# Assembly of a Library of Pel-Oligosaccharides Featuring *α*-Glucosamine and *α*-Galactosamine Linkages

**DOI:** 10.3389/fchem.2022.842238

**Published:** 2022-01-26

**Authors:** Yongzhen Zhang, Liming Wang, Herman S. Overkleeft, Gijsbert A. van der Marel, Jeroen D. C. Codée

**Affiliations:** ^1^ Institute of Chemistry, Leiden University, Leiden, Netherlands; ^2^ National Research Centre for Carbohydrate Synthesis, Jiangxi Normal University, Nanchang, China

**Keywords:** glycosylation, sereoselectivity, bacterial polysaccharides, *pseudomonas aeruginosa*, biofilm

## Abstract

*Pseudomonas aeruginosa*, a pathogenic Gram-negative bacterium for which currently antibiotic resistance is posing a significant problem and for which no vaccines are available, protects itself by the formation of a biofilm. The Pel polysaccharide, a cationic polymer composed of *cis*-linked galactosamine (GalN), *N*-acetyl galactosamine (GalNAc), glucosamine (GlcN) and *N*-acetyl glucosamine (GlcNAc) monosaccharides, is an important constituent of the biofilm. Well-defined Pel oligosaccharides will be valuable tools to probe the biosynthesis machinery of this polysaccharide and may serve as diagnostic tools or be used as components of glycoconjugate vaccines. We here, report on the development of synthetic chemistry to access well-defined Pel-oligosaccharides. The chemistry hinges on the use of di-*tert*-butylsilylidene protected GalN and GlcN building blocks, which allow for completely *cis*-selective glycosylation reactions. We show the applicability of the chemistry by the assembly of a matrix of 3 × 6 Pel heptasaccharides, which has been generated from a single set of suitably protected Pel heptasaccharides, in which a single glucosamine residue is incorporated and positioned at different places along the Pel oligo-galactosamine chain.

## Introduction

**FIGURE 1:**
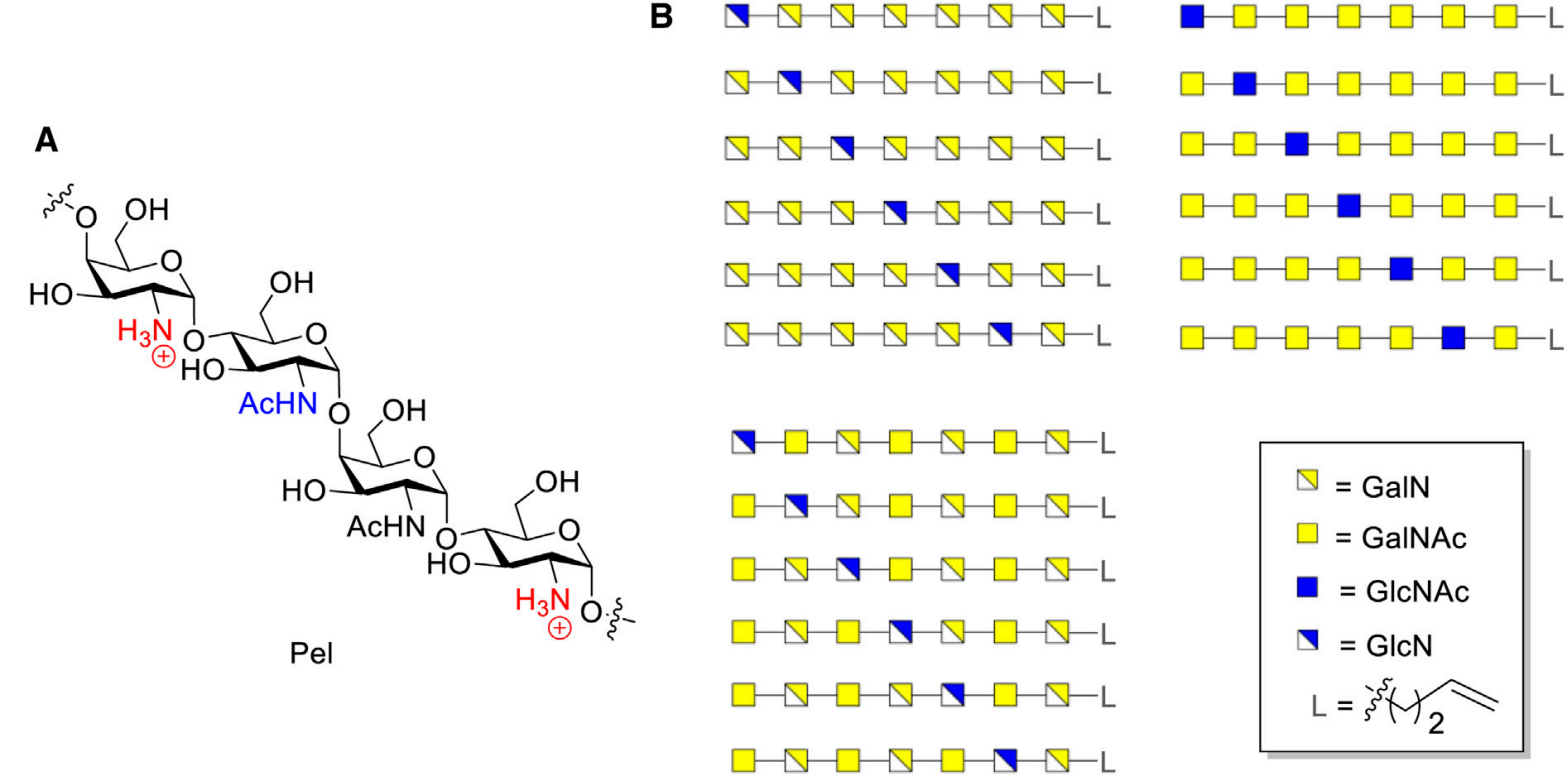
**(A)** Structure of Pel. **(B)** Structures of designed Pel oligomers.

*Pseudomonas aeruginosa* is an opportunistic Gram-negative pathogen that can cause both acute and chronic infections in immunocompromised patients (Franklin et al., 2011; Ghafoor et al., 2011; Ma et al., 2012; Jennings et al., 2015; Marmont et al., 2017; Whitfield et al., 2020). *P. aeruginosa* can become resistant to antibiotics due to its ability to form a biofilm which complicates the treatment of its infections. As part of the biofilm three exopolysaccharides are synthesized, alginate, Pel and Psl (Franklin et al., 2011). Alginate is a negatively charged polymer of mannuronic and guluronic acid (Flo et al., 2002), while Psl is a neutral polysaccharide composed of a pentasaccharide repeat containing glucose, rhamnose and mannose (Jackson et al., 2004). Pel is a positively charged polymer, and although its structure has not been fully characterized it is thought to be composed of *α*-1,4-linked *N*-acetylgalactosamine (GalNAc) and *N*-acetyl-glucosamine (GlcNAc), both of which can be de-acetylated to give galactosamine (GalN) and glucosamine (GlcN) residues, respectively (Figure 1A). The GalN(Ac): GlcN(Ac) ratio has been reported to be ±6:1 (Jennings et al., 2015). Pel plays an important role in maintaining cell-cell interactions in biofilms and affords protection to the bacterium by enhancing resistance to aminoglycoside antibiotics (Colvin et al., 2011). Well-defined fragments of the Pel polymer can serve as powerful research tools in various interconnected fields of research. They may serve as synthetic antigens in the generation of potential *pseudomonas* vaccines and they can be used in elucidating biosynthesis pathways and characterizing the enzymes involved therein. This may open up avenues to interfere with the biosynthesis and eventually generate anti-bacterial compounds. Because of the seemingly random distribution of monosaccharides in Pel, it is impossible to isolate well defined fragments from natural sources and therefore organic synthesis is the method of choice to provide these.

The key to the assembly of Pel fragments is the stereoselective introduction of *α*-GalN, *α*-GalNAc, *α*-GlcN and *α*-GlcNAc linkages. We have previously described the successful application of the 4,6-*O*-di-*tert*-butylsilylidene (DTBS) directed *α*-galactosylation methodology, developed by Kiso’s group (Imamura et al., 2003; Imamura et al., 2005; Sato et al., 2009; Imamura et al., 2016; Kazakova et al., 2020; Wang et al., 2020), for the synthesis of the structurally related galactosaminogalactan (GAG) homo- and hetero-oligosaccharides that occur in the cell wall of *Aspergillus fumigatus* and that are composed of 1,4-linked *α*-Gal, *α*-GalN, *α*-GalNAc residues (Bamford et al., 2019; Le Mauff et al., 2019; Bamford et al., 2020; Zhang et al., 2020a). The application of 4,6-*O*-DTBS protected GalN_3_ and GalNHTCA donors resulted in glycosylations with high *α*-stereoselectivity to give a row of GAG fragments, having both GalN and GalNAc constituents. The high *α*-stereoselectivity of the glycosylations proved to be insensitive to the nature of the C-2-*N*-acyl group, capable of neighboring group participation. On the basis of these results, we selected DTBS-protected GalN donors as building blocks for the construction of *α*-GalN and *α*-GalNAc linkages in Pel. The formation of similar *α*-GlcN linkages is more challenging and substantial effort has been expended to develop a procedure for the stereoselective introduction of *α*-GlcN linkages (Benakli et al., 2001; Wei and Kerns, 2005; Manabe et al., 2006; Park et al., 2007; Geng et al., 2008; Olsson et al., 2008; Mensah et al., 2010; Ye and Geng, 2010; Ingle et al., 2013; van der Vorm et al., 2017; Zhang et al., 2020). Recently, we have reported on an effective synthetic strategy to assemble a Pel-type oligosaccharide, containing 1,4-linked GalNAc and GlcNAc residues (Wang et al., 2020). A [2 + 2 + 2] strategy was developed for the synthesis of a hexasaccharide in which the glucosamine linkages were constructed using a *N*-methyl-*N*-phenylformamide (MPF)-modulated glycosylation methodology. We have previously also systematically evaluated a set of glycosylation reactions between a series of 4,6-tethered glucosazide donors and a panel of acceptors to find that with increasing reactivity of the studied GlcN_3_-donors and decreasing nucleophilicity of the acceptors, the *α*-selectivity of the glycosylations increased (van der Vorm et al., 2017). The most reactive GlcN_3_ donor that was studied carried a 4,6-DTBS group and its reaction with the model nucleophile trifluoroethanol (TFE) gave the *α*-linked product exclusively. As the nucleophilicity of the C-4-OH in GalN moieties is relatively low, the DTBS-GlcN_3_ donors may represent promising building blocks for the construction of *α*-GlcN-(1→4)-GalN linkages.

We here describe the synthesis of a library of Pel fragments with DTBS-directed glycosylation methodology. A library of hetero-oligomers containing *α*-GalN/*α*-GalNAc and *α*-GlcN/*α*-GlcNAc residues at predetermined positions, was designed (Figure 1B). A set of heptamers, each of which contains one GlcN/GlcNAc-residue and six GalN/GalNAc residues, was selected because of the GalNAc:GlcNAc ratio that is present in naturally occurring Pel polysaccharides, while some of the residues have been deacetylated (Jennings et al., 2015). Also a spacer was incorporated at the reducing end of the heptamers for future conjugation purposes.

## Results and Discussion

As the DTBS-directed *α*-galactosylation methodology is well established, attention was first paid to the formation of *α*-GlcN_3_-(1→4)-GalN_3_ linkages. A set of glycosylation reactions was investigated using GlcN_3_ donors **1–4** (van der Vorm et al., 2017; Revuelta et al., 2018; Wang et al., 2018) and GalN_3_ acceptors **5–7** (Wang et al., 2020; Zhang et al., 2020a) (Table 1). First, the additive *N*-methyl-*N*-phenylformamide (MPF) controlled *α*-glycosylation methodology was attempted to introduce the *α*-GlcN linkage. With this methodology, glycosylation of benzylated GlcN_3_ donor **1** with benzylated GalN_3_ acceptor **7** led to the disaccharide **8** with 7:1 *α*/*β*-selectivity, but the yield was only 47% (Table 1, entry 1). Using the same conditions, coupling of 4,6-DTBS-protected GlcN_3_ donor **4** with GalN_3_ **7** afforded the dimer in a mere 5% yield (entry 2), owing to the low reactivity of the GalN_3_ C4-OH acceptor. Next, a pre-activation strategy, using GlcN_3_ donors **2** and **3** as donors was explored. Benzylidene-protected donor **2** reacted with acceptor **5**, to afford disaccharide **9** in 38% yield and with a 3.5/1 *α*/*β* ratio (entry 3). When the more reactive DTBS-protected donor **3** was treated with **5**, a slightly better *α*-selectivity was obtained (*α*/*β* = 5/1, entry 4). Condensation of donor **3** with 6-O-Bn protected acceptor **6** led to **11** (Wang et al., 2020) in excellent yield and *α*-selectivity (entry 5). By contrast, changing the linker of the acceptor to 3-buten-ol, which is more convenient for future conjugation purposes, gave no glycosylation product (entry 6). Condensation of GlcN_3_ donor **3** and acceptor **7**, promoted by NIS and TfOH at −40°C, also failed to afford the product (entry 7). To further improve the reaction, imidate donor **4** was coupled with acceptor **5**, under influence of TBSOTf, giving dimer **10** with moderate *α*-selectivity (*α*/*β* = 3.7/1, entry 8). Gratifyingly, performing the glycosylation of donor **4** and acceptor **7**, at −10°C with TfOH as promotor, furnished the desired disaccharide **12** in 77% yield with excellent *α*-selectivity (>20:1, entry 9). Based on these model reactions, the DTBS-tethered GlcN_3_ donor **4** was chosen for the construction of *α*-GlcN_3_-(1→4)-GalN_3_ linkages, and the benzyl group was preferred for the protection of C6-OH of the GalN acceptors. Notably, the implementation of this strategy would match exceptionally well with the strategy we previously developed for the introduction of *α*-GalN and *α*-GalNAc linkages in the synthesis of the related GAG oligosaccharides.

**TABLE 1:** Glycosylation between GlcN_3_ donors and GalN_3_ acceptors.

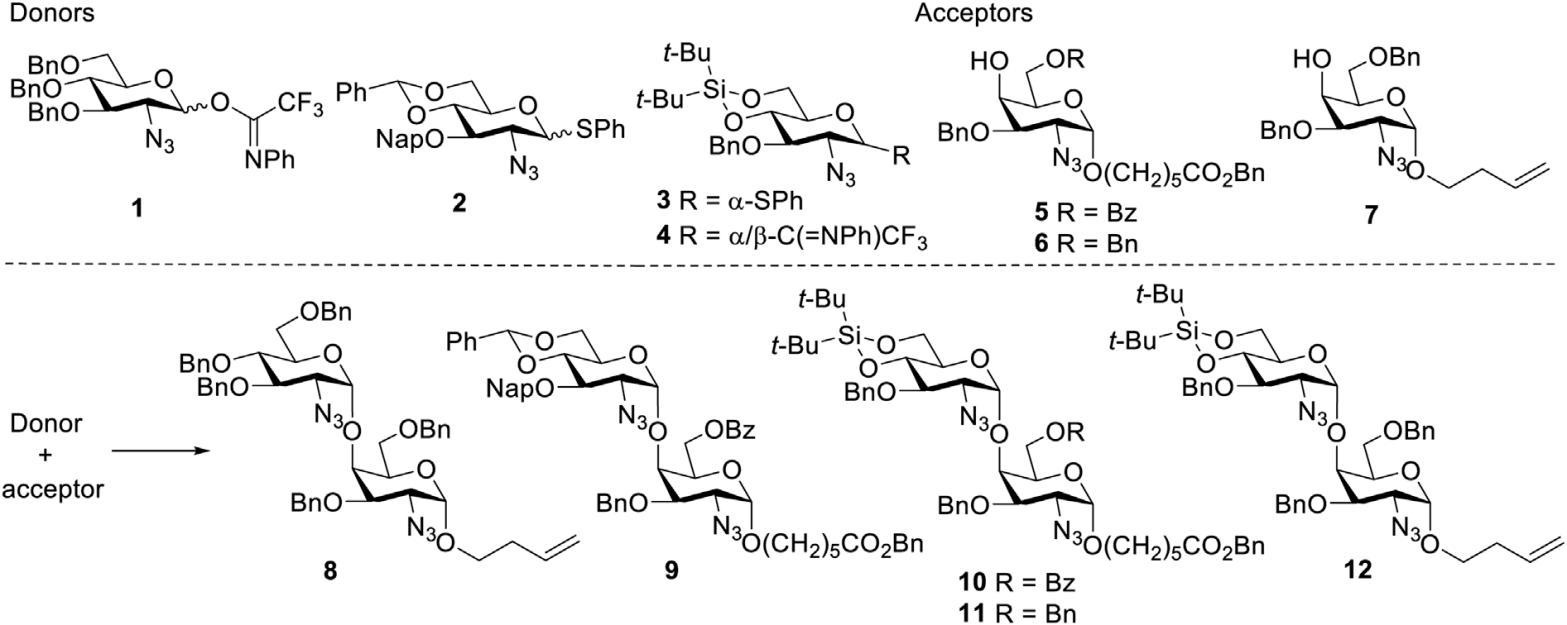

Entry	Donor	Acceptor	Reagents and conditions	Temperature	Product	*α*/*β*	Yield
1	**1**	**7**	TfOH, MPF, DCM	−78 to 0°C	**8**	7/1	47%
2	**4**			−78 to 0°C	**12**	>10/1	5%
3	**2**	**5**	Tf_2_O, Ph_2_SO, TTBP, DCM	−78 to −40°C	**9**	3.5/1	38%
4	**3**	**5**		−40°C	**10**	5/1	65%
5		**6**		−40°C	**11**	13/1	81%
6		**7**		−40°C	**12**	—	—
7	**3**	**7**	NIS, TfOH, DCM	−40 to 0°C	**12**	—	—
8	**4**	**5**	TBSOTf, DCM	−78 to −40°C	**10**	3.7/1	62%
9		**7**	TfOH, DCM	−10°C	**12**	>20/1	77%

The bold numbers are the sequence numbers of compounds.

**FIGURE 2:**
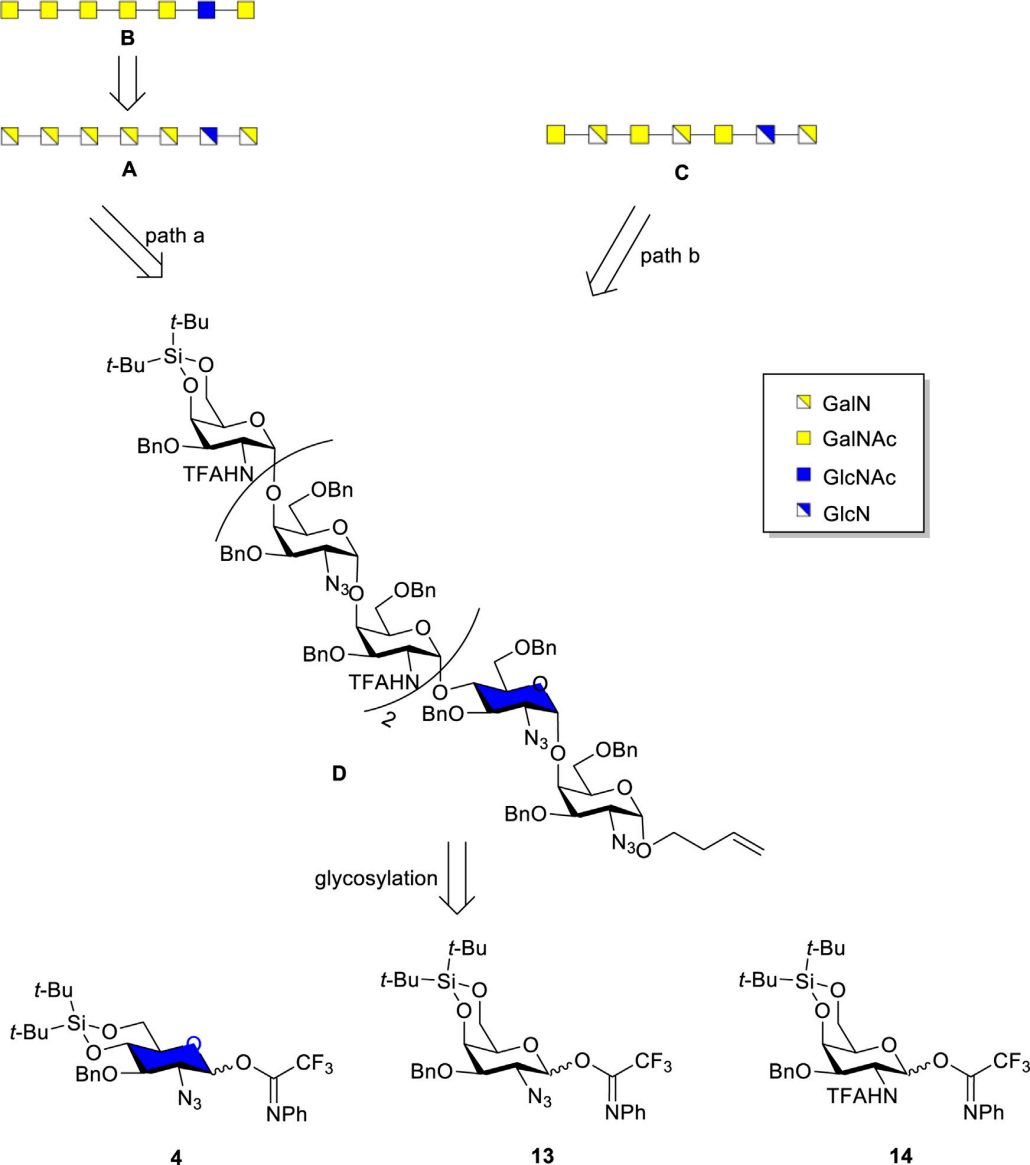
Retrosynthetic analysis of Pel heptasaccharides.

With conditions in hand to construct the required *α*-GalN and *α*-GlcN linkages, attention was directed to the assembly of a library of Pel heptamers, consisting of (3 × 6) members, that can be achieved by the synthesis of six protected heptameric precursors and subjecting these to different deprotection procedures. The projected eighteen heptamers contain one GlcN or GlcNAc, differently positioned in the heptameric chain, while the remaining residues are all GalN, all GalNAc or alternating GalN and GalNAc (Figure 1B). The retrosynthesis of Pel heptamers **A–C** with either GlcN or GlcNAc at the second position from the reducing end of the heptamer is depicted in Figure 2. This retrosynthesis also applies to the remaining members of the projected library that can be accessed using the same strategy. The deprotected heptamers **A–C** are derived from protected heptamer **D** through different procedures for the removal of the protecting groups. In path a, the sequence of deprotection steps include DTBS removal, reduction of azido groups, and removal of Bn and TFA groups via Birch reduction to afford compound **A**, containing GalN and GlcN residues. Birch reduction is chosen to avoid reduction of C-C double bond in the linker (Zhang et al., 2019). Acetylation of free amine groups in **A** can furnish heptamer **B**. In path b, the C2-*N*-TFA groups are first removed, followed by desilylation and acetylation of the released amino groups, after which reduction the azido and Bn-groups should give the heptamer **C**. The common protected heptamer **D** can be constructed with GlcN_3_ donor **4**, GalN_3_ donor **13** and GalNHTFA donor **14**, which would serve as precursors for the GlcN, GlcNAc, GalN and GalNAc residues.

Table 2 summarizes the syntheses of the six fully protected Pel heptasaccharides (**20**, **26**, **31**, **35**, **38**, **40**) with one GlcN_3_ residue at different positions. The elongation cycle consisted of the following three-steps: 1) Glycosylation using the donor of choice, 2) DTBS-removal with HF/pyridine and 3) selective benzylation of the primary alcohol group. The Bn group can be regioselectively introduced under the aegis of Taylor’s borinic acid catalyst (Chan and Taylor, 2011; Lee and Taylor, 2011; Lee et al., 2012). As can be seen from the Table, the heptasaccharide **20** (or **D** in Figure 2) with the GlcN_3_ moiety at the second position from the reducing end of the heptamer was first synthesized. Condensation of GlcN_3_ donor **4** and acceptor **7** led to the disaccharide **12** using a TfOH promoted condensation at −10°C (Table 1). Next the DTBS group was cleaved and the liberated 6-OH was benzylated selectively to form the desired 4-OH acceptor, which was reacted with GalNHTFA donor **14** giving the trisaccharide **16** in 73% yield over the three steps. However, the relatively moderate yield of the glycosylation for the tetra- and pentamer (56% for **17** and 51% for **18**) was an incentive to optimize the glycosylation reaction conditions. It was found that implementation of a “reverse addition sequence” strategy, in which the acceptor and activator are mixed, after which the donor is slowly added, greatly improved the reaction yields (71% for **17** and 72% for **18**). Elongation of the pentamer with another copy of the GalN_3_ donor **13** and subsequently the GalNHTFA building block **14**, delivered heptasaccharide **20** in excellent yields. It should be noted that the desilylation reactions and regioselective benzylations all occurred with excellent chemo- and regioselectivity, showing the effectivity of these reactions to be independent on glycan length.

**TABLE 2:** Synthesis of Pel oligomers.

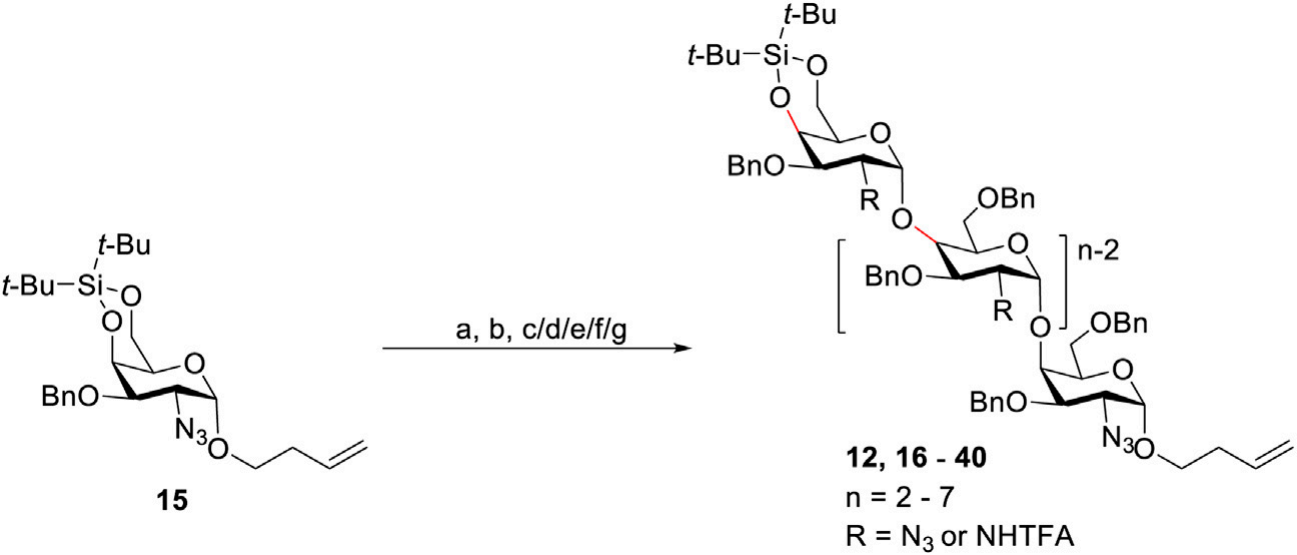

*n*	(GluR)_m_ GalN_3_	Yield^(^ ^h^ ^]^
2	GlcN_3_ GalN_3_	**12** (71%)^(^ ^c^ ^)^
3	GalNHTFA GlcN_3_ GalN_3_	**16** (73%)^(^ ^a^ ^,^ ^b^ ^,^ ^e^ ^)^
4	GalN_3_ GalNHTFA GlcN_3_ GalN_3_	**17** (56%)^(^ ^a^ ^,^ ^b^ ^,^ ^d^ ^]^ (71%)^(^ ^a^ ^,^ ^b^ ^,^ ^f^ ^)^
5	GalNHTFA GalN_3_ GalNHTFA GlcN_3_ GalN_3_	**18** (51%)^(^ ^a^ ^,^ ^b^ ^,^ ^e^ ^)^ (72%)^(^ ^a^ ^,^ ^b^ ^,^ ^g^ ^)^
6	GalN_3_ GalNHTFA GalN_3_ GalNHTFA GlcN_3_ GalN_3_	**19** (69%)^(^ ^a^ ^,^ ^b^ ^,^ ^f^ ^)^
7	GalNHTFA GalN_3_ GalNHTFA GalN_3_ GalNHTFA GlcN_3_ GalN_3_	**20** (67%)^(^ ^a^ ^,^ ^b^ ^,^ ^g^ ^)^
2	GalNHTFA GalN_3_	**21** (79%)^(^ ^a^ ^,^ ^b^ ^,^ ^e^ ^)^
3	GlcN_3_ GalNHTFA GalN_3_	**22** (74%)^(^ ^a^ ^,^ ^b^ ^,^ ^c^ ^)^
4	GalN_3_ GlcN_3_ GalNHTFA GalN_3_	**23** (68%)^(^ ^a^ ^,^ ^b^ ^,^ ^f^ ^)^
5	GalNHTFA GalN_3_ GlcN_3_ GalNHTFA GalN_3_	**24** (78%)^(^ ^a^ ^,^ ^b^ ^,^ ^g^ ^)^
6	GalN_3_ GalNHTFA GalN_3_ GlcN_3_ GalNHTFA GalN_3_	**25** (68%)^(^ ^a^ ^,^ ^b^ ^,^ ^f^ ^)^
7	GalNHTFA GalN_3_ GalNHTFA GalN_3_ GlcN_3_ GalNHTFA GalN_3_	**26** (76%)^(^ ^a^ ^,^ ^b^ ^,^ ^g^ ^)^
3	GalN_3_ GalNHTFA GalN_3_	**27** (79%)^(^ ^a^ ^,^ ^b^ ^,^ ^f^ ^)^
4	GlcN_3_ GalN_3_ GalNHTFA GalN_3_	**28** (73%)^(^ ^a^ ^,^ ^b^ ^,^ ^c^ ^)^
5	GalNHTFA GlcN_3_ GalN_3_ GalNHTFA GalN_3_	**29** (70%)^(^ ^a^ ^,^ ^b^ ^,^ ^g^ ^)^
6	GalN_3_ GalNHTFA GlcN_3_ GalN_3_ GalNHTFA GalN_3_	**30** (73%)^(^ ^a^ ^,^ ^b^ ^,^ ^f^ ^)^
7	GalNHTFA GalN_3_ GalNHTFA GlcN_3_ GalN_3_ GalNHTFA GalN_3_	**31** (74%)^(^ ^a^ ^,^ ^b^ ^,^ ^g^ ^)^
4	GalNHTFA GalN_3_ GalNHTFA GalN_3_	**32** (66%)^(^ ^a^ ^,^ ^b^ ^,^ ^g^ ^)^
5	GlcN_3_ GalNHTFA GalN_3_ GalNHTFA GalN_3_	**33** (74%)^(^ ^a^ ^,^ ^b^ ^,^ ^c^ ^)^
6	GalN_3_ GlcN_3_ GalNHTFA GalN_3_ GalNHTFA GalN_3_	**34** (56%)^(^ ^a^ ^,^ ^b^ ^,^ ^f^ ^)^
7	GalNHTFA GalN_3_ GlcN_3_ GalNHTFA GalN_3_ GalNHTFA GalN_3_	**35** (52%)^(^ ^a^ ^,^ ^b^ ^,^ ^g^ ^)^
5	GalN_3_ GalNHTFA GalN_3_ GalNHTFA GalN_3_	**36** (74%)^(^ ^a^ ^,^ ^b^ ^,^ ^f^ ^)^
6	GlcN_3_ GalN_3_ GalNHTFA GalN_3_ GalNHTFA GalN_3_	**37** (77%)^(^ ^a^ ^,^ ^b^ ^,^ ^c^ ^)^
7	GalNHTFA GlcN_3_ GalN_3_ GalNHTFA GalN_3_ GalNHTFA GalN_3_	**38** (50%)^(^ ^a^ ^,^ ^b^ ^,^ ^g^ ^)^
6	GalNHTFA GalN_3_ GalNHTFA GalN_3_ GalNHTFA GalN_3_	**39** (67%)^(^ ^a^ ^,^ ^b^ ^,^ ^g^ ^)^
7	GlcN_3_ GalNHTFA GalN_3_ GalNHTFA GalN_3_ GalNHTFA GalN_3_	**40** (60%)^(^ ^a^ ^,^ ^b^ ^,^ ^c^ ^)^

a HF/pyridine, THF, 0°C to rt.

b Ph_2_BO(CH_2_)_2_NH_2_, KI, K_2_CO_3_, BnBr, MeCN, 60°C.

c **4**, TfOH, 4Å MS, DCM, −10°C.

d **13**, TfOH, 4Å MS, DCM, 0°C.

e **14**, TfOH, 4Å MS, DCM, 0°C.

f TfOH, 4Å MS, DCM, then **13** added in 1 h, 0°C for 3, 4 and 5-mers, −20°C for 6 and 7-mers.

g TfOH, 4Å MS, DCM, then **14** added in 1 h, 0°C for 4 and 5-mers, −20°C for 6 and 7-mers.

h yields for over three steps.

i The bold numbers are the sequence numbers of compounds.

In an analogous way, the assembly of target heptasaccharides **26**, **31**, **35**, **38**, and **40** with a GlcN_3_ moiety at the positions 3-7 was accomplished with building blocks **4**, **13** and **14**. Repetition of the elongation cycle, comprising the same three steps as described above led to all target heptasaccharides. The glycosylation reactions proceeded efficiently providing the intermediate and target oligosaccharides (*n* = 2–7) with excellent stereoselectivity and good yields (50–79% yields for three steps). The mixed sequence structures were generated uneventfully, showing the chemistry developed to be applicable to any type of Pel-target.

**SCHEME 1:**
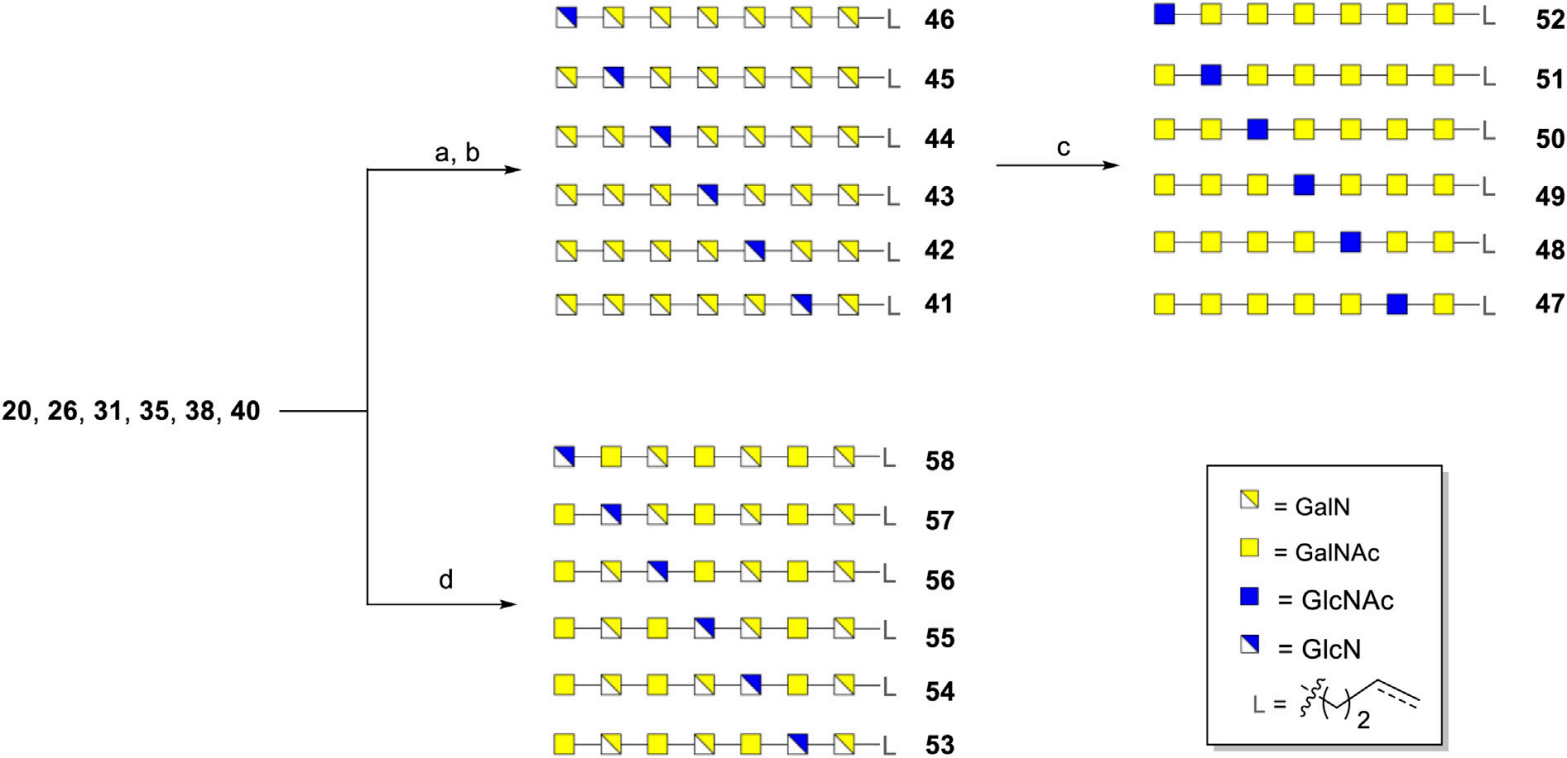
Deprotection of synthetic Pel heptasaccharides. a) i) HF/pyridine, THF, rt; ii) HS(CH_2_)_3_SH, Et_3_N, pyridine/H_2_O, rt. b) Na, NH_3_ (liq.), THF, t-BuOH, 3-buten-1-ol, −78°C, yields for **41**: 69% (12/1 with:without C=C); **42**: 48% (23/1); **43**: 84% (19/1); **44**: 53% (50/1); **45**: 59% (25/1); **46**: 85% (43/1). c) Ac_2_O, H_2_O, NaHCO_3_, rt, yields for **47**: 90%; **48**: 91% (11/1); **49**: 91% (32/1); **50**: 90% (32/1); **51**: 89% (21/1); **52**: 88% (12/1). d) i) HF/pyridine, THF, rt; ii) Pd(OH)_2_/C, H_2_, AcOH, THF/t-BuOH/H_2_O, rt; iii) Boc_2_O, NaHCO_3_, H_2_O, rt; iv) NH_3_^**.**^H_2_O, 60°C; v) Ac_2_O, NaHCO_3_, H_2_O, rt; vi) 30% TFA in H_2_O, L = (CH_2_)_3_CH_3_, yields for **53**: 31%; **54**: 25%; **55**: 24%; **56**: 18%; **57**: 30%; **58**: 18%.

With all six protected heptasaccharides in hand, deprotection conditions were explored to complete the assembly of all projected Pel oligomers (Scheme 1). First, the set of 7-mers containing solely *α*-GalN and *α*-GlcN moieties was generated. Removal of the DTBS-group in heptamers **20**, **26**, **31**, **35**, **38** and **40** was performed with HF/pyridine and the azido-groups could be reduced with HS(CH_2_)_3_SH, after which the Bn groups together with the TFA groups were cleaved using sodium in ammonia and THF, affording the 7-mers **41–46** in 48–85% yields. In the Birch reduction, allyl carbinol was used as a scavenger to prevent reduction of the linker alkene. A portion of the 7-mers **41–46** was chemoselectively acetylated to provide the second set of heptamers **47–52**, composed of *α*-GalNAc and *α*-GlcNAc moieties. Furthermore, the heptamers **20**, **26**, **31**, **35**, **38** and **40** were transformed into the third set of GalN-, GalNAc and GlcN-containing heptamers **53–58**. Similar to the first series, the silylidene groups were first removed. However, we then found that the TFA groups could not be cleaved even with strong basic/nucleophilic conditions and high temperatures (4M NaOH, 80°C). Also attempts to remove the TFA groups with the assistance of microwave failed (see experimental section, Supplementary Table S1). A solution for this problem was found by first removing the benzyl ethers and concomitant reduction of the azido groups using hydrogenation over Pd(OH)_2_/C, followed by the temporary protection of the generated free amino groups with a Boc group. At this stage, the TFA groups could be removed with NH_3_^**.**^H_2_O at 60°C, after which acetylation of generated amines and subsequent removal of the Boc groups with 30% TFA provided the heptamers **53–58** in 18–31% yields. Although this sequence of reactions sacrificed the alkene group in the linker moiety it did grant access to the last series of Pel-oligosaccharides.

## Conclusion

In conclusion, synthetic methodology enabling the assembly of Pel fragments has been developed. Key features of the synthetic strategy include the use of DTBS-directed *α*-glycosylation methodology and a regioselective benzylation procedure. The DTBS-directed glycosylation was not only successfully applied for the construction of *α*-GalN_3_ and *α*-GalNTFA linkages, as already previously described, it also proved applicable for the synthesis of *α*-GlcN_3_ linkages. With the increasing length of the oligosaccharides, the glycosylation yields decreased significantly, owing to the reduced nucleophilicity of the acceptors. Application of a reverse-addition-sequence strategy adequately improved the yields of the glycosylations providing the longer oligosaccharides in good yield. Six protected heptamers with different composition were subjected to different deprotection protocols, providing three sets of heptamers containing *α*-GlcN-*α*-GalN, *α*-GlcNAc-*α*-GalNAc and *α*-GlcN-*α*-GalNAc-*α*-GalN combinations. Unexpectedly, it proved impossible to remove the *N*-TFA groups in the heptamers carrying benzyl protecting groups. Fortunately, a protocol in which the benzyl and azide groups were first reduced, after which the liberated amines were temporarily masked as Boc-carbamates, allowed for removal of the TFA groups, using aqueous ammonia hydroxide. The synthetic Pel heptamers will be valuable for the define establishment of the Pel structure, studies of the biosynthesis machinery and biofilm forming process as well as the development of vaccines and diagnostic tools to combat and monitor *P. aeruginosa* infections.

## Data Availability

The original contributions presented in the study are included in the article/Supplementary Material, further inquiries can be directed to the corresponding author.
