# Supplementary material for: Assembly of a Library of Pel-Oligosaccharides Featuring α-Glucosamine and α-Galactosamine Linkages
Source: Front Chem. 2022 Jan 26;10:842238. doi: 10.3389/fchem.2022.842238 (PMC8826555; doi:10.3389/fchem.2022.842238)
Supplement: Supplementary file 1 [file DataSheet1.PDF]

Supplementary Material

Table of Contents

Table of Contents S1
Experimental Procedures and Characterization Data of Synthesized Pel Oligomers S2
General experimental procedures S2
Experimental Procedures and Characterization Data of Products S2
References S35
NMR Spectrum S36

Experimental Procedures and Characterization Data of Synthesized Pel Oligomers

General experimental procedures

All reagents were of commercial grade and used as received. All moisture sensitive reactions were performed under an argon atmosphere. DCM used in the glycosylation reactions was dried with flamed 4Å molecular sieves before being used. Reactions were monitored by TLC analysis with detection by UV (254 nm) and where applicable by spraying with 20% sulfuric acid in EtOH or with a solution of $(NH_4)_6Mo_7O_{24}\cdot 4H_2O$ (25 g/L) and $(NH_4)_4Ce(SO_4)_4\cdot 2H_2O$ (10 g/L) in 10% sulfuric acid (aq.) followed by charring at ~150 °C. Flash column chromatography was performed on silica gel (40-63μm). ^{1}H and ^{13}C spectra were recorded on a Bruker AV 400 and Bruker AV 500 in $CDCl_3$ or D_2O. Chemical shifts (δ) are given in ppm relative to tetramethylsilane as internal standard (^{1}H NMR in $CDCl_3$) or the residual signal of the deuterated solvent. Coupling constants (*J*) are given in Hz. All ^{13}C spectra are proton decoupled. NMR peak assignments were made using COSY and HSQC experiments, where applicable Clean TOCSY, HMBC and GATED experiments were used to further elucidate the structure. Size-exclusion chromatography was carried out using Sephadex LH-20.

General procedure for glycosylation with imidate donors 4, 13 and 14 (procedure A)

The donor (1.5 – 3.0 eq) and acceptor (1.0 eq) were co-evaporated with toluene (three times). The residue was dissolved in dry DCM (0.1 M acceptor in DCM) under nitrogen and stirred over fresh flame-dried molecular sieves 4Å. The solution was cooled to 0 ºC, after which TfOH (0.1 – 0.3 eq) was added. The reaction was stirred at 0 ºC until TLC-analysis showed complete conversion of the acceptor. The reaction was quenched with Et_3N, diluted with DCM, washed with saturated $NaHCO_3$ and brine. The organic phase was dried with anhydrous $MgSO_4$, filtered and concentrated *in vacuo*. The products were purified by silica gel column chromatography (See experimental description below for eluent system).

General procedure for glycosylation with imidate donors 13 and 14 (Reverse-addition sequence, procedure B)

The acceptor (1.0 eq) was co-evaporated with toluene (three times), and the residue was dissolved in dry DCM (0.1 M acceptor in DCM) under nitrogen and stirred over fresh flame-dried molecular sieves 4Å. The solution was cooled to 0 ºC, after which TfOH (0.1 – 0.3 eq) was added. The solution of donor (1.5 -4.0 eq) in dry DCM was added slowly into the reaction mixture within 1 hour. The reaction was stirred at 0 ºC until TLC-analysis showed complete conversion of the acceptor. The reaction was quenched with Et_3N, diluted with DCM, washed with saturated $NaHCO_3$ and brine. The organic phase was dried with anhydrous $MgSO_4$, filtered and concentrated *in vacuo*. The products were purified by silica gel column chromatography (See experimental description below for eluent system).

General procedure for the deprotection of di-*tert*-butyl silylidene group (general procedure C)

HF/pyridine (16 eq) solution was added to a solution of starting material in THF at 0 ºC. The reaction was warmed to room temperature and stirred until TLC-analysis indicated full consumption of the starting material (± 1h). Then the mixture was diluted with DCM and washed with saturated $NaHCO_3$ and brine, dried with anhydrous $MgSO_4$, filtered and concentrated *in vacuo*. The product was purified by silica gel column chromatography (See experimental description below for eluent system).

General procedure for selective benzylation of primary alcohol (general procedure D)

K_2CO_3 (1.1 eq), KI (1.5 eq) and $Ph_2BO(CH_2)_2NH_2$ (0.1-0.2 eq) were added to the solution of starting material in MeCN (0.05 M). Then BnBr was added in the solution. The reaction was allowed to stirred at 60 ºC until TLC-analysis showed complete conversion of the starting material. Then reaction was quenched with H2O after completed checking by TLC, filtered and concentrated *in vacuo*. The product was purified by silica gel column chromatography (See experimental description below for eluent system).

Experimental Procedures and Characterization Data of Products

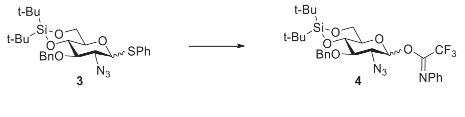

2-Azido-3-*O*-benzyl-2-deoxy-4,6-di-*tert*-butylsilylidene-1-*O*-(*N*-phenyl-trifluoroacetimidoyl)-α/β-D-glucopyranoside (4)

NIS (525 mg, 2.33 mmol) was added to the solution of compound **3**[1] (820 mg, 1.55 mmol) in Acetone/H_2O (16 ml/1.6 ml) at 0 °C. The reaction was slowly warmed to room temperature and stirred until TLC-analysis indicated full consumption of the starting material (± 1H). Then the mixture was diluted with DCM and washed with saturated $Na_2S_2O_3$ and brine, dried with anhydrous $MgSO_4$, filtered and concentrated *in vacuo*. The product S1 was purified by silica gel column chromatography (pentane:EtOAc = 8:1). Cs_2CO_3 (440 mg, 1.35 mmol) was added to the solution of the residue in 15 ml acetone. The mixture was stirred at 0 °C for 15 minutes. Then CF_3C(=NPh)Cl (420 mg, 2.03 mmol) was added to the solution. which was slowly warmed to room temperature and stirred overnight. The reaction was quenched with Et_3N and concentrated *in vacuo*. The product **4** was purified by silica gel column chromatography (pentane:Et_2O = 30:1 – 10:1). Compound **4** (828 mg, α:β = 2:1, 88% yield) was obtained as yellow syrup. α-Isomer: ^{1}H NMR ($CDCl_3$, 400 MHz) δ 7.46 – 7.39 (m, 2H), 7.38 – 7.24 (m, 6H), 7.13 – 7.04 (m, 1H), 6.83 (d, *J* = 7.8 Hz, 2H, *aromatic* H), 6.25 (s, 1H, H-1), 5.09 (d, *J* = 10.6 Hz, 1H, *PhC*H*H*O), 4.84 (d, *J* = 10.6 Hz, 1H, *PhC*H*H*O), 4.15 (dd, *J* = 9.2, 3.9 Hz, 1H, H-6), 4.07 – 3.82 (m, 4H, H-3, 4, 5, 6), 3.61 – 3.47 (m, 1H, H-2), 1.09 (s, 9H, C*H*$_3$), 1.04 (s, 9H, C*H*$_3$). ^{13}C NMR (100 MHz, $CDCl_3$) δ 143.22, 137.92, 129.36, 128.89, 128.55, 128.47, 128.44, 128.09, 126.36, 124.66, 119.44 (*aromatic* C/C*H*), 116.03 (*a*d, *J* = 286 Hz, C*F*$_3$), 93.58 (C-1), 79.42 (C-3), 78.36 (C-4), 75.70 (C*H*$_2$*Ph*), 68.96 (C-5), 66.38 (C-6), 61.79 (C-2), 27.46, 26.99 (2 C*H*$_3$), 22.74, 20.03 (2 C-*Si*). β-Isomer: ^{1}H NMR ($CDCl_3$, 400 MHz) δ 7.42 (d, *J* = 6.9 Hz, 2H, *aromatic* H), 7.38 – 7.24 (m, 5H, *aromatic* H), 7.11 – 7.05 (m, 1H, *aromatic* H), 6.83 (d, *J* = 7.7 Hz, 2H, *aromatic* H), 5.60 (bs, 1H, H-1), 5.01 (d, *J* = 11.0 Hz, 1H, *PhC*H*H*O), 4.83 (d, *J* = 11.0 Hz, 1H, *PhC*H*H*O), 4.24 – 4.09 (m, 1H, H-6), 4.03 – 3.85 (m, 2H, H-4, 6), 3.67 – 3.20 (m, 3H, H-2, 3, 5), 1.08 (s, 9H, C*H*$_3$), 1.00 (s, 9H, C*H*$_3$). ^{13}C NMR (100 MHz, $CDCl_3$) δ 143.16, 137.91, 128.86, 128.50, 128.40, 128.07, 124.61, 119.28 (*aromatic* C/C*H*), 116.03 (*a*d, *J* = 286 Hz, C*F*$_3$), 95.54 (C-1), 82.08 (C-3), 77.63 (C-4), 75.37 (C*H*$_2$*Ph*), 71.15 (C-5), 66.04 (C-6), 64.47 (C-2), 27.45 (C*H*$_3$), 27.05 (C*H*$_3$), 22.73, 20.01 (2 C-*Si*). HR-MS: Calculated for $C_{29}H_{37}N_4O_5F_3Si$ $[M+Na]^+$: 629.23775, found: 629.23788.

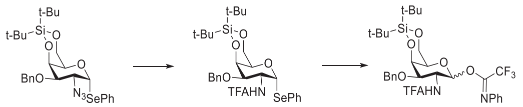

S1 S2 14

Phenyl 3-*O*-benzyl-2-deoxy-1-seleno-4,6-di-*tert*-butylsilylidene-2-trifluoroacetamido-α-D-galactopyranoside (S2)

1,3-Dithiolpropane (10.1 ml, 100 mmol) and trimethylamine (11.6 ml, 83.5 mmol) were added to the solution of compound **S1**[2] (9.6 g, 16.7 mmol) in pyridine/water (80 ml/20 ml). The mixture was protected from light and stirred at room temperature overnight. The fluent was evaporated and co-evaporated with toluene. The residue was dissolved in 50 ml pyridine, after which TFA_2O (3.5 ml, 25 mmol) was added at 0 °C. The reaction was slowly warmed to room temperature and stirred overnight. The reaction was quenched with Methanol and concentrated *in vacuo*. The product was purified by silica gel column chromatography (pentane:EtOAc = 50:1 – 10:1). Compound **S2** (9.58 g, 89% yield) was obtained as yellow syrup. $[\alpha]_D^{25}$ +205.4 (c=1, $CHCl_3$). ^{1}H NMR (400 MHz, $CDCl_3$) δ 7.55 – 7.46 (m, 2H), 7.44 – 7.21 (m, 8H, *aromatic* H), 6.59 (d, *J* = 7.0 Hz, 1H, NH), 6.12 (d, *J* = 4.8 Hz, 1H, H-1), 4.83 – 4.73 (m, 2H, H-2, *PhC*H*H*O), 4.71 (d, *J* = 2.7 Hz, 1H, H-5), 4.50 (d, *J* = 11.7 Hz, 1H, *PhC*H*H*O), 4.33 (dd, *J* = 12.7, 2.3 Hz, 1H, H-6), 4.17 (dd, *J* = 12.7, 1.7 Hz, 1H, H-6), 4.05 (d, *J* = 2.3 Hz, 1H, H-4), 3.51 (dd, *J* = 11.0, 2.7 Hz, 1H, H-3), 1.07 (d, *J* = 4.2 Hz, 18H, C*H*$_3$). ^{13}C NMR (100 MHz, $CDCl_3$) δ 157.26 (*a*d, *J* = 37 Hz, *CF*$_3$*CO*), 149.79, 137.33, 136.16, 134.43, 134.38, 134.33, 129.46, 128.85, 128.59, 128.37, 128.32, 128.24, 127.95, 127.89, 123.86 (*aromatic* C/C*H*), 115.68 (*a*d, *J* = 286 Hz, C*F*$_3$), 88.66 (C-1), 76.06 (C-3), 70.90 (C-4), 69.65 (C*H*$_2$*Ph*), 68.82 (C-5), 67.23 (C-6), 49.95 (C-2), 27.72, 27.37 (2 C*H*$_3$), 23.50, 20.87 (2 C-*Si*). HR-MS: Calculated for $C_{29}H_{38}NO_5F_3SiSe$ $[M+NH_4]^+$: 663.19749, found: 663.19784.

3-*O*-benzyl-2-deoxy-4,6-di-*tert*-butylsilylidene-2-trifluoroacetamido-1-*O*-(*N*-phenyl-trifluoroacetimidoyl)-α/β-D-galactopyranoside (14)

NIS (944 mg, 4.19 mmol) was added to the solution of compound **S2** (1.65 g, 2.8 mmol) in Acetone/H_2O (15 ml/3 ml) at 0 °C. The reaction was slowly warmed to room temperature and stirred until TLC-analysis indicated full consumption of the starting material (± 1H). Then the mixture was diluted with DCM and washed with saturated $Na_2S_2O_3$ and brine, dried with anhydrous $MgSO_4$, filtered and concentrated *in vacuo*. The product was purified by silica gel column chromatography (pentane:EtOAc = 4:1). Cs_2CO_3 (2.77 g, 8.5 mmol) was added to the solution of the hemiacetal (4.3 g, 8.5 mmol) in 45 ml acetone. The mixture was stirred at 0 °C for 15 minutes. Then CF_3C(=NPh)Cl (2.29 g, 11.06 mmol) was added to the solution. which was slowly warmed to room temperature and stirred overnight. The reaction was quenched with Et_3N and concentrated *in vacuo*. The product **14** was purified by silica gel column chromatography (pentane:Et_2O = 50:1 – 10:1). Compound **14** (5.15 g, α:β = 7:1, 90% yield) was obtained as syrup. α-Isomer: ^{1}H NMR (400 MHz, $CDCl_3$) δ 7.45 – 7.22 (m, 7H), 7.10 (t, *J* = 7.4 Hz, 1H), 6.76 (d, *J* = 7.7

Hz, 2H, *aromatic* H), 6.57 (bs, H-1), 6.11 (d, *J* = 7.3 Hz, 1H, *N*H), 4.80 (d, *J* = 11.8 Hz, 1H, *PhCHH*O), 4.72 (s, 2H, H-2, 5), 4.52 (d, *J* = 11.8 Hz, 1H, *PhCHH*O), 4.33 – 4.12 (m, 2H, H-6), 3.82 – 3.66 (m, 2H, H-3, 4), 1.14 – 0.97 (m, 18H, *C*H$_3$). ^{13}C NMR (100 MHz, CDCl$_3$) δ 157.53 (*a*d, *J* = 37 Hz, *CF$_3$CO*), 143.09, 137.25, 128.90, 128.44, 128.01, 119.33 (*aromatic* C/C*H*), 115.72(*a*d, *J* = 286 Hz, C*F$_3$*), 96.81 (C-1), 73.91 (C-3), 70.16 (C-4), 69.96 (C*H$_2$Ph*), 68.77 (C-5), 66.87 (C-6), 48.40 (C-2), 27.72, 27.30 (2 C*H$_3$*), 23.51, 20.85 (2 C-*Si*). β-Isomer: ^{1}H NMR (400 MHz, CDCl$_3$) δ 7.48 – 7.27 (m, 7H), 7.20 – 7.08 (m, 1H), 6.84 (d, *J* = 7.7 Hz, 2H, *aromatic* H), 6.56 (d, *J* = 7.2 Hz, 1H, NH), 6.19 (bs, 1H, H-1), 4.73 (d, *J* = 11.6 Hz, 1H, *PhCHHO*), 4.64 – 4.47 (m, 2H, *PhCHHO*, H-5), 4.42 – 3.90 (m, 5H, H-2, 3, 4, 6), 1.20 – 1.03 (m, 18H, *C*H$_3$). ^{13}C NMR (100 MHz, CDCl$_3$) δ 157.68 (*a*d, *J* = 37 Hz, *CF$_3$CO*), 143.28, 137.42, 128.85, 128.78, 128.33, 128.05, 124.57, 119.38 (*aromatic* C/C*H*), 115.60 (*a*d, *J* = 286 Hz, C*F$_3$*), 93.70 (C-1), 75.46 (C-3), 72.36 (C-4), 70.61 (C*H$_2$Ph*), 68.61 (C-5), 66.90 (C-6), 52.93 (C-2), 27.74, 27.44 (2 C*H$_3$*), 23.54, 20.91 (2 C-*Si*). HR-MS: Calculated for $C_{31}H_{38}N_2O_6F_6Si$ [M+Na]$^+$: 699.22955, found: 699.22946.

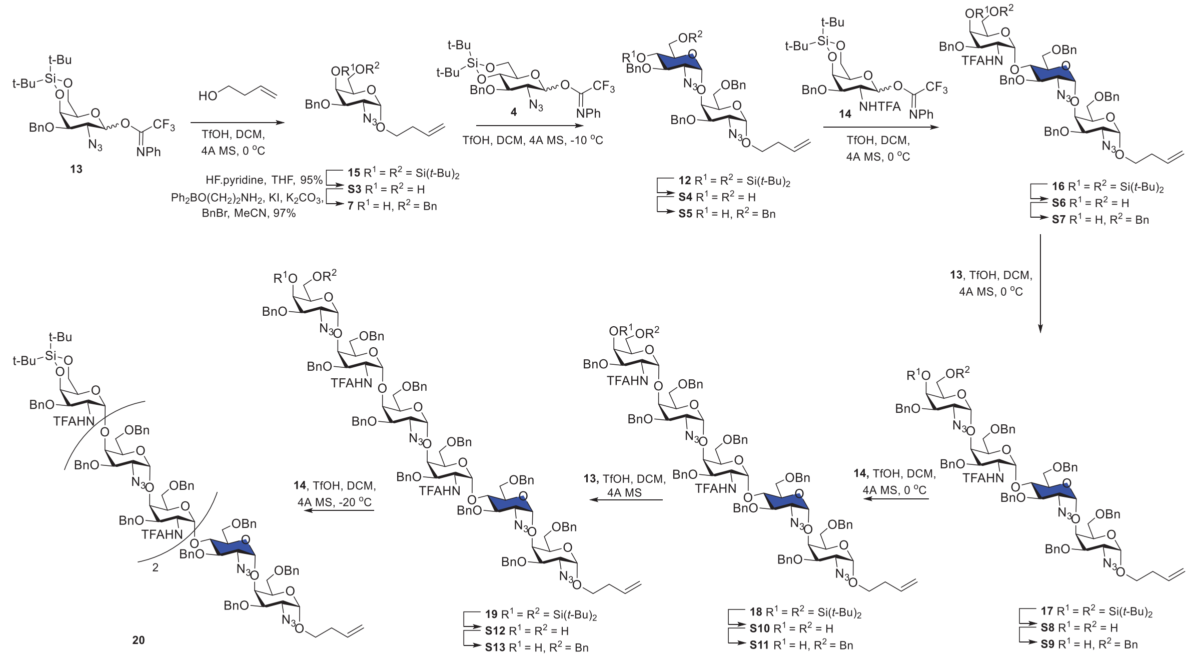

3-Butenyl 2-azido-3-*O*-benzyl-2-deoxy-4,6-di-*tert*-butylsilylidene-α-D-galactopyranoside (15)

The reaction was carried out according to the general procedure A. The donor **13**[3] (1.8 g, 2.97 mmol) was co-evaporated with toluene (three times). The the linker alcohol (511 ul, 5.94 mmol) was added, the mixture was dissolved in dry 30 ml DCM under nitrogen and stirred over fresh flame-dried molecular sieves 4Å. The solution was cooled to 0 °C, after which TfOH (26 µl, 0.23 mmol) was added. The reaction was stirred at 0 °C for 2 h. Then the reaction was quenched with Et_3N, diluted with DCM, washed with saturated $NaHCO_3$ and brine. The organic phase was dried with anhydrous $MgSO_4$, filtered and concentrated *in vacuo*. The product was purified by silica gel column chromatography (pentane:EtOAc = 50:1). Compound **15** (1.30 g, α-only, 89% yield) was obtained as colorless syrup. $[\alpha]_D^{25}$ +146.7 (c=2, CHCl$_3$). ^{1}H NMR (400 MHz, CDCl$_3$) δ 7.48 – 7.22 (m, 5H, *aromatic* H), 5.86 – 5.71 (m, 1H, H-9), 5.13 – 5.00 (m, 2H, H-10), 4.93 (d, *J* = 3.5 Hz, 1H, H-1), 4.74 (d, *J* = 11.5 Hz, 1H, *PhCHHO*), 4.65 (d, *J* = 11.5 Hz, 1H, *PhCHHO*), 4.57 (dd, *J* = 2.9, 1.0 Hz, 1H, H-4), 4.24 (dd, *J* = 12.6, 2.1 Hz, 1H, H-6), 4.13 (dd, *J* = 12.5, 1.7 Hz, 1H, H-6), 3.87 (dd, *J* = 10.6, 2.8 Hz, 1H, H-3), 3.77 (dd, *J* = 10.6, 3.5 Hz, 1H, H-2), 3.73 – 3.62 (m, 2H, H-7, 5), 3.59 – 3.48 (m, 1H, H-7), 2.41 – 2.30 (m, 2H, H-8), 1.07 (s, 9H, *C*H$_3$), 1.04 (s, 9H, *C*H$_3$). ^{13}C NMR (100 MHz, CDCl$_3$) δ 137.85 (*aromatic* C), 134.66 (C-9), 128.49, 127.91, 127.83 (*aromatic* C*H*), 116.89 (C-10), 98.40 (C-1), 75.36 (C-3), 70.40 (C*H$_2$Ph*), 69.82 (C-4), 67.67 (C-7), 67.46 (C-5), 67.17 (C-6), 58.23 (C-2), 33.90 (C-8), 27.66, 27.34 (2 C*H$_3$*), 23.42, 20.73 (2 C-*Si*). HR-MS: Calculated for $C_{25}H_{39}N_3O_5Si$ [M+Na]$^+$: 512.25512, found: 512.25511.

3-Butenyl 2-azido-3-*O*-benzyl-2-deoxy-α-D-galactopyranoside (S3)

The reaction was carried out according to the general procedure C using compound **15** (1.27 g, 2.59 mmol) and HF/pyridine (70%, 1.1 ml, 41.5 mmol). The product was purified by column chromatography (pentane:EtOAc = 3:2). Compound **S3** (860 mg, 95% yield) was obtained as white solid. $[\alpha]_D^{25}$ +135.4 (c=1, CHCl$_3$). ^{1}H NMR (400 MHz, Chloroform-*d*) δ 7.47 – 7.26 (m, 5H, *aromatic* H), 5.80 (ddt, *J* = 17.0, 10.2, 6.7 Hz, 1H, H-9), 5.18 – 5.01 (m, 2H, H-10), 4.93 (d, *J* = 3.5 Hz, 1H, H-1), 4.77 – 4.61 (m, 2H, 2x*PhCHHO*), 4.10 (d, *J* = 3.1 Hz, 1H, H-4), 3.94 – 3.83

(m, 2H, H-3, 6), 3.83 – 3.68 (m, 3H, H-5, 6, 7), 3.65 (dd, *J* = 10.4, 3.6 Hz, 1H, H-2), 3.52 (dt, *J* = 9.7, 6.6 Hz, 1H, H-7), 2.97 (bs, 1H, OH), 2.71 (bs, 1H, OH), 2.37 (qt, *J* = 6.8, 1.4 Hz, 2H, H-8). ^{13}C NMR (100 MHz, $CDCl_3$) δ 137.12 (*aromatic* C), 134.62 (C-9), 128.73, 128.34, 128.11 (*aromatic* C*H*), 117.03 (C-10), 98.07 (C-1), 75.81 (C-3), 72.02 (C*H₂Ph*), 69.50 (C-5), 67.68 (C-7), 67.50 (C-4), 62.80 (C-6), 58.97 (C-2), 33.87 (C-8). HR-MS: Calculated for $C_{17}H_{23}N_3O_5$ $[M+Na]^+$: 372.15299, found: 372.15306.

3-Butenyl 2-azido-3,6-di-*O*-benzyl-2-deoxy-α-D-galactopyranoside (7)

The reaction was carried out according to the general procedure D using compound **S3** (900 mg, 2.58 mmol), K_2CO_3 (392 mg, 2.84 mmol), KI (428 mg, 2.58 mmol) and $Ph_2BO(CH_2)_2NH_2$ (58 mg, 0.26 mmol). The product was purified by column chromatography (pentane:EtOAc = 7:1). Compound **7** (1.07 g, 94% yield) was obtained as yellow syrup. $[\alpha]_D^{25}$ +75 (c=2, $CHCl_3$). ^{1}H NMR (400 MHz, $CDCl_3$) δ 7.43 – 7.26 (m, 10H, *aromatic* H), 5.80 (ddt, *J* = 17.1, 10.2, 6.7 Hz, 1H, H-9), 5.14 – 5.01 (m, 2H, H-10), 4.91 (d, *J* = 3.6 Hz, 1H, H-1), 4.66 (s, 2H, *PhCHHO*), 4.58 (d, *J* = 11.9 Hz, 1H, *PhCHHO*), 4.56 (d, 1H, *PhCHHO*), 4.10 (dd, *J* = 3.2, 1.3 Hz, 1H, H-4), 3.93 (td, *J* = 5.8, 1.3 Hz, 1H, H-5), 3.87 (dd, *J* = 10.4, 3.1 Hz, 1H, H-3), 3.79 – 3.64 (m, 4H, H-2, 6, 7), 3.52 (dt, *J* = 9.7, 6.5 Hz, 1H, H-7), 2.73 (bs, 1H, OH), 2.37 (qt, *J* = 6.8, 1.4 Hz, 2H, H-8). ^{13}C NMR (100 MHz, $CDCl_3$) δ 137.87, 137.24 (*aromatic* C), 134.67 (C-9), 128.97, 128.61, 128.42, 128.15, 128.01, 127.75, 127.66 (*aromatic* C*H*), 116.87 (C-10), 98.00 (C-1), 75.97 (C-3), 73.60, 71.77 (2 C*H₂Ph*), 69.43 (C-6), 68.80 (C-5), 67.58 (C-7), 66.55 (C-4), 58.97 (C-2), 33.84 (C-8). HR-MS: Calculated for $C_{24}H_{29}N_3O_5$ $[M+Na]^+$: 462.19994, found: 462.19997.

3-Butenyl 2-azido-3-*O*-benzyl-2-deoxy-4,6-di-*tert*-butylsilylidene-α-D-glucopyranosyl-(1→4)-2-azido-3,6-di-*O*-benzyl-2-deoxy-α-D-galacto-pyranoside (12)

The reaction was carried out according to the general procedure A. The donor **4** (808 mg, 1.33 mmol) and acceptor **7** (293 mg, 0.67 mmol) were co-evaporated with toluene (three times). The residue was dissolved in dry 6 ml DCM under nitrogen and stirred over fresh flame-dried molecular sieves 4Å. The solution was cooled to -10 ºC, after which TfOH (12 µl, 0.13 mmol) was added. The reaction was stirred at -10 ºC for 2 h. Then the reaction was quenched with Et_3N, diluted with DCM, washed with saturated $NaHCO_3$ and brine. The organic phase was dried with anhydrous $MgSO_4$, filtered and concentrated *in vacuo*. The product was purified by silica gel column chromatography (pentane:Et_2O = 10:1). Compound **12** (422 mg, α-only, 74% yield) was obtained as yellow syrup. $[\alpha]_D^{25}$ +121.5 (c=1, $CHCl_3$). ^{1}H NMR (400 MHz, $CDCl_3$) δ 7.58 – 7.33 (m, 15H, *aromatic* H), 5.87 (ddt, *J* = 17.1, 10.2, 6.8 Hz, 1H, H-9), 5.23 – 5.10 (m, 3H, H-10), 5.04 (d, *J* = 3.5 Hz, 1H, H-1^A), 4.92 (d, *J* = 5.4 Hz, 1H, *PhCHHO*), 4.91 – 4.87 (m, 2H, H-1^B), 4.76 – 4.63 (m, 2H, *PhCHHO*), 4.58 (d, *J* = 11.8 Hz, 1H, *PhCHHO*), 4.43 (td, *J* = 9.7, 4.7 Hz, 1H), 4.27 (d, *J* = 2.7 Hz, 1H, H-4^A), 4.12 – 4.03 (m, 1H), 4.03 – 3.88 (m, 5H), 3.86 – 3.71 (m, 3H), 3.67 – 3.56 (m, 2H), 3.37 – 3.27 (m, 1H, H-2^B), 2.43 (qt, *J* = 6.8, 1.4 Hz, 2H, H-8), 1.15 (s, 9H, C*H₃*), 1.08 (s, 9H, C*H₃*). ^{13}C NMR (100 MHz, $CDCl_3$) δ 138.13, 137.38, 137.26 (*aromatic* C), 134.60 (C-9), 128.50, 128.38, 128.35, 128.32, 128.21, 128.10, 127.88, 127.82, 127.63 (*aromatic* C*H*), 116.88 (C-10), 98.64 (C-1^B), 97.85 (C-1^A), 79.26, 79.17, 75.46, 75.37, 73.59, 73.51, 71.97, 69.02, 67.56, 66.76, 66.74, 66.64, 62.92 (C-2^B), 60.01 (C-2^A), 33.83 (C-8), 27.35, 27.04 (2 C*H₃*), 22.55, 19.98 (2 C-*Si*). ^{13}C-HMBC ($CDCl_3$, 100 MHz): 98.64 ($J_{C1,H1}$ = 172 Hz), 97.85 ($J_{C1,H1}$ = 171 Hz). HR-MS: Calculated for $C_{45}H_{60}N_6O_9Si$ $[M+Na]^+$: 879.40833, found: 879.40858.

3-Butenyl 2-azido-3-*O*-benzyl-2-deoxy-α-D-glucopyranosyl-(1→4)-2-azido-3,6-di-*O*-benzyl-2-deoxy-α-D-galactopyranoside (S4)

The reaction was carried out according to the general procedure C using compound **12** (422 mg, 0.49 mmol) and HF/pyridine (70%, 205 µl, 7.88 mmol). The product was purified by column chromatography (pentane:EtOAc = 3:1). Compound **S4** (342 mg, 97% yield) was obtained as white solid. $[\alpha]_D^{25}$ +146.8 (c=1, $CHCl_3$). ^{1}H NMR (400 MHz, $CDCl_3$) δ 7.43 – 7.20 (m, 15H, *aromatic* H), 5.78 (ddt, *J* = 17.0, 10.2, 6.8 Hz, 1H, H-9), 5.11 – 5.00 (m, 2H, H-10), 4.93 (d, *J* = 3.5 Hz, 1H, H-1^A), 4.89 – 4.77 (m, 3H, H-1^B, *PhCHHO*), 4.72 (d, *J* = 12.0 Hz, 1H, *PhCHHO*), 4.61 – 4.46 (m, 3H), 4.21 (d, *J* = 2.7 Hz, 1H, H-4^A), 3.99 – 3.76 (m, 5H), 3.73 – 3.46 (m, 5H), 3.35 (dd, *J* = 12.2, 2.7 Hz, 1H), 3.19 (td, *J* = 11.1, 10.3, 3.5 Hz, 2H), 2.41 – 2.26 (m, 2H, H-8). ^{13}C NMR (101 MHz, $CDCl_3$) δ 138.04, 137.34, 137.26 (*aromatic* C), 134.56 (C-9), 128.42, 128.36, 127.99, 127.97, 127.90, 127.77, 127.73, 127.20 (*aromatic* C*H*), 116.80 (C-10), 98.58 (C-1^B), 98.01 (C-1^A), 79.59, 75.17, 75.00, 73.42, 73.00, 71.57, 71.32, 70.88, 69.01, 67.56, 66.88, 63.35, 61.24, 59.31, 33.77 (C-8). HR-MS: Calculated for $C_{37}H_{44}N_6O_9$ $[M+Na]^+$: 739.30620, found: 739.30573.

3-Butenyl 2-azido-3,6-di-*O*-benzyl-2-deoxy-α-D-glucopyranosyl-(1→4)-2-azido-3,6-di-*O*-benzyl-2-deoxy-α-D-galactopyranoside (S5)

The reaction was carried out according to the general procedure D using compound **S4** (337 mg, 0.47 mmol), K_2CO_3 (71 mg, 0.52 mmol), KI (78 mg, 0.47 mmol) and $Ph_2BO(CH_2)_2NH_2$ (11 mg, 0.047 mmol). The product was purified by column chromatography (pentane:EtOAc = 10:1).

Compound **S5** (363 g, 96% yield) was obtained as colorless syrup. $[\alpha]_D^{25}$ +135.8 (c=2, $CHCl_3$). ^1H NMR (400 MHz, $CDCl_3$) δ 7.58 – 7.28 (m, 20H, *aromatic* H), 5.93 (ddt, *J* = 17.1, 10.2, 6.7 Hz, 1H, H-9), 5.27 – 5.15 (m, 2H, H-10), 5.10 (d, *J* = 3.6 Hz, 1H, H-1^A), 5.06 (d, *J* = 3.7 Hz, 1H, H-1^B), 5.05 – 4.95 (m, 2H, *PhCHHO*), 4.90 (d, *J* = 12.0 Hz, 1H, *PhCHHO*), 4.74 – 4.62 (m, 3H), 4.44 – 4.35 (m, 2H), 4.33 – 4.20 (m, 2H), 4.17 – 4.05 (m, 2H), 4.04 – 3.79 (m, 5H), 3.75 – 3.61 (m, 3H), 3.40 (dd, *J* = 10.1, 3.6 Hz, 1H), 3.35 (dd, *J* = 10.3, 3.2 Hz, 1H), 3.24 (dd, *J* = 10.4, 4.4 Hz, 1H, H-6^B), 2.84 (bs, 1H, OH), 2.54 – 2.45 (m, 2H, H-8). ^{13}C NMR (100 MHz, $CDCl_3$) δ 138.13, 137.64, 137.49, 137.39 (*aromatic* C), 134.59 (C-9), 128.88, 128.44, 128.39, 128.35, 128.33, 128.26, 128.02, 127.98, 127.95, 127.75, 127.62, 127.56, 127.14, 127.07 (*aromatic* C*H*), 116.82 (C-10), 98.74 (C-1^B), 98.07 (C-1^A), 79.59 (C-3^B), 75.53 (C-3^A), 74.99, 73.46, 73.25 (3 C*H₂Ph*), 73.20 (C-4^A), 72.45 (C-4^B), 71.66 (C*H₂Ph*), 69.96 (C-5^B), 69.09 (C-$6^{B)}$, 69.07 (C-5^A), 67.60 (C-7), 66.85 (C-6^A), 63.27 (C-2^B), 59.42 (C-2^A), 33.81 (C-8). HR-MS: Calculated for $C_{44}H_{50}N_6O_9$ $[M+Na]^+$: 829.35315, found: 829.35326.

3-Butenyl 3-*O*-benzyl-2-deoxy-4,6-di-*tert*-butylsilylidene-2-trifluoroacetamido-α-D-galactopyranosyl-(1→4)-2-azido-3,6-di-*O*-benzyl-2-deoxy-α-D-glucopyranosyl-(1→4)-2-azido-3,6-di-*O*-benzyl-2-deoxy-α-D-galactopyranoside (16)

The reaction was carried out according to the general procedure A. The donor **14** (559 mg, 0.83 mmol) and acceptor **S5** (370 mg, 0.46 mmol) were co-evaporated with toluene (three times). The residue was dissolved in dry 4.5 ml DCM under nitrogen and stirred over fresh flame-dried molecular sieves 4Å. The solution was cooled to 0 °C, after which TfOH (7.5 µl, 0.083 mmol) was added. The reaction was stirred at 0 °C for 2 h. Then the reaction was quenched with Et_3N, diluted with DCM, washed with saturated $NaHCO_3$ and brine. The organic phase was dried with anhydrous $MgSO_4$, filtered and concentrated *in vacuo*. The product was purified by silica gel column chromatography (pentane:EtOAc = 8:1). Compound **16** (506 mg, α-only, 85% yield) was obtained as yellow syrup. $[\alpha]_D^{25}$ +117.9 (c=1, $CHCl_3$). ^1H NMR (400 MHz, $CDCl_3$) δ 7.42 – 7.22 (m, 23H, *aromatic* H), 7.18 – 7.12 (m, 2H, *aromatic* H), 6.98 (d, *J* = 9.5 Hz, 1H, NH), 5.89 – 5.72 (m, 1H, H-9), 5.45 (d, *J* = 3.5 Hz, 1H, H-1^C), 5.14 – 5.02 (m, 2H, H-10), 5.01 – 4.97 (m, 2H, H-1^A, 1^B), 4.82 – 4.42 (m, 10H), 4.28 (dd, *J* = 18.5, 3.6 Hz, 3H), 4.04 – 3.79 (m, 8H), 3.71 (dt, *J* = 9.8, 6.8 Hz, 1H, H-7), 3.62 (dd, *J* = 10.9, 3.5 Hz, 1H, H-2^A), 3.60 – 3.51 (m, 3H), 3.47 (dd, *J* = 10.9, 2.6 Hz, 1H), 3.31 (dd, *J* = 10.0, 3.6 Hz, 1H, H-2^B), 3.20 (dd, *J* = 11.5, 2.3 Hz, 1H, H-6), 3.08 (dd, *J* = 11.4, 1.8 Hz, 1H, H-6), 2.37 (qt, *J* = 6.7, 1.4 Hz, 2H, H-8), 1.03 (s, 9H, *C*H_3), 1.02 (s, 9H, *C*H_3). ^{13}C NMR (100 MHz, $CDCl_3$) δ 157.38 (*a*d, *J* = 37 Hz, *CF₃CO*), 138.07, 137.59, 137.45, 137.38, 136.35 (*aromatic* C), 134.66 (C-9), 128.67, 128.65, 128.53, 128.51, 128.47, 128.33, 128.24, 128.17, 127.94, 127.91, 127.80, 127.77, 127.45, 127.25 (*aromatic* C*H*), 117.04 (C-10), 115.89 (*a*d, *J* = 287 Hz, C*F₃*), 98.53 (C-1^B), 98.22 (C-1^A), 97.45 (C-1^C), 79.79, 75.58, 75.11, 74.69, 73.70, 73.53 (3 C*H₂Ph*), 73.21, 72.94, 72.03, 70.86, 69.74, 69.58, 69.13, 68.59, 67.94 (C-6), 67.86 (C-7), 67.12 (C-6), 66.89 (C-6), 64.39 (C-2^B), 59.72 (C-2^A), 48.67 (C-2^C), 33.96 (C-8), 27.67, 27.36 (2 C*H₃*), 23.41, 20.81 (2 C-*Si*). HR-MS: Calculated for $C_{67}H_{82}N_7O_{14}F_3Si$ $[M+Na]^+$: 1316.55333, found: 1316.55366.

3-Butenyl 3-*O*-benzyl-2-deoxy-2-trifluoroacetamido-α-D-galactopyranosyl-(1→4)-2-azido-3,6-di-*O*-benzyl-2-deoxy-α-D-glucopyranosyl-(1→4)-2-azido-3,6-di-*O*-benzyl-2-deoxy-α-D-galactopyranoside (S6)

The reaction was carried out according to the general procedure C using compound **16** (417 g, 0.33 mmol) and HF/pyridine (70%, 134 µl, 5.15 mmol). The product was purified by column chromatography (pentane:EtOAc = 3:2). Compound **S6** (341 mg, 92% yield) was obtained as yellow solid. $[\alpha]_D^{25}$ +132.2 (c=1, $CHCl_3$).^1H NMR (400 MHz, $CDCl_3$) δ 7.42 – 7.16 (m, 25H, *aromatic* H), 7.08 (d, *J* = 9.9 Hz, 1H, NH), 5.79 (ddt, *J* = 17.0, 10.2, 6.7 Hz, 1H, H-9), 5.25 (d, *J* = 3.6 Hz, 1H, H-1^C), 5.14 – 5.02 (m, 2H, H-10), 5.01 – 4.95 (m, 2H, H-1^A, 1^B), 4.75 (dd, *J* = 14.9, 11.5 Hz, 2H), 4.62 – 4.46 (m, 6H), 4.40 – 4.23 (m, 4H), 4.06 (d, *J* = 2.9 Hz, 1H), 4.03 – 3.93 (m, 2H), 3.93 – 3.80 (m, 4H), 3.76 – 3.50 (m, 7H), 3.44 (dd, *J* = 10.7, 2.9 Hz, 1H), 3.32 – 3.21 (m, 2H), 3.10 (dd, *J* = 11.6, 2.1 Hz, 1H), 2.94 (s, 1H, OH), 2.43 – 2.30 (m, 2H, H-8). ^{13}C NMR (100 MHz, $CDCl_3$) δ 157.38 (*a*d, *J* = 37 Hz, *CF₃CO*), 137.55, 137.34, 137.09, 136.49 (*aromatic* C), 134.63 (C-9), 128.60, 128.52, 128.43, 128.23, 128.17, 128.12, 128.09, 127.98, 127.89, 127.88, 127.83, 127.43, 127.24 (*aromatic* C*H*), 116.98 (C-10), 115.89 (*a*d, *J* = 286 Hz, C*F₃*), 98.45 (C-1^B), 98.21 (C-1^A), 97.76 (C-1^C), 79.64, 75.81, 75.39, 74.50, 74.38, 73.62, 73.47, 73.04, 71.98, 70.96, 70.92, 70.60, 69.07, 67.79 (C-7), 67.48 (C-6), 66.90 (C-6), 66.22, 64.23 (C-2), 62.52 (C-6), 59.56 (C-2), 49.25 (C-2^C), 33.90 (C-8). HR-MS: Calculated for $C_{59}H_{66}N_7O_{14}F_3$ $[M+Na]^+$: 1176.45121, found: 1176.45119.

3-Butenyl 3,6-di-*O*-benzyl-2-deoxy-2-trifluoroacetamido-α-D-galactopyranosyl-(1→4)-2-azido-3,6-di-*O*-benzyl-2-deoxy-α-D-glucopyranosyl-(1→4)-2-azido-3,6-di-*O*-benzyl-2-deoxy-α-D-galactopyranoside (S7)

The reaction was carried out according to the general procedure D using compound **S6** (286 mg, 0.25 mmol), K_2CO_3 (38 mg, 0.27 mmol), KI (41 mg, 0.25 mmol) and $Ph_2BO(CH_2)_2NH_2$ (5.6 mg, 0.025 mmol). The product was purified by column chromatography (pentane:EtOAc = 4:1).

Compound **S7** (279 mg, 90% yield) was obtained as yellow syrup. $[\alpha]_D^{25}$ +118 (c=0.6, $CHCl_3$). ^{1}H NMR (400 MHz, $CDCl_3$) δ 7.48 – 7.13 (m, 30H, *aromatic* H), 7.09 (d, *J* = 9.9 Hz, 1H, NH), 5.78 (ddt, *J* = 17.0, 10.2, 6.7 Hz, 1H, H-9), 5.25 (d, *J* = 3.6 Hz, 1H, H-1^C), 5.13 – 5.00 (m, 2H, H-10), 5.00 – 4.96 (m, 2H, H-1^A, 1^B), 4.81 – 4.66 (m, 2H), 4.63 – 4.21 (m, 13H), 4.17 – 3.79 (m, 9H), 3.75 – 3.42 (m, 8H), 3.40 – 3.23 (m, 2H), 3.14 (dd, *J* = 11.3, 2.1 Hz, 1H), 2.62 (s, *O*H), 2.35 (qt, *J* = 6.7, 1.4 Hz, 2H, H-8). ^{13}C NMR (100 MHz, $CDCl_3$) δ 157.34 (*a*d, *J* = 37 Hz, *CF_3CO*), 137.90, 137.74, 137.35, 137.32, 137.23, 136.51 (*aromatic* C), 134.63 (C-9), 128.58, 128.54, 128.48, 128.43, 128.36, 128.34, 128.22, 128.14, 128.07, 128.03, 127.94, 127.90, 127.86, 127.82, 127.81, 127.76, 127.72, 127.69, 127.39, 127.33 (*aromatic* C*H*), 116.96 (*a*d, *J* = 286 Hz, C*F_3*), 98.49 (C-1^B), 98.18 (C-1^A), 97.91 (C-$^{1C)}$, 79.59, 76.01, 75.13, 74.70, 74.53, 73.70, 73.60, 73.17, 73.03, 71.84, 70.90, 70.87, 69.63, 69.06, 68.81, 67.75, 67.69, 66.93, 65.22, 64.27 (C-2), 59.55 (C-2), 49.40 (C-2^C), 33.89 (C-8). HR-MS: Calculated for $C_{66}H_{72}N_7O_{14}F_3$ $[M+Na]^+$: 1266.49816, found: 1266.49839.

3-Butenyl 2-azido-3-*O*-benzyl-2-deoxy-4,6-di-*tert*-butylsilylidene-α-D-galactopyranosyl-(1→4)-3,6-di-*O*-benzyl-2-deoxy-2-trifluoroacetamido-α-D-galactopyranosyl-(1→4)-2-azido-3,6-di-*O*-benzyl-2-deoxy-α-D-glucopyranosyl-(1→4)-2-azido-3,6-di-*O*-benzyl-2-deoxy-α-D-galactopyranoside (17)

The reaction was carried out according to the general procedure A. The donor **13** (535 mg, 0.88 mmol) and acceptor **S7** (366 mg, 0.29 mmol) were co-evaporated with toluene (three times). The residue was dissolved in dry 3 ml DCM under nitrogen and stirred over fresh flame-dried molecular sieves 4Å. The solution was cooled to 0 °C, after which TfOH (8 µl, 0.088 mmol) was added. The reaction was stirred at 0 °C for 2 h. Then the reaction was quenched with Et_3N, diluted with DCM, washed with saturated $NaHCO_3$ and brine. The organic phase was dried with anhydrous $MgSO_4$, filtered and concentrated *in vacuo*. The product was purified by silica gel column chromatography (pentane:EtOAc = 6:1). Compound **17** (343 mg, α-only, 70% yield) was obtained as yellow solid. $[\alpha]_D^{25}$ +159.3 (c=1, $CHCl_3$). ^{1}H NMR (400 MHz, $CDCl_3$) δ 7.47 – 7.12 (m, 35H, *aromatic* H), 7.05 (d, *J* = 10.0 Hz, 1H, NH), 5.79 (ddt, *J* = 17.0, 10.2, 6.8 Hz, 1H, H-9), 5.36 (d, *J* = 3.6 Hz, 1H, H-1C), 5.13 – 5.02 (m, 2H, H-10), 5.01 – 4.97 (m, 2H, 2xH-1), 4.94 (d, *J* = 3.6 Hz, 1H, H-1), 4.81 – 4.52 (m, 10H), 4.50 (d, *J* = 2.9 Hz, 1H), 4.45 – 4.36 (m, 2H), 4.35 – 4.21 (m, 5H), 4.10 – 3.79 (m, 11H), 3.77 – 3.45 (m, 8H), 3.38 (dd, *J* = 8.3, 5.0 Hz, 1H), 3.34 – 3.25 (m, 2H), 3.14 (dd, *J* = 11.5, 1.9 Hz, 1H), 2.41 – 2.32 (m, 2H, H-8), 1.02 (s, 9H, *C*H_3), 0.96 (s, 9H, *C*H_3). ^{13}C NMR (100 MHz, $CDCl_3$) δ 157.52 (*a*d, *J* = 37 Hz, *CF_3*CO), 137.94, 137.39, 137.35, 137.29, 137.23, 136.42 (*aromatic* C), 134.66 (C-9), 128.65, 128.63, 128.60, 128.55, 128.49, 128.46, 128.30, 128.22, 128.17, 128.14, 127.99, 127.79, 127.76, 127.68, 127.64, 127.33, 126.21, 124.43, 123.56 (*aromatic* C*H*), 117.04 (C-10), 115.94 (*a*d, *J* = 286 Hz, C*F_3*), 98.85 (C-1), 98.58 (C-1), 98.28 (C-1), 97.75 (C-1C), 79.78, 76.20, 75.66, 75.39, 74.55, 73.91, 73.69, 73.30, 73.06, 72.00, 71.03, 70.91, 70.64, 70.61, 70.31, 69.61, 69.11, 67.85, 67.57, 67.08, 66.94, 66.63, 64.32, 59.58, 58.51, 49.73 (4 C-2), 33.96 (C-8), 27.64, 27.47 (2 C*H_3*), 23.31, 20.77 (2 C-*Si*). ^{13}C-HMBC ($CDCl_3$, 100 MHz): 98.85 ($J_{C1,H1}$ = 174 Hz), 98.58 ($J_{C1,H1}$ = 171 Hz), 97.75 ($J_{C1,H1}$ = 171 Hz). HR-MS: Calculated for $C_{87}H_{103}N_{10}O_{18}F_3Si$ $[M+Na]^+$: 1683.70654, found: 1683.70758.

3-Butenyl 2-azido-3-*O*-benzyl-2-deoxy-α-D-galactopyranosyl-(1→4)-3,6-di-*O*-benzyl-2-deoxy-2-trifluoroacetamido-α-D-galactopyranosyl -(1→4)-2-azido-3,6-di-*O*-benzyl-2-deoxy-α-D-glucopyranosyl-(1→4)-2-azido-3,6-di-*O*-benzyl-2-deoxy-α-D-galactopyranoside (S8)

The reaction was carried out according to the general procedure C using compound **17** (112 mg, 0.067 mmol) and HF/pyridine (70%, 28 µl, 1.1 mmol). The product was purified by column chromatography (pentane:EtOAc = 2:1). Compound **S8** (89 mg, 87% yield) was obtained as yellow syrup. $[\alpha]_D^{25}$ +133.7 (c=0.7, $CHCl_3$). ^{1}H NMR (500 MHz, $CDCl_3$) δ 7.63 – 7.27 (m, 35H, *aromatic* H), 7.23 (d, *J* = 9.9 Hz, 1H, NH), 5.89 (ddt, *J* = 17.0, 10.2, 6.8 Hz, 1H, H-9), 5.44 (d, *J* = 3.6 Hz, 1H, H-1^C), 5.23 – 5.12 (m, 2H, H-10), 5.12 – 5.08 (m, 2H, 2xH-1), 5.06 (d, *J* = 3.6 Hz, 1H, H-1), 4.88 (d, *J* = 11.1 Hz, 1H), 4.81 (dd, *J* = 12.1, 4.0 Hz, 2H), 4.76 (s, 2H), 4.75 – 4.69 (m, 1H), 4.68 – 4.59 (m, 4H), 4.53 – 4.44 (m, 3H), 4.43 – 4.37 (m, 2H), 4.37 – 4.30 (m, 2H), 4.30 – 4.24 (m, 1H), 4.24 – 4.18 (m, 1H), 4.15 – 3.92 (m, 9H), 3.85 – 3.75 (m, 2H), 3.71 (dd, *J* = 10.9, 3.5 Hz, 1H), 3.69 – 3.57 (m, 3H), 3.54 – 3.43 (m, 2H), 3.44 – 3.33 (m, 3H), 3.24 (dd, *J* = 11.4, 2.0 Hz, 1H), 3.03 (s, 1H, OH), 2.46 (q, *J* = 6.8 Hz, 2H, H-8). ^{13}C NMR (125 MHz, $CDCl_3$) δ 157.50 (*a*d, *J* = 37 Hz, *CF_3CO*), 137.79, 137.34, 137.27, 137.20, 137.11, 136.38 (*aromatic* C), 134.54 (C-9), 128.51, 128.46, 128.44, 128.41, 128.31, 128.20, 128.07, 128.05, 127.99, 127.84, 127.73, 127.71, 127.62, 127.57, 127.19, 126.44 (*aromatic* C*H*), 116.86 (C-10), 115.77 (*a*d, *J* = 286 Hz, C*F_3*), 99.15 (C-1), 98.43 (C-1), 98.14 (C-1), 97.61 (C-1^C), 79.58, 76.19, 75.93, 75.21, 74.23, 73.91, 73.51, 73.46, 73.15, 72.97, 72.03, 71.84, 71.64, 71.02, 70.73, 70.33, 69.16, 68.96, 67.69, 67.66, 67.33 (C-7), 66.81, 66.63 (2 C-6), 64.13 (C-2), 62.37 (C-6), 59.42, 59.36 (2 C-2), 49.73 (C-2^C), 33.81 (C-8). HR-MS: Calculated for $C_{79}H_{87}N_{10}O_{18}F_3$ $[M+Na]^+$: 1543.60441, found: 1543.60362.

3-Butenyl 2-azido-3,6-di-*O*-benzyl-2-deoxy-*α*-D-galactopyranosyl-(1→4)-3,6-di-*O*-benzyl-2-deoxy-2-trifluoroacetamido-*α*-D-galacto-pyranosyl-(1→4)-2-azido-3,6-di-*O*-benzyl-2-deoxy-*α*-D-glucopyranosyl-(1→4)-2-azido-3,6-di-*O*-benzyl-2-deoxy-*α*-D-galactopyranoside (S9)

The reaction was carried out according to the general procedure D using compound **S8** (129 mg, 0.085 mmol), K_2CO_3 (13 mg, 0.09 mmol), KI (14 mg, 0.085 mmol) and $Ph_2BO(CH_2)_2NH_2$ (1.9 mg, 0.0085 mmol). The product was purified by column chromatography (pentane:EtOAc = 4:1). Compound **S9** (129 mg, 94% yield) was obtained as yellow syrup. $[\alpha]_D^{25}$ +136.4 (c=0.5, $CHCl_3$). ^{1}H NMR (500 MHz, $CDCl_3$) δ 7.62 – 7.23 (m, 40H, *aromatic* H), 7.17 (d, *J* = 9.9 Hz, 1H, NH), 5.92 (ddt, *J* = 17.0, 10.3, 6.8 Hz, 1H, H-9), 5.50 (d, *J* = 3.6 Hz, 1H, H-1^C), 5.25 – 5.16 (m, 2H, H-10), 5.15 – 5.10 (m, 3H, 3xH-1), 4.90 (d, *J* = 11.6 Hz, 2H, *PhCHH*O), 4.87 – 4.81 (m, 2H, *PhCHH*O, H-2^C), 4.81 – 4.74 (m, 2H), 4.72 – 4.61 (m, 5H), 4.57 – 4.47 (m, 3H), 4.46 – 4.39 (m, 4H), 4.37 (d, *J* = 2.9 Hz, 1H), 4.35 – 4.25 (m, 2H), 4.22 – 3.93 (m, 12H), 3.83 (dt, *J* = 9.8, 6.9 Hz, 1H, H-7), 3.76 (dd, *J* = 10.9, 3.5 Hz, 1H, H-2), 3.72 – 3.60 (m, 3H), 3.59 – 3.51 (m, 1H), 3.50 – 3.36 (m, 4H), 3.32 (dd, *J* = 10.0, 3.9 Hz, 1H), 3.29 – 3.22 (m, 1H), 2.48 (q, *J* = 6.9 Hz, 2H, H-8). ^{13}C NMR (125 MHz, $CDCl_3$) δ 157.25 (*a*d, *J* = 37 Hz, *CF_3CO*), 137.73, 137.53, 137.45, 137.38, 137.31, 137.22, 137.19, 136.32 (*aromatic* C), 134.50 (C-9), 128.46, 128.42, 128.38, 128.34, 128.32, 128.26, 128.18, 128.16, 128.03, 128.00, 127.98, 127.95, 127.89, 127.76, 127.71, 127.58, 127.56, 127.53, 127.51, 127.45, 127.28, 127.08, 126.71, 126.39 (*aromatic* C*H*), 116.85 (C-10), 115.72 (*a*d, *J* = 286 Hz, C*F_3*), 99.35 (C-1), 98.37 (C-1), 98.08 (C-1), 97.48 (C-1^C), 79.52, 76.15, 76.04, 75.20, 74.20 (C*H_2Ph*), 73.70, 73.45 (C*H_2Ph*), 73.41 (C*H_2Ph*), 73.20 (C*H_2Ph*), 73.10 (C*H_2Ph*), 72.89, 71.99, 71.75 (C*H_2Ph*), 71.16 (C*H_2Ph*), 70.99 (C*H_2Ph*), 70.69, 70.24, 69.76 (C-6), 68.92, 68.21, 67.63 (C-7), 66.84, 66.76 (C-6), 66.57 (C-6), 64.79 (C-6), 64.07 (C-2), 59.40 (C-2), 59.20 (C-2), 49.60 (C-2^C), 33.77 (C-8). HR-MS: Calculated for $C_{86}H_{93}N_{10}O_{18}F_3$ $[M+Na]^+$: 1633.65136, found: 1633.65160.

Pentasaccharide 18

The reaction was carried out according to the general procedure B using donor **14** (586 mg, 0.87 mmol) and acceptor **S9** (558 mg, 0.35 mmol). The product was purified by column chromatography (pentane:EtOAc = 6:1). Compound **18** (642 mg, α-only, 88% yield) was obtained as yellow syrup. $[\alpha]_D^{25}$ +134.2 (c=0.6, $CHCl_3$). ^{1}H NMR (500 MHz, $CDCl_3$) δ 7.38 – 7.18 (m, 41H, *aromatic* H), 7.17 – 7.15 (m, 2H, *aromatic* H), 7.05 – 7.01 (m, 2H, *aromatic* H), 6.99 (d, *J* = 10.0 Hz, 1H, NH), 6.23 (d, *J* = 9.5 Hz, 1H, NH), 5.79 (ddt, *J* = 17.1, 10.3, 6.8 Hz, 1H, H-9), 5.34 (d, *J* = 3.6 Hz, 1H, H-1), 5.12 – 5.02 (m, 2H, H-10), 4.99 (d, *J* = 3.6 Hz, 3H, 3xH-1), 4.82 (d, *J* = 3.8 Hz, 1H, H-1), 4.80 – 4.68 (m, 5H), 4.65 – 4.33 (m, 13H), 4.32 – 4.17 (m, 5H), 4.05 – 3.80 (m, 11H), 3.76 – 3.53 (m, 7H), 3.50 (dd, *J* = 11.1, 2.5 Hz, 1H), 3.44 (dd, *J* = 12.7, 2.1 Hz, 1H), 3.40 (dd, *J* = 8.3, 4.9 Hz, 1H), 3.37 – 3.28 (m, 2H), 3.26 (dd, *J* = 11.5, 2.1 Hz, 1H), 3.12 (dd, *J* = 11.5, 1.9 Hz, 1H), 2.96 (t, *J* = 9.5 Hz, 1H), 2.86 (dd, *J* = 8.9, 5.4 Hz, 1H), 2.41 – 2.32 (m, 2H, H-8), 1.06 (s, 9H, C*H_3*), 0.97 (s, 9H, C*H_3*). ^{13}C NMR (125 MHz, $CDCl_3$) δ 157.41 (*a*d, *J* = 37 Hz, *CF_3CO*), 156.57 (*a*d, *J* = 37 Hz, *CF_3CO*), 138.03, 137.94, 137.61, 137.56, 137.41, 137.36, 137.28, 136.92, 136.44 (*aromatic* C), 134.67 (C-9), 128.65, 128.62, 128.60, 128.54, 128.49, 128.44, 128.34, 128.22, 128.20, 128.16, 128.14, 128.12, 127.97, 127.93, 127.80, 127.77, 127.74, 127.72, 127.64, 127.34, 126.83, 126.41 (*aromatic* C*H*), 117.04 (C-10), 115.92 (*a*d, *J* = 286 Hz, 2xC*F_3*), 99.03 (C-1), 98.56 (C-1), 98.29 (C-1), 97.62 (C-1), 96.74 (C-1), 79.79, 76.46, 75.98, 75.42, 74.90, 74.55, 73.78, 73.69, 73.48, 73.31, 73.11, 73.09, 72.02, 71.95, 71.63, 71.46, 70.90, 70.17, 69.55, 69.51, 69.39, 69.13, 68.79, 67.87 (C-7), 67.79, 67.02, 66.95, 66.76, 65.76, 64.40, 60.27, 59.60, 49.67, 48.25 (5 C-2), 33.96, 27.64 (2 C*H_3*), 27.46, 23.35 (2 C-*Si*). ^{13}C-HMBC ($CDCl_3$, 125 MHz): 99.03 ($J_{C1,H1}$ = 171 Hz), 98.56 ($J_{C1,H1}$ = 173 Hz), 98.29 ($J_{C1,H1}$ = 170 Hz), 97.62 ($J_{C1,H1}$ = 176 Hz), 96.74 ($J_{C1,H1}$ = 174 Hz). HR-MS: Calculated for $C_{109}H_{125}N_{11}O_{23}F_6Si$ $[M+Na]^+$: 2120.85155, found: 2120.85651.

Pentasaccharide S10

The reaction was carried out according to the general procedure C using compound **18** (641 mg, 0.31 mmol) and HF/pyridine (70%, 130 µl, 5.0 mmol). The product was purified by column chromatography (pentane:EtOAc = 3:2). Compound **S10** (547 mg, 91% yield) was obtained as white solid. $[\alpha]_D^{25}$ +164.8 (c=0.4, $CHCl_3$). ^{1}H NMR (500 MHz, $CDCl_3$) δ 7.48 – 7.21 (m, 43H, *aromatic* H), 7.14 (dd, *J* = 8.1, 1.6 Hz, 3H), 6.53 (d, *J* = 9.5 Hz, 1H, NH), 5.87 (ddt, *J* = 17.0, 10.3, 6.8 Hz, 1H, H-9), 5.45 (d, *J* = 3.6 Hz, 1H, H-1), 5.21 – 5.10 (m, 2H, H-10), 5.09 – 5.05 (m, 2H, 2xH-1), 5.02 (d, *J* = 3.6 Hz, 1H, H-1), 4.92 (d, *J* = 3.7 Hz, 1H, H-1), 4.88 – 4.76 (m, 3H), 4.76 – 4.58 (m, 8H), 4.52 – 4.41 (m, 5H), 4.41 – 4.34 (m, 4H), 4.31 (d, *J* = 2.4 Hz, 1H), 4.26 (d, *J* = 12.4 Hz, 1H), 4.18 (d, *J* = 1.9 Hz, 1H), 4.12 – 3.89 (m, 12H), 3.82 (d, *J* = 11.8 Hz, 1H), 3.81 – 3.75 (m, 1H), 3.69 (dd, *J* = 10.9, 3.5 Hz, 1H), 3.67 – 3.33 (m, 11H), 3.26 – 3.18 (m, 1H), 3.11 – 2.95 (m, 3H), 2.44 (q, *J* = 6.8 Hz, 2H, H-8). ^{13}C NMR (125 MHz, $CDCl_3$) δ 157.34 (*a*d, *J* = 37 Hz, *CF_3CO*), 156.65 (*a*d, *J* = 37 Hz, *CF_3CO*), 137.88, 137.56, 137.39, 137.34, 137.26, 137.14, 136.98, 136.38 (*aromatic* C), 134.60 (C-9), 128.58, 128.55, 128.51, 128.46, 128.36, 128.34, 128.14, 128.11, 128.06, 128.03, 128.01, 127.96, 127.89, 127.86, 127.84, 127.82, 127.78, 127.67, 127.56, 127.28, 127.11, 126.48 (*aromatic* C*H*), 116.97 (C-10), 115.88 (*a*d, *J* = 286 Hz, C*F_3*), 98.97, 98.52, 98.20,

97.57, 96.90 (5 C-1), 79.75, 76.18, 75.85, 75.25, 75.22, 74.43, 73.63, 73.59, 73.34, 73.23, 73.09, 73.03, 72.02, 71.89, 71.77, 71.37, 70.81, 70.64, 70.52, 70.08, 69.32, 69.04, 68.70, 67.76 (C-7), 67.72, 66.86, 66.67 (3 C-6), 66.30, 65.81 (C-6), 64.29 (C-2), 62.37 (C-6), 60.17, 59.48, 49.58, 48.77 (4 C-2), 33.88 (C-8). HR-MS: Calculated for $C_{101}H_{109}N_{11}O_{23}F_6$ $[M+NH_4]^+$: 1975.79402, found: 1980.79644.

Pentasaccharide S11

The reaction was carried out according to the general procedure D using compound **S10** (546 mg, 0.28 mmol), K_2CO_3 (42.6 mg), KI (46.5 mg) and $Ph_2BO(CH_2)_2NH_2$ (6.3 mg). The product was purified by column chromatography (pentane:EtOAc = 4:1). Compound **S11** (531 mg, 92% yield) was obtained as white foam. $[\alpha]_D^{25}$ +127 (c=0.3, $CHCl_3$). ^{1}H NMR (500 MHz, $CDCl_3$) δ 7.44 – 7.22 (m, 48H, *aromatic* H), 7.19 – 7.14 (m, 2H, *aromatic* H), 7.10 (d, *J* = 9.9 Hz, 1H, NH), 6.52 (d, *J* = 9.6 Hz, 1H, NH), 5.89 (ddt, *J* = 17.0, 10.2, 6.8 Hz, 1H, H-9), 5.44 (d, *J* = 3.6 Hz, 1H, H-1), 5.22 – 5.12 (m, 2H, H-10), 5.08 (t, *J* = 3.1 Hz, 2H, 2xH-1), 5.04 (d, *J* = 3.7 Hz, 1H, H-1), 4.92 (d, *J* = 3.8 Hz, 1H), 4.90 (d, *J* = 4.5 Hz, 1H), 4.86 (d, *J* = 10.9 Hz, 1H), 4.83 – 4.73 (m, 3H), 4.73 – 4.55 (m, 7H), 4.53 – 4.24 (m, 15H), 4.13 – 3.90 (m, 11H), 3.86 (d, *J* = 11.7 Hz, 1H), 3.80 (dt, *J* = 9.8, 6.9 Hz, 1H), 3.71 (dd, *J* = 10.9, 3.5 Hz, 1H), 3.68 – 3.62 (m, 3H), 3.61 – 3.54 (m, 2H), 3.52 – 3.43 (m, 2H), 3.39 (dd, *J* = 10.2, 3.6 Hz, 1H), 3.35 (dd, *J* = 11.5, 2.1 Hz, 1H), 3.30 (dd, *J* = 9.5, 4.5 Hz, 1H), 3.22 (dd, *J* = 11.4, 1.9 Hz, 1H), 3.12 (t, *J* = 9.5 Hz, 1H), 3.07 – 3.00 (m, 2H), 2.46 (qt, *J* = 6.8, 1.4 Hz, 2H, H-8). ^{13}C NMR (125 MHz, $CDCl_3$) δ 157.28 (*a*d, *J* = 37 Hz, *CF₃CO*), 156.60 (*a*d, *J* = 37 Hz, *CF₃CO*), 137.85, 137.68, 137.53, 137.41, 137.38, 137.33, 137.28, 137.04, 136.37 (*aromatic* C), 134.60 (C-9), 128.58, 128.52, 128.50, 128.48, 128.46, 128.42, 128.36, 128.33, 128.15, 128.12, 128.06, 128.04, 127.89, 127.87, 127.78, 127.76, 127.74, 127.69, 127.67, 127.61, 127.57, 127.23, 126.50 (*aromatic* C*H*), 116.97 (C-10), 115.89 (*a*d, *J* = 286 Hz, C*F₃*), 99.02, 98.48, 98.20, 97.55, 97.08 (5 C-1), 79.70, 76.16, 75.77, 75.44, 75.34, 74.43, 73.67, 73.60, 73.46, 73.35, 73.22, 73.00, 72.96, 71.90, 71.87, 71.79, 71.33, 70.79, 70.57, 70.45, 70.08, 69.09 (C-6), 69.03, 68.79, 68.60, 67.77 (C-7), 67.74, 66.86, 66.66, 65.76 (4 C-6), 65.63, 64.27, 60.00, 59.51, 49.59, 48.86 (5 C-2), 33.88 (C-8). HR-MS: Calculated for $C_{108}H_{115}N_{11}O_{23}F_6$ $[M+NH_4]^+$: 2065.84097, found: 2065.84186.

Hexasaccharide 19

The reaction was carried out according to the general procedure B using donor **13** (164 mg, 0.27 mmol) and acceptor **S11** (185 mg, 0.09 mmol). The product was purified by column chromatography (pentane:EtOAc = 6:1). Compound **19** (546 mg, α-only, 82% yield) was obtained as yellow syrup. $[\alpha]_D^{25}$ +155.0 (c=0.4, $CHCl_3$). ^{1}H NMR (500 MHz, $CDCl_3$) δ 7.57 – 7.52 (m, 4H, *aromatic* H), 7.47 – 7.25 (m, 50H, *aromatic* H), 7.18 – 7.14 (m, 2H, *aromatic* H), 7.12 (d, *J* = 9.9 Hz, 1H, NH), 6.40 (d, *J* = 9.8 Hz, 1H, NH), 5.90 (ddt, *J* = 17.0, 10.2, 6.8 Hz, 1H, H-9), 5.45 (d, *J* = 3.6 Hz, 1H, H-1), 5.24 – 5.13 (m, 2H, H-10), 5.10 (d, *J* = 3.6 Hz, 2H, 2xH-1), 5.04 (dd, *J* = 7.9, 4.6 Hz, 3H, 2xH-1), 4.91 (d, *J* = 3.8 Hz, 1H, H-1), 4.90 – 4.60 (m, 14H), 4.57 – 4.22 (m, 15H), 4.15 – 3.92 (m, 14H), 3.88 – 3.57 (m, 12H), 3.55 – 3.48 (m, 1H), 3.46 – 3.39 (m, 2H), 3.39 – 3.33 (m, 1H), 3.26 – 3.20 (m, 1H), 3.16 (dd, *J* = 8.3, 5.1 Hz, 1H), 3.08 (t, *J* = 9.4 Hz, 1H), 3.00 (dd, *J* = 9.0, 5.4 Hz, 1H), 2.50 – 2.43 (m, 2H, H-8), 1.13 (s, 9H, C*H₃*), 1.09 (s, 9H, C*H₃*). ^{13}C NMR (125 MHz, $CDCl_3$) δ 157.33 (*a*d, *J* = 37 Hz, *CF₃CO*), 156.56 (*a*d, *J* = 37 Hz, *CF₃CO*), 137.92, 137.85, 137.61, 137.58, 137.43, 137.33, 137.30, 137.17, 136.92, 136.34 (*aromatic* C), 134.60 (C-9), 128.65, 128.60, 128.54, 128.51, 128.46, 128.44, 128.37, 128.24, 128.17, 128.16, 128.08, 128.06, 128.02, 127.98, 127.96, 127.88, 127.75, 127.69, 127.59, 127.56, 127.23, 126.85, 126.70, 126.46 (*aromatic* C*H*), 117.00 (C-10), 115.88 (*a*d, *J* = 286 Hz, C*F₃*), 99.13, 98.50, 98.43, 98.22, 97.64, 97.04 (6 C-1), 79.67, 76.30, 76.23, 75.90, 75.35, 75.24, 74.49, 73.85, 73.62, 73.36, 73.23, 73.09, 73.00, 72.03, 71.92, 71.63, 71.35, 70.83, 70.63, 70.60, 70.30, 70.21, 70.11, 69.64, 69.55, 69.04, 68.77, 67.79 (C-7), 67.71 (C-6), 67.42, 67.14, 66.85, 66.70, 66.38, 65.56 (5 C-6), 64.29, 59.97, 59.53, 58.46, 49.64, 49.11 (6 C-2), 33.90 (C-8), 27.60, 27.43 (2 C*H₃*), 23.27, 20.73 (2 C-*Si*). ^{13}C-HMBC ($CDCl_3$, 125 MHz): 99.13 ($J_{C1,H1}$ = 171 Hz), 98.50 ($J_{C1,H1}$ = 173 Hz), 98.43 ($J_{C1,H1}$ = 172 Hz), 98.22 ($J_{C1,H1}$ = 171 Hz), 97.64 ($J_{C1,H1}$ = 174 Hz), 97.04 ($J_{C1,H1}$ = 175 Hz). MALDI-MS: Calculated for $C_{129}H_{146}N_{14}O_{27}F_6Si$ [M+Na]+: 2488.0048, found: 2487.9692.

Hexasaccharide S12

The reaction was carried out according to the general procedure C using compound **19** (215 mg, 0.087 mmol) and HF/pyridine (70%, 36 µl, 1.39 mmol). The product was purified by column chromatography (pentane:EtOAc = 3:2). Compound **S12** (177 mg, 87% yield) was obtained as white solid. ^{1}H NMR (500 MHz, $CDCl_3$) δ 7.39 – 7.12 (m, 53H, *aromatic* H), 7.08 – 7.02 (m, 3H), 6.42 (d, *J* = 9.7 Hz, 1H, NH), 5.79 (ddt, *J* = 17.1, 10.2, 6.8 Hz, 1H, H-9), 5.33 (d, *J* = 3.6 Hz, 1H, H-1), 5.13 – 5.02 (m, 2H, H-10), 4.98 (t, *J* = 2.8 Hz, 2H, 2xH-1), 4.92 (dd, *J* = 5.7, 3.7 Hz, 2H, 2xH-1), 4.89 (d, *J* = 12.3 Hz, 1H), 4.81 (d, *J* = 3.8 Hz, 1H, H-1), 4.79 – 4.73 (m, 2H), 4.74 – 4.64 (m, 4H), 4.63 – 4.48 (m, 6H), 4.45 – 4.13 (m, 16H), 4.03 – 3.81 (m, 14H), 3.80 – 3.64 (m, 4H), 3.61 (dd, *J* = 10.9, 3.5 Hz, 1H), 3.58 – 3.36 (m, 7H), 3.34 – 3.22 (m, 3H), 3.16 – 3.05 (m,

2H), 2.99 (t, *J* = 9.5 Hz, 1H), 2.90 (dd, *J* = 9.2, 5.4 Hz, 1H), 2.78 (bs, 1H, OH), 2.36 (q, *J* = 6.8 Hz, 2H, H-8), 2.28 (bs, 1H, OH). ^{13}C NMR (125 MHz, $CDCl_3$) δ 157.34 (*a*d, *J* = 37 Hz, *CF_3CO*), 156.82 (*a*d, *J* = 37 Hz, *CF_3CO*), 137.85, 137.58, 137.52, 137.47, 137.45, 137.42, 137.33, 137.28, 137.16, 137.03, 136.36 (*aromatic* C), 134.61 (C-9), 128.64, 128.60, 128.53, 128.52, 128.50, 128.47, 128.40, 128.38, 128.34, 128.29, 128.17, 128.13, 128.09, 128.03, 127.97, 127.95, 127.90, 127.87, 127.86, 127.77, 127.76, 127.69, 127.63, 127.59, 127.25, 127.13, 126.86, 126.50 (*aromatic* C*H*), 116.99 (C-10), 115.87 (*a*d, *J* = 286 Hz, C*F_3*), 99.13, 98.94, 98.50, 98.21, 97.64, 97.05 (6 C-1), 79.69, 76.62, 76.27, 76.12, 75.33, 75.22, 74.47, 73.82, 73.62, 73.36, 73.23, 73.15, 73.03, 72.08, 71.93, 71.89, 71.83, 71.71, 71.37, 70.56, 70.10, 69.71, 69.18, 69.04, 68.74, 67.79 (C-7), 67.72 (C-6), 67.47, 66.86, 66.71, 66.52, 65.64 (4 C-6), 64.28 (C-2), 62.61 (C-6), 59.98, 59.51, 59.48, 49.63, 49.32 (5 C-2), 33.90 (C-8). MALDI-MS: Calculated for $C_{121}H_{130}N_{14}O_{27}F_6$ [M+Na]+: 2347.9026, found: 2347.8708.

Hexasaccharide S13

The reaction was carried out according to the general procedure D using compound **S12** (173 mg, 0.074 mmol), K_2CO_3 (11 mg, 0.081 mmol), KI (12 mg, 0.074 mmol) and $Ph_2BO(CH_2)_2NH_2$ (1.7 mg, 0.0074 mmol). The product was purified by column chromatography (pentane:EtOAc = 4:1). Compound **S13** (170 mg, 95% yield) was obtained as white foam. ^{1}H NMR (500 MHz, $CDCl_3$) δ 7.46 – 7.39 (m, 4H, *aromatic* H), 7.36 – 7.14 (m, 54H, *aromatic* H), 7.08 – 7.04 (m, 2H, *aromatic* H), 7.02 (d, *J* = 9.9 Hz, 1H, NH), 6.35 (d, *J* = 9.7 Hz, 1H, NH), 5.79 (ddt, *J* = 17.1, 10.2, 6.8 Hz, 1H, H-9), 5.33 (d, *J* = 3.6 Hz, 1H, H-1), 5.12 – 5.02 (m, 2H, H-10), 5.01 – 4.96 (m, 2H), 4.94 (t, *J* = 3.8 Hz, 2H, 2xH-1), 4.89 (d, *J* = 12.3 Hz, 1H, *PhC*H*H*O), 4.80 (d, *J* = 3.8 Hz, 1H, H-1), 4.78 – 4.50 (m, 12H), 4.46 – 4.21 (m, 15H), 4.18 – 4.13 (m, 2H), 4.02 – 3.74 (m, 17H), 3.70 (dt, *J* = 9.8, 6.9 Hz, 1H), 3.62 – 3.37 (m, 7H), 3.33 – 3.21 (m, 4H), 3.15 – 3.04 (m, 3H), 2.99 (t, *J* = 9.4 Hz, 1H), 2.91 (dd, *J* = 9.1, 5.4 Hz, 1H), 2.36 (q, *J* = 6.8 Hz, 2H, H-8). ^{13}C NMR (125 MHz, $CDCl_3$) δ 157.32 (*a*d, *J* = 37 Hz, *CF_3CO*), 156.61 (*a*d, *J* = 37 Hz, *CF_3CO*), 137.84, 137.68, 137.60, 137.58, 137.55, 137.47, 137.44, 137.39, 137.32, 137.28, 137.02, 136.35 (*aromatic* C), 134.60 (C-9), 128.58, 128.52, 128.50, 128.48, 128.46, 128.39, 128.36, 128.33, 128.32, 128.31, 128.16, 128.12, 128.07, 128.01, 127.96, 127.94, 127.89, 127.86, 127.75, 127.72, 127.67, 127.65, 127.58, 127.23, 127.16, 126.80, 126.52 (*aromatic* C*H*), 116.98 (C-10), 115.88 (*a*d, *J* = 286 Hz, C*F_3*), 99.10, 99.00, 98.48, 98.20, 97.60, 97.11 (6 C-1), 79.67, 76.58, 76.19, 76.16, 75.33, 75.12, 74.45, 73.79, 73.60, 73.38, 73.35, 73.22, 73.13, 73.01, 72.98, 72.00, 71.91, 71.69, 71.59, 71.44, 71.32, 70.81, 70.78, 70.49, 70.09, 69.86, 69.65, 69.03, 68.79, 68.21, 67.78, 67.72, 66.97, 66.85, 66.71, 66.50, 65.65, 64.26, 59.97, 59.51, 59.33, 49.62, 49.23 (6 C-2), 33.89 (C-8). MALDI-MS: Calculated for $C_{128}H_{136}N_{14}O_{27}F_6$ [M+Na]+: 2437.9496, found: 2437.9159.

Heptasaccharide 20

The reaction was carried out according to the general procedure B using donor **14** (142 mg, 0.21 mmol) and acceptor **S13** (170 mg, 0.07 mmol). The product was purified by column chromatography (pentane:EtOAc = 6:1). Compound **20** (165 mg, α-only, 81% yield) was obtained as yellow syrup. $[\alpha]_D^{25}$ +176 (c=0.5, $CHCl_3$). ^{1}H NMR (500 MHz, $CDCl_3$) δ 7.47 – 7.18 (m, 61H, *aromatic* H), 7.16 – 7.12 (m, 2H, *aromatic* H), 7.11 – 7.06 (m, 2H, *aromatic* H), 7.05 (d, *J* = 9.7 Hz, 1H, NH), 6.34 (t, *J* = 12.4, 9.6 Hz, 2H, 2xNH), 5.92 – 5.78 (m, 1H, H-9), 5.38 (d, *J* = 3.6 Hz, 1H, H-1), 5.18 – 5.08 (m, 2H, H-10), 5.06 (d, *J* = 3.6 Hz, 1H, H-1), 5.05 – 5.02 (m, 2H, 2xH-1), 4.99 (d, *J* = 3.7 Hz, 1H, H-1), 4.95 (d, *J* = 12.3 Hz, 1H, *PhC*H*H*O), 4.87 (d, *J* = 3.7 Hz, 1H, H-1), 4.85 (d, *J* = 3.7 Hz, 1H, H-1), 4.83 – 4.24 (m, 32H), 4.22 (d, *J* = 12.4 Hz, 1H), 4.09 – 3.42 (m, 31H), 3.40 – 3.27 (m, 4H), 3.21 – 2.92 (m, 6H), 2.42 (qt, *J* = 6.7, 1.4 Hz, 2H, H-8), 1.11 (s, 9H, *C*H_3), 1.03 (s, 9H, *C*H_3). ^{13}C NMR (125 MHz, $CDCl_3$) δ 157.40 (*a*d, *J* = 37 Hz, *CF_3CO*), 156.65 (*a*d, *J* = 37 Hz, *CF_3CO*), 137.97, 137.92, 137.70, 137.68, 137.64, 137.56, 137.46, 137.41, 137.36, 137.29, 137.09, 136.96, 136.43 (*aromatic* C), 134.68 (C-9), 128.67, 128.60, 128.58, 128.55, 128.52, 128.48, 128.44, 128.43, 128.39, 128.36, 128.24, 128.22, 128.16, 128.12, 128.10, 128.02, 127.97, 127.94, 127.92, 127.90, 127.85, 127.83, 127.80, 127.76, 127.66, 127.32, 127.01, 126.83, 126.59 (*aromatic* C*H*), 117.06 (C-10), 115.96 (*a*d, *J* = 286 Hz, 3xC*F_3*), 99.19, 98.59, 98.47, 98.29, 97.71, 97.23, 96.77 (7 C-1), 79.77, 76.29, 76.22, 75.43, 75.40, 74.87, 74.57, 73.90, 73.70, 73.43, 73.30, 73.13, 73.11, 73.10, 73.01, 72.07, 72.00, 71.86, 71.77, 71.42, 71.13, 71.05, 70.90, 70.67, 70.17, 69.64, 69.59, 69.42, 69.35, 69.12, 68.86, 68.66, 67.88, 67.80, 67.77, 67.02, 66.94, 66.80, 66.59, 65.85, 65.81, 64.38, 60.23, 60.08, 59.60, 49.70, 49.21, 48.28 (7 C-2), 33.98(C-8), 27.66, 27.47 (2 C*H_3*), 23.37, 20.77 (2 C-*Si*). ^{13}C-HMBC ($CDCl_3$, 125 MHz): 99.19 ($J_{C1,H1}$ = 172 Hz), 98.59 ($J_{C1,H1}$ = 172 Hz), 98.47 ($J_{C1,H1}$ = 177 Hz), 98.29 ($J_{C1,H1}$ = 172 Hz), 97.71 ($J_{C1,H1}$ = 175 Hz), 97.23 ($J_{C1,H1}$ = 175 Hz), 96.77 ($J_{C1,H1}$ = 177 Hz). MALDI-MS: Calculated for $C_{151}H_{168}N_{15}O_{32}F_9Si$ [M+Na]+: 2925.1498, found: 2925.1039.

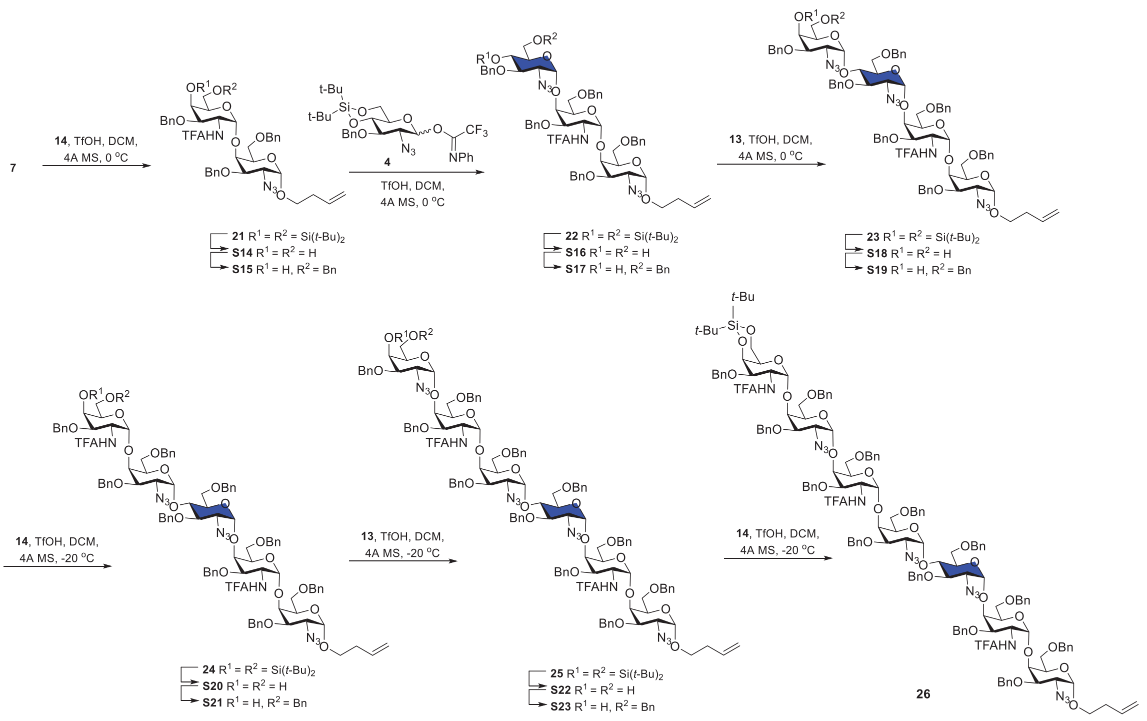

3-Butenyl 3-*O*-benzyl-2-deoxy-4,6-di-*tert*-butylsilylidene-2-trifluoroacetamido-α-D-galactopyranosyl-(1→4)-2-azido-3,6-di-*O*-benzyl-2-deoxy-α-D-galactopyranoside (21)

The reaction was carried out according to the general procedure A. The donor **14** (2.98 g, 4.4 mmol) and acceptor **7** (1.29 g, 2.94 mmol) were co-evaporated with toluene (three times). The residue was dissolved in dry 29 ml DCM under nitrogen and stirred over fresh flame-dried molecular sieves 4Å. The solution was cooled to 0 ºC, after which TfOH (39 µl, 0.44 mmol) was added. The reaction was stirred at 0 ºC for 2 h. Then the reaction was quenched with Et_3N, diluted with DCM, washed with saturated $NaHCO_3$ and brine. The organic phase was dried with anhydrous $MgSO_4$, filtered and concentrated *in vacuo*. The product was purified by silica gel column chromatography (pentane:EtOAc = 15:1). Compound **21** (2.34 g, α-only, 86% yield) was obtained as yellow syrup. $[\alpha]_D^{25}$ +165.7 (c=0.4, $CHCl_3$). ^{1}H NMR (400 MHz, $CDCl_3$) δ 7.38 – 7.25 (m, 15H, *aromatic* H), 6.32 (d, *J* = 9.4 Hz, 1H, NH), 5.77 (ddt, *J* = 17.0, 10.2, 6.7 Hz, 1H, H-9), 5.14 – 5.00 (m, 3H, H-10, 1^{B}), 4.84 (d, *J* = 3.6 Hz, 1H, H-1^{A}), 4.75 (d, *J* = 12.0 Hz, 1H, *PhC*H*H*O), 4.69 (d, *J* = 11.9 Hz, 1H, *PhC*H*H*O), 4.66 – 4.61 (m, 1H, H-2^{B}), 4.60 (d, *J* = 11.8 Hz, 1H, *PhC*H*H*O), 4.49 (d, *J* = 2.4 Hz, 1H, H-4^{B}), 4.47 (d, *J* = 11.8 Hz, 2H, *PhC*H*H*O), 4.39 (d, *J* = 11.4 Hz, 1H, *PhC*H*H*O), 4.36 (d, *J* = 2.7 Hz, 1H, H-4^{A}), 4.04 (d, *J* = 2.4 Hz, 1H, , H-5^{B}), 3.91 (dd, *J* = 9.2, 5.8 Hz, 1H, H-5^{A}), 3.81 (dd, *J* = 10.7, 2.8 Hz, 1H, , H-3^{A}), 3.76 (dd, *J* = 12.8, 1.6 Hz, 1H, , H-6^{B}), 3.72 – 3.65 (m, 1H, H-7), 3.65 – 3.56 (m, 2H, H-3^{B}, 6^{B}), 3.55 – 3.48 (m, 1H, H-7), 3.45 – 3.30 (m, 3H, , H-2^{A}, 6^{A}), 2.34 (qt, *J* = 6.7, 1.3 Hz, 2H, H-8), 1.09 (s, 9H, *C*H$_3$), 1.00 (s, 9H, *C*H$_3$). ^{13}C NMR (100 MHz, $CDCl_3$) δ 156.94 (*a*d, *J* = 37 Hz, *CF$_3$CO*), 137.88, 137.12, 136.90 (*aromatic* C), 134.59 (C-9), 128.75, 128.71, 128.68, 128.62, 128.52, 128.43, 128.35, 128.07, 128.01, 127.96, 127.72, 127.67, 127.19, 127.13 (*aromatic* C*H*), 117.08 (C-10), 115.96 (*a*d, *J* = 286 Hz, C*F$_3$*), 98.03 (C-1^{A}), 97.27 (C-1^{B}), 75.79 (C-3^{A}), 74.34 (C-3^{B}), 73.79 (C*H$_2$Ph*), 71.82 (C*H$_2$Ph*), 70.33 (C-4^{A}), 69.65 (C*H$_2$Ph*), 69.30 (C-4^{B}), 68.87 (C-5^{A}), 68.03 (C-5^{B}), 67.88 (C-7), 67.05 (C-6^{B}), 66.63 (C-6^{A}), 59.81 (C-2^{A}), 48.45 (C-2^{B}), 33.93 (C-8), 27.69, 27.47 (2 C*H$_3$*), 23.41, 20.80 (2 C-*Si*). HR-MS: Calculated for $C_{47}H_{61}N_4O_{10}F_3Si$ $[M+NH_4]^+$: 944.44473, found: 944.44512.

3-Butenyl 3-*O*-benzyl-2-deoxy-2-trifluoroacetamido-α-D-galactopyranosyl-(1→4)-2-azido-3,6-di-*O*-benzyl-2-deoxy-α-D-galactopyranoside (S14)

The reaction was carried out according to the general procedure C using compound **21** (3.6 g, 3.88 mmol) and HF/pyridine (70%, 1.6 ml, 62.1 mmol). The product was purified by column chromatography (pentane:EtOAc = 2:1). Compound **S14** (2.78 g, 91% yield) was obtained as yellow syrup. $[\alpha]_D^{25}$ +128.2 (c=0.4, $CHCl_3$). ^{1}H NMR (400 MHz, $CDCl_3$) δ 7.41 – 7.22 (m, 15H), 6.56 (d, *J* = 9.4 Hz, 1H), 5.77 (ddt, *J* = 17.0, 10.2, 6.7 Hz, 1H), 5.11 – 5.01 (m, 2H), 4.98 (d, *J* = 3.6 Hz, 1H), 4.85 (d, *J* = 3.6 Hz, 1H), 4.79 (d, *J* = 11.8 Hz, 1H), 4.66 (d, *J* = 11.9 Hz, 1H), 4.62 (d, *J* = 11.8 Hz, 1H), 4.55 – 4.46 (m, 1H), 4.45 – 4.37 (m, 3H), 4.31 (d, *J* = 2.7 Hz, 1H), 4.15 (dd, *J* = 2.9, 1.4 Hz, 1H), 4.12 – 4.06 (m, 1H), 3.89 (dd, *J* =

8.8, 5.9 Hz, 1H), 3.83 (dd, *J* = 10.8, 2.7 Hz, 1H), 3.71 – 3.28 (m, 8H), 3.13 (s, 1H), 2.34 (qt, *J* = 6.8, 1.4 Hz, 2H). ^{13}C NMR (100 MHz, $CDCl_3$) δ 157.08 (*a*d, *J* = 37 Hz, *CF₃CO*), 137.21, 137.13, 136.94 (*aromatic* C), 134.53 (C-9), 128.65, 128.63, 128.59, 128.23, 128.15, 128.10, 128.08, 127.94, 127.38 (*aromatic* C*H*), 116.99 (C-10), 115.87 (*a*d, *J* = 286 Hz, C*F₃*), 97.99, 97.40, 75.48, 74.70, 73.58, 71.89, 71.18, 70.75, 69.50, 68.93, 67.80, 66.63, 66.60, 62.55, 59.64, 48.93, 33.82. HR-MS: Calculated for $C_{39}H_{45}N_4O_{10}F_3$ $[M+Na]^+$: 809.29800, found: 809.29837.

3-Butenyl 3,6-di-*O*-benzyl-2-deoxy-2-trifluoroacetamido-*α*-D-galactopyranosyl-(1→4)-2-azido-3,6-di-*O*-benzyl-2-deoxy-*α*-D-galacto-pyranoside (S15)

The reaction was carried out according to the general procedure D using compound **S14** (1.83 g, 2.33 mmol), K_2CO_3 (354 mg, 2.56 mmol), KI (387 mg, 2.33 mmol) and $Ph_2BO(CH_2)_2NH_2$ (53 mg, 0.233 mmol). The product was purified by column chromatography (pentane:EtOAc = 5:1). Compound **S15** (1.96 g, 96% yield) was obtained as yellow syrup. $[\alpha]_D^{25}$ +129.9 (c=1, $CHCl_3$). ^{1}H NMR (400 MHz, $CDCl_3$) δ 7.40 – 7.18 (m, 20H), 6.44 (d, *J* = 9.4 Hz, 1H), 5.75 (ddt, *J* = 17.0, 10.2, 6.8 Hz, 1H), 5.11 – 5.00 (m, 2H), 4.97 (d, *J* = 3.6 Hz, 1H), 4.83 (d, *J* = 3.6 Hz, 1H), 4.79 (d, *J* = 12.3 Hz, 1H), 4.70 (d, *J* = 12.0 Hz, 1H), 4.62 – 4.50 (m, 2H), 4.45 – 4.38 (m, 3H), 4.37 – 4.23 (m, 4H), 4.22 – 4.17 (m, 1H), 3.87 (dd, *J* = 8.8, 6.0 Hz, 1H), 3.78 (dd, *J* = 10.8, 2.7 Hz, 1H), 3.69 – 3.58 (m, 2H), 3.58 – 3.45 (m, 2H), 3.43 – 3.29 (m, 4H), 2.93 (s, 1H), 2.32 (qt, *J* = 6.8, 1.4 Hz, 2H). ^{13}C NMR (100 MHz, $CDCl_3$) δ 156.99 (*a*d, *J* = 37 Hz, *CF₃CO*), 137.67, 137.37, 137.31, 137.01 (*aromatic* C), 134.57 (C-9), 129.04, 128.63, 128.56, 128.47, 128.40, 128.25, 128.12, 127.99, 127.94, 127.89, 127.86, 127.79, 127.48 (*aromatic* C*H*), 117.00 (C-10), 115.83 (*a*d, *J* = 286 Hz, C*F₃*), 98.06, 97.47, 75.33, 74.88, 73.60, 73.59, 71.74, 71.14, 70.64, 69.23, 68.96, 67.79, 66.68, 65.72, 59.60, 49.02, 33.86. HR-MS: Calculated for $C_{46}H_{51}N_4O_{10}F_3$ $[M+ NH_4]^+$: 894.38955, found: 894.39005.

3-Butenyl 2-azido-3-*O*-benzyl-2-deoxy-4,6-di-*tert*-butylsilylidene-*α*-D-glucopyranosyl-(1→4)-3,6-di-*O*-benzyl-2-deoxy-2-trifluoroacet-amido-*α*-D-galactopyranosyl-(1→4)-2-azido-3,6-di-*O*-benzyl-2-deoxy-*α*-D-galactopyranoside (22)

The reaction was carried out according to the general procedure A. The donor **4** (934 mg, 1.54 mmol) and acceptor **S15** (450 mg, 0.51 mmol) were co-evaporated with toluene (three times). The residue was dissolved in dry 5 ml DCM under nitrogen and stirred over fresh flame-dried molecular sieves 4Å. The solution was cooled to -10 °C, after which TfOH (14 µl, 0.15 mmol) was added. The reaction was stirred at -10 °C for overnight. Then the reaction was quenched with Et_3N, diluted with DCM, washed with saturated $NaHCO_3$ and brine. The organic phase was dried with anhydrous $MgSO_4$, filtered and concentrated *in vacuo*. The product was purified by silica gel column chromatography (pentane:EtOAc = 12:1). Compound **22** (583 mg, α-only, 85% yield) was obtained as yellow syrup. $[\alpha]_D^{25}$ +114.3 (c=1, $CHCl_3$). ^{1}H NMR (400 MHz, $CDCl_3$) δ 7.48 – 7.18 (m, 25H), 6.03 (d, *J* = 9.2 Hz, 1H), 5.75 (ddt, *J* = 17.0, 10.2, 6.7 Hz, 1H), 5.10 (d, *J* = 10.4 Hz, 1H), 5.08 – 5.00 (m, 3H), 4.89 – 4.76 (m, 4H), 4.73 (d, *J* = 3.6 Hz, 1H), 4.60 – 4.41 (m, 4H), 4.40 – 4.28 (m, 3H), 4.25 (d, *J* = 2.6 Hz, 1H), 4.22 (d, *J* = 2.4 Hz, 1H), 4.10 (d, *J* = 11.6 Hz, 1H), 4.00 – 3.73 (m, 8H), 3.68 – 3.57 (m, 2H), 3.48 (dt, *J* = 9.7, 6.5 Hz, 1H), 3.33 (dd, *J* = 9.1, 5.8 Hz, 1H), 3.29 – 3.10 (m, 4H), 2.31 (qt, *J* = 6.8, 1.4 Hz, 2H), 1.05 (s, 9H), 1.02 (s, 9H). ^{13}C NMR (100 MHz, $CDCl_3$) δ 156.62 (*a*d, *J* = 37 Hz, *CF₃CO*), 138.32, 137.51, 137.33, 137.19 (*aromatic* C), 134.62 (C-9), 128.84, 128.67, 128.63, 128.55, 128.52, 128.48, 128.34, 128.29, 128.15, 128.14, 127.98, 127.89, 127.11 (*aromatic* C*H*), 117.02 (C-10), 115.84 (*a*d, *J* = 286 Hz, C*F₃*), 98.20, 98.08, 97.55, 79.40, 79.32, 75.79, 75.76, 73.53, 73.32, 72.59, 71.71, 71.33, 71.14, 70.68, 69.74, 68.99, 67.80, 67.12, 66.79, 66.65, 66.29, 62.90, 59.60, 49.40, 33.92, 27.45, 27.06, 22.74, 20.07. HR-MS: Calculated for $C_{67}H_{82}N_7O_{14}F_3Si$ $[M+Na]^+$: 1316.55333, found: 1316.55334.

3-Butenyl 2-azido-3-*O*-benzyl-2-deoxy-*α*-D-glucopyranosyl-(1→4)-3,6-di-*O*-benzyl-2-deoxy-2-trifluoroacet-amido-*α*-D-galactopyranosyl -(1→4)-2-azido-3,6-di-*O*-benzyl-2-deoxy-*α*-D-galactopyranoside (S16)

The reaction was carried out according to the general procedure C using compound **22** (655 mg, 0.51 mmol) and HF/pyridine (70%, 210 µl, 8.1 mmol). The product was purified by column chromatography (pentane:EtOAc = 2:1). Compound **S16** (497 mg, 84% yield) was obtained as white foam. $[\alpha]_D^{25}$ +139.7 (c=1, $CHCl_3$). ^{1}H NMR (400 MHz, $CDCl_3$) δ 7.46 – 7.16 (m, 25H), 6.40 (d, *J* = 9.5 Hz, 1H), 5.75 (ddt, *J* = 17.0, 10.2, 6.7 Hz, 1H), 5.11 – 4.96 (m, 3H), 4.93 – 4.79 (m, 4H), 4.78 (d, *J* = 3.7 Hz, 1H), 4.71 (d, *J* = 12.3 Hz, 1H), 4.55 – 4.37 (m, 4H), 4.33 – 4.24 (m, 3H), 4.21 (d, *J* = 2.3 Hz, 1H), 4.09 (s, 3H), 3.94 (dd, *J* = 10.3, 8.8 Hz, 1H), 3.89 – 3.74 (m, 3H), 3.73 – 3.44 (m, 5H), 3.43 – 3.17 (m, 7H), 2.32 (qt, *J* = 6.8, 1.5 Hz, 3H). ^{13}C NMR (100 MHz, $CDCl_3$) δ 157.09 (*a*d, *J* = 37 Hz, *CF₃CO*), 138.30, 137.43, 137.39, 137.24, 136.82 (*aromatic* C), 134.55 (C-9), 128.67, 128.63, 128.62, 128.52, 128.44, 128.38, 128.31, 128.27, 128.19, 128.03, 127.96, 127.89, 127.85, 127.63, 127.15 (*aromatic* C*H*), 117.02 (C-10), 115.83 (*a*d, *J* = 286 Hz, C*F₃*), 98.73, 98.06, 97.25, 80.00, 75.56, 75.22, 74.15, 73.65, 73.28, 72.24, 71.90, 71.59, 71.47, 70.90, 70.61,

69.91, 68.81, 67.83, 66.55, 66.33, 63.53, 62.20, 59.54, 49.49, 33.86. HR-MS: Calculated for $C_{59}H_{66}N_7O_{14}F_3$ $[M+Na]^+$: 1176.45121, found: 1176.45147.

3-Butenyl 2-azido-3,6-di-*O*-benzyl-2-deoxy-α-D-glucopyranosyl-(1→4)-3,6-di-*O*-benzyl-2-deoxy-2-trifluoro-acetamido-α-D-galactopyranosyl-(1→4)-2-azido-3,6-di-*O*-benzyl-2-deoxy-α-D-galactopyranoside (S17)

The reaction was carried out according to the general procedure D using compound **S16** (482 mg, 0.42 mmol), K_2CO_3 (64 mg, 0.46 mmol), KI (70 mg, 0.42 mmol) and $Ph_2BO(CH_2)_2NH_2$ (9.4 mg, 0.042 mmol). The product was purified by column chromatography (pentane:EtOAc = 6:1). Compound **S17** (506 mg, 97% yield) was obtained as colorless syrup. $[\alpha]_D^{25}$ +139.8 (c=1, $CHCl_3$). ^1H NMR (400 MHz, $CDCl_3$) δ 7.46 – 7.12 (m, 30H), 6.36 (d, *J* = 9.5 Hz, 1H), 5.74 (ddt, *J* = 17.0, 10.2, 6.7 Hz, 1H), 5.10 – 4.97 (m, 3H), 4.95 (d, *J* = 3.6 Hz, 1H), 4.91 (d, *J* = 3.6 Hz, 1H), 4.88 (d, *J* = 11.0 Hz, 1H), 4.84 – 4.73 (m, 2H), 4.67 (d, *J* = 12.4 Hz, 1H), 4.55 (td, *J* = 10.3, 3.6 Hz, 1H), 4.51 – 4.36 (m, 3H), 4.35 – 4.16 (m, 7H), 4.13 – 4.00 (m, 2H), 3.93 (t, *J* = 9.5 Hz, 1H), 3.90 – 3.71 (m, 4H), 3.68 – 3.14 (m, 10H), 2.76 (d, *J* = 4.2 Hz, 1H), 2.30 (q, *J* = 6.8 Hz, 2H). ^{13}C NMR (100 MHz, $CDCl_3$) δ 156.03 (*a*d, *J* = 37 Hz, *CF*$_3$*CO*), 138.22, 137.79, 137.33, 137.29, 136.74 (*aromatic* C), 134.41 (C-9), 128.80, 128.49, 128.45, 128.37, 128.34, 128.26, 128.18, 128.16, 128.11, 128.08, 127.99, 127.83, 127.68, 127.64, 127.58, 127.54, 127.48, 127.22, 126.91 (*aromatic* C*H*), 116.83 (C-10), 115.84 (*a*d, *J* = 286 Hz, C*F*$_3$), 98.67, 97.94, 97.21, 79.77, 75.48, 74.97, 74.24, 73.45, 73.19, 73.07, 72.35, 71.78, 71.43, 70.77, 70.45, 70.36, 69.69, 69.06, 68.70, 67.62, 66.42, 66.23, 63.28, 59.37, 58.71, 58.10, 54.60, 49.25, 33.70. HR-MS: Calculated for $C_{66}H_{72}N_7O_{14}F_3$ $[M+Na]^+$: 1266.49816, found: 1266.49820.

3-Butenyl 2-azido-3-*O*-benzyl-2-deoxy-4,6-di-*tert*-butylsilylidene-α-D-galactopyranosyl-(1→4)-2-azido-3,6-di-*O*-benzyl-2-deoxy-α-D-gluco-pyranosyl-(1→4)-3,6-di-*O*-benzyl-2-deoxy-2-trifluoroacetamido-α-D-galactopyranosyl-(1→4)-2-azido-3,6-di-*O*-benzyl-2-deoxy-α-D-galacto pyranoside (23)

The reaction was carried out according to the general procedure B using donor **13** (722 mg, 1.19 mmol) and acceptor **S17** (494 mg, 0.40 mmol). The product was purified by column chromatography (pentane:EtOAc = 8:1). Compound **23** (547 mg, α-only, 83% yield) was obtained as yellow syrup. $[\alpha]_D^{25}$ +126.6 (c=1, $CHCl_3$). ^1H NMR (500 MHz, $CDCl_3$) δ 7.46 – 7.17 (m, 35H), 6.39 (d, *J* = 9.6 Hz, 1H), 5.75 (ddt, *J* = 17.1, 10.2, 6.7 Hz, 1H), 5.52 (d, *J* = 3.6 Hz, 1H), 5.10 – 4.99 (m, 3H), 4.99 – 4.90 (m, 3H), 4.84 (d, *J* = 12.4 Hz, 1H), 4.81 (d, *J* = 3.6 Hz, 1H), 4.78 – 4.72 (m, 2H), 4.68 (d, *J* = 11.5 Hz, 1H), 4.63 – 4.56 (m, 1H), 4.53 (d, *J* = 12.4 Hz, 1H), 4.49 – 4.40 (m, 3H), 4.37 – 4.19 (m, 7H), 4.14 (d, *J* = 11.4 Hz, 1H), 4.12 – 4.03 (m, 2H), 4.00 (dd, *J* = 9.7, 8.5 Hz, 1H), 3.96 – 3.76 (m, 7H), 3.68 – 3.55 (m, 3H), 3.53 – 3.44 (m, 2H), 3.43 – 3.32 (m, 4H), 3.23 (dd, *J* = 8.4, 5.2 Hz, 1H), 3.12 (dd, *J* = 11.3, 1.9 Hz, 1H), 2.32 (qt, *J* = 6.7, 1.3 Hz, 2H), 1.02 (s, 9H), 0.97 (s, 9H). ^{13}C NMR (125 MHz, $CDCl_3$) δ 156.64 (*a*d, *J* = 37 Hz, *CF*$_3$*CO*), 138.22, 137.87, 137.65, 137.62, 137.46, 137.01 (*aromatic* C), 134.56 (C-9), 128.66, 128.51, 128.48, 128.36, 128.27, 128.20, 128.17, 128.05, 128.01, 127.93, 127.89, 127.78, 127.71, 127.56, 127.49, 127.37, 127.14 (*aromatic* C*H*), 117.02 (C-10), 116.02 (*a*d, *J* = 286 Hz, C*F*$_3$), 98.98, 98.14, 97.94, 97.58, 80.94, 75.87, 75.64, 74.97, 74.69, 74.31, 73.64, 73.29, 73.16, 72.93, 71.70, 71.16, 70.98, 70.66, 69.99, 69.89, 68.92, 68.86, 67.89, 67.86, 67.04, 66.71, 66.58, 64.81, 59.61, 58.65, 49.49, 33.88, 27.67, 27.36, 23.39, 20.77. HR-MS: Calculated for $C_{87}H_{103}N_{10}O_{18}F_3Si$ $[M+Na]^+$: 1683.70654, found: 1683.70808.

3-Butenyl 2-azido-3-*O*-benzyl-2-deoxy-α-D-galactopyranosyl-(1→4)-2-azido-3,6-di-*O*-benzyl-2-deoxy-α-D-glucopyranosyl-(1→4)-3,6-di-*O*-benzyl-2-deoxy-2-trifluoroacetamido-α-D-galactopyranosyl-(1→4)-2-azido-3,6-di-*O*-benzyl-2-deoxy-α-D-galactopyranoside (S18)

The reaction was carried out according to the general procedure C using compound **23** (530 mg, 0.32 mmol) and HF/pyridine (70%, 133 µl, 5.1 mmol). The product was purified by column chromatography (pentane:EtOAc = 2:1). Compound **S18** (436 mg, 89% yield) was obtained as white foam. $[\alpha]_D^{25}$ +120.5 (c=1, $CHCl_3$). ^1H NMR (400 MHz, $CDCl_3$) δ 7.48 – 7.12 (m, 35H), 6.46 (d, *J* = 9.6 Hz, 1H), 5.76 (ddt, *J* = 17.0, 10.2, 6.7 Hz, 1H), 5.36 (d, *J* = 3.6 Hz, 1H), 5.12 – 4.99 (m, 3H), 4.94 – 4.87 (m, 3H), 4.84 (d, *J* = 12.4 Hz, 1H), 4.81 (d, *J* = 3.6 Hz, 1H), 4.72 (d, *J* = 12.3 Hz, 1H), 4.68 (s, 2H), 4.57 – 4.39 (m, 4H), 4.37 – 4.19 (m, 7H), 4.18 – 4.01 (m, 5H), 3.97 (dd, *J* = 10.5, 3.0 Hz, 1H), 3.94 – 3.76 (m, 4H), 3.71 (dd, *J* = 10.5, 3.6 Hz, 1H), 3.65 (td, *J* = 6.9, 3.1 Hz, 4H), 3.57 (dd, *J* = 11.0, 2.2 Hz, 1H), 3.50 (dt, *J* = 9.7, 6.5 Hz, 1H), 3.43 – 3.29 (m, 4H), 3.23 (dd, *J* = 8.3, 5.2 Hz, 1H), 3.13 (d, *J* = 11.4 Hz, 1H), 2.67 (d, *J* = 7.1 Hz, 2H), 2.32 (q, *J* = 6.7 Hz, 2H). ^{13}C NMR (100 MHz, $CDCl_3$) δ 156.97 (*a*d, *J* = 37 Hz, *CF*$_3$*CO*), 138.19, 137.97, 137.58, 137.48, 137.40, 137.30, 136.86 (*aromatic* C), 134.53 (C-9), 128.66, 128.65, 128.49, 128.46, 128.33, 128.29, 128.26, 128.17, 128.01, 127.89, 127.84, 127.75, 127.70, 127.55, 127.35, 127.10 (*aromatic* C*H*), 117.03 (C-10), 115.98 (*a*d, *J* = 286 Hz, C*F*$_3$), 99.00, 98.10, 97.99, 97.34, 80.15, 76.36, 75.94, 75.60, 75.05, 74.66, 73.63, 73.27, 73.20, 73.00, 71.81, 71.65, 71.08, 70.96, 70.71,

70.32, 69.92, 68.76, 68.47, 67.86, 67.02, 66.65, 66.31, 64.54, 62.51, 59.61, 59.56, 49.47, 33.85. HR-MS: Calculated for $C_{79}H_{87}N_{10}O_{18}F_3$ $[M+Na]^+$: 1543.60441, found: 1543.60408.

3-Butenyl 2-azido-3,6-di-*O*-benzyl-2-deoxy-*α*-D-galactopyranosyl-(1→4)-2-azido-3,6-di-*O*-benzyl-2-deoxy-*α*-D-glucopyranosyl-(1→4)-3,6-di-*O*-benzyl-2-deoxy-2-trifluoroacetamido-*α*-D-galactopyranosyl-(1→4)-2-azido-3,6-di-*O*-benzyl-2-deoxy-*α*-D-galactopyranoside (S19)

The reaction was carried out according to the general procedure D using compound **S18** (421 mg, 0.28 mmol), K_2CO_3 (42 mg, 0.30 mmol), KI (46 mg, 0.28 mmol) and $Ph_2BO(CH_2)_2NH_2$ (6.2 mg, 0.028 mmol). The product was purified by column chromatography (pentane:EtOAc = 4:1). Compound **S19** (436 mg, 98% yield) was obtained as white foam. $[\alpha]_D^{25}$ +136.2 (c=1, $CHCl_3$). ^1H NMR (500 MHz, $CDCl_3$) δ 7.56 – 7.26 (m, 40H), 6.46 (d, *J* = 9.5 Hz, 1H), 5.87 (ddt, *J* = 17.0, 10.2, 6.7 Hz, 1H), 5.69 (d, *J* = 3.7 Hz, 1H), 5.22 – 5.12 (m, 3H), 5.12 – 5.04 (m, 3H), 4.96 (d, *J* = 12.4 Hz, 1H), 4.91 (d, *J* = 3.6 Hz, 1H), 4.82 (d, *J* = 12.0 Hz, 3H), 4.70 (ddd, *J* = 13.2, 9.7, 3.6 Hz, 1H), 4.64 (d, *J* = 12.4 Hz, 1H), 4.59 – 4.51 (m, 2H), 4.51 – 4.33 (m, 9H), 4.30 – 4.22 (m, 3H), 4.21 – 4.13 (m, 3H), 4.07 – 3.96 (m, 3H), 3.91 (dd, *J* = 10.8, 2.6 Hz, 1H), 3.86 (dd, *J* = 10.6, 3.6 Hz, 1H), 3.79 – 3.41 (m, 11H), 3.39 – 3.31 (m, 2H), 2.88 (s, 1H), 2.49 – 2.38 (m, 2H). ^{13}C NMR (125 MHz, $CDCl_3$) δ 156.52 (*a*d, *J* = 37 Hz, *CF₃CO*), 138.47, 137.92, 137.79, 137.65, 137.56, 137.40, 136.89 (*aromatic* C), 134.51 (C-9), 128.97, 128.60, 128.58, 128.53, 128.43, 128.41, 128.32, 128.25, 128.21, 128.17, 128.14, 128.12, 127.95, 127.91, 127.83, 127.82, 127.71, 127.67, 127.49, 127.34, 127.30, 127.07 (*aromatic* C*H*), 116.95 (C-10), 116.02 (*a*d, *J* = 286 Hz, C*F₃*), 98.89, 98.05, 98.03, 97.42, 80.62, 76.17, 75.57, 74.61, 74.57, 74.44, 73.57, 73.56, 73.23, 72.95, 72.70, 71.69, 71.58, 70.94, 70.82, 70.48, 69.90, 69.42, 68.86, 68.83, 67.77, 66.70, 66.67, 66.43, 64.86, 59.53, 59.21, 58.60, 58.19, 54.74, 49.39, 33.81. HR-MS: Calculated for $C_{86}H_{93}N_{10}O_{18}F_3$ $[M+Na]^+$: 1633.65136, found: 1633.65245.

Pentasaccharide 24

The reaction was carried out according to the general procedure B using donor **14** (542 mg, 0.80 mmol) and acceptor **S19** (430 mg, 0.27 mmol). The product was purified by column chromatography (pentane:EtOAc = 7:1). Compound **24** (501 mg, α-only, 89% yield) was obtained as yellow foam. $[\alpha]_D^{25}$ +150.6 (c=0.5, $CHCl_3$). ^1H NMR (500 MHz, $CDCl_3$) δ 7.63 – 7.21 (m, 45H), 6.42 (d, *J* = 9.6 Hz, 2H), 5.86 (ddt, *J* = 17.0, 10.3, 6.7 Hz, 1H), 5.59 (d, *J* = 3.7 Hz, 1H), 5.22 – 5.02 (m, 7H), 4.94 (d, *J* = 12.4 Hz, 1H), 4.90 (d, *J* = 3.6 Hz, 1H), 4.86 – 4.71 (m, 5H), 4.70 – 4.33 (m, 14H), 4.32 – 4.15 (m, 6H), 4.15 – 4.04 (m, 3H), 4.04 – 3.93 (m, 2H), 3.90 (dd, *J* = 10.7, 2.5 Hz, 1H), 3.81 (d, *J* = 12.6 Hz, 1H), 3.78 – 3.66 (m, 3H), 3.65 – 3.56 (m, 4H), 3.54 (dd, *J* = 10.1, 3.3 Hz, 1H), 3.51 – 3.40 (m, 3H), 3.40 – 3.33 (m, 2H), 3.32 – 3.23 (m, 2H), 2.48 – 2.37 (m, 2H), 1.20 (s, 10H), 1.10 (s, 9H). ^{13}C NMR (125 MHz, $CDCl_3$) δ 156.71 (*a*d, *J* = 37 Hz, 2x*CF₃CO*), 138.38, 138.17, 137.85, 137.52, 137.45, 137.39, 136.96, 136.78 (*aromatic* C), 134.51 (C-9), 128.61, 128.55, 128.53, 128.50, 128.47, 128.44, 128.27, 128.25, 128.15, 128.07, 127.99, 127.85, 127.78, 127.76, 127.68, 127.66, 127.48, 127.42, 127.20, 127.08, 126.63 (*aromatic* C*H*), 116.97 (C-10), 115.89 (*a*d, *J* = 286 Hz, 2xC*F₃*), 98.82, 98.06, 97.47, 97.39, 96.90, 79.98, 76.10, 75.59, 74.63, 74.29, 73.90, 73.49, 73.27, 73.03, 72.51, 71.77, 71.62, 70.97, 70.62, 70.60, 69.87, 69.54, 69.34, 69.22, 68.81, 68.73, 67.78, 67.75, 66.99, 66.65, 66.44, 66.17, 64.72, 60.20, 59.56, 49.37, 48.29, 33.83, 27.59, 27.41, 23.30, 20.72. HR-MS: Calculated for $C_{109}H_{125}N_{11}O_{23}F_6Si$ $[M+ NH_4]^+$: 2115.89615, found: 2120.89919.

Pentasaccharide S20

The reaction was carried out according to the general procedure C using compound **24** (490 mg, 0.23 mmol) and HF/pyridine (70%, 97 µl, 3.7 mmol). The product was purified by column chromatography (pentane:EtOAc = 3:2). Compound **S20** (395 mg, 86% yield) was obtained as white foam. $[\alpha]_D^{25}$ +117.6 (c=0.5, $CHCl_3$). ^1H NMR (500 MHz, $CDCl_3$) δ 7.46 (d, *J* = 7.2 Hz, 2H), 7.43 – 7.09 (m, 43H), 6.44 (d, *J* = 9.5 Hz, 1H), 6.40 (d, *J* = 9.6 Hz, 1H), 5.74 (ddt, *J* = 17.1, 10.2, 6.7 Hz, 1H), 5.48 (d, *J* = 3.7 Hz, 1H), 5.09 – 4.90 (m, 7H), 4.87 – 4.74 (m, 3H), 4.72 – 4.48 (m, 5H), 4.46 – 4.20 (m, 12H), 4.19 – 4.01 (m, 8H), 3.99 – 3.91 (m, 2H), 3.90 – 3.82 (m, 2H), 3.78 (dd, *J* = 10.7, 2.6 Hz, 1H), 3.66 – 3.52 (m, 3H), 3.52 – 3.39 (m, 5H), 3.39 – 3.27 (m, 4H), 3.27 – 3.19 (m, 2H), 3.19 – 3.09 (m, 2H), 2.90 (s, 1H), 2.36 – 2.25 (m, 2H), 2.02 (s, 1H). ^{13}C NMR (125 MHz, $CDCl_3$) δ 156.78 (*a*d, *J* = 37 Hz, 2x*CF₃CO*), 138.34, 138.16, 137.60, 137.55, 137.42, 137.40, 137.05, 136.98, 136.84 (*aromatic* C), 134.53 (C-9), 128.59, 128.58, 128.54, 128.49, 128.47, 128.42, 128.22, 128.17, 128.14, 128.12, 128.05, 127.96, 127.88, 127.85, 127.75, 127.67, 127.46, 127.42, 127.25, 127.10, 126.99 (*aromatic* C*H*), 116.95 (C-10), 115.93 (*a*d, *J* = 286 Hz, C*F₃*), 98.80, 98.05, 97.53, 97.43, 97.13, 80.03, 75.92, 75.58, 74.84, 74.58, 74.24, 73.82, 73.48, 73.45, 73.25, 73.05, 72.58, 71.97, 71.65, 71.07, 70.84, 70.60, 70.58, 69.86, 69.29, 69.24, 68.84, 68.71, 67.79, 66.67, 66.24, 64.71, 62.45, 60.11, 59.56, 49.41, 48.83, 33.81. HR-MS: Calculated for $C_{101}H_{109}N_{11}O_{23}F_6$ $[M+ NH_4]^+$: 1975.79402, found: 1975.79608.

Pentasaccharide S21

The reaction was carried out according to the general procedure D using compound **S20** (389 mg, 0.2 mmol), K_2CO_3 (30 mg, 0.22 mmol), KI (33 mg, 0.2 mmol) and $Ph_2BO(CH_2)_2NH_2$ (4.5 mg, 0.02 mmol). The product was purified by column chromatography (pentane:EtOAc = 4:1). Compound **S21** (369 mg, 90% yield) was obtained as yellow syrup. $[\alpha]_D^{25}$ +143.7 (c=0.5, $CHCl_3$). ^{1}H NMR (500 MHz, $CDCl_3$) δ 7.45 (d, *J* = 7.6 Hz, 2H), 7.41 – 7.06 (m, 48H), 6.44 (dd, *J* = 14.6, 9.5 Hz, 2H), 5.73 (ddt, *J* = 17.0, 10.2, 6.7 Hz, 1H), 5.47 (d, *J* = 3.7 Hz, 1H), 5.08 – 4.90 (m, 7H), 4.86 – 4.76 (m, 3H), 4.57 (ddd, *J* = 53.0, 25.7, 12.7 Hz, 6H), 4.42 – 4.00 (m, 21H), 3.98 – 3.82 (m, 4H), 3.78 (dd, *J* = 10.6, 2.5 Hz, 1H), 3.60 (dq, *J* = 13.3, 6.6 Hz, 3H), 3.55 – 3.29 (m, 9H), 3.29 – 3.09 (m, 5H), 2.95 (s, 1H), 2.29 (q, *J* = 6.8 Hz, 2H). ^{13}C NMR (125 MHz, $CDCl_3$) δ 156.55 (*a*d, *J* = 37 Hz, 2x*CF_3CO*), 138.29, 138.07, 137.72, 137.66, 137.48, 137.34, 137.33, 137.24, 136.90 (*aromatic* C), 134.44 (C-9), 128.86, 128.49, 128.45, 128.40, 128.37, 128.36, 128.33, 128.31, 128.29, 128.21, 128.11, 128.05, 128.02, 127.99, 127.87, 127.85, 127.73, 127.66, 127.62, 127.57, 127.55, 127.49, 127.41, 127.36, 127.30, 127.15, 127.09, 126.99 (*aromatic* C*H*), 116.84 (C-10), 115.83 (*a*d, *J* = 286 Hz, 2xC*F_3*), 98.70, 97.98, 97.48, 97.33, 97.22, 79.87, 75.75, 75.52, 74.83, 74.49, 74.18, 73.72, 73.37, 73.24, 73.14, 72.93, 72.47, 71.75, 71.54, 70.98, 70.86, 70.52, 70.49, 70.47, 69.77, 69.25, 69.07, 68.78, 68.70, 68.61, 67.68, 66.60, 66.41, 66.19, 65.56, 64.58, 59.95, 59.47, 58.61, 58.12, 54.68, 49.34, 48.89, 33.72. HR-MS: Calculated for $C_{108}H_{115}N_{11}O_{23}F_6$ $[M+Na]^+$: 2070.79637, found: 2070.79976.

Hexasaccharide 25

The reaction was carried out according to the general procedure B using donor **13** (346 mg, 0.57 mmol) and acceptor **S21** (390 mg, 0.19 mmol). The product was purified by column chromatography (pentane:EtOAc = 6:1). Compound **25** (415 mg, α-only, 88% yield) was obtained as white foam. $[\alpha]_D^{25}$ +130.6 (c=0.5, $CHCl_3$). ^{1}H NMR (500 MHz, $CDCl_3$) δ 7.50 – 7.09 (m, 57H), 7.03 – 6.96 (m, 1H), 6.31 (d, *J* = 9.5 Hz, 1H), 6.25 (d, *J* = 9.7 Hz, 1H), 5.73 (ddt, *J* = 17.0, 10.2, 6.7 Hz, 1H), 5.41 (d, *J* = 3.7 Hz, 1H), 5.08 – 4.89 (m, 9H), 4.85 – 4.72 (m, 3H), 4.70 – 4.43 (m, 8H), 4.37 – 4.18 (m, 14H), 4.17 – 3.93 (m, 10H), 3.92 – 3.82 (m, 3H), 3.81 – 3.52 (m, 8H), 3.50 – 3.19 (m, 9H), 3.18 – 3.06 (m, 3H), 2.29 (q, *J* = 6.6 Hz, 2H), 1.03 (s, 10H), 0.98 (s, 9H). ^{13}C NMR (125 MHz, $CDCl_3$) δ 156.60 (*a*d, *J* = 37 Hz, 2x*CF_3CO*), 138.34, 138.10, 137.89, 137.82, 137.51, 137.39, 137.35, 137.14, 137.06, 136.87, 136.71 (*aromatic* C), 134.45 (C-9), 128.55, 128.50, 128.48, 128.42, 128.40, 128.37, 128.29, 128.16, 128.14, 128.08, 128.01, 127.91, 127.79, 127.69, 127.66, 127.62, 127.48, 127.36, 127.34, 127.12, 127.00, 126.49 (*aromatic* C*H*), 116.92 (C-10), 115.84 (*a*d, *J* = 286 Hz, C*F_3*), 98.85, 98.34, 98.03, 97.77, 97.34, 97.29, 80.22, 76.19, 75.76, 75.55, 74.73, 74.42, 74.37, 74.01, 73.44, 73.42, 73.18, 72.91, 72.70, 71.64, 71.56, 70.91, 70.75, 70.68, 70.56, 70.52, 70.14, 69.83, 69.61, 69.56, 69.21, 68.75, 68.65, 67.73, 67.36, 67.07, 66.63, 66.34, 65.97, 64.78, 59.87, 59.50, 58.44, 49.33, 49.10, 33.78, 27.55, 27.39, 23.20, 20.67. MALDI-MS: Calculated for $C_{129}H_{146}N_{14}O_{27}F_6Si$ [M+Na]+: 2488.0048, found: 2487.9742.

Hexasaccharide S22

The reaction was carried out according to the general procedure C using compound **25** (409 mg, 0.17 mmol) and HF/pyridine (70%, 69 µl, 2.65 mmol). The product was purified by column chromatography (pentane:EtOAc = 3:2). Compound **S22** (347 mg, 90% yield) was obtained as white foam. $[\alpha]_D^{25}$ +147 (c=0.5, $CHCl_3$). ^{1}H NMR (500 MHz, $CDCl_3$) δ 7.52 – 7.09 (m, 55H), 7.02 (td, *J* = 6.3, 2.9 Hz, 1H), 6.39 (t, *J* = 11.1 Hz, 2H), 5.81 – 5.68 (m, 1H), 5.42 (d, *J* = 3.7 Hz, 1H), 5.09 – 4.76 (m, 11H), 4.73 – 4.61 (m, 4H), 4.61 – 4.48 (m, 3H), 4.44 (td, *J* = 10.4, 3.6 Hz, 1H), 4.40 – 4.24 (m, 9H), 4.23 – 4.08 (m, 10H), 4.08 – 4.01 (m, 2H), 4.01 – 3.91 (m, 5H), 3.90 – 3.82 (m, 2H), 3.81 – 3.73 (m, 2H), 3.68 – 3.53 (m, 4H), 3.51 – 3.27 (m, 9H), 3.27 – 3.19 (m, 2H), 3.18 – 3.05 (m, 3H), 2.84 (s, 1H), 2.35 – 2.24 (m, 2H). ^{13}C NMR (125 MHz, $CDCl_3$) δ 156.77 (*a*d, *J* = 37 Hz, *CF_3CO*), 138.36, 138.13, 137.75, 137.56, 137.46, 137.45, 137.38, 137.22, 136.95, 136.87 (*aromatic* C), 134.49 (C-9), 128.59, 128.56, 128.53, 128.49, 128.46, 128.44, 128.41, 128.39, 128.34, 128.20, 128.18, 128.14, 128.08, 128.04, 127.92, 127.88, 127.81, 127.67, 127.65, 127.58, 127.39, 127.35, 127.30, 127.16, 127.07, 126.71 (*aromatic* C*H*), 116.92 (C-10), 115.87 (*a*d, *J* = 286 Hz, C*F_3*), 98.84, 98.75, 98.05, 97.80, 97.41, 97.31, 80.24, 76.38, 76.02, 75.55, 74.70, 74.37, 74.34, 74.01, 73.45, 73.38, 73.21, 73.13, 72.97, 72.72, 71.76, 71.73, 71.63, 71.59, 71.04, 70.81, 70.69, 70.59, 69.84, 69.73, 69.20, 69.08, 68.83, 68.64, 67.76, 67.45, 66.67, 66.54, 66.45, 66.09, 64.79, 62.60, 59.91, 59.53, 59.41, 49.40, 49.32, 33.79. MALDI-MS: Calculated for $C_{121}H_{130}N_{14}O_{27}F_6$ [M+Na]+: 2347.9026, found: 2347.8750.

Heptasaccharide 26

The reaction was carried out according to the general procedure B using donor **14** (381 mg, 0.56 mmol) and acceptor **S23** (340 mg, 0.14 mmol). The product was purified by column chromatography (pentane:EtOAc = 6:1). Compound **26** (358 mg, α-only, 88% yield) was obtained as yellow foam. $[\alpha]_D^{25}$ +157.6 (c=0.5, $CHCl_3$). ^{1}H NMR (500 MHz, $CDCl_3$) δ 7.52 – 7.02 (m, 69H), 6.42 – 6.34 (m, 2H), 6.31 (d, *J* = 9.4 Hz, 1H), 5.73 (ddt, *J* = 17.0, 10.2, 6.7 Hz, 1H), 5.43 (d, *J* = 3.6 Hz, 1H), 5.08 – 4.87 (m, 9H), 4.86 – 4.76 (m, 3H), 4.72 (dd, *J* = 12.0, 7.2 Hz, 2H), 4.68 –

4.42 (m, 10H), 4.41 – 4.19 (m, 15H), 4.12 (d, *J* = 11.1 Hz, 4H), 4.09 – 3.82 (m, 12H), 3.81 – 3.72 (m, 2H), 3.71 – 3.53 (m, 5H), 3.50 – 2.96 (m, 16H), 2.35 – 2.26 (m, 2H), 1.07 (s, 9H), 0.98 (s, 9H). ^{13}C NMR (125 MHz, $CDCl_3$) δ 156.69 (*a*d, *J* = 37 Hz, 3x*CF₃CO*), 138.32, 138.13, 137.87, 137.80, 137.62, 137.52, 137.41, 137.37, 137.29, 137.21, 136.90, 136.84 (*aromatic* C), 134.47 (C-9), 128.56, 128.52, 128.49, 128.46, 128.44, 128.41, 128.39, 128.31, 128.28, 128.17, 128.16, 128.11, 128.09, 128.05, 127.97, 127.93, 127.81, 127.79, 127.74, 127.72, 127.67, 127.64, 127.39, 127.36, 127.16, 127.09, 127.02, 126.73, 126.57 (*aromatic* C*H*), 116.94 (C-10), 115.83 (*a*d, *J* = 286 Hz, C*F₃*), 98.83, 98.31, 98.05, 97.75, 97.49, 97.40, 96.73, 80.24, 76.20, 76.05, 75.57, 74.70, 74.51, 74.31, 73.97, 73.44, 73.39, 73.20, 73.05, 72.94, 72.90, 72.68, 71.85, 71.61, 71.19, 71.06, 70.99, 70.95, 70.64, 70.58, 69.83, 69.57, 69.49, 69.36, 69.27, 68.79, 68.56, 67.76, 67.72, 66.91, 66.63, 66.49, 66.36, 66.14, 65.74, 64.78, 60.15, 59.92, 59.51, 49.37, 49.20, 48.21, 33.80, 27.56, 27.38, 23.25, 20.67. MALDI-MS: Calculated for $C_{151}H_{168}N_{15}O_{32}F_9Si$ [M+Na]+: 2925.1498, found: 2925.1018.

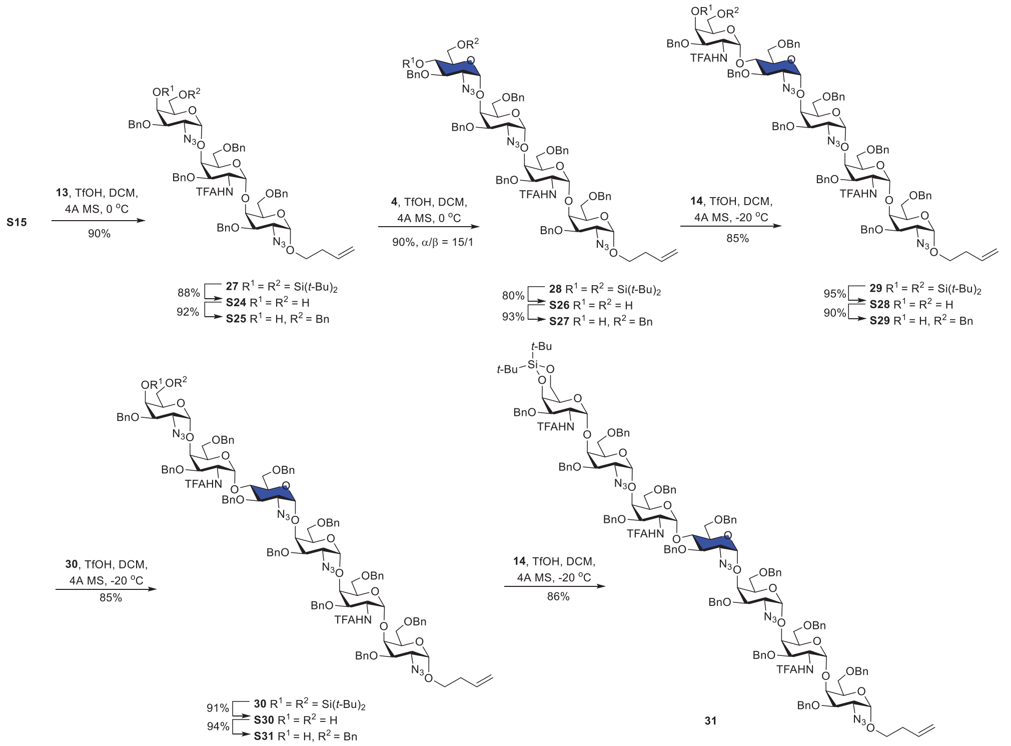

3-Butenyl 2-azido-3-*O*-benzyl-2-deoxy-4,6-di-*tert*-butylsilylidene-α-D-galactopyranosyl-(1→4)-3,6-di-*O*-benzyl-2-deoxy-2-trifluoro-acetamido-α-D-galactopyranosyl-(1→4)-2-azido-3,6-di-*O*-benzyl-2-deoxy-α-D-galactopyranoside (27)

The reaction was carried out according to the general procedure B using donor **13** (1.07 g, 1.77 mmol) and acceptor **S15** (620 mg, 0.71 mmol). The product was purified by column chromatography (pentane:EtOAc = 10:1). Compound **27** (828 mg, α-only, 90% yield) was obtained as white foam. $[\alpha]_D^{25}$ +176.3 (c=1, $CHCl_3$). ^{1}H NMR (400 MHz, $CDCl_3$) δ 7.50 – 7.14 (m, 26H), 6.31 (d, *J* = 9.7 Hz, 1H), 5.76 (ddt, *J* = 17.0, 10.2, 6.8 Hz, 1H), 5.12 – 4.99 (m, 2H), 4.95 (dd, *J* = 5.6, 3.6 Hz, 2H), 4.87 (d, *J* = 12.3 Hz, 1H), 4.81 – 4.62 (m, 4H), 4.59 – 4.23 (m, 9H), 4.20 – 4.06 (m, 2H), 4.02 – 3.92 (m, 2H), 3.87 (dd, *J* = 9.0, 5.9 Hz, 1H), 3.85 – 3.60 (m, 6H), 3.57 (dd, *J* = 11.1, 2.4 Hz, 1H), 3.55 – 3.45 (m, 1H), 3.43 – 3.26 (m, 3H), 3.16 (dd, *J* = 8.4, 5.2 Hz, 1H), 2.33 (qt, *J* = 6.8, 1.4 Hz, 2H), 1.03 (s, 9H), 1.00 (s, 9H). ^{13}C NMR (100 MHz, $CDCl_3$) δ 156.96 (*a*d, *J* = 37 Hz, *CF₃*CO), 137.99, 137.49, 137.24, 137.20, 136.89 (*aromatic* C), 134.58 (C-9), 128.70, 128.69, 128.65, 128.54, 128.36, 128.29, 128.20, 128.13, 127.90, 127.87, 127.83, 127.28, 127.06 (*aromatic* C*H*), 117.06 (C-10), 115.95 (*a*d, *J* = 286 Hz, C*F₃*), 98.55, 98.14, 97.51, 76.02, 75.80, 74.48, 73.76, 73.41, 71.68, 70.94, 70.69, 70.64, 70.37, 69.88, 69.67, 68.89, 67.89, 67.55, 67.23, 66.50, 66.44, 59.55, 58.60, 49.27, 33.91, 27.68, 27.50, 23.36, 20.80. HR-MS: Calculated for $C_{67}H_{82}N_7O_{14}F_3Si$ $[M+Na]^+$: 1316.55333, found: 1316.55390.

3-Butenyl 2-azido-3-*O*-benzyl-2-deoxy-α-D-galactopyranosyl-(1→4)-3,6-di-*O*-benzyl-2-deoxy-2-trifluoroacetamido-α-D-galactopyranosyl-(1→4)-2-azido-3,6-di-*O*-benzyl-2-deoxy-α-D-galactopyranoside (S24)

The reaction was carried out according to the general procedure C using compound **27** (910 mg, 0.70 mmol) and HF/pyridine (70%, 292 µl, 11.2 mmol). The product was purified by column chromatography (pentane:EtOAc = 2:1). Compound **S24** (710 mg, 88% yield) was obtained as white foam. $[\alpha]_D^{25}$ +155.4 (c=1, $CHCl_3$). ^{1}H NMR (500 MHz, $CDCl_3$) δ 7.54 – 7.20 (m, 25H), 6.54 (d, *J* = 10.3, 3.9 Hz, 1H), 5.82 (ddt, *J* = 17.0, 10.4, 6.7 Hz, 1H), 5.18 – 5.06 (m, 2H), 5.04 (d, *J* = 3.6 Hz, 1H), 5.01 (d, *J* = 3.6 Hz, 1H), 4.93 – 4.73 (m, 5H), 4.62 – 4.54 (m, 2H), 4.49 (s, 2H), 4.42 – 4.27 (m, 5H), 4.27 – 4.22 (m, 1H), 4.20 – 4.12 (m, 2H), 4.06 (dd, *J* = 10.5, 3.0 Hz, 1H), 3.97 – 3.81 (m, 3H), 3.76 (dd, *J* = 10.5, 3.5 Hz, 1H), 3.73 – 3.64 (m, 2H), 3.63 – 3.49 (m, 3H), 3.48 – 3.34 (m, 3H), 3.28 (dd, *J* = 8.6, 5.3 Hz, 1H), 2.95 (d, *J* = 8.1 Hz, 1H), 2.44 (s, 1H), 2.38 (q, *J* = 6.7 Hz, 2H). ^{13}C NMR (125 MHz, $CDCl_3$) δ 157.11 (*a*d, *J* = 37 Hz, *CF*$_3$*CO*), 137.38, 137.37, 137.35, 137.20, 136.91 (*aromatic* C), 134.52 (C-9), 128.65, 128.61, 128.41, 128.27, 128.22, 128.17, 128.14, 128.09, 128.00, 127.95, 127.87, 127.44, 127.14 (*aromatic* C*H*), 116.98 (C-10), 115.81 (*a*d, *J* = 286 Hz, C*F*$_3$), 98.86, 98.07, 97.42, 76.53, 75.51, 74.37, 73.62, 73.26, 71.84, 71.69, 71.65, 71.10, 70.81, 69.94, 69.16, 68.82, 67.81, 67.56, 66.53, 66.49, 62.71, 59.51, 59.47, 49.40, 33.83. HR-MS: Calculated for $C_{59}H_{66}N_7O_{14}F_3$ $[M+Na]^+$: 1176.45121, found: 1176.45086.

3-Butenyl 2-azido-3,6-di-*O*-benzyl-2-deoxy-α-D-galactopyranosyl-(1→4)-3,6-di-*O*-benzyl-2-deoxy-2-tri-fluoroacetamido-α-D-galacto-pyranosyl-(1→4)-2-azido-3,6-di-*O*-benzyl-2-deoxy-α-D-galactopyranoside (S25)

The reaction was carried out according to the general procedure D using compound **S24** (983 mg, 0.85 mmol), K_2CO_3 (129 mg, 0.94 mmol), KI (141 mg, 0.85 mmol) and $Ph_2BO(CH_2)_2NH_2$ (19 mg, 0.085 mmol). The product was purified by column chromatography (pentane:EtOAc = 4:1). Compound **S25** (973 mg, 92% yield) was obtained as white foam. $[\alpha]_D^{25}$ +141.7 (c=1, $CHCl_3$). ^{1}H NMR (400 MHz, $CDCl_3$) δ 7.48 – 7.14 (m, 32H), 6.32 (d, *J* = 9.5 Hz, 1H), 5.75 (ddt, *J* = 17.0, 10.2, 6.8 Hz, 1H), 5.10 – 5.00 (m, 2H), 4.98 (d, *J* = 3.7 Hz, 1H), 4.95 (d, *J* = 3.7 Hz, 1H), 4.82 (d, *J* = 12.4 Hz, 1H), 4.79 – 4.66 (m, 4H), 4.52 (t, *J* = 12.7 Hz, 2H), 4.42 (s, 2H), 4.37 (q, *J* = 5.3, 3.9 Hz, 1H), 4.34 – 4.17 (m, 7H), 4.07 (d, *J* = 1.5 Hz, 2H), 4.00 (dd, *J* = 10.5, 2.9 Hz, 1H), 3.90 – 3.80 (m, 2H), 3.77 (dd, *J* = 10.6, 3.1 Hz, 2H), 3.67 – 3.55 (m, 2H), 3.53 – 3.43 (m, 2H), 3.40 – 3.25 (m, 4H), 3.19 (dd, *J* = 8.5, 5.2 Hz, 1H), 3.09 (s, 1H), 2.31 (q, *J* = 6.7 Hz, 2H). ^{13}C NMR (100 MHz, $CDCl_3$) δ 156.86 (*a*d, *J* = 37 Hz, *CF*$_3$*CO*), 137.61, 137.55, 137.47, 137.39, 137.37, 136.93 (*aromatic* C), 134.53 (C-9), 128.98, 128.61, 128.55, 128.52, 128.44, 128.42, 128.35, 128.31, 128.22, 128.14, 128.03, 128.01, 127.94, 127.85, 127.78, 127.72, 127.53, 127.24, 127.08 (*aromatic* C*H*), 116.98 (C-10), 115.92 (*a*d, *J* = 286 Hz, C*F*$_3$), 98.94, 98.04, 97.42, 76.56, 75.62, 74.20, 73.61, 73.46, 73.25, 71.62, 71.50, 71.41, 71.00, 70.74, 69.89, 69.77, 68.84, 68.32, 67.78, 66.88, 66.50, 59.52, 59.36, 49.33, 33.84. HR-MS: Calculated for $C_{66}H_{72}N_7O_{14}F_3$ $[M+Na]^+$: 1266.49816, found: 1266.49778.

3-Butenyl 2-azido-3-*O*-benzyl-2-deoxy-4,6-di-*tert*-butylsilylidene-α-D-glucopyranosyl-(1→4)-2-azido-3,6-di-*O*-benzyl-2-deoxy-α-D-galacto-pyranosyl-(1→4)-3,6-di-*O*-benzyl-2-deoxy-2-trifluoroacetamido-α-D-galactopyranosyl-(1→4)-2-azido-3,6-di-*O*-benzyl-2-deoxy-α-D-galacto pyranoside (28)

The reaction was carried out according to the general procedure A. The donor **4** (585 mg, 0.96 mmol) and acceptor **S25** (400 mg, 0.32 mmol) were co-evaporated with toluene (three times). The residue was dissolved in dry 4 ml DCM under nitrogen and stirred over fresh flame-dried molecular sieves 4Å. The solution was cooled to -10 ºC, after which TfOH (9 µl, 0.01 mmol) was added. The reaction was stirred at -10 ºC for overnight. Then the reaction was quenched with Et_3N, diluted with DCM, washed with saturated $NaHCO_3$ and brine. The organic phase was dried with anhydrous $MgSO_4$, filtered and concentrated *in vacuo*. The product was purified by silica gel column chromatography (pentane:EtOAc = 8:1). Compound **28** (487 mg, α-only, 90% yield) was obtained as yellow syrup. $[\alpha]_D^{25}$ +134.9 (c=1, $CHCl_3$). ^{1}H NMR (400 MHz, $CDCl_3$) δ 7.47 – 7.16 (m, 39H), 6.28 (d, *J* = 9.6 Hz, 1H), 5.75 (ddt, *J* = 17.0, 10.2, 6.7 Hz, 1H), 5.13 – 4.98 (m, 3H), 4.96 (d, *J* = 3.8 Hz, 1H), 4.94 (d, *J* = 3.7 Hz, 1H), 4.88 – 4.70 (m, 6H), 4.61 (d, *J* = 12.0 Hz, 1H), 4.55 – 4.19 (m, 12H), 4.17 – 4.02 (m, 3H), 3.94 (dd, *J* = 11.7, 3.3 Hz, 3H), 3.88 – 3.74 (m, 6H), 3.72 – 3.56 (m, 5H), 3.48 (dt, *J* = 9.7, 6.5 Hz, 1H), 3.42 – 3.25 (m, 3H), 3.22 – 3.13 (m, 2H), 3.08 (dd, *J* = 8.7, 5.2 Hz, 1H), 2.36 – 2.26 (m, 2H), 1.03 (s, 9H), 0.98 (s, 9H). ^{13}C NMR (100 MHz, $CDCl_3$) δ 156.79 (*a*d, *J* = 37 Hz, *CF*$_3$*CO*), 138.23, 137.72, 137.60, 137.44, 137.32, 136.98 (*aromatic* C), 134.57 (C-9), 128.70, 128.64, 128.56, 128.50, 128.46, 128.44, 128.38, 128.34, 128.30, 128.27, 128.25, 128.19, 128.15, 128.10, 127.98, 127.90, 127.87, 127.79, 127.76, 127.66, 127.62, 127.24, 127.15, 127.11 (*aromatic* C*H*), 117.02 (C-10), 115.86 (*a*d, *J* = 286 Hz, C*F*$_3$), 98.64, 98.47, 98.11, 97.53, 79.33, 79.24, 76.28, 75.60, 75.51, 74.59, 73.66, 73.28, 73.06, 72.99, 72.24, 71.64, 71.16, 71.10, 70.74, 69.91, 69.07, 68.91, 67.82, 66.77, 66.71, 66.59, 66.38, 66.07, 62.99, 60.27, 59.54, 49.33, 33.89, 27.45, 27.19, 22.64, 20.04. HR-MS: Calculated for $C_{87}H_{103}N_{10}O_{18}F_3Si$ $[M+Na]^+$: 1683.70654, found: 1683.70788.

3-Butenyl 2-azido-3-*O*-benzyl-2-deoxy-α-D-glucopyranosyl-(1→4)-2-azido-3,6-di-*O*-benzyl-2-deoxy-α-D-galactopyranosyl-(1→4)-3,6-di-*O*-benzyl-2-deoxy-2-trifluoroacetamido-α-D-galactopyranosyl-(1→4)-2-azido-3,6-di-*O*-benzyl-2-deoxy-α-D-galactopyranoside (S26)

The reaction was carried out according to the general procedure C using compound **28** (638 mg, 0.38 mmol) and HF/pyridine (70%, 160 µl, 6.14 mmol). The product was purified by column chromatography (pentane:EtOAc = 3:1). Compound **S26** (469 mg, 80% yield) was obtained as white foam. $[\alpha]_D^{25}$ +187.5 (c=1, $CHCl_3$). ^{1}H NMR (400 MHz, $CDCl_3$) δ 7.44 – 7.14 (m, 37H), 6.52 (d, *J* = 9.6 Hz, 1H), 5.74 (ddt, *J* = 17.1, 10.2, 6.7 Hz, 1H), 5.10 – 4.98 (m, 3H), 4.95 (d, *J* = 3.7 Hz, 1H), 4.90 (d, *J* = 3.7 Hz, 1H), 4.87 (s, 2H), 4.82 (d, *J* = 4.3 Hz, 1H), 4.81 – 4.76 (m, 2H), 4.72 (d, *J* = 12.7 Hz, 1H), 4.65 (d, *J* = 11.9 Hz, 1H), 4.56 (td, *J* = 10.4, 3.6 Hz, 1H), 4.49 (d, *J* = 12.4 Hz, 1H), 4.46 – 4.37 (m, 3H), 4.34 – 4.23 (m, 5H), 4.14 – 3.97 (m, 5H), 3.95 – 3.71 (m, 6H), 3.69 – 3.57 (m, 4H), 3.48 (dt, *J* = 9.7, 6.5 Hz, 1H), 3.41 – 3.26 (m, 3H), 3.20 (dq, *J* = 8.4, 5.0, 4.6 Hz, 2H), 3.15 – 3.02 (m, 4H), 2.31 (q, *J* = 6.7 Hz, 2H). ^{13}C NMR (100 MHz, $CDCl_3$) δ 156.94 (*a*d, *J* = 37 Hz, *CF*$_3$*C*O), 138.21, 137.63, 137.59, 137.39, 137.34, 136.93 (*aromatic* C), 134.53 (C-9), 128.61, 128.54, 128.49, 128.45, 128.37, 128.35, 128.21, 128.14, 128.02, 128.00, 127.92, 127.87, 127.86, 127.78, 127.69, 127.22, 127.12, 126.90 (*aromatic* C*H*), 117.00 (C-10), 115.95 (*a*d, *J* = 286 Hz, C*F*$_3$), 98.79, 98.28, 98.08, 97.61, 79.64, 76.04, 75.55, 75.06, 74.52, 73.61, 73.27, 72.97, 72.15, 71.71, 71.68, 71.63, 71.21, 71.18, 70.81, 70.75, 69.82, 68.98, 68.92, 67.81, 66.54, 66.44, 66.23, 63.34, 61.97, 59.99, 59.48, 49.36, 33.84. HR-MS: Calculated for $C_{79}H_{87}N_{10}O_{18}F_3$ $[M+Na]^+$: 1543.60441, found: 1543.60491.

3-Butenyl 2-azido-3,6-di-*O*-benzyl-2-deoxy-*α*-D-glucopyranosyl-(1→4)-2-azido-3,6-di-*O*-benzyl-2-deoxy-*α*-D-galactopyranosyl-(1→4)-3,6-di-*O*-benzyl-2-deoxy-2-trifluoroacetamido-*α*-D-galactopyranosyl-(1→4)-2-azido-3,6-di-*O*-benzyl-2-deoxy-*α*-D-galactopyranoside (S27)

The reaction was carried out according to the general procedure D using compound **S26** (452 mg, 0.30 mmol), K_2CO_3 (45 mg, 0.33 mmol), KI (49 mg, 0.30 mmol) and $Ph_2BO(CH_2)_2NH_2$ (6.7 mg, 0.03 mmol). The product was purified by column chromatography (pentane:EtOAc = 5:1). Compound **S27** (445 mg, 93% yield) was obtained as white foam. $[\alpha]_D^{25}$ +147.6 (c=0.5, $CHCl_3$). ^{1}H NMR (500 MHz, $CDCl_3$) δ 7.42 – 7.11 (m, 43H), 6.36 (d, *J* = 9.6 Hz, 1H), 5.80 – 5.69 (m, 1H), 5.09 – 4.99 (m, 3H), 4.96 (d, *J* = 3.7 Hz, 2H), 4.92 (d, *J* = 10.9 Hz, 1H), 4.88 – 4.83 (m, 2H), 4.80 (d, *J* = 12.4 Hz, 1H), 4.76 (d, *J* = 3.6 Hz, 1H), 4.72 (d, *J* = 12.7 Hz, 1H), 4.62 (d, *J* = 11.8 Hz, 1H), 4.56 (ddd, *J* = 13.3, 9.8, 3.7 Hz, 1H), 4.48 (d, *J* = 12.4 Hz, 1H), 4.45 – 4.36 (m, 3H), 4.36 – 4.23 (m, 5H), 4.18 (d, *J* = 12.0 Hz, 1H), 4.13 – 4.03 (m, 6H), 4.01 (dd, *J* = 11.0, 2.6 Hz, 1H), 3.88 – 3.70 (m, 6H), 3.67 (dd, *J* = 11.0, 3.6 Hz, 1H), 3.65 – 3.56 (m, 2H), 3.47 (dt, *J* = 9.7, 6.5 Hz, 1H), 3.39 – 3.25 (m, 3H), 3.25 – 3.18 (m, 2H), 3.15 (dd, *J* = 8.7, 5.2 Hz, 1H), 3.09 (dd, *J* = 10.3, 3.3 Hz, 1H), 3.00 (dd, *J* = 10.3, 4.8 Hz, 1H), 2.68 (d, *J* = 2.8 Hz, 1H), 2.38 – 2.25 (m, 2H). ^{13}C NMR (125 MHz, $CDCl_3$) δ 156.82 (*a*d, *J* = 37 Hz, *CF*$_3$*C*O), 138.27, 137.74, 137.73, 137.72, 137.54, 137.37, 137.33, 136.91 (*aromatic* C), 134.51 (C-9), 128.57, 128.53, 128.47, 128.43, 128.37, 128.35, 128.32, 128.17, 128.12, 128.07, 128.02, 127.97, 127.82, 127.78, 127.76, 127.70, 127.65, 127.43, 127.27, 127.10, 126.88 (*aromatic* C*H*), 116.95 (C-10), 115.89 (*a*d, *J* = 286 Hz, C*F*$_3$), 98.78, 98.59, 98.04, 97.49, 79.66, 76.27, 75.54, 75.04, 74.40, 73.59, 73.28, 73.26, 72.95, 72.93, 72.75, 71.79, 71.59, 71.10, 71.05, 70.68, 69.81, 69.67, 69.38, 69.08, 68.87, 67.76, 66.53, 66.44, 66.27, 63.32, 59.96, 59.49, 49.32. HR-MS: Calculated for $C_{86}H_{93}N_{10}O_{18}F_3$ $[M+Na]^+$: 1633.65136, found: 1633.65226.

Pentasaccharide 29

The reaction was carried out according to the general procedure B using donor **14** (550 mg, 0.81 mmol) and acceptor **S27** (437 mg, 0.27 mmol). The product was purified by column chromatography (pentane:EtOAc = 7:1). Compound **29** (488 mg, α-only, 85% yield) was obtained as yellow foam. $[\alpha]_D^{25}$ +157.8 (c=0.5, $CHCl_3$). ^{1}H NMR (500 MHz, $CDCl_3$) δ 7.45 – 7.16 (m, 47H), 7.15 – 7.09 (m, 2H), 6.89 (d, *J* = 9.5 Hz, 1H), 6.31 (d, *J* = 9.6 Hz, 1H), 5.75 (ddt, *J* = 17.1, 10.2, 6.8 Hz, 1H), 5.48 (d, *J* = 3.5 Hz, 1H), 5.11 – 4.99 (m, 4H), 4.96 (d, *J* = 3.6 Hz, 1H), 4.86 – 4.63 (m, 8H), 4.61 – 4.37 (m, 10H), 4.37 – 4.17 (m, 7H), 4.16 – 4.01 (m, 5H), 4.00 – 3.58 (m, 13H), 3.54 – 3.41 (m, 3H), 3.40 – 3.14 (m, 6H), 3.07 – 3.01 (m, 1H), 2.97 (d, *J* = 10.7 Hz, 1H), 2.31 (qt, *J* = 6.7, 1.4 Hz, 2H), 1.03 (d, *J* = 4.7 Hz, 18H). ^{13}C NMR (125 MHz, $CDCl_3$) δ 157.08 (*a*d, *J* = 37 Hz, 2x*CF*$_3$*C*O), 138.07, 137.63, 137.55, 137.45, 137.38, 136.92, 136.42 (*aromatic* C), 134.51 (C-9), 128.61, 128.59, 128.49, 128.44, 128.42, 128.38, 128.33, 128.22, 128.19, 128.13, 128.08, 127.93, 127.89, 127.86, 127.84, 127.83, 127.74, 127.72, 127.33, 127.10, 126.83 (*aromatic* C*H*), 116.98 (C-10), 115.96 (*a*d, *J* = 286 Hz, 2xC*F*$_3$), 98.75, 98.07, 98.01, 97.48, 97.04, 76.12, 75.57, 75.26, 74.39, 74.20, 73.62, 73.49, 73.27, 73.00, 72.40, 71.90, 71.88, 71.64, 71.20, 71.06, 70.75, 70.61, 69.79, 69.68, 69.57, 68.98, 68.81, 68.44, 67.80, 67.05, 66.55, 66.52, 66.23, 63.95, 60.35, 59.52, 49.30, 48.57, 33.84, 27.62, 27.32. HR-MS: Calculated for $C_{109}H_{125}N_{11}O_{23}F_6Si$ $[M+NH_4]^+$: 2115.89615, found: 2115.89868.

Pentasaccharide S28

The reaction was carried out according to the general procedure C using compound **29** (478 mg, 0.23 mmol) and HF/pyridine (70%, 95 µl, 3.64 mmol). The product was purified by column chromatography (pentane:EtOAc = 3:2). Compound **S28** (423 mg, 95% yield) was obtained as white foam. $[\alpha]_D^{25}$ +135.4 (c=0.5, $CHCl_3$). ^{1}H NMR (500 MHz, $CDCl_3$) δ 7.43 – 7.14 (m, 46H), 7.08 (d, *J* = 9.8 Hz, 1H), 6.40 (d, *J* = 9.6 Hz, 1H), 5.74 (ddt, *J* = 17.0, 10.2, 6.7 Hz, 1H), 5.26 (d, *J* = 3.6 Hz, 1H), 5.11 – 4.98 (m, 4H), 4.95 (d, *J* = 3.6 Hz, 1H), 4.85 – 4.74 (m, 4H), 4.69 (d, *J* = 12.6

Hz, 1H), 4.63 (d, *J* = 11.8 Hz, 1H), 4.59 – 4.43 (m, 6H), 4.39 (d, *J* = 12.5 Hz, 3H), 4.35 – 4.22 (m, 7H), 4.15 – 4.01 (m, 6H), 3.97 (d, *J* = 9.0 Hz, 1H), 3.92 – 3.82 (m, 3H), 3.78 (dq, *J* = 9.4, 2.7 Hz, 2H), 3.73 – 3.58 (m, 7H), 3.48 (dt, *J* = 9.7, 6.5 Hz, 1H), 3.42 (dd, *J* = 10.6, 2.8 Hz, 1H), 3.39 – 3.26 (m, 3H), 3.25 – 3.14 (m, 3H), 3.11 (d, *J* = 10.4 Hz, 1H), 3.00 (d, *J* = 11.0 Hz, 1H), 2.35 – 2.24 (m, 2H). ^{13}C NMR (125 MHz, $CDCl_3$) δ 157.08 (*a*d, *J* = 37 Hz, 2x*CF*$_3$*CO*), 137.60, 137.54, 137.46, 137.43, 137.36, 137.14, 136.88, 136.55 (*aromatic* C), 134.49 (C-9), 128.55, 128.54, 128.37, 128.33, 128.17, 128.14, 128.09, 128.03, 127.99, 127.92, 127.86, 127.82, 127.79, 127.59, 127.34, 127.25, 127.05, 126.92 (*aromatic* C*H*), 116.91 (C-10), 115.89 (*a*d, *J* = 286 Hz, 2xC*F*$_3$), 98.73, 98.03, 97.96, 97.49, 97.46, 79.52, 75.91, 75.56, 74.39, 74.05, 73.87, 73.58, 73.38, 73.21, 72.94, 72.05, 71.90, 71.61, 71.12, 71.08, 70.87, 70.74, 70.71, 70.57, 69.72, 68.93, 68.82, 67.76, 67.32, 66.55, 66.52, 66.26, 66.10, 63.81, 62.38, 60.18, 59.48, 49.28, 49.20, 33.79. HR-MS: Calculated for $C_{101}H_{109}N_{11}O_{23}F_6$ $[M+NH_4]^+$: 1975.79402, found: 1975.79547.

Pentasaccharide S29

The reaction was carried out according to the general procedure D using compound **S28** (416 mg, 0.21 mmol), K_2CO_3 (32 mg, 0.23 mmol), KI (35 mg, 0.21 mmol) and $Ph_2BO(CH_2)_2NH_2$ (4.8 mg, 0.021 mmol). The product was purified by column chromatography (pentane:EtOAc = 4:1). Compound **S29** (391 mg, 90% yield) was obtained as yellow syrup. $[\alpha]_D^{25}$ +169 (c=0.5, $CHCl_3$). ^{1}H NMR (500 MHz, $CDCl_3$) δ 7.47 – 7.05 (m, 54H), 6.42 (d, *J* = 9.5 Hz, 1H), 5.74 (ddt, *J* = 17.0, 10.2, 6.7 Hz, 1H), 5.28 (d, *J* = 3.6 Hz, 1H), 5.10 – 4.99 (m, 4H), 4.95 (d, *J* = 3.6 Hz, 1H), 4.85 – 4.72 (m, 4H), 4.67 (d, *J* = 12.6 Hz, 1H), 4.63 – 4.19 (m, 20H), 4.16 – 3.96 (m, 7H), 3.94 – 3.83 (m, 4H), 3.82 – 3.74 (m, 2H), 3.73 – 3.57 (m, 5H), 3.56 – 3.41 (m, 3H), 3.33 (ddt, *J* = 26.5, 10.2, 4.8 Hz, 3H), 3.24 – 3.11 (m, 4H), 3.04 (d, *J* = 10.8 Hz, 1H), 2.76 (s, 1H), 2.29 (q, *J* = 6.8 Hz, 2H). ^{13}C NMR (125 MHz, $CDCl_3$) δ 156.93 (*a*d, *J* = 37 Hz, 2x*CF*$_3$*CO*), 137.88, 137.74, 137.49, 137.37, 137.31, 137.29, 137.23, 136.83, 136.53 (*aromatic* C), 134.43 (C-9), 128.46, 128.43, 128.41, 128.30, 128.29, 128.24, 128.19, 128.13, 128.06, 128.02, 127.90, 127.86, 127.78, 127.71, 127.68, 127.65, 127.59, 127.53, 127.49, 127.24, 127.22, 126.96 (*aromatic* C*H*), 116.82 (C-10), 115.77 (*a*d, *J* = 286 Hz, C*F*$_3$), 98.62, 97.97, 97.94, 97.46, 97.38, 79.38, 75.98, 75.71, 75.51, 74.25, 73.88, 73.53, 73.49, 73.14, 73.04, 72.84, 72.01, 71.74, 71.52, 70.99, 70.69, 70.65, 70.60, 69.67, 69.49, 68.85, 68.76, 68.69, 67.67, 67.55, 66.46, 66.22, 65.10, 63.71, 60.08, 59.41, 49.25, 49.21, 33.71. HR-MS: Calculated for $C_{108}H_{115}N_{11}O_{23}F_6$ $[M+NH_4]^+$: 2065.84097, found: 2065.84196.

Hexasaccharide 30

The reaction was carried out according to the general procedure B using donor **13** (344 mg, 0.57 mmol) and acceptor **S29** (388 mg, 0.19 mmol). The product was purified by column chromatography (pentane:EtOAc = 6:1). Compound **30** (401 mg, α-only, 85% yield) was obtained as white foam. $[\alpha]_D^{25}$ +150 (c=0.5, $CHCl_3$). ^{1}H NMR (500 MHz, $CDCl_3$) δ 7.49 – 7.41 (m, 2H), 7.40 – 7.12 (m, 55H), 7.01 (d, *J* = 9.8 Hz, 1H), 6.28 (d, *J* = 9.6 Hz, 1H), 5.80 – 5.68 (m, 1H), 5.38 (d, *J* = 3.5 Hz, 1H), 5.11 – 4.99 (m, 4H), 4.96 (dd, *J* = 5.9, 3.6 Hz, 2H), 4.84 – 4.21 (m, 29H), 4.15 – 3.95 (m, 9H), 3.94 – 3.44 (m, 16H), 3.40 – 3.09 (m, 8H), 3.03 (d, *J* = 11.0 Hz, 1H), 2.30 (q, *J* = 6.7 Hz, 2H), 1.03 (s, 9H), 0.96 (s, 9H). ^{13}C NMR (125 MHz, $CDCl_3$) δ 157.05 (*a*d, *J* = 37 Hz, 2x*CF*$_3$*CO*), 137.90, 137.84, 137.50, 137.41, 137.37, 137.31, 137.29, 137.23, 137.14, 136.81, 136.39 (*aromatic* C), 134.44 (C-9), 128.53, 128.51, 128.47, 128.44, 128.37, 128.34, 128.31, 128.29, 128.18, 128.15, 128.09, 128.05, 127.98, 127.89, 127.86, 127.81, 127.78, 127.76, 127.67, 127.62, 127.57, 127.51, 127.26, 126.99, 126.84, 126.04 (*aromatic* C*H*), 116.92 (C-10), 115.81 (*a*d, *J* = 286 Hz, 2xC*F*$_3$), 98.74, 98.68, 98.01, 97.40, 97.37, 79.54, 76.28, 75.88, 75.53, 74.31, 73.98, 73.56, 73.30, 73.20, 73.17, 72.91, 71.96, 71.82, 71.55, 71.01, 70.95, 70.91, 70.64, 70.53, 70.50, 70.10, 69.70, 69.55, 68.90, 68.73, 67.72, 67.51, 67.46, 66.96, 66.48, 66.41, 66.21, 63.78, 60.12, 59.44, 58.42, 49.60, 49.20, 33.77, 27.52, 27.37, 23.17. MALDI-MS: Calculated for $C_{129}H_{146}N_{14}O_{27}F_6Si$ [M+Na]+: 2488.0048, found: 2487.9692.

Hexasaccharide S30

The reaction was carried out according to the general procedure C using compound **30** (394 mg, 0.16 mmol) and HF/pyridine (70%, 66 μl, 2.56 mmol). The product was purified by column chromatography (pentane:EtOAc = 3:2). Compound **S30** (340 mg, 91% yield) was obtained as white foam. $[\alpha]_D^{25}$ +149.2 (c=0.5, $CHCl_3$). ^{1}H NMR (500 MHz, $CDCl_3$) δ 7.42 – 7.12 (m, 63H), 7.08 (d, *J* = 9.8 Hz, 1H), 6.42 (d, *J* = 9.6 Hz, 1H), 5.74 (ddt, *J* = 17.0, 10.3, 6.7 Hz, 1H), 5.34 (d, *J* = 3.5 Hz, 1H), 5.11 – 4.91 (m, 7H), 4.85 – 4.74 (m, 4H), 4.72 – 4.52 (m, 9H), 4.52 – 3.57 (m, 41H), 3.52 – 3.43 (m, 2H), 3.43 – 3.07 (m, 11H), 3.01 (d, *J* = 10.9 Hz, 1H), 2.90 (s, 1H), 2.30 (q, *J* = 6.7 Hz, 2H). ^{13}C NMR (125 MHz, $CDCl_3$) δ 157.11 (*a*d, *J* = 37 Hz, 2x*CF*$_3$*CO*), 137.88, 137.54, 137.44, 137.42, 137.39, 137.33, 137.15, 136.86, 136.49 (*aromatic* C), 134.46 (C-9), 128.52, 128.47, 128.44, 128.33, 128.29, 128.19, 128.14, 128.11, 128.06, 128.04, 127.92, 127.83, 127.77, 127.73, 127.72, 127.60, 127.58, 127.20, 127.02, 126.88, 126.40 (*aromatic* C*H*), 116.88 (C-10), 115.84 (*a*d, *J* = 286 Hz, 2xC*F*$_3$), 99.15, 98.69, 98.02, 97.41, 79.47, 76.15, 75.84, 75.51, 74.35, 73.82, 73.54, 73.47, 73.18, 73.16, 72.89, 72.10, 72.04, 71.85, 71.66, 71.58, 71.09, 71.05, 71.01, 70.65, 70.27, 69.68, 69.17, 68.89, 68.77, 67.72,

67.49, 67.43, 66.65, 66.53, 66.48, 63.76, 62.43, 60.13, 59.44, 59.40, 49.76, 49.24, 33.76. MALDI-MS: Calculated for $C_{121}H_{130}N_{14}O_{27}F_6$ [M+Na]+: 2347.9026, found: 2347.8702.

Hexasaccharide S31

The reaction was carried out according to the general procedure D using compound **S30** (333 mg, 0.14 mmol), K_2CO_3 (22 mg, 0.16 mmol), KI (24 mg, 0.14 mmol) and $Ph_2BO(CH_2)_2NH_2$ (6.4 mg, 0.029 mmol). The product was purified by column chromatography (pentane:EtOAc = 7:2). Compound **S31** (325 mg, 94% yield) was obtained as white foam. $[\alpha]_D^{25}$ +146.2 (c=0.5, $CHCl_3$). ^{1}H NMR (500 MHz, $CDCl_3$) δ 7.45 – 7.09 (m, 62H), 6.99 (d, *J* = 9.7 Hz, 1H), 6.32 (d, *J* = 9.5 Hz, 1H), 5.80 – 5.67 (m, 1H), 5.37 (d, *J* = 3.5 Hz, 1H), 5.09 – 5.00 (m, 4H), 4.99 (d, *J* = 3.7 Hz, 1H), 4.95 (d, *J* = 3.6 Hz, 1H), 4.83 – 4.73 (m, 5H), 4.72 – 4.52 (m, 7H), 4.51 – 4.43 (m, 2H), 4.43 – 4.17 (m, 15H), 4.16 – 3.57 (m, 21H), 3.50 – 3.42 (m, 2H), 3.42 – 3.07 (m, 11H), 3.01 (d, *J* = 11.0 Hz, 1H), 2.29 (q, *J* = 6.7 Hz, 2H). ^{13}C NMR (125 MHz, $CDCl_3$) δ 156.99 (*a*d, *J* = 37 Hz, 2x*CF*$_3$*CO*), 137.82, 137.61, 137.51, 137.46, 137.40, 137.37, 137.35, 137.28, 137.25, 136.77, 136.42 (*aromatic* C), 134.41 (C-9), 128.48, 128.44, 128.40, 128.34, 128.29, 128.28, 128.26, 128.23, 128.19, 128.17, 128.14, 128.10, 128.04, 128.00, 127.98, 127.91, 127.81, 127.75, 127.73, 127.70, 127.56, 127.50, 127.45, 127.19, 126.94, 126.77, 126.37 (*aromatic* C*H*), 116.86 (C-10), 115.83 (*a*d, *J* = 286 Hz, C*F*$_3$), 99.39, 98.63, 97.97, 97.34, 97.27, 79.45, 76.26, 76.16, 75.83, 75.49, 74.27, 73.82, 73.50, 73.42, 73.21, 73.14, 73.11, 72.85, 72.02, 71.94, 71.75, 71.50, 71.19, 71.07, 70.96, 70.89, 70.59, 70.17, 69.78, 69.65, 68.84, 68.69, 68.24, 67.67, 67.42, 66.86, 66.56, 66.44, 66.37, 66.17, 63.71, 60.06, 59.39, 59.25, 58.09, 49.60, 49.17, 33.72. MALDI-MS: Calculated for $C_{128}H_{136}N_{14}O_{27}F_6$ [M+Na]+: 2437.9496, found: 2437.9144.

Heptasaccharide 31

The reaction was carried out according to the general procedure B using donor **14** (381 mg, 0.56 mmol) and acceptor **S31** (330 mg, 0.14 mmol). The product was purified by column chromatography (pentane:EtOAc = 6:1). Compound **31** (341 mg, α-only, 86% yield) was obtained as yellow foam. $[\alpha]_D^{25}$ +149.8 (c=0.5, $CHCl_3$). ^{1}H NMR (500 MHz, $CDCl_3$) δ 7.45 – 7.09 (m, 67H), 7.08 – 7.02 (m, 2H), 7.00 (d, *J* = 9.7 Hz, 1H), 6.37 (d, *J* = 9.5 Hz, 1H), 6.27 (d, *J* = 9.4 Hz, 1H), 5.74 (ddt, *J* = 17.0, 10.3, 6.7 Hz, 1H), 5.36 (d, *J* = 3.5 Hz, 1H), 5.10 – 4.98 (m, 5H), 4.97 – 4.93 (m, 1H), 4.86 – 4.66 (m, 9H), 4.66 – 4.52 (m, 6H), 4.52 – 4.19 (m, 20H), 4.14 – 3.83 (m, 15H), 3.81 – 3.55 (m, 9H), 3.53 – 3.26 (m, 8H), 3.26 – 3.08 (m, 4H), 3.06 – 2.93 (m, 2H), 2.92 – 2.85 (m, 1H), 2.30 (q, *J* = 6.8 Hz, 2H), 1.07 (s, 9H), 0.98 (s, 9H). ^{13}C NMR (125 MHz, $CDCl_3$) δ 156.80 (*a*d, *J* = 37 Hz, *CF*$_3$C*O*), 137.89, 137.51, 137.50, 137.42, 137.39, 137.33, 137.31, 137.15, 136.84, 136.80, 136.39 (*aromatic* C), 134.45 (C-9), 128.60, 128.53, 128.50, 128.47, 128.42, 128.37, 128.34, 128.32, 128.28, 128.23, 128.18, 128.14, 128.08, 128.06, 128.01, 127.98, 127.86, 127.83, 127.81, 127.79, 127.76, 127.70, 127.67, 127.63, 127.61, 127.57, 127.53, 127.24, 127.00, 126.84, 126.72, 126.21 (*aromatic* C*H*), 116.91 (C-10), 115.83 (*a*d, *J* = 286 Hz, C*F*$_3$), 98.89, 98.68, 98.03, 97.97, 97.43, 97.28, 96.59, 79.56, 76.55, 75.91, 75.84, 75.55, 74.74, 74.35, 74.02, 73.55, 73.34, 73.19, 73.17, 72.98, 72.91, 71.95, 71.83, 71.58, 71.46, 71.38, 71.05, 70.99, 70.68, 70.61, 69.95, 69.69, 69.42, 69.34, 69.23, 68.89, 68.75, 68.65, 67.74, 67.67, 67.53, 66.89, 66.58, 66.42, 66.20, 65.64, 63.86, 60.16, 60.12, 59.44, 53.44, 49.54, 49.23, 48.13, 33.77, 27.52, 27.35, 23.21, 20.64. MALDI-MS: Calculated for $C_{151}H_{168}N_{15}O_{32}F_9Si$ [M+Na]+: 2925.1498, found: 2925.1031.

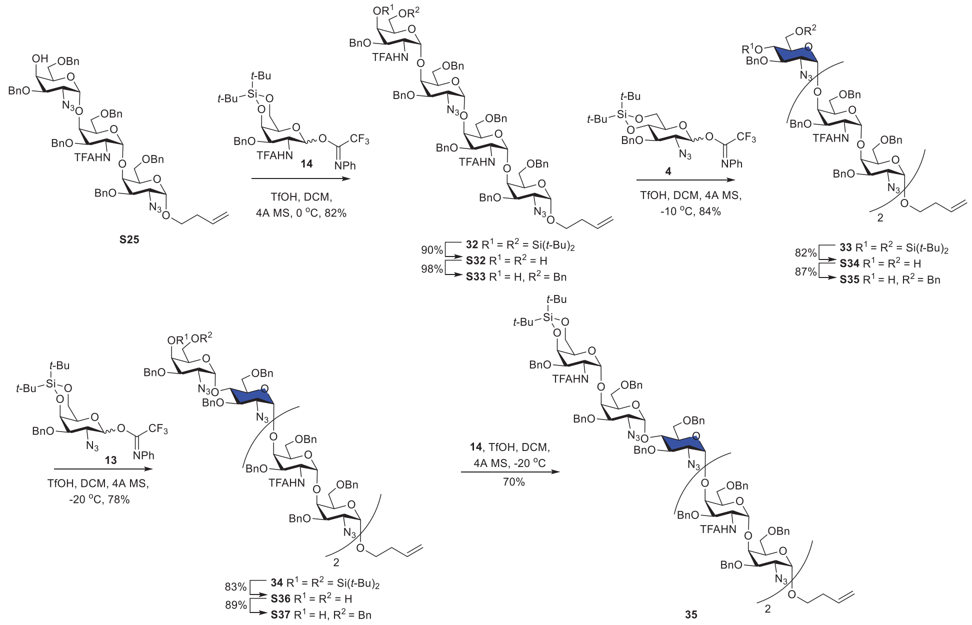

3-Butenyl 3-*O*-benzyl-2-deoxy-4,6-di-*tert*-butylsilylidene-2-trifluoroacetamido-α-D-galactopyranosyl-(1→4)-2-azido-3,6-di-*O*-benzyl-2-deoxy-α-D-galactopyranosyl-(1→4)-3,6-di-*O*-benzyl-2-deoxy-2-trifluoro-acetamido-α-D-galactopyranosyl-(1→4)-2-azido-3,6-di-*O*-benzyl-2-deoxy-α-D-galactopyranoside (32)

The reaction was carried out according to the general procedure B using donor **14** (2.44 g, 3.61 mmol) and acceptor **S25** (1.5 g, 1.2 mmol). The product was purified by column chromatography (pentane:EtOAc = 8:1). Compound **32** (1.70 g, α-only, 82% yield) was obtained as yellow foam. ^{1}H NMR (500 MHz, $CDCl_3$) δ 7.44 – 7.10 (m, 37H), 6.28 (dd, *J* = 9.7, 3.6 Hz, 2H), 5.75 (ddt, *J* = 17.1, 10.2, 6.8 Hz, 1H), 5.09 – 5.00 (m, 3H), 4.95 (d, *J* = 3.6 Hz, 1H), 4.87 – 4.83 (m, 1H), 4.82 (s, 1H), 4.79 (d, *J* = 3.6 Hz, 1H), 4.75 (d, *J* = 3.5 Hz, 1H), 4.73 (d, *J* = 3.3 Hz, 1H), 4.67 – 4.62 (m, 2H), 4.62 – 4.56 (m, 1H), 4.55 – 4.44 (m, 4H), 4.44 – 4.34 (m, 4H), 4.31 (dd, *J* = 9.9, 5.3 Hz, 1H), 4.27 (d, *J* = 2.9 Hz, 2H), 4.25 – 4.22 (m, 1H), 4.11 (d, *J* = 11.6 Hz, 1H), 4.06 (d, *J* = 11.6 Hz, 1H), 4.02 (d, *J* = 11.5 Hz, 1H), 3.98 – 3.89 (m, 3H), 3.85 (dd, *J* = 9.0, 5.8 Hz, 1H), 3.83 – 3.76 (m, 2H), 3.70 – 3.57 (m, 4H), 3.53 – 3.43 (m, 2H), 3.40 – 3.28 (m, 4H), 3.21 (dd, *J* = 8.6, 5.2 Hz, 1H), 3.12 – 3.01 (m, 2H), 2.32 (qt, *J* = 6.7, 1.3 Hz, 2H), 1.07 (s, 9H), 0.98 (s, 9H). ^{13}C NMR (125 MHz, $CDCl_3$) δ 156.81 (*a*d, *J* = 37 Hz, 2x*CF₃C*O), 137.98, 137.61, 137.45, 137.40, 137.29, 136.97, 136.94 (*aromatic* C), 134.57 (C-9), 128.69, 128.66, 128.65, 128.57, 128.52, 128.50, 128.45, 128.37, 128.25, 128.19, 128.17, 128.10, 127.97, 127.90, 127.83, 127.81, 127.75, 127.37, 127.11, 126.80 (*aromatic* C*H*), 117.02 (C-10), 115.87 (*a*d, *J* = 286 Hz, 2xC*F₃*), 98.47, 98.12, 97.54, 96.85, 76.25, 75.76, 74.77, 74.53, 73.67, 73.18, 73.12, 71.79, 71.77, 71.21, 71.14, 70.97, 69.68, 69.65, 69.56, 69.36, 68.88, 68.73, 67.87, 67.83, 67.00, 66.58, 65.86, 60.29, 59.60, 49.31, 48.30, 33.89, 27.64, 27.45, 23.34, 20.75. HR-MS: Calculated for $C_{89}H_{104}N_8O_{19}F_6Si$ [M+Na]$^+$: 1753.69834, found: 1753.69945.

3-Butenyl 3-*O*-benzyl-2-deoxy-2-trifluoroacetamido-α-D-galactopyranosyl-(1→4)-2-azido-3,6-di-*O*-benzyl-2-deoxy-α-D-galacto-pyranosyl-(1→4)-3,6-di-*O*-benzyl-2-deoxy-2-trifluoroacetamido-α-D-galactopyranosyl-(1→4)-2-azido-3,6-di-*O*-benzyl-2-deoxy-α-D-galact-opyranoside (S32)

The reaction was carried out according to the general procedure C using compound **32** (2.33 g, 1.35 mmol) and HF/pyridine (70%, 560 µl, 21.5 mmol). The product was purified by column chromatography (pentane:EtOAc = 2:1). Compound **S32** (1.9 g, 90% yield) was obtained as white foam. $[\alpha]_D^{25}$ +179.4 (c=1, $CHCl_3$). ^{1}H NMR (500 MHz, $CDCl_3$) δ 7.50 – 7.10 (m, 37H), 6.52 (d, *J* = 9.5 Hz, 1H), 6.33 (d, *J* = 9.7 Hz, 1H), 5.75 (ddt, *J* = 17.0, 10.2, 6.7 Hz, 1H), 5.09 – 5.00 (m, 2H), 4.99 (d, *J* = 3.7 Hz, 1H), 4.93 (d, *J* = 3.6 Hz, 1H), 4.87 (d, *J* = 3.7 Hz, 1H), 4.84 – 4.75 (m, 3H), 4.65 (dd, *J* = 11.8, 3.1 Hz, 2H), 4.51 (dp, *J* = 13.4, 3.6, 3.1 Hz, 3H), 4.47 – 4.35 (m, 5H), 4.33 (d, *J* = 2.6 Hz, 1H), 4.30 (dd, *J* = 9.9, 5.4 Hz, 1H), 4.26 (d, *J* = 2.6 Hz, 1H), 4.23 – 4.17 (m, 2H), 4.14 – 3.98 (m, 6H), 3.96 (dd, *J* = 10.9, 2.6 Hz, 1H), 3.85 (dd, *J* = 9.0, 5.8 Hz, 1H), 3.82 – 3.75 (m, 2H), 3.67 – 3.53 (m, 3H), 3.53 – 3.43 (m, 2H), 3.40 – 3.32 (m, 3H), 3.32 – 3.23 (m, 2H), 3.20 (dd, *J* = 8.6, 5.2 Hz, 1H), 3.09 (d, *J* = 7.5 Hz, 2H), 2.90 (s, 1H), 2.32 (qt, *J* = 6.7, 1.4 Hz, 2H). ^{13}C NMR (125 MHz, $CDCl_3$) δ 156.88 (*a*d, *J* = 37 Hz, 2x*CF₃C*O), 137.63, 137.42, 137.29, 137.15, 137.03, 136.95 (*aromatic* C), 134.56 (C-9), 128.66, 128.64, 128.46, 128.42, 128.24, 128.22, 128.16, 128.11, 128.02, 127.99, 127.96,

127.92, 127.87, 127.63, 127.18, 127.08 (*aromatic* C*H*), 117.00 (C-10), 115.87 (*a*d, *J* = 286 Hz, 2xC*F*$_3$), 98.49, 98.07, 97.51, 97.12, 76.14, 75.74, 75.20, 74.06, 73.65, 73.12, 73.05, 72.02, 71.74, 71.26, 71.14, 70.89, 70.84, 70.76, 69.63, 69.42, 68.86, 68.70, 67.84, 66.60, 66.55, 66.40, 65.86, 62.44, 60.21, 59.57, 49.26, 48.89, 33.86. HR-MS: Calculated for $C_{81}H_{88}N_8O_{19}F_6$ [M+Na]$^+$: 1613.59621, found: 1613.59665.

3-Butenyl 3,6-di-*O*-benzyl-2-deoxy-2-trifluoroacetamido-*α*-D-galactopyranosyl-(1→4)-2-azido-3,6-di-*O*-benzyl-2-deoxy-*α*-D-galacto-pyranosyl-(1→4)-3,6-di-*O*-benzyl-2-deoxy-2-trifluoroacetamido-*α*-D-galactopyranosyl-(1→4)-2-azido-3,6-di-*O*-benzyl-2-deoxy-*α*-D-galact-opyranoside (S33)

The reaction was carried out according to the general procedure D using compound **S32** (1.84 g, 1.16 mmol), K_2CO_3 (240 mg, 1.73 mmol), KI (249 mg, 1.5 mmol) and $Ph_2BO(CH_2)_2NH_2$ (53 mg, 0.23 mmol). The product was purified by column chromatography (pentane:EtOAc = 4:1). Compound **S33** (1.90 g, 98% yield) was obtained as yellow syrup. $[\alpha]_D^{25}$ +77.4 (c=3, $CHCl_3$). ^{1}H NMR (500 MHz, $CDCl_3$) δ 7.43 – 7.11 (m, 46H), 6.42 (d, *J* = 9.5 Hz, 1H), 6.24 (d, *J* = 9.7 Hz, 1H), 5.75 (ddt, *J* = 17.0, 10.2, 6.7 Hz, 1H), 5.09 – 5.00 (m, 2H), 4.99 (d, *J* = 3.8 Hz, 1H), 4.91 (d, *J* = 3.6 Hz, 1H), 4.87 – 4.78 (m, 3H), 4.76 (d, *J* = 3.6 Hz, 1H), 4.71 (d, *J* = 12.1 Hz, 1H), 4.58 (d, *J* = 12.0 Hz, 1H), 4.56 – 4.32 (m, 10H), 4.31 – 4.16 (m, 9H), 4.10 (d, *J* = 11.6 Hz, 1H), 4.07 – 4.01 (m, 3H), 3.94 (dd, *J* = 10.9, 2.6 Hz, 1H), 3.84 (dd, *J* = 9.0, 5.8 Hz, 1H), 3.80 – 3.73 (m, 2H), 3.66 – 3.53 (m, 4H), 3.48 (dq, *J* = 9.5, 6.4 Hz, 2H), 3.39 – 3.31 (m, 2H), 3.31 – 3.17 (m, 4H), 3.15 – 3.05 (m, 2H), 2.91 (s, 1H), 2.32 (qt, *J* = 6.7, 1.4 Hz, 2H). ^{13}C NMR (125 MHz, $CDCl_3$) δ 156.85 (*a*d, *J* = 37 Hz, 2xC*F*$_3$C*O*), 137.76, 137.62, 137.55, 137.44, 137.31, 137.12, 136.96 (*aromatic* C), 134.59 (C-9), 128.68, 128.66, 128.60, 128.54, 128.51, 128.49, 128.44, 128.43, 128.28, 128.22, 128.07, 128.04, 127.96, 127.90, 127.87, 127.83, 127.79, 127.73, 127.71, 127.28, 127.11 (*aromatic* C*H*), 117.03 (C-10), 115.92 (*a*d, *J* = 286 Hz, C*F*$_3$), 98.57, 98.09, 97.50, 97.30, 76.12, 75.77, 75.44, 74.03, 73.68, 73.57, 73.11, 72.03, 71.74, 71.13, 71.07, 70.88, 70.86, 70.59, 69.68, 69.23, 68.87, 68.81, 68.70, 67.87, 66.61, 66.57, 65.85, 65.78, 60.09, 59.60, 49.26, 48.99, 33.89. HR-MS: Calculated for $C_{88}H_{94}N_8O_{19}F_6$ [M+Na]$^+$: 1703.64316, found: 1703.64321.

Pentasaccharide 33

The reaction was carried out according to the general procedure A. The donor **4** (713 mg, 0.36 mmol) and acceptor **S33** (495 mg, 0.089 mmol) were co-evaporated with toluene (three times). The residue was dissolved in dry 5 ml DCM under nitrogen and stirred over fresh flame-dried molecular sieves 4Å. The solution was cooled to -10 ºC, after which TfOH (10 µl, 0.036 mmol) was added. The reaction was stirred at -10 ºC for overnight. Then the reaction was quenched with Et_3N, diluted with DCM, washed with saturated $NaHCO_3$ and brine. The organic phase was dried with anhydrous $MgSO_4$, filtered and concentrated *in vacuo*. The product was purified by silica gel column chromatography (pentane:EtOAc = 8:1). Compound **33** (676 mg, α-only, 84% yield) was obtained as white foam. $[\alpha]_D^{25}$ +119.6 (c=1, $CHCl_3$). ^{1}H NMR (500 MHz, $CDCl_3$) δ 7.50 – 7.11 (m, 48H), 7.07 (h, *J* = 4.2 Hz, 1H), 6.13 (d, *J* = 9.6 Hz, 1H), 6.08 (d, *J* = 9.4 Hz, 1H), 5.74 (ddt, *J* = 17.0, 10.2, 6.8 Hz, 1H), 5.12 – 4.99 (m, 4H), 4.97 – 4.89 (m, 2H), 4.87 – 4.77 (m, 4H), 4.76 (d, *J* = 3.7 Hz, 1H), 4.74 (d, *J* = 3.7 Hz, 1H), 4.62 – 4.31 (m, 11H), 4.30 – 4.09 (m, 8H), 4.06 – 3.70 (m, 13H), 3.65 – 3.57 (m, 2H), 3.53 (dd, *J* = 11.1, 2.4 Hz, 1H), 3.51 – 3.44 (m, 1H), 3.34 (dd, *J* = 9.3, 5.8 Hz, 1H), 3.29 – 3.17 (m, 4H), 3.16 – 3.01 (m, 4H), 2.31 (qt, *J* = 6.7, 1.3 Hz, 2H), 1.04 (s, 9H), 1.01 (s, 9H). ^{13}C NMR (125 MHz, $CDCl_3$) δ 156.61 (*a*d, *J* = 37 Hz, 2xC*F*$_3$C*O*), 138.33, 137.71, 137.69, 137.41, 137.38, 137.25, 136.94 (*aromatic* C), 134.56 (C-9), 128.67, 128.64, 128.59, 128.54, 128.50, 128.46, 128.44, 128.41, 128.34, 128.28, 128.26, 128.24, 128.23, 128.18, 128.12, 128.10, 128.05, 127.94, 127.91, 127.89, 127.84, 127.81, 127.64, 127.12, 126.81 (*aromatic* C*H*), 117.01 (C-10), 115.93 (*a*d, *J* = 286 Hz, 2xC*F*$_3$), 98.57, 98.11, 98.06, 97.40, 79.41, 79.29, 76.46, 75.73, 75.73, 75.71, 73.65, 73.55, 73.21, 73.17, 73.05, 73.01, 71.93, 71.69, 71.30, 70.95, 70.79, 70.66, 69.62, 69.53, 68.88, 68.84, 67.82, 67.09, 66.78, 66.73, 66.54, 66.26, 65.75, 62.90, 60.02, 59.58, 49.31, 49.22, 33.87, 27.43, 27.05, 22.69, 20.02. HR-MS: Calculated for $C_{109}H_{125}N_{11}O_{23}F_6Si$ [M+NH$_4$]$^+$: 2115.89615, found: 2115.89649.

Pentasaccharide S34

The reaction was carried out according to the general procedure C using compound **33** (740 mg, 0.35 mmol) and HF/pyridine (70%, 146 µl, 5.64 mmol). The product was purified by column chromatography (pentane:EtOAc = 2:1). Compound **S34** (568 mg, 82% yield) was obtained as white foam. $[\alpha]_D^{25}$ +144.6 (c=1, $CHCl_3$). ^{1}H NMR (500 MHz, $CDCl_3$) δ 7.49 – 7.10 (m, 46H), 7.06 (dd, *J* = 8.1, 6.4 Hz, 1H), 6.40 (d, *J* = 9.5 Hz, 1H), 6.36 (d, *J* = 9.5 Hz, 1H), 5.74 (ddt, *J* = 17.1, 10.3, 6.7 Hz, 1H), 5.10 – 4.97 (m, 3H), 4.97 – 4.76 (m, 8H), 4.67 (d, *J* = 12.4 Hz, 1H), 4.61 – 4.46 (m, 4H), 4.45 – 4.32 (m, 5H), 4.32 – 3.89 (m, 16H), 3.88 – 3.73 (m, 4H), 3.72 – 3.44 (m, 6H), 3.40 – 2.97 (m, 11H), 2.41 (s, 1H), 2.30 (q, *J* = 6.7 Hz, 2H). ^{13}C NMR (125 MHz, $CDCl_3$) δ 156.80 (*a*d, *J* = 37 Hz, 2xC*F*$_3$C*O*), 138.30, 137.57, 137.53, 137.47, 137.36, 137.31, 137.24, 136.87, 136.84 (*aromatic* C), 134.49 (C-9), 128.56, 128.52, 128.49, 128.45, 128.42, 128.36, 128.34, 128.31, 128.29, 128.28, 128.17, 128.13, 128.11,

127.94, 127.89, 127.87, 127.83, 127.81, 127.76, 127.73, 127.61, 127.42, 127.32, 127.01, 126.82 (*aromatic* C*H*), 116.93 (C-10), 115.79 (*a*d, *J* = 286 Hz, 2xC*F*$_3$), 98.68, 98.51, 98.03, 97.50, 96.88, 79.97, 76.28, 75.67, 75.12, 74.66, 74.21, 73.57, 73.15, 73.07, 72.98, 72.17, 71.88, 71.73, 71.68, 71.43, 71.17, 71.12, 70.84, 70.50, 70.41, 69.67, 69.54, 68.81, 68.63, 67.78, 66.56, 66.45, 65.44, 63.47, 62.03, 59.95, 59.51, 49.38, 49.22, 33.79. HR-MS: Calculated for $C_{101}H_{109}N_{11}O_{23}F_6$ $[M+NH_4]^+$: 1975.79402, found: 1975.79601.

Pentasaccharide S35

The reaction was carried out according to the general procedure D using compound **S34** (552 mg, 0.28 mmol), K_2CO_3 (58 mg, 0.42 mmol), KI (60 mg, 0.36 mmol) and $Ph_2BO(CH_2)_2NH_2$ (12.7 mg, 0.056 mmol). The product was purified by column chromatography (pentane:EtOAc = 4:1). Compound **S35** (501 mg, 87% yield) was obtained as white foam. $[\alpha]_D^{25}$ +144 (c=1, $CHCl_3$). ^{1}H NMR (500 MHz, $CDCl_3$) δ 7.54 – 6.99 (m, 64H), 6.45 – 6.25 (m, 2H), 5.80 – 5.65 (m, 1H), 5.12 – 4.63 (m, 14H), 4.60 – 3.70 (m, 38H), 3.66 – 2.98 (m, 19H), 2.78 (d, *J* = 4.7 Hz, 1H), 2.51 (d, *J* = 5.8 Hz, 1H), 2.28 (q, *J* = 6.7 Hz, 2H). ^{13}C NMR (125 MHz, $CDCl_3$) δ 156.53 (*a*d, *J* = 37 Hz, 2x*CF*$_3$*CO*), 138.70, 138.24, 137.84, 137.44, 137.42, 137.38, 137.24, 137.13, 136.81, 136.71 (*aromatic* C), 134.37 (C-9), 128.75, 128.43, 128.38, 128.28, 128.25, 128.22, 128.18, 128.15, 128.12, 128.10, 128.05, 128.00, 127.97, 127.94, 127.79, 127.76, 127.68, 127.65, 127.57, 127.48, 127.44, 127.40, 127.01, 126.97, 126.89, 126.81, 126.69 (*aromatic* C*H*), 116.79 (C-10), 115.74 (*a*d, *J* = 286 Hz, C*F*$_3$), 98.67, 98.38, 97.92, 97.26, 96.96, 79.83, 76.11, 75.58, 74.92, 74.86, 73.92, 73.43, 73.11, 72.97, 72.92, 72.83, 72.35, 71.88, 71.58, 71.48, 70.91, 70.80, 70.61, 70.47, 70.37, 69.52, 69.42, 69.04, 68.65, 68.56, 67.61, 66.44, 66.27, 65.41, 63.30, 59.78, 59.37, 58.71, 58.07, 54.59, 49.19, 49.06, 33.66. HR-MS: Calculated for $C_{108}H_{115}N_{11}O_{23}F_6$ $[M+Na]^+$: 2070.79637, found: 2070.79871.

Hexasaccharide 34

The reaction was carried out according to the general procedure B using donor **13** (568 mg, 0.94 mmol) and acceptor **S35** (480 mg, 0.23 mmol). The product was purified by column chromatography (pentane:EtOAc = 6:1). Compound **34** (450 mg, α-only, 78% yield) was obtained as white foam. $[\alpha]_D^{25}$ +140.9 (c=1, $CHCl_3$). ^{1}H NMR (500 MHz, $CDCl_3$) δ 7.50 – 7.06 (m, 59H), 6.41 (d, *J* = 9.5 Hz, 1H), 6.21 (d, *J* = 9.6 Hz, 1H), 5.74 (ddt, *J* = 17.1, 10.3, 6.7 Hz, 1H), 5.51 (d, *J* = 3.6 Hz, 1H), 5.09 – 4.98 (m, 4H), 4.97 – 4.88 (m, 4H), 4.84 (d, *J* = 3.7 Hz, 1H), 4.82 – 4.72 (m, 4H), 4.69 (d, *J* = 11.5 Hz, 1H), 4.63 – 4.53 (m, 2H), 4.53 – 4.35 (m, 8H), 4.29 (td, *J* = 14.0, 13.3, 8.9 Hz, 5H), 4.23 – 4.13 (m, 5H), 4.13 – 3.73 (m, 18H), 3.66 – 3.53 (m, 4H), 3.47 (q, *J* = 6.3 Hz, 2H), 3.42 – 3.05 (m, 10H), 2.30 (q, *J* = 6.7 Hz, 2H), 1.03 (s, 10H), 0.97 (d, *J* = 1.5 Hz, 10H). ^{13}C NMR (125 MHz, $CDCl_3$) δ 156.55 (*a*d, *J* = 37 Hz, 2x*CF*$_3$*CO*), 138.18, 138.16, 137.82, 137.65, 137.58, 137.52, 137.33, 137.20, 137.02, 136.82 (*aromatic* C), 134.46 (C-9), 128.55, 128.52, 128.45, 128.41, 128.39, 128.38, 128.33, 128.30, 128.25, 128.18, 128.13, 128.11, 127.99, 127.96, 127.92, 127.89, 127.86, 127.81, 127.78, 127.68, 127.66, 127.59, 127.51, 127.43, 127.39, 127.04, 126.97, 126.77 (*aromatic* C*H*), 116.93 (C-10), 115.90 (*a*d, *J* = 286 Hz, C*F*$_3$), 98.93, 98.48, 98.01, 97.90, 97.34, 97.25, 80.90, 76.30, 75.83, 75.67, 75.53, 74.67, 74.47, 73.88, 73.57, 73.10, 73.04, 73.00, 72.95, 71.78, 71.61, 70.99, 70.90, 70.87, 70.72, 70.64, 70.61, 70.58, 69.84, 69.71, 69.57, 68.78, 68.73, 68.67, 67.79, 67.74, 66.96, 66.66, 66.51, 66.41, 65.63, 64.78, 59.91, 59.50, 49.36, 49.15, 33.79, 27.58, 27.28, 23.28, 20.67. MALDI-MS: Calculated for $C_{129}H_{146}N_{14}O_{27}F_6Si$ [M+Na]+: 2488.0048, found: 2487.9691.

Hexasaccharide S36

The reaction was carried out according to the general procedure C using compound **34** (440 mg, 0.18 mmol) and HF/pyridine (70%, 74 µl, 2.85 mmol). The product was purified by column chromatography (pentane:EtOAc = 2:1). Compound **S36** (346 mg, 83% yield) was obtained as white foam. $[\alpha]_D^{25}$ +118.6 (c=0.5, $CHCl_3$). ^{1}H NMR (500 MHz, $CDCl_3$) δ 7.61 – 7.01 (m, 59H), 6.44 (d, *J* = 9.5 Hz, 1H), 6.31 (d, *J* = 9.6 Hz, 1H), 5.74 (ddt, *J* = 17.0, 10.3, 6.7 Hz, 1H), 5.32 (d, *J* = 3.6 Hz, 1H), 5.10 – 4.98 (m, 3H), 4.96 – 4.85 (m, 5H), 4.83 (d, *J* = 3.7 Hz, 1H), 4.80 (d, *J* = 12.3 Hz, 1H), 4.77 (d, *J* = 3.6 Hz, 1H), 4.73 (d, *J* = 12.3 Hz, 1H), 4.66 (s, 2H), 4.61 – 4.47 (m, 4H), 4.47 – 4.34 (m, 5H), 4.34 – 4.00 (m, 18H), 4.01 – 3.75 (m, 10H), 3.74 – 3.42 (m, 9H), 3.42 – 3.02 (m, 11H), 2.88 – 2.64 (m, 2H), 2.30 (q, *J* = 6.7 Hz, 2H). ^{13}C NMR (125 MHz, $CDCl_3$) δ 156.73 (*a*d, *J* = 37 Hz, 2x*CF*$_3$*CO*), 138.15, 137.94, 137.61, 137.49, 137.45, 137.31, 137.23, 137.22, 136.90, 136.80 (*aromatic* C), 134.46 (C-9), 128.52, 128.49, 128.38, 128.36, 128.33, 128.32, 128.27, 128.16, 128.14, 128.08, 128.02, 128.01, 127.91, 127.86, 127.78, 127.76, 127.58, 127.55, 127.44, 127.40, 127.01, 126.94, 126.72 (*aromatic* C*H*), 116.89 (C-10), 115.81 (*a*d, *J* = 286 Hz, 2xC*F*$_3$), 98.94, 98.47, 97.98, 97.36, 96.90, 80.07, 76.25, 76.19, 75.64, 75.59, 74.68, 74.15, 73.54, 73.08, 73.00, 72.96, 71.69, 71.61, 71.12, 71.02, 70.96, 70.79, 70.63, 70.40, 70.26, 69.63, 69.51, 68.72, 68.53, 68.40, 67.72, 66.80, 66.62, 66.46, 66.39, 65.37, 64.49, 62.27, 59.89, 59.60, 59.46, 49.35, 49.15, 33.75. MALDI-MS: Calculated for $C_{121}H_{130}N_{14}O_{27}F_6$ [M+Na]+: 2347.9026, found: 2347.8721.

Hexasaccharide S37

The reaction was carried out according to the general procedure D using compound **S36** (340 mg, 0.15 mmol), K_2CO_3 (30 mg, 0.22 mmol), KI (32 mg, 0.19 mmol) and $Ph_2BO(CH_2)_2NH_2$ (6.6 mg, 0.029 mmol). The product was purified by column chromatography (pentane:EtOAc = 4:1). Compound **S37** (309 g, 88% yield) was obtained as white foam. $[\alpha]_D^{25}$ +128 (c=0.2, $CHCl_3$). ^{1}H NMR (500 MHz, Chloroform-*d*) δ 7.52 – 7.03 (m, 65H), 6.37 (d, *J* = 9.5 Hz, 1H), 6.26 (d, *J* = 9.6 Hz, 1H), 5.74 (ddt, *J* = 17.0, 10.3, 6.7 Hz, 1H), 5.55 (d, *J* = 3.7 Hz, 1H), 5.10 – 4.89 (m, 8H), 4.83 (d, *J* = 3.7 Hz, 1H), 4.80 (d, *J* = 12.3 Hz, 1H), 4.77 – 4.68 (m, ^{1}H NMR (500 MHz, $CDCl_3$) δ 7.52 – 7.03 (m, 65H), 6.37 (d, *J* = 9.5 Hz, 1H), 6.26 (d, *J* = 9.6 Hz, 1H), 5.74 (ddt, *J* = 17.0, 10.3, 6.7 Hz, 1H), 5.55 (d, *J* = 3.7 Hz, 1H), 5.10 – 4.89 (m, 8H), 4.83 (d, *J* = 3.7 Hz, 1H), 4.80 (d, *J* = 12.3 Hz, 1H), 4.77 – 4.68 (m, 4H), 4.62 – 4.45 (m, 5H), 4.43 – 4.00 (m, 26H), 4.00 – 3.70 (m, 9H), 3.66 – 3.03 (m, 18H), 2.78 (s, 1H), 2.30 (q, *J* = 6.8 Hz, 2H).^{13}C NMR (125 MHz, $CDCl_3$) δ 156.52 (*a*d, *J* = 37 Hz, 2x*CF$_3$CO*), 138.48, 137.90, 137.78, 137.65, 137.62, 137.56, 137.51, 137.32, 137.20, 136.93, 136.81 (*aromatic* C), 134.46 (C-9), 128.53, 128.49, 128.47, 128.37, 128.36, 128.29, 128.27, 128.20, 128.15, 128.09, 128.02, 127.93, 127.88, 127.85, 127.81, 127.79, 127.76, 127.65, 127.60, 127.56, 127.50, 127.28, 127.22, 127.18, 126.95, 126.75 (*aromatic* C*H*), 116.90 (C-10), 115.85 (*a*d, *J* = 286 Hz, C*F$_3$*), 98.89, 98.45, 97.99, 97.35, 97.15, 80.62, 76.27, 76.14, 75.66, 75.22, 74.63, 74.42, 73.89, 73.55, 73.50, 73.09, 73.01, 72.97, 72.85, 72.76, 71.72, 71.63, 71.60, 70.98, 70.91, 70.78, 70.70, 70.46, 69.66, 69.54, 69.37, 68.80, 68.74, 68.65, 67.72, 66.69, 66.64, 66.50, 66.40, 65.53, 64.87, 59.89, 59.48, 59.19, 58.14, 49.31, 49.15, 33.76. MALDI-MS: Calculated for $C_{128}H_{136}N_{14}O_{27}F_6$ [M+Na]+: 2437.9496, found: 2437.9146.

Heptasaccharide 35

The reaction was carried out according to the general procedure B using donor **14** (338 mg, 0.50 mmol) and acceptor **S37** (302 mg, 0.13 mmol). The product was purified by column chromatography (pentane:EtOAc = 6:1). Compound **35** (276 mg, α-only, 76% yield) was obtained as yellow syrup. $[\alpha]_D^{25}$ +110.5 (c=0.2, $CHCl_3$). ^{1}H NMR (500 MHz, $CDCl_3$) δ 7.50 – 7.41 (m, 4H), 7.40 – 7.07 (m, 64H), 6.36 (d, *J* = 9.6 Hz, 1H), 6.30 (d, *J* = 9.3 Hz, 1H), 6.19 (d, *J* = 9.6 Hz, 1H), 5.83 – 5.67 (m, 1H), 5.44 (d, *J* = 3.6 Hz, 1H), 5.09 – 4.89 (m, 9H), 4.84 – 4.78 (m, 2H), 4.75 (d, *J* = 3.6 Hz, 1H), 4.73 – 4.67 (m, 3H), 4.66 – 4.55 (m, 3H), 4.54 – 4.43 (m, 6H), 4.42 – 3.91 (m, 30H), 3.89 – 3.72 (m, 4H), 3.73 – 3.31 (m, 11H), 3.30 – 3.04 (m, 10H), 2.31 (qt, *J* = 6.7, 1.4 Hz, 2H), 1.07 (s, 9H), 0.97 (s, 9H). ^{13}C NMR (125 MHz, $CDCl_3$) δ 156.73 (*a*d, *J* = 37 Hz, 3x*CF$_3$CO*), 138.50, 138.24, 137.92, 137.71, 137.68, 137.64, 137.59, 137.53, 137.44, 137.29, 137.13, 136.97, 136.89 (*aromatic* C), 134.59 (C-9), 128.68, 128.64, 128.61, 128.58, 128.56, 128.52, 128.51, 128.46, 128.44, 128.34, 128.31, 128.28, 128.25, 128.23, 128.21, 128.06, 128.03, 127.94, 127.92, 127.88, 127.82, 127.75, 127.72, 127.70, 127.56, 127.47, 127.24, 127.21, 127.14, 126.87, 126.72 (*aromatic* C*H*), 117.04 (C-10), 116.00 (*a*d, *J* = 286 Hz, 3xC*F$_3$*), 98.90, 98.59, 98.10, 97.68, 97.47, 97.29, 97.02, 80.37, 76.46, 76.18, 75.76, 75.43, 74.37, 74.22, 73.91, 73.70, 73.55, 73.24, 73.11, 73.06, 73.02, 72.72, 71.92, 71.89, 71.75, 71.15, 71.04, 70.84, 70.77, 70.75, 70.70, 70.03, 69.74, 69.68, 69.65, 69.43, 69.27, 68.87, 68.76, 68.70, 67.88, 67.83, 67.06, 66.76, 66.64, 66.58, 66.23, 65.68, 64.94, 60.23, 60.04, 59.62, 49.44, 49.26, 48.35, 33.91, 27.66, 27.47, 23.38, 20.79. MALDI-MS: Calculated for $C_{151}H_{168}N_{15}O_{32}F_9Si$ [M+Na]+: 2925.1498, found: 2925.0999.

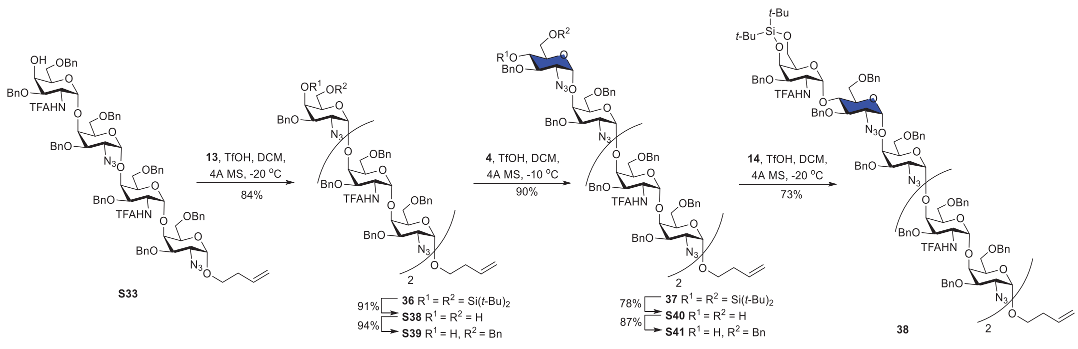

Pentasaccharide 36

The reaction was carried out according to the general procedure B using donor **13** (1.33 g, 2.19 mmol) and acceptor **S33** (1.23 g, 0.73 mmol). The product was purified by column chromatography (pentane:EtOAc = 8:1). Compound **36** (1.29 g, α-only, 84% yield) was obtained as white foam. $[\alpha]_D^{25}$ +144 (c=1, $CHCl_3$). ^{1}H NMR (500 MHz, $CDCl_3$) δ 7.50 – 7.08 (m, 50H), 6.32 (d, *J* = 9.8 Hz, 1H), 6.22 (d, *J* = 9.7 Hz, 1H), 5.75 (ddt, *J* = 17.1, 10.3, 6.8 Hz, 1H), 5.09 – 5.00 (m, 2H), 4.98 (d, *J* = 3.7 Hz, 1H), 4.97 – 4.91 (m, 3H), 4.85 – 4.81 (m, 1H), 4.80 – 4.71 (m, 3H), 4.71 –

4.55 (m, 3H), 4.54 – 4.45 (m, 5H), 4.42 – 4.32 (m, 5H), 4.32 – 4.18 (m, 6H), 4.16 – 4.02 (m, 4H), 4.00 (s, 1H), 3.98 (s, 1H), 3.96 (dd, *J* = 10.7, 2.6 Hz, 2H), 3.88 – 3.59 (m, 9H), 3.55 (ddd, *J* = 10.7, 7.9, 2.4 Hz, 2H), 3.49 (dt, *J* = 9.7, 6.5 Hz, 1H), 3.35 (dd, *J* = 9.2, 5.8 Hz, 1H), 3.31 – 3.19 (m, 4H), 3.13 – 3.03 (m, 3H), 2.31 (qt, *J* = 6.7, 1.4 Hz, 2H), 1.02 (s, 9H), 0.98 (s, 9H). ^{13}C NMR (125 MHz, $CDCl_3$) δ 156.81 (*a*d, *J* = 37 Hz, 2x*CF_3CO*), 138.02, 137.70, 137.66, 137.42, 137.33, 137.31, 137.26, 137.00, 136.95 (*aromatic* C), 134.58 (C-9), 128.71, 128.69, 128.67, 128.62, 128.55, 128.51, 128.43, 128.35, 128.28, 128.21, 128.16, 128.13, 128.12, 128.02, 127.96, 127.91, 127.80, 127.72, 127.66, 127.64, 127.13, 126.96, 126.77 (*aromatic* C*H*), 117.03 (C-10), 115.95 (*a*d, *J* = 286 Hz, 2xC*F_3*), 98.65, 98.49, 98.10, 97.48, 97.28, 76.51, 76.07, 75.73, 75.16, 74.20, 73.69, 73.32, 73.23, 73.09, 71.85, 71.74, 71.21, 71.06, 70.88, 70.71, 70.69, 70.64, 70.34, 69.69, 69.66, 68.86, 68.79, 67.87, 67.50, 67.22, 66.66, 66.56, 66.45, 65.70, 60.01, 59.61, 58.60, 49.27, 49.25, 33.89, 27.66, 27.49, 23.33, 20.79. HR-MS: Calculated for $C_{109}H_{125}N_{11}O_{23}F_6Si$ $[M+NH_4]^+$: 2115.89615, found: 2115.89912.

Pentasaccharide S38

The reaction was carried out according to the general procedure C using compound **36** (1.20 g, 0.57 mmol) and HF/pyridine (70%, 238 µl, 9.14 mmol). The product was purified by column chromatography (pentane:EtOAc = 2:1). Compound **S38** (1.02 g, 91% yield) was obtained as white foam. $[\alpha]_D^{25}$ +158.2 (c=1, $CHCl_3$). ^{1}H NMR (500 MHz, $CDCl_3$) δ 7.49 – 7.09 (m, 48H), 6.47 (d, *J* = 9.7 Hz, 1H), 6.36 (d, *J* = 9.6 Hz, 1H), 5.75 (ddt, *J* = 17.0, 10.2, 6.7 Hz, 1H), 5.09 – 4.96 (m, 3H), 4.96 – 4.89 (m, 3H), 4.85 (d, *J* = 3.7 Hz, 1H), 4.80 (d, *J* = 12.4 Hz, 1H), 4.77 (d, *J* = 3.6 Hz, 1H), 4.75 – 4.65 (m, 3H), 4.58 (d, *J* = 12.2 Hz, 1H), 4.54 – 4.43 (m, 4H), 4.41 – 4.34 (m, 4H), 4.34 – 4.23 (m, 4H), 4.23 – 4.04 (m, 8H), 4.03 – 3.94 (m, 5H), 3.87 – 3.75 (m, 4H), 3.67 (dd, *J* = 10.5, 3.5 Hz, 1H), 3.65 – 3.54 (m, 3H), 3.47 (dq, *J* = 8.4, 5.9, 5.3 Hz, 2H), 3.43 – 3.24 (m, 5H), 3.21 (dd, *J* = 8.7, 5.2 Hz, 1H), 3.15 – 3.02 (m, 3H), 2.84 (s, 1H), 2.37 – 2.29 (m, 3H). ^{13}C NMR (125 MHz, $CDCl_3$) δ 156.86 (*a*d, *J* = 37 Hz, 2x*CF_3CO*), 137.58, 137.54, 137.45, 137.38, 137.35, 137.29, 137.19, 137.00, 136.87 (*aromatic* C), 134.50 (C-9), 128.61, 128.57, 128.54, 128.49, 128.39, 128.38, 128.34, 128.31, 128.28, 128.18, 128.12, 128.05, 127.96, 127.94, 127.90, 127.86, 127.83, 127.81, 127.64, 127.52, 127.17, 127.03, 126.85 (*aromatic* C*H*), 116.95 (C-10), 115.85 (*a*d, *J* = 286 Hz, 2xC*F_3*), 98.85, 98.56, 98.04, 97.45, 97.17, 76.58, 76.29, 75.65, 74.97, 74.13, 73.60, 73.16, 73.06, 72.98, 71.84, 71.79, 71.68, 71.18, 71.06, 70.81, 70.79, 70.75, 69.74, 69.55, 69.12, 68.81, 68.67, 67.78, 67.50, 66.58, 66.54, 66.48, 65.66, 62.63, 59.95, 59.51, 59.48, 49.36, 49.21, 33.81. HR-MS: Calculated for $C_{101}H_{109}N_{11}O_{23}F_6$ $[M+NH_4]^+$: 1975.79402, found: 1975.79588.

Pentasaccharide S39

The reaction was carried out according to the general procedure D using compound **S38** (1.06 g, 0.54 mmol), K_2CO_3 (112 mg, 0.81 mmol), KI (117 mg, 0.7 mmol) and $Ph_2BO(CH_2)_2NH_2$ (24 mg, 0.11 mmol). The product was purified by column chromatography (pentane:EtOAc = 4:1). Compound **S39** (1.05 g, 94% yield) was obtained as white foam. $[\alpha]_D^{25}$ +162.6 (c=1, $CHCl_3$). ^{1}H NMR (500 MHz, $CDCl_3$) δ 7.49 – 7.07 (m, 57H), 6.34 (d, *J* = 9.7 Hz, 1H), 6.23 (d, *J* = 9.6 Hz, 1H), 5.74 (ddt, *J* = 17.1, 10.2, 6.7 Hz, 1H), 5.09 – 4.98 (m, 3H), 4.96 (d, *J* = 3.7 Hz, 1H), 4.94 – 4.90 (m, 2H), 4.83 (d, *J* = 3.7 Hz, 1H), 4.80 (d, *J* = 12.3 Hz, 1H), 4.77 – 4.65 (m, 4H), 4.57 (d, *J* = 12.3 Hz, 1H), 4.54 – 4.45 (m, 4H), 4.42 – 4.33 (m, 5H), 4.32 – 4.23 (m, 7H), 4.22 – 4.11 (m, 4H), 4.10 – 4.02 (m, 3H), 4.01 – 3.94 (m, 4H), 3.87 – 3.74 (m, 5H), 3.66 – 3.54 (m, 3H), 3.51 – 3.41 (m, 2H), 3.39 – 3.18 (m, 6H), 3.14 – 3.05 (m, 4H), 2.30 (qt, *J* = 6.8, 1.4 Hz, 2H). ^{13}C NMR (125 MHz, $CDCl_3$) δ 156.69 (*a*d, *J* = 37 Hz, 2x*CF_3CO*), 137.62, 137.59, 137.57, 137.56, 137.47, 137.44, 137.33, 137.22, 137.00, 136.84 (*aromatic* C), 134.49 (C-9), 128.56, 128.52, 128.46, 128.41, 128.38, 128.33, 128.29, 128.18, 128.11, 127.98, 127.95, 127.93, 127.86, 127.81, 127.70, 127.62, 127.61, 127.21, 127.01, 126.78 (*aromatic* C*H*), 116.94 (C-10), 115.75 (*a*d, *J* = 286 Hz, 2xC*F_3*), 98.94, 98.53, 98.00, 97.37, 97.20, 77.36, 76.61, 76.33, 75.65, 74.89, 73.97, 73.58, 73.36, 73.13, 73.06, 72.98, 71.81, 71.63, 71.55, 71.44, 71.08, 70.95, 70.77, 70.75, 70.66, 69.78, 69.69, 69.57, 68.77, 68.70, 68.24, 67.76, 66.89, 66.58, 66.50, 66.45, 65.65, 59.92, 59.51, 59.36, 49.27, 49.17, 33.80. HR-MS: Calculated for $C_{108}H_{115}N_{11}O_{23}F_6$ $[M+Na]^+$: 2070.79637, found: 2070.79877.

Hexasaccharide 37

The reaction was carried out according to the general procedure A. The donor **4** (178 mg, 0.29 mmol) and acceptor **S39** (150 mg, 0.073 mmol) were co-evaporated with toluene (three times). The residue was dissolved in dry 2 ml DCM under nitrogen and stirred over fresh flame-dried molecular sieves 4Å. The solution was cooled to -10 °C, after which TfOH (2.6 µl, 0.03 mmol) was added. The reaction was stirred at -10 °C for overnight. Then the reaction was quenched with Et_3N, diluted with DCM, washed with saturated $NaHCO_3$ and brine. The organic phase was dried with anhydrous $MgSO_4$, filtered and concentrated *in vacuo*. The product was purified by silica gel column chromatography (pentane:EtOAc = 6:1). Compound **37** (162 mg, α-only, 90% yield) was obtained as white foam. $[\alpha]_D^{25}$ +135.6 (c=1, $CHCl_3$). ^{1}H NMR (500 MHz, $CDCl_3$) δ 7.47 – 7.08 (m, 64H), 6.31 (d, *J* = 9.7 Hz, 1H), 6.17 (d, *J* = 9.7 Hz, 1H), 5.75 (ddt, *J* = 17.0, 10.3, 6.7 Hz, 1H), 5.12 – 5.00 (m, 3H), 4.98

(d, J = 3.7 Hz, 1H), 4.95 – 4.89 (m, 3H), 4.86 – 4.73 (m, 7H), 4.58 (dd, J = 14.7, 12.1 Hz, 2H), 4.53 – 4.44 (m, 4H), 4.41 – 4.18 (m, 15H), 4.18 – 3.87 (m, 13H), 3.86 – 3.73 (m, 8H), 3.70 – 3.52 (m, 7H), 3.49 (dt, J = 9.7, 6.5 Hz, 1H), 3.34 (dd, J = 9.2, 5.8 Hz, 1H), 3.31 – 3.18 (m, 4H), 3.16 – 3.00 (m, 5H), 2.31 (qt, J = 6.8, 1.4 Hz, 2H), 1.02 (s, 10H), 0.97 (s, 10H). ^{13}C NMR (125 MHz, $CDCl_3$) δ 156.77 (*a*d, J = 37 Hz, 2x*CF$_3$CO*), 138.29, 137.84, 137.74, 137.68, 137.58, 137.43, 137.35, 137.32, 137.12, 136.97 (*aromatic* C), 134.59 (C-9), 128.68, 128.62, 128.57, 128.52, 128.48, 128.46, 128.45, 128.37, 128.32, 128.29, 128.25, 128.22, 128.14, 128.05, 127.97, 127.95, 127.92, 127.85, 127.83, 127.79, 127.73, 127.67, 127.50, 127.15, 126.98, 126.91, 119.42, 119.33 (*aromatic* C*H*), 117.05 (C-10), 116.00 (*a*d, J = 286 Hz, 2xC*F$_3$*), 98.68, 98.66, 98.48, 98.11, 97.47, 97.42, 79.36, 79.27, 76.41, 76.36, 75.73, 75.53, 75.36, 73.98, 73.71, 73.22, 73.19, 73.11, 72.97, 72.30, 71.93, 71.74, 71.31, 71.14, 71.03, 70.87, 70.84, 69.77, 69.69, 69.06, 68.89, 68.85, 67.88, 66.79, 66.72, 66.60, 66.43, 66.13, 65.82, 63.04, 60.34, 60.00, 59.63, 49.34, 49.28, 33.92, 27.48, 27.22, 22.67, 20.07. MALDI-MS: Calculated for $C_{129}H_{146}N_{14}O_{27}F_6Si$ [M+Na]+: 2488.0048, found: 2487.9691.

Hexasaccharide S40

The reaction was carried out according to the general procedure C using compound **37** (555 mg, 0.23 mmol) and HF/pyridine (70%, 94 µl, 3.6 mmol). The product was purified by column chromatography (pentane:EtOAc = 2:1). Compound **S40** (463 mg, 88% yield) was obtained as white foam. $[\alpha]_D^{25}$ +148.5 (c=1, $CHCl_3$). ^{1}H NMR (500 MHz, $CDCl_3$) δ 7.43 – 7.11 (m, 59H), 6.45 (d, J = 9.0 Hz, 2H), 5.73 (ddd, J = 16.9, 10.6, 5.1 Hz, 1H), 5.12 – 4.62 (m, 16H), 4.62 – 3.69 (m, 40H), 3.60 (dq, J = 30.8, 12.9, 11.5 Hz, 6H), 3.44 (q, J = 7.2 Hz, 1H), 3.38 – 2.91 (m, 13H), 2.28 (q, J = 6.9 Hz, 2H), 1.91 (s, 1H). ^{13}C NMR (125 MHz, $CDCl_3$) δ 156.57 (*a*d, J = 37 Hz, 2x*CF$_3$CO*), 138.14, 137.55, 137.50, 137.44, 137.27, 137.22, 137.19, 136.85, 136.73 (*aromatic* C), 134.37 (C-9), 128.63, 128.44, 128.39, 128.32, 128.27, 128.24, 128.21, 128.15, 128.06, 127.99, 127.97, 127.88, 127.82, 127.79, 127.75, 127.69, 127.65, 127.62, 127.52, 127.46, 127.28, 126.88, 126.84, 126.70 (*aromatic* C*H*), 116.84 (C-10), 115.78 (*a*d, J = 286 Hz, 2xC*F$_3$*), 98.58, 98.41, 98.15, 97.93, 97.39, 97.12, 79.37, 76.11, 75.77, 75.53, 74.71, 74.02, 73.43, 73.01, 72.92, 72.83, 72.79, 71.98, 71.57, 71.38, 70.99, 70.64, 70.46, 69.48, 69.39, 68.73, 68.72, 67.64, 66.51, 66.28, 65.58, 63.15, 61.33, 59.78, 59.37, 49.12, 49.10, 33.66. MALDI-MS: Calculated for $C_{121}H_{130}N_{14}O_{27}F_6$ [M+Na]+: 2347.9026, found: 2347.8711.

Hexasaccharide S41

The reaction was carried out according to the general procedure D using compound **S40** (450 mg, 0.19 mmol), K_2CO_3 (40 mg, 0.29 mmol), KI (42 mg, 0.25 mmol) and $Ph_2BO(CH_2)_2NH_2$ (8.7 mg, 0.039 mmol). The product was purified by column chromatography (pentane:EtOAc = 4:1). Compound **S41** (402 mg, 97% yield) was obtained as white foam. $[\alpha]_D^{25}$ +148.6 (c=0.5, $CHCl_3$). ^{1}H NMR (500 MHz, $CDCl_3$) δ 7.46 – 7.13 (m, 62H), 6.40 (d, J = 9.4 Hz, 1H), 6.32 (d, J = 9.4 Hz, 1H), 5.73 (ddt, J = 17.0, 10.3, 6.7 Hz, 1H), 5.10 – 4.70 (m, 15H), 4.63 – 3.93 (m, 35H), 3.89 – 3.70 (m, 7H), 3.69 – 3.41 (m, 7H), 3.38 – 2.95 (m, 13H), 2.76 (s, 1H), 2.28 (q, J = 6.8 Hz, 2H). ^{13}C NMR (125 MHz, $CDCl_3$) δ 156.55 (*a*d, J = 37 Hz, *CF$_3$CO*), 138.20, 137.67, 137.65, 137.58, 137.48, 137.43, 137.27, 137.24, 137.14, 136.86, 136.71 (*aromatic* C), 134.37 (C-9), 128.79, 128.45, 128.40, 128.34, 128.30, 128.28, 128.26, 128.22, 128.20, 128.15, 128.08, 128.02, 127.91, 127.87, 127.83, 127.81, 127.78, 127.74, 127.70, 127.68, 127.64, 127.59, 127.54, 127.50, 127.46, 127.28, 127.07, 126.89, 126.85, 126.78, 126.72 (*aromatic* C*H*), 116.83 (C-10), 115.79 (*a*d, J = 286 Hz, C*F$_3$*), 98.62, 98.44, 97.93, 97.28, 97.15, 79.46, 76.13, 75.58, 74.90, 74.83, 73.88, 73.46, 73.12, 73.04, 72.94, 72.85, 72.75, 72.61, 71.63, 71.51, 70.95, 70.82, 70.62, 70.55, 69.60, 69.51, 69.44, 69.23, 68.91, 68.67, 68.61, 67.64, 66.49, 66.29, 66.17, 65.57, 63.18, 59.84, 59.78, 59.39, 58.56, 58.04, 54.55, 49.15, 49.08, 33.68. MALDI-MS: Calculated for $C_{128}H_{136}N_{14}O_{27}F_6$ [M+Na]+: 2437.9496, found: 2437.9159.

Heptasaccharide 38

The reaction was carried out according to the general procedure B using donor **14** (442 mg, 0.65 mmol) and acceptor **S41** (395 mg, 0.16 mmol). The product was purified by column chromatography (pentane:EtOAc = 6:1). Compound **38** (380 mg, α-only, 80% yield) was obtained as white foam. $[\alpha]_D^{25}$ +120 (c=0.3, $CHCl_3$). ^{1}H NMR (500 MHz, $CDCl_3$) δ 7.46 – 7.42 (m, 2H), 7.39 – 7.11 (m, 69H), 6.89 (d, J = 9.5 Hz, 1H), 6.36 (d, J = 9.7 Hz, 1H), 6.22 (d, J = 9.6 Hz, 1H), 5.75 (ddt, J = 17.0, 10.2, 6.7 Hz, 1H), 5.45 (d, J = 3.5 Hz, 1H), 5.10 – 4.97 (m, 5H), 4.95 – 4.89 (m, 2H), 4.84 (d, J = 3.7 Hz, 1H), 4.83 – 4.62 (m, 8H), 4.61 – 4.33 (m, 15H), 4.33 – 4.13 (m, 11H), 4.11 – 3.87 (m, 14H), 3.87 – 3.74 (m, 5H), 3.72 – 3.41 (m, 9H), 3.35 (dd, J = 9.2, 5.8 Hz, 1H), 3.31 – 3.18 (m, 5H), 3.15 – 3.06 (m, 4H), 3.05 – 2.99 (m, 1H), 2.95 (d, J = 10.5 Hz, 1H), 2.31 (qt, J = 6.7, 1.3 Hz, 2H), 1.02 (d, J = 6.0 Hz, 17H). ^{13}C NMR (125 MHz, $CDCl_3$) δ 156.99 (*a*d, J = 37 Hz, 3x*CF$_3$CO*), 138.12, 137.68, 137.66, 137.62, 137.59, 137.52, 137.39, 137.29, 137.08, 136.92, 136.46 (*aromatic* C), 134.55 (C-9), 128.64, 128.60, 128.56, 128.53, 128.48, 128.44, 128.42, 128.38, 128.36, 128.26, 128.22, 128.19, 128.07, 128.01, 128.00, 127.91, 127.89, 127.86, 127.78, 127.75, 127.74, 127.69, 127.62, 127.37, 127.11, 127.04, 126.87 (*aromatic* C*H*), 117.03 (C-10), 115.97 (*a*d, J = 286 Hz, 3xC*F$_3$*), 98.80, 98.63, 98.08, 98.01, 97.45, 97.38, 97.13, 79.80, 76.38,

76.22, 75.70, 75.31, 75.19, 74.27, 74.06, 73.67, 73.52, 73.21, 73.14, 73.06, 72.99, 72.52, 71.95, 71.92, 71.71, 71.35, 71.19, 71.02, 70.87, 70.84, 70.62, 69.74, 69.65, 69.61, 68.98, 68.84, 68.77, 68.48, 67.85, 67.80, 67.10, 66.68, 66.62, 66.54, 66.30, 65.79, 63.99, 60.43, 59.99, 59.59, 49.31, 49.24, 48.61, 33.88, 27.66, 27.36, 23.39, 20.80. MALDI-MS: Calculated for $C_{151}H_{168}N_{15}O_{32}F_9Si$ [M+Na]+: 2925.1498, found: 2925.1007.

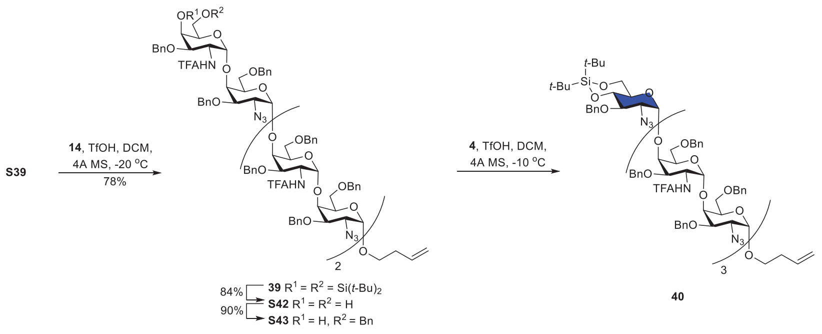

Hexasaccharide 39

The reaction was carried out according to the general procedure B using donor **14** (597 mg, 0.94 mmol) and acceptor **S39** (480 mg, 0.23 mmol). The product was purified by column chromatography (pentane:EtOAc = 6:1). Compound **39** (462 mg, α-only, 78% yield) was obtained as white foam. $[\alpha]_D^{25}$ +149.7 (c=1, $CHCl_3$). ^1H NMR (500 MHz, $CDCl_3$) δ 7.44 (d, *J* = 7.6 Hz, 2H), 7.37 – 7.10 (m, 56H), 6.34 (d, *J* = 9.5 Hz, 1H), 6.26 (dd, *J* = 15.4, 9.4 Hz, 2H), 5.81 – 5.67 (m, 1H), 5.08 – 4.98 (m, 4H), 4.96 – 4.89 (m, 2H), 4.89 – 4.55 (m, 11H), 4.54 – 4.42 (m, 7H), 4.42 – 4.33 (m, 6H), 4.33 – 4.09 (m, 9H), 4.06 – 3.91 (m, 9H), 3.88 – 3.74 (m, 5H), 3.71 – 3.53 (m, 5H), 3.46 (dt, *J* = 14.0, 8.6 Hz, 2H), 3.38 – 3.17 (m, 6H), 3.16 – 2.96 (m, 5H), 2.30 (q, *J* = 6.8 Hz, 2H), 1.07 (s, 11H), 0.98 (s, 10H). ^{13}C NMR (125 MHz, $CDCl_3$) δ 156.59 (*a*d, *J* = 37 Hz, 3x*CF$_3$CO*), 137.84, 137.59, 137.57, 137.52, 137.36, 137.31, 137.18, 137.17, 136.95, 136.84, 136.80 (*aromatic* C), 134.44 (C-9), 128.60, 128.53, 128.48, 128.43, 128.39, 128.35, 128.33, 128.30, 128.24, 128.15, 128.13, 128.08, 128.04, 128.01, 127.90, 127.88, 127.81, 127.79, 127.77, 127.71, 127.70, 127.62, 127.56, 126.98, 126.94, 126.71 (*aromatic* C*H*), 116.90 (C-10), 115.81 (*a*d, *J* = 286 Hz, 3xC*F$_3$*), 98.49, 98.31, 98.01, 97.33, 97.22, 96.65, 76.35, 76.14, 75.66, 75.07, 74.65, 73.97, 73.55, 73.02, 72.94, 72.91, 71.83, 71.60, 71.05, 70.93, 70.88, 70.72, 69.52, 69.44, 69.38, 69.22, 68.72, 68.66, 68.56, 67.73, 67.69, 66.87, 66.57, 66.48, 66.38, 65.73, 65.66, 60.20, 59.94, 59.49, 49.15, 48.19, 33.77, 27.52, 27.35, 23.21, 20.63. MALDI-MS: Calculated for $C_{131}H_{147}N_{12}O_{28}F_9Si$ [M+Na]+: 2557.9966, found: 2557.9584.

Hexasaccharide S42

The reaction was carried out according to the general procedure C using compound **39** (445 mg, 0.18 mmol) and HF/pyridine (70%, 73 µl, 2.8 mmol). The product was purified by column chromatography (pentane:EtOAc = 3:2). Compound **S42** (353 mg, 84% yield) was obtained as white foam. $[\alpha]_D^{25}$ +141.8 (c=0.5, $CHCl_3$). ^1H NMR (500 MHz, $CDCl_3$) δ 7.46 – 7.11 (m, 64H), 6.46 (d, *J* = 9.5 Hz, 1H), 6.33 (d, *J* = 9.6 Hz, 1H), 6.30 (d, *J* = 9.6 Hz, 1H), 5.83 – 5.67 (m, 1H), 5.09 – 5.00 (m, 2H), 4.99 (d, *J* = 3.6 Hz, 1H), 4.97 (d, *J* = 3.7 Hz, 1H), 4.95 – 4.89 (m, 2H), 4.86 (d, *J* = 3.7 Hz, 1H), 4.84 (d, *J* = 3.7 Hz, 1H), 4.80 (d, *J* = 12.3 Hz, 1H), 4.77 (d, *J* = 3.6 Hz, 1H), 4.73 (d, *J* = 11.8 Hz, 1H), 4.65 – 4.46 (m, 9H), 4.45 – 4.33 (m, 8H), 4.32 – 4.12 (m, 10H), 4.07 – 3.90 (m, 12H), 3.87 – 3.70 (m, 5H), 3.65 – 3.42 (m, 7H), 3.39 – 3.20 (m, 8H), 3.14 – 3.02 (m, 5H), 2.90 (s, 1H), 2.31 (q, *J* = 6.7 Hz, 2H). ^{13}C NMR (125 MHz, $CDCl_3$) δ 156.73 (*a*d, *J* = 37 Hz, 3x*CF$_3$CO*), 137.67, 137.63, 137.57, 137.37, 137.31, 137.28, 137.10, 137.01, 136.89 (*aromatic* C), 134.52 (C-9), 128.58, 128.53, 128.51, 128.39, 128.37, 128.34, 128.18, 128.13, 128.11, 128.03, 127.95, 127.88, 127.84, 127.81, 127.60, 127.53, 127.29, 127.12, 127.05, 126.75 (*aromatic* C*H*), 116.95 (C-10), 115.86 (*a*d, *J* = 286 Hz, 3xC*F$_3$*), 98.53, 98.40, 98.04, 97.43, 97.24, 96.93, 76.42, 76.09, 75.68, 75.14, 74.71, 74.08, 73.60, 73.08, 73.06, 72.97, 72.91, 71.92, 71.90, 71.68, 71.20, 71.11, 71.06, 70.93, 70.78, 70.71, 70.67, 69.53, 69.42, 69.29, 68.80, 68.70, 68.59, 67.78, 66.64, 66.58, 66.51, 66.39, 65.86, 65.73, 62.44, 60.18, 60.00, 59.53, 49.22, 49.18, 48.82, 33.82. MALDI-MS: Calculated for $C_{123}H_{131}N_{12}O_{28}F_9$ [M+Na]+: 2417.8944, found: 2417.8591.

Hexasaccharide S43

The reaction was carried out according to the general procedure D using compound **S42** (347 mg, 0.15 mmol), K_2CO_3 (30 mg, 0.22 mmol), KI (31 mg, 0.19 mmol) and $Ph_2BO(CH_2)_2NH_2$ (6.5 g, 0.03 mmol). The product was purified by column chromatography (pentane:EtOAc = 4:1). Compound **S43** (330 mg, 92% yield) was obtained as white foam. $[\alpha]_D^{25}$ +145.6 (c=0.5, $CHCl_3$). ^1H NMR (500 MHz, $CDCl_3$) δ 7.46 – 7.08 (m, 66H), 6.43 (d, *J* = 9.5 Hz, 1H), 6.31 (t, *J* = 8.8 Hz, 2H), 5.74 (ddt, *J* = 17.0, 10.3, 6.7 Hz, 1H), 5.08 – 4.87 (m, 6H), 4.86 – 4.78 (m, 4H), 4.76 (d, *J*

= 3.6 Hz, 1H), 4.66 (d, *J* = 12.1 Hz, 1H), 4.60 – 4.43 (m, 8H), 4.42 – 4.12 (m, 20H), 4.07 – 3.90 (m, 9H), 3.89 – 3.71 (m, 4H), 3.68 – 3.43 (m, 7H), 3.40 – 3.18 (m, 7H), 3.15 – 3.02 (m, 5H), 2.96 (s, 1H), 2.30 (q, *J* = 6.7 Hz, 2H). ^{13}C NMR (125 MHz, $CDCl_3$) δ 156.62 (*a*d, *J* = 37 Hz, 3x*C*F_3*C*O), 137.68, 137.59, 137.51, 137.40, 137.32, 137.25, 137.22, 137.05, 136.93, 136.83 (*aromatic* C), 134.46 (C-9), 128.89, 128.52, 128.46, 128.44, 128.38, 128.34, 128.32, 128.31, 128.27, 128.14, 128.07, 128.05, 127.96, 127.91, 127.89, 127.86, 127.78, 127.76, 127.72, 127.62, 127.58, 127.54, 127.50, 127.31, 127.20, 126.96, 126.70 (*aromatic* C*H*), 116.89 (C-10), 115.87 (*a*d, *J* = 286 Hz, C*F_3*), 98.49, 98.41, 97.99, 97.39, 97.16, 97.09, 76.35, 75.97, 75.65, 75.25, 74.55, 74.05, 73.54, 73.41, 73.02, 72.93, 72.91, 72.87, 71.85, 71.79, 71.13, 71.04, 71.01, 70.81, 70.75, 70.69, 70.59, 70.38, 69.52, 69.38, 69.08, 68.76, 68.65, 68.62, 67.73, 66.58, 66.50, 66.43, 65.75, 65.65, 65.58, 59.95, 59.48, 58.14, 49.17, 49.11, 48.88, 33.76. MALDI-MS: Calculated for $C_{130}H_{137}N_{12}O_{28}F_9$ [M+Na]+: 2507.9414, found: 2507.9043.

Heptasaccharide 40

The reaction was carried out according to the general procedure A. The donor **4** (312 mg, 0.51 mmol) and acceptor **S43** (320 mg, 0.13 mmol) were co-evaporated with toluene (three times). The residue was dissolved in dry 3 ml DCM under nitrogen and stirred over fresh flame-dried molecular sieves 4Å. The solution was cooled to -10 ^oC, after which TfOH (7.7 µl, 0.05 mmol) was added. The reaction was stirred at -10 ^oC for overnight. Then the reaction was quenched with Et_3N, diluted with DCM, washed with saturated $NaHCO_3$ and brine. The organic phase was dried with anhydrous $MgSO_4$, filtered and concentrated *in vacuo*. The product was purified by silica gel column chromatography (pentane:EtOAc = 6:1). Compound **40** (320 mg, α-only, 85% yield) was obtained as white foam. $[\alpha]_D^{25}$ +131.6 (c=0.5, $CHCl_3$). ^{1}H NMR (500 MHz, $CDCl_3$) δ 7.47 – 7.03 (m, 81H), 6.19 (d, *J* = 9.6 Hz, 2H), 6.07 (d, *J* = 9.4 Hz, 1H), 5.82 – 5.67 (m, 1H), 5.12 – 4.99 (m, 5H), 4.96 (d, *J* = 3.6 Hz, 1H), 4.94 – 4.88 (m, 4H), 4.85 – 4.73 (m, 8H), 4.61 – 4.11 (m, 32H), 4.10 – 3.97 (m, 6H), 3.96 – 3.71 (m, 17H), 3.66 – 3.44 (m, 6H), 3.35 (dd, *J* = 9.2, 5.8 Hz, 1H), 3.30 – 3.19 (m, 6H), 3.17 – 3.00 (m, 8H), 2.31 (qt, *J* = 6.8, 1.4 Hz, 2H), 1.04 (s, 9H), 1.01 (s, 10H). ^{13}C NMR (125 MHz, $CDCl_3$) δ 156.69 (*a*d, *J* = 37 Hz, 3x*C*F_3*C*O), 138.33, 137.81, 137.71, 137.68, 137.61, 137.40, 137.32, 137.29, 137.28, 137.22, 137.04, 136.93 (*aromatic* C), 134.56 (C-9), 128.65, 128.58, 128.53, 128.44, 128.38, 128.32, 128.26, 128.24, 128.18, 128.10, 128.08, 128.02, 128.00, 127.92, 127.90, 127.87, 127.84, 127.79, 127.75, 127.72, 127.69, 127.67, 127.63, 127.11, 126.84, 126.82 (*aromatic* C*H*), 117.03 (C-10), 115.96 (*a*d, *J* = 286 Hz, 3xC*F_3*), 98.60, 98.54, 98.11, 98.08, 97.45, 97.34, 97.24, 79.40, 79.29, 76.50, 76.45, 75.74, 74.28, 74.04, 73.67, 73.23, 73.20, 73.13, 73.06, 72.98, 72.93, 71.93, 71.73, 71.28, 71.16, 71.04, 71.02, 70.83, 70.78, 70.76, 70.67, 69.64, 69.49, 69.44, 68.84, 68.77, 67.85, 67.10, 66.81, 66.55, 66.27, 65.76, 62.89, 60.04, 59.59, 49.30, 49.24, 49.19, 33.88, 27.44, 27.06, 22.70. MALDI-MS: Calculated for $C_{151}H_{168}N_{15}O_{32}F_9Si$ [M+Na]+: 2925.1498, found: 2925.1035.

General procedure for desilylation and Birch reduction of the oligosaccharides towards 41 - 46 (general procedure E)

HF/pyridine (16 eq) solution was added to a solution of starting material in THF at 0 ^oC. The reaction was warmed to room temperature and stirred until TLC-analysis indicated full consumption of the starting material (± 1h). Then the mixture was diluted with DCM and washed with saturated $NaHCO_3$ and brine, dried with anhydrous $MgSO_4$, filtered and concentrated *in vacuo*. The residue was purified by silica gel column chromatography. Ammonia (10 ml) was condensed at -78 oC, the residue was dissolved in THF (2 ml) and tert-butanol (0.8 ml) and slowly added to the flask containing ammonia. Allyl carbinol (200 ul) was added to the reaction mixture. Small pieces of sodium was added to the reaction mixture one by one to keep deep blue for 15 min. Then ammonia acetate (100 mg) was added. The solution was allowed to warm to room temperature and stirred until all of the ammonia was evaporated. The solution was concentrated *in vacuo* and purified by gel filtration (HW-40, 0.15M NH_4OAc in H_2O). The product containing fractions were pooled and lyophilized (4x) to yield the final products as a white solid.

Heptasaccharide (41)

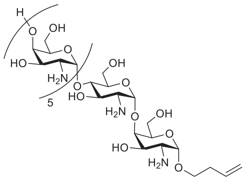

(69% yield, 12/1 with:without C=C). The reaction was carried out according to the general procedure E. ^{1}H NMR (500 MHz, Deuterium Oxide) δ 5.88 (ddt, *J* = 17.1, 10.3, 6.6 Hz, 1H), 5.67 (d, *J* = 4.0 Hz, 1H), 5.33 – 5.23 (m, 4H), 5.19 – 5.12 (m, 3H), 5.11 – 5.07 (m, 1H), 4.48 (q, *J* = 5.4 Hz, 3H), 4.41 (t, *J* = 6.4 Hz, 1H), 4.31 – 4.20 (m, 5H), 4.19 – 4.00 (m, 11H), 3.86 – 3.71 (m, 16H), 3.66 – 3.53 (m, 6H), 3.49 (dd, *J* = 10.9, 3.8 Hz, 1H), 3.19 (dd, *J* = 10.8, 3.6 Hz, 1H), 2.42 – 2.35 (m, 2H). ^{13}C NMR (125 MHz, D_2O) δ 135.71, 116.71, 97.36, 97.19, 96.91, 96.79, 96.53, 95.49, 76.54, 76.28, 76.23, 76.15, 75.34, 71.53, 71.44, 70.98, 70.90, 70.72, 67.90, 67.65, 66.76, 66.68, 66.52, 60.56, 60.47, 60.26, 60.10, 54.77, 50.98, 50.91, 33.10. Calculated for $C_{46}H_{85}N_7O_{29}$ $[M+2H]^{2+}$: 600.77686, found: 600.44676.

Heptasaccharide (42)

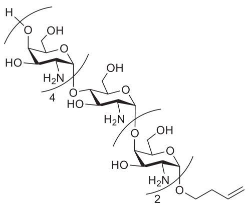

(48% yield, 25/1 with:without C=C). The reaction was carried out according to the general procedure E. ^{1}H NMR (500 MHz, Deuterium Oxide) δ 5.88 (ddt, J = 17.1, 10.4, 6.6 Hz, 1H), 5.70 (d, J = 3.9 Hz, 1H), 5.32 (d, J = 3.8 Hz, 1H), 5.29 (dd, J = 5.7, 3.9 Hz, 2H), 5.26 (d, J = 3.8 Hz, 1H), 5.22 (d, J = 3.8 Hz, 1H), 5.19 (d, J = 3.8 Hz, 1H), 5.18 – 5.13 (m, 1H), 5.09 (ddt, J = 10.4, 2.3, 1.3 Hz, 1H), 4.53 – 4.46 (m, 3H), 4.42 (t, J = 6.4 Hz, 1H), 4.33 – 4.00 (m, 19H), 3.87 – 3.69 (m, 18H), 3.67 – 3.59 (m, 5H), 3.59 – 3.50 (m, 3H), 3.26 (dd, J = 11.0, 3.5 Hz, 1H), 2.39 (q, J = 6.6 Hz, 2H). ^{13}C NMR (125 MHz, D_2O) δ 135.70, 116.72, 97.13, 96.81, 96.65, 96.48, 96.31, 95.33, 76.50, 76.30, 76.19, 76.13, 75.17, 71.44, 71.22, 70.97, 70.72, 70.66, 67.87, 67.67, 66.59, 66.40, 60.58, 60.54, 60.47, 60.26, 60.13, 60.01, 54.67, 51.00, 50.91, 50.85, 33.11. HR-MS: Calculated for $C_{46}H_{85}N_7O_{29}$ $[M+2H]^{2+}$: 600.77686, found: 600.46824.

Heptasaccharide (43)

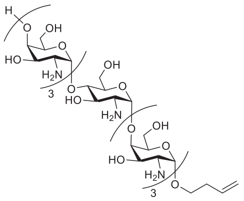

(84% yield, 19/1). The reaction was carried out according to the general procedure E. ^{1}H NMR (500 MHz, Chloroform-*d*) δ 5.88 (ddt, J = 17.0, 10.3, 6.6 Hz, 1H), 5.71 (d, J = 3.9 Hz, 1H), 5.31 (q, J = 4.2, 3.5 Hz, 3H), 5.28 (d, J = 3.8 Hz, 1H), 5.24 (d, J = 3.7 Hz, 1H), 5.19 (d, J = 3.8 Hz, 1H), 5.15 (dq, J = 17.3, 1.7 Hz, 1H), 5.09 (ddt, J = 10.3, 2.4, 1.3 Hz, 1H), 4.49 (t, J = 5.7 Hz, 3H), 4.42 (t, J = 6.4 Hz, 1H), 4.33 – 4.00 (m, 18H), 3.90 – 3.70 (m, 18H), 3.69 – 3.58 (m, 7H), 3.53 (dd, J = 11.0, 3.8 Hz, 1H), 3.32 (dd, J = 10.6, 3.6 Hz, 1H), 2.38 (q, J = 6.6 Hz, 2H). ^{13}C NMR (125 MHz, $CDCl_3$) δ 138.23, 119.25, 99.41, 98.91, 98.75, 98.71, 97.82, 78.92, 78.80, 78.70, 78.61, 77.47, 73.94, 73.49, 73.36, 73.13, 73.11, 73.05, 70.39, 70.20, 69.01, 68.85, 68.76, 68.62, 68.56, 63.11, 63.05, 63.01, 62.76, 62.60, 57.09, 53.52, 53.40, 53.37, 53.32, 35.64. HR-MS: Calculated for $C_{46}H_{85}N_7O_{29}$ $[M+3H]^{3+}$: 400.85367, found: 400.85307.

Heptasaccharide (44)

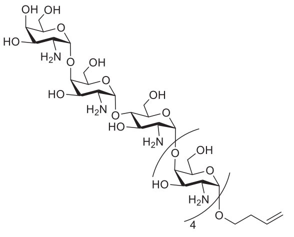

(53% yield, 50/1). The reaction was carried out according to the general procedure E. ^{1}H NMR (850 MHz, Deuterium Oxide) δ 5.85 (ddt, J = 17.1, 10.3, 6.7 Hz, 1H), 5.64 (d, J = 4.0 Hz, 1H), 5.23 (d, J = 3.9 Hz, 1H), 5.22 (d, J = 3.9 Hz, 1H), 5.20 (d, J = 3.9 Hz, 1H), 5.18 (d, J = 3.9 Hz, 1H), 5.15 (d, J = 3.8 Hz, 1H), 5.14 – 5.11 (m, 1H), 5.10 (d, J = 3.7 Hz, 1H), 5.06 (d, J = 10.3 Hz, 1H), 4.44 (p, J = 6.5, 6.1 Hz, 3H), 4.37 (t, J = 6.4 Hz, 1H), 4.25 (dt, J = 10.1, 3.2 Hz, 1H), 4.19 (dd, J = 9.3, 2.9 Hz, 3H), 4.15 (t, J = 3.6 Hz, 2H), 4.11 – 4.06 (m, 5H), 4.05 (t, J = 5.7 Hz, 1H), 4.03 – 3.97 (m, 5H), 3.82 – 3.72 (m, 15H), 3.70 – 3.67 (m, 2H), 3.62 – 3.52 (m, 4H), 3.51 – 3.45 (m, 3H), 3.41 (dd, J = 10.9, 3.8 Hz, 1H), 3.09 (dd, J = 10.6, 3.7 Hz, 1H), 2.36 (q, J = 7.2 Hz, 2H). ^{13}C NMR (214 MHz, D_2O) δ 135.63, 116.60, 97.66, 97.35, 97.12, 96.79, 95.47, 76.44, 76.41, 76.34, 76.25, 76.22, 75.43, 71.97, 71.48, 71.32, 71.04, 70.85, 70.84, 70.78, 70.74, 67.85, 67.51, 67.22, 66.98, 66.66, 66.62, 60.54, 60.52, 60.49, 60.06, 60.01, 59.96, 54.82, 50.94, 50.92, 50.89, 50.88, 50.76, 33.02. HR-MS: Calculated for $C_{46}H_{85}N_7O_{29}$ $[M+H]^{+}$: 1200.5470, found: 1200.5464. HR-MS: Calculated for $C_{46}H_{85}N_7O_{29}$ $[M+3H]^{3+}$: 400.85367, found: 400.85340.

Heptasaccharide (45)

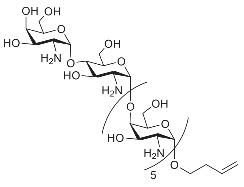

(59% yield, 25/1). The reaction was carried out according to the general procedure E. ^{1}H NMR (500 MHz, Deuterium Oxide) δ 5.91 (ddt, J = 17.1, 10.3, 6.6 Hz, 1H), 5.44 (d, J = 4.0 Hz, 1H), 5.17 (dq, J = 17.3, 1.7 Hz, 1H), 5.12 – 5.08 (m, 1H), 5.06 – 4.93 (m, 6H), 4.41 – 4.33 (m, 4H), 4.21 – 3.95 (m, 11H), 3.87 – 3.72 (m, 23H), 3.67 – 3.58 (m, 2H), 3.18 – 3.09 (m, 6H), 2.85 – 2.79 (m, 1H), 2.42 – 2.35 (m, 2H). ^{13}C NMR (214 MHz, D_2O) δ 135.78, 116.48, 100.11, 100.09, 99.97, 99.65, 98.56, 77.46, 77.45, 77.40, 77.34, 76.84, 76.57, 73.88, 72.00, 71.94, 71.92, 71.80, 71.79, 71.61, 71.09, 70.10, 69.83, 69.76, 69.65, 68.41, 68.39, 67.45, 61.20, 61.18, 60.67, 60.32, 60.23, 60.18, 60.14, 55.19, 51.41, 51.39, 51.32, 51.05, 50.75, 33.09. HR-MS: Calculated for $C_{46}H_{85}N_7O_{29}$ $[M+3H]^{3+}$: 400.85367, found: 400.85366.

Heptasaccharide (46)

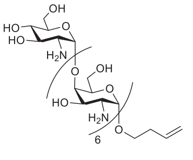

(85% yield, 43/1). The reaction was carried out according to the general procedure E. ^{1}H NMR (850 MHz, Deuterium Oxide) δ 5.90 – 5.82 (m, 1H), 5.28 – 5.23 (m, 4H), 5.23 – 5.14 (m, 3H), 5.14 – 5.11 (m, 1H), 5.06 (d, J = 10.3 Hz, 1H), 4.45 (q, J = 5.7, 4.9 Hz, 5H), 4.24 – 4.08 (m, 13H), 4.01 (t, J = 5.8 Hz, 1H), 3.84 – 3.72 (m, 15H), 3.59 (dt, J = 9.9, 6.3 Hz, 1H), 3.58 – 3.52 (m, 6H), 3.51 – 3.45 (m, 1H), 3.20 (dd, J = 10.7, 3.6 Hz, 1H), 2.36 (q, J = 7.2 Hz, 2H). ^{13}C NMR (214

MHz, D_2O) δ 135.63, 116.61, 96.85, 96.74, 95.37, 76.36, 76.30, 76.28, 76.21, 72.36, 70.78, 70.70, 70.65, 70.30, 69.15, 67.52, 66.71, 66.58, 66.50, 60.53, 60.09, 60.07, 60.06, 60.02, 59.95, 54.34, 50.84, 50.74, 33.02. HR-MS: Calculated for $C_{46}H_{85}N_7O_{29}$ $[M+2H]^{2+}$: 600.77686, found: 600.77660.

General procedure for acetylation of the oligosaccharides towards 47 - 52 (general procedure F)

To a solution of starting material in H_2O (1 ml) was added Ac_2O at 0 °C. Then $NaHCO_3$ was added to the solution until the pH is 8~9. The reaction was warmed to room temperature and stirred for 3h. Then the mixture was neutralized with AcOH and then concentrated *in vacuo*, which was purified by gel filtration (HW-40, 0.15M NH_4OAc in H_2O). The product containing fractions were pooled and lyophilized (4x) to yield the final products as a white solid.

Heptasaccharide (47)

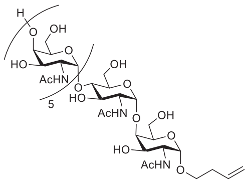

(90% yield). The reaction was carried out according to the general procedure F. ^{1}H NMR (850 MHz, Deuterium Oxide) δ 5.85 (ddt, *J* = 17.1, 10.3, 6.7 Hz, 1H), 5.42 (d, *J* = 3.9 Hz, 1H), 5.13 – 5.04 (m, 2H), 5.02 – 4.98 (m, 2H), 4.97 – 4.92 (m, 3H), 4.86 (d, *J* = 3.7 Hz, 1H), 4.40 – 4.35 (m, 3H), 4.33 (t, *J* = 6.6 Hz, 1H), 4.29 – 4.23 (m, 4H), 4.22 – 4.16 (m, 3H), 4.14 – 3.96 (m, 16H), 3.94 – 3.88 (m, 1H), 3.83 (dd, *J* = 12.4, 3.2 Hz, 1H), 3.77 – 3.72 (m, 2H), 3.71 – 3.53 (m, 15H), 2.38 – 2.29 (m, 2H), 2.07 – 1.97 (m, 21H). ^{13}C NMR (214 MHz, D_2O) δ 174.64, 174.52, 174.49, 174.48, 174.42, 174.34, 135.78, 116.39, 98.23, 98.15, 98.12, 98.07, 98.01, 97.98, 96.67, 77.18, 76.66, 76.33, 76.16, 76.07, 75.29, 72.24, 71.52, 71.22, 71.17, 71.13, 70.59, 70.53, 68.09, 67.10, 67.07, 66.94, 66.80, 66.60, 66.53, 66.40, 60.36, 60.21, 59.90, 59.47, 59.35, 54.33, 50.19, 50.03, 49.91, 32.96, 21.93, 21.82, 21.78, 21.77. HR-MS: Calculated for $C_{60}H_{99}N_7O_{36}$ $[M+2H]^{2+}$: 747.81384, found: 747.81376.

Heptasaccharide (48)

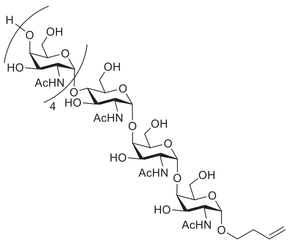

(91% yield, 7/1). The reaction was carried out according to the general procedure F. ^{1}H NMR (850 MHz, Deuterium Oxide) δ 5.85 (ddt, *J* = 17.1, 10.4, 6.7 Hz, 1H), 5.43 (d, *J* = 3.9 Hz, 1H), 5.13 – 5.04 (m, 2H), 5.00 (d, *J* = 3.8 Hz, 1H), 4.97 – 4.95 (m, 2H), 4.94 (d, *J* = 3.8 Hz, 1H), 4.93 (d, *J* = 3.8 Hz, 1H), 4.92 (d, *J* = 3.7 Hz, 1H), 4.40 – 4.36 (m, 3H), 4.33 (t, *J* = 6.5 Hz, 1H), 4.29 – 4.23 (m, 4H), 4.21 – 4.16 (m, 3H), 4.15 – 3.96 (m, 16H), 3.92 (dd, *J* = 10.8, 3.7 Hz, 1H), 3.84 (dd, *J* = 12.5, 3.2 Hz, 1H), 3.77 – 3.72 (m, 2H), 3.71 – 3.57 (m, 14H), 3.54 (dt, *J* = 10.4, 6.2 Hz, 1H), 2.39 – 2.27 (m, 2H), 2.05 – 1.98 (m, 21H). ^{13}C NMR (214 MHz, D_2O) δ 174.63, 174.56, 174.48, 174.42, 174.35, 135.78, 116.38, 98.24, 98.17, 98.10, 98.08, 97.98, 96.61, 76.68, 76.53, 76.36, 76.08, 75.27, 72.23, 71.47, 71.29, 71.22, 71.16, 70.60, 70.54, 68.11, 68.09, 67.10, 67.04, 66.96, 66.82, 66.61, 66.40, 60.35, 60.21, 59.92, 59.47, 59.38, 59.34, 54.28, 50.21, 50.08, 50.03, 49.94, 32.95, 21.93, 21.82, 21.77. HR-MS: Calculated for $C_{60}H_{99}N_7O_{36}$ $[M+2H]^{2+}$: 747.81384, found: 747.81423.

Heptasaccharide (49)

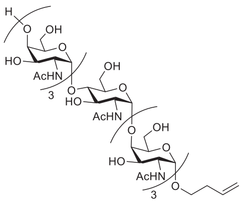

(91% yield, 32/1). The reaction was carried out according to the general procedure F. ^{1}H NMR (850 MHz, Deuterium Oxide) δ 5.85 (ddt, *J* = 17.0, 10.4, 6.6 Hz, 1H), 5.43 (d, *J* = 3.9 Hz, 1H), 5.13 – 5.08 (m, 1H), 5.07 – 5.04 (m, 1H), 5.00 (d, *J* = 3.8 Hz, 1H), 4.97 – 4.90 (m, 5H), 4.40 – 4.36 (m, 3H), 4.34 – 4.31 (m, 1H), 4.30 – 4.22 (m, 4H), 4.22 – 4.15 (m, 3H), 4.14 – 4.10 (m, 3H), 4.09 – 3.96 (m, 12H), 3.92 (dd, *J* = 10.8, 3.6 Hz, 1H), 3.84 (dd, *J* = 12.4, 3.2 Hz, 1H), 3.78 – 3.71 (m, 2H), 3.71 – 3.56 (m, 14H), 3.54 (dt, *J* = 10.4, 6.2 Hz, 1H), 2.37 – 2.28 (m, 2H), 2.05 – 1.99 (m, 21H). ^{13}C NMR (214 MHz, D_2O) δ 174.63, 174.56, 174.48, 174.46, 174.43, 174.41, 174.36, 135.79, 116.38, 98.19, 98.17, 98.12, 98.08, 98.03, 97.97, 96.61, 76.77, 76.68, 76.24, 76.02, 75.27, 72.23, 71.49, 71.28, 71.23, 71.16, 71.12, 70.59, 70.58, 68.11, 68.09, 67.11, 67.04, 66.98, 66.82, 66.53, 66.48, 66.38, 60.36, 60.35, 60.23, 59.93, 59.50, 59.32, 54.29, 50.23, 50.20, 50.03, 49.95, 32.95, 21.82, 21.81, 21.78. HR-MS: Calculated for $C_{60}H_{99}N_7O_{36}$ $[M+2H]^{2+}$: 747.81384, found: 747.81387.

Heptasaccharide (50)

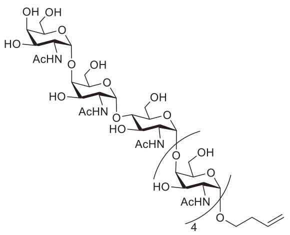

(90% yield, 32/1). The reaction was carried out according to the general procedure F. ^{1}H NMR (850 MHz, Deuterium Oxide) δ 5.85 (ddt, *J* = 17.0, 10.4, 6.6 Hz, 1H), 5.43 (d, *J* = 4.0 Hz, 1H), 5.13 – 5.08 (m, 1H), 5.08 – 5.04 (m, 1H), 5.01 (d, *J* = 3.8 Hz, 1H), 5.00 (d, *J* = 3.8 Hz, 1H), 4.95 – 4.90 (m, 4H), 4.40 – 4.36 (m, 3H), 4.33 (t, *J* = 6.6 Hz, 1H), 4.30 – 4.22 (m, 4H), 4.22 – 4.14 (m, 3H), 4.14 – 4.10 (m, 3H), 4.09 – 3.95 (m, 13H), 3.92 (dd, *J* = 10.9, 3.6 Hz, 1H), 3.84 (dd, *J* = 12.6, 3.2 Hz, 1H), 3.77 – 3.56 (m, 17H), 3.54 (dt, *J* = 10.4, 6.1 Hz, 1H), 2.36 – 2.29 (m, 2H), 2.06 – 2.02 (m, 15H), 2.02 – 2.00 (m, 6H). ^{13}C NMR (214 MHz, D_2O) δ 174.64, 174.55, 174.47, 174.46, 174.44, 174.42, 174.35, 135.79, 116.38, 98.28, 98.15, 98.08, 98.02, 97.98, 96.61, 76.87, 76.75, 76.74, 76.65, 76.09, 75.25, 72.33, 71.49, 71.29, 71.15, 71.10, 70.60, 70.58, 70.55, 68.09, 68.07, 67.03, 66.97, 66.90, 66.51, 66.40, 60.39, 60.37, 60.34, 59.91, 59.34, 59.30, 54.29, 50.20, 50.16, 50.07, 50.04, 49.97, 32.95, 21.94, 21.93, 21.84, 21.83, 21.82, 21.81, 21.79, 21.78, 21.77. HR-MS: Calculated for $C_{60}H_{99}N_7O_{36}$ $[M+2H]^{2+}$: 747.81384, found: 747.81383.

Heptasaccharide (51)

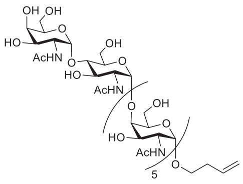

(89% yield, 13/1). The reaction was carried out according to the general procedure F. ^{1}H NMR (850 MHz, Deuterium Oxide) δ 5.76 (ddt, *J* = 17.1, 10.4, 6.7 Hz, 1H), 5.27 (d, *J* = 4.0 Hz, 1H), 5.04 – 4.99 (m, 1H), 4.98 – 4.95 (m, 1H), 4.93 – 4.89 (m, 3H), 4.86 – 4.82 (m, 3H), 4.31 – 4.26 (m, 4H), 4.22 – 4.13 (m, 4H), 4.13 – 4.05 (m, 3H), 4.05 – 3.96 (m, 8H), 3.93 – 3.87 (m, 6H), 3.84 – 3.80 (m, 1H), 3.79 – 3.76 (m, 2H), 3.67 – 3.47 (m, 16H), 3.45 (dt, *J* = 10.4, 6.2 Hz, 1H), 2.29 – 2.19 (m, 2H), 1.97 – 1.93 (m, 15H), 1.91 (s, 3H), 1.90 (s, 3H). ^{13}C NMR (214 MHz, D_2O) δ 174.56, 174.51, 174.49, 174.46, 174.44, 174.35, 135.79, 116.38, 98.16, 98.12, 98.09, 98.07, 98.00, 96.61, 76.74, 76.61, 76.17, 76.08, 75.54, 71.54, 71.49, 71.15, 70.61, 68.34, 68.33, 67.48, 67.03, 66.97, 66.52, 66.39, 61.05, 60.37, 59.93, 59.33, 54.28, 50.20, 50.16, 50.04, 49.84, 32.95, 21.96, 21.83, 21.78, 21.77. HR-MS: Calculated for $C_{60}H_{99}N_7O_{36}$ $[M+2H]^{2+}$: 747.81384, found: 747.81345.

Heptasaccharide (52)

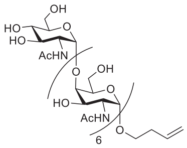

(88% yield, 12/1). The reaction was carried out according to the general procedure F. ^{1}H NMR (850 MHz, Deuterium Oxide) δ 5.85 (ddt, *J* = 17.1, 10.3, 6.7 Hz, 1H), 5.13 – 5.08 (m, 1H), 5.07 – 5.04 (m, 1H), 5.01 – 4.98 (m, 4H), 4.95 – 4.92 (m, 3H), 4.40 – 4.35 (m, 5H), 4.28 – 4.22 (m, 5H), 4.18 (dd, *J* = 11.2, 3.7 Hz, 1H), 4.14 – 4.04 (m, 10H), 4.01 – 3.95 (m, 3H), 3.89 (dd, *J* = 10.9, 3.6 Hz, 1H), 3.83 – 3.77 (m, 2H), 3.76 – 3.71 (m, 1H), 3.70 – 3.49 (m, 15H), 2.37 – 2.28 (m, 2H), 2.05 – 2.02 (m, 17H), 1.99 (s, 7H). ^{13}C NMR (214 MHz, D_2O) δ 174.55, 174.48, 174.45, 174.41, 174.23, 135.79, 116.39, 98.23, 98.11, 98.08, 96.61, 76.76, 76.74, 76.63, 76.61, 76.15, 76.08, 71.87, 71.49, 71.15, 71.13, 70.38, 69.47, 67.03, 66.97, 66.52, 66.40, 60.37, 59.75, 59.34, 53.96, 50.20, 50.17, 50.11, 50.03, 32.95, 21.84, 21.83, 21.78, 21.77, 21.73, 21.72. HR-MS: Calculated for $C_{60}H_{99}N_7O_{36}$ $[M+2H]^{2+}$: 747.81384, found: 747.81386.

Removal of TFA group was first attempted on trisaccharide **S6** in 1 M NaOH solution at 40 °C, giving S45 in 92% yield (Table S1, entry 1). However, the TFA groups in heptamer **S44** could not be cleaved even with strong basic conditions and high temperature (4M KOH, 80 °C, entry 2). Also attempts to remove the TFA groups with the assistance of microwave failed (entry 3). Considering the possible solubility problem of the intermediates, ammonia in methanol and 1,4-dioxane was applied, but only afforded a mixture of incompletely deprotected products (entries 4 and 5). Another attempt by the combination of KOH and H_2O_2 in 37 °C led to a mixture (entry 6). When the reaction was proceed at 100 °C with ammonium salt and ethylenediamine as reagents, which could be used for the deacylation of unactivated amides to generate amines[4], still failed to give the target **S46** (entry 7).

Table S1. Attempts of N-TFA removal of heptasaccharide **S44**

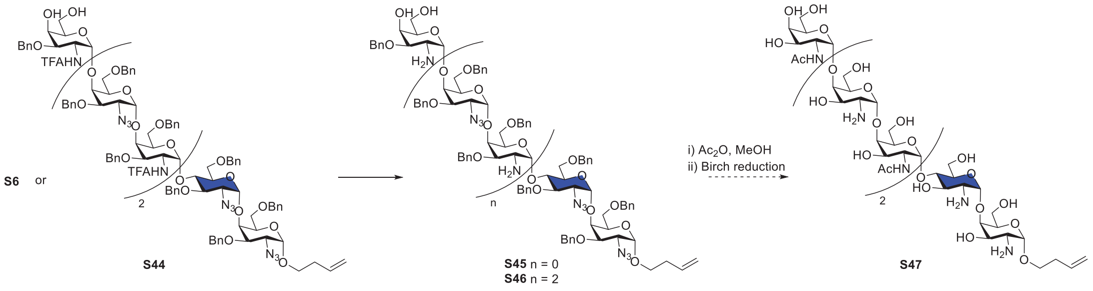

Entry	RN-TFA	Reagents and conditions	Yield
1	**S6**	1M NaOH, THF, MeOH, 40 °C, 24h	92% (**S45**)
2	**S44**	4M KOH, THF, MeOH, 80 °C, 5d	mixture
3	**S44**	4M KOH, THF, MeOH, 60 °C, microwave, 6h	mixture
4	**S44**	NH_3 in MeOH, 65 °C, 7d	mixture
5	**S44**	$NH_3.H_2O$, 1,4-dioxane, 60 °C, 4d	mixture
6	**S44**	KOH, H_2O_2, H_2O, THF, 37 °C, 2d	mixture
7	**S44**	$H_2N(CH_2)_2NH_2$, NH_4Br, 100 °C, 24h	mixture

General procedure for heptasaccharides 53 - 58 (general procedure G)

HF/pyridine (16 eq) solution was added to a solution of starting material in THF at 0 °C. The reaction was warmed to room temperature and stirred until TLC-analysis indicated full consumption of the starting material (± 1h). Then the mixture was diluted with DCM and washed with saturated $NaHCO_3$ and brine, dried with anhydrous $MgSO_4$, filtered and concentrated *in vacuo*. The residue was purified by silica gel column chromatography. The residue was dissolved in THF/H_2O/*tert*-BuOH (2 ml/2 ml/0.8 ml) before a catalytic amount of $Pd(OH)_2$/C was added. The reaction mixture was stirred for 3 days under a H_2 atmosphere, filtered and concentrated *in vacuo*. Then Boc_2O and Et_3N were added to the solution of the residue in methanol at 0 °C. The reaction was slowly warmed to room temperature and stirred for overnight. The reaction was concentrated *in vacuo* and co-evaporated with toluene for 3 times. The residue was dissolved in $NH_3 \cdot H_2O$ (2 ml), which was warmed to 60 °C and stirred for overnight. The solution was concentrated *in vacuo* and then dissolved in H_2O. Ac_2O was added at 0 °C and $NaHCO_3$ was added to the solution until the pH is 8~9. The reaction was warmed to room temperature and stirred for 3h. Then the mixture was neutralized with AcOH and then concentrated *in vacuo*. The residue was dissolved in 30% TFA in H_2O, and allowed to stirred at rt for overnight. The solution was concentrated *in vacuo*, which was purified by gel filtration (HW-40, 0.15M NH_4OAc in H_2O). The product containing fractions were pooled and lyophilized (4x) to yield the final products as a white solid.

Heptasaccharide (53)

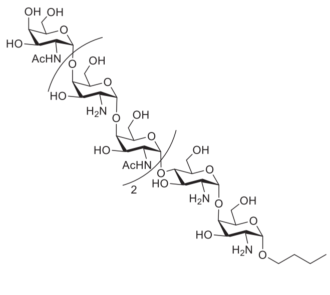

(31% yield). The reaction was carried out according to the general procedure G. ^{1}H NMR (500 MHz, Deuterium Oxide) δ 5.44 (d, *J* = 3.9 Hz, 1H), 5.28 (d, *J* = 3.9 Hz, 1H), 5.20 (d, *J* = 3.8 Hz, 1H), 5.13 (d, *J* = 3.8 Hz, 1H), 5.05 (d, *J* = 3.6 Hz, 1H), 5.00 (d, *J* = 3.8 Hz, 1H), 4.96 (d, *J* = 3.9 Hz, 1H), 4.42 (q, *J* = 4.4, 2.8 Hz, 3H), 4.36 (t, *J* = 6.4 Hz, 1H), 4.31 – 4.22 (m, 4H), 4.21 – 3.91 (m, 17H), 3.84 – 3.58 (m, 18H), 3.58 – 3.43 (m, 5H), 3.01 (d, *J* = 10.6 Hz, 1H), 2.09 – 1.98 (m, 9H), 1.64 – 1.53 (m, 2H), 1.41 – 1.28 (m, 2H), 0.87 (t, *J* = 7.4 Hz, 3H). ^{13}C NMR (214 MHz, D_2O) δ 175.70, 175.48, 175.39, 99.27, 99.08, 98.38, 96.38, 96.29, 77.50, 77.13, 76.71, 75.78, 72.70, 72.58, 72.39, 71.84, 71.21, 70.09, 69.30, 69.04, 68.07, 67.75, 67.64, 67.51, 61.59, 61.49, 61.43, 61.14, 61.07, 60.61, 60.39, 56.02, 52.07, 51.99, 51.79, 51.02, 50.98, 50.81, 31.62, 22.93, 22.79, 22.74, 19.66, 13.95. HR-MS: Calculated for $C_{52}H_{93}N_7O_{32}$ $[M+2H]^{2+}$: 664.80053, found: 664.80019.

Heptasaccharide (54)

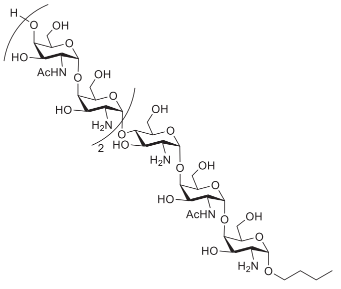

(25% yield). The reaction was carried out according to the general procedure G. ^{1}H NMR (850 MHz, Deuterium Oxide) δ 5.46 (d, *J* = 4.0 Hz, 1H), 5.03 (d, *J* = 3.7 Hz, 1H), 4.95 – 4.92 (m, 4H), 4.90 (d, *J* = 3.8 Hz, 1H), 4.40 – 4.33 (m, 4H), 4.30 (t, *J* = 6.4 Hz, 1H), 4.26 – 4.20 (m, 3H), 4.17 – 4.11 (m, 4H), 4.10 (d, *J* = 2.8 Hz, 1H), 4.04 – 4.00 (m, 5H), 3.99 – 3.95 (m, 5H), 3.93 – 3.90 (m, 2H), 3.89 – 3.86 (m, 2H), 3.82 – 3.62 (m, 25H), 3.48 (dt, *J* = 9.8, 6.4 Hz, 1H), 2.04 – 2.01 (m, 9H), 1.58 – 1.52 (m, 2H), 1.37 – 1.32 (m, 2H), 0.86 (t, *J* = 7.4 Hz, 3H). ^{13}C NMR (214 MHz, D_2O) δ 175.45, 175.41, 100.99, 100.94, 100.59, 99.30, 99.22, 99.15, 78.43, 77.93, 77.82, 76.95, 74.73, 73.37, 72.84, 72.64, 72.24, 72.17, 72.04, 71.92, 70.62, 70.42, 69.24, 69.14, 68.09, 67.76, 67.73, 61.56, 61.45, 61.36, 60.98, 60.83, 56.17, 52.33, 52.00, 51.91, 51.32, 51.17, 51.06, 31.66, 22.79, 22.74, 19.72, 13.99. HR-MS: Calculated for $C_{52}H_{93}N_7O_{32}$ $[M+2H]^{2+}$: 664.80053, found: 664.80024.

Heptasaccharide (55)

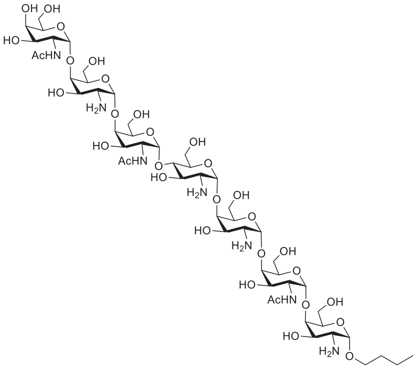

(24% yield). The reaction was carried out according to the general procedure G. ^{1}H NMR (850 MHz, Deuterium Oxide) δ 5.43 (d, *J* = 3.9 Hz, 1H), 5.21 – 5.14 (m, 2H), 5.09 (d, *J* = 3.8 Hz, 1H), 5.06 – 5.02 (m, 1H), 4.95 (d, *J* = 4.0 Hz, 1H), 4.93 (d, *J* = 3.9 Hz, 1H), 4.40 (dt, *J* = 11.4, 5.6 Hz, 3H), 4.33 (t, *J* = 6.4 Hz, 1H), 4.28 – 4.20 (m, 4H), 4.20 – 4.11 (m, 5H), 4.10 – 3.95 (m, 13H), 3.95 – 3.87 (m, 3H), 3.84 – 3.59 (m, 21H), 3.50 (dt, *J* = 9.7, 6.4 Hz, 1H), 3.47 – 3.32 (m, 3H), 2.95 (s, 1H), 2.05 – 1.99 (m, 11H), 1.60 – 1.52 (m, 2H), 1.37 – 1.29 (m, 2H), 0.86 (t, *J* = 7.4 Hz, 3H). ^{13}C NMR (214 MHz, D_2O) δ 174.64, 174.45, 174.39, 98.26, 98.13, 97.35, 76.72, 76.63, 76.34, 74.96, 71.74, 71.59, 71.06, 70.88, 70.81, 70.63, 68.23, 68.07, 67.08, 66.88, 66.55, 60.57, 60.47, 60.23, 60.16, 59.91, 59.82, 59.69, 51.16, 51.08, 50.89, 50.04, 49.89, 30.62, 18.67, 12.96. HR-MS: Calculated for $C_{52}H_{93}N_7O_{32}$ $[M+2H]^{2+}$: 664.80053, found: 664.80000.

Heptasaccharide (56)

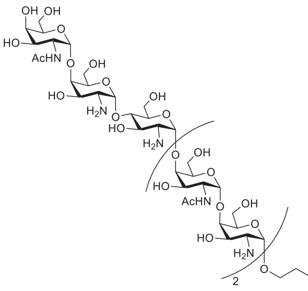

(18% yield). The reaction was carried out according to the general procedure G. ^{1}H NMR (500 MHz, Deuterium Oxide) δ 5.53 (d, *J* = 4.0 Hz, 1H), 5.08 (d, *J* = 3.9 Hz, 1H), 5.03 – 4.93 (m, 5H), 4.45 – 4.37 (m, 3H), 4.34 (t, *J* = 6.4 Hz, 1H), 4.32 – 4.24 (m, 2H), 4.22 – 4.14 (m, 4H), 4.11 – 3.65 (m, 34H), 3.53 (dt, *J* = 9.8, 6.3 Hz, 1H), 3.26 (dd, *J* = 11.1, 3.9 Hz, 1H), 3.21 – 3.14 (m, 2H), 2.85 (dd, *J* = 10.5, 3.6 Hz, 1H), 2.11 – 2.02 (m, 9H), 1.64 – 1.56 (m, 2H), 1.43 – 1.34 (m, 2H), 0.90 (t, *J* = 7.4 Hz, 3H). ^{13}C NMR (214 MHz, D_2O) δ 174.44, 174.41, 100.08, 99.72, 98.54, 98.24, 98.23, 98.12, 77.56, 77.09, 76.92, 76.55, 75.95, 73.77, 72.46, 71.75, 71.67, 71.31, 71.23, 71.07, 70.87, 70.86, 69.89, 69.44, 69.38, 68.39, 68.23, 68.13, 68.11, 67.00, 66.89, 66.73, 60.53, 60.52, 60.00, 59.84, 59.68, 55.18, 51.27, 51.07, 50.93, 50.33, 50.17, 50.09, 30.67, 21.80, 21.75, 19.95, 18.73, 13.00. HR-MS: Calculated for $C_{52}H_{93}N_7O_{32}$ $[M+3H]^{3+}$: 443.53611, found: 443.53617.

Heptasaccharide (57)

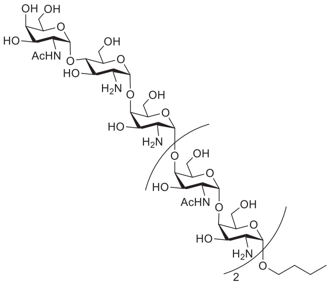

(30% yield). The reaction was carried out according to the general procedure G. ^{1}H NMR (850 MHz, Deuterium Oxide) δ 5.26 (d, *J* = 3.9 Hz, 1H), 5.20 – 5.14 (m, 2H), 5.06 (s, 1H), 5.03 (d, *J* = 3.8 Hz, 1H), 4.89 (d, *J* = 3.9 Hz, 1H), 4.84 (d, *J* = 3.9 Hz, 1H), 4.36 – 4.29 (m, 4H), 4.26 – 4.21 (m, 1H), 4.19 – 4.14 (m, 2H), 4.13 – 4.08 (m, 5H), 4.05 (dd, *J* = 11.2, 3.9 Hz, 2H), 4.01 – 3.95 (m, 6H), 3.94 (t, *J* = 6.3 Hz, 1H), 3.92 – 3.88 (m, 2H), 3.87 (d, *J* = 3.2 Hz, 1H), 3.79 (dd, *J* = 11.2, 3.2 Hz, 1H), 3.74 (dd, *J* = 12.3, 4.0 Hz, 1H), 3.70 – 3.59 (m, 13H), 3.59 – 3.52 (m, 4H), 3.50 (dd, *J* = 10.9, 6.3 Hz, 1H), 3.47 – 3.38 (m, 4H), 1.96 – 1.89 (m, 9H), 1.51 – 1.43 (m, 2H), 1.28 – 1.22 (m, 2H), 0.77 (t, *J* = 7.4 Hz, 3H). ^{13}C NMR (214 MHz, D_2O) δ 174.47, 98.10, 96.60, 95.30, 76.73, 76.51, 76.16, 75.97, 75.70, 75.13, 71.60, 71.56, 70.66, 70.54, 70.32, 70.26, 68.23, 67.21, 66.56, 66.46, 61.09, 60.16, 60.04, 59.90, 59.37, 54.83, 50.99, 50.87, 50.00, 49.95, 49.88, 30.61, 23.13, 23.12, 21.91, 21.74, 18.66, 12.95. HR-MS: Calculated for $C_{52}H_{93}N_7O_{32}$ $[M+2H]^{2+}$: 664.80053, found: 664.80042.

Heptasaccharide (58)

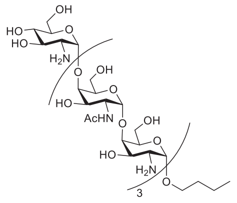

(18% yield). The reaction was carried out according to the general procedure G. ^{1}H NMR (850 MHz, Deuterium Oxide) δ 5.29 – 5.22 (m, 2H), 5.20 (d, *J* = 4.0 Hz, 1H), 5.12 (d, *J* = 3.8 Hz, 1H), 4.99 (d, *J* = 4.1 Hz, 2H), 4.94 (d, *J* = 3.9 Hz, 1H), 4.44 – 4.38 (m, 5H), 4.29 – 4.23 (m, 3H), 4.23 – 4.17 (m, 3H), 4.15 – 4.03 (m, 10H), 4.02 – 3.96 (m, 3H), 3.86 (t, *J* = 9.9 Hz, 1H), 3.81 – 3.56 (m, 19H), 3.54 – 3.46 (m, 5H), 3.20 (d, *J* = 10.3 Hz, 1H), 2.05 – 1.99 (m, 11H), 1.60 – 1.52 (m, 2H), 1.36 – 1.29 (m, 2H), 0.86 (t, *J* = 7.5, 3.2 Hz, 3H). ^{13}C NMR (214 MHz, D_2O) δ 174.49, 174.48, 174.43, 98.11, 95.39, 95.13, 77.01, 76.60, 76.55, 76.20, 75.77, 72.39, 71.58, 71.02, 70.36, 69.04, 68.24, 66.85, 66.58, 66.48, 61.04, 60.44, 60.19, 60.08, 59.65, 59.41, 54.41, 51.01, 50.89, 50.02, 49.95, 30.62, 21.75, 18.67, 12.96. HR-MS: Calculated for $C_{52}H_{93}N_7O_{32}$ $[M+2H]^{2+}$: 664.80053, found: 664.80022.

References

[1] S. van der Vorm, H. S. Overkleeft, G. A. van der Marel, J. D. C. Codée, *J. Org. Chem.* **2017**, *82*, 4793-4811.

[2] Y. Zhang, M. Gómez-Redondo, G. Jiménez-Osés, A. Arda, H. S. Overkleeft, G. A. van der Marel, J. Jiménez-Barbero, J. D. C. Codée, *Angew. Chem. Int. Ed.* **2020**, *59*, 12746-12750.

[3] L. Wang, Y. Zhang, H. S. Overkleeft, G. A. van der Marel, J. D. C. Codee, *J. Org. Chem.* **2020**, *85*, 15872-15884.

[4] Y. Shimizu, H. Morimoto, M. Zhang, T. Ohshima, *Angew. Chem. Int. Ed.* **2012**, *51*, 8564-8567.

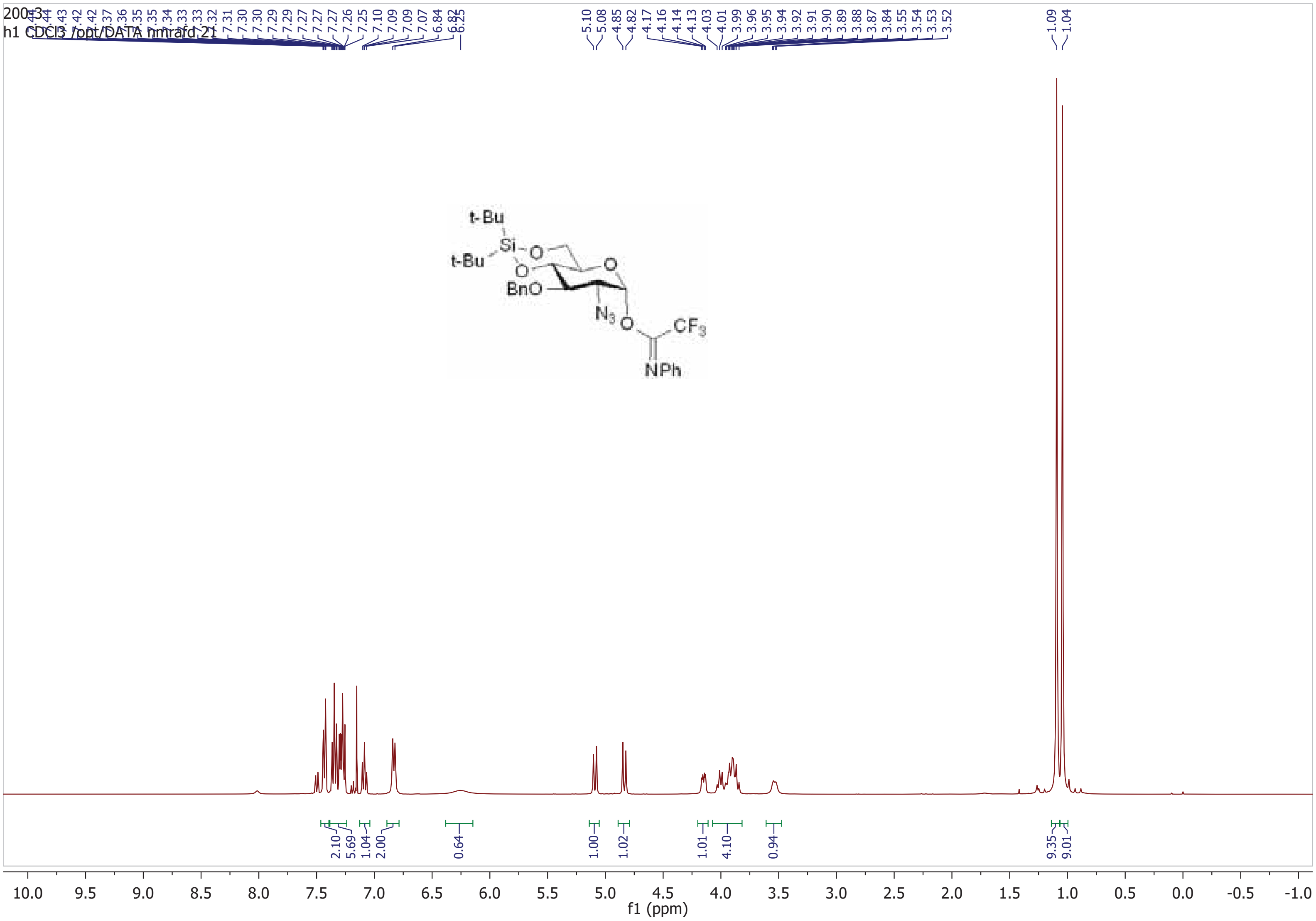

200-3
h1 CDCl3 /opt/DATA nmrafd.21
7.43 7.42 7.42 7.37 7.36 7.35 7.35 7.34 7.33 7.33 7.32 7.31 7.30 7.30 7.29 7.29 7.27 7.27 7.27 7.26 7.25 7.10 7.09 7.09 7.07 6.84 6.82 6.25
5.10 5.08 4.85 4.82 4.17 4.16 4.14 4.13 4.03 4.01 3.99 3.96 3.95 3.94 3.92 3.91 3.90 3.89 3.88 3.87 3.84 3.55 3.54 3.53 3.52
1.09 1.04
t-Bu
Si
t-Bu
O
O
O
BnO
N_3
O
CF_3
NPh
2.10 5.69 1.04 2.00 0.64 1.00 1.02 1.01 4.10 0.94 9.35 9.01
10.0 9.5 9.0 8.5 8.0 7.5 7.0 6.5 6.0 5.5 5.0 4.5 4.0 3.5 3.0 2.5 2.0 1.5 1.0 0.5 0.0 -0.5 -1.0
f1 (ppm)

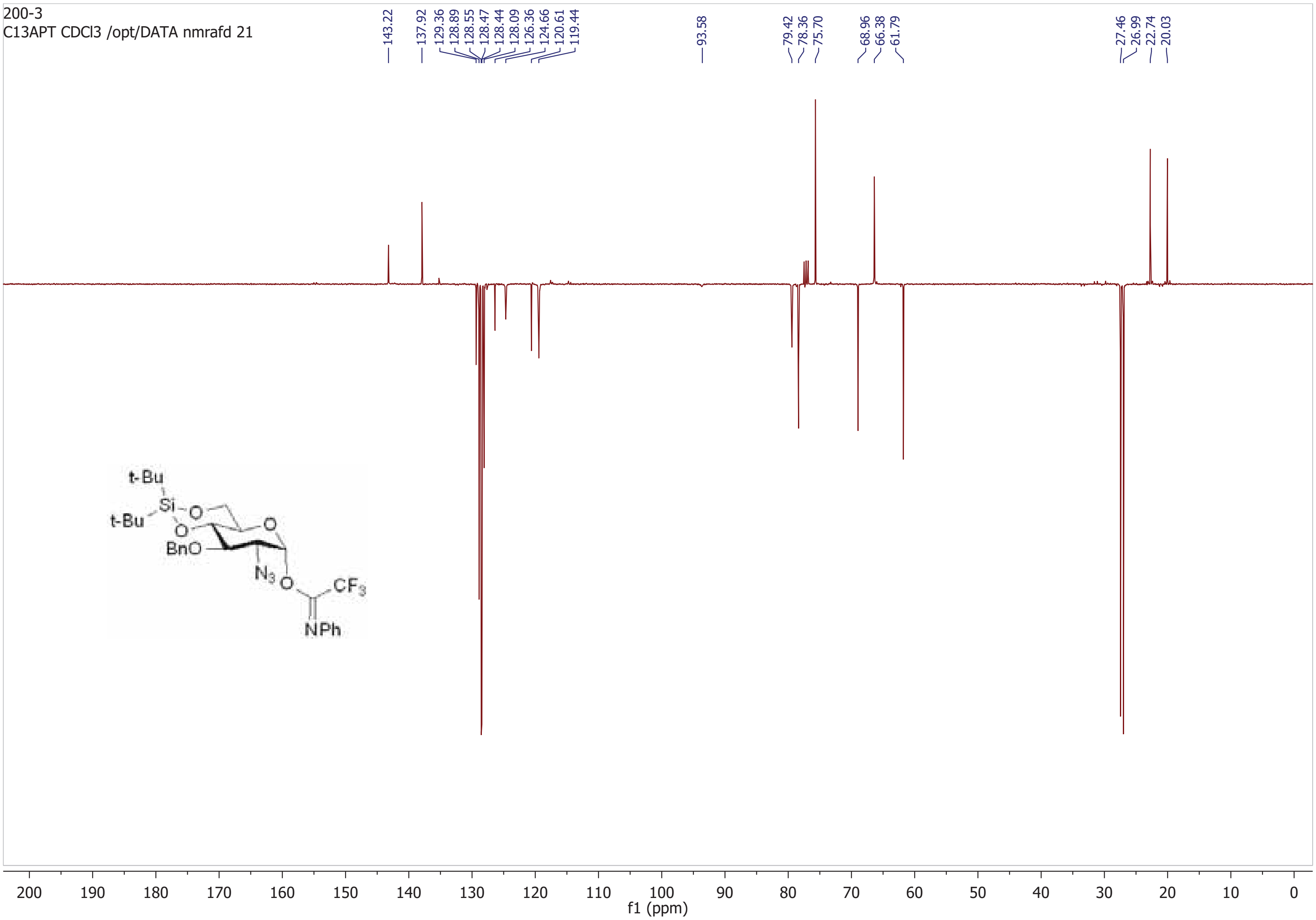
200-3
C13APT CDCl3 /opt/DATA nmrafd 21
143.22
137.92
129.36
128.89
128.55
128.47
128.44
128.09
126.36
124.66
120.61
119.44
93.58
79.42
78.36
75.70
68.96
66.38
61.79
27.46
26.99
22.74
20.03
t-Bu
t-Bu
Si
O
O
O
BnO
N3
O
CF3
NPh
200
190
180
170
160
150
140
130
120
110
100
90
80
70
60
50
40
30
20
10
0
f1 (ppm)

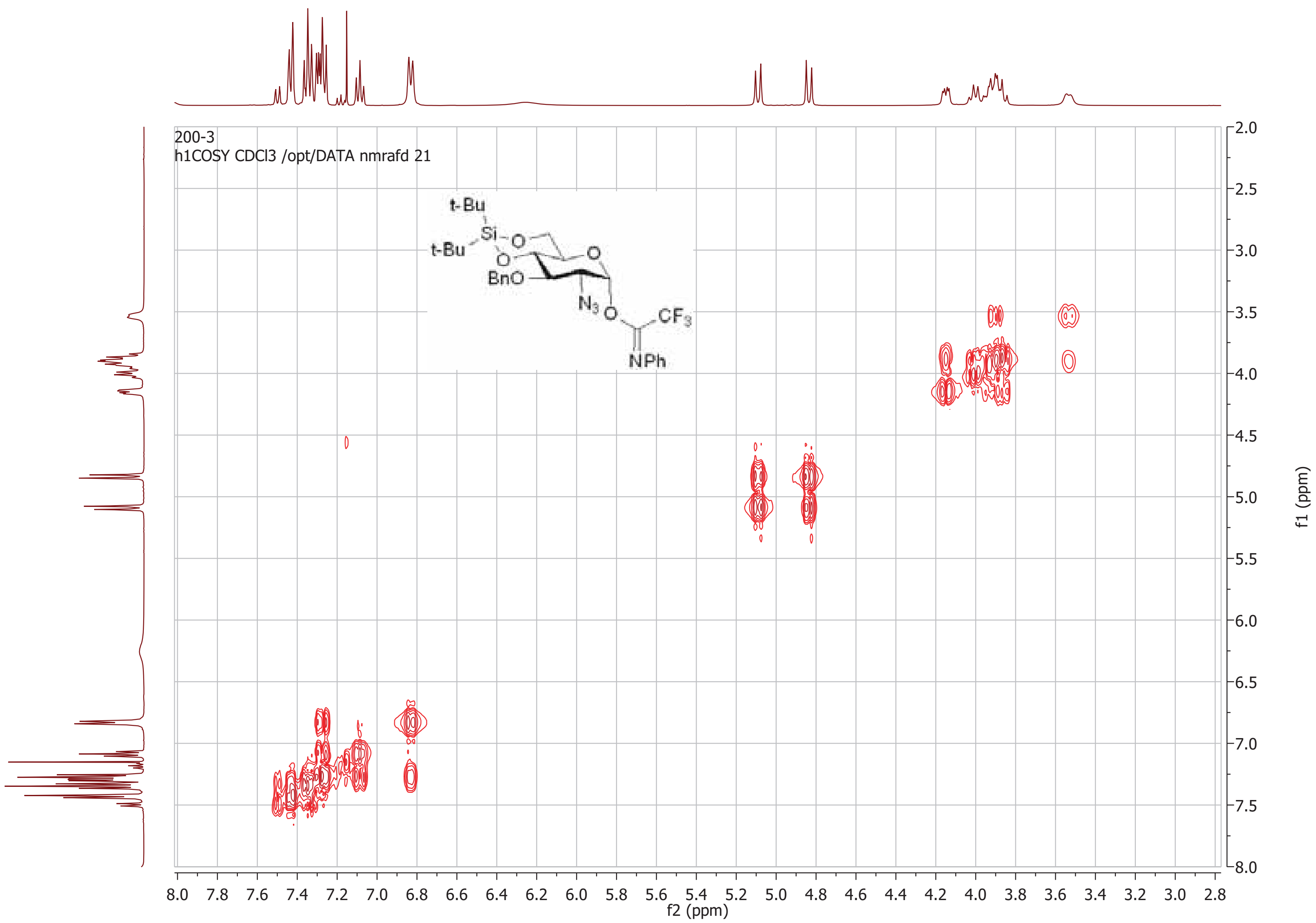
200-3
h1COSY CDCl3 /opt/DATA nmrafd 21
t-Bu
Si
O
t-Bu
O
BnO
O
N3
O
CF3
NPh
f2 (ppm)
f1 (ppm)

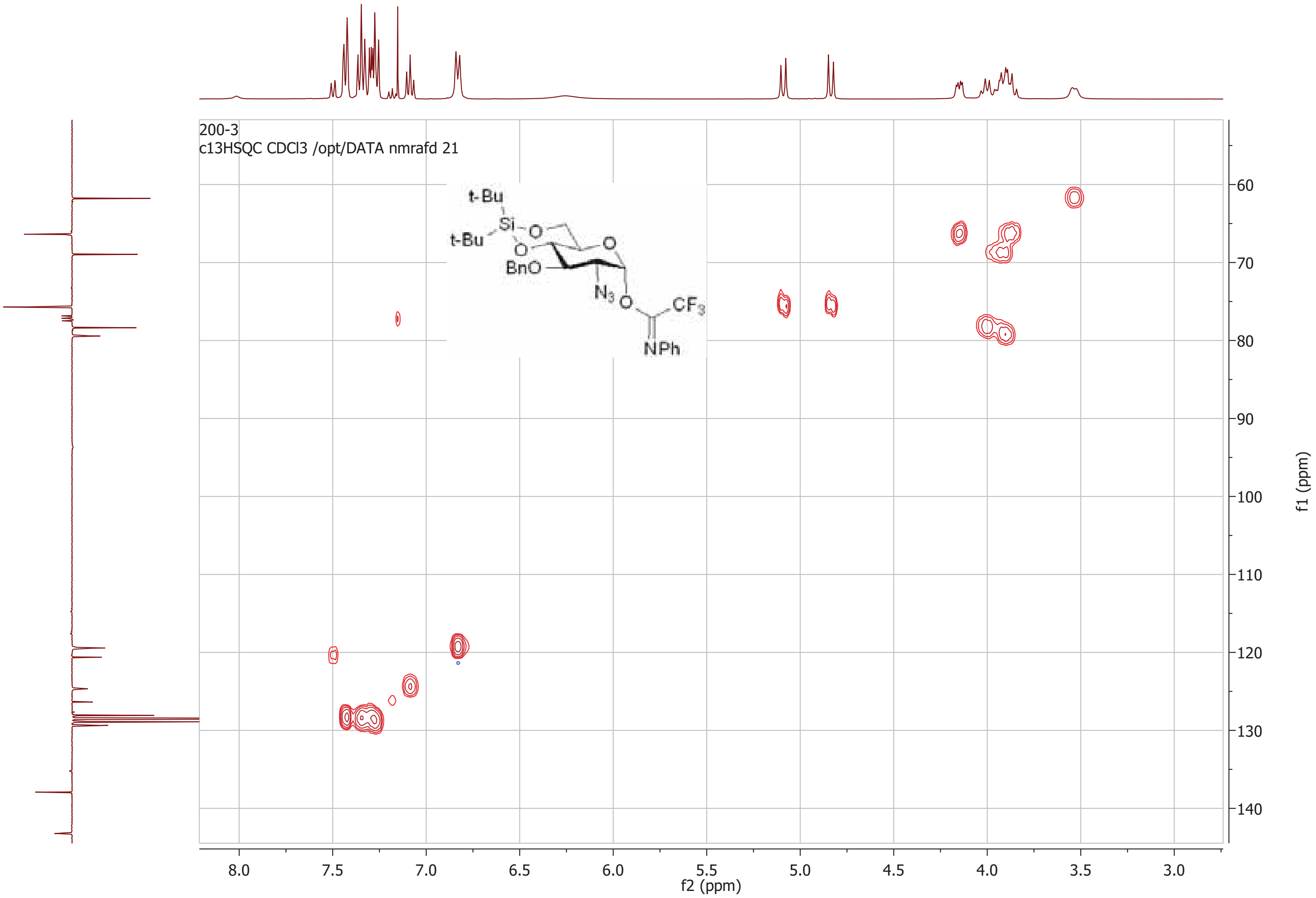
200-3
c13HSQC CDCl3 /opt/DATA nmrafd 21
t-Bu
Si
O
t-Bu
O
BnO
O
N3
O
CF3
NPh
8.0
7.5
7.0
6.5
6.0
5.5
5.0
4.5
4.0
3.5
3.0
f2 (ppm)
60
70
80
90
100
110
120
130
140
f1 (ppm)

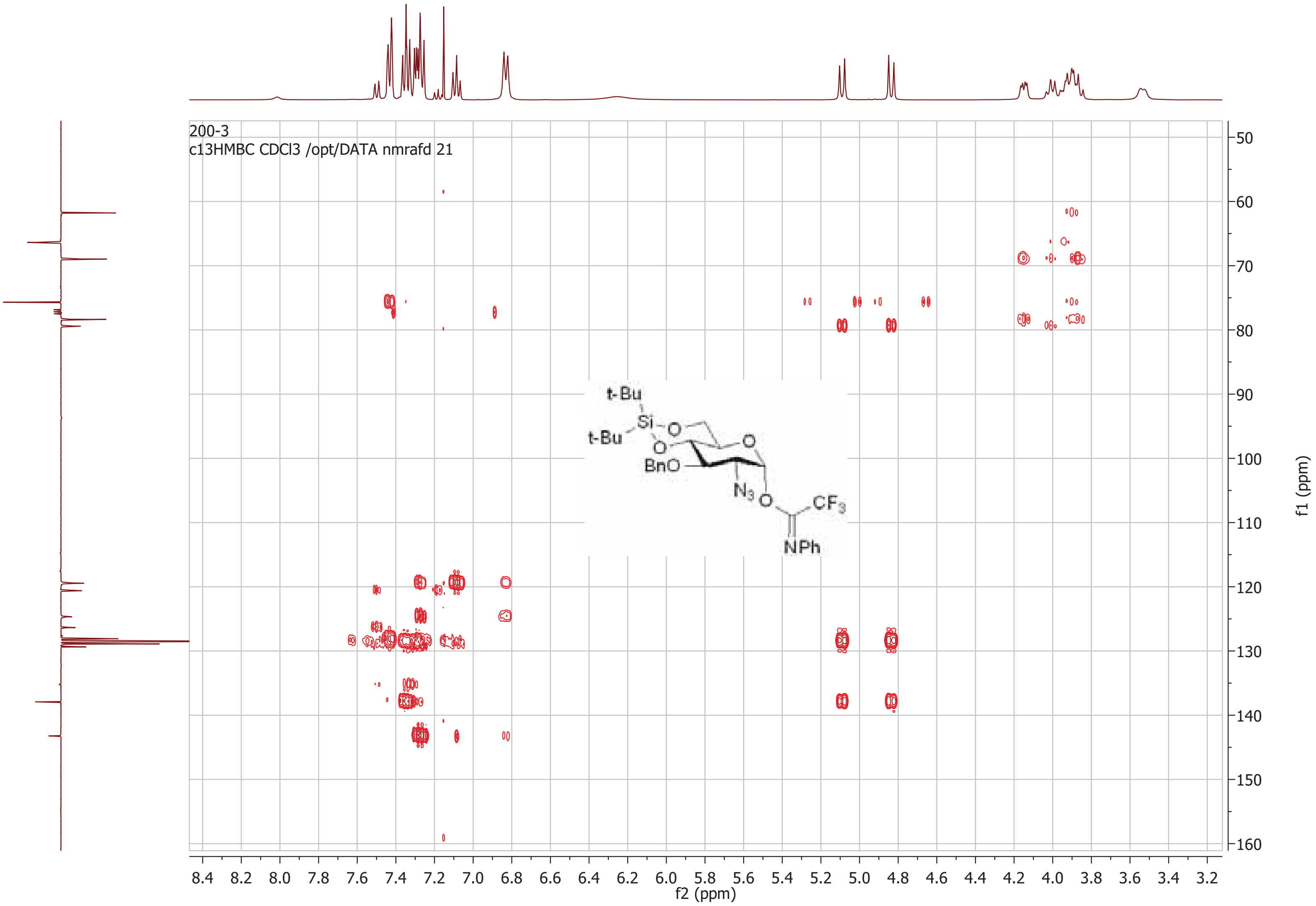

200-3
c13HMBC CDCl3 /opt/DATA nmrafd 21
t-Bu
t-Bu
Si
O
O
BnO
O
N3
O
CF3
NPh
f2 (ppm)
f1 (ppm)

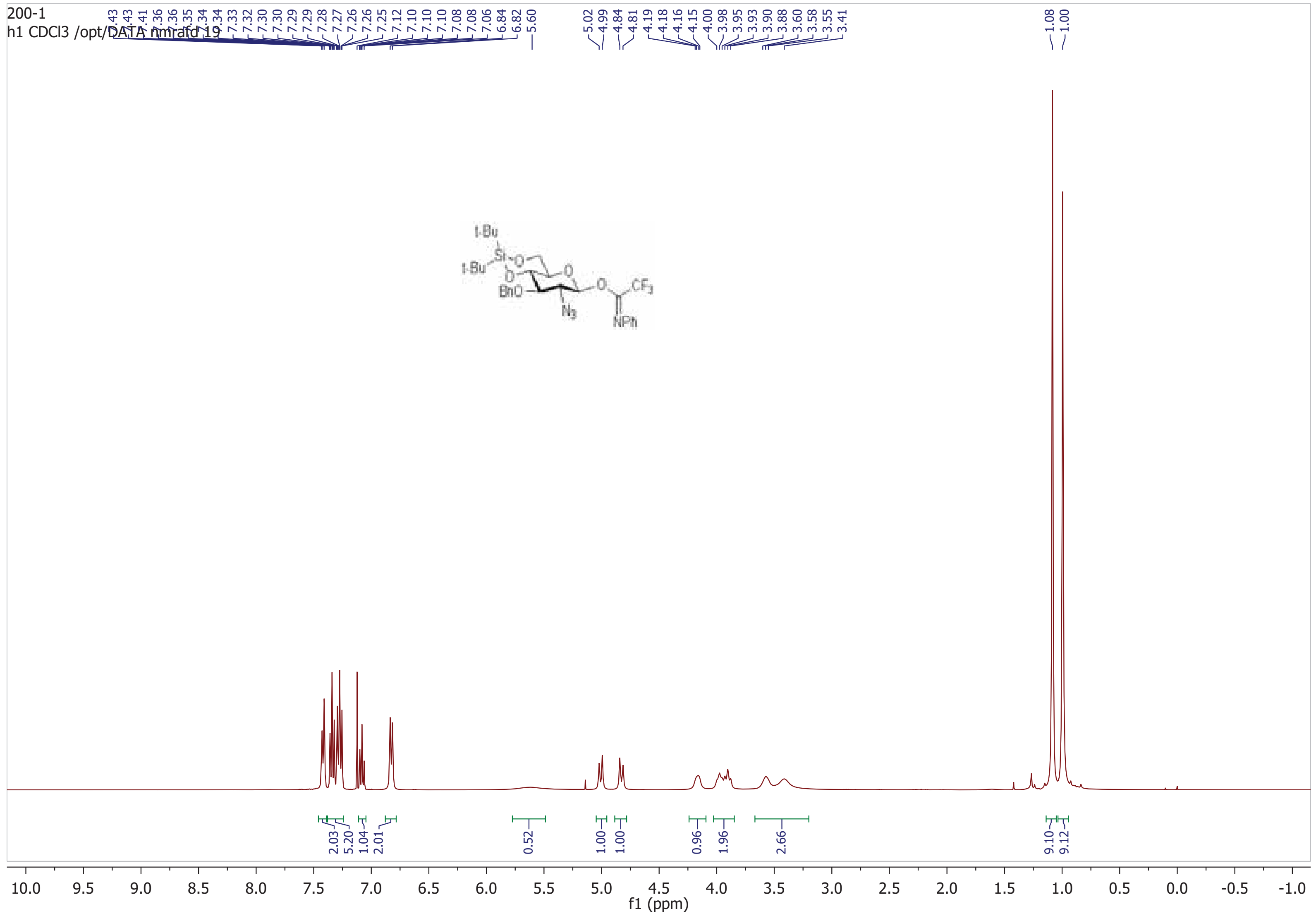
200-1
h1 CDCl3 /opt/DATA nmrafd 19
7.43 7.43 7.41 7.36 7.36 7.35 7.34 7.34 7.33 7.32 7.30 7.30 7.29 7.29 7.28 7.27 7.26 7.26 7.25 7.12 7.10 7.10 7.10 7.08 7.08 7.06 6.84 6.82 5.60
5.02 4.99 4.84 4.81 4.19 4.18 4.16 4.15 4.00 3.98 3.95 3.93 3.90 3.88 3.60 3.58 3.55 3.41
1.08 1.00
t-Bu
Si
O
t-Bu
BnO
N3
CF3
NPh
2.03 5.20 1.04 2.01 0.52 1.00 1.00 0.96 1.96 2.66 9.10 9.12
10.0 9.5 9.0 8.5 8.0 7.5 7.0 6.5 6.0 5.5 5.0 4.5 4.0 3.5 3.0 2.5 2.0 1.5 1.0 0.5 0.0 -0.5 -1.0
f1 (ppm)

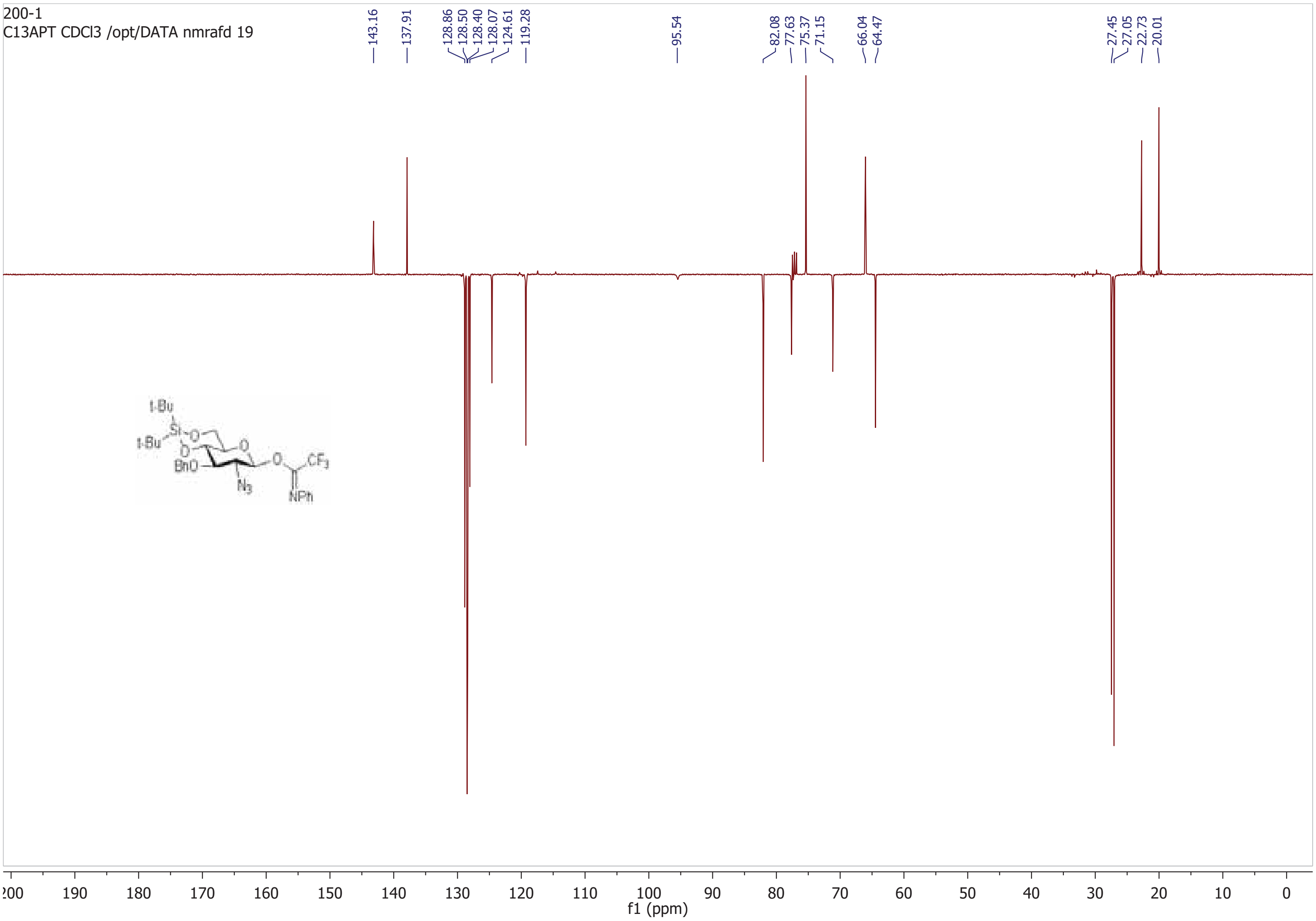

200-1
C13APT CDCl3 /opt/DATA nmrafd 19
143.16
137.91
128.86
128.50
128.40
128.07
124.61
119.28
95.54
82.08
77.63
75.37
71.15
66.04
64.47
27.45
27.05
22.73
20.01
t-Bu
Si
t-Bu
O
O
BnO
N3
O
O
CF3
NPh
200 190 180 170 160 150 140 130 120 110 100 90 80 70 60 50 40 30 20 10 0
f1 (ppm)

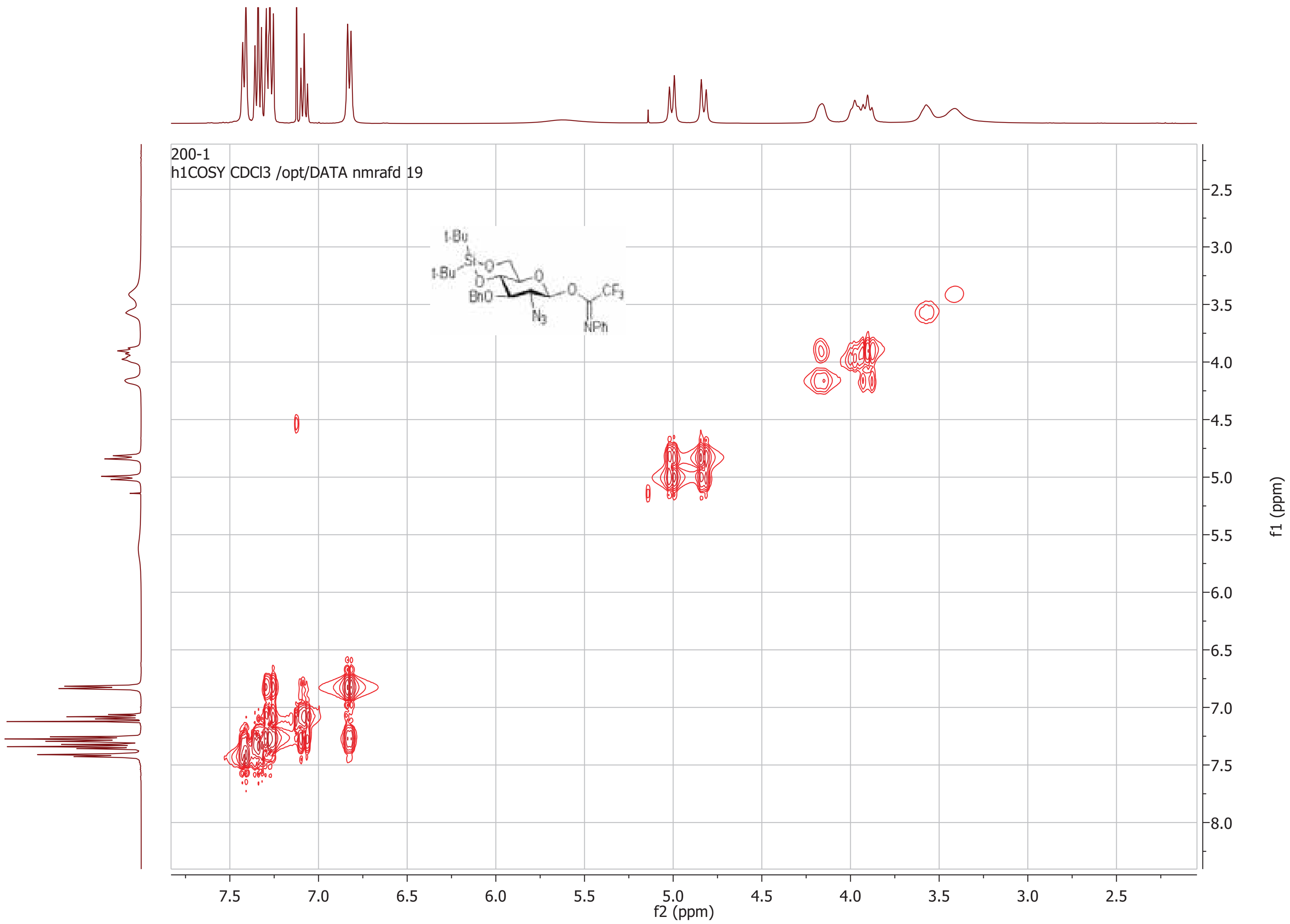
200-1
h1COSY CDCl3 /opt/DATA nmrafd 19
t-Bu
Si
t-Bu
O
O
BnO
O
O
CF3
N3
NPh
f2 (ppm)
f1 (ppm)

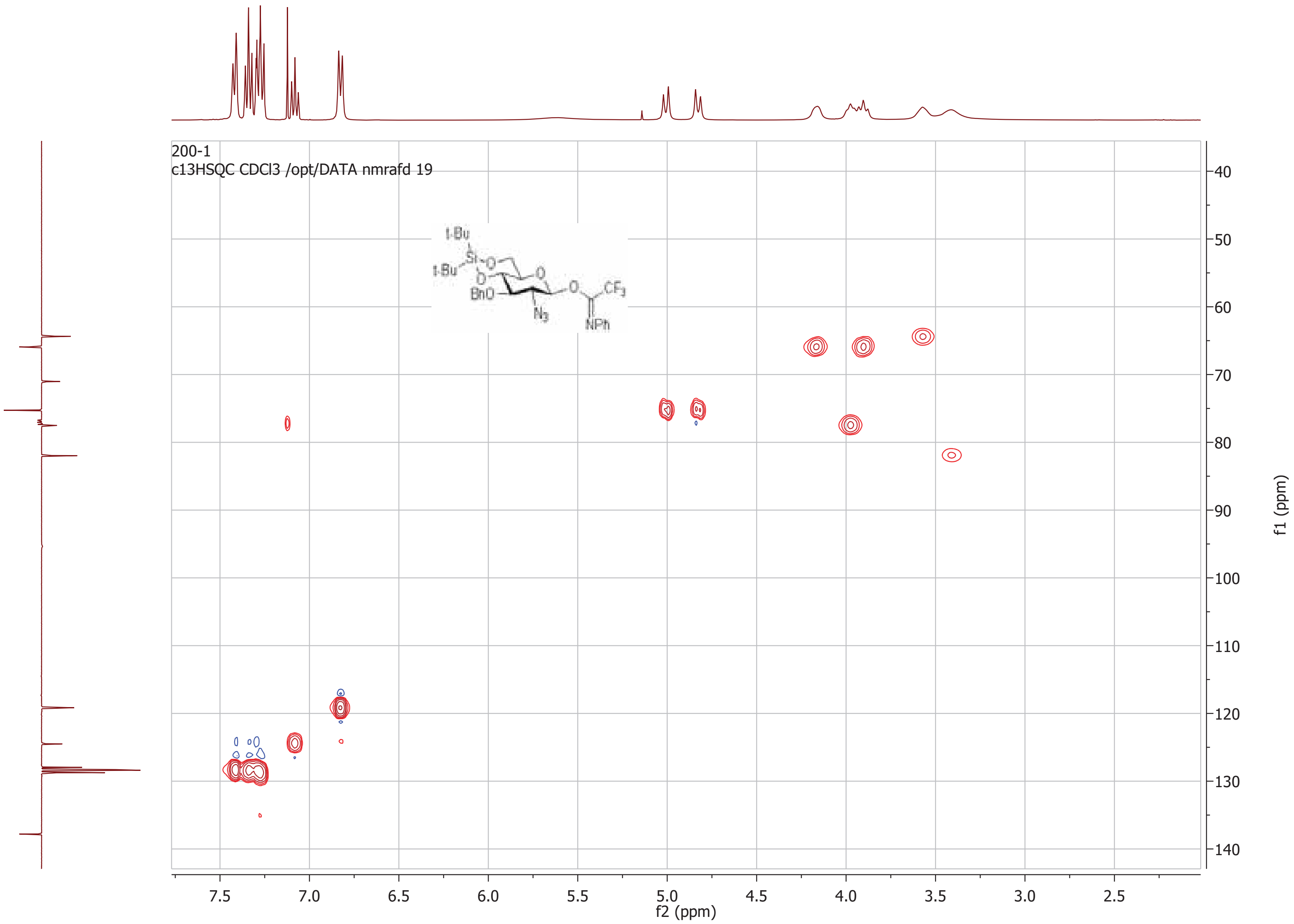

200-1
c13HSQC CDCl3 /opt/DATA nmrafd 19
t-Bu
t-Bu
Si
O
O
BnO
O
N3
O
CF3
NPh
f2 (ppm)
f1 (ppm)

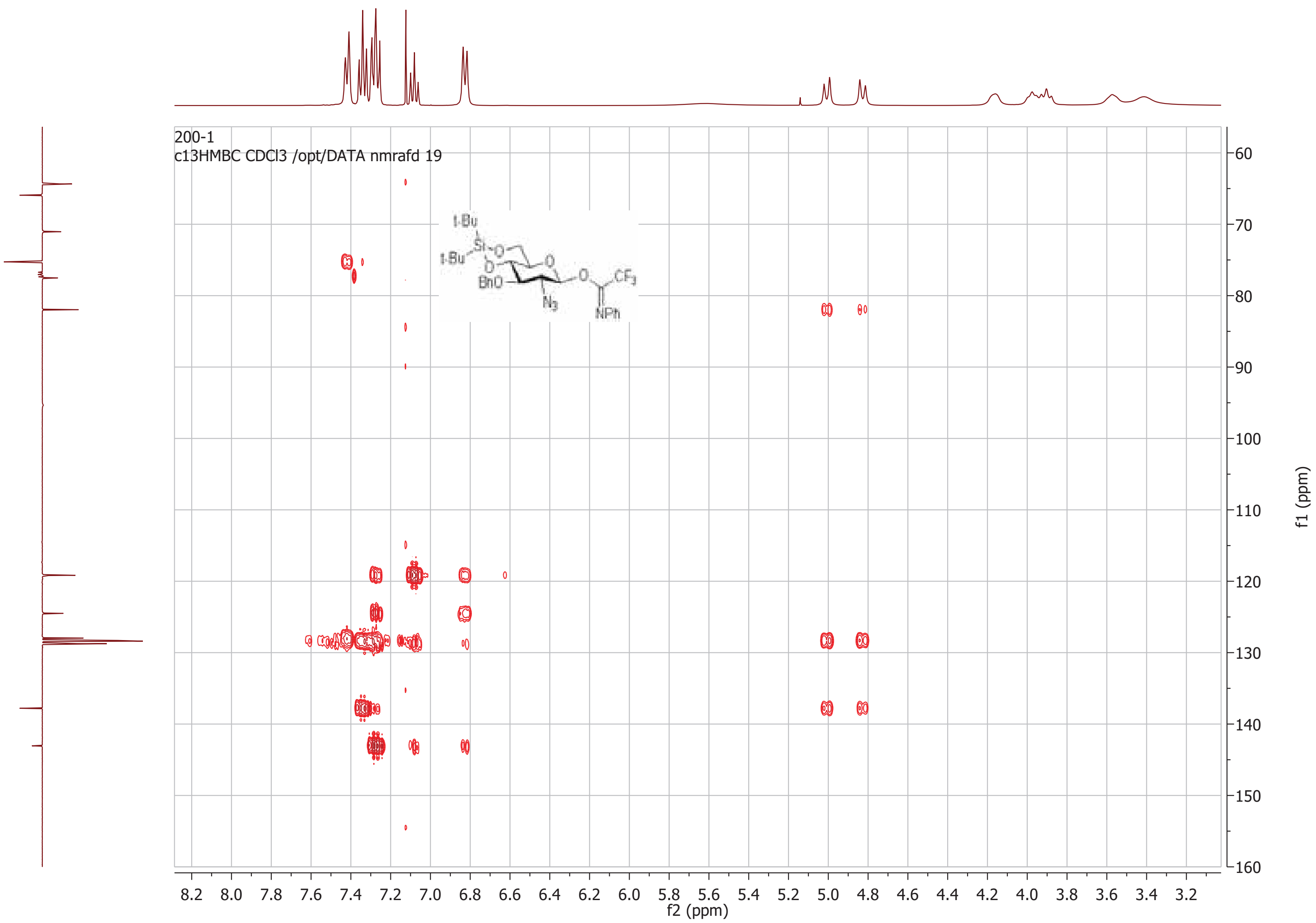

200-1
c13HMBC CDCl3 /opt/DATA nmrafd 19
t-Bu
Si
t-Bu
O
O
O
BnO
N3
O
CF3
NPh
f2 (ppm)
f1 (ppm)

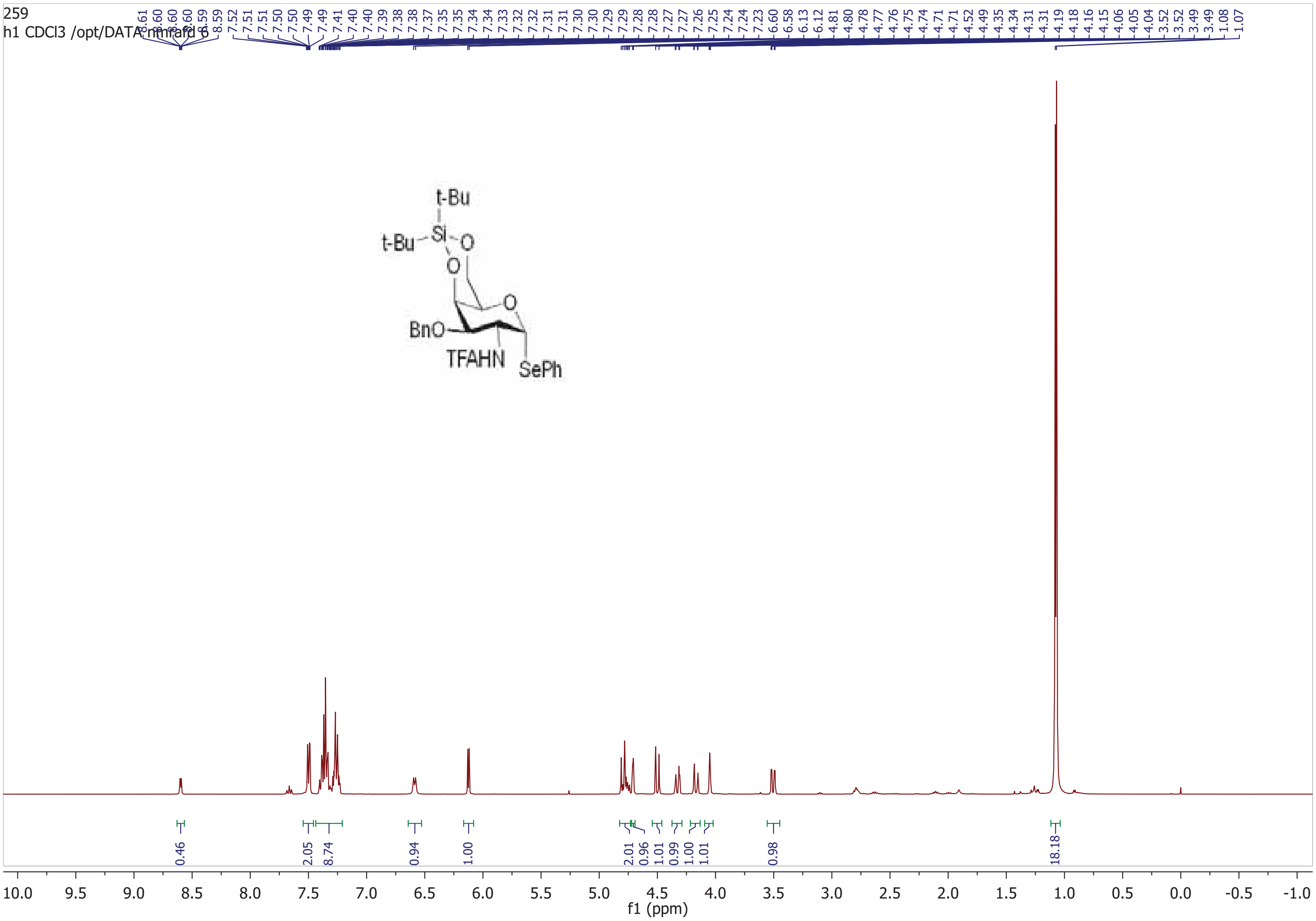

259
h1 CDCl3 /opt/DATA nmrafd 6
8.61 8.60 8.60 8.60 8.59 7.52 7.51 7.50 7.50 7.49 7.49 7.41 7.40 7.40 7.39 7.38 7.38 7.37 7.35 7.35 7.34 7.34 7.33 7.32 7.32 7.31 7.31 7.30 7.30 7.29 7.28 7.28 7.27 7.27 7.26 7.25 7.24 7.24 7.23 6.60 6.58 6.13 6.12 4.81 4.80 4.78 4.77 4.76 4.75 4.74 4.71 4.71 4.52 4.49 4.35 4.34 4.31 4.31 4.19 4.18 4.16 4.15 4.06 4.05 4.04 3.52 3.49 3.49 1.08 1.07
t-Bu
t-Bu
Si
O
O
O
BnO
TFAHN
SePh
0.46
2.05
8.74
0.94
1.00
2.01
0.96
1.01
0.99
1.00
1.01
0.98
18.18
10.0 9.5 9.0 8.5 8.0 7.5 7.0 6.5 6.0 5.5 5.0 4.5 4.0 3.5 3.0 2.5 2.0 1.5 1.0 0.5 0.0 -0.5 -1.0
f1 (ppm)

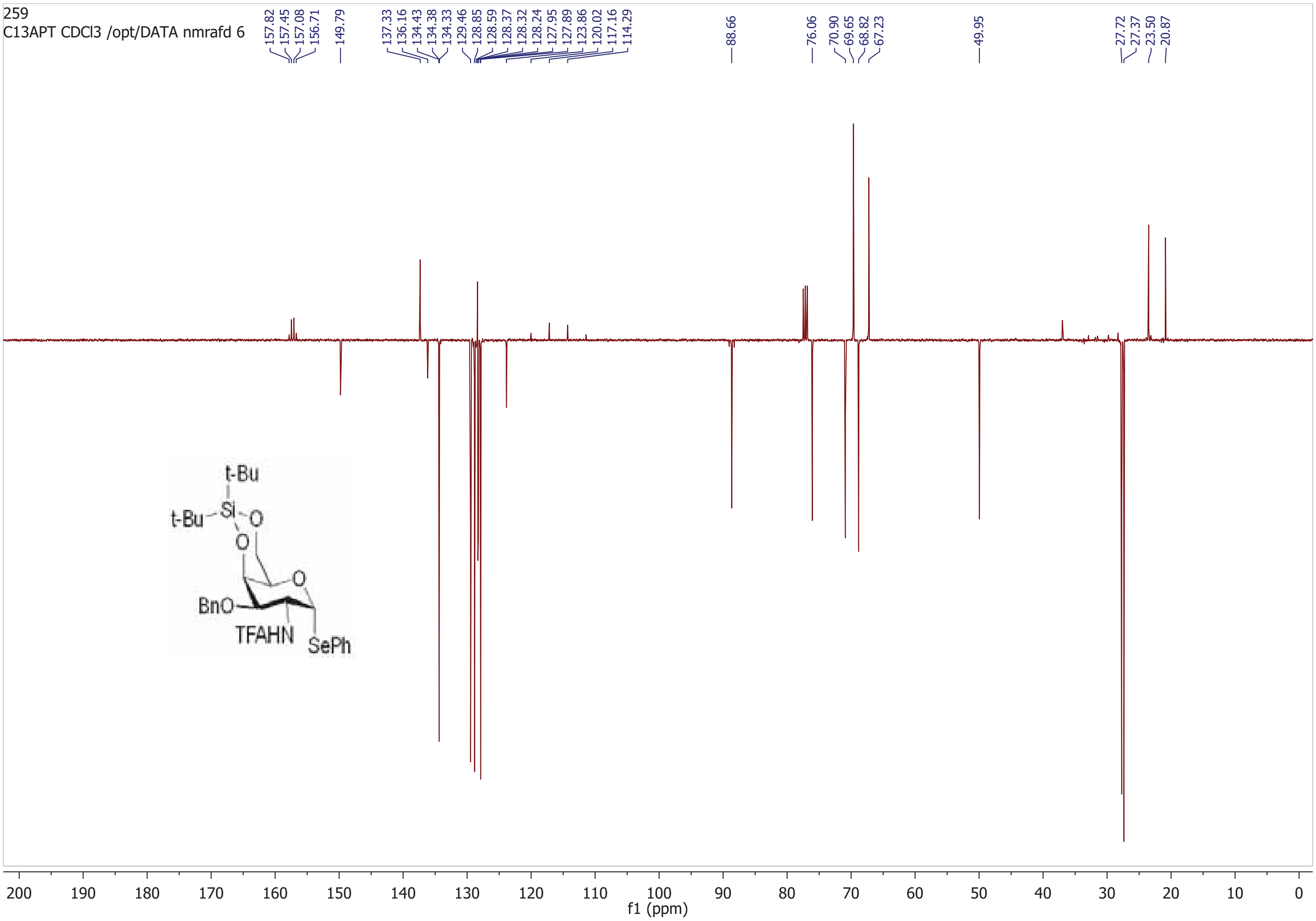
259
C13APT CDCl3 /opt/DATA nmrafd 6
157.82
157.45
157.08
156.71
149.79
137.33
136.16
134.43
134.38
134.33
129.46
128.85
128.59
128.37
128.32
128.24
127.95
127.89
123.86
120.02
117.16
114.29
88.66
76.06
70.90
69.65
68.82
67.23
49.95
27.72
27.37
23.50
20.87
t-Bu
t-Bu
Si
O
O
O
BnO
TFAHN
SePh
200 190 180 170 160 150 140 130 120 110 100 90 80 70 60 50 40 30 20 10 0
f1 (ppm)

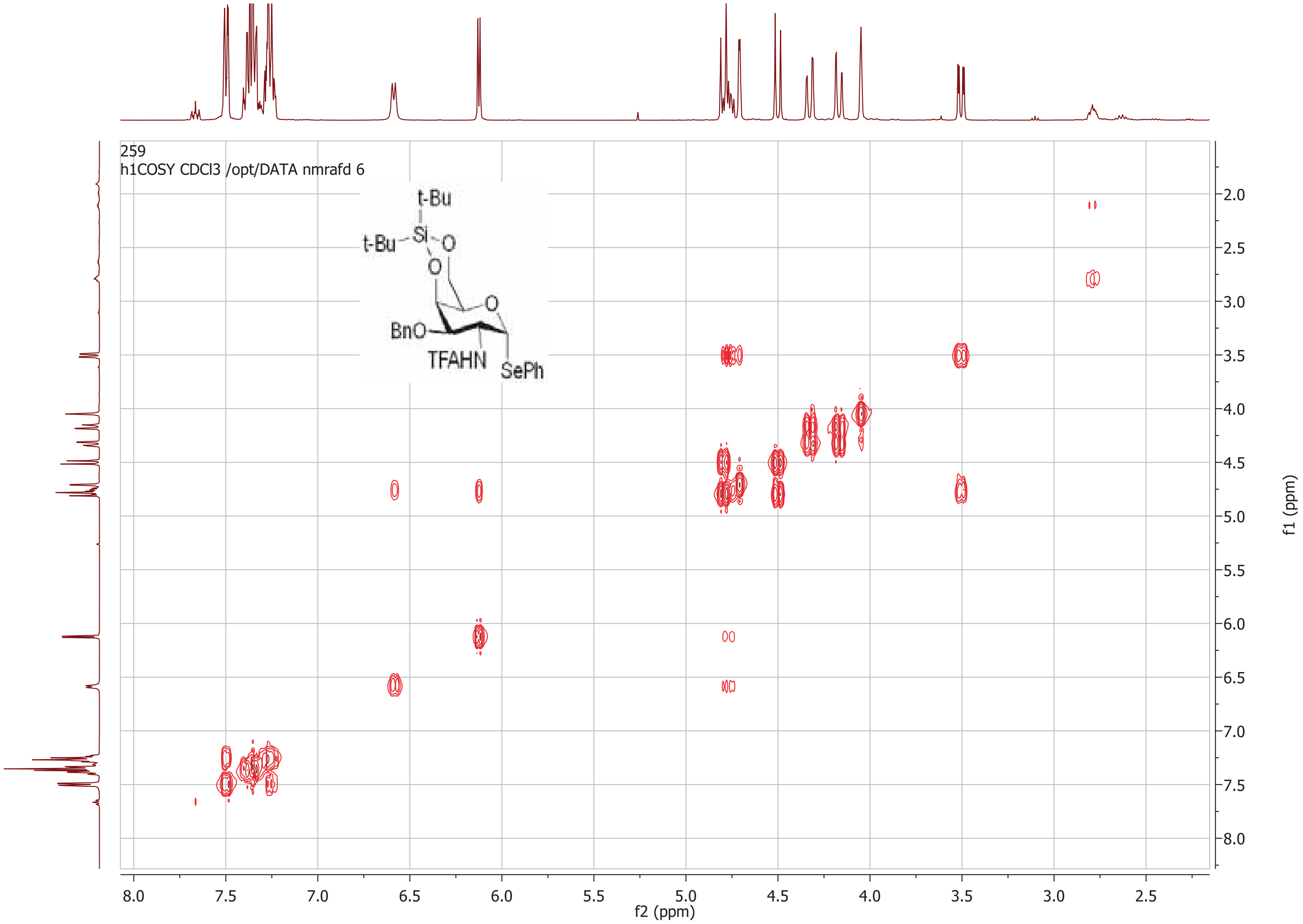
259
h1COSY CDCl3 /opt/DATA nmrafd 6
t-Bu
t-Bu
Si
O
O
O
BnO
TFAHN
SePh
f2 (ppm)
f1 (ppm)
8.0
7.5
7.0
6.5
6.0
5.5
5.0
4.5
4.0
3.5
3.0
2.5
2.0

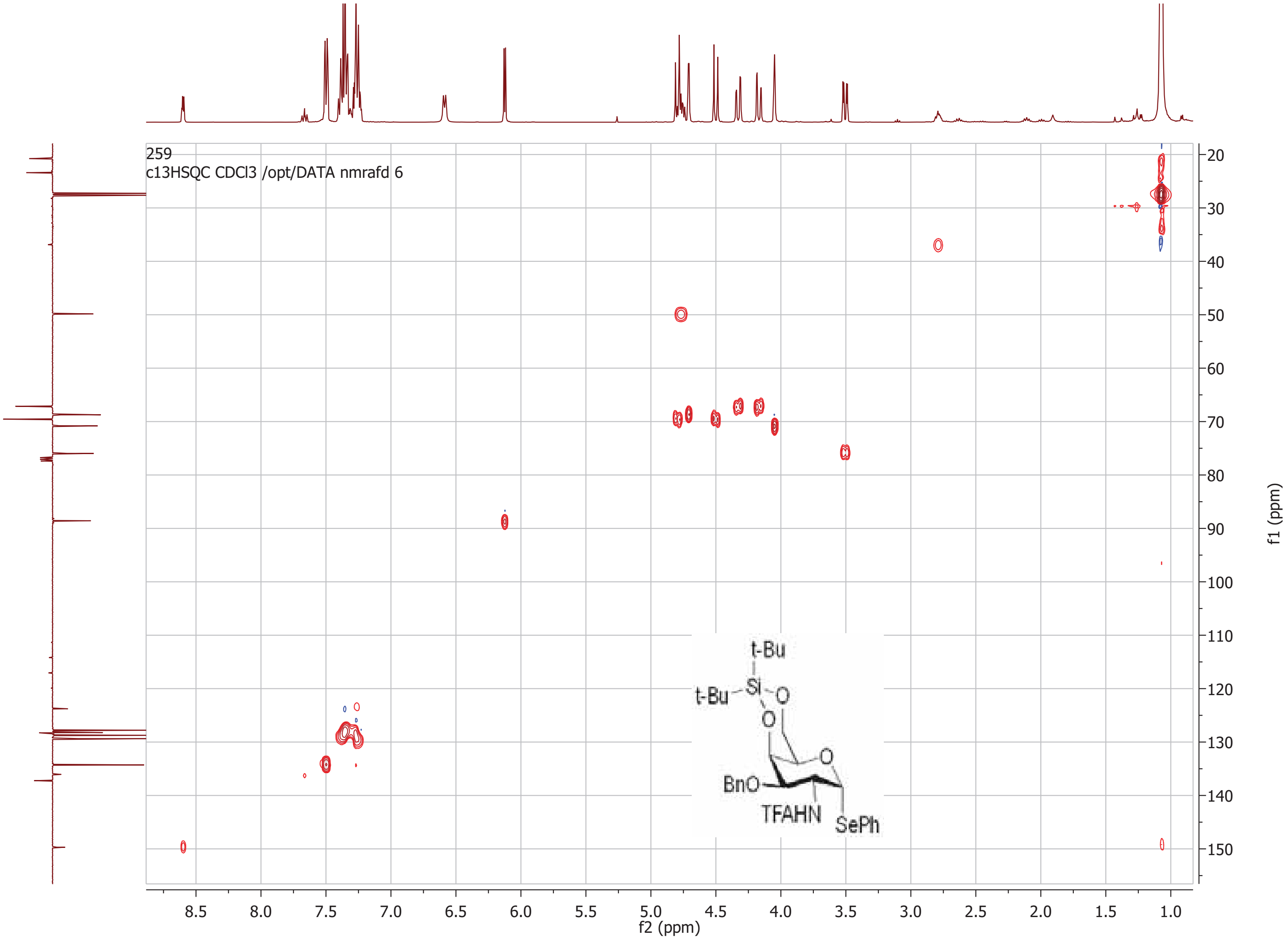
259
c13HSQC CDCl3 /opt/DATA nmrafd 6
t-Bu
t-Bu
Si
O
O
O
BnO
TFAHN
SePh
f2 (ppm)
f1 (ppm)

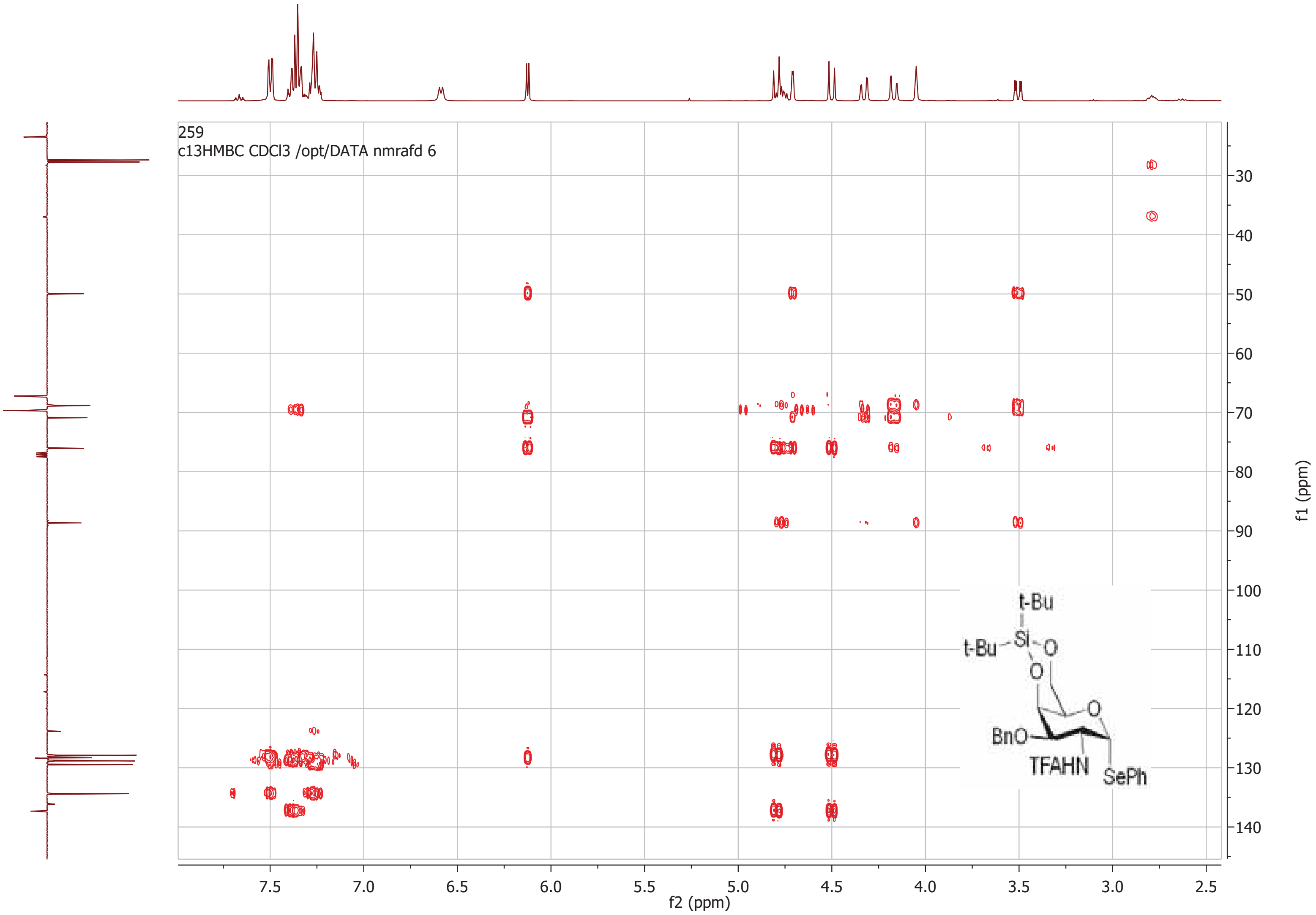

259
c13HMBC CDCl3 /opt/DATA nmrafd 6
f2 (ppm)
f1 (ppm)
t-Bu
t-Bu
Si
O
O
O
BnO
TFAHN
SePh

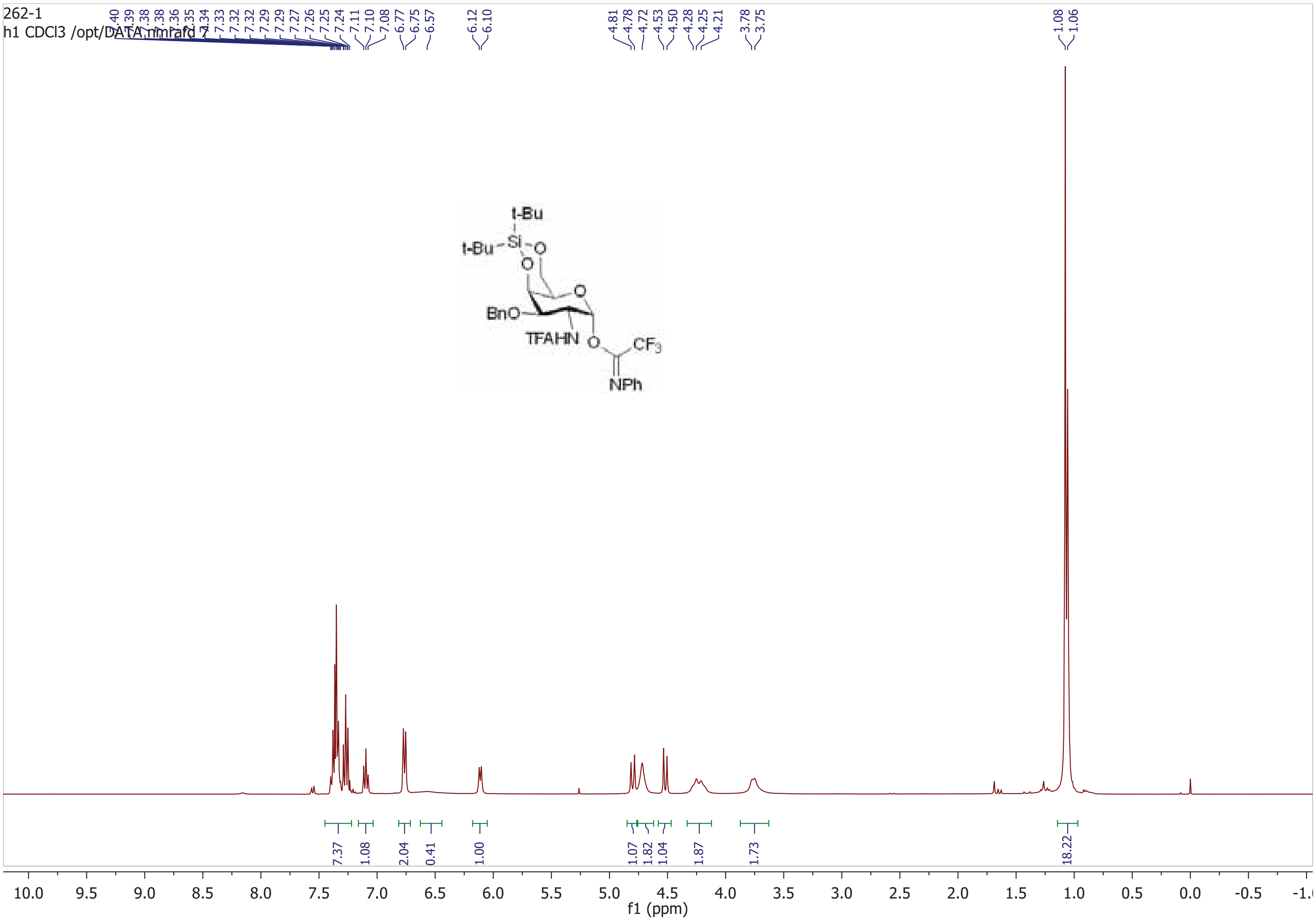
262-1
h1 CDCl3 /opt/DATA.nmrafd 7
7.40 7.39 7.38 7.38 7.36 7.35 7.34 7.33 7.32 7.32 7.29 7.29 7.27 7.26 7.25 7.24 7.11 7.10 7.08 6.77 6.75 6.57
6.12 6.10
4.81 4.78 4.72 4.53 4.50 4.28 4.25 4.21 3.78 3.75
1.08 1.06
t-Bu
t-Bu
Si
O
O
O
BnO
TFAHN
O
CF3
NPh
7.37
1.08
2.04
0.41
1.00
1.07
1.82
1.04
1.87
1.73
18.22
10.0 9.5 9.0 8.5 8.0 7.5 7.0 6.5 6.0 5.5 5.0 4.5 4.0 3.5 3.0 2.5 2.0 1.5 1.0 0.5 0.0 -0.5
f1 (ppm)

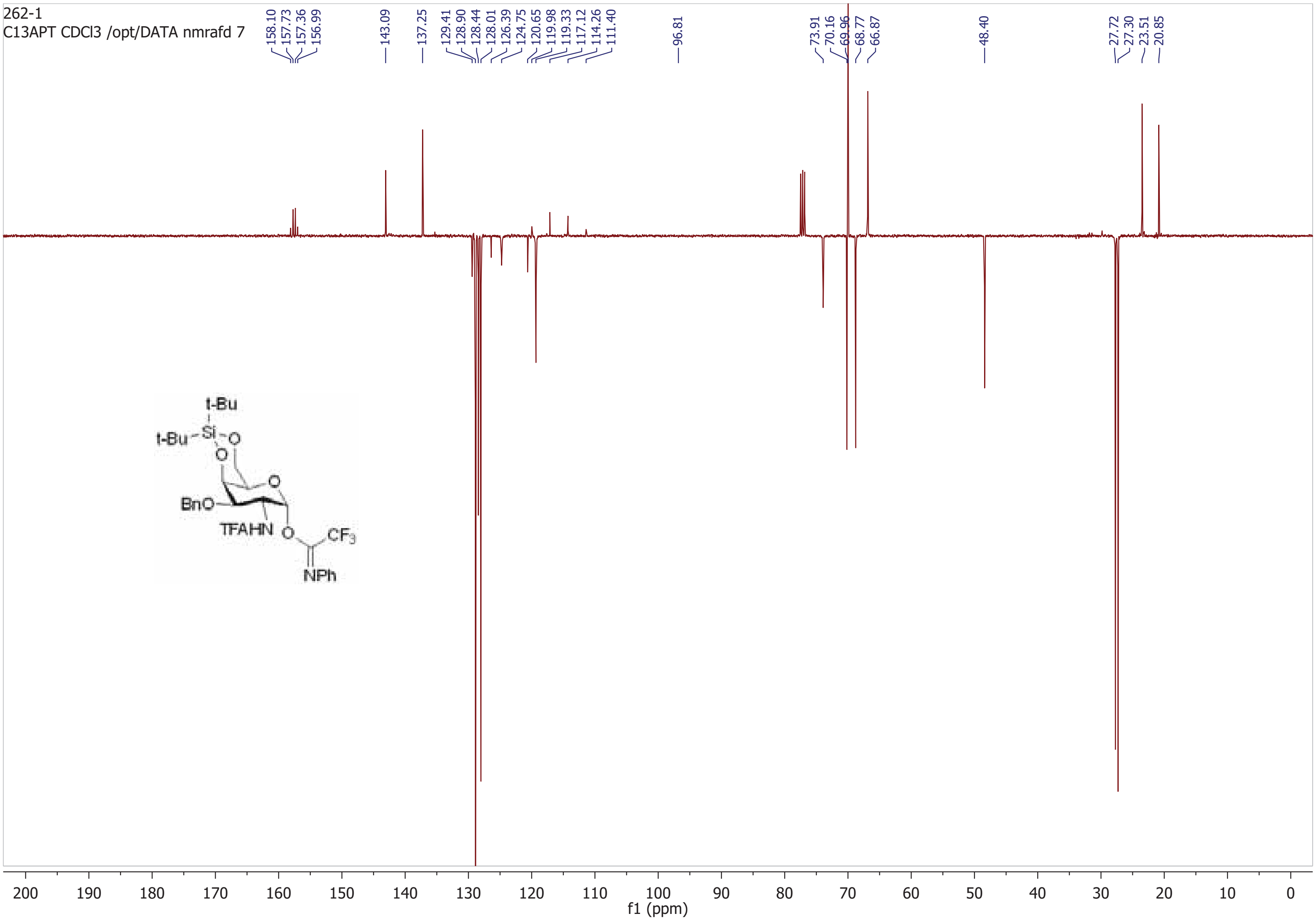
262-1
C13APT CDCl3 /opt/DATA nmrafd 7
158.10
157.73
157.36
156.99
143.09
137.25
129.41
128.90
128.44
128.01
126.39
124.75
120.65
119.98
119.33
117.12
114.26
111.40
96.81
73.91
70.16
69.96
68.77
66.87
48.40
27.72
27.30
23.51
20.85
t-Bu
t-Bu
Si
O
O
O
BnO
TFAHN
O
CF3
NPh
200
190
180
170
160
150
140
130
120
110
100
90
80
70
60
50
40
30
20
10
0
f1 (ppm)

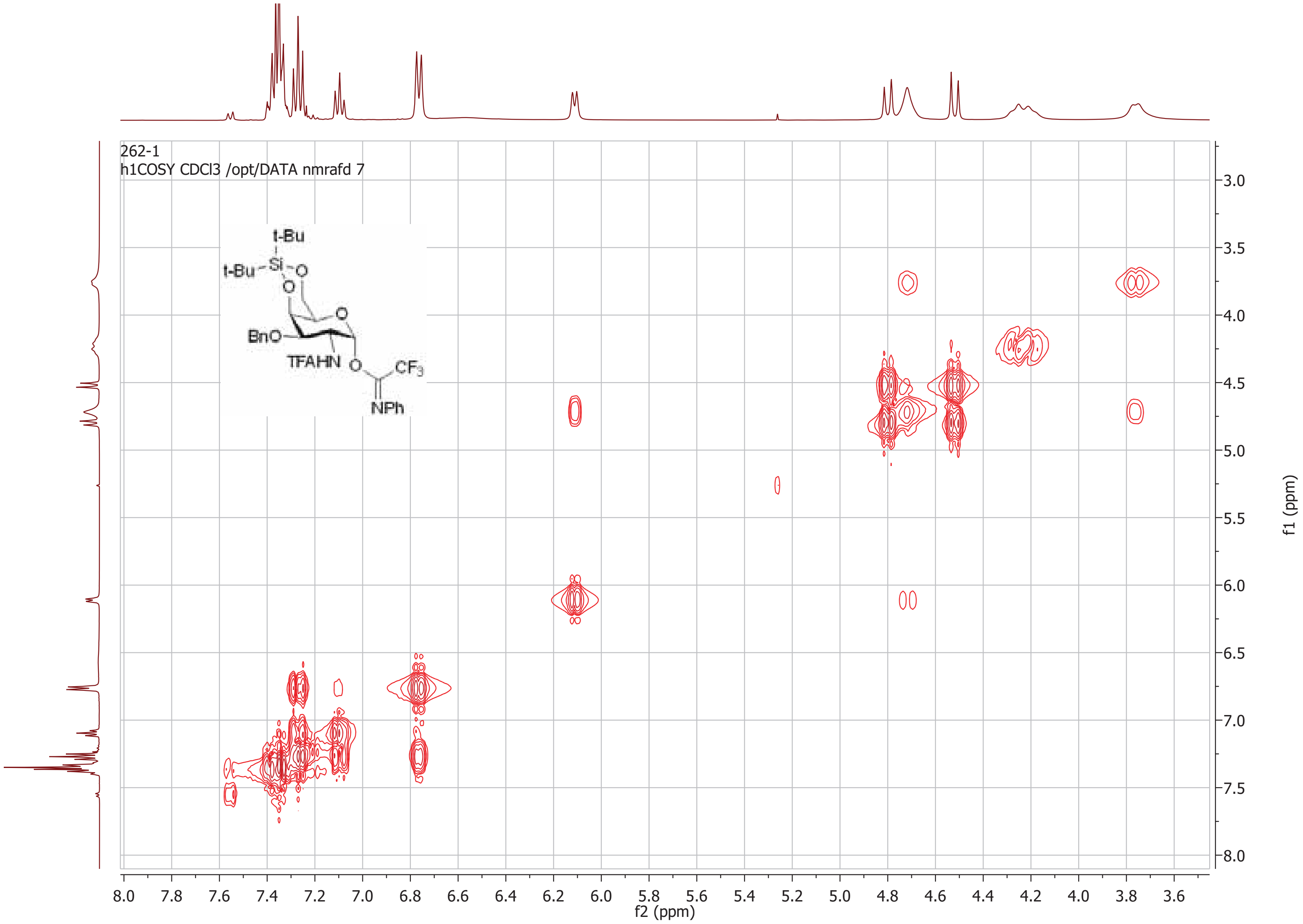
262-1
h1COSY CDCl3 /opt/DATA nmrafd 7
t-Bu
t-Bu
Si
O
O
O
BnO
TFAHN
O
CF_3
NPh
f2 (ppm)
f1 (ppm)
8.0
7.8
7.6
7.4
7.2
7.0
6.8
6.6
6.4
6.2
6.0
5.8
5.6
5.4
5.2
5.0
4.8
4.6
4.4
4.2
4.0
3.8
3.6
3.0
3.5
4.0
4.5
5.0
5.5
6.0
6.5
7.0
7.5
8.0

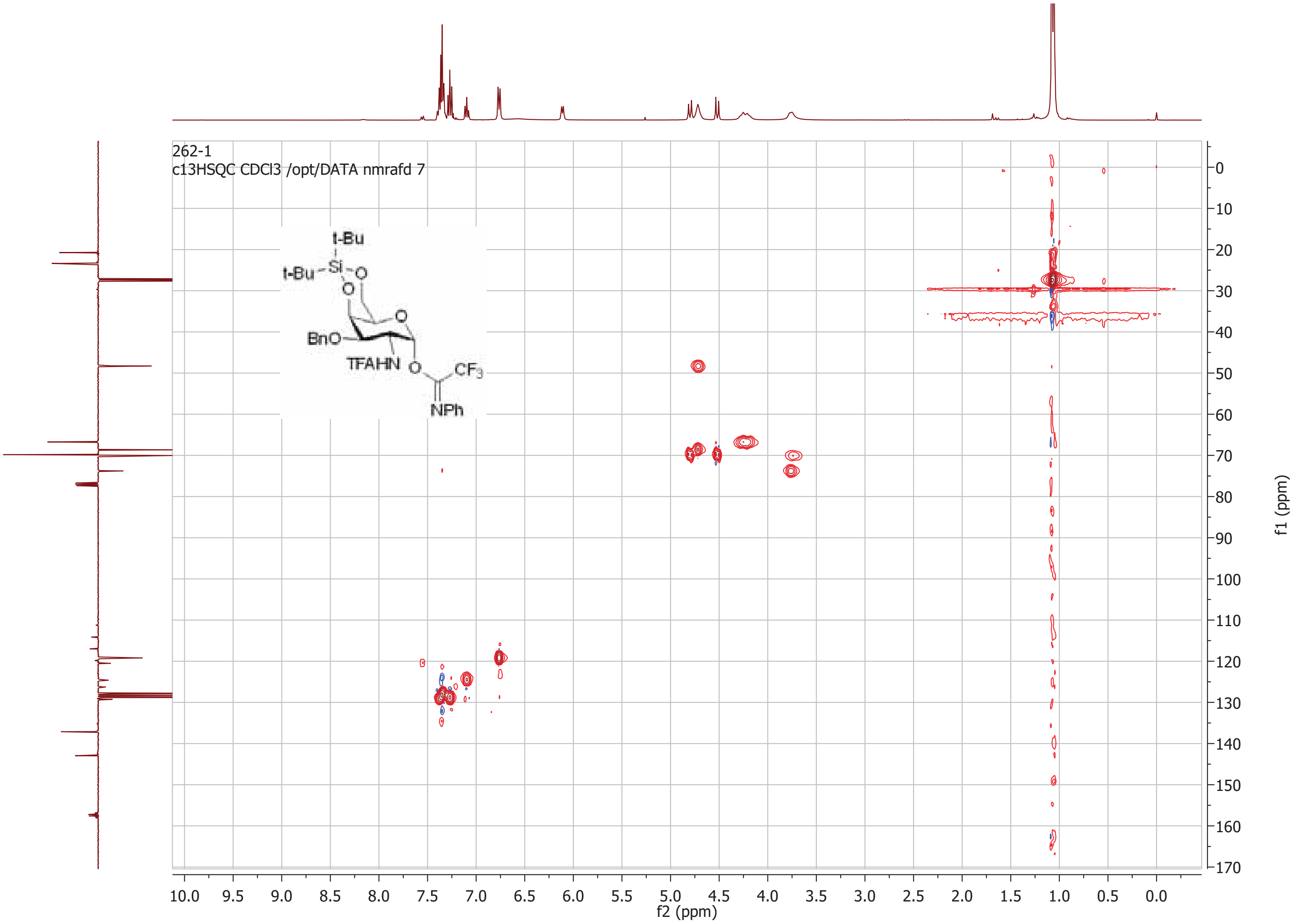

262-1
c13HSQC CDCl3 /opt/DATA nmrafd 7
t-Bu
t-Bu
Si
O
O
O
BnO
TFAHN
O
CF3
NPh
f2 (ppm)
f1 (ppm)

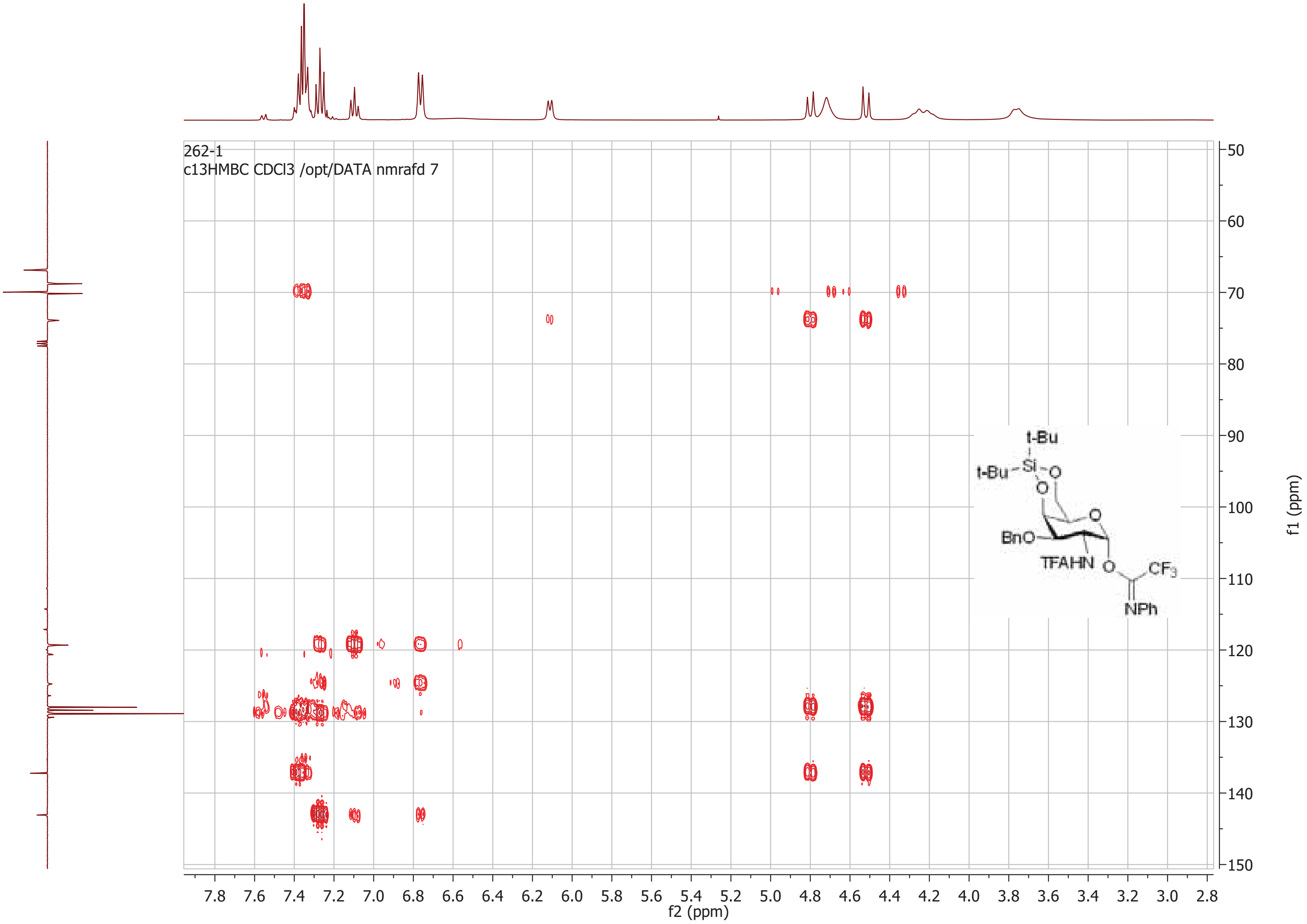
262-1
c13HMBC CDCl3 /opt/DATA nmrafd 7
f2 (ppm)
f1 (ppm)
t-Bu
t-Bu
Si
O
O
O
BnO
TFAHN
O
CF3
NPh

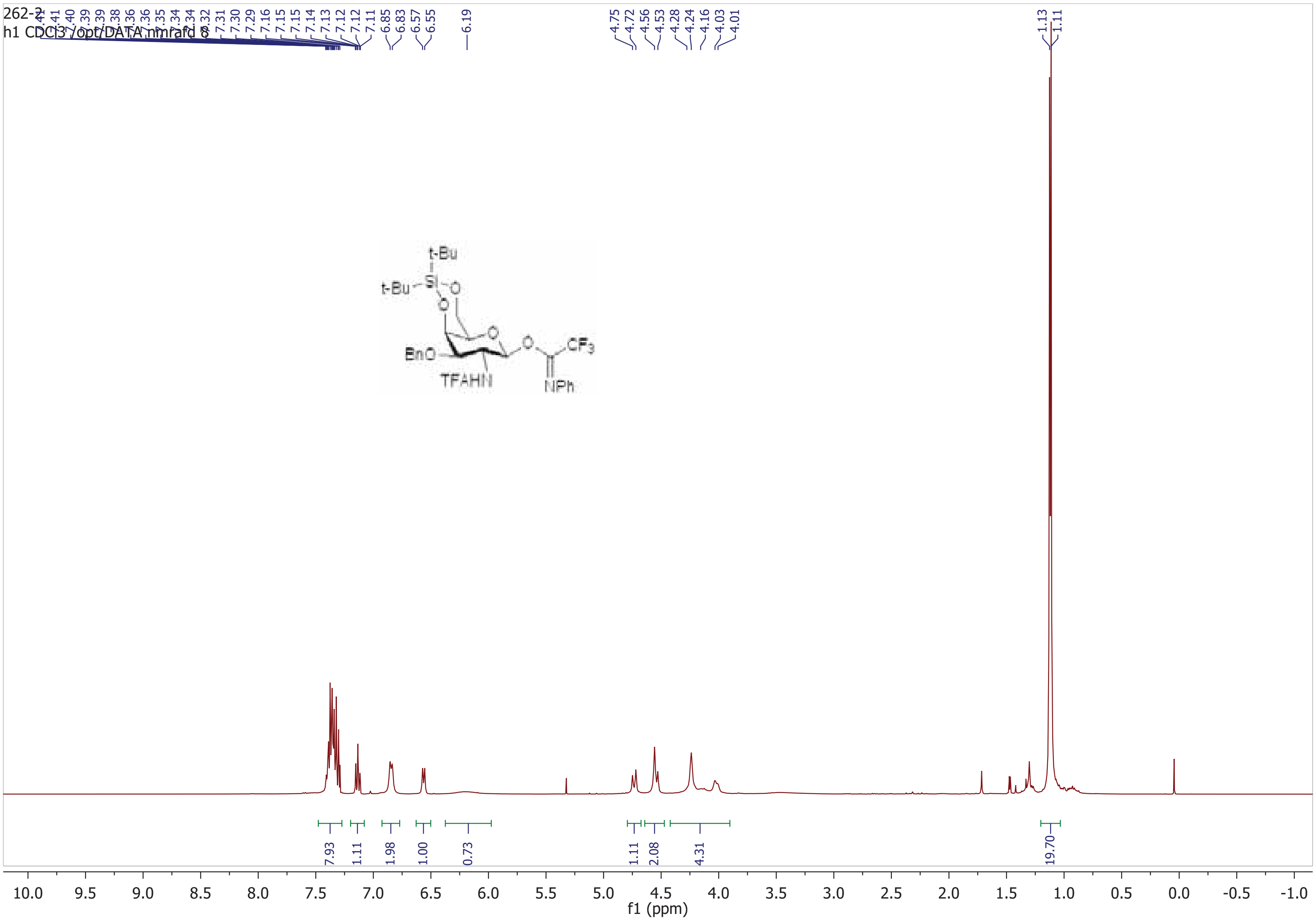
262-2
h1 CDCl3 /opt/DATA nmrafd 8
7.41
7.41
7.40
7.39
7.39
7.38
7.36
7.36
7.35
7.34
7.34
7.32
7.31
7.30
7.29
7.16
7.15
7.15
7.14
7.13
7.12
7.11
6.85
6.83
6.57
6.55
6.19
4.75
4.72
4.56
4.53
4.28
4.24
4.16
4.03
4.01
1.13
1.11
t-Bu
t-Bu
Si
O
O
BnO
O
O
CF3
TFAHN
NPh
7.93
1.11
1.98
1.00
0.73
1.11
2.08
4.31
19.70
10.0
9.5
9.0
8.5
8.0
7.5
7.0
6.5
6.0
5.5
5.0
4.5
4.0
3.5
3.0
2.5
2.0
1.5
1.0
0.5
0.0
-0.5
-1.0
f1 (ppm)

262-2
C13APT CDCl3 /opt/DATA nmrafd 8

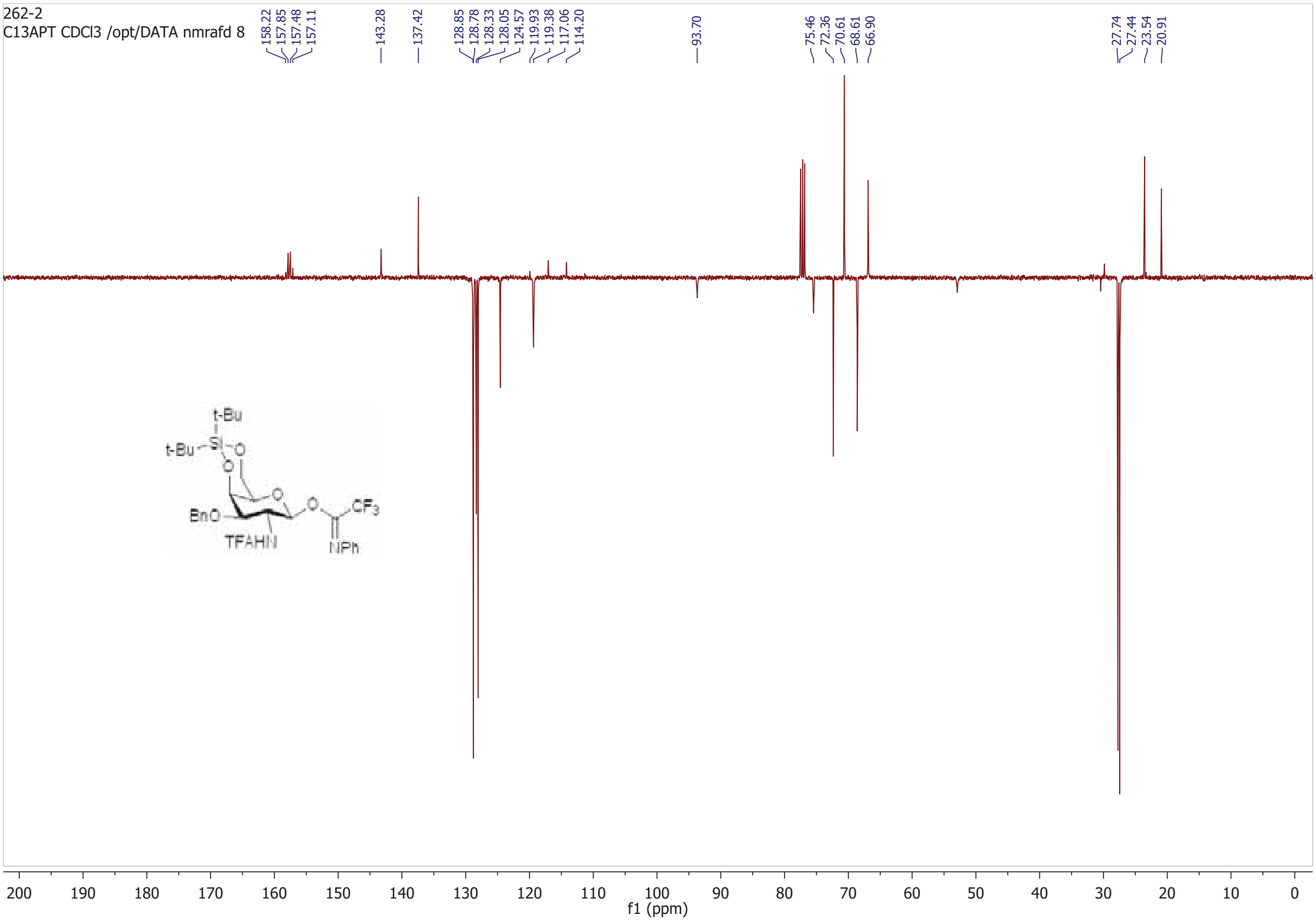

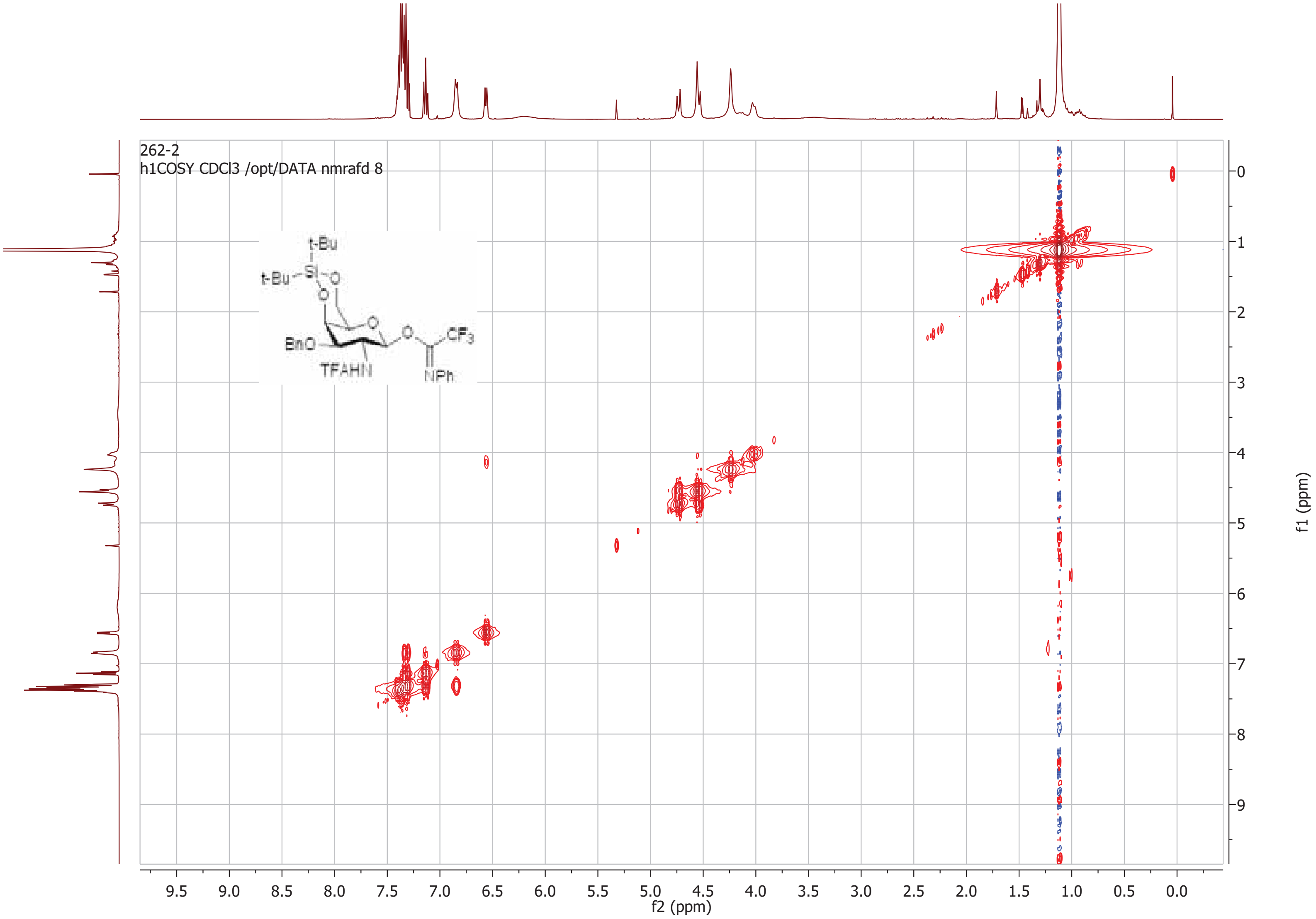

262-2
h1COSY CDCl3 /opt/DATA nmrafd 8
t-Bu
t-Bu
Si
O
O
O
O
BnO
TFAHN
CF3
NPh
f2 (ppm)
f1 (ppm)

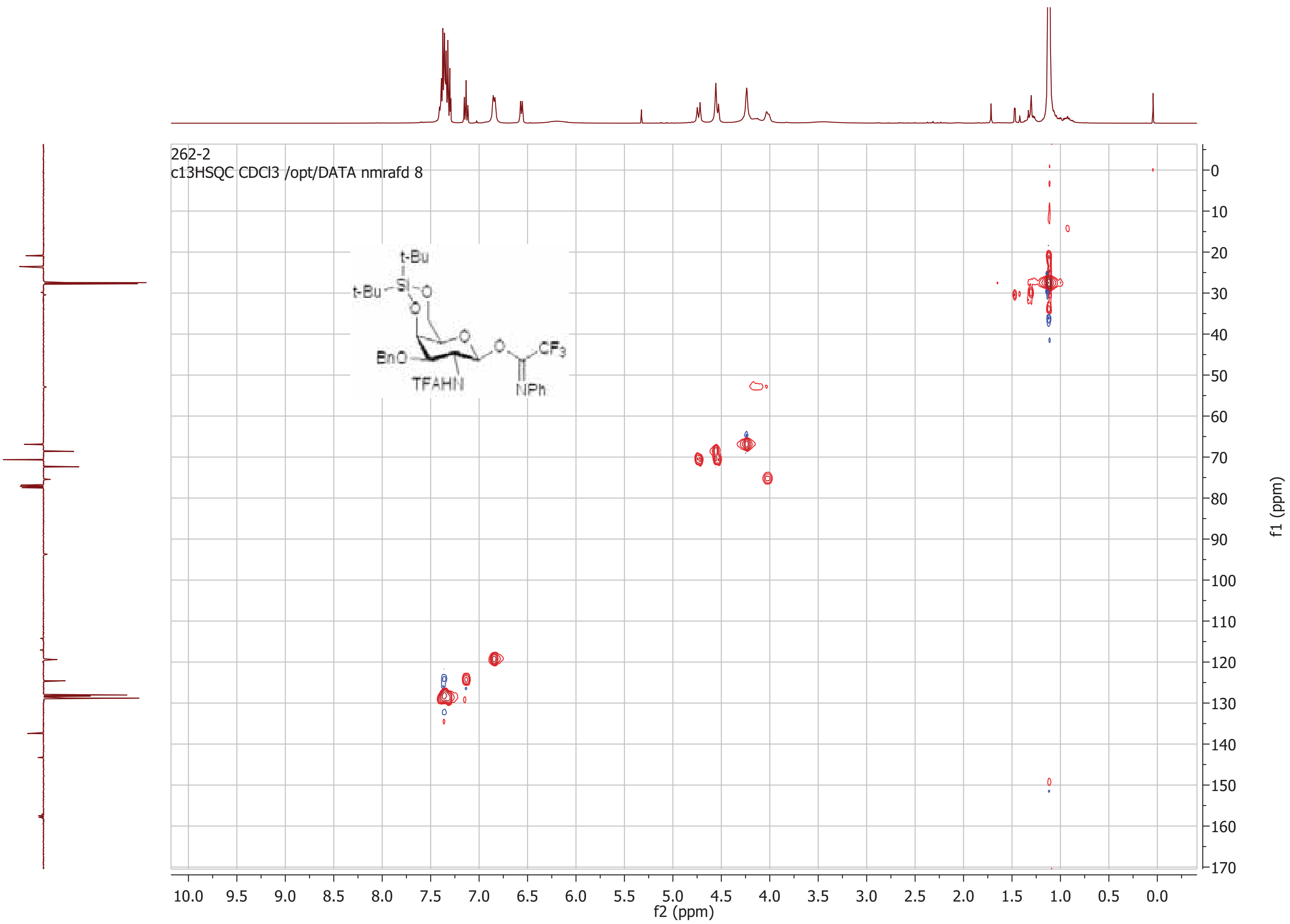
262-2
c13HSQC CDCl3 /opt/DATA nmrafd 8
t-Bu
t-Bu
Si
O
O
O
BnO
TFAHN
O
CF3
NPh
f2 (ppm)
f1 (ppm)

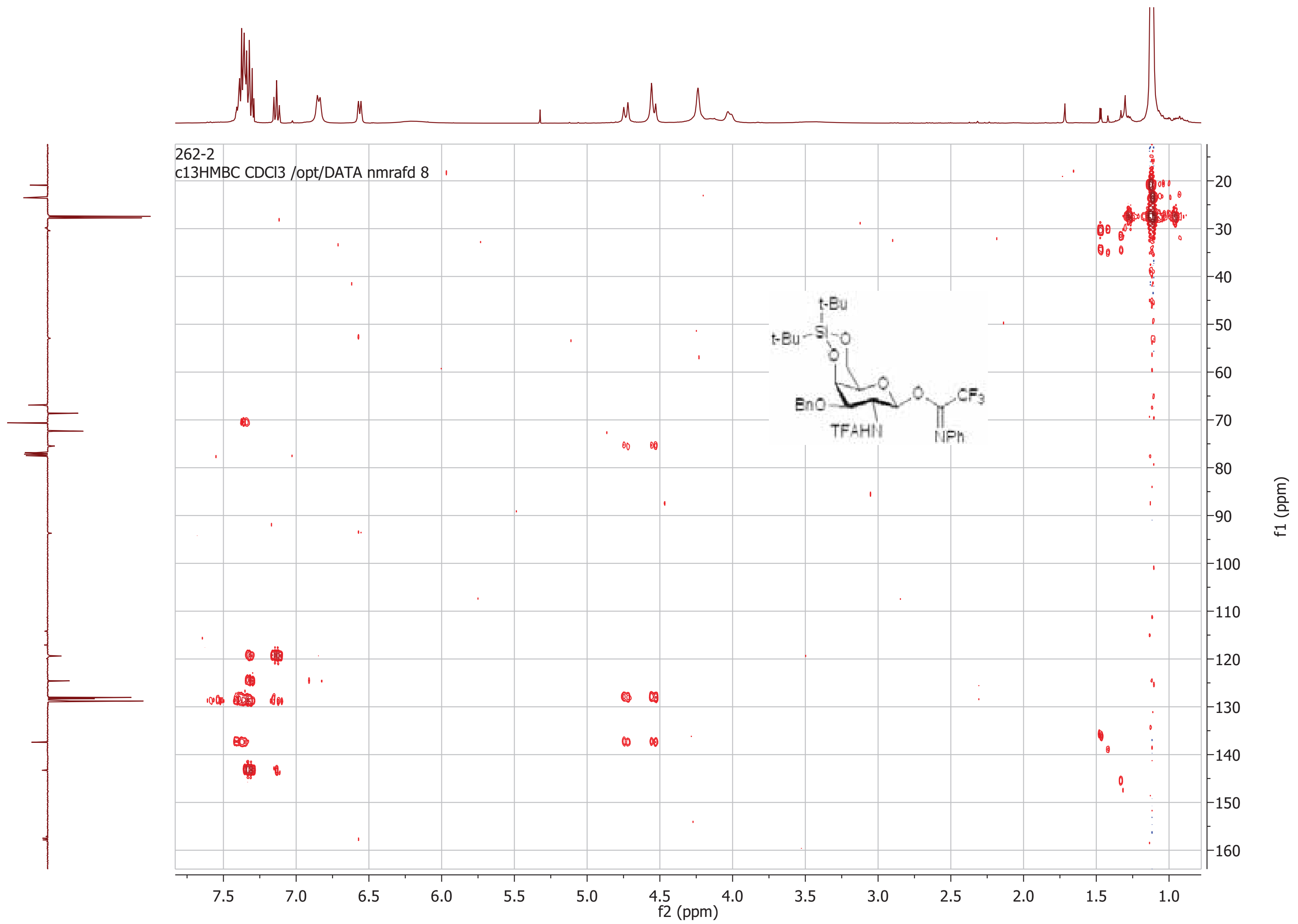
262-2
c13HMBC CDCl3 /opt/DATA nmrafd 8
t-Bu
t-Bu
Si
BnO
TFAHN
CF3
NPh
f2 (ppm)
f1 (ppm)

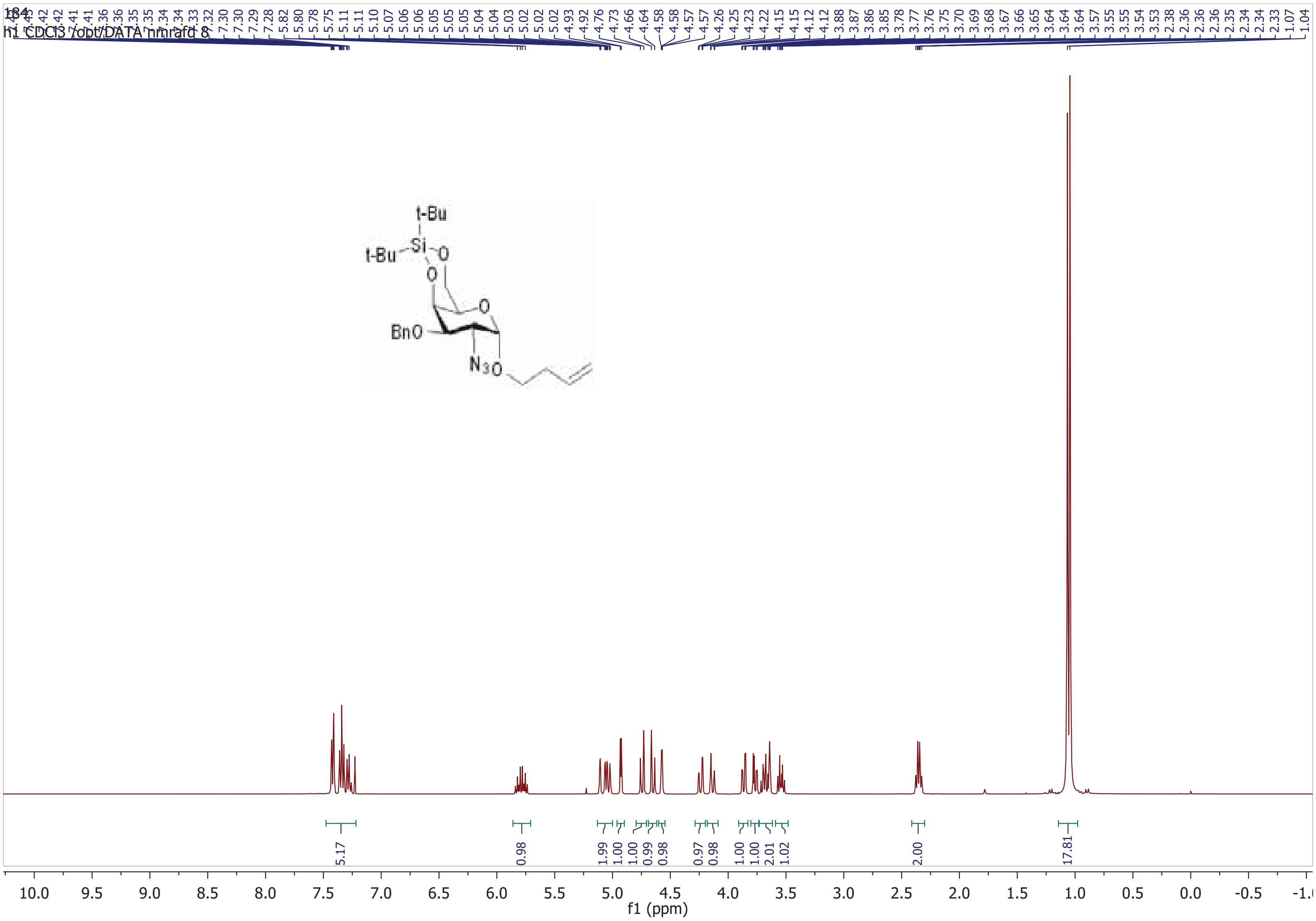
184
h1 CDCl3 /opt/DATA nmrafd 8
7.45 7.42 7.42 7.41 7.41 7.36 7.36 7.35 7.35 7.34 7.34 7.33 7.32 7.30 7.30 7.29 7.28 5.82 5.80 5.78 5.75 5.11 5.11 5.10 5.07 5.06 5.06 5.05 5.05 5.05 5.04 5.04 5.03 5.02 5.02 5.02 4.93 4.92 4.76 4.73 4.66 4.64 4.58 4.58 4.57 4.57 4.26 4.25 4.23 4.22 4.15 4.15 4.12 4.12 3.88 3.87 3.86 3.85 3.78 3.77 3.76 3.75 3.70 3.69 3.68 3.67 3.66 3.65 3.64 3.64 3.64 3.57 3.55 3.55 3.54 3.53 2.38 2.36 2.36 2.35 2.34 2.34 2.33 1.07 1.04
t-Bu
t-Bu
Si
O
O
O
BnO
N3
O
5.17
0.98
1.99
1.00
1.00
0.99
0.98
0.97
0.98
1.00
1.00
2.01
1.02
2.00
17.81
10.0 9.5 9.0 8.5 8.0 7.5 7.0 6.5 6.0 5.5 5.0 4.5 4.0 3.5 3.0 2.5 2.0 1.5 1.0 0.5 0.0 -0.5
f1 (ppm)

184
C13APT CDCl3 /opt/DATA nmrafd 8

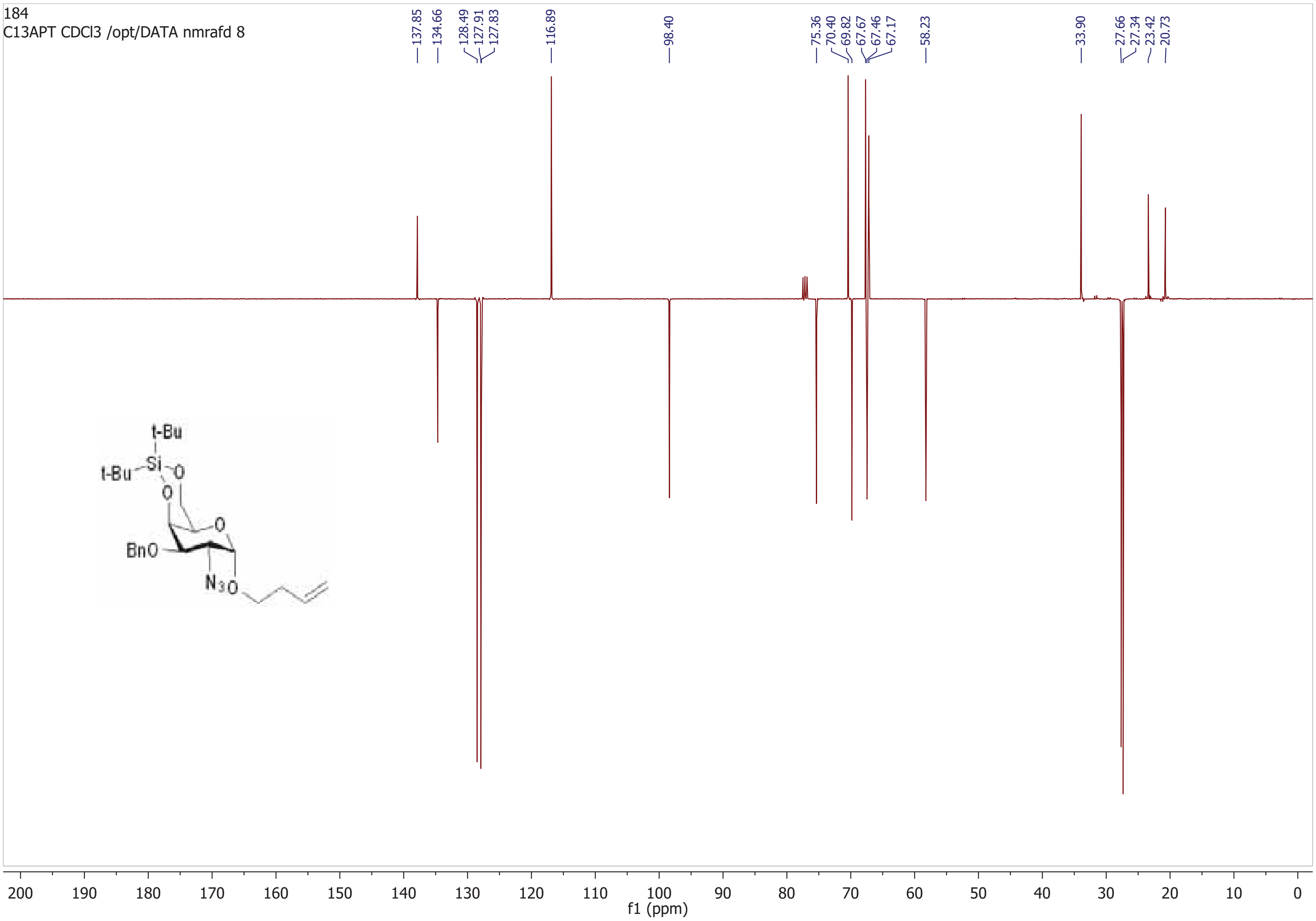

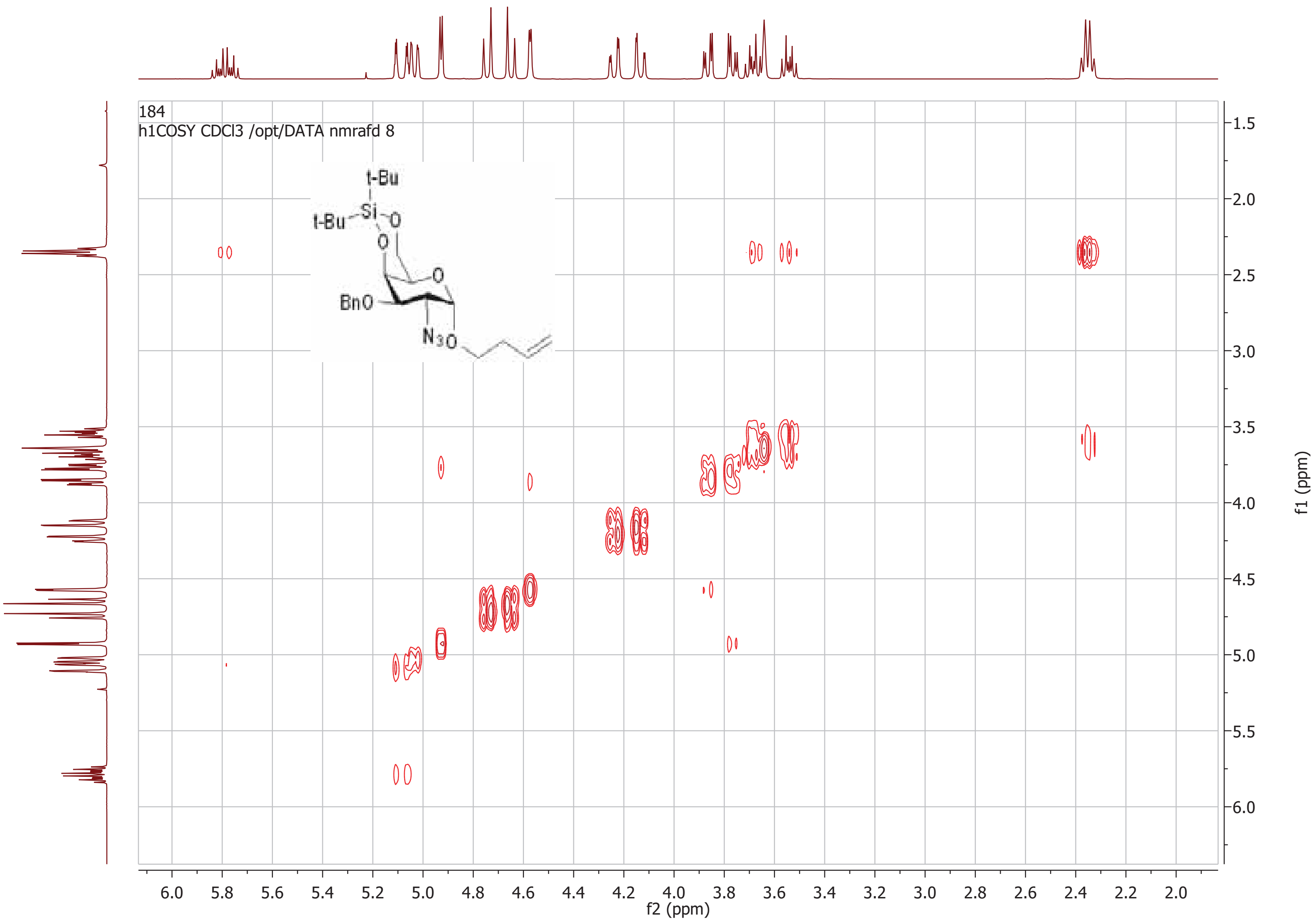
184
h1COSY CDCl3 /opt/DATA nmrafd 8
t-Bu
t-Bu
Si
O
O
O
BnO
N3
O
f2 (ppm)
f1 (ppm)

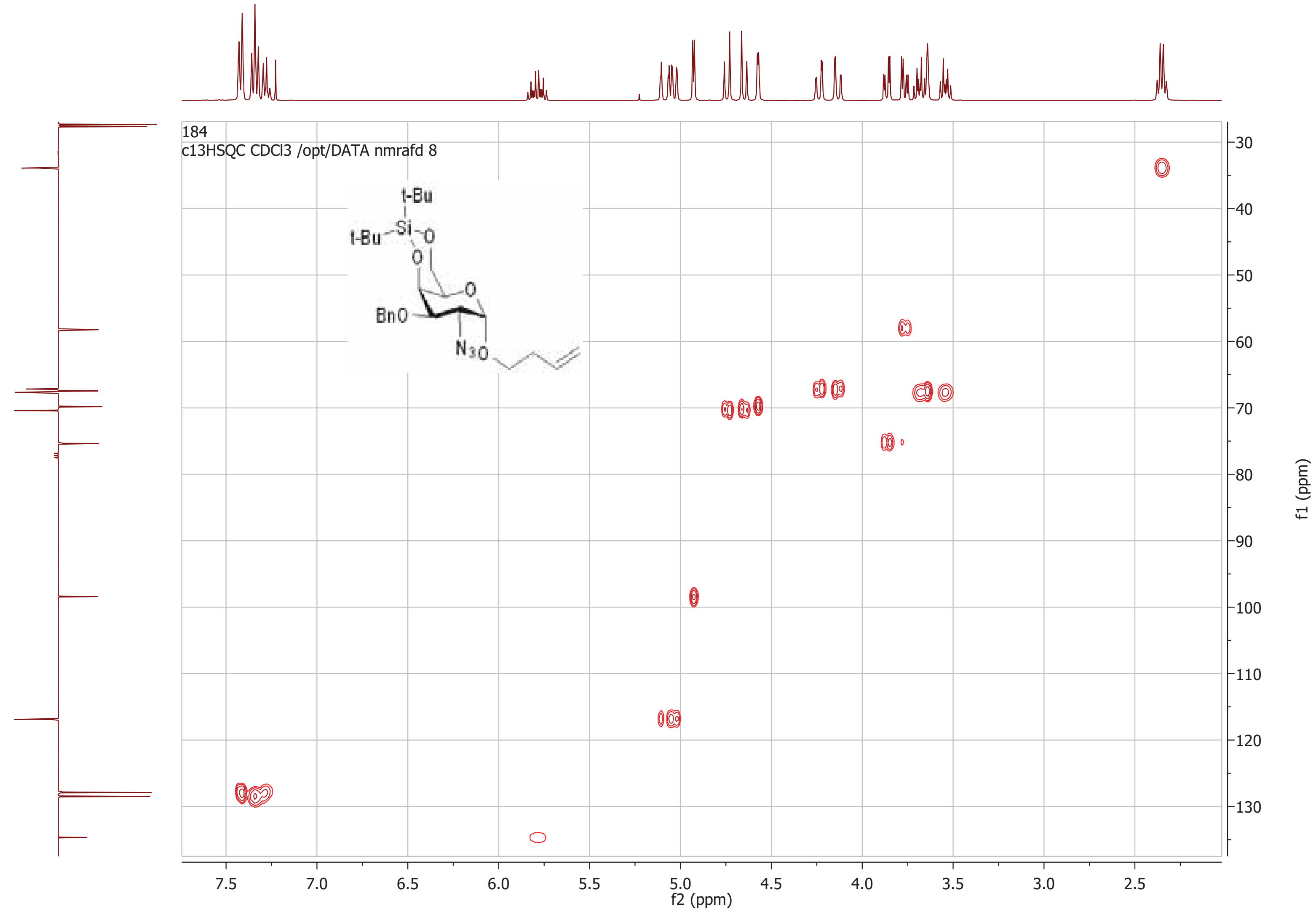
184
c13HSQC CDCl3 /opt/DATA nmrafd 8
t-Bu
t-Bu
Si
O
O
O
BnO
N_3
O
f2 (ppm)
f1 (ppm)
7.5
7.0
6.5
6.0
5.5
5.0
4.5
4.0
3.5
3.0
2.5
30
40
50
60
70
80
90
100
110
120
130

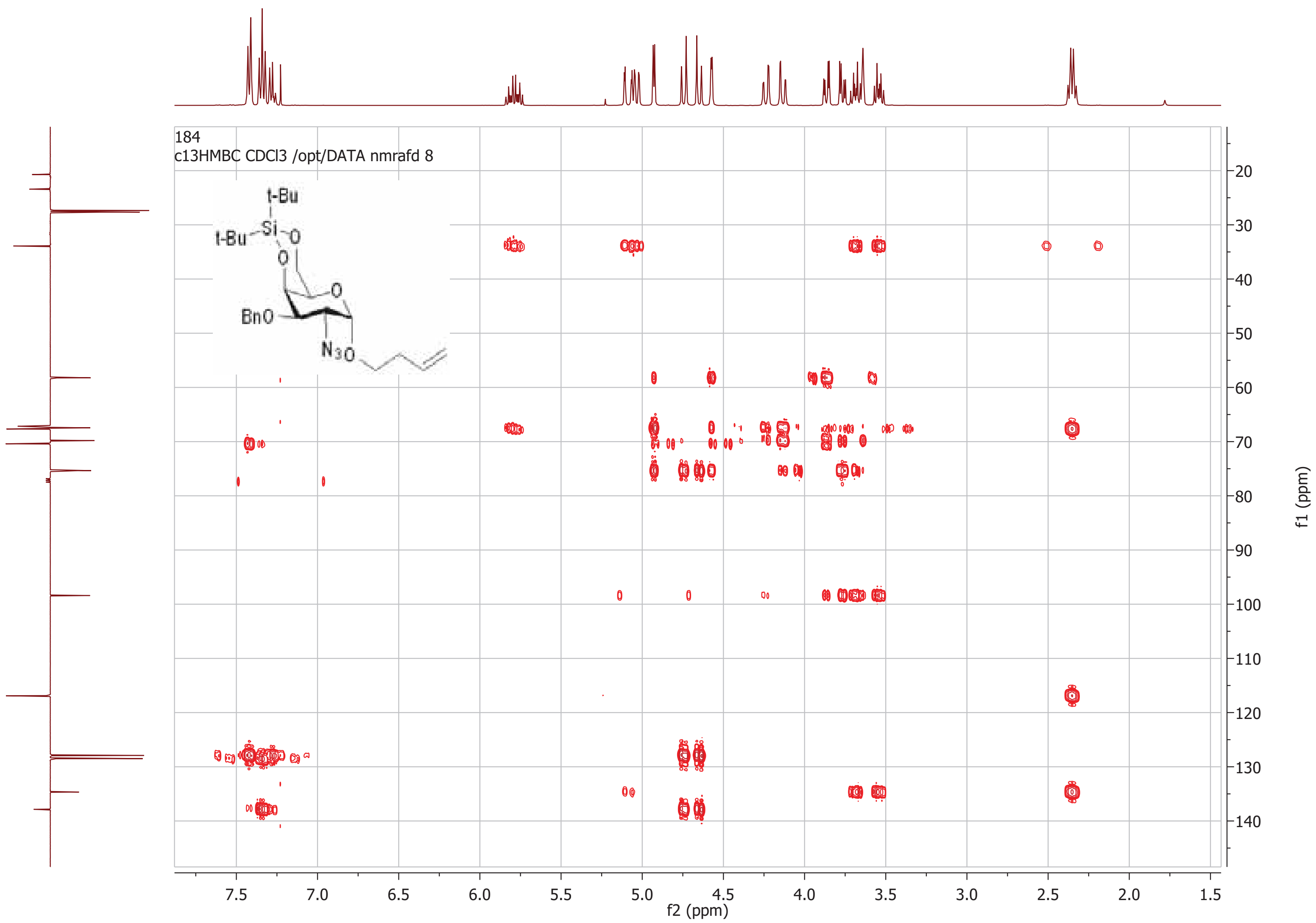

184
c13HMBC CDCl3 /opt/DATA nmrafd 8
t-Bu
t-Bu
Si
O
O
O
BnO
N3O
f2 (ppm)
f1 (ppm)

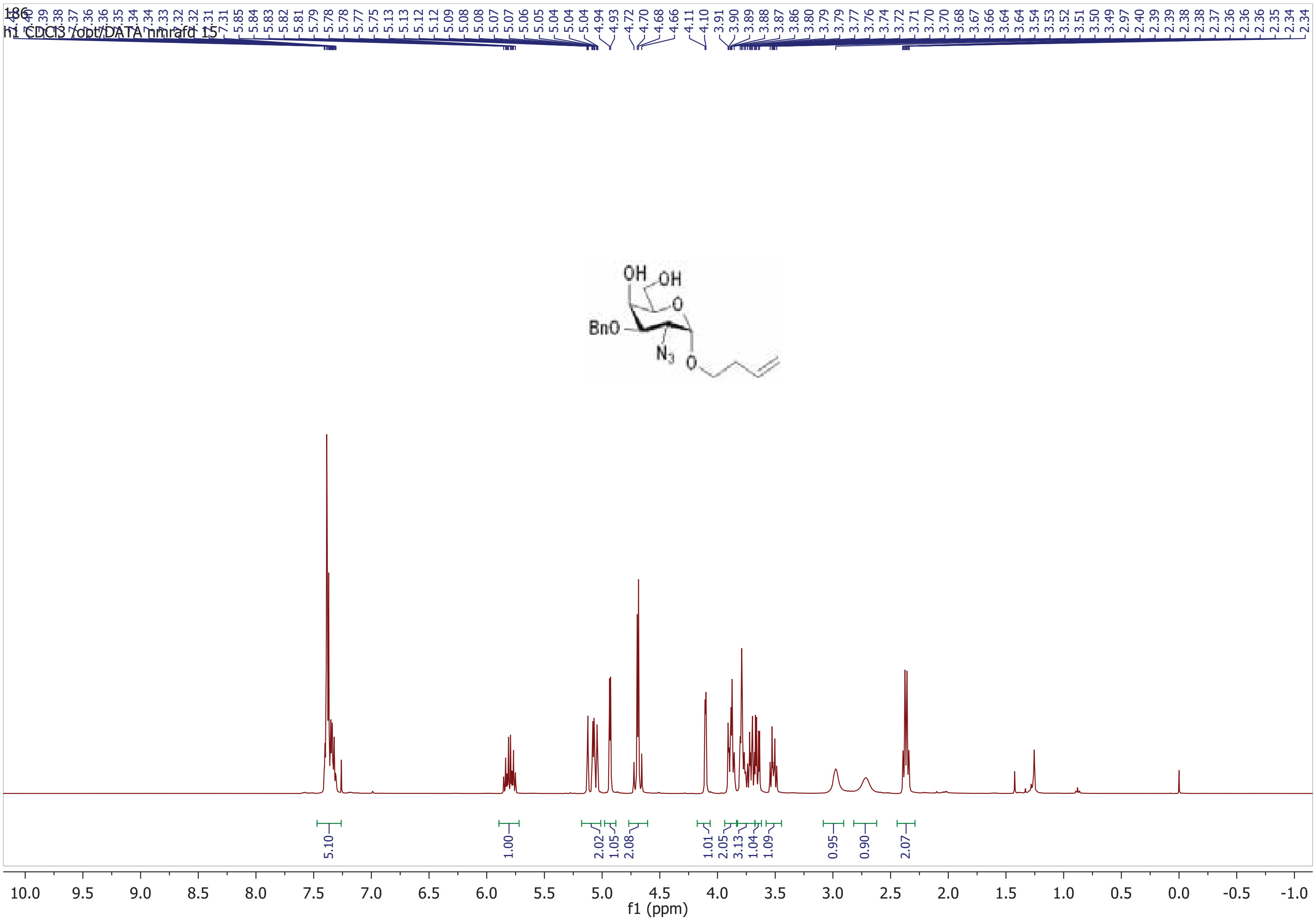
186
h1 CDCl3 /opt/DATA nmrafd 15
7.39 7.38 7.37 7.36 7.36 7.35 7.34 7.34 7.33 7.32 7.32 7.31 5.85 5.84 5.83 5.82 5.81 5.79 5.78 5.78 5.77 5.75 5.13 5.13 5.12 5.12 5.09 5.08 5.08 5.07 5.07 5.06 5.05 5.04 5.04 5.04 4.94 4.93 4.72 4.70 4.68 4.66 4.11 4.10 3.91 3.90 3.89 3.88 3.87 3.86 3.80 3.79 3.79 3.77 3.76 3.74 3.72 3.71 3.70 3.70 3.68 3.67 3.66 3.64 3.54 3.53 3.52 3.51 3.50 3.49 2.97 2.40 2.39 2.39 2.39 2.38 2.38 2.37 2.36 2.36 2.36 2.35 2.34 2.34
OH
OH
O
BnO
N3
O
5.10
1.00
2.02
1.05
2.08
1.01
2.05
3.13
1.04
1.09
0.95
0.90
2.07
10.0 9.5 9.0 8.5 8.0 7.5 7.0 6.5 6.0 5.5 5.0 4.5 4.0 3.5 3.0 2.5 2.0 1.5 1.0 0.5 0.0 -0.5 -1.0
f1 (ppm)

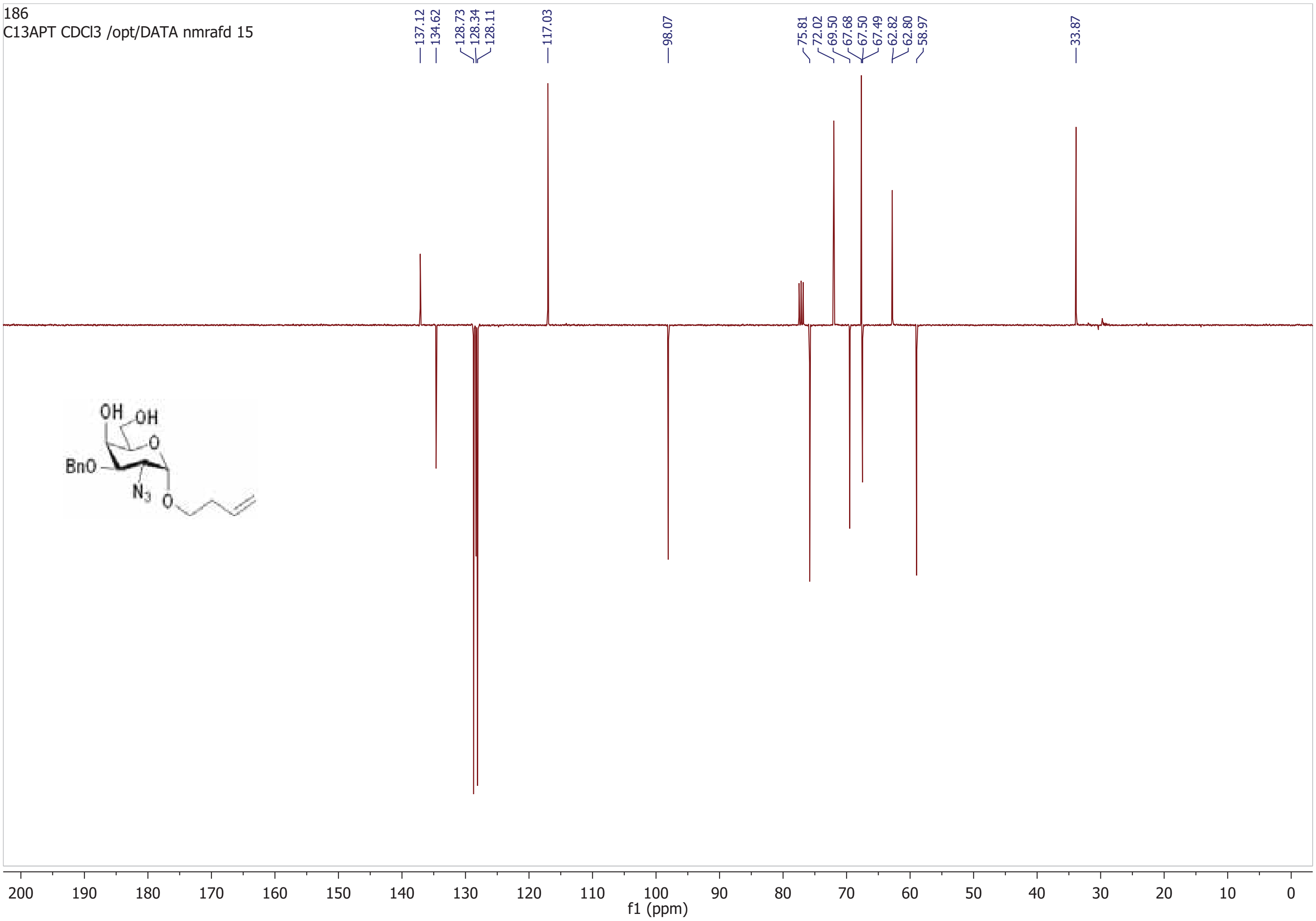
186
C13APT CDCl3 /opt/DATA nmrafd 15
137.12
134.62
128.73
128.34
128.11
117.03
98.07
75.81
72.02
69.50
67.68
67.50
67.49
62.82
62.80
58.97
33.87
OH
OH
O
BnO
N3
O
200 190 180 170 160 150 140 130 120 110 100 90 80 70 60 50 40 30 20 10 0
f1 (ppm)

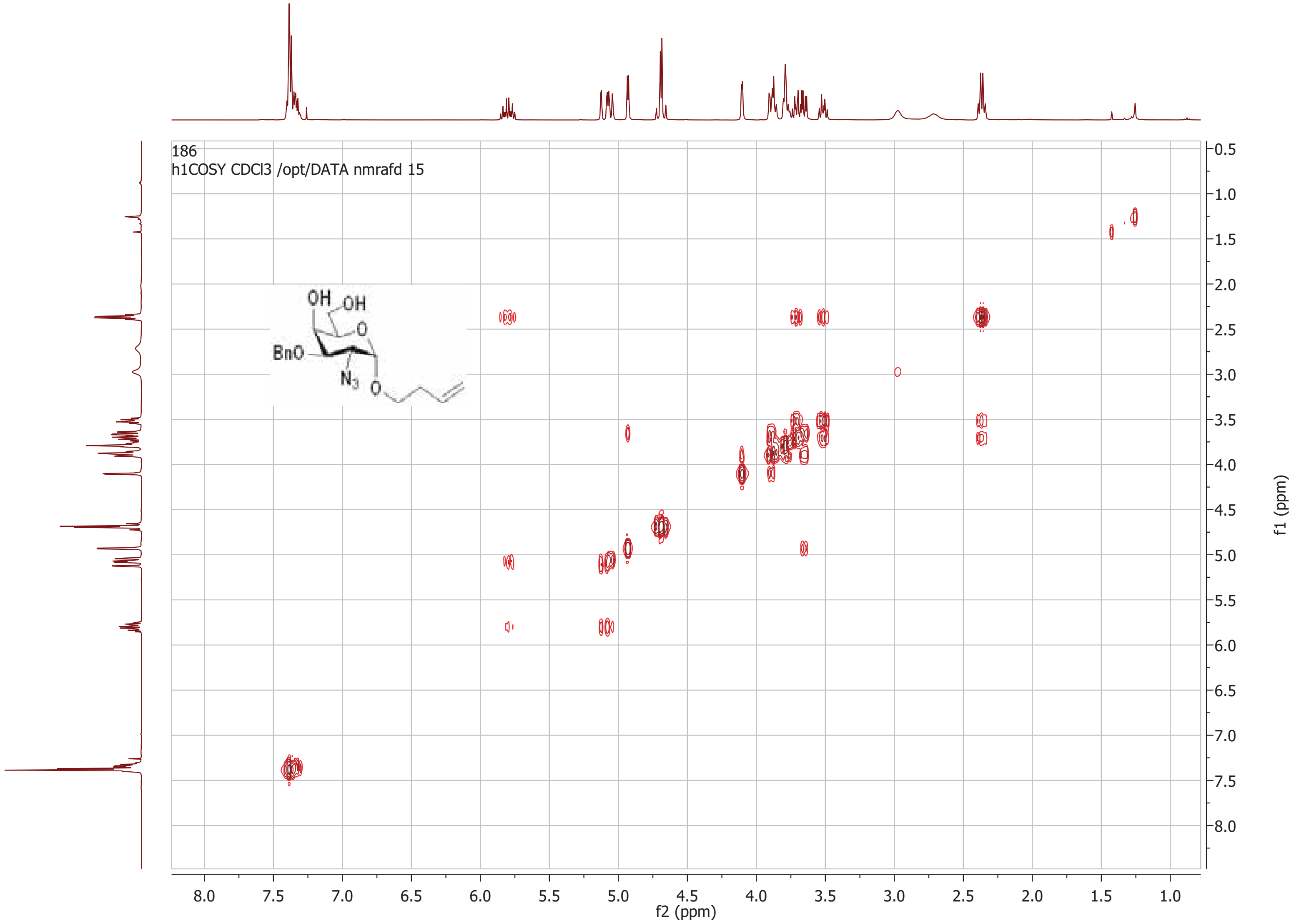
186
h1COSY CDCl3 /opt/DATA nmrafd 15
OH
OH
O
BnO
N3
O
f2 (ppm)
f1 (ppm)

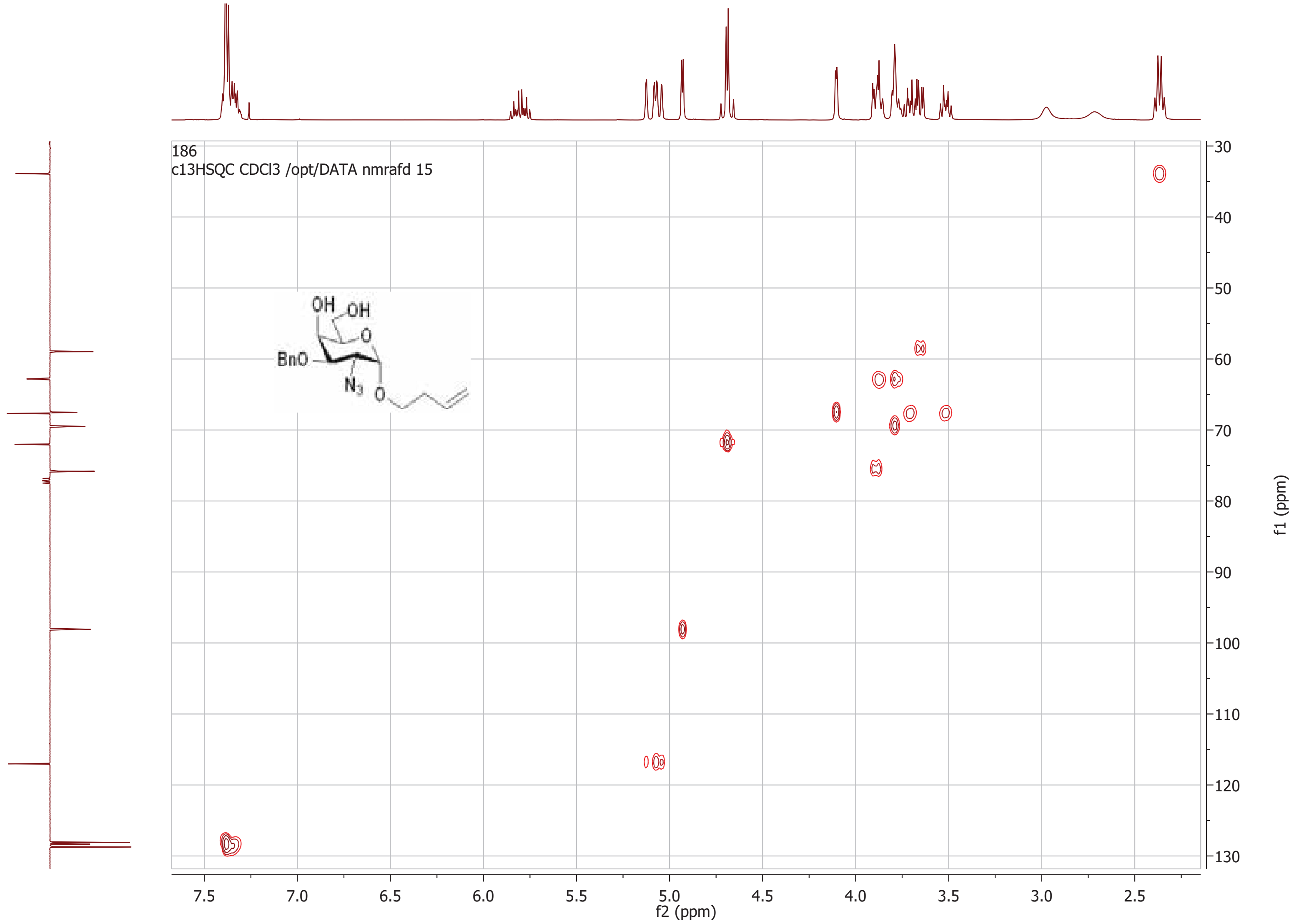
186
c13HSQC CDCl3 /opt/DATA nmrafd 15
OH
OH
O
BnO
N3
O
f2 (ppm)
f1 (ppm)
7.5 7.0 6.5 6.0 5.5 5.0 4.5 4.0 3.5 3.0 2.5
30 40 50 60 70 80 90 100 110 120 130

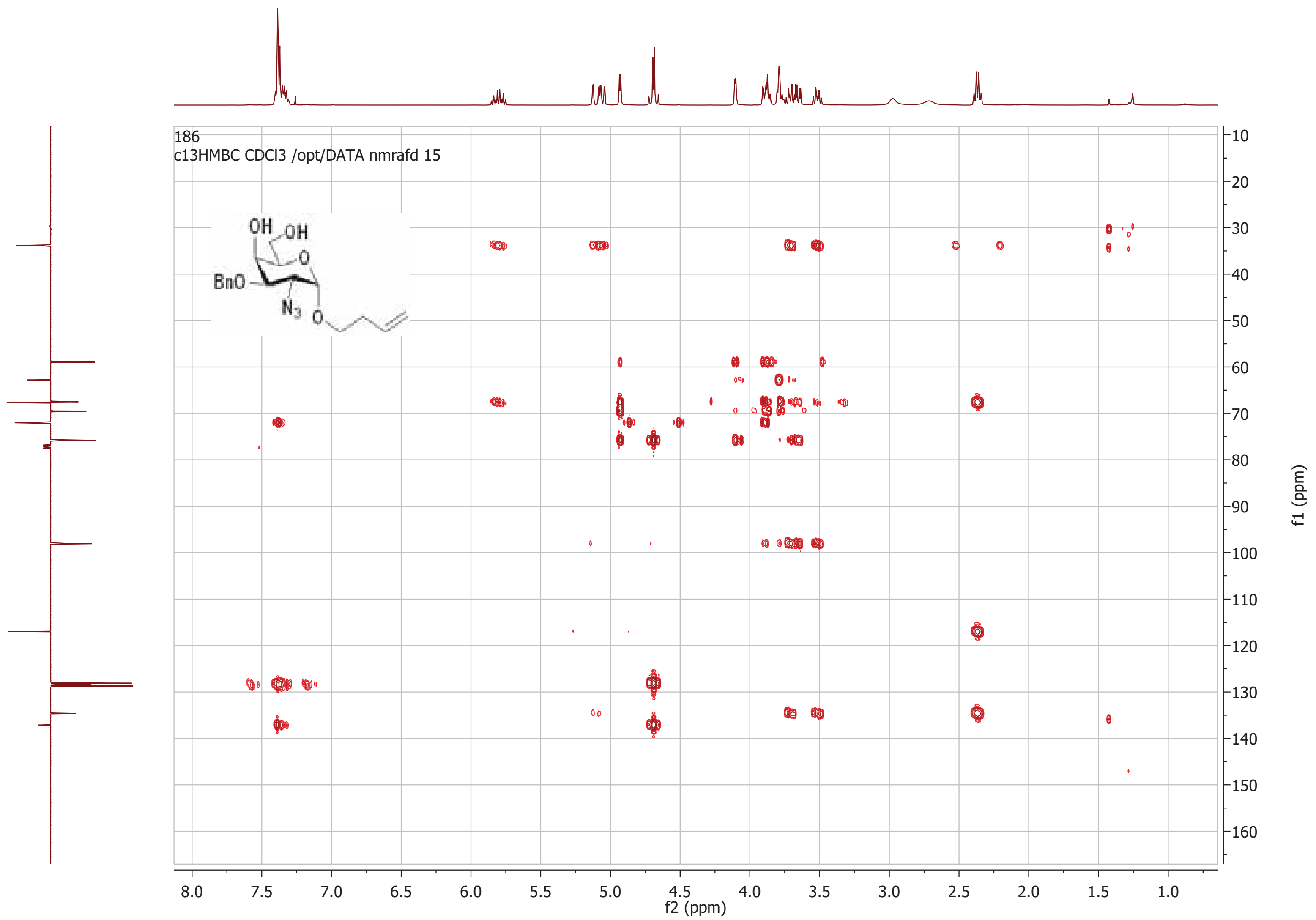
186
c13HMBC CDCl3 /opt/DATA nmrafd 15
OH
OH
O
BnO
N3
O
f2 (ppm)
f1 (ppm)
8.0
7.5
7.0
6.5
6.0
5.5
5.0
4.5
4.0
3.5
3.0
2.5
2.0
1.5
1.0
10
20
30
40
50
60
70
80
90
100
110
120
130
140
150
160

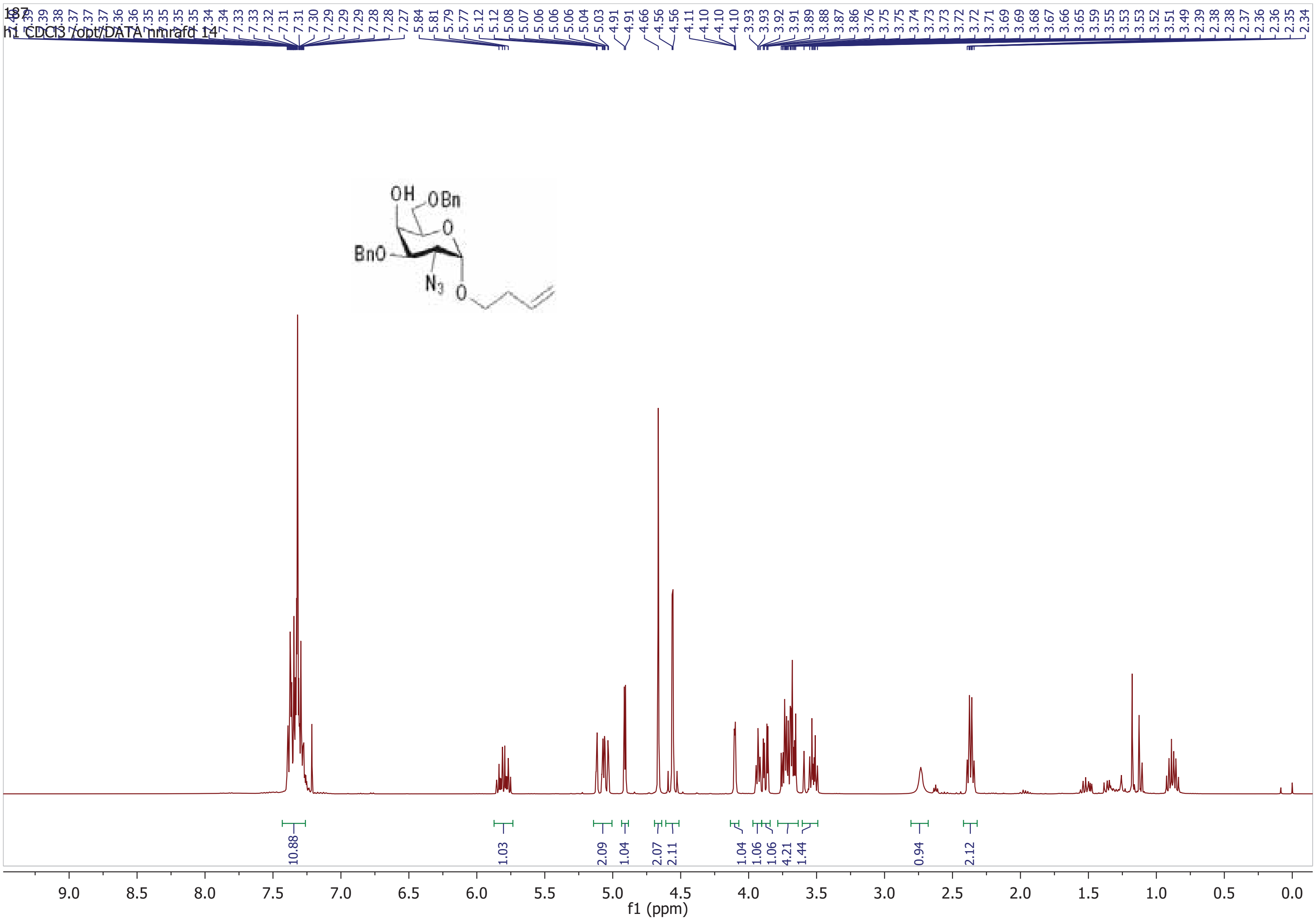
187
h1 CDCl3 /opt/DATA nmrafd 14
7.38 7.37 7.37 7.37 7.36 7.36 7.35 7.35 7.35 7.35 7.34 7.33 7.33 7.32 7.31 7.31 7.30 7.29 7.29 7.29 7.28 7.28 7.27 5.84 5.81 5.79 5.77 5.12 5.12 5.08 5.07 5.06 5.06 5.04 5.03 4.91 4.91 4.66 4.56 4.56 4.11 4.10 4.10 4.10 3.93 3.93 3.92 3.91 3.89 3.88 3.87 3.86 3.76 3.75 3.75 3.74 3.73 3.73 3.72 3.72 3.71 3.69 3.68 3.67 3.66 3.65 3.59 3.55 3.53 3.53 3.52 3.51 3.49 2.39 2.38 2.38 2.37 2.36 2.36 2.35 2.34
OH
OBn
O
BnO
N3
O
10.88
1.03
2.09
1.04
2.07
2.11
1.04
1.06
1.06
4.21
1.44
0.94
2.12
9.0 8.5 8.0 7.5 7.0 6.5 6.0 5.5 5.0 4.5 4.0 3.5 3.0 2.5 2.0 1.5 1.0 0.5 0.0
f1 (ppm)

187
C13APT CDCl3 /opt/DATA nmrafd 14

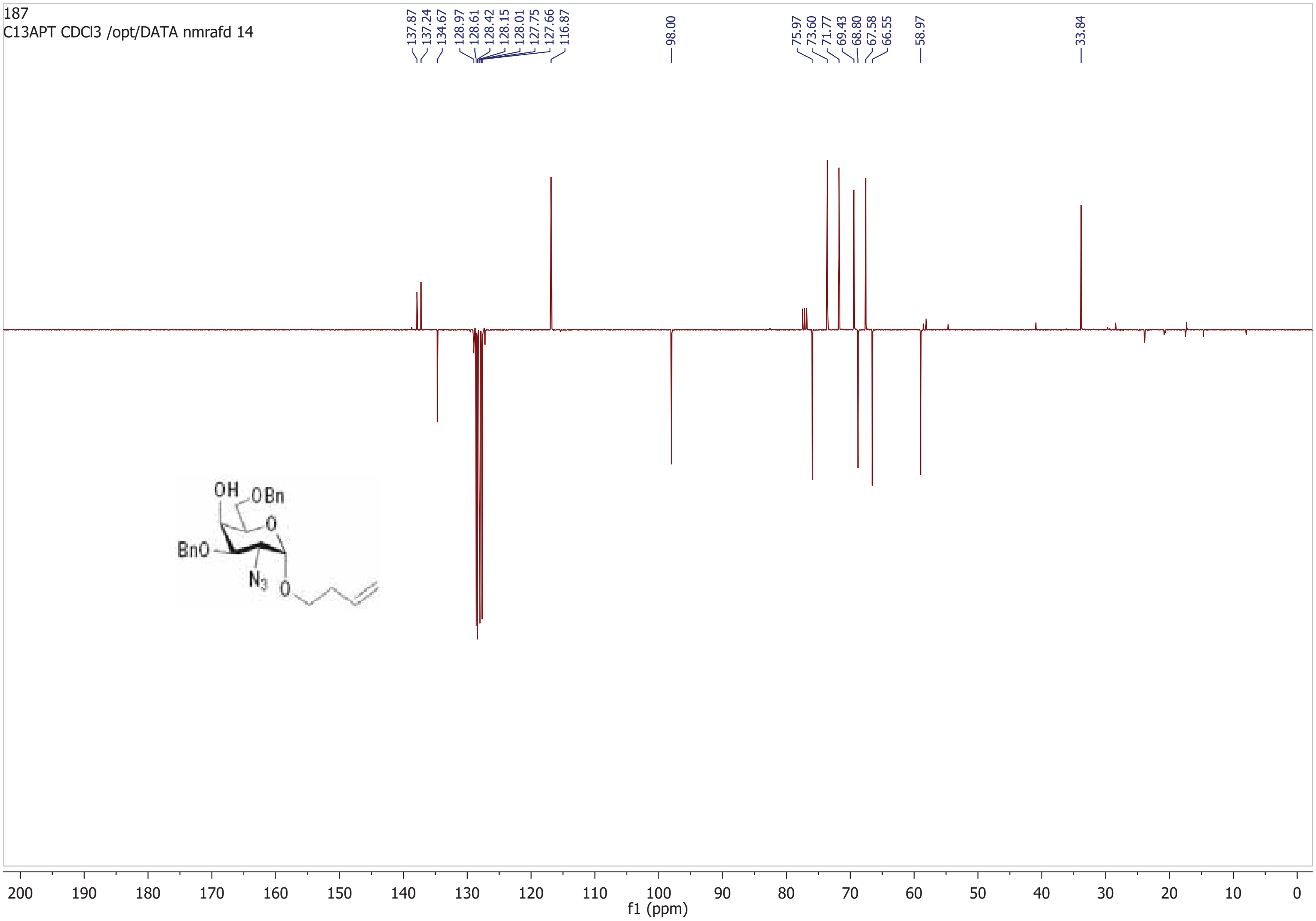

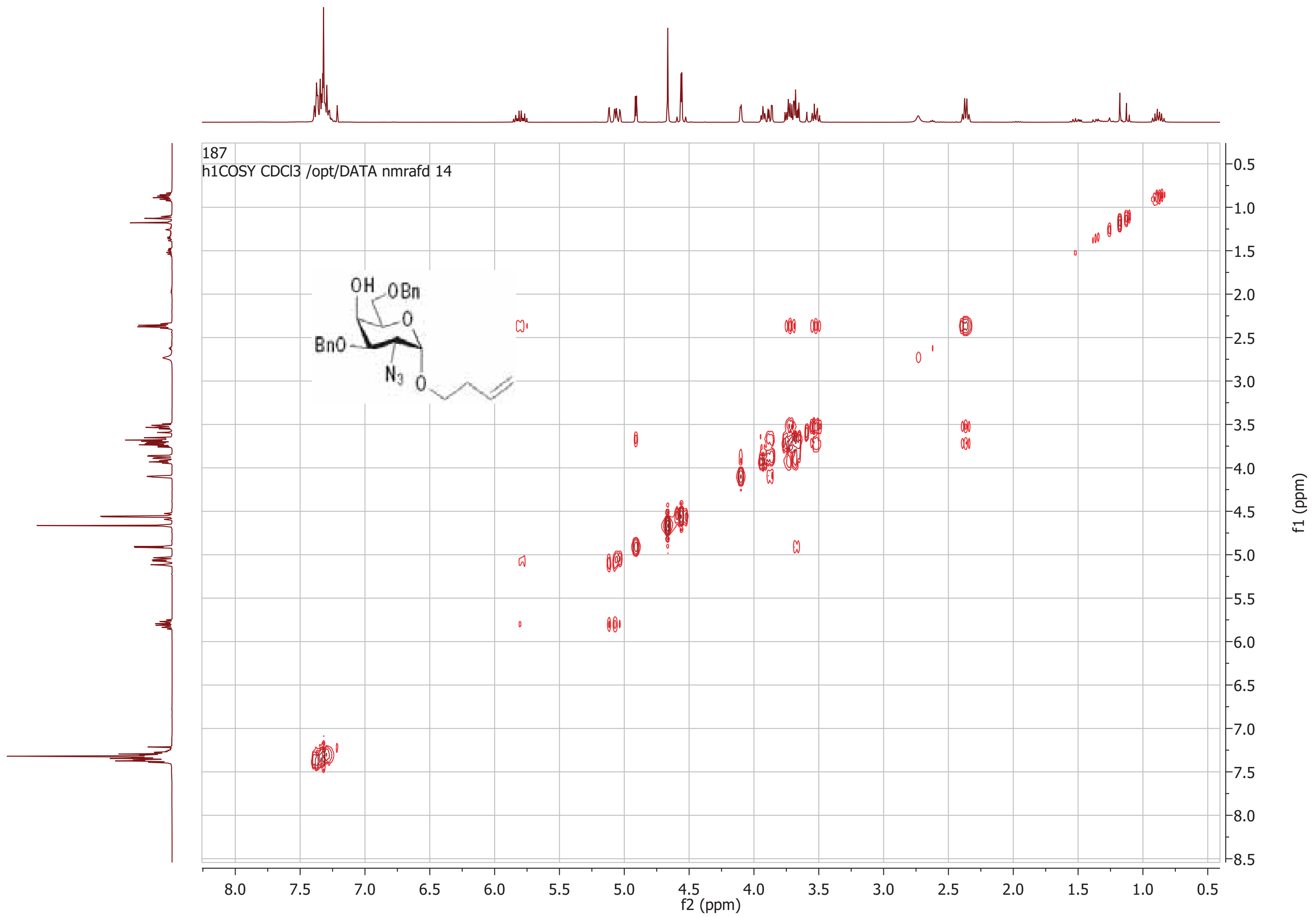
187
h1COSY CDCl3 /opt/DATA nmrafd 14
OH
OBn
O
BnO
N3
O
f2 (ppm)
f1 (ppm)

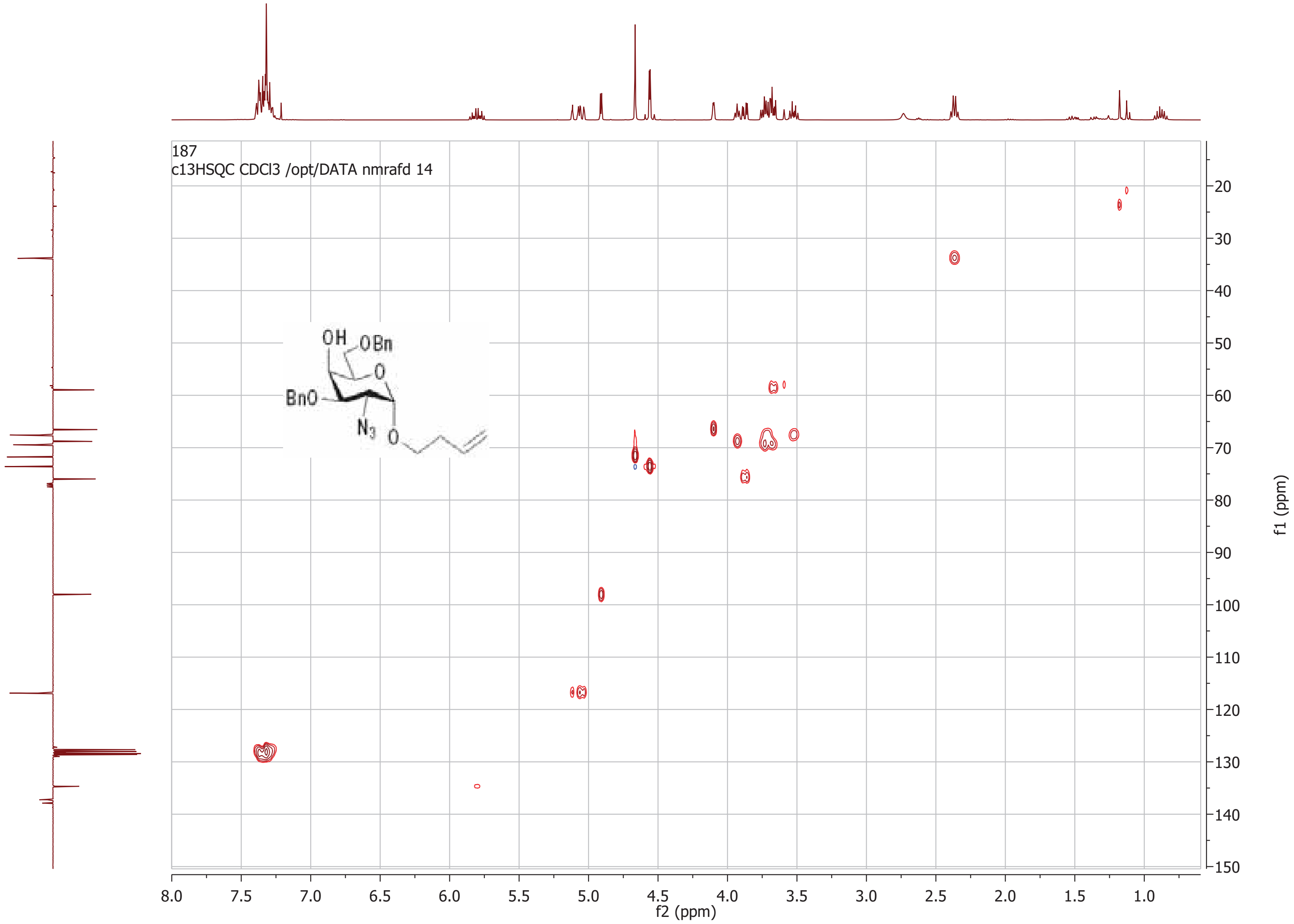
187
c13HSQC CDCl3 /opt/DATA nmrafd 14
OH
OBn
O
BnO
N3
O
f2 (ppm)
f1 (ppm)
8.0
7.5
7.0
6.5
6.0
5.5
5.0
4.5
4.0
3.5
3.0
2.5
2.0
1.5
1.0
20
30
40
50
60
70
80
90
100
110
120
130
140
150

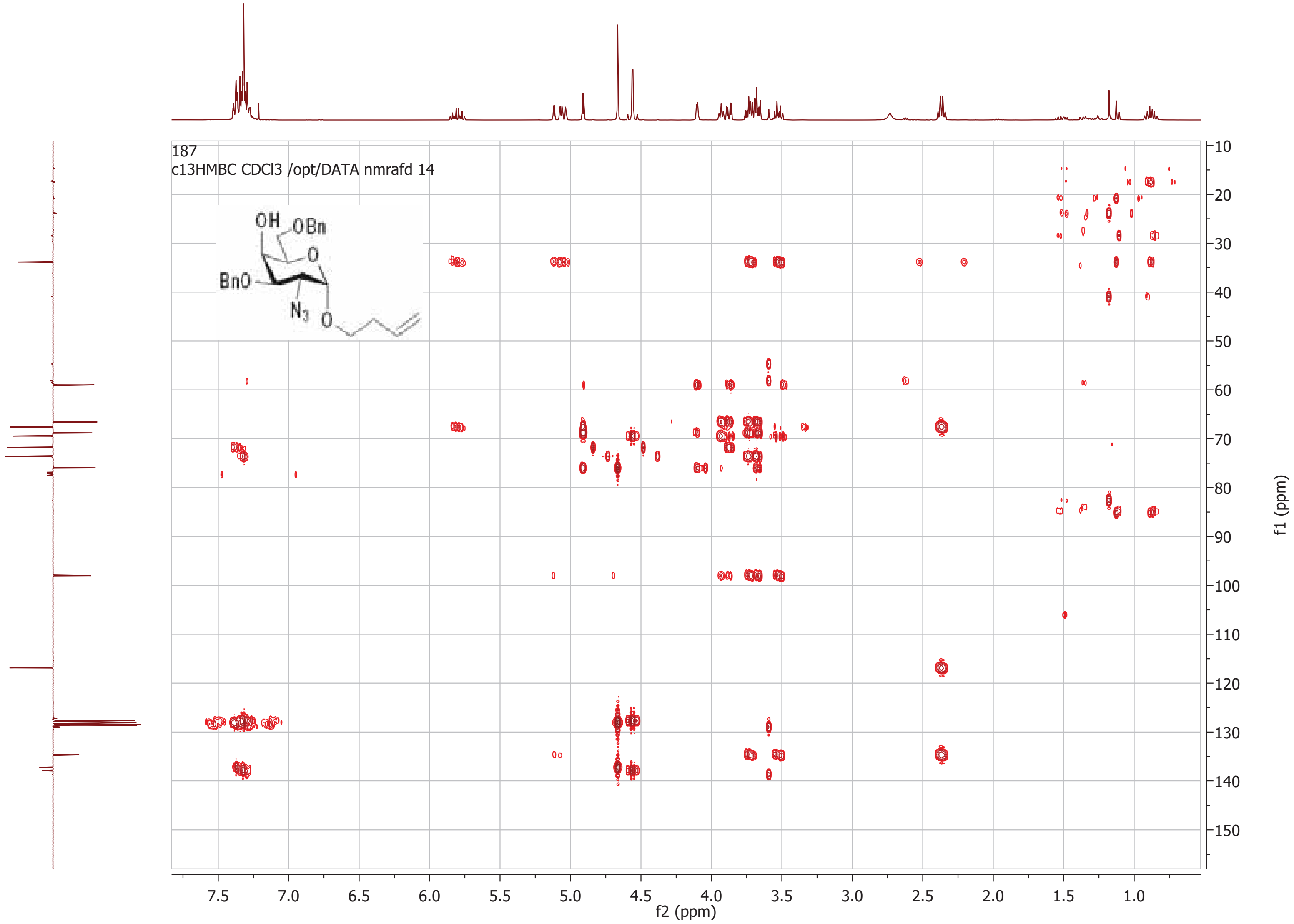
187
c13HMBC CDCl3 /opt/DATA nmrafd 14
OH
OBn
O
BnO
N3
O
f2 (ppm)
f1 (ppm)

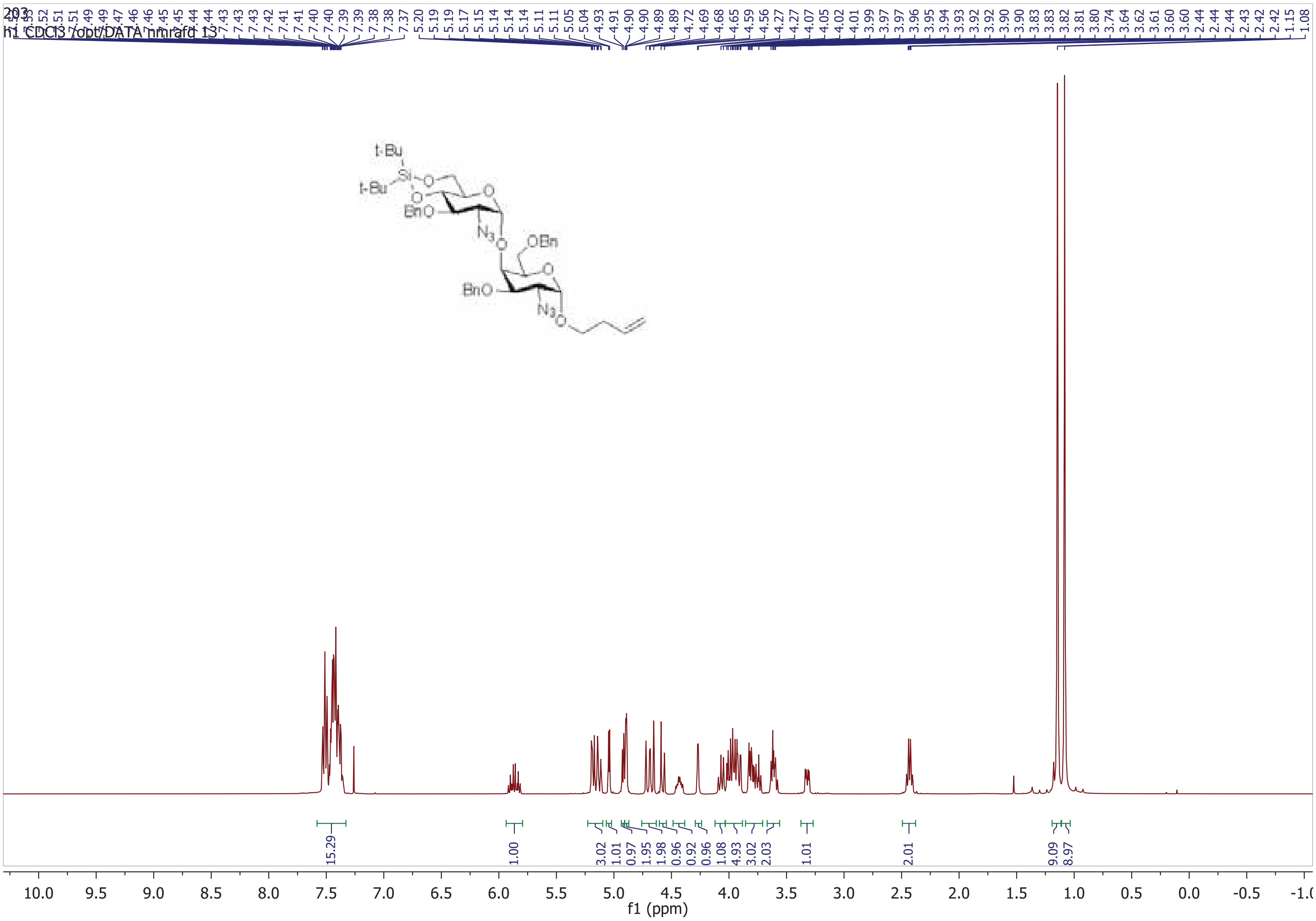
203
h1 CDCl3 /opt/DATA nmrafd 13
7.52
7.51
7.51
7.49
7.47
7.46
7.46
7.45
7.45
7.44
7.44
7.43
7.43
7.43
7.42
7.41
7.41
7.40
7.40
7.39
7.39
7.38
7.38
7.37
5.20
5.19
5.19
5.17
5.15
5.14
5.14
5.14
5.11
5.11
5.05
5.04
4.93
4.91
4.90
4.90
4.89
4.89
4.72
4.69
4.68
4.65
4.59
4.56
4.27
4.27
4.07
4.05
4.02
4.01
3.99
3.97
3.97
3.96
3.95
3.94
3.93
3.92
3.92
3.90
3.90
3.83
3.83
3.82
3.81
3.80
3.74
3.64
3.62
3.61
3.60
2.44
2.44
2.43
2.42
2.42
1.15
1.08
t-Bu
Si
t-Bu
O
O
BnO
O
N3
O
OBn
O
BnO
N3
O
15.29
1.00
3.02
1.01
0.97
1.95
1.98
0.96
0.92
0.96
1.08
4.93
3.02
2.03
1.01
2.01
9.09
8.97
10.0
9.5
9.0
8.5
8.0
7.5
7.0
6.5
6.0
5.5
5.0
4.5
4.0
3.5
3.0
2.5
2.0
1.5
1.0
0.5
0.0
-0.5
f1 (ppm)

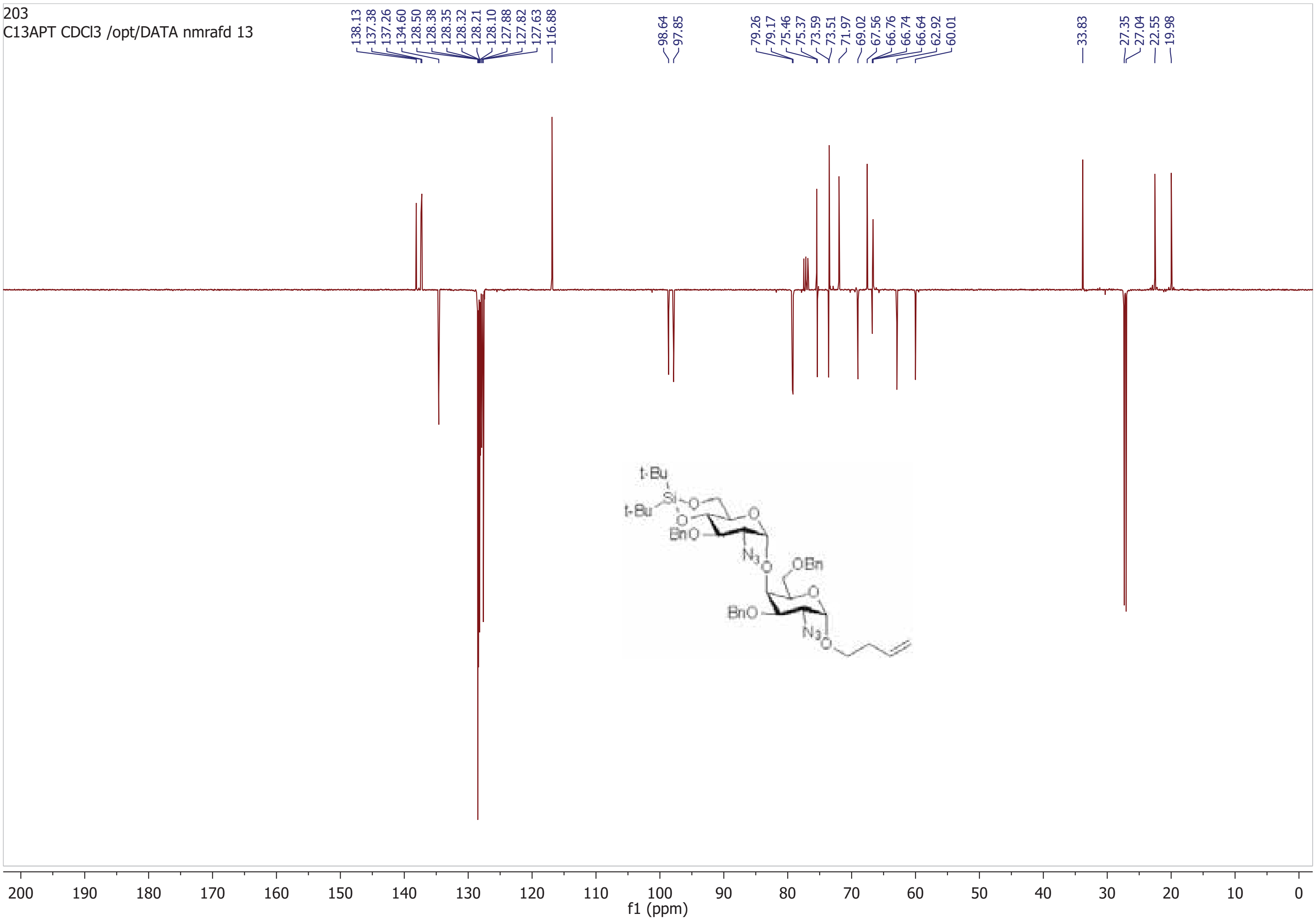
203
C13APT CDCl3 /opt/DATA nmrafd 13
138.13
137.38
137.26
134.60
128.50
128.38
128.35
128.32
128.21
128.10
127.88
127.82
127.63
116.88
98.64
97.85
79.26
79.17
75.46
75.37
73.59
73.51
71.97
69.02
67.56
66.76
66.74
66.64
62.92
60.01
33.83
27.35
27.04
22.55
19.98
t-Bu
Si
t-Bu
O
O
O
BnO
N3
O
OBn
O
BnO
N3
O
200 190 180 170 160 150 140 130 120 110 100 90 80 70 60 50 40 30 20 10 0
f1 (ppm)

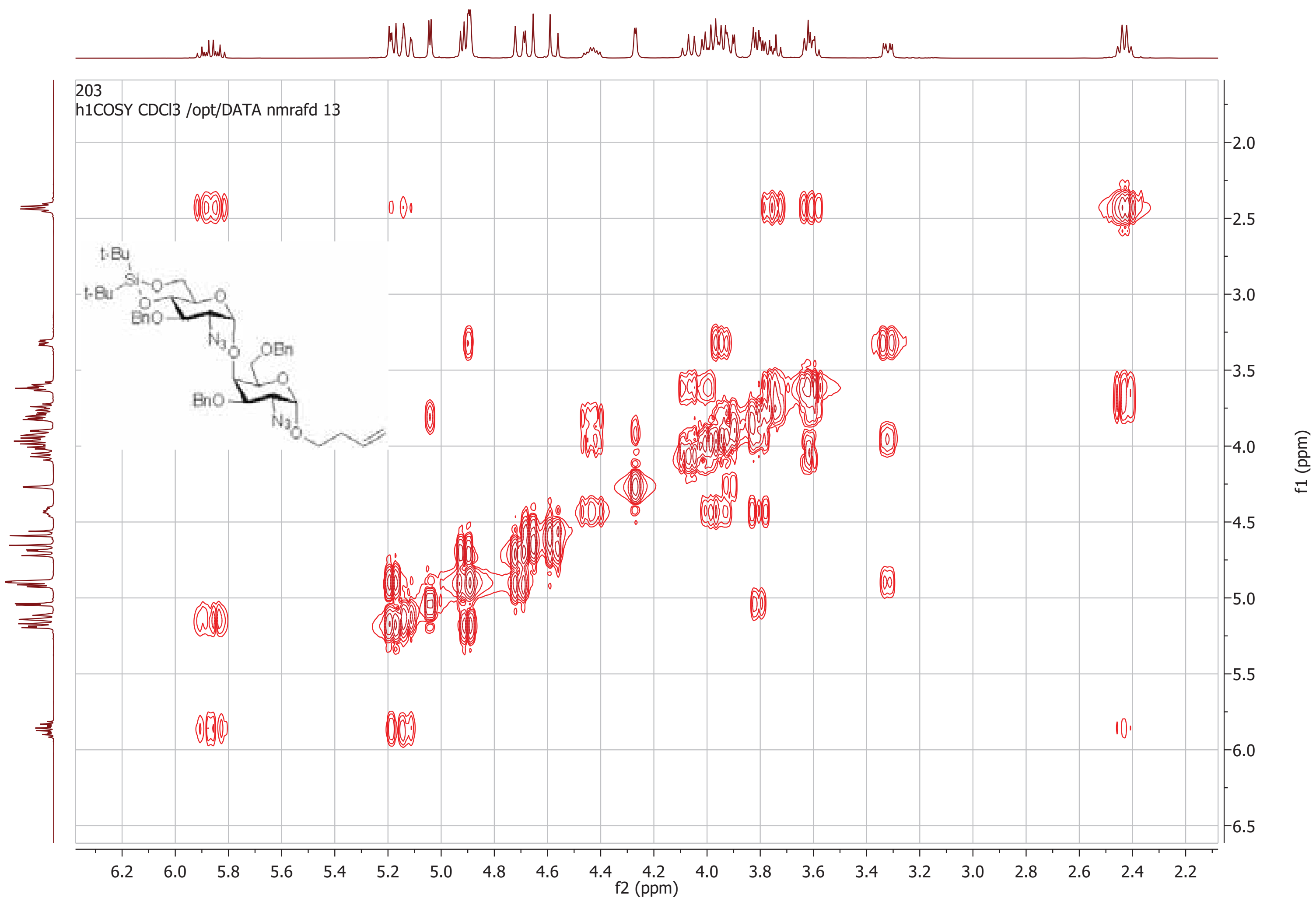

203
h1COSY CDCl3 /opt/DATA nmrafd 13
t-Bu
t-Bu
Si
O
O
BnO
N3
O
OBn
BnO
N3
O
O
f2 (ppm)
f1 (ppm)

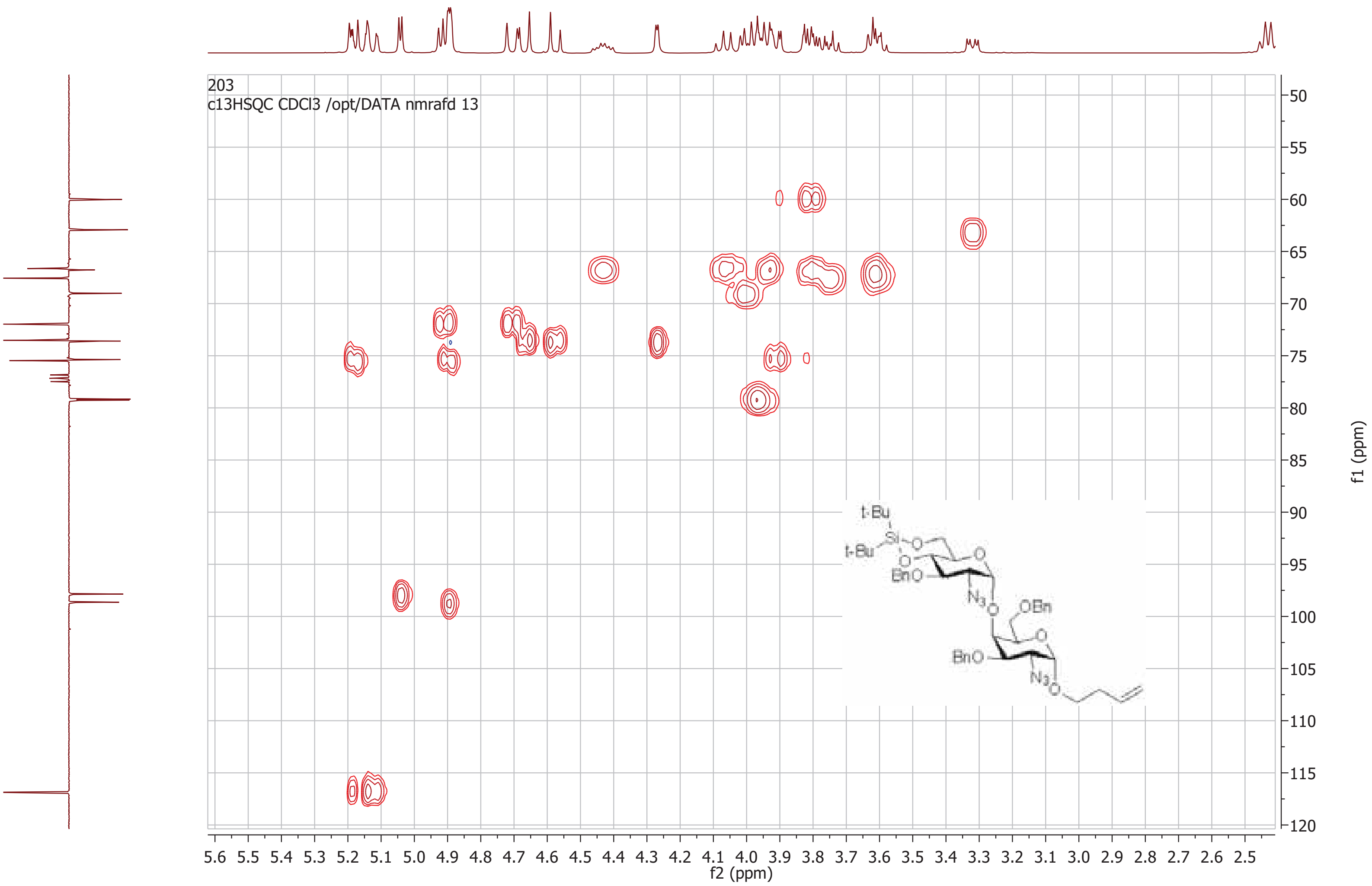
203
c13HSQC CDCl3 /opt/DATA nmrafd 13
t-Bu
Si
t-Bu
BnO
N3
OBn
BnO
N3
f2 (ppm)
f1 (ppm)

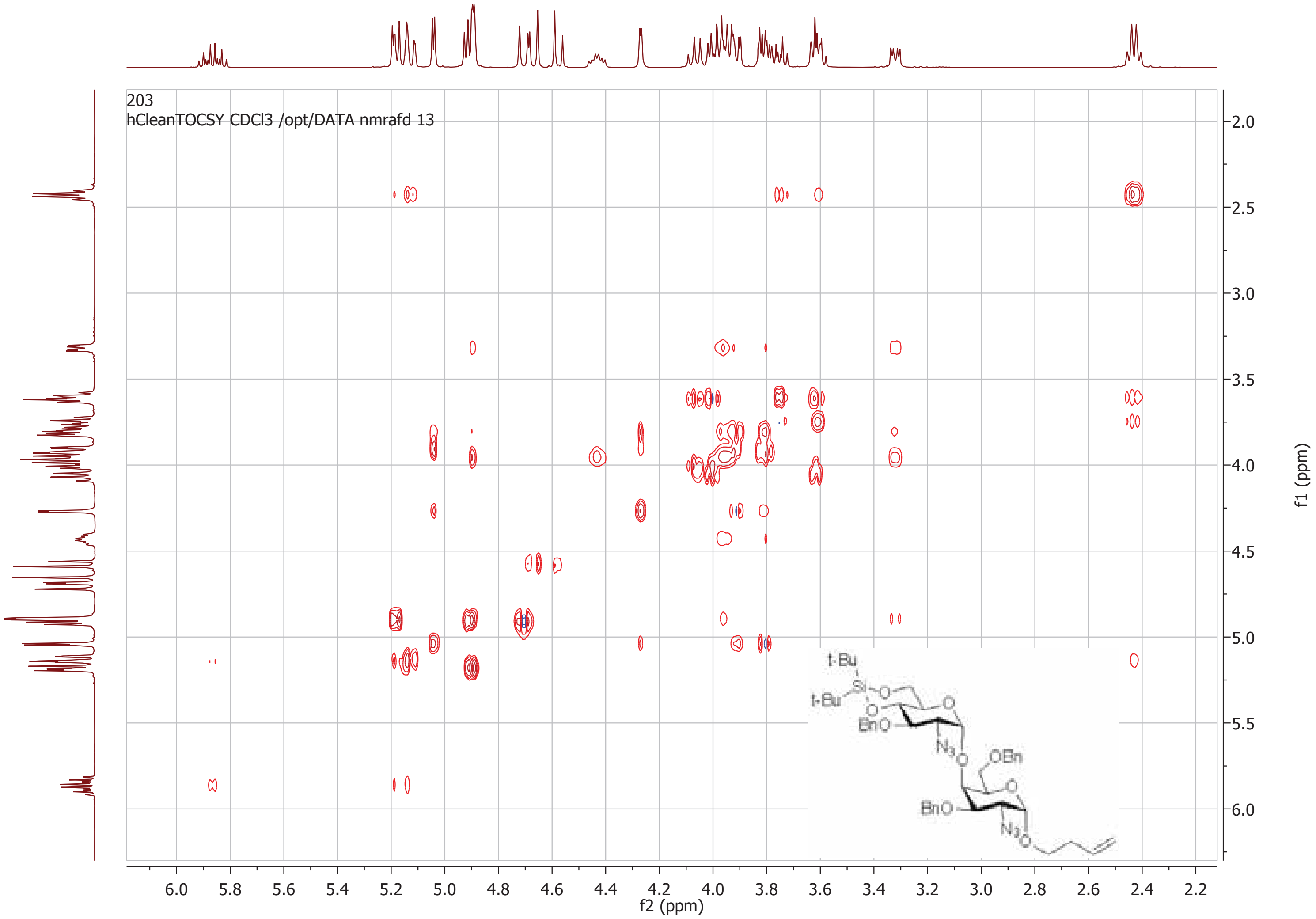
203
hCleanTOCSY CDCl3 /opt/DATA nmrafd 13
f2 (ppm)
f1 (ppm)
t-Bu
Si
t-Bu
O
BnO
N3
OBn
BnO
N3

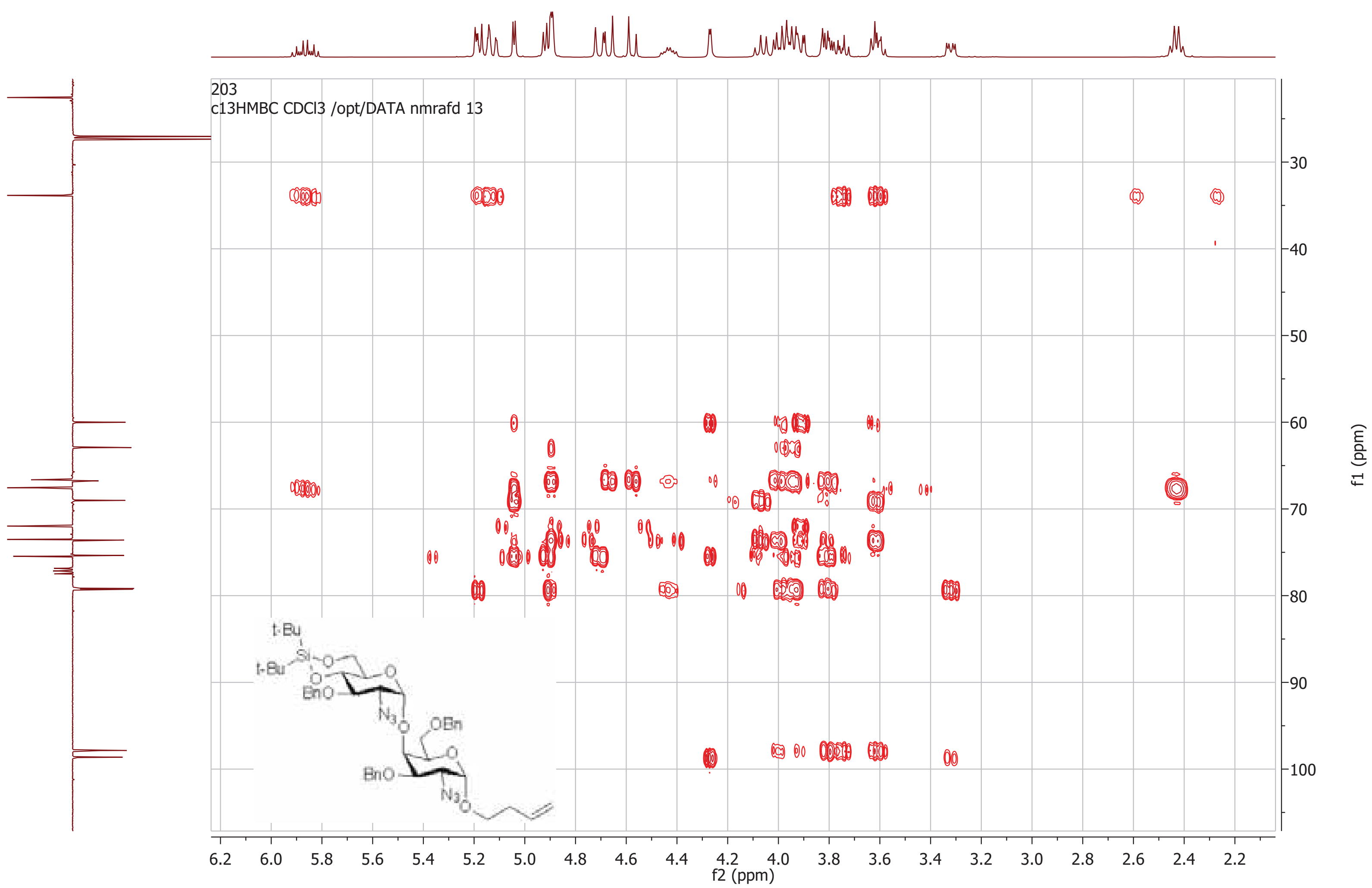

203
c13HMBC CDCl3 /opt/DATA nmrafd 13
f2 (ppm)
f1 (ppm)
t-Bu
Si
t-Bu
BnO
N3
OBn
BnO
N3

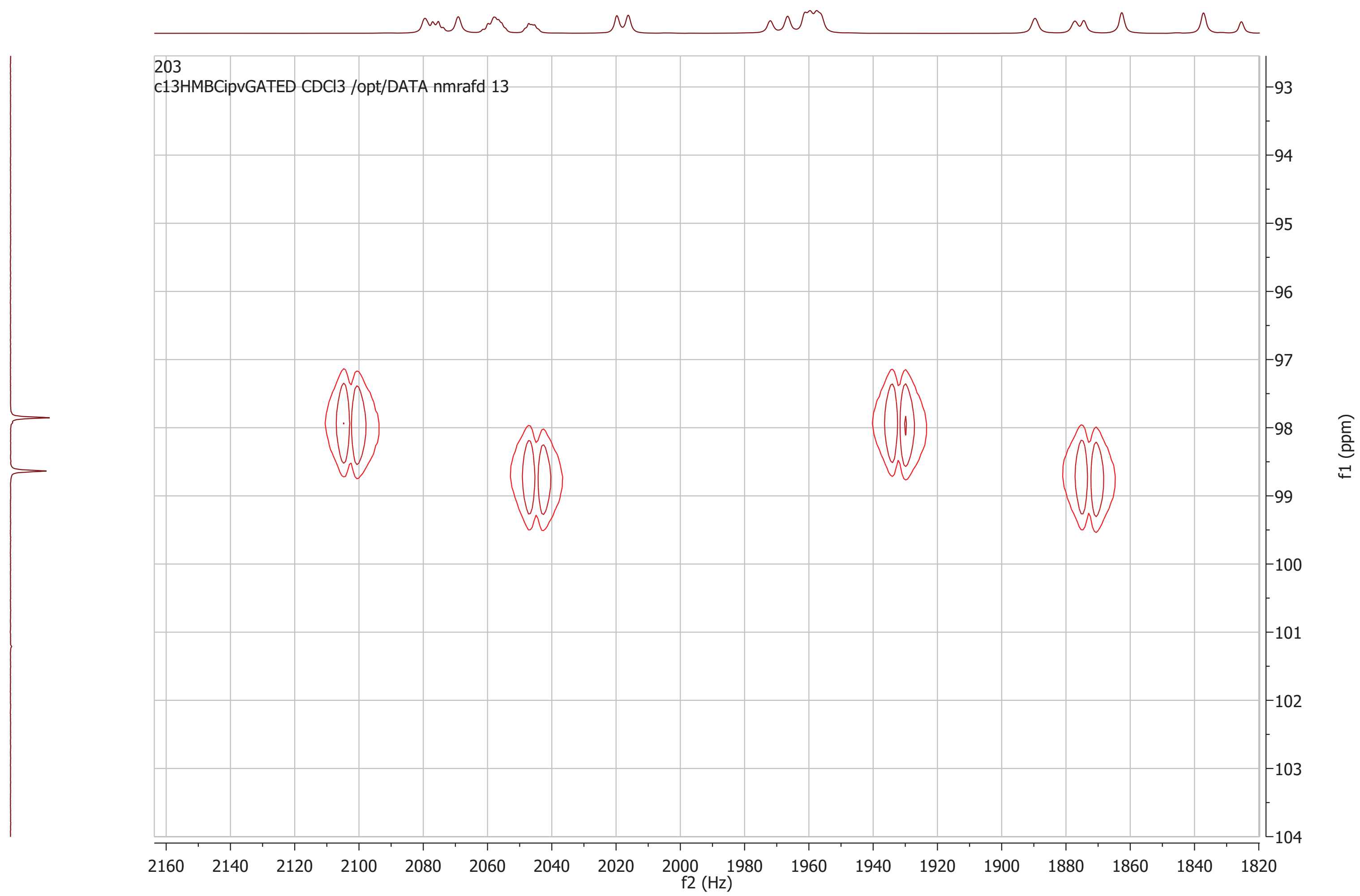
203
c13HMBCipvGATED CDCl3 /opt/DATA nmrafd 13
f2 (Hz)
f1 (ppm)

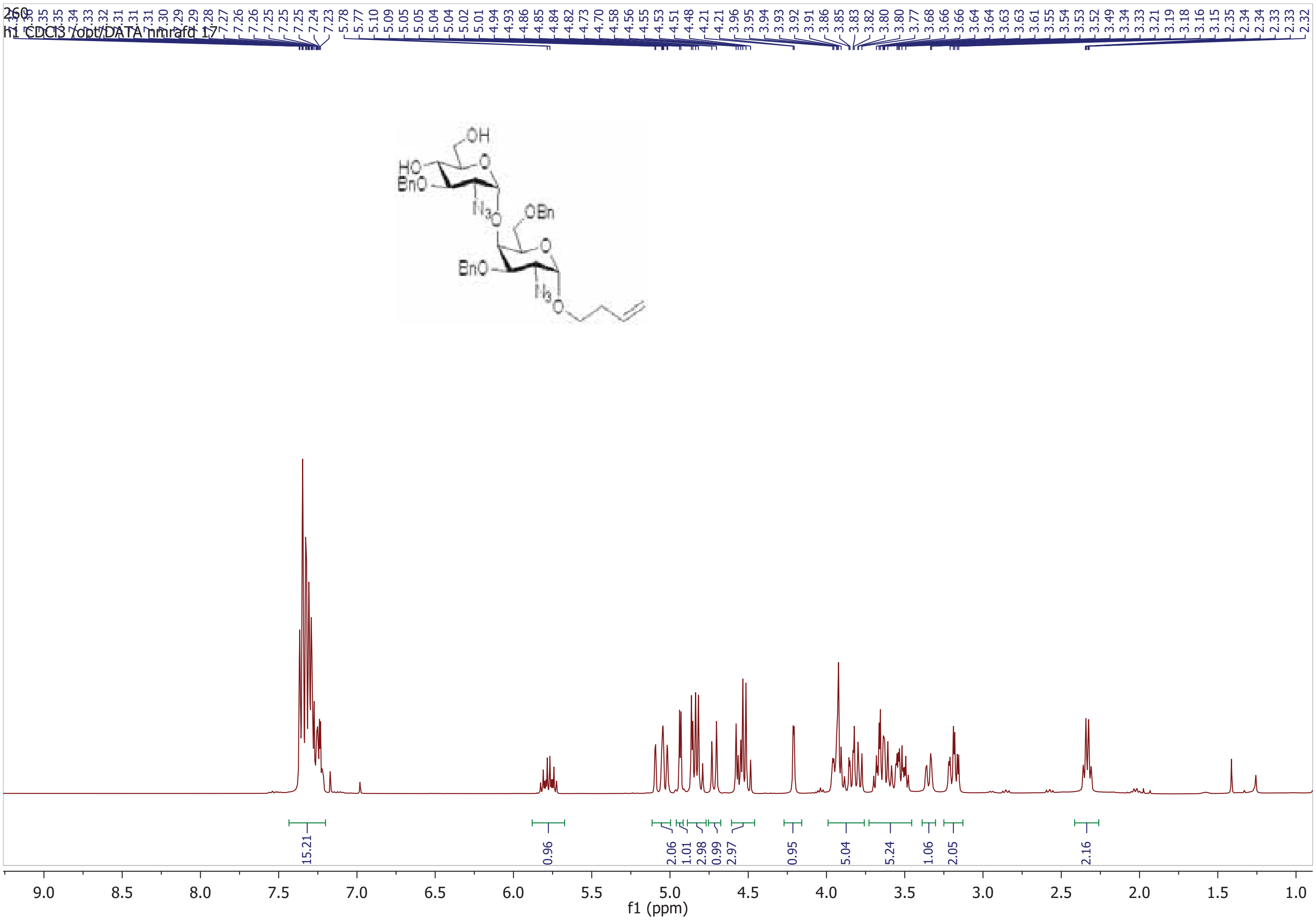
260
h1 CDCl3 /opt/DATA nmrafd 17
7.35 7.35 7.34 7.33 7.32 7.31 7.31 7.30 7.29 7.28 7.27 7.26 7.26 7.25 7.25 7.24 7.23 5.78 5.77 5.10 5.09 5.05 5.04 5.04 5.02 5.01 4.94 4.93 4.86 4.85 4.84 4.82 4.73 4.70 4.58 4.56 4.55 4.53 4.51 4.48 4.21 4.21 3.96 3.95 3.94 3.93 3.92 3.91 3.86 3.85 3.83 3.82 3.80 3.80 3.77 3.68 3.66 3.66 3.64 3.64 3.63 3.63 3.61 3.55 3.54 3.53 3.52 3.49 3.34 3.33 3.21 3.19 3.18 3.16 3.15 2.35 2.34 2.34 2.33 2.33 2.32
OH
HO
BnO
N3
OBn
BnO
N3
15.21
0.96
2.06
1.01
2.98
0.99
2.97
0.95
5.04
5.24
1.06
2.05
2.16
9.0 8.5 8.0 7.5 7.0 6.5 6.0 5.5 5.0 4.5 4.0 3.5 3.0 2.5 2.0 1.5 1.0
f1 (ppm)

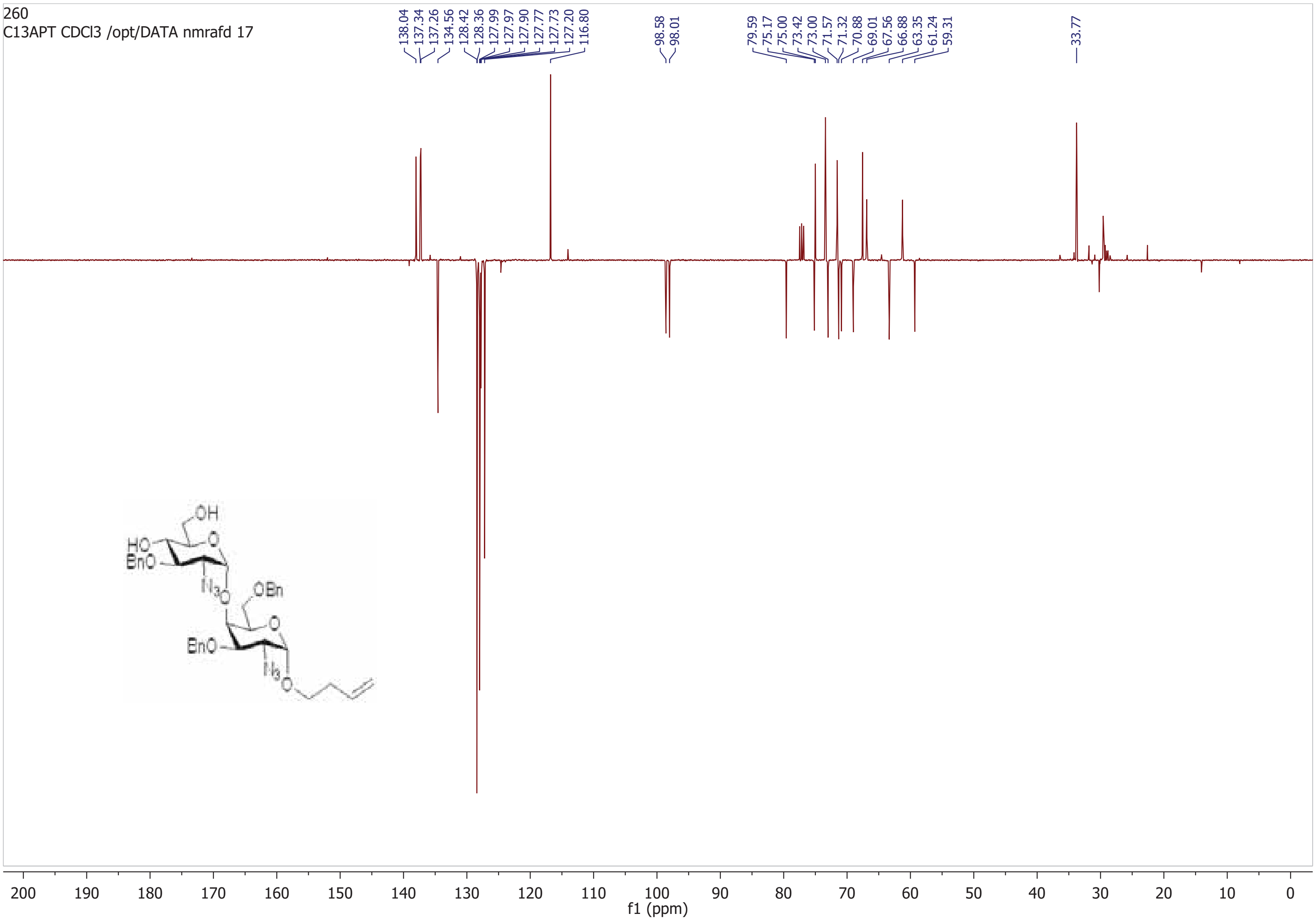
260
C13APT CDCl3 /opt/DATA nmrafd 17
138.04
137.34
137.26
134.56
128.42
128.36
127.99
127.97
127.90
127.77
127.73
127.20
116.80
98.58
98.01
79.59
75.17
75.00
73.42
73.00
71.57
71.32
70.88
69.01
67.56
66.88
63.35
61.24
59.31
33.77
OH
HO
BnO
N3
O
OBn
BnO
N3
200 190 180 170 160 150 140 130 120 110 100 90 80 70 60 50 40 30 20 10 0
f1 (ppm)

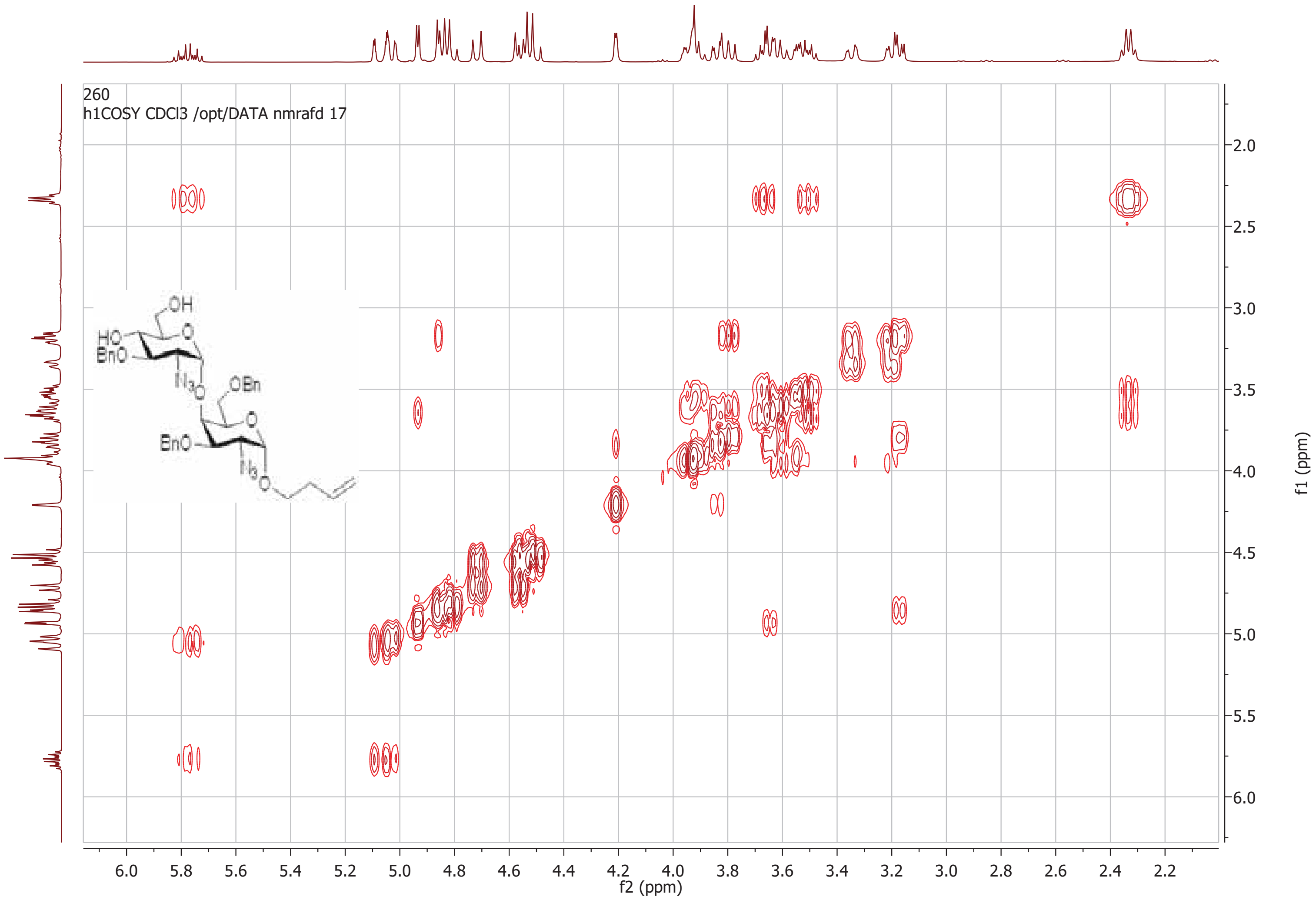
260
h1COSY CDCl3 /opt/DATA nmrafd 17
OH
HO
BnO
N3
OBn
BnO
N3
6.0 5.8 5.6 5.4 5.2 5.0 4.8 4.6 4.4 4.2 4.0 3.8 3.6 3.4 3.2 3.0 2.8 2.6 2.4 2.2
f2 (ppm)
2.0 2.5 3.0 3.5 4.0 4.5 5.0 5.5 6.0
f1 (ppm)

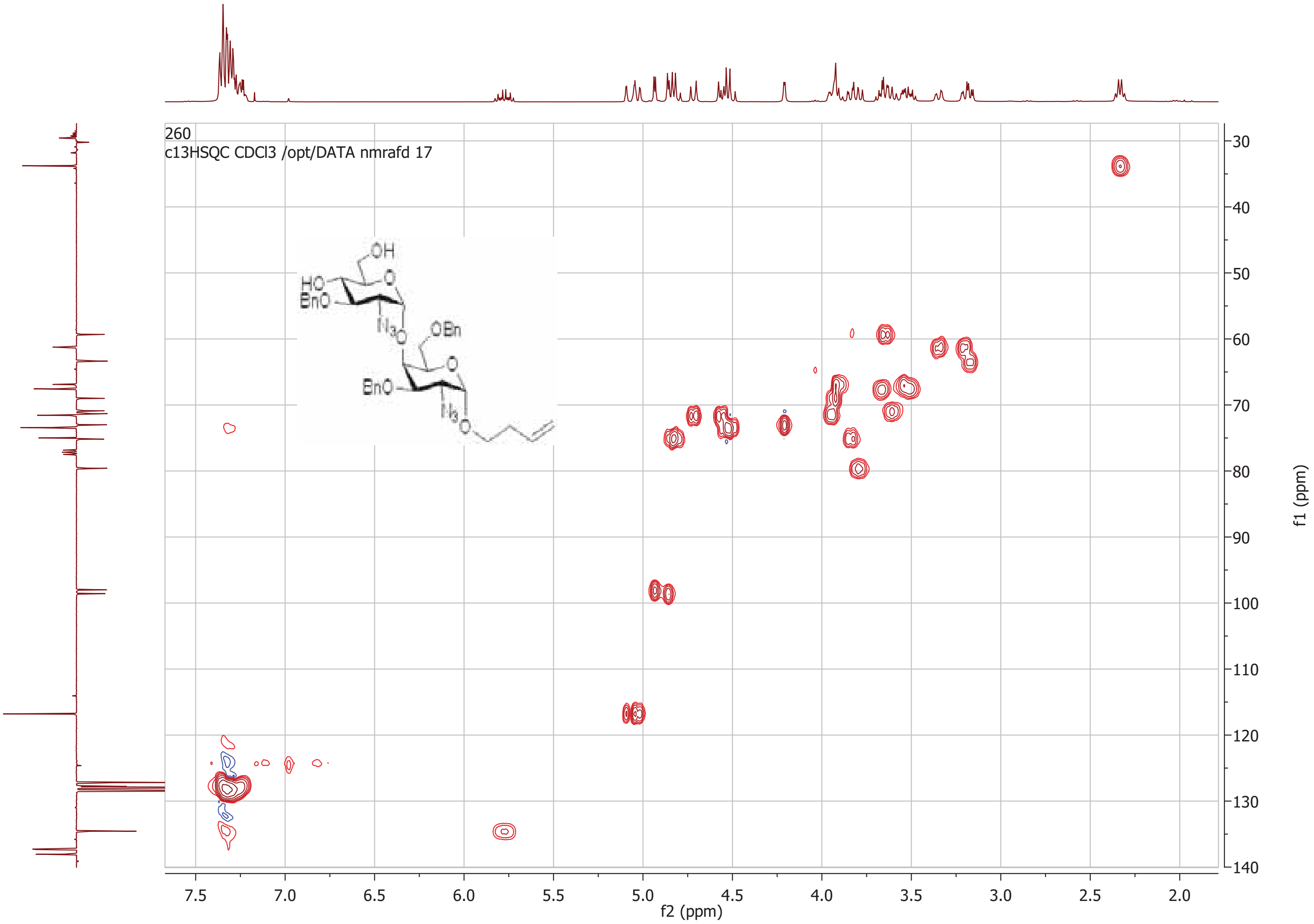
260
c13HSQC CDCl3 /opt/DATA nmrafd 17
OH
HO
BnO
N3
OBn
BnO
N3
f2 (ppm)
f1 (ppm)
7.5
7.0
6.5
6.0
5.5
5.0
4.5
4.0
3.5
3.0
2.5
2.0
30
40
50
60
70
80
90
100
110
120
130
140

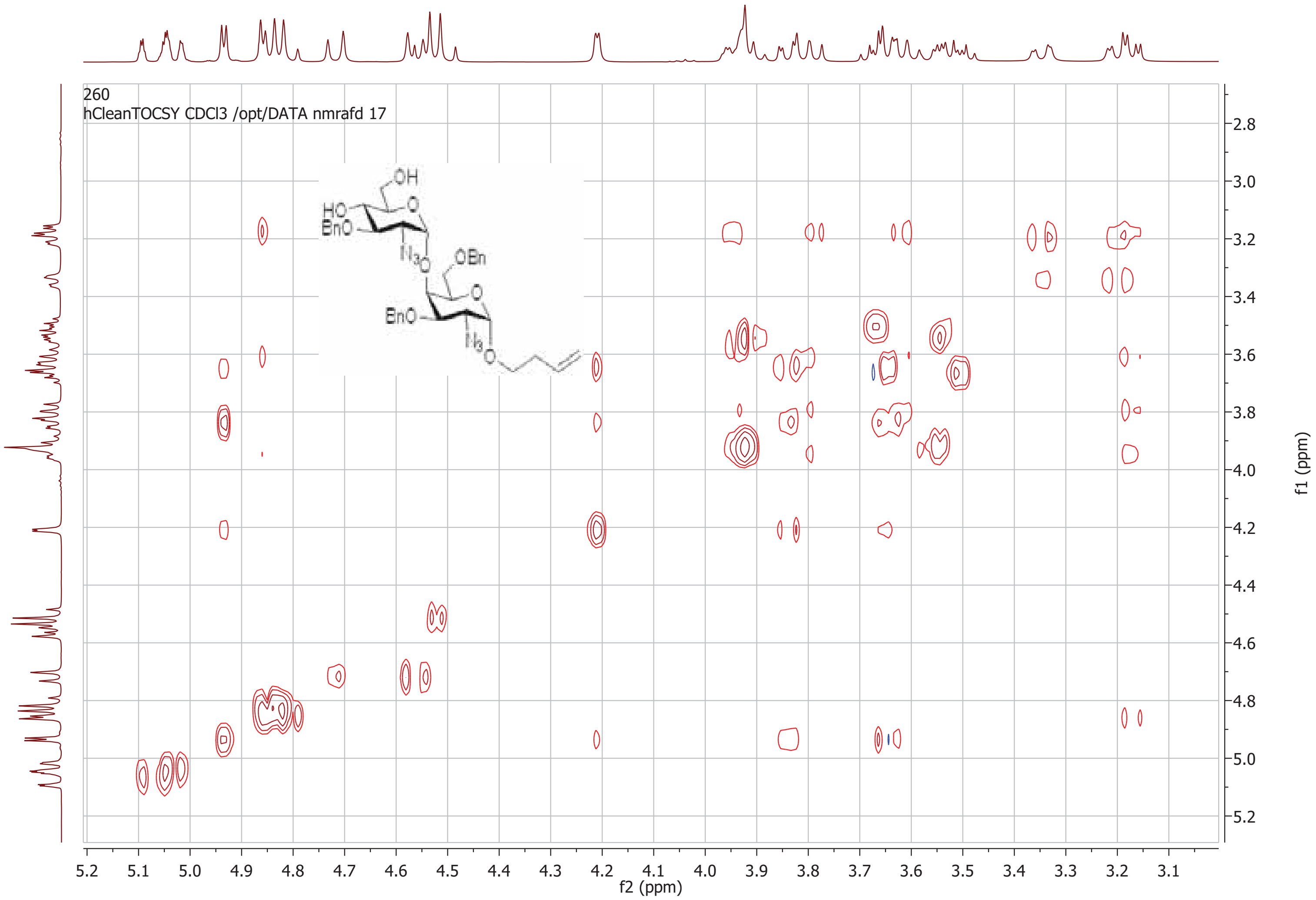
260
hCleanTOCSY CDCl3 /opt/DATA nmrafd 17
f2 (ppm)
f1 (ppm)

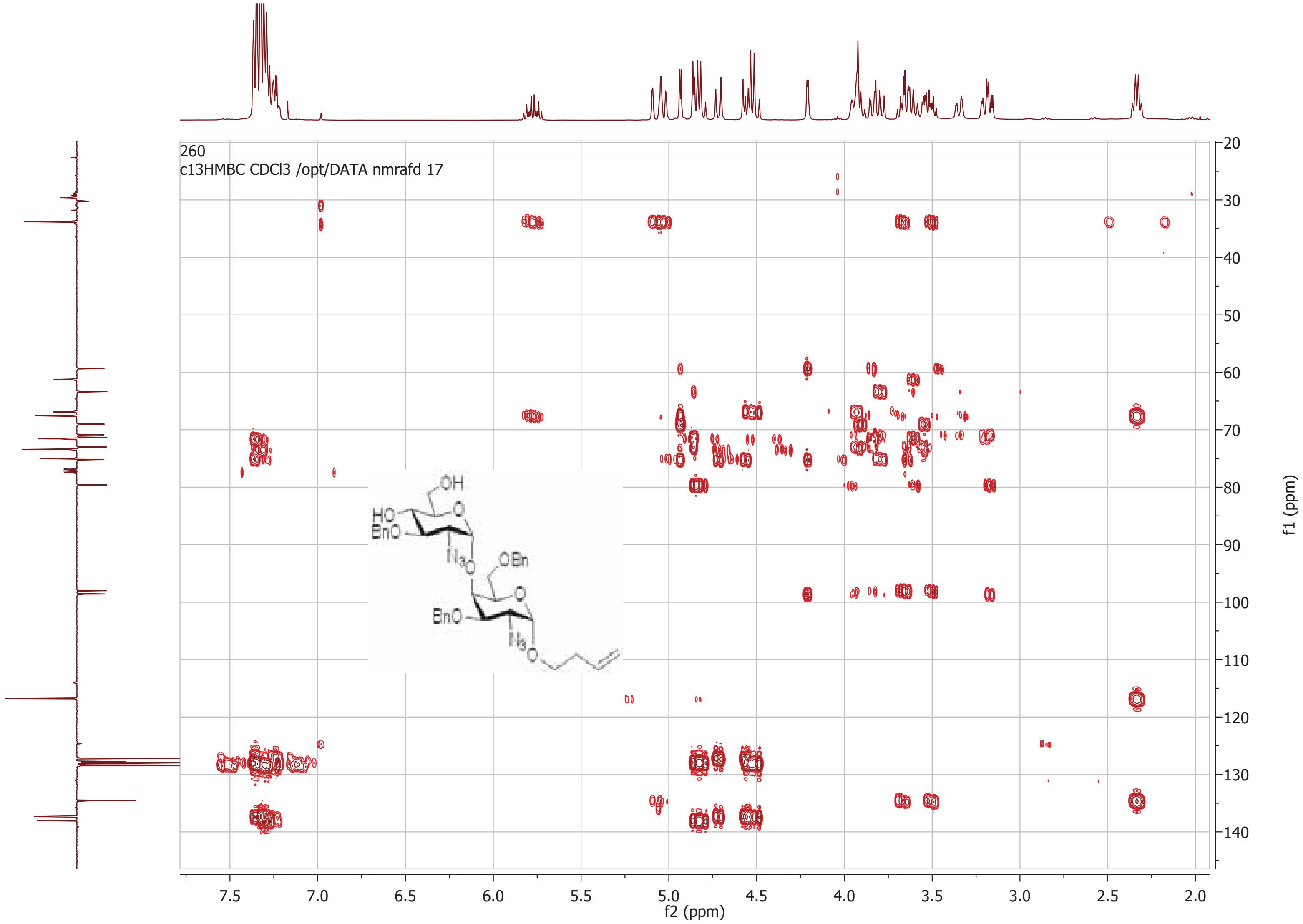

260
c13HMBC CDCl3 /opt/DATA nmrafd 17
f2 (ppm)
f1 (ppm)
7.5
7.0
6.5
6.0
5.5
5.0
4.5
4.0
3.5
3.0
2.5
2.0
20
30
40
50
60
70
80
90
100
110
120
130
140
OH
HO
BnO
N3
OBn
BnO
N3

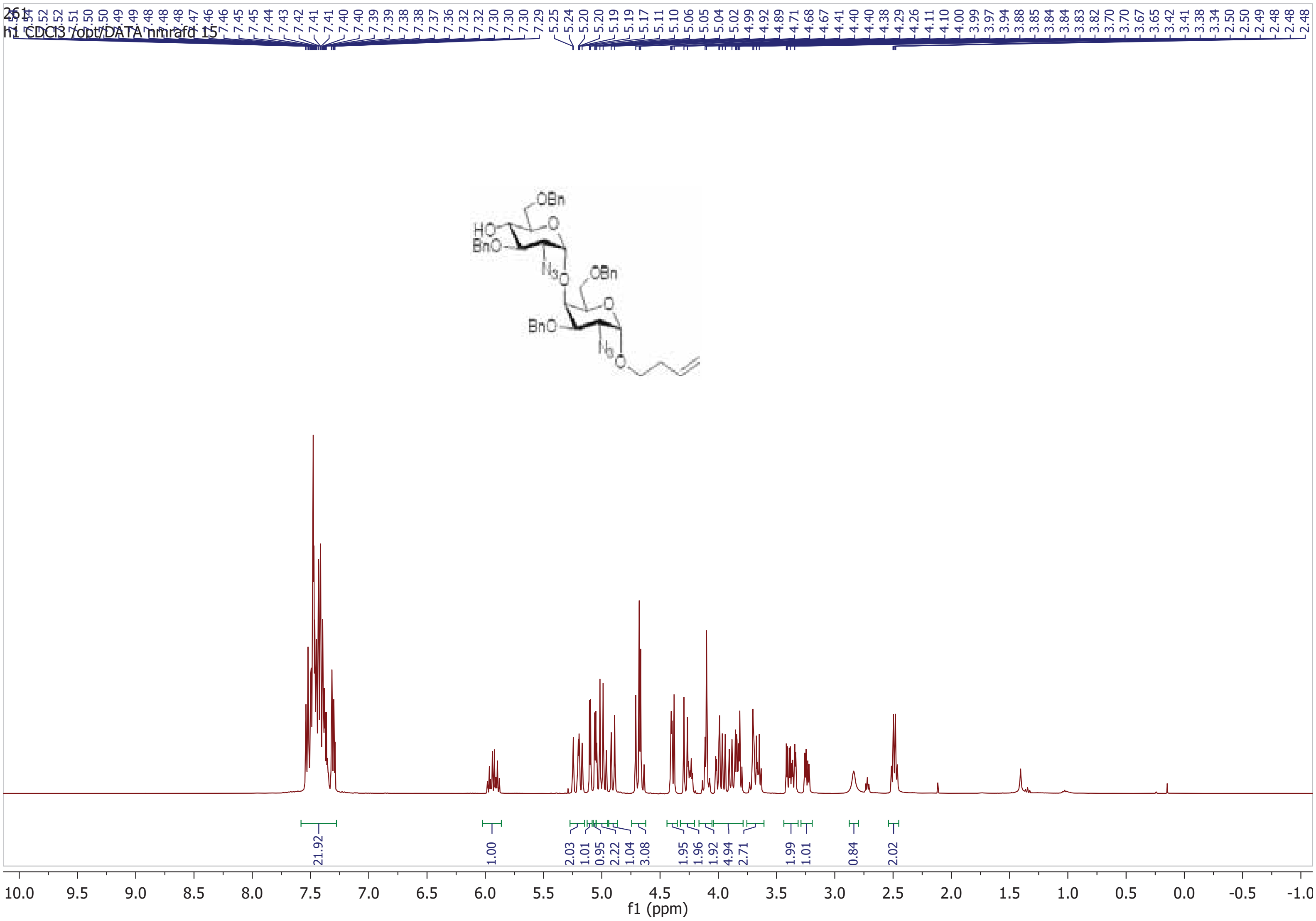
261
h1 CDCl3 /opt/DATA nmrafd 15
7.54 7.52 7.52 7.51 7.50 7.49 7.49 7.48 7.48 7.48 7.47 7.46 7.45 7.45 7.44 7.43 7.42 7.41 7.41 7.40 7.40 7.39 7.39 7.38 7.38 7.37 7.36 7.32 7.32 7.30 7.30 7.30 7.29
5.25 5.24 5.20 5.20 5.19 5.19 5.17 5.11 5.10 5.06 5.05 5.04 5.02 4.99 4.92 4.89 4.71 4.68 4.67 4.41 4.40 4.40 4.38 4.29 4.26 4.11 4.10 4.00 3.99 3.97 3.94 3.88 3.85 3.84 3.84 3.83 3.82 3.70 3.70 3.67 3.65 3.42 3.41 3.38 3.34 2.50 2.50 2.49 2.48 2.48 2.48
OBn
HO
O
BnO
N3
O
OBn
O
BnO
N3
O
21.92
1.00
2.03
1.01
0.95
2.22
1.04
3.08
1.95
1.96
1.92
4.94
2.71
1.99
1.01
0.84
2.02
10.0 9.5 9.0 8.5 8.0 7.5 7.0 6.5 6.0 5.5 5.0 4.5 4.0 3.5 3.0 2.5 2.0 1.5 1.0 0.5 0.0 -0.5 -1.0
f1 (ppm)

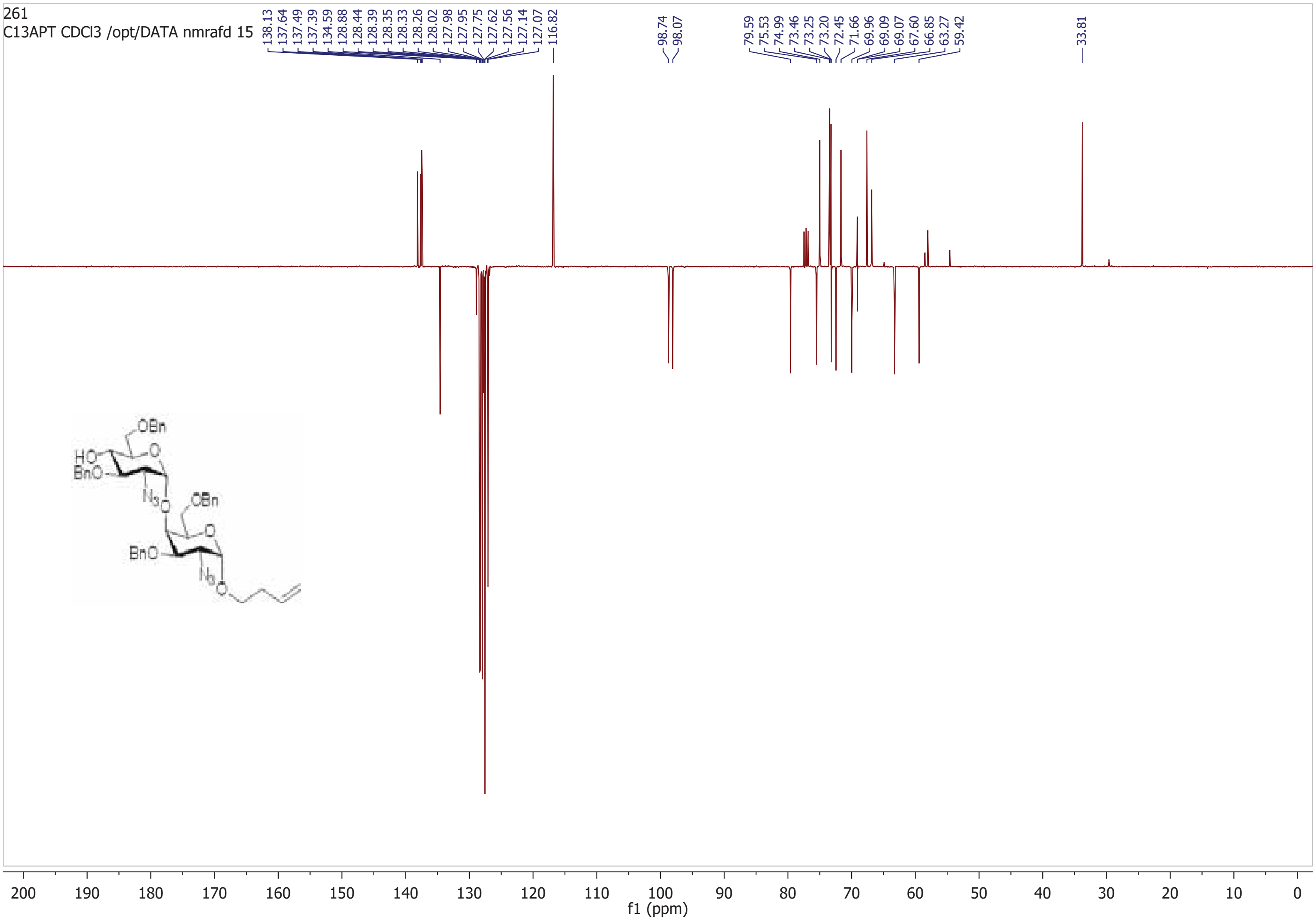
261
C13APT CDCl3 /opt/DATA nmrafd 15
138.13
137.64
137.49
137.39
134.59
128.88
128.44
128.39
128.35
128.33
128.26
128.02
127.98
127.95
127.75
127.62
127.56
127.14
127.07
116.82
98.74
98.07
79.59
75.53
74.99
73.46
73.25
73.20
72.45
71.66
69.96
69.09
69.07
67.60
66.85
63.27
59.42
33.81
OBn
HO
BnO
N3
O
OBn
BnO
N3
O
200
190
180
170
160
150
140
130
120
110
100
90
80
70
60
50
40
30
20
10
0
f1 (ppm)

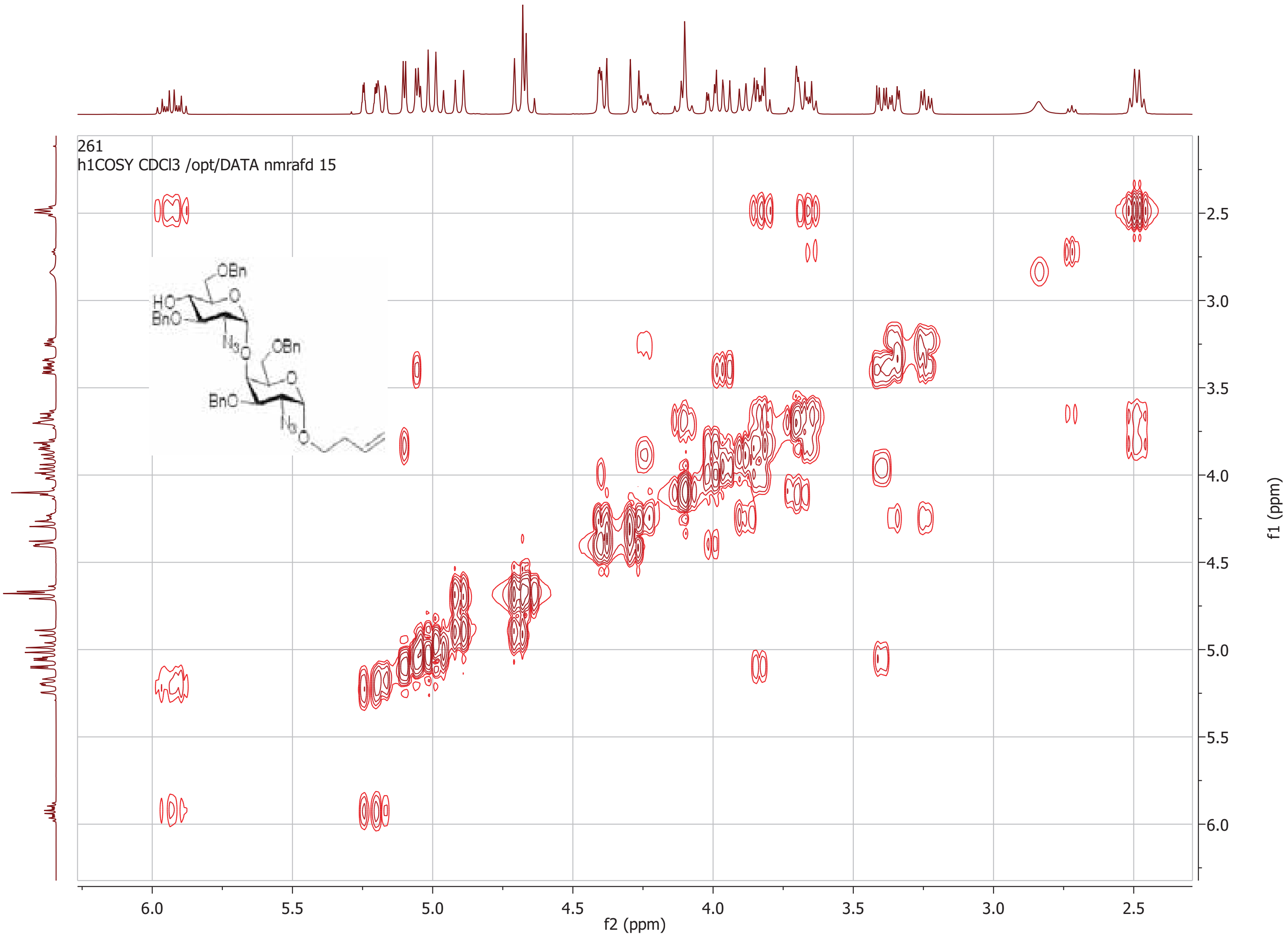
261
h1COSY CDCl3 /opt/DATA nmrafd 15
OBn
HO
BnO
O
N3
OBn
O
BnO
O
N3
O
f2 (ppm)
f1 (ppm)
6.0
5.5
5.0
4.5
4.0
3.5
3.0
2.5

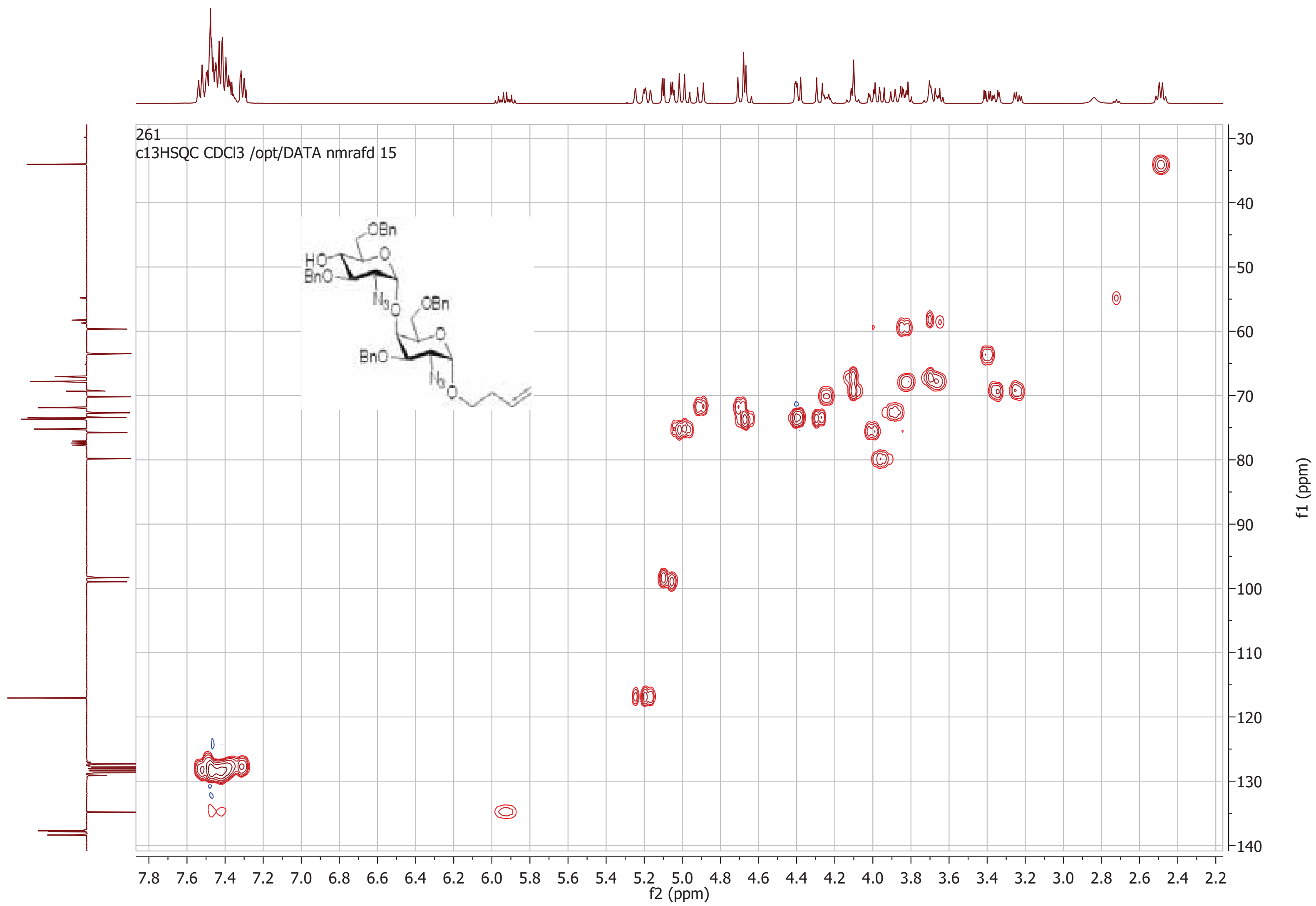

261
c13HSQC CDCl3 /opt/DATA nmrafd 15
OBn
HO
BnO
N3
O
OBn
BnO
N3
O
f2 (ppm)
f1 (ppm)

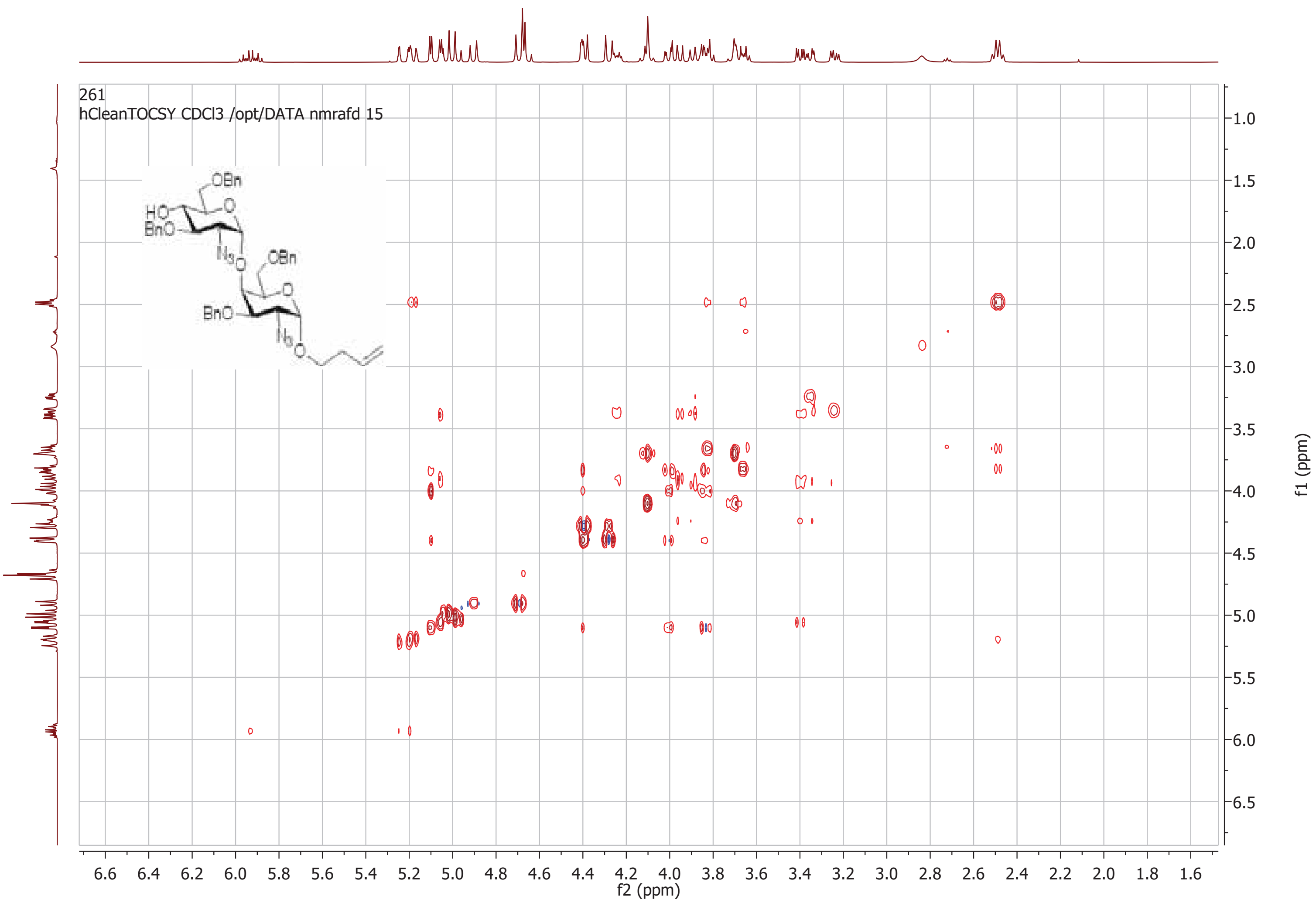
261
hCleanTOCSY CDCl3 /opt/DATA nmrafd 15
OBn
HO
BnO
N3
O
OBn
BnO
N3
f2 (ppm)
f1 (ppm)

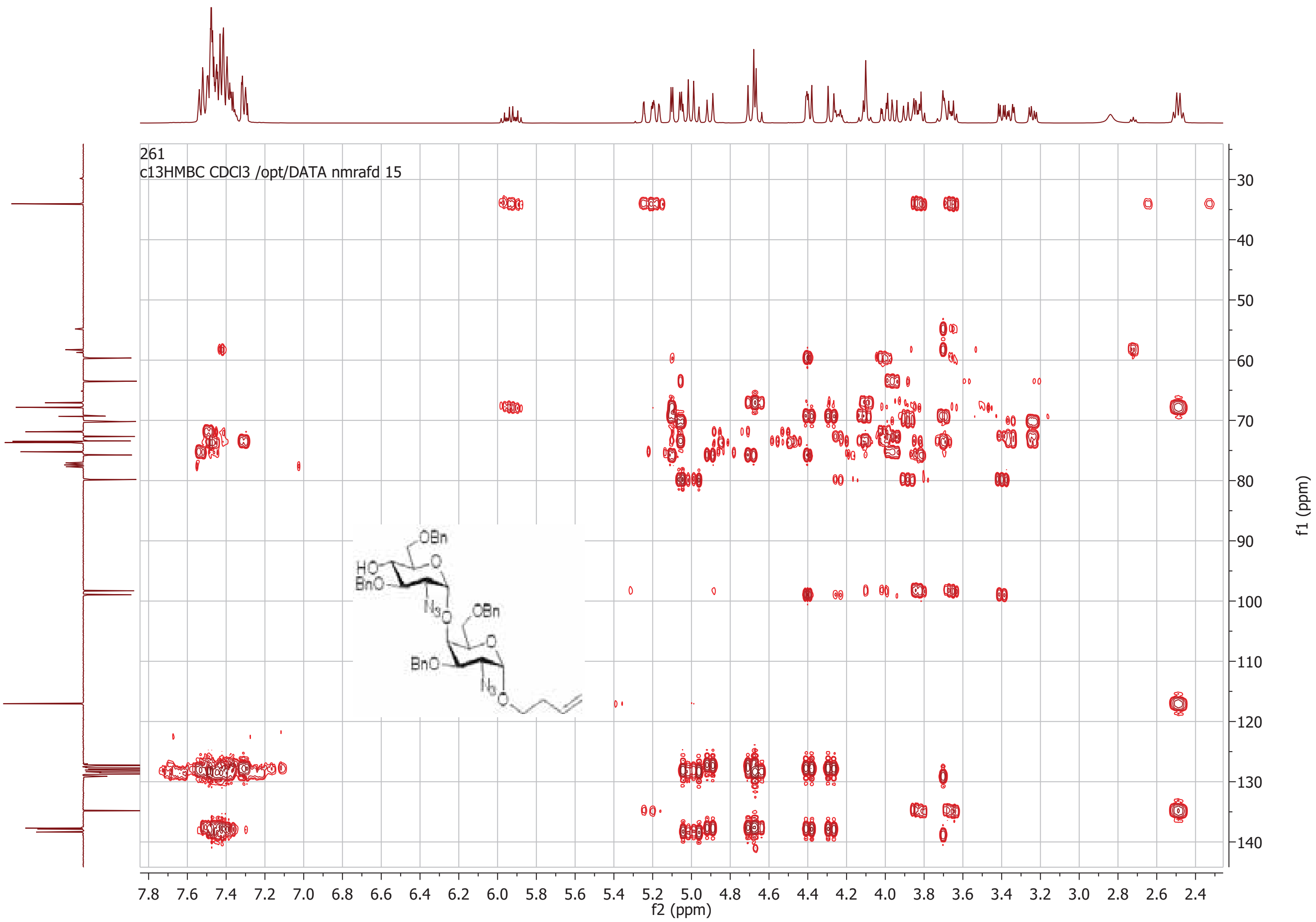

261
c13HMBC CDCl3 /opt/DATA nmrafd 15
OBn
HO
BnO
N3
O
OBn
BnO
N3
f2 (ppm)
f1 (ppm)

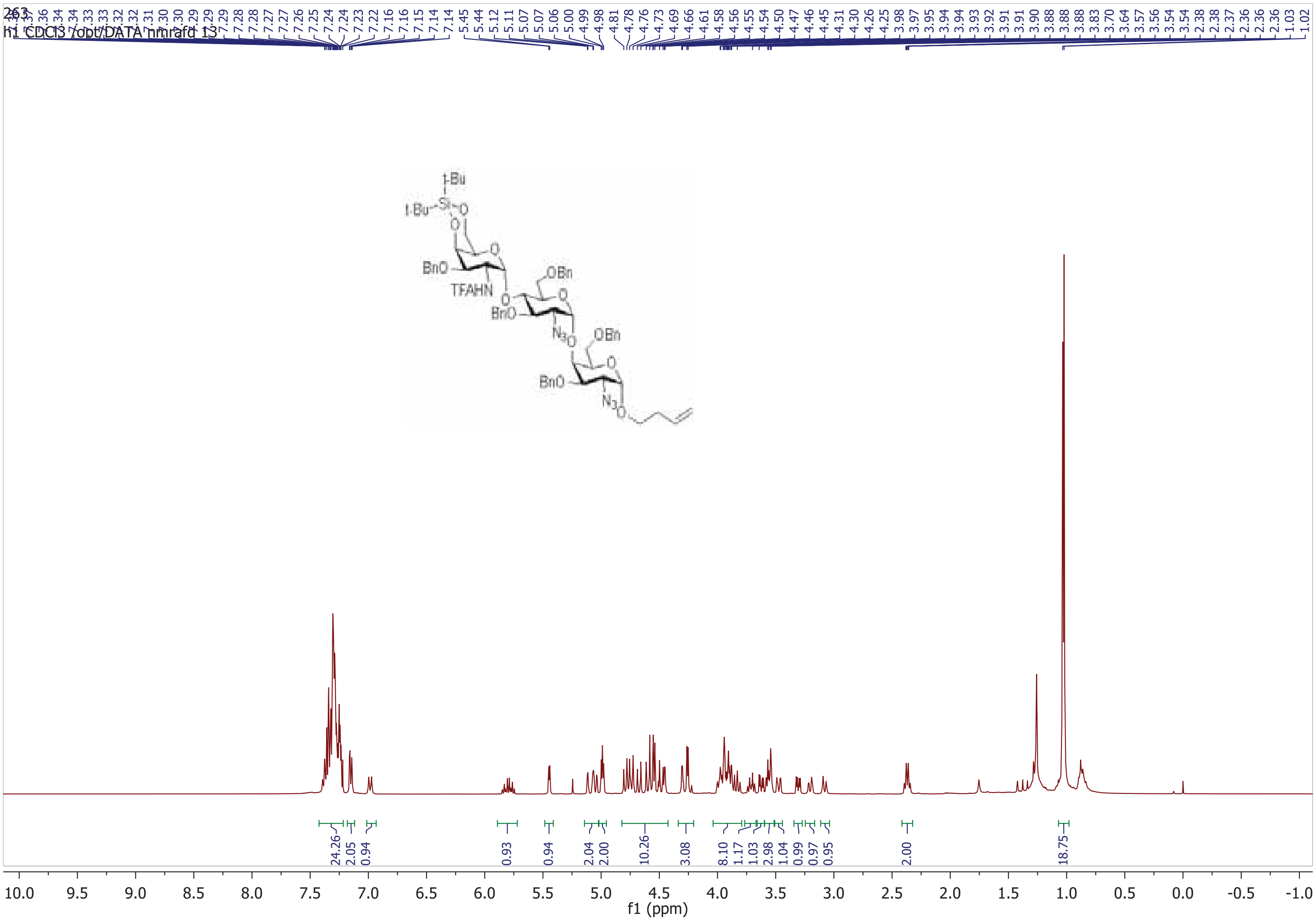
263
h1 CDCl3 /opt/DATA nmrafd 13
7.36 7.34 7.34 7.33 7.33 7.32 7.32 7.31 7.30 7.30 7.29 7.29 7.28 7.28 7.27 7.26 7.25 7.24 7.24 7.23 7.22 7.16 7.15 7.14
5.45 5.44 5.12 5.11 5.07 5.07 5.06 5.00 4.99 4.98 4.81 4.78 4.76 4.73 4.69 4.66 4.61 4.58 4.56 4.55 4.54 4.50 4.47 4.46 4.45 4.31 4.30 4.26 4.25 3.98 3.97 3.95 3.94 3.94 3.93 3.92 3.91 3.91 3.90 3.88 3.88 3.88 3.83 3.70 3.64 3.57 3.56 3.54 2.38 2.38 2.37 2.36 2.36 2.36 1.03 1.02
t-Bu
t-Bu
Si
BnO
TFAHN
OBn
BnO
N3
OBn
BnO
N3
24.26 2.05 0.94 0.93 0.94 2.04 2.00 10.26 3.08 8.10 1.17 1.03 2.98 1.04 0.99 0.97 0.95 2.00 18.75
10.0 9.5 9.0 8.5 8.0 7.5 7.0 6.5 6.0 5.5 5.0 4.5 4.0 3.5 3.0 2.5 2.0 1.5 1.0 0.5 0.0 -0.5 -1.0
f1 (ppm)

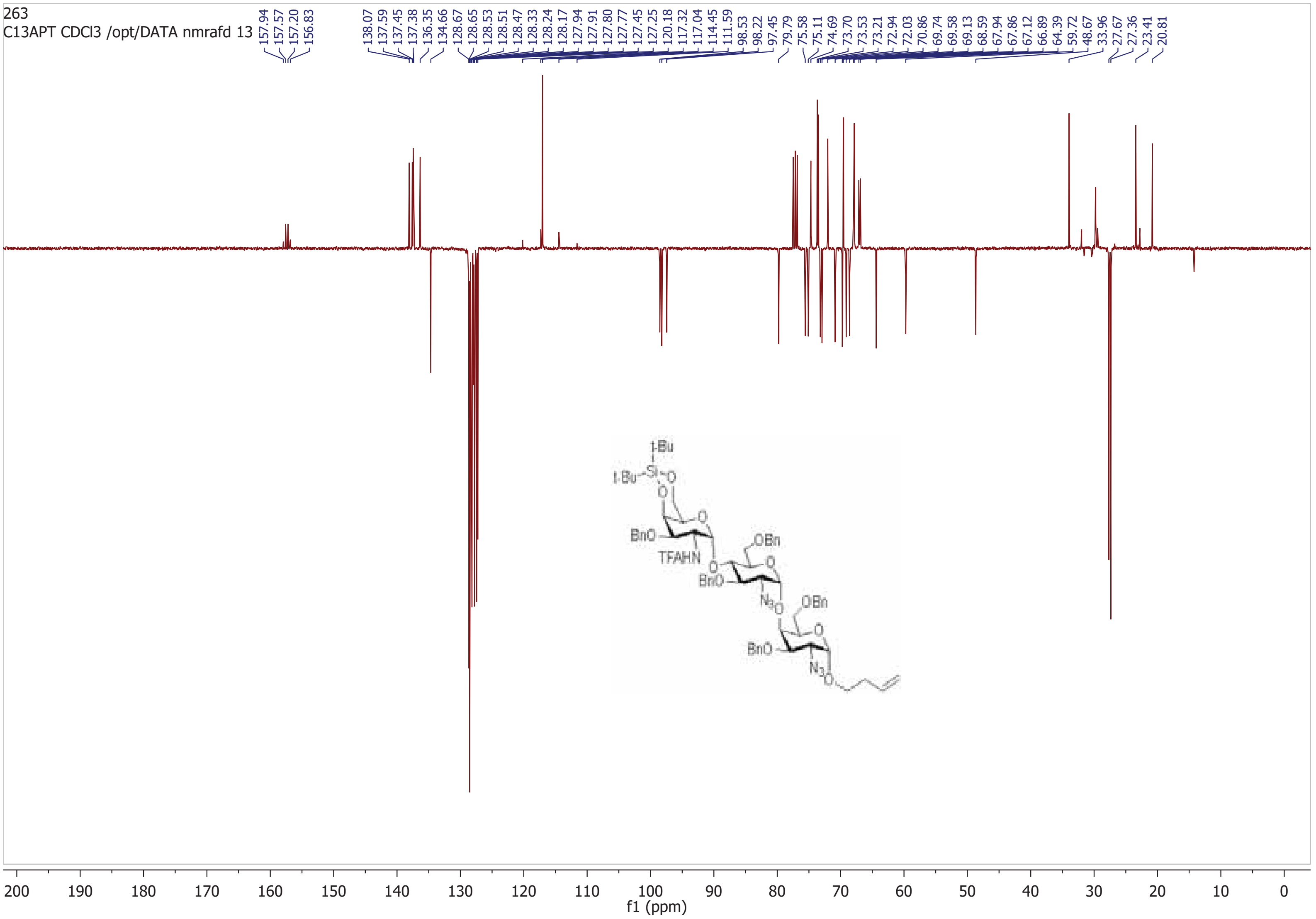
263
C13APT CDCl3 /opt/DATA nmrafd 13
157.94
157.57
157.20
156.83
138.07
137.59
137.45
137.38
136.35
134.66
128.67
128.65
128.53
128.51
128.47
128.33
128.24
128.17
127.94
127.91
127.80
127.77
127.45
127.25
120.18
117.32
117.04
114.45
111.59
98.53
98.22
97.45
79.79
75.58
75.11
74.69
73.70
73.53
73.21
72.94
72.03
70.86
69.74
69.58
69.13
68.59
67.94
67.86
67.12
66.89
64.39
59.72
48.67
33.96
27.67
27.36
23.41
20.81
t-Bu
t-Bu
Si
O
O
O
BnO
TFAHN
O
OBn
O
BnO
N3
O
OBn
O
BnO
N3
O
200
190
180
170
160
150
140
130
120
110
100
90
80
70
60
50
40
30
20
10
0
f1 (ppm)

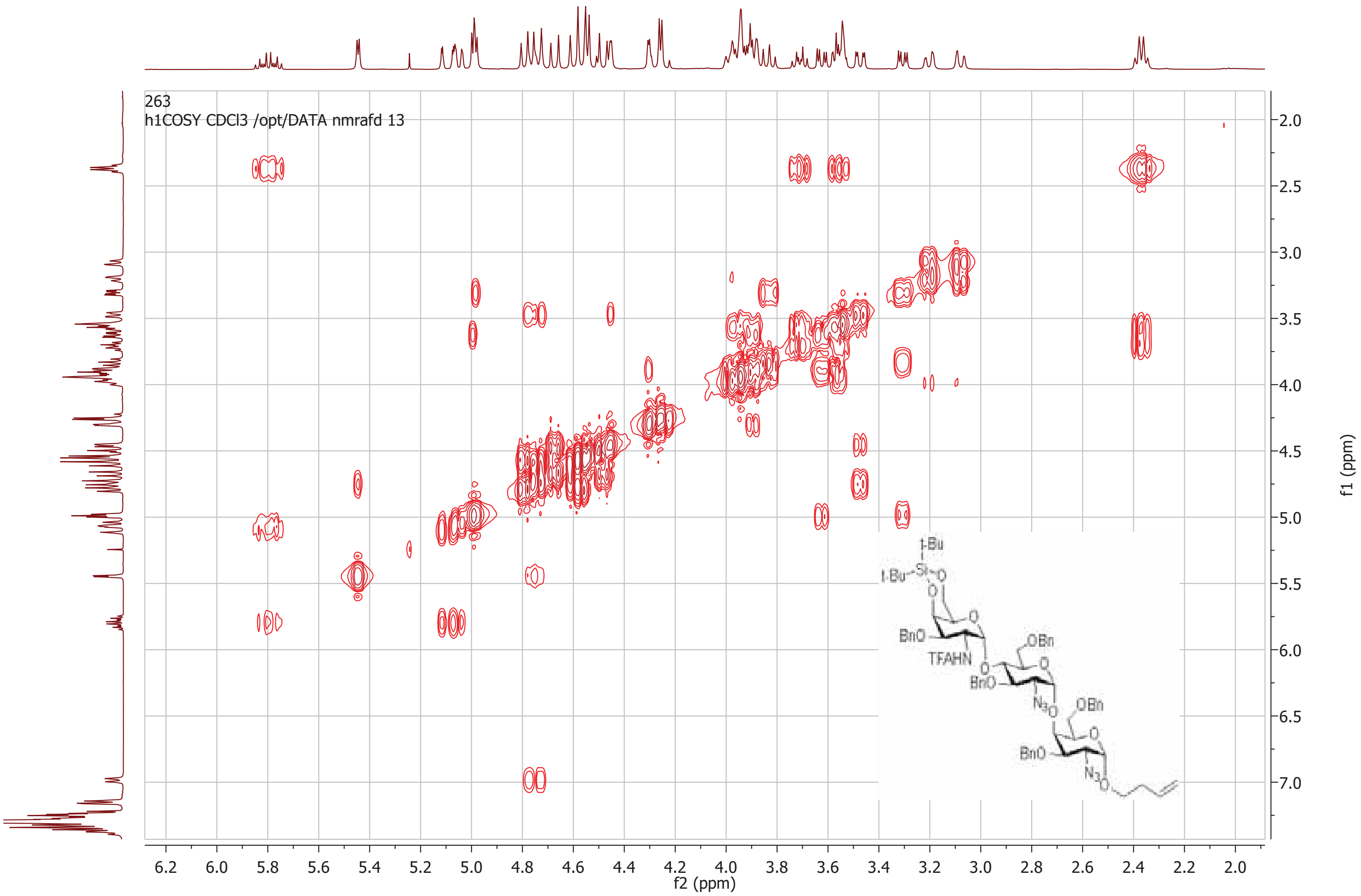
263
h1COSY CDCl3 /opt/DATA nmrafd 13
f2 (ppm)
f1 (ppm)
t-Bu
t-Bu
Si
BnO
TFAHN
OBn
BnO
N3
OBn
BnO
N3

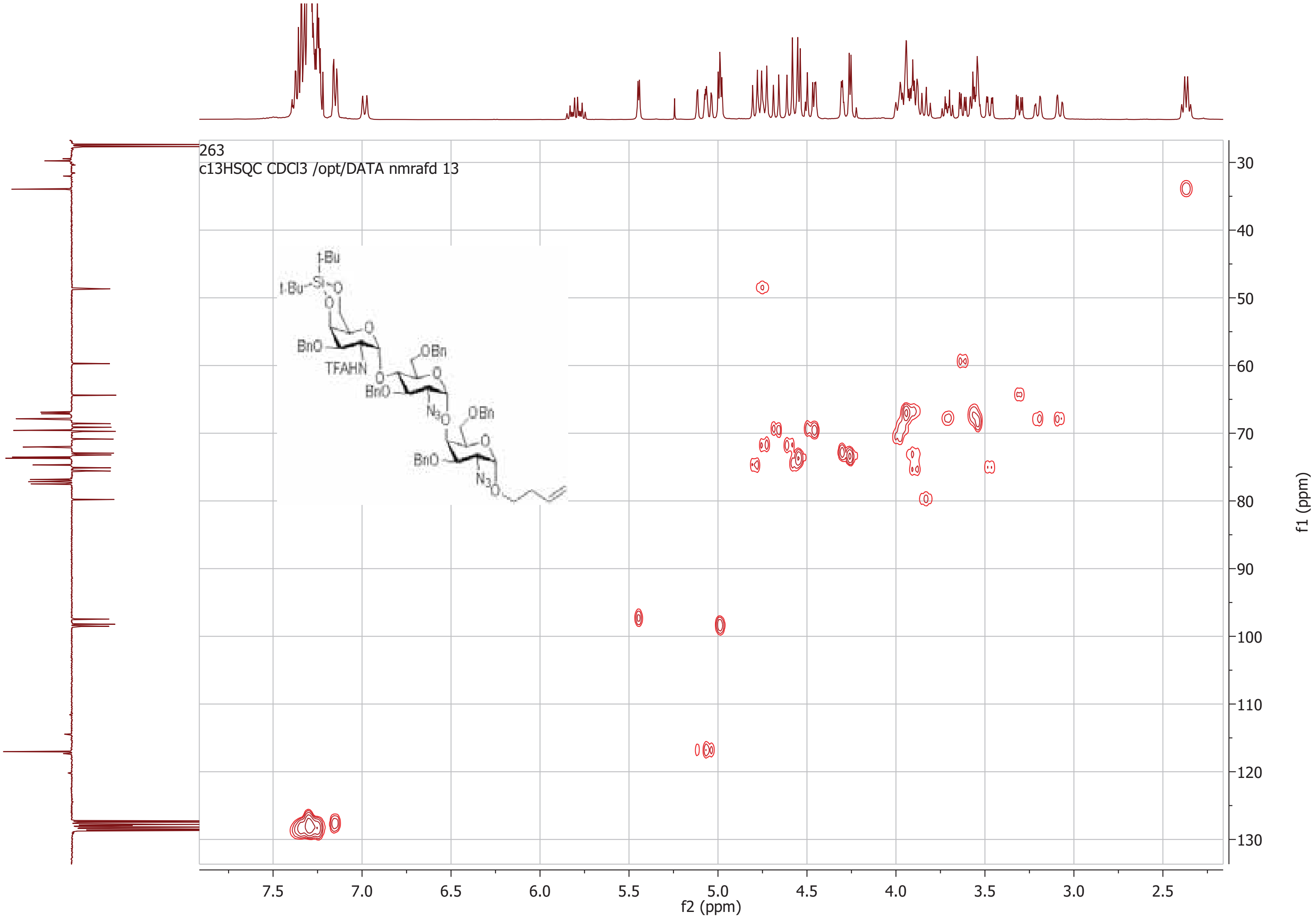
263
c13HSQC CDCl3 /opt/DATA nmrafd 13
t-Bu
t-Bu
Si
BnO
TFAHN
OBn
BnO
N_3
OBn
BnO
N_3
f2 (ppm)
f1 (ppm)

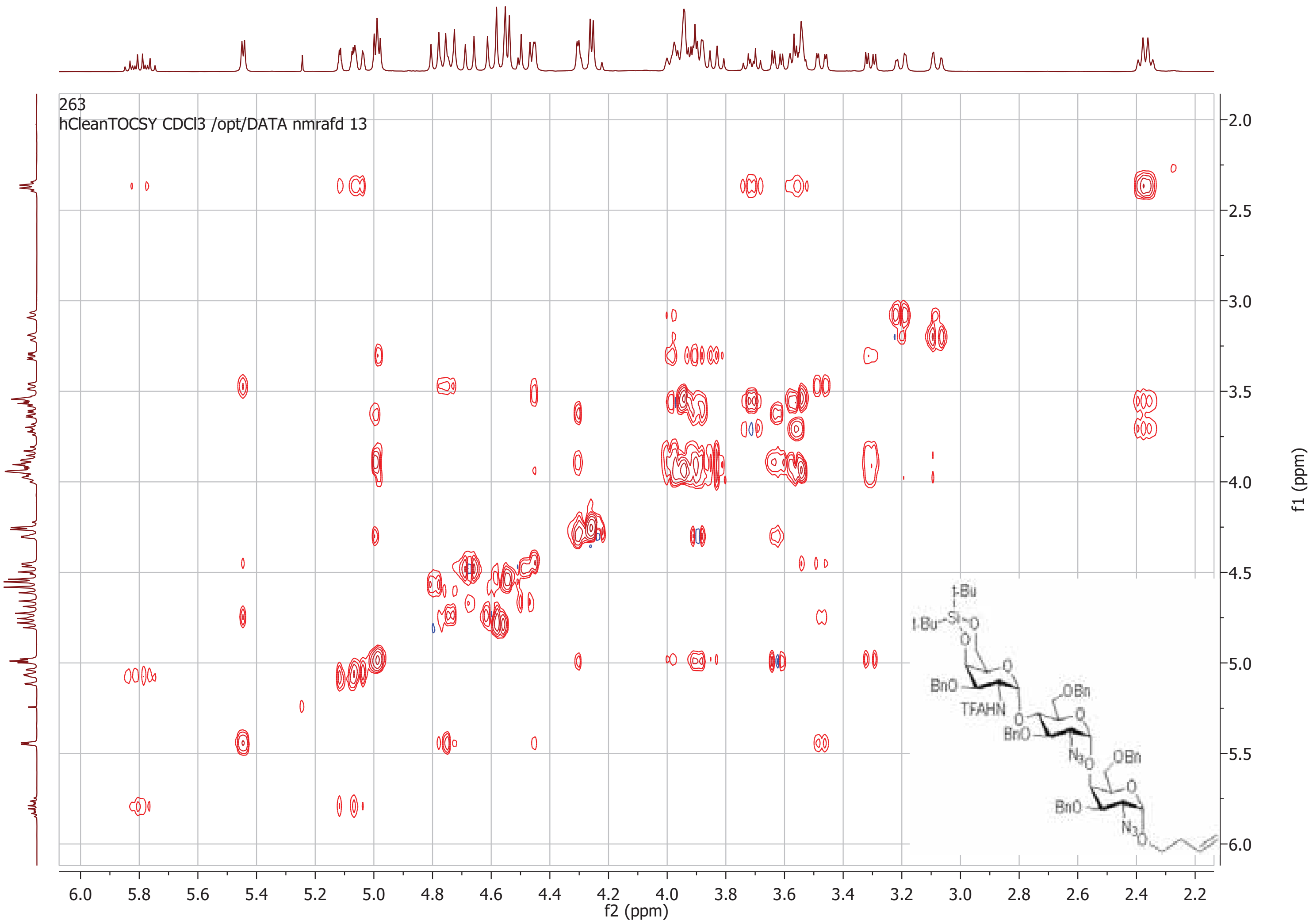
263
hCleanTOCSY CDCl3 /opt/DATA nmrafd 13
f2 (ppm)
f1 (ppm)
t-Bu
t-Bu
Si
BnO
TFAHN
OBn
BnO
N3
OBn
BnO
N3

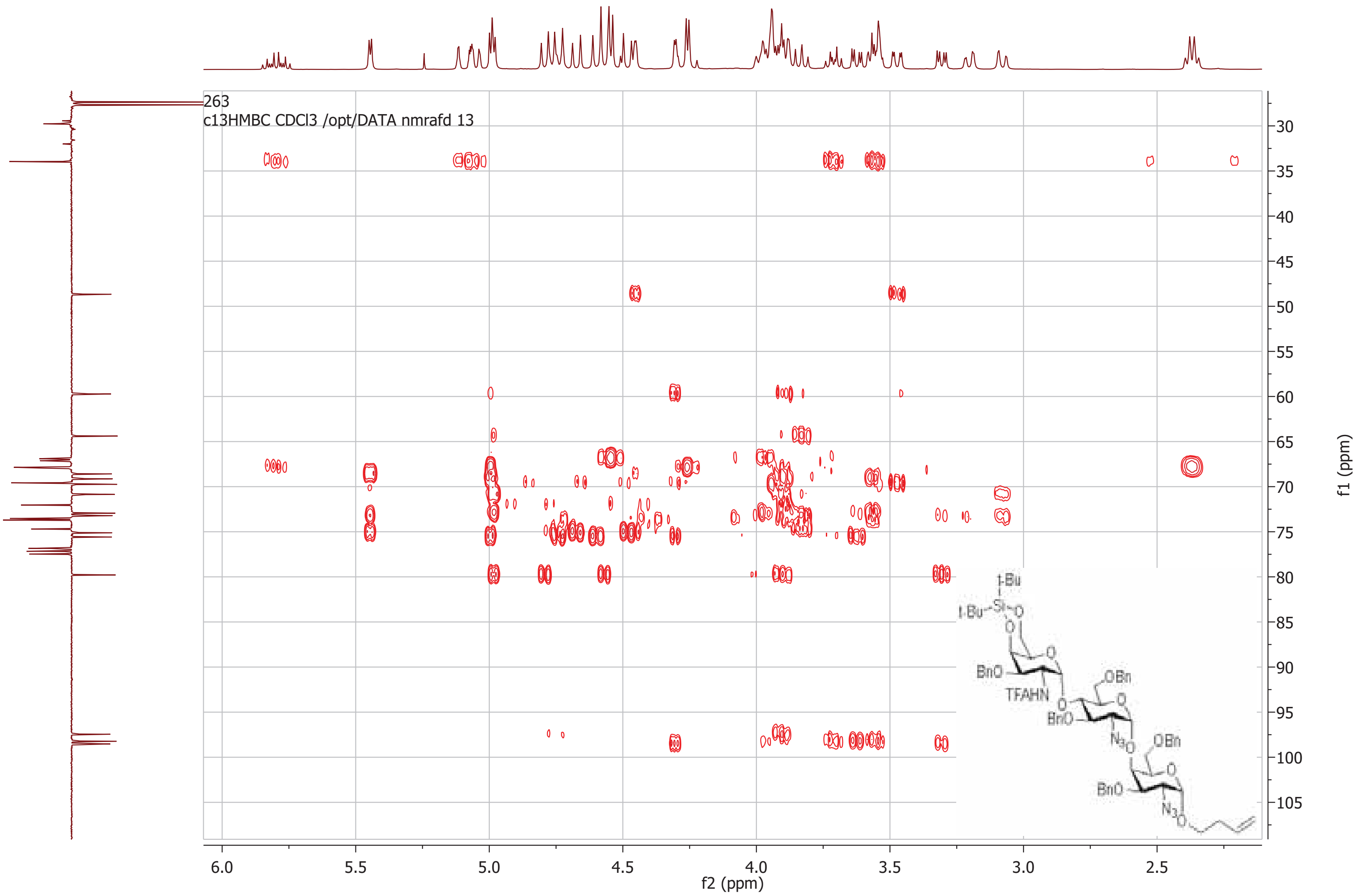
263
c13HMBC CDCl3 /opt/DATA nmrafd 13
f2 (ppm)
f1 (ppm)
t-Bu
t-Bu
Si
BnO
TFAHN
OBn
BnO
N3
OBn
BnO
N3

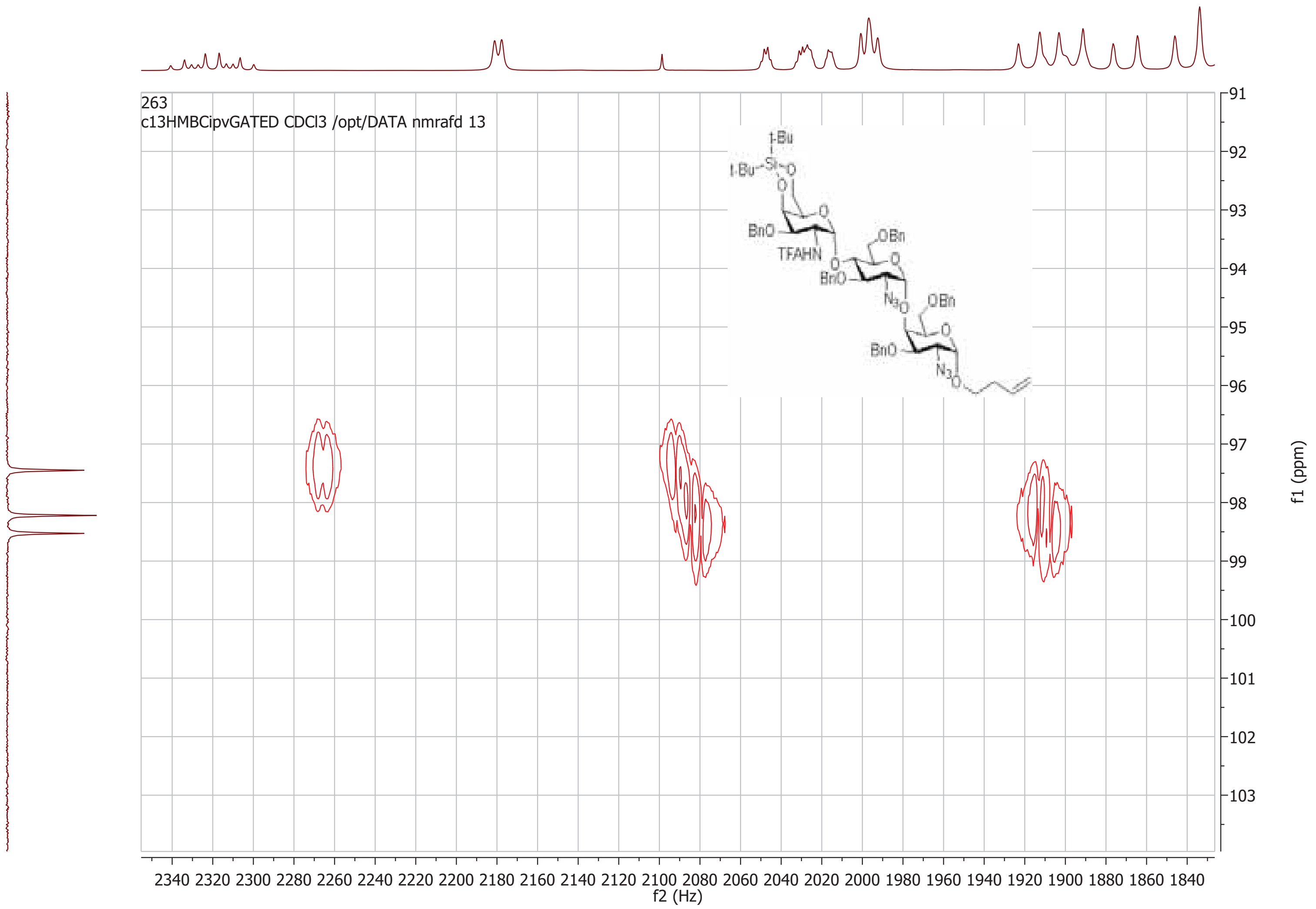
263
c13HMBCipvGATED CDCl3 /opt/DATA nmrafd 13
t-Bu
t-Bu
Si
BnO
TFAHN
OBn
BnO
N3
OBn
BnO
N3
f2 (Hz)
f1 (ppm)

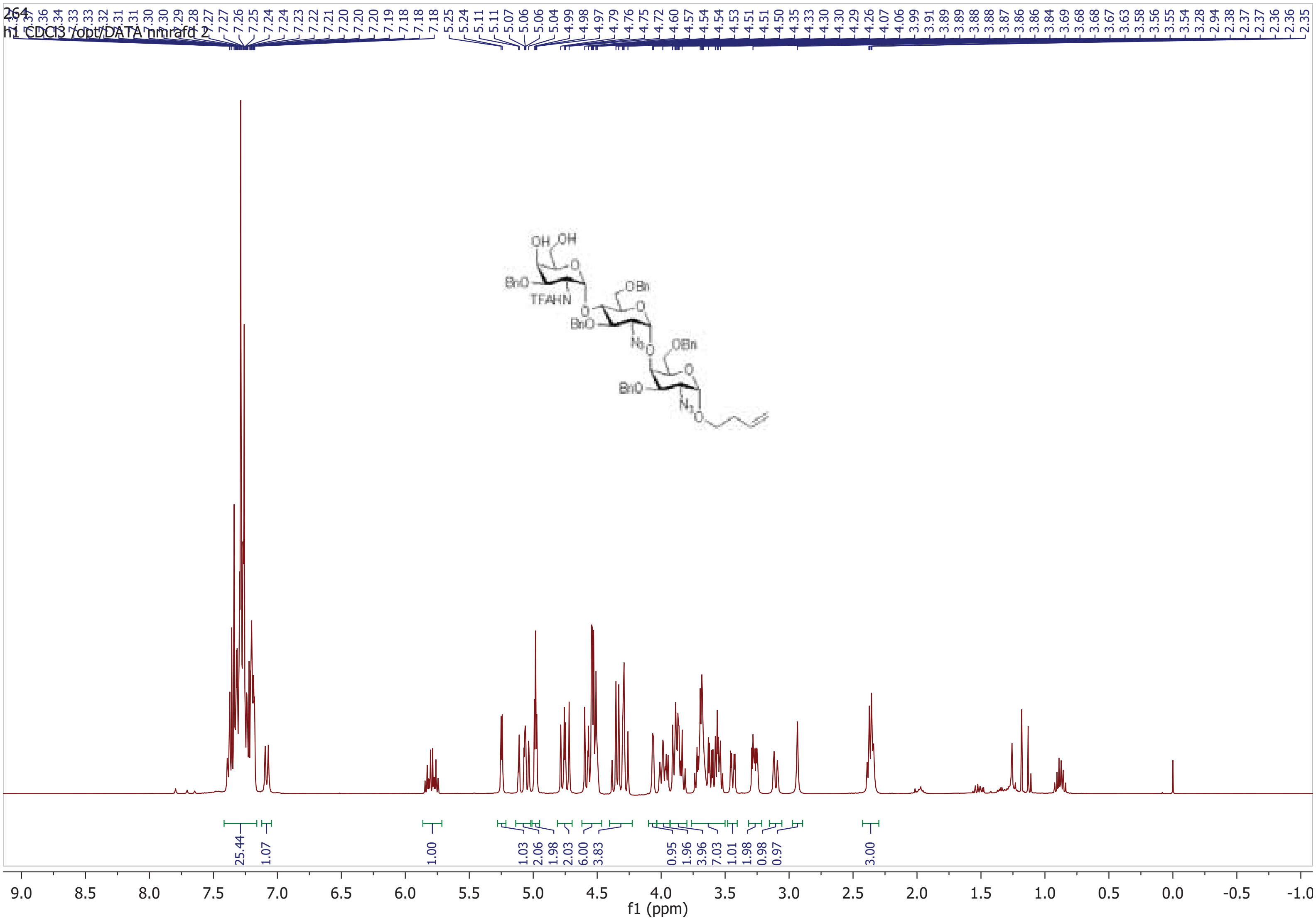
264
h1 CDCl3 /opt/DATA nmrafd 2
7.36 7.34 7.33 7.33 7.32 7.31 7.31 7.30 7.30 7.29 7.28 7.27 7.26 7.25 7.24 7.24 7.23 7.22 7.21 7.20 7.20 7.20 7.19 7.18 7.18 5.25 5.24 5.11 5.11 5.07 5.06 5.06 5.04 4.99 4.98 4.97 4.79 4.76 4.75 4.72 4.60 4.57 4.54 4.54 4.53 4.51 4.51 4.50 4.35 4.33 4.30 4.30 4.29 4.26 4.07 4.06 3.99 3.91 3.89 3.89 3.88 3.87 3.86 3.86 3.84 3.69 3.68 3.67 3.63 3.58 3.56 3.55 3.54 3.28 2.94 2.38 2.37 2.37 2.36 2.35
OH OH
BnO
TFAHN
OBn
BnO
N3
OBn
BnO
N3
25.44 1.07 1.00 1.03 2.06 1.98 2.03 6.00 3.83 0.95 1.96 3.96 7.03 1.01 1.98 0.98 0.97 3.00
9.0 8.5 8.0 7.5 7.0 6.5 6.0 5.5 5.0 4.5 4.0 3.5 3.0 2.5 2.0 1.5 1.0 0.5 0.0 -0.5 -1.0
f1 (ppm)

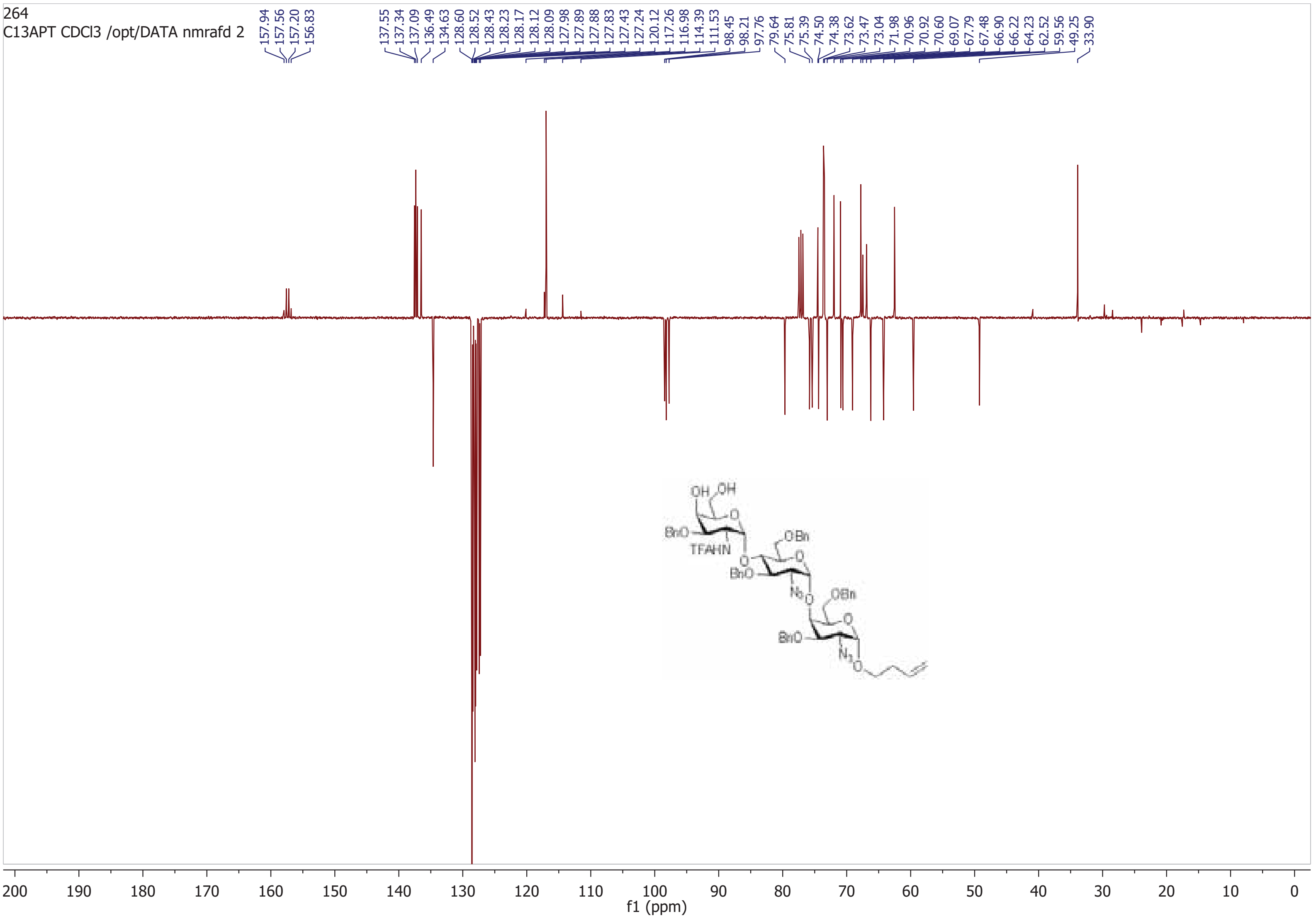
264
C13APT CDCl3 /opt/DATA nmrafd 2
157.94
157.56
157.20
156.83
137.55
137.34
137.09
136.49
134.63
128.60
128.52
128.43
128.23
128.17
128.12
128.09
127.98
127.89
127.88
127.83
127.43
127.24
120.12
117.26
116.98
114.39
111.53
98.45
98.21
97.76
79.64
75.81
75.39
74.50
74.38
73.62
73.47
73.04
71.98
70.96
70.92
70.60
69.07
67.79
67.48
66.90
66.22
64.23
62.52
59.56
49.25
33.90
200 190 180 170 160 150 140 130 120 110 100 90 80 70 60 50 40 30 20 10 0
f1 (ppm)
OH
OH
BnO
TFAHN
OBn
BnO
N3
OBn
BnO
N3

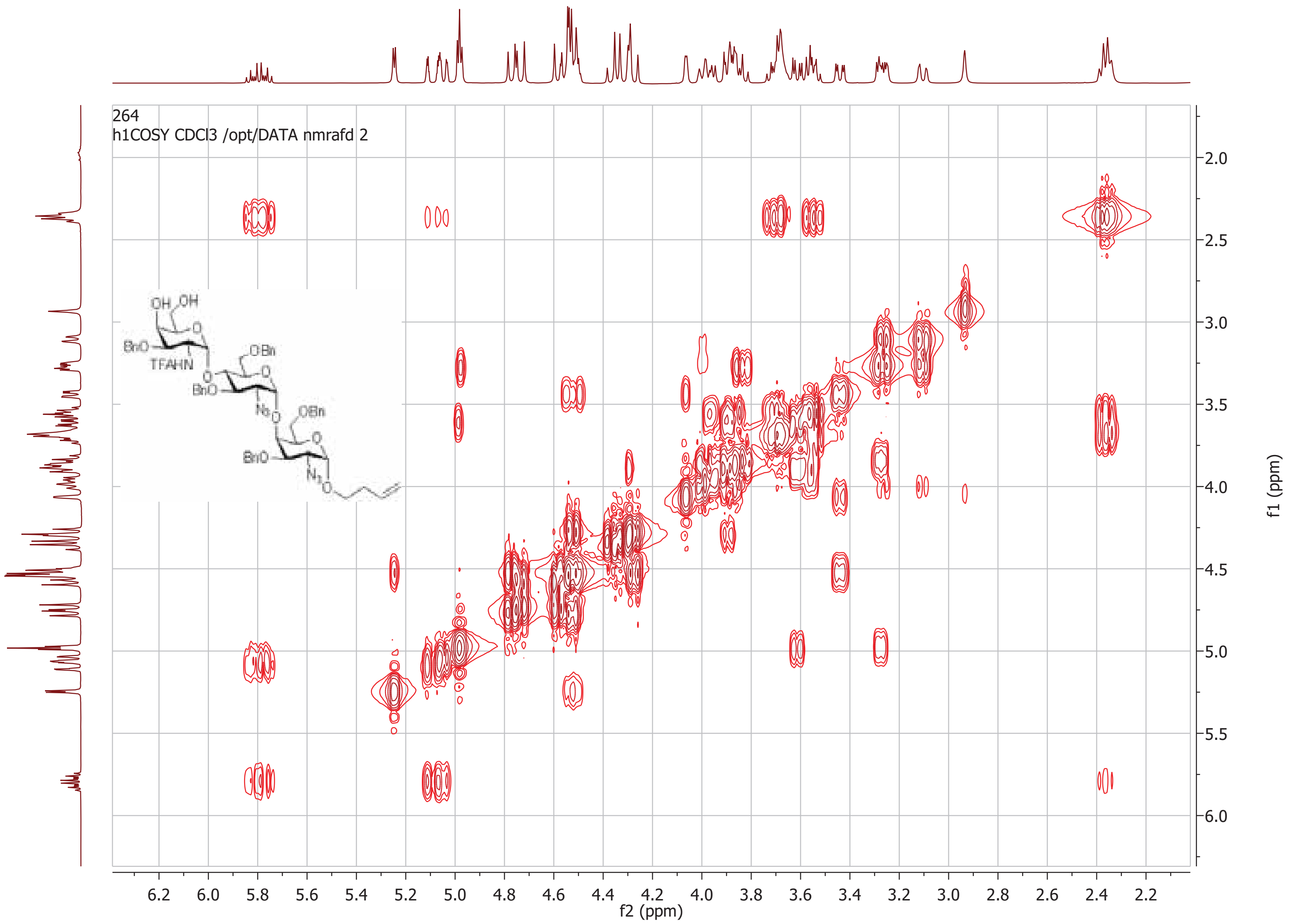
264
h1COSY CDCl3 /opt/DATA nmrafd 2
f2 (ppm)
f1 (ppm)

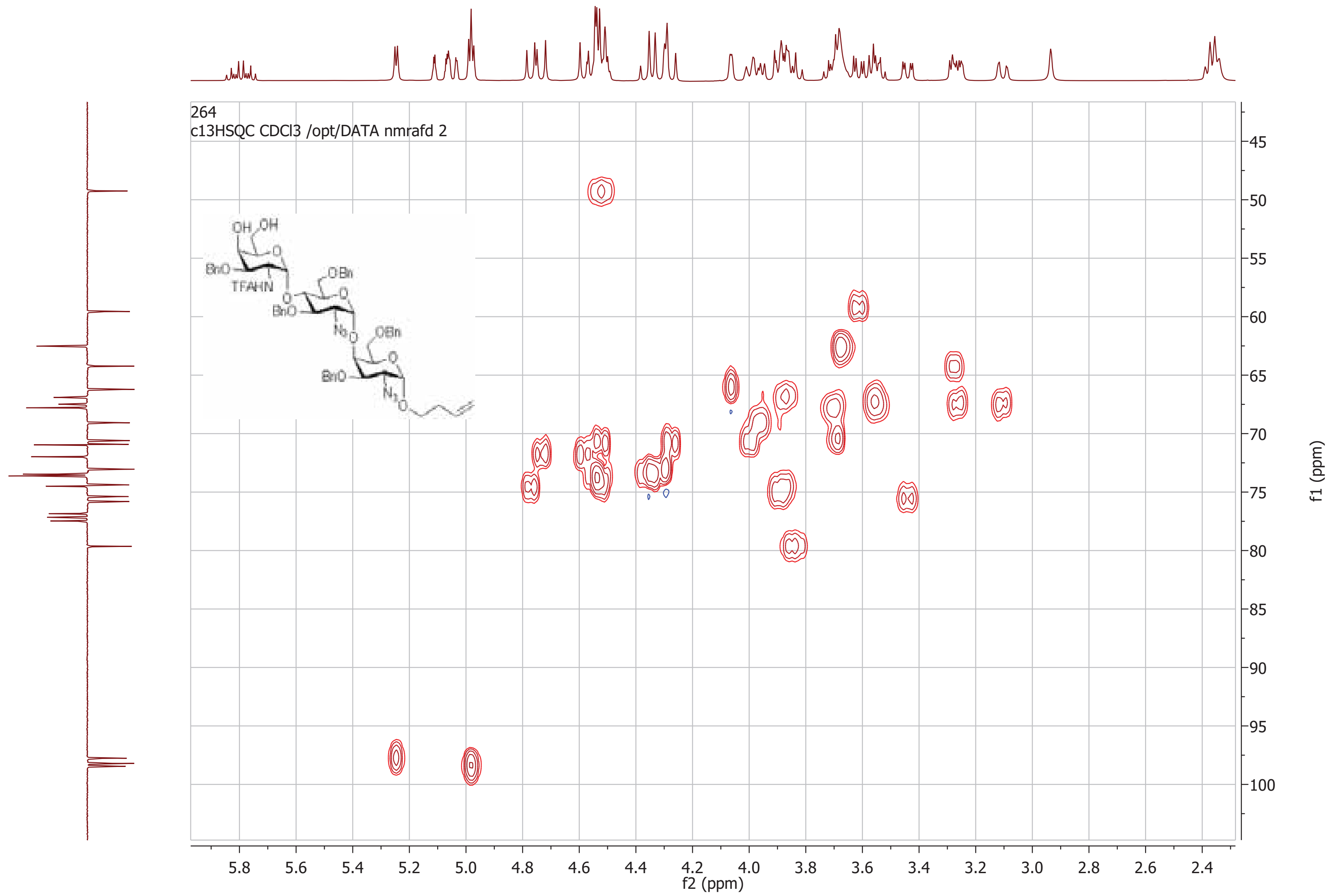
264
c13HSQC CDCl3 /opt/DATA nmrafd 2
f2 (ppm)
f1 (ppm)

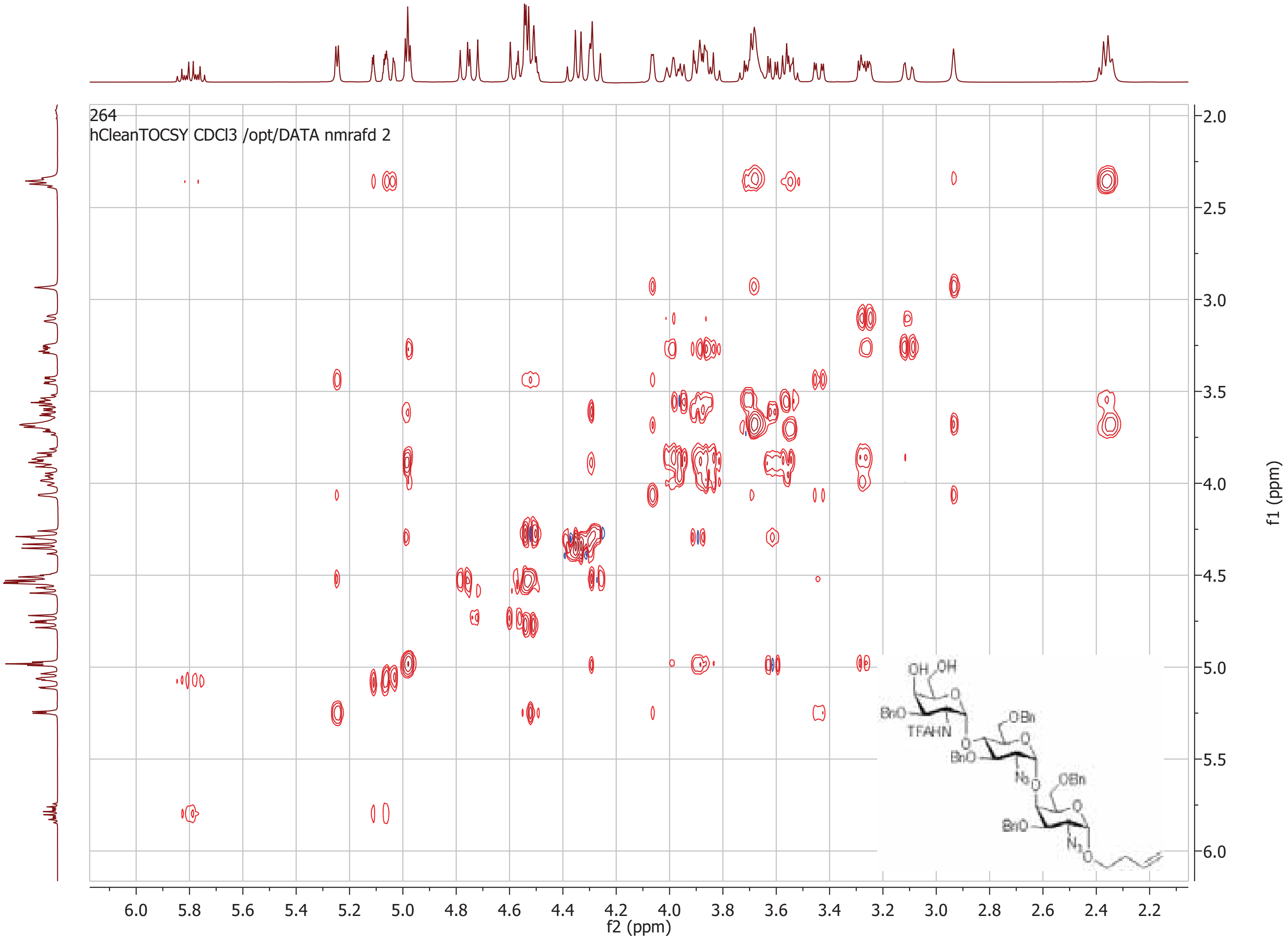
264
hCleanTOCSY CDCl3 /opt/DATA nmrafd 2
f2 (ppm)
f1 (ppm)
OH OH
BnO
TFAHN
OBn
BnO
OBn
BnO

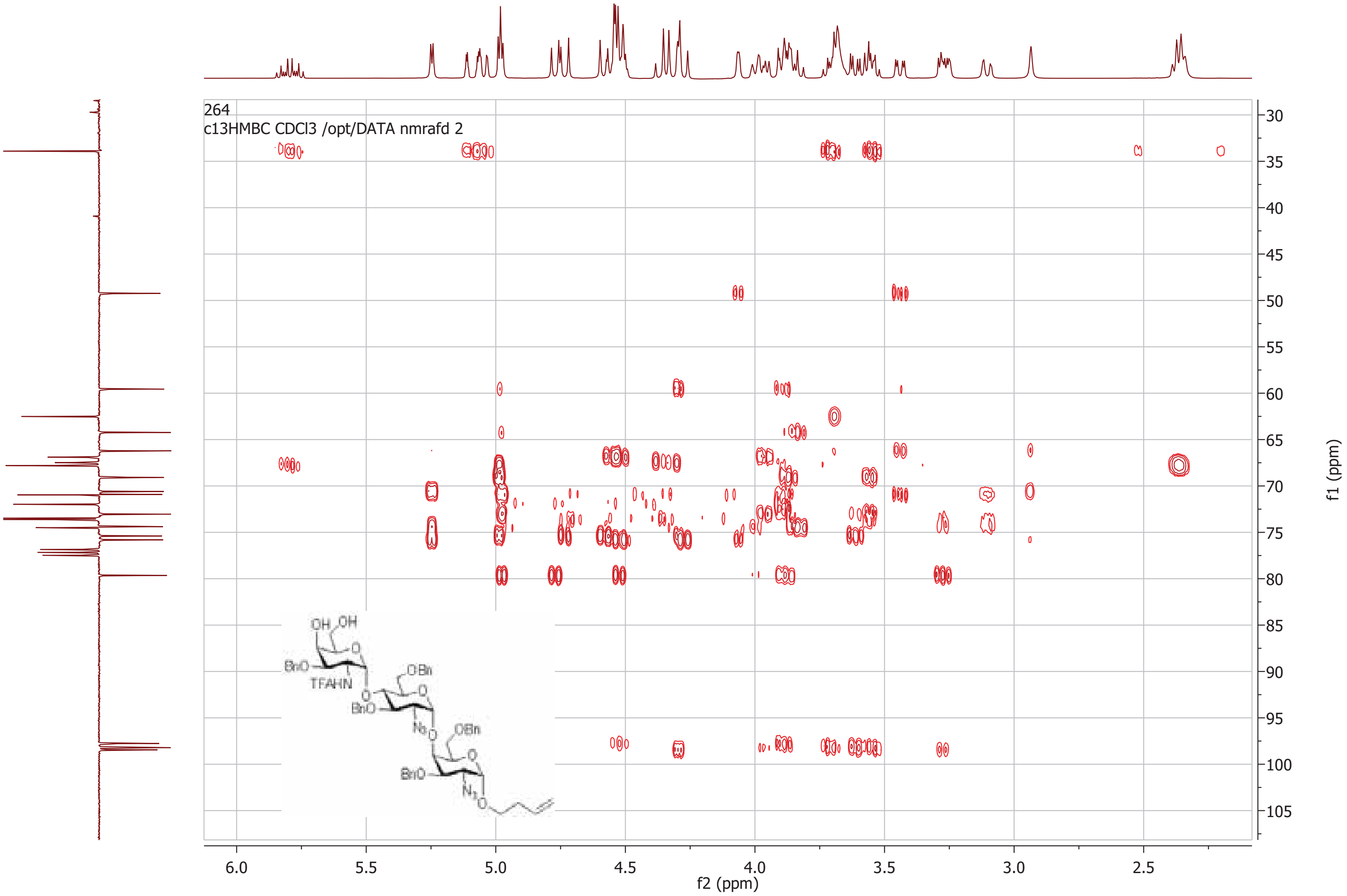
264
c13HMBC CDCl3 /opt/DATA nmrafd 2
f2 (ppm)
f1 (ppm)
OH OH
BnO
TFAHN
OBn
BnO
N3
OBn
BnO
N3

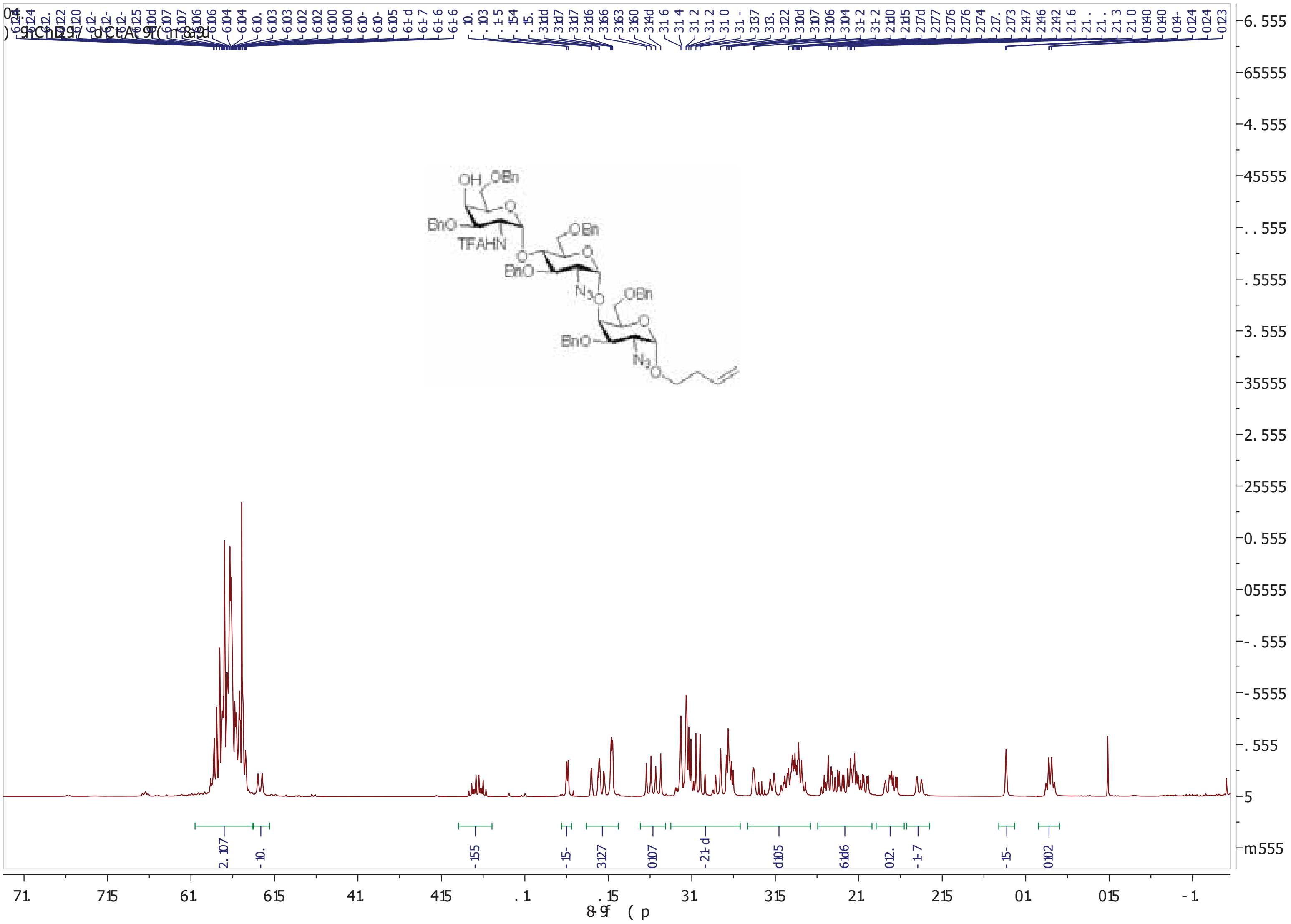
OH
OBn
BnO
TFAHN
OBn
BnO
N3
OBn
BnO
N3

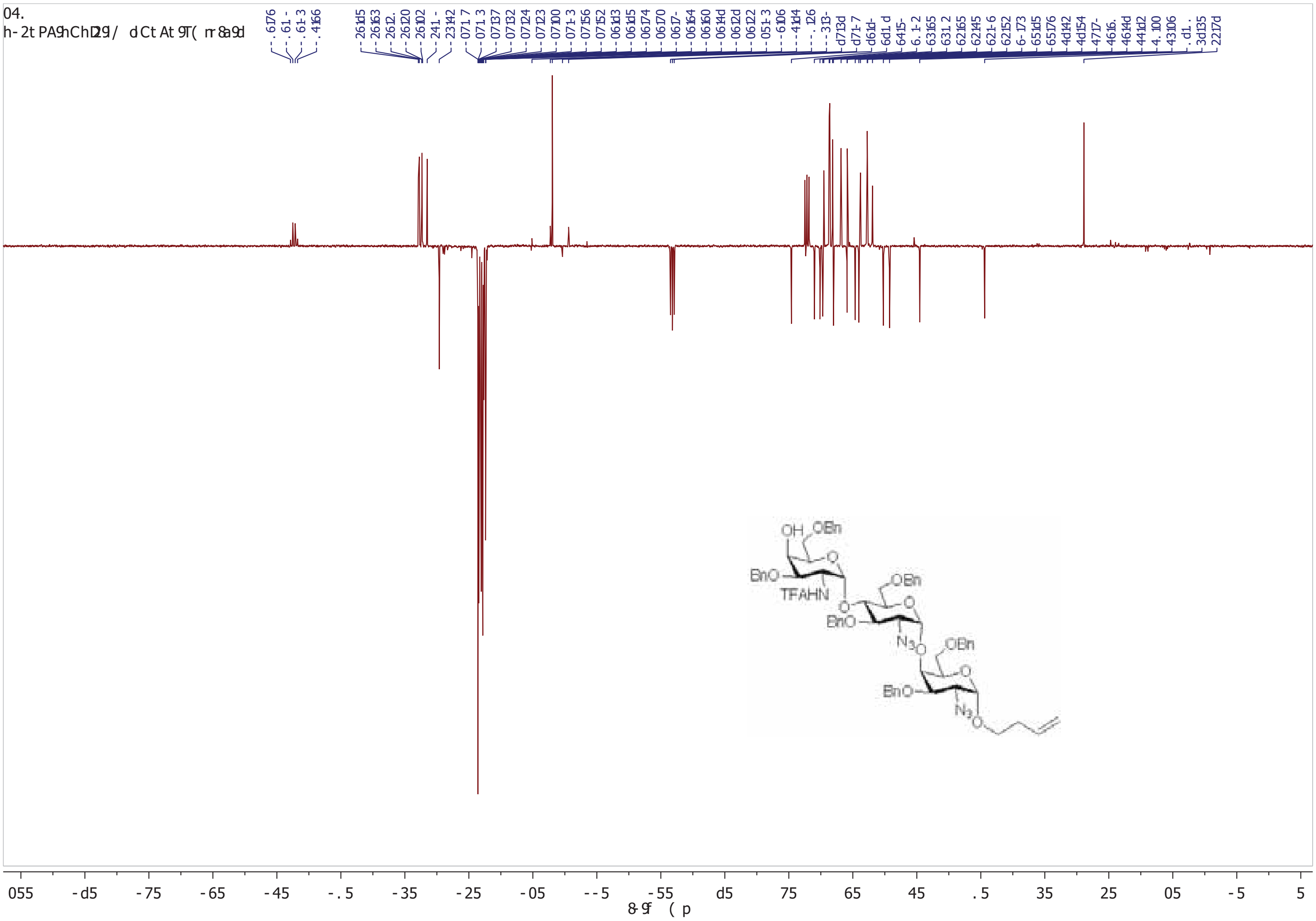
OH
OBn
BnO
TFAHN
OBn
BnO
N3
OBn
BnO
N3

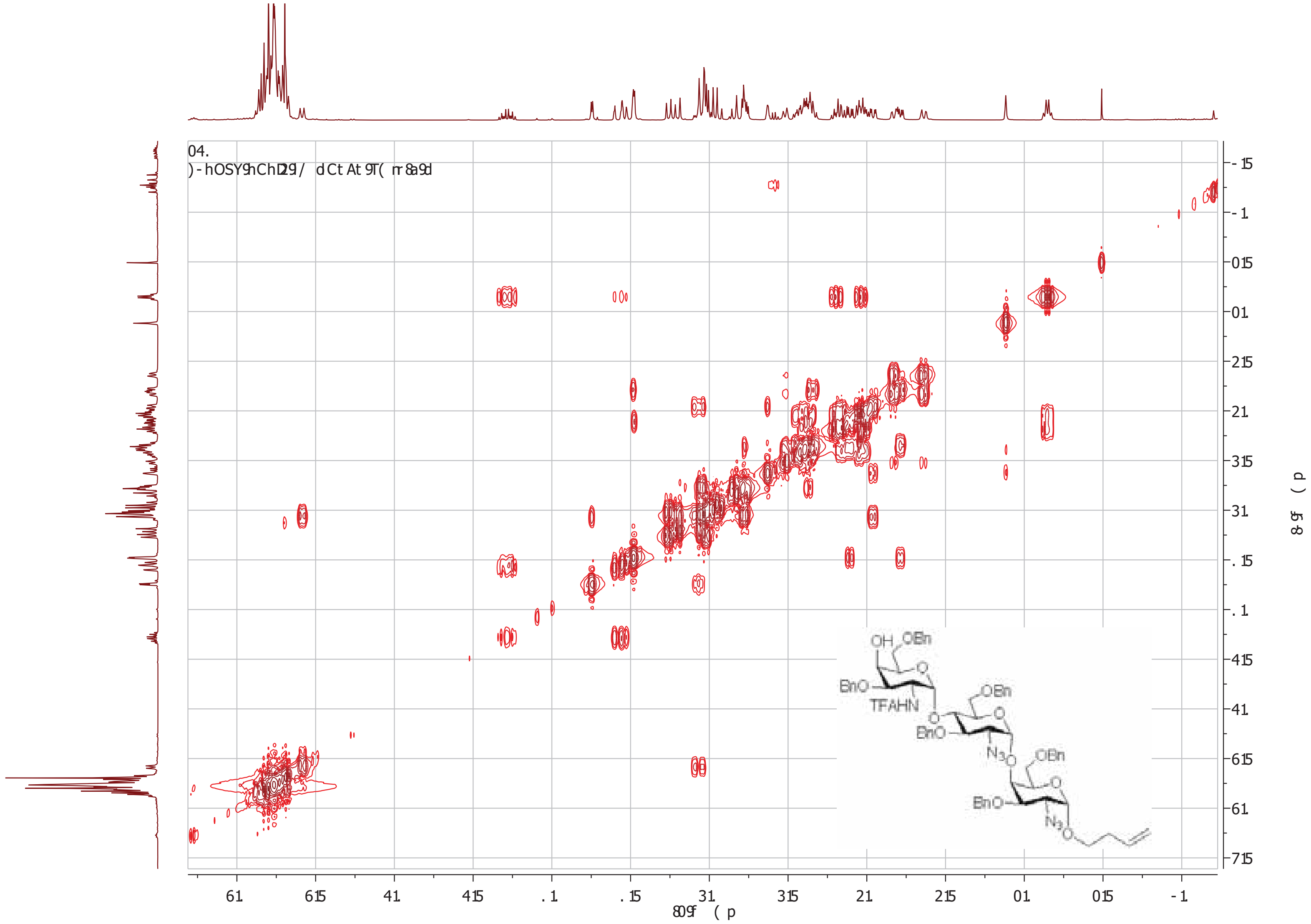
OH
OBn
BnO
TFAHN
OBn
BnO
N3
OBn
BnO
N3

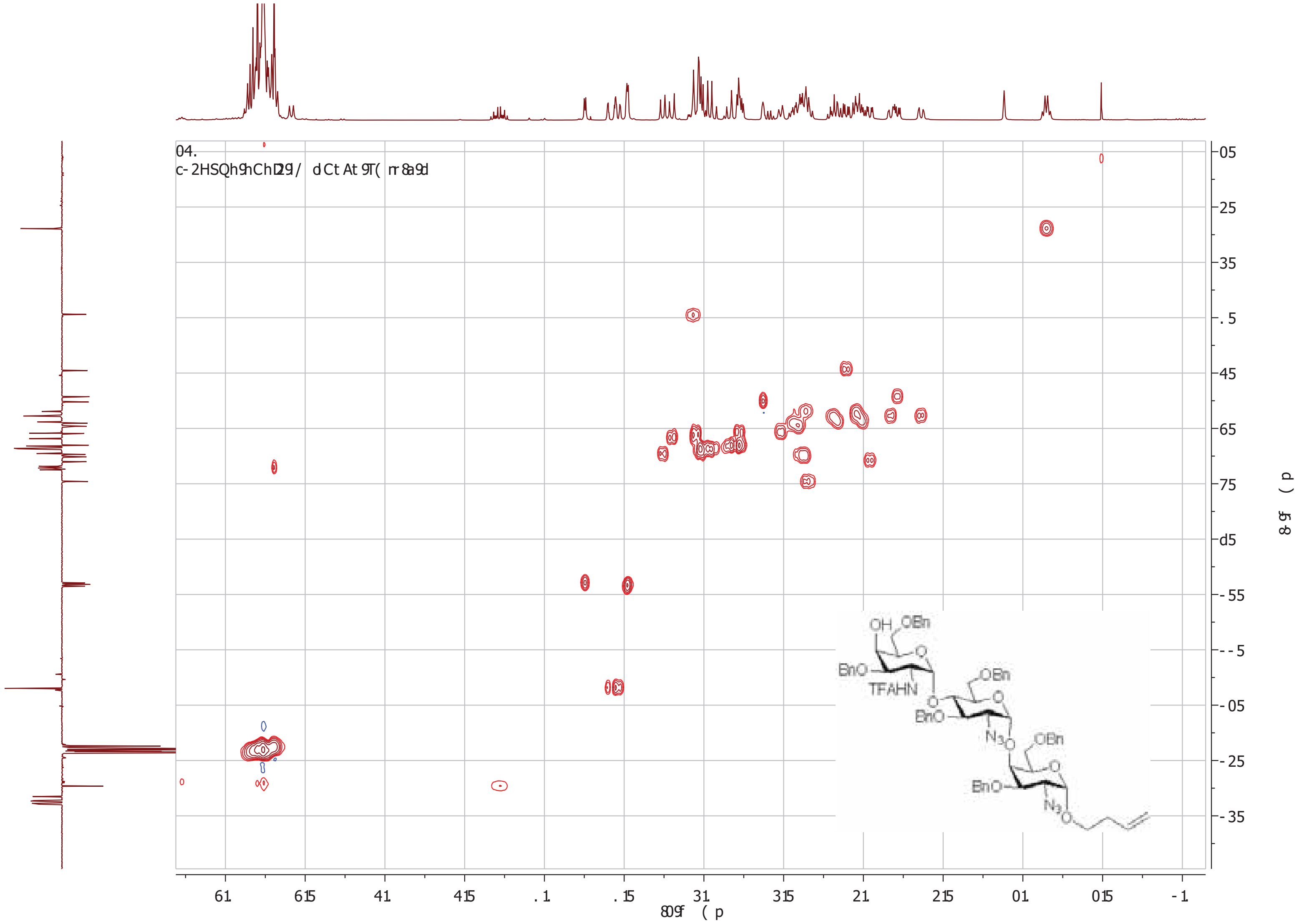
OH
OBn
BnO
TFAHN
OBn
BnO
N3
OBn
BnO
N3

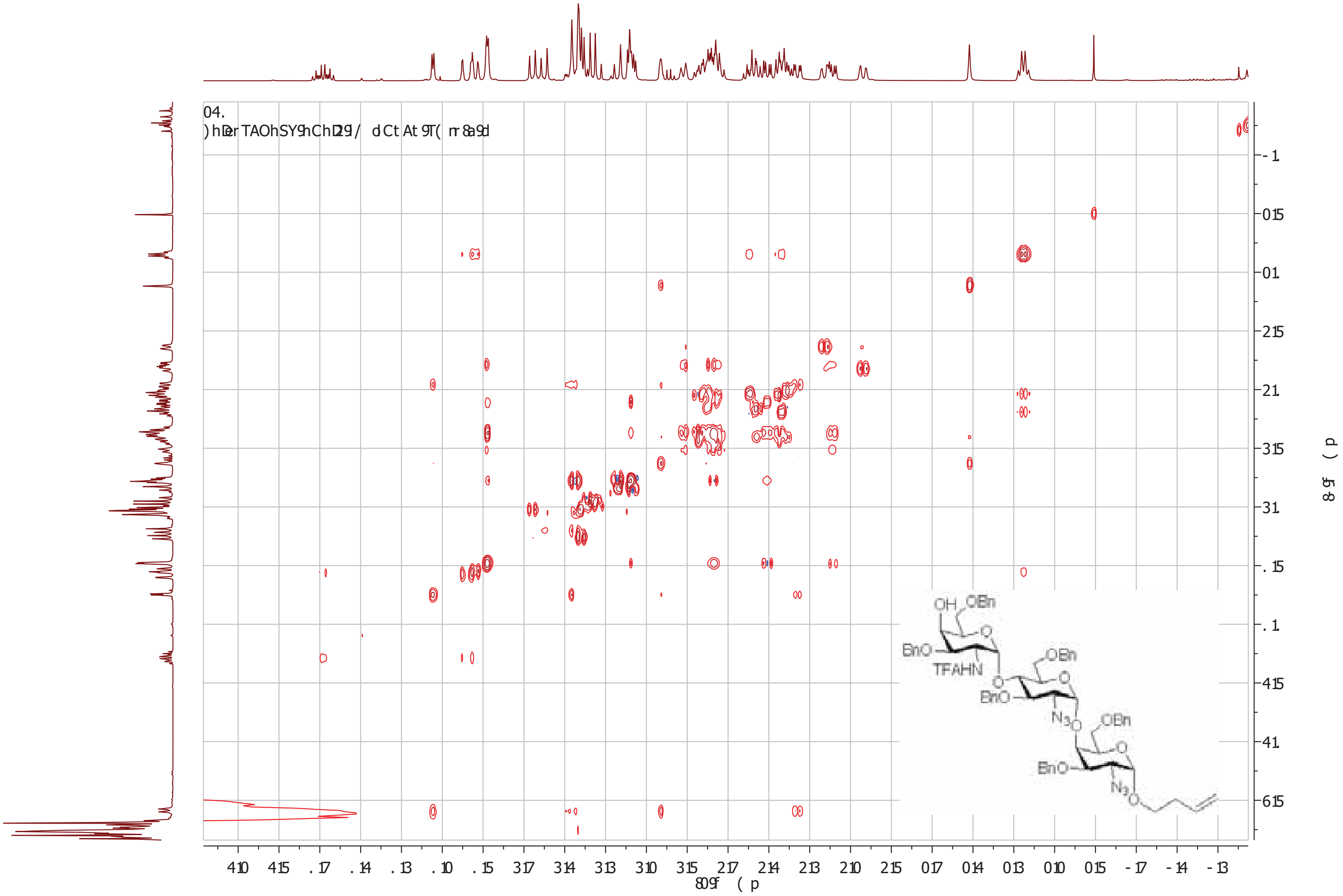
OH
OBn
BnO
TFAHN
OBn
BnO
N3
OBn
BnO
N3

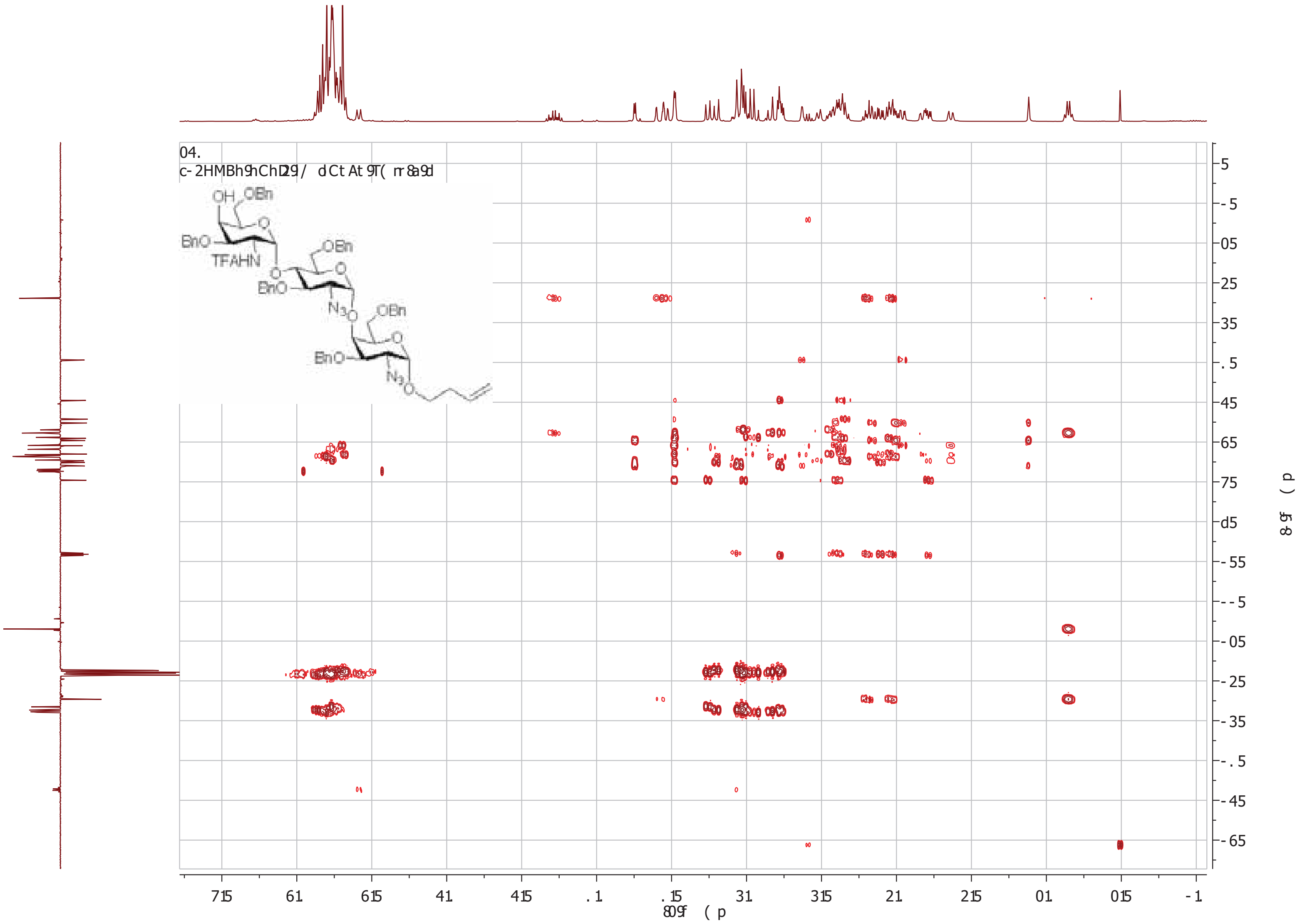
OH
OBn
BnO
TFAHN
O
OBn
BnO
N3
O
OBn
BnO
N3
O

266

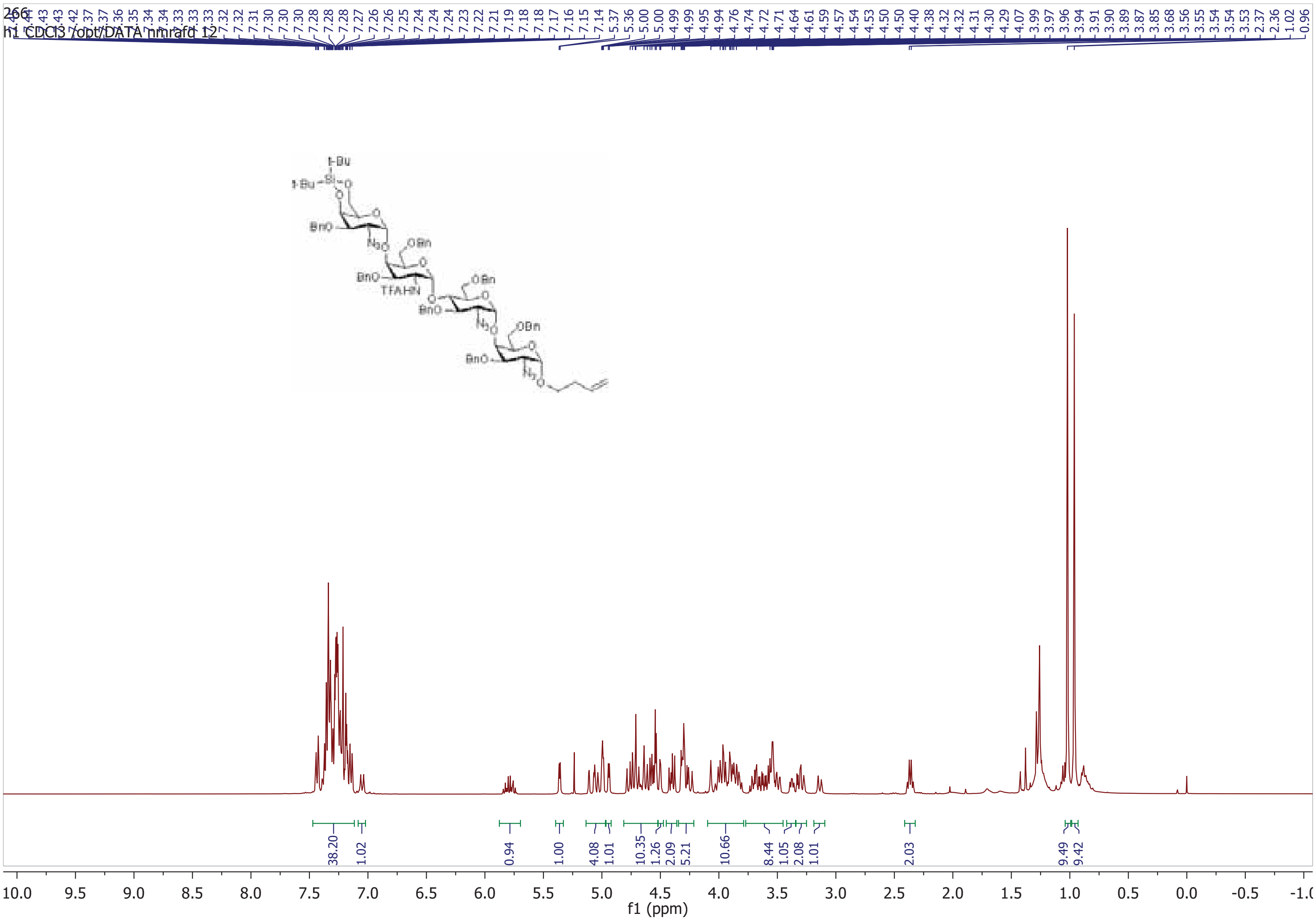

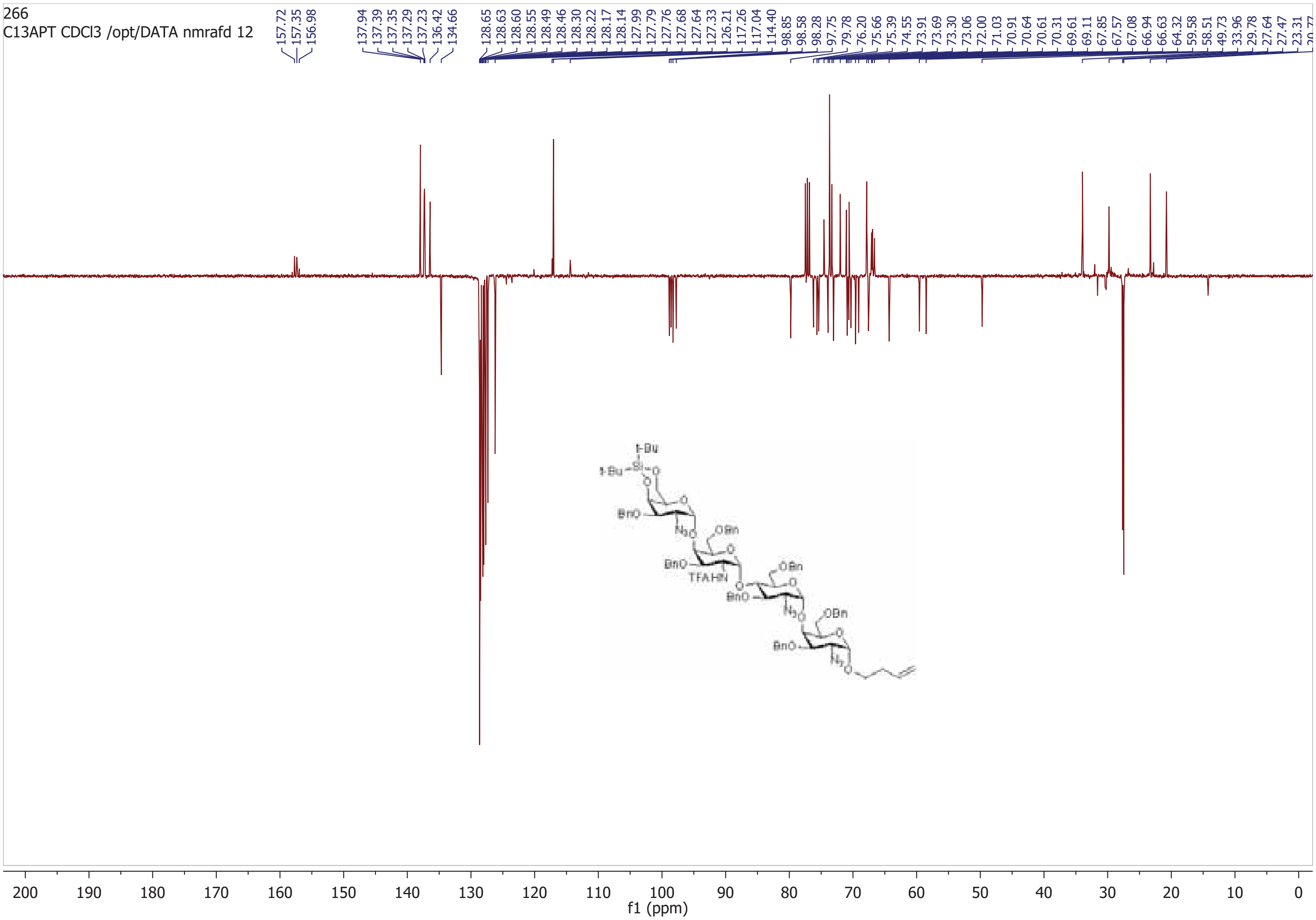
266
C13APT CDCl3 /opt/DATA nmrafd 12
157.72
157.35
156.98
137.94
137.39
137.35
137.29
137.23
136.42
134.66
128.65
128.63
128.60
128.55
128.49
128.46
128.30
128.22
128.17
128.14
127.99
127.79
127.76
127.68
127.64
127.33
126.21
117.26
117.04
114.40
98.85
98.58
98.28
97.75
79.78
76.20
75.66
75.39
74.55
73.91
73.69
73.30
73.06
72.00
71.03
70.91
70.64
70.61
70.31
69.61
69.11
67.85
67.57
67.08
66.94
66.63
64.32
59.58
58.51
49.73
33.96
29.78
27.64
27.47
23.31
t-Bu
t-Bu
Si
BnO
N3
OBn
BnO
TFAHN
OBn
BnO
N3
OBn
BnO
N3
200
190
180
170
160
150
140
130
120
110
100
90
80
70
60
50
40
30
20
10
0
f1 (ppm)

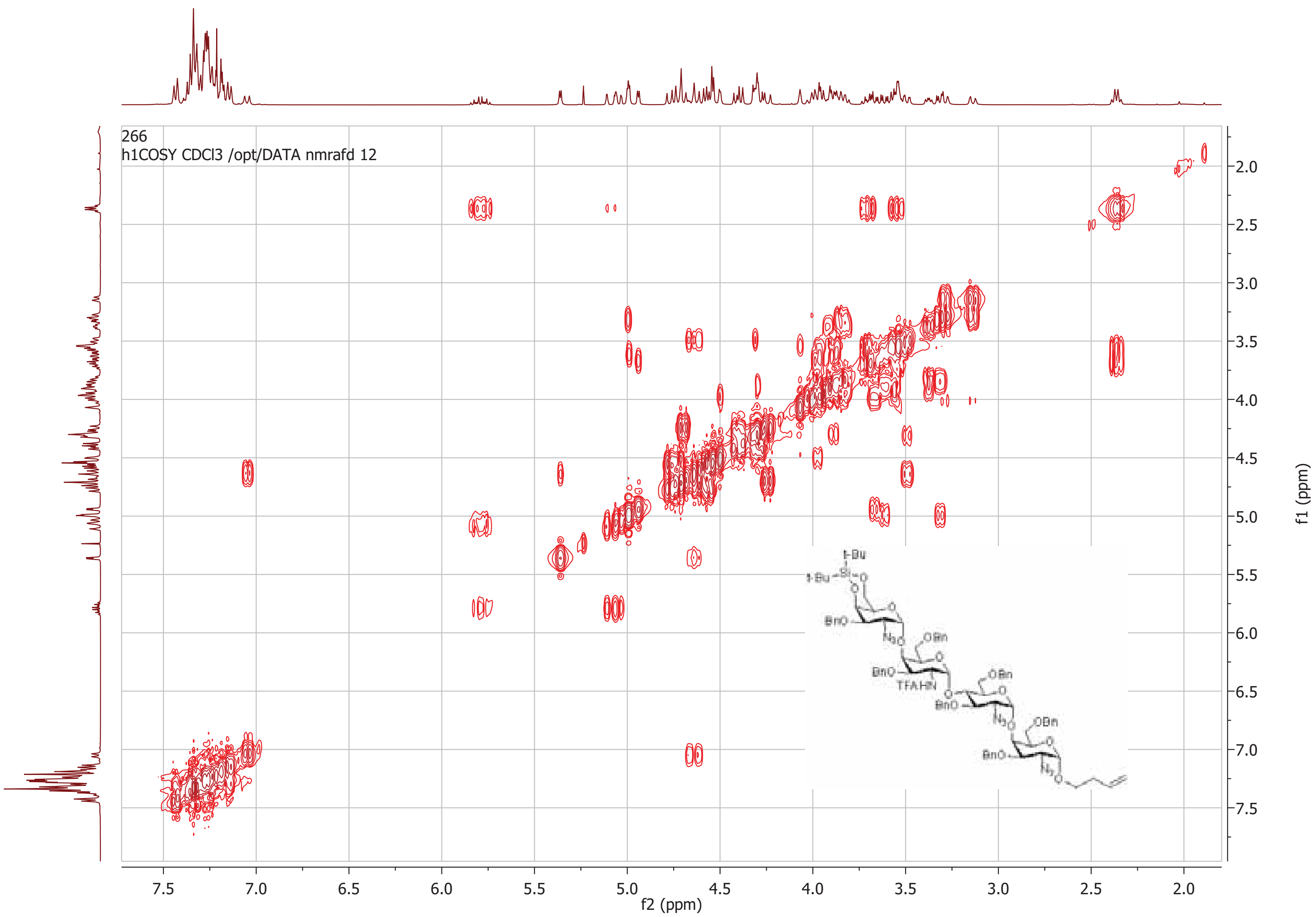

266
h1COSY CDCl3 /opt/DATA nmrafd 12
f2 (ppm)
f1 (ppm)
t-Bu
BnO
TFAHN
OBn

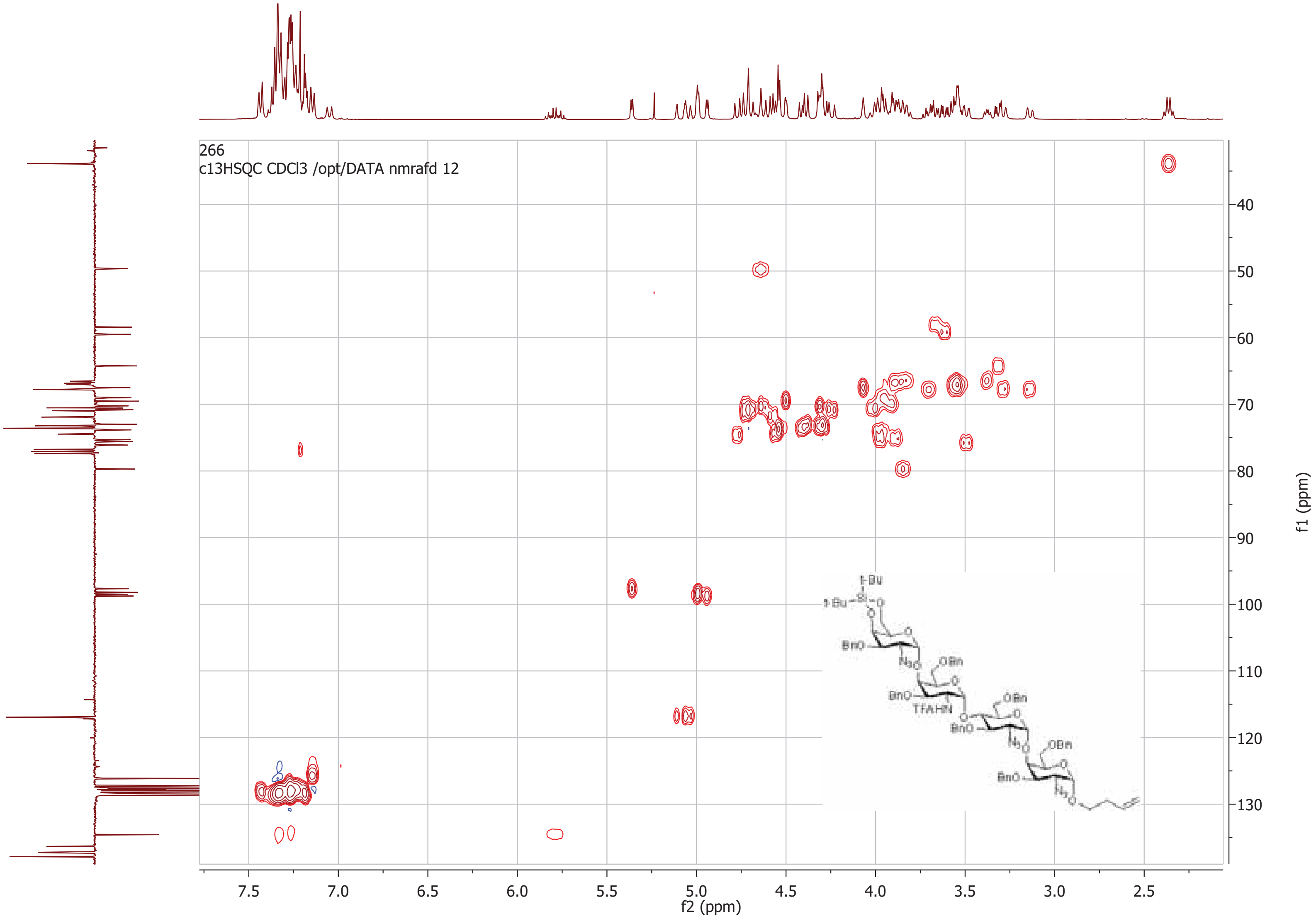
266
c13HSQC CDCl3 /opt/DATA nmrafd 12
f1 (ppm)
f2 (ppm)
t-Bu
t-Bu
Si
BnO
N3
OBn
BnO
TFAHN
OBn
BnO
N3
OBn
BnO
N3

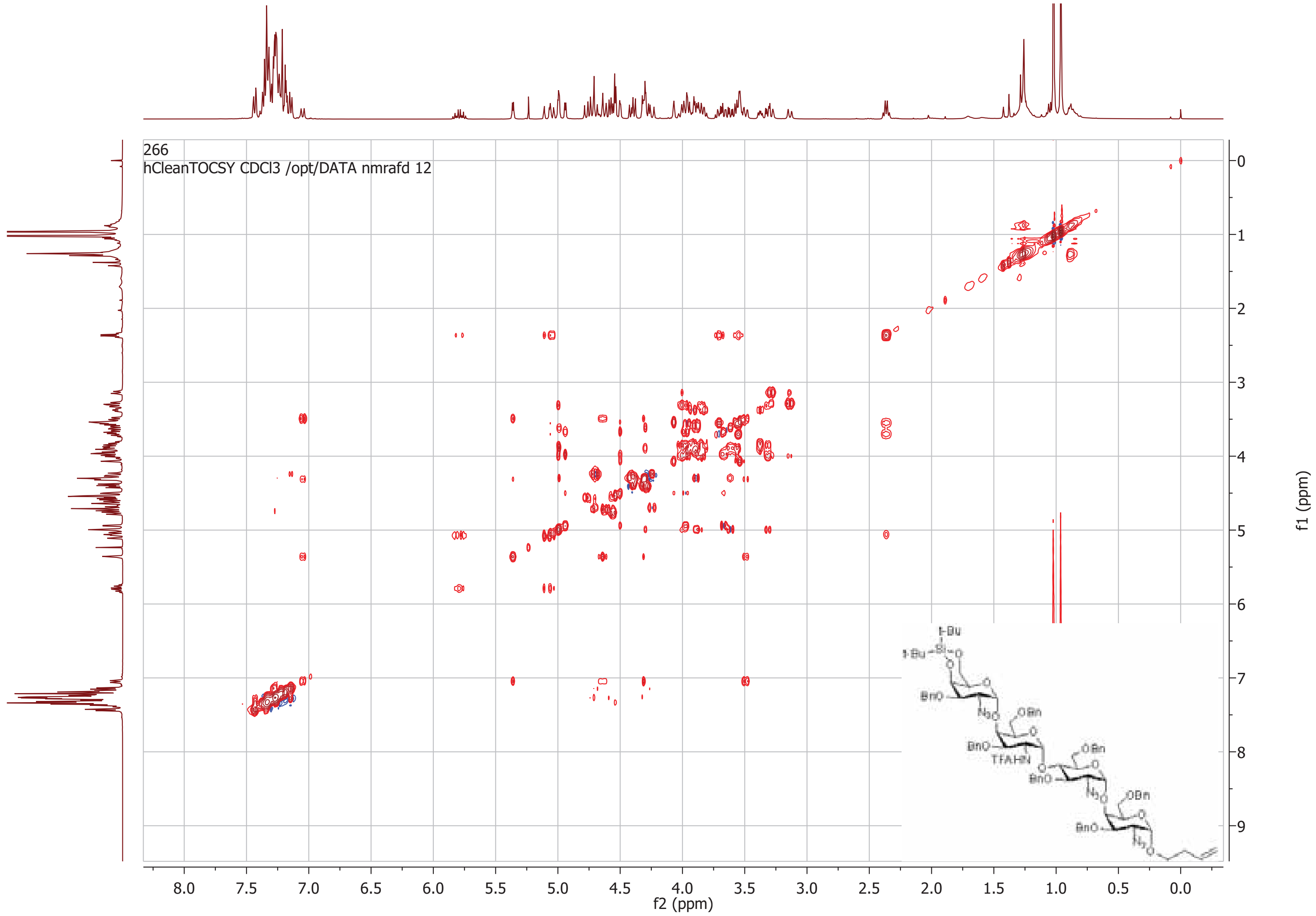
266
hCleanTOCSY CDCl3 /opt/DATA nmrafd 12
f2 (ppm)
f1 (ppm)
t-Bu
t-Bu
Si
BnO
N3
OBn
BnO
TFAHN
OBn
BnO
N3
OBn
BnO
N3

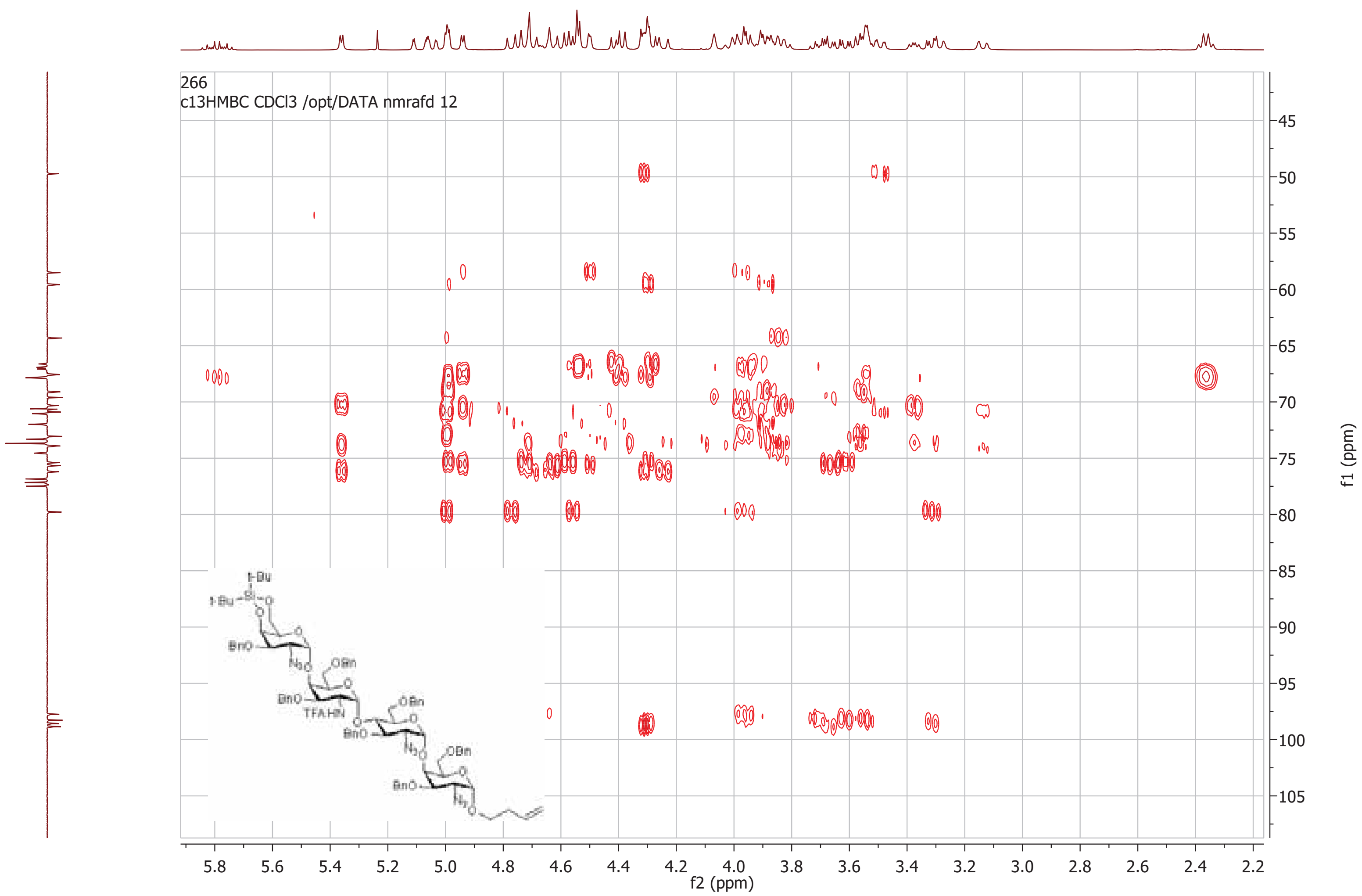
266
c13HMBC CDCl3 /opt/DATA nmrafd 12
f2 (ppm)
f1 (ppm)
t-Bu
Si
O
BnO
N3
OBn
TFAHN
N3
OBn
OBn

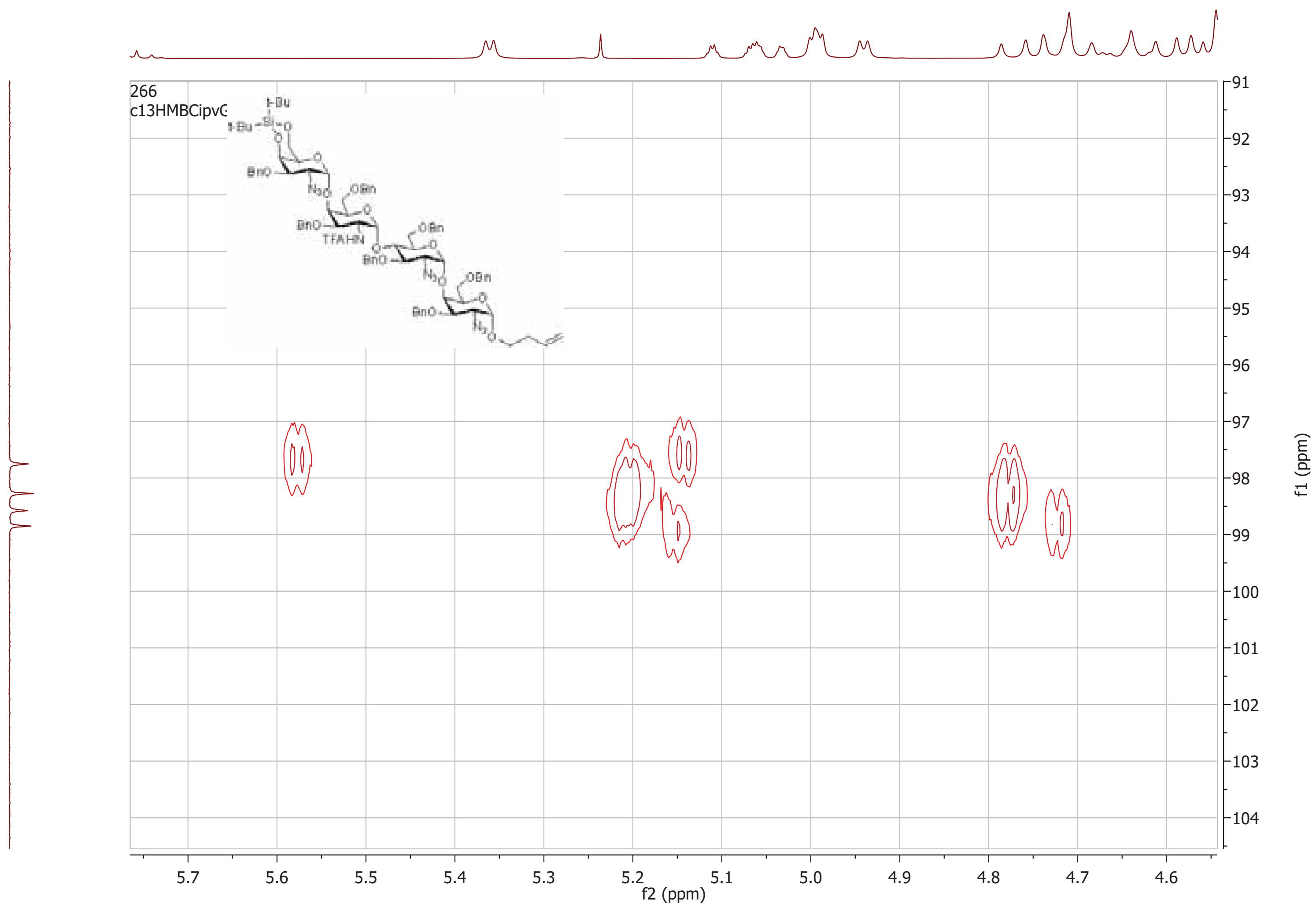
266
c13HMBCipvG
t-Bu
t-Bu
Si
BnO
N3
OBn
BnO
TFAHN
OBn
BnO
N3
OBn
BnO
N3
f2 (ppm)
f1 (ppm)

267

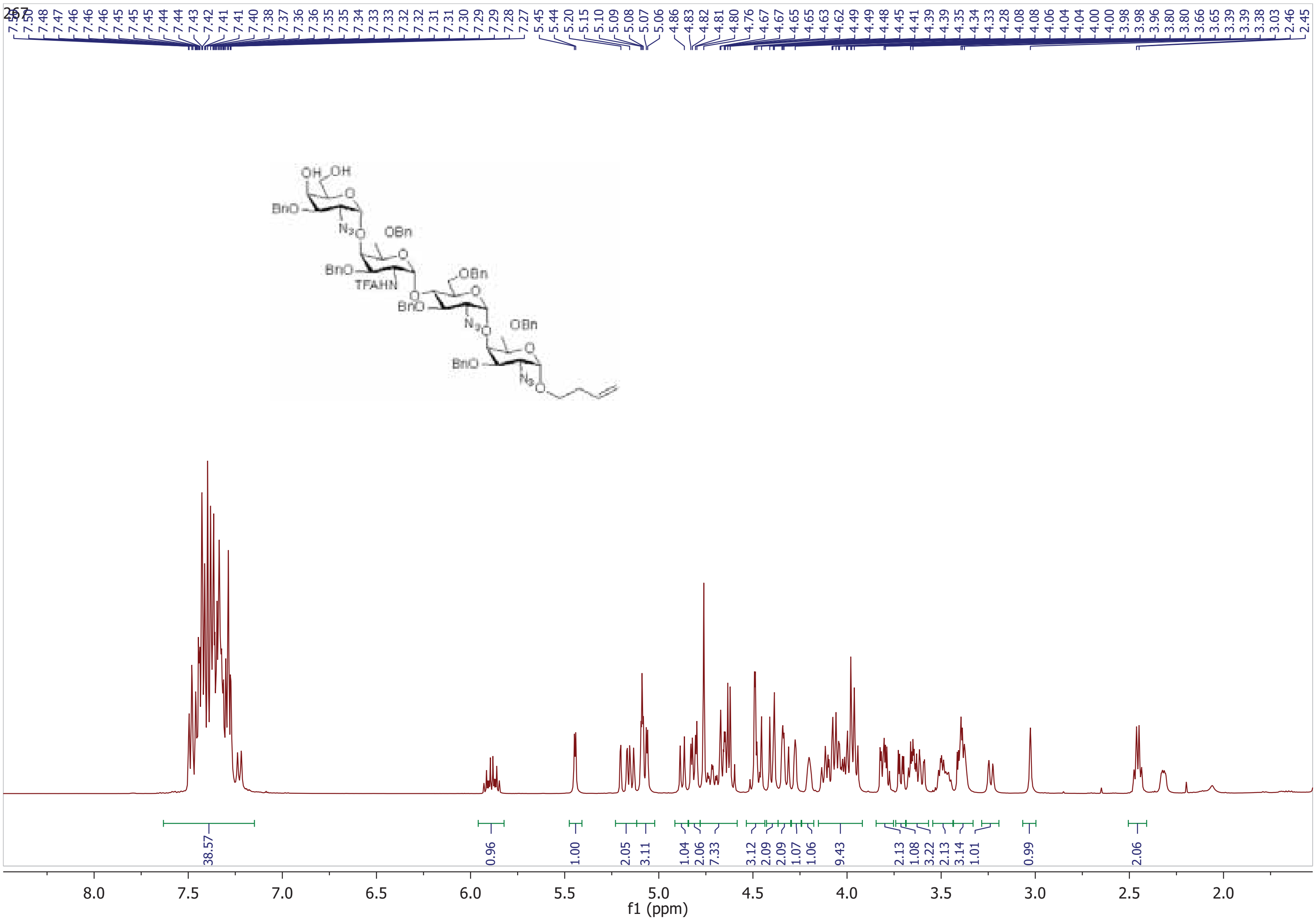
7.50 7.48 7.47 7.46 7.46 7.45 7.45 7.44 7.44 7.43 7.42 7.41 7.41 7.40 7.38 7.37 7.36 7.36 7.35 7.35 7.34 7.33 7.33 7.32 7.32 7.31 7.30 7.29 7.28 7.27 5.45 5.44 5.20 5.15 5.10 5.09 5.08 5.07 5.06 4.86 4.83 4.82 4.81 4.80 4.76 4.67 4.67 4.65 4.65 4.63 4.62 4.49 4.49 4.48 4.45 4.41 4.39 4.39 4.35 4.34 4.33 4.28 4.08 4.08 4.06 4.04 4.04 4.00 4.00 3.98 3.98 3.96 3.80 3.80 3.66 3.65 3.39 3.39 3.38 3.03 2.46 2.45
OH OH
BnO
N3
OBn
BnO
TFAHN
OBn
BnO
N3
OBn
BnO
N3
38.57 0.96 1.00 2.05 3.11 1.04 2.06 7.33 3.12 2.09 2.09 1.07 1.06 9.43 2.13 1.08 3.22 2.13 3.14 1.01 0.99 2.06
8.0 7.5 7.0 6.5 6.0 5.5 5.0 4.5 4.0 3.5 3.0 2.5 2.0
f1 (ppm)

267
13C APT, bbo, av500,

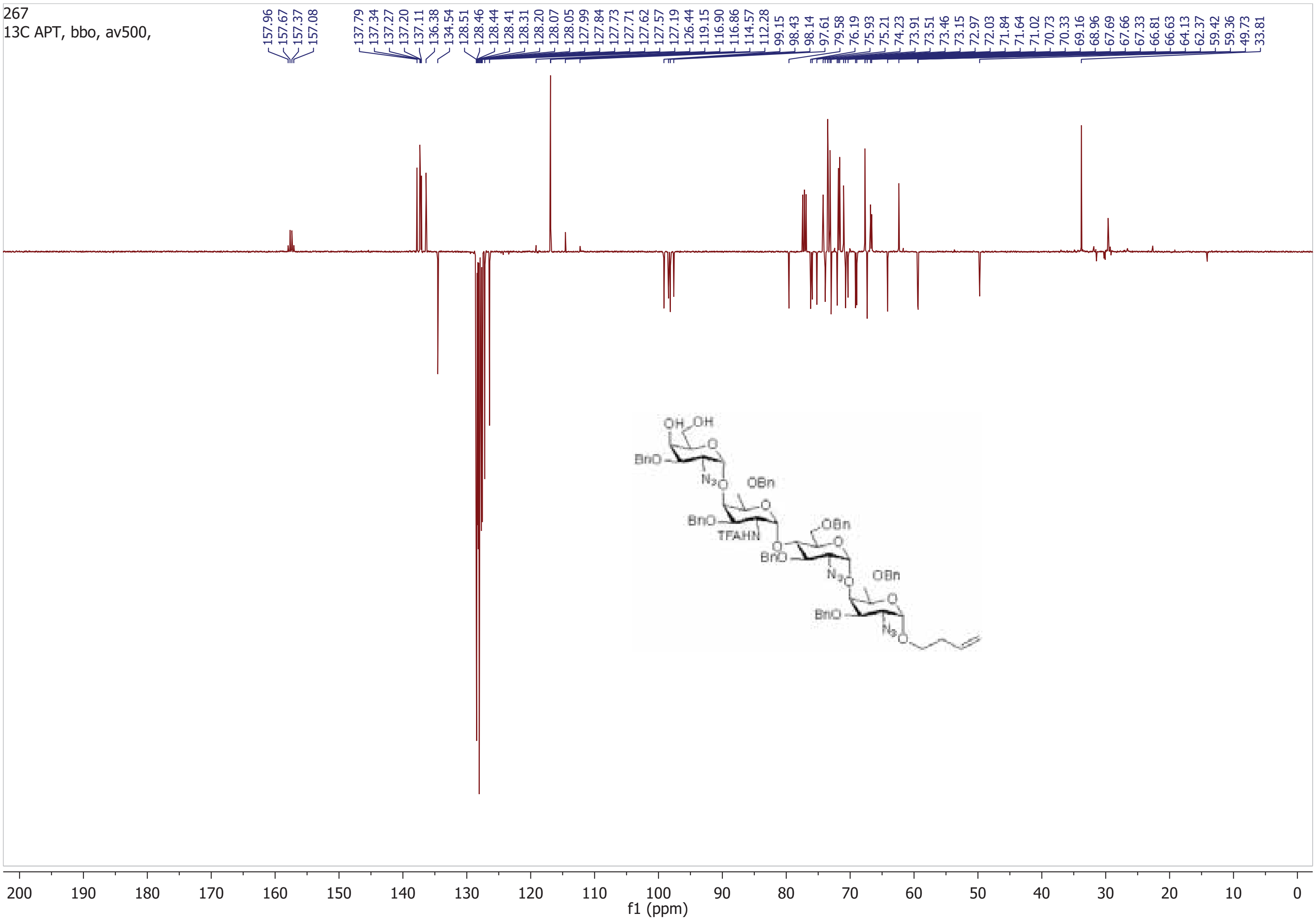

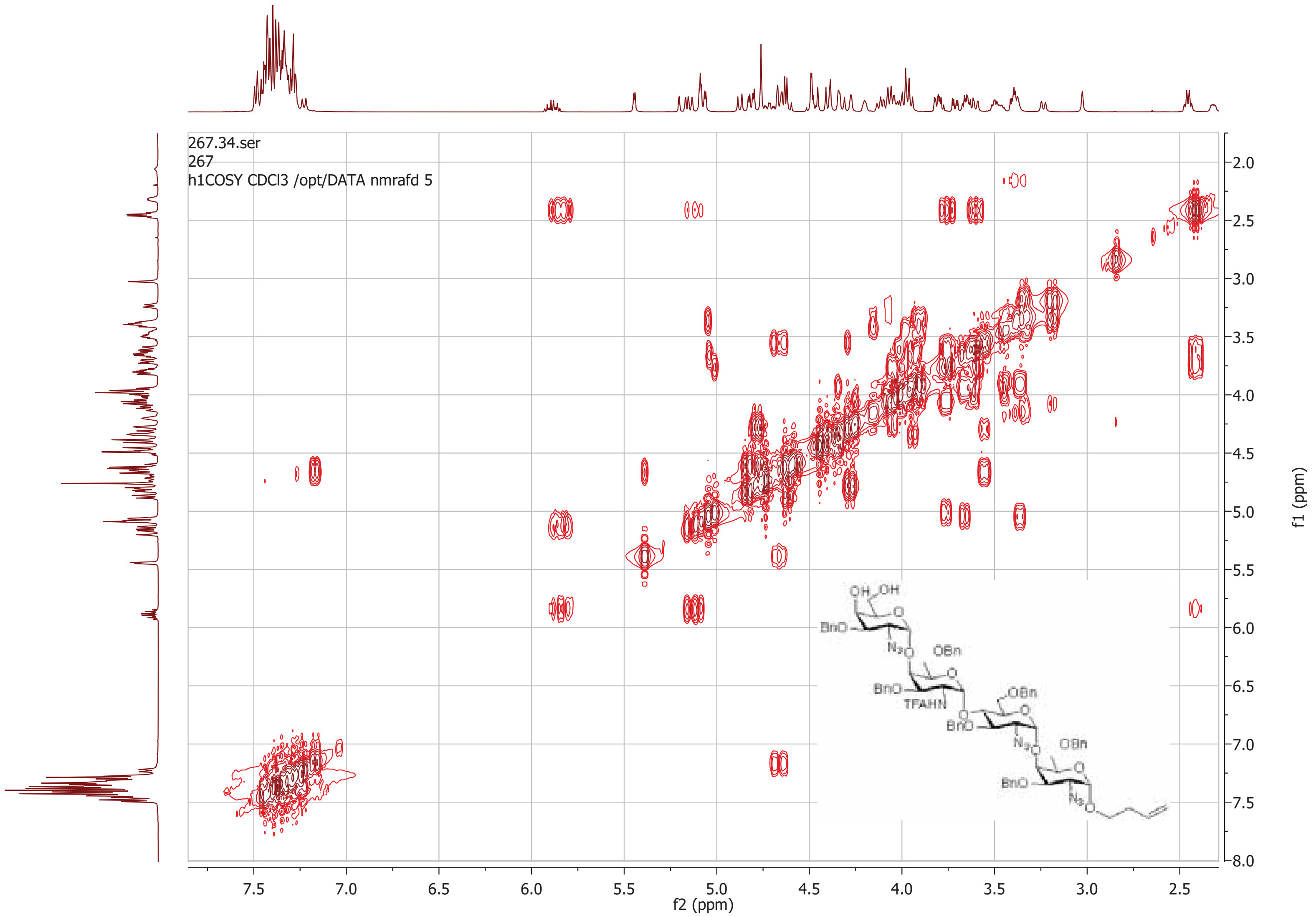

267.34.ser
267
h1COSY CDCl3 /opt/DATA nmrafd 5
f2 (ppm)
f1 (ppm)
OH
OH
BnO
N3
OBn
BnO
TFAHN
OBn
BnO
N3
OBn
BnO
N3

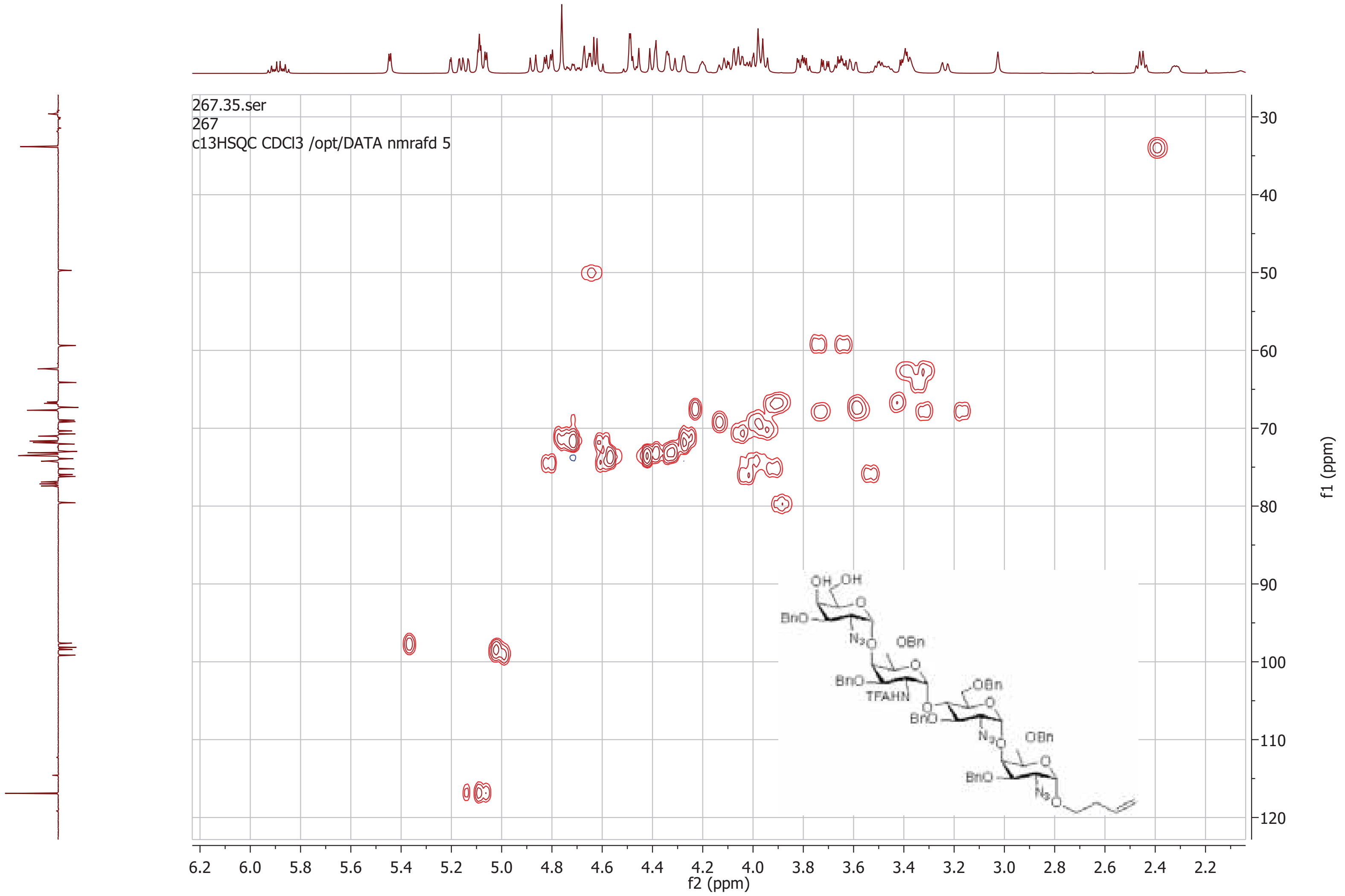
267.35.ser
267
c13HSQC CDCl3 /opt/DATA nmrafd 5
f2 (ppm)
f1 (ppm)
OH OH
BnO
N3
OBn
BnO
TFAHN
OBn
BnO
N3
OBn
BnO
N3

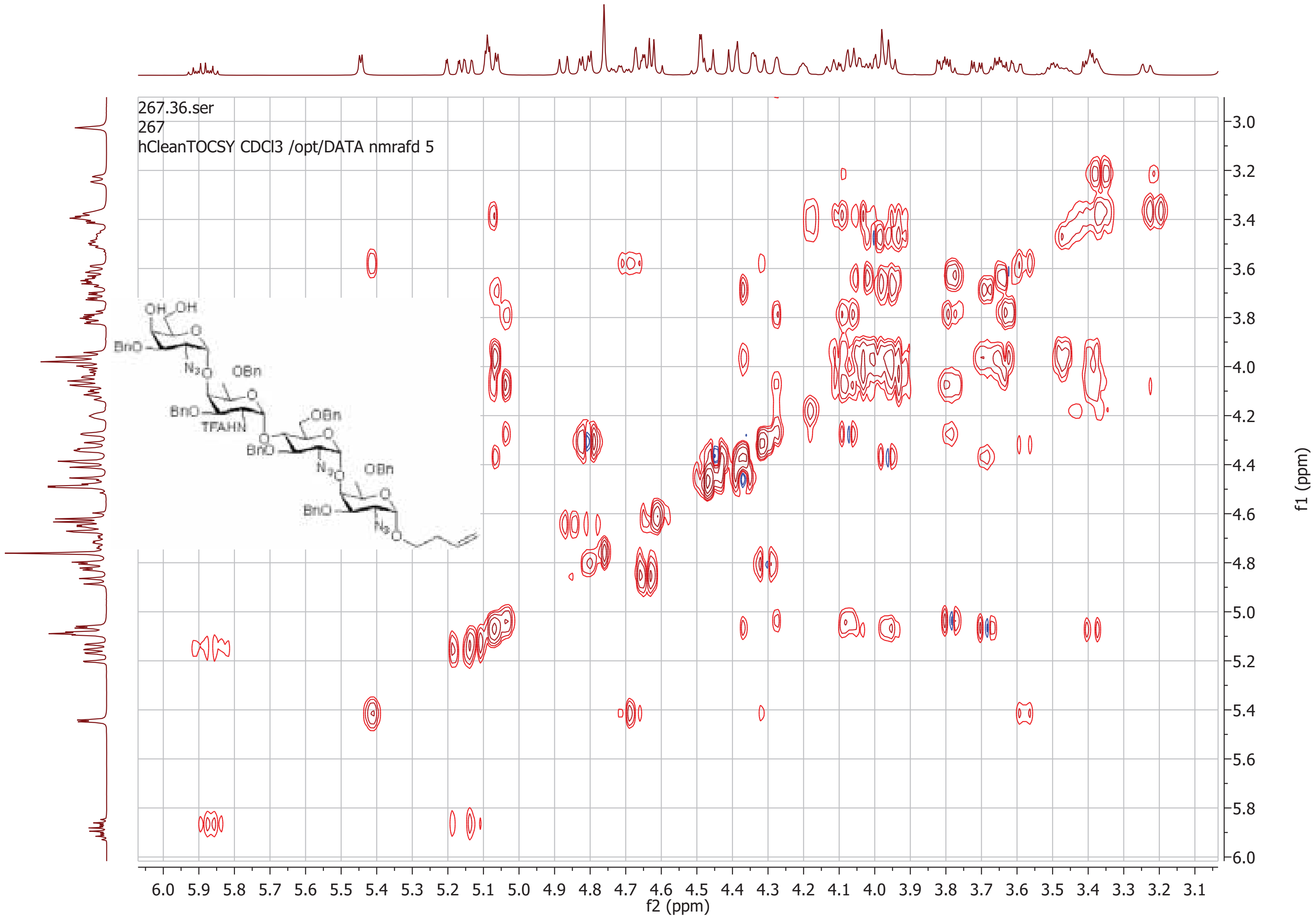
267.36.ser
267
hCleanTOCSY CDCl3 /opt/DATA nmrafd 5
OH OH
BnO
N3
OBn
BnO
TFAHN
OBn
BnO
N3
OBn
BnO
N3
f2 (ppm)
f1 (ppm)

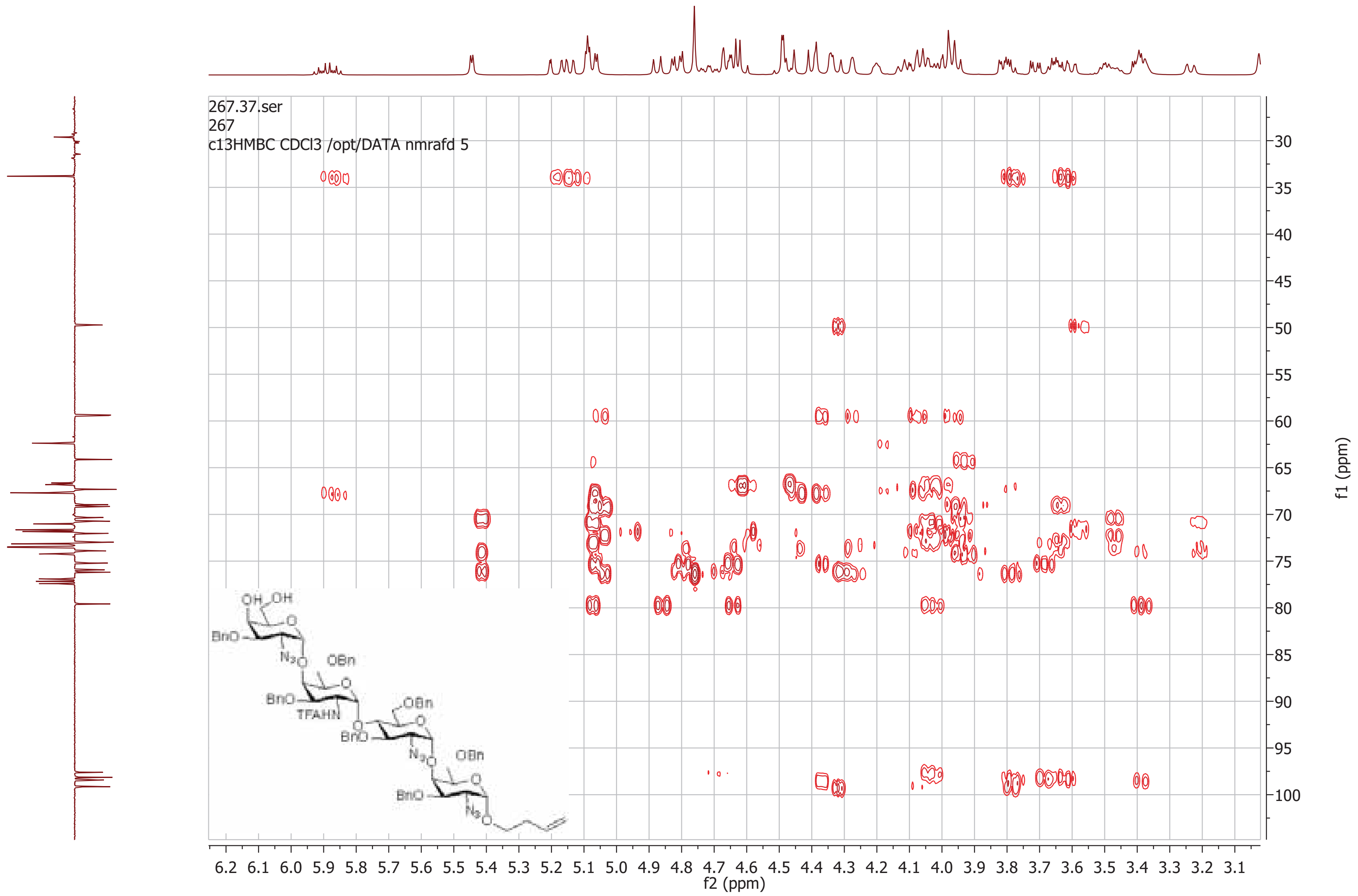
267.37.ser
267
c13HMBC CDCl3 /opt/DATA nmrafd 5
f2 (ppm)
f1 (ppm)
OH
OH
BnO
N_3
OBn
BnO
TFAHN
OBn
BnO
N_3
OBn
BnO
N_3

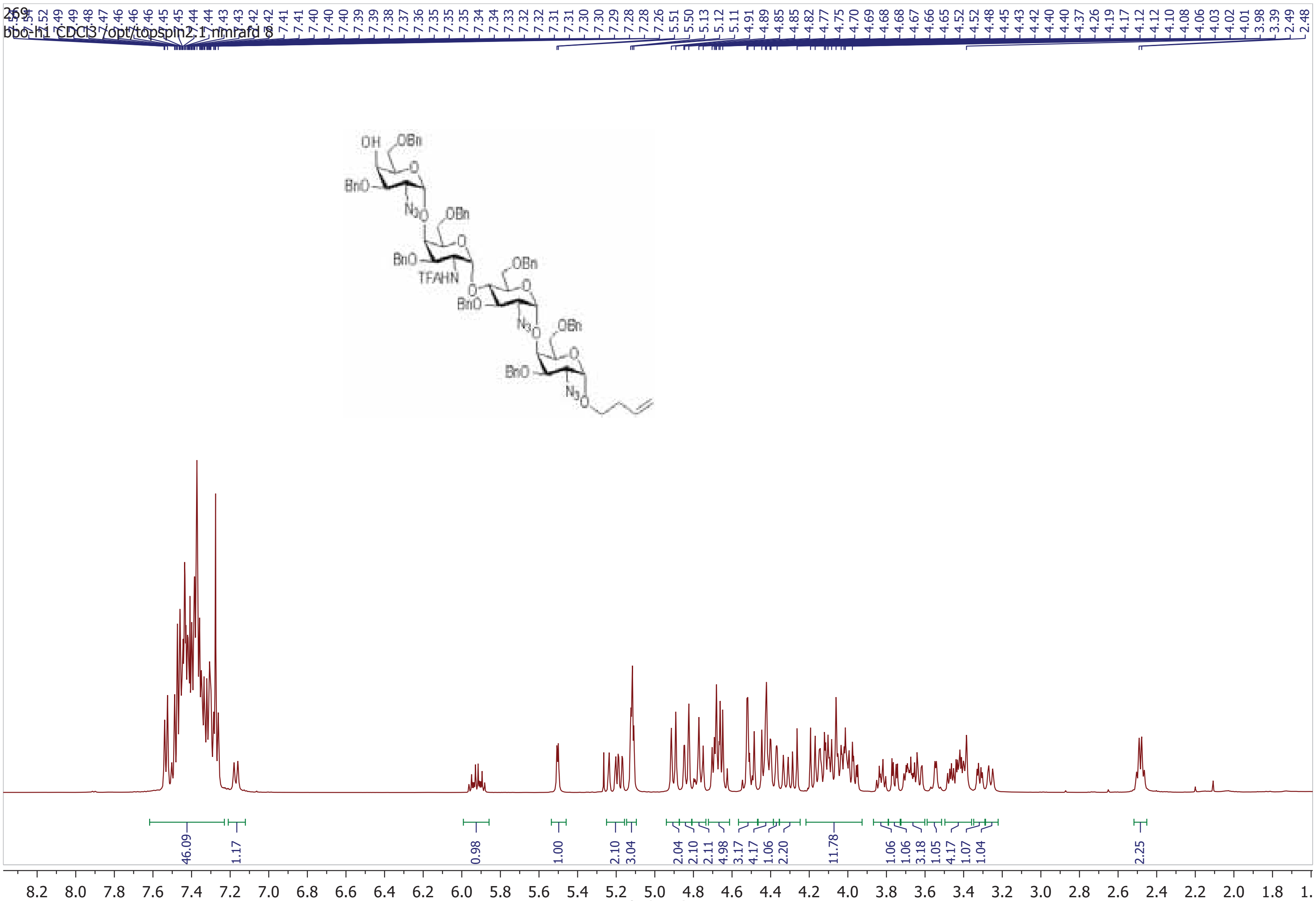

269
bbo-h1 CDCl3 /opt/topspin2.1 nmrafd 8
7.52 7.49 7.49 7.48 7.47 7.46 7.46 7.46 7.45 7.45 7.44 7.44 7.43 7.43 7.42 7.42 7.41 7.41 7.40 7.40 7.39 7.39 7.38 7.37 7.36 7.35 7.35 7.34 7.34 7.33 7.32 7.32 7.31 7.31 7.30 7.30 7.29 7.28 7.28 7.26 5.51 5.50 5.13 5.12 5.11 4.91 4.89 4.85 4.85 4.82 4.77 4.75 4.70 4.69 4.68 4.68 4.67 4.66 4.65 4.52 4.52 4.48 4.45 4.43 4.42 4.40 4.40 4.37 4.26 4.19 4.17 4.12 4.12 4.10 4.08 4.06 4.03 4.02 4.01 3.98 3.39 2.49 2.48
OH
OBn
BnO
N3
O
TFAHN
46.09
1.17
0.98
1.00
2.10
3.04
2.04
2.10
2.11
4.98
3.17
4.17
1.06
2.20
11.78
1.06
1.06
3.18
1.05
4.17
1.07
1.04
2.25
8.2 8.0 7.8 7.6 7.4 7.2 7.0 6.8 6.6 6.4 6.2 6.0 5.8 5.6 5.4 5.2 5.0 4.8 4.6 4.4 4.2 4.0 3.8 3.6 3.4 3.2 3.0 2.8 2.6 2.4 2.2 2.0 1.8
f1 (ppm)

269

bbo-c13-APT CDCl3 /opt/topspin2.1 nmrafd18

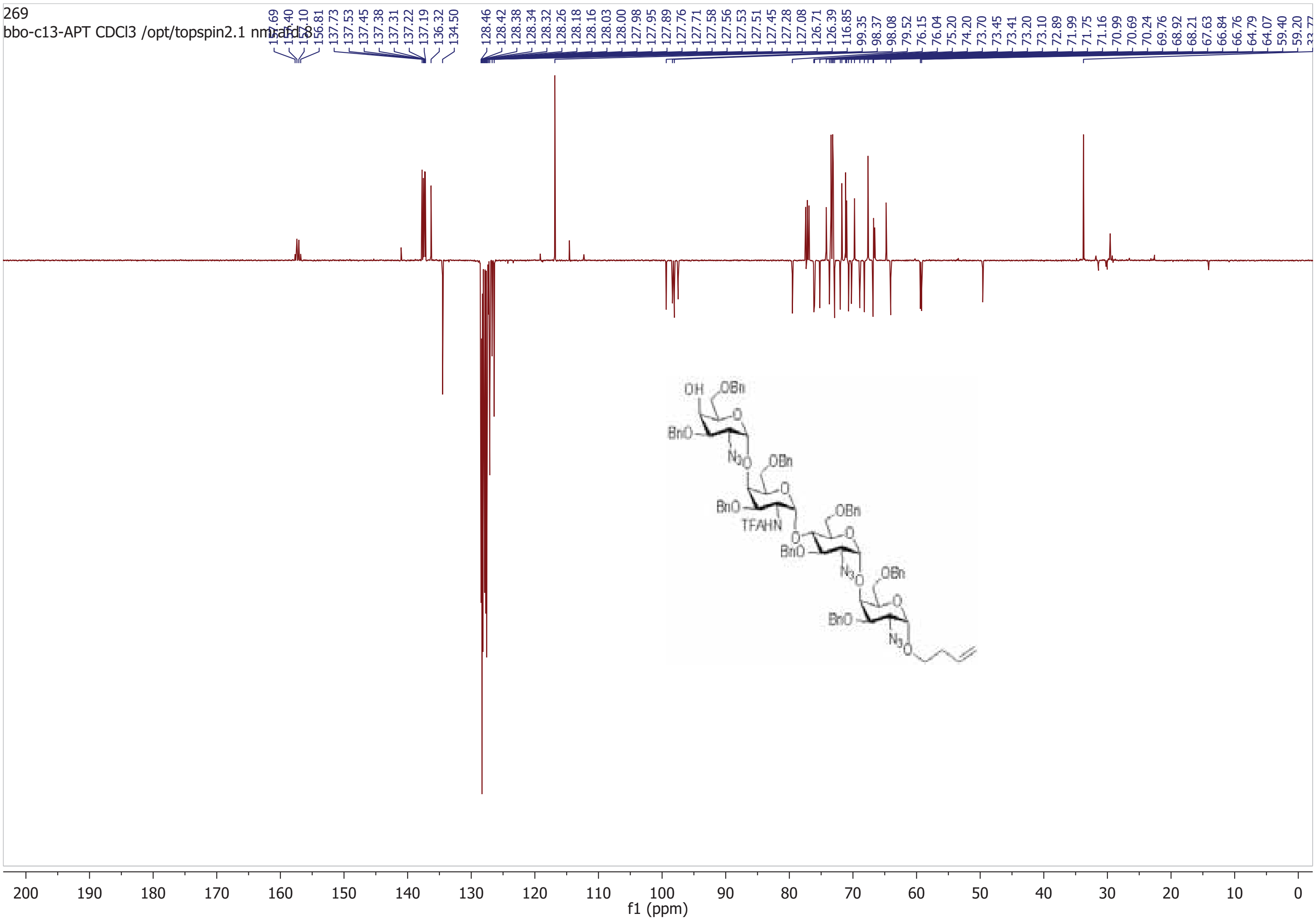

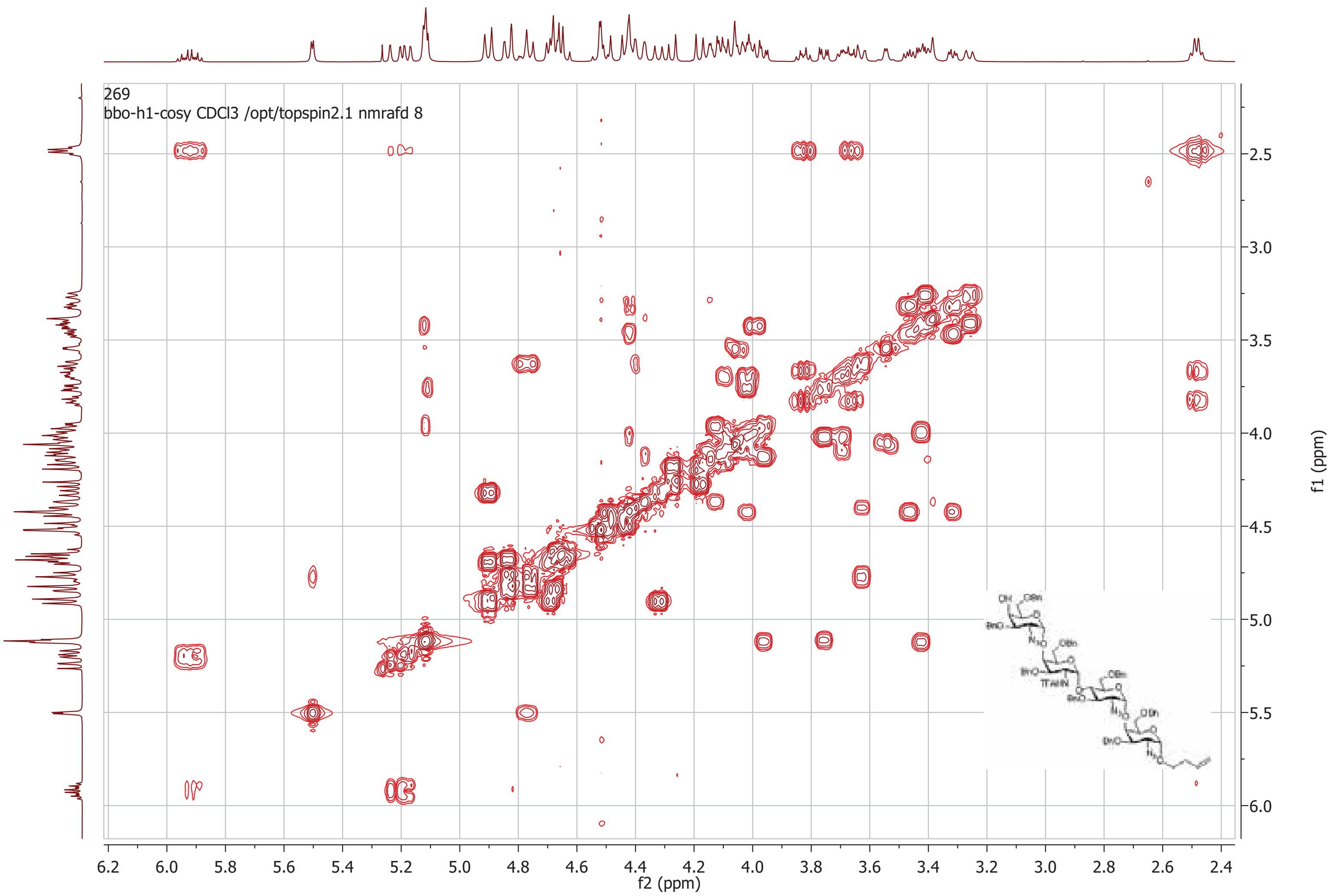

269
bbo-h1-cosy CDCl3 /opt/topspin2.1 nmrafd 8
f2 (ppm)
f1 (ppm)

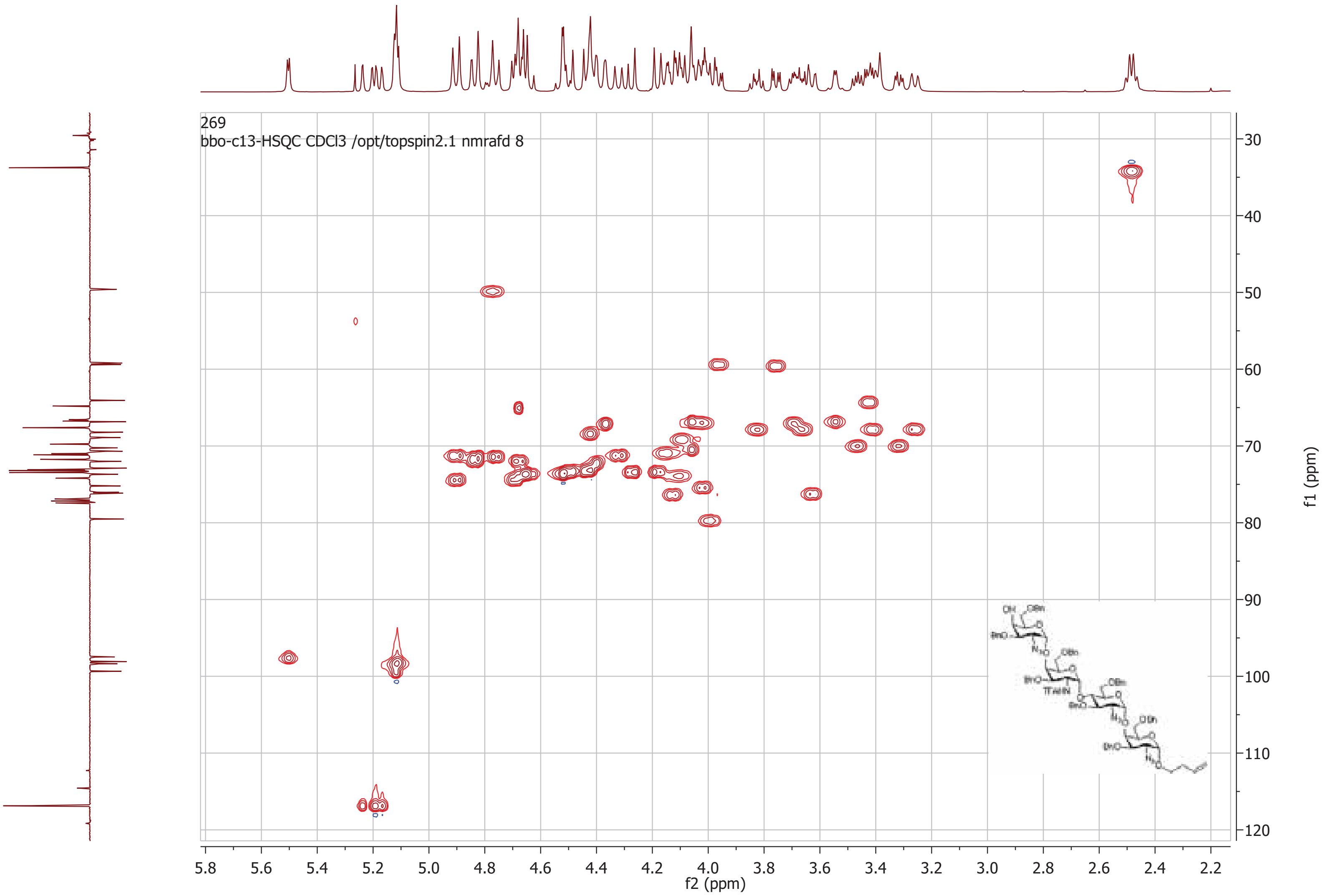
269
bbo-c13-HSQC CDCl3 /opt/topspin2.1 nmrafd 8
f2 (ppm)
f1 (ppm)

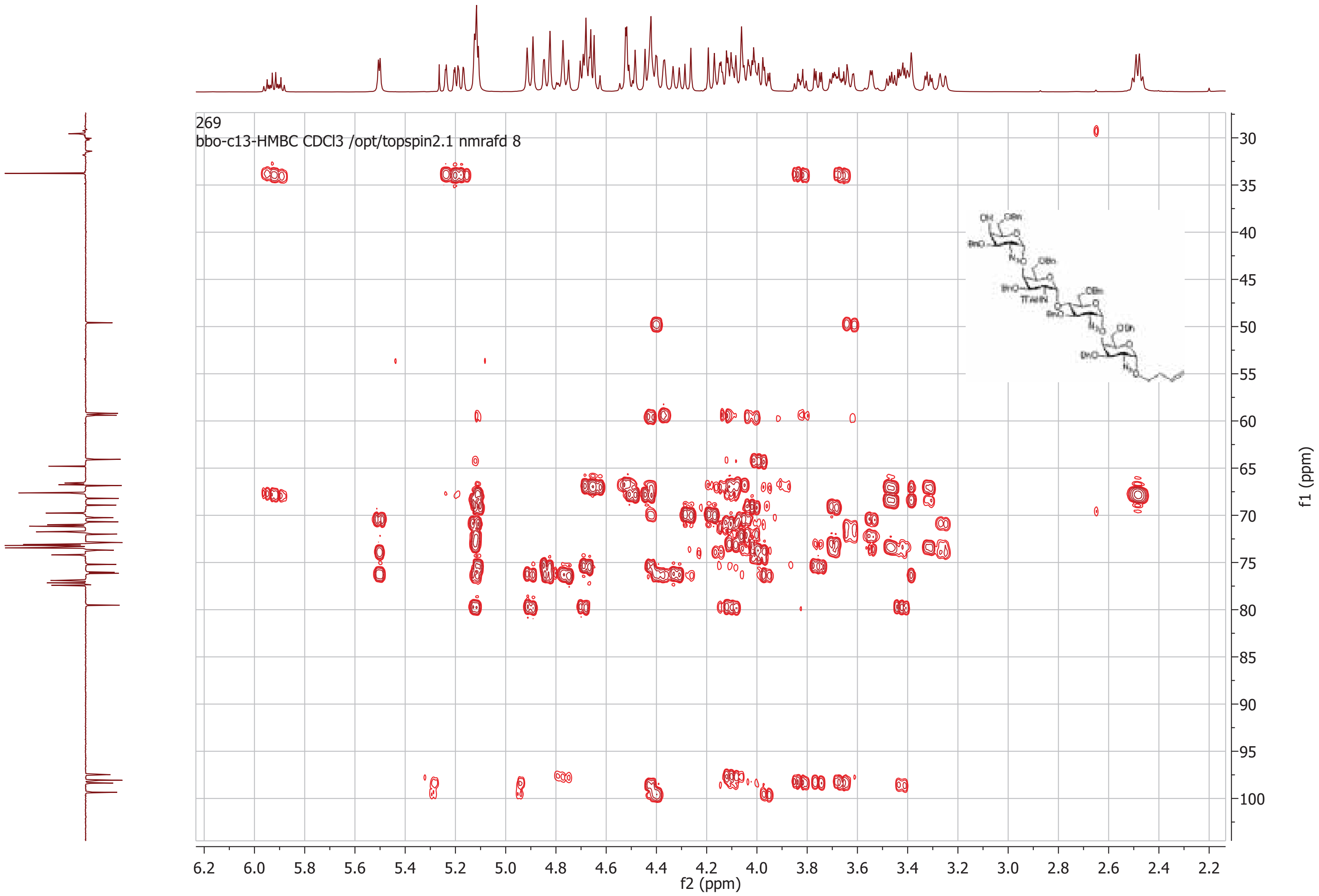
269
bbo-c13-HMBC CDCl3 /opt/topspin2.1 nmrafd 8
f2 (ppm)
f1 (ppm)

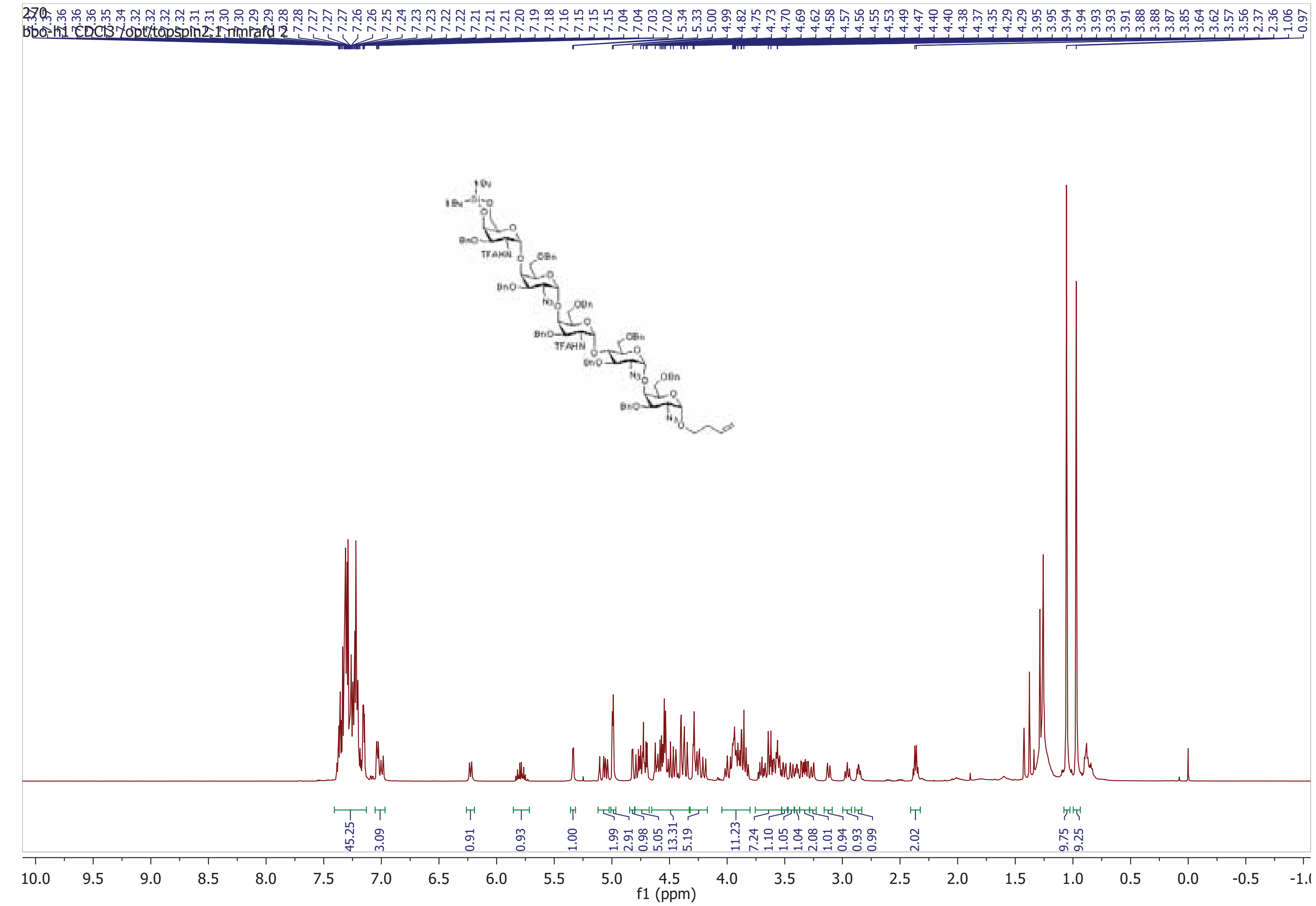
270
bbo-h1 CDCl3 /opt/topspin2.1 nmrafd 2
7.37 7.36 7.36 7.35 7.34 7.32 7.32 7.32 7.32 7.31 7.30 7.30 7.29 7.29 7.28 7.27 7.27 7.27 7.26 7.25 7.24 7.23 7.23 7.22 7.21 7.21 7.21 7.20 7.19 7.18 7.16 7.15 7.15 7.15 7.04 7.04 7.03 7.02 5.34 5.33 5.00 4.99 4.82 4.75 4.73 4.70 4.69 4.62 4.58 4.57 4.56 4.55 4.53 4.49 4.47 4.40 4.40 4.38 4.37 4.35 4.29 4.29 3.95 3.95 3.94 3.94 3.93 3.93 3.91 3.88 3.87 3.85 3.64 3.62 3.57 3.56 2.37 2.36 1.06 0.97
tBu
Si
O
BnO
TFAHN
OBn
N3
45.25
3.09
0.91
0.93
1.00
1.99
2.91
0.98
5.05
13.31
5.19
11.23
7.24
1.10
1.05
1.04
2.08
1.01
0.94
0.93
0.99
2.02
9.75
9.25
10.0 9.5 9.0 8.5 8.0 7.5 7.0 6.5 6.0 5.5 5.0 4.5 4.0 3.5 3.0 2.5 2.0 1.5 1.0 0.5 0.0 -0.5
f1 (ppm)

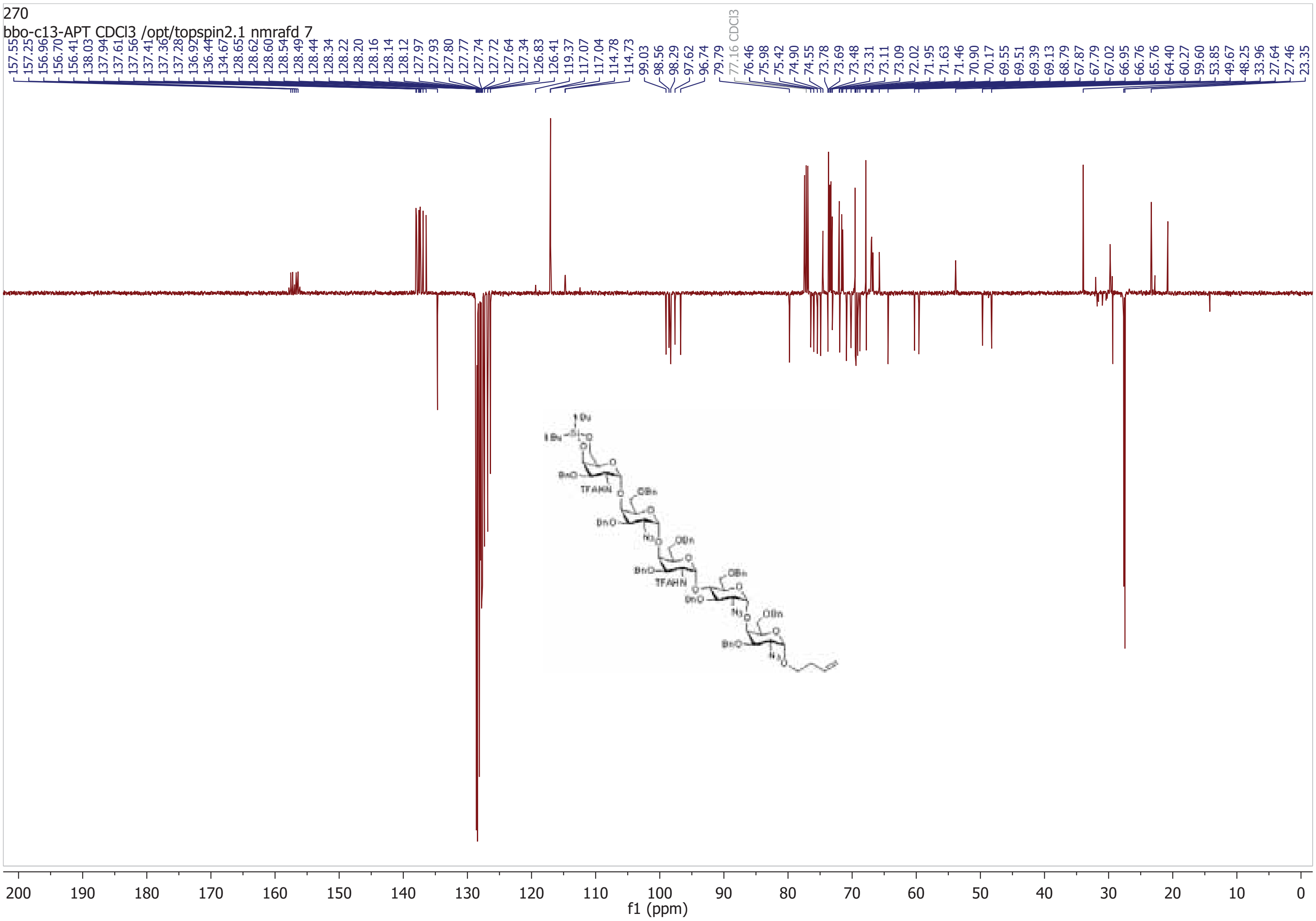
270
bbo-c13-APT CDCl3 /opt/topspin2.1 nmrafd 7
157.55
157.25
156.96
156.70
156.41
138.03
137.94
137.61
137.56
137.41
137.36
137.28
136.92
136.44
134.67
128.65
128.62
128.60
128.54
128.49
128.44
128.34
128.22
128.20
128.16
128.14
128.12
127.97
127.93
127.80
127.77
127.74
127.72
127.64
127.34
126.83
126.41
119.37
117.07
117.04
114.78
114.73
99.03
98.56
98.29
97.62
96.74
79.79
77.16 CDCl3
76.46
75.98
75.42
74.90
74.55
73.78
73.69
73.48
73.31
73.11
73.09
72.02
71.95
71.63
71.46
70.90
70.17
69.55
69.51
69.39
69.13
68.79
67.87
67.79
67.02
66.95
66.76
65.76
64.40
60.27
59.60
53.85
49.67
48.25
33.96
27.64
27.46
23.35
200
190
180
170
160
150
140
130
120
110
100
90
80
70
60
50
40
30
20
10
0
f1 (ppm)

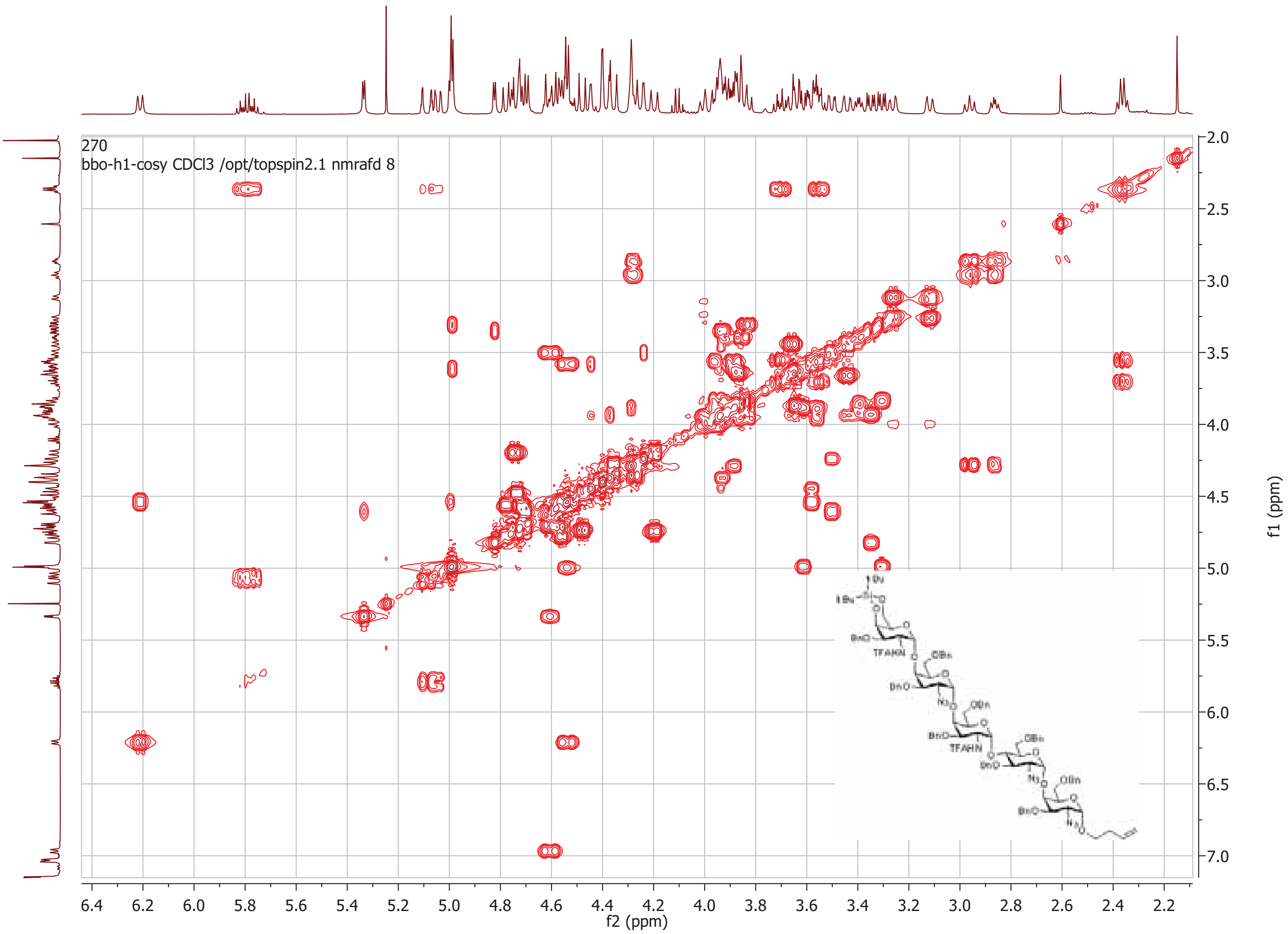
270
bbo-h1-cosy CDCl3 /opt/topspin2.1 nmrafd 8
f2 (ppm)
f1 (ppm)
6.4 6.2 6.0 5.8 5.6 5.4 5.2 5.0 4.8 4.6 4.4 4.2 4.0 3.8 3.6 3.4 3.2 3.0 2.8 2.6 2.4 2.2
2.0 2.5 3.0 3.5 4.0 4.5 5.0 5.5 6.0 6.5 7.0
TFAHN
OBn
BnO
N3

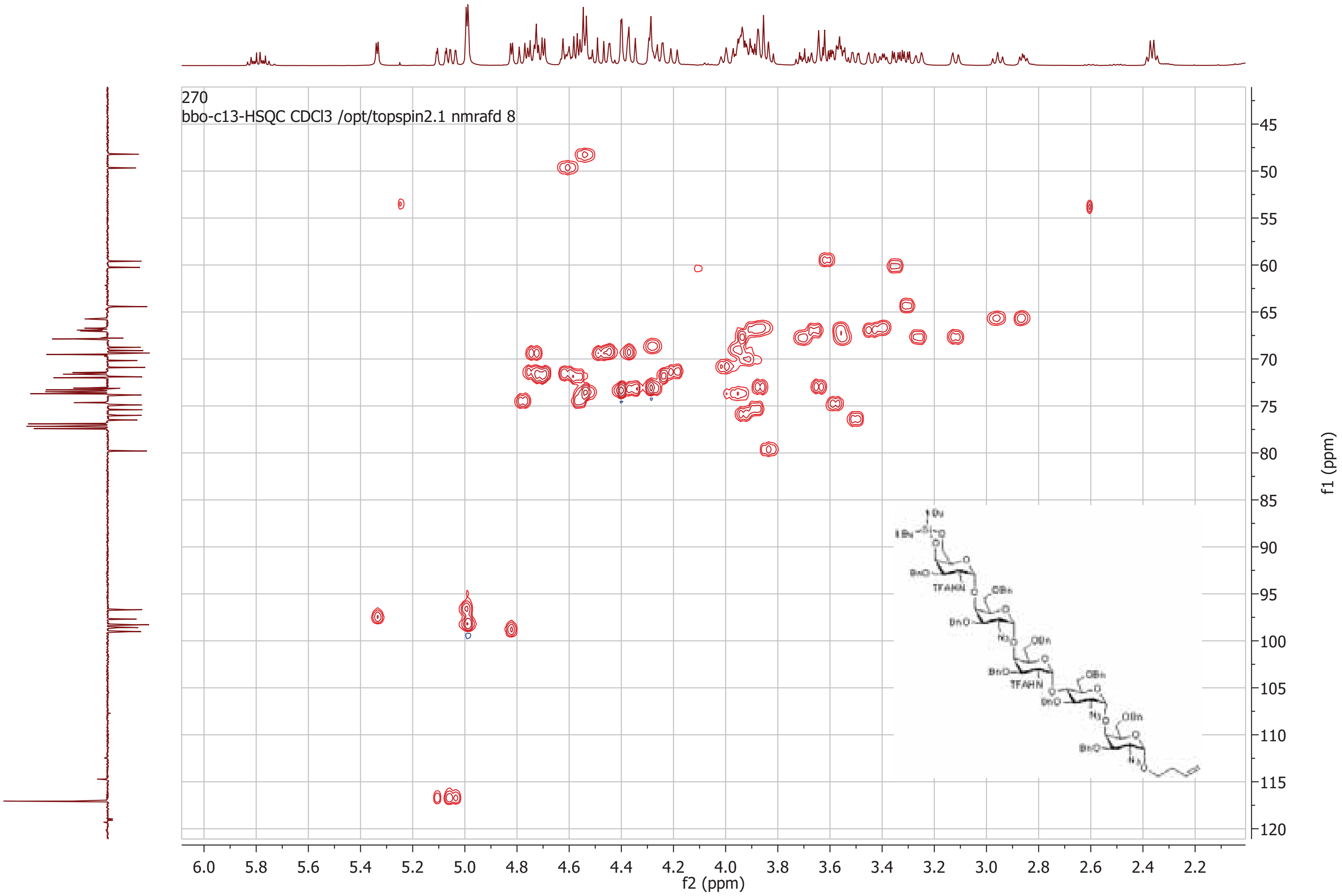
270
bbo-c13-HSQC CDCl3 /opt/topspin2.1 nmrafd 8
f2 (ppm)
f1 (ppm)

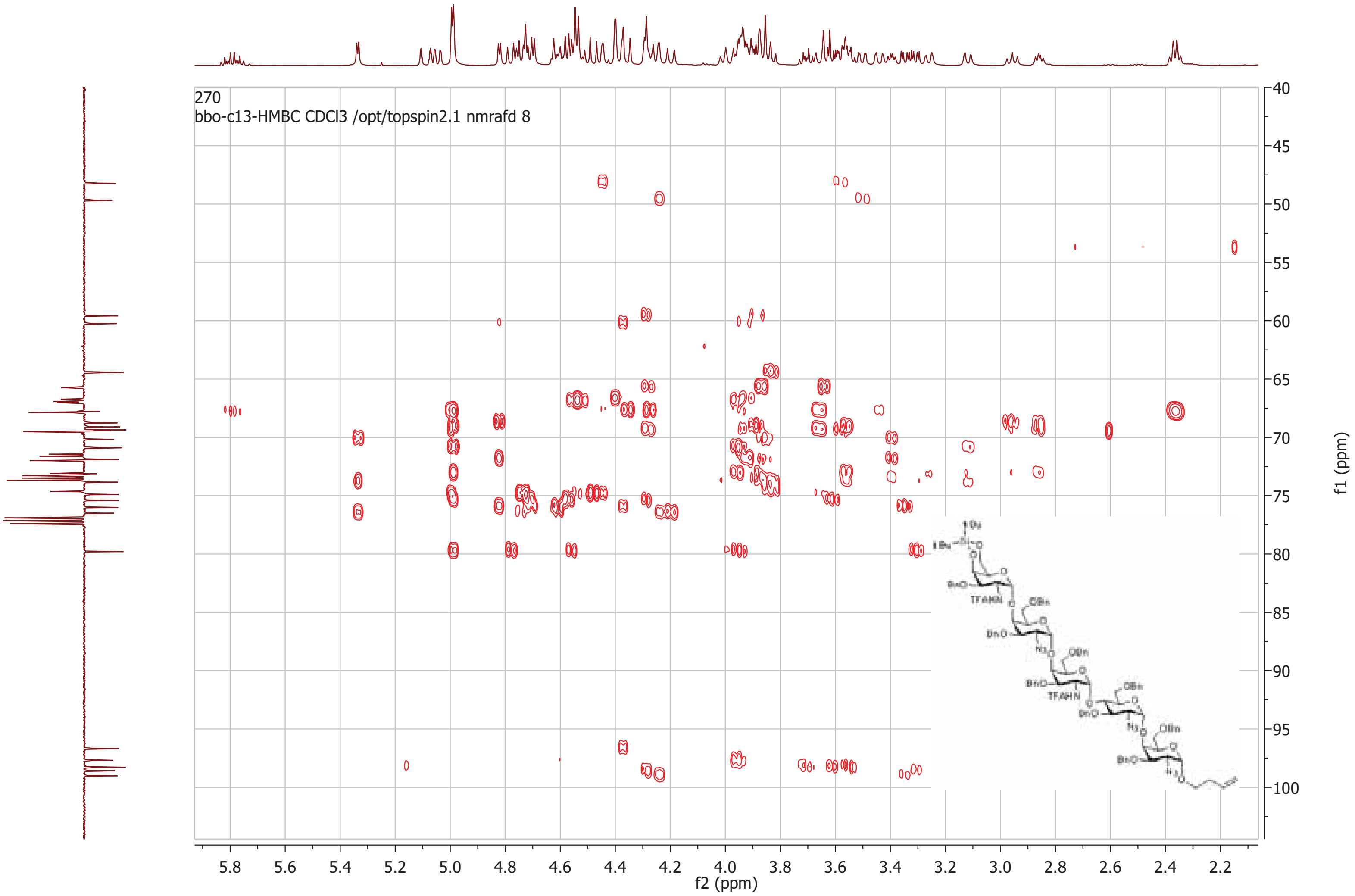
270
bbo-c13-HMBC CDCl3 /opt/topspin2.1 nmrafd 8
f2 (ppm)
f1 (ppm)
TFAHN
BnO
OBn

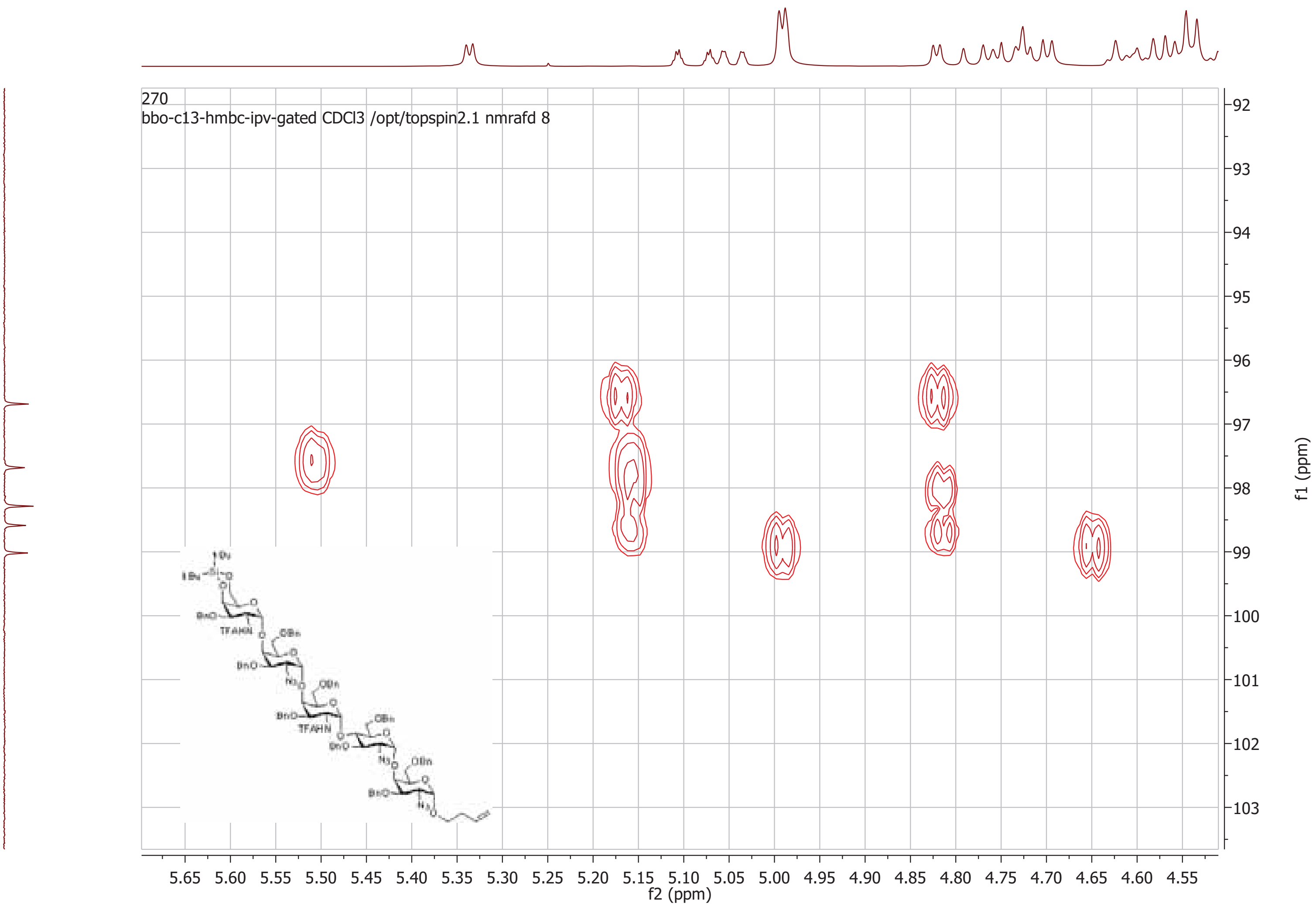
270
bbo-c13-hmbc-ipv-gated CDCl3 /opt/topspin2.1 nmrafd 8
f2 (ppm)
f1 (ppm)
TFAHN
BnO
N3
OBn

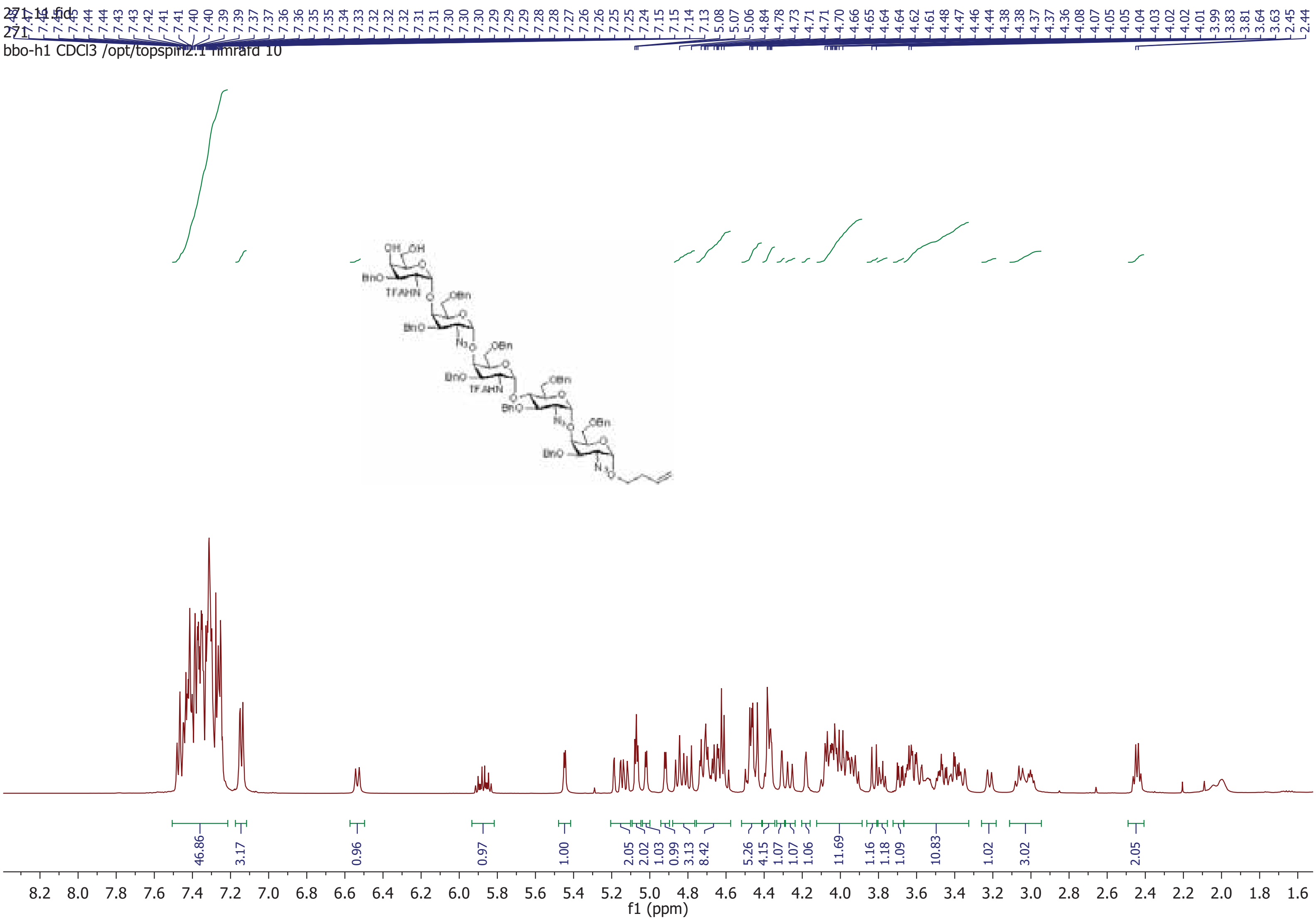

271 1I fid
271
bbo-h1 CDCl3 /opt/topspin2.1 nmrafd 10
7.45 7.44 7.44 7.43 7.43 7.42 7.41 7.41 7.40 7.40 7.39 7.39 7.37 7.37 7.36 7.36 7.35 7.35 7.34 7.33 7.32 7.32 7.32 7.31 7.31 7.30 7.30 7.30 7.29 7.29 7.29 7.28 7.28 7.27 7.26 7.26 7.25 7.25 7.24 7.15 7.15 7.14 7.13 5.08 5.07 5.06 4.84 4.78 4.73 4.71 4.71 4.70 4.66 4.65 4.64 4.64 4.62 4.61 4.48 4.47 4.46 4.44 4.38 4.38 4.37 4.37 4.36 4.08 4.07 4.05 4.05 4.04 4.03 4.02 4.02 4.01 3.99 3.83 3.81 3.64 3.63 2.45 2.44
OH
OH
BnO
TFAHN
OBn
BnO
N3
OBn
BnO
TFAHN
OBn
BnO
N3
OBn
BnO
N3
46.86 3.17 0.96 0.97 1.00 2.05 2.02 1.03 0.99 3.13 8.42 5.26 4.15 1.07 1.07 1.06 11.69 1.16 1.18 1.09 10.83 1.02 3.02 2.05
8.2 8.0 7.8 7.6 7.4 7.2 7.0 6.8 6.6 6.4 6.2 6.0 5.8 5.6 5.4 5.2 5.0 4.8 4.6 4.4 4.2 4.0 3.8 3.6 3.4 3.2 3.0 2.8 2.6 2.4 2.2 2.0 1.8 1.6
f1 (ppm)

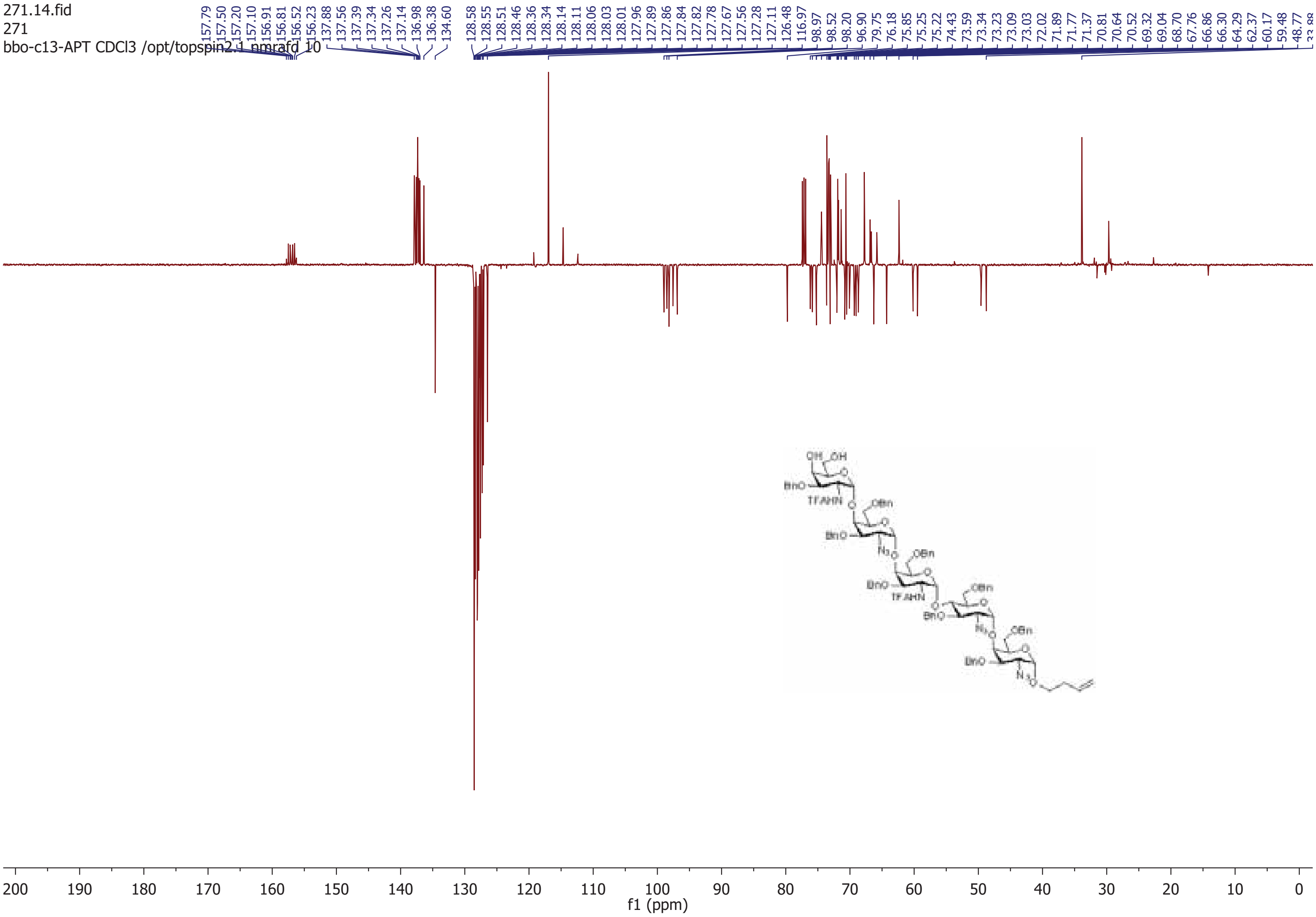
271.14.fid
271
bbo-c13-APT CDCl3 /opt/topspin2.1 nmrafd 10
157.79
157.50
157.20
157.10
156.91
156.81
156.52
156.23
137.88
137.56
137.39
137.34
137.26
137.14
136.98
136.38
134.60
128.58
128.55
128.51
128.46
128.36
128.34
128.14
128.11
128.06
128.03
128.01
127.96
127.89
127.86
127.84
127.82
127.78
127.67
127.56
127.28
127.11
126.48
116.97
98.97
98.52
98.20
96.90
79.75
76.18
75.85
75.25
75.22
74.43
73.59
73.34
73.23
73.09
73.03
72.02
71.89
71.77
71.37
70.81
70.64
70.52
69.32
69.04
68.70
67.76
66.86
66.30
64.29
62.37
60.17
59.48
48.77
OH
OH
BnO
TFAHN
OBn
BnO
N3
OBn
BnO
TFAHN
OBn
BnO
N3
OBn
BnO
N3
200
190
180
170
160
150
140
130
120
110
100
90
80
70
60
50
40
30
20
10
0
f1 (ppm)

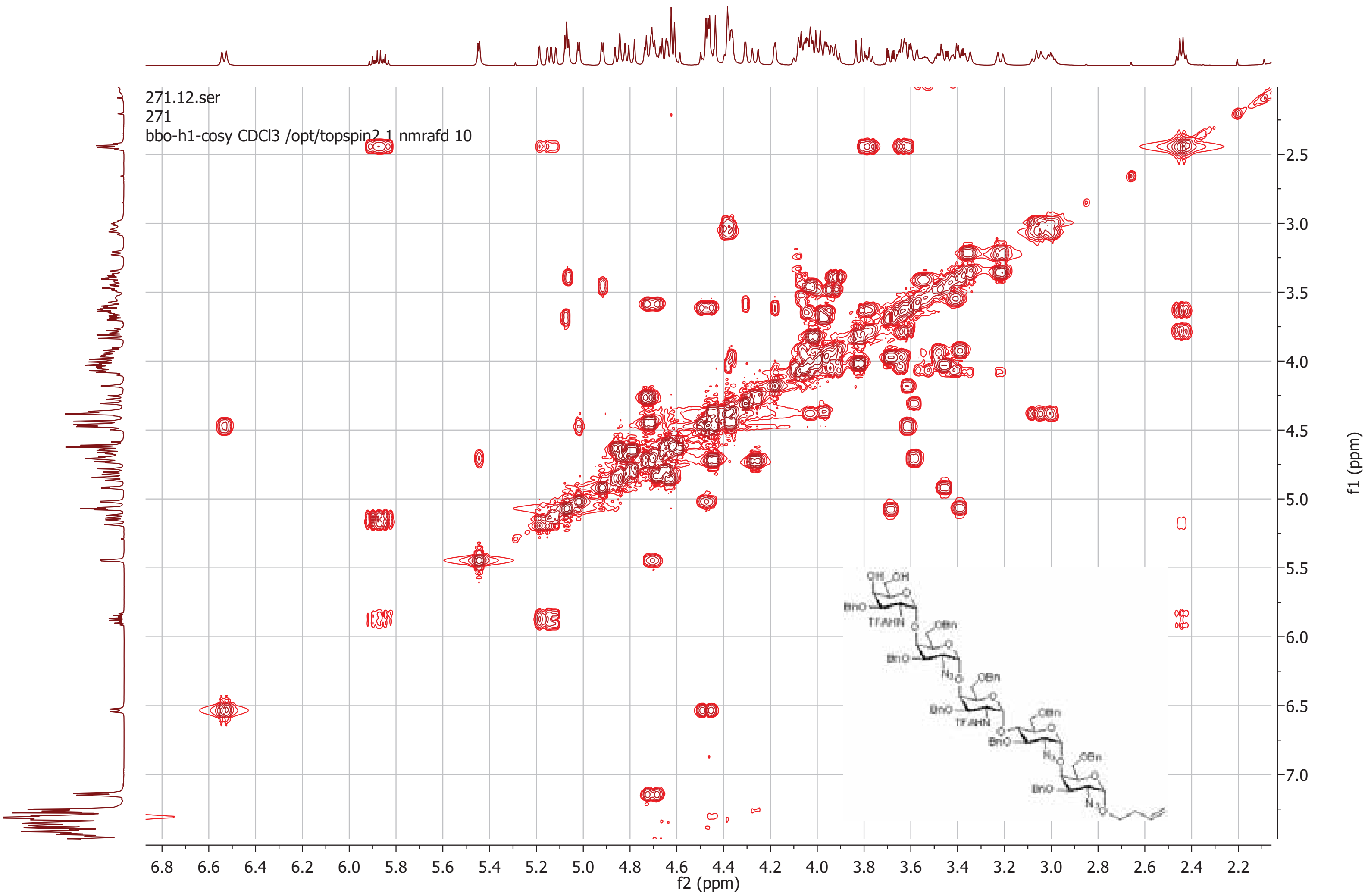
271.12.ser
271
bbo-h1-cosy CDCl3 /opt/topspin2.1 nmrafd 10
f2 (ppm)
f1 (ppm)
OH
OBn
BnO
TFAHN
N3

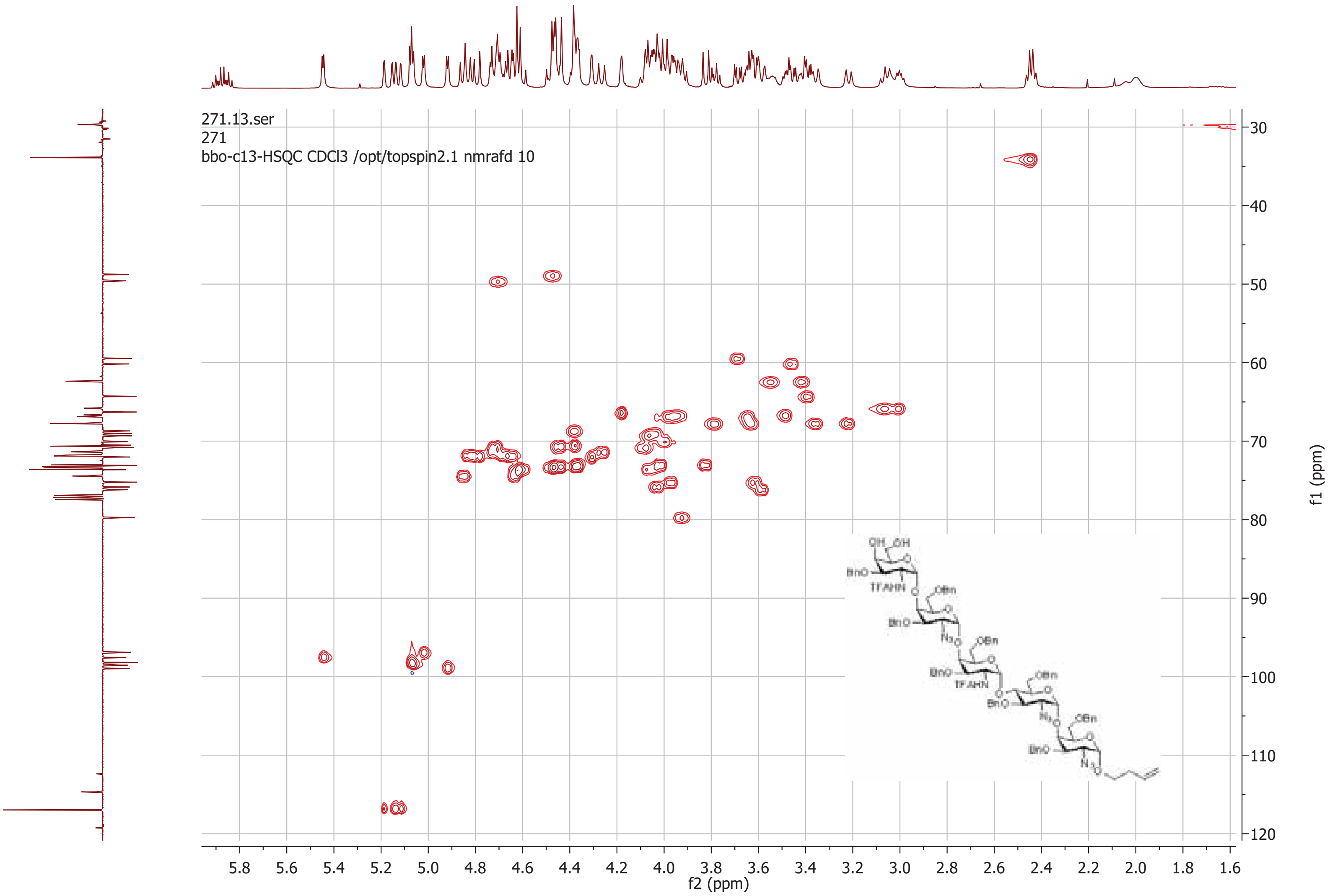
271.13.ser
271
bbo-c13-HSQC CDCl3 /opt/topspin2.1 nmrafd 10
f1 (ppm)
f2 (ppm)
OH
OH
BnO
TFAHN
OBn
BnO
N3
OBn
BnO
TFAHN
OBn
BnO
N3
OBn
BnO
N3

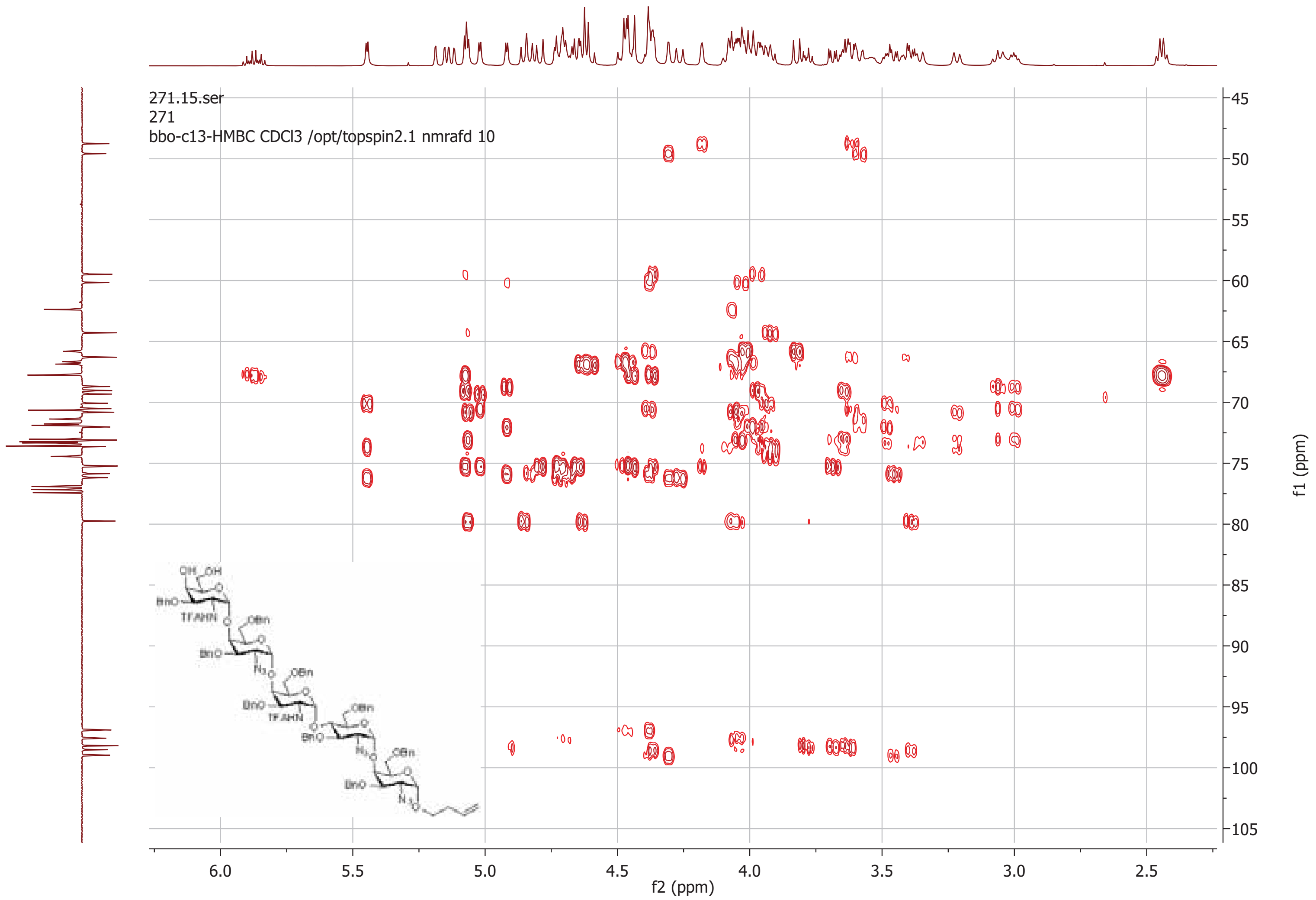
271.15.ser
271
bbo-c13-HMBC CDCl3 /opt/topspin2.1 nmrafd 10
f2 (ppm)
f1 (ppm)
OH
OH
BnO
TFAHN
OBn
BnO
N3
OBn
BnO
TFAHN
OBn
BnO
N3
OBn
BnO
N3

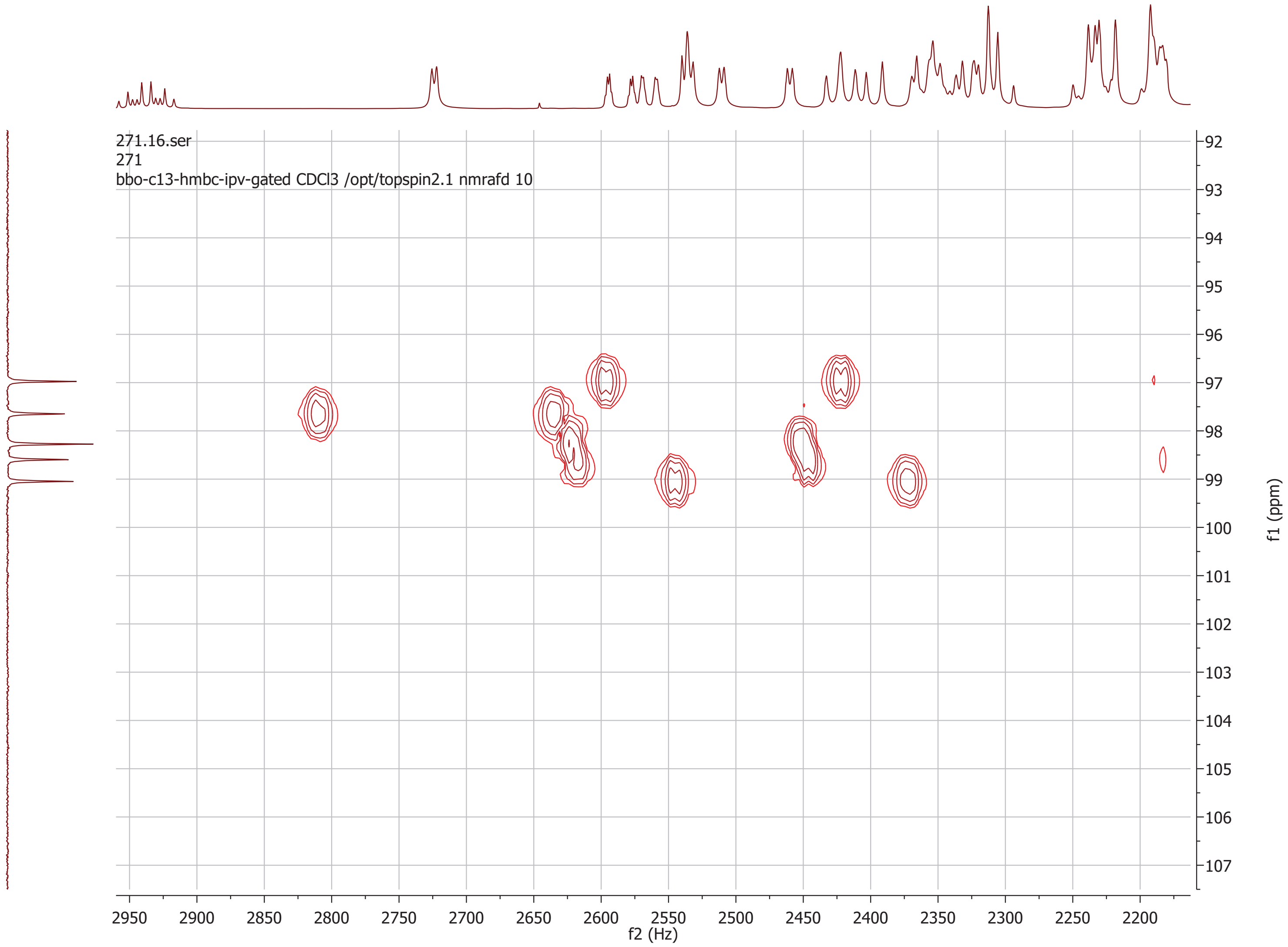

271.16.ser
271
bbo-c13-hmbc-ipv-gated CDCl3 /opt/topspin2.1 nmrafd 10
92
93
94
95
96
97
98
99
100
101
102
103
104
105
106
107
f1 (ppm)
2950
2900
2850
2800
2750
2700
2650
2600
2550
2500
2450
2400
2350
2300
2250
2200
f2 (Hz)

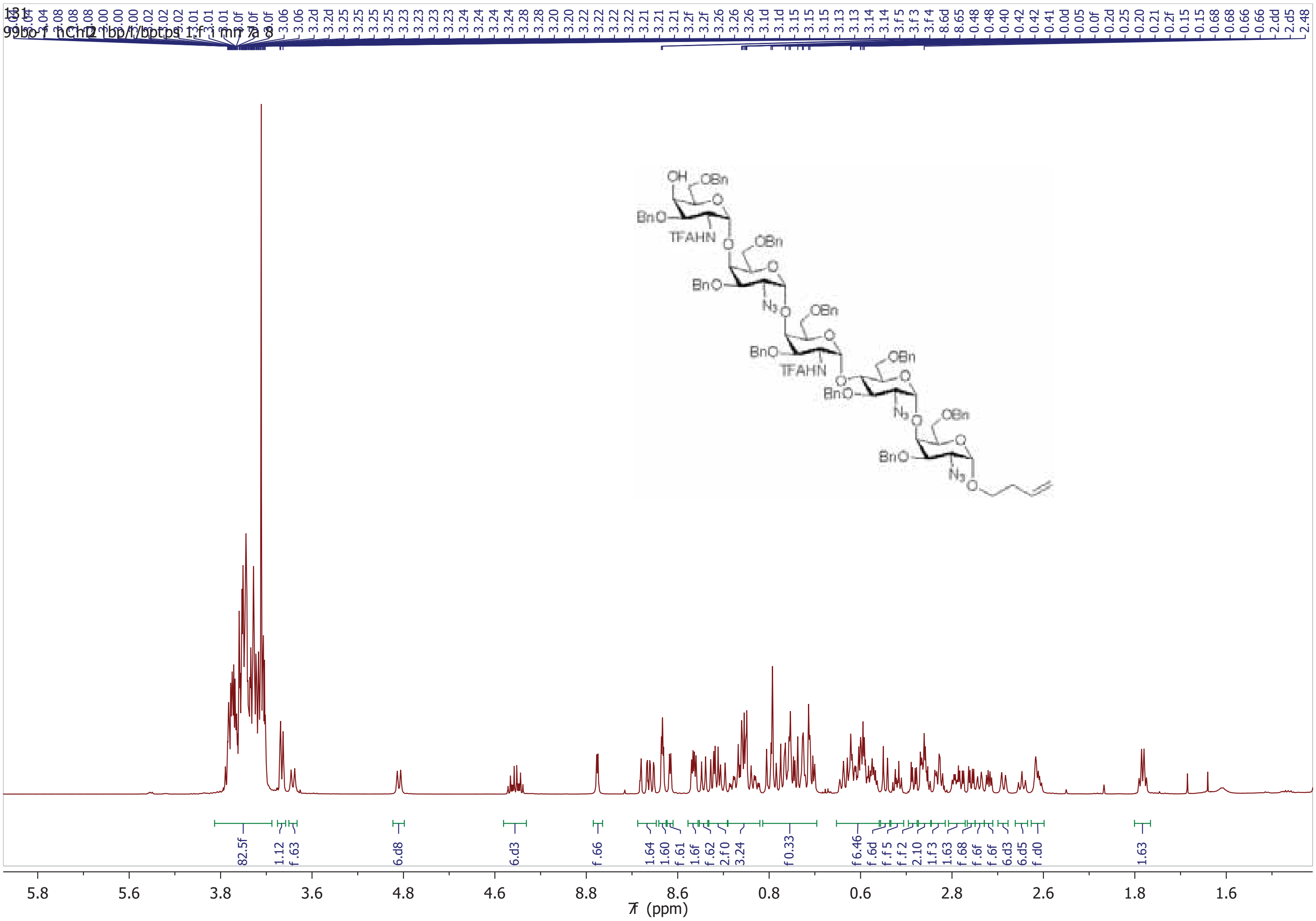
OH
OBn
BnO
TFAHN
N3

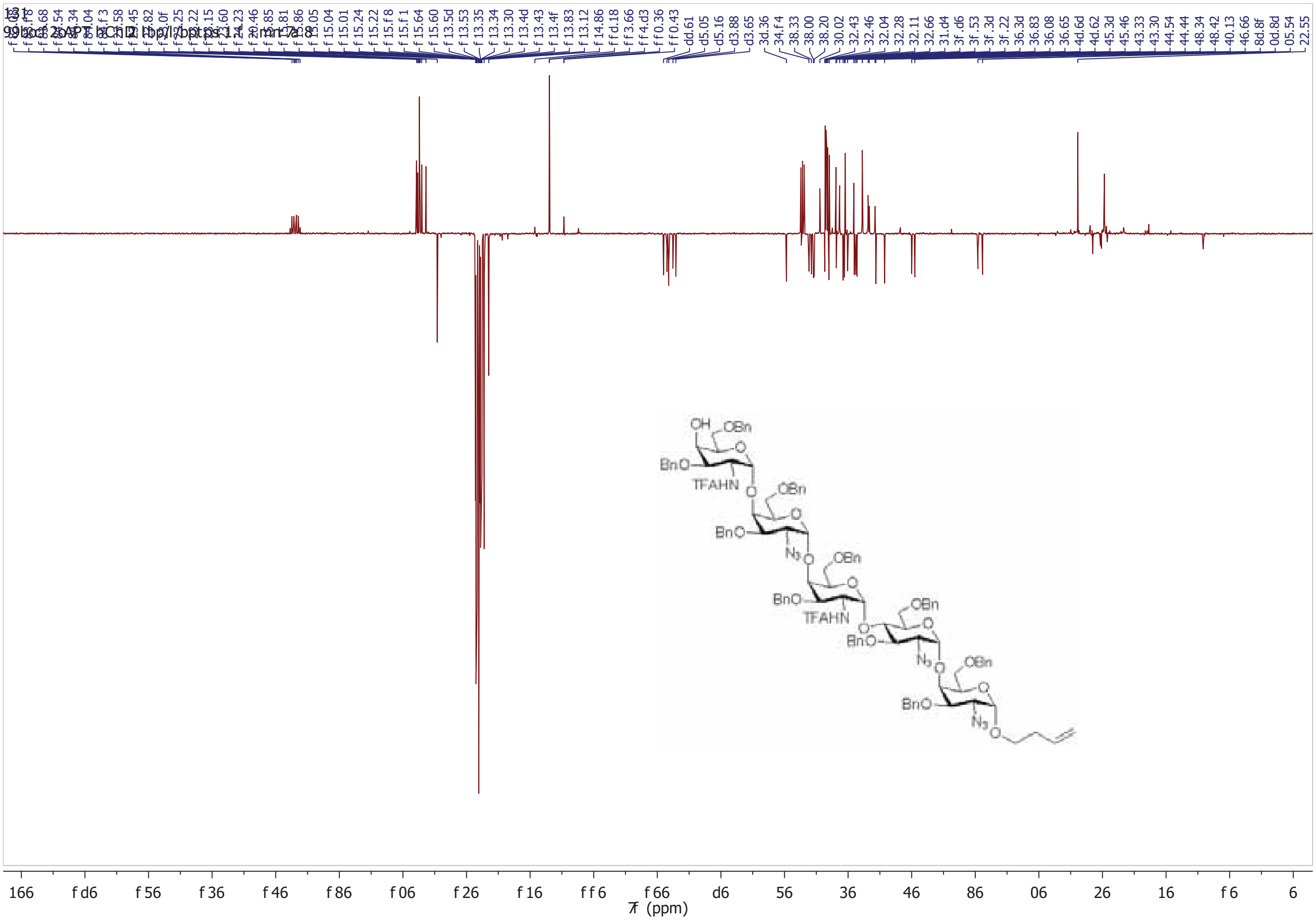
OH
OBn
BnO
TFAHN
N3
(ppm)

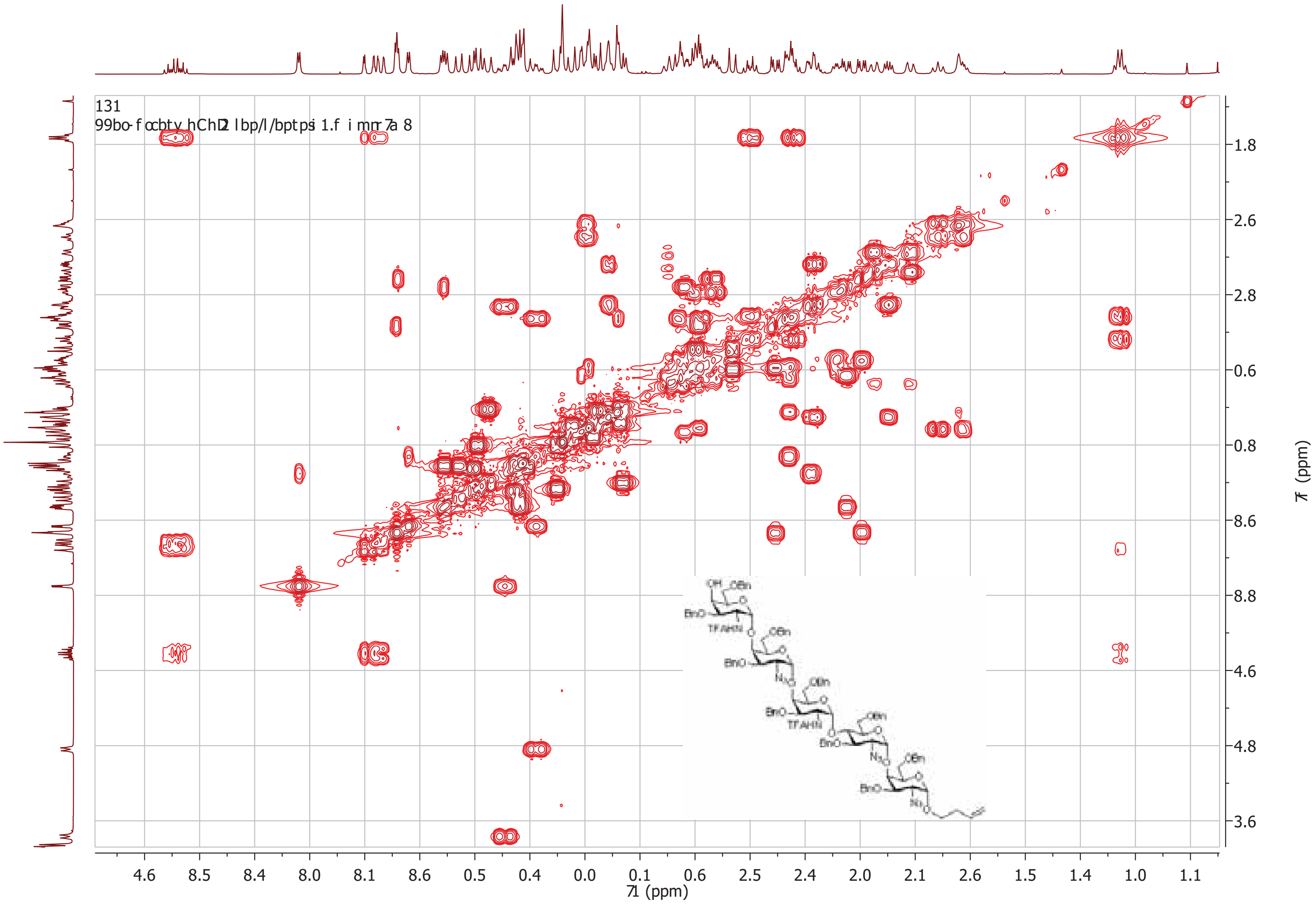
131
99bo-f ocbty hChl2 lbp/l/bptpsi 1.f i mrr7a 8
7l (ppm)
7f (ppm)
OH
OBn
BnO
TFAHN
N3
OBn

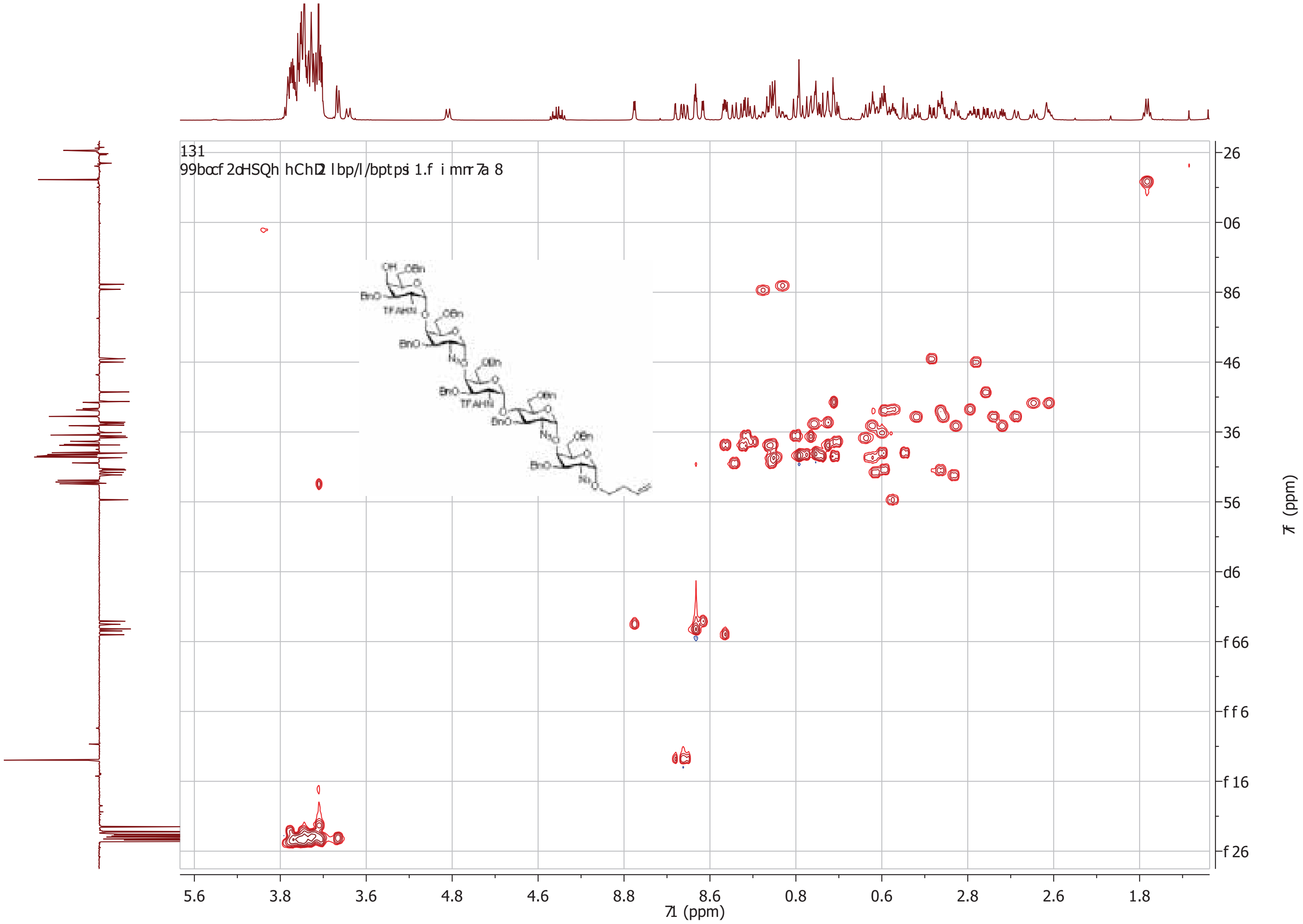
131
99bocf 2oHSQh hChl2 lbp/l/bptpsi 1.f i mm 7a 8
7l (ppm)
7 (ppm)

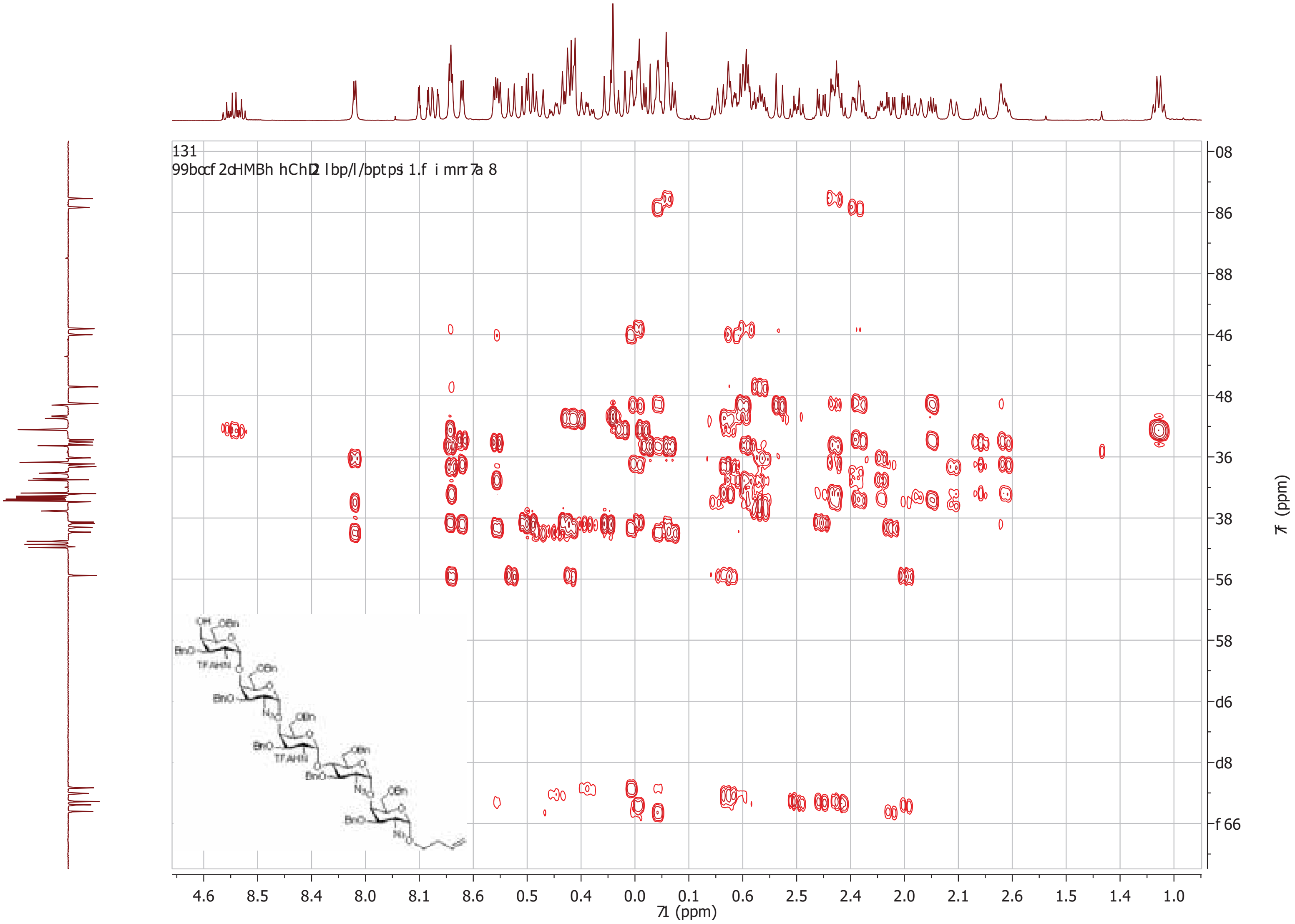

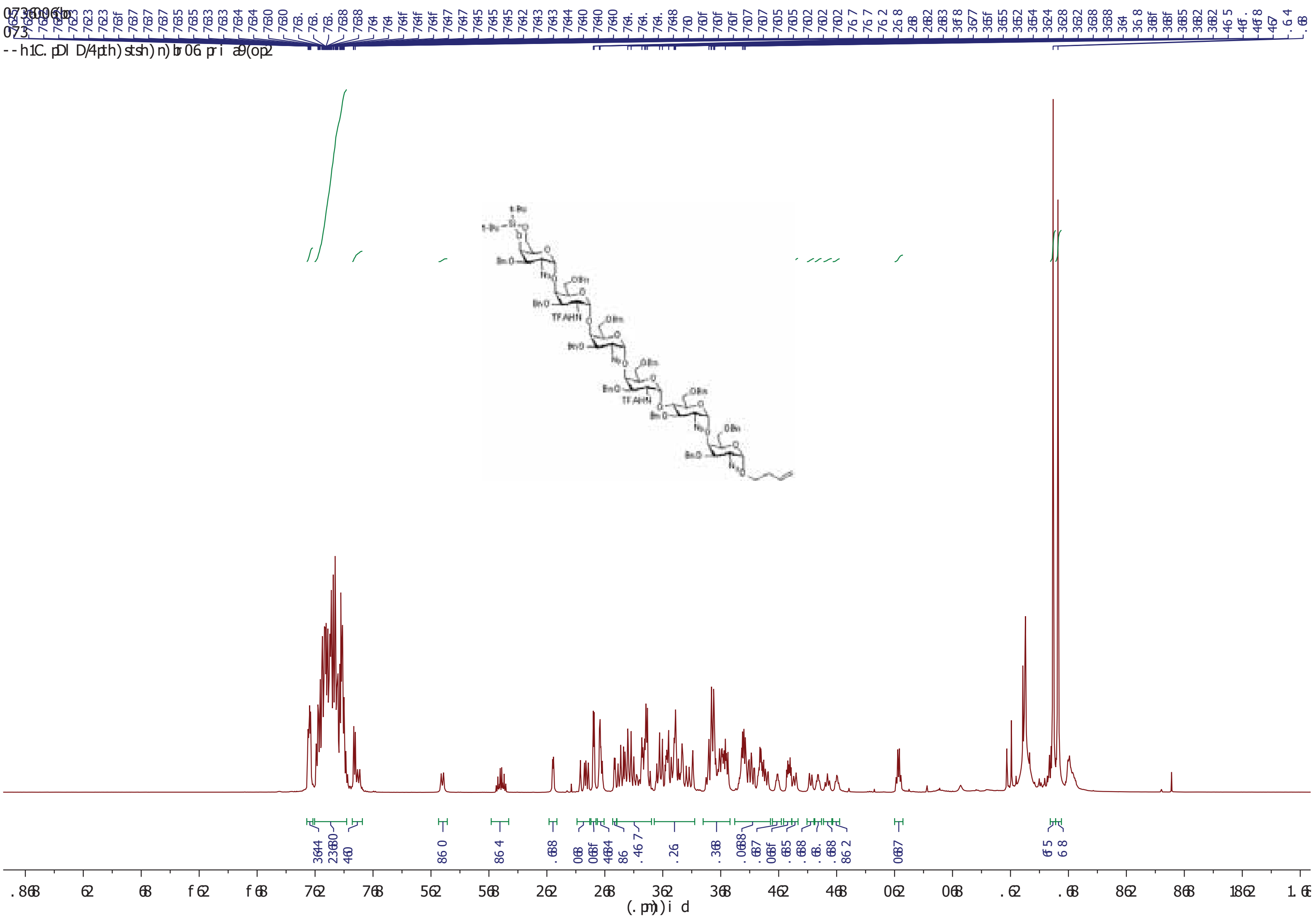

t-Bu
Si
BnO
TFAHN
OBn

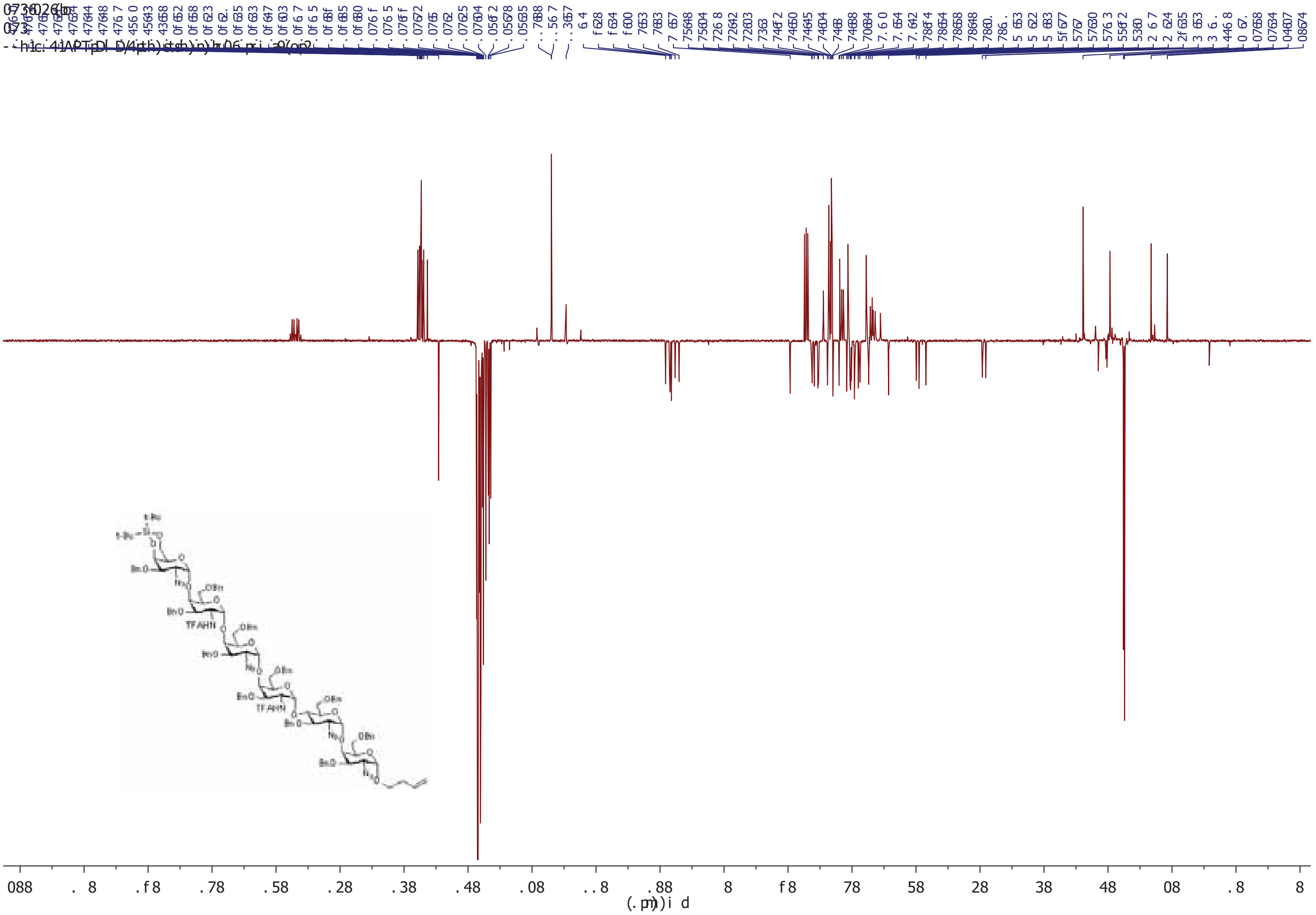

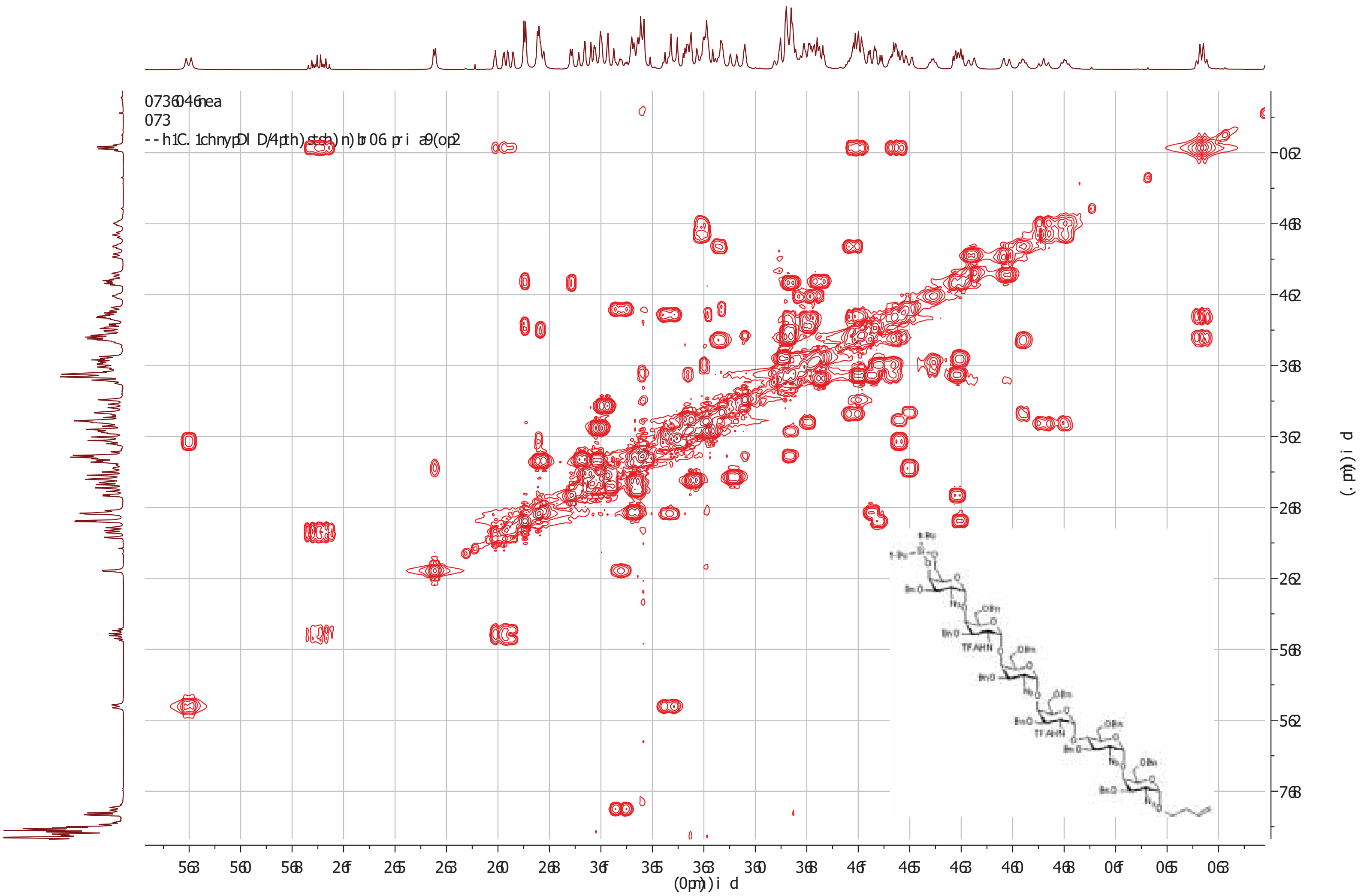

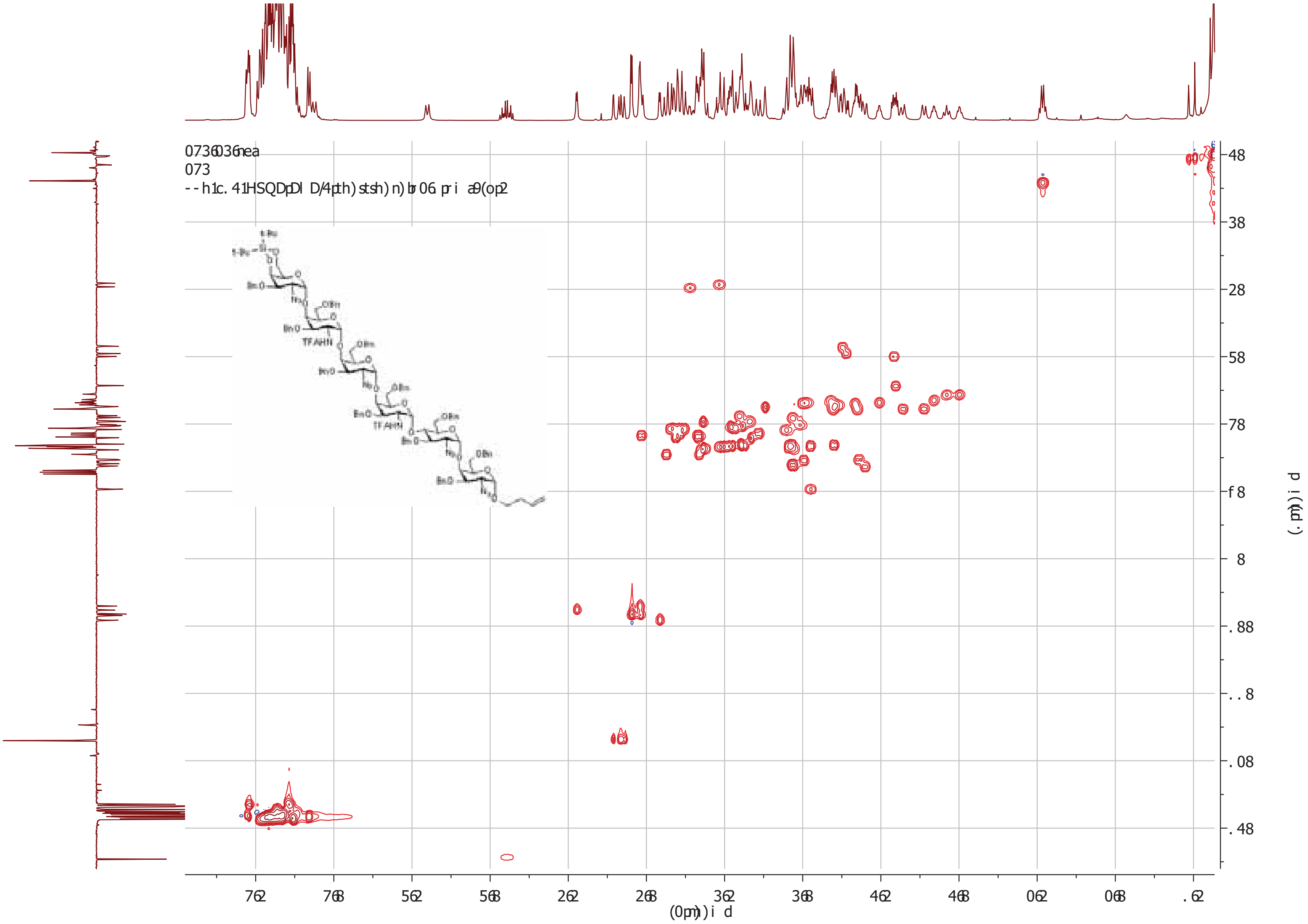

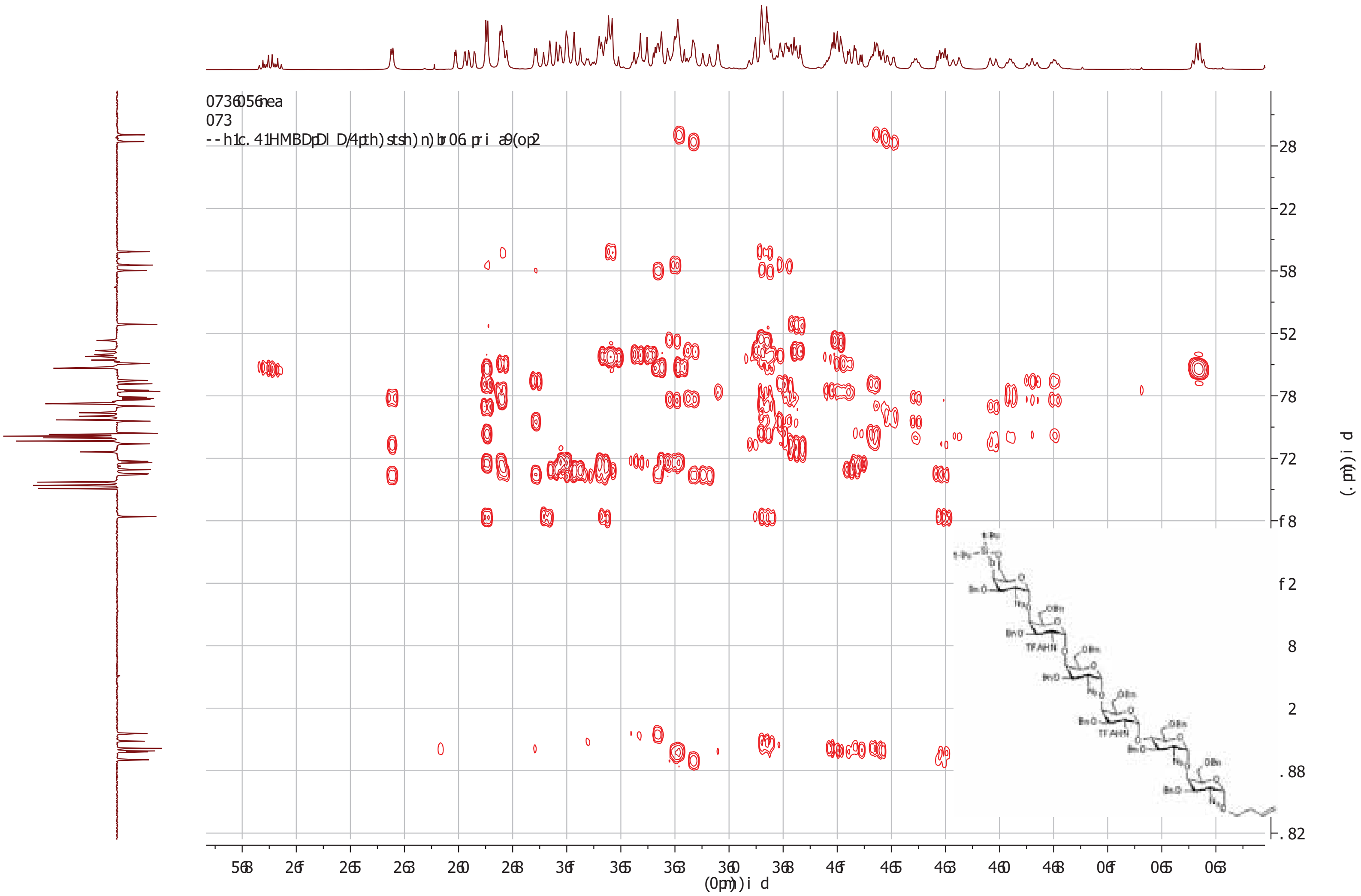

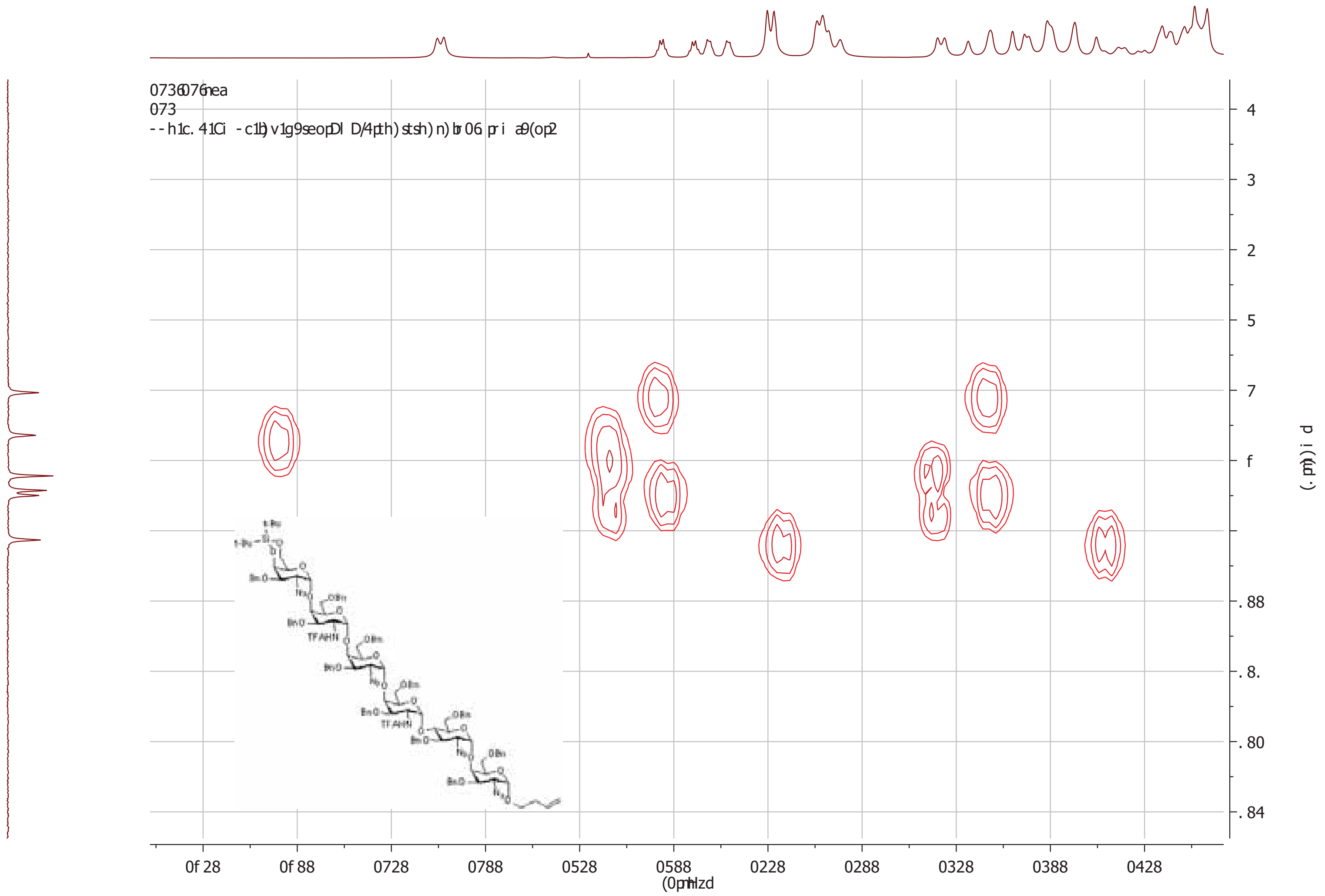

0736076nea
073
0f 28
0f 88
0728
0788
0528
0588
0228
0288
0328
0388
0428
(0pmHzd

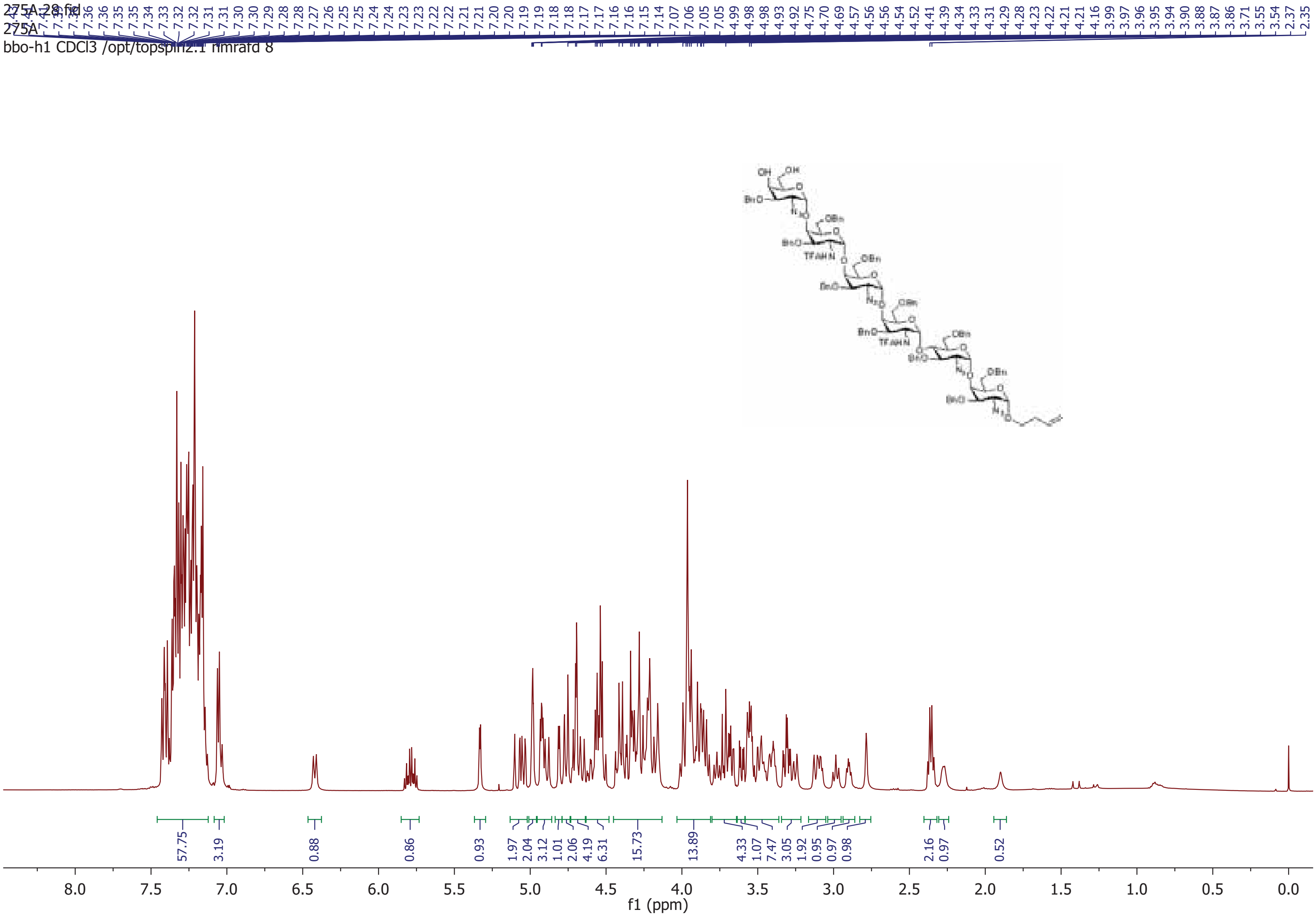
275A-28.fid
275A
bbo-h1 CDCl3 /opt/topspin2.1 nmrafd 8
7.36 7.35 7.35 7.34 7.33 7.32 7.32 7.31 7.31 7.30 7.29 7.28 7.28 7.27 7.26 7.25 7.25 7.24 7.24 7.23 7.23 7.22 7.22 7.21 7.21 7.20 7.20 7.19 7.19 7.18 7.18 7.17 7.17 7.16 7.16 7.15 7.14 7.07 7.06 7.05 7.05 4.99 4.98 4.93 4.92 4.75 4.70 4.69 4.57 4.56 4.56 4.54 4.52 4.41 4.39 4.34 4.33 4.31 4.29 4.28 4.23 4.22 4.21 4.21 4.16 3.99 3.97 3.96 3.95 3.94 3.90 3.88 3.87 3.86 3.71 3.55 3.54 2.37 2.35
57.75 3.19 0.88 0.86 0.93 1.97 2.04 3.12 1.01 2.06 4.19 6.31 15.73 13.89 4.33 1.07 7.47 3.05 1.92 0.95 0.97 0.98 2.16 0.97 0.52
8.0 7.5 7.0 6.5 6.0 5.5 5.0 4.5 4.0 3.5 3.0 2.5 2.0 1.5 1.0 0.5 0.0
f1 (ppm)
OH
OH
BnO
N3
OBn
TFAHN

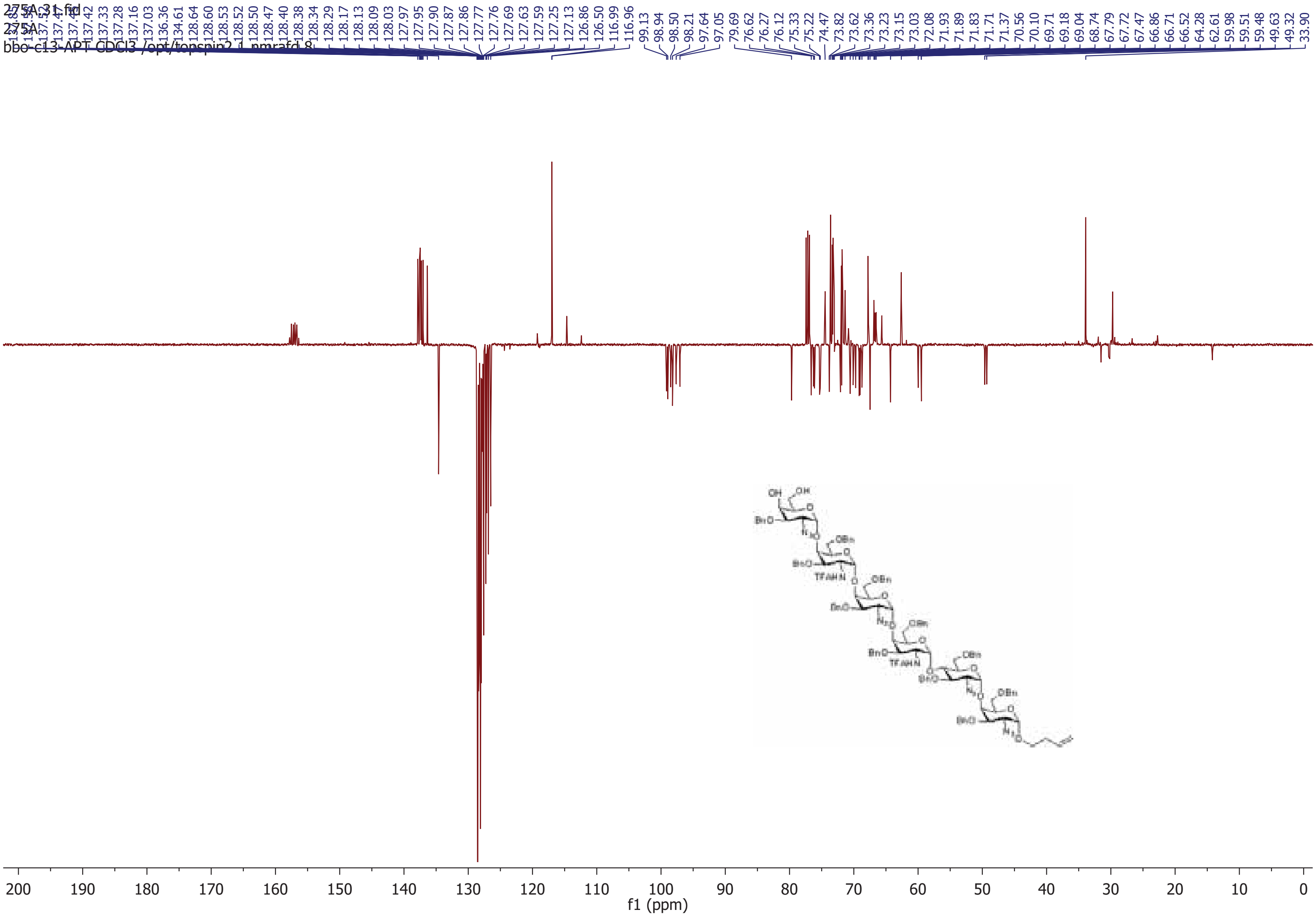
275A.31.fid
275A
bbo-c13-APT CDCl3 /opt/topspin2.1 nmrafd 8
137.47
137.42
137.33
137.28
137.16
137.03
136.36
134.61
128.64
128.60
128.53
128.52
128.50
128.47
128.40
128.38
128.34
128.29
128.17
128.13
128.09
128.03
127.97
127.95
127.90
127.87
127.86
127.77
127.76
127.69
127.63
127.59
127.25
127.13
126.86
126.50
116.99
116.96
99.13
98.94
98.50
98.21
97.64
97.05
79.69
76.62
76.27
76.12
75.33
75.22
74.47
73.82
73.62
73.36
73.23
73.15
73.03
72.08
71.93
71.89
71.83
71.71
71.37
70.56
70.10
69.71
69.18
69.04
68.74
67.79
67.72
67.47
66.86
66.71
66.52
64.28
62.61
59.98
59.51
59.48
49.63
49.32
33.90
200
190
180
170
160
150
140
130
120
110
100
90
80
70
60
50
40
30
20
10
0
f1 (ppm)

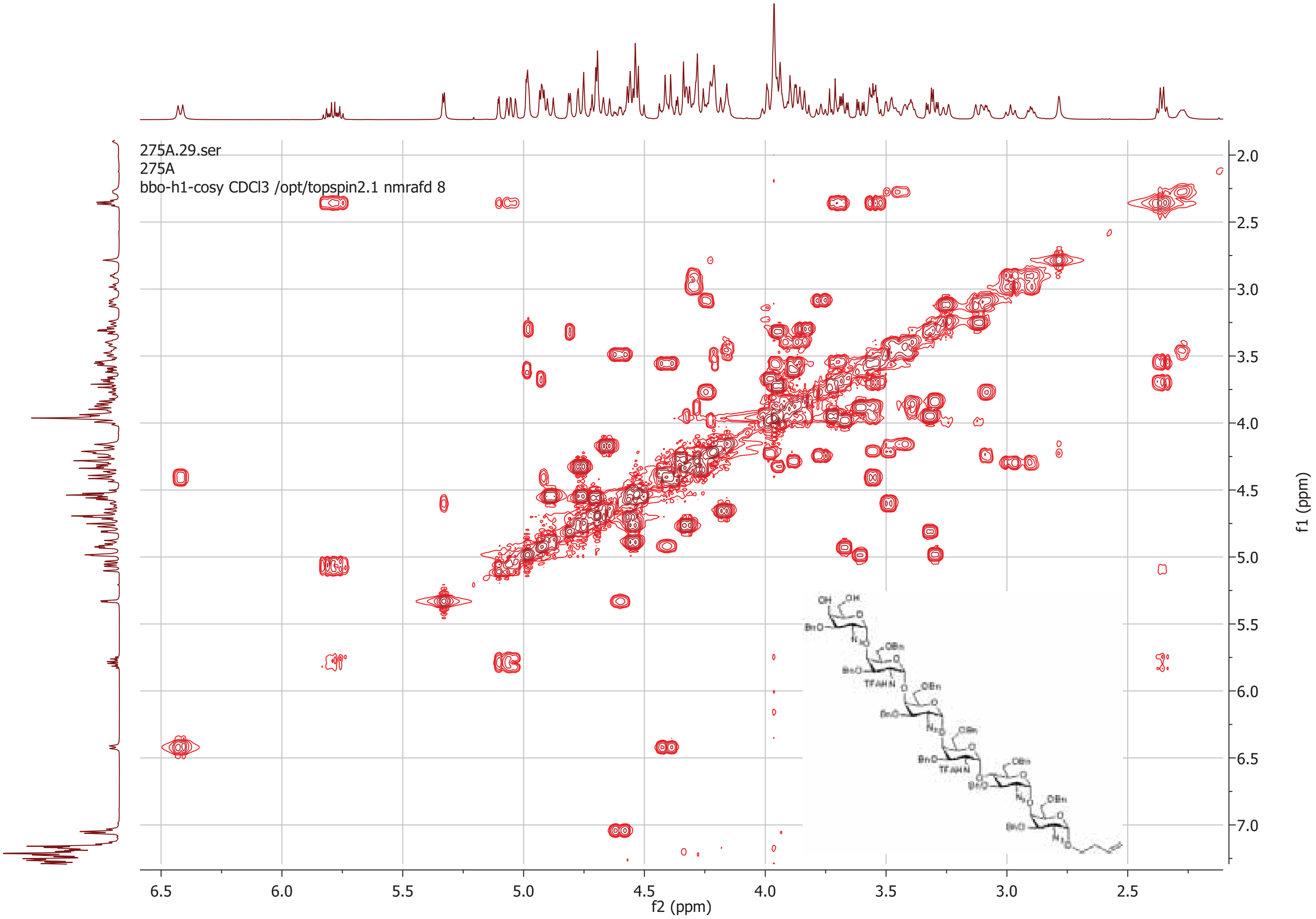

275A.29.ser
275A
bbo-h1-cosy CDCl3 /opt/topspin2.1 nmrafd 8
f2 (ppm)
f1 (ppm)

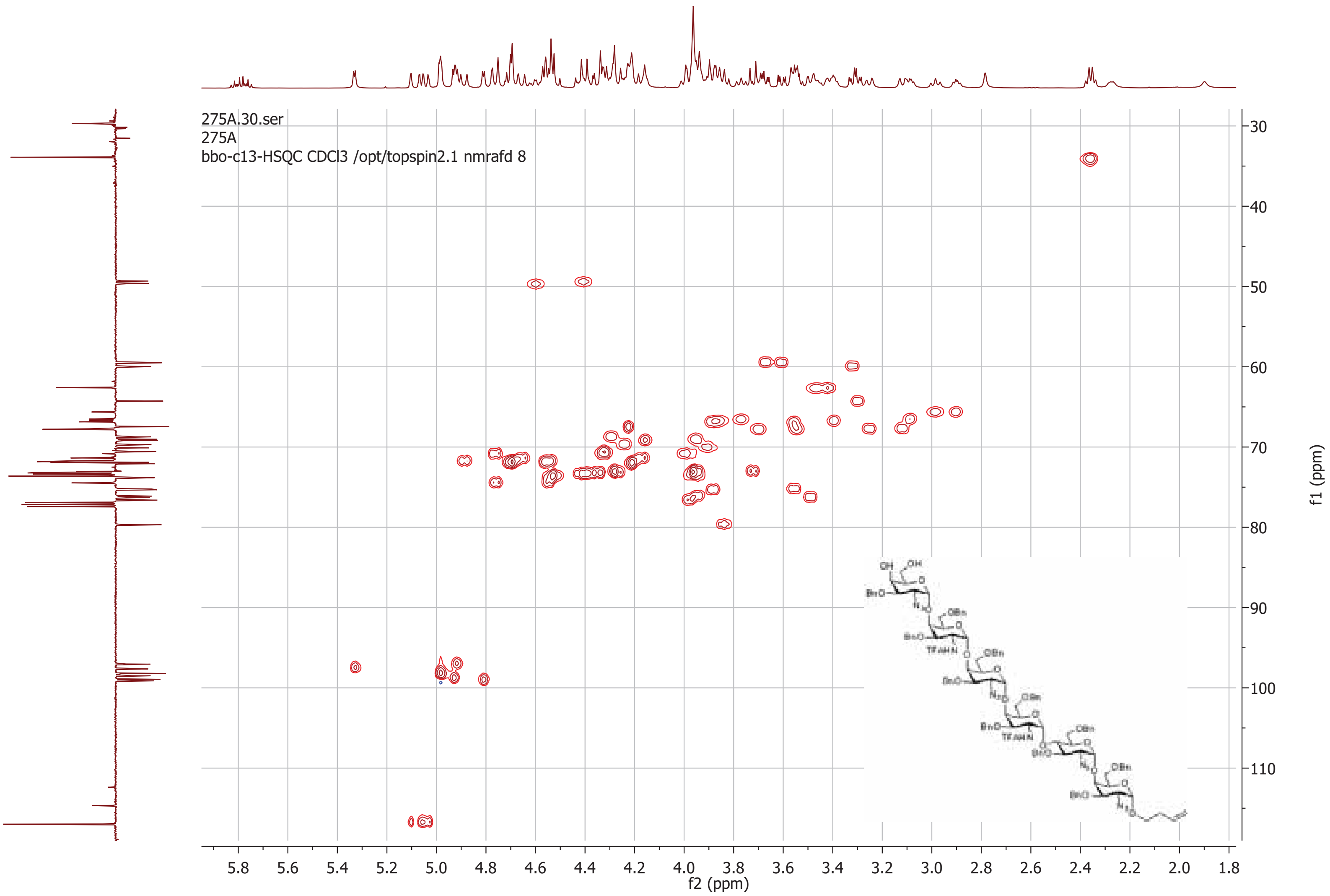
275A.30.ser
275A
bbo-c13-HSQC CDCl3 /opt/topspin2.1 nmrafd 8
f2 (ppm)
f1 (ppm)
5.8
5.6
5.4
5.2
5.0
4.8
4.6
4.4
4.2
4.0
3.8
3.6
3.4
3.2
3.0
2.8
2.6
2.4
2.2
2.0
1.8
30
40
50
60
70
80
90
100
110

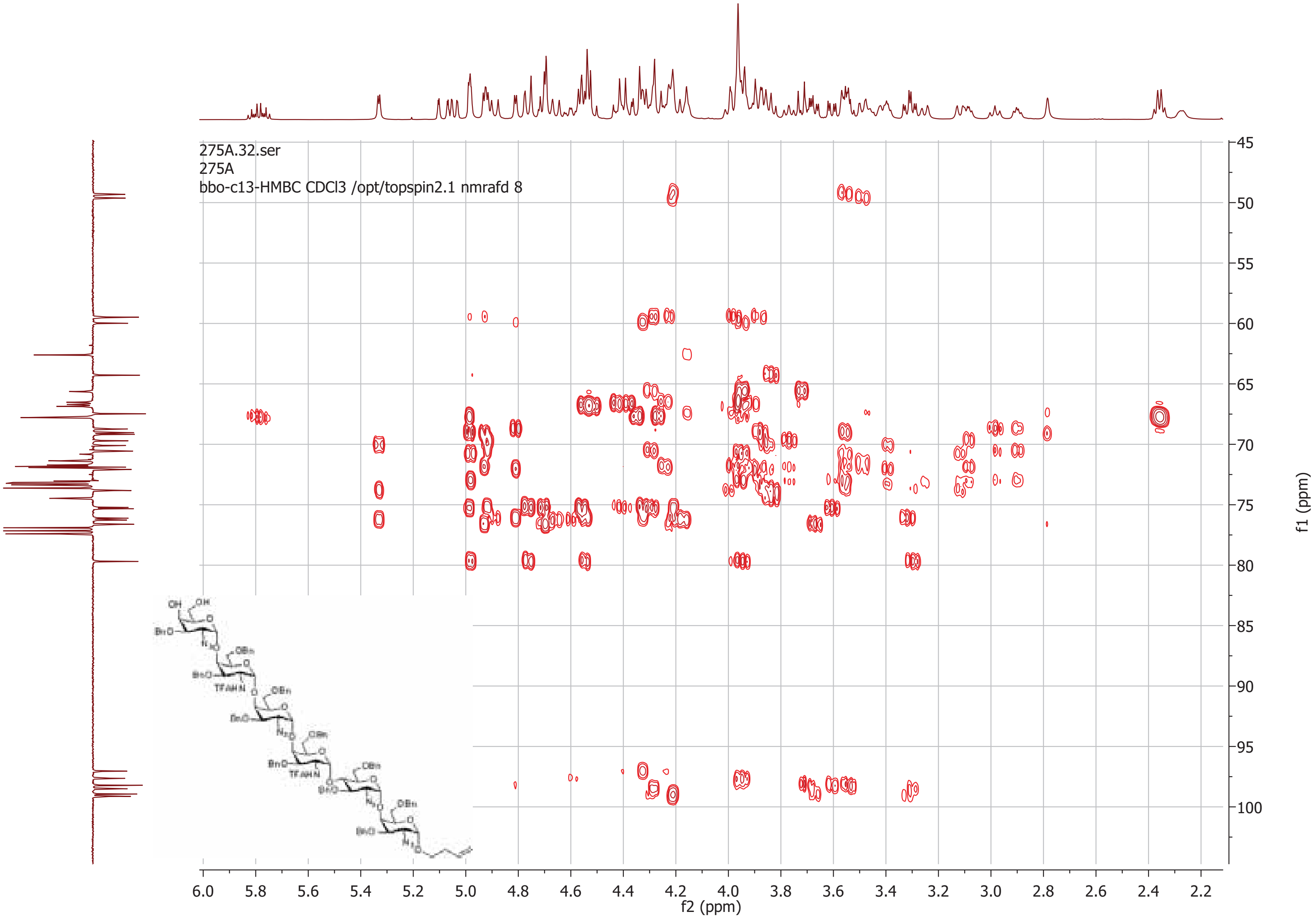
275A.32.ser
275A
bbo-c13-HMBC CDCl3 /opt/topspin2.1 nmrafd 8
f1 (ppm)
f2 (ppm)
45
50
55
60
65
70
75
80
85
90
95
100
6.0
5.8
5.6
5.4
5.2
5.0
4.8
4.6
4.4
4.2
4.0
3.8
3.6
3.4
3.2
3.0
2.8
2.6
2.4
2.2

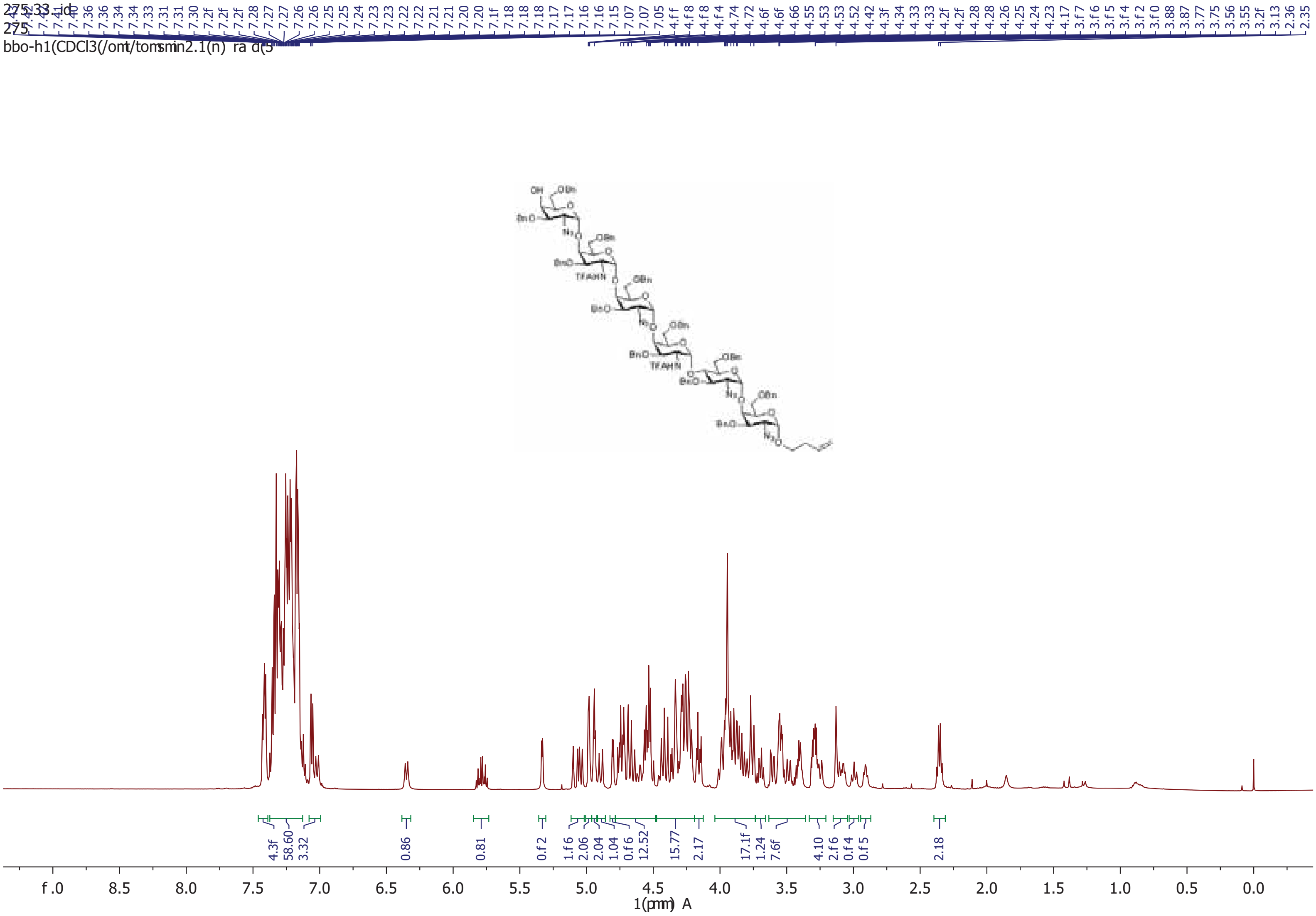
8.5
8.0
7.5
7.0
6.5
6.0
5.5
5.0
4.5
4.0
3.5
3.0
2.5
2.0
1.5
1.0
0.5
0.0

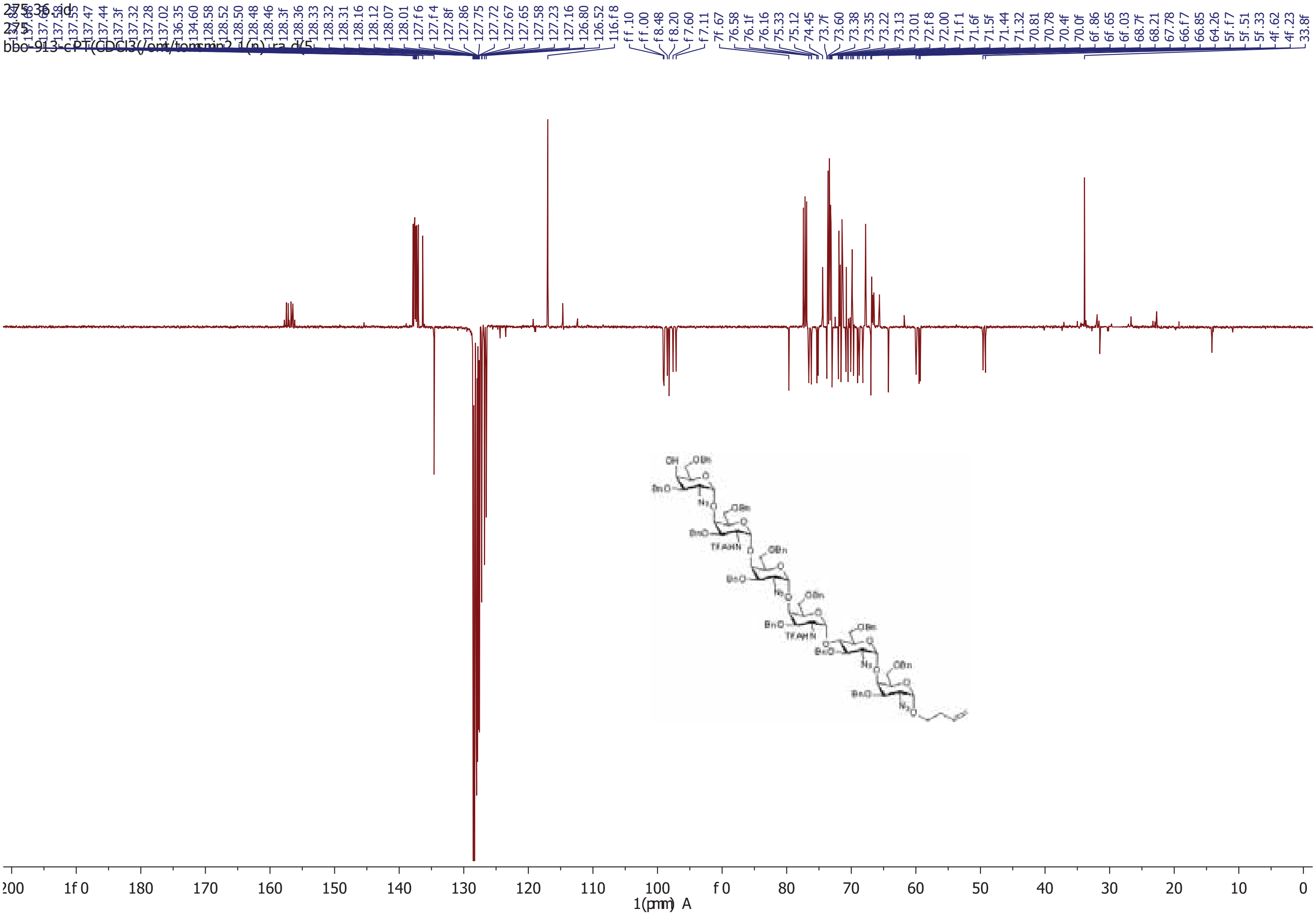

275.36.id
bbo-913-cPT(CDCl3)
1(ppm) A
200
180
170
160
150
140
130
120
110
100
80
70
60
50
40
30
20
10
0
OH
OBn
BnO
N3
TFAHN

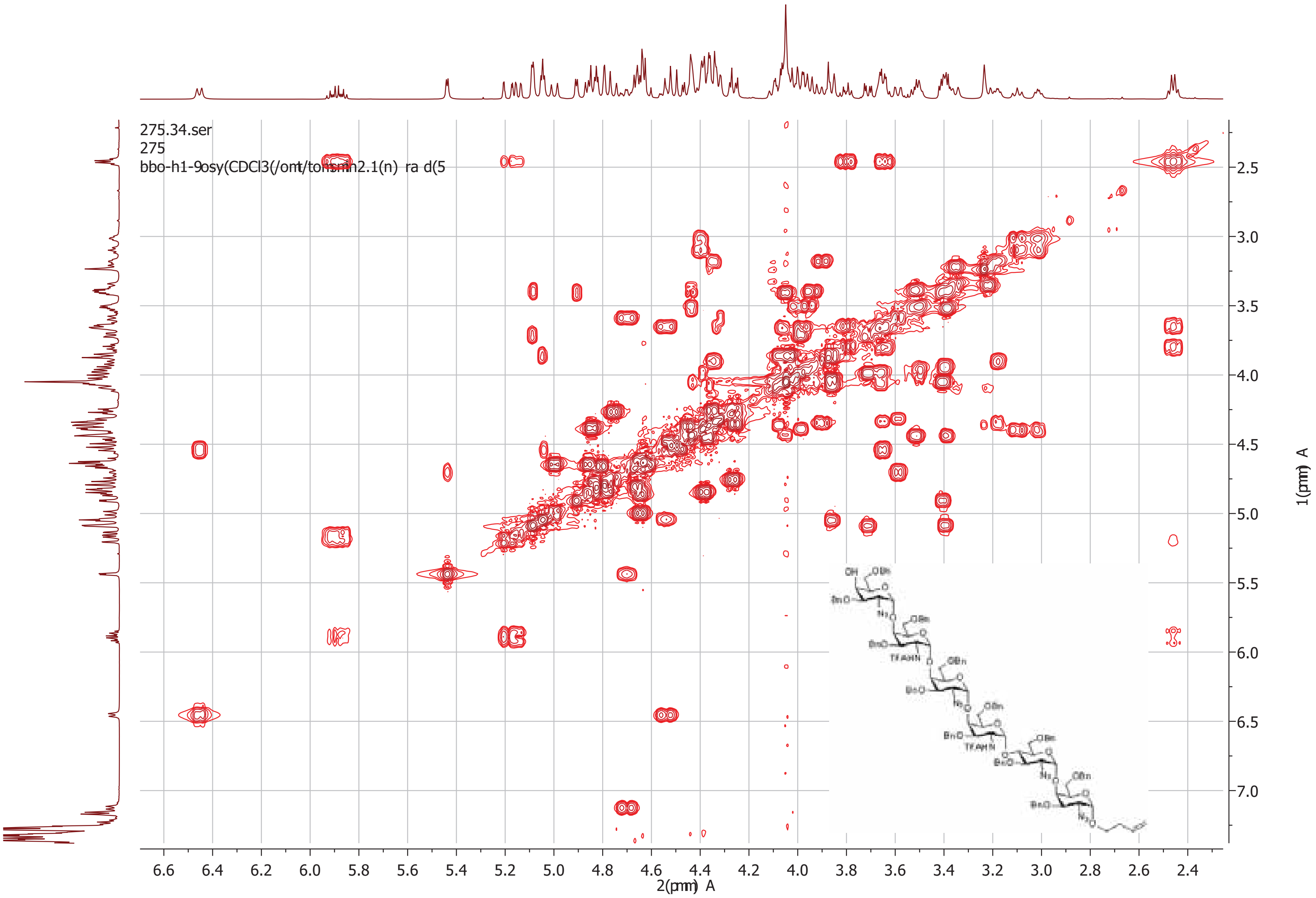
275.34.ser
275
bbo-h1-9osy(CDCl3(/omt/tonsmh2.1(n) ra d(5
2(pm) A
1(pm) A

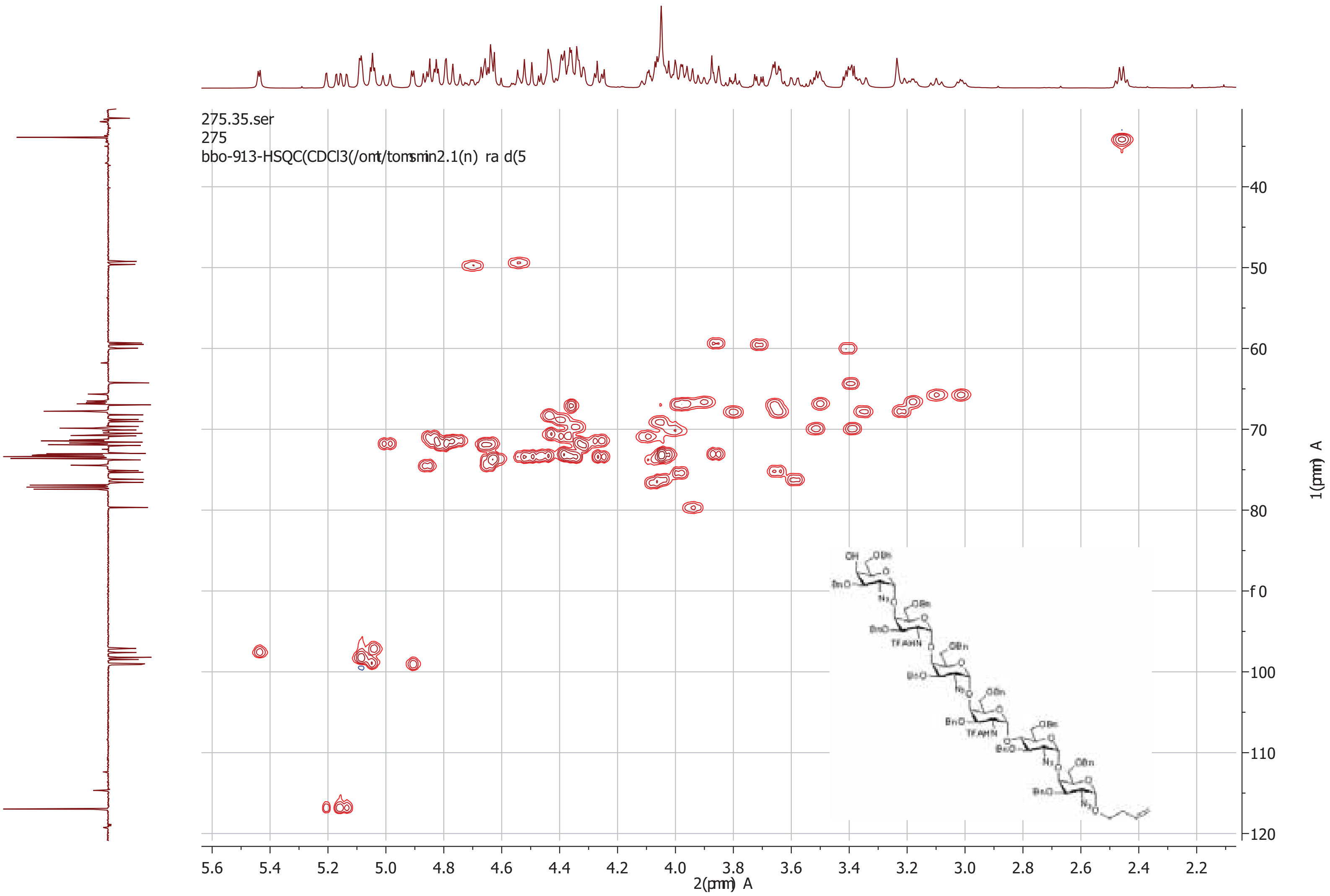
275.35.ser
275
bbo-913-HSQC(CDCl3(/ont/tomsmin2.1(n) ra d(5
2(ppm) A
1(ppm) A
f 0
OH
OBn
BnO
N3
TFAHN

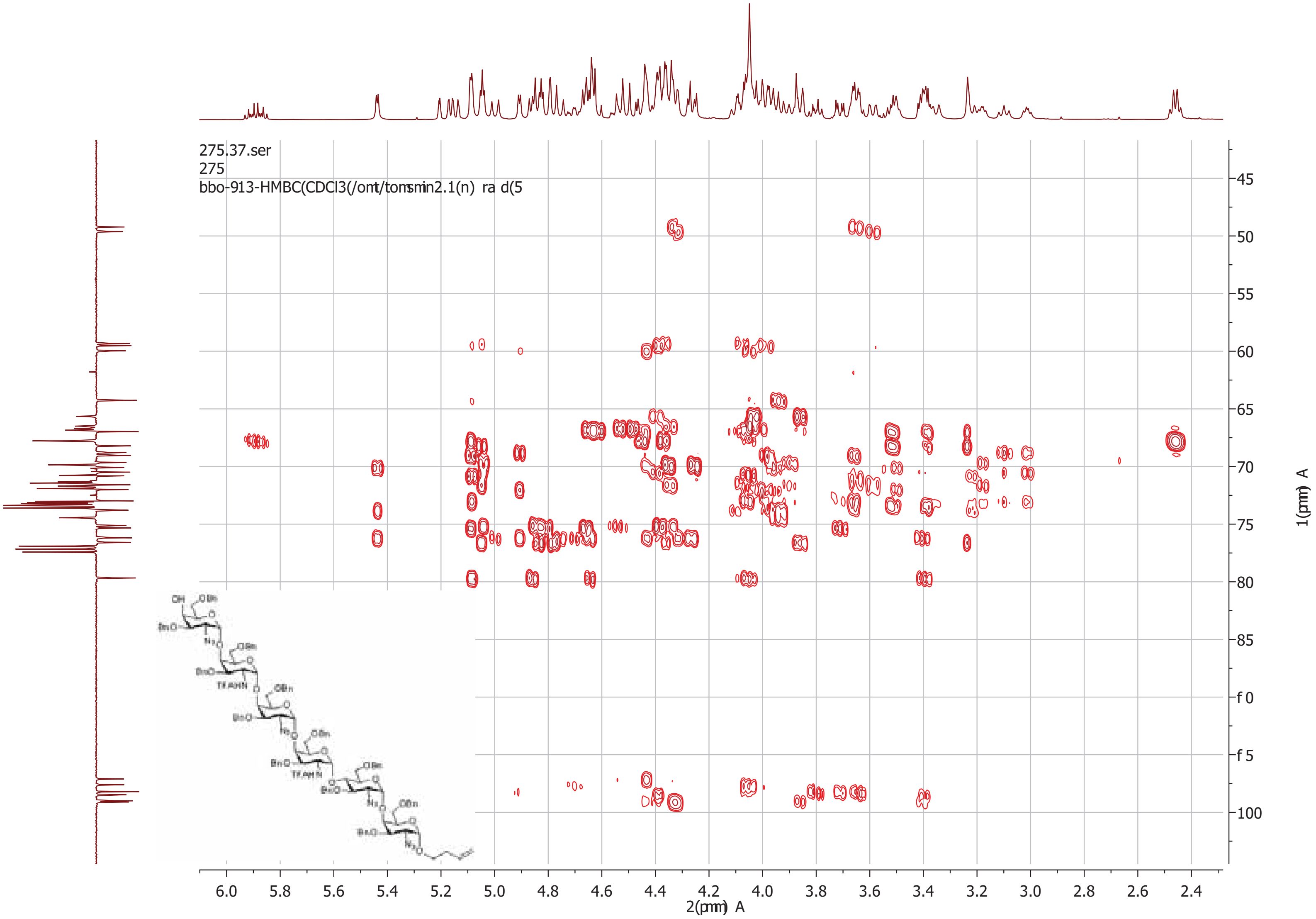
275.37.ser
275
bbo-913-HMBC(CDCl3(/omt/tomsmin2.1(n) ra d(5
2(ppm) A
1(ppm) A

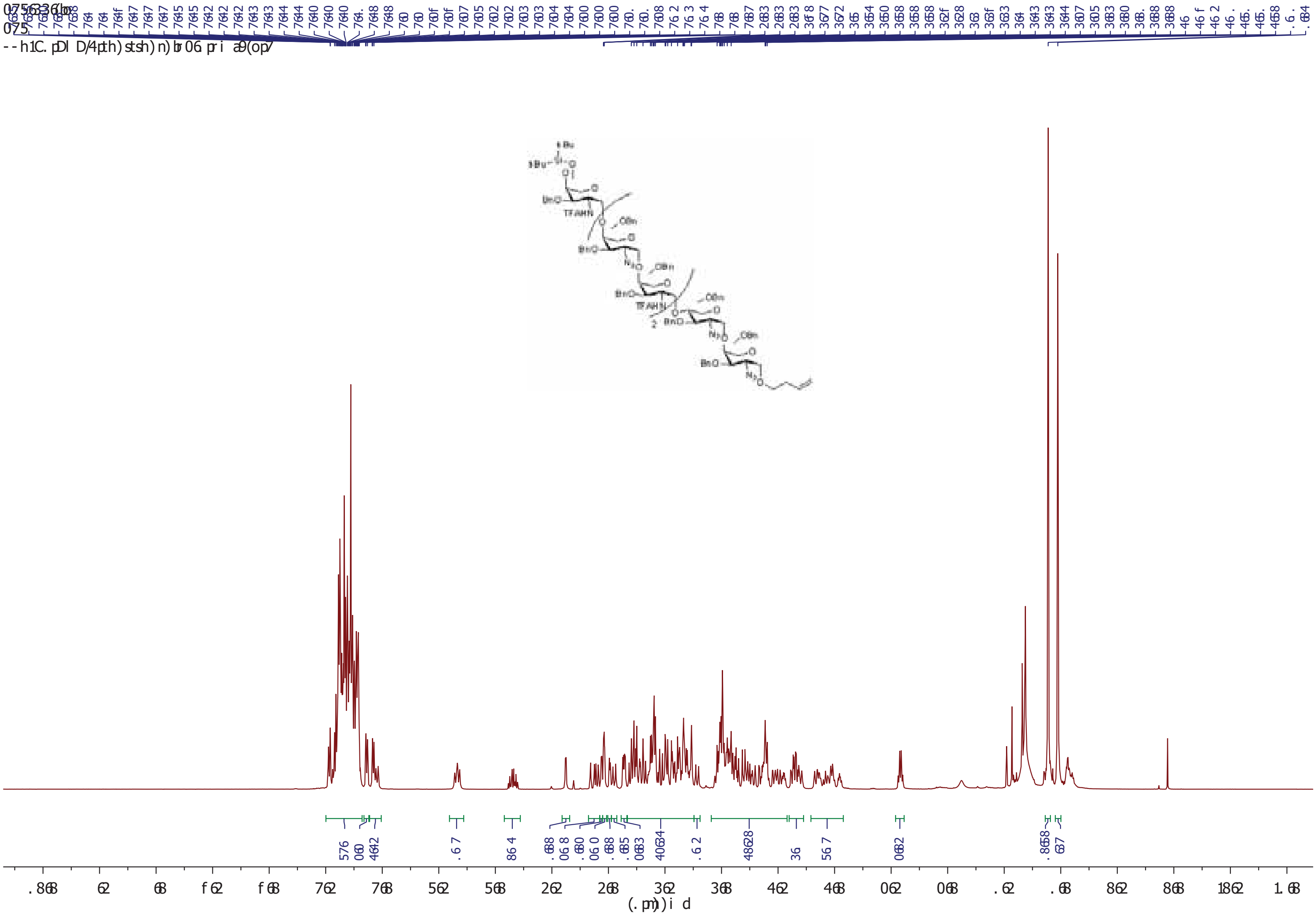

TFAHN
BnO
OBn

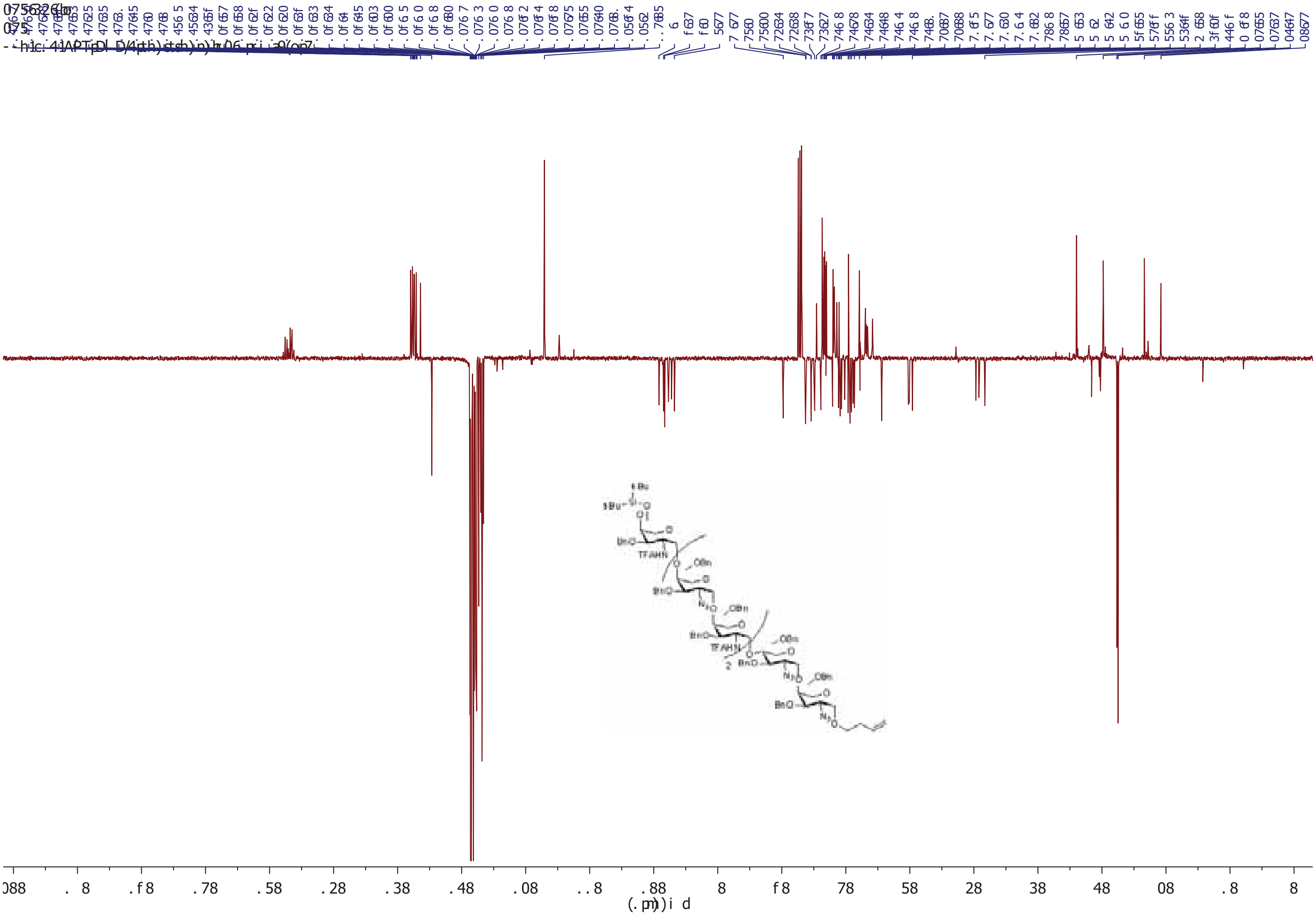

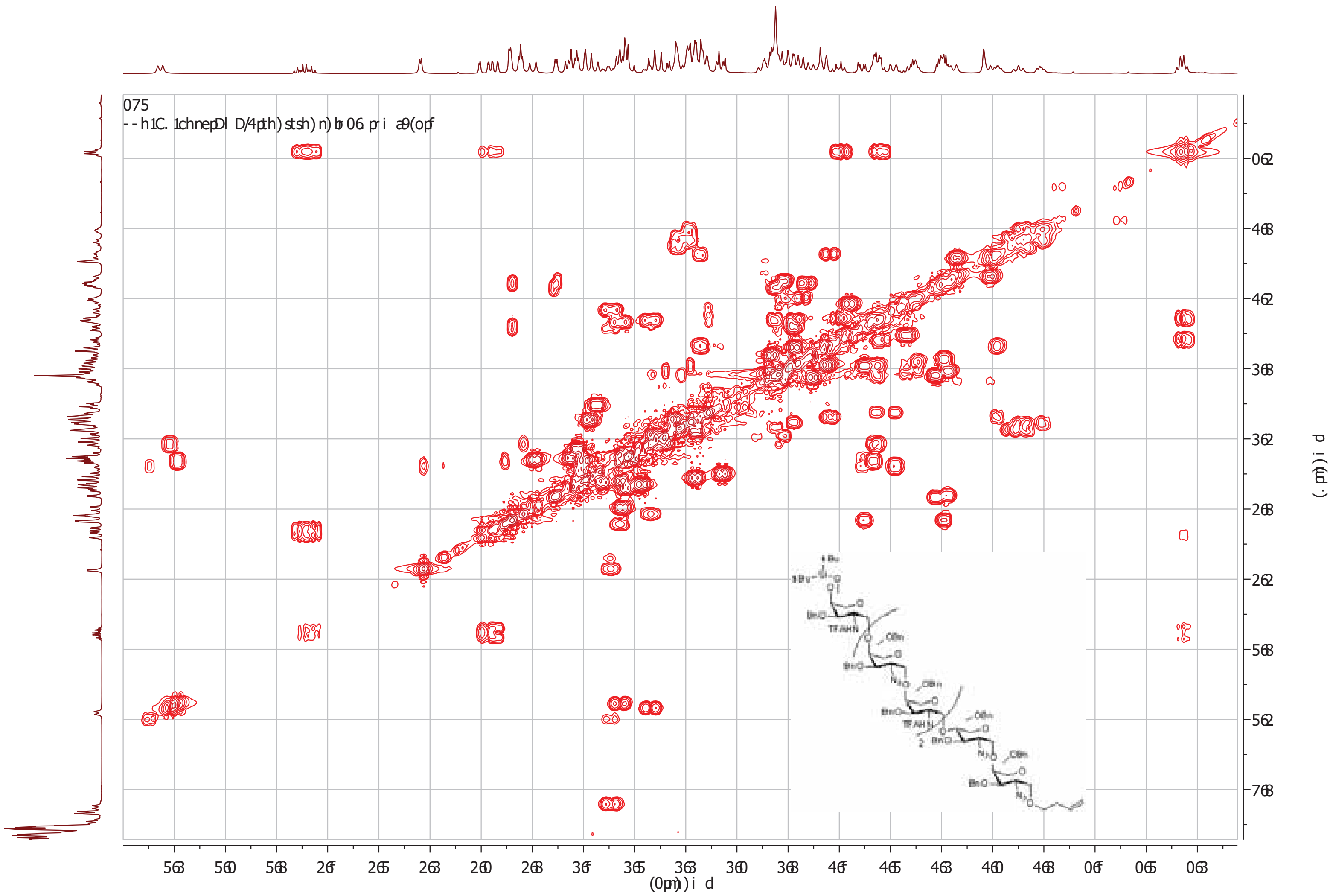

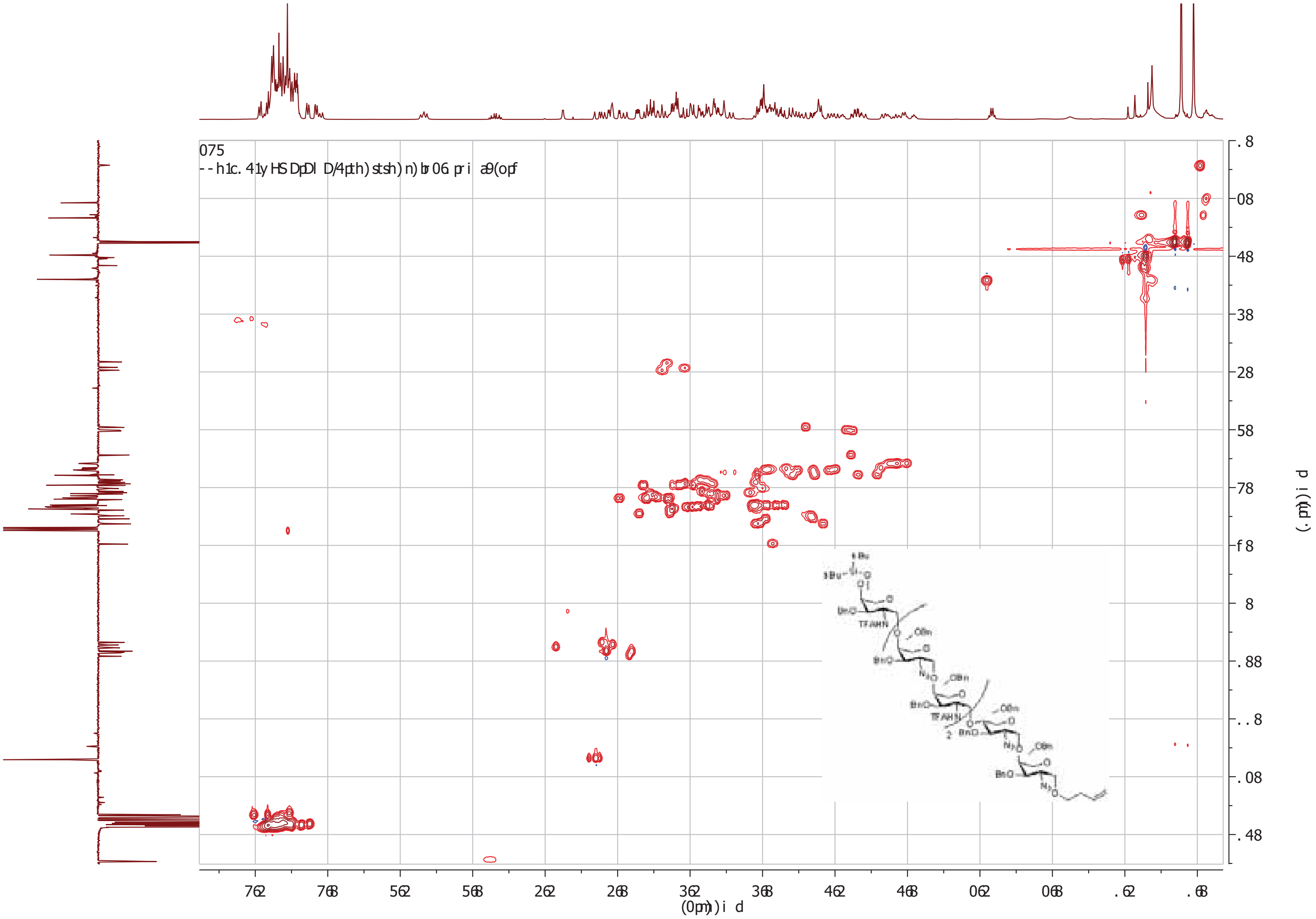

BnO
TFAHN
OBn

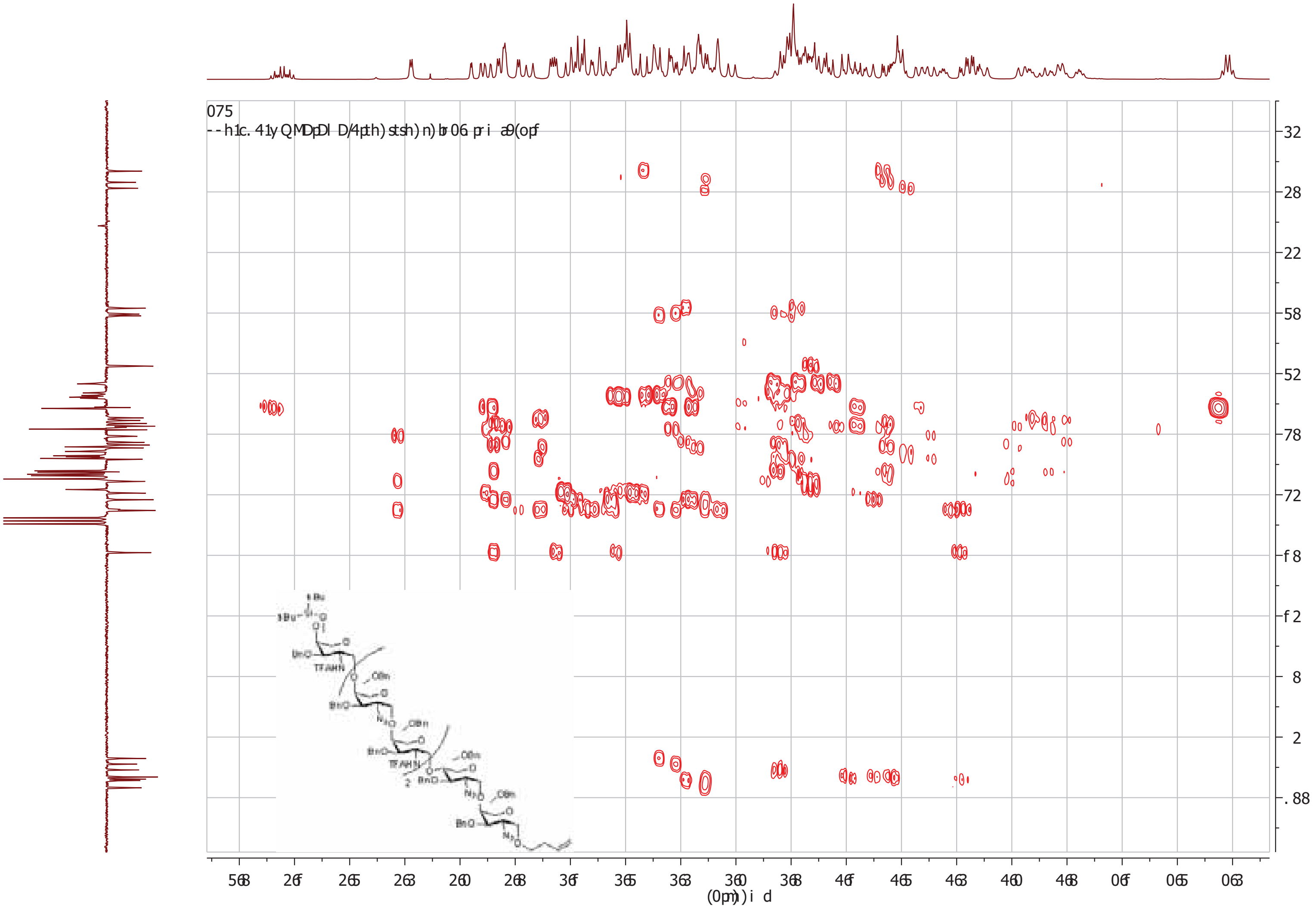

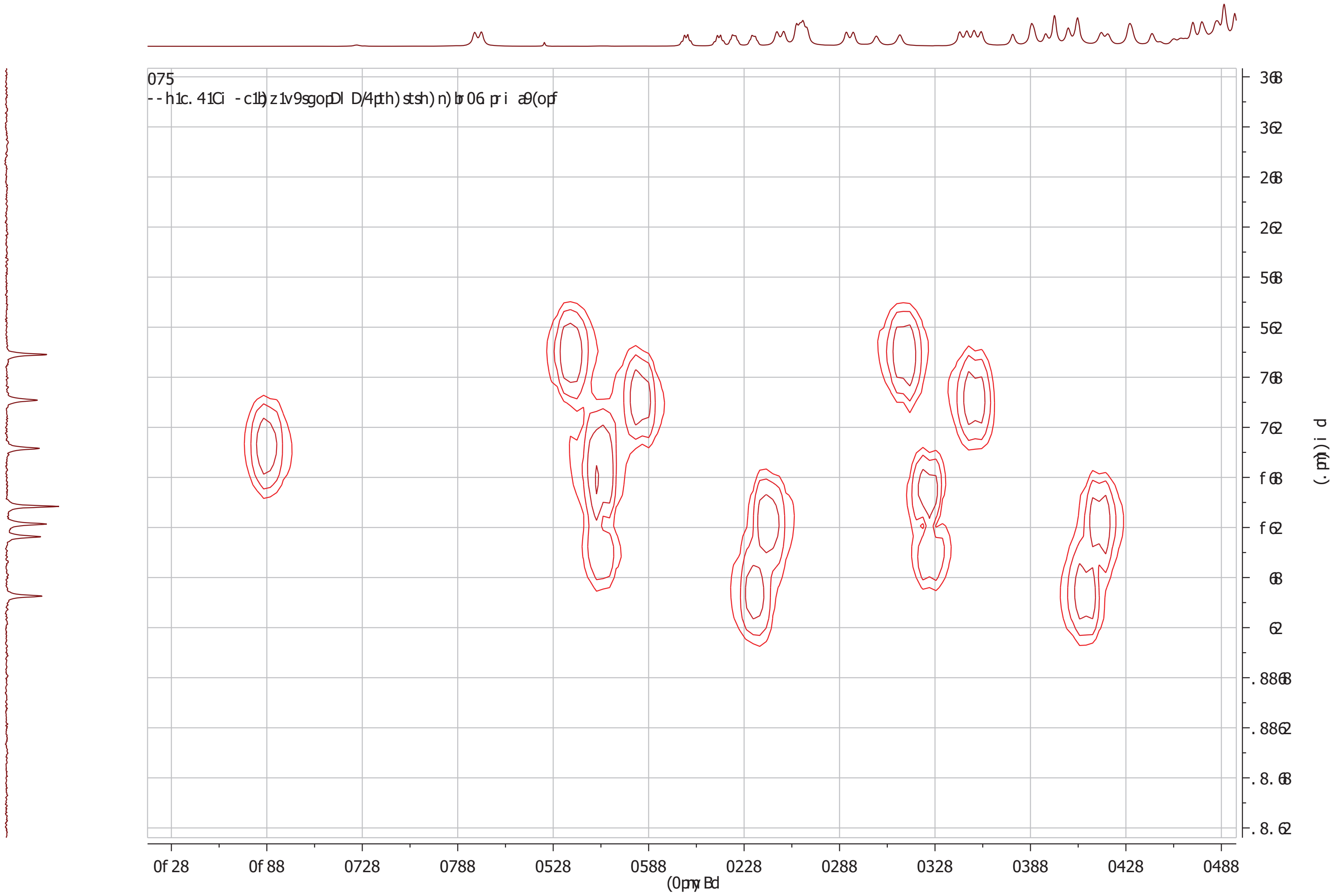

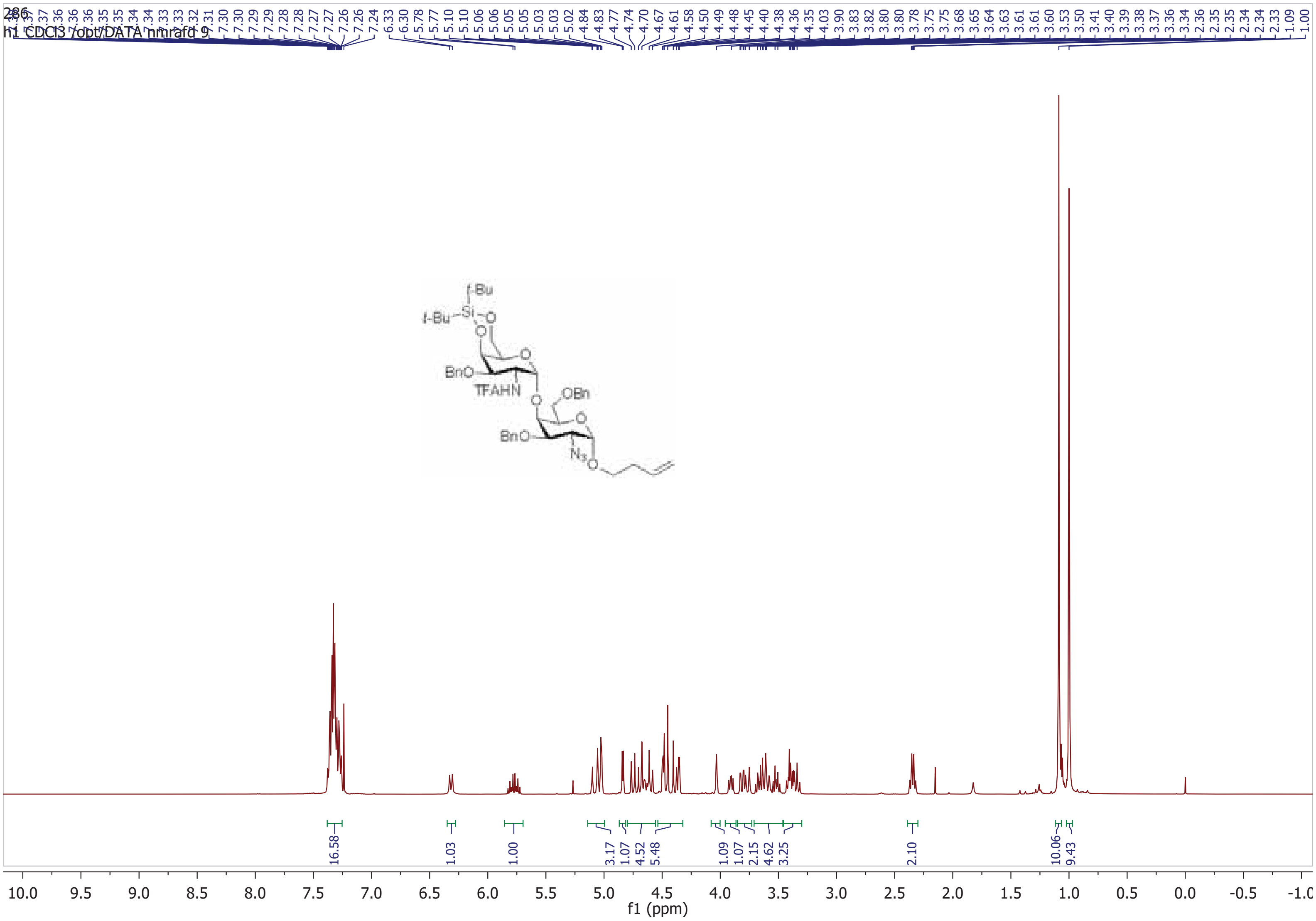

286
h1 CDCl3 /opt/DATA nmrafd 9
7.37 7.36 7.36 7.36 7.35 7.35 7.34 7.34 7.33 7.33 7.32 7.31 7.30 7.30 7.29 7.28 7.28 7.27 7.27 7.26 7.26 7.24
6.33 6.30 5.78 5.77 5.10 5.10 5.06 5.06 5.05 5.05 5.03 5.03 5.02 4.84 4.83 4.77 4.74 4.70 4.67 4.61 4.58 4.50 4.49 4.48 4.45 4.40 4.38 4.36 4.35 4.03 3.90 3.83 3.82 3.80 3.80 3.78 3.75 3.75 3.68 3.65 3.64 3.63 3.61 3.61 3.60 3.53 3.50 3.41 3.40 3.39 3.38 3.37 3.36 3.34 2.36 2.35 2.34 2.34 2.33 1.09 1.00
t-Bu
t-Bu
Si
O
BnO
TFAHN
OBn
BnO
N3
16.58
1.03
1.00
3.17
1.07
4.52
5.48
1.09
1.07
2.15
4.62
3.25
2.10
10.06
9.43
10.0 9.5 9.0 8.5 8.0 7.5 7.0 6.5 6.0 5.5 5.0 4.5 4.0 3.5 3.0 2.5 2.0 1.5 1.0 0.5 0.0 -0.5 -1.0
f1 (ppm)

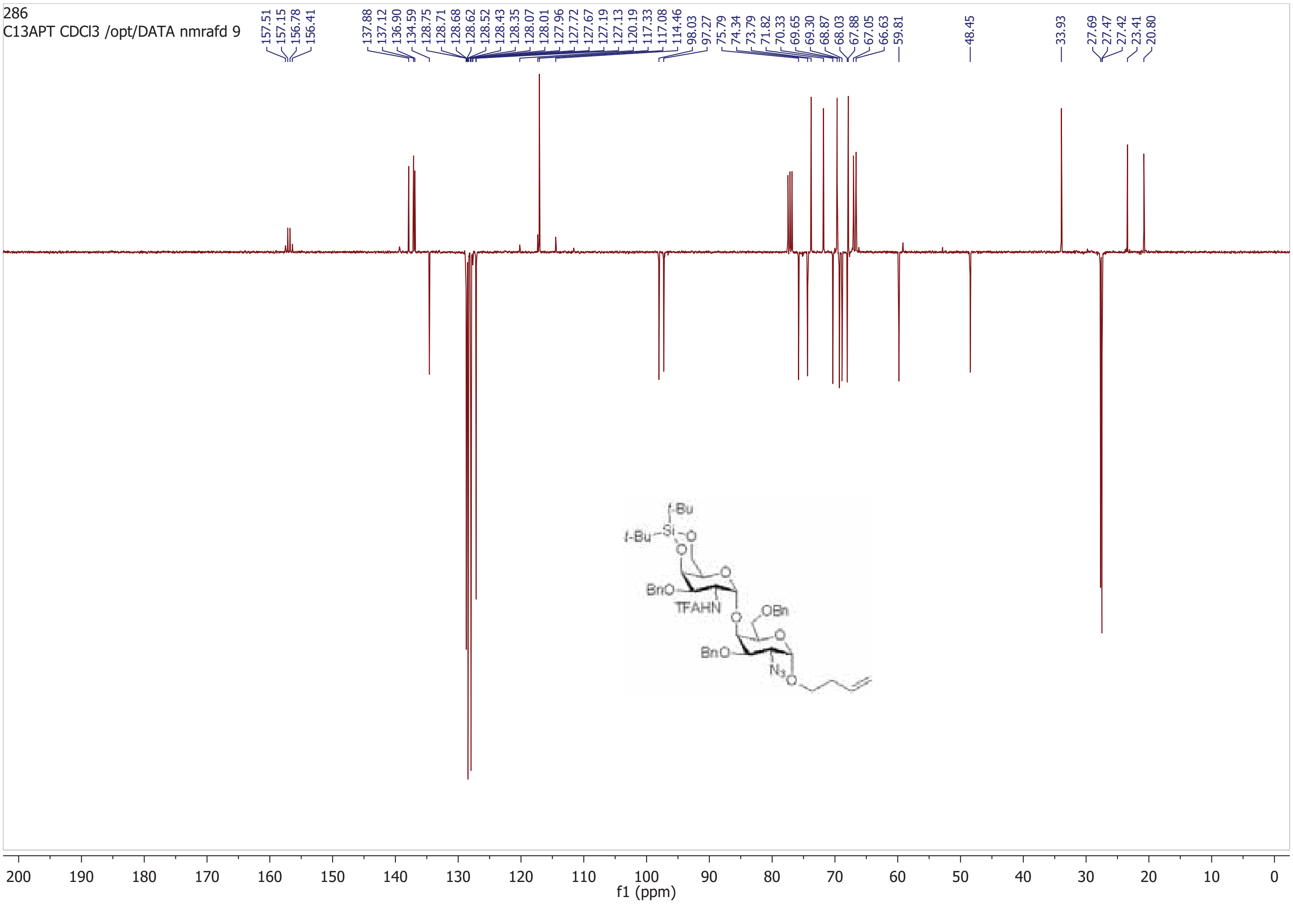
286
C13APT CDCl3 /opt/DATA nmrafd 9
157.51
157.15
156.78
156.41
137.88
137.12
136.90
134.59
128.75
128.71
128.68
128.62
128.52
128.43
128.35
128.07
128.01
127.96
127.72
127.67
127.19
127.13
120.19
117.33
117.08
114.46
98.03
97.27
75.79
74.34
73.79
71.82
70.33
69.65
69.30
68.87
68.03
67.88
67.05
66.63
59.81
48.45
33.93
27.69
27.47
27.42
23.41
20.80
t-Bu
t-Bu
Si
O
O
BnO
TFAHN
O
O
OBn
BnO
N3
O
200 190 180 170 160 150 140 130 120 110 100 90 80 70 60 50 40 30 20 10 0
f1 (ppm)

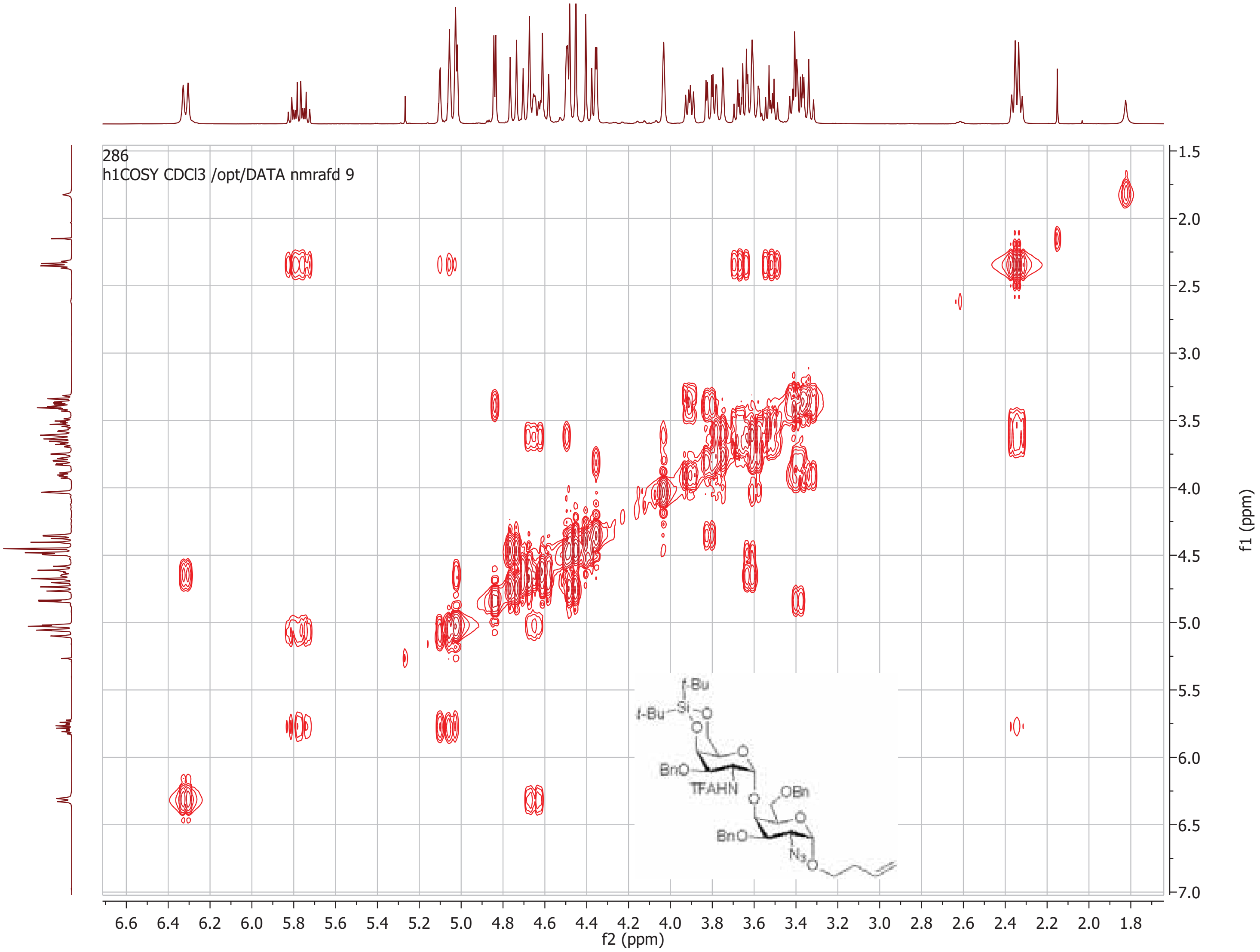

286
h1COSY CDCl3 /opt/DATA nmrafd 9
f2 (ppm)
f1 (ppm)
t-Bu
t-Bu
Si
O
BnO
TFAHN
OBn
BnO
N3

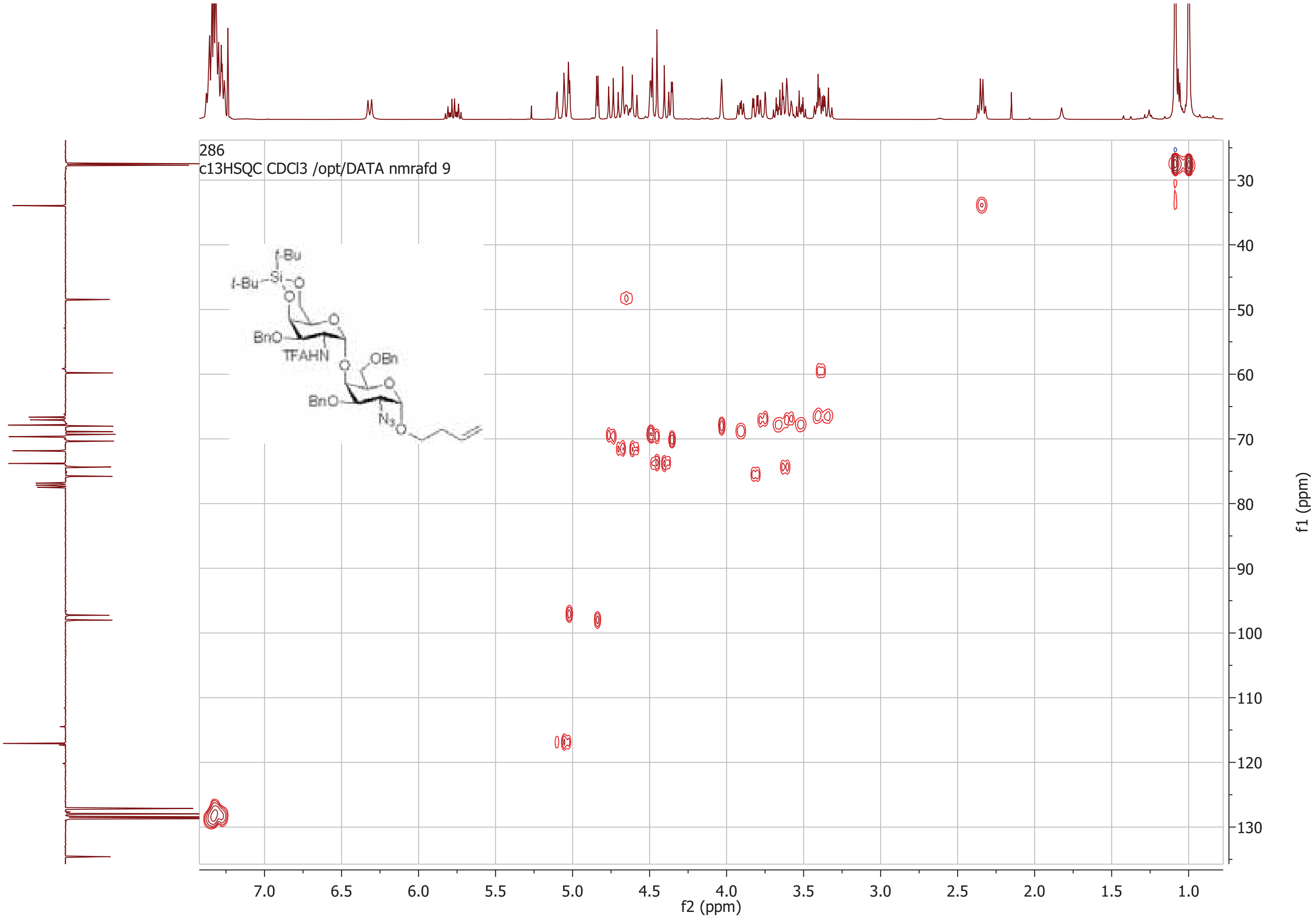
286
c13HSQC CDCl3 /opt/DATA nmrafd 9
t-Bu
t-Bu
Si
BnO
TFAHN
OBn
BnO
N3
f2 (ppm)
f1 (ppm)

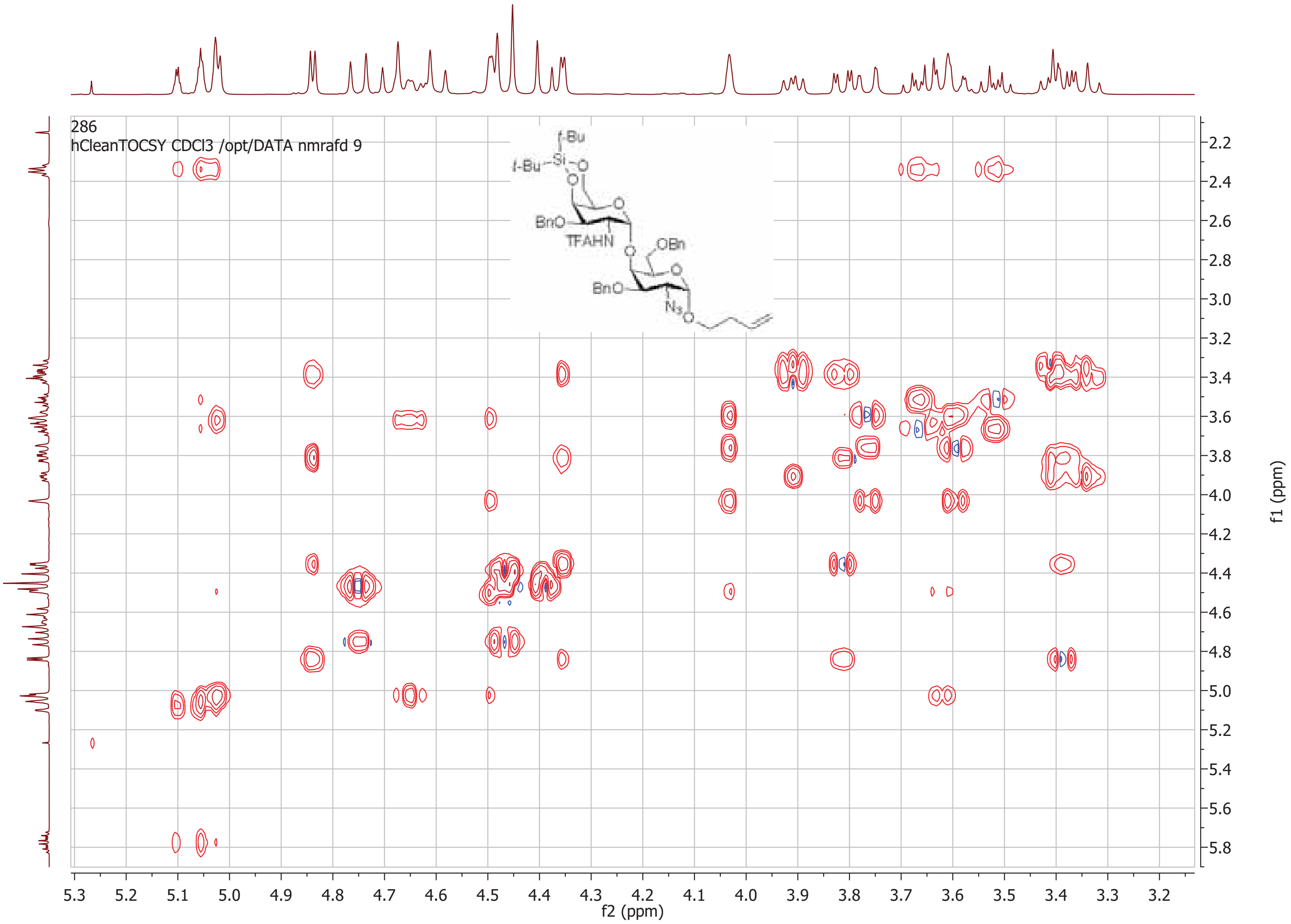

286
hCleanTOCSY CDCl3 /opt/DATA nmrafd 9
t-Bu
t-Bu
Si
BnO
TFAHN
OBn
BnO
N3
f2 (ppm)
f1 (ppm)

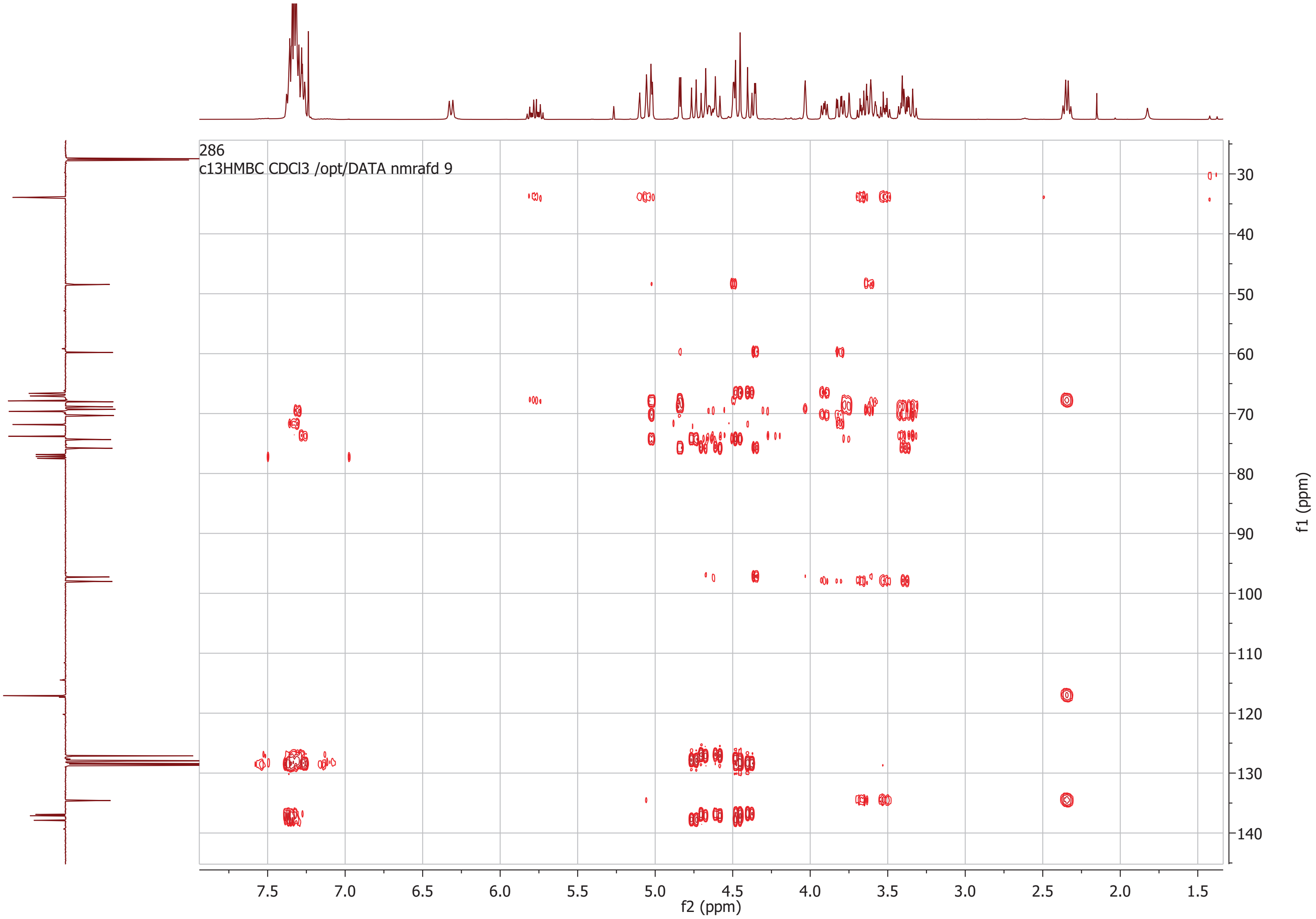
286
c13HMBC CDCl3 /opt/DATA nmrafd 9
f2 (ppm)
f1 (ppm)
7.5
7.0
6.5
6.0
5.5
5.0
4.5
4.0
3.5
3.0
2.5
2.0
1.5
30
40
50
60
70
80
90
100
110
120
130
140

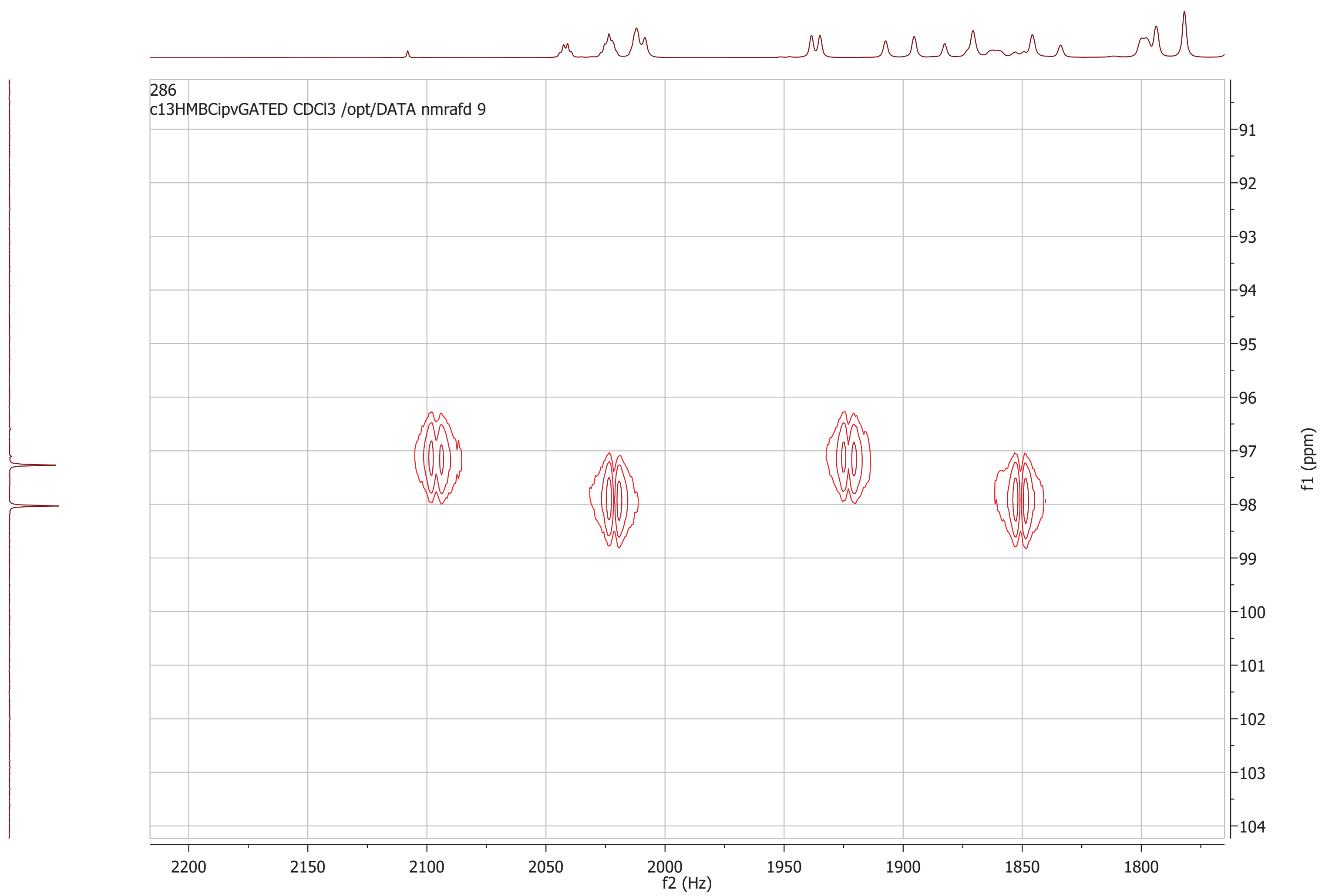

286
c13HMBCipvGATED CDCl3 /opt/DATA nmrafd 9
f2 (Hz)
f1 (ppm)

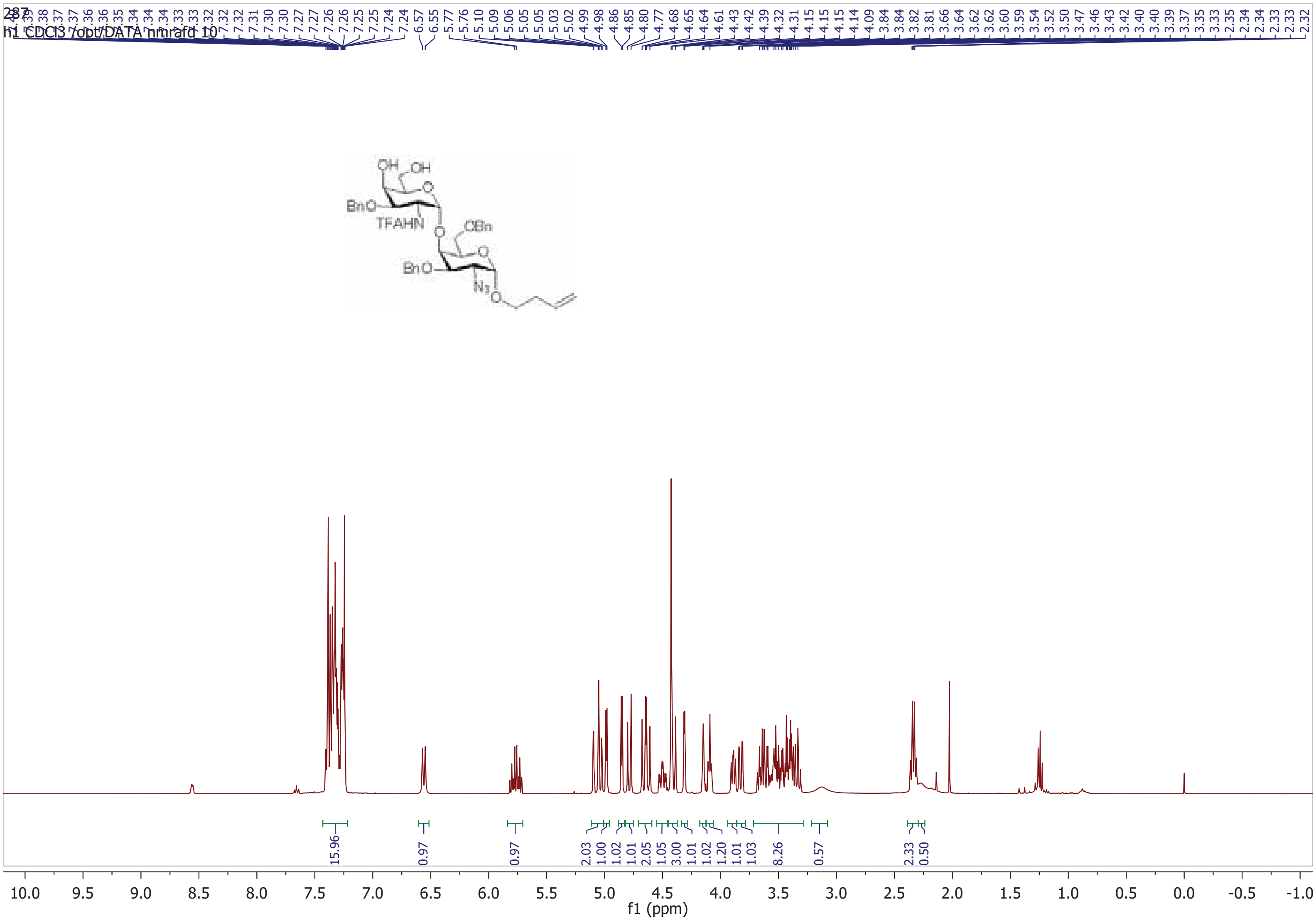
287
h1 CDCl3 /opt/DATA nmrafd 10
7.38 7.37 7.37 7.36 7.36 7.35 7.34 7.34 7.34 7.33 7.33 7.32 7.32 7.31 7.30 7.30 7.27 7.27 7.26 7.26 7.25 7.25 7.24 7.24 6.57 6.55 5.77 5.76 5.10 5.09 5.06 5.05 5.05 5.03 5.02 4.99 4.98 4.86 4.85 4.80 4.77 4.68 4.65 4.64 4.61 4.43 4.42 4.39 4.32 4.31 4.15 4.15 4.15 4.14 4.09 3.84 3.84 3.82 3.81 3.66 3.64 3.62 3.62 3.60 3.59 3.54 3.52 3.50 3.47 3.46 3.43 3.42 3.40 3.39 3.37 3.35 3.33 2.35 2.34 2.34 2.33 2.33 2.32
OH
OH
BnO
TFAHN
OBn
BnO
N3
15.96
0.97
0.97
2.03
1.00
1.02
1.01
2.05
1.05
3.00
1.01
1.02
1.20
1.01
1.03
8.26
0.57
2.33
0.50
10.0 9.5 9.0 8.5 8.0 7.5 7.0 6.5 6.0 5.5 5.0 4.5 4.0 3.5 3.0 2.5 2.0 1.5 1.0 0.5 0.0 -0.5 -1.0
f1 (ppm)

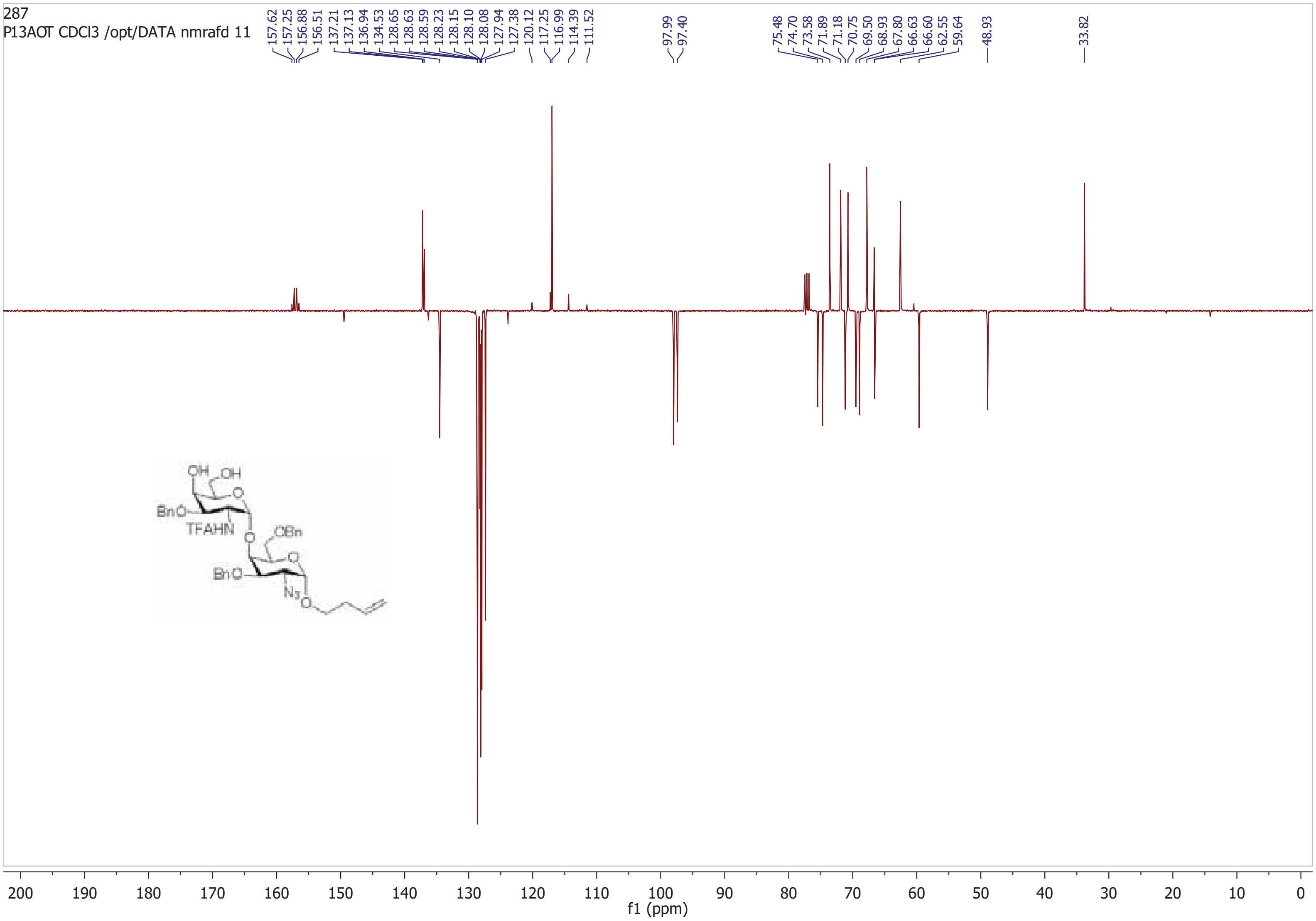
287
P13AOT CDCl3 /opt/DATA nmrafd 11
157.62
157.25
156.88
156.51
137.21
137.13
136.94
134.53
128.65
128.63
128.59
128.23
128.15
128.10
128.08
127.94
127.38
120.12
117.25
116.99
114.39
111.52
97.99
97.40
75.48
74.70
73.58
71.89
71.18
70.75
69.50
68.93
67.80
66.63
66.60
62.55
59.64
48.93
33.82
OH
OH
BnO
TFAHN
OBn
BnO
N3
200
190
180
170
160
150
140
130
120
110
100
90
80
70
60
50
40
30
20
10
0
f1 (ppm)

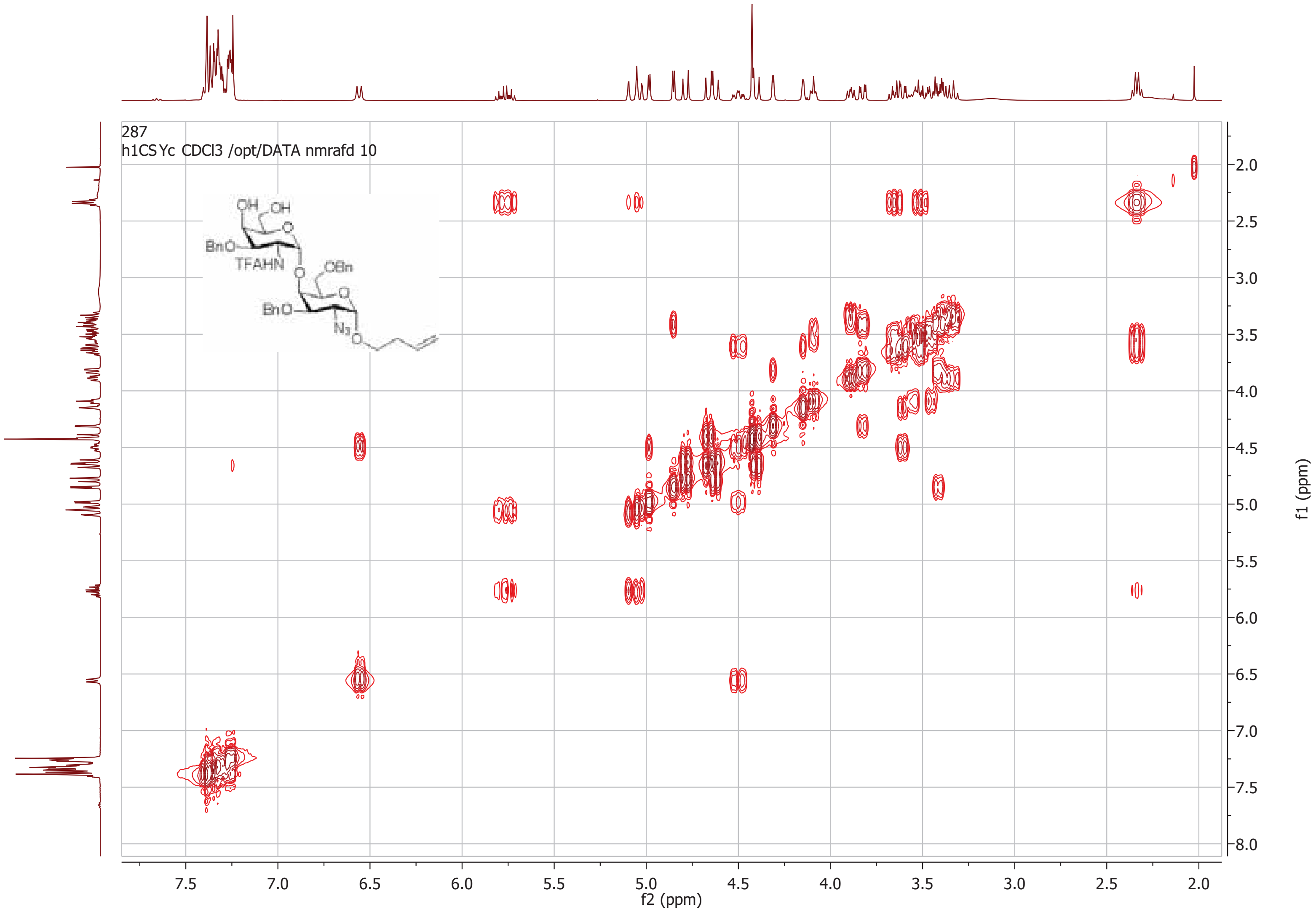
287
h1CSYc CDCl3 /opt/DATA nmrafd 10
OH
OH
BnO
TFAHN
OBn
BnO
N3
f2 (ppm)
f1 (ppm)
7.5
7.0
6.5
6.0
5.5
5.0
4.5
4.0
3.5
3.0
2.5
2.0
8.0

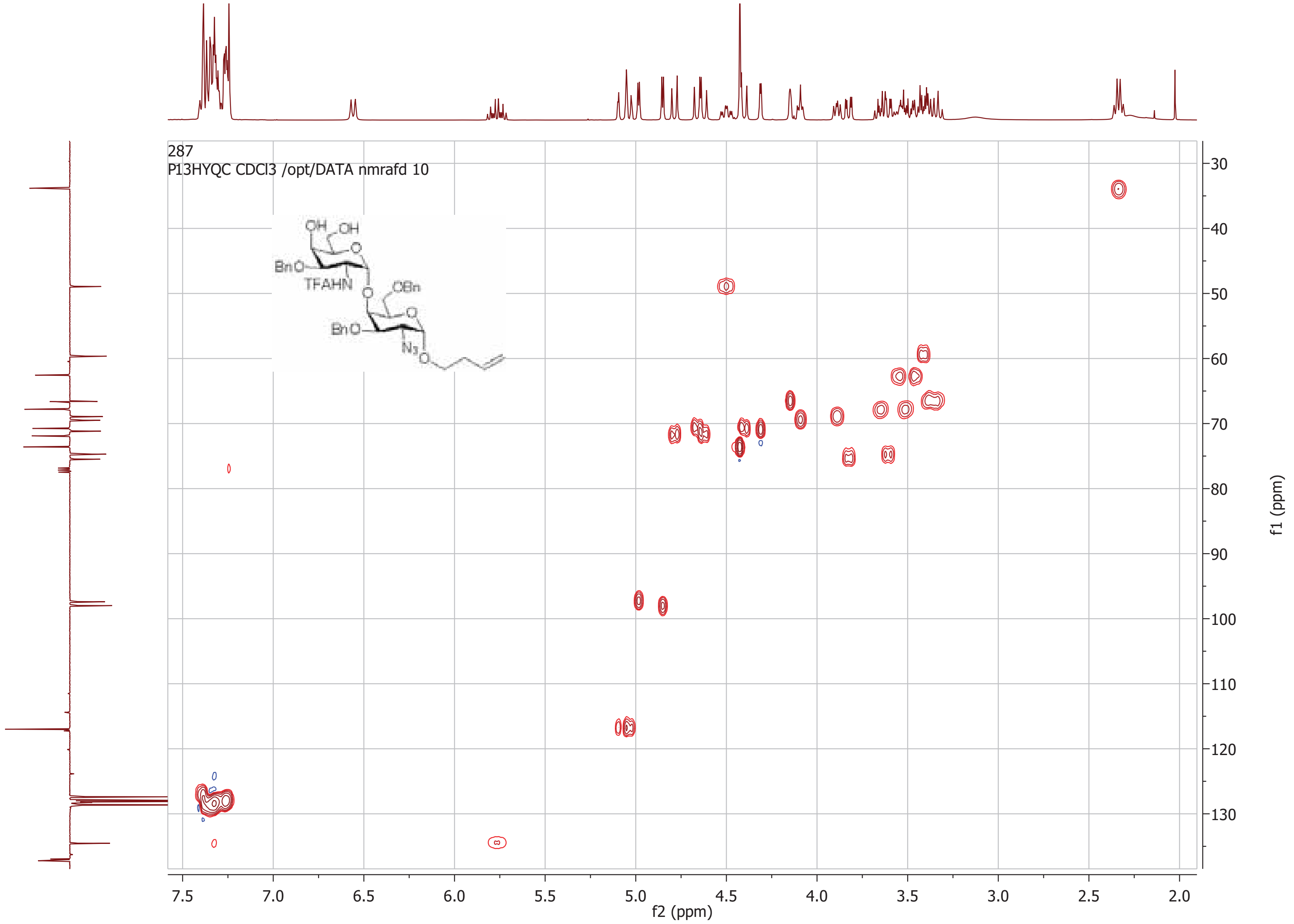
287
P13HYQC CDCl3 /opt/DATA nmrafd 10
OH
OH
BnO
TFAHN
OBn
BnO
N3
f2 (ppm)
f1 (ppm)

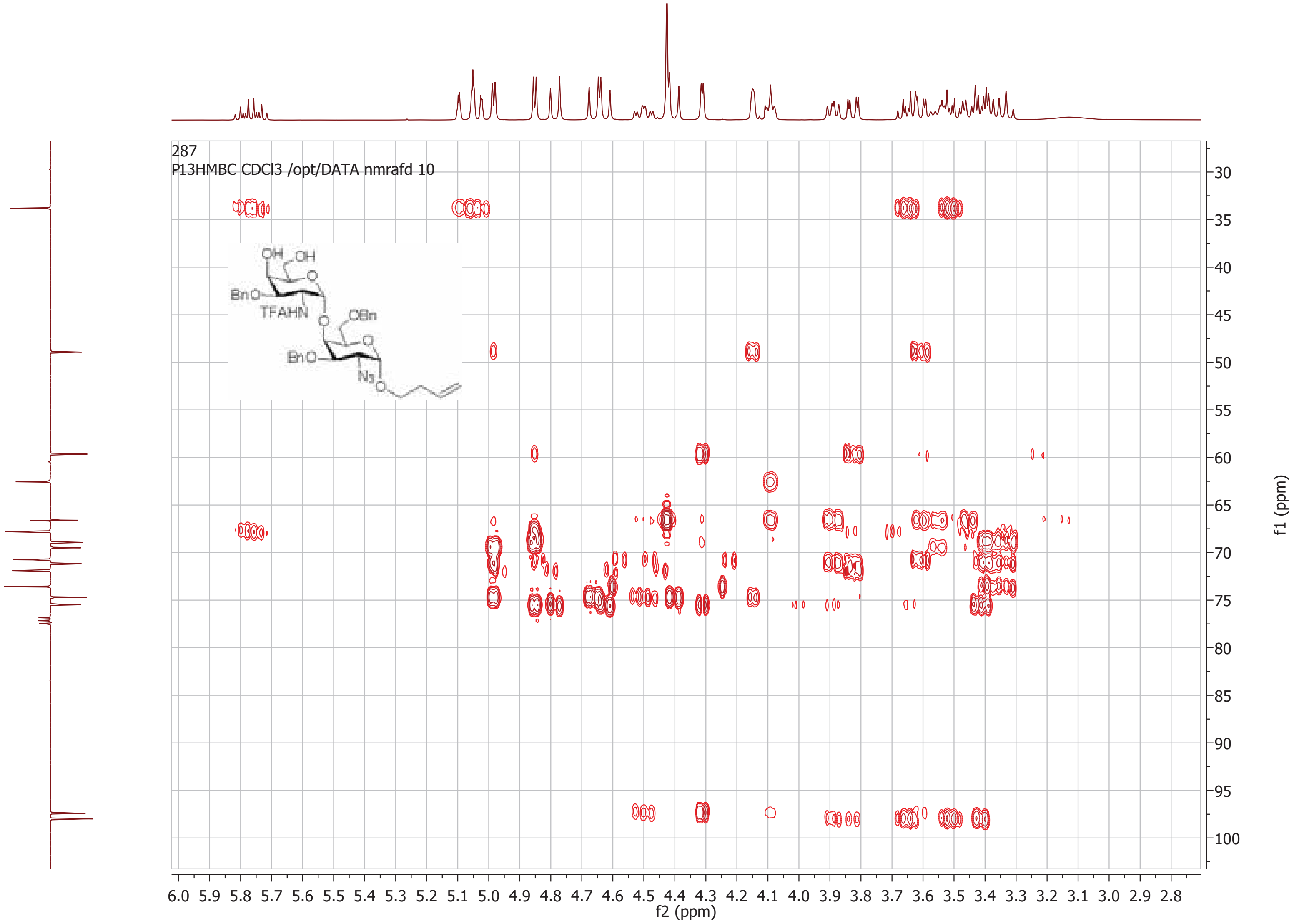

287
P13HMBC CDCl3 /opt/DATA nmrafd 10
OH
OH
BnO
TFAHN
OBn
BnO
N3
f2 (ppm)
f1 (ppm)

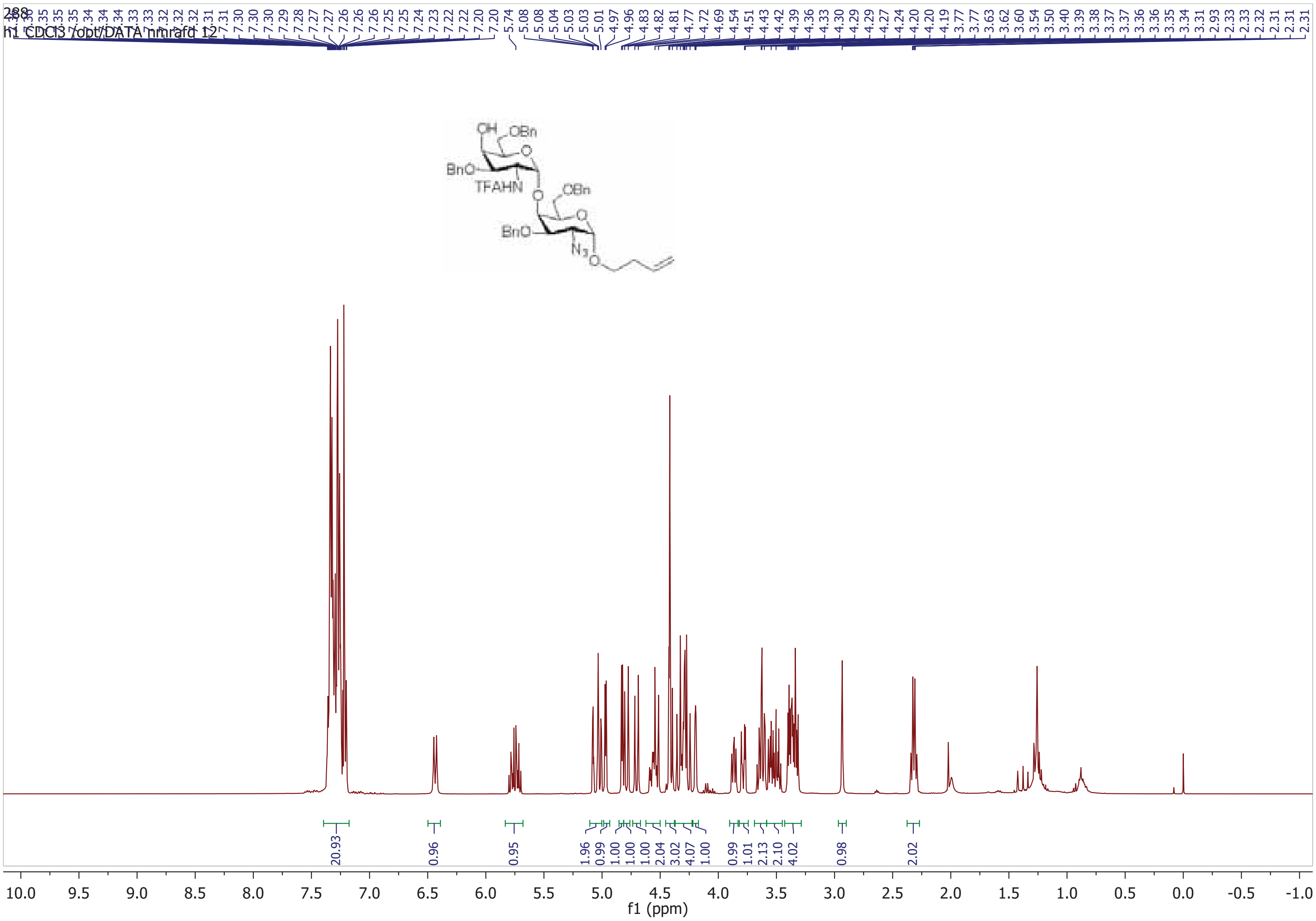
288
h1 CDCl3 /opt/DATA nmrafd 12
7.35 7.35 7.34 7.34 7.33 7.33 7.32 7.32 7.32 7.31 7.31 7.30 7.30 7.29 7.28 7.27 7.27 7.26 7.26 7.25 7.25 7.24 7.23 7.22 7.22 7.20 7.20 5.74 5.08 5.08 5.04 5.03 5.03 5.01 4.97 4.96 4.83 4.82 4.81 4.77 4.72 4.69 4.54 4.51 4.43 4.42 4.39 4.36 4.33 4.30 4.29 4.29 4.27 4.24 4.20 4.20 4.19 3.77 3.77 3.63 3.62 3.60 3.54 3.50 3.40 3.39 3.38 3.37 3.37 3.36 3.36 3.35 3.34 3.31 2.93 2.33 2.33 2.32 2.31 2.31 2.31
OH
OBn
BnO
TFAHN
OBn
BnO
N3
20.93
0.96
0.95
1.96
0.99
1.00
1.00
1.00
2.04
3.02
4.07
1.00
0.99
1.01
2.13
2.10
4.02
0.98
2.02
10.0 9.5 9.0 8.5 8.0 7.5 7.0 6.5 6.0 5.5 5.0 4.5 4.0 3.5 3.0 2.5 2.0 1.5 1.0 0.5 0.0 -0.5 -1.0
f1 (ppm)

288
C13APT CDCl3 /opt/DATA nmrafd 12

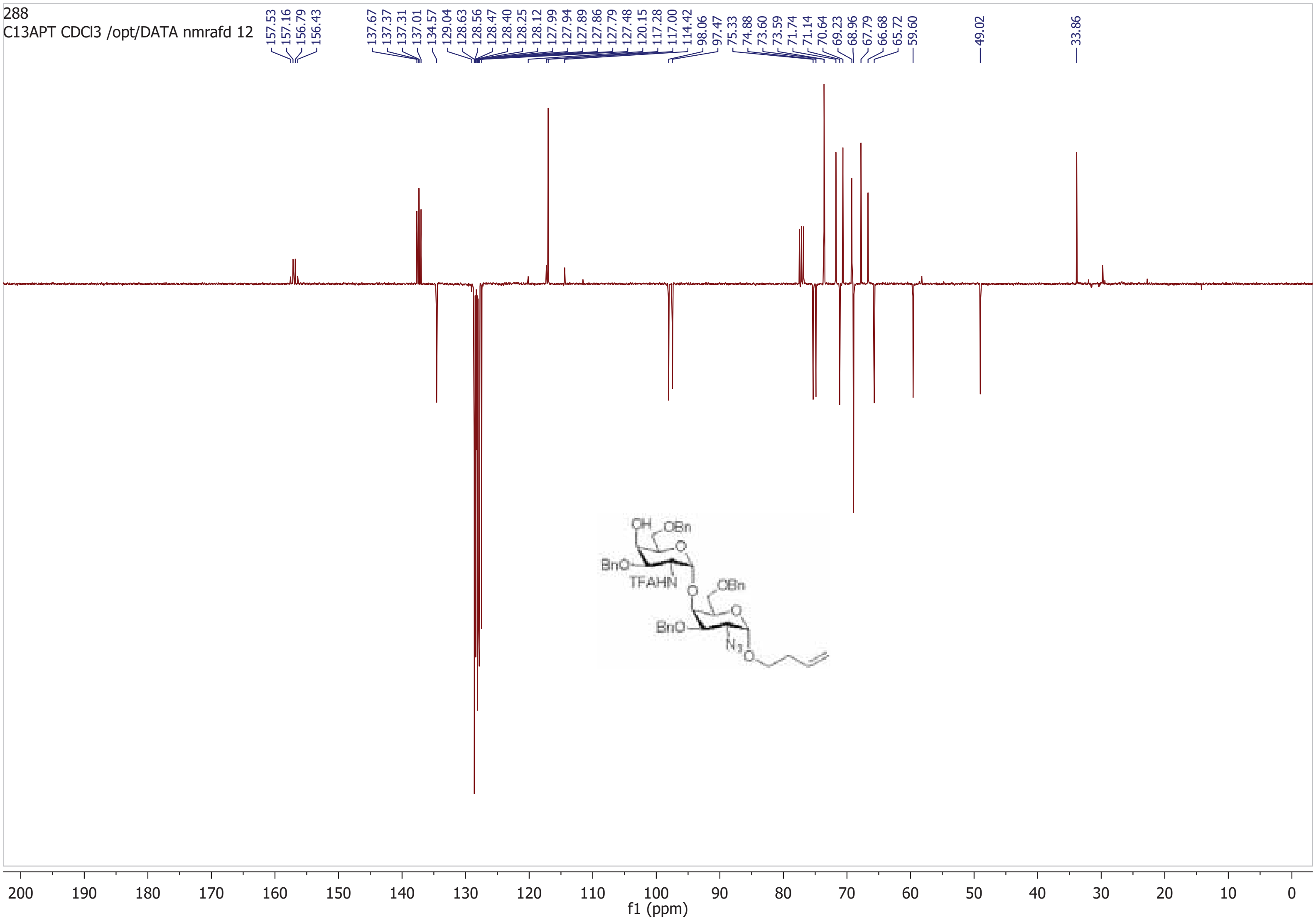

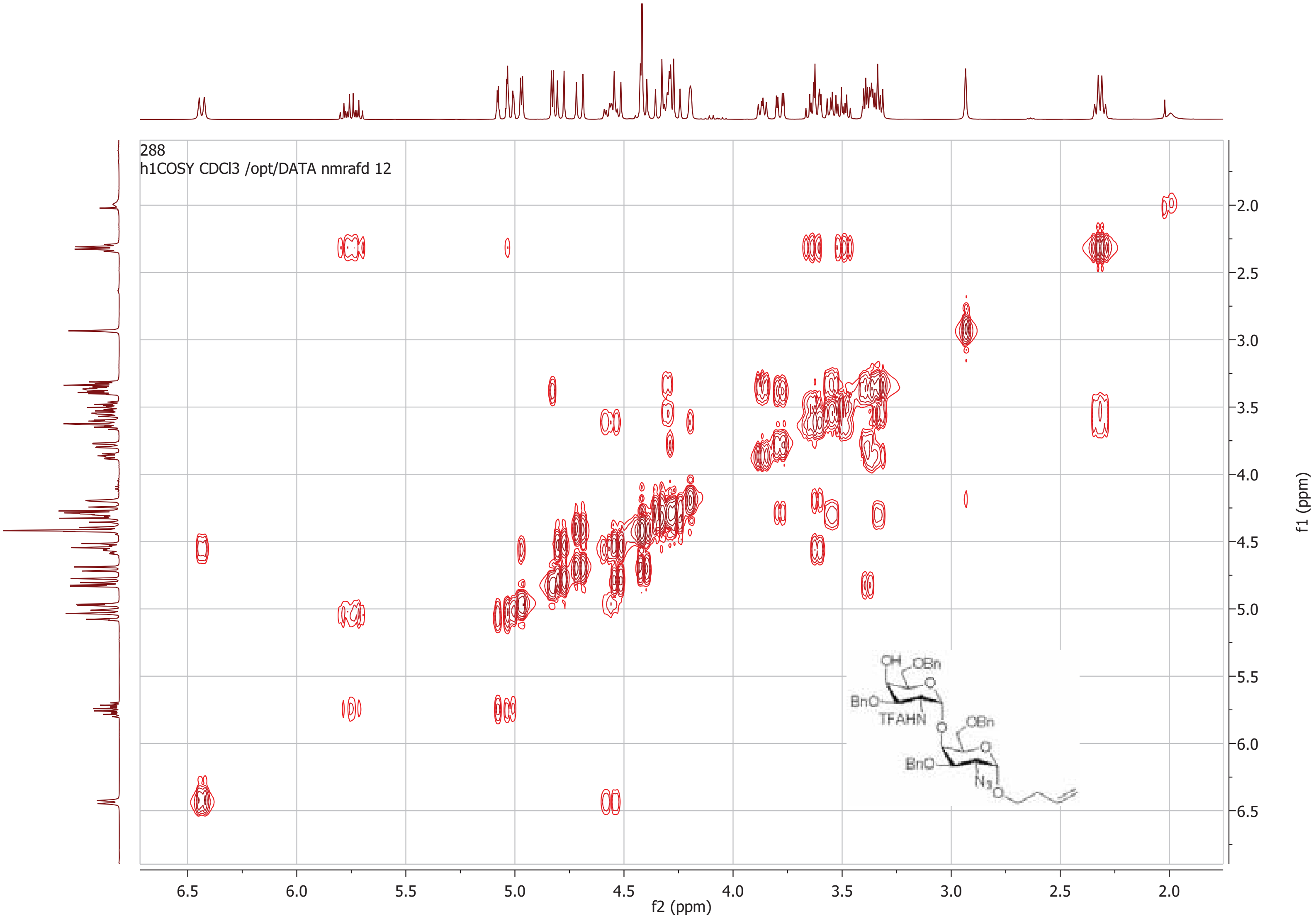
288
h1COSY CDCl3 /opt/DATA nmrafd 12
f2 (ppm)
f1 (ppm)
OH
OBn
BnO
TFAHN
O
OBn
BnO
N_3

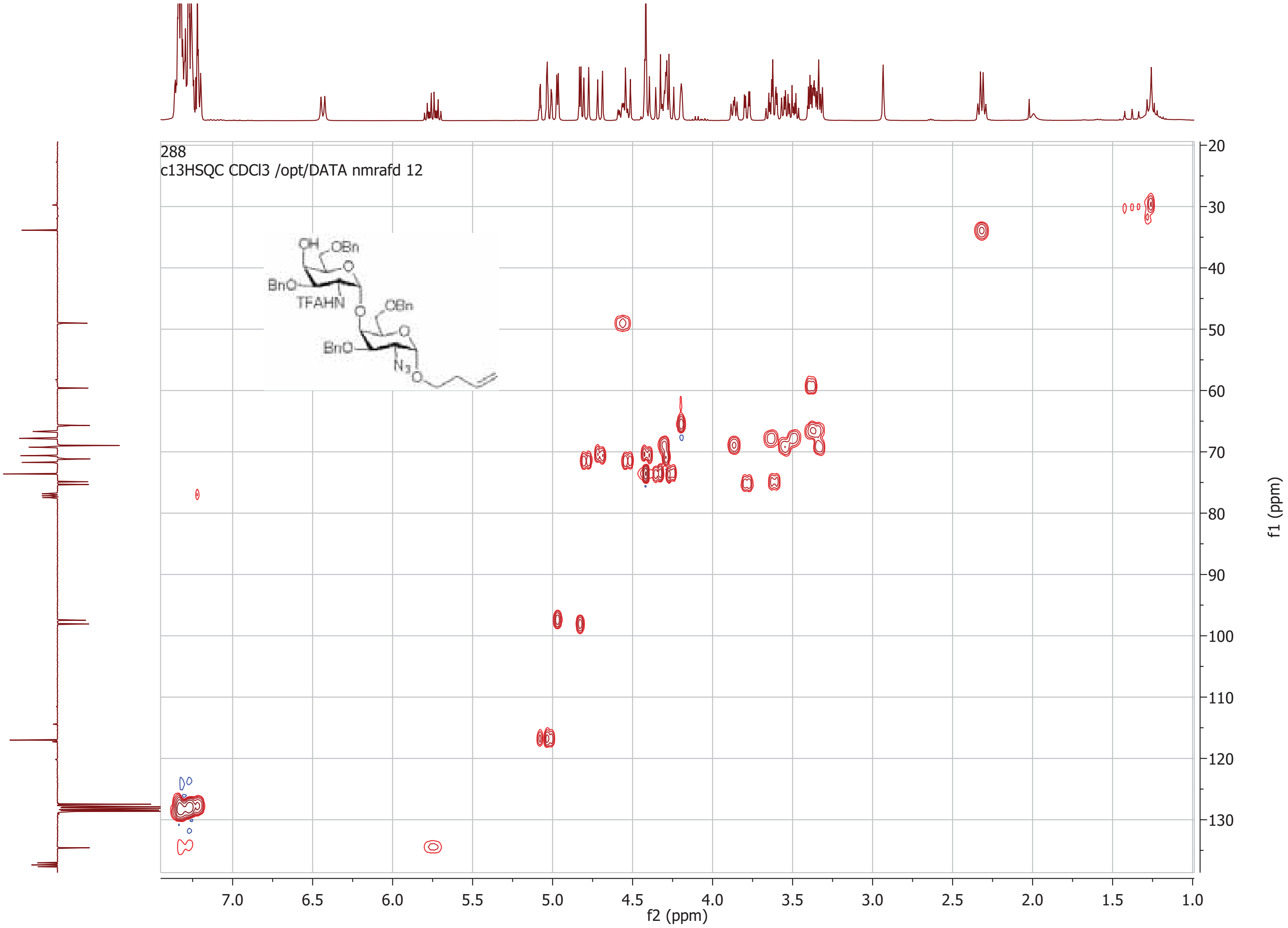
288
c13HSQC CDCl3 /opt/DATA nmrafd 12
OH
OBn
BnO
TFAHN
O
OBn
BnO
N3
f2 (ppm)
f1 (ppm)

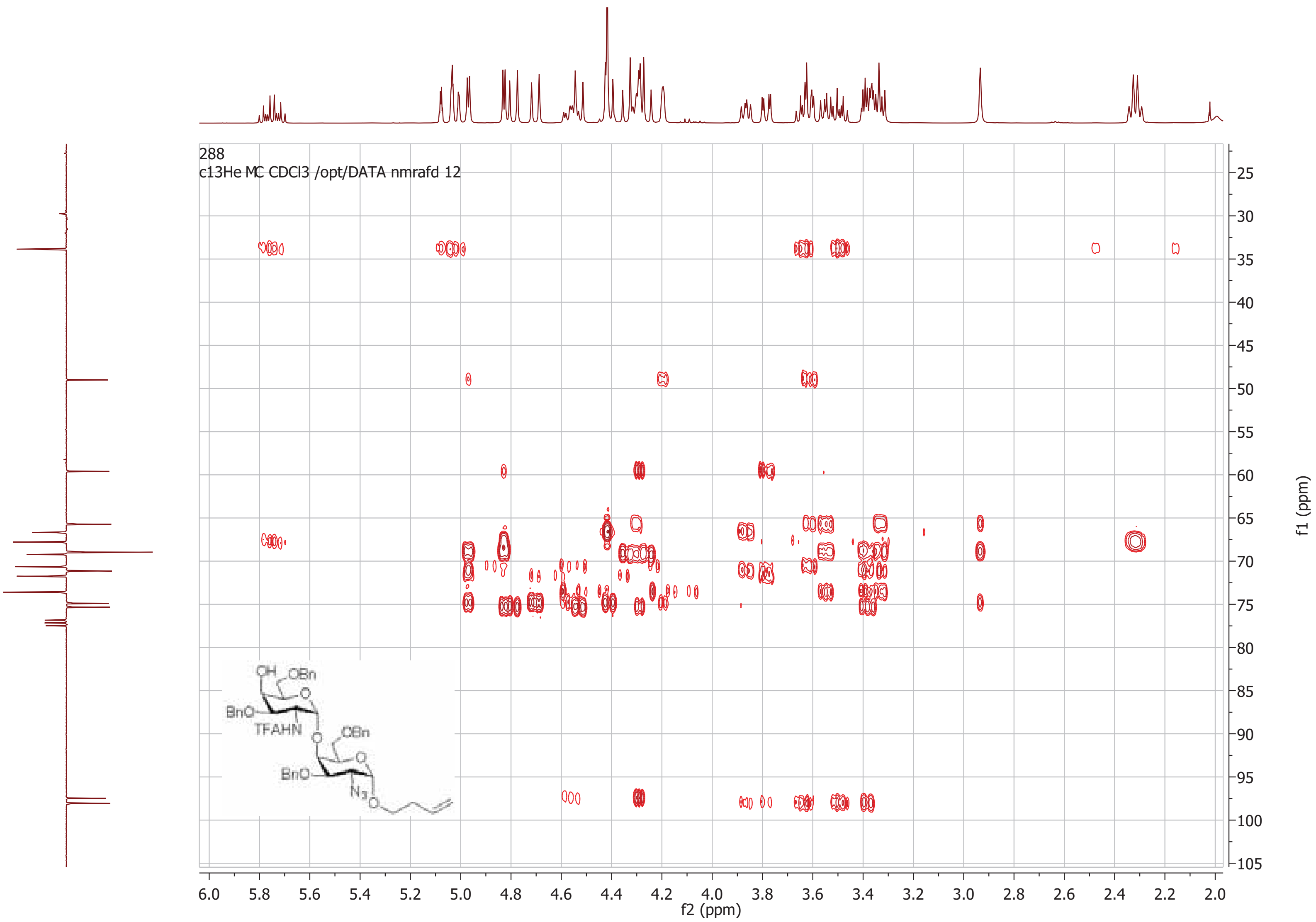
288
c13He MC CDCl3 /opt/DATA nmrafd 12
f2 (ppm)
f1 (ppm)
OH
OBn
BnO
TFAHN
O
BnO
N3

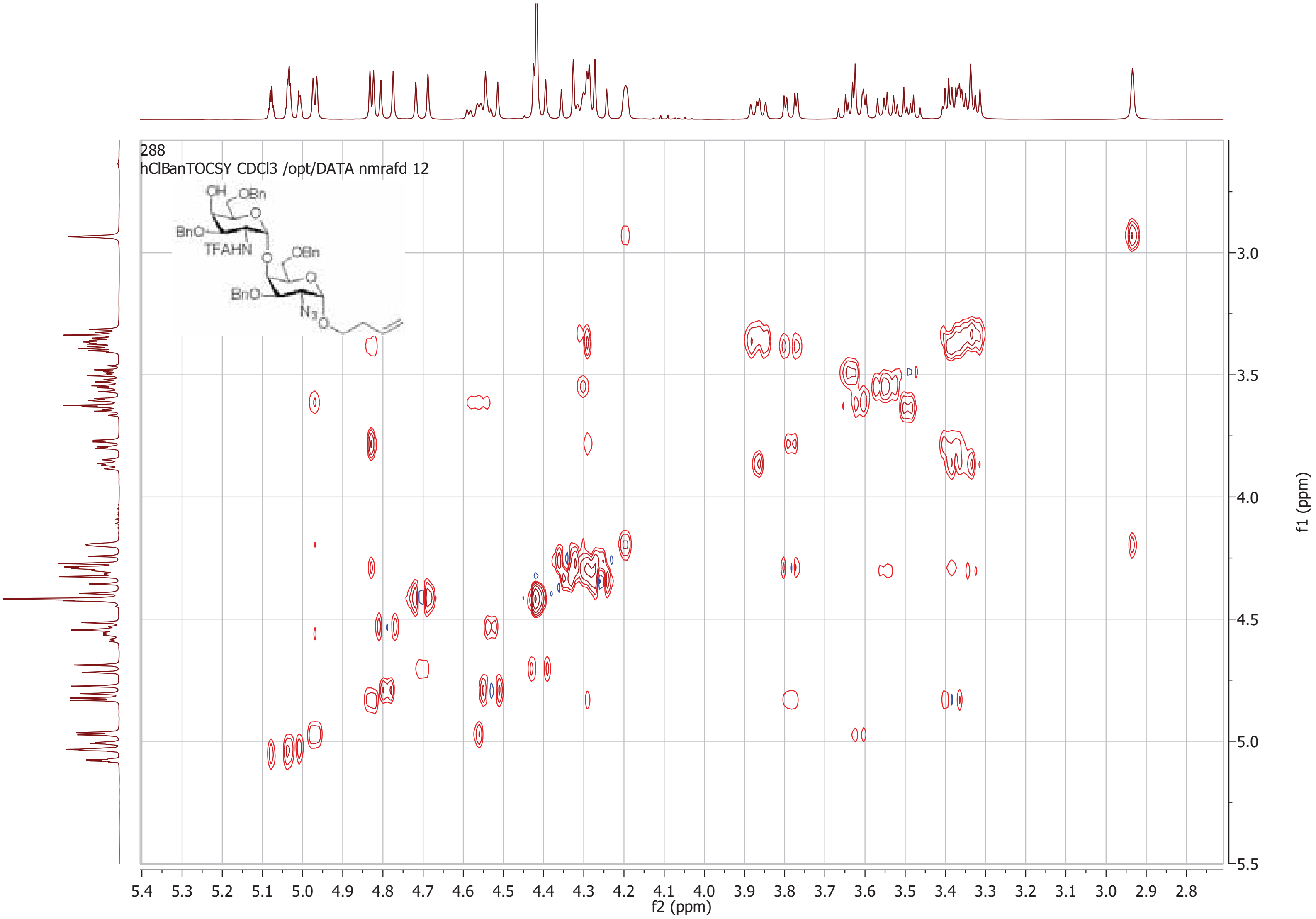

288
hClBanTOCSY CDCl3 /opt/DATA nmrafd 12
OH
OBn
BnO
TFAHN
O
OBn
BnO
N3
f2 (ppm)
f1 (ppm)
5.4 5.3 5.2 5.1 5.0 4.9 4.8 4.7 4.6 4.5 4.4 4.3 4.2 4.1 4.0 3.9 3.8 3.7 3.6 3.5 3.4 3.3 3.2 3.1 3.0 2.9 2.8
3.0 3.5 4.0 4.5 5.0 5.5

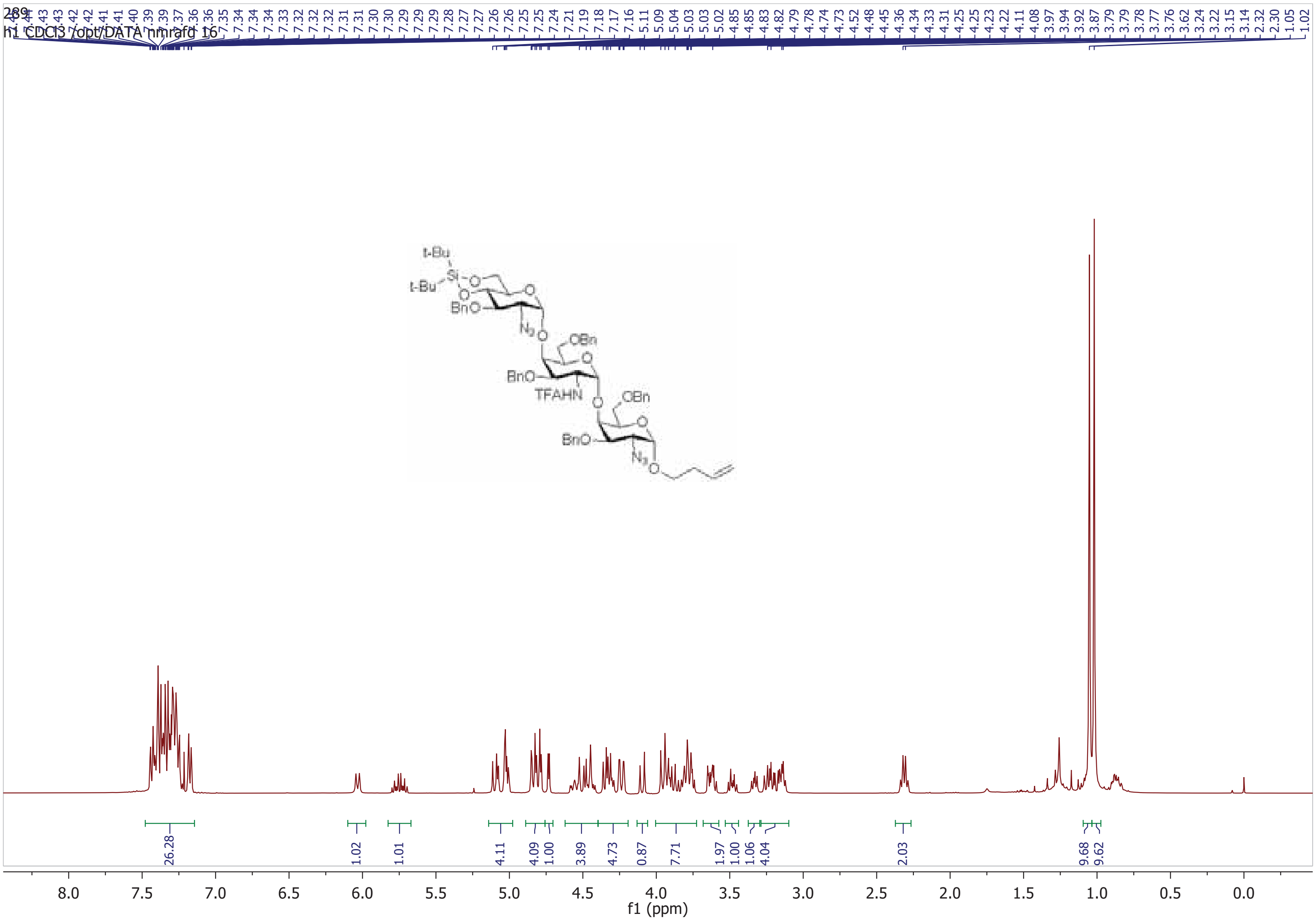

289
h1 CDCl3 /opt/DATA nmrafd 16
7.43
7.43
7.42
7.42
7.41
7.41
7.40
7.39
7.39
7.37
7.36
7.36
7.35
7.34
7.34
7.33
7.32
7.32
7.32
7.31
7.31
7.30
7.30
7.29
7.29
7.29
7.28
7.27
7.27
7.26
7.26
7.25
7.25
7.24
7.21
7.19
7.18
7.17
7.16
5.11
5.09
5.04
5.03
5.03
5.02
4.85
4.85
4.83
4.82
4.79
4.78
4.74
4.73
4.52
4.48
4.45
4.36
4.34
4.33
4.31
4.25
4.25
4.23
4.22
4.11
4.08
3.97
3.94
3.92
3.87
3.79
3.79
3.78
3.77
3.76
3.62
3.24
3.22
3.15
3.14
2.32
2.30
1.05
1.02
t-Bu
Si
t-Bu
O
O
O
BnO
N3
O
OBn
O
BnO
TFAHN
O
OBn
O
BnO
N3
O
26.28
1.02
1.01
4.11
4.09
1.00
3.89
4.73
0.87
7.71
1.97
1.00
1.06
4.04
2.03
9.68
9.62
8.0
7.5
7.0
6.5
6.0
5.5
5.0
4.5
4.0
3.5
3.0
2.5
2.0
1.5
1.0
0.5
0.0
f1 (ppm)

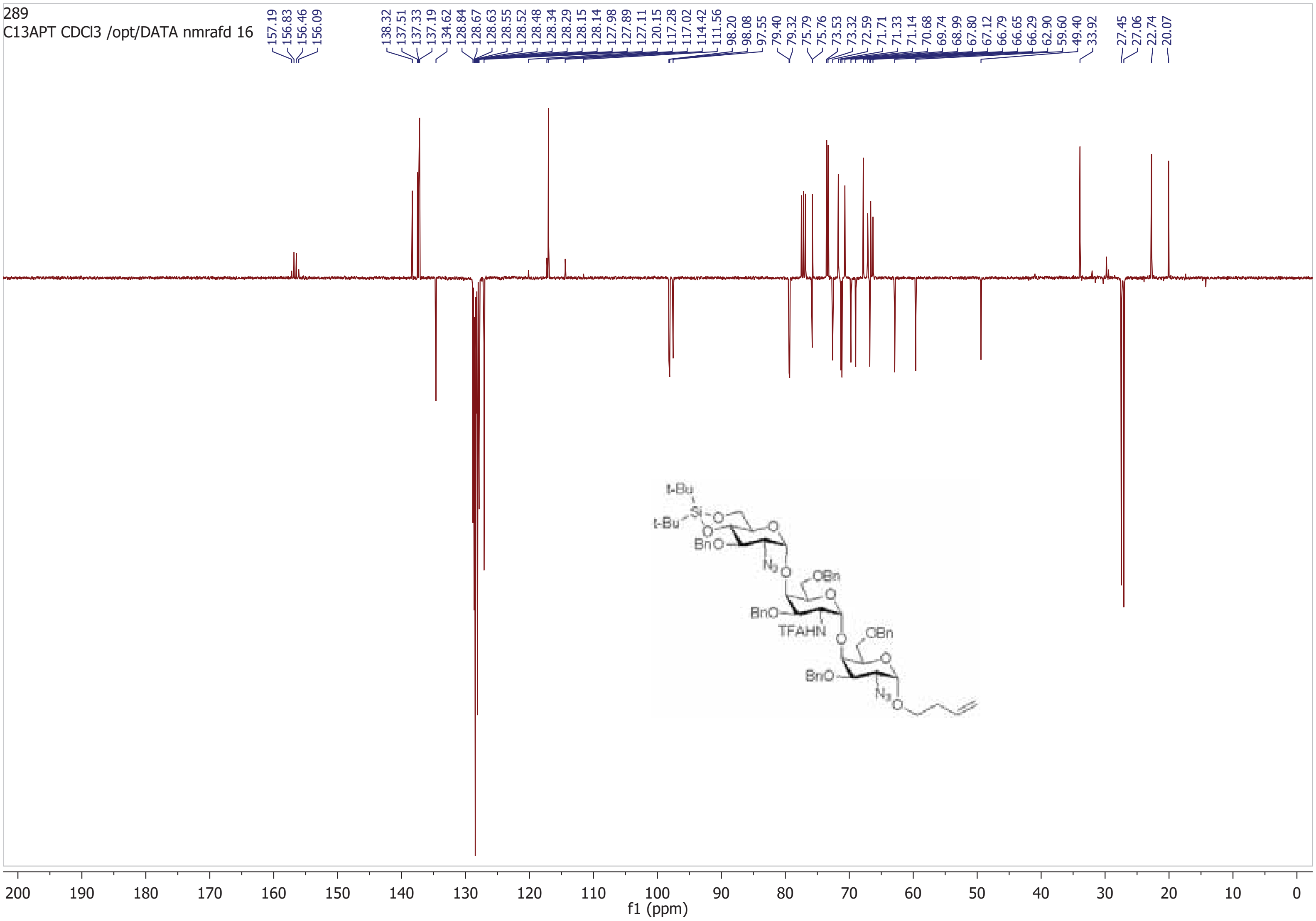

289
C13APT CDCl3 /opt/DATA nmrafd 16
157.19
156.83
156.46
156.09
138.32
137.51
137.33
137.19
134.62
128.84
128.67
128.63
128.55
128.52
128.48
128.34
128.29
128.15
128.14
127.98
127.89
127.11
120.15
117.28
117.02
114.42
111.56
98.20
98.08
97.55
79.40
79.32
75.79
75.76
73.53
73.32
72.59
71.71
71.33
71.14
70.68
69.74
68.99
67.80
67.12
66.79
66.65
66.29
62.90
59.60
49.40
33.92
27.45
27.06
22.74
20.07
t-Bu
Si
t-Bu
BnO
N3
OBn
BnO
TFAHN
OBn
BnO
N3
200
190
180
170
160
150
140
130
120
110
100
90
80
70
60
50
40
30
20
10
0
f1 (ppm)

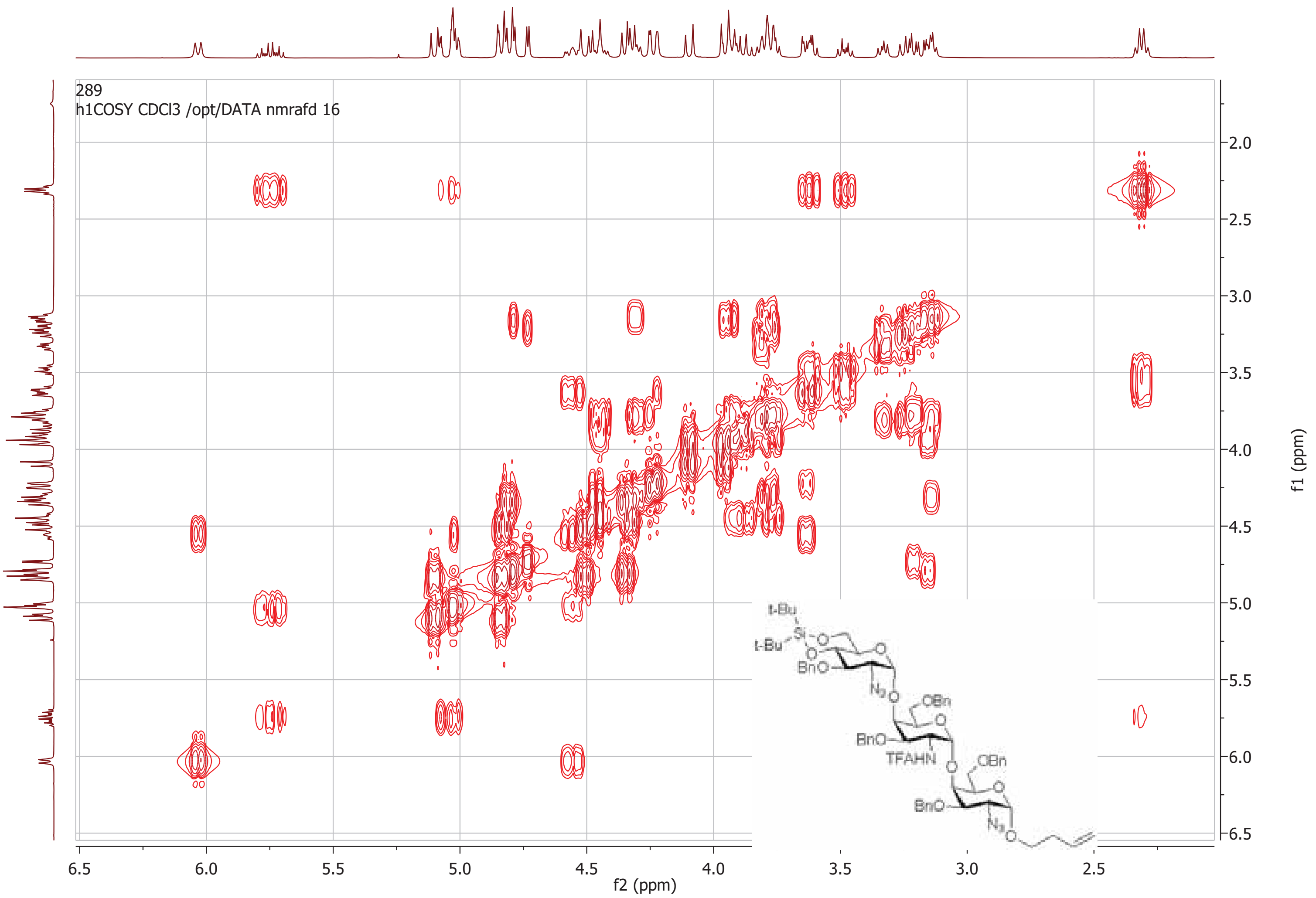
289
h1COSY CDCl3 /opt/DATA nmrafd 16
f2 (ppm)
f1 (ppm)
t-Bu
t-Bu
Si
BnO
N3
OBn
BnO
TFAHN
OBn
BnO
N3

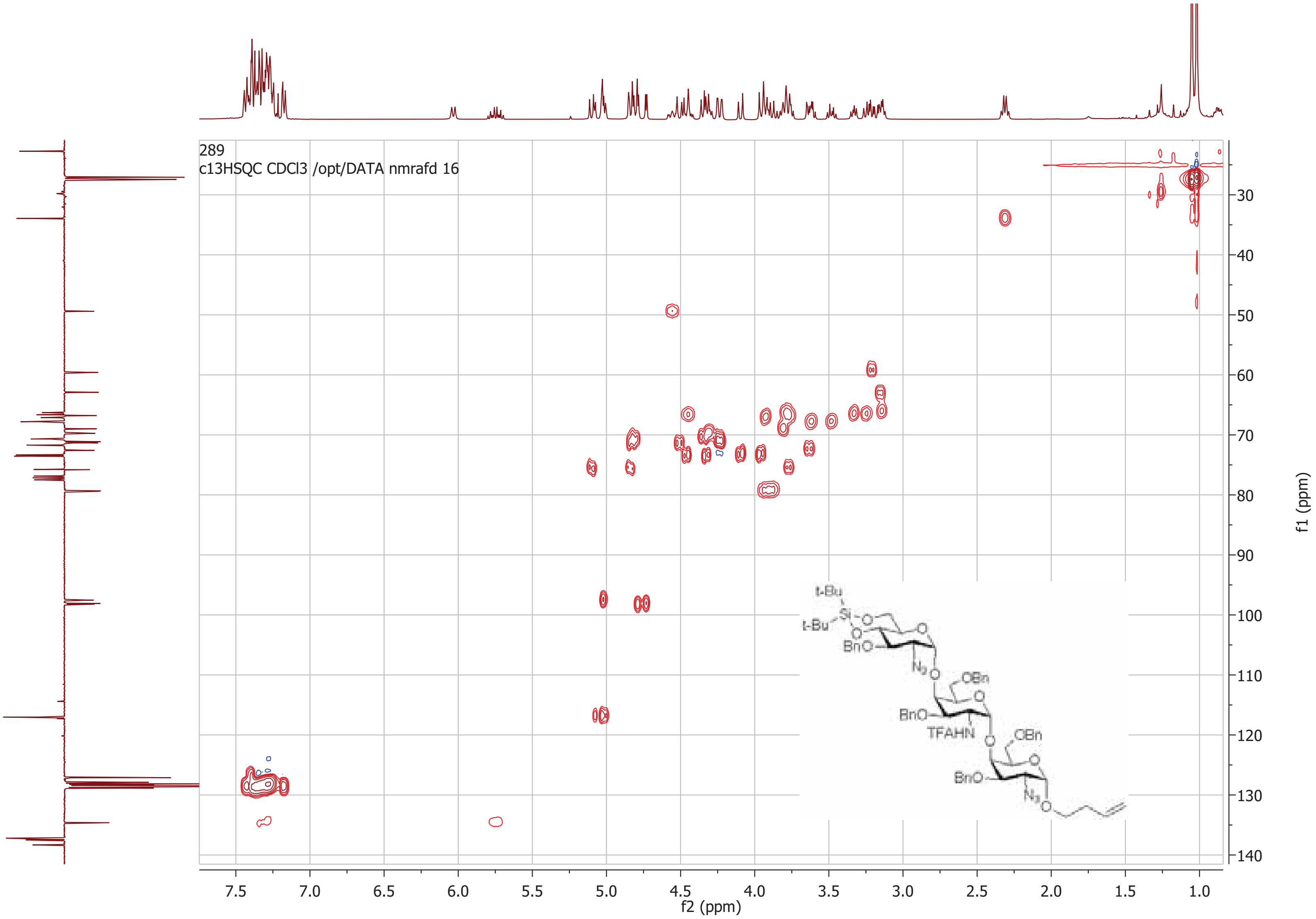

289
c13HSQC CDCl3 /opt/DATA nmrafd 16
f2 (ppm)
f1 (ppm)
t-Bu
Si
t-Bu
BnO
N3
OBn
BnO
TFAHN
OBn
BnO
N3

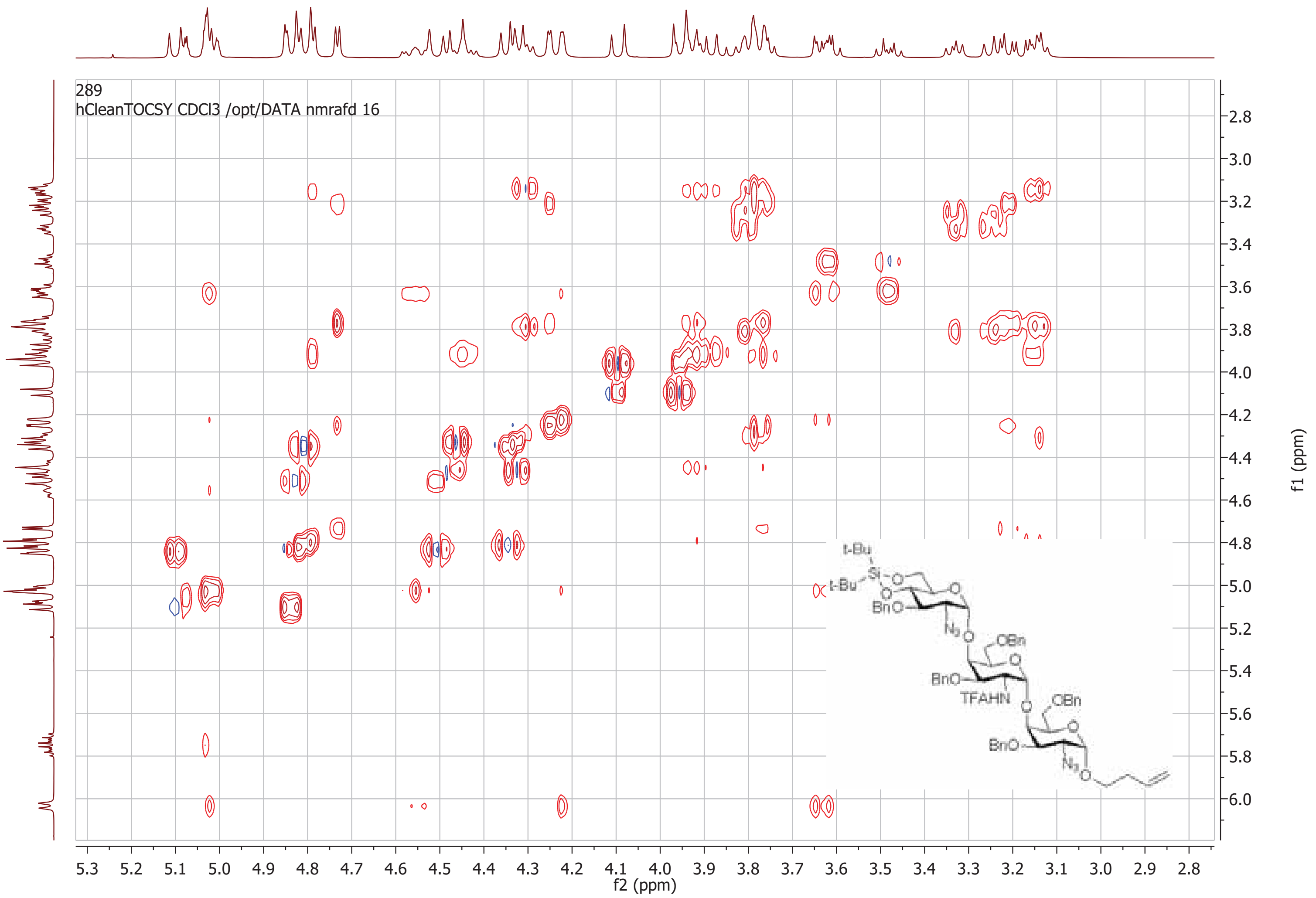
289
hCleanTOCSY CDCl3 /opt/DATA nmrafd 16
f2 (ppm)
f1 (ppm)
t-Bu
Si
t-Bu
BnO
N3
OBn
BnO
TFAHN
OBn
BnO
N3

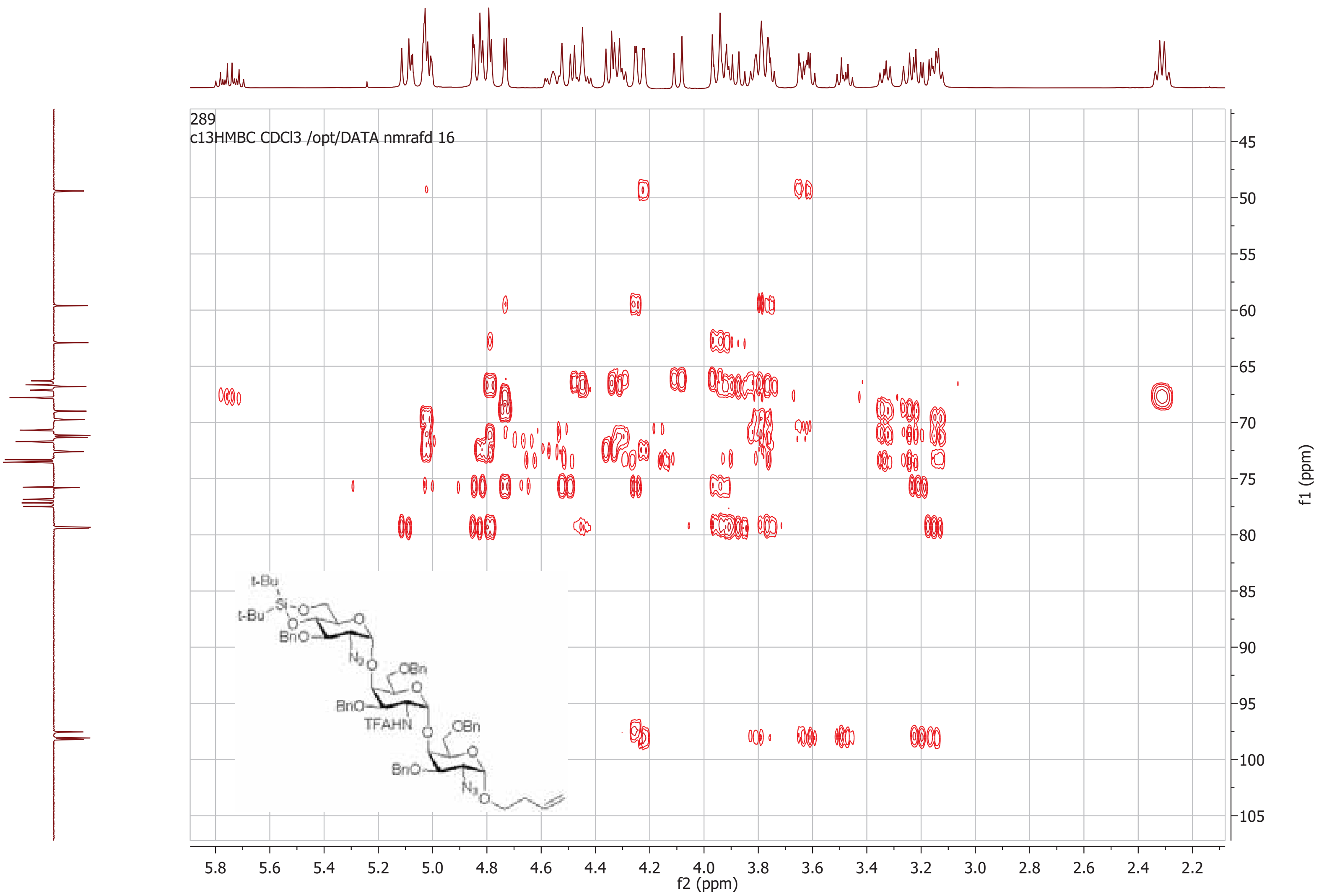
289
c13HMBC CDCl3 /opt/DATA nmrafd 16
f2 (ppm)
f1 (ppm)
t-Bu
t-Bu
Si
BnO
N3
OBn
BnO
TFAHN
OBn
BnO
N3

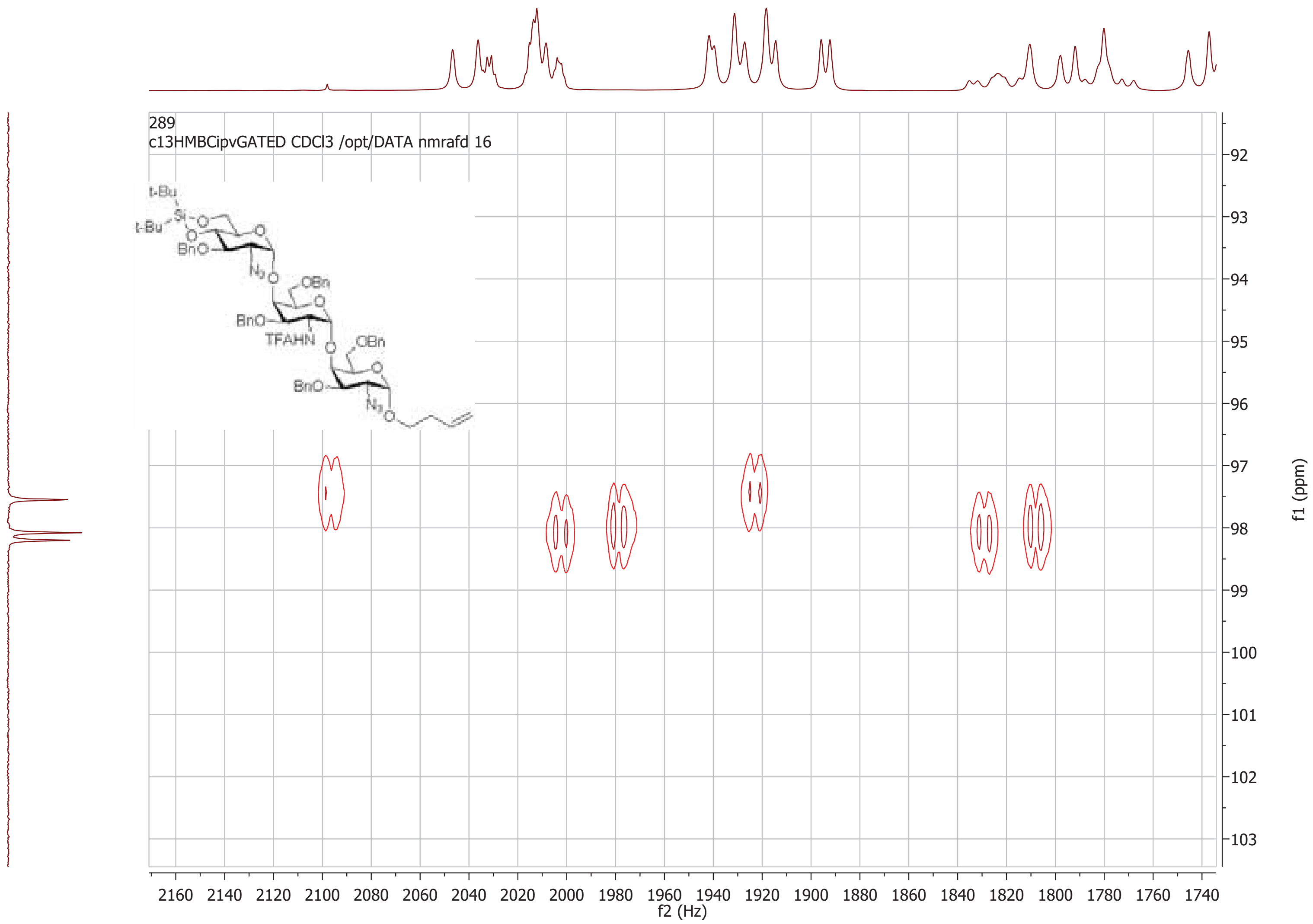
289
c13HMBCipvGATED CDCl3 /opt/DATA nmrafd 16
t-Bu
Si
t-Bu
BnO
N_3
OBn
BnO
TFAHN
OBn
BnO
N_3
f2 (Hz)
f1 (ppm)

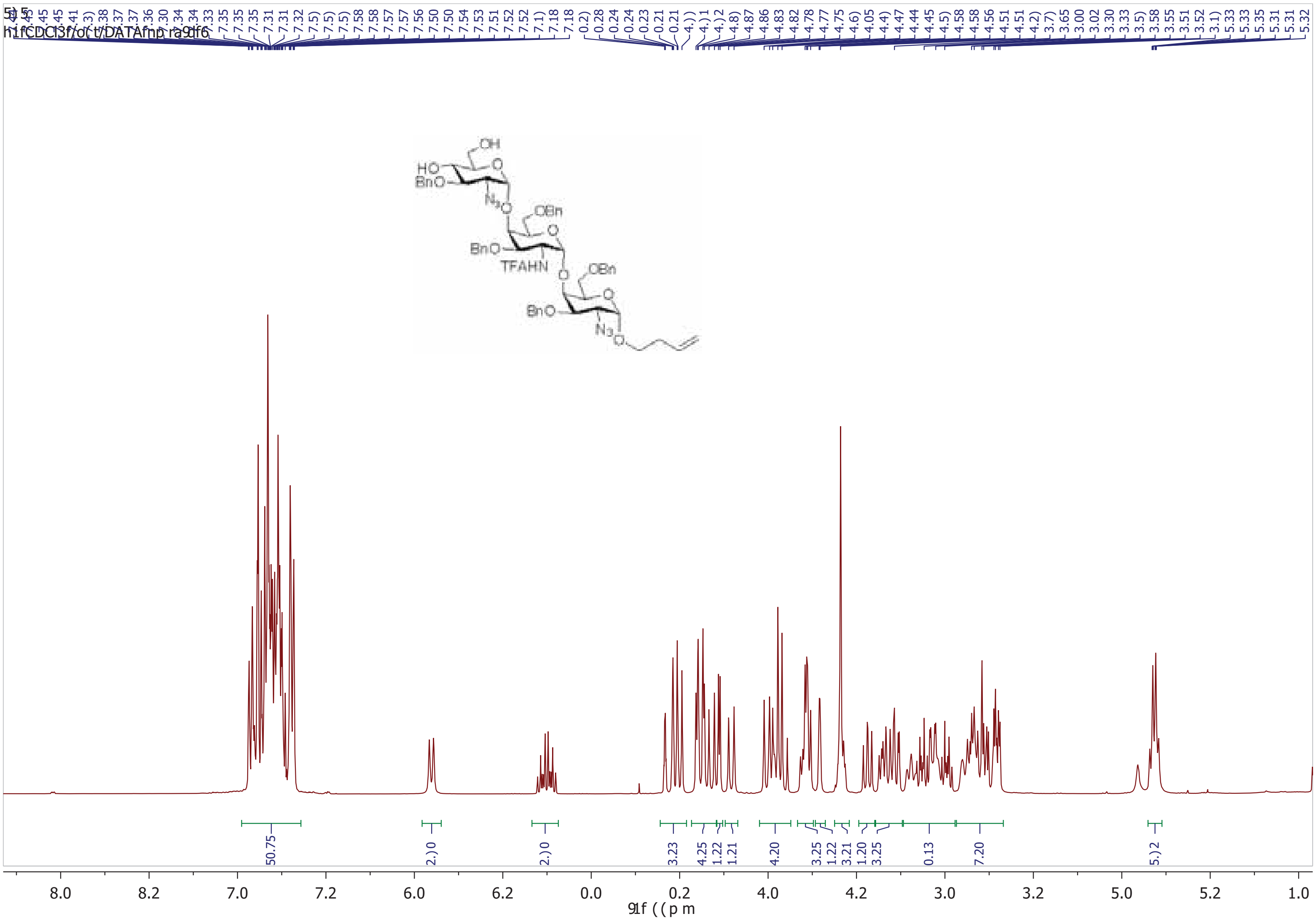
OH
HO
BnO
N3
OBn
BnO
TFAHN
OBn
BnO
N3

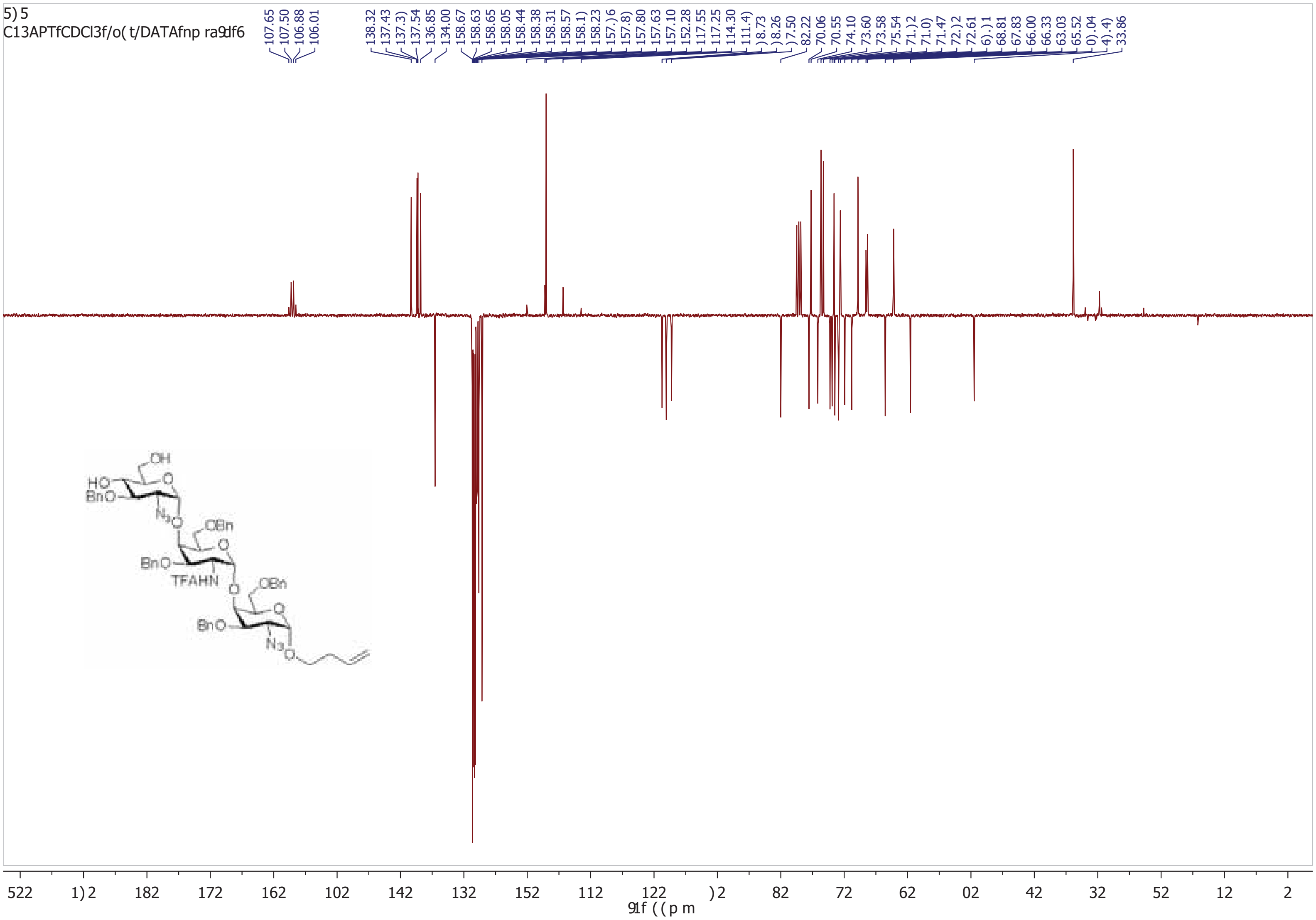
5) 5
C13APTfCDCl3f/o(t/DATAfnp ra9df6
107.65
107.50
106.88
106.01
138.32
137.43
137.3)
137.54
136.85
134.00
158.67
158.63
158.65
158.05
158.44
158.38
158.31
158.57
158.1)
158.23
157.)6
157.8)
157.80
157.63
157.10
152.28
117.55
117.25
114.30
111.4)
)8.73
)8.26
)7.50
82.22
70.06
70.55
74.10
73.60
73.58
75.54
71.)2
71.0)
71.47
72.)2
72.61
6).)1
68.81
67.83
66.00
66.33
63.03
65.52
0).04
4).4)
33.86
HO
BnO
OH
N3
OBn
BnO
TFAHN
OBn
BnO
N3
522
1)2
182
172
162
102
142
132
152
112
122
)2
82
72
62
02
42
32
52
12
2
9f1 ((p m

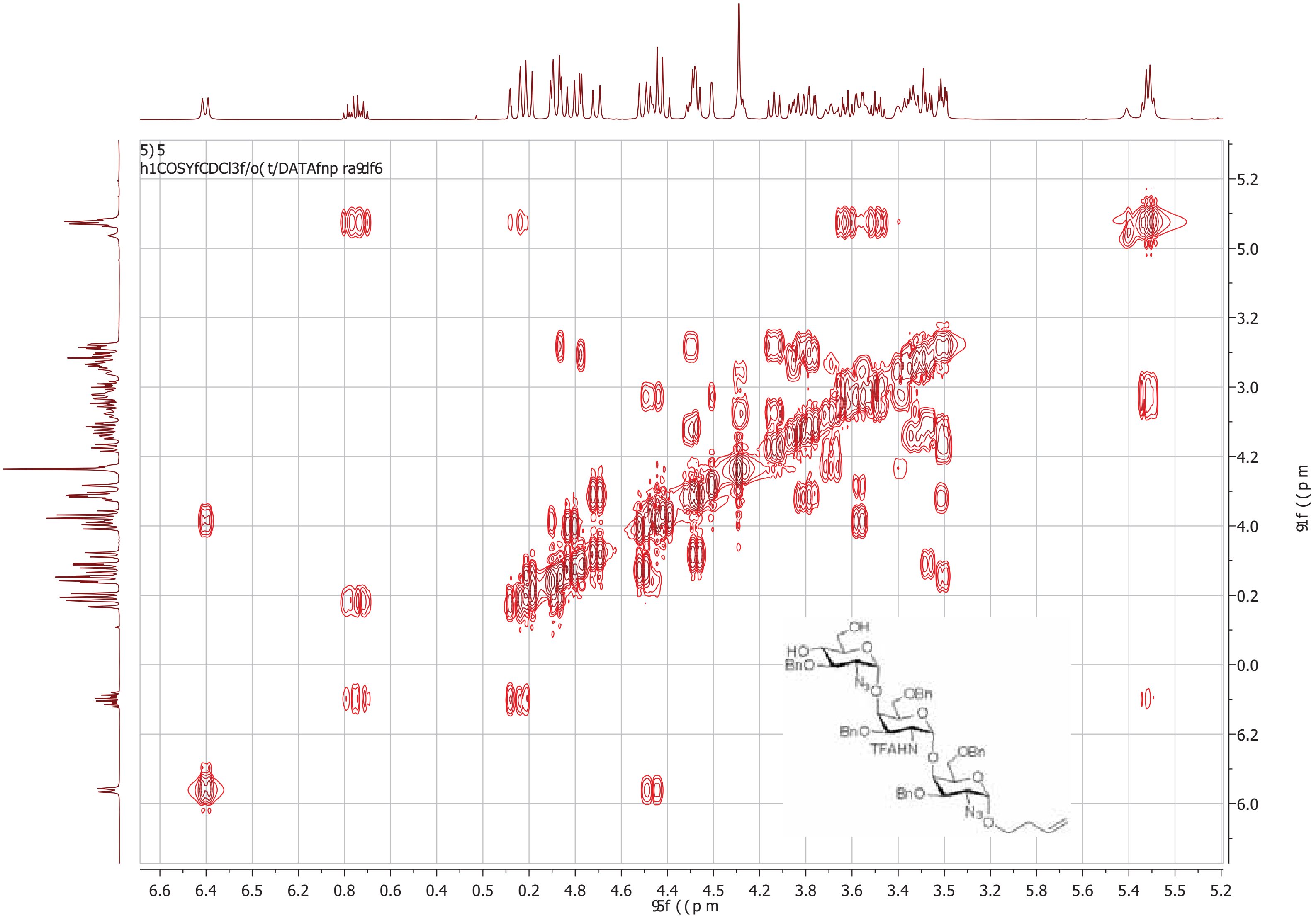
5) 5
h1COSYfCDCl3f/o(t/DATAfnp ra9df6
9f ((p m
91f ((p m
HO
BnO
OH
OBn
BnO
TFAHN
OBn
BnO

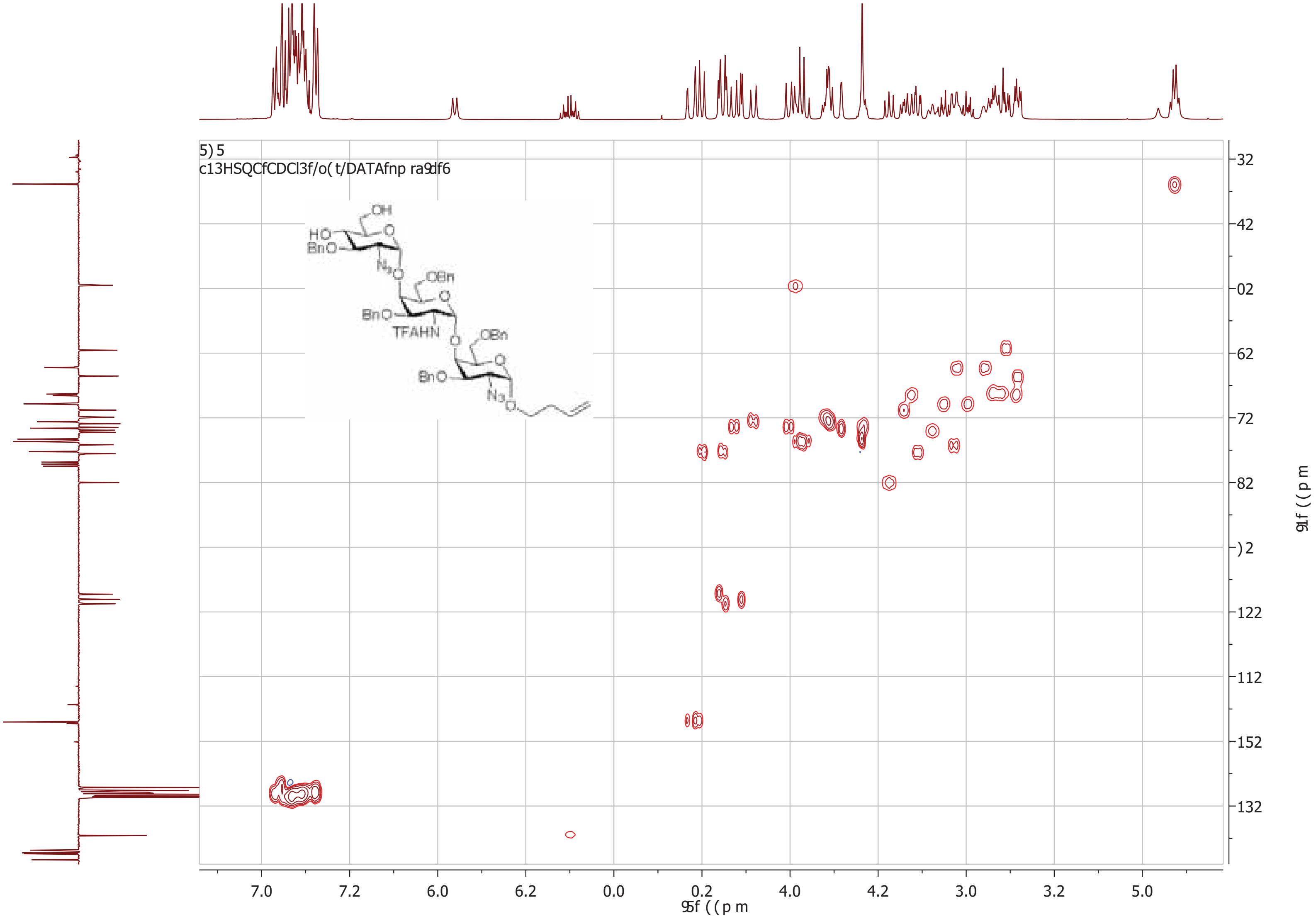
5) 5
c13HSQCfCDCl3f/o(t/DATAfnp ra9df6
OH
HO
BnO
N3
OBn
BnO
TFAHN
OBn
BnO
N3
O

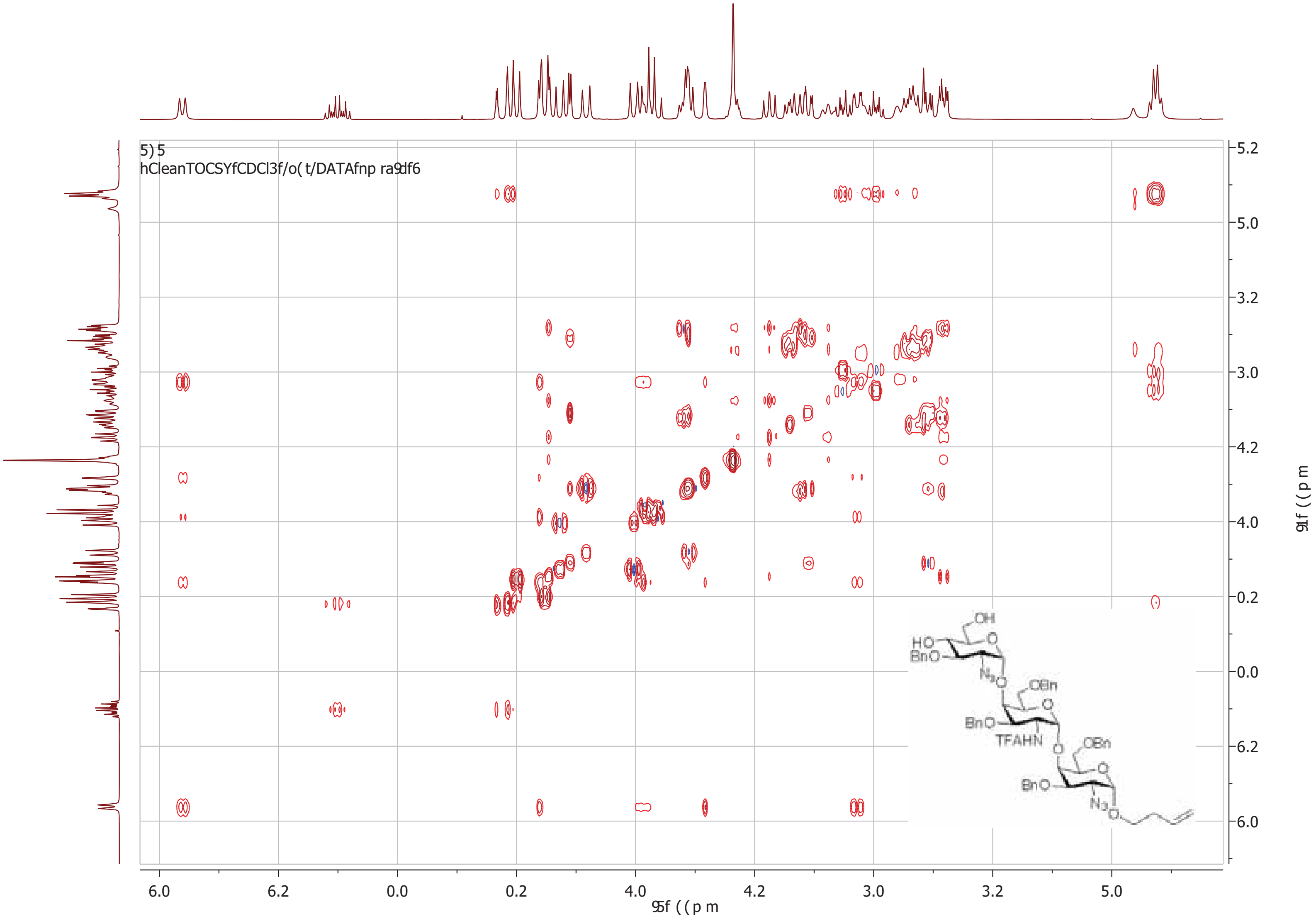

5) 5
hCleanTOCSYfCDCl3f/o(t/DATAfnp ra9df6
9f1 ((p m
95f ((p m
OH
HO
BnO
N3
OBn
BnO
TFAHN
OBn
BnO
N3

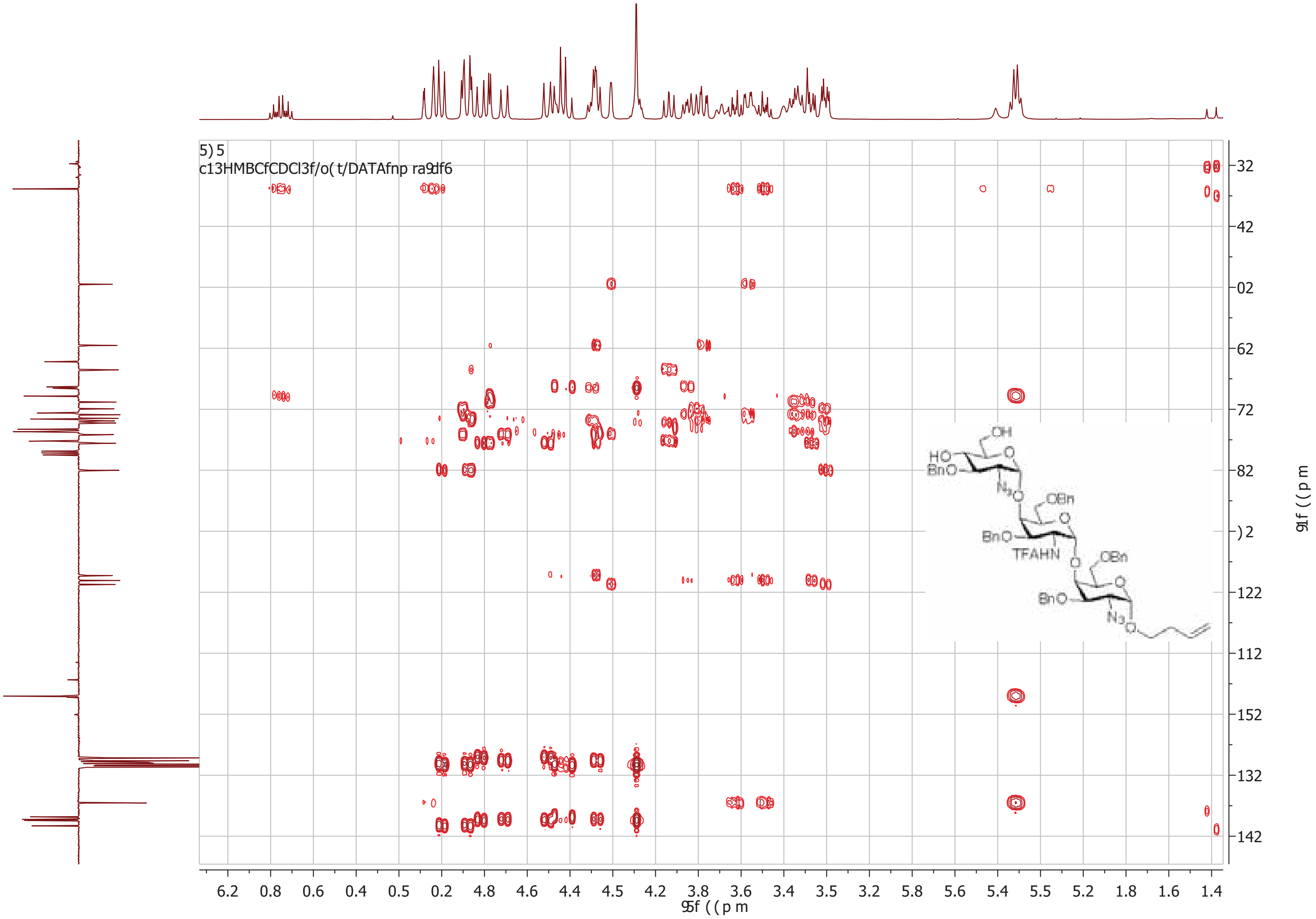
5) 5
c13HMBCfCDCl3f/o(t/DATAfnp ra9df6
OH
HO
BnO
N3
OBn
BnO
TFAHN
OBn
BnO
N3

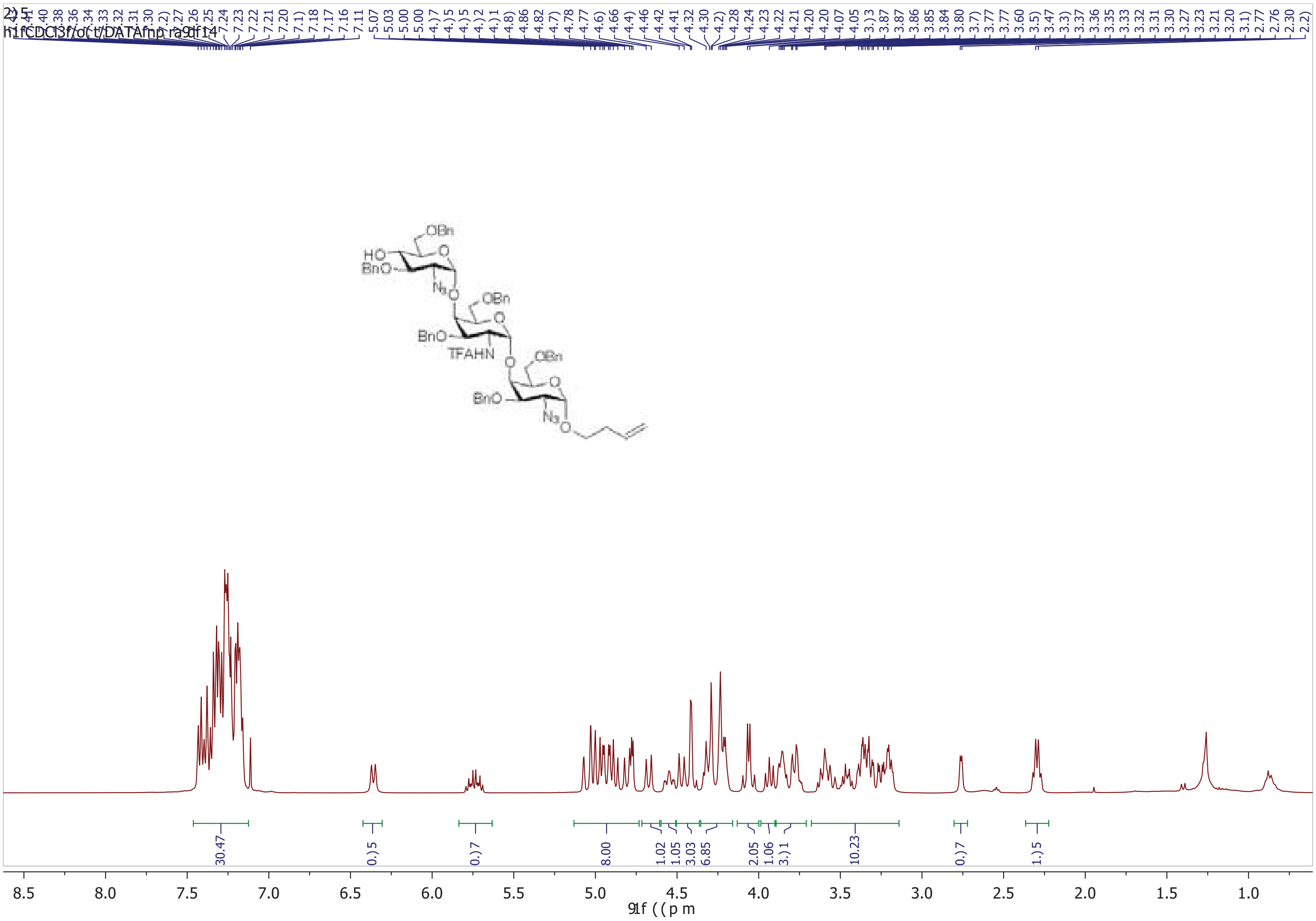

OBn
HO
BnO
N3
OBn
BnO
TFAHN
OBn
BnO
N3
30.47
8.00
1.02
1.05
3.03
6.85
2.05
1.06
10.23
8.5
8.0
7.5
7.0
6.5
6.0
5.5
5.0
4.5
4.0
3.5
3.0
2.5
2.0
1.5
1.0

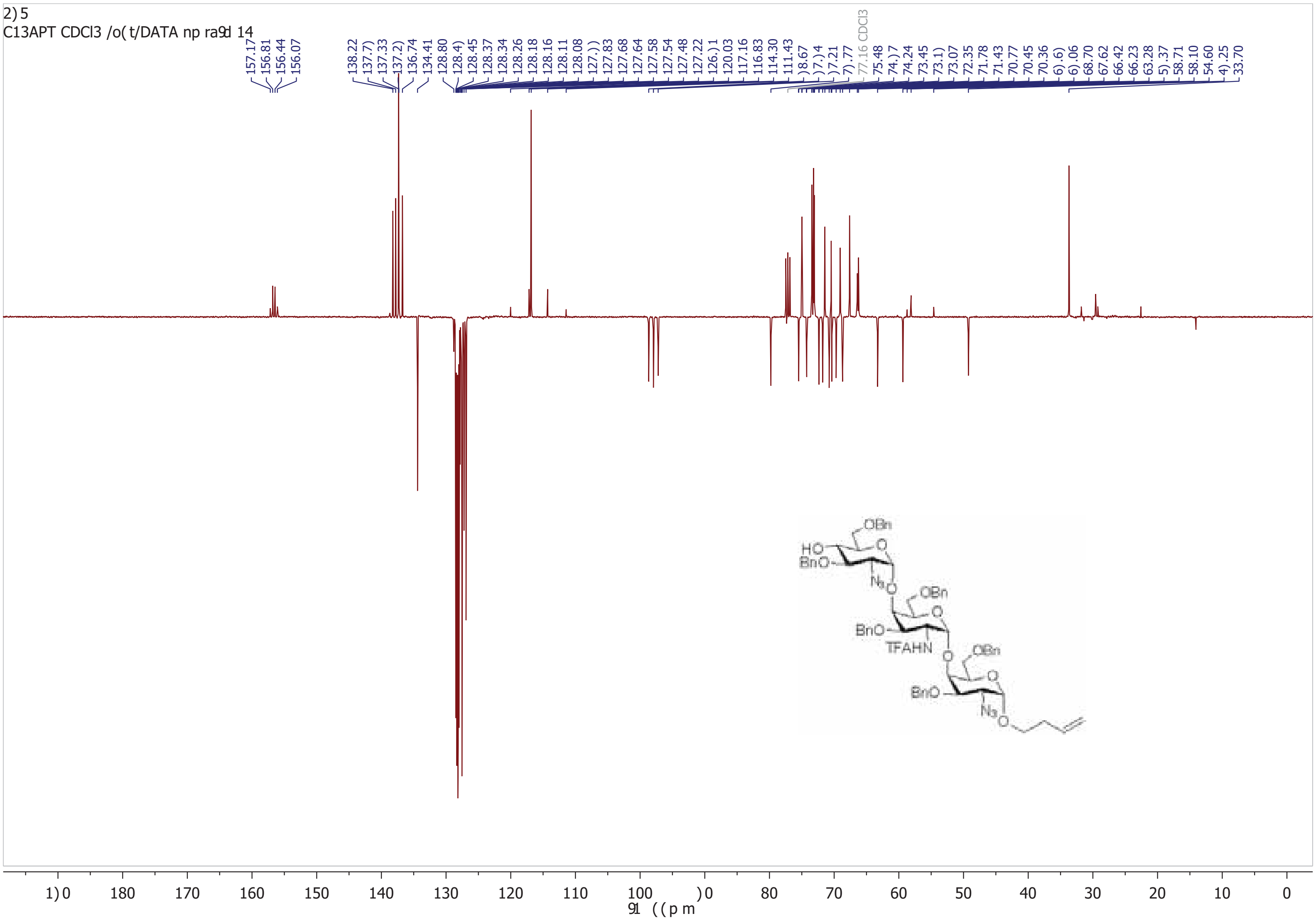
2)5
C13APT CDCl3 /o(t/DATA np ra9d 14
157.17
156.81
156.44
156.07
138.22
137.7)
137.33
137.2)
136.74
134.41
128.80
128.4)
128.45
128.37
128.34
128.26
128.18
128.16
128.11
128.08
127.))
127.83
127.68
127.64
127.58
127.54
127.48
127.22
126.)1
120.03
117.16
116.83
114.30
111.43
)8.67
)7.)4
)7.21
7).77
77.16 CDCl3
75.48
74.)7
74.24
73.45
73.1)
73.07
72.35
71.78
71.43
70.77
70.45
70.36
6).6)
6).06
68.70
67.62
66.42
66.23
63.28
5).37
58.71
58.10
54.60
4).25
33.70
1)0
180
170
160
150
140
130
120
110
100
)0
80
70
60
50
40
30
20
10
0
91 ((p m
OBn
HO
O
BnO
N3
O
OBn
O
BnO
TFAHN
O
OBn
O
BnO
N3
O

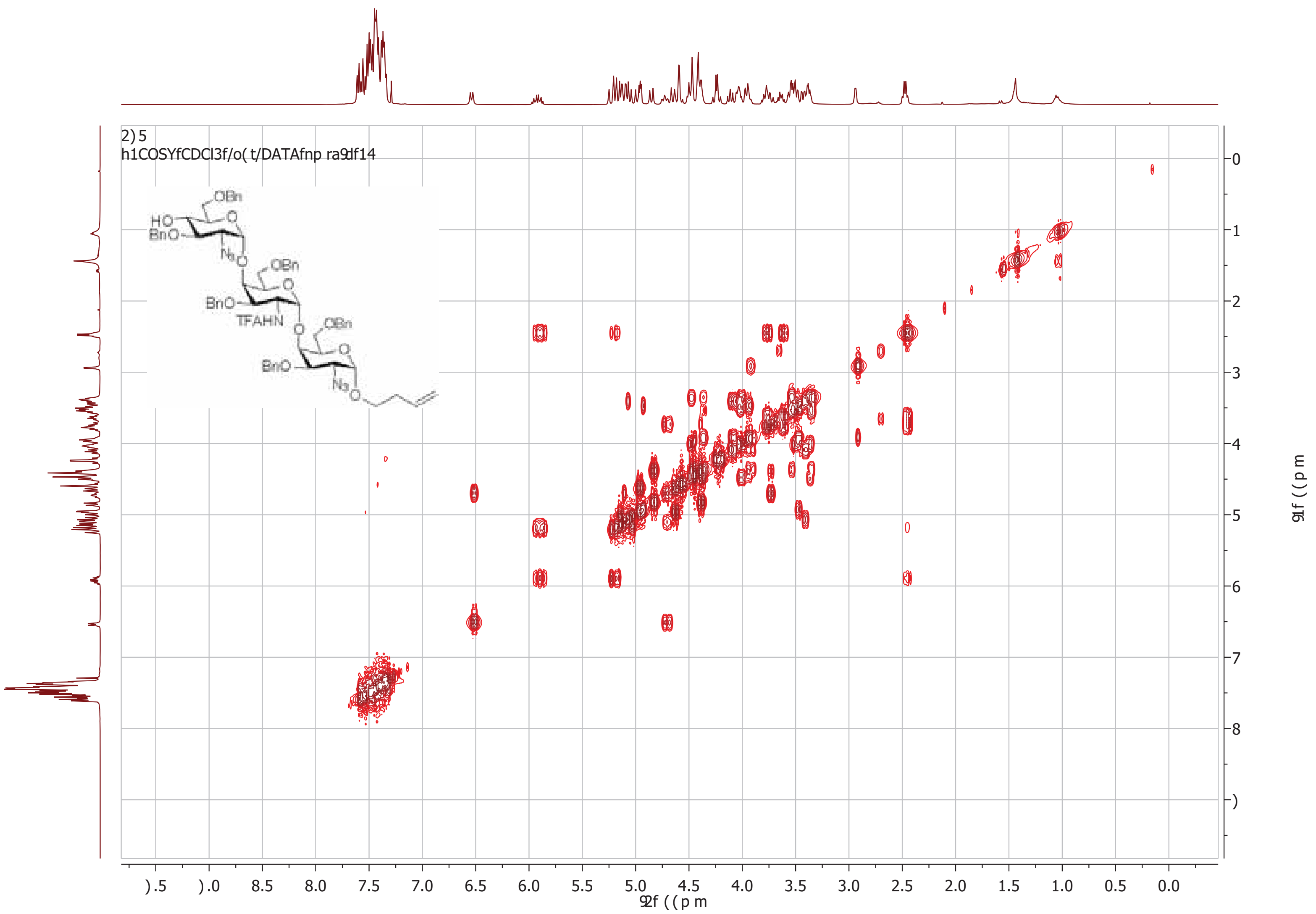

2) 5
h1COSYfCDCl3f/o(t/DATAfnp ra9df14
OBn
HO
BnO
O
N3
O
OBn
O
BnO
TFAHN
O
OBn
O
BnO
N3
O
).5
).0
8.5
8.0
7.5
7.0
6.5
6.0
5.5
5.0
4.5
4.0
3.5
3.0
2.5
2.0
1.5
1.0
0.5
0.0
92f ((p m
0
1
2
3
4
5
6
7
8
)
91f ((p m

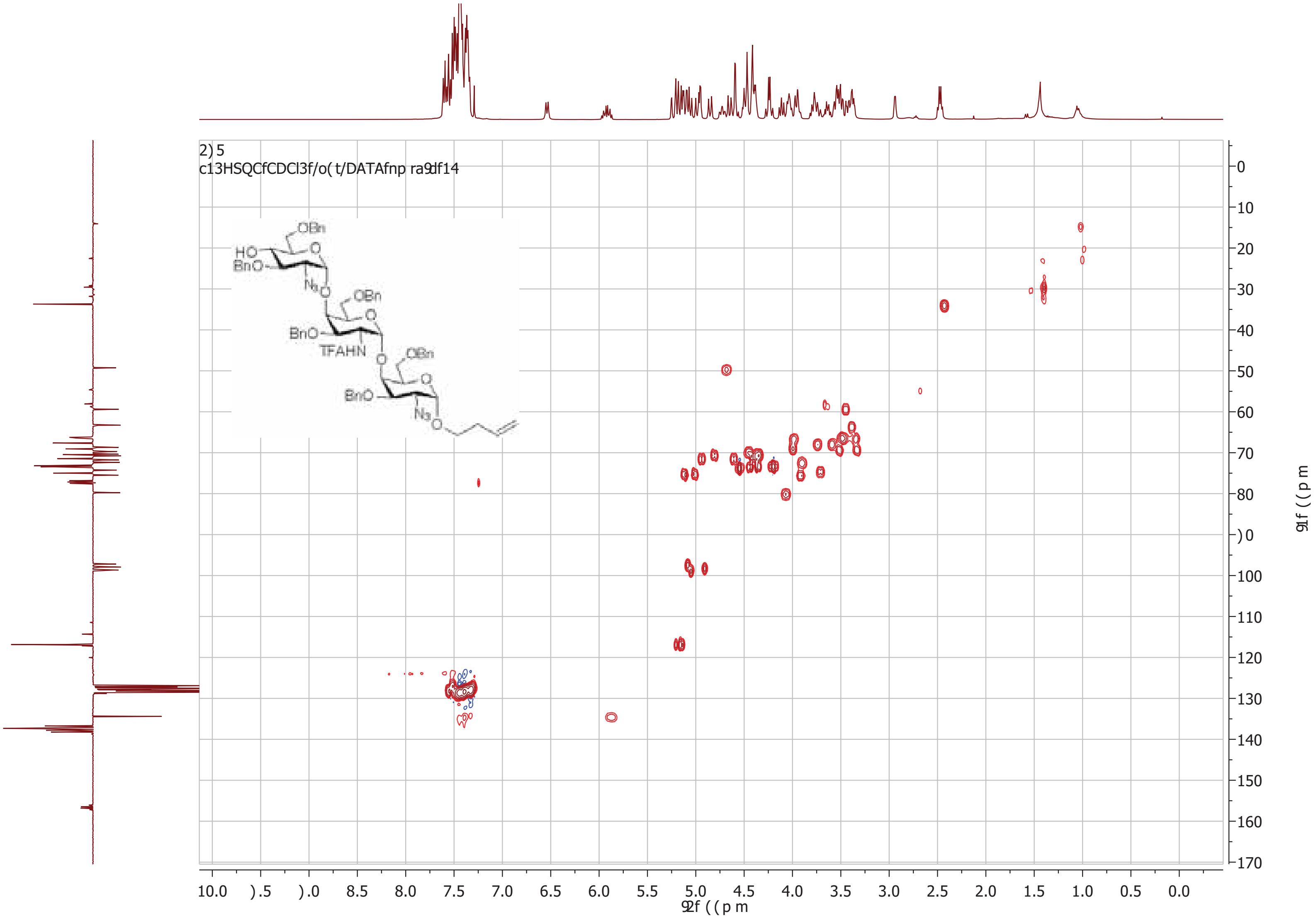
2)5
c13HSQCfCDCl3f/o(t/DATAfnp ra9df14
OBn
HO
BnO
N3
OBn
BnO
TFAHN
OBn
BnO
N3
9f2 ((p m
9f1 ((p m

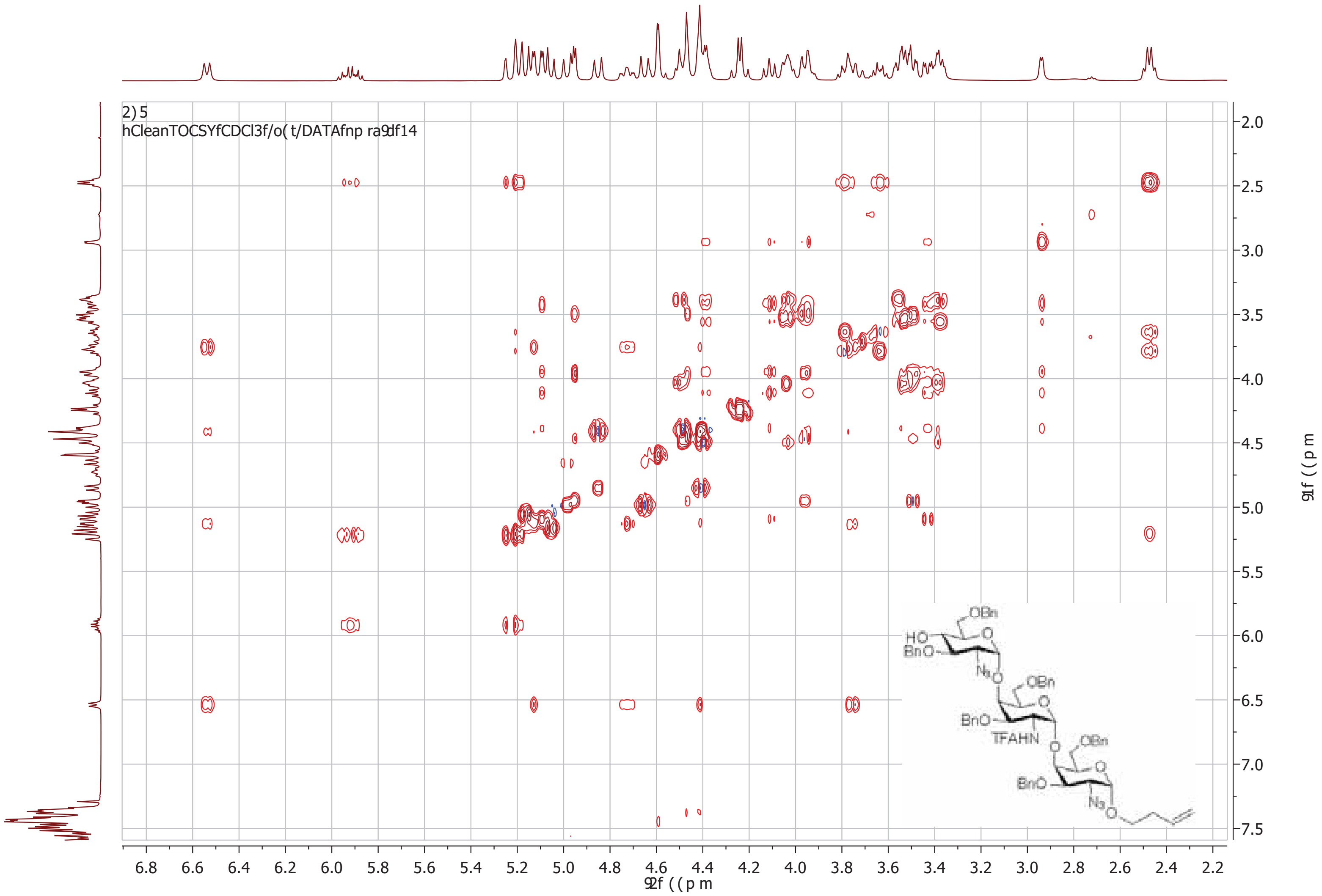
2) 5
hCleanTOCSYfCDCl3f/o(t/DATAfnp ra9df14
9f1 ((p m
92f ((p m
OBn
HO
BnO
N3
OBn
BnO
TFAHN
OBn
BnO
N3

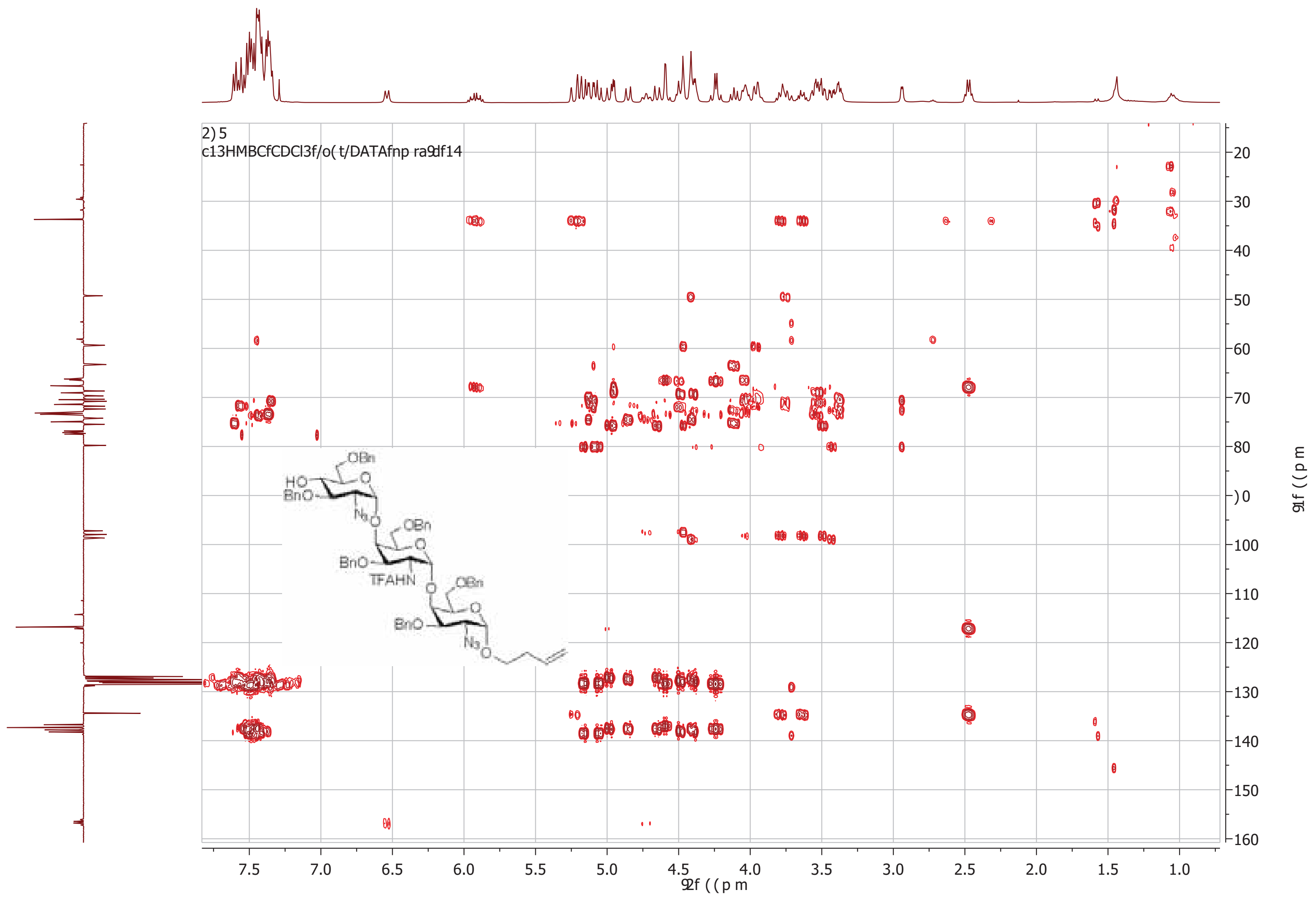
2) 5
c13HMBCfCDCl3f/o(t/DATAfnp ra9df14
OBn
HO
BnO
N3
OBn
BnO
TFAHN
OBn
BnO
N3
7.5 7.0 6.5 6.0 5.5 5.0 4.5 4.0 3.5 3.0 2.5 2.0 1.5 1.0
9f2 ((p m
20 30 40 50 60 70 80)0 100 110 120 130 140 150 160
9f1 ((p m

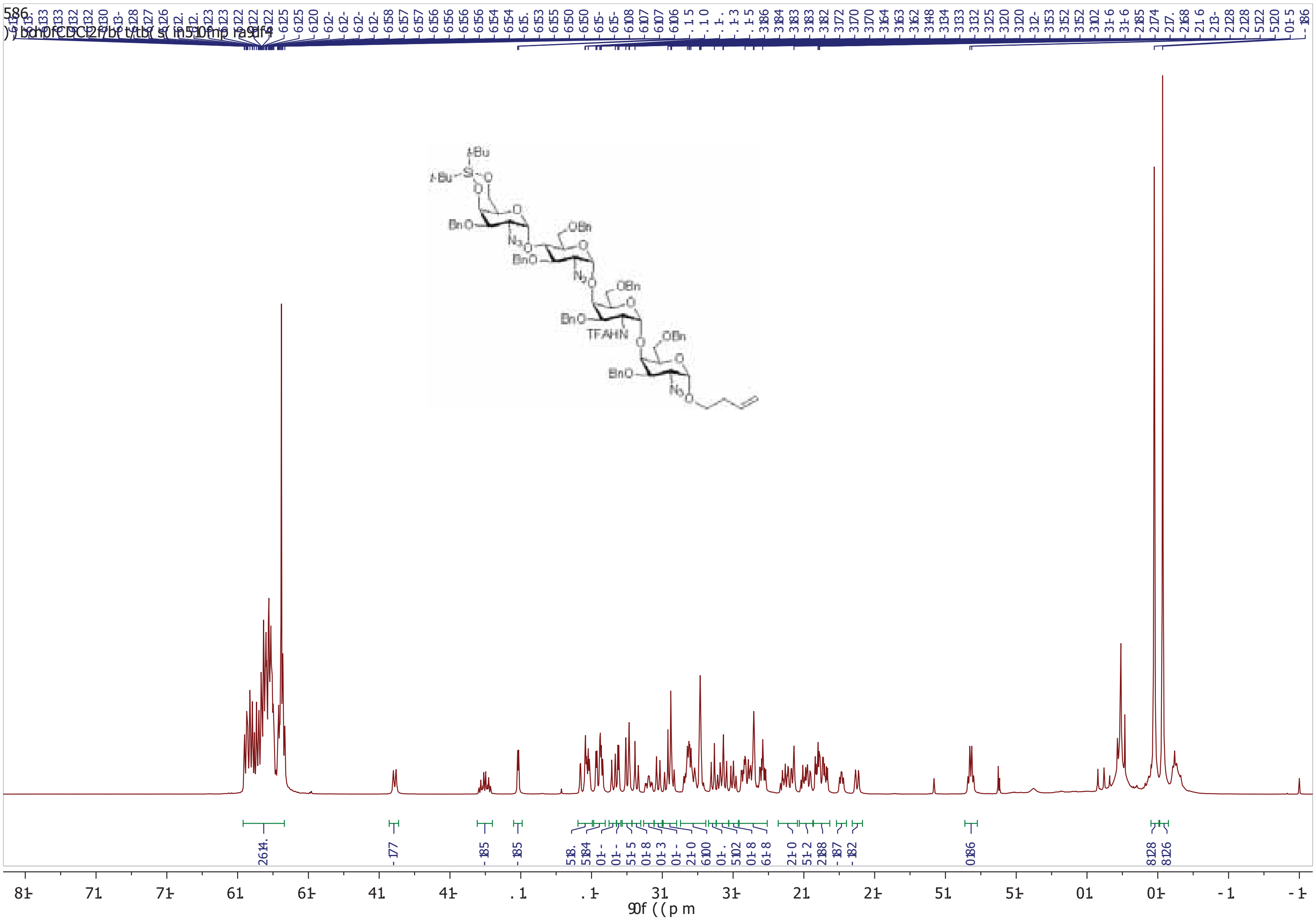
t-Bu
t-Bu
Si
BnO
N3
OBn
BnO
N3
OBn
BnO
TFAHN
OBn
BnO
N3

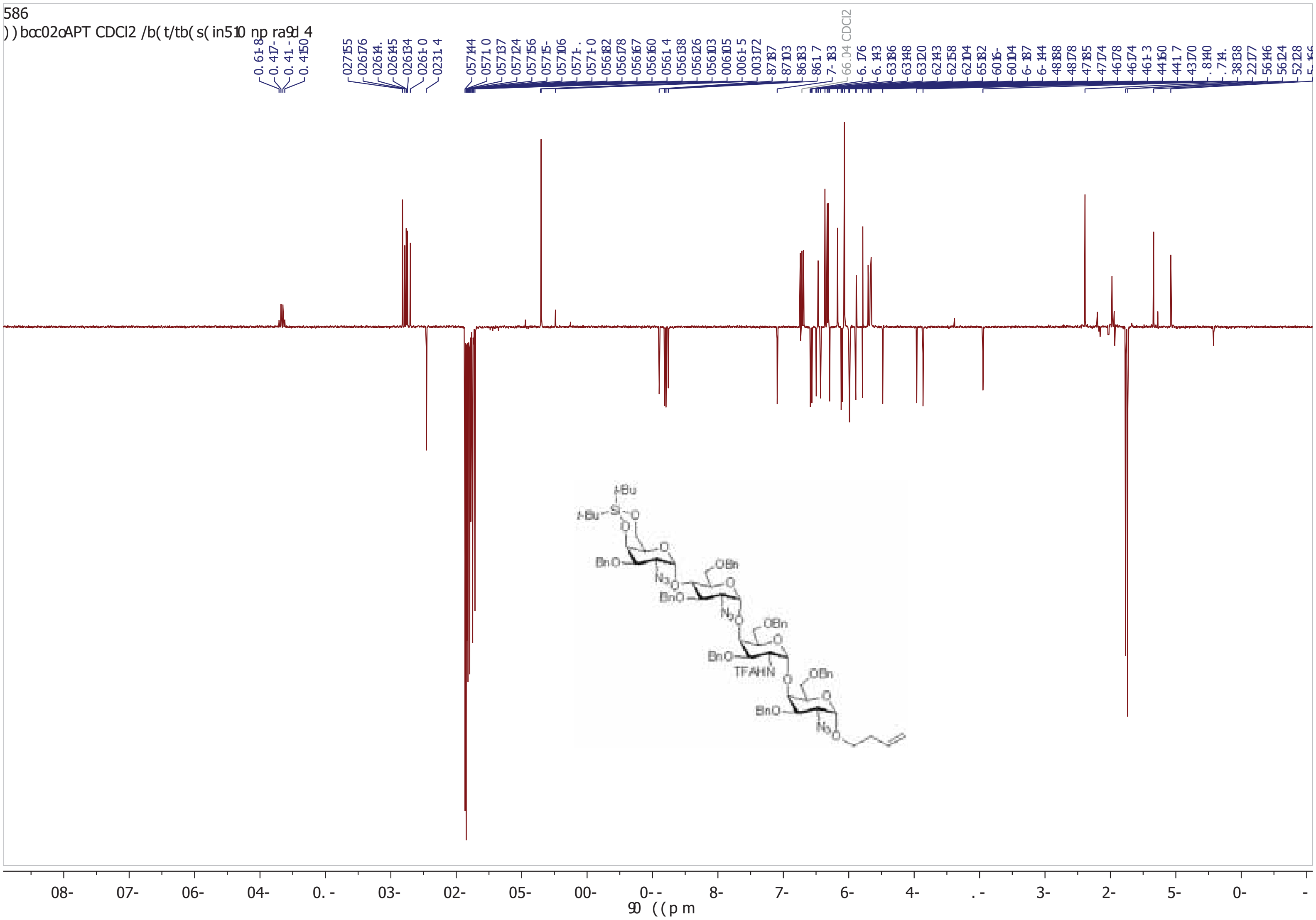
586
CDCl2
t-Bu
t-Bu
Si
BnO
N3
OBn
TFAHN

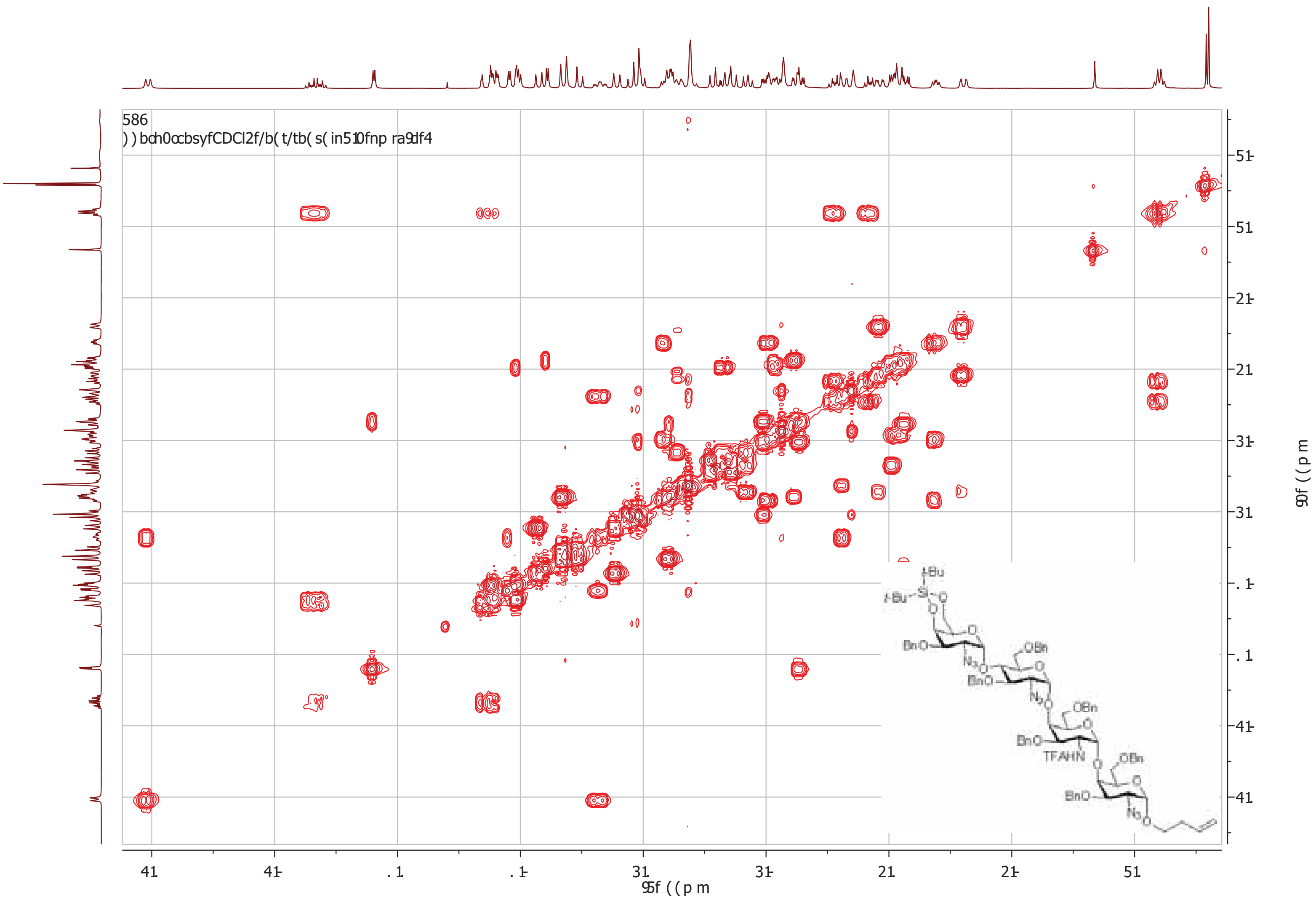
t-Bu
t-Bu
Si
BnO
N3
OBn
TFAHN

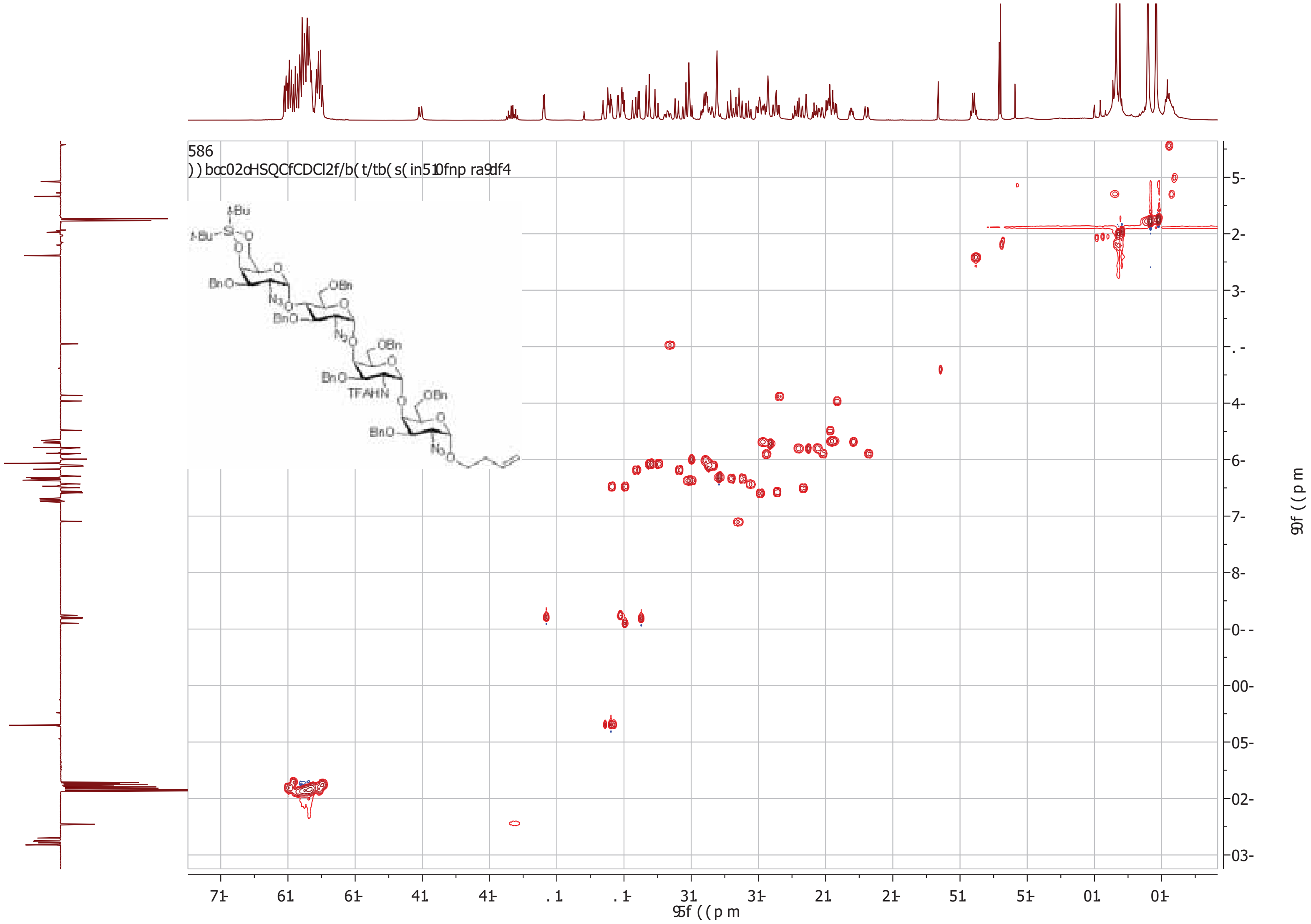

586
t-Bu
t-Bu
Si
BnO
N3
OBn
TFAHN

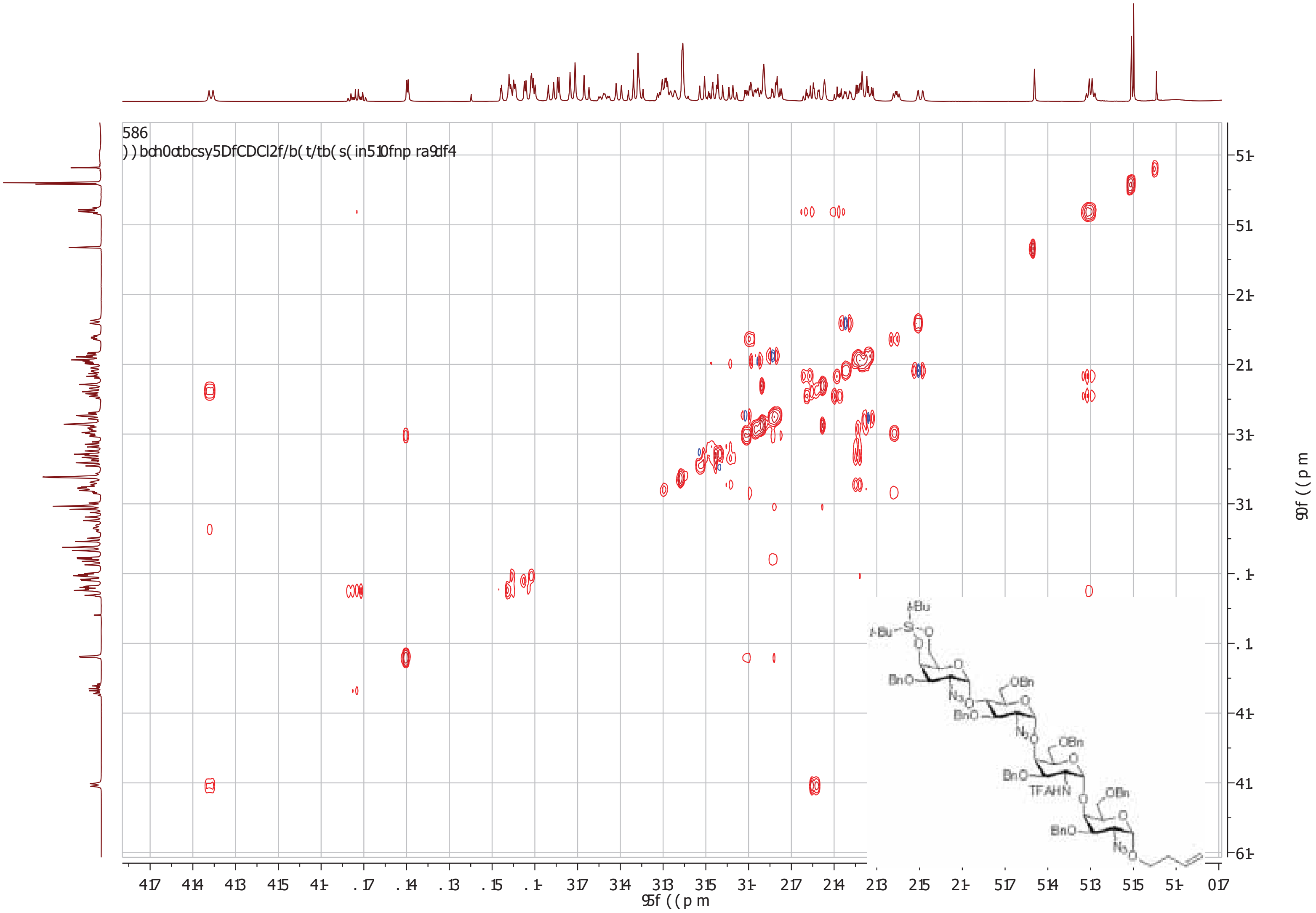

586
t-Bu
t-Bu
Si
BnO
N3
OBn
TFAHN

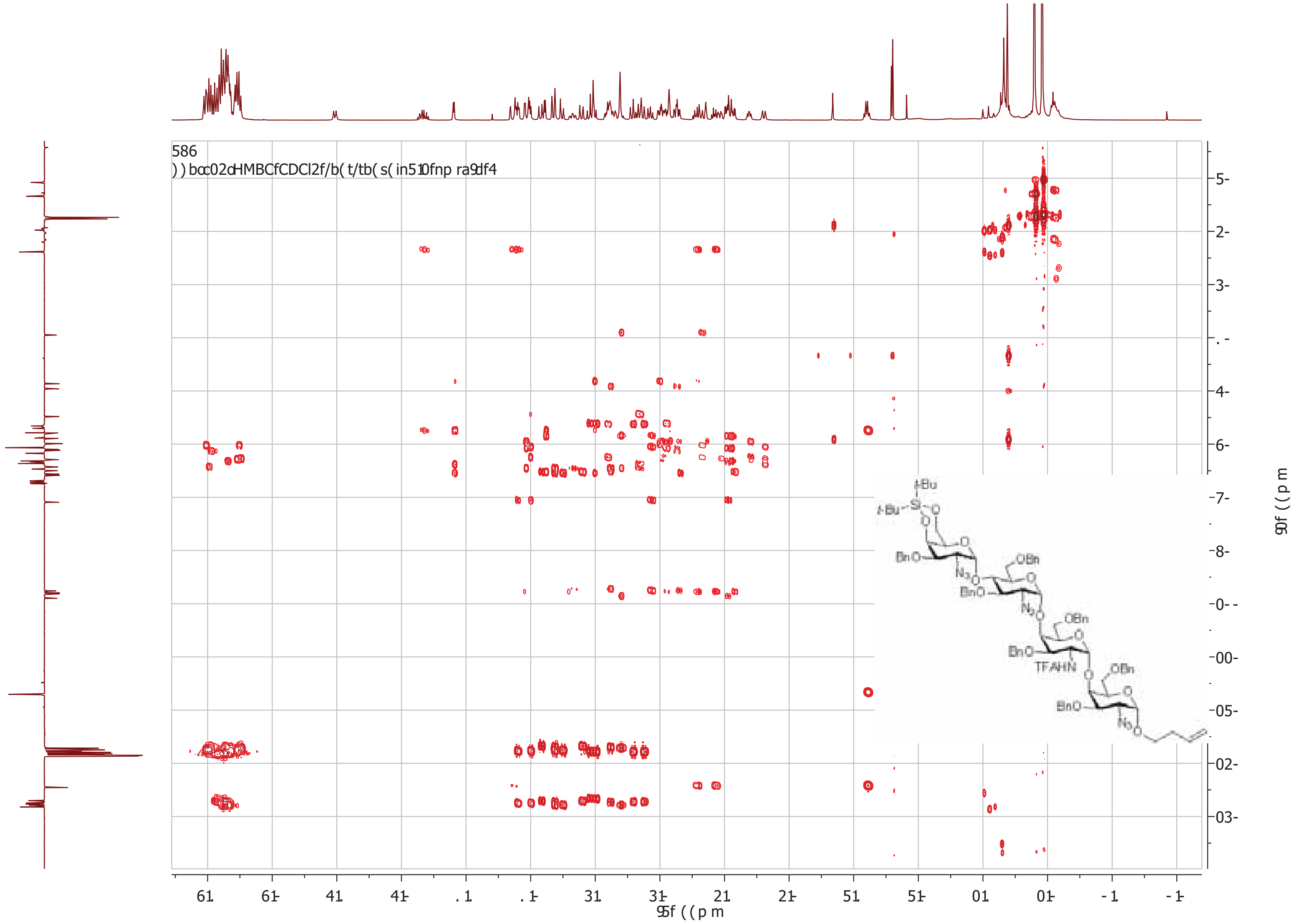
586
))bcc02cHMBCfCDCl2f/b(t/tb(s(in510fnp ra9df4
t-Bu
t-Bu
Si
BnO
N3
OBn
TFAHN

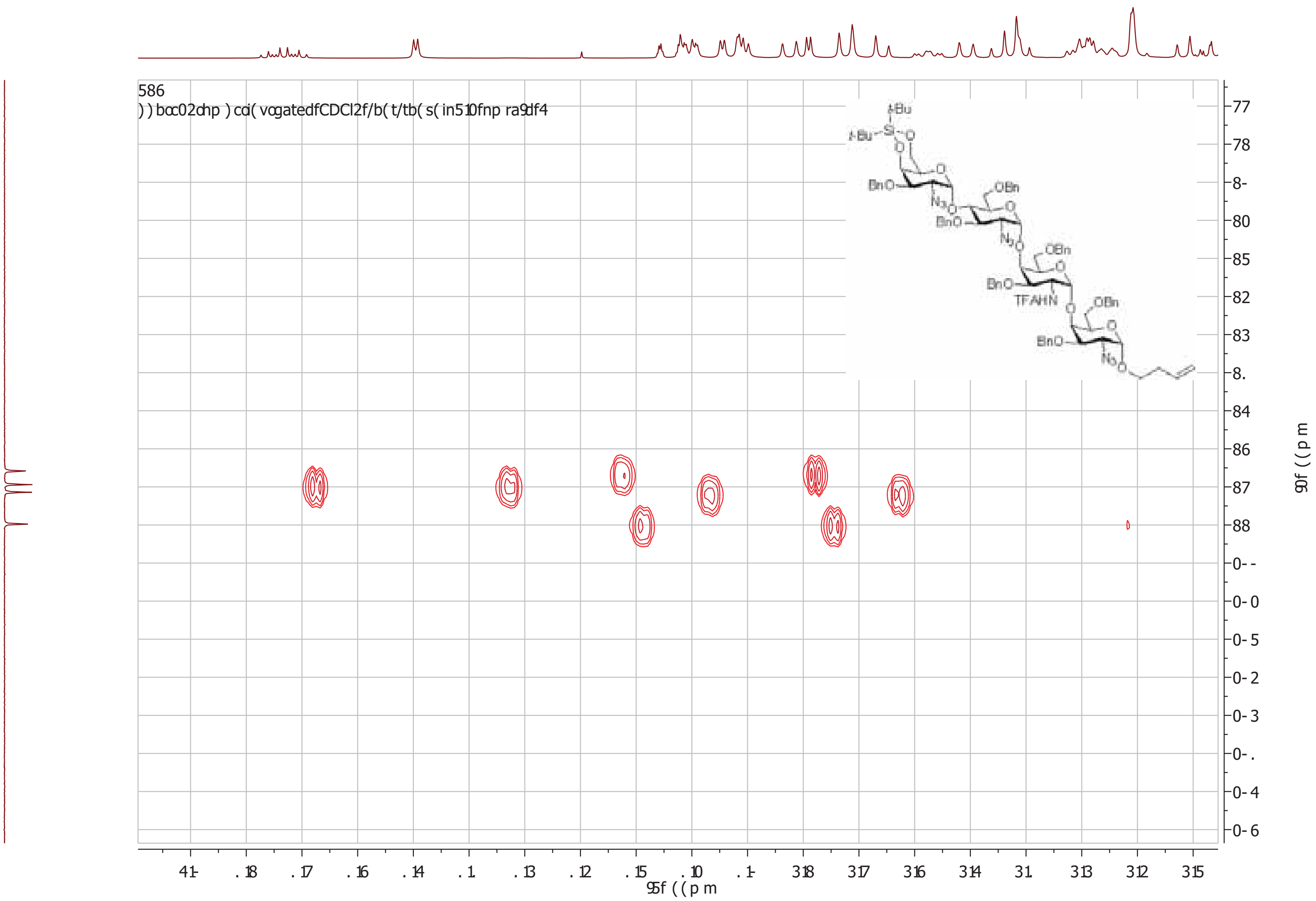
t-Bu
t-Bu
Si
O
BnO
N3
OBn
TFAHN

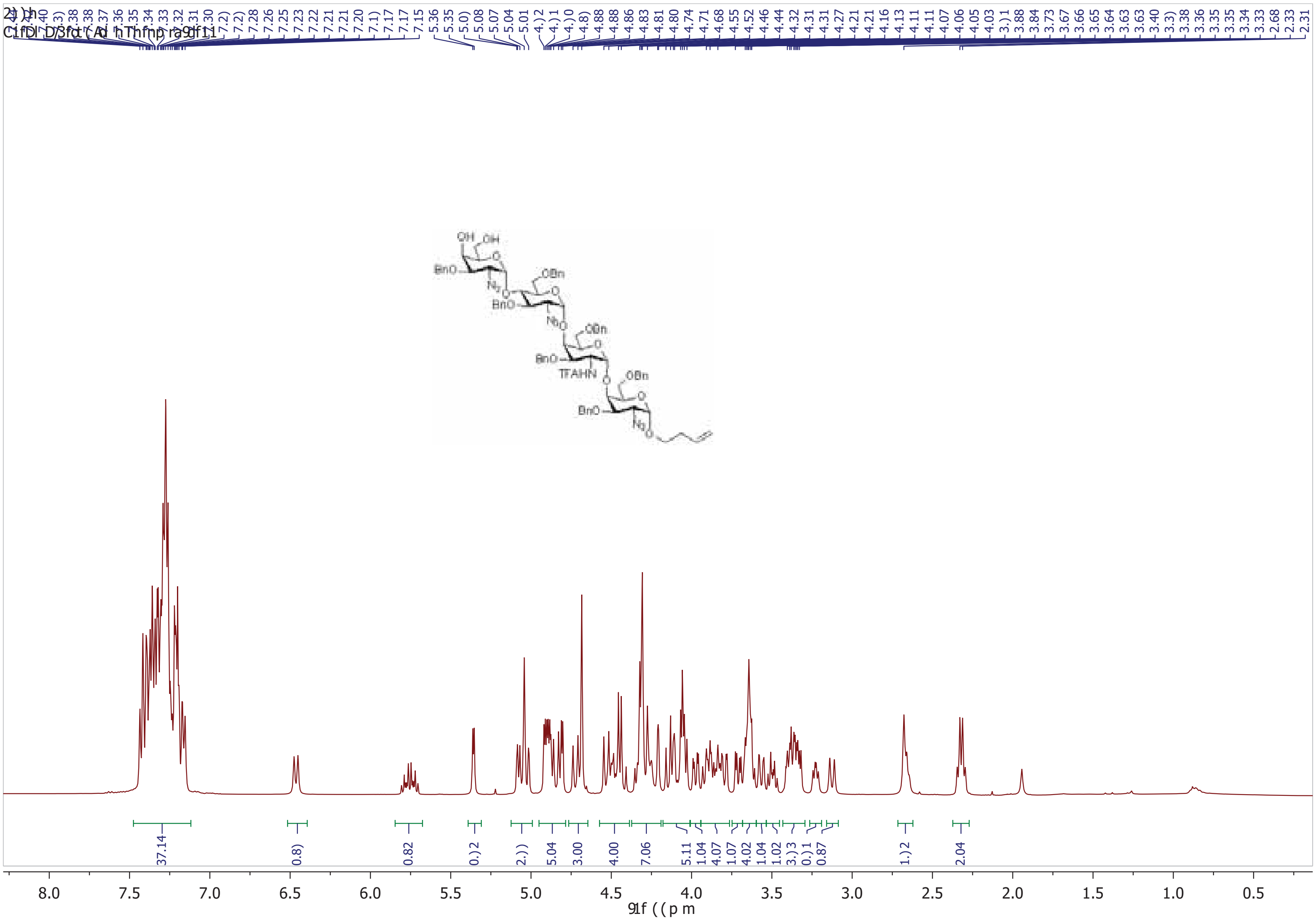

OH
OH
BnO
OBn
TFAHN
37.14
0.82
5.04
3.00
4.00
7.06
2.04
8.0
7.5
7.0
6.5
6.0
5.5
5.0
4.5
4.0
3.5
3.0
2.5
2.0
1.5
1.0
0.5

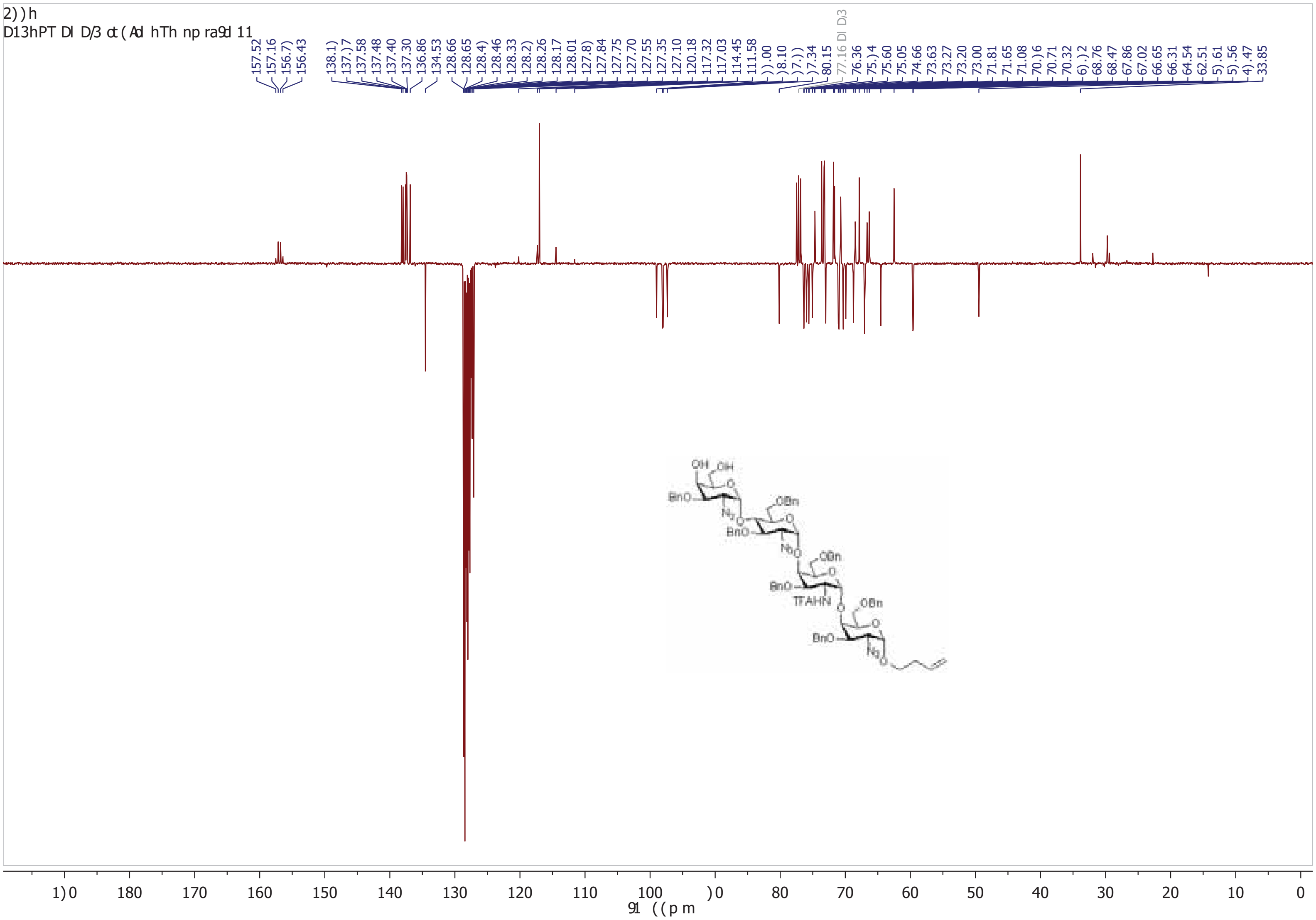
2))h
D13hPT DI D/3 ct (Ad hTh np ra9d 11
157.52
157.16
156.7)
156.43
138.1)
137.)7
137.58
137.48
137.40
137.30
136.86
134.53
128.66
128.65
128.4)
128.46
128.33
128.2)
128.26
128.17
128.01
127.8)
127.84
127.75
127.70
127.55
127.35
127.10
120.18
117.32
117.03
114.45
111.58
)).00
)8.10
)7.))
)7.34
80.15
77.16 DI D3
76.36
75.)4
75.60
75.05
74.66
73.63
73.27
73.20
73.00
71.81
71.65
71.08
70.)6
70.71
70.32
6).)2
68.76
68.47
67.86
67.02
66.65
66.31
64.54
62.51
5).61
5).56
4).47
33.85
OH
OH
BnO
N3
OBn
BnO
N3
OBn
BnO
TFAHN
OBn
BnO
N3
1)0
180
170
160
150
140
130
120
110
100
)0
80
70
60
50
40
30
20
10
0
91 ((pm

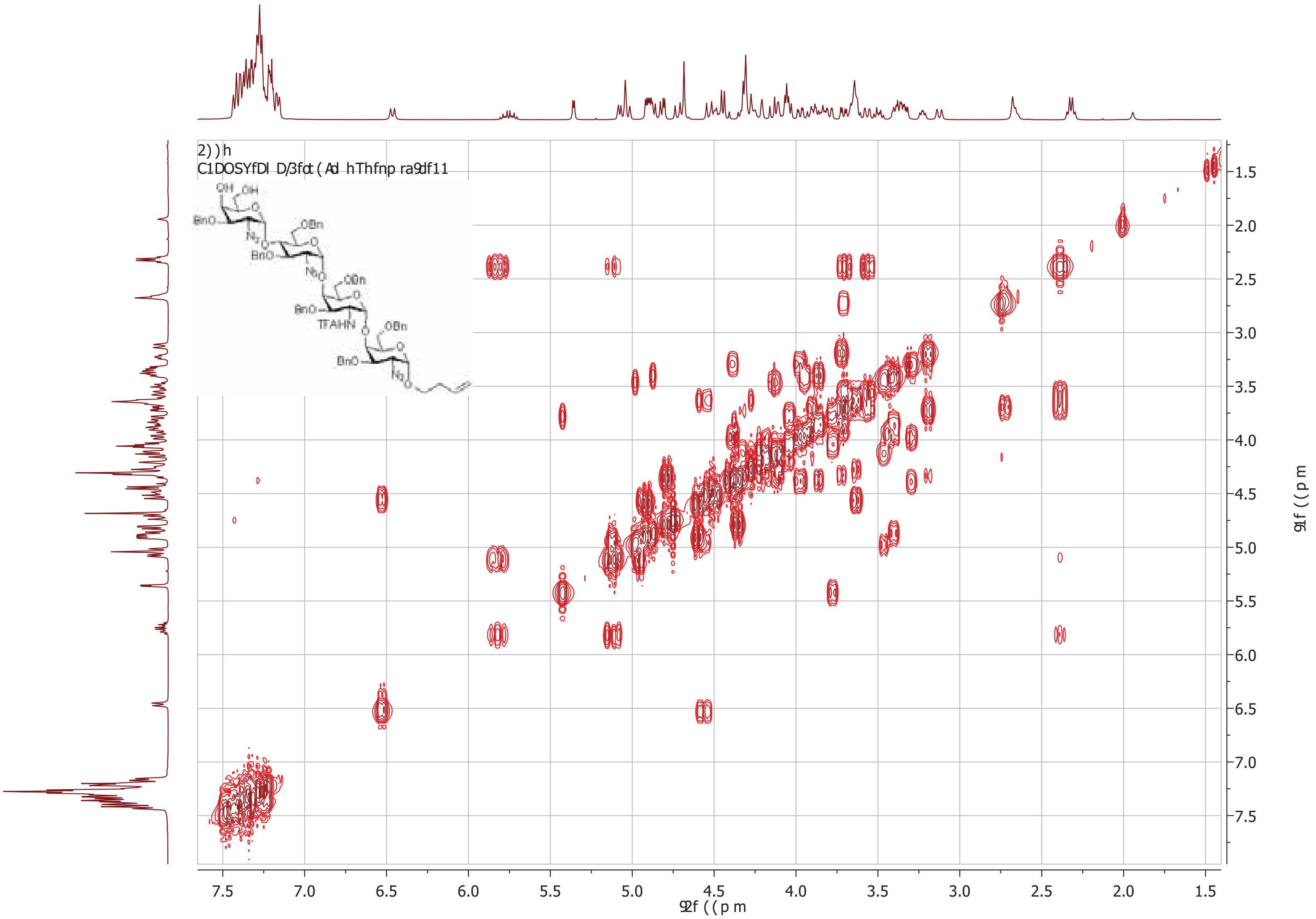

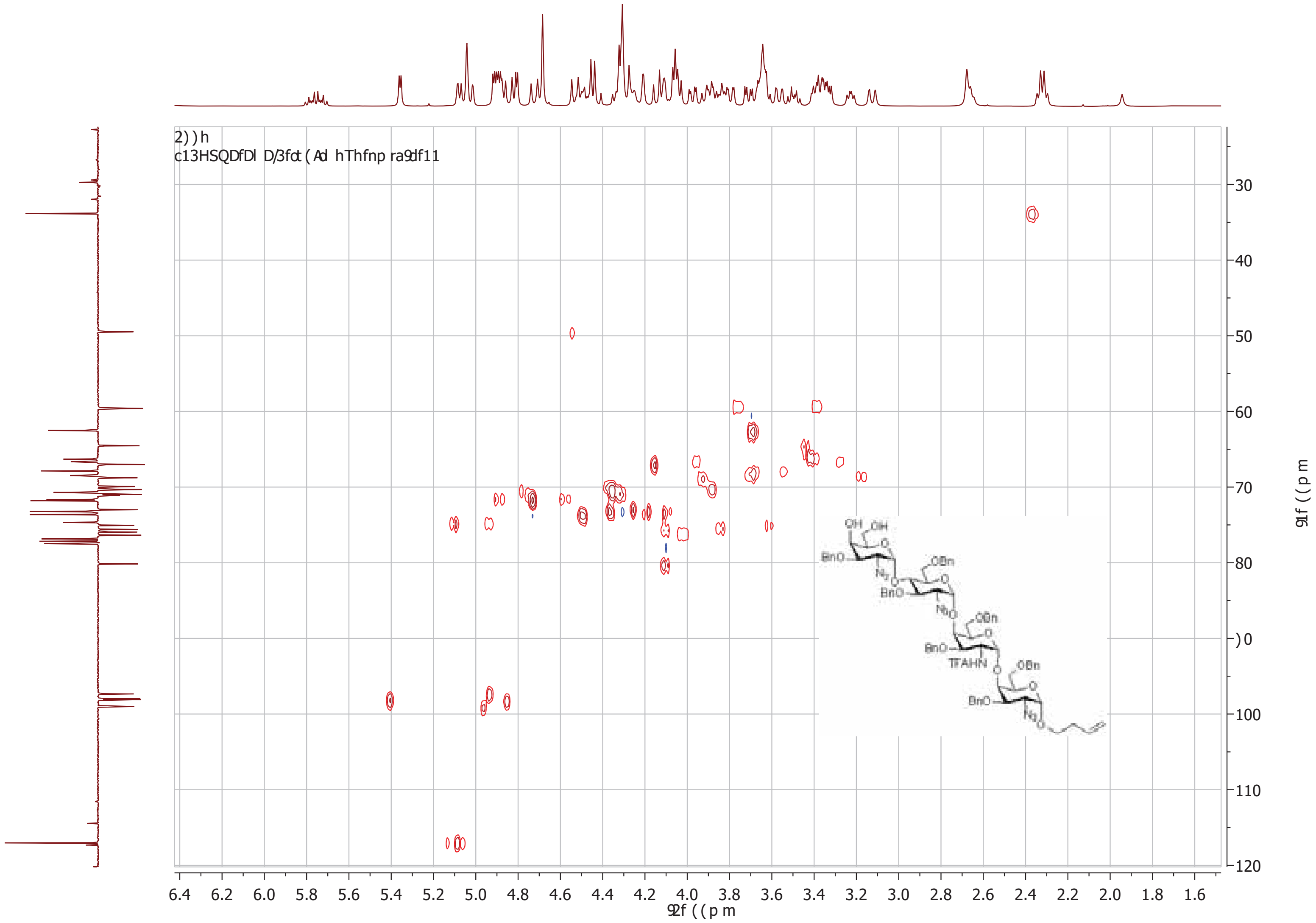

2))h
c13HSQDfDl D/3fct (Ad hThfnp ra9df11
OH
OH
BnO
N3
OBn
BnO
N3
OBn
BnO
TFAHN
OBn
BnO
N3
9f ((p m
91f ((p m

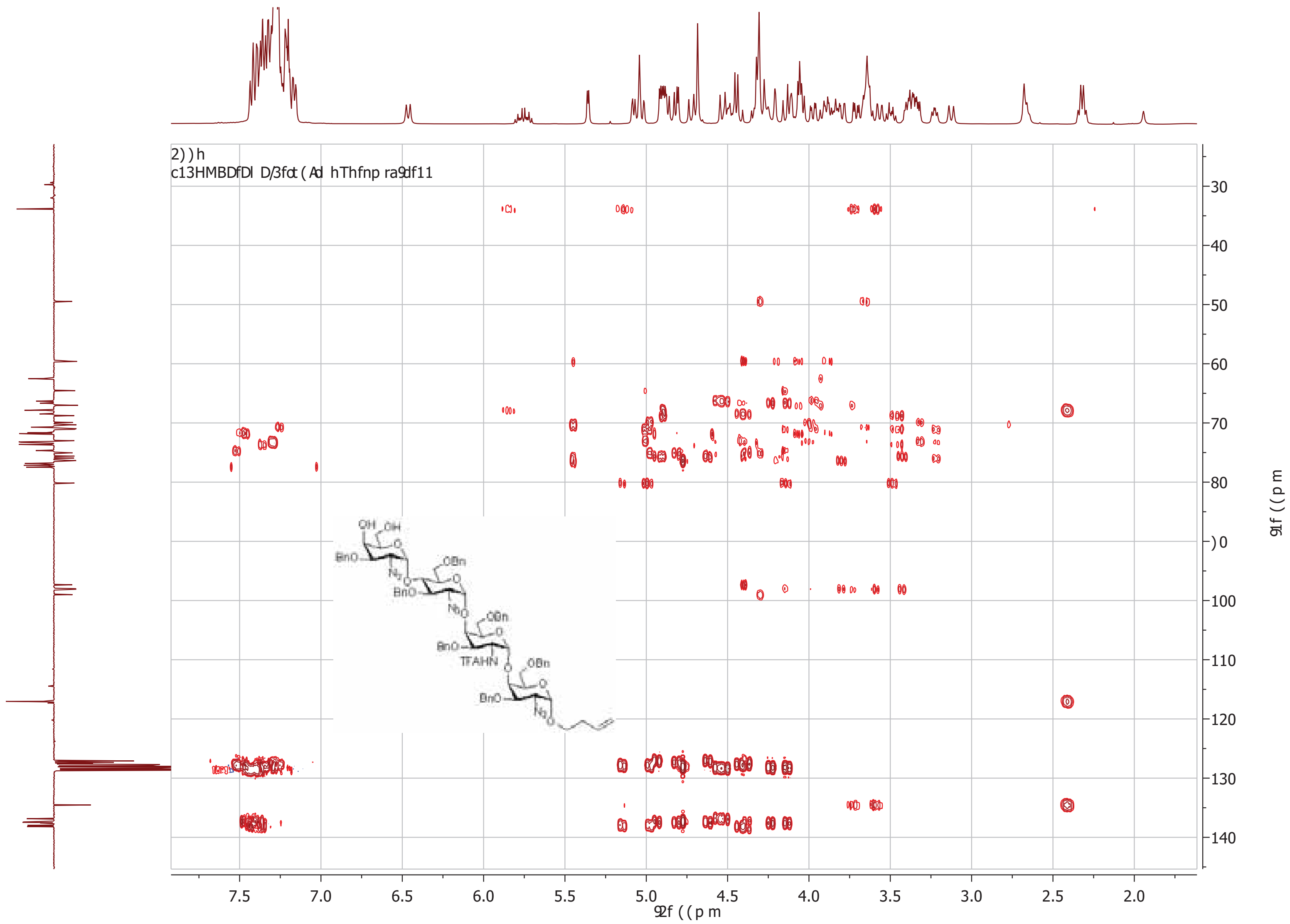
OH
OH
BnO
N3
OBn
BnO
N3
OBn
BnO
TFAHN
OBn
BnO
N3
30
40
50
60
70
80
100
110
120
130
140
7.5
7.0
6.5
6.0
5.5
5.0
4.5
4.0
3.5
3.0
2.5
2.0

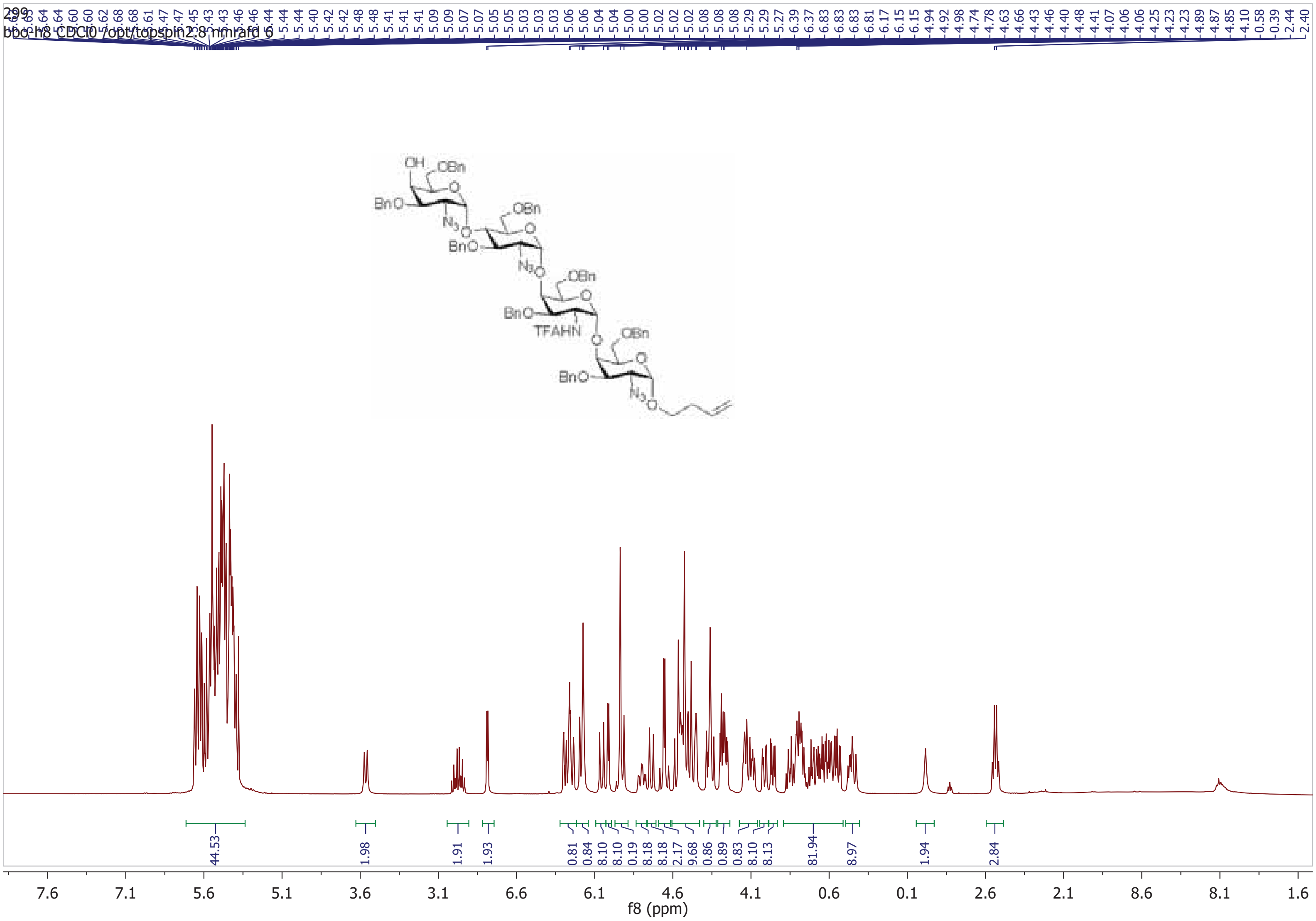
299
bbo-h8 CDCl0 /opt/topspin2.8 nmrafd 6
OH
OBn
BnO
N_3
OBn
BnO
N_3
OBn
BnO
TFAHN
OBn
BnO
N_3
44.53
1.98
1.91
1.93
0.81
0.84
8.10
8.10
0.19
8.18
8.18
2.17
9.68
0.86
0.89
0.83
8.10
8.13
81.94
8.97
1.94
2.84
7.6 7.1 5.6 5.1 3.6 3.1 6.6 6.1 4.6 4.1 0.6 0.1 2.6 2.1 8.6 8.1 1.6
f8 (ppm)

299
bbo-c80-APT CDCl0 /opt/topspin2.8 nmrafd 6

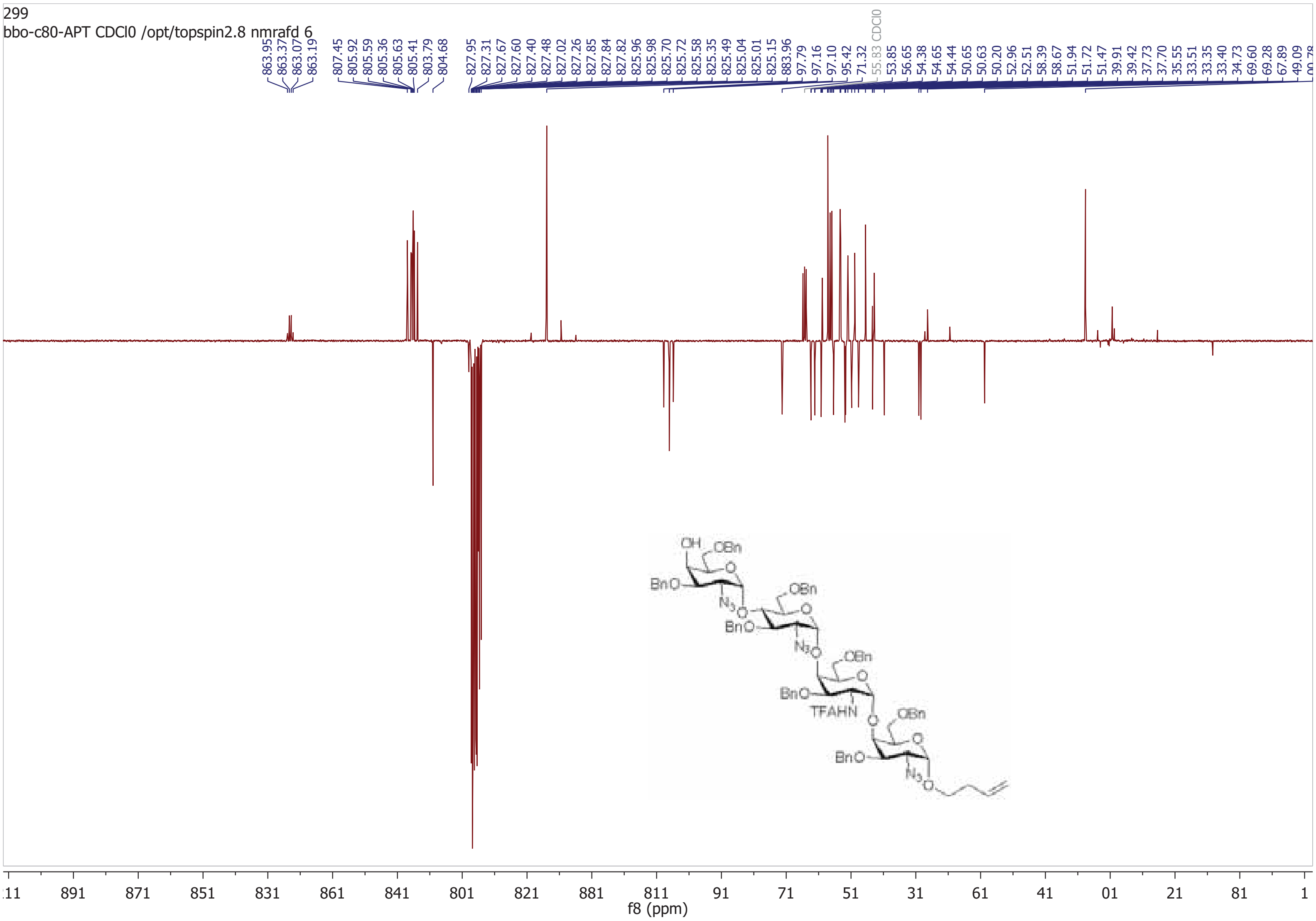

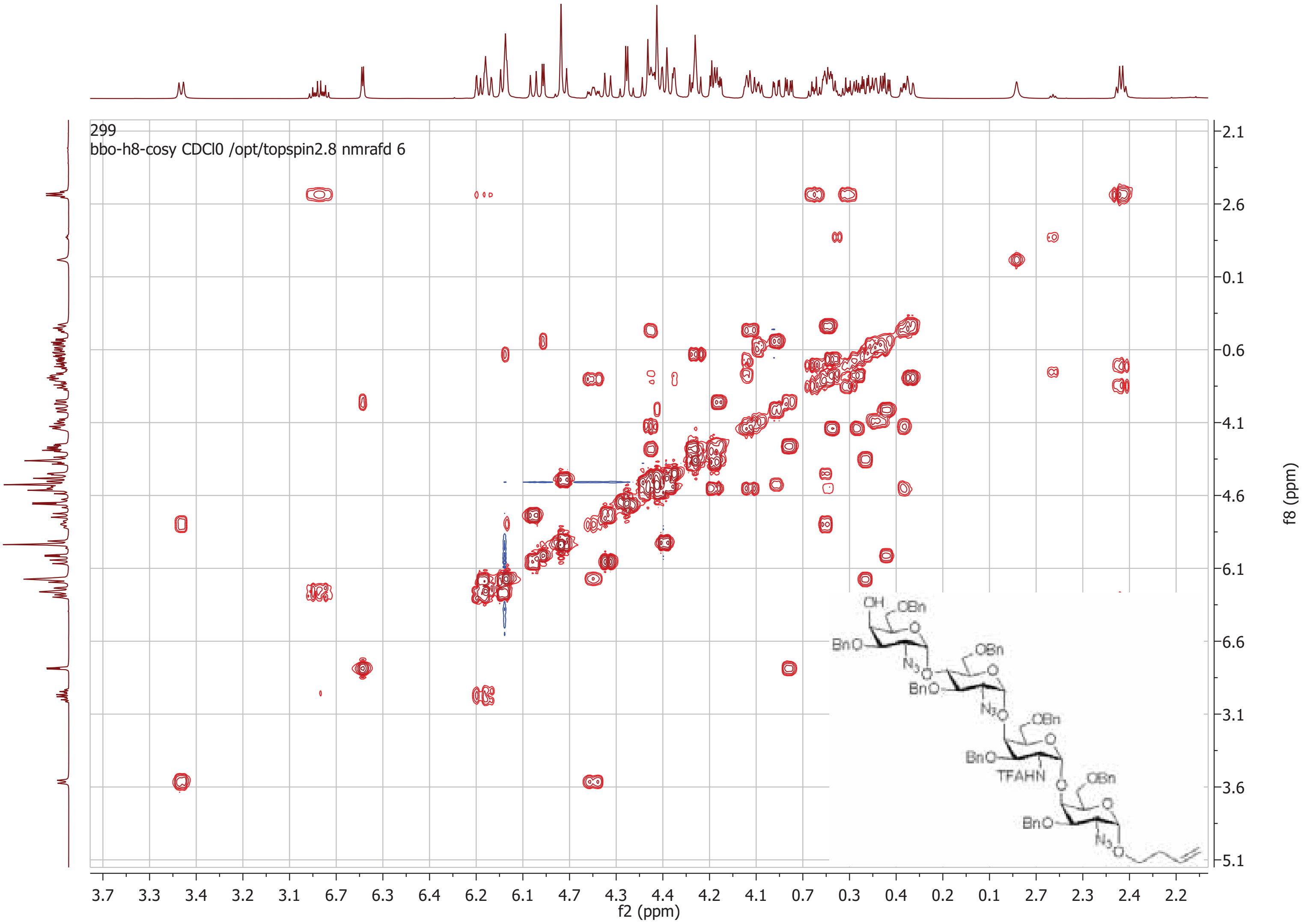

299
bbo-h8-cosy CDCl0 /opt/topspin2.8 nmrafd 6
3.7 3.3 3.4 3.2 3.1 6.7 6.3 6.4 6.2 6.1 4.7 4.3 4.4 4.2 4.1 0.7 0.3 0.4 0.2 0.1 2.7 2.3 2.4 2.2
f2 (ppm)
2.1 2.6 0.1 0.6 4.1 4.6 6.1 6.6 3.1 3.6 5.1
f8 (ppm)
OH
OBn
BnO
N3
OBn
BnO
N3
OBn
BnO
TFAHN
OBn
BnO
N3

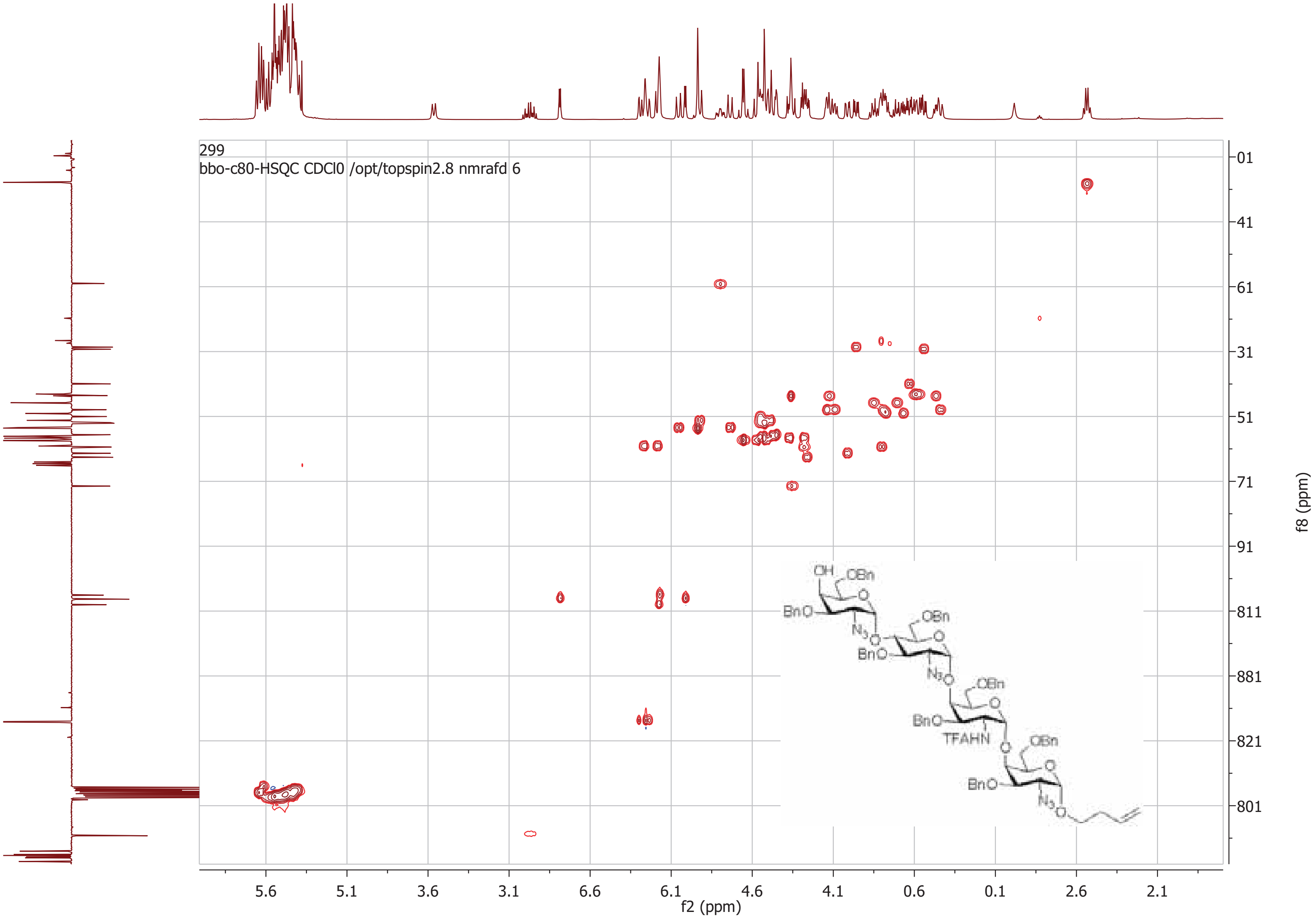
299
bbo-c80-HSQC CDCl0 /opt/topspin2.8 nmrafd 6
f2 (ppm)
f8 (ppm)
OH
OBn
BnO
N3
TFAHN

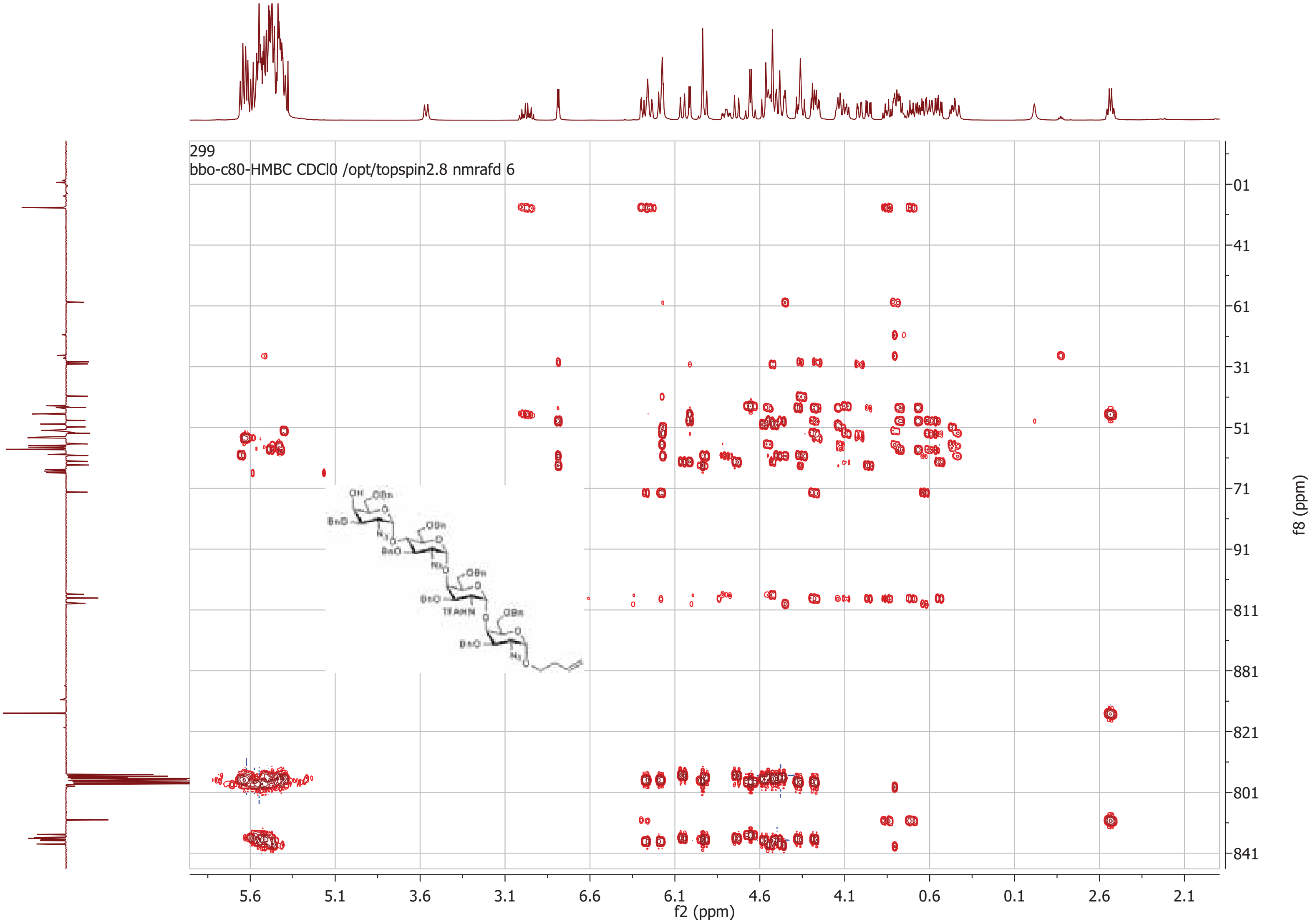
299
bbo-c80-HMBC CDCl0 /opt/topspin2.8 nmrafd 6
f2 (ppm)
f8 (ppm)
5.6
5.1
3.6
3.1
6.6
6.1
4.6
4.1
0.6
0.1
2.6
2.1
01
41
61
31
51
71
91
811
881
821
801
841

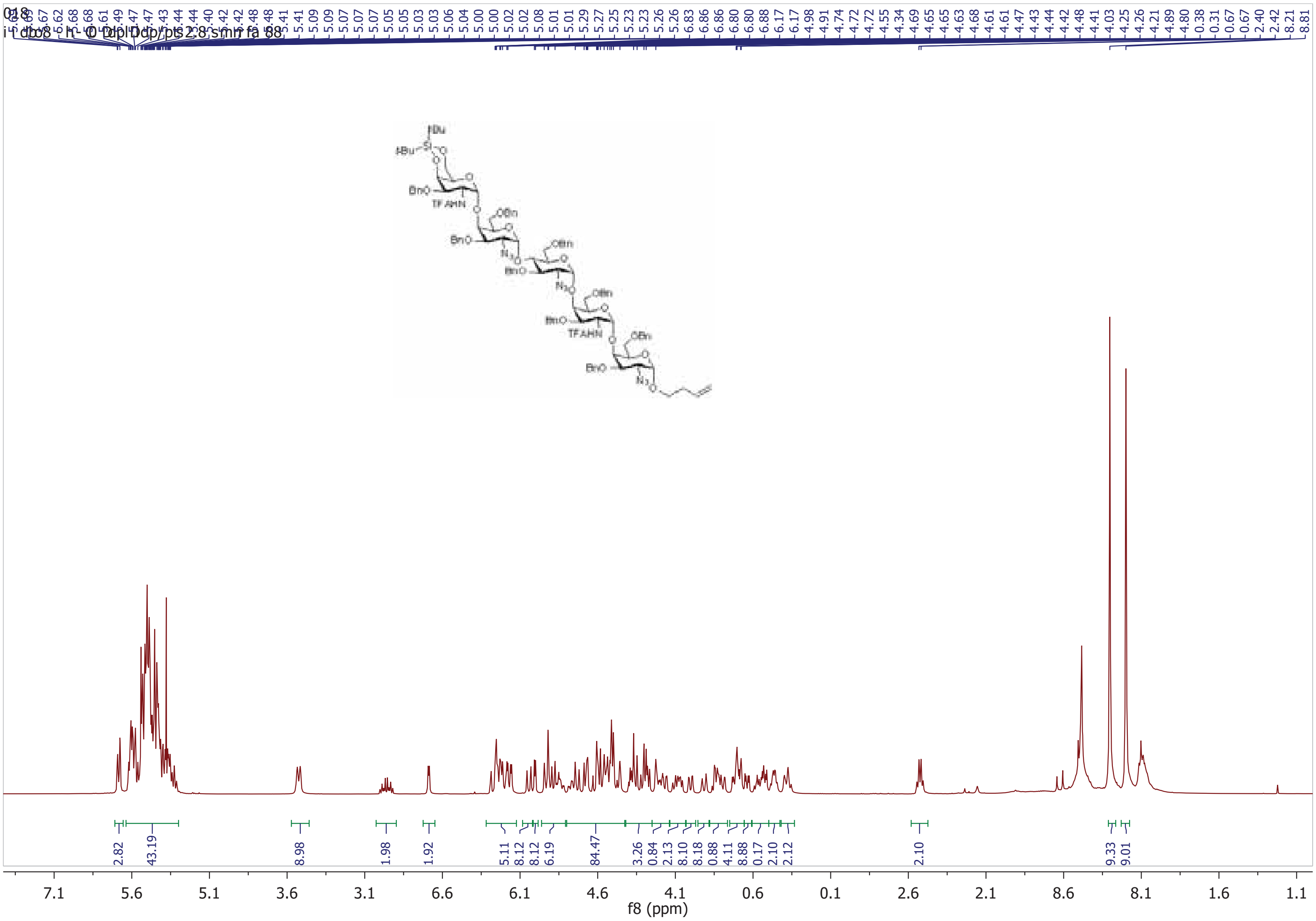
t-Bu
BnO
TFAHN
OBn
N3
2.82
43.19
8.98
1.98
1.92
5.11
8.12
8.12
6.19
84.47
3.26
0.84
2.13
8.10
8.18
0.88
4.11
8.88
0.17
2.10
2.12
2.10
9.33
9.01
7.1 5.6 5.1 3.6 3.1 6.6 6.1 4.6 4.1 0.6 0.1 2.6 2.1 8.6 8.1 1.6 1.1
f8 (ppm)

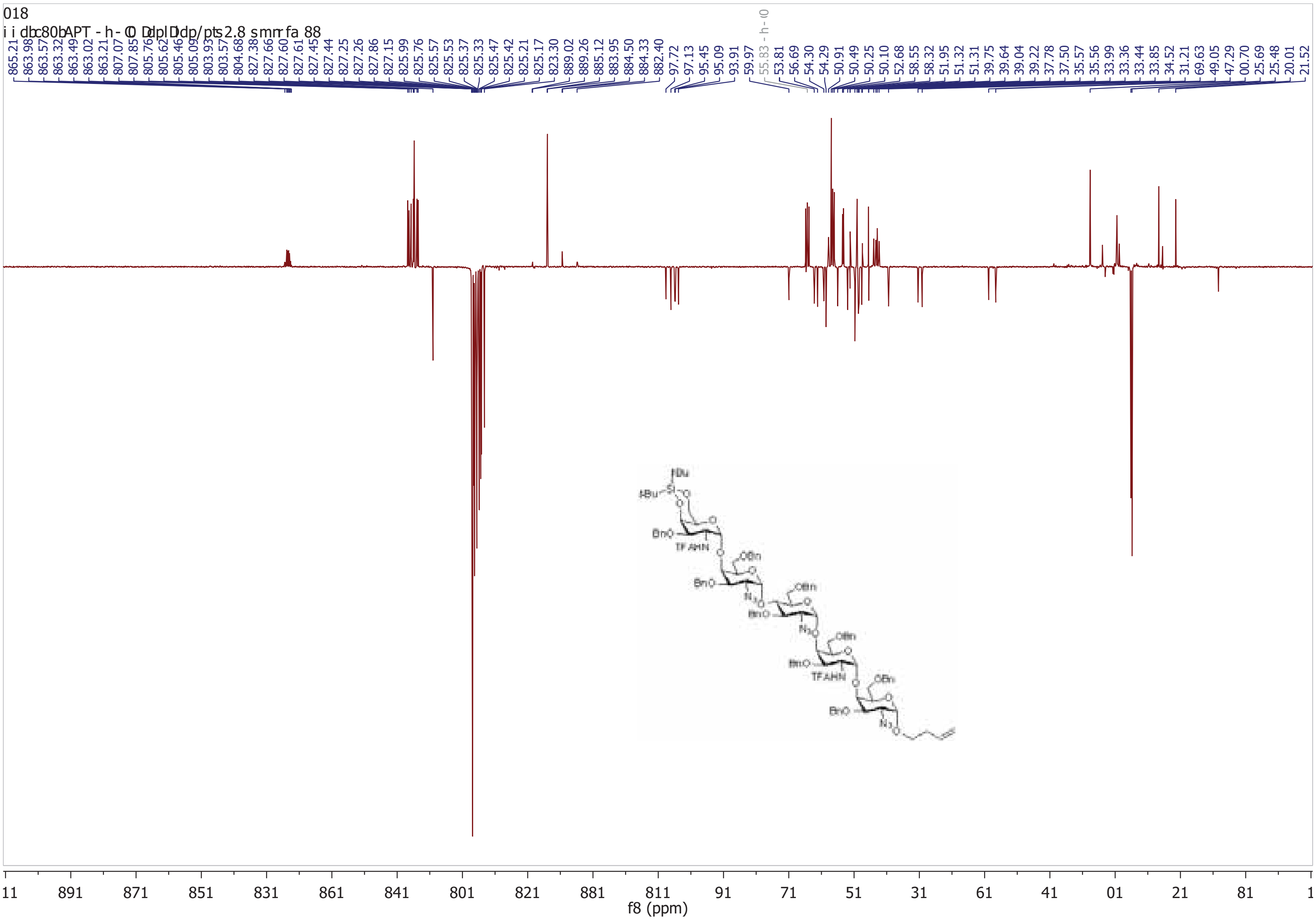
018
i i dbc80bAPT - h- (I) Ddpl Ddp/pts2.8 smrrfa 88
865.21
863.98
863.57
863.32
863.49
863.02
863.21
807.07
807.85
805.76
805.62
805.46
805.09
803.93
803.57
804.68
827.38
827.66
827.60
827.61
827.45
827.44
827.25
827.26
827.86
827.15
825.99
825.76
825.57
825.53
825.37
825.33
825.47
825.42
825.21
825.17
823.30
889.02
889.26
885.12
883.95
884.50
884.33
882.40
97.72
97.13
95.45
95.09
93.91
59.97
55.83 - h- i0
53.81
56.69
54.30
54.29
50.91
50.49
50.25
50.10
52.68
58.55
58.32
51.95
51.32
51.31
39.75
39.64
39.04
39.22
37.78
37.50
35.57
35.56
33.99
33.36
33.44
33.85
34.52
31.21
69.63
49.05
47.29
00.70
25.69
25.48
20.01
21.52
tBu
tBu
Si
BnO
TFAHN
OBn
BnO
N3
OBn
BnO
N3
OBn
BnO
TFAHN
OBn
BnO
N3
11
891
871
851
831
861
841
801
821
881
811
91
71
51
31
61
41
01
21
81
1
f8 (ppm)

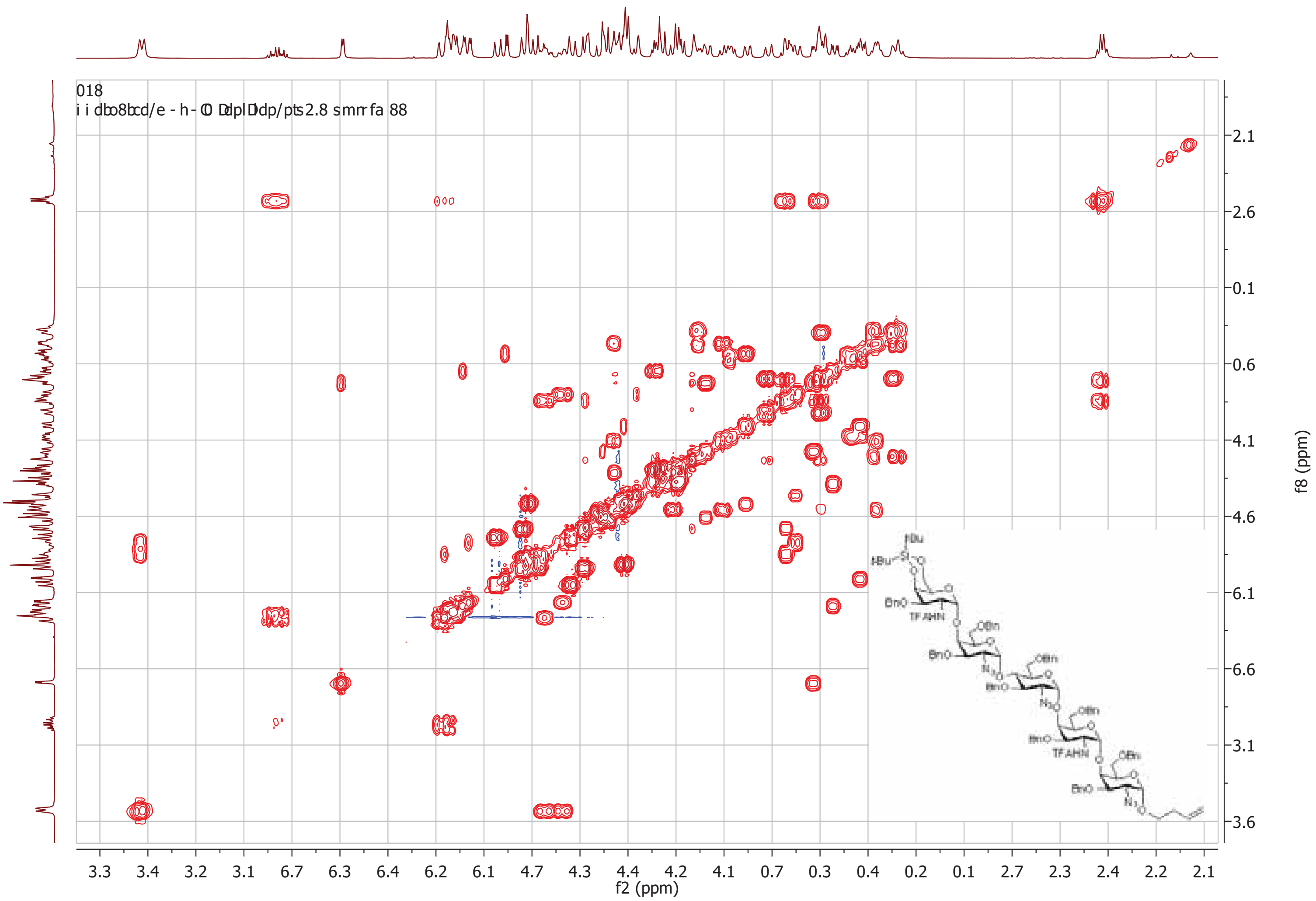
018
f2 (ppm)
f8 (ppm)
BnO
TFAHN
OBn
N3

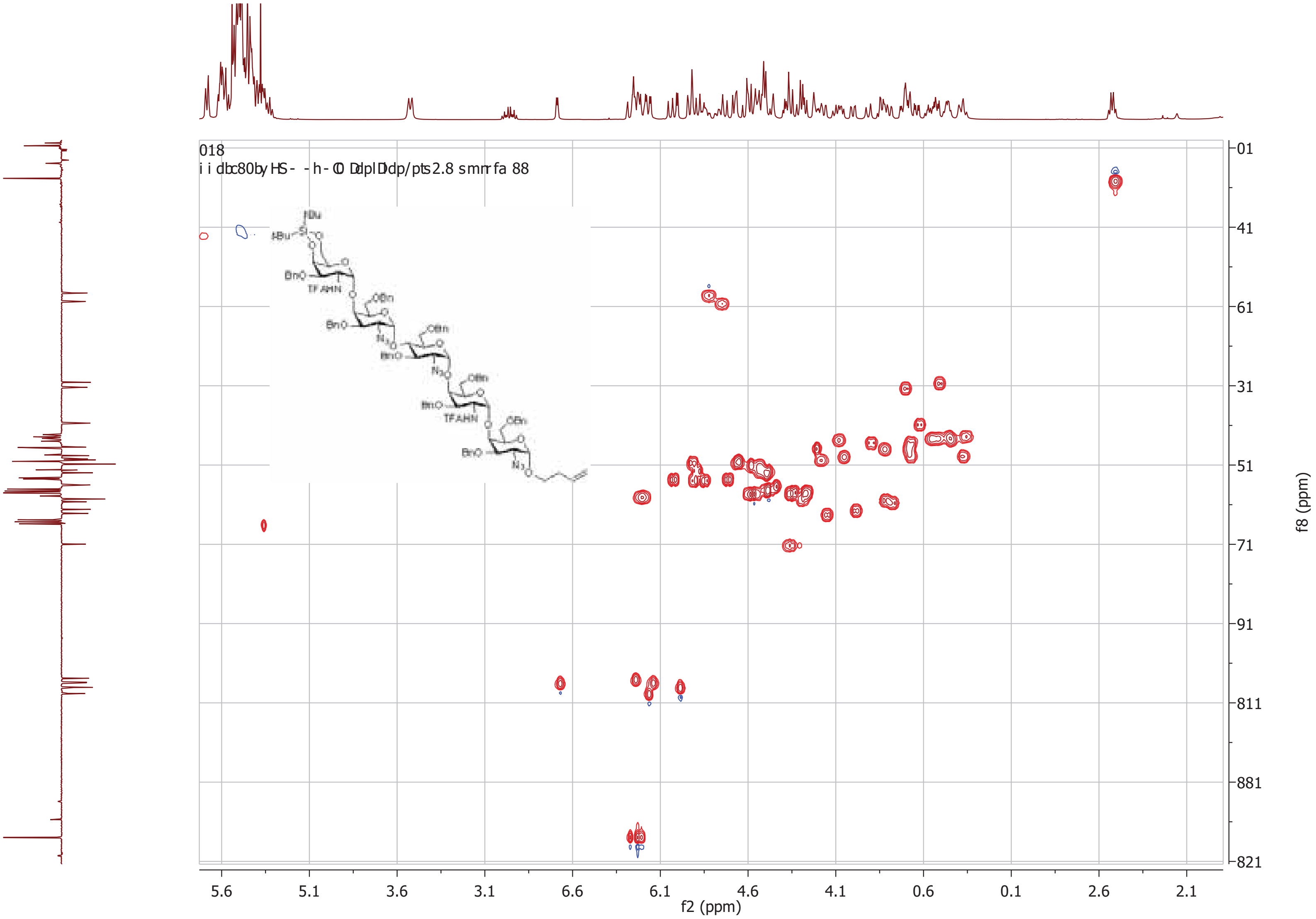
018
i i dbc80by HS- -h-Cl Ddp lDdp/pts 2.8 smrr fa 88
f2 (ppm)
f8 (ppm)
BnO
TFAHN
OBn
N3

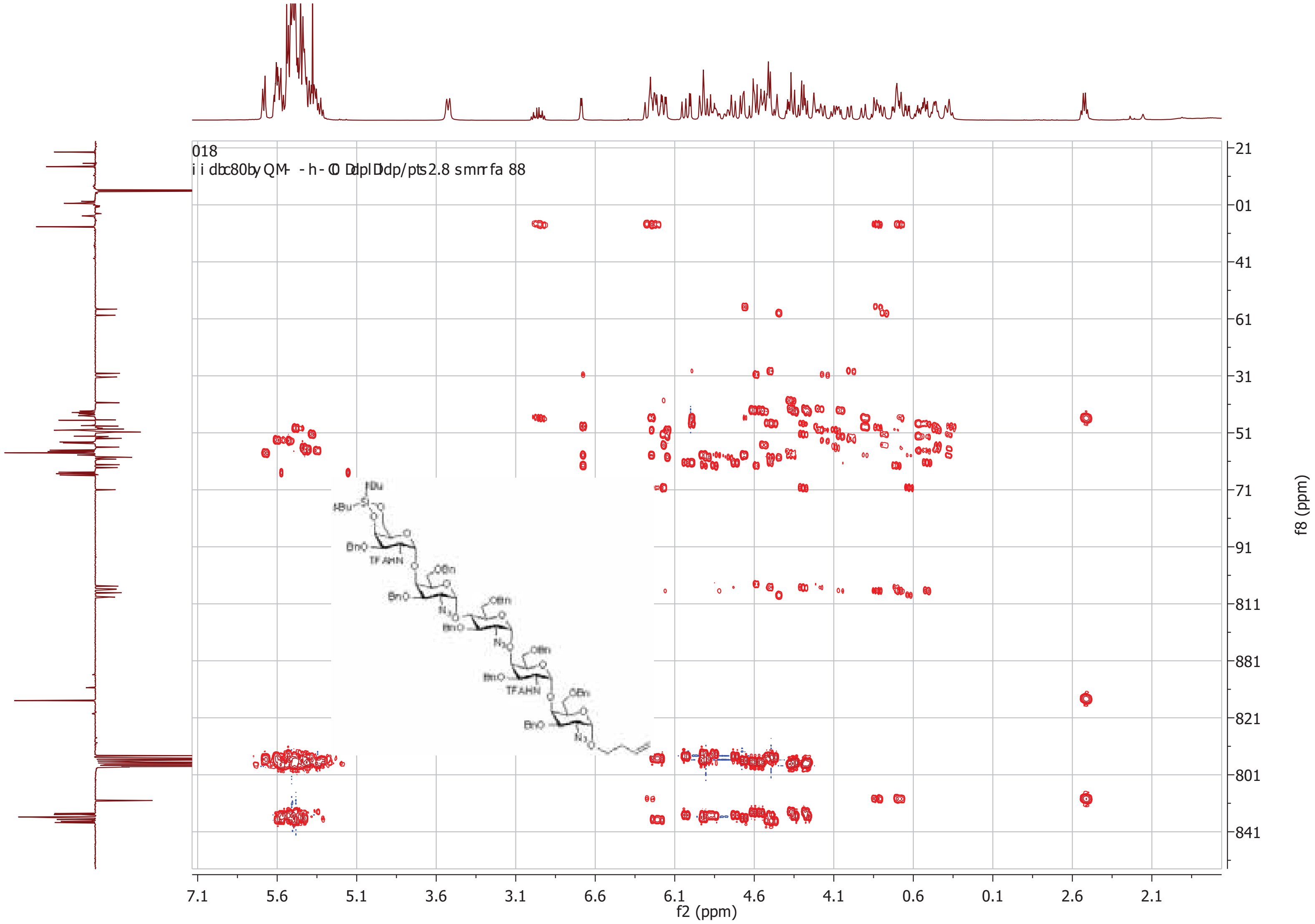
018
f2 (ppm)
f8 (ppm)
tBu
BnO
TFAHN
OBn
N3

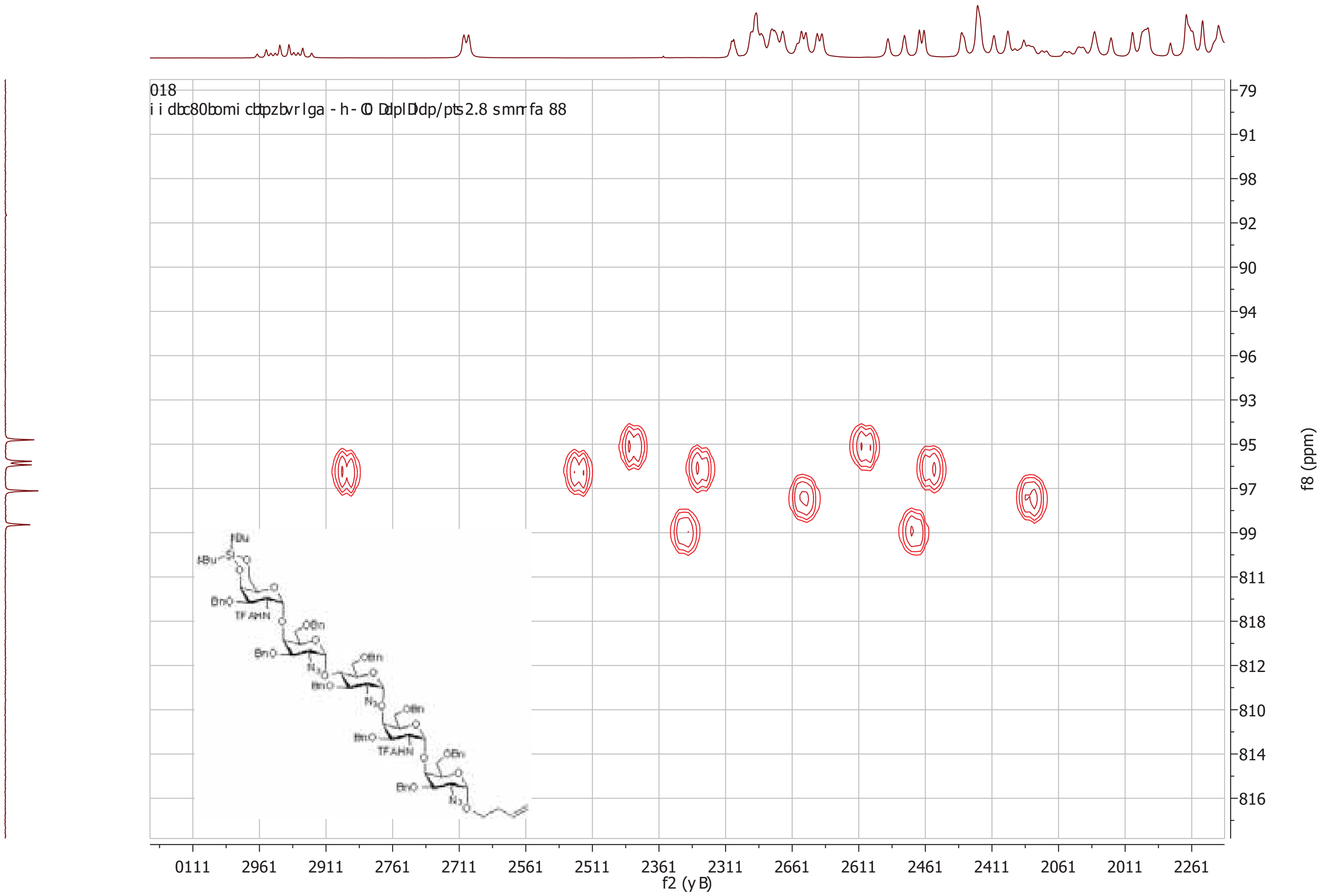
f2 (y B)
f8 (ppm)
t-Bu
Si
BnO
TFAHN
OBn
N3
OBn

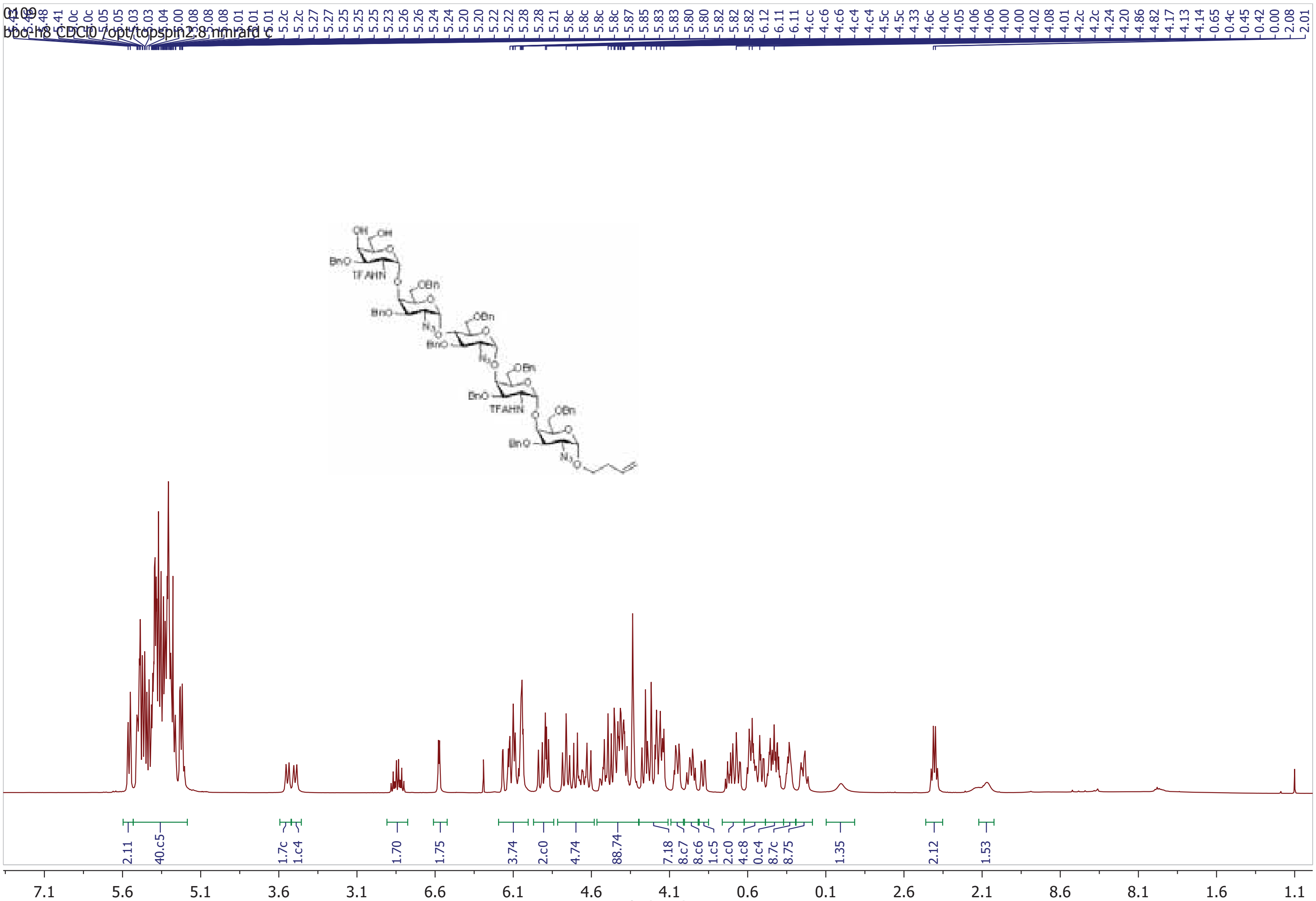
0109
OH
OH
BnO
TFAHN
OBn
BnO
N3
OBn
BnO
N3
OBn
BnO
TFAHN
OBn
BnO
N3
2.11
40.c5
1.7c
1.c4
1.70
1.75
3.74
2.c0
4.74
88.74
7.18
8.c7
8.c6
1.c5
2.c0
4.c8
0.c4
8.7c
8.75
1.35
2.12
1.53
7.1
5.6
5.1
3.6
3.1
6.6
6.1
4.6
4.1
0.6
0.1
2.6
2.1
8.6
8.1
1.6
1.1
f8 (ppm)

0109
bbo-A80-9PT CDCl0 /opt/topspin2.8 nmrafd c

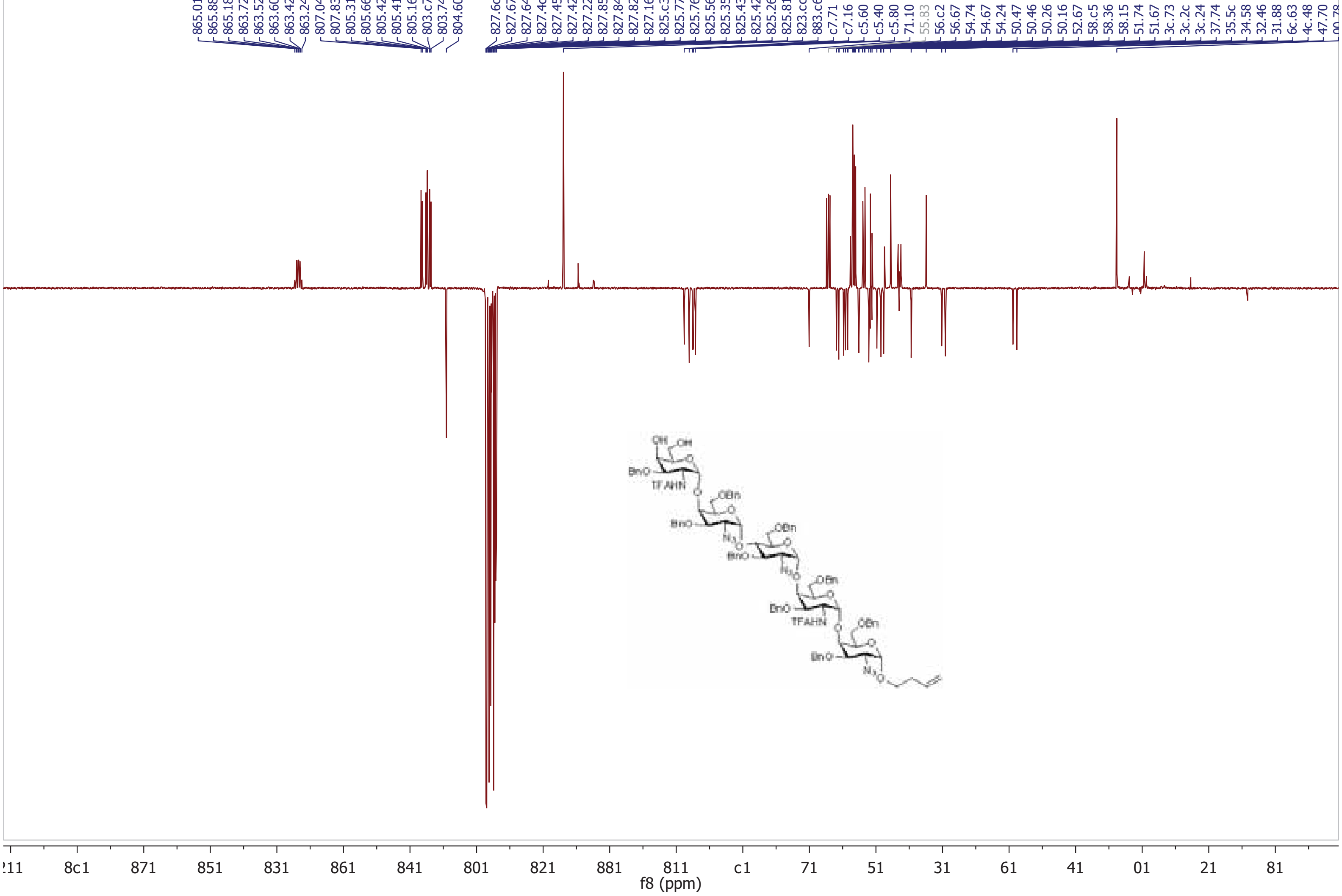

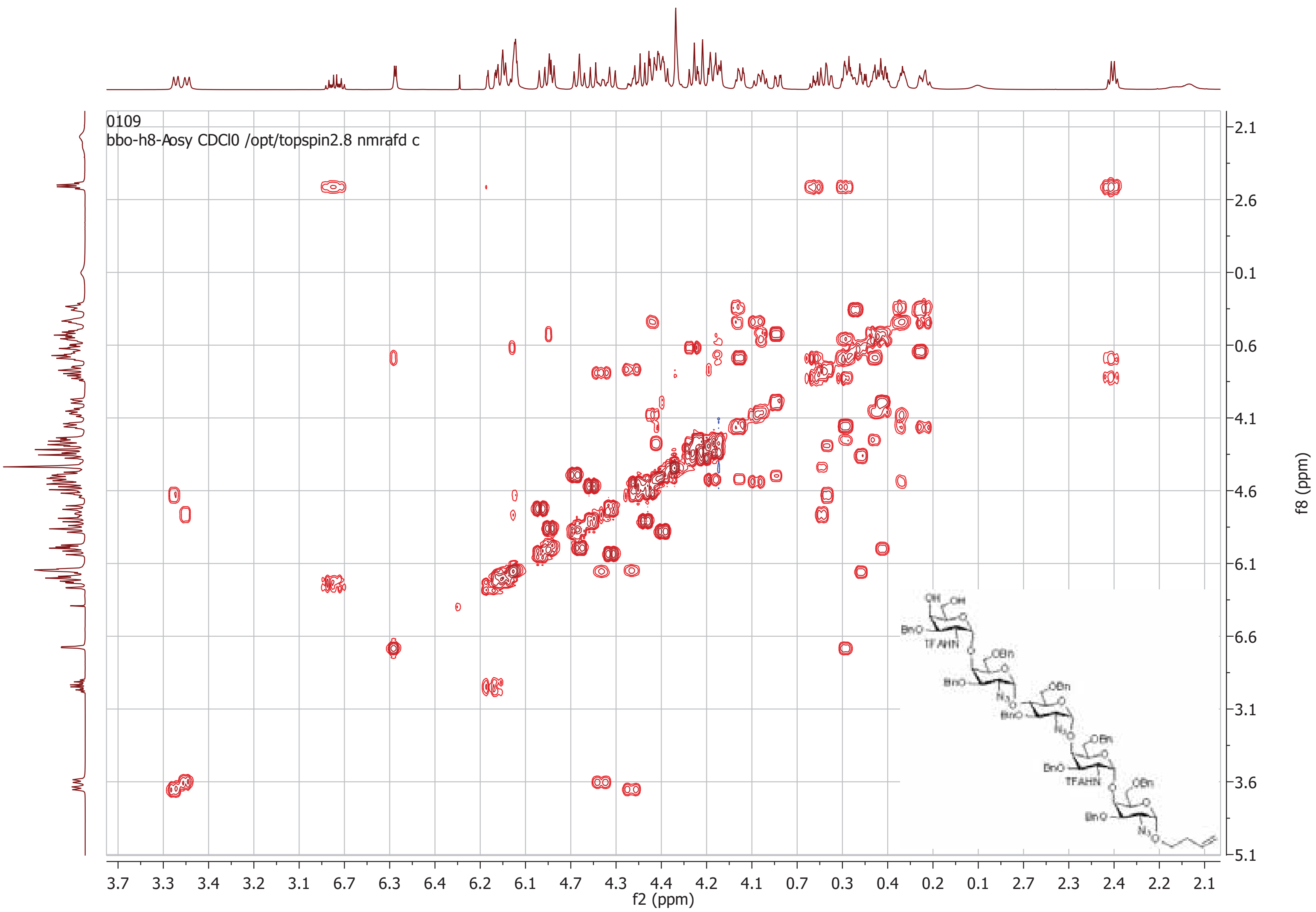
0109
bbo-h8-Aosy CDCl0 /opt/topspin2.8 nmrafd c
f2 (ppm)
f8 (ppm)
3.7 3.3 3.4 3.2 3.1 6.7 6.3 6.4 6.2 6.1 4.7 4.3 4.4 4.2 4.1 0.7 0.3 0.4 0.2 0.1 2.7 2.3 2.4 2.2 2.1
2.1 2.6 0.1 0.6 4.1 4.6 6.1 6.6 3.1 3.6 5.1
OH
OH
BnO
TFAHN
OBn
TFAHN

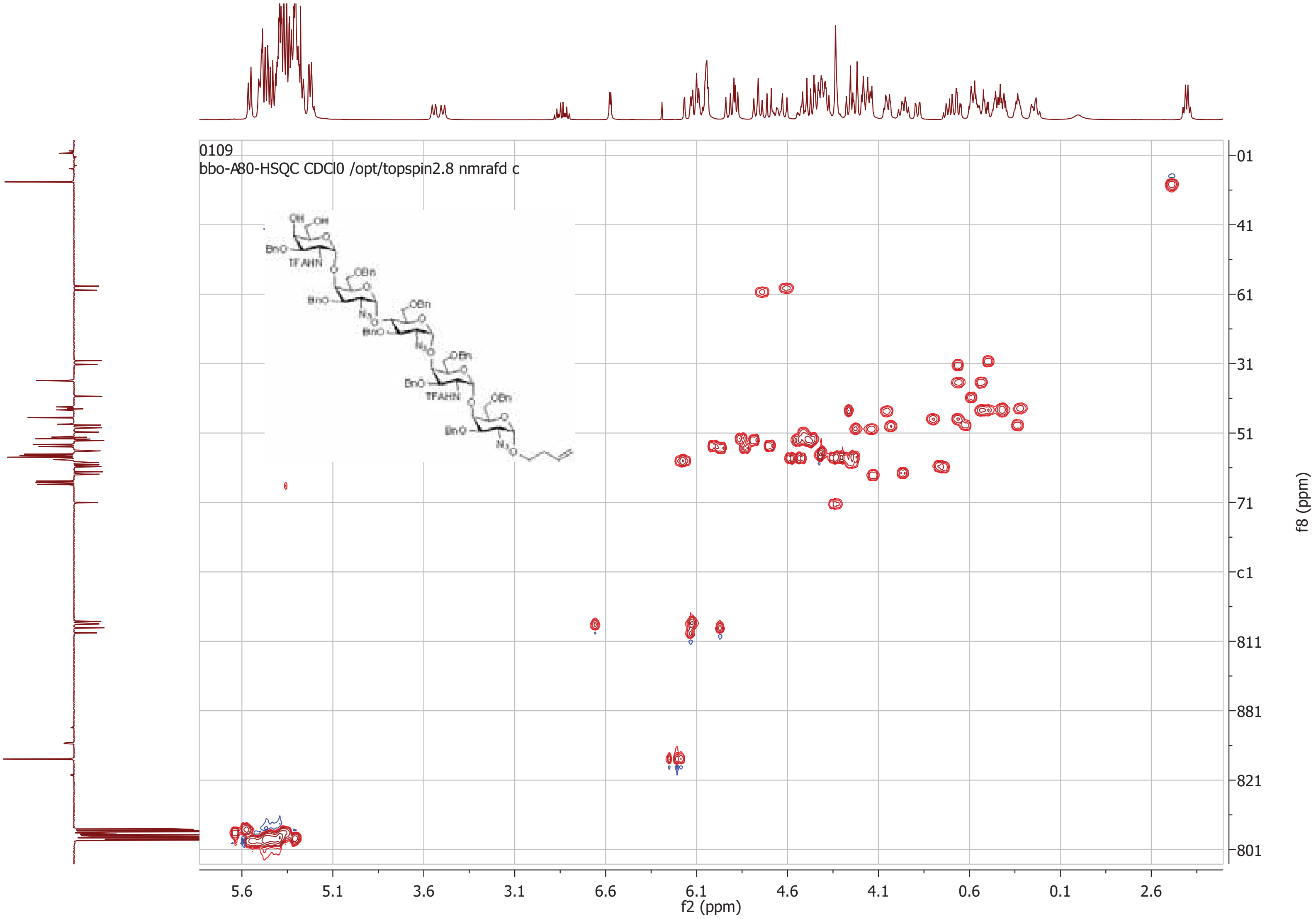
0109
bbo-A80-HSQC CDCl0 /opt/topspin2.8 nmrafd c
OH
OH
BnO
TFAHN
OBn
BnO
N3
OBn
BnO
N3
OBn
BnO
TFAHN
OBn
BnO
N3
01
41
61
31
51
71
c1
811
881
821
801
f8 (ppm)
5.6
5.1
3.6
3.1
6.6
6.1
4.6
4.1
0.6
0.1
2.6
f2 (ppm)

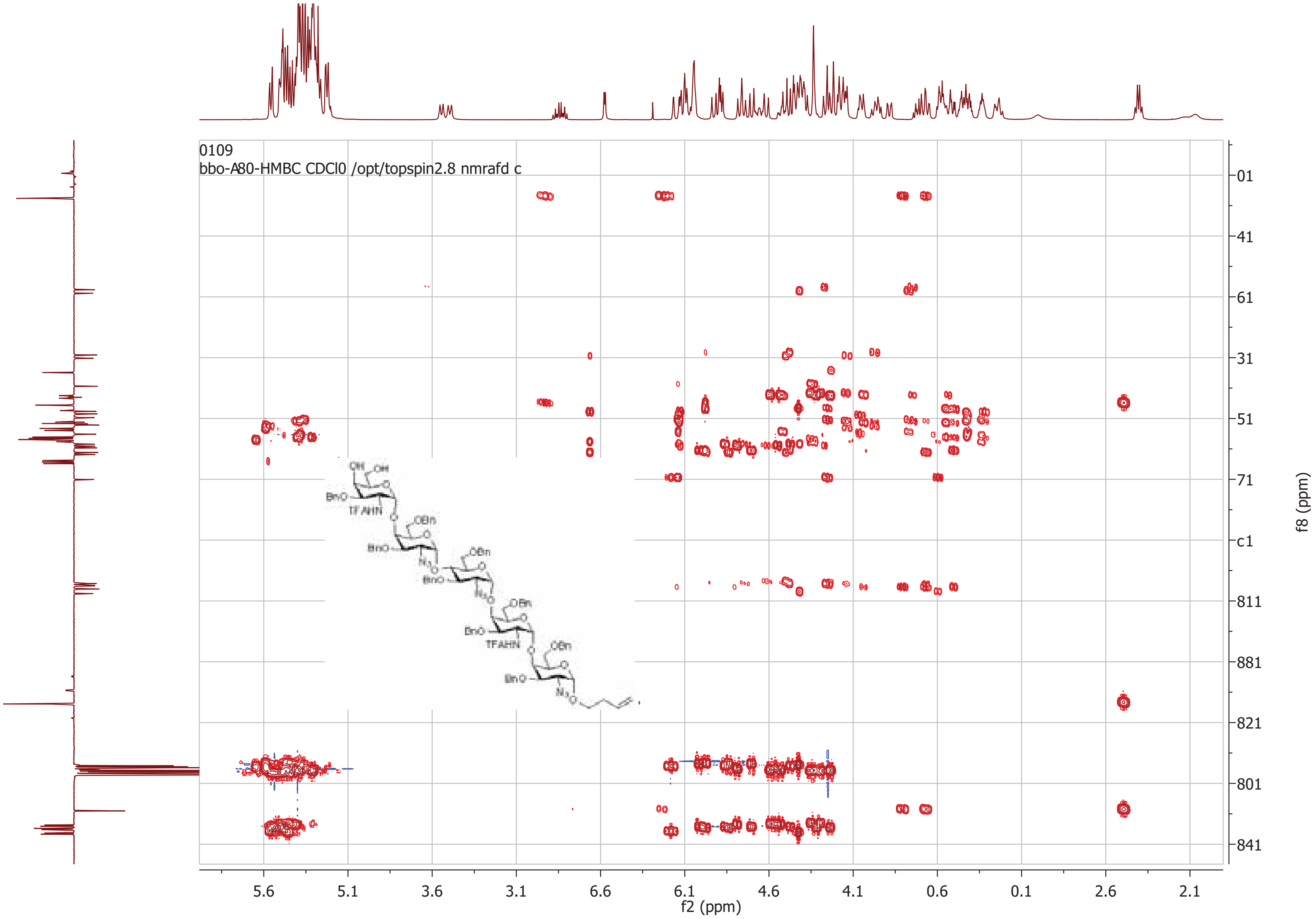
0109
bbo-A80-HMBC CDCl0 /opt/topspin2.8 nmrafd c
f2 (ppm)
f8 (ppm)

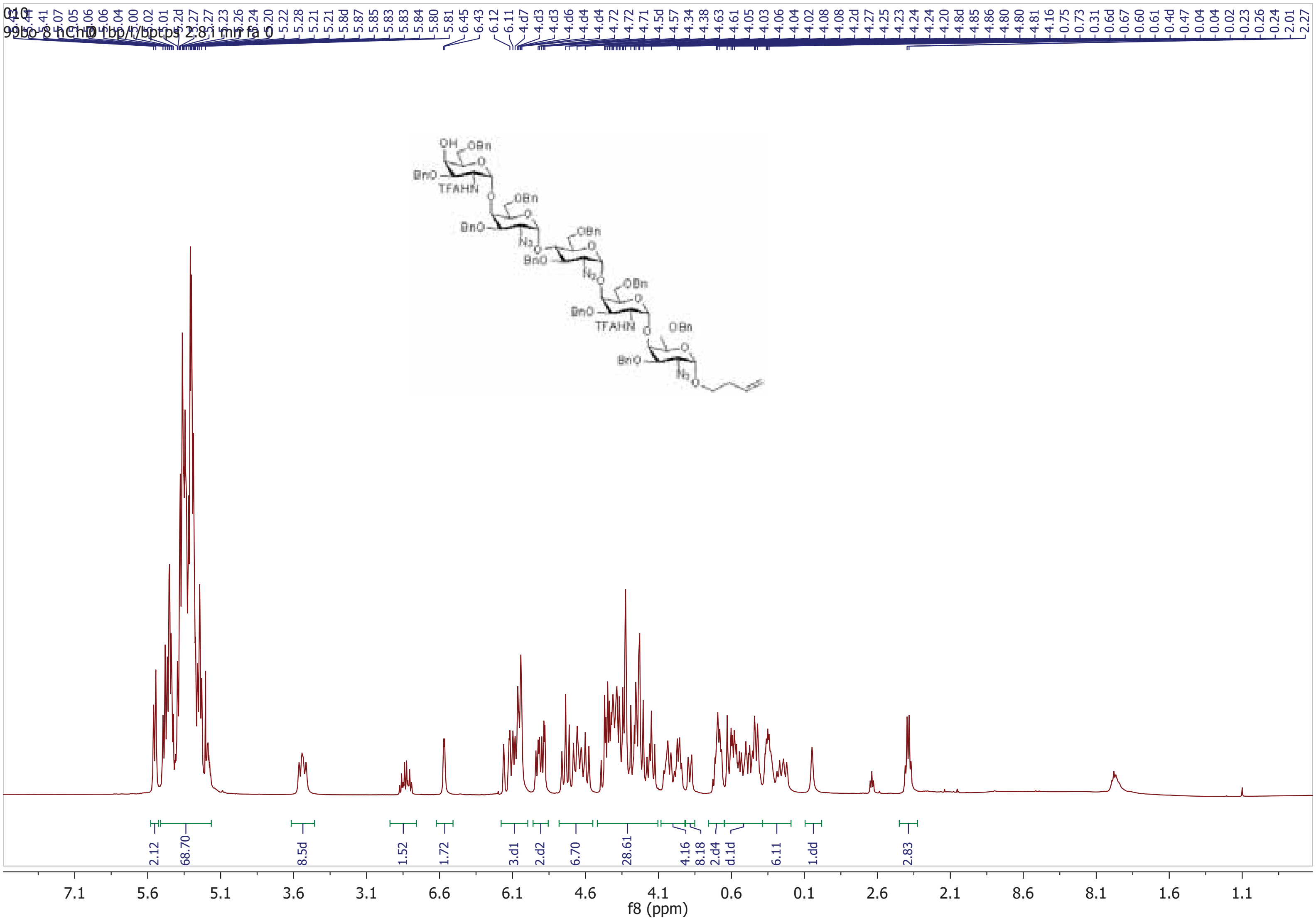

OH
OBn
BnO
TFAHN
N3
OBn
2.12
68.70
8.5d
1.52
1.72
3.d1
2.d2
6.70
28.61
4.16
8.18
2.d4
d.1d
6.11
1.dd
2.83
7.1
5.6
5.1
3.6
3.1
6.6
6.1
4.6
4.1
0.6
0.1
2.6
2.1
8.6
8.1
1.6
1.1
f8 (ppm)

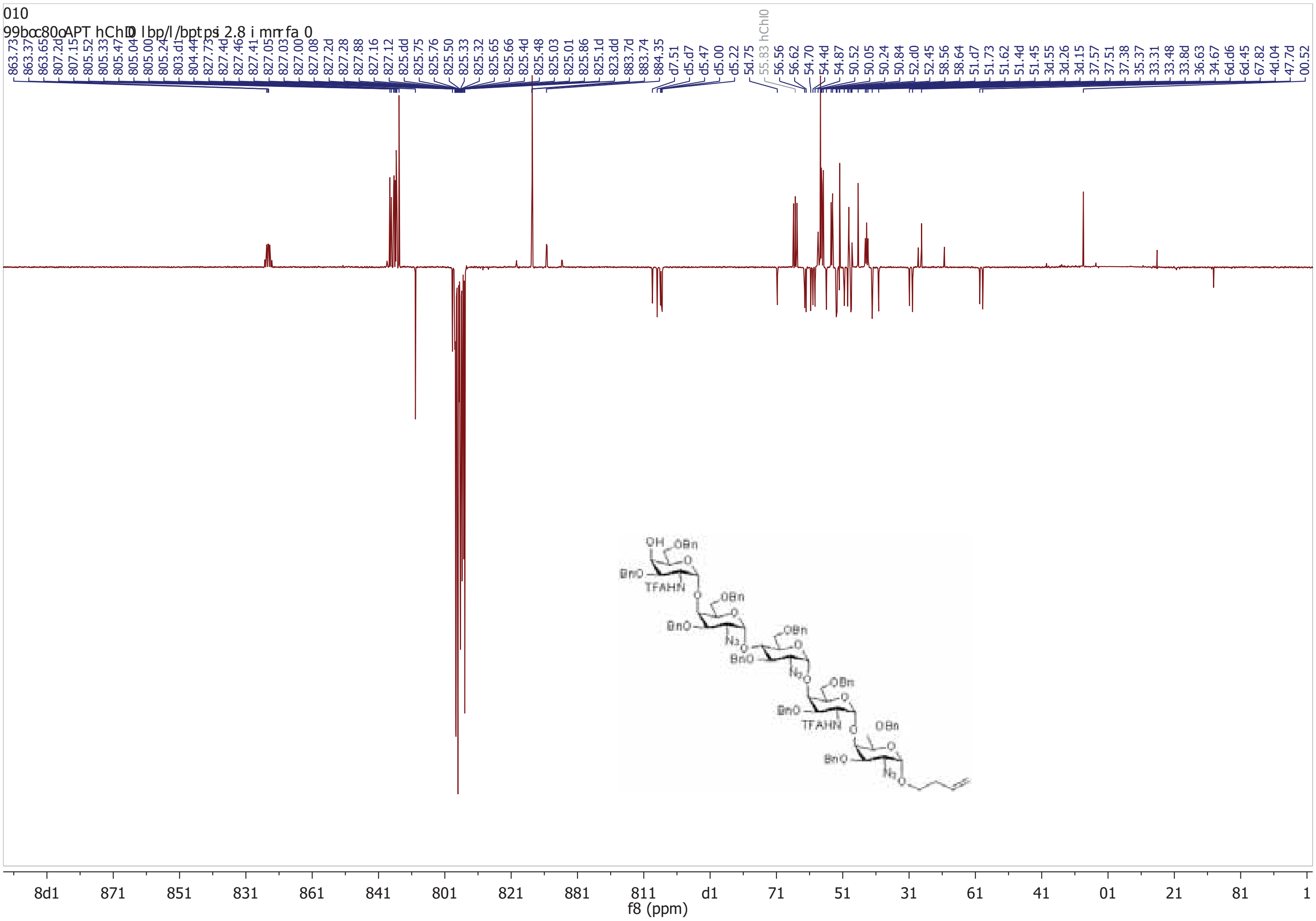

010
99bc c80c APT hChl0 lbp/l/bptps 2.8 i mrr fa 0
863.73 863.37 863.65 807.2d 807.15 805.52 805.33 805.47 805.04 805.00 805.24 803.d1 804.44 827.73 827.4d 827.46 827.41 827.05 827.03 827.00 827.08 827.2d 827.28 827.88 827.16 827.12 825.dd 825.75 825.76 825.50 825.33 825.32 825.65 825.66 825.4d 825.48 825.03 825.01 825.86 825.1d 823.dd 883.7d 883.74 884.35 d7.51 d5.d7 d5.47 d5.00 d5.22 5d.75 55.83 hChl0 56.56 56.62 54.70 54.4d 54.87 50.52 50.05 50.24 50.84 52.d0 52.45 58.56 58.64 51.d7 51.73 51.62 51.4d 51.45 3d.55 3d.26 3d.15 37.57 37.51 37.38 35.37 33.31 33.48 33.8d 36.63 34.67 6d.d6 6d.45 67.82 4d.04 47.7d 00.52
8d1
871
851
831
861
841
801
821
881
811
d1
71
51
31
61
41
01
21
81
1
f8 (ppm)
OH
OBn
BnO
TFAHN
O
OBn
BnO
N3
O
OBn
BnO
N3
O
OBn
BnO
TFAHN
O
OBn
BnO
N3
O

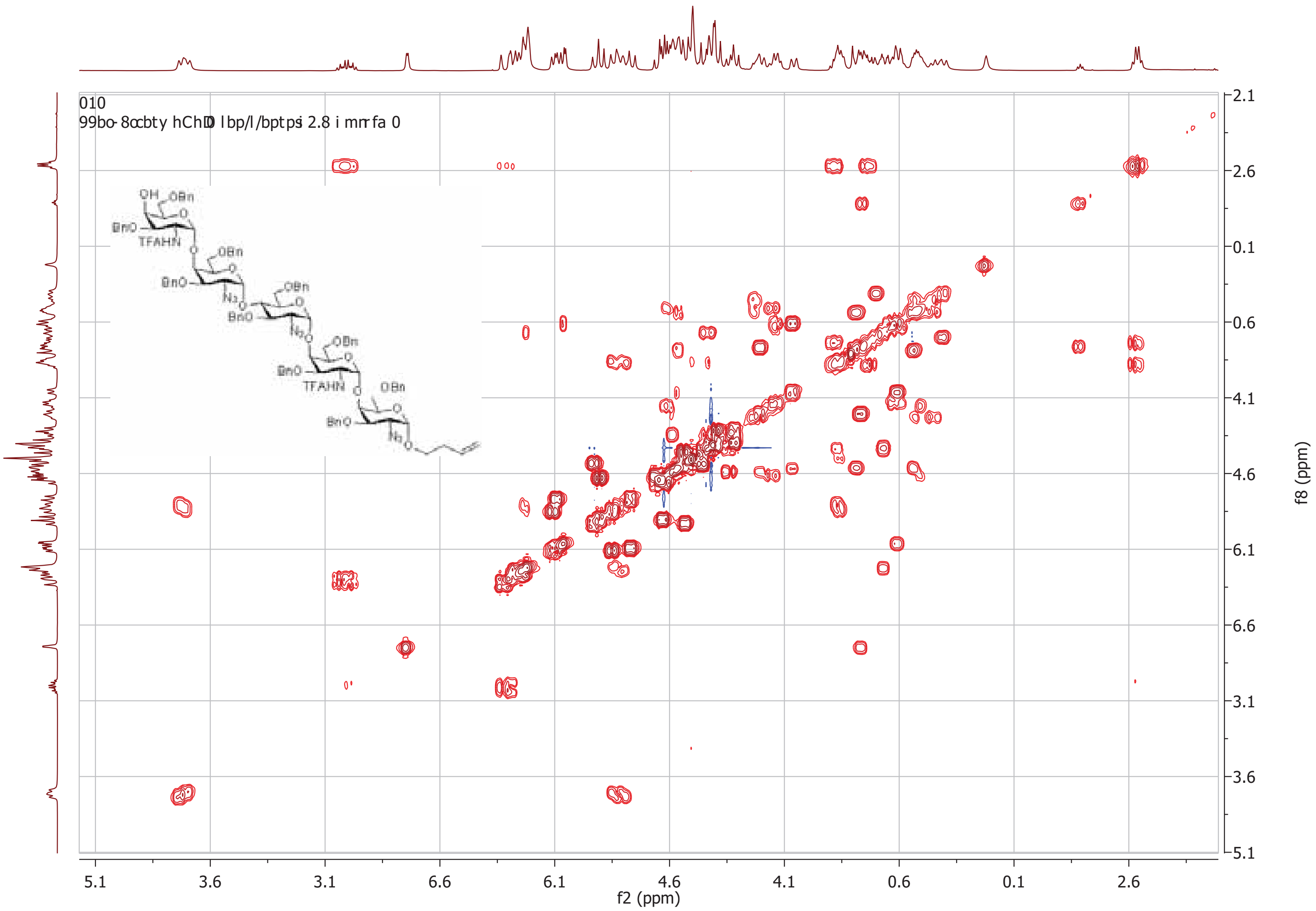
010
99bo-8ccbty hChD lbp/l/bptpsi 2.8 i mrrfa 0
OH
OBn
BnO
TFAHN
N3
TFAHN
f2 (ppm)
f8 (ppm)

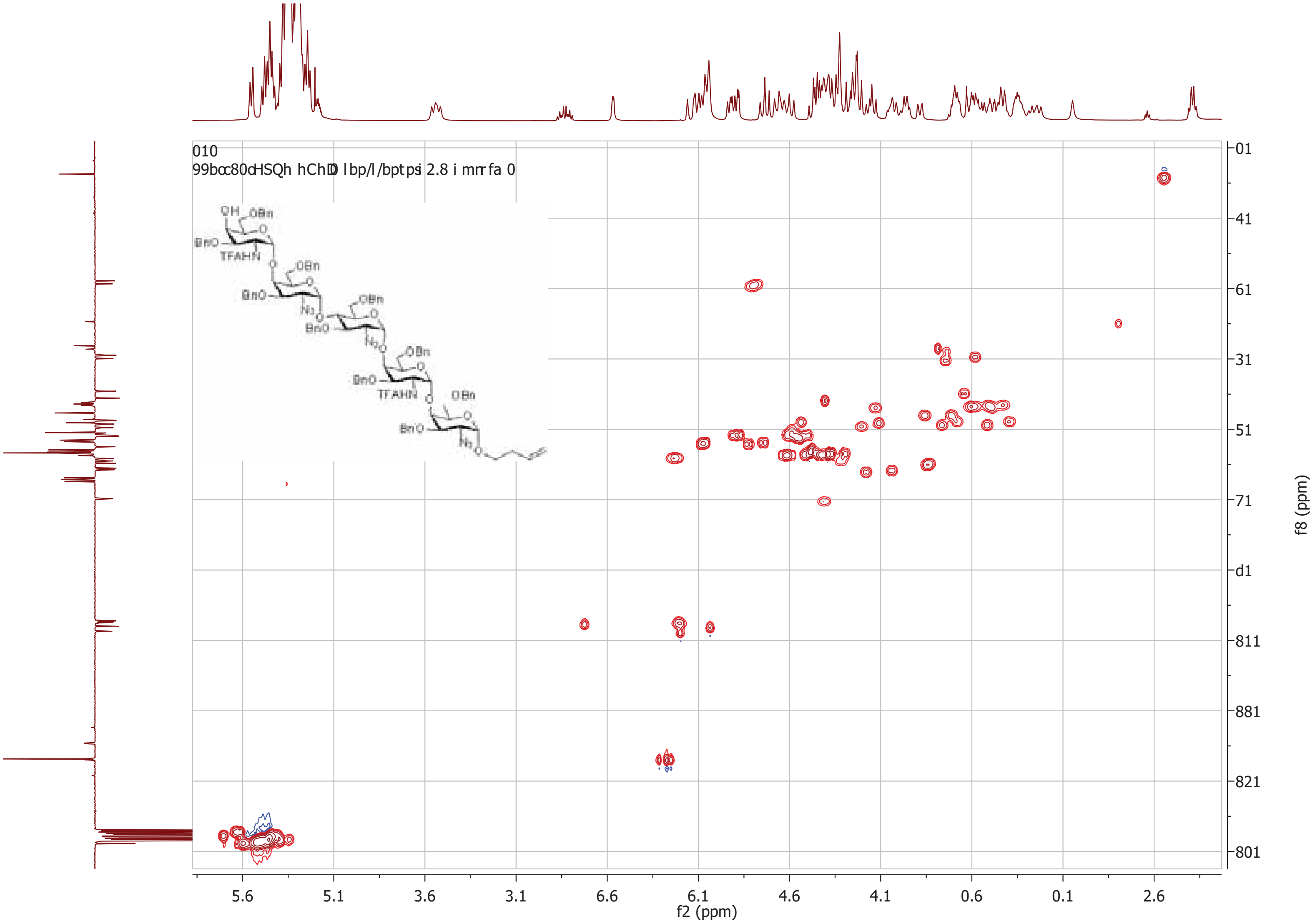

010
OH
OBn
BnO
TFAHN
N3
TFAHN
f2 (ppm)
f8 (ppm)

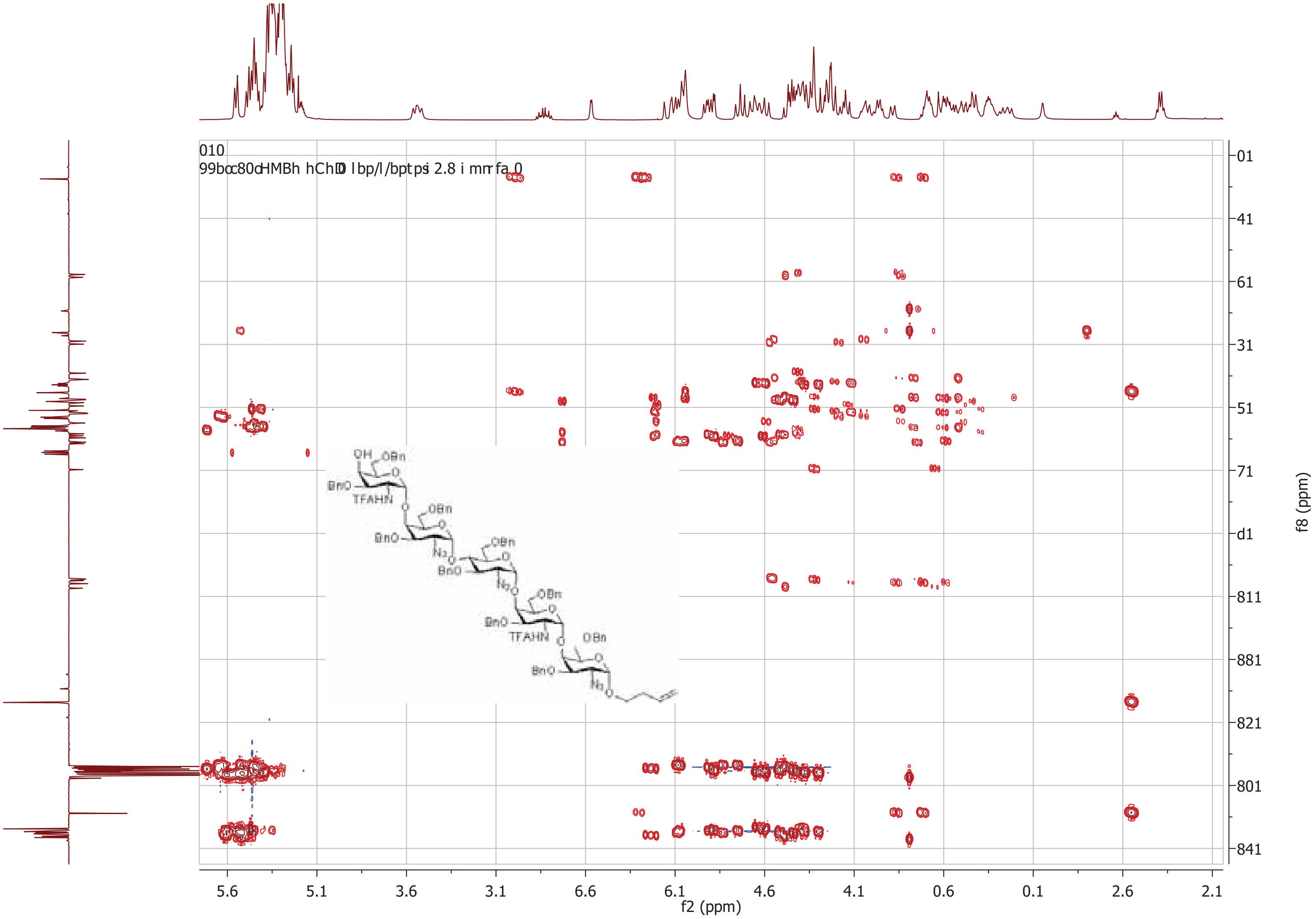
OH
OBn
BnO
TFAHN
N3
f2 (ppm)

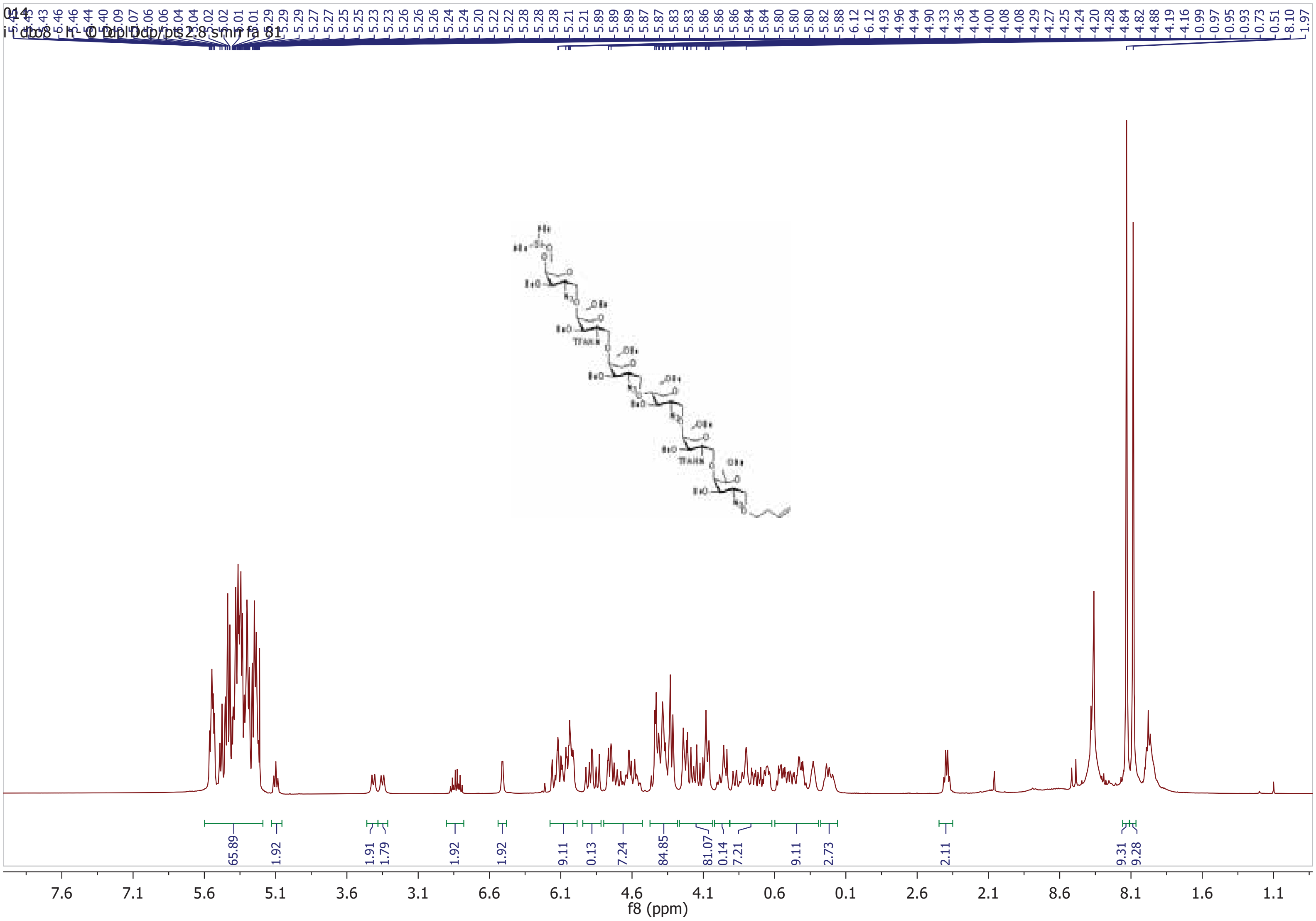
7.6 7.1 5.6 5.1 3.6 3.1 6.6 6.1 4.6 4.1 0.6 0.1 2.6 2.1 8.6 8.1 1.6 1.1
f8 (ppm)

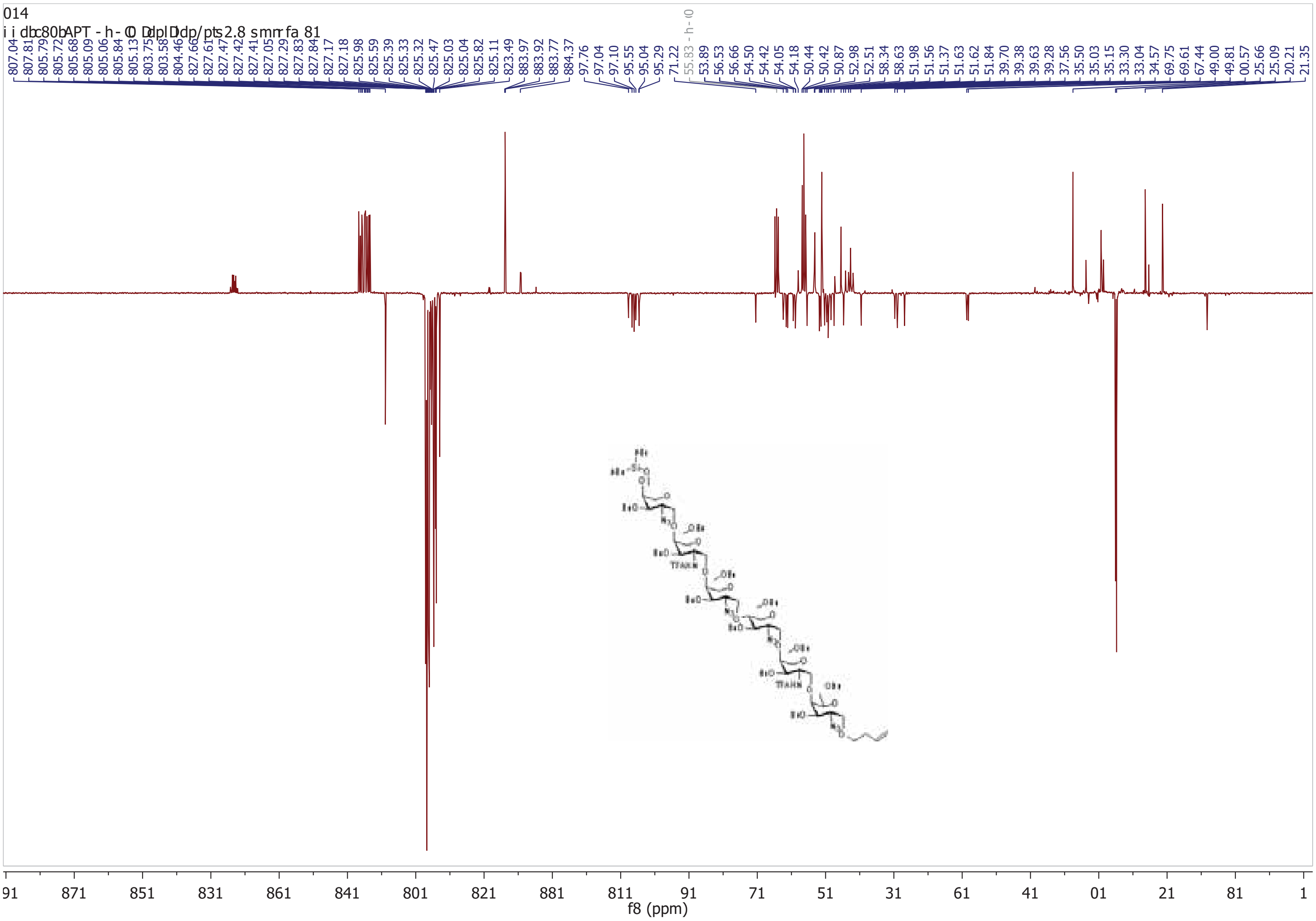
014
i i dbc80bAPT - h- Ó Ddpl Ddp/pts2.8 smrr fa 81
807.04 807.81 805.79 805.72 805.68 805.09 805.06 805.84 805.13 803.75 803.58 804.46 827.66 827.61 827.47 827.42 827.41 827.05 827.29 827.83 827.84 827.17 827.18 825.98 825.59 825.39 825.33 825.32 825.47 825.03 825.04 825.82 825.11 823.49 883.97 883.92 883.77 884.37 97.76 97.04 97.10 95.55 95.04 95.29 71.22 55.83 - h- i0 53.89 56.53 56.66 54.50 54.42 54.05 54.18 50.44 50.42 50.87 52.98 52.51 58.34 58.63 51.98 51.56 51.37 51.63 51.62 51.84 39.70 39.38 39.63 39.28 37.56 35.50 35.03 35.15 33.30 33.04 34.57 69.75 69.61 67.44 49.00 49.81 00.57 25.66 25.09 20.21 21.35
91 871 851 831 861 841 801 821 881 811 91 71 51 31 61 41 01 21 81 1
f8 (ppm)

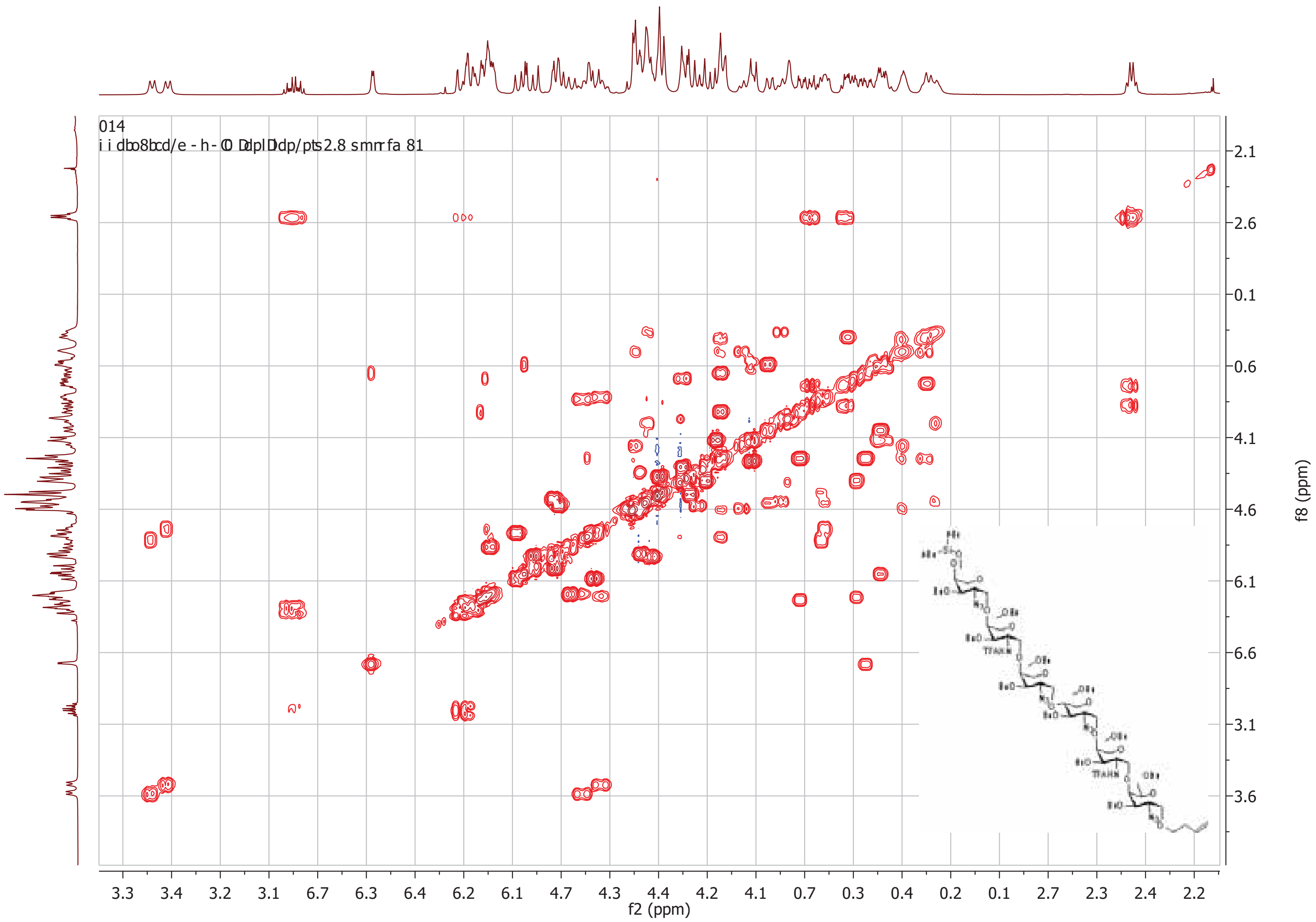
014
f2 (ppm)
f8 (ppm)

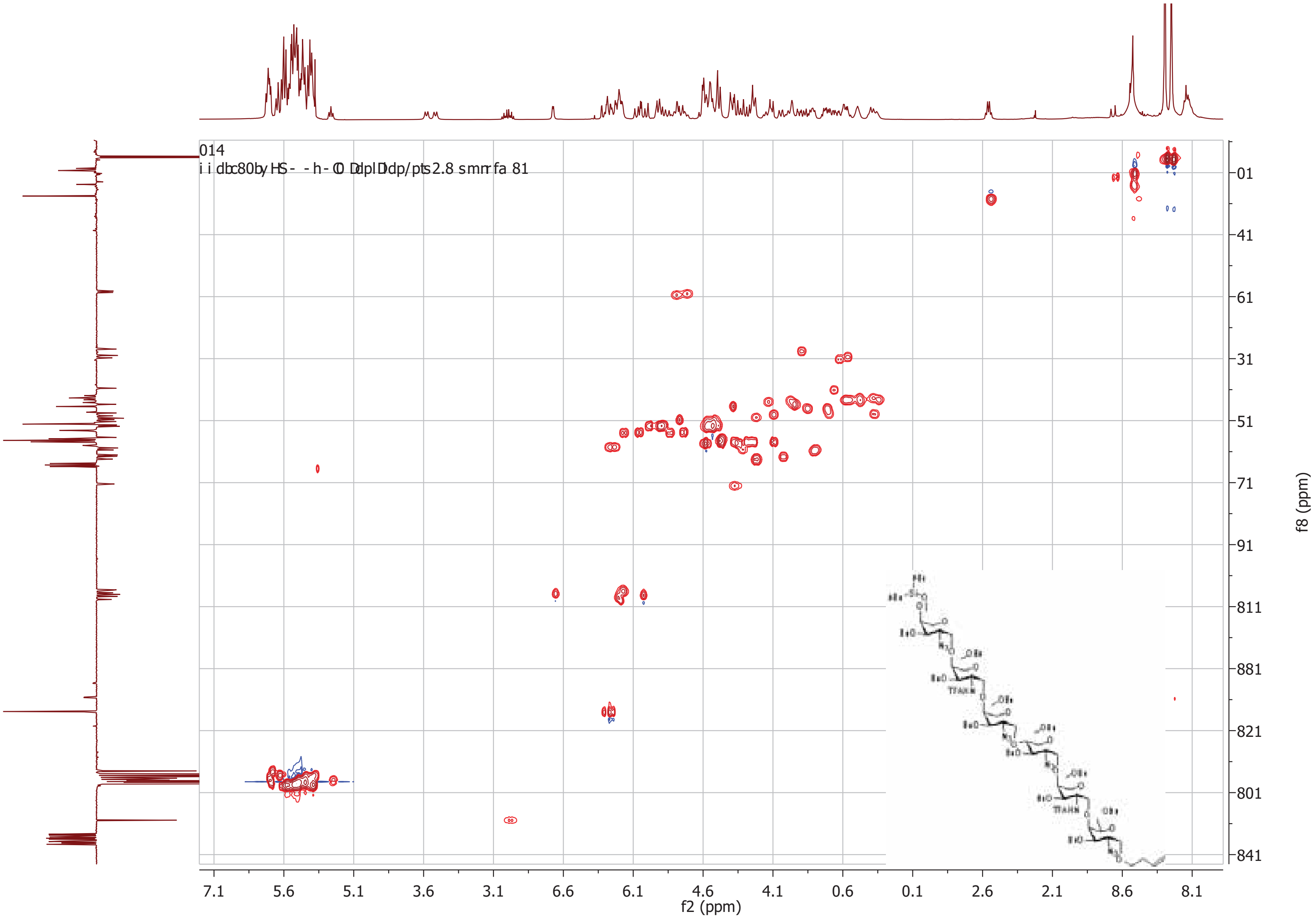
014
f2 (ppm)
f8 (ppm)

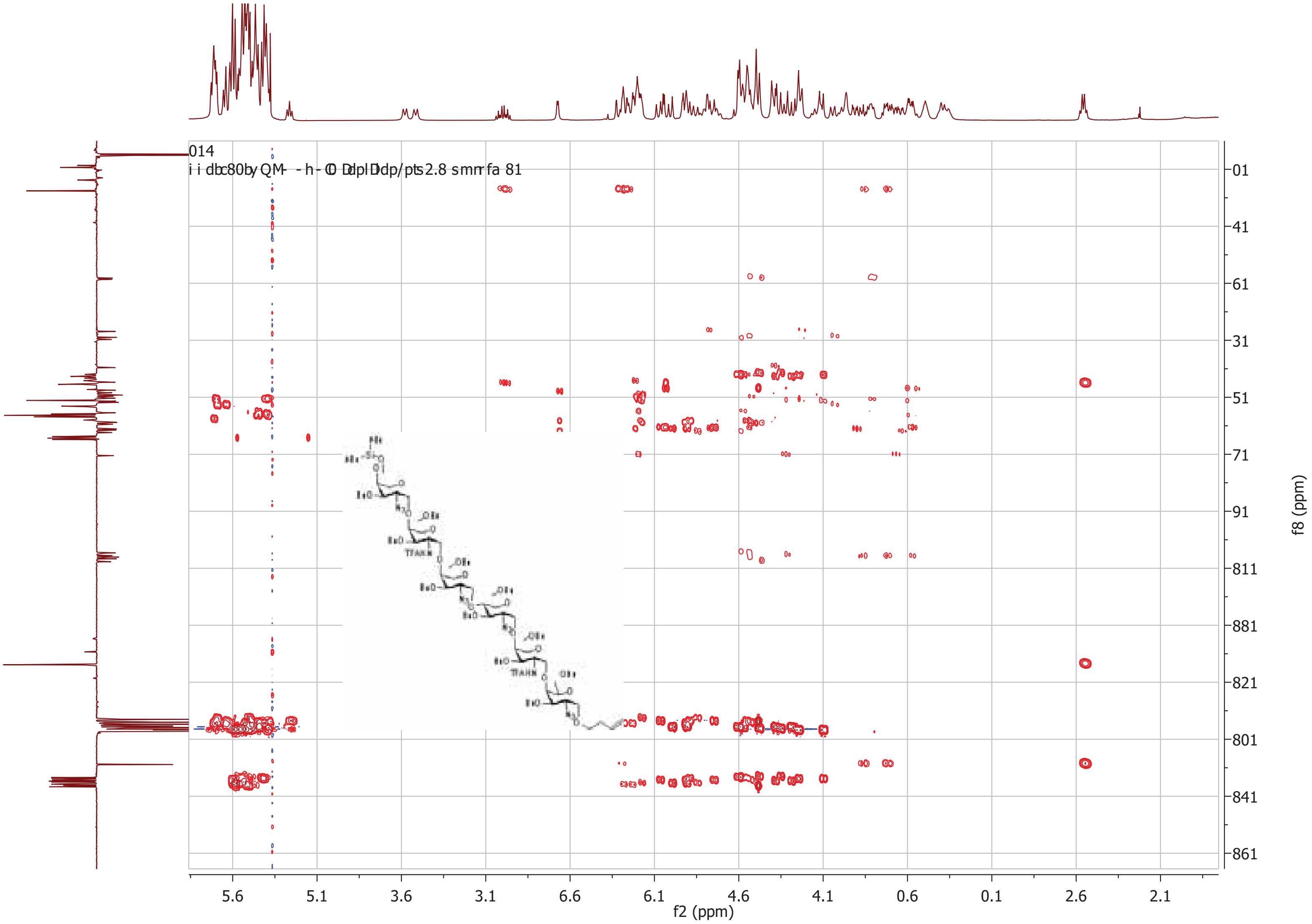
f2 (ppm)
f8 (ppm)

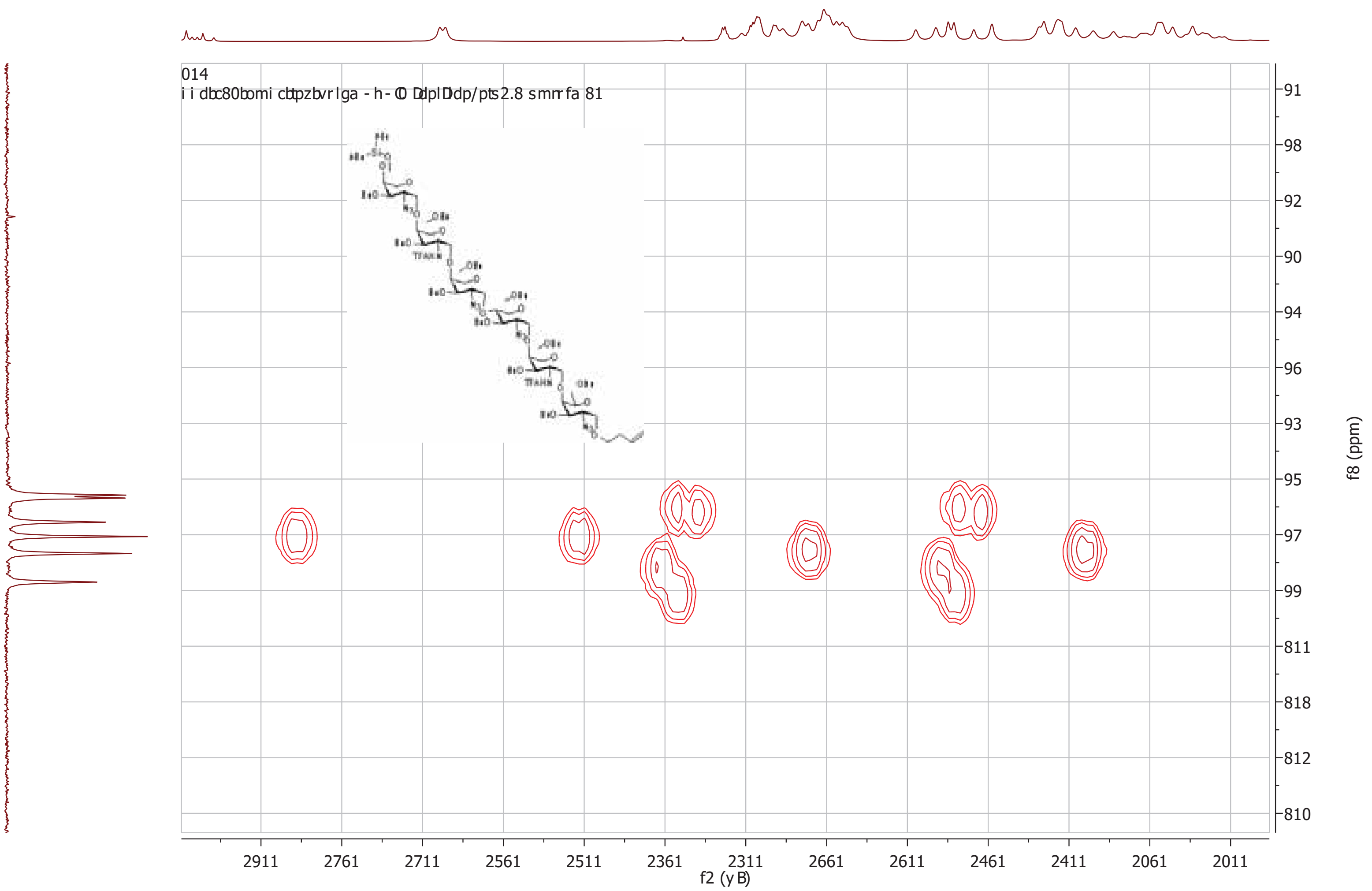
014
f2 (y B)
f8 (ppm)

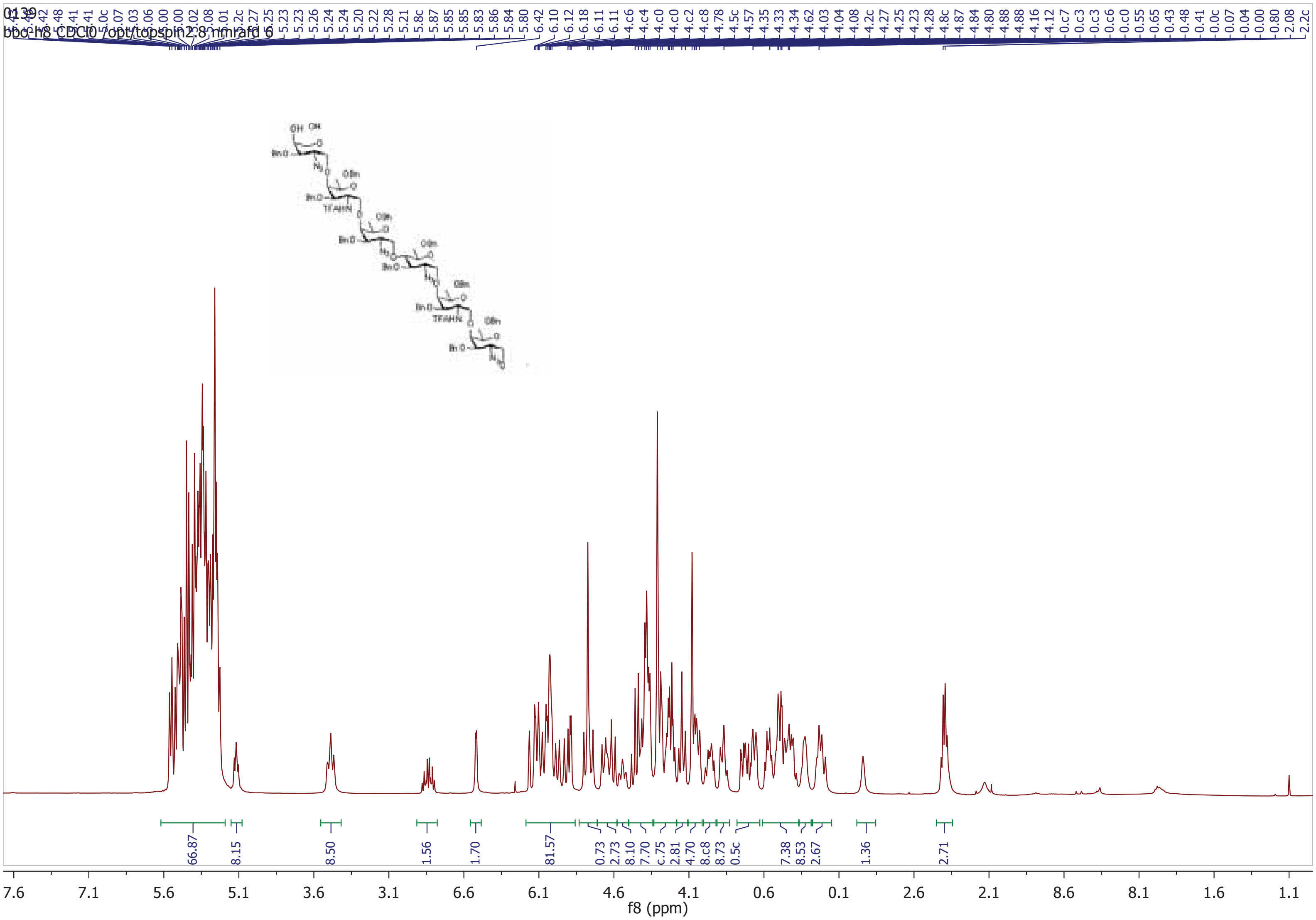
0139
bbo-h8 CDCl0 /opt/topspin2.8 nmrafd 6
TFAHN
f8 (ppm)

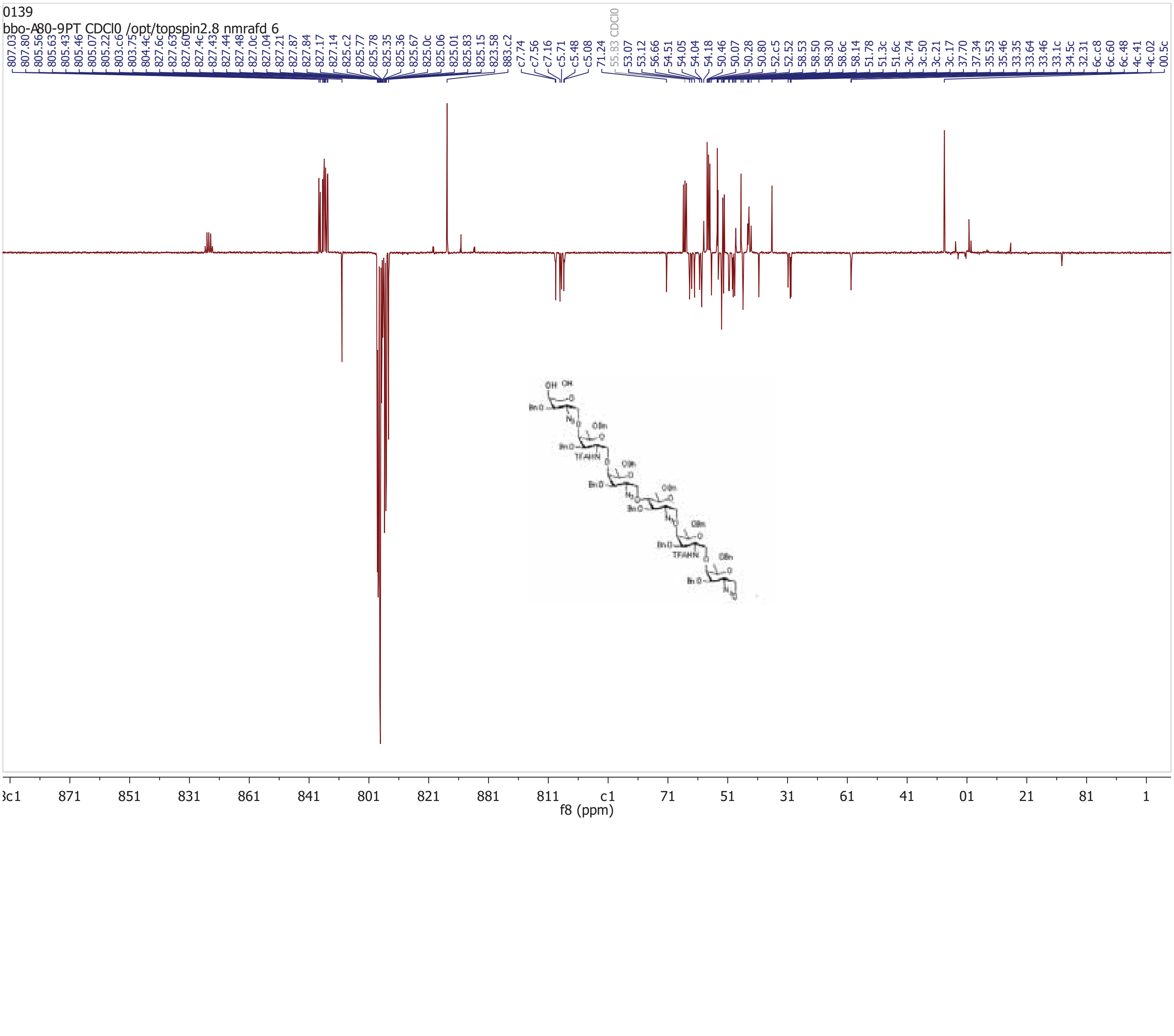
0139
bbo-A80-9PT CDCl0 /opt/topspin2.8 nmrafd 6
f8 (ppm)

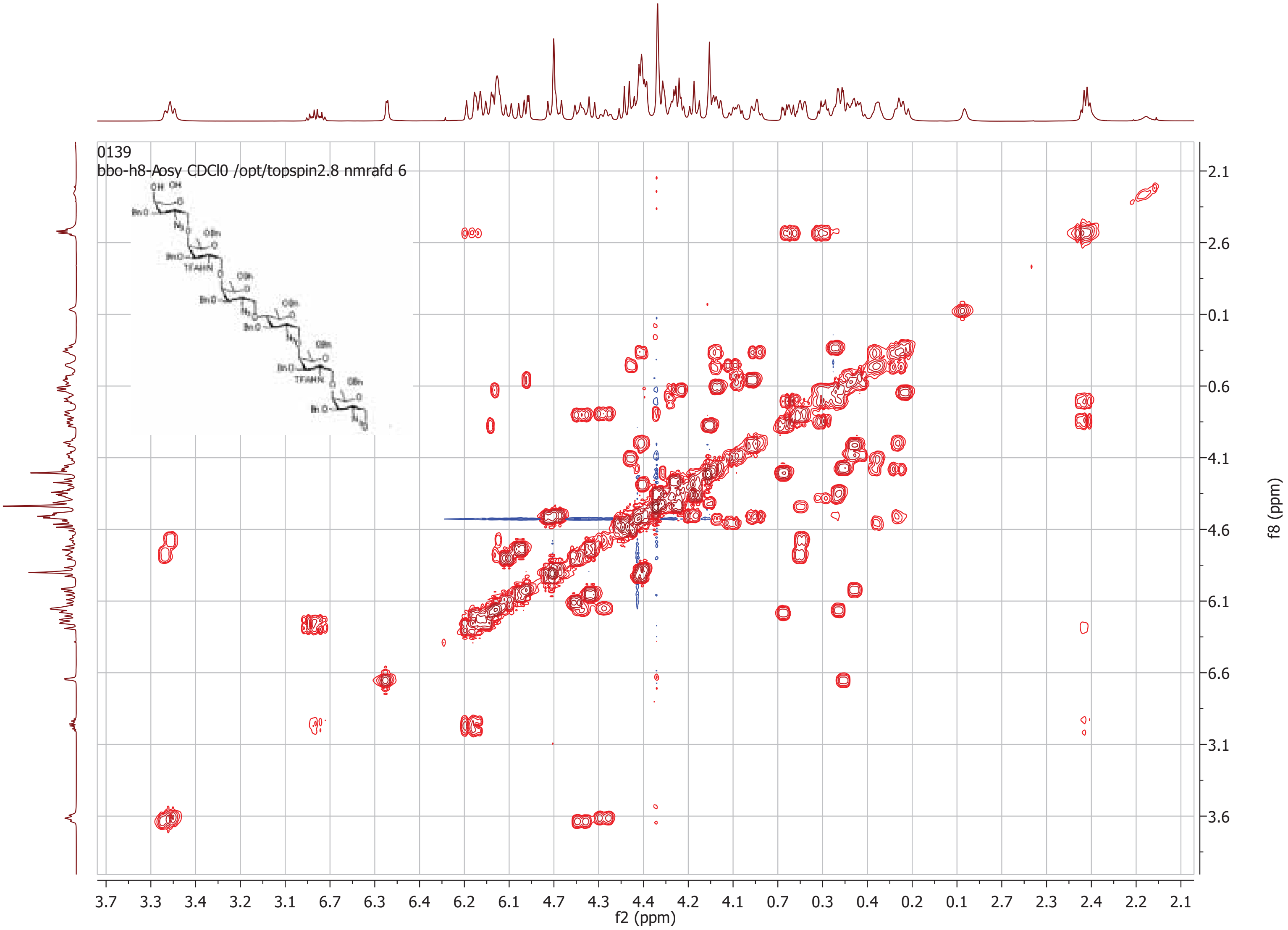
0139
bbo-h8-Aosy CDCl0 /opt/topspin2.8 nmrafd 6
f2 (ppm)
f8 (ppm)

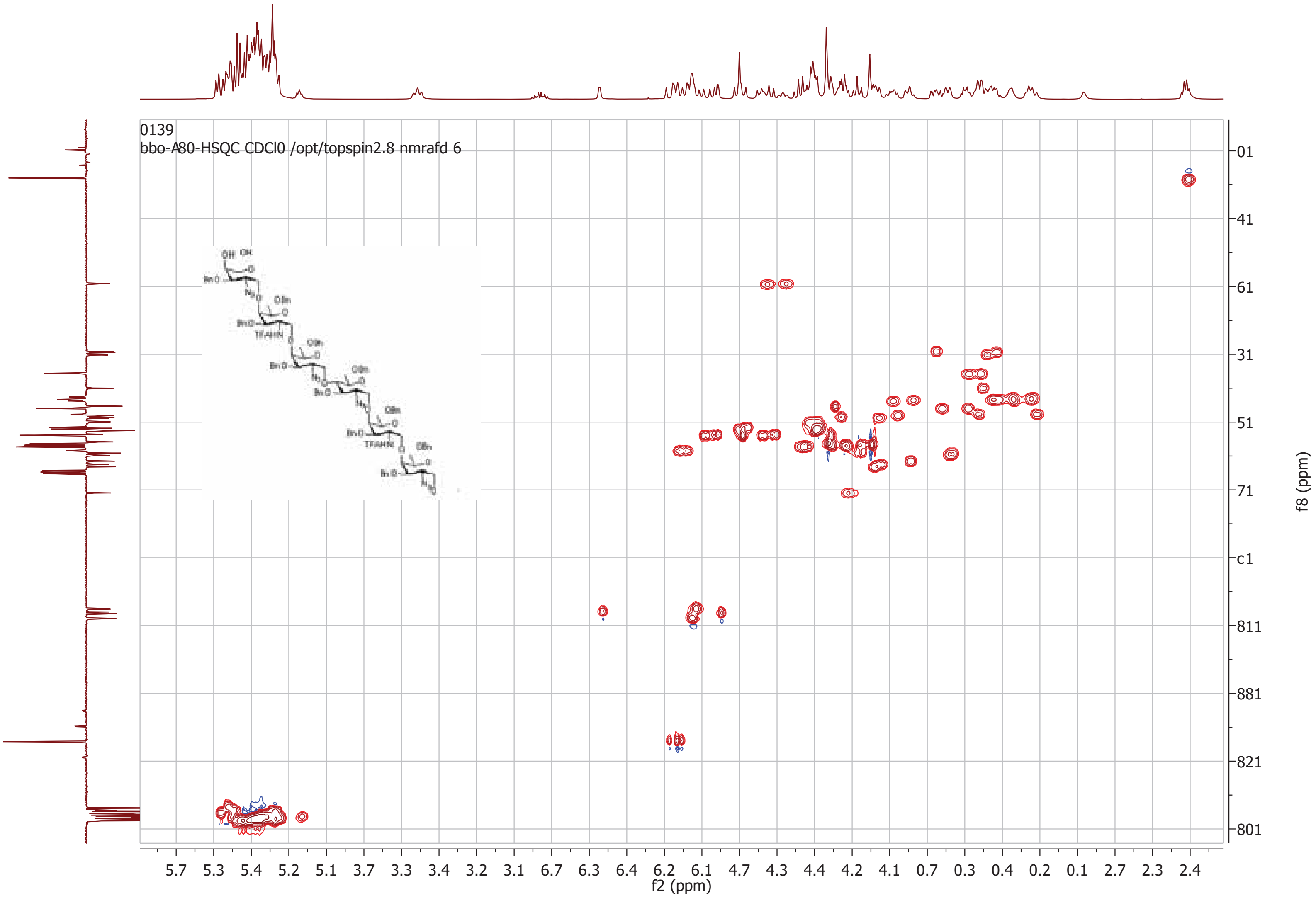
0139
bbo-A80-HSQC CDCl0 /opt/topspin2.8 nmrafd 6
5.7 5.3 5.4 5.2 5.1 3.7 3.3 3.4 3.2 3.1 6.7 6.3 6.4 6.2 6.1 4.7 4.3 4.4 4.2 4.1 0.7 0.3 0.4 0.2 0.1 2.7 2.3 2.4
f2 (ppm)
01 41 61 31 51 71 c1 811 881 821 801
f8 (ppm)

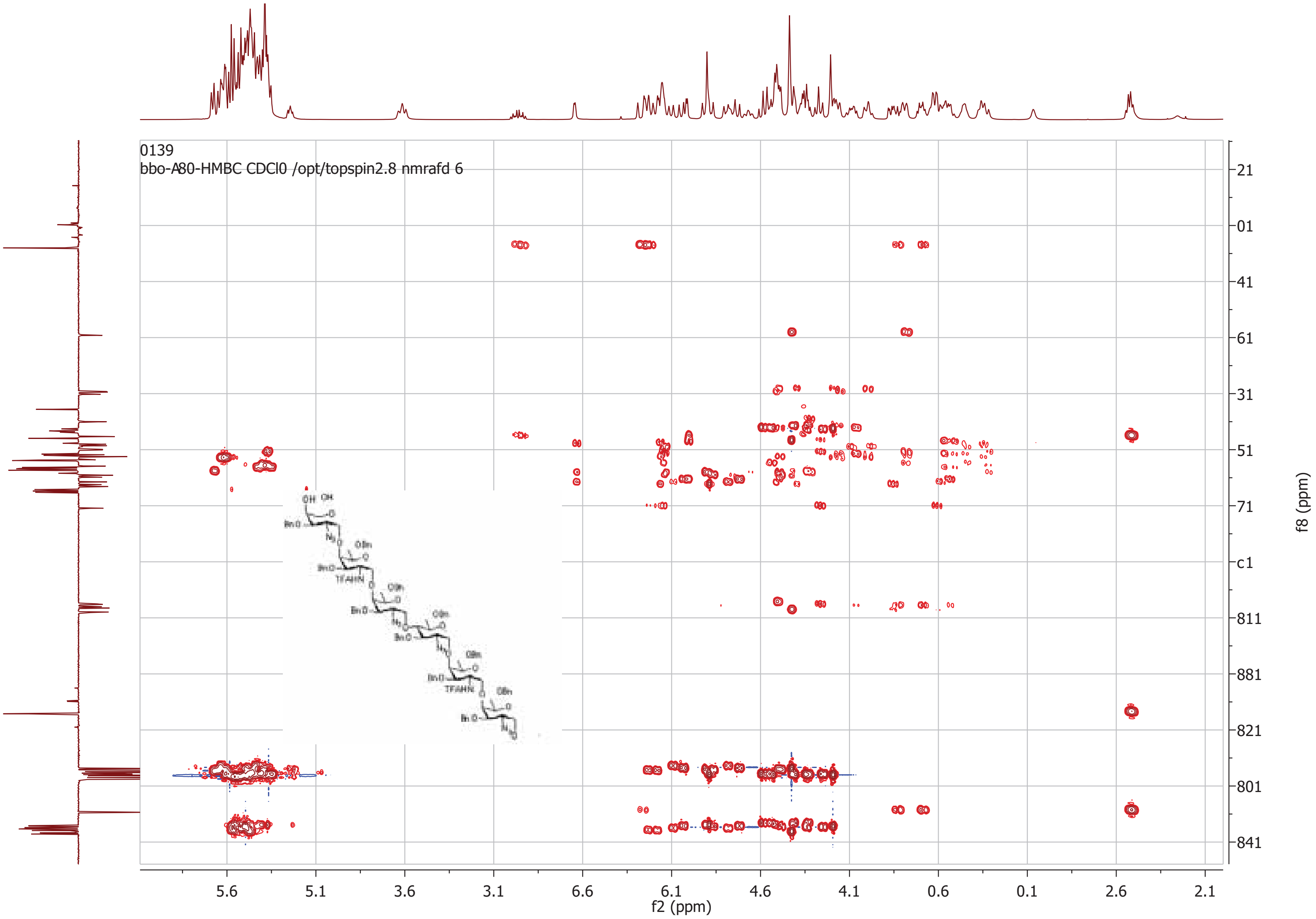
0139
bbo-A80-HMBC CDCl0 /opt/topspin2.8 nmrafd 6
f2 (ppm)
f8 (ppm)

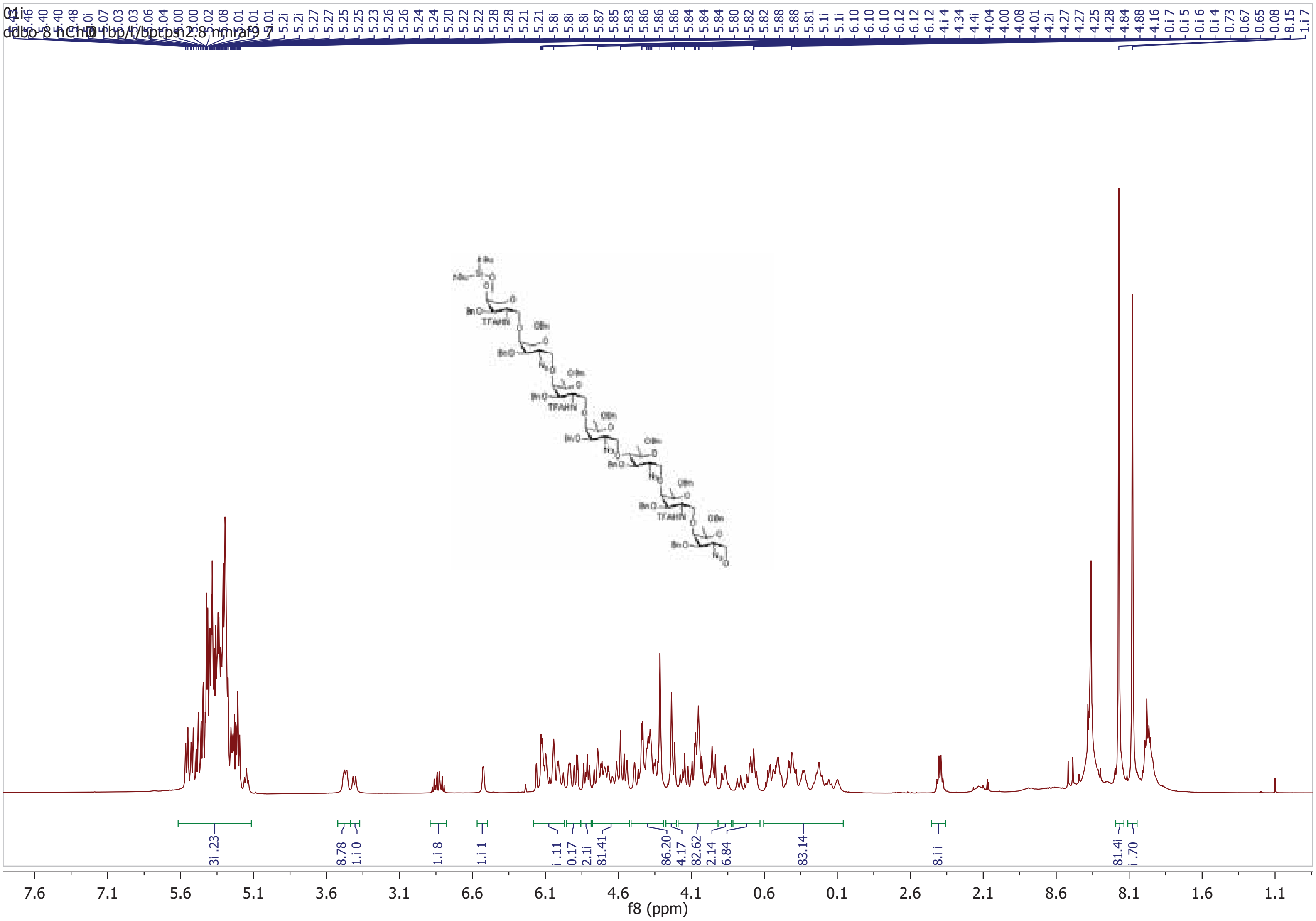
7.6 7.1 5.6 5.1 3.6 3.1 6.6 6.1 4.6 4.1 0.6 0.1 2.6 2.1 8.6 8.1 1.6 1.1
f8 (ppm)

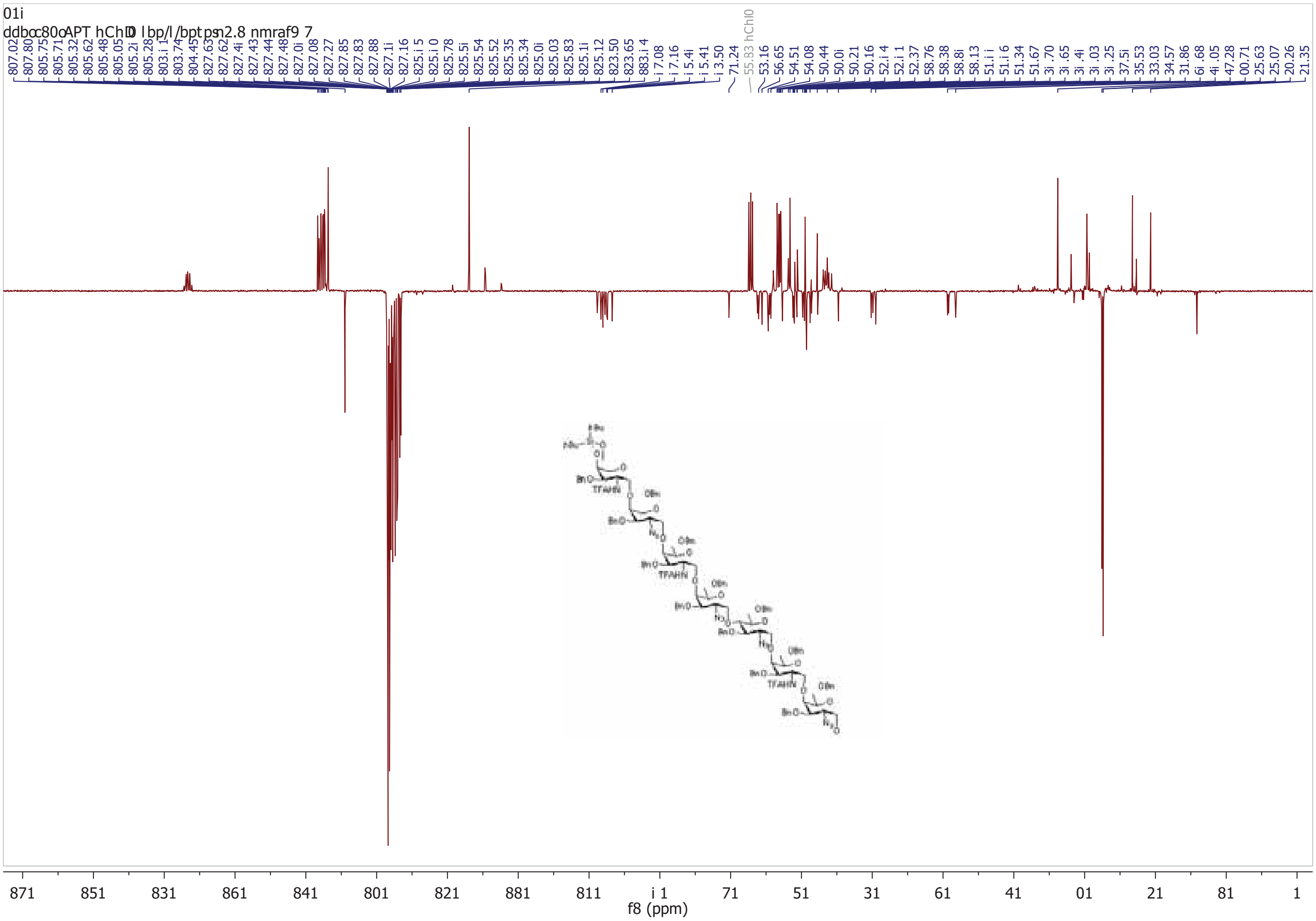
01i
ddbcc80oAPT hChl0 lbp/l/bptps n2.8 nmraf9 7
f8 (ppm)

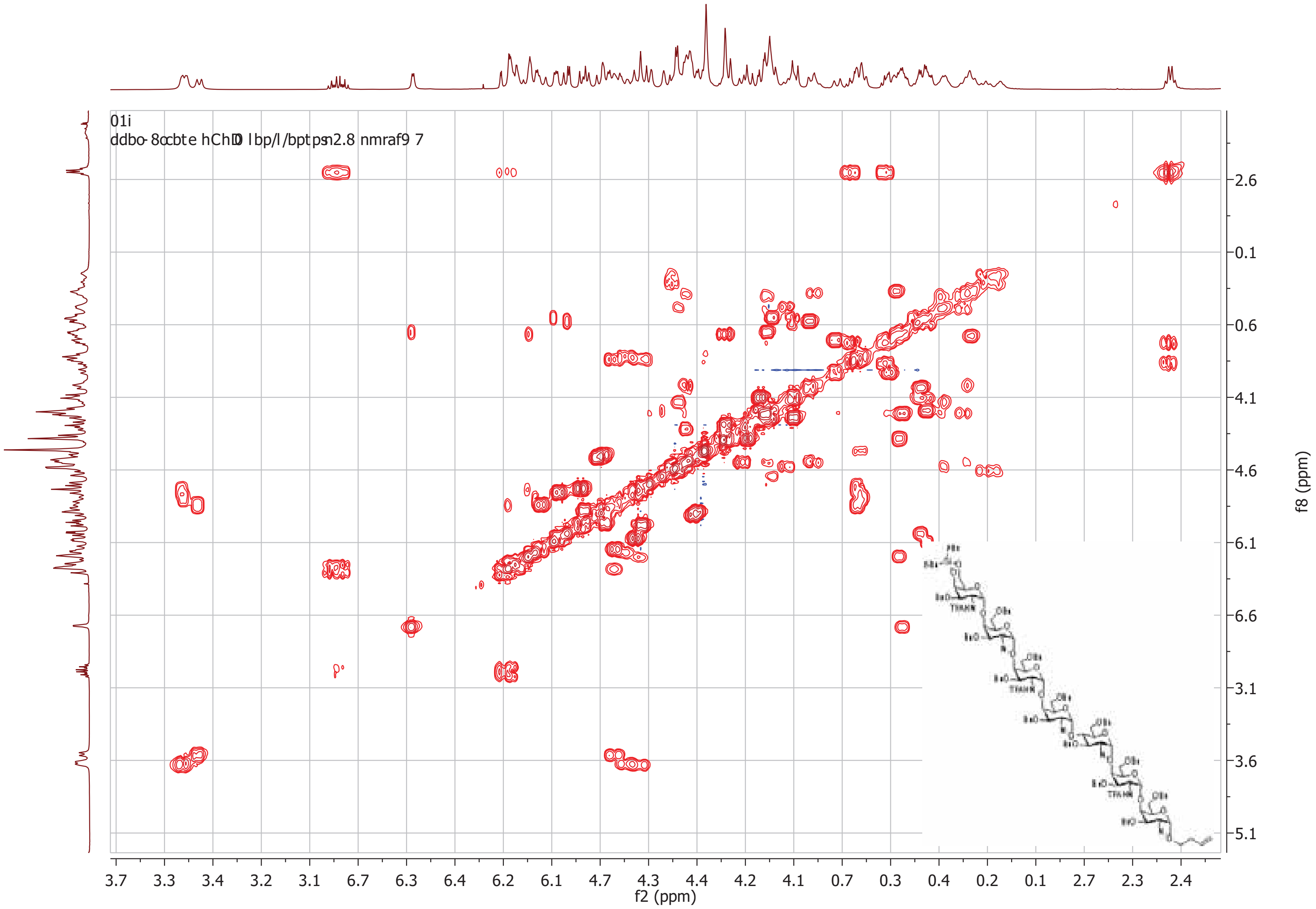
01i
f2 (ppm)
f8 (ppm)

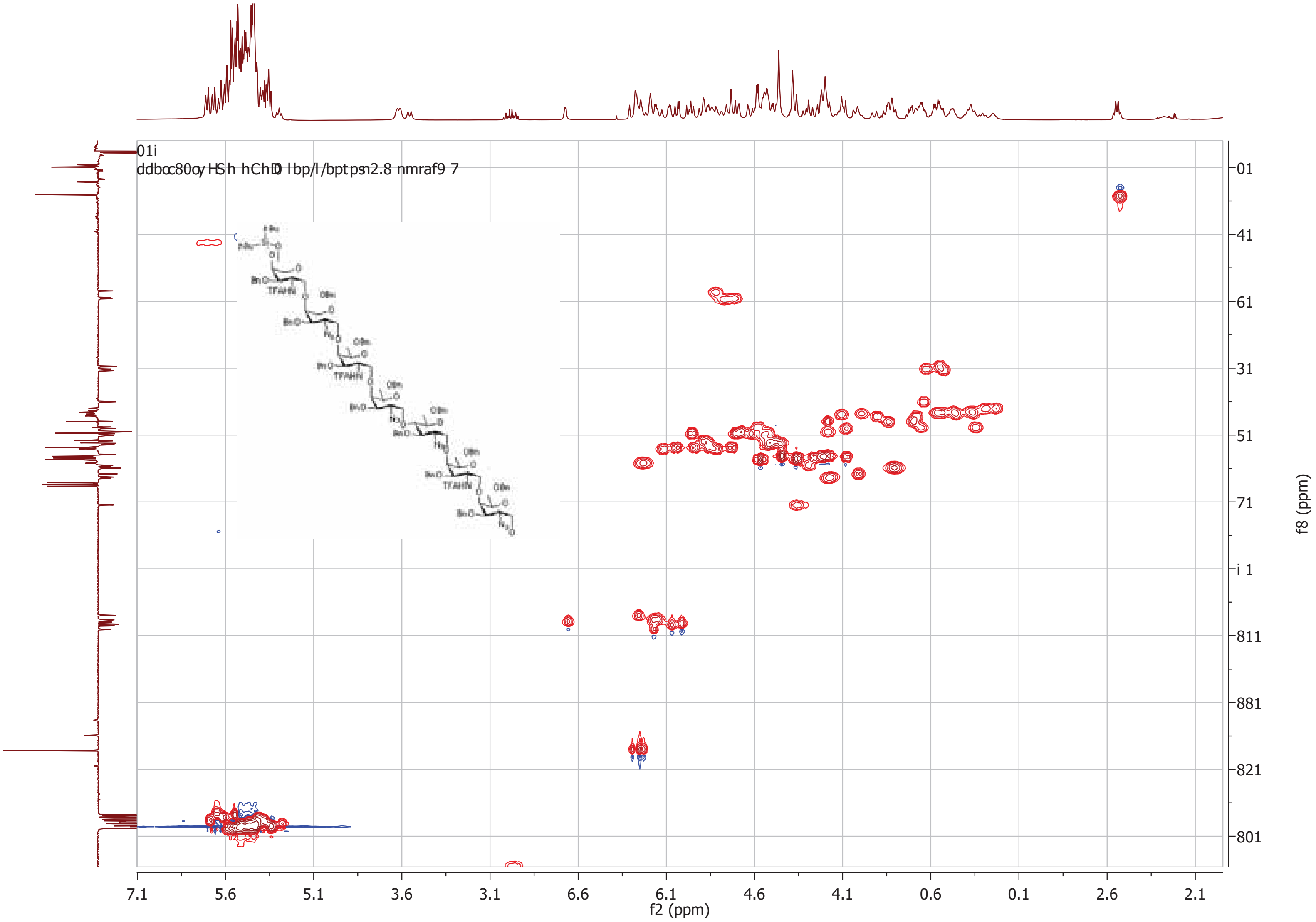
01i
ddbcc80oy HS h hChD lbp/l/bptpsn2.8 nmraf9 7
f2 (ppm)
f8 (ppm)

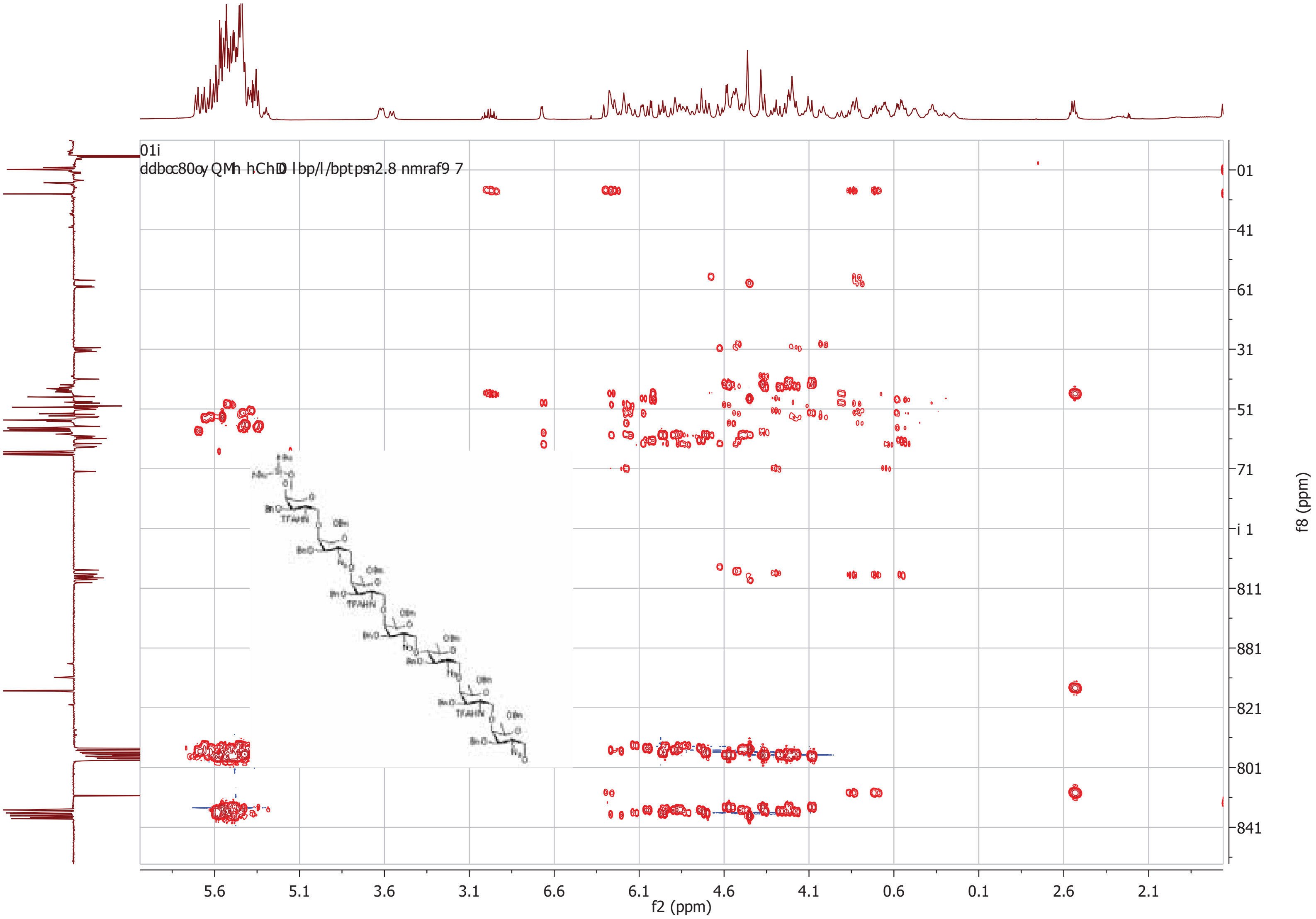
01i
ddbcc80oy QMn hChD lbp/l/bptpsn2.8 nmraf9 7
f2 (ppm)
f8 (ppm)

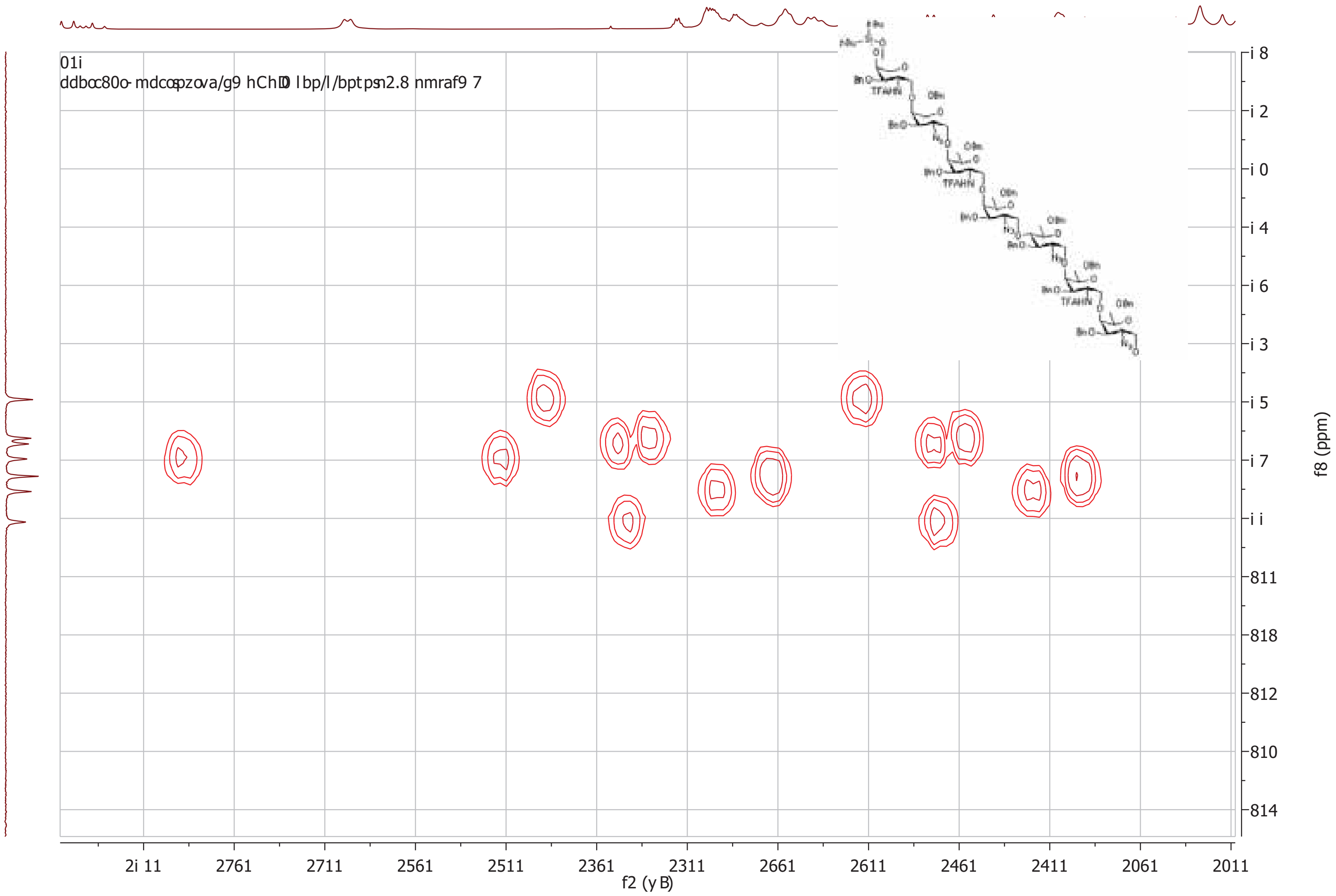
01i
ddbcc80o-mdcospzova/g9 hChlD lbp/l/bptpsn2.8 nmraf9 7
f2 (y B)
f8 (ppm)
TFAHN
BnO
OBn

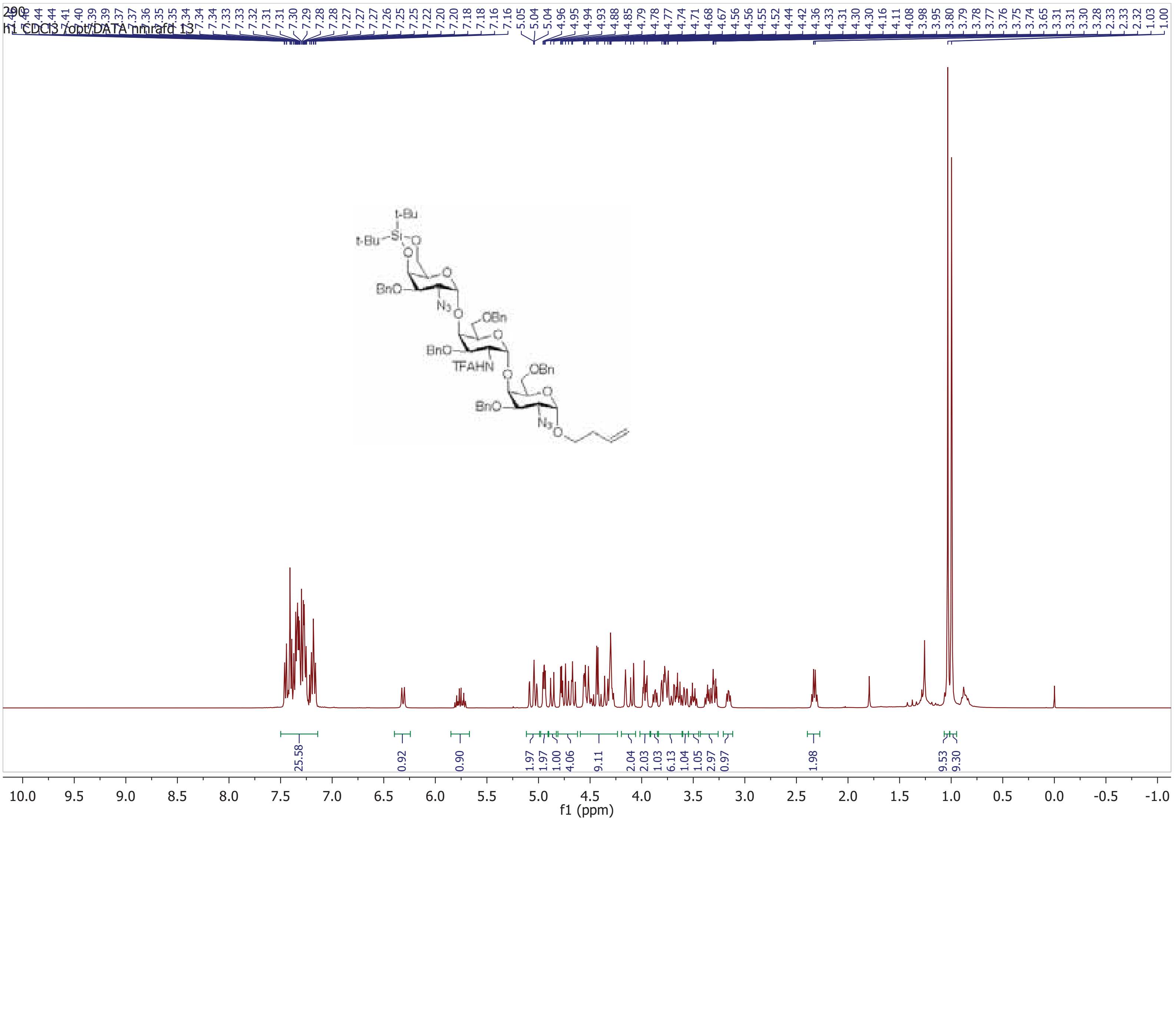
290
h1 CDCl3 /opt/DATA nmrafd 13
t-Bu
t-Bu
Si
BnO
N3
OBn
BnO
TFAHN
OBn
BnO
N3
25.58
0.92
0.90
1.97
1.97
1.00
4.06
9.11
2.04
2.03
1.03
6.13
1.04
1.05
2.97
0.97
1.98
9.53
9.30
f1 (ppm)

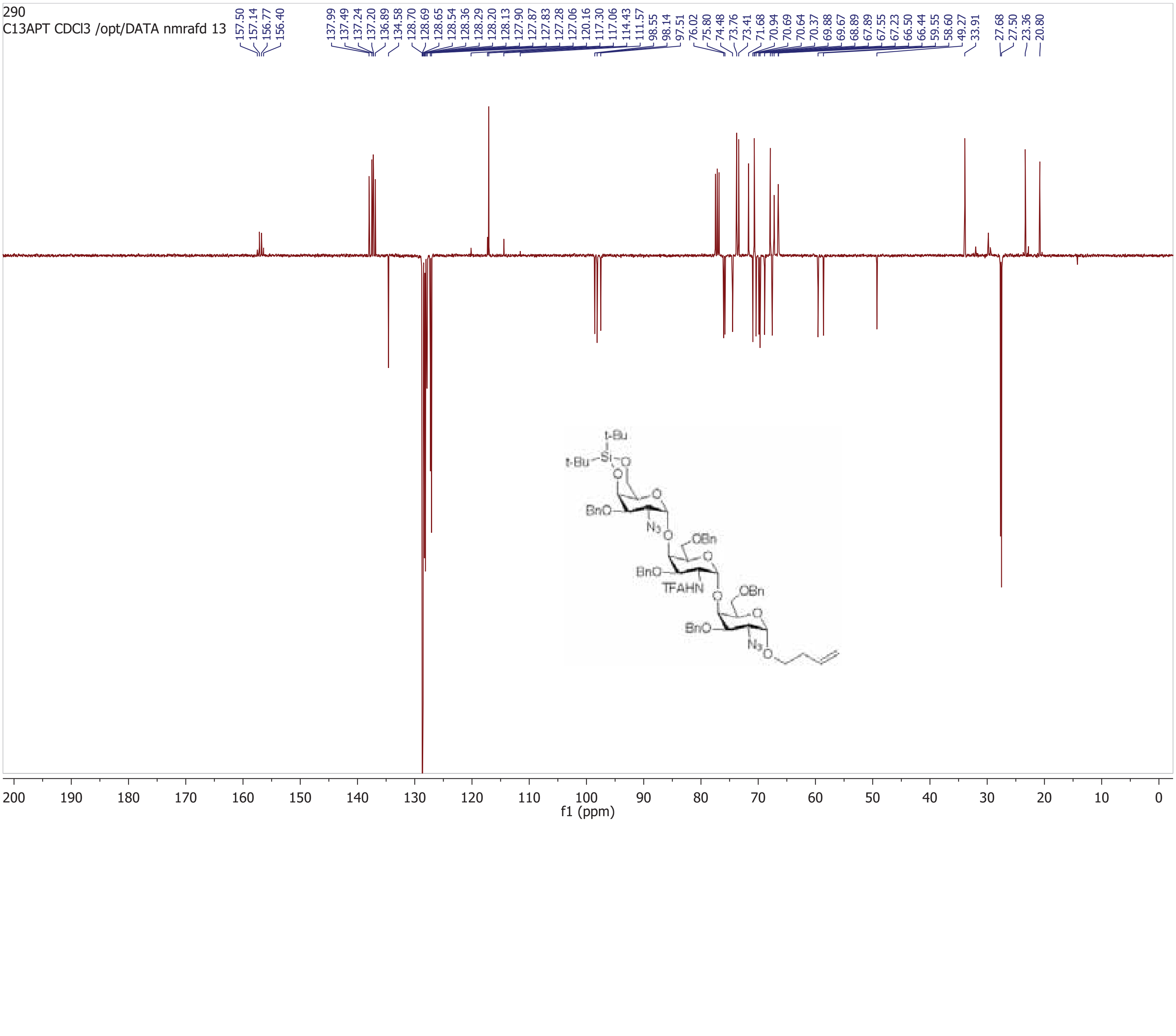
290
C13APT CDCl3 /opt/DATA nmrafd 13
157.50
157.14
156.77
156.40
137.99
137.49
137.24
137.20
136.89
134.58
128.70
128.69
128.65
128.54
128.36
128.29
128.20
128.13
127.90
127.87
127.83
127.28
127.06
120.16
117.30
117.06
114.43
111.57
98.55
98.14
97.51
76.02
75.80
74.48
73.76
73.41
71.68
70.94
70.69
70.64
70.37
69.88
69.67
68.89
67.89
67.55
67.23
66.50
66.44
59.55
58.60
49.27
33.91
27.68
27.50
23.36
20.80
t-Bu
t-Bu
Si
O
O
O
BnO
N3
O
OBn
O
BnO
TFAHN
O
OBn
O
BnO
N3
O
200
190
180
170
160
150
140
130
120
110
100
90
80
70
60
50
40
30
20
10
0
f1 (ppm)

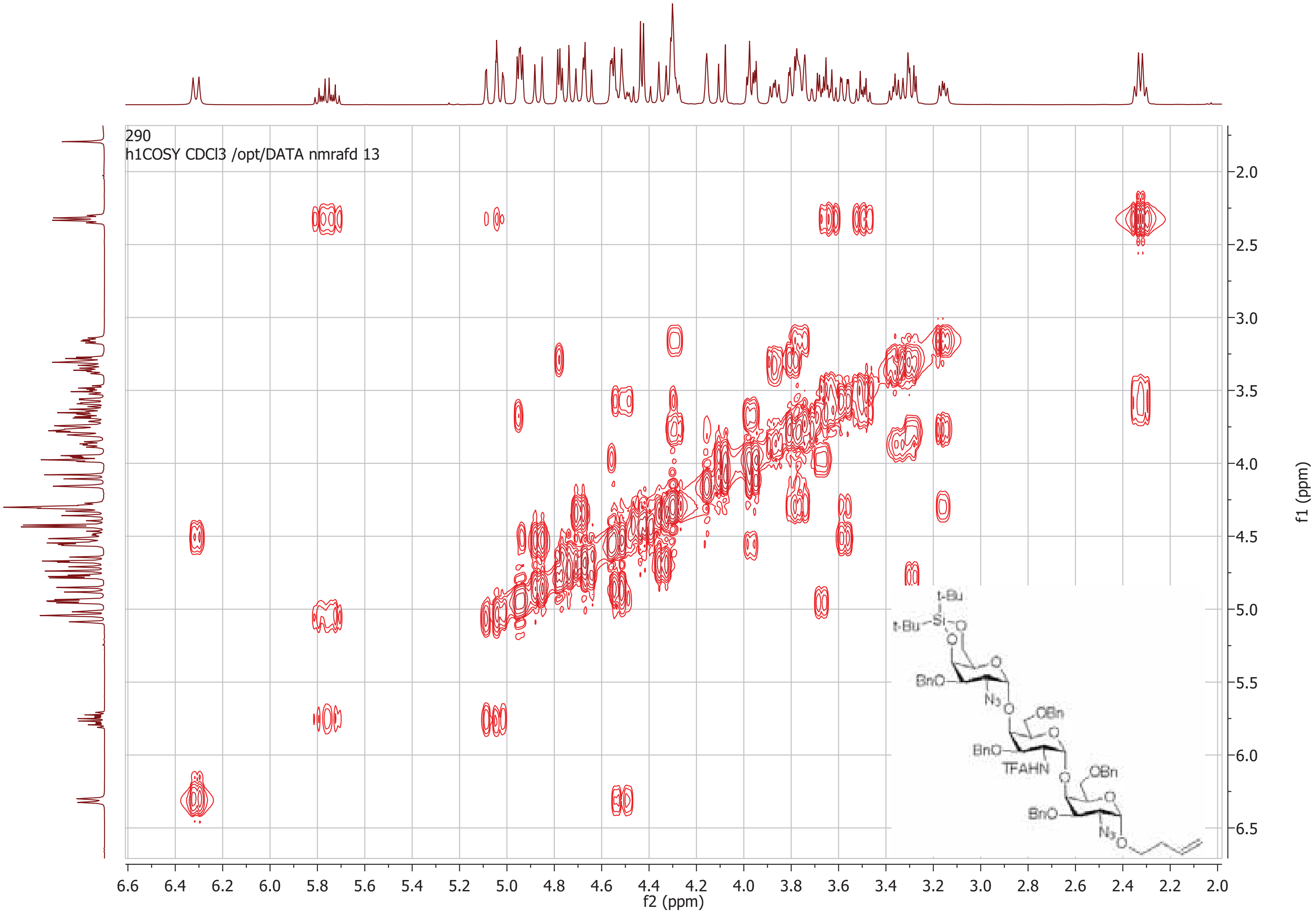
290
h1COSY CDCl3 /opt/DATA nmrafd 13
f2 (ppm)
f1 (ppm)
t-Bu
t-Bu
Si
BnO
N3
OBn
TFAHN
OBn
BnO
N3
BnO

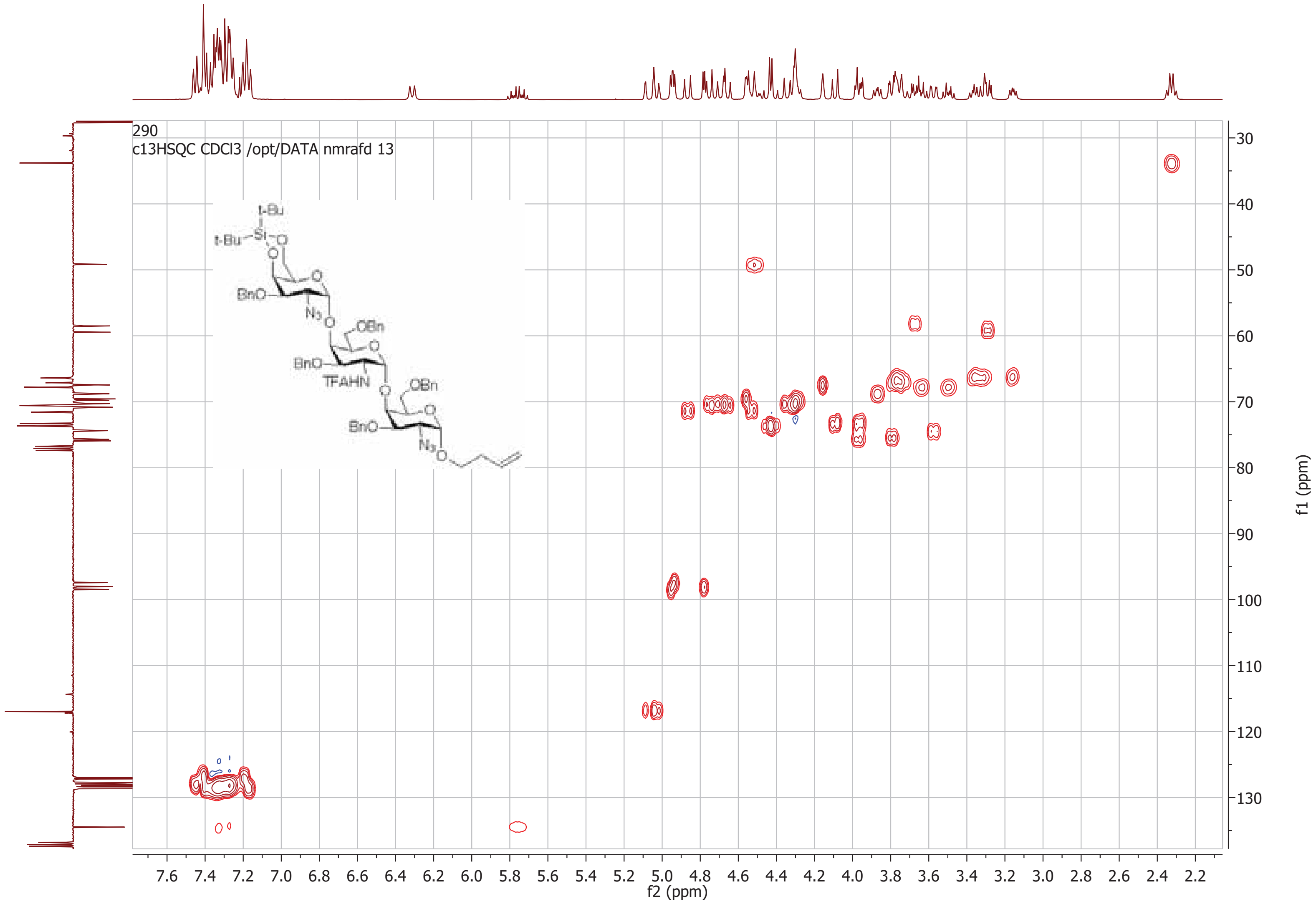
290
c13HSQC CDCl3 /opt/DATA nmrafd 13
t-Bu
t-Bu
Si
BnO
N3
OBn
BnO
TFAHN
OBn
BnO
N3
f2 (ppm)
f1 (ppm)

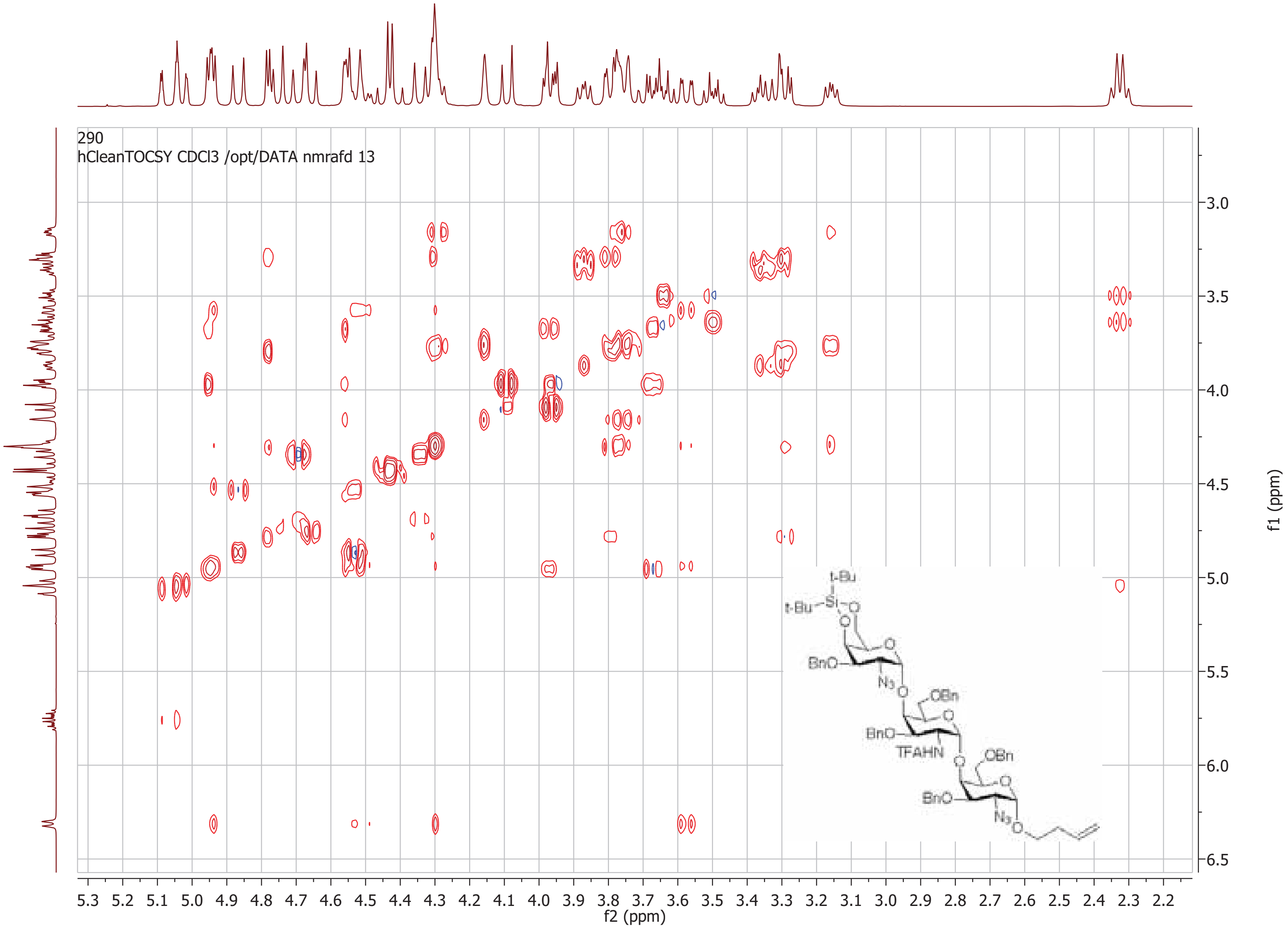
290
hCleanTOCSY CDCl3 /opt/DATA nmrafd 13
f2 (ppm)
f1 (ppm)
5.3 5.2 5.1 5.0 4.9 4.8 4.7 4.6 4.5 4.4 4.3 4.2 4.1 4.0 3.9 3.8 3.7 3.6 3.5 3.4 3.3 3.2 3.1 3.0 2.9 2.8 2.7 2.6 2.5 2.4 2.3 2.2
3.0 3.5 4.0 4.5 5.0 5.5 6.0 6.5
t-Bu
t-Bu
Si
BnO
N3
OBn
BnO
TFAHN
OBn
BnO
N3

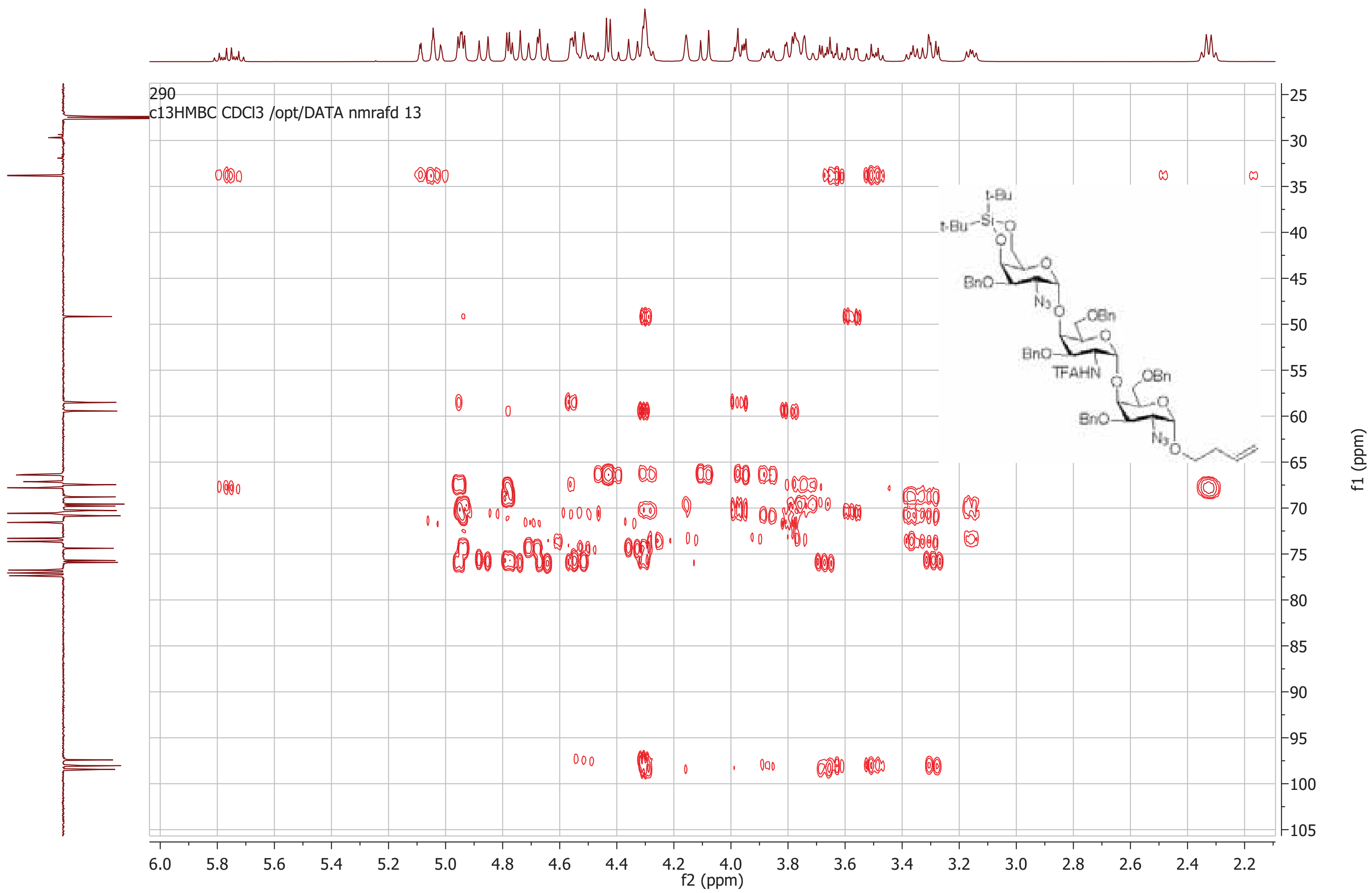
290
c13HMBC CDCl3 /opt/DATA nmrafd 13
f2 (ppm)
f1 (ppm)
t-Bu
Si
BnO
N3
OBn
TFAHN

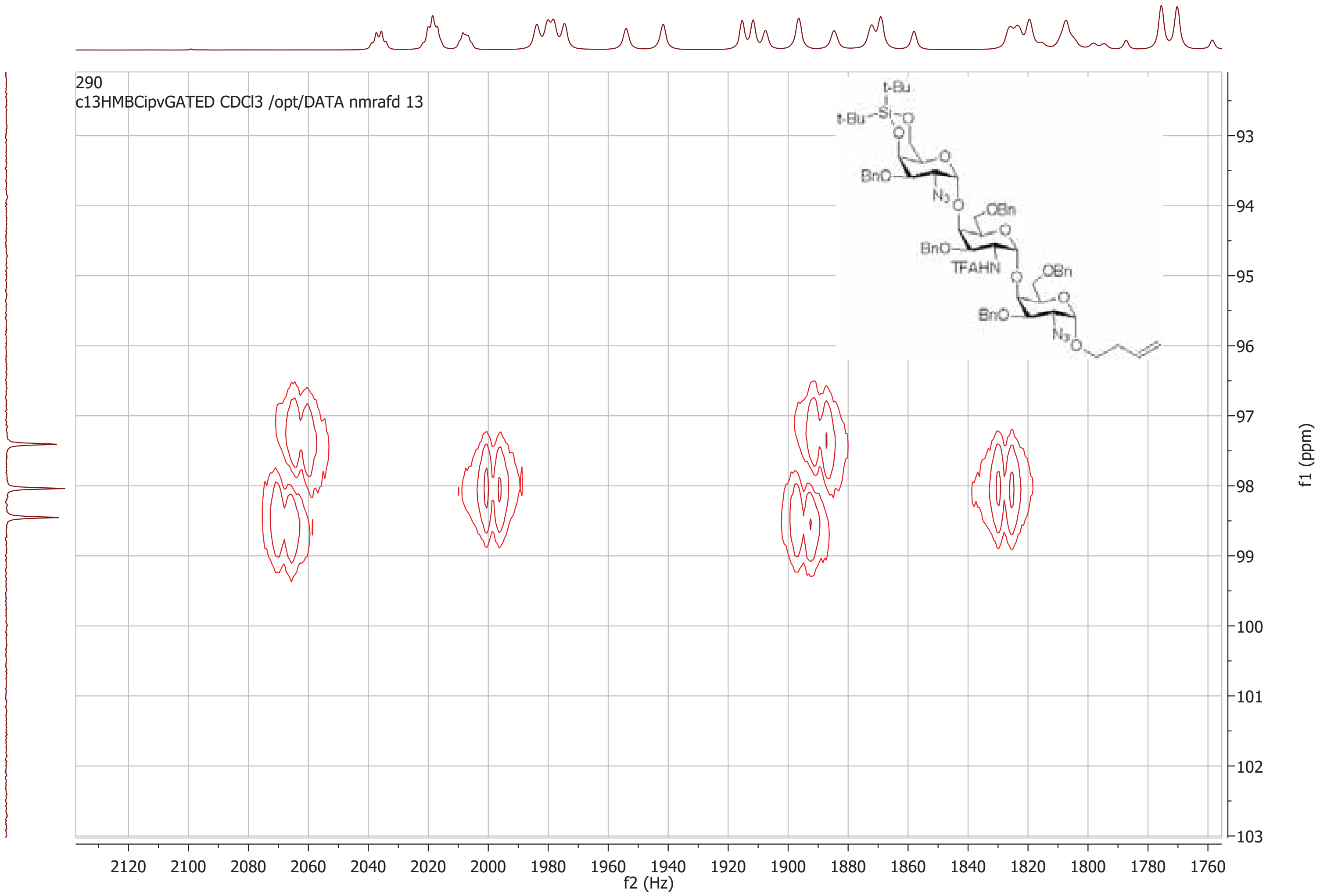
290
c13HMBCipvGATED CDCl3 /opt/DATA nmrafd 13
t-Bu
t-Bu
Si
BnO
N3
OBn
BnO
TFAHN
OBn
BnO
N3
f2 (Hz)
f1 (ppm)

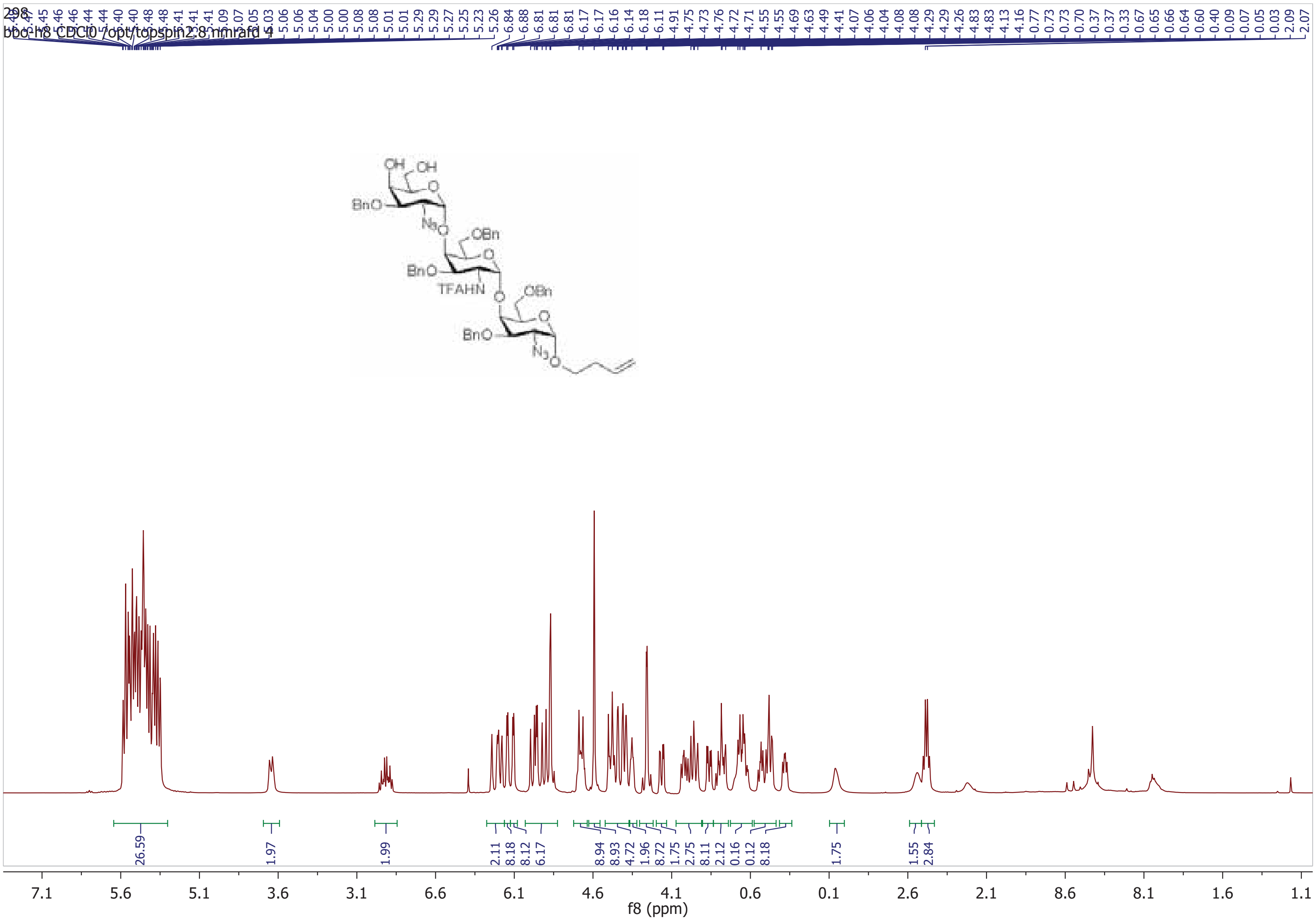
298
OH
OH
BnO
N3
OBn
BnO
TFAHN
OBn
BnO
N3
26.59
1.97
1.99
2.11
8.18
8.12
6.17
8.94
8.93
4.72
1.96
8.72
1.75
2.75
8.11
2.12
0.16
0.12
8.18
1.75
1.55
2.84
7.1
5.6
5.1
3.6
3.1
6.6
6.1
4.6
4.1
0.6
0.1
2.6
2.1
8.6
8.1
1.6
1.1
f8 (ppm)

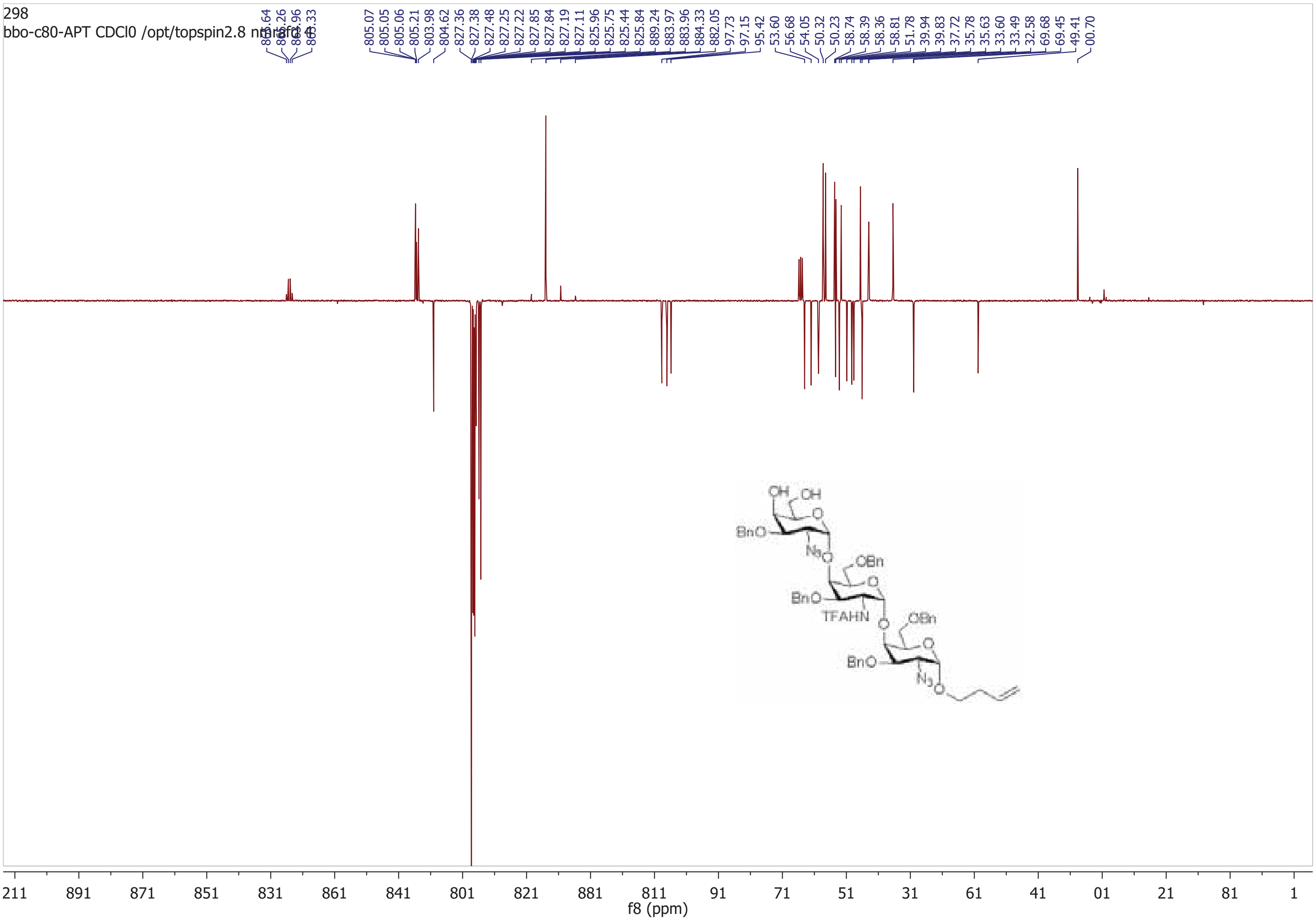
298
bbo-c80-APT CDCl0 /opt/topspin2.8 nmrafd 4
805.07
805.05
805.06
805.21
803.98
804.62
827.36
827.38
827.48
827.25
827.22
827.85
827.84
827.19
827.11
825.96
825.75
825.44
825.84
889.24
883.97
883.96
884.33
882.05
97.73
97.15
95.42
53.60
56.68
54.05
50.32
50.23
58.74
58.39
58.36
58.81
51.78
39.94
39.83
37.72
35.78
35.63
33.60
33.49
32.58
69.68
69.45
49.41
00.70
211
891
871
851
831
861
841
801
821
881
811
91
71
51
31
61
41
01
21
81
1
f8 (ppm)
OH
OH
BnO
OBn
BnO
TFAHN
OBn
BnO
N3

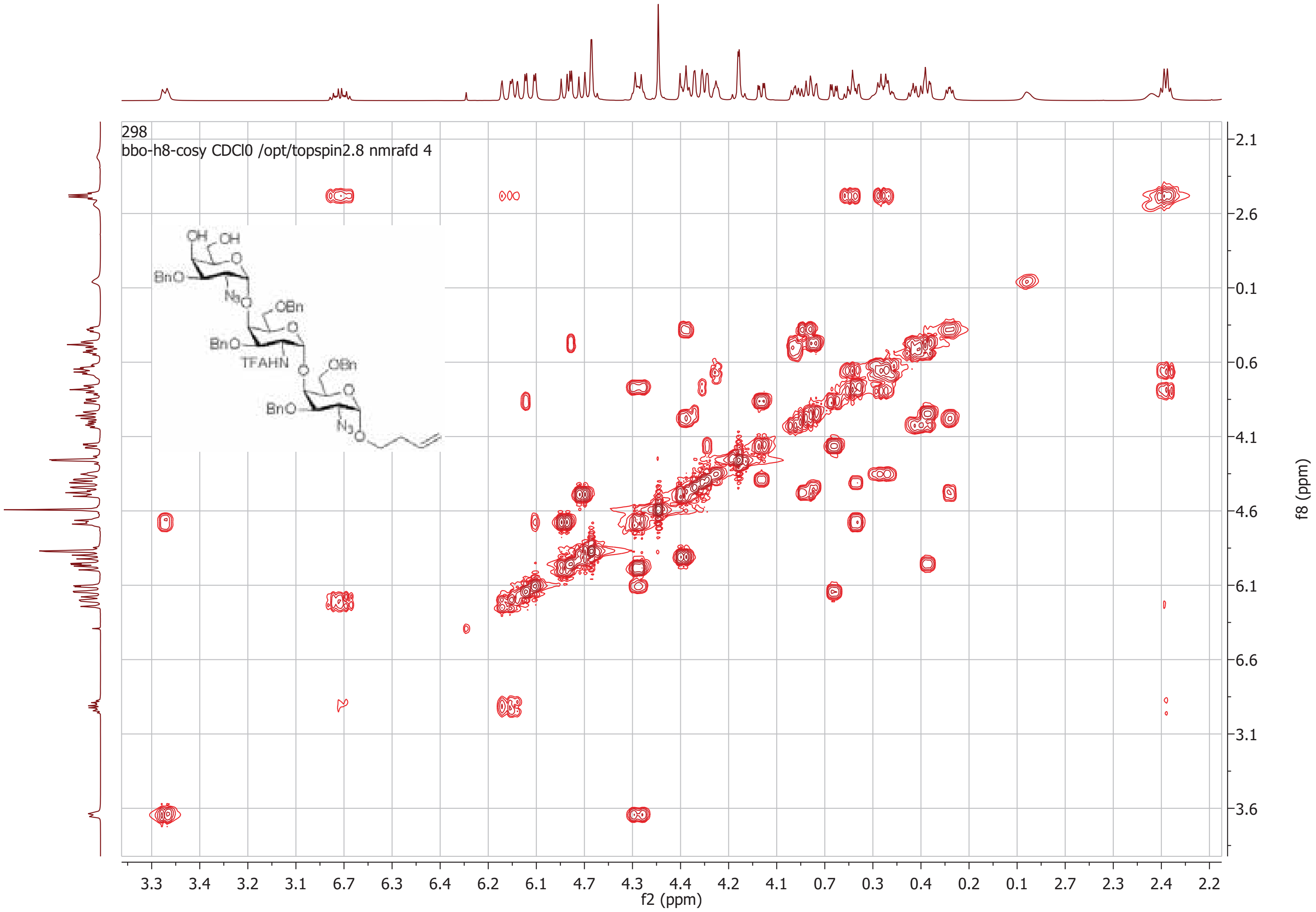
298
bbo-h8-cosy CDCl0 /opt/topspin2.8 nmrafd 4
BnO
TFAHN
OBn
3.3 3.4 3.2 3.1 6.7 6.3 6.4 6.2 6.1 4.7 4.3 4.4 4.2 4.1 0.7 0.3 0.4 0.2 0.1 2.7 2.3 2.4 2.2
f2 (ppm)
2.1 2.6 0.1 0.6 4.1 4.6 6.1 6.6 3.1 3.6
f8 (ppm)

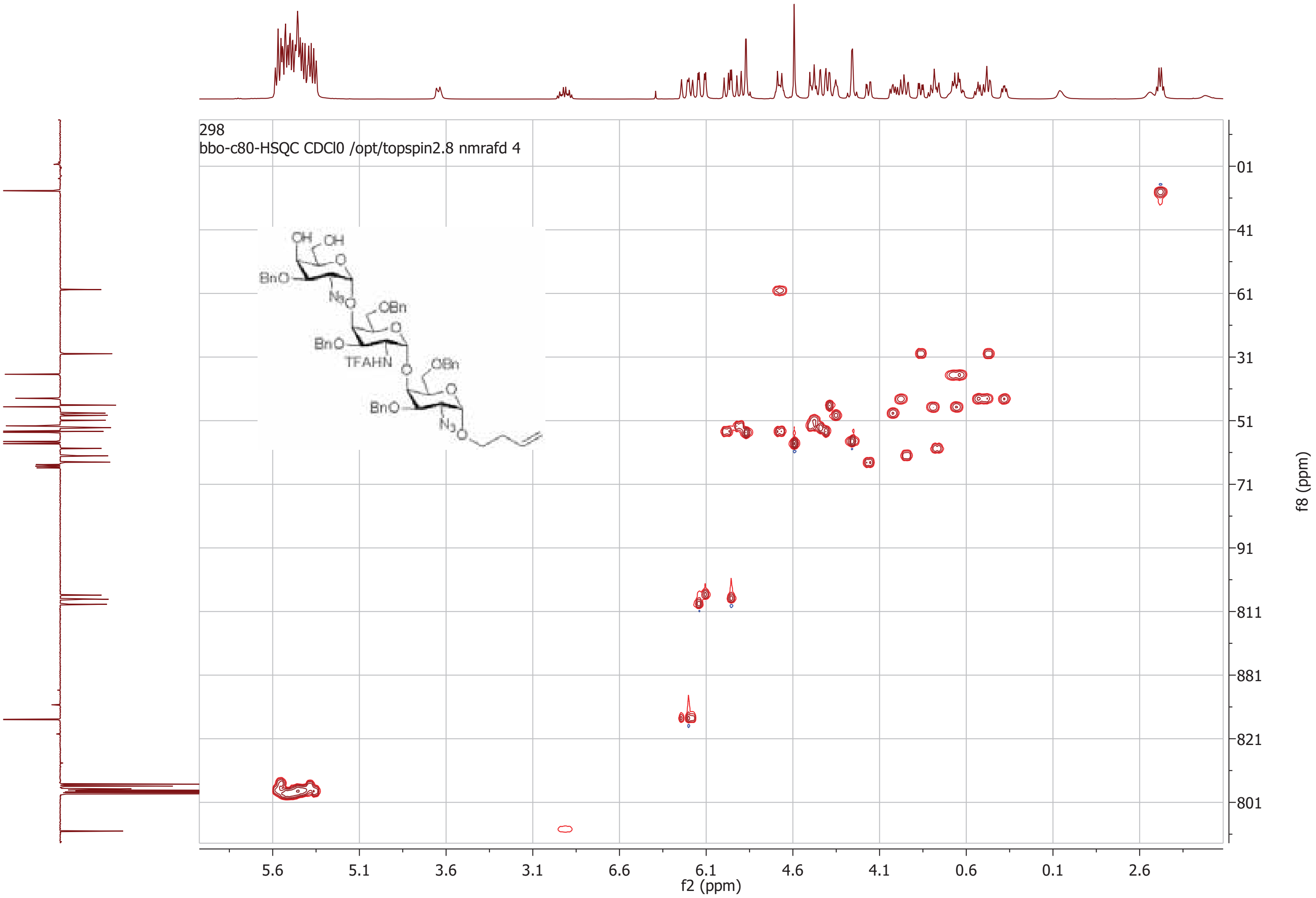
298
bbo-c80-HSQC CDCl0 /opt/topspin2.8 nmrafd 4
OH
OH
BnO
N3
OBn
BnO
TFAHN
OBn
BnO
N3
5.6 5.1 3.6 3.1 6.6 6.1 4.6 4.1 0.6 0.1 2.6
f2 (ppm)
01 41 61 31 51 71 91 811 881 821 801
f8 (ppm)

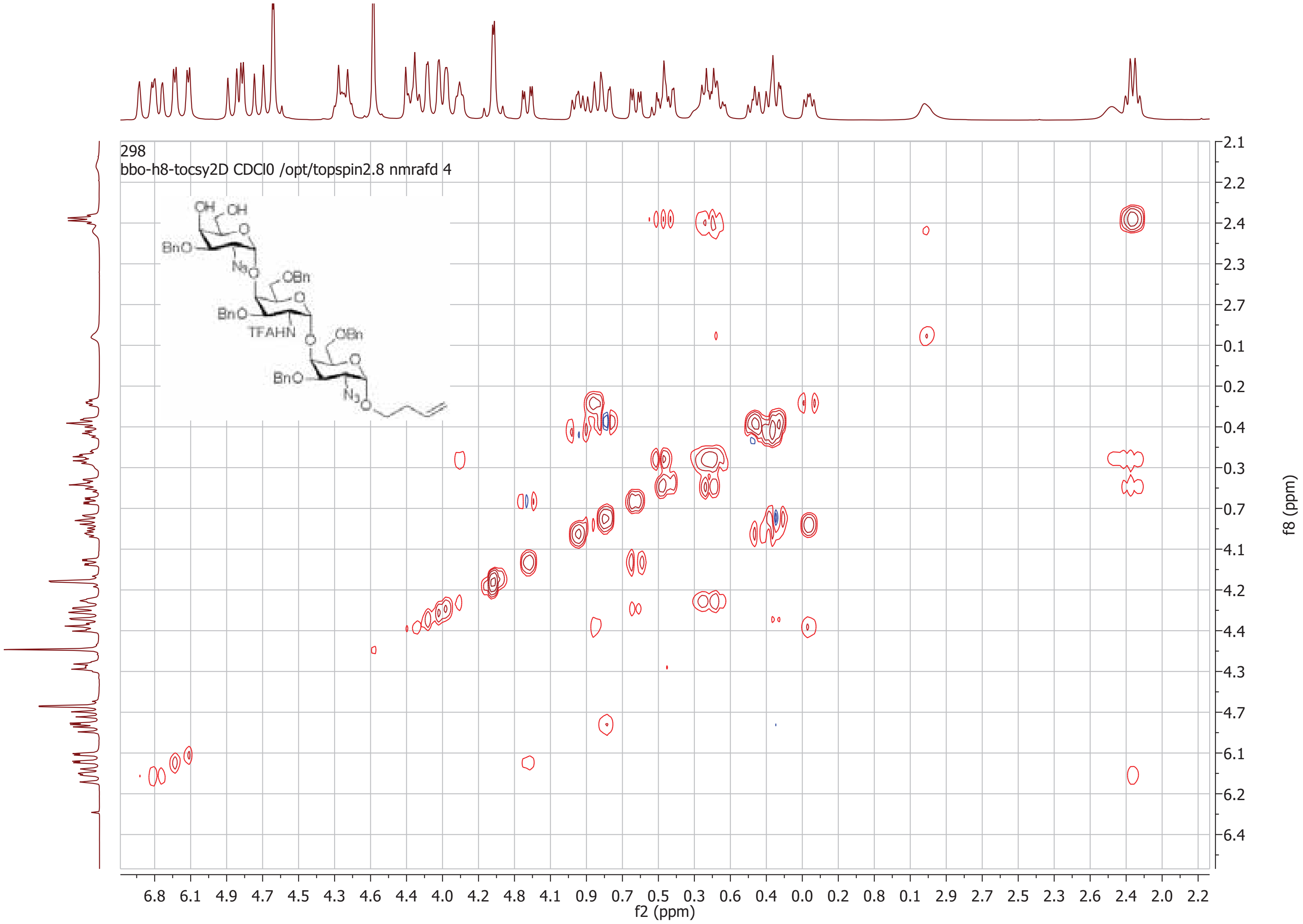
298
bbo-h8-tocsy2D CDCl0 /opt/topspin2.8 nmrafd 4
OH
OH
BnO
N3
OBn
BnO
TFAHN
OBn
BnO
N3
f2 (ppm)
f8 (ppm)

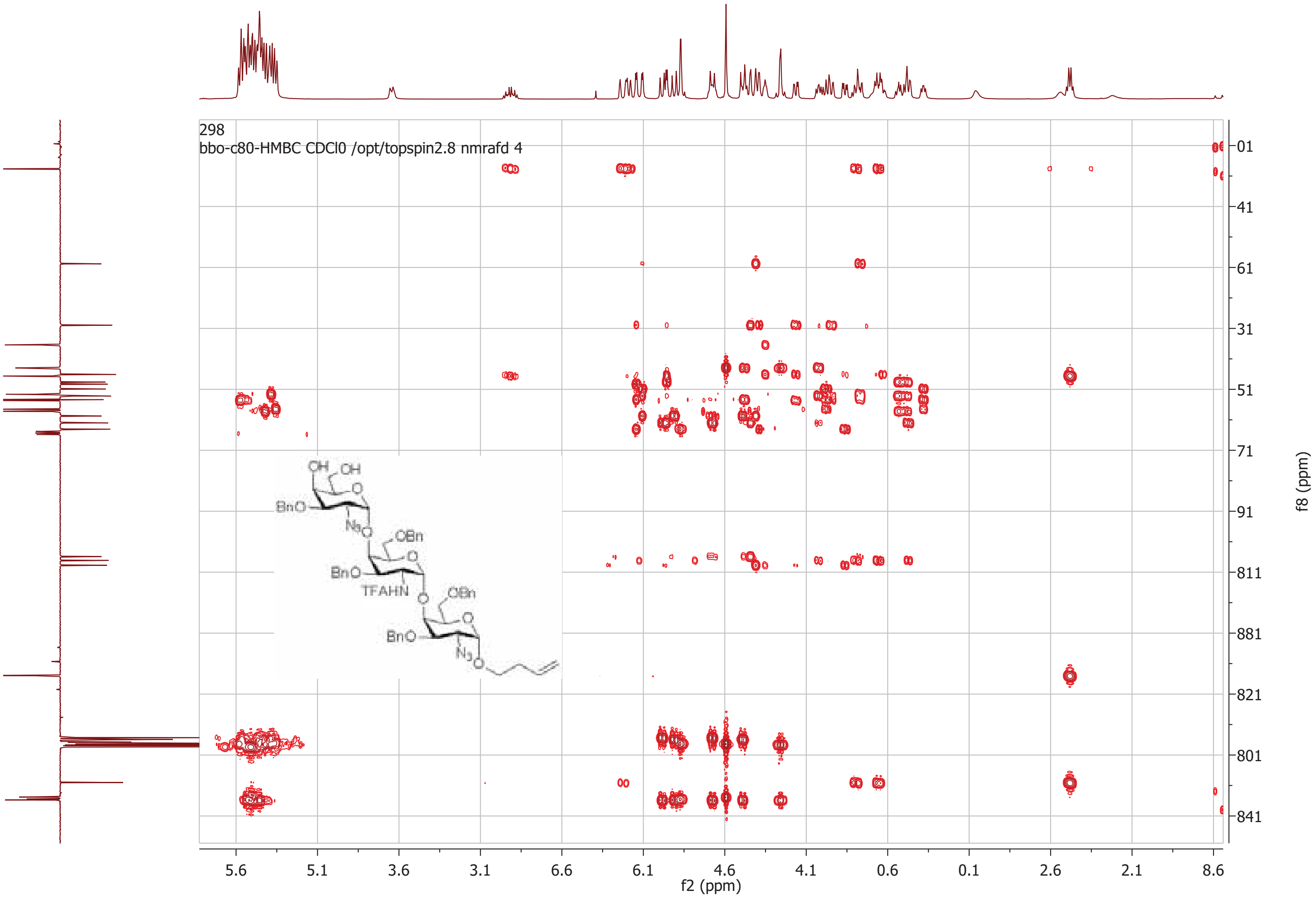
298
bbo-c80-HMBC CDCl0 /opt/topspin2.8 nmrafd 4
OH
OH
BnO
O
N3
O
OBn
BnO
O
TFAHN
O
OBn
BnO
O
N3
O
5.6
5.1
3.6
3.1
6.6
6.1
4.6
4.1
0.6
0.1
2.6
2.1
8.6
f2 (ppm)
01
41
61
31
51
71
91
811
881
821
801
841
f8 (ppm)

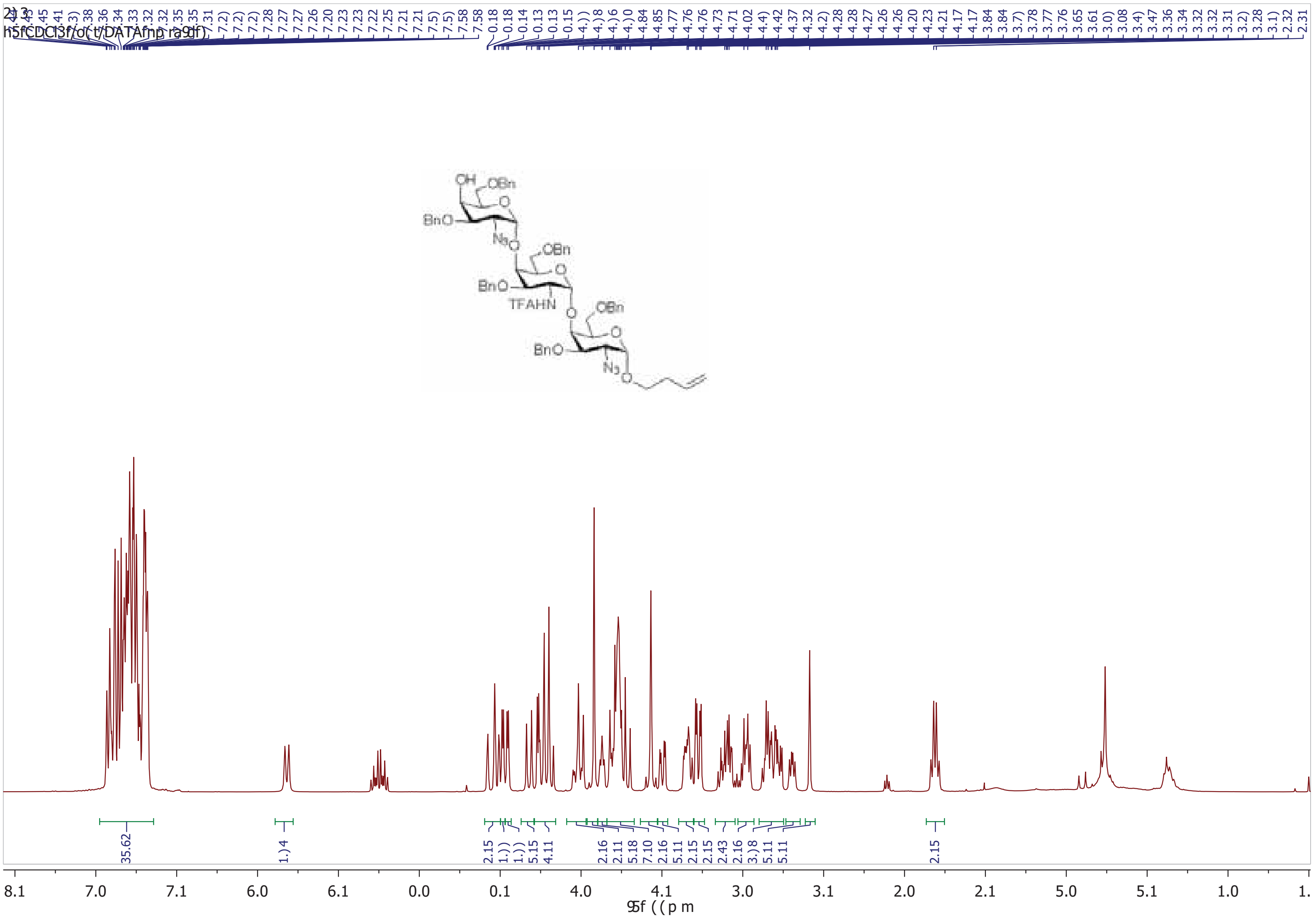

OH
OBn
BnO
N3
OBn
BnO
TFAHN
OBn
BnO
N3

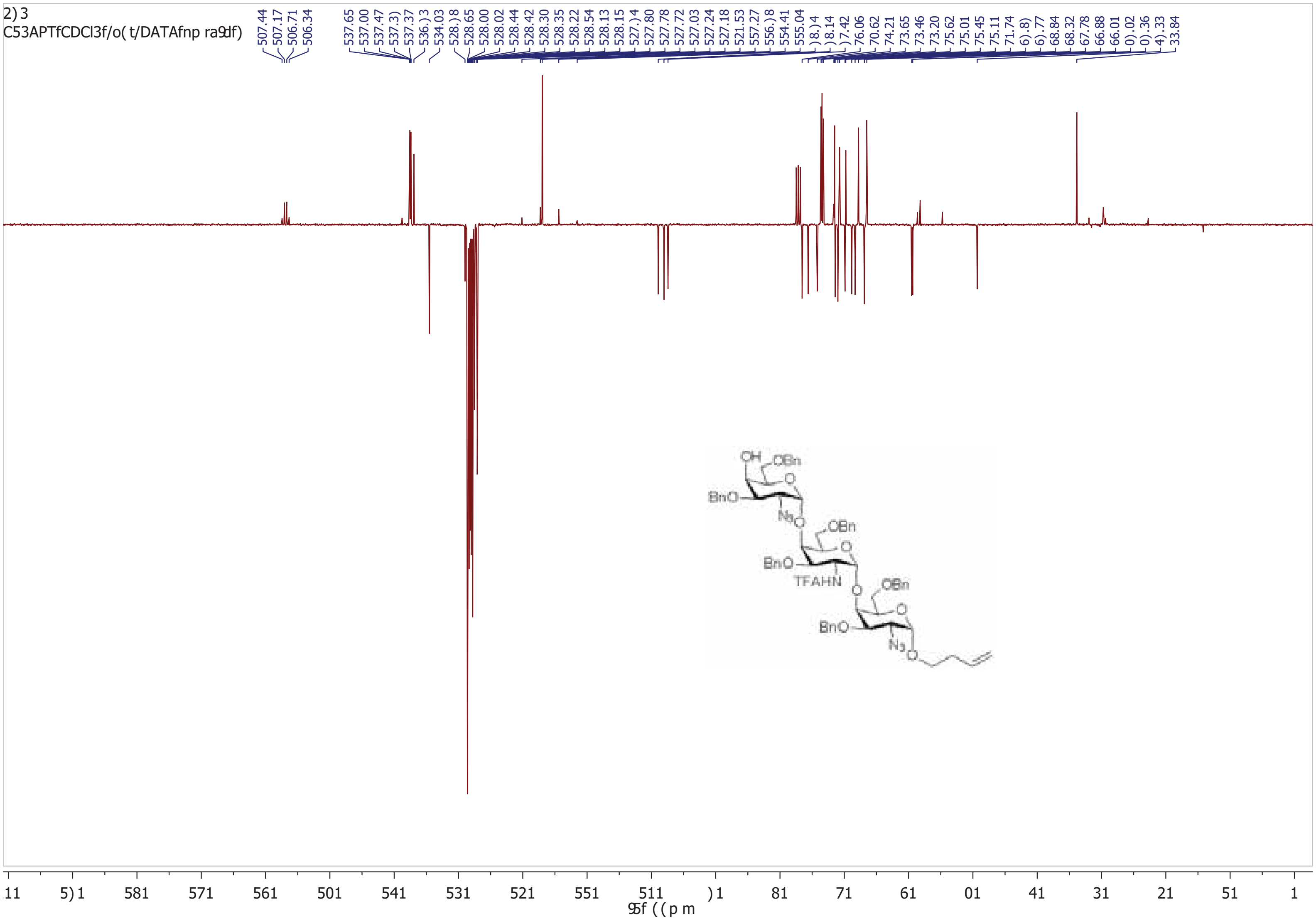

2) 3
C53APTfCDCl3f/o(t/DATAfnp ra9df)
507.44 507.17 506.71 506.34
537.65 537.00 537.47 537.3) 537.37 536.)3 534.03 528.)8 528.65 528.00 528.02 528.44 528.42 528.30 528.35 528.22 528.54 528.13 528.15 527.)4 527.80 527.78 527.72 527.03 527.24 527.18 521.53 557.27 556.)8 554.41 555.04
)8.)4)8.14)7.42 76.06 70.62 74.21 73.65 73.46 73.20 75.62 75.01 75.45 75.11 71.74 6).8) 6).77 68.84 68.32 67.78 66.88 66.01 0).02 0).36 4).33 33.84
OH OBn
BnO
N3
OBn
BnO
TFAHN
OBn
BnO
N3
11 5)1 581 571 561 501 541 531 521 551 511)1 81 71 61 01 41 31 21 51 1
9f ((p m

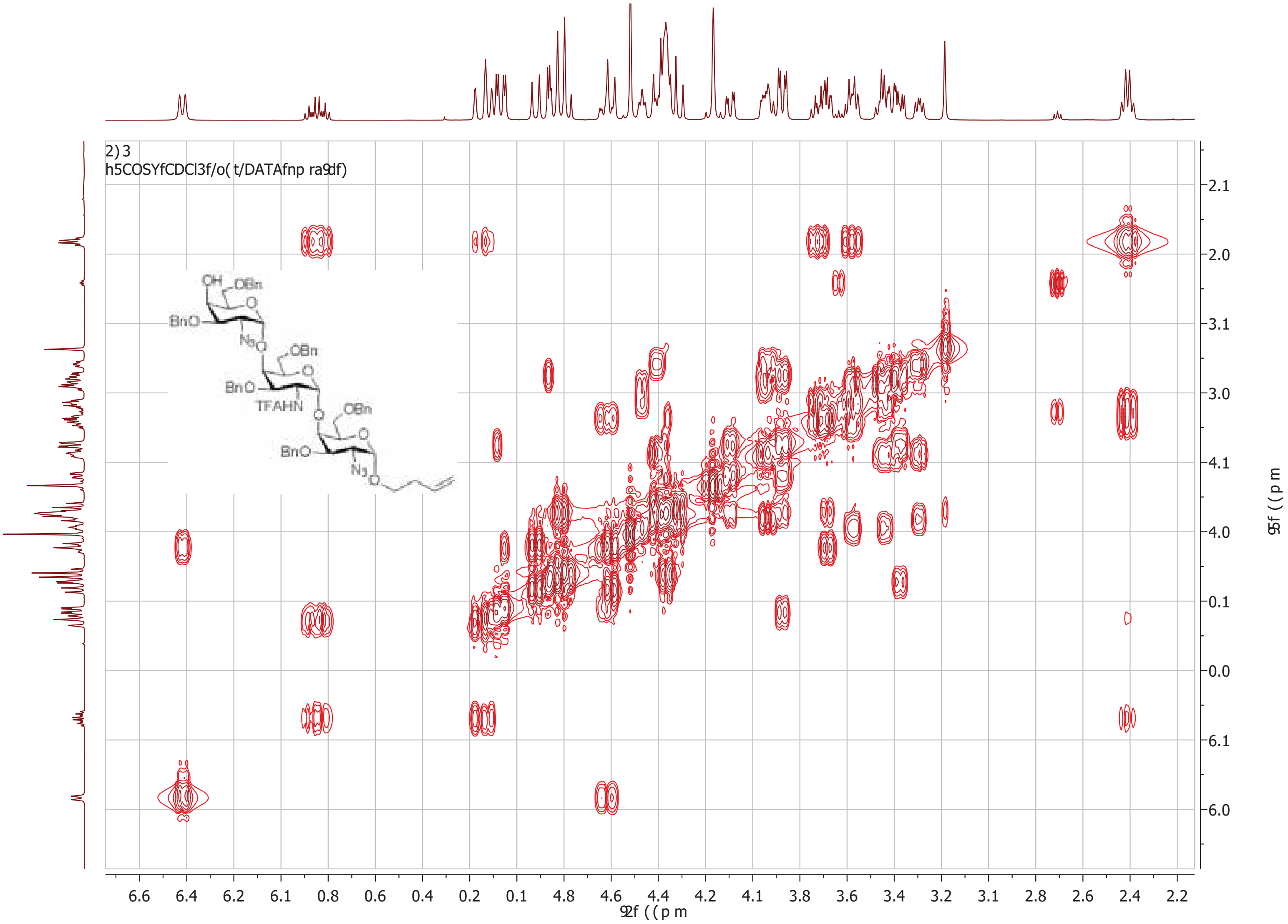

2) 3
h5COSYfCDCl3f/o(t/DATAfnp ra9df)
OH
OBn
BnO
N3
OBn
BnO
TFAHN
OBn
BnO
N3
9f ((p m
92f ((p m

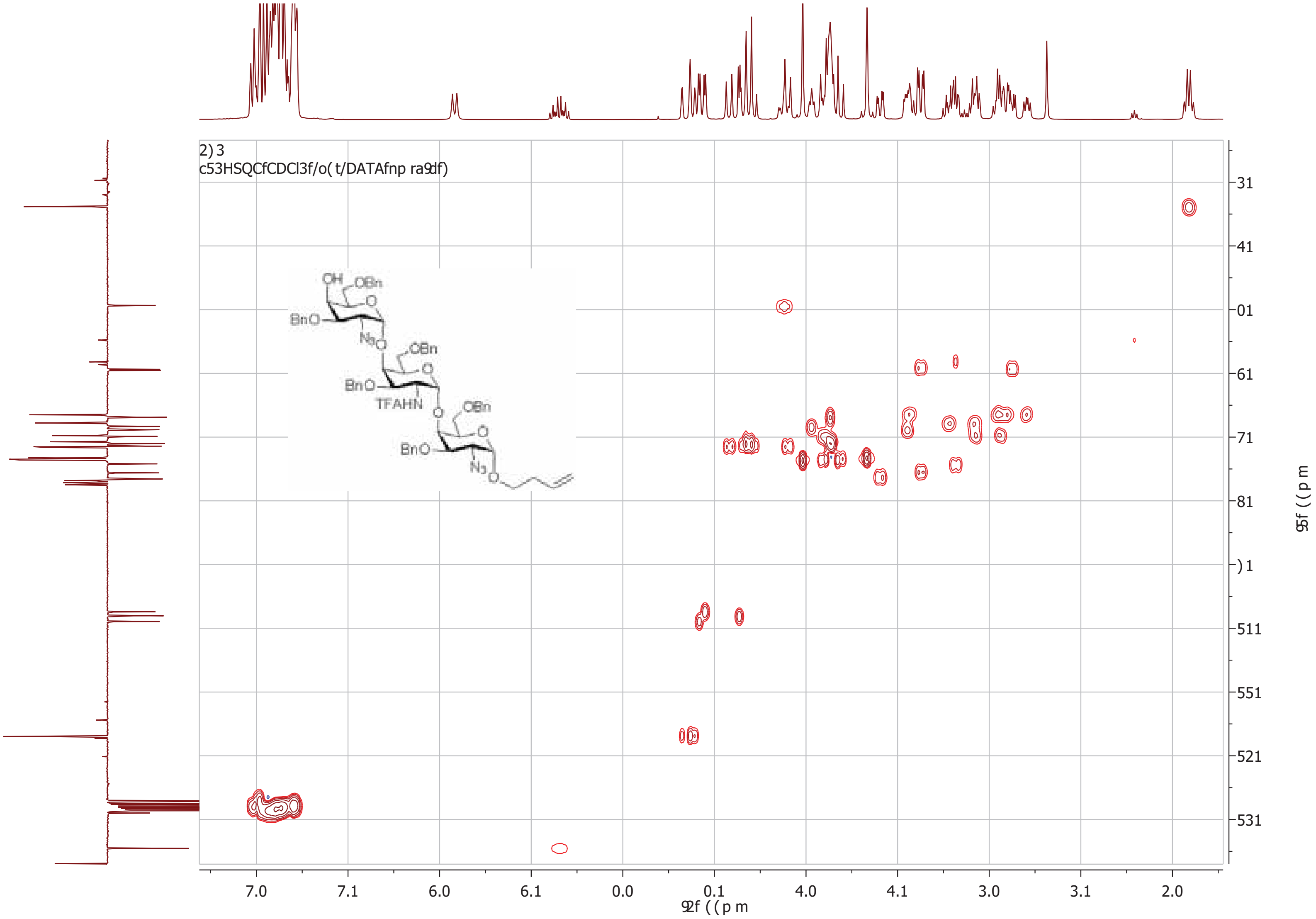
2) 3
c53HSQCfCDCl3f/o(t/DATAfnp ra9df)
OH
OBn
BnO
N3
TFAHN
9f ((p m
92f ((p m

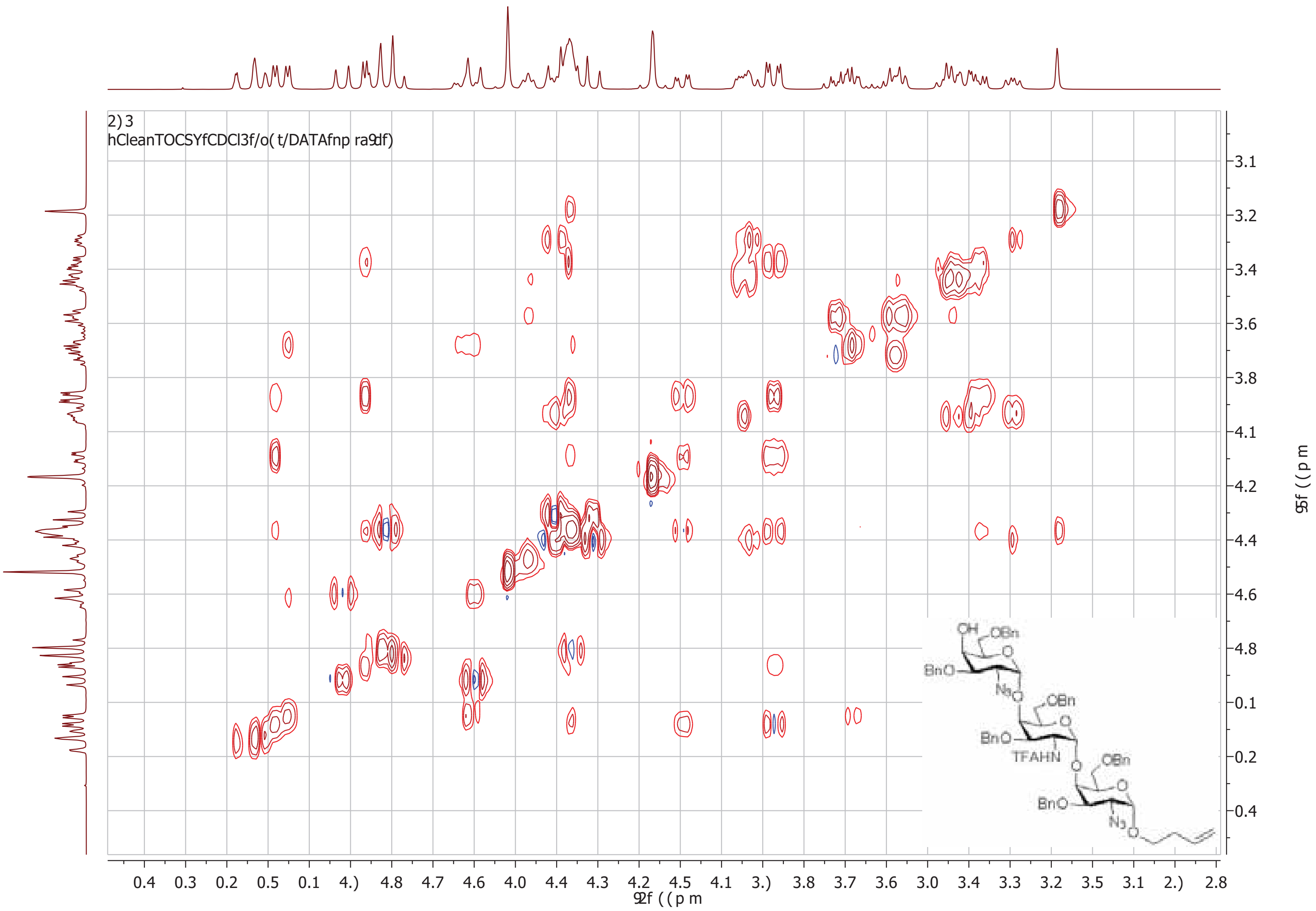
2)3
hCleanTOCSYfCDCl3f/o(t/DATAfnp ra9df)
OH
OBn
BnO
N3
TFAHN

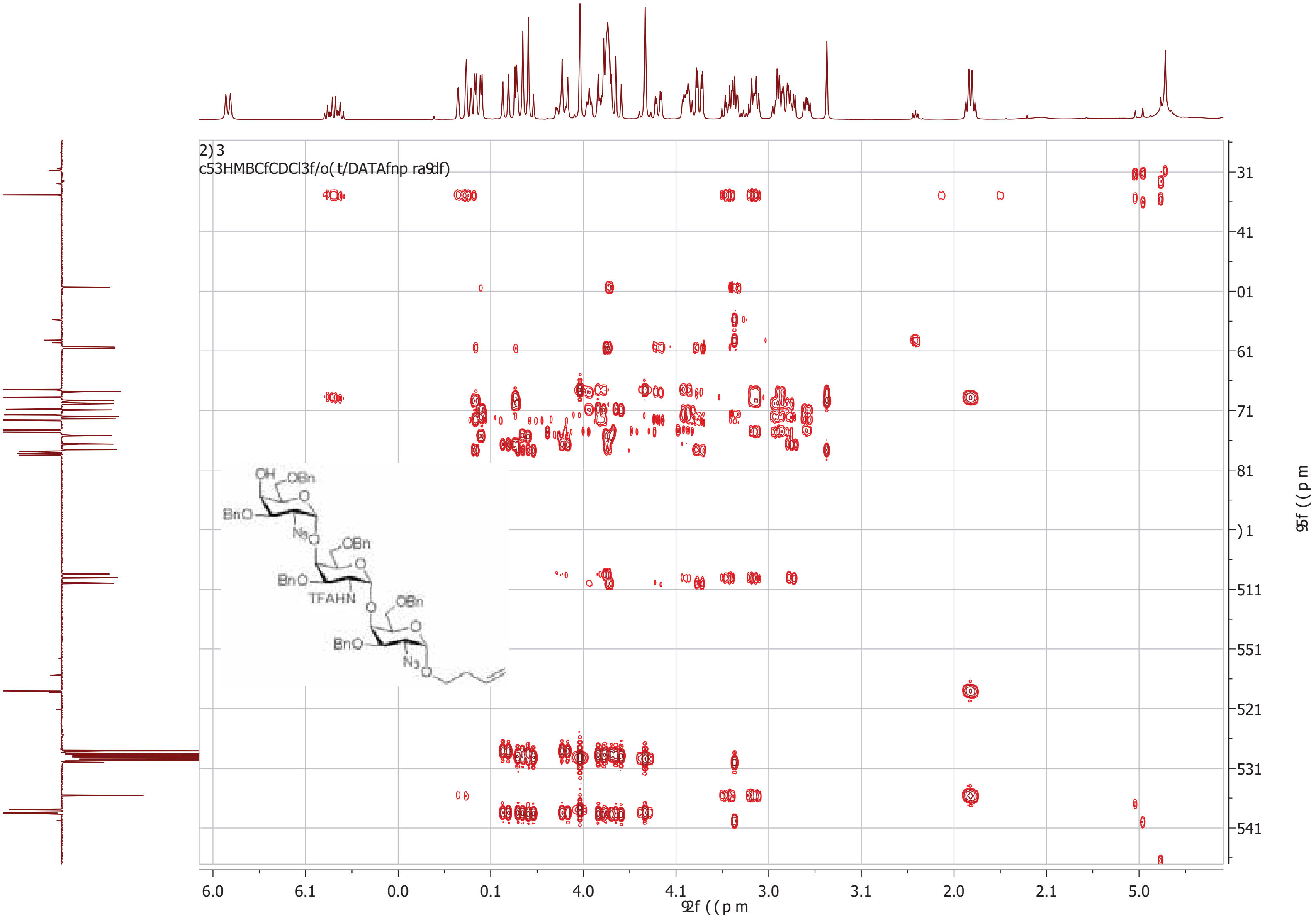
2) 3
c53HMBCfCDCl3f/o(t/DATAfnp ra9df)
OH
OBn
BnO
N3
OBn
BnO
TFAHN
OBn
BnO
N3
9f ((p m
9f ((p m

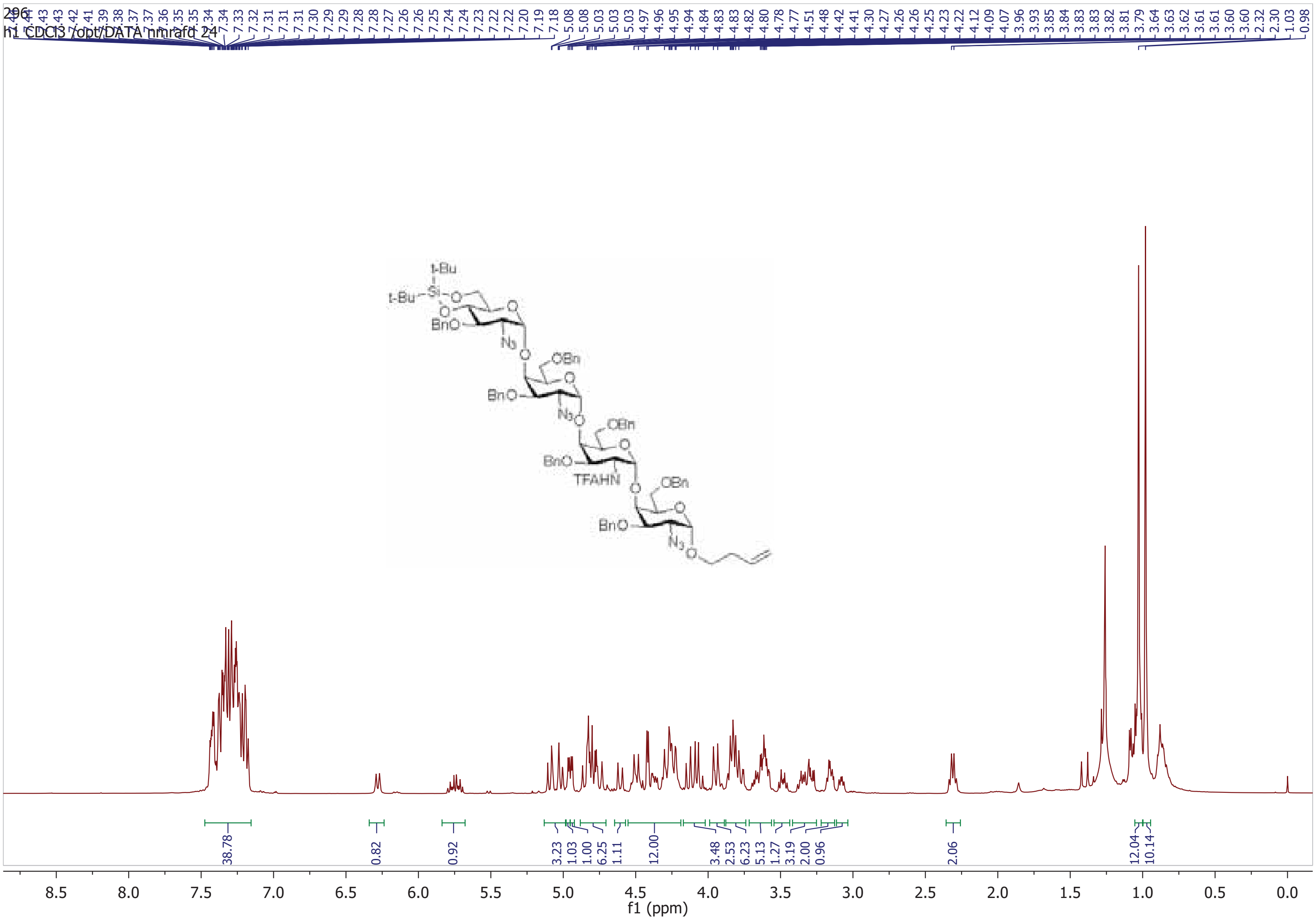
296
h1 CDCl3 /opt/DATA nmrafd 24
7.44 7.43 7.43 7.42 7.41 7.39 7.38 7.37 7.37 7.36 7.35 7.34 7.34 7.33 7.32 7.31 7.31 7.30 7.29 7.29 7.28 7.28 7.27 7.26 7.26 7.25 7.24 7.24 7.23 7.22 7.22 7.20 7.19 7.18 5.08 5.08 5.03 5.03 4.97 4.96 4.95 4.94 4.84 4.83 4.83 4.82 4.80 4.78 4.77 4.51 4.48 4.42 4.41 4.30 4.27 4.26 4.25 4.23 4.22 4.12 4.09 4.07 3.96 3.93 3.85 3.84 3.83 3.83 3.82 3.81 3.79 3.64 3.63 3.62 3.61 3.61 3.60 3.60 2.32 2.30 1.03 0.98
t-Bu
t-Bu
Si
O
BnO
N3
OBn
BnO
N3
OBn
BnO
TFAHN
OBn
BnO
N3
38.78
0.82
0.92
3.23
1.03
1.00
6.25
1.11
12.00
3.48
2.53
6.23
5.13
1.27
3.19
2.00
0.96
2.06
12.04
10.14
8.5 8.0 7.5 7.0 6.5 6.0 5.5 5.0 4.5 4.0 3.5 3.0 2.5 2.0 1.5 1.0 0.5 0.0
f1 (ppm)

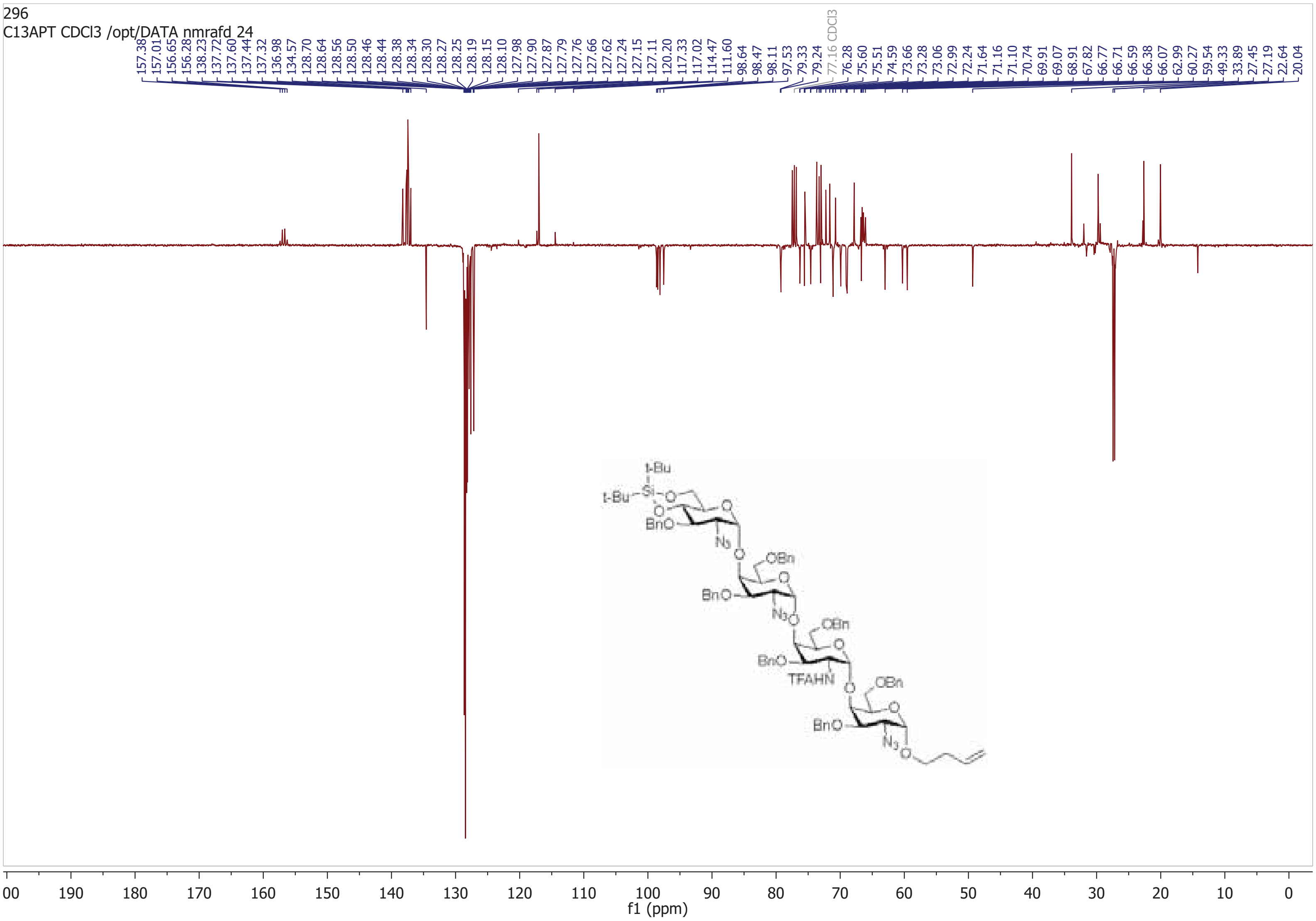

296
C13APT CDCl3 /opt/DATA nmrafd 24
157.38
157.01
156.65
156.28
138.23
137.72
137.60
137.44
137.32
136.98
134.57
128.70
128.64
128.56
128.50
128.46
128.44
128.38
128.34
128.30
128.27
128.25
128.19
128.15
128.10
127.98
127.90
127.87
127.79
127.76
127.66
127.62
127.24
127.15
127.11
120.20
117.33
117.02
114.47
111.60
98.64
98.47
98.11
97.53
79.33
79.24
77.16 CDCl3
76.28
75.60
75.51
74.59
73.66
73.28
73.06
72.99
72.24
71.64
71.16
71.10
70.74
69.91
69.07
68.91
67.82
66.77
66.71
66.59
66.38
66.07
62.99
60.27
59.54
49.33
33.89
27.45
27.19
22.64
20.04
t-Bu
t-Bu
Si
O
O
BnO
N3
O
OBn
O
BnO
N3
O
OBn
O
BnO
TFAHN
O
OBn
O
BnO
N3
O
00
190
180
170
160
150
140
130
120
110
100
90
80
70
60
50
40
30
20
10
0
f1 (ppm)

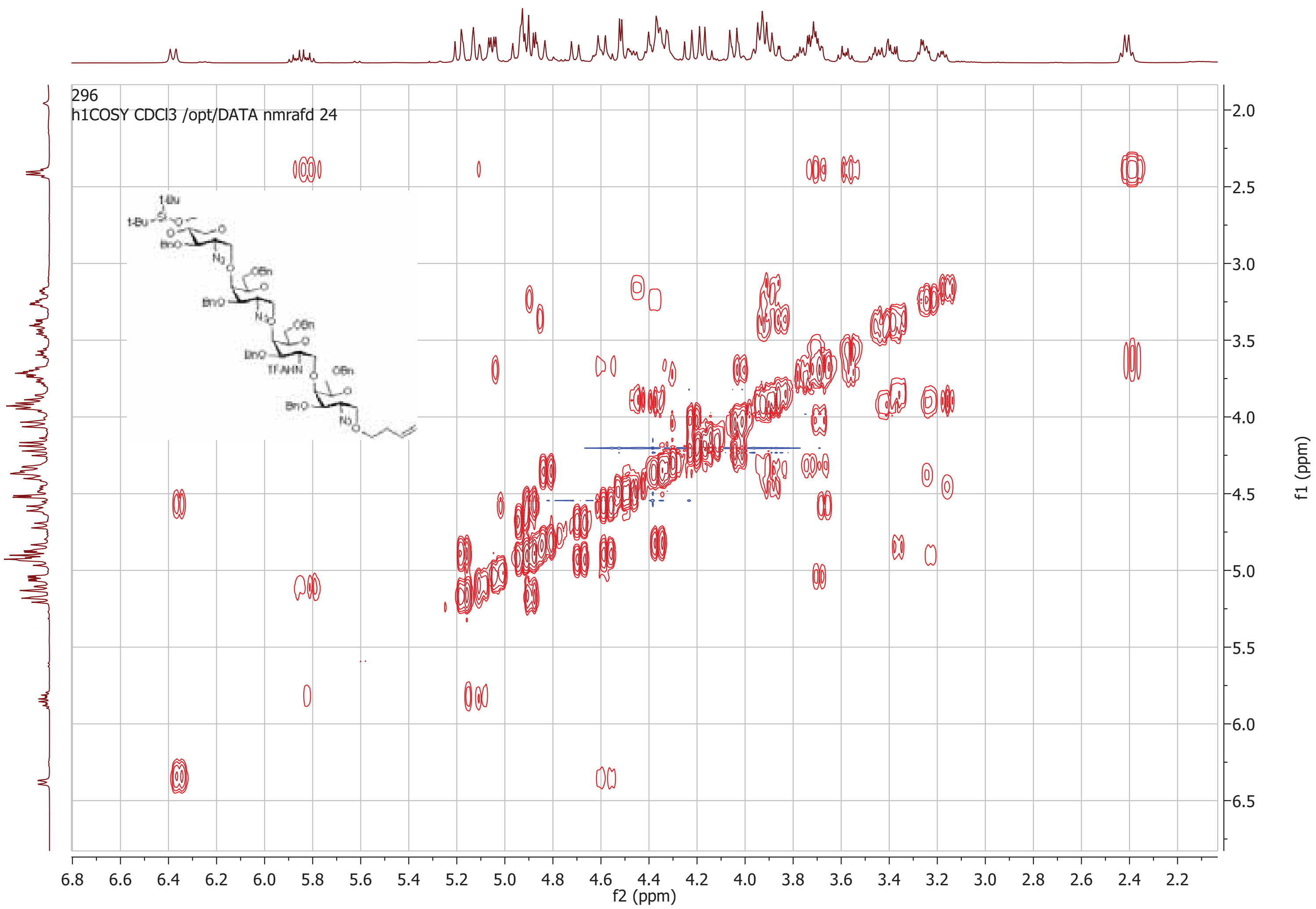
296
h1COSY CDCl3 /opt/DATA nmrafd 24
f2 (ppm)
f1 (ppm)

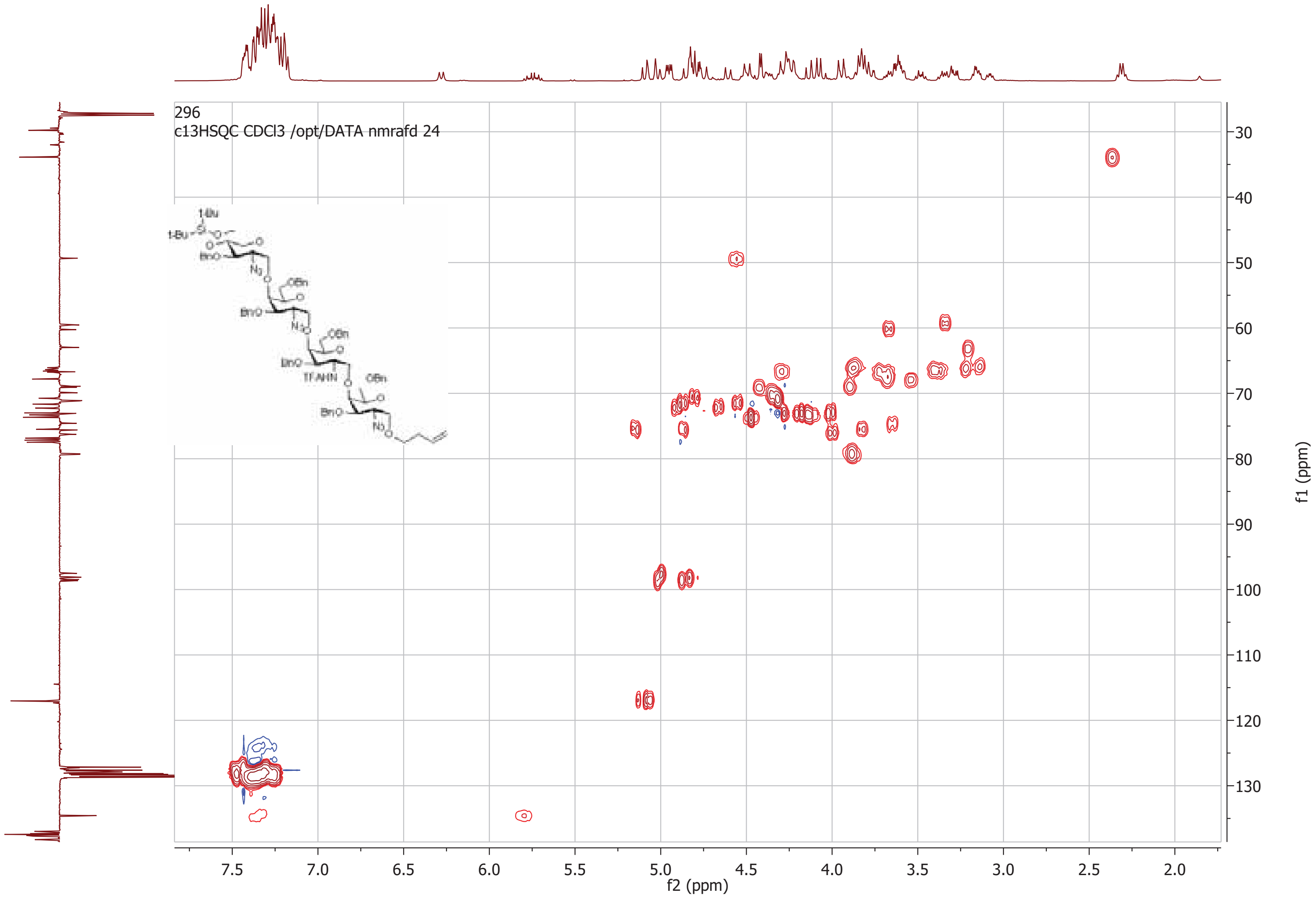
296
c13HSQC CDCl3 /opt/DATA nmrafd 24
t-Bu
t-Bu
Si
BnO
N3
OBn
BnO
N3
OBn
BnO
TFAHN
OBn
BnO
N3
f1 (ppm)
f2 (ppm)
30
40
50
60
70
80
90
100
110
120
130
7.5
7.0
6.5
6.0
5.5
5.0
4.5
4.0
3.5
3.0
2.5
2.0

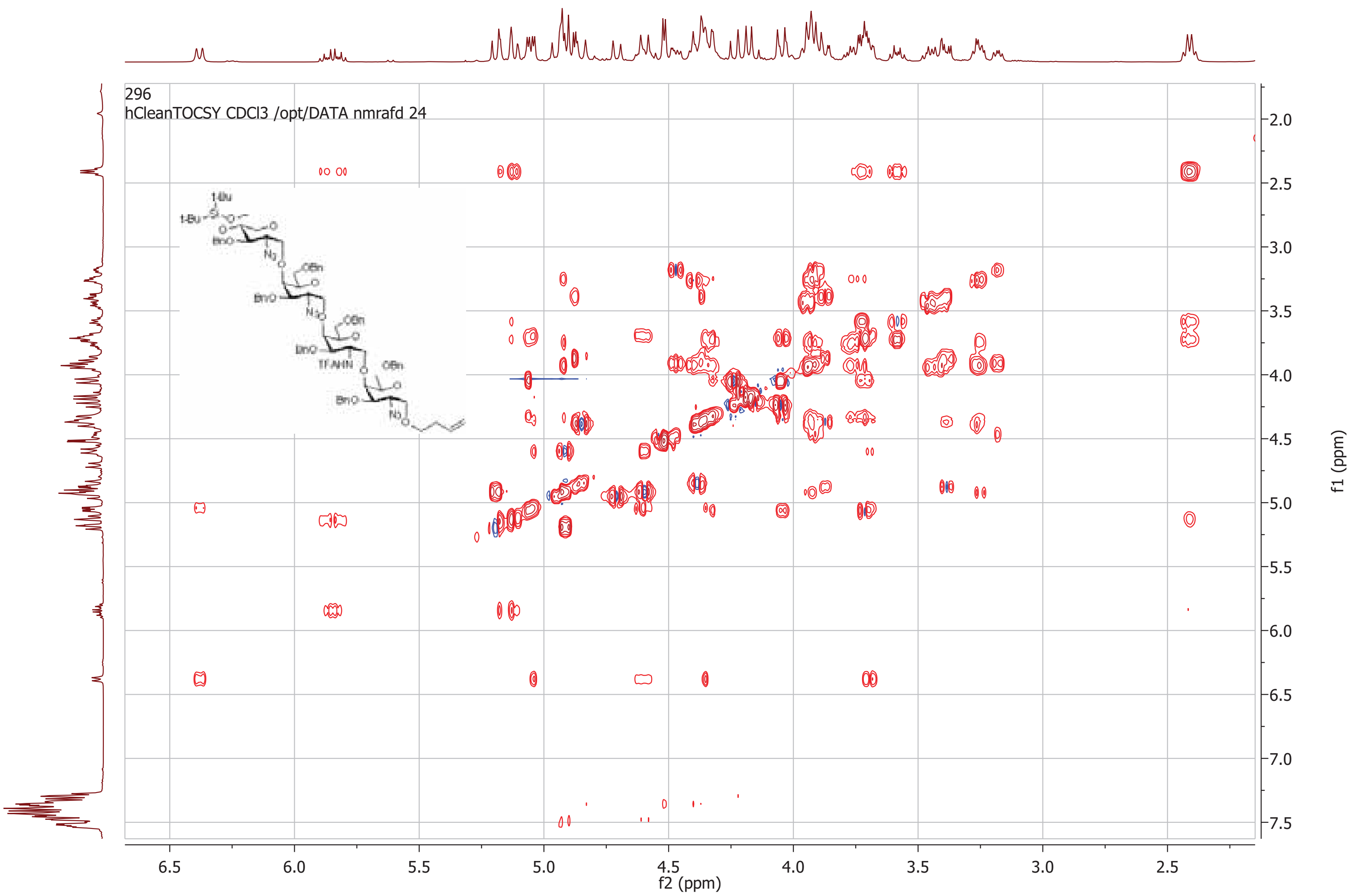
296
hCleanTOCSY CDCl3 /opt/DATA nmrafd 24
t-Bu
t-Bu
Si
BnO
N3
OBn
BnO
N3
OBn
BnO
TFAHN
OBn
BnO
N3
f2 (ppm)
f1 (ppm)

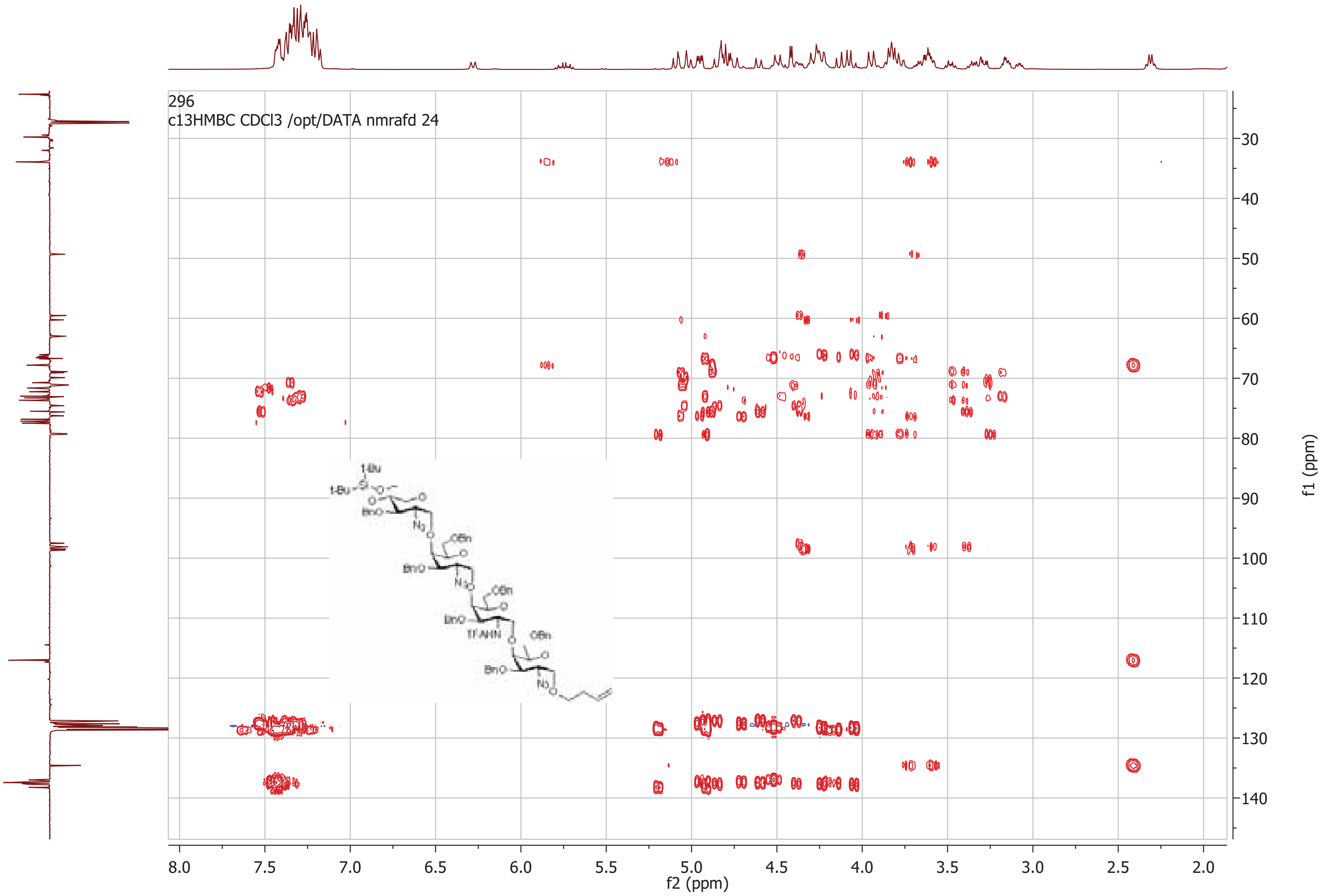
296
c13HMBC CDCl3 /opt/DATA nmrafd 24
f2 (ppm)
f1 (ppm)
t-Bu
t-Bu
BnO
OBn
TFAHN

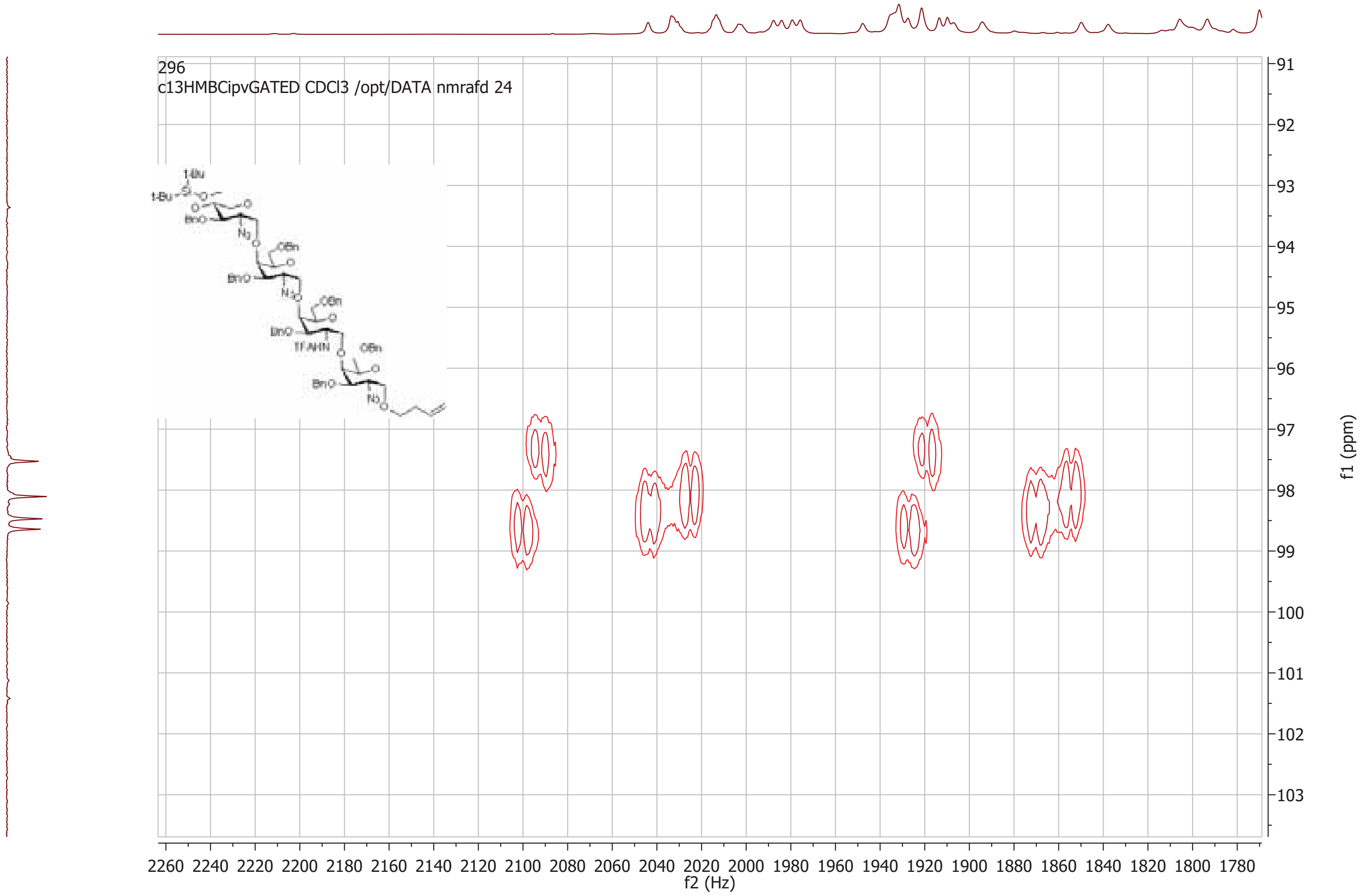
296
c13HMBCipvGATED CDCl3 /opt/DATA nmrafd 24
f2 (Hz)
f1 (ppm)

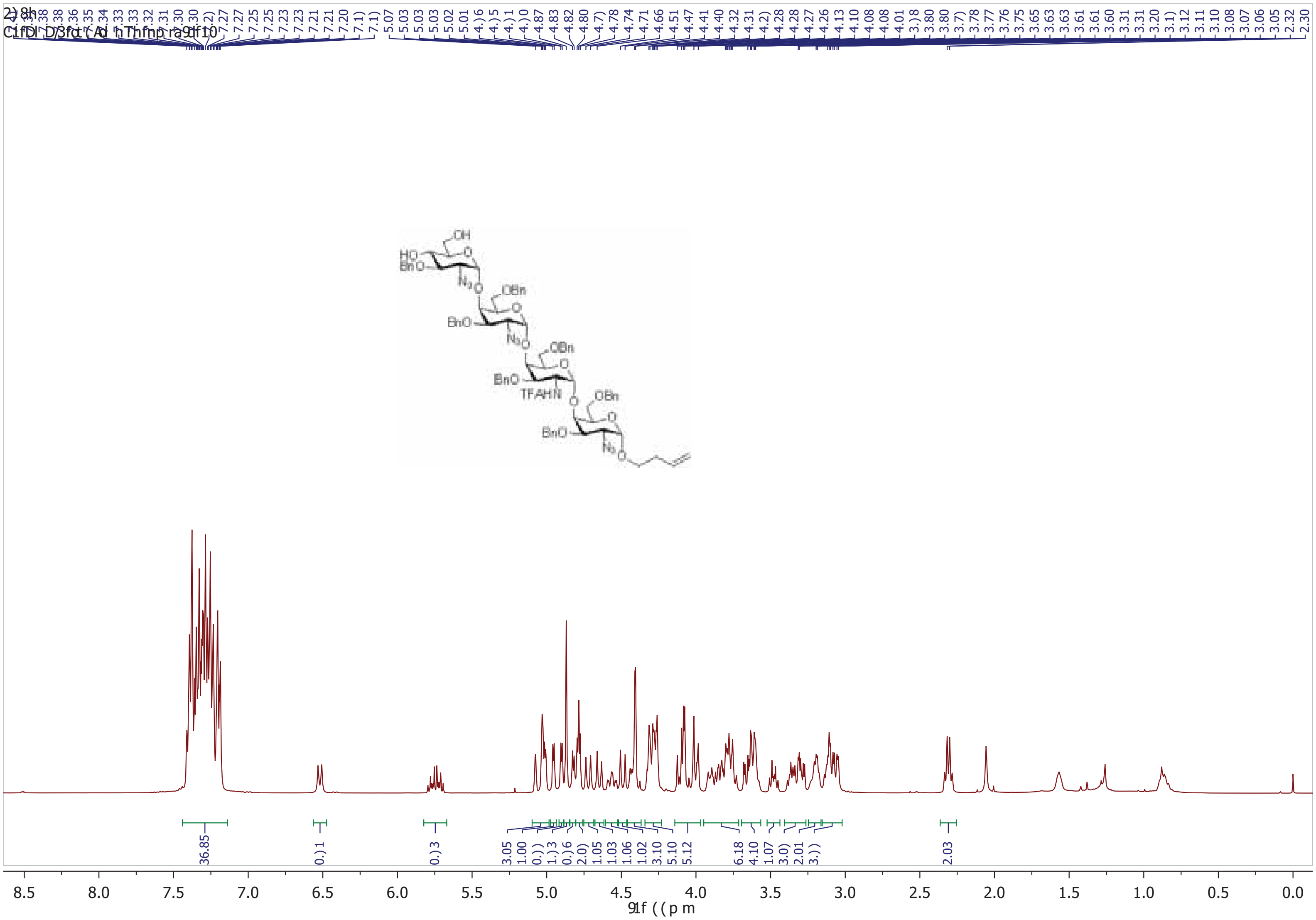

HO
BnO
OH
N3
OBn
BnO
N3
OBn
BnO
TFAHN
OBn
BnO
N3
36.85
2.03
8.5 8.0 7.5 7.0 6.5 6.0 5.5 5.0 4.5 4.0 3.5 3.0 2.5 2.0 1.5 1.0 0.5 0.0

2)8h
D13hPT DI D/3 ct(Ad hTh np ra9d 10

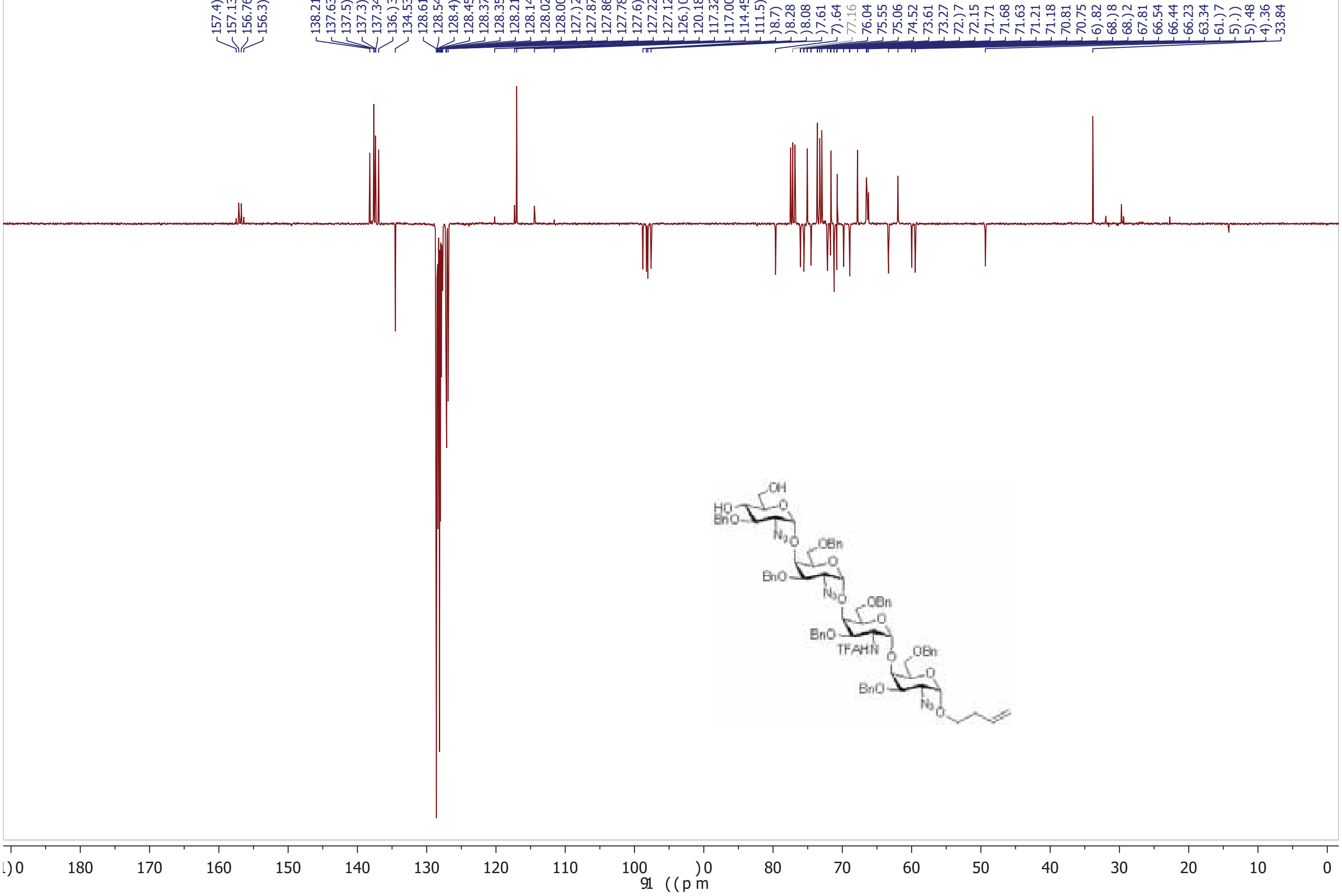

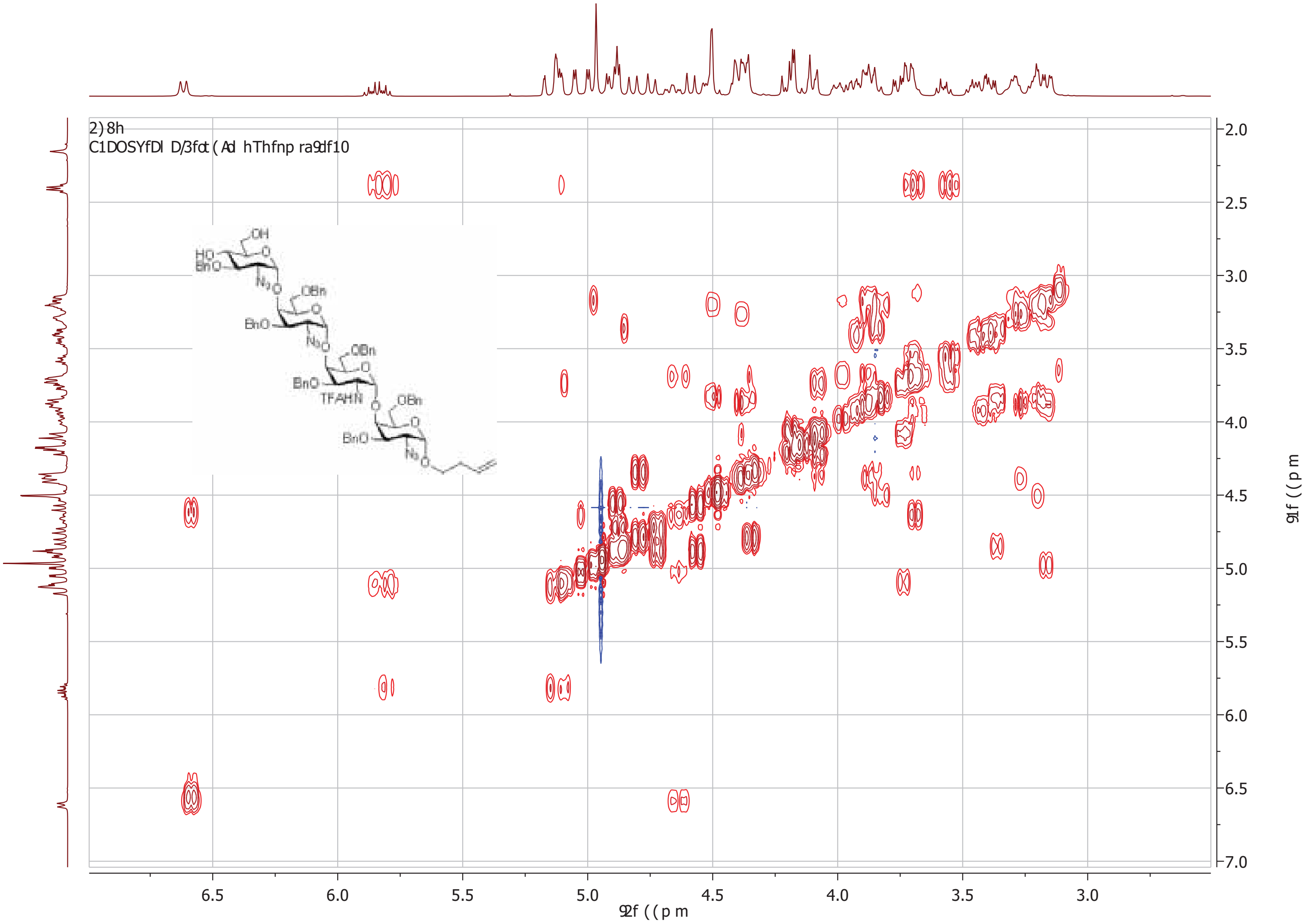
6.5
6.0
5.5
5.0
4.5
4.0
3.5
3.0
2.0
2.5
3.0
3.5
4.0
4.5
5.0
5.5
6.0
6.5
7.0
HO
OH
BnO
OBn
BnO
TFAHN

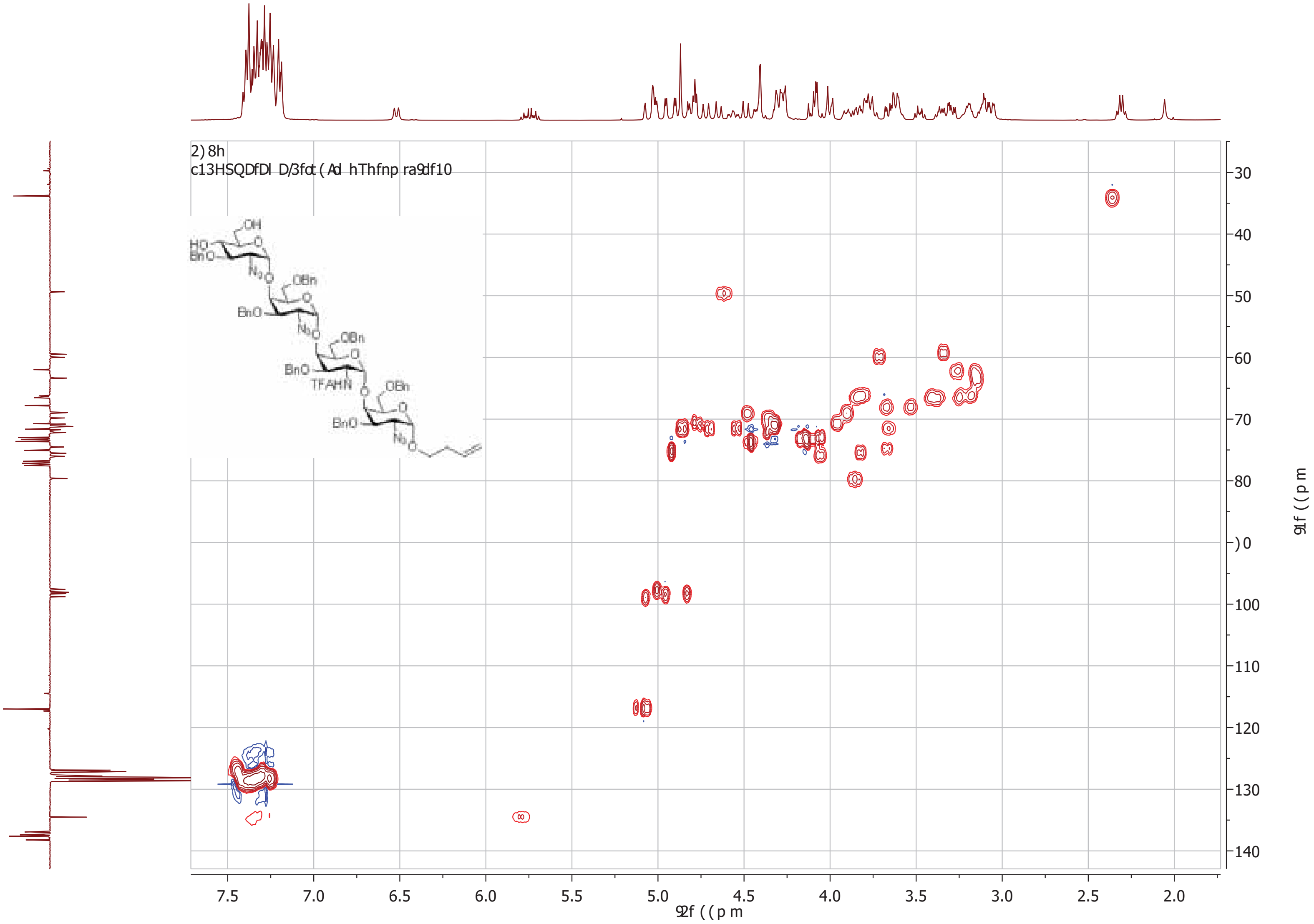
2) 8h
c13HSQDfDl D/3fot (Ad hThfnp ra9df10
OH
HO
BnO
N3
OBn
BnO
N3
OBn
BnO
TFAHN
OBn
BnO
N3
9f ((p m
92f ((p m

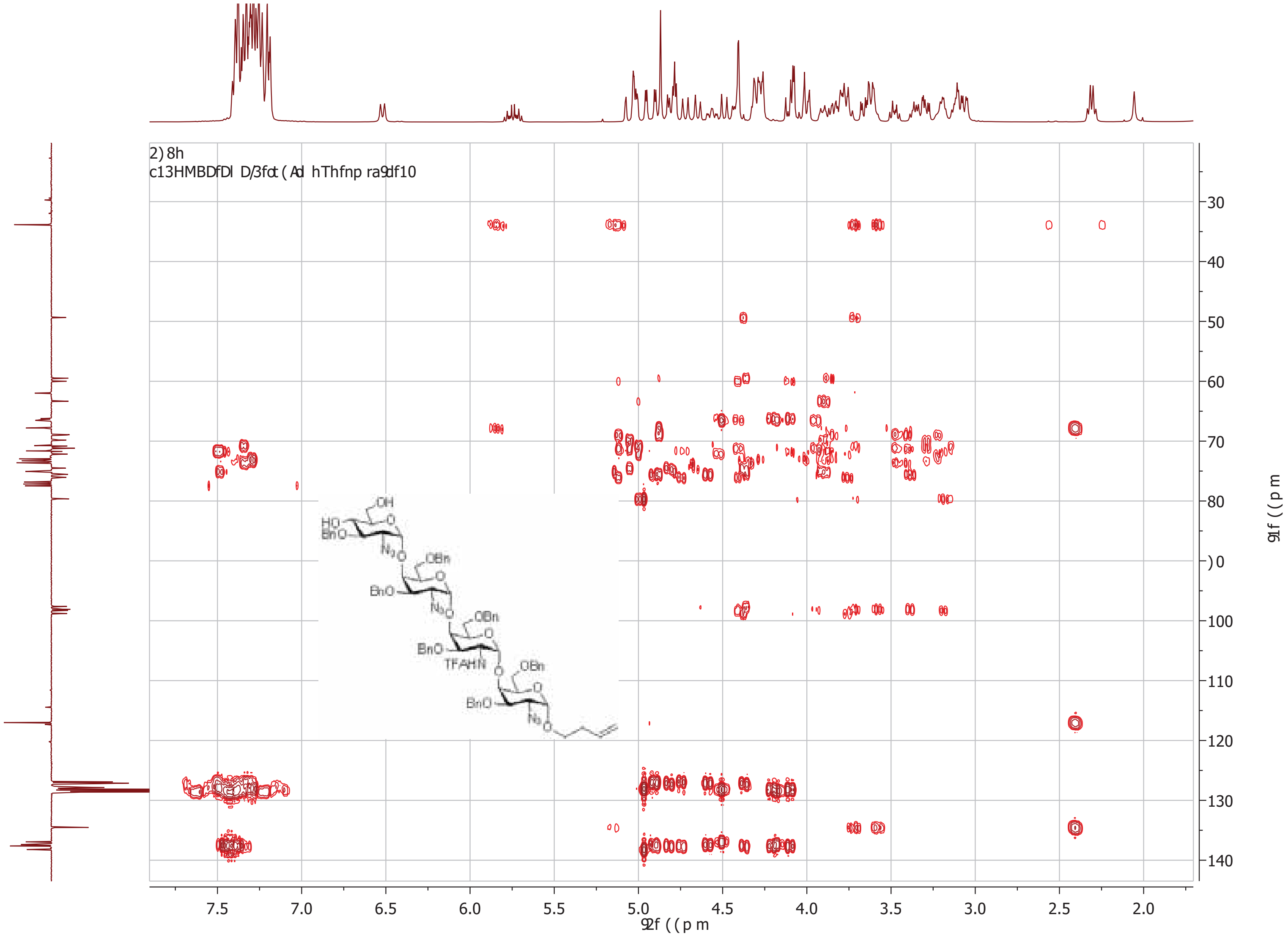
2) 8h
c13HMBDfDl D/3fct (Ad hThfnp ra9df10
OH
HO
BnO
N3
OBn
BnO
N3
OBn
BnO
TFAHN
OBn
BnO
N3
7.5
7.0
6.5
6.0
5.5
5.0
4.5
4.0
3.5
3.0
2.5
2.0
30
40
50
60
70
80
100
110
120
130
140

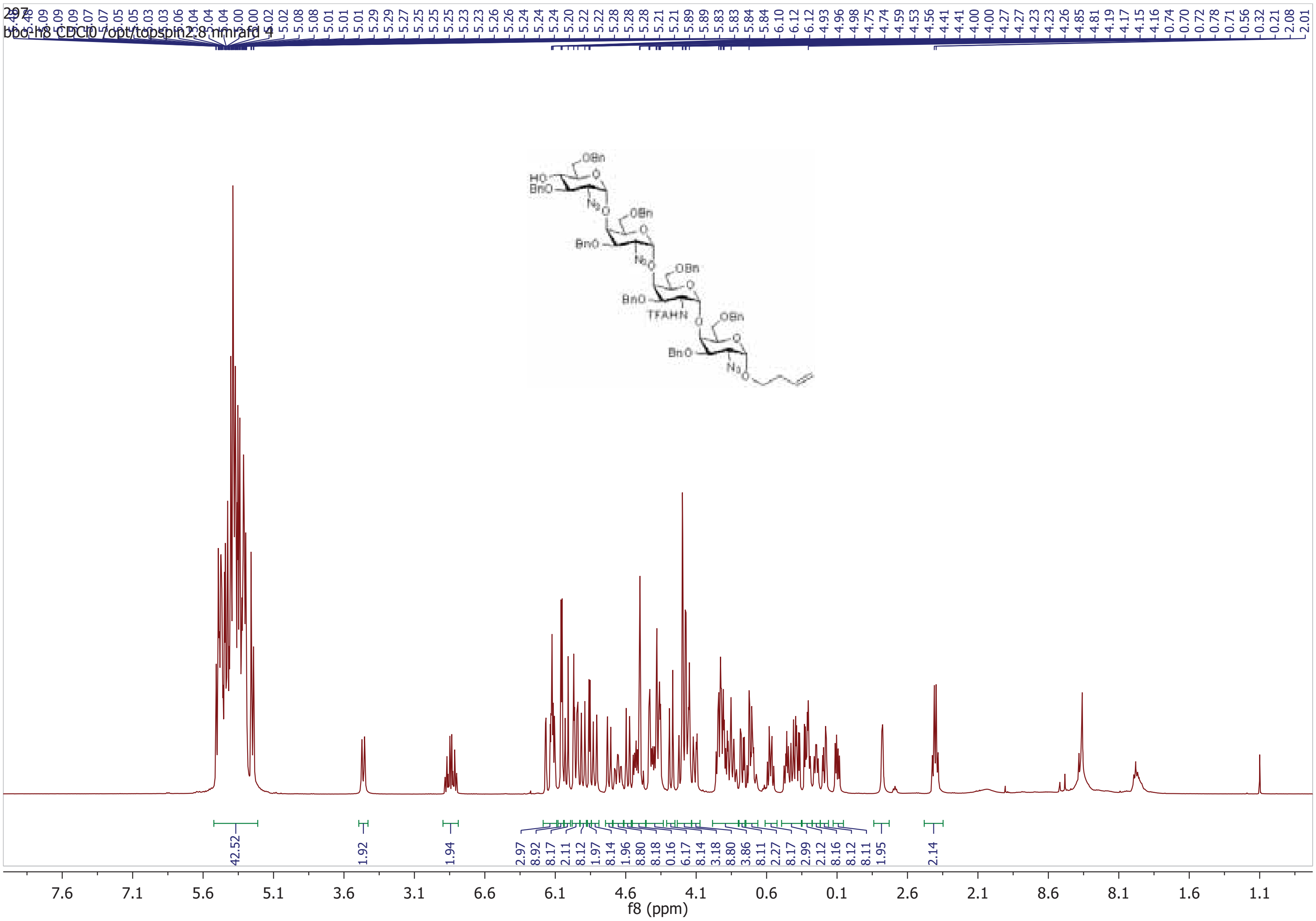
297
bbo-h8 CDCl0 /opt/topspin2.8 nmrafd 4
OBn
HO
BnO
N3
TFAHN
42.52
1.92
1.94
2.97
8.92
8.17
2.11
8.12
1.97
8.14
1.96
8.80
8.18
0.16
6.17
8.14
3.18
8.80
3.86
8.11
2.27
8.17
2.99
2.12
8.16
8.12
8.11
1.95
2.14
7.6
7.1
5.6
5.1
3.6
3.1
6.6
6.1
4.6
4.1
0.6
0.1
2.6
2.1
8.6
8.1
1.6
1.1
f8 (ppm)

297
bbo-c80-APT CDCl0 /opt/topspin2.8 nmrafd 4

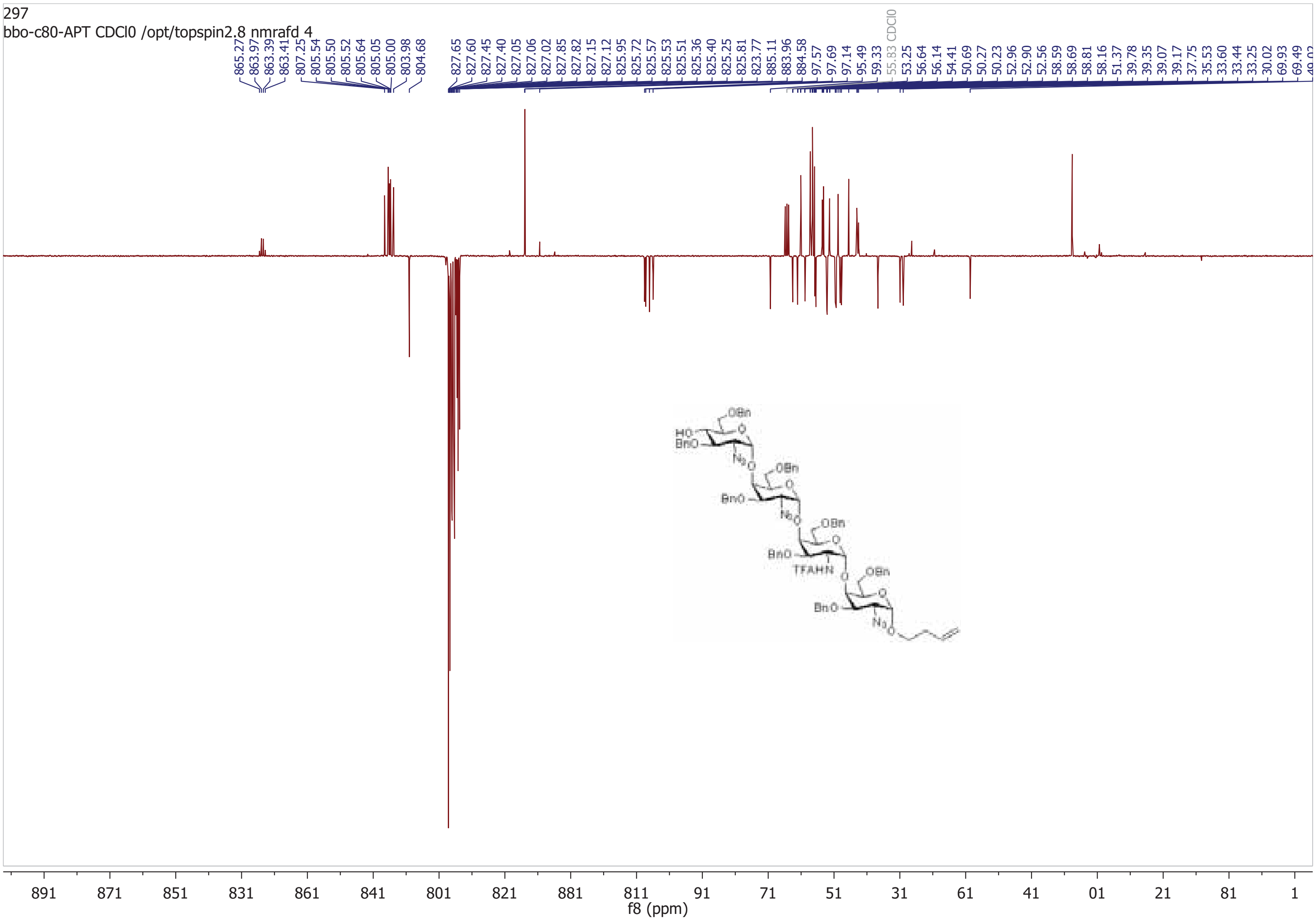

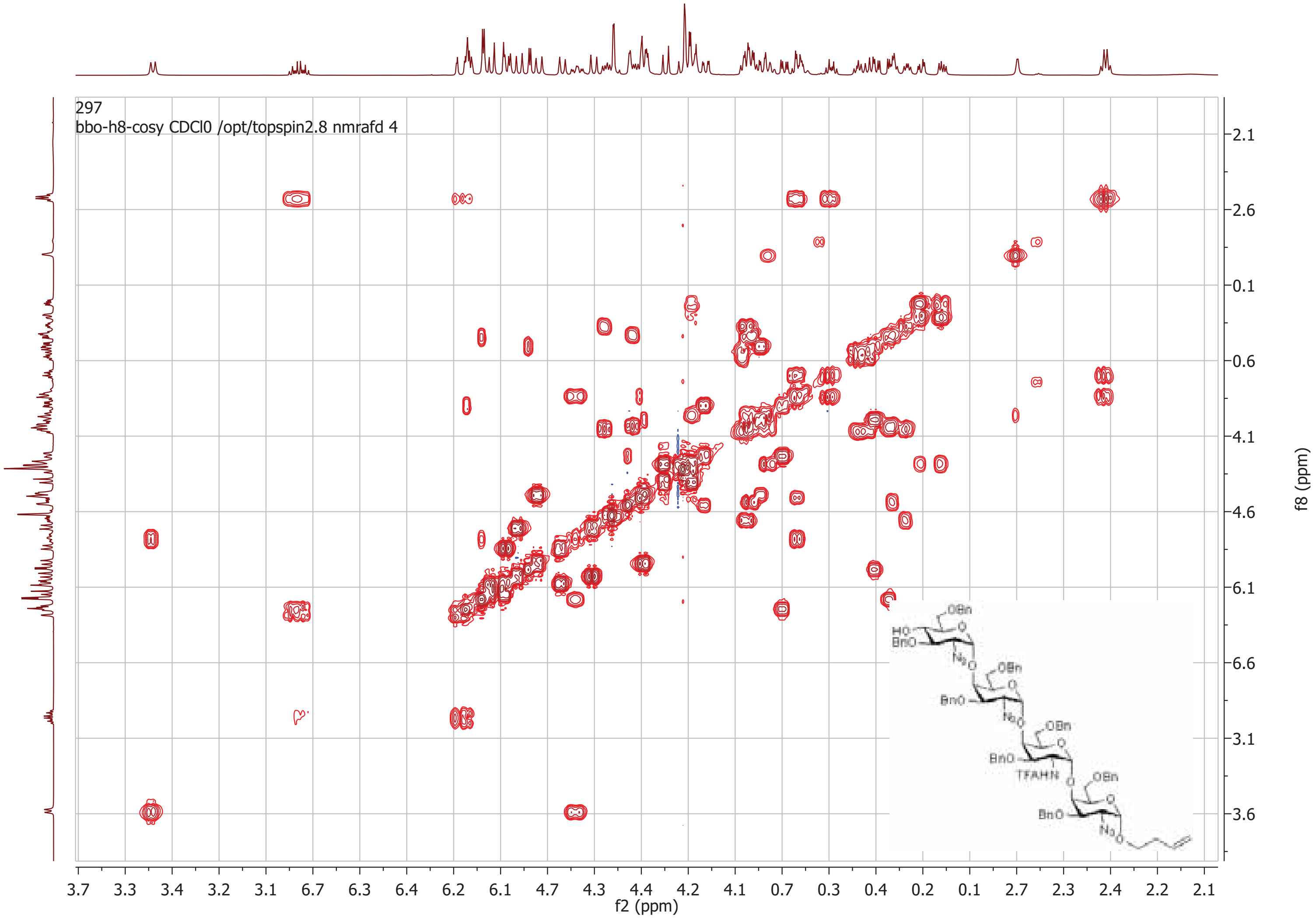
297
bbo-h8-cosy CDCl0 /opt/topspin2.8 nmrafd 4
f2 (ppm)
f8 (ppm)
OBn
HO
BnO
BnO
TFAHN

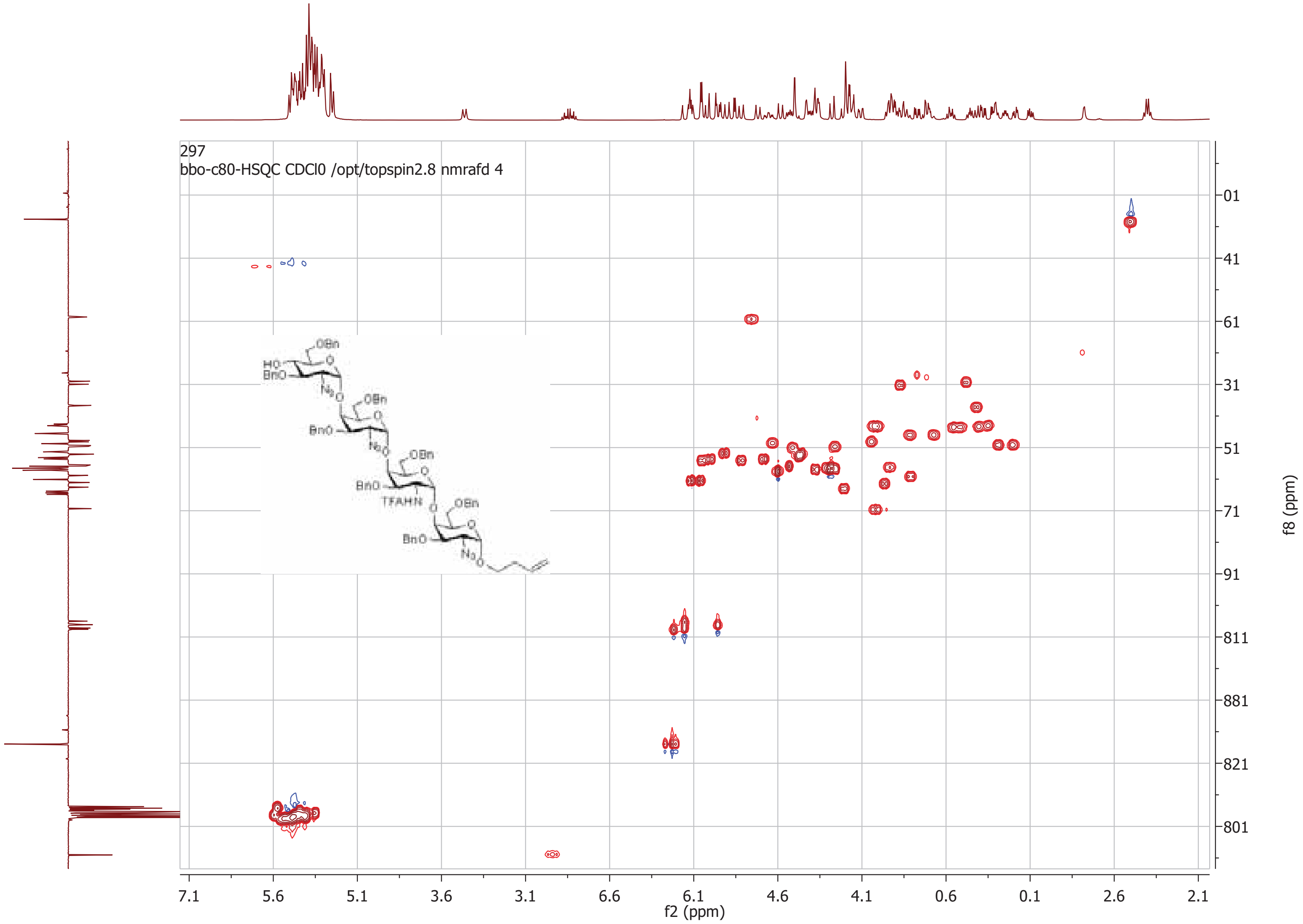
297
bbo-c80-HSQC CDCl0 /opt/topspin2.8 nmrafd 4
OBn
HO
BnO
N3
OBn
BnO
N3
OBn
BnO
TFAHN
OBn
BnO
N3
7.1
5.6
5.1
3.6
3.1
6.6
6.1
4.6
4.1
0.6
0.1
2.6
2.1
f2 (ppm)
01
41
61
31
51
71
91
811
881
821
801
f8 (ppm)

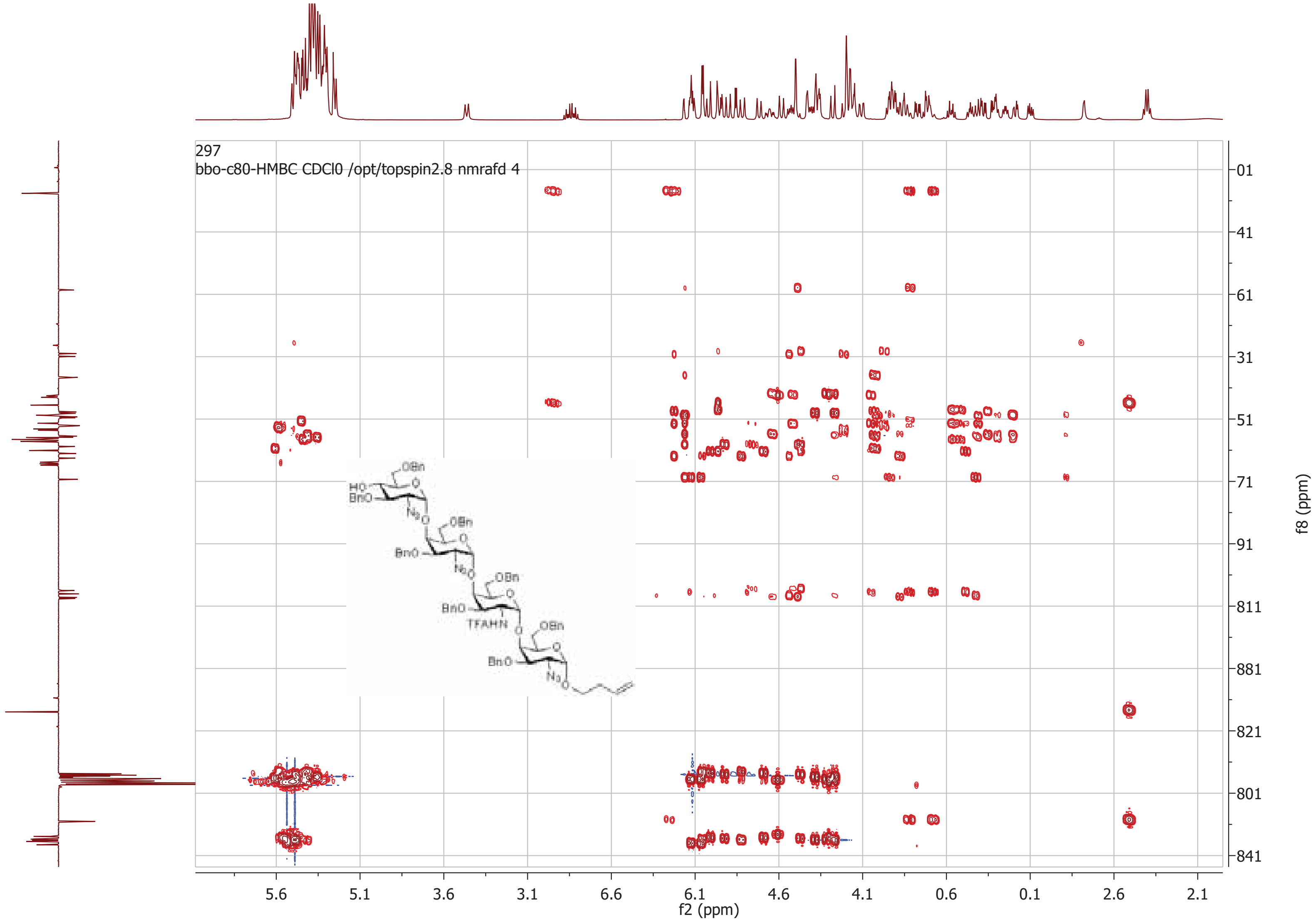

297
bbo-c80-HMBC CDCl0 /opt/topspin2.8 nmrafd 4
5.6
5.1
3.6
3.1
6.6
6.1
4.6
4.1
0.6
0.1
2.6
2.1
f2 (ppm)
01
41
61
31
51
71
91
811
881
821
801
841
f8 (ppm)
OBn
HO
BnO
N3
TFAHN

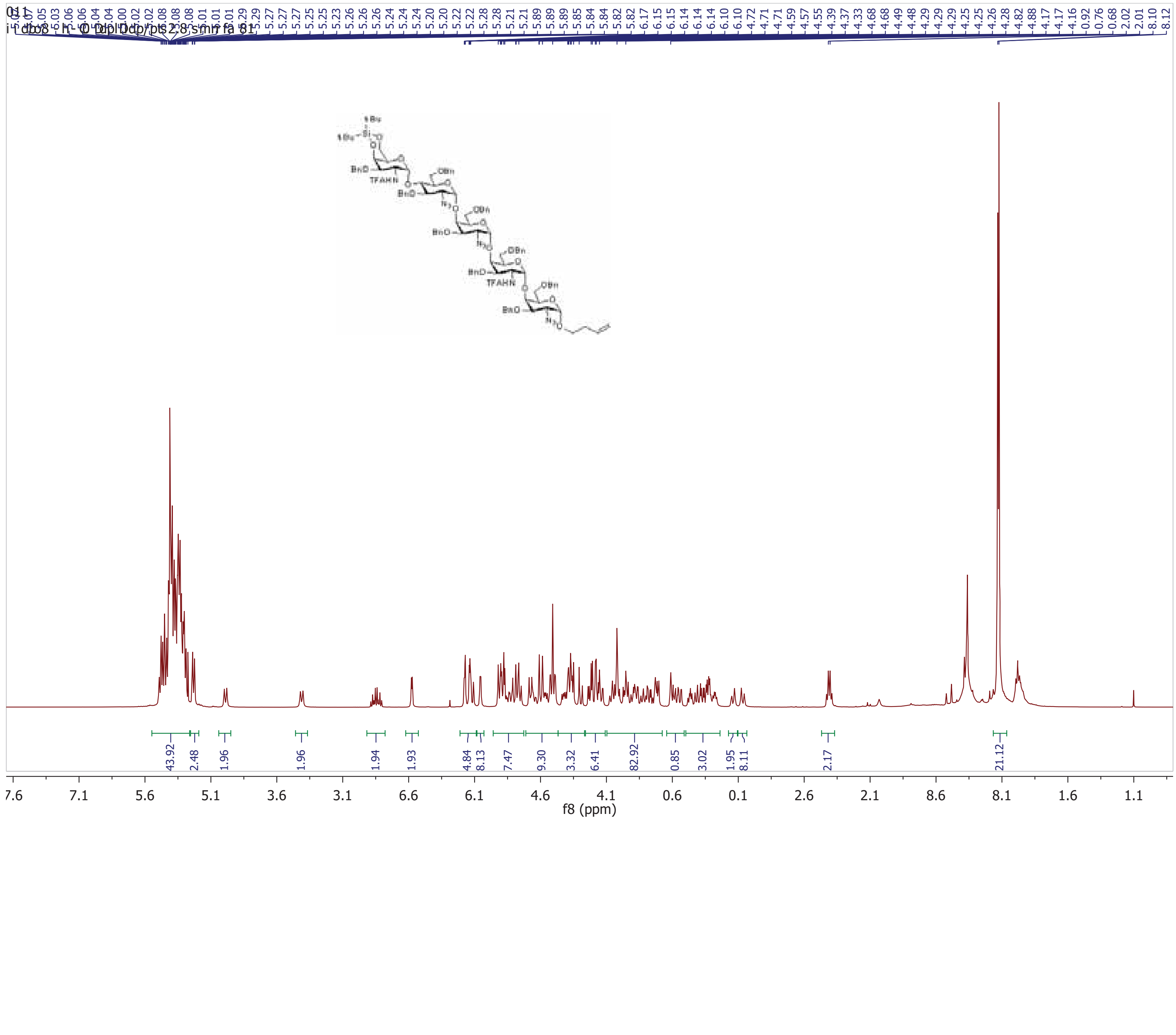

tBu
tBu
Si
BnO
TFAHN
OBn
BnO
N3
OBn
BnO
N3
OBn
BnO
TFAHN
OBn
BnO
N3
43.92
2.48
1.96
1.96
1.94
1.93
4.84
8.13
7.47
9.30
3.32
6.41
82.92
0.85
3.02
1.95
8.11
2.17
21.12
7.6
7.1
5.6
5.1
3.6
3.1
6.6
6.1
4.6
4.1
0.6
0.1
2.6
2.1
8.6
8.1
1.6
1.1
f8 (ppm)

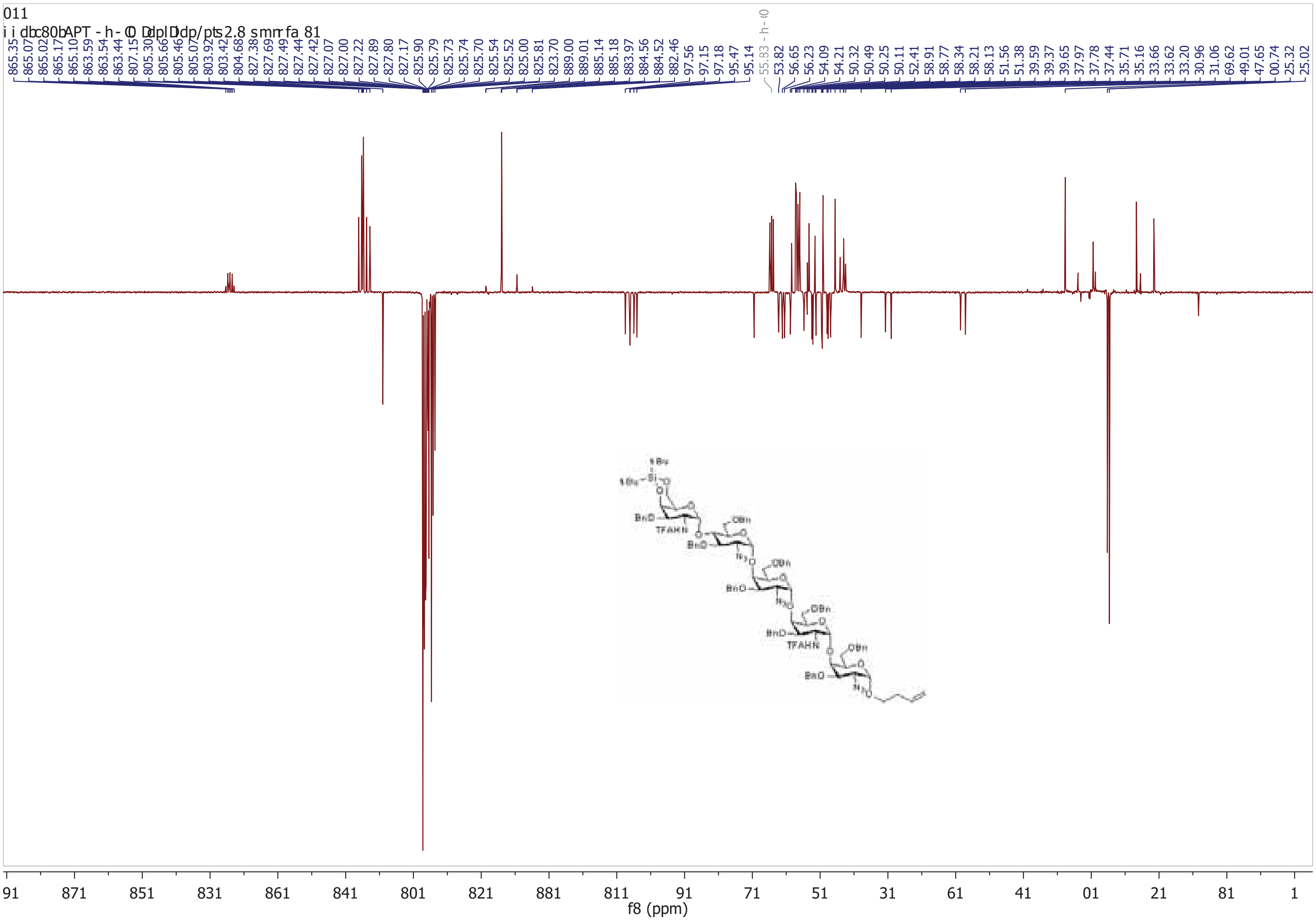
011
i i dbc80bAPT - h- Ф Ddpl Ddp/pts2.8 smrr fa 81
55.83 - h- (0
91 871 851 831 861 841 801 821 881 811 91 71 51 31 61 41 01 21 81 1
f8 (ppm)
tBu
tBu
Si
BnO
TFAHN
OBn
BnO
N3
OBn
BnO
N3
OBn
BnO
TFAHN
OBn
BnO
N3

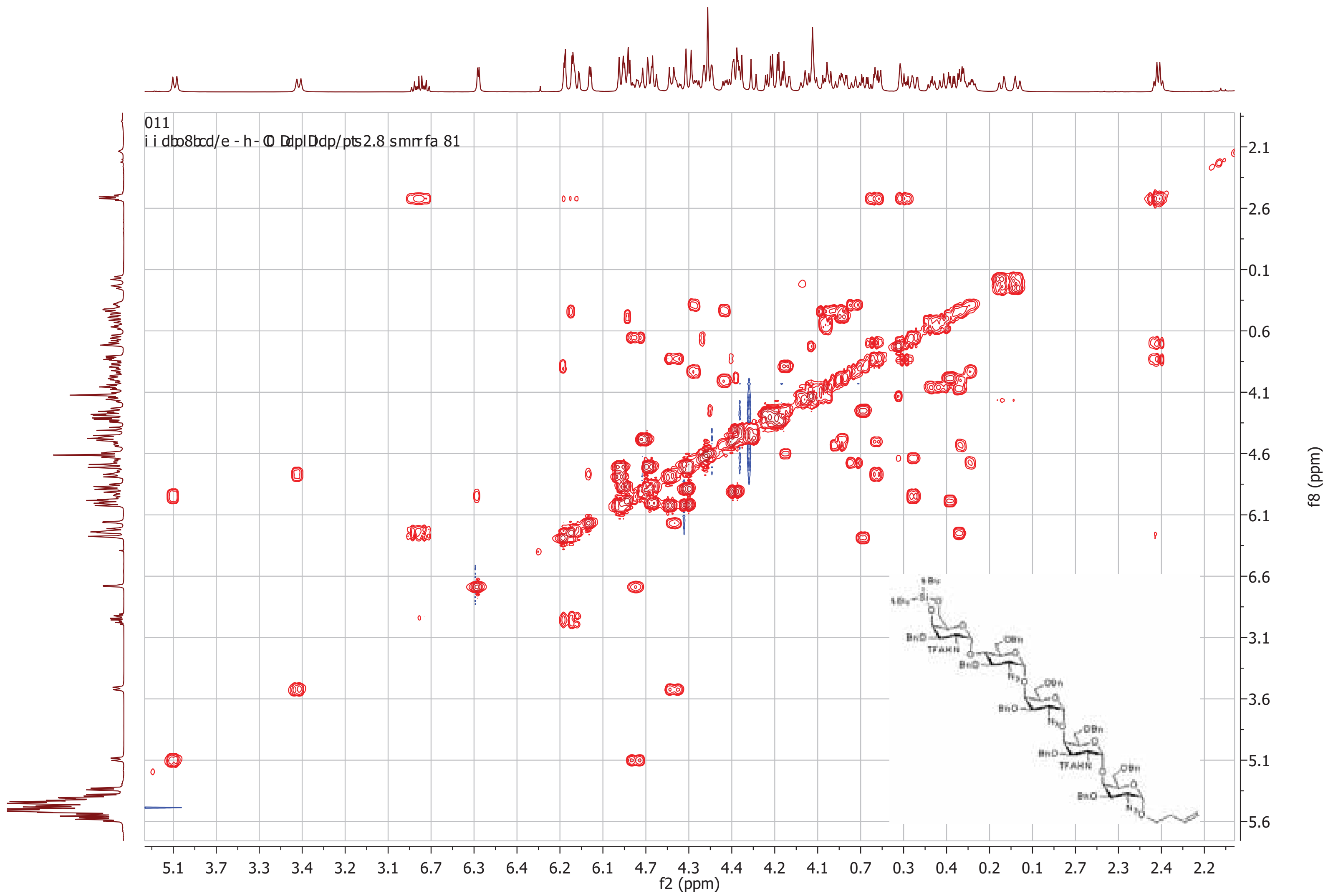
011
i i db o8bcd/e - h- C D dplDdp/pts2.8 smrrfa 81
f2 (ppm)
f8 (ppm)
BnO
TFAHN
OBn
N3

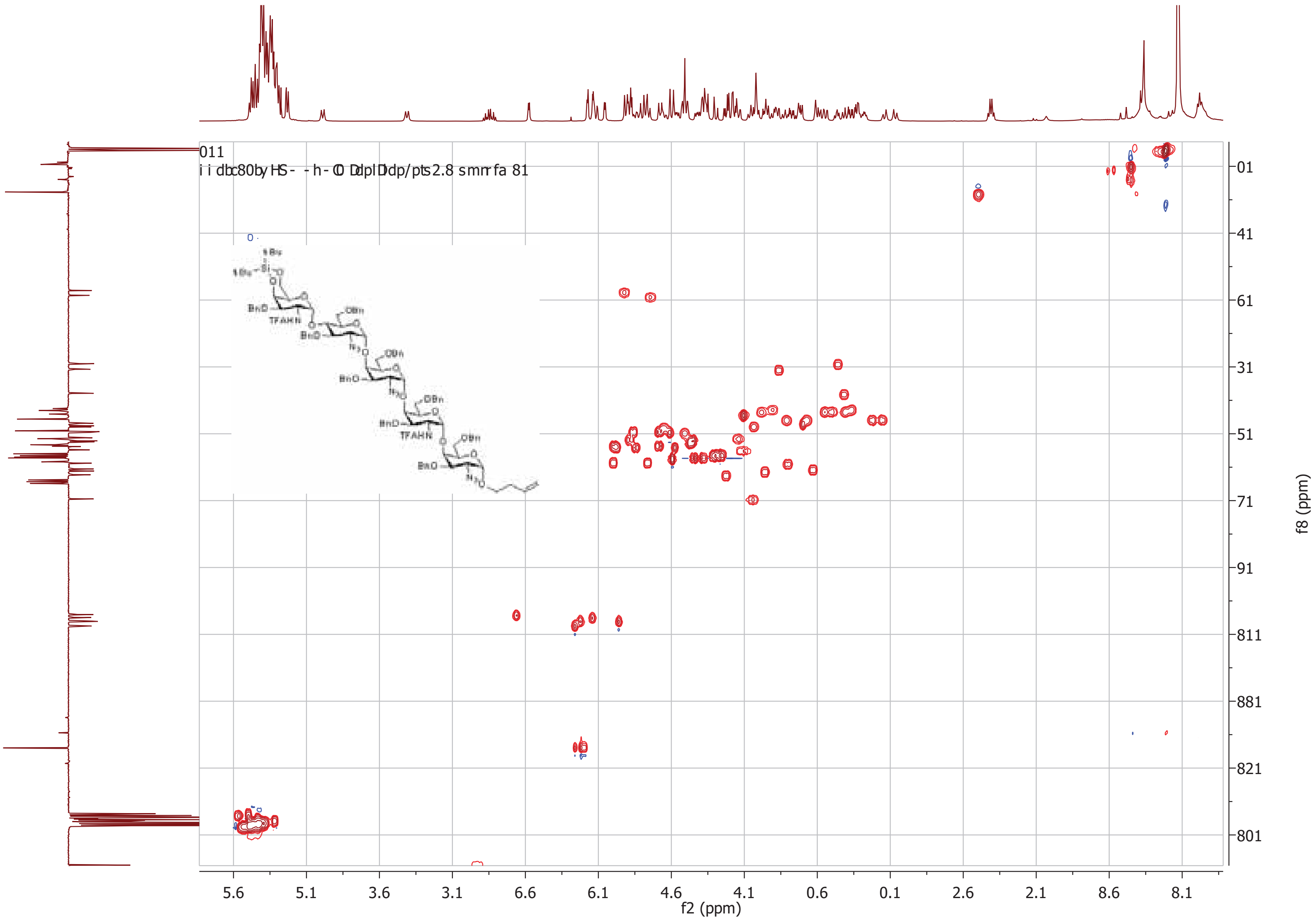

011
i i dbc80by HS- -h-O DdplDdp/pts2.8 smrrfa 81
5.6
5.1
3.6
3.1
6.6
6.1
4.6
4.1
0.6
0.1
2.6
2.1
8.6
8.1
f2 (ppm)
01
41
61
31
51
71
91
811
881
821
801
f8 (ppm)

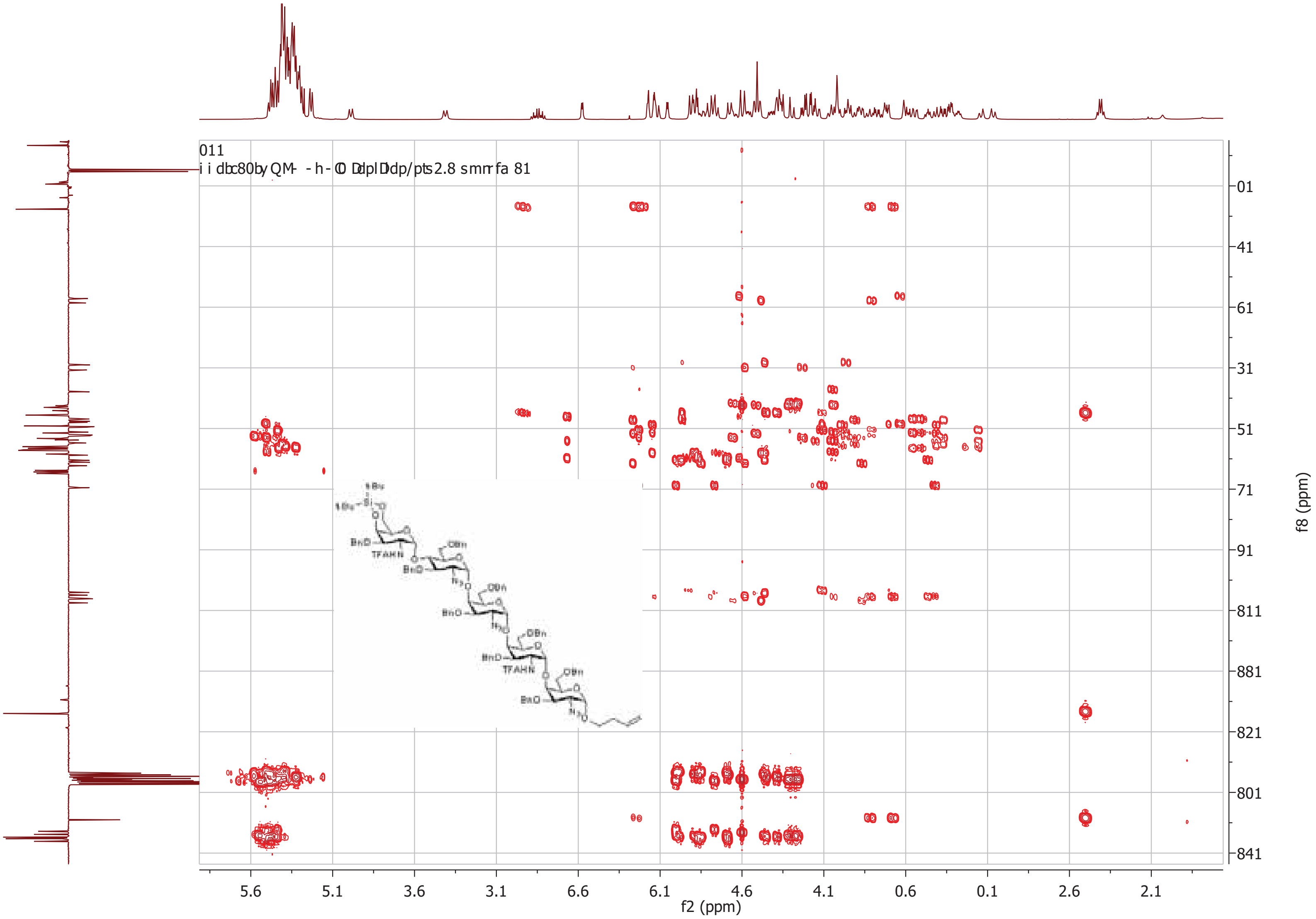
f2 (ppm)
f8 (ppm)
BnO
TFAHN
OBn

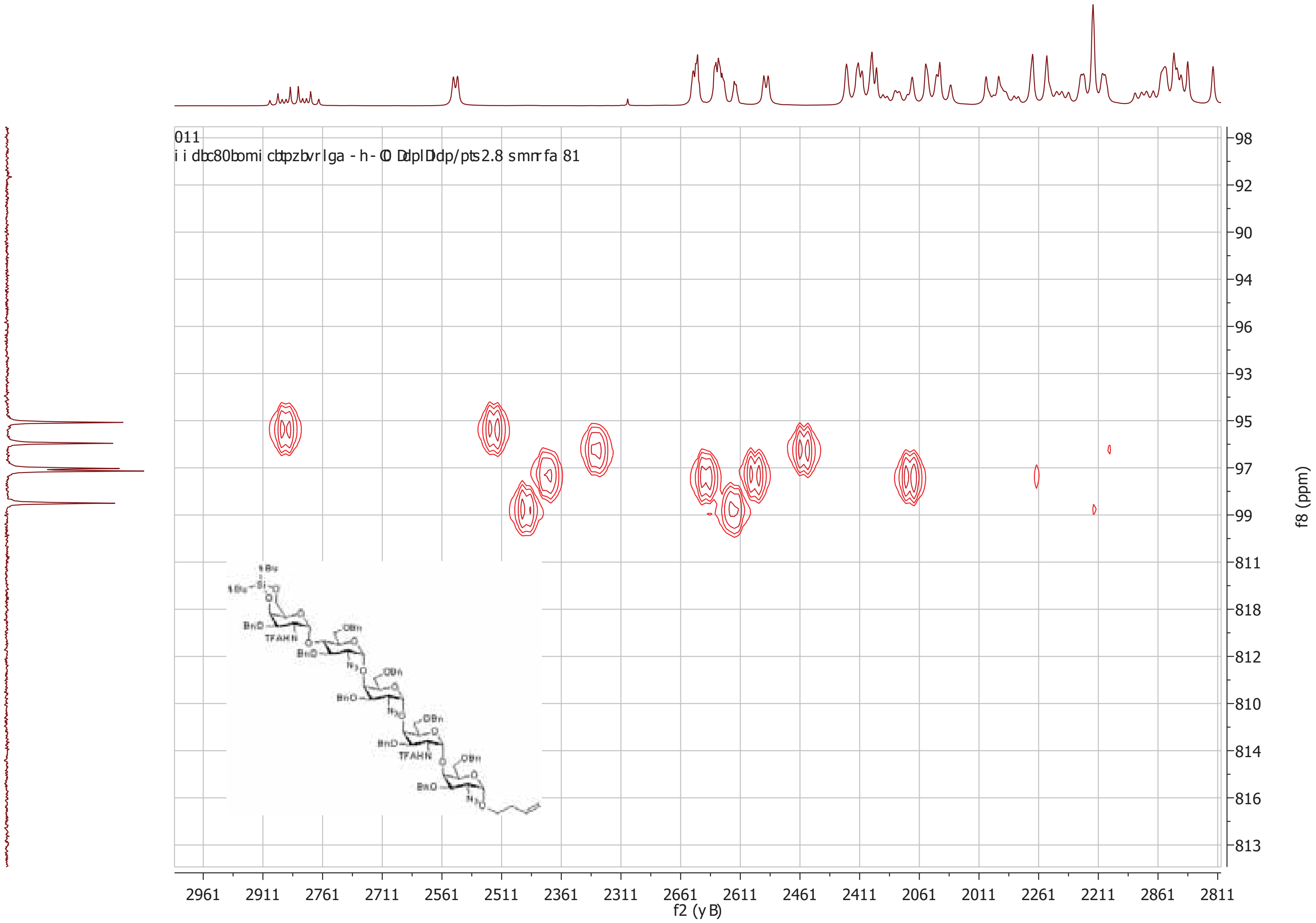

011
f2 (y B)
f8 (ppm)
TFAHN
BnO
OBn

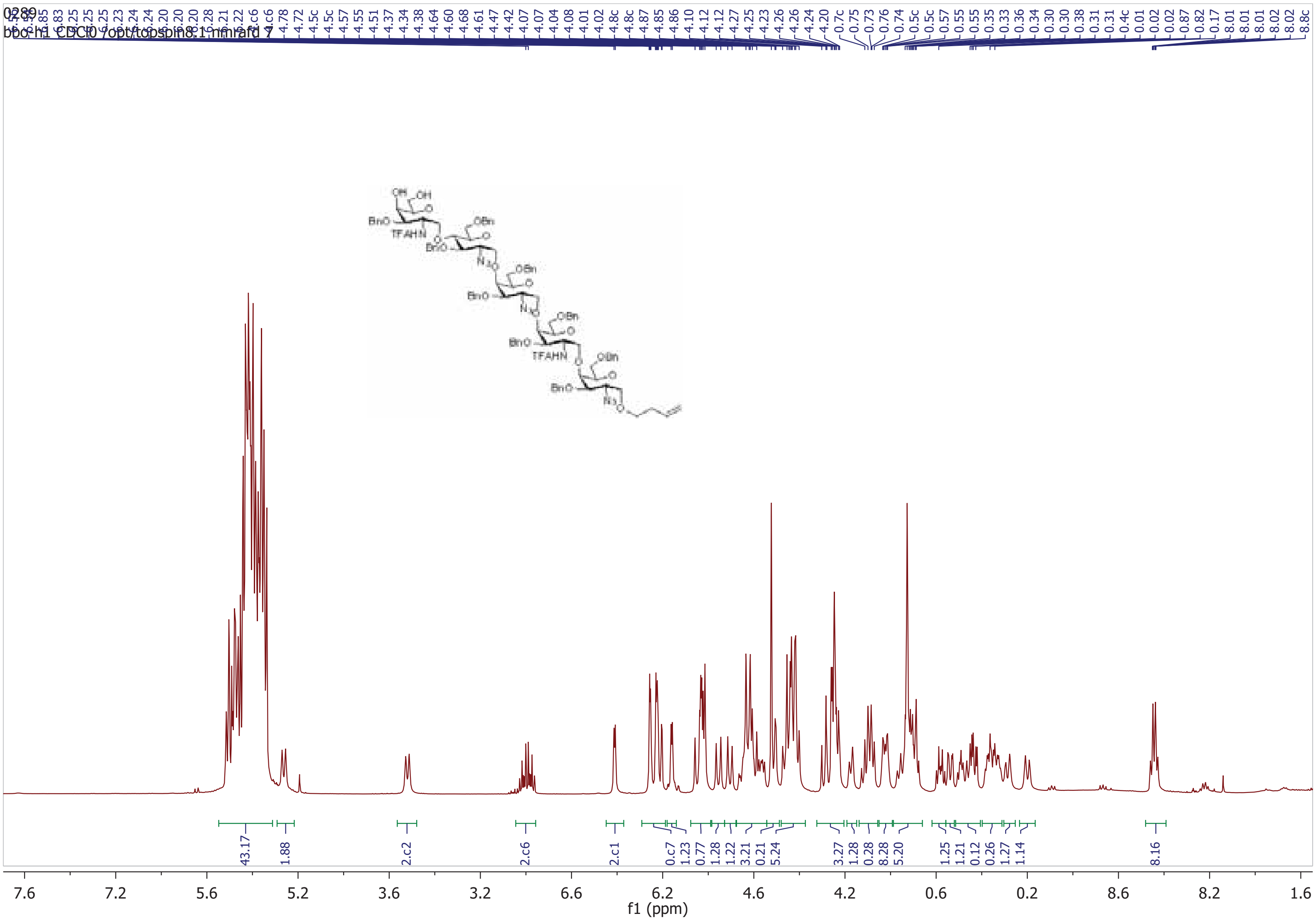
f1 (ppm)
7.6
7.2
5.6
5.2
3.6
3.2
6.6
6.2
4.6
4.2
0.6
0.2
8.6
8.2
1.6
43.17
1.88
2.c2
2.c6
2.c1
8.16
BnO
TFAHN
N3
OBn
OH

0289
bbo-A10-9PT CDCl0 /opt/topspin8.1 nmrafd 7

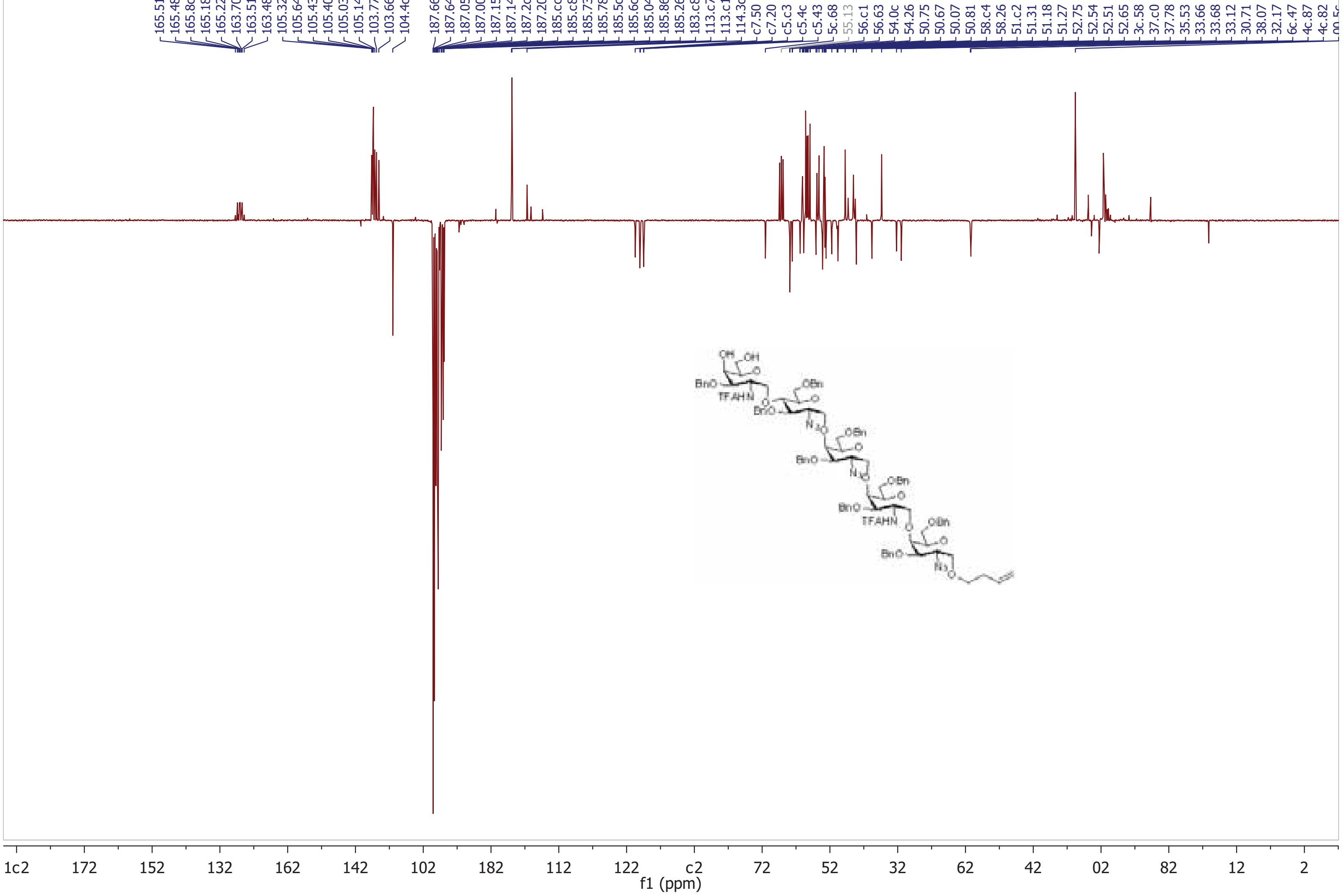

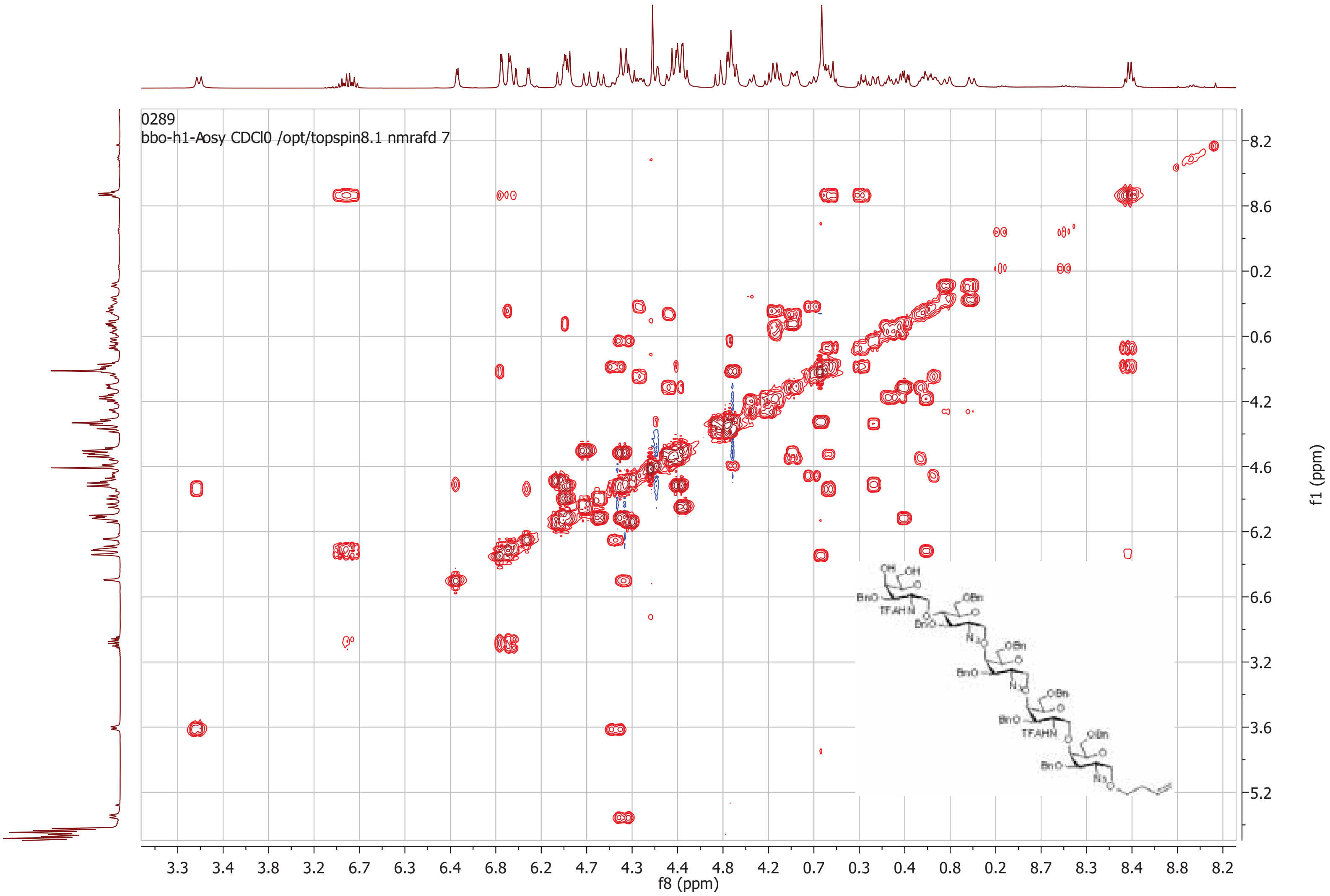

0289
bbo-h1-Aosy CDCl0 /opt/topspin8.1 nmrafd 7
f8 (ppm)
f1 (ppm)

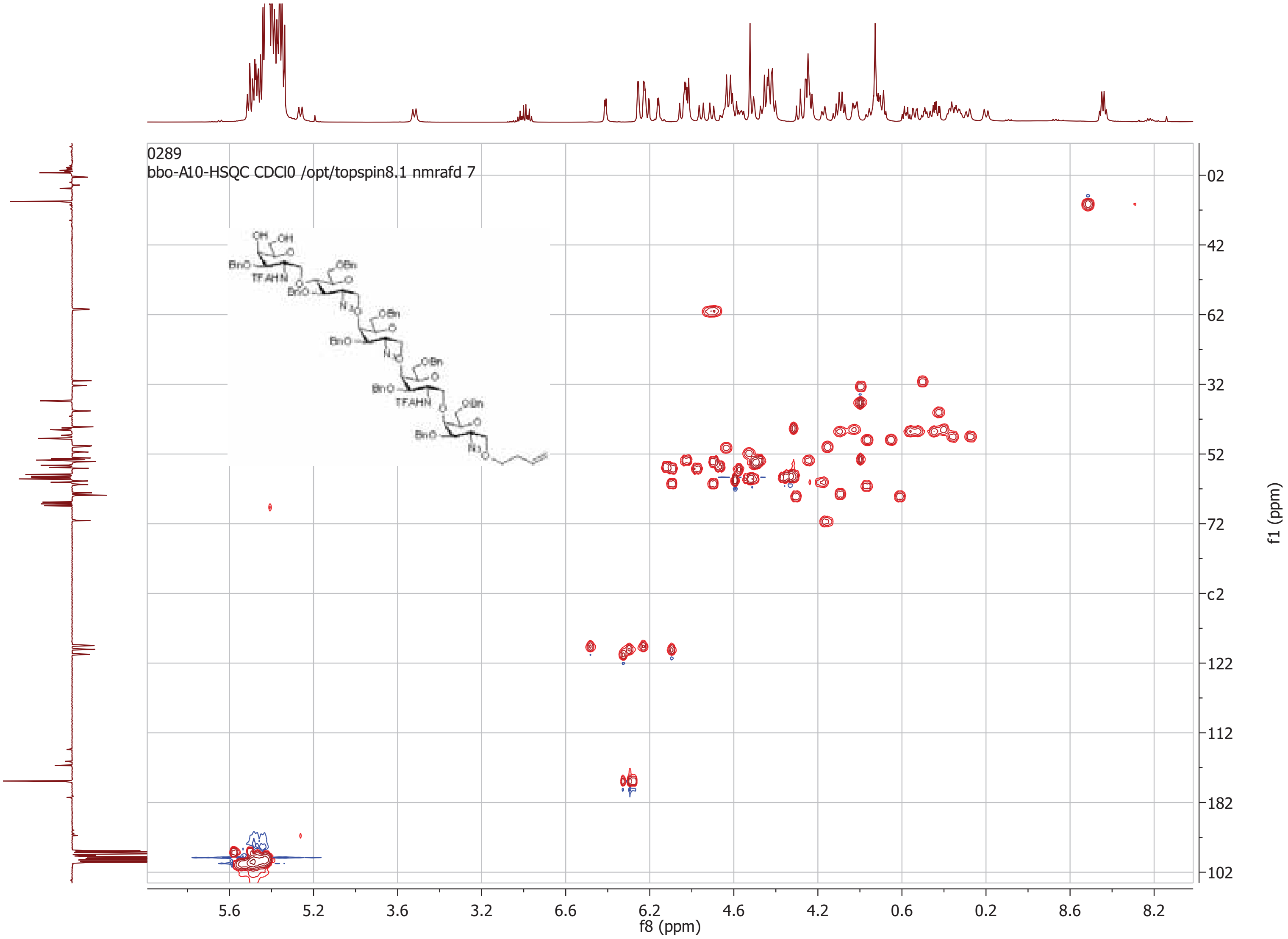
0289
bbo-A10-HSQC CDCl0 /opt/topspin8.1 nmrafd 7
f8 (ppm)
f1 (ppm)

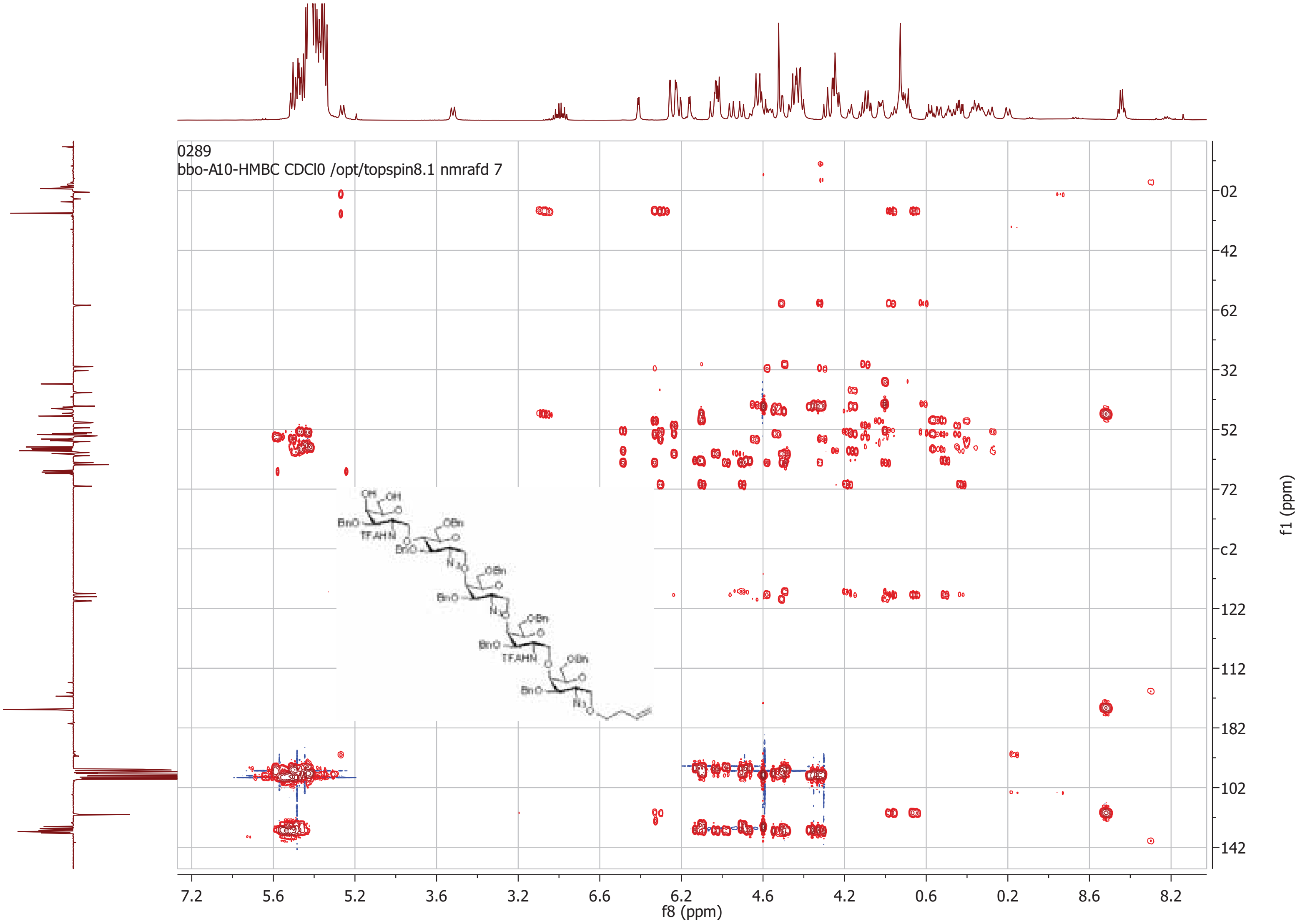
0289
bbo-A10-HMBC CDCl0 /opt/topspin8.1 nmrafd 7
f8 (ppm)
f1 (ppm)
7.2
5.6
5.2
3.6
3.2
6.6
6.2
4.6
4.2
0.6
0.2
8.6
8.2
02
42
62
32
52
72
c2
122
112
182
102
142
OH
OH
BnO
TFAHN
OBn
N3
TFAHN

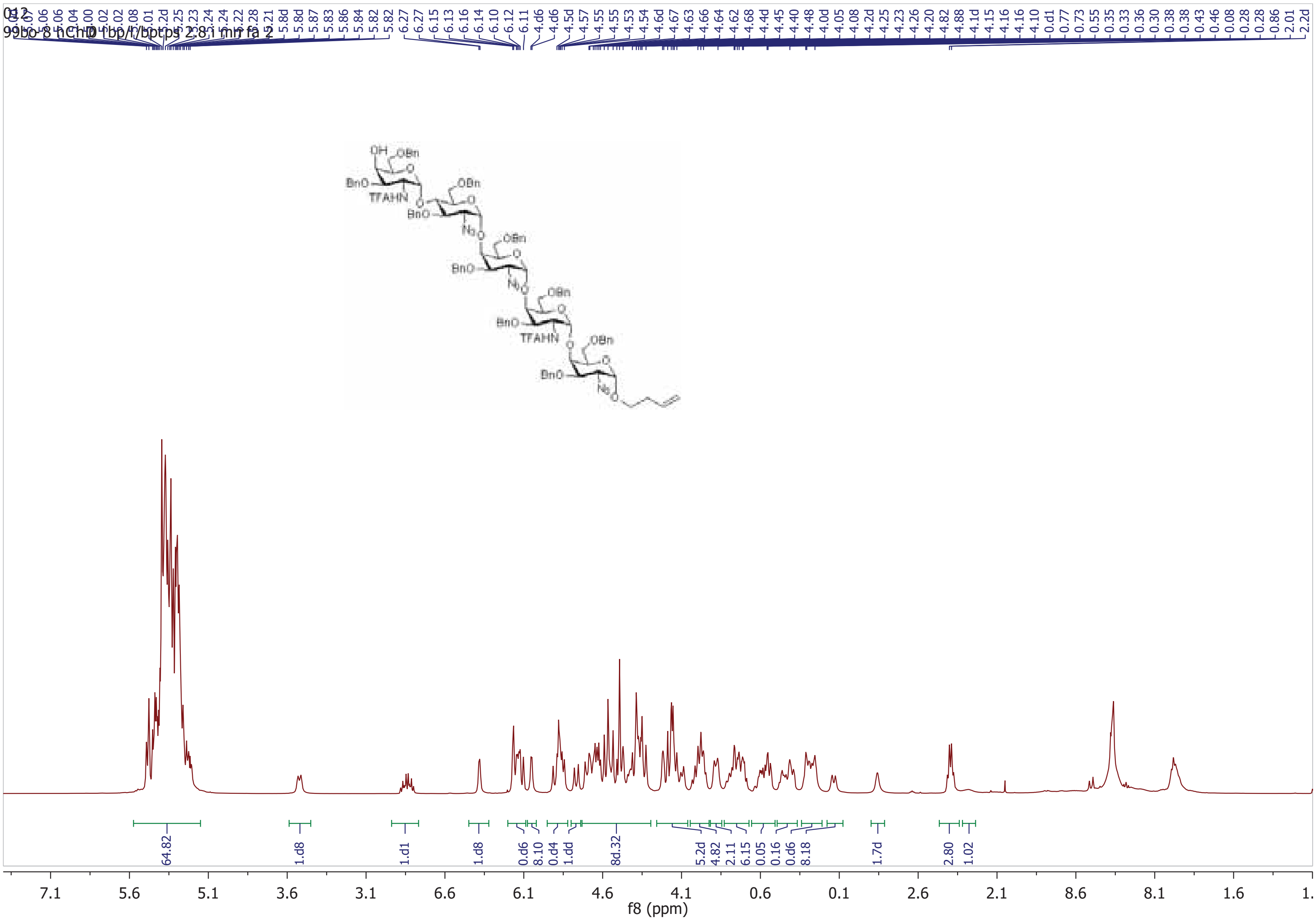

BnO
TFAHN
OBn
OH
N3
64.82
1.d8
1.d1
1.d8
0.d6
8.10
0.d4
1.dd
8d.32
5.2d
4.82
2.11
6.15
0.05
0.16
0.d6
8.18
1.7d
2.80
1.02
7.1
5.6
5.1
3.6
3.1
6.6
6.1
4.6
4.1
0.6
0.1
2.6
2.1
8.6
8.1
1.6
f8 (ppm)

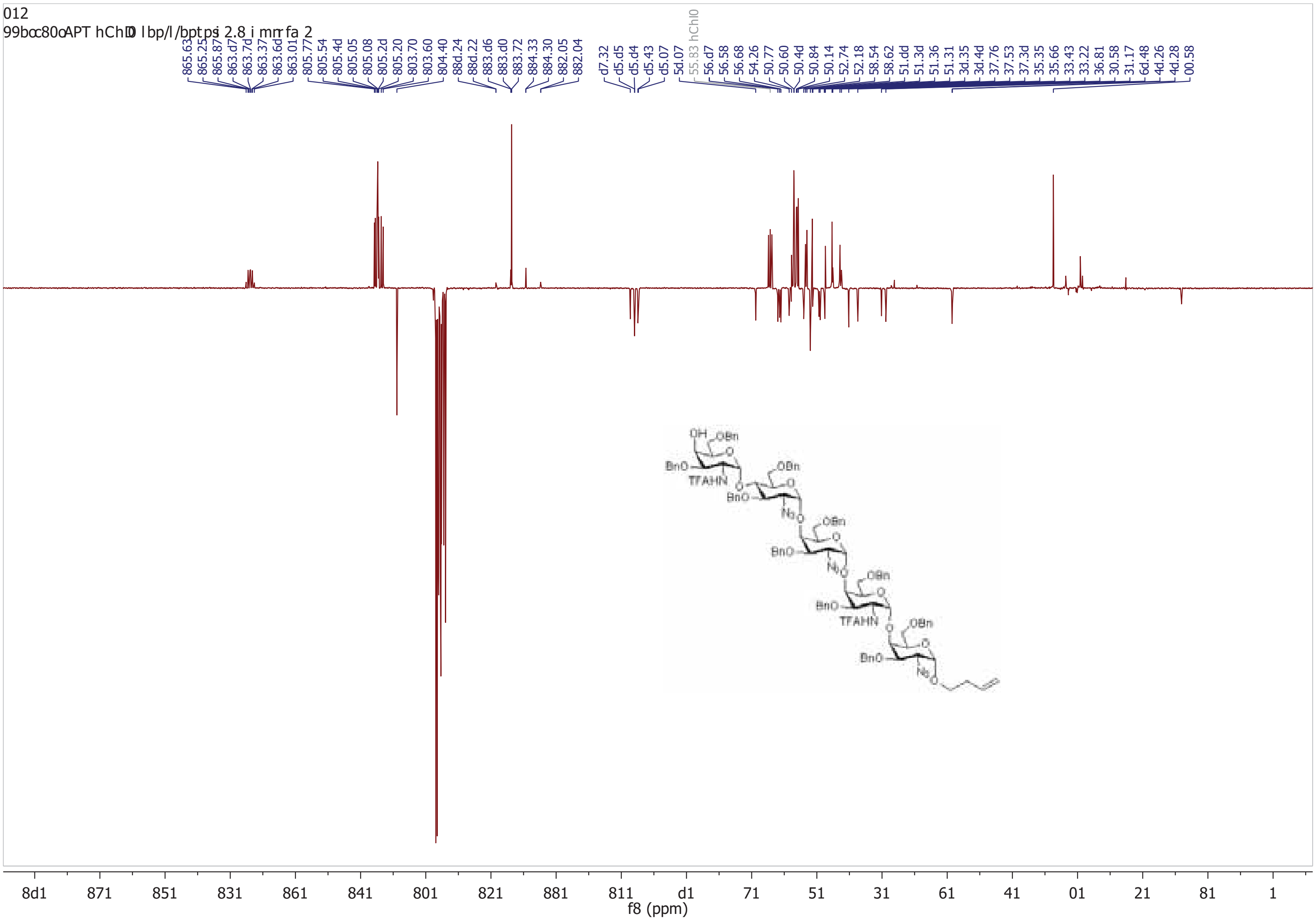
012
APT
f8 (ppm)
BnO
TFAHN
OBn
N3

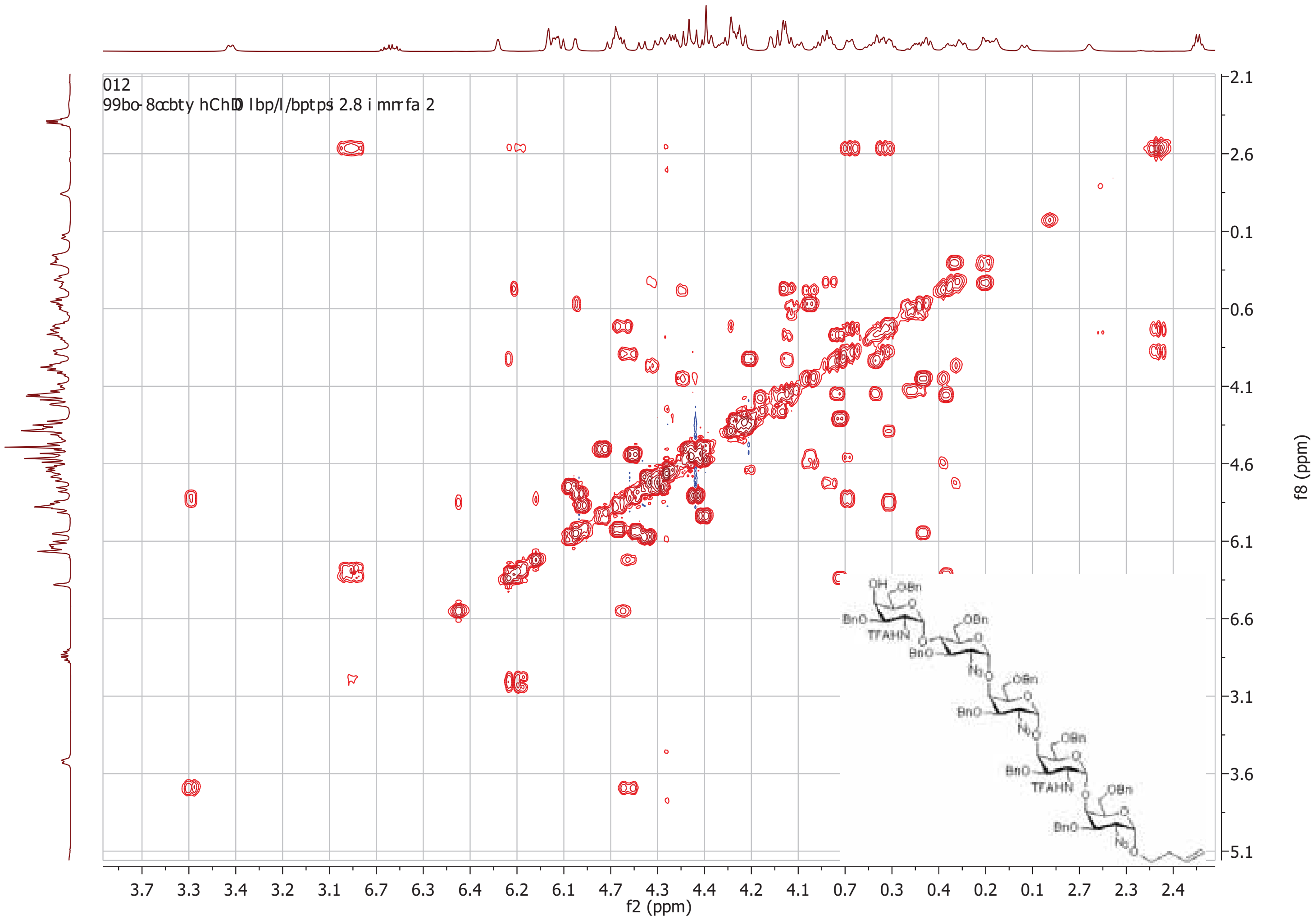
012
99bo-8ccbty hChD lbp/l/bptpsi 2.8 i mrr fa 2
f2 (ppm)
f8 (ppm)
OH
OBn
BnO
TFAHN
N3

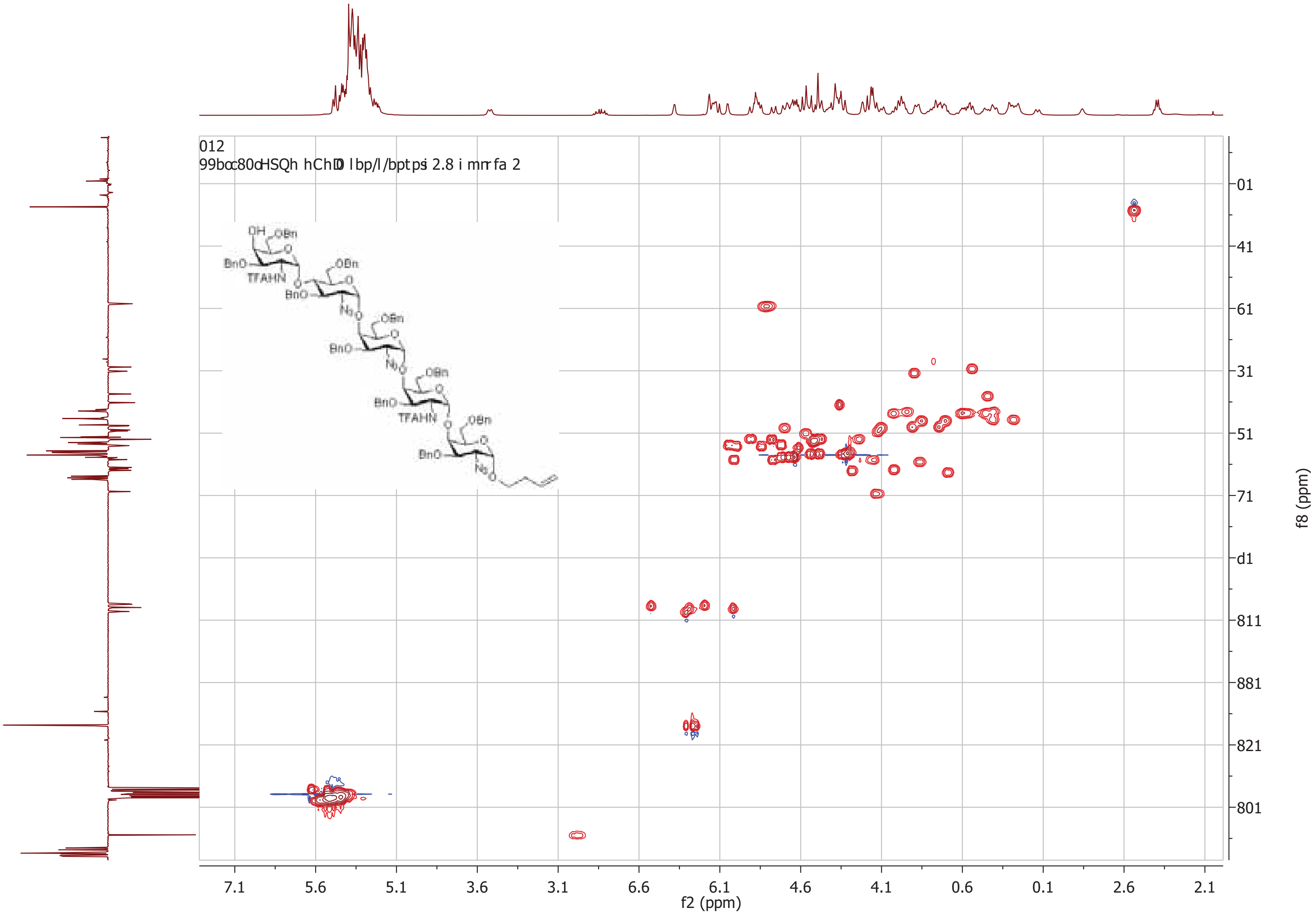
012
99bcc80cHSQh hChD lbp/l/bptps 2.8 i mrrfa 2
OH
OBn
BnO
TFAHN
N3
f2 (ppm)
f8 (ppm)

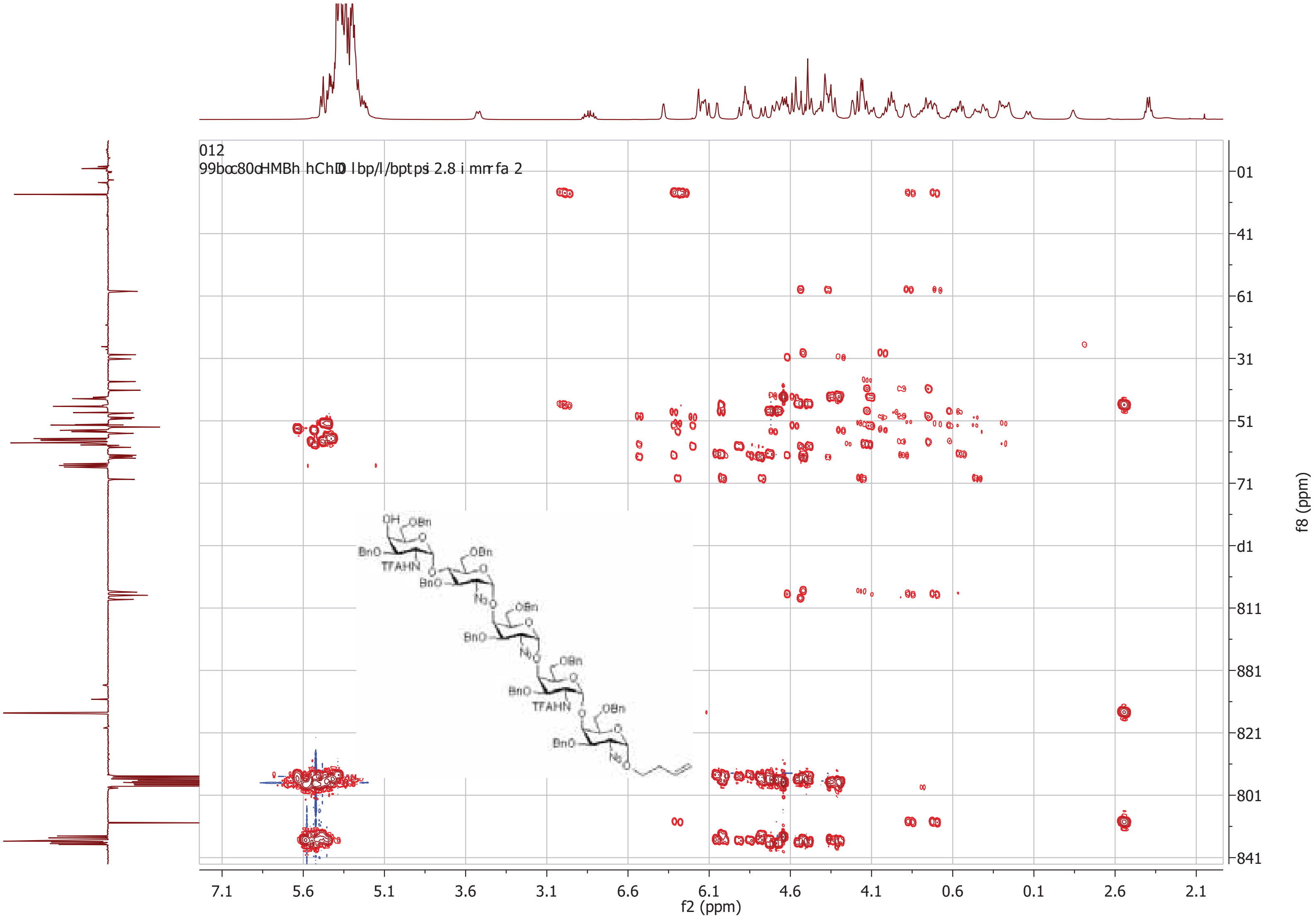
012
99bcc80cHMBh hChD lbp/l/bptpsi 2.8 i mmfa 2
f2 (ppm)
f8 (ppm)
OH
OBn
BnO
TFAHN
N3
OBn
BnO
N3
BnO
TFAHN
BnO
N3

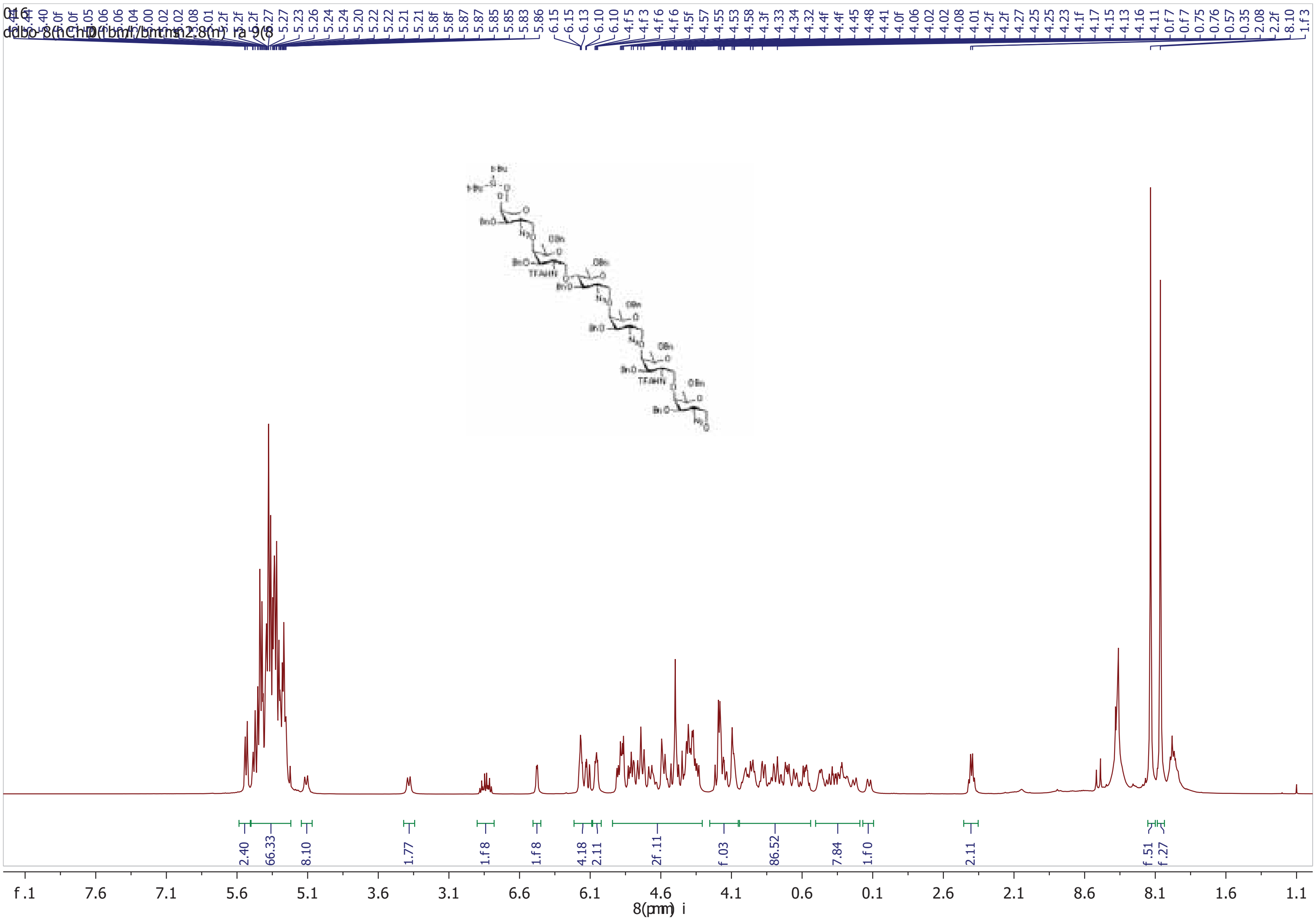

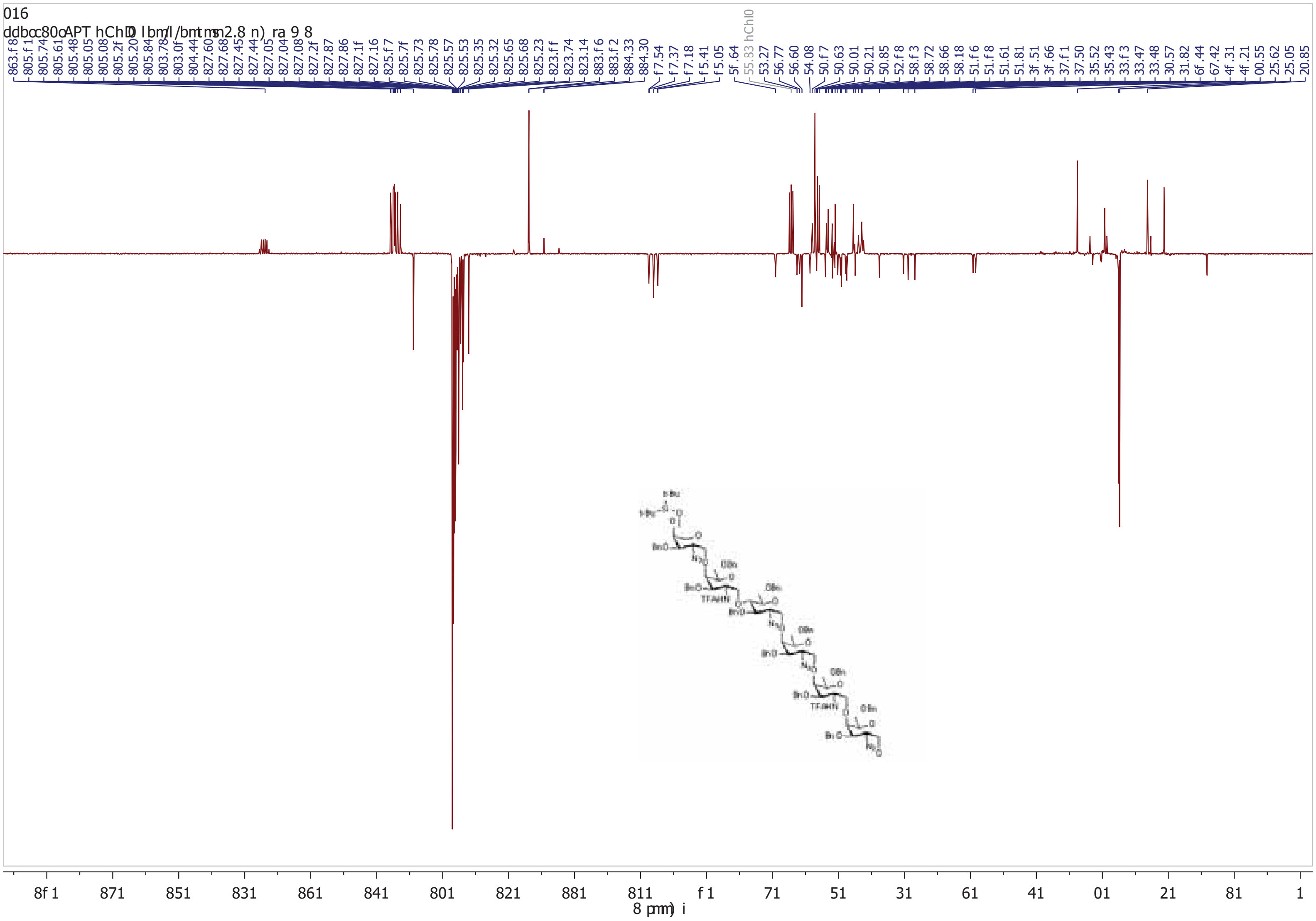
016
ddbo c80o APT hChl0 lbm/l/bmt ms 2.8 n) ra 9 8
863.f 8
805.f 1
805.74
805.61
805.48
805.05
805.08
805.2f
805.20
805.84
803.78
803.0f
804.44
827.60
827.68
827.45
827.44
827.05
827.04
827.08
827.2f
827.87
827.86
827.1f
827.16
825.7f
825.7f
825.73
825.78
825.57
825.53
825.35
825.32
825.65
825.68
825.23
823.f f
823.74
823.14
883.f 6
883.f 2
884.33
884.30
f 7.54
f 7.37
f 7.18
f 5.41
f 5.05
5f. 64
55.83 hChl0
53.27
56.77
56.60
54.08
50.f 7
50.63
50.01
50.21
50.85
52.f 8
58.f 3
58.72
58.66
58.18
51.f 6
51.f 8
51.61
51.81
3f. 51
3f. 66
37.f 1
37.50
35.52
35.43
33.f 3
33.47
33.48
30.57
31.82
6f. 44
67.42
4f. 31
4f. 21
00.55
25.62
25.05
20.85
8f 1
871
851
831
861
841
801
821
881
811
f 1
71
51
31
61
41
01
21
81
1
8 pmm) i

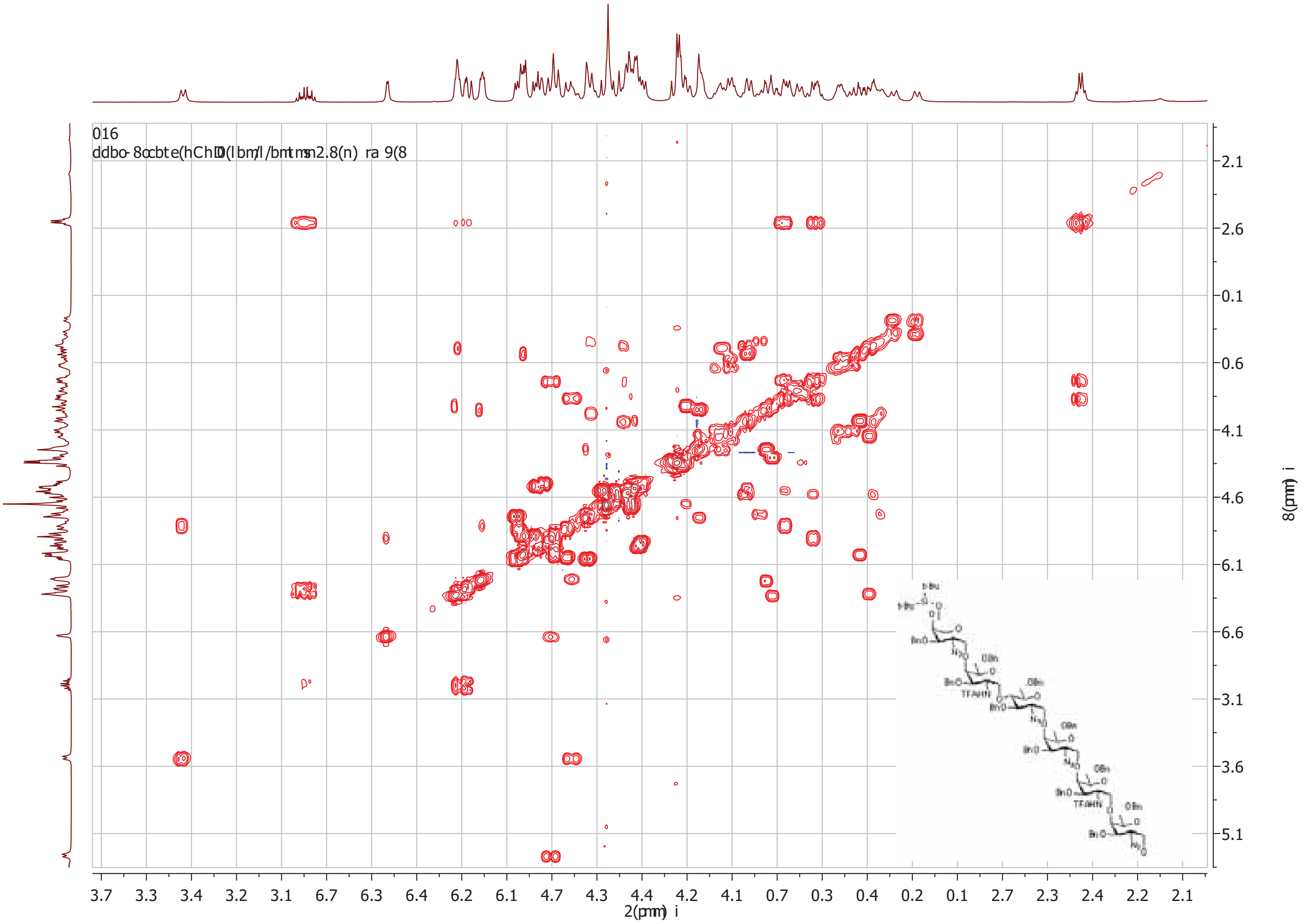
016
ddbo- 8ccbte(hChD0(lbm/l/bmtms2.8(n) ra 9(8
2(pmm) i
8(pmm) i

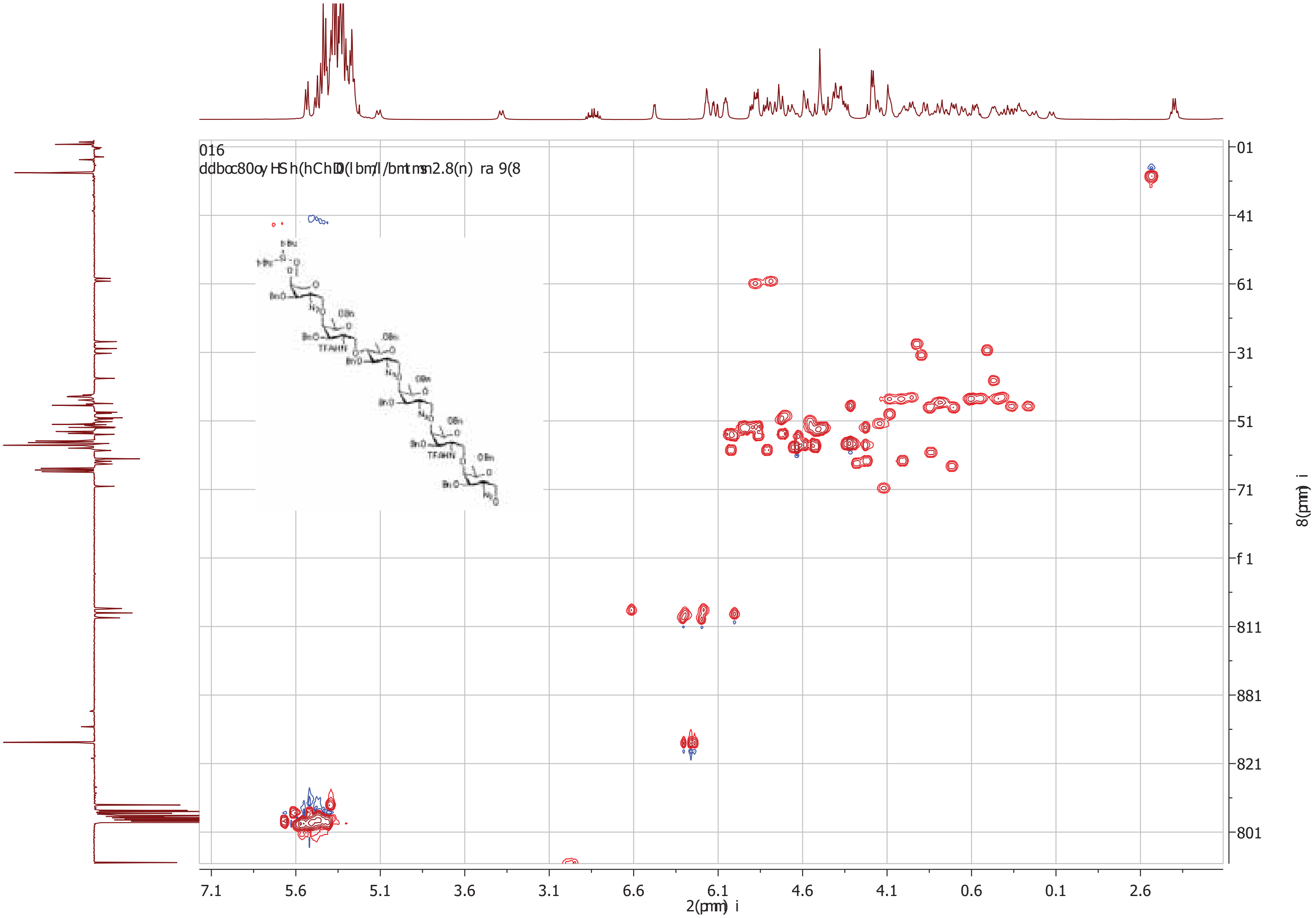

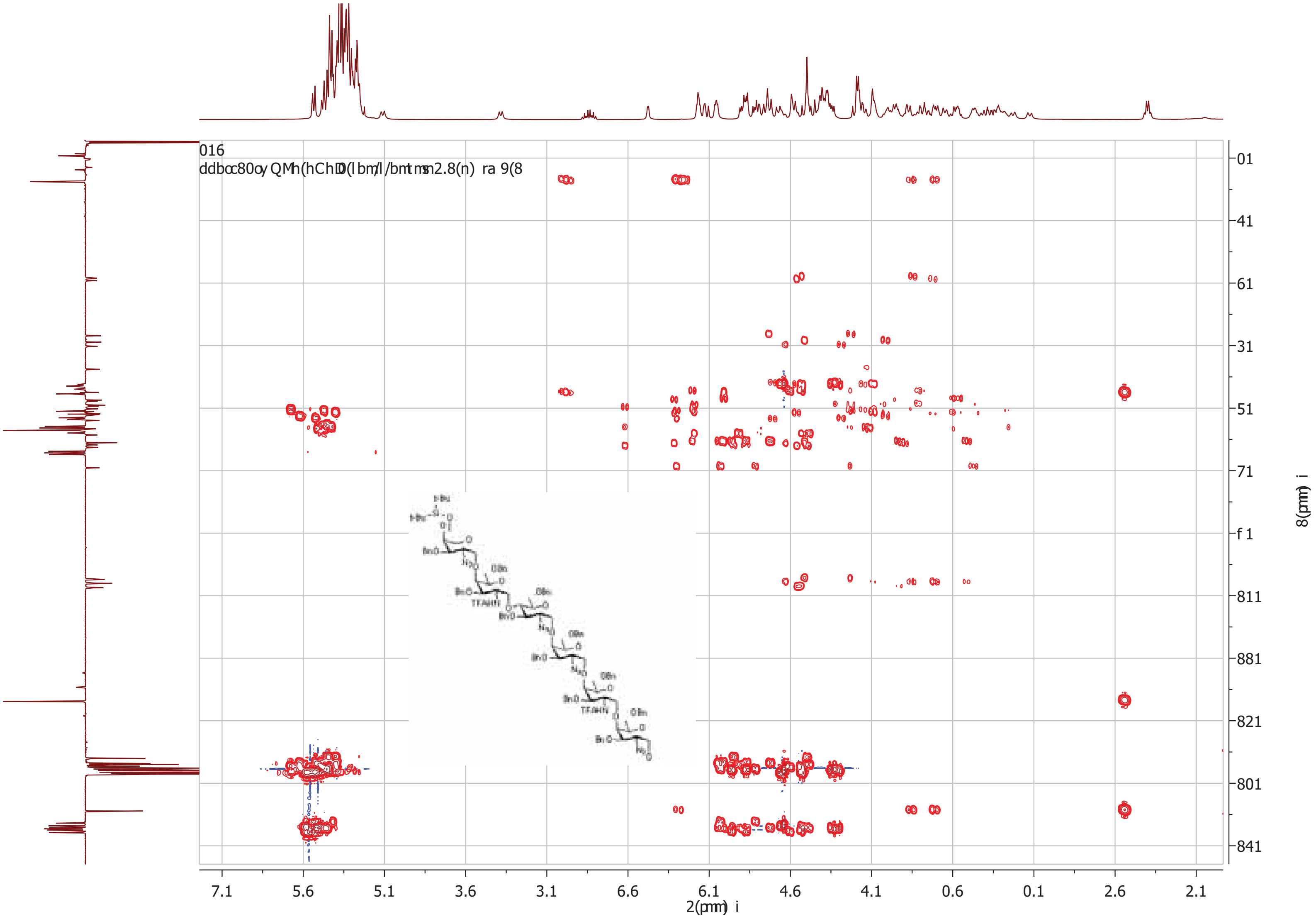

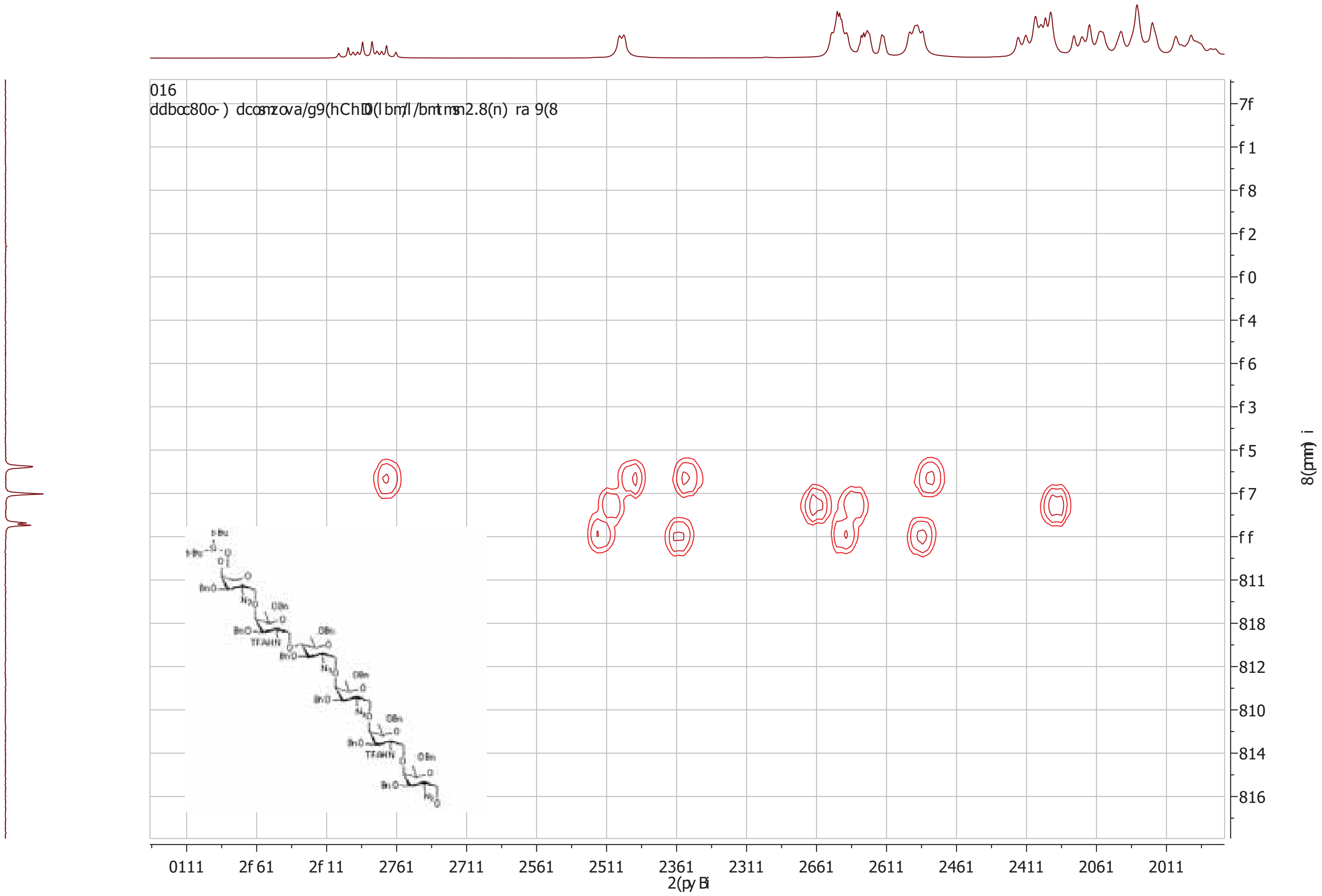
016
ddboc80o-) dcosmzova/g9(hChD(lbm/l/bmtms2.8(n) ra 9(8
t-Bu
BnO
OBn
TFAHN
2(pyBi
8(pm) i

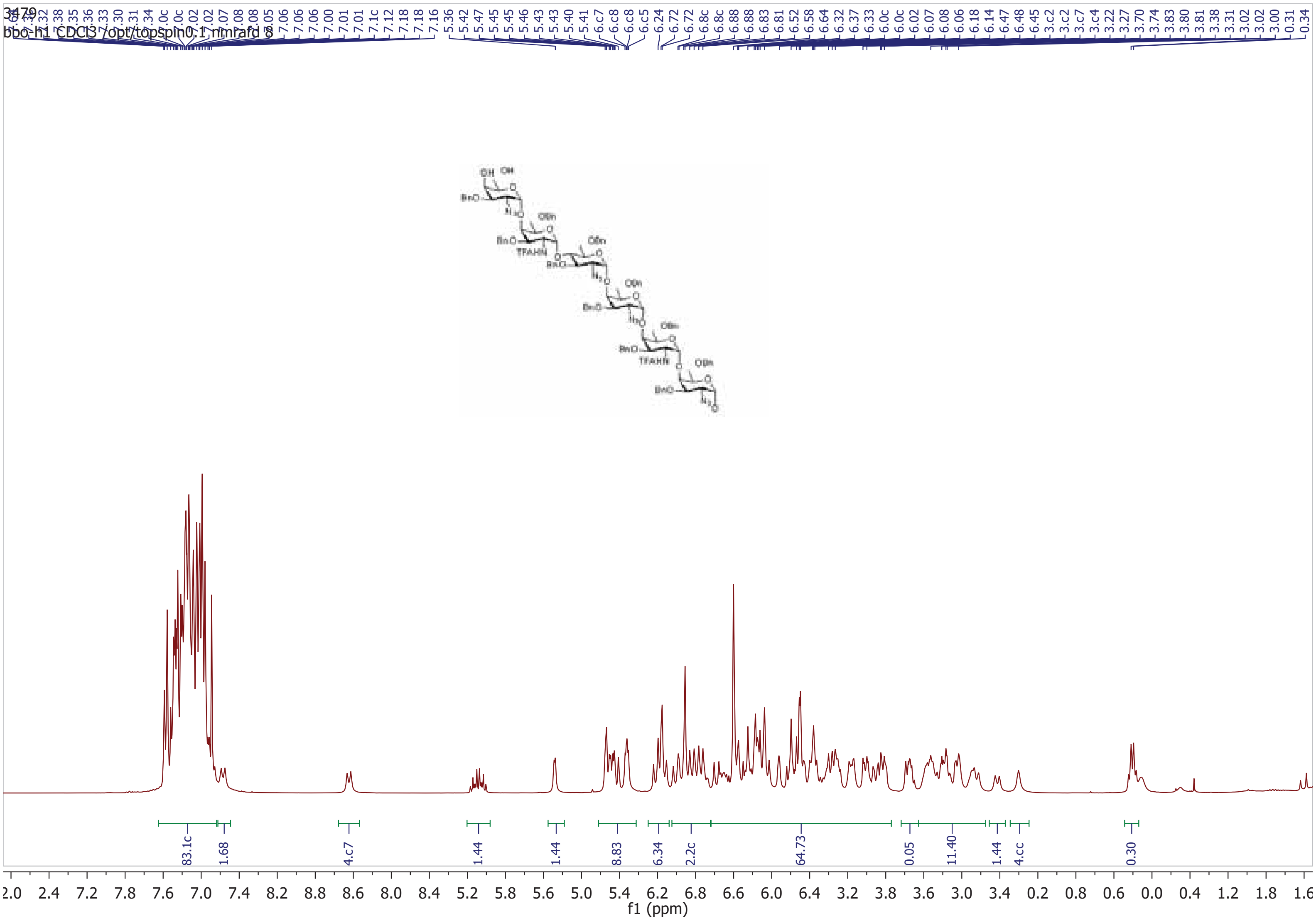

f1 (ppm)

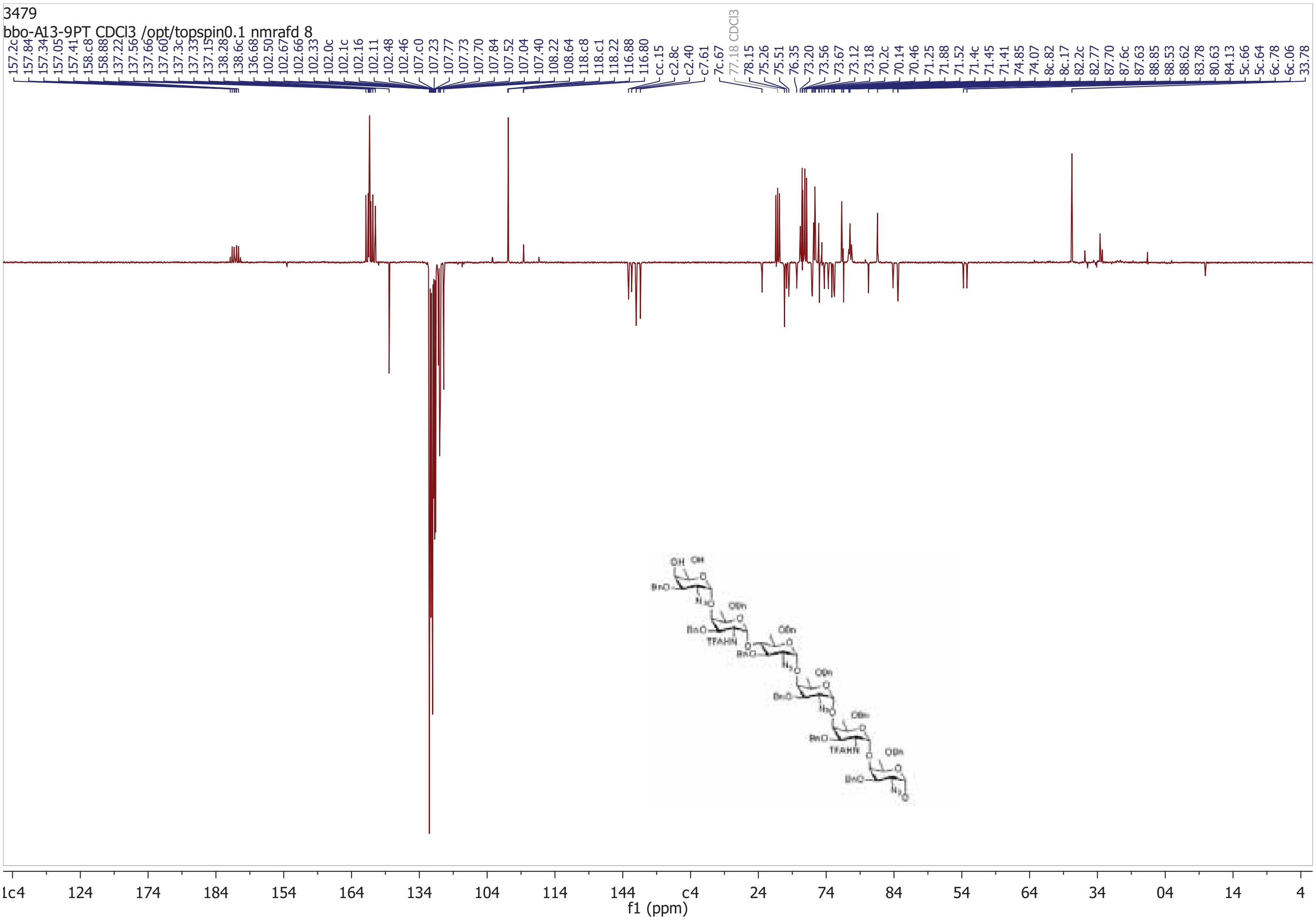

3479
bbo-A13-9PT CDCl3 /opt/topspin0.1 nmrafd 8
77.18 CDCl3
f1 (ppm)

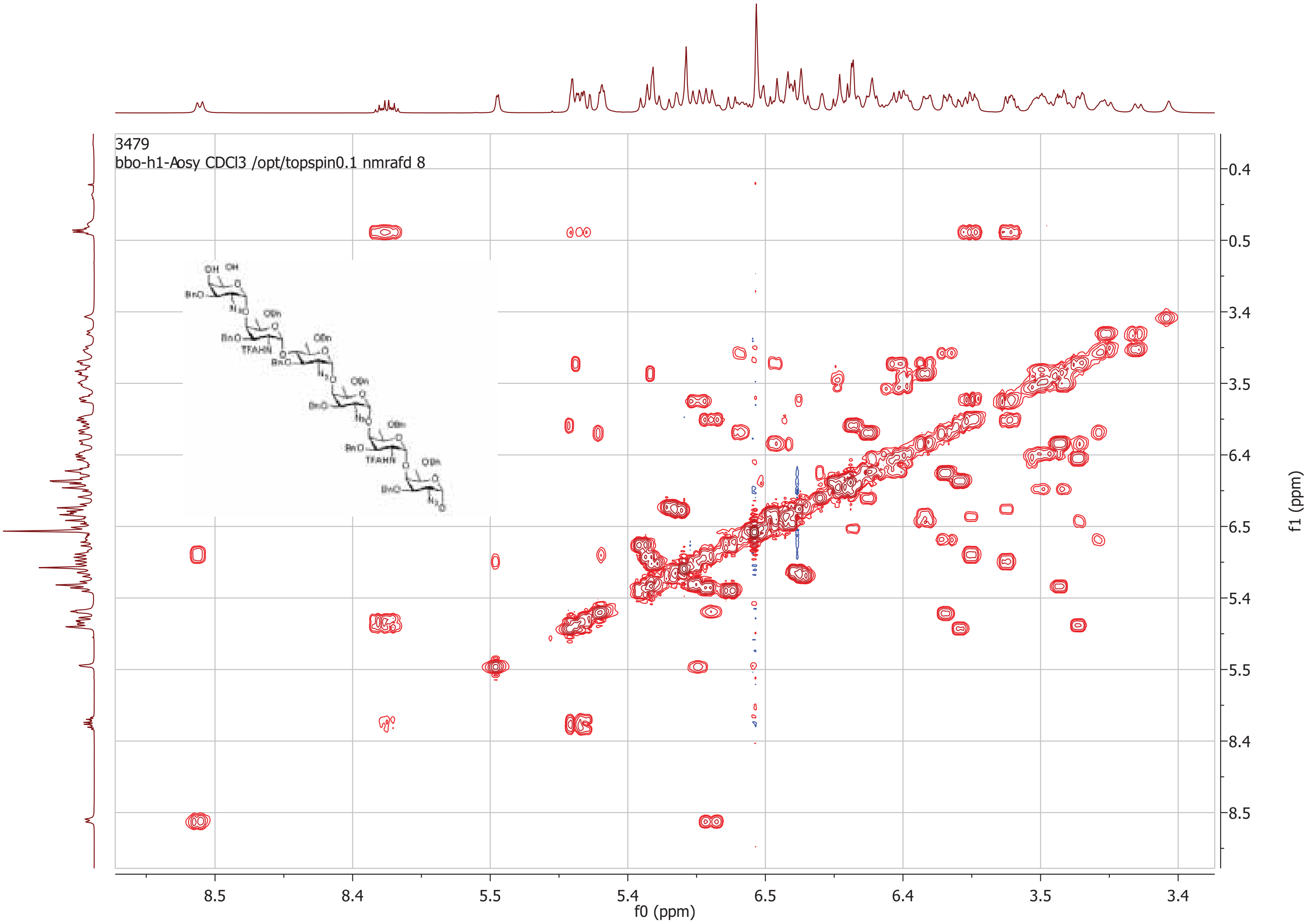
3479
bbo-h1-Aosy CDCl3 /opt/topspin0.1 nmrafd 8
f0 (ppm)
f1 (ppm)

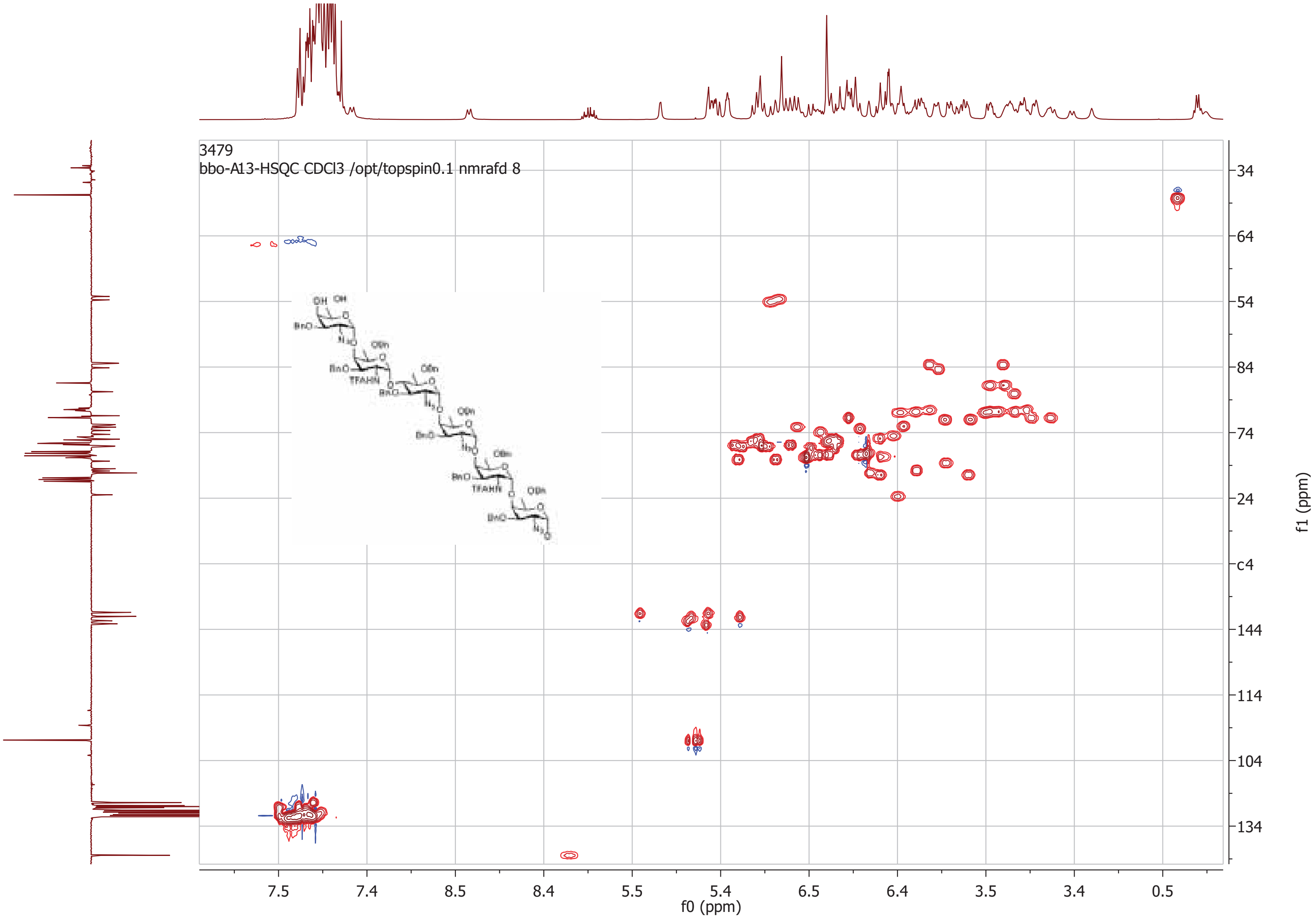
3479
bbo-A13-HSQC CDCl3 /opt/topspin0.1 nmrafd 8
OH
OH
BnO
N3
OBn
BnO
TFAHN
OBn
BnO
OBn
BnO
OBn
BnO
TFAHN
OBn
BnO
34
64
54
84
74
24
c4
144
114
104
134
f1 (ppm)
7.5
7.4
8.5
8.4
5.5
5.4
6.5
6.4
3.5
3.4
0.5
f0 (ppm)

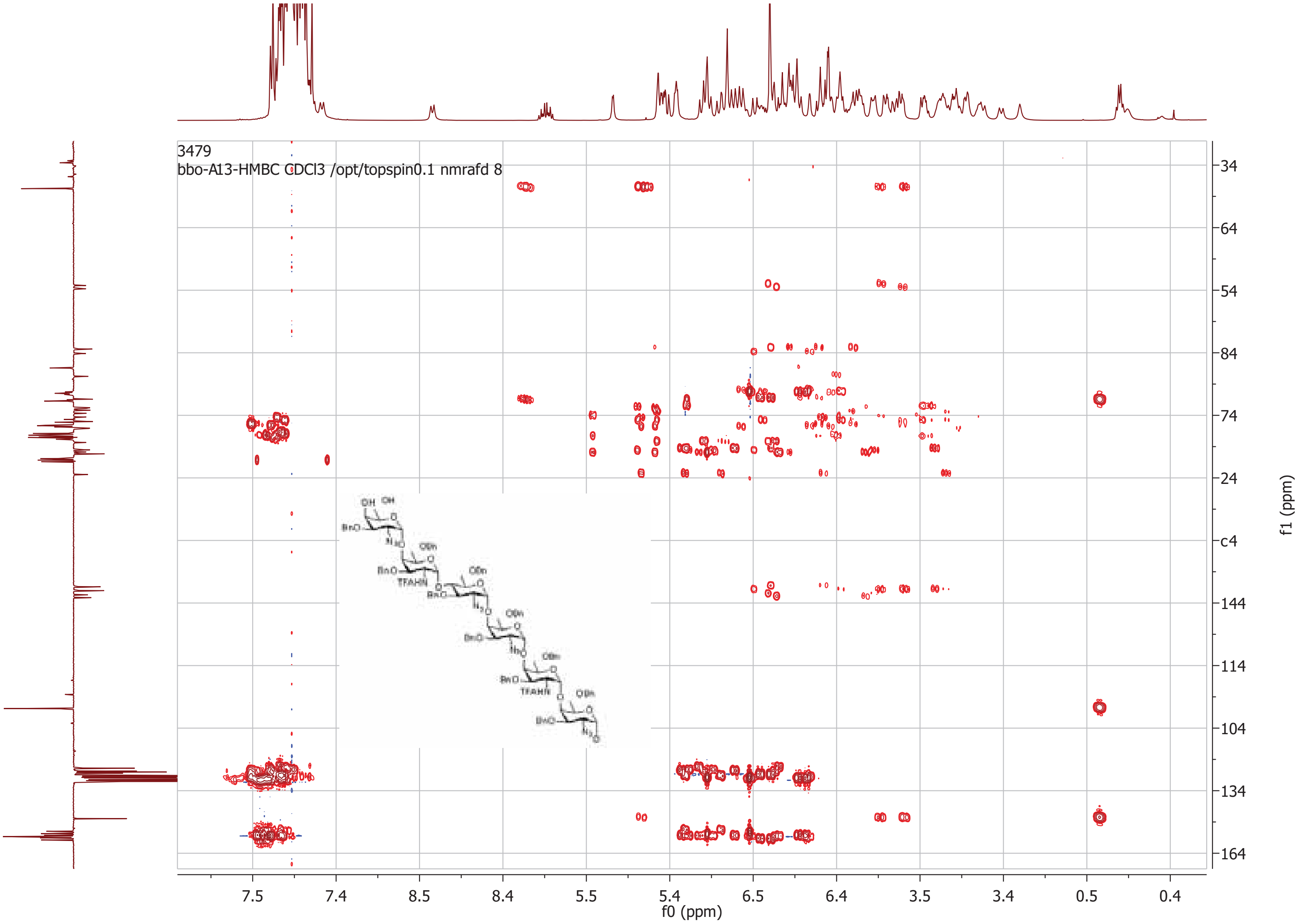
3479
bbo-A13-HMBC CDCl3 /opt/topspin0.1 nmrafd 8
f0 (ppm)
f1 (ppm)

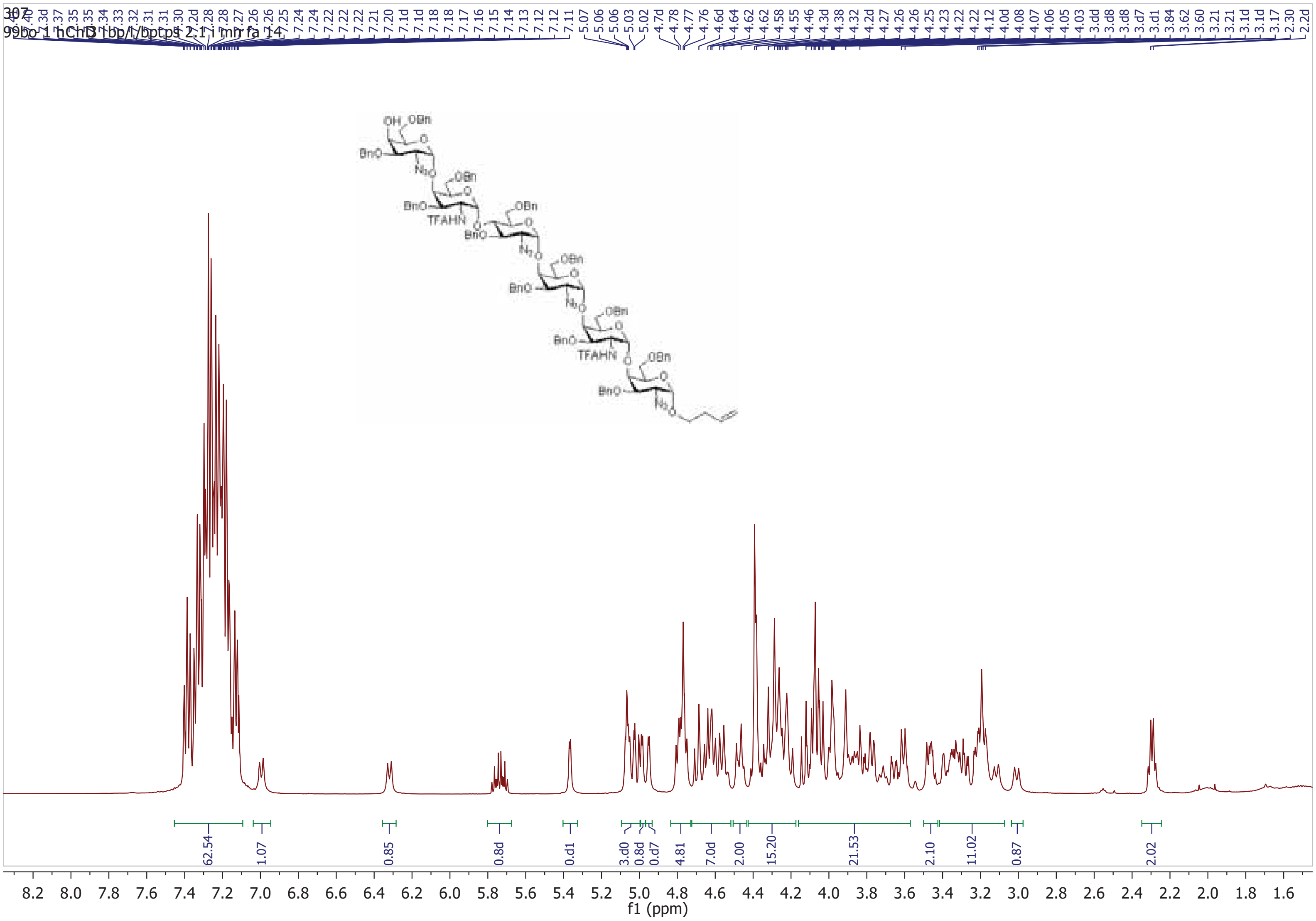
307
OH
OBn
BnO
TFAHN
62.54
1.07
0.85
4.81
2.00
15.20
21.53
2.10
11.02
0.87
2.02
f1 (ppm)

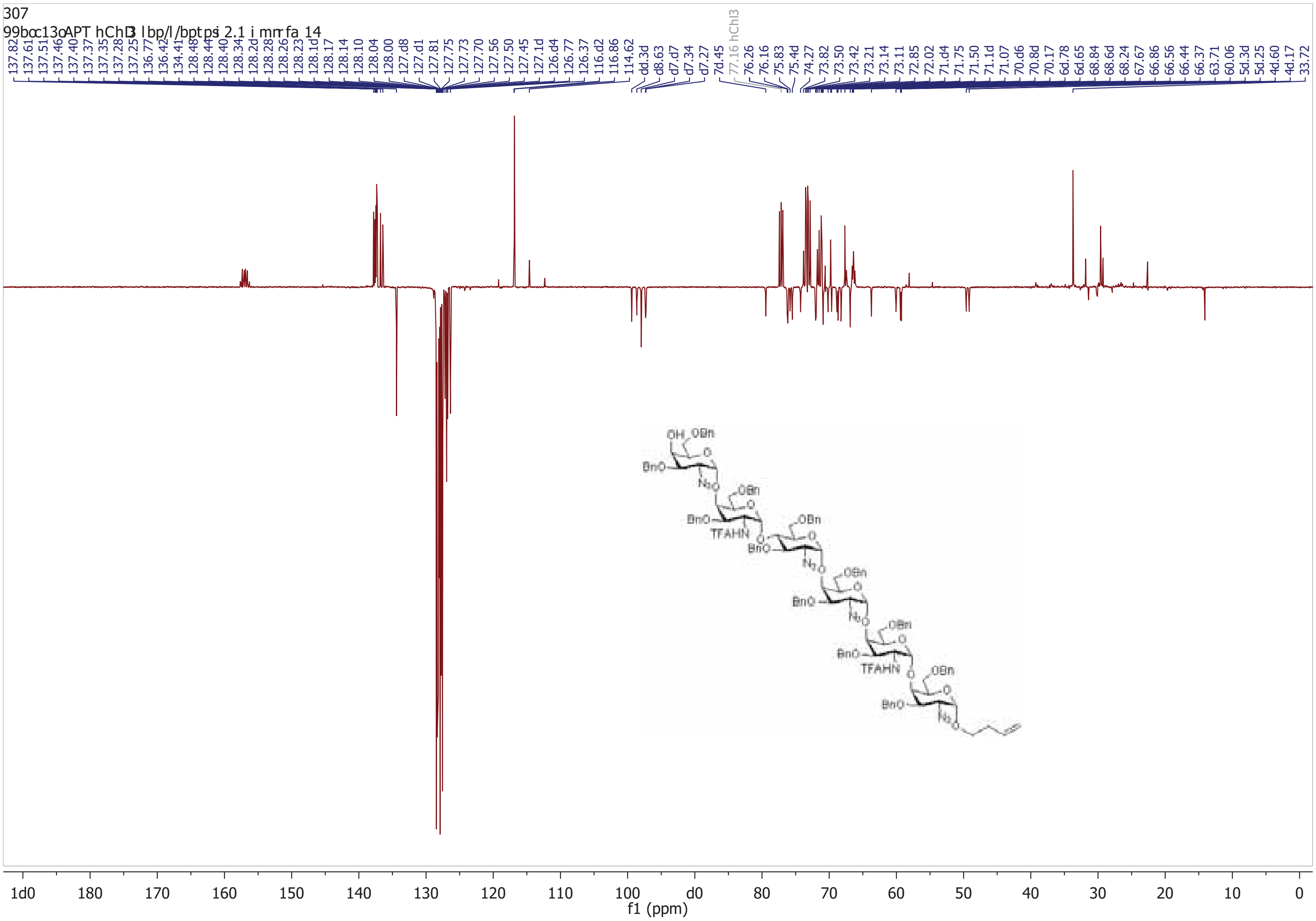

307
99bcc13oAPT hChl3 lbp/l/bptpsi 2.1 i mrrfa 14
137.82
137.61
137.51
137.46
137.40
137.37
137.35
137.28
137.25
136.77
136.42
134.41
128.48
128.44
128.40
128.34
128.2d
128.28
128.26
128.23
128.1d
128.17
128.14
128.10
128.04
128.00
127.d8
127.d1
127.81
127.75
127.73
127.70
127.56
127.50
127.45
127.1d
126.d4
126.77
126.37
116.d2
116.86
114.62
dd.3d
d8.63
d7.d7
d7.34
d7.27
7d.45
77.16 hChl3
76.26
76.16
75.83
75.4d
74.27
73.82
73.50
73.42
73.21
73.14
73.11
72.85
72.02
71.d4
71.75
71.50
71.1d
71.07
70.d6
70.8d
70.17
6d.78
6d.65
68.84
68.6d
68.24
67.67
66.86
66.56
66.44
66.37
63.71
60.06
5d.3d
5d.25
4d.60
4d.17
33.72
OH
OBn
BnO
N3
TFAHN
1d0
180
170
160
150
140
130
120
110
100
d0
80
70
60
50
40
30
20
10
0
f1 (ppm)

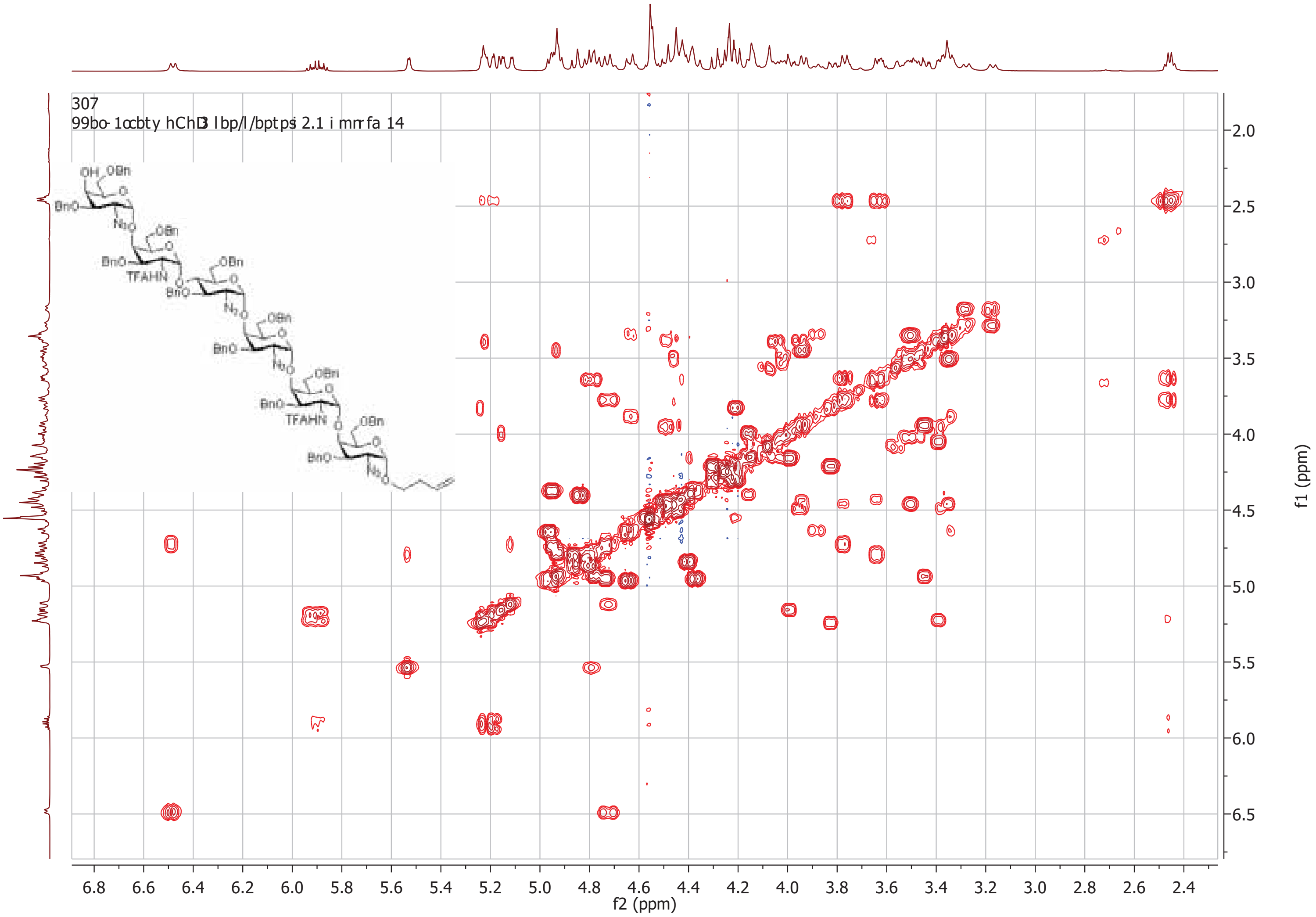
307
99bo-1ccbty hChl3 lbp/l/bptps 2.1 i mrrfa 14
OH
OBn
BnO
N3
TFAHN
f2 (ppm)
f1 (ppm)

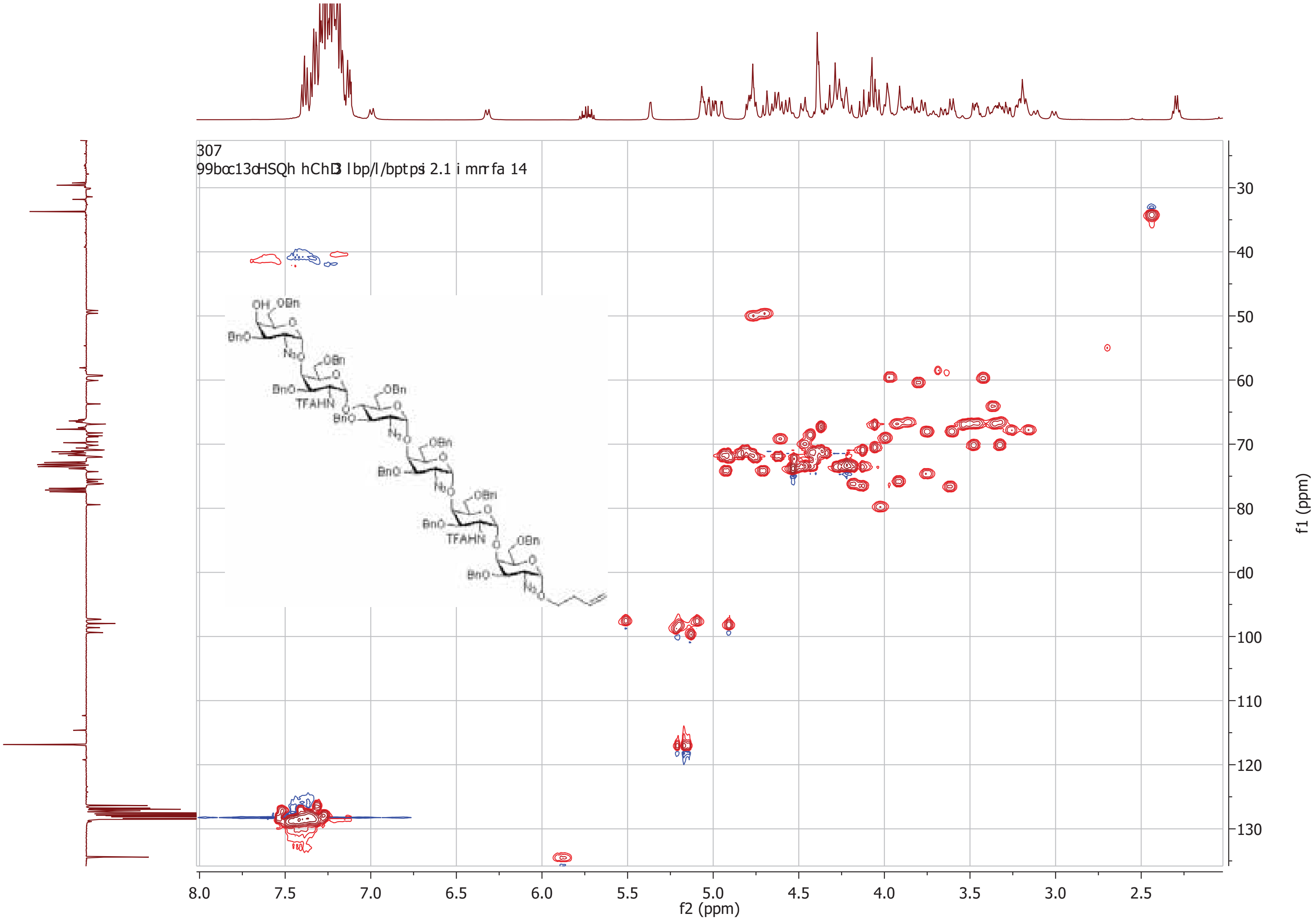

307
f2 (ppm)
f1 (ppm)
8.0
7.5
7.0
6.5
6.0
5.5
5.0
4.5
4.0
3.5
3.0
2.5
30
40
50
60
70
80
d0
100
110
120
130
OH
OBn
BnO
N3
TFAHN

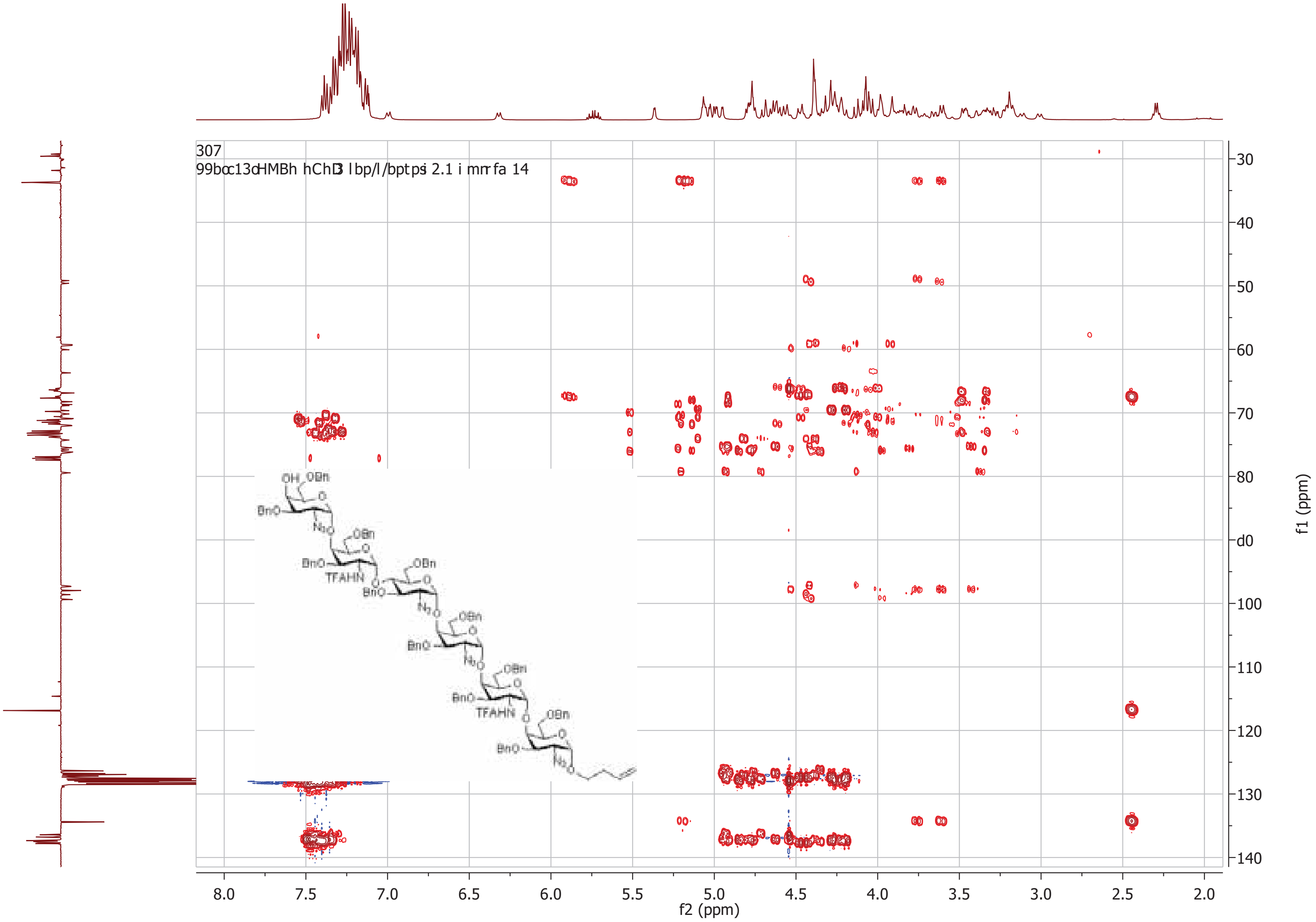
307
f2 (ppm)
f1 (ppm)
OH
OBn
BnO
TFAHN

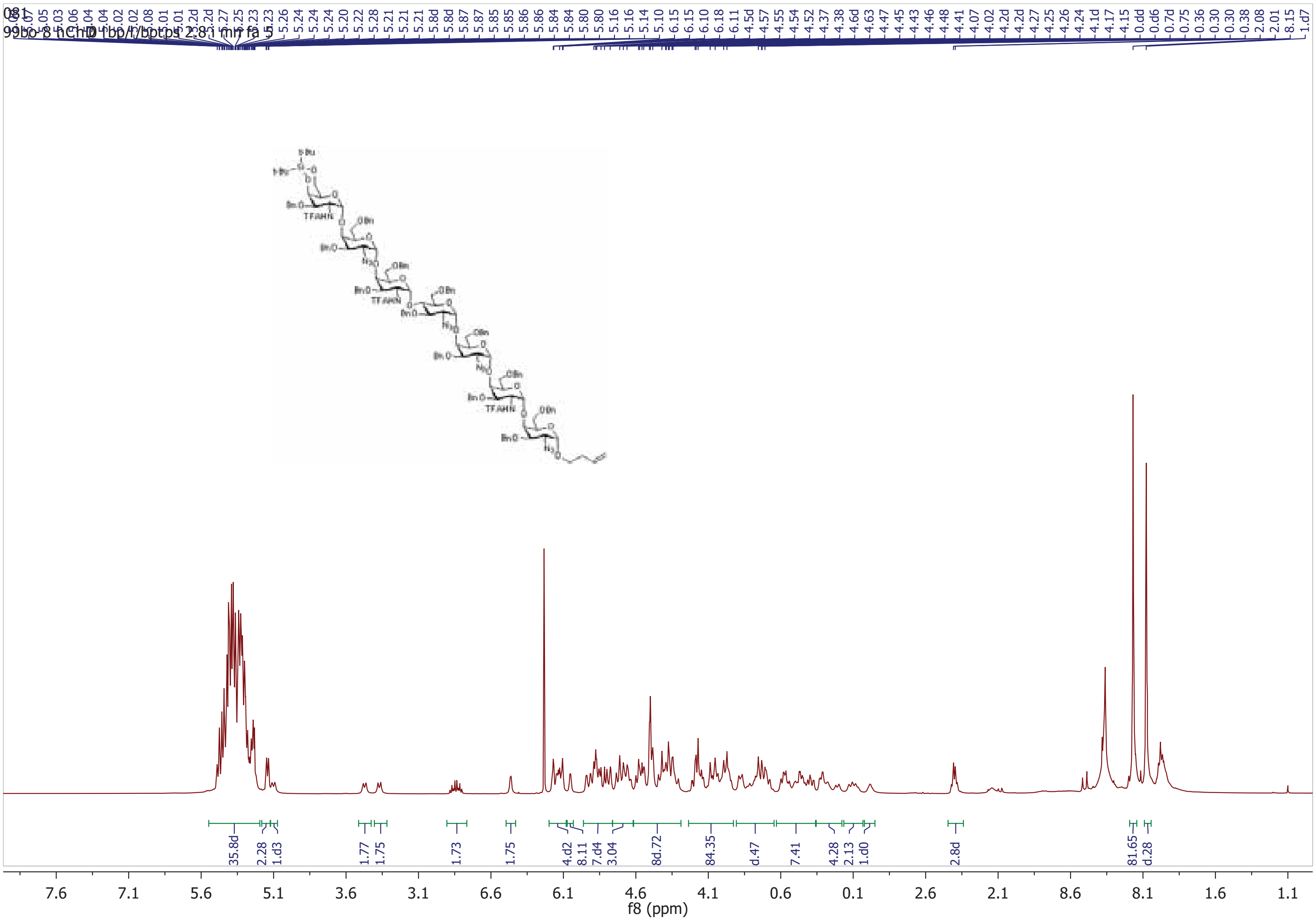
f8 (ppm)

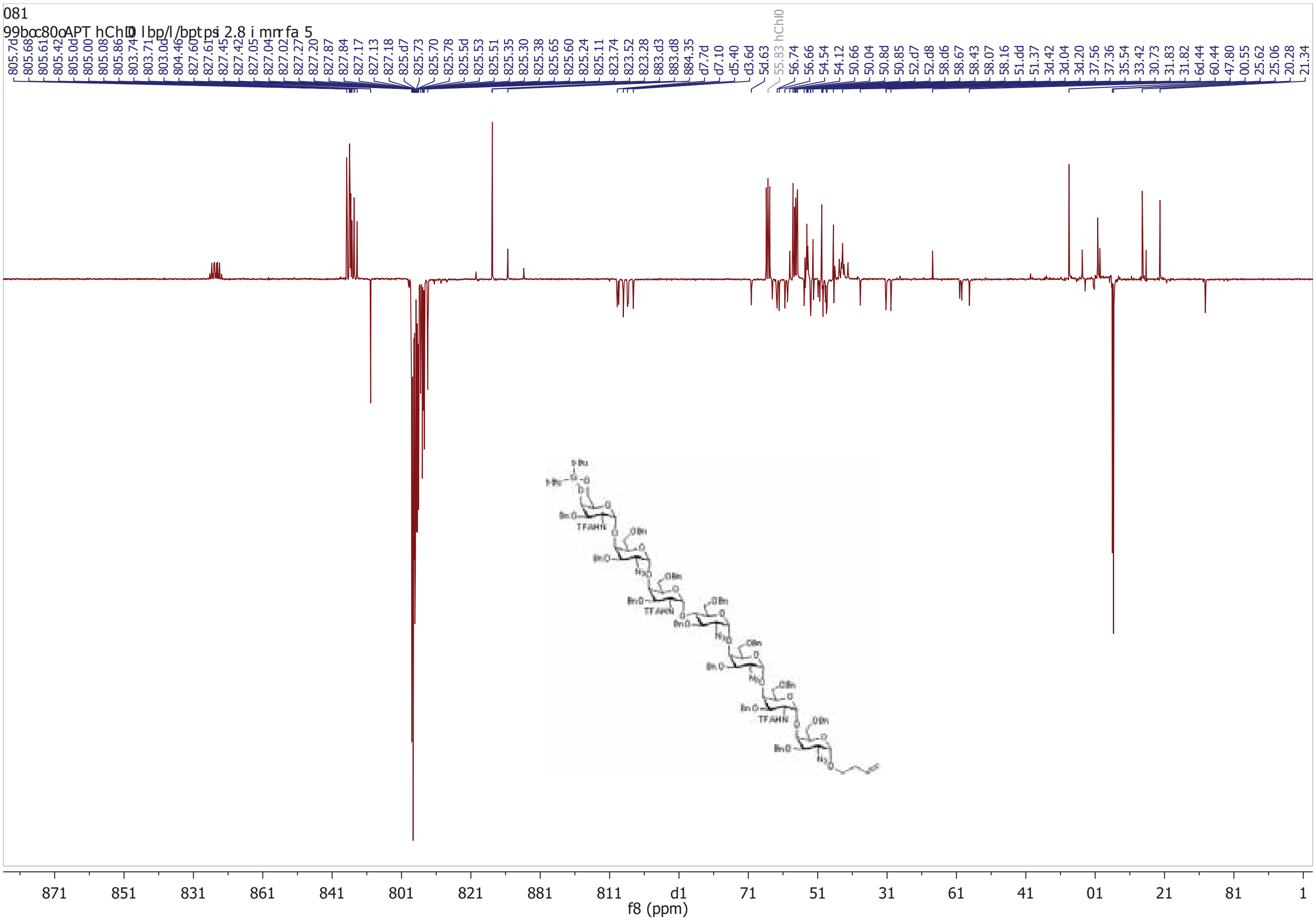
081
99bc c80o APT hChl0 lbp/l/bptps 2.8 i mrr fa 5
f8 (ppm)

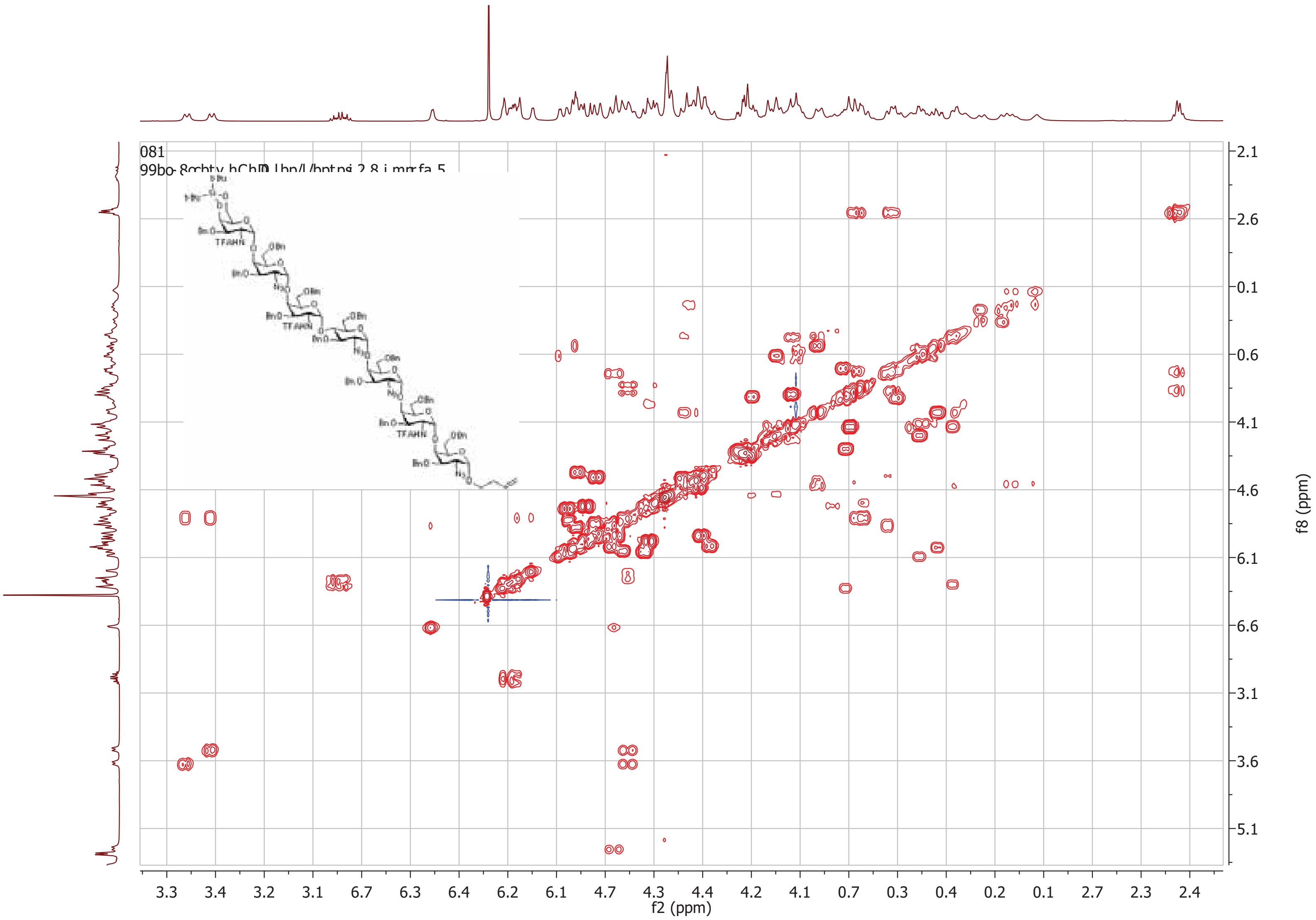
081
f2 (ppm)
f8 (ppm)

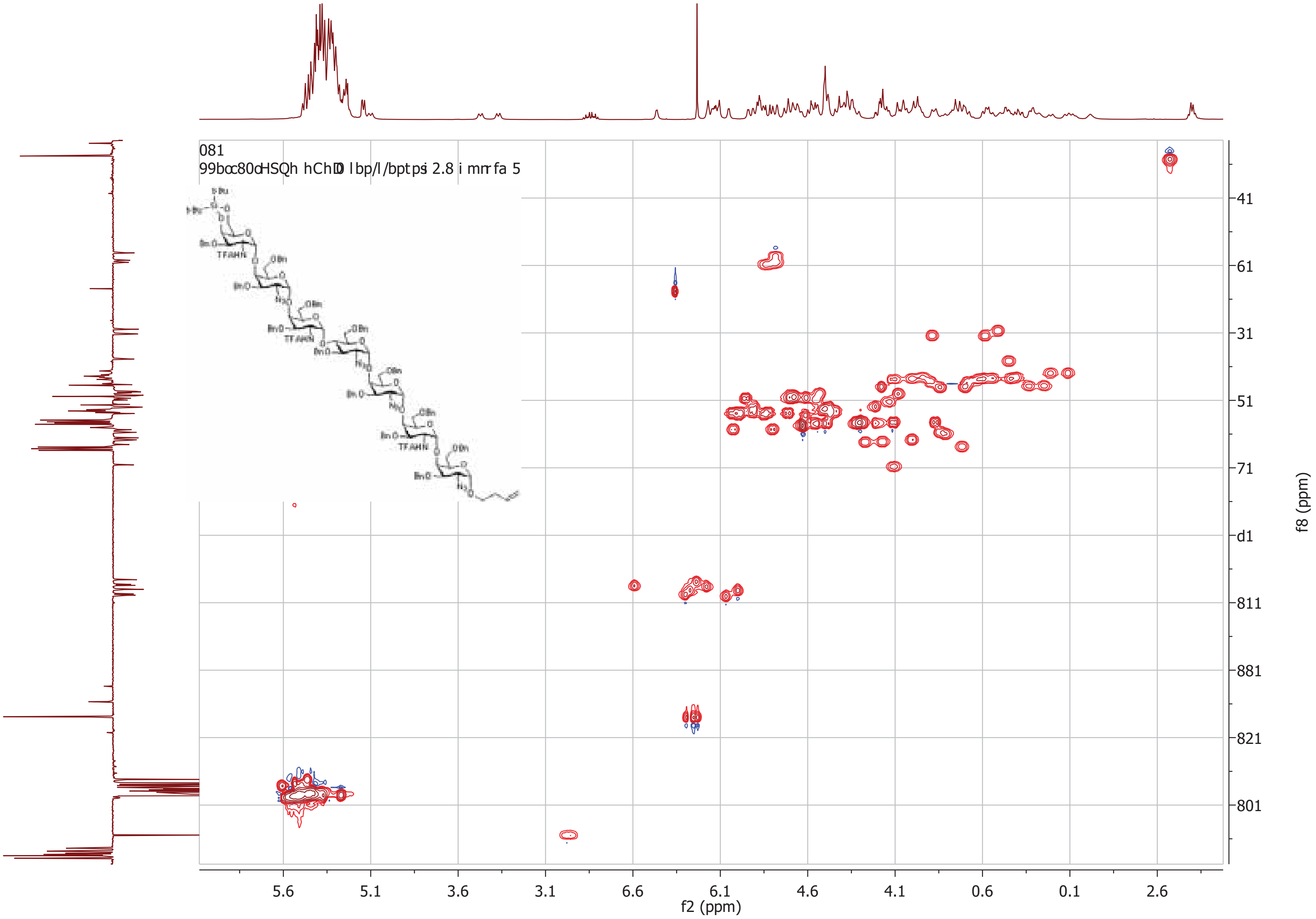
081
TFAHN
BnO
OBn
f2 (ppm)
f8 (ppm)

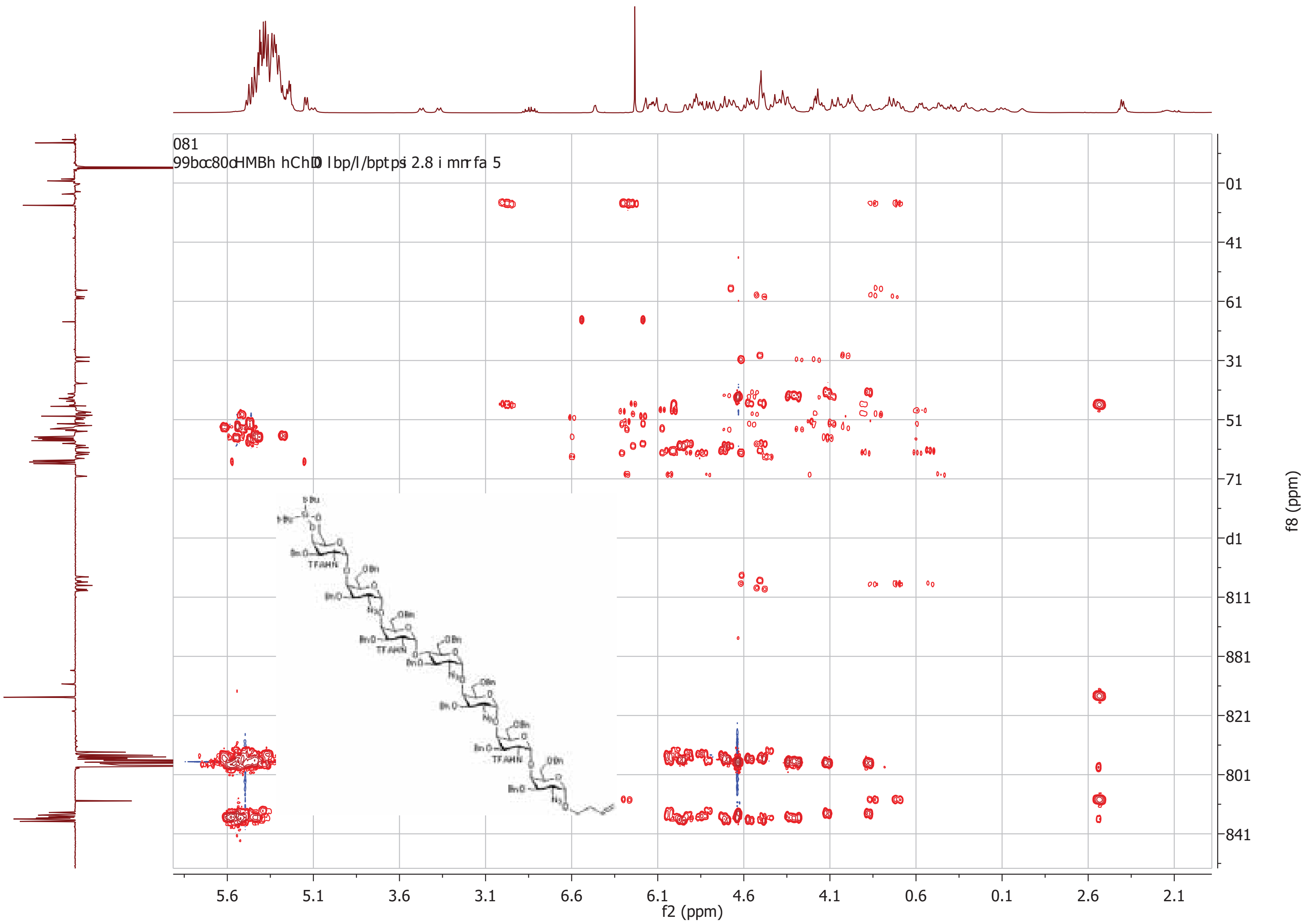

f2 (ppm)
f8 (ppm)
TFAHN

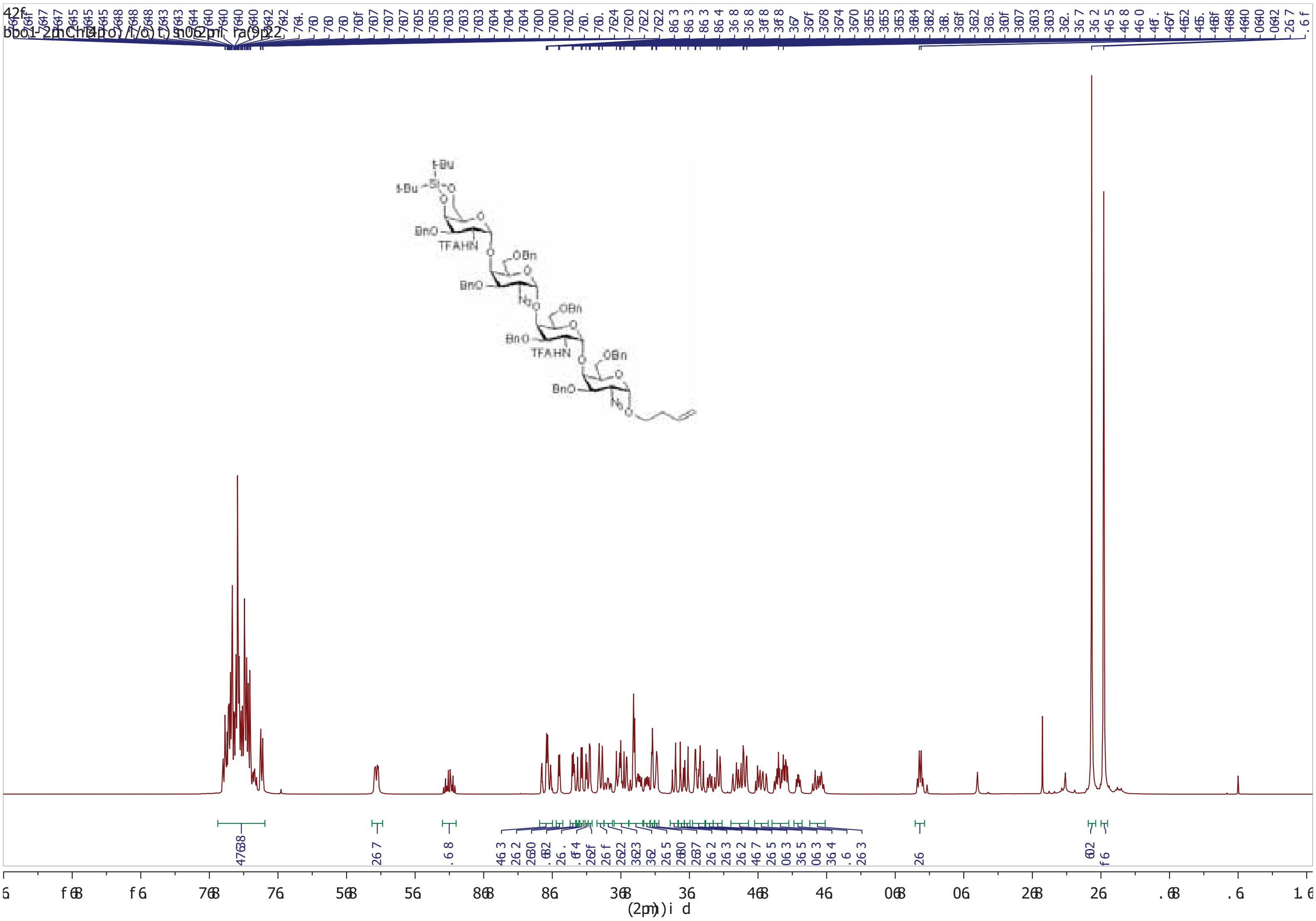
t-Bu
t-Bu
Si
BnO
TFAHN
OBn
BnO
OBn
BnO
TFAHN
OBn
BnO

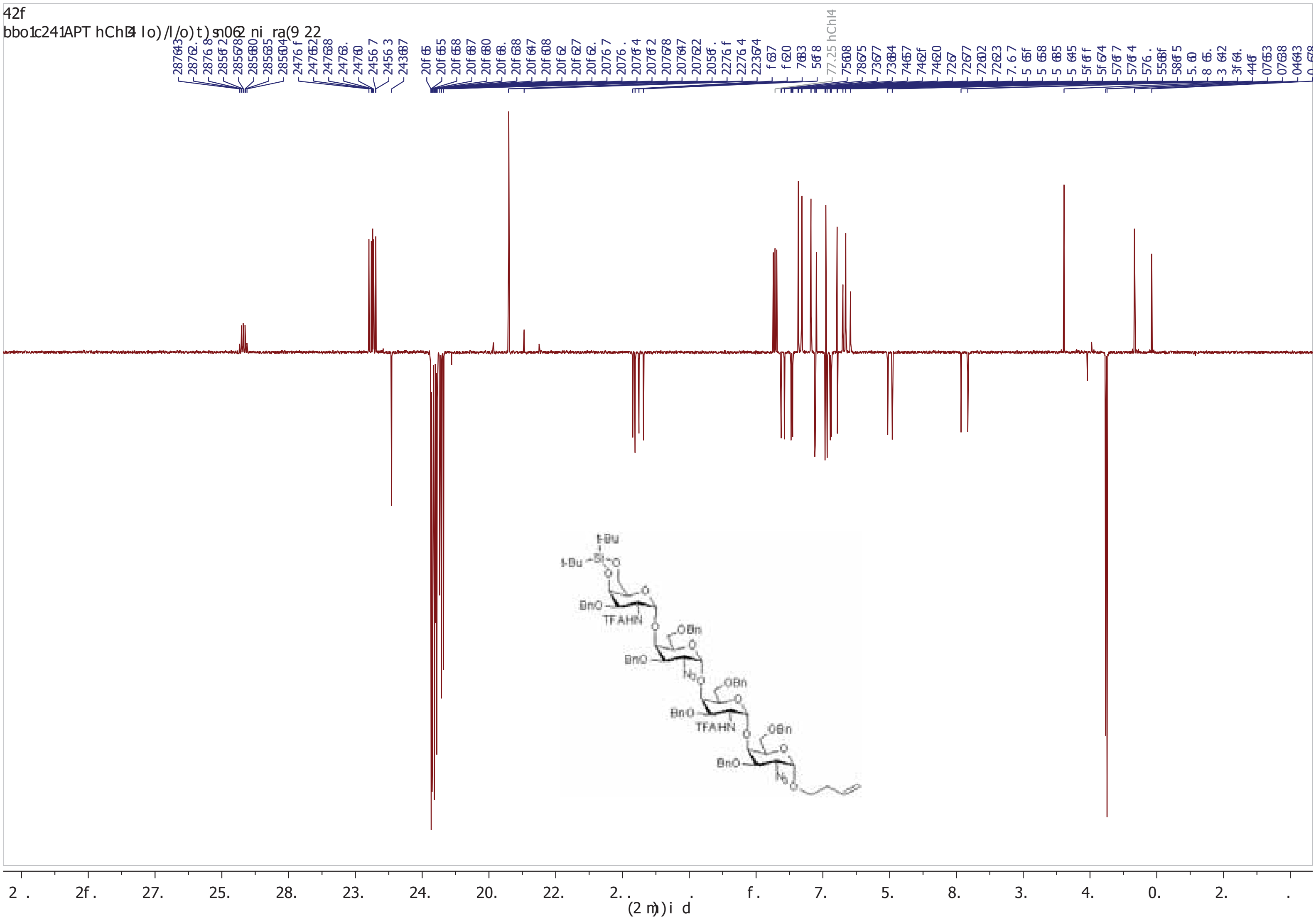
42f
77.25 hChl4
t-Bu
t-Bu
Si
BnO
TFAHN
OBn
BnO
OBn
BnO
TFAHN
OBn
BnO

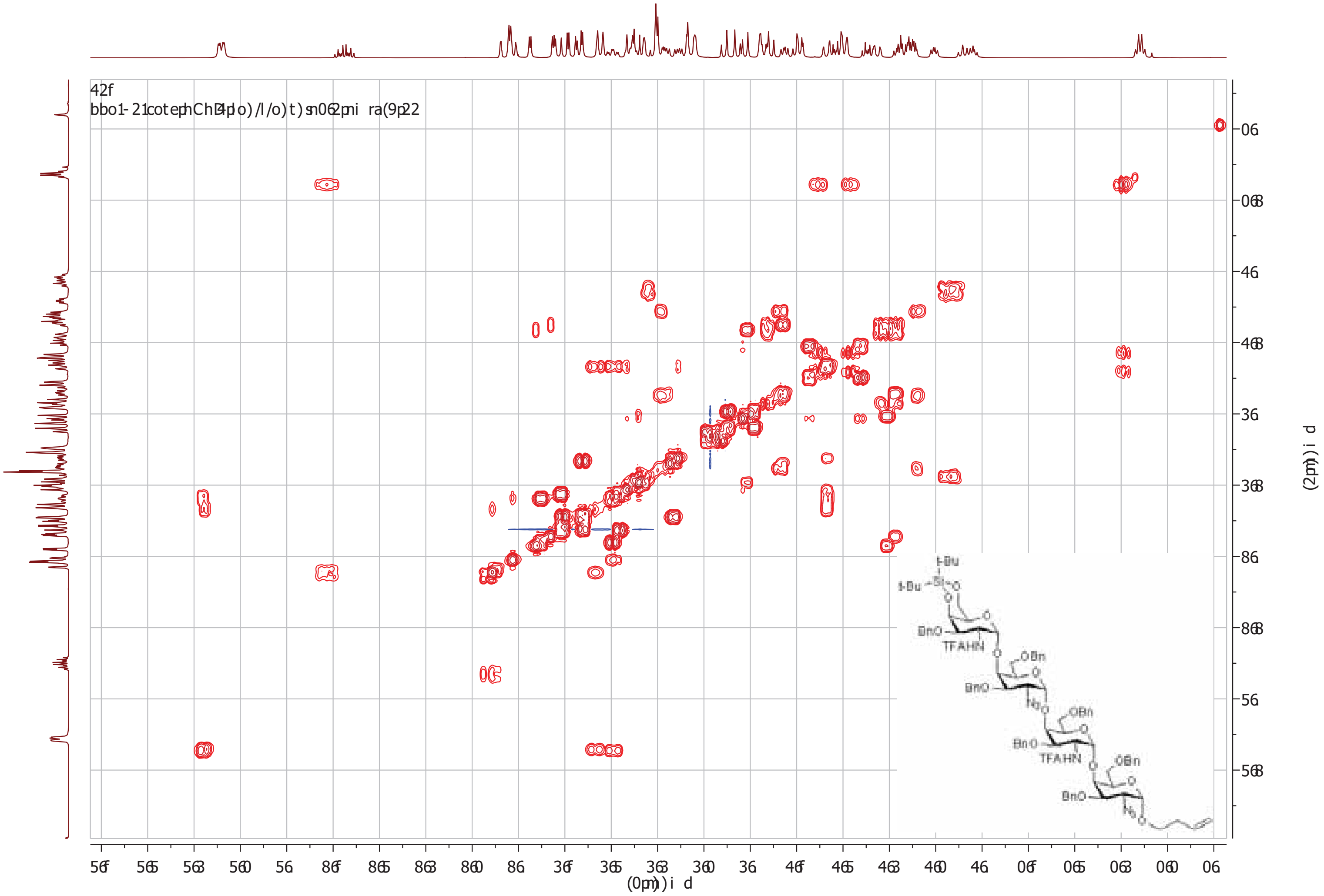
t-Bu
t-Bu
Si
BnO
TFAHN
OBn
BnO
BnO
TFAHN
OBn
BnO

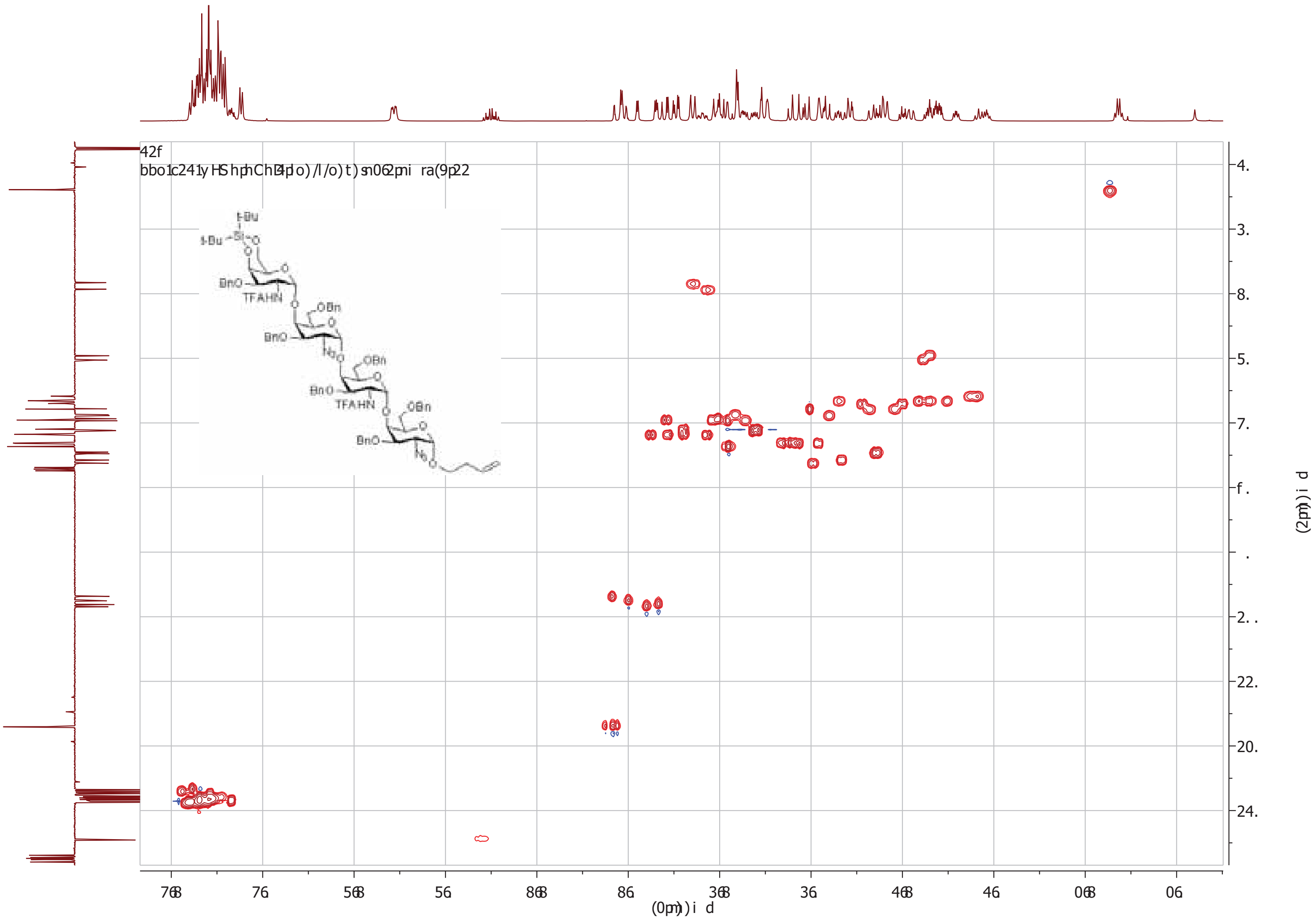
t-Bu
t-Bu
Si
BnO
TFAHN
OBn
TFAHN

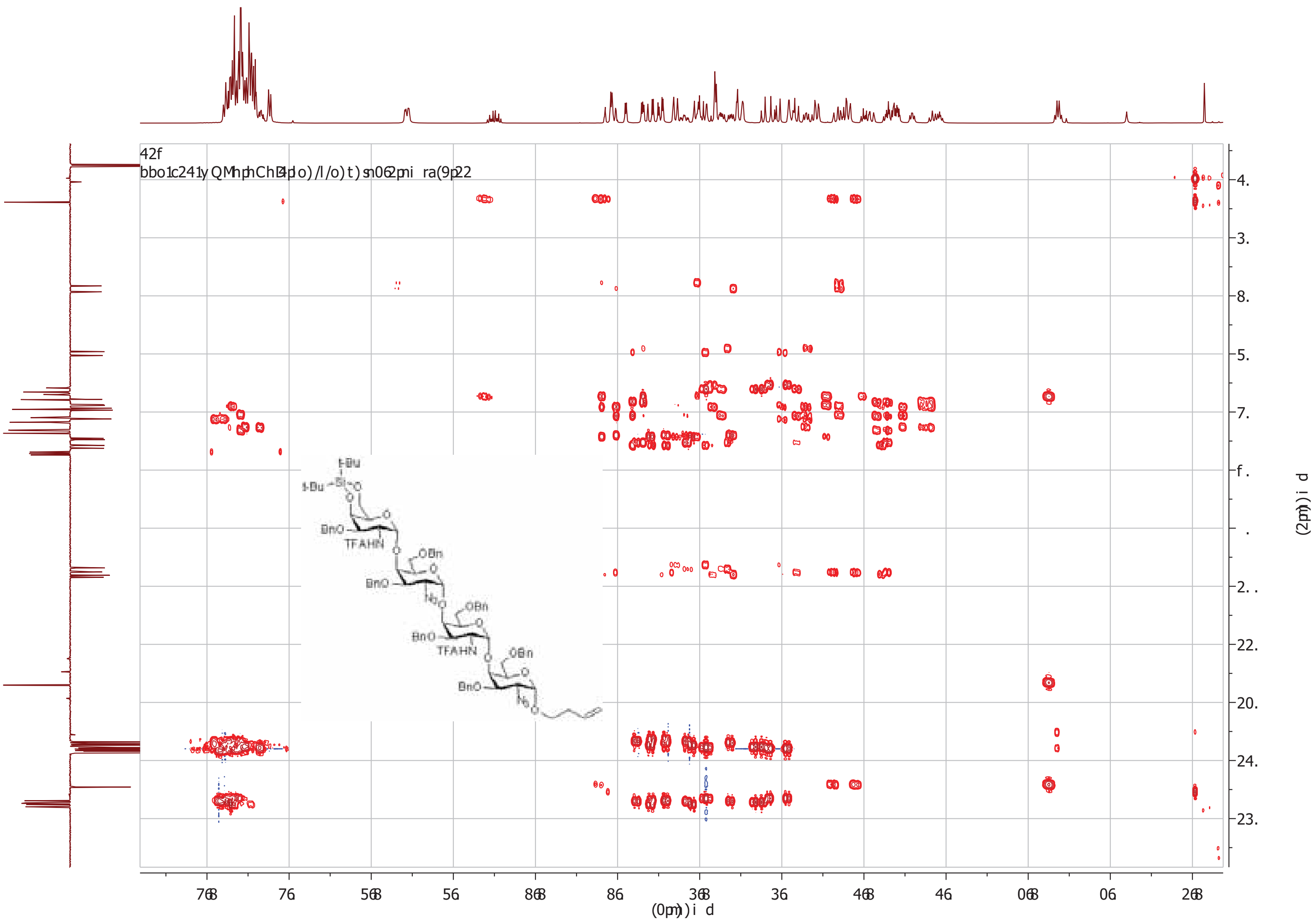

t-Bu
t-Bu
Si
BnO
TFAHN
OBn
BnO
BnO
TFAHN
OBn
BnO

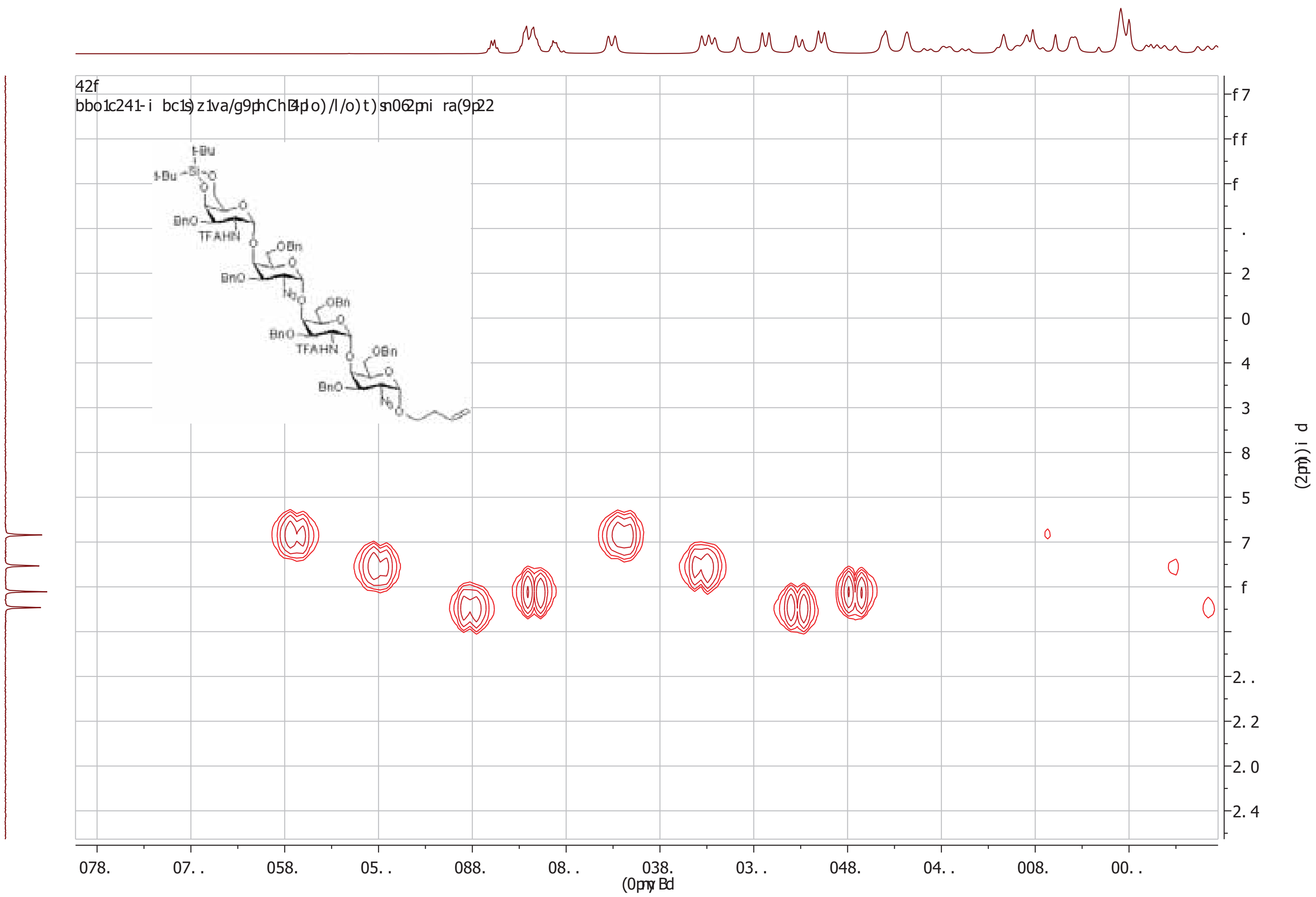
42f
t-Bu
t-Bu
Si
BnO
TFAHN
OBn
BnO
OBn
BnO
TFAHN
OBn
BnO

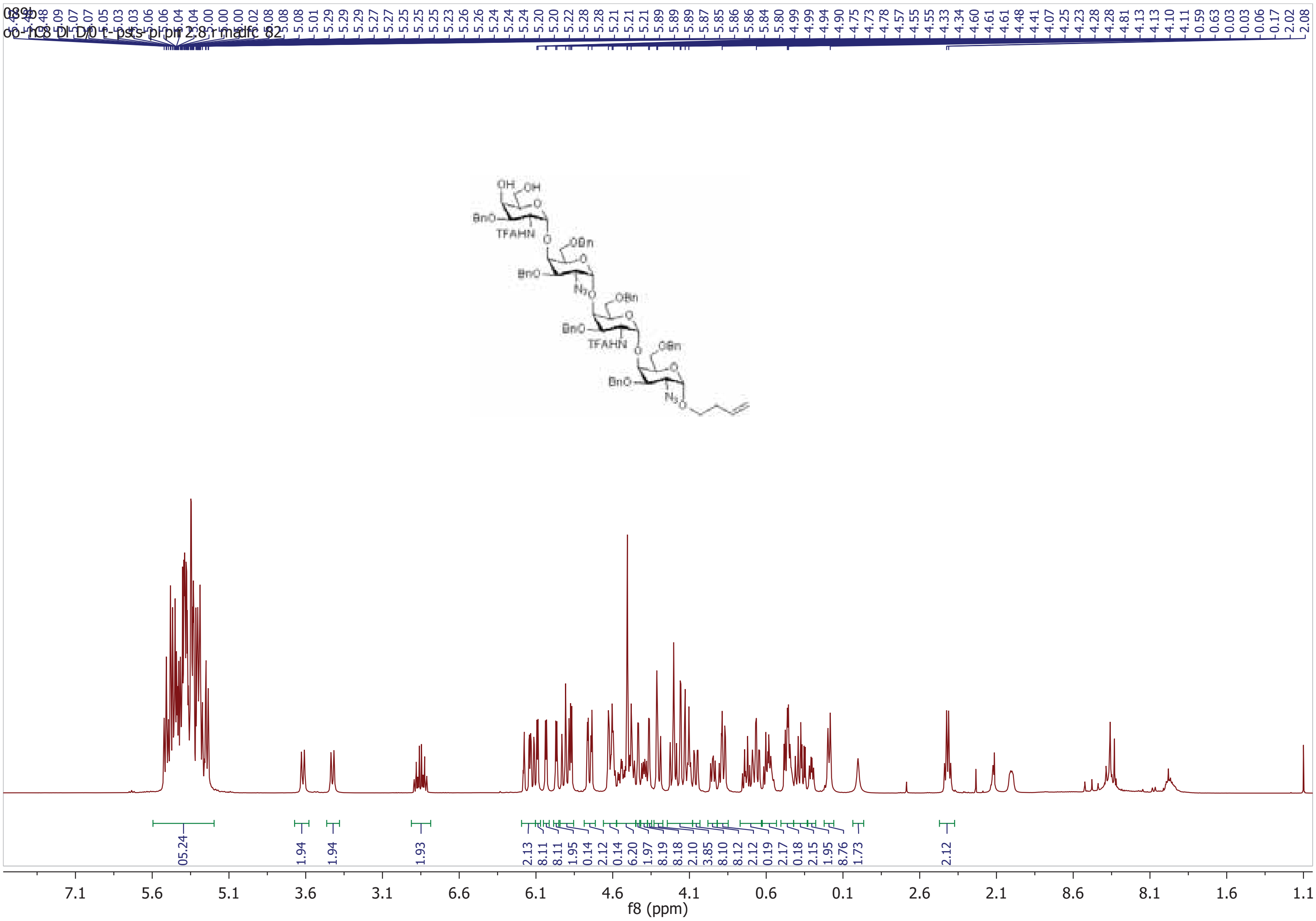
f8 (ppm)

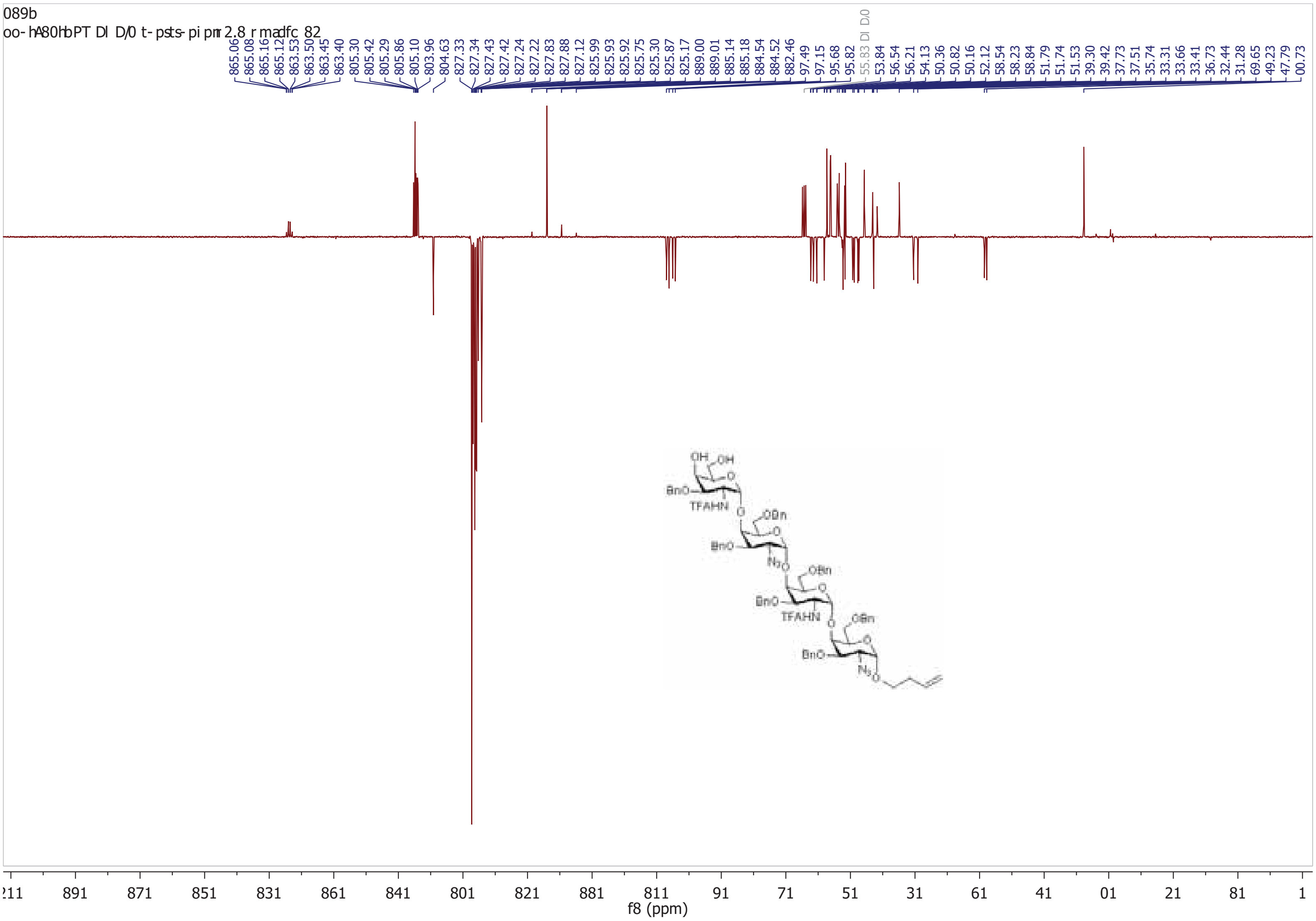
089b
oo-hA80hbPT DI D/0 t-psts-pi pm 2.8 r madfc 82
865.06
865.08
865.16
865.12
863.53
863.50
863.45
863.40
805.30
805.42
805.29
805.86
805.10
803.96
804.63
827.33
827.34
827.43
827.42
827.24
827.22
827.83
827.88
827.12
825.99
825.93
825.92
825.75
825.30
825.87
825.17
889.00
889.01
885.14
885.18
884.54
884.52
882.46
97.49
97.15
95.68
95.82
55.83 DI D0
53.84
56.54
56.21
54.13
50.36
50.82
50.16
52.12
58.54
58.23
58.84
51.79
51.74
51.53
39.30
39.42
37.73
37.51
35.74
33.31
33.66
33.41
36.73
32.44
31.28
69.65
49.23
47.79
00.73
OH
OH
BnO
TFAHN
OBn
BnO
N3
OBn
BnO
TFAHN
OBn
BnO
N3
211 891 871 851 831 861 841 801 821 881 811 91 71 51 31 61 41 01 21 81 1
f8 (ppm)

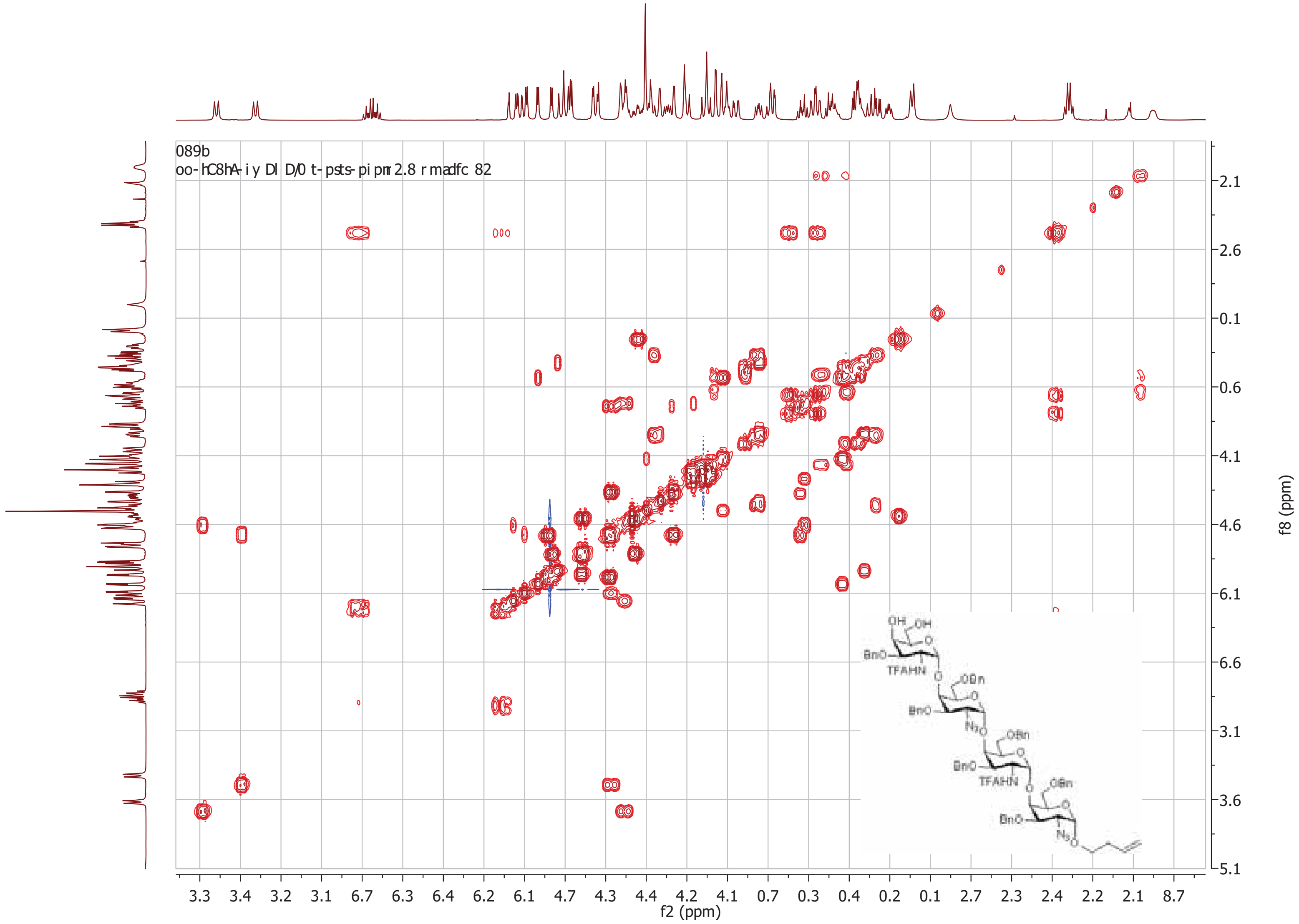
089b
oo- hC8hA- i y DI D/0 t- psts- pi pmr 2.8 r madfc 82
f2 (ppm)
f8 (ppm)
OH
OH
BnO
TFAHN
OBn
N3

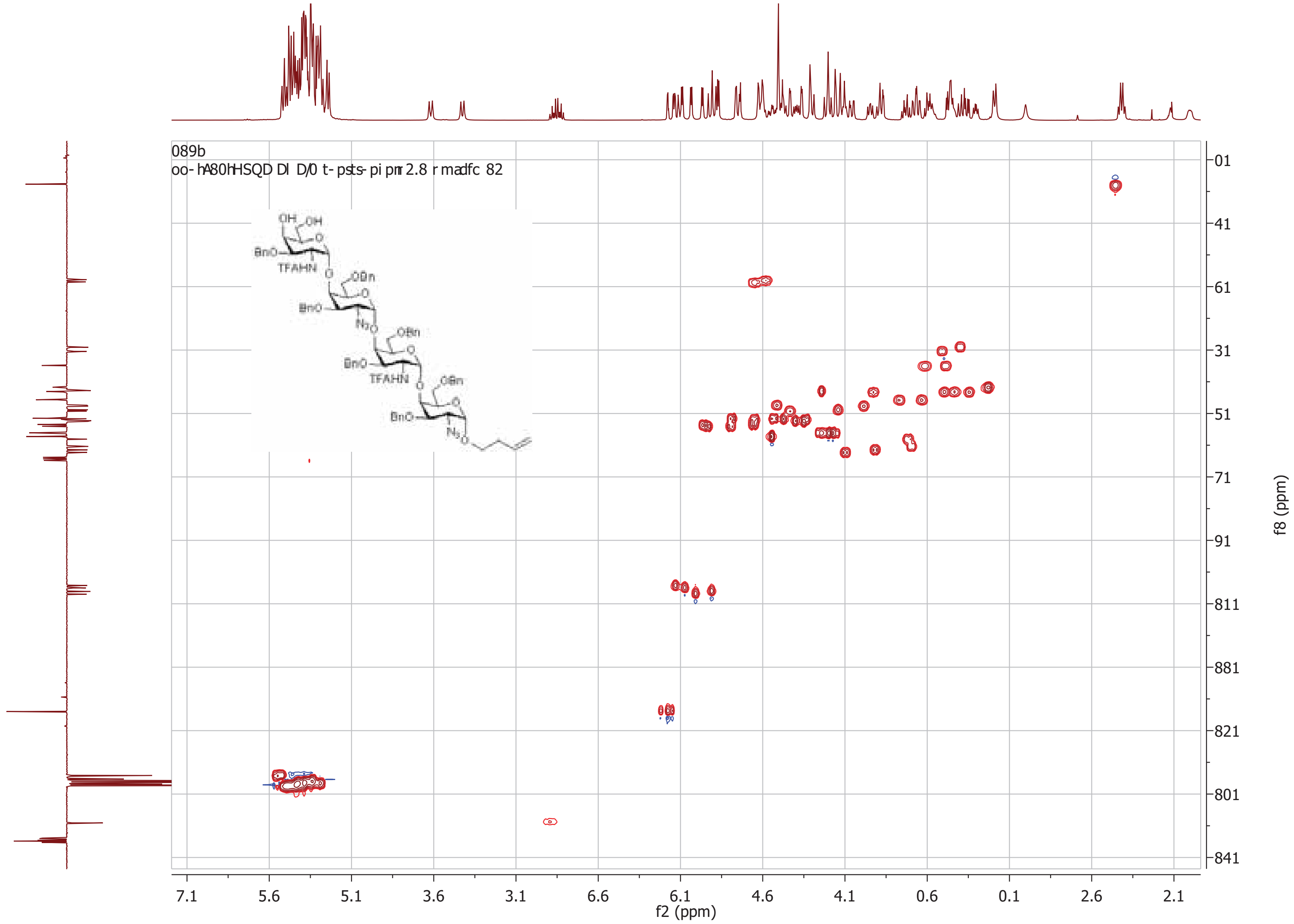
089b
BnO
TFAHN
OBn
N3
f2 (ppm)
f8 (ppm)

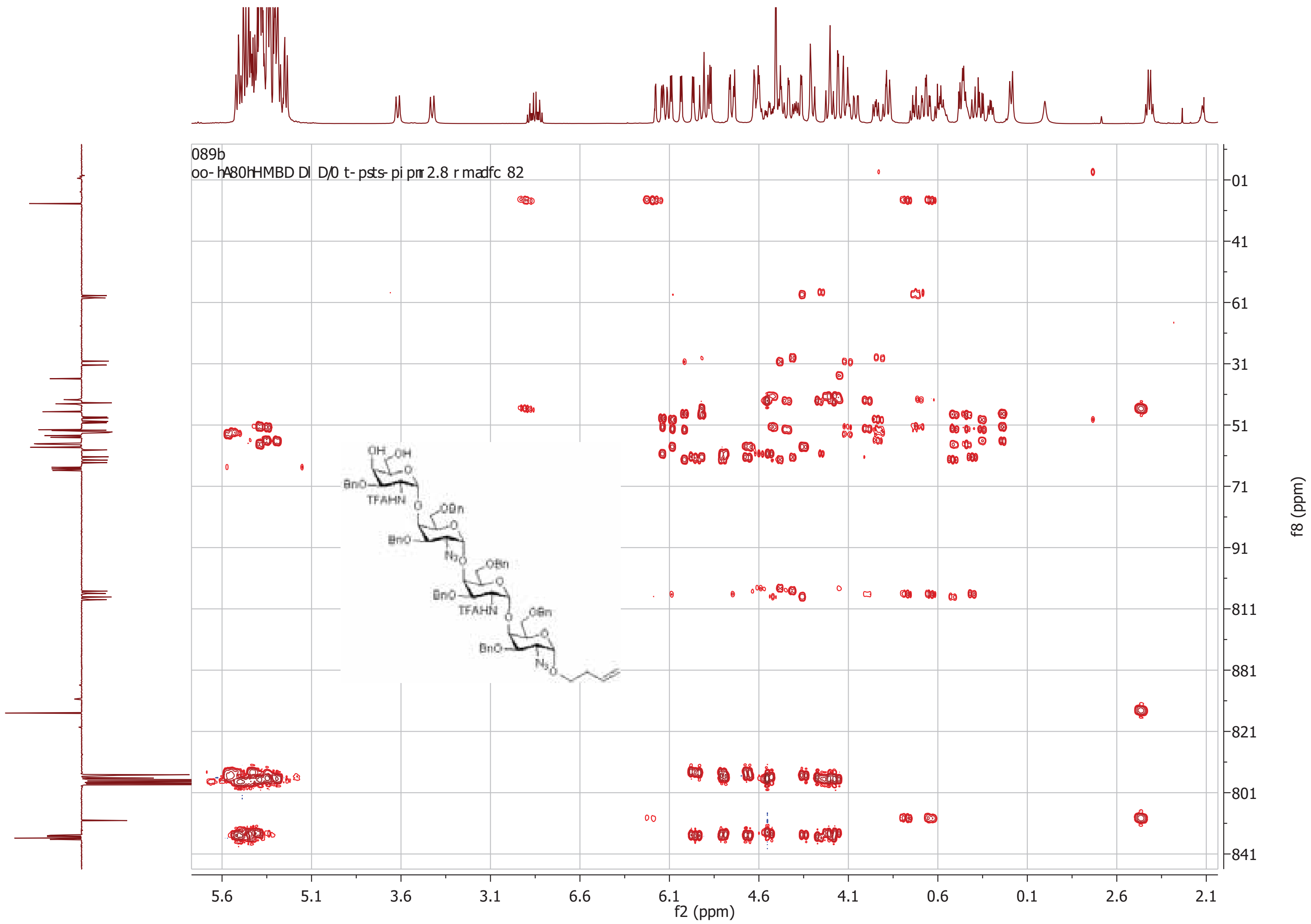
089b
oo- hA80hHMBD Dl D/0 t- psts- pi pnr 2.8 r madfc 82
f2 (ppm)
f8 (ppm)
BnO
TFAHN
OBn
N3
OH

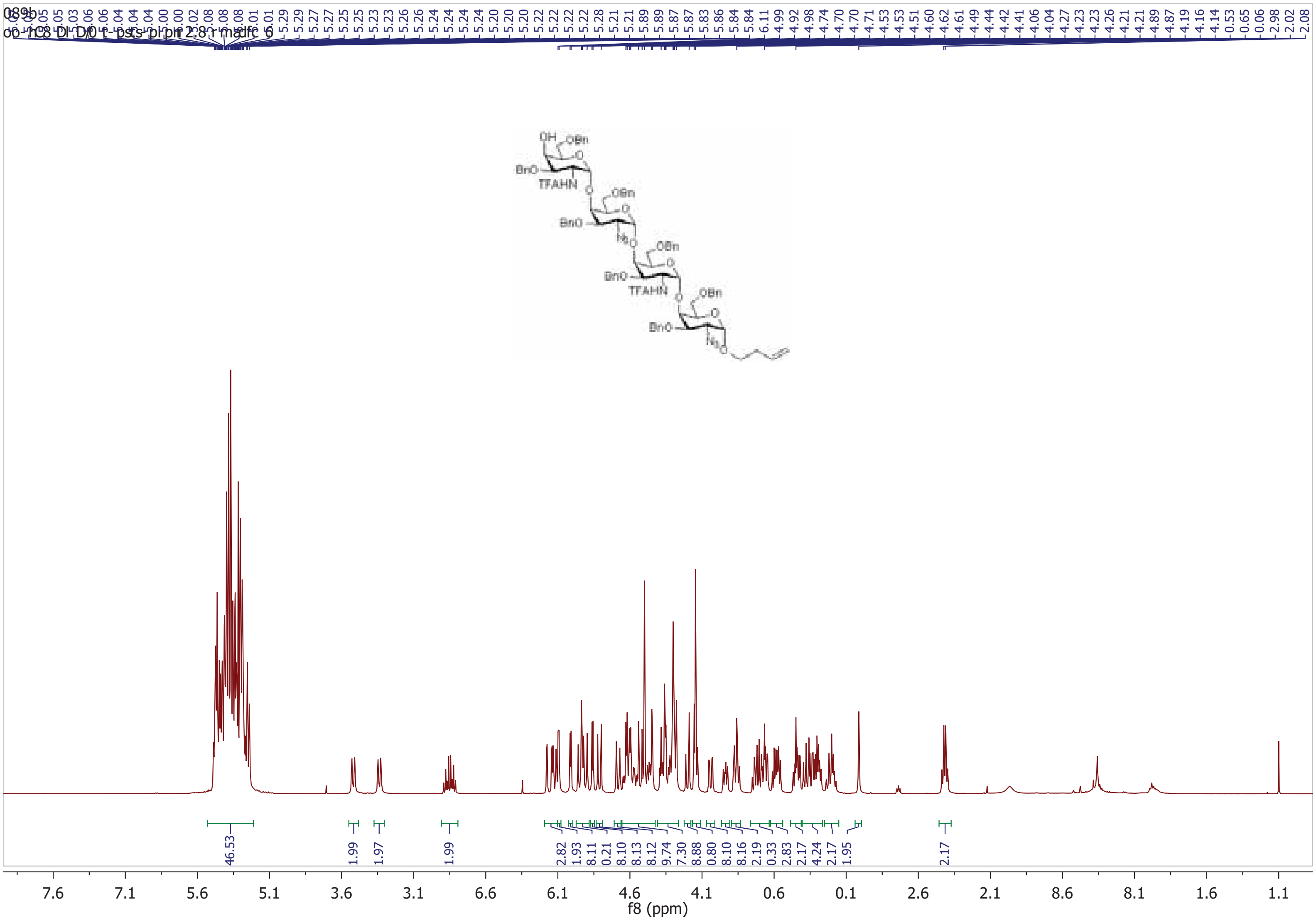

OH
OBn
BnO
TFAHN
N3
46.53
1.99
1.97
1.99
2.17
f8 (ppm)

089b
oo- hA80hPTy DI D/0 t- psts- pi pm 2.8 r madfc 6

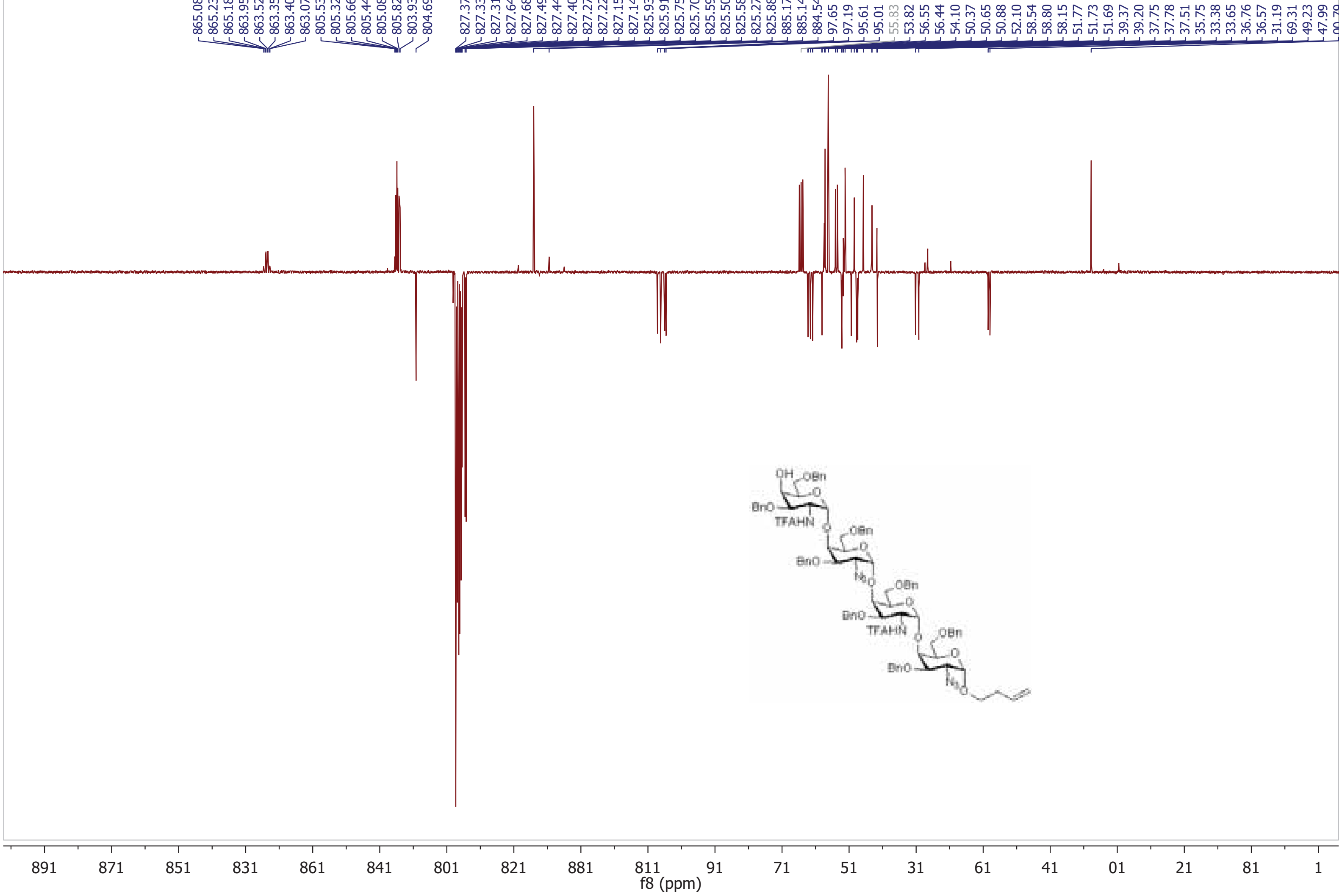

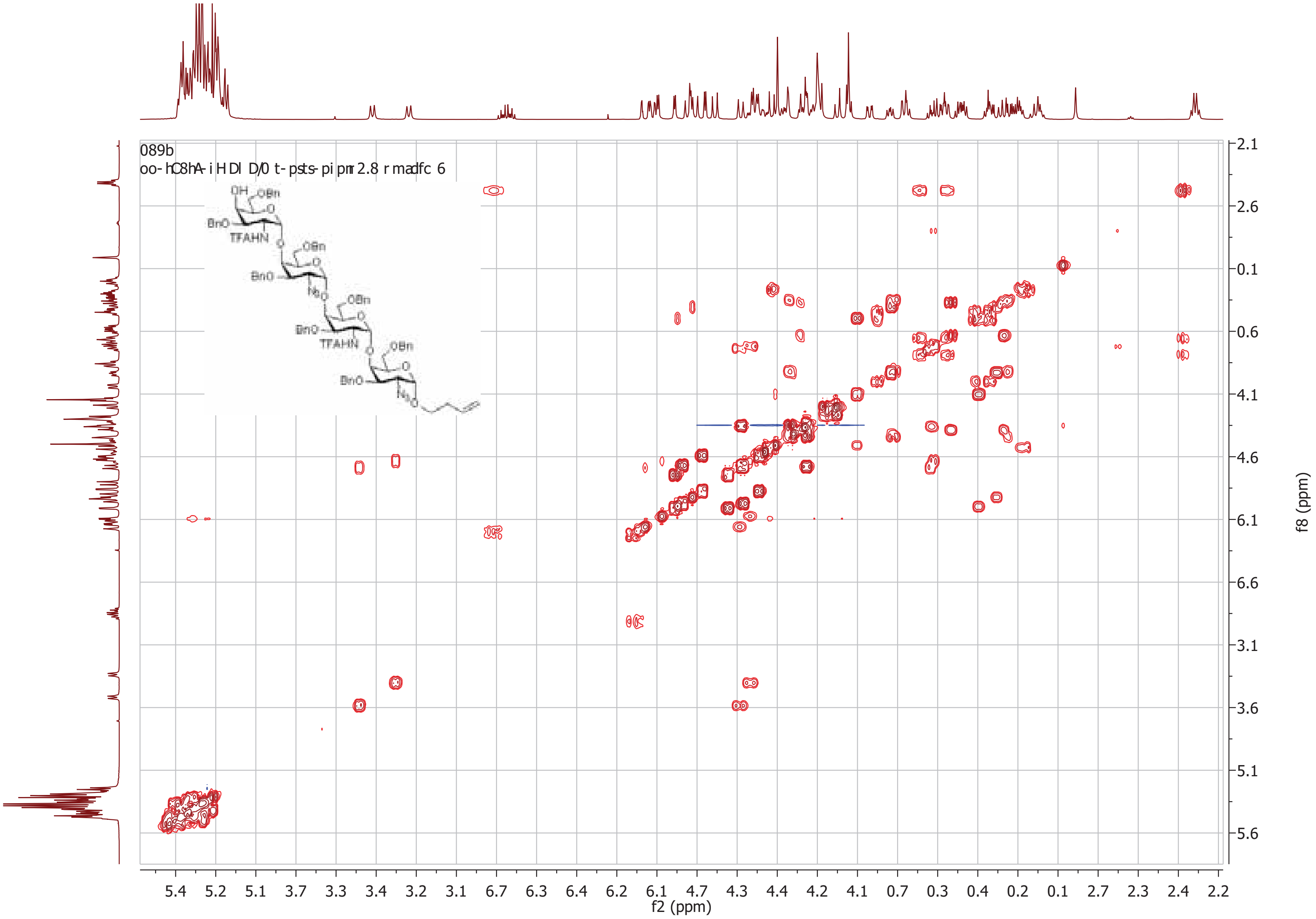
089b
f2 (ppm)
f8 (ppm)
OH
OBn
BnO
TFAHN
N3

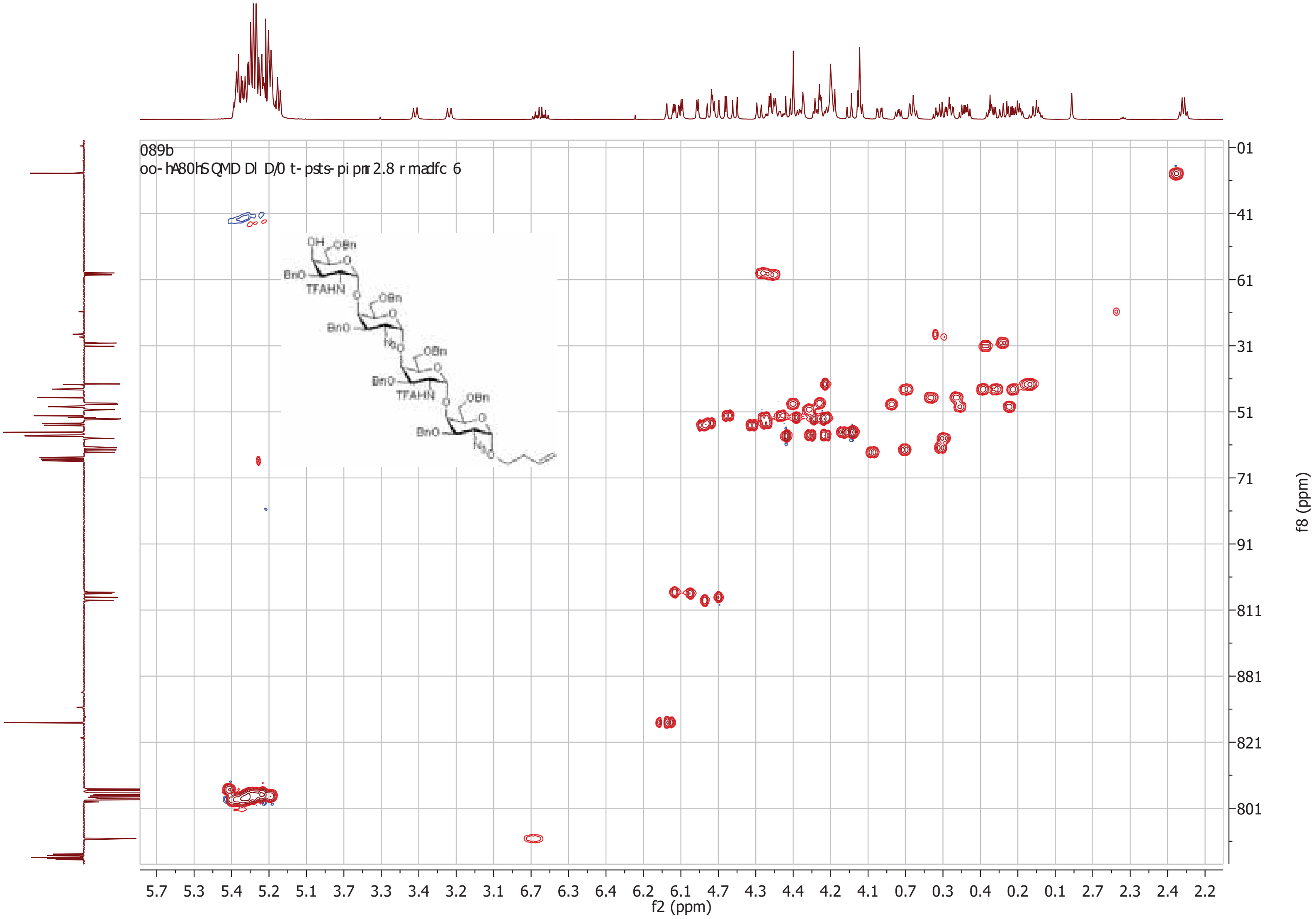

089b
oo- hA80hSQMD Dl D/0 t- psts- pi pmr 2.8 r madfc 6
OH
OBn
BnO
TFAHN
OBn
BnO
N3
OBn
BnO
TFAHN
OBn
BnO
N3
f2 (ppm)
f8 (ppm)

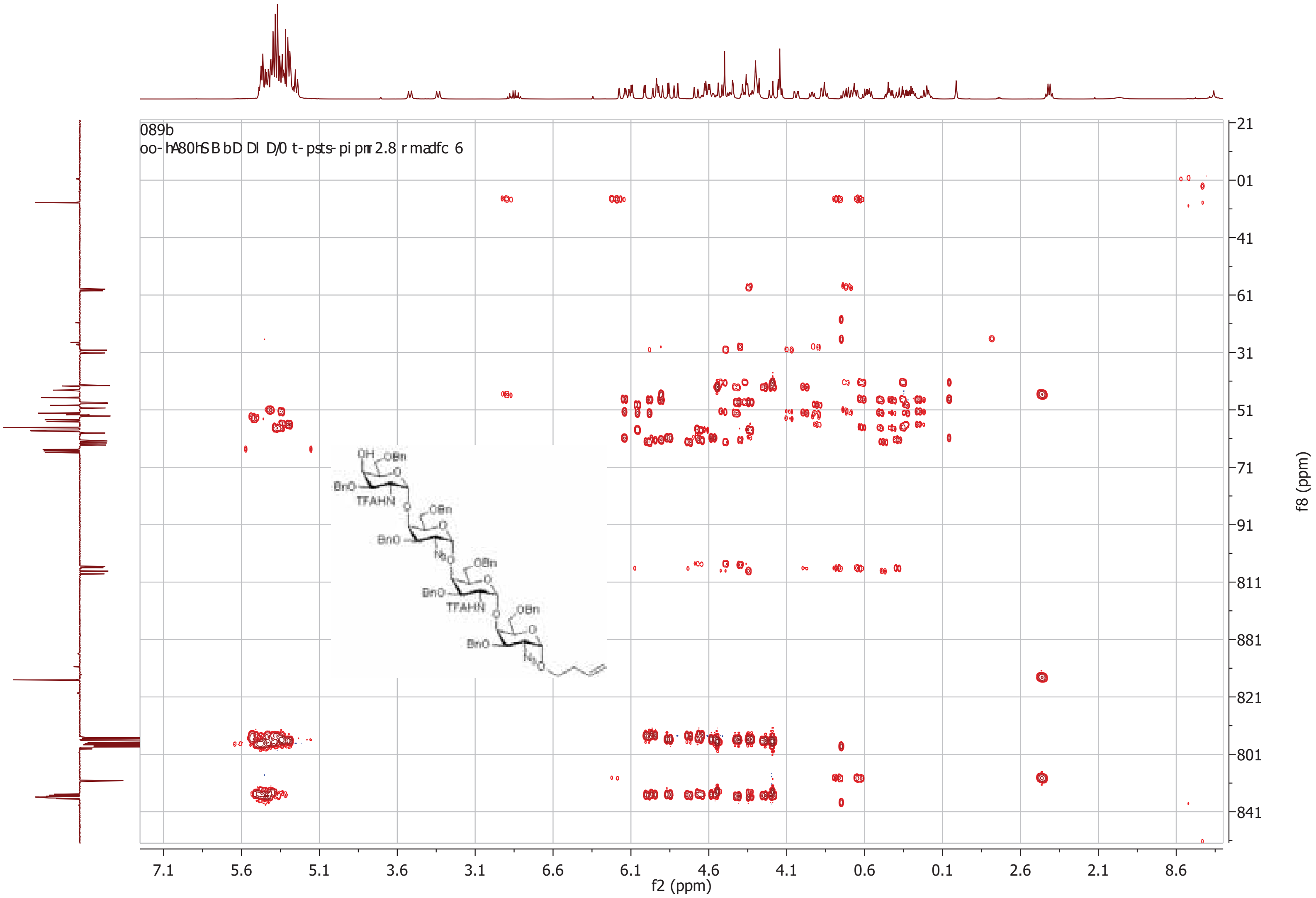
089b
oo- hA80hSB bD DI D/0 t- psts- pi pm 2.8 r madfc 6
7.1
5.6
5.1
3.6
3.1
6.6
6.1
4.6
4.1
0.6
0.1
2.6
2.1
8.6
f2 (ppm)
21
01
41
61
31
51
71
91
811
881
821
801
841
f8 (ppm)
OH
OBn
BnO
TFAHN
OBn
BnO
OBn
BnO
TFAHN
OBn
BnO

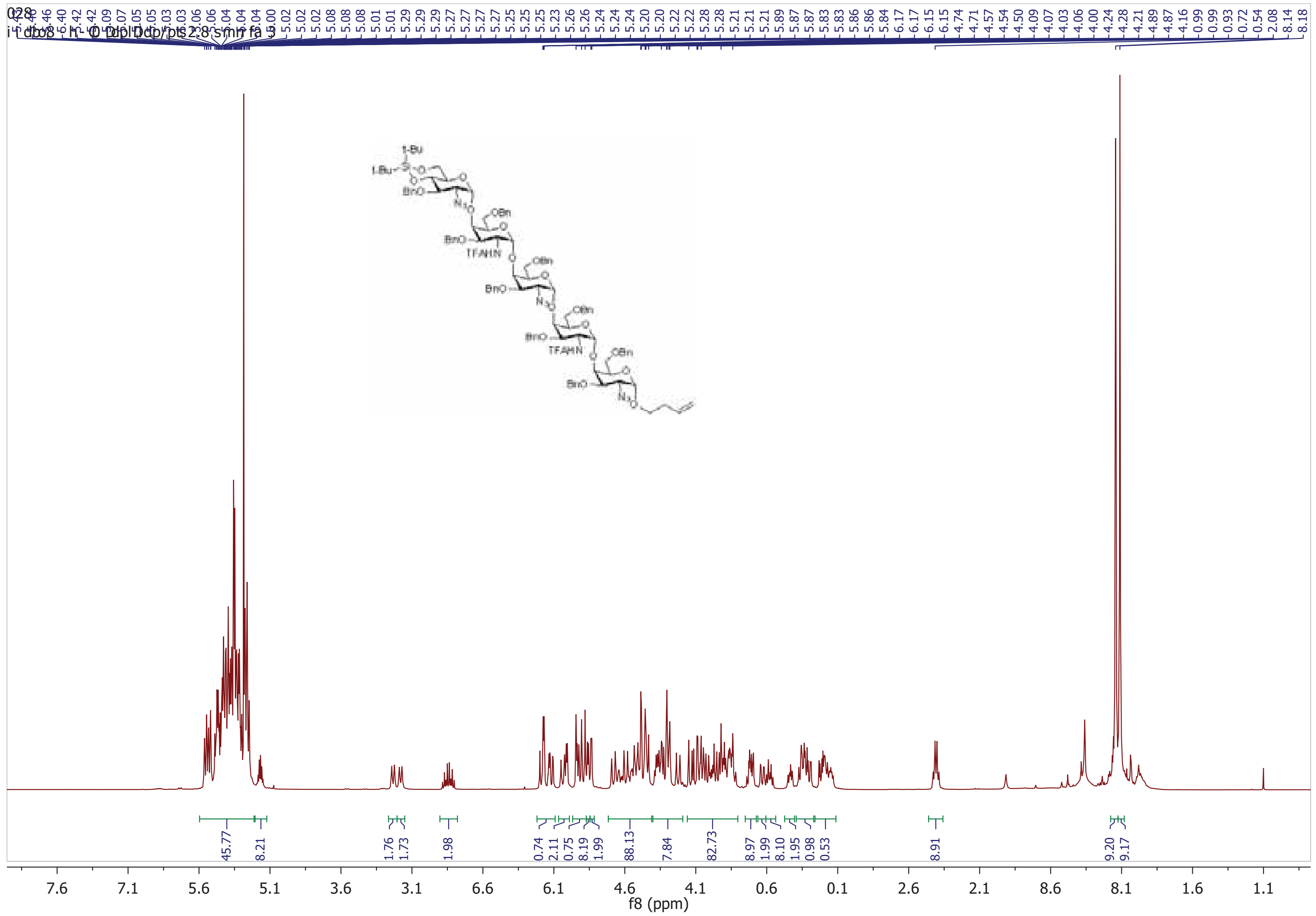
t-Bu
t-Bu
Si
BnO
N3
OBn
BnO
TFAHN
OBn
BnO
N3
OBn
BnO
TFAHN
OBn
BnO
N3
45.77
8.21
1.76
1.73
1.98
0.74
2.11
0.75
8.19
1.99
88.13
7.84
82.73
8.97
1.99
8.10
1.95
0.98
0.53
8.91
9.20
9.17
7.6 7.1 5.6 5.1 3.6 3.1 6.6 6.1 4.6 4.1 0.6 0.1 2.6 2.1 8.6 8.1 1.6 1.1
f8 (ppm)

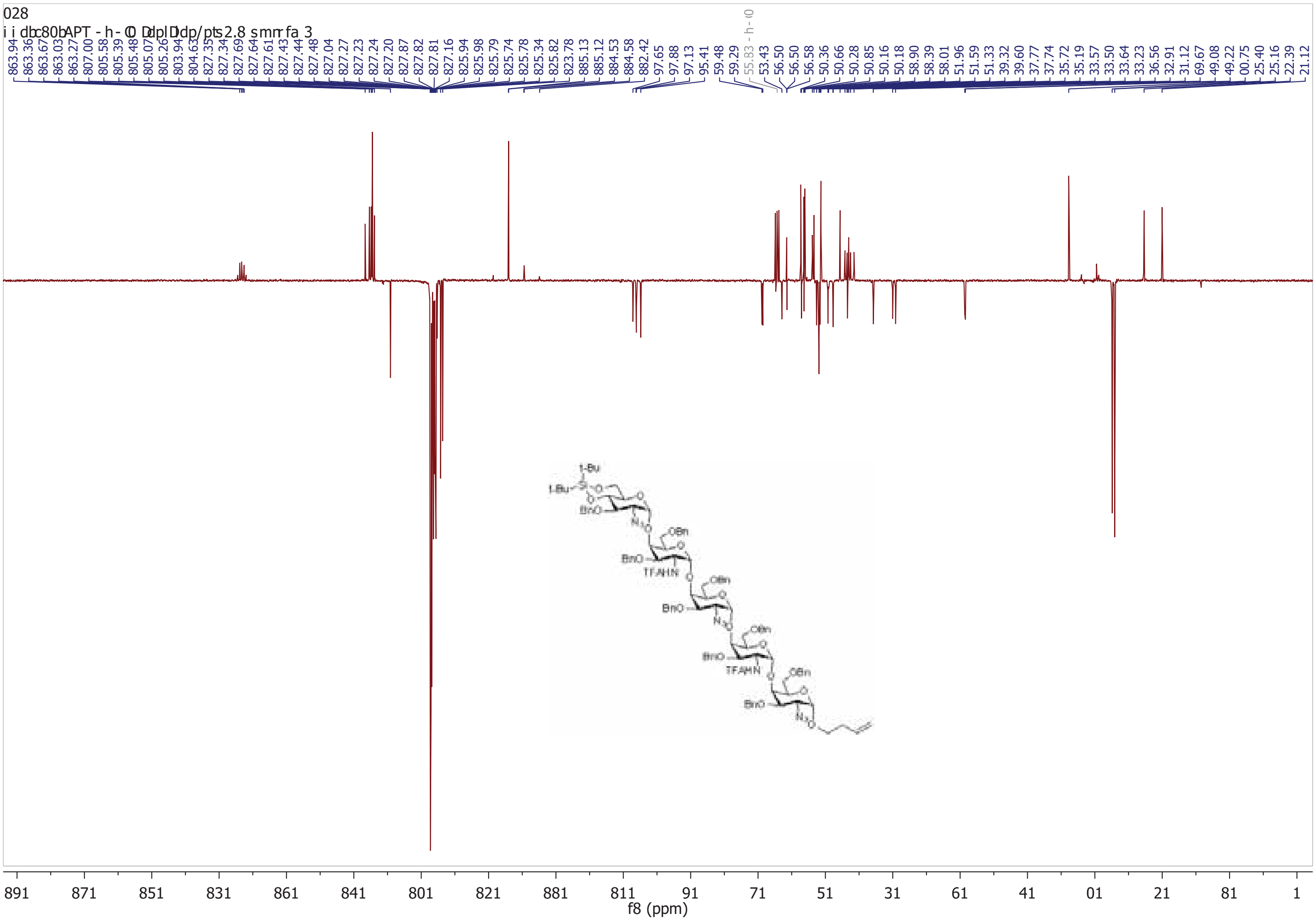

028
i i dbc80bAPT - h- 0 Ddp|Ddp/pts2.8 smrrfa 3
863.94
863.36
863.67
863.03
863.27
807.00
805.58
805.39
805.48
805.07
805.26
803.94
804.63
827.35
827.34
827.69
827.64
827.61
827.43
827.44
827.48
827.04
827.27
827.23
827.24
827.20
827.87
827.82
827.81
827.16
825.94
825.98
825.79
825.74
825.78
825.34
825.82
823.78
885.13
885.12
884.53
884.58
882.42
97.65
97.88
97.13
95.41
59.48
59.29
55.83 - h- 0
53.43
56.50
56.50
56.58
50.36
50.66
50.28
50.85
50.16
50.18
58.90
58.39
58.01
51.96
51.59
51.33
39.32
39.60
37.77
37.74
35.72
35.19
33.57
33.50
33.64
33.23
36.56
32.91
31.12
69.67
49.08
49.22
00.75
25.40
25.16
22.39
21.12
t-Bu
t-Bu
Si
O
BnO
N3
OBn
BnO
TFAHN
OBn
BnO
N3
OBn
BnO
TFAHN
OBn
BnO
N3
891
871
851
831
861
841
801
821
881
811
91
71
51
31
61
41
01
21
81
1
f8 (ppm)

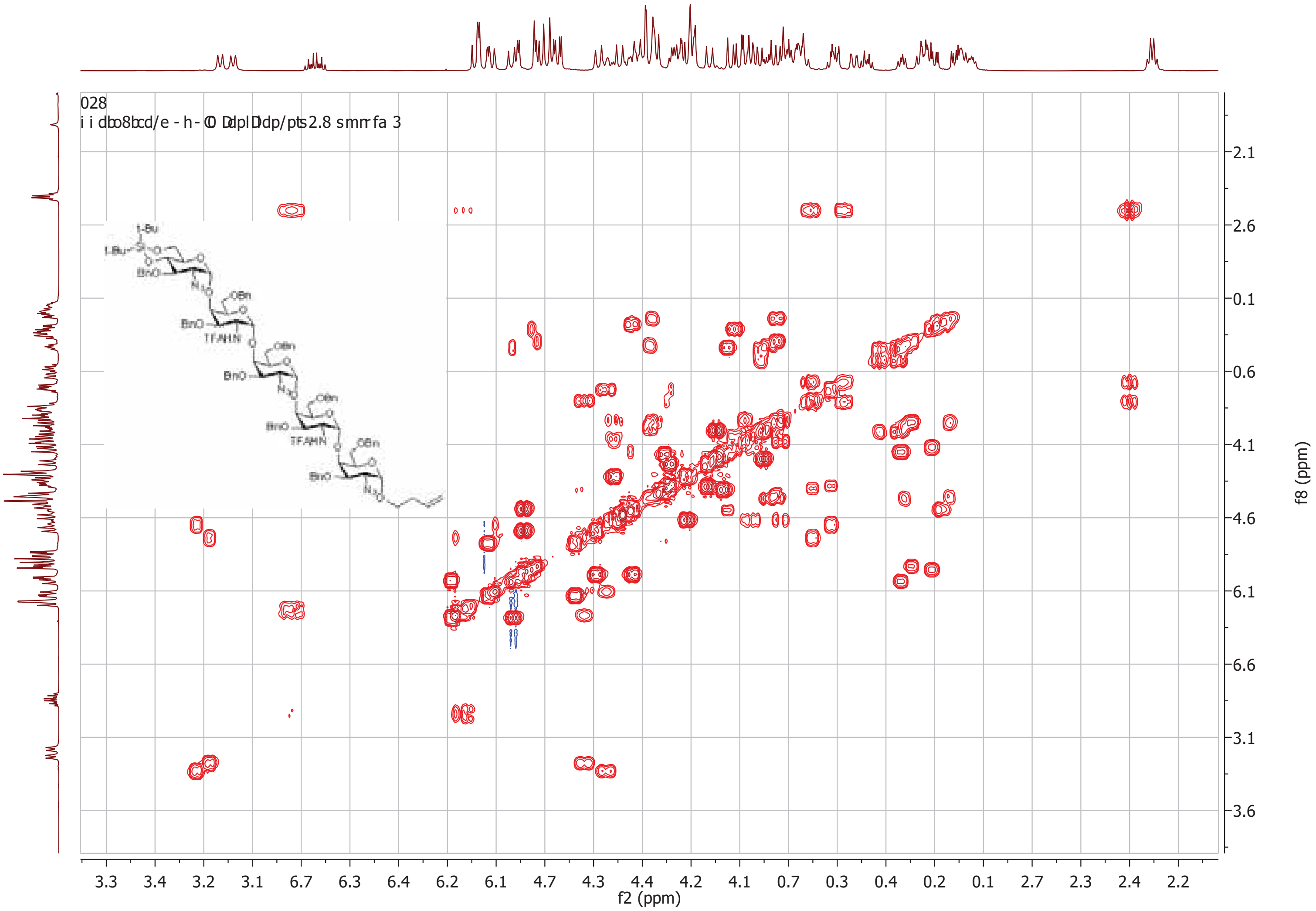
028
i i dbo8bcd/e - h- 0 Ddpl Ddp/pts2.8 smmfa 3
t-Bu
Si
t-Bu
BnO
N3
OBn
TFAHN
f2 (ppm)
f8 (ppm)

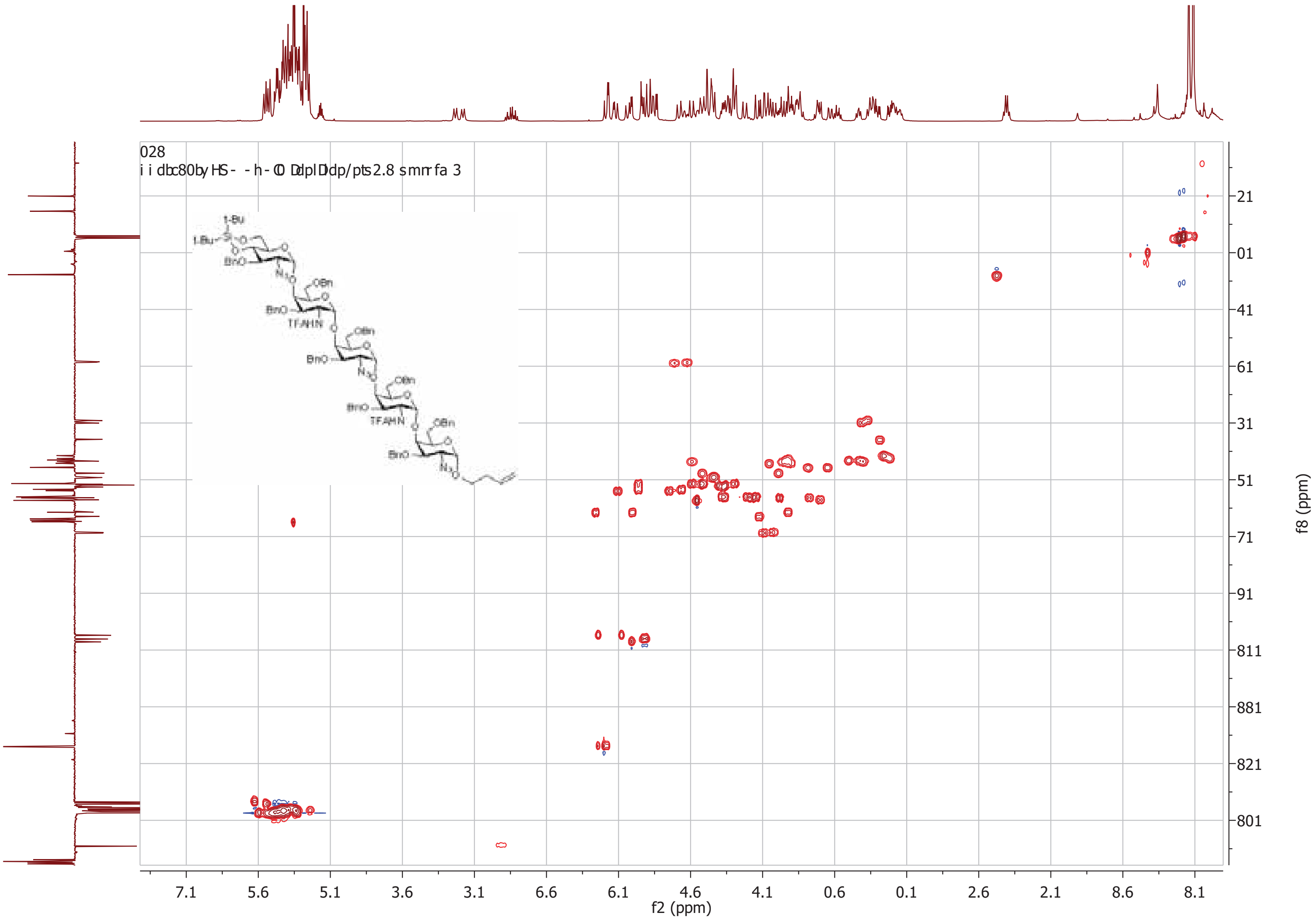
028
f2 (ppm)
f8 (ppm)
t-Bu
t-Bu
BnO
OBn
TFAHN
N3

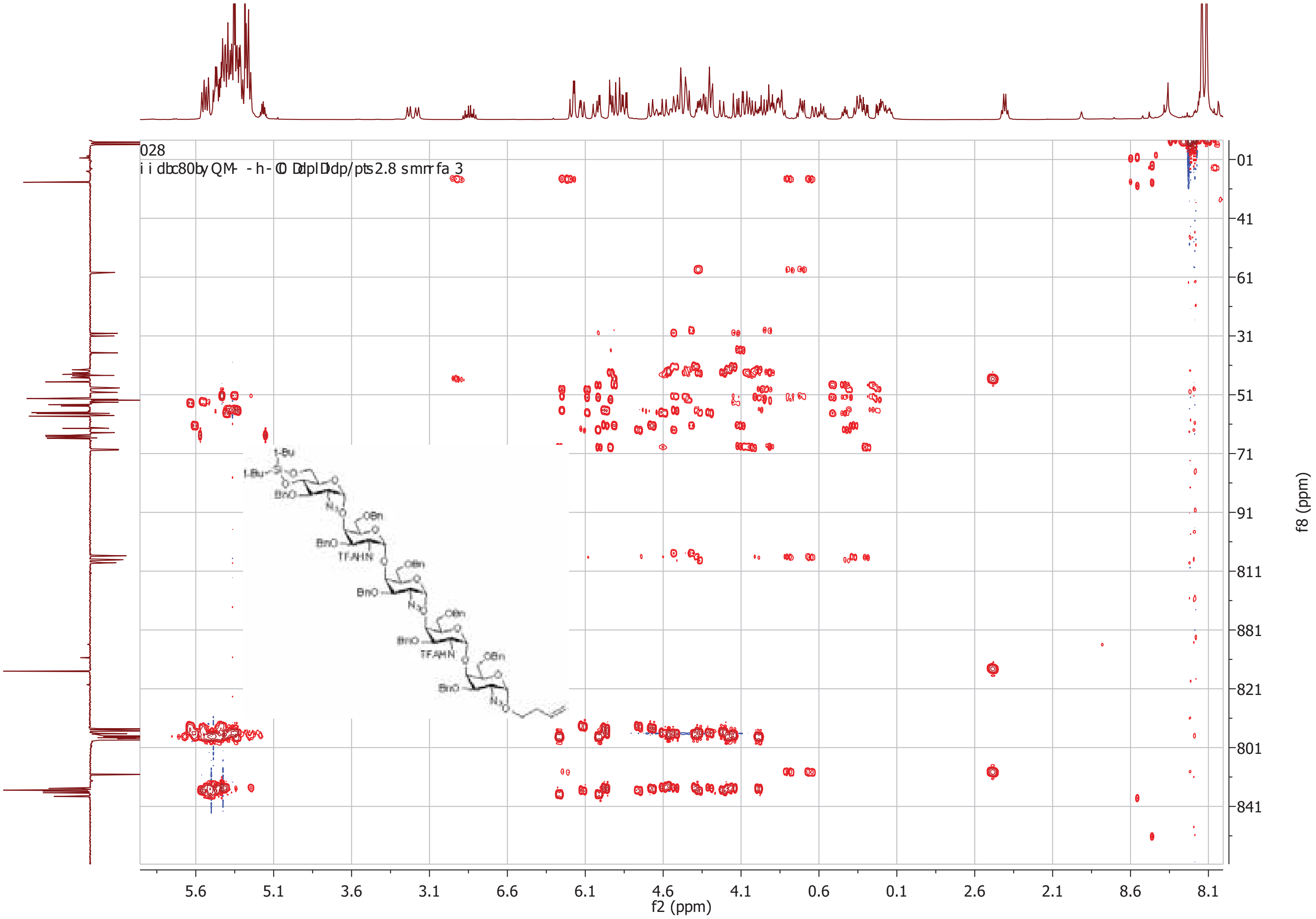
028
f2 (ppm)
f8 (ppm)
t-Bu
t-Bu
BnO
OBn
TFAHN

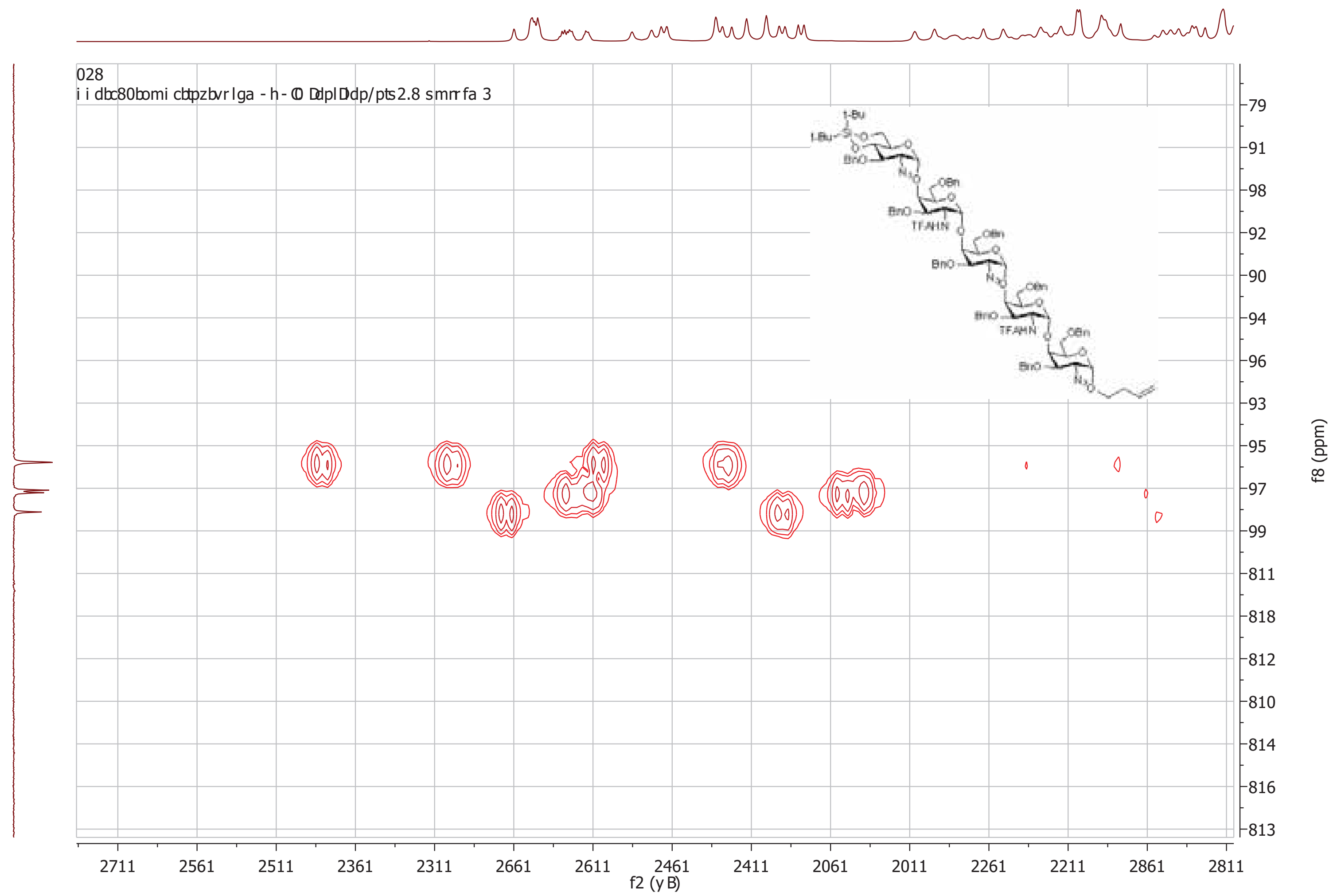
028
i i dbc80bomi cbtpzbvrlga -h-ClO DdplDdp/pts2.8 smmfa 3
f2 (y B)
f8 (ppm)
t-Bu
t-Bu
BnO
N3
OBn
TFAHN

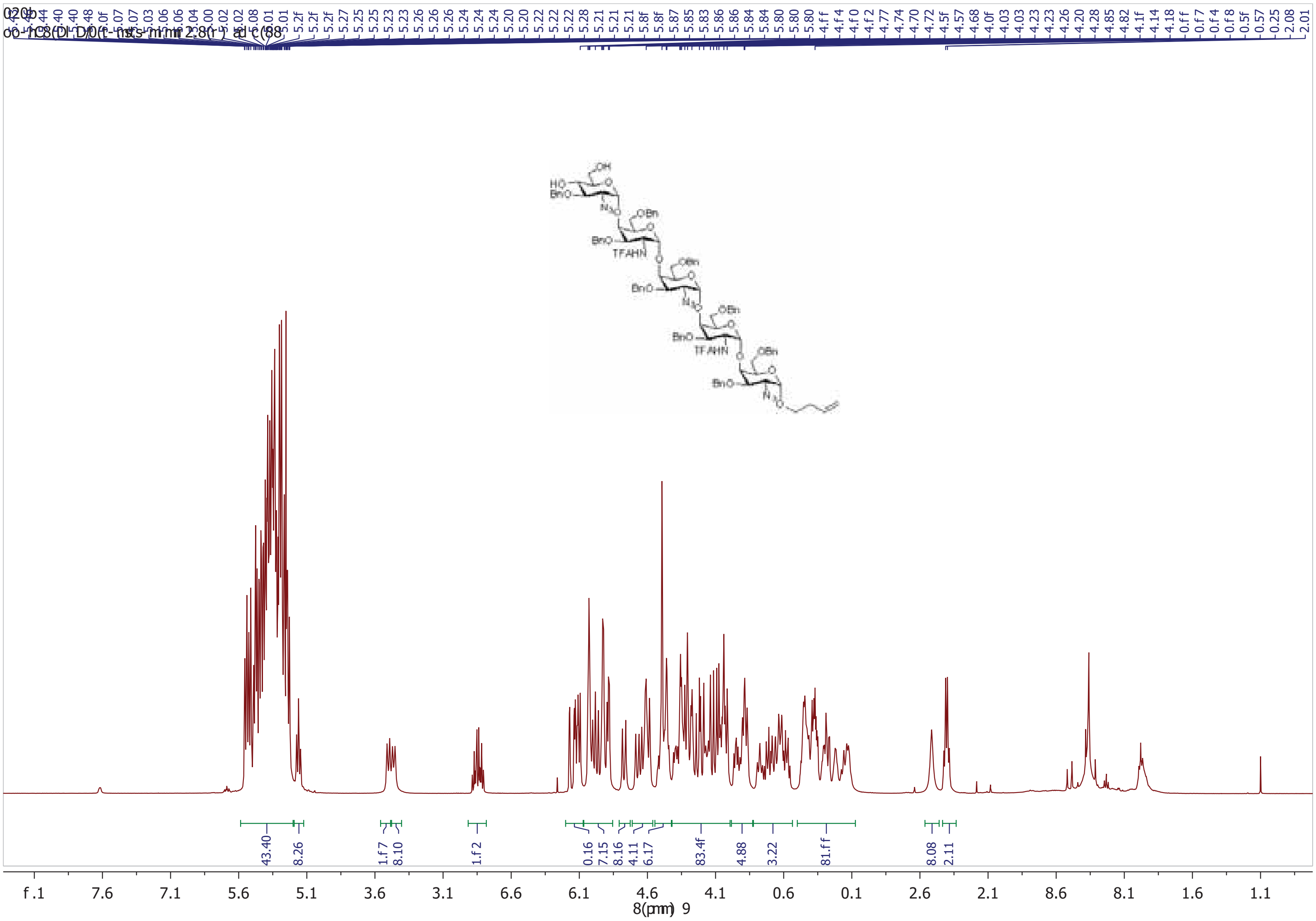
HO
BnO
OH
OBn
TFAHN
BnO
43.40
8.26
8.10
0.16
7.15
8.16
4.11
6.17
4.88
3.22
8.08
2.11

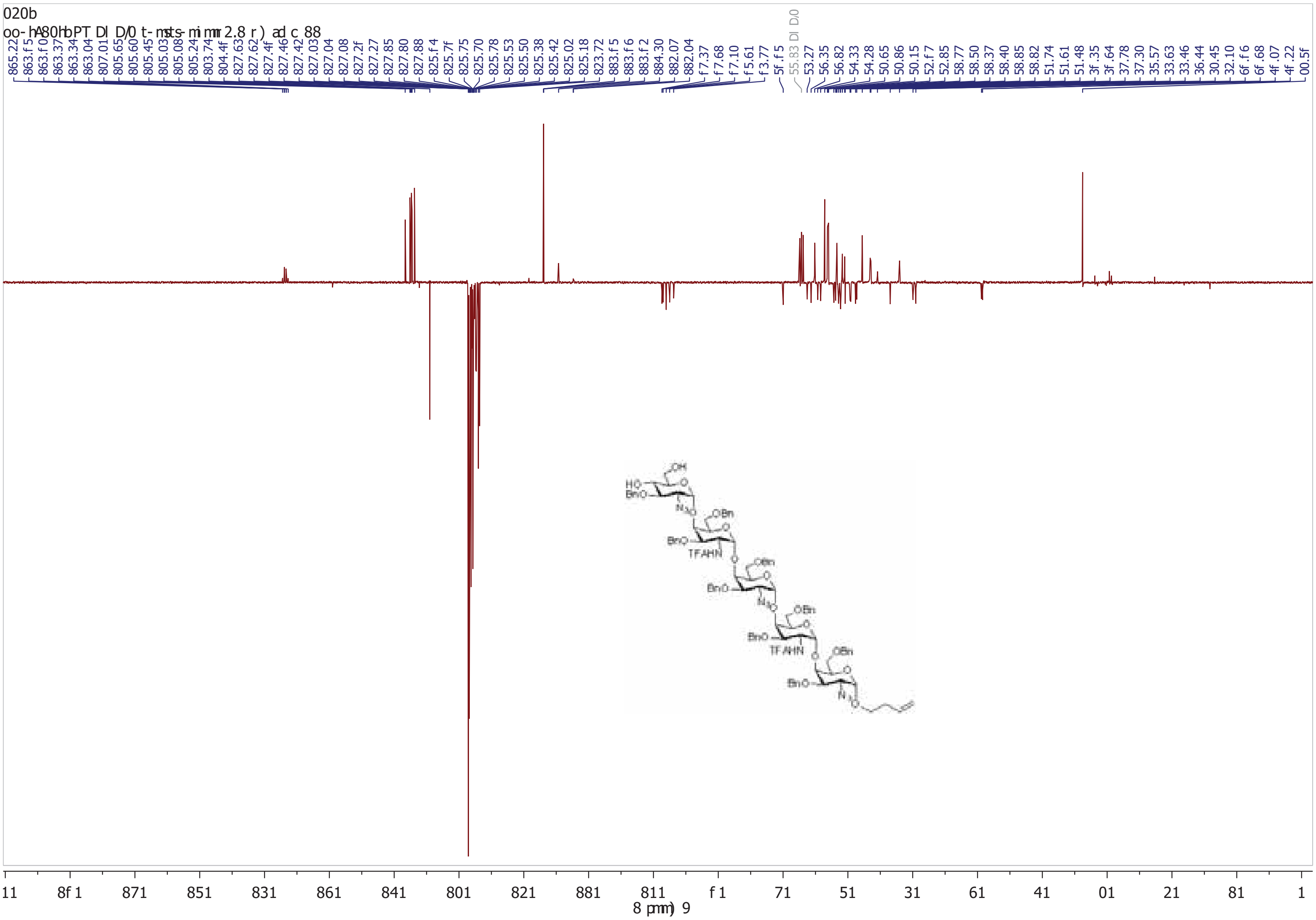
020b
oo-hA80hbPT DI D/0 t-msts-mimr2.8 r) ad c 88
8 pmm) 9
HO
BnO
N3
OBn
BnO
TFAHN
OBn
BnO
N3
OBn
BnO
TFAHN
OBn
BnO
N3

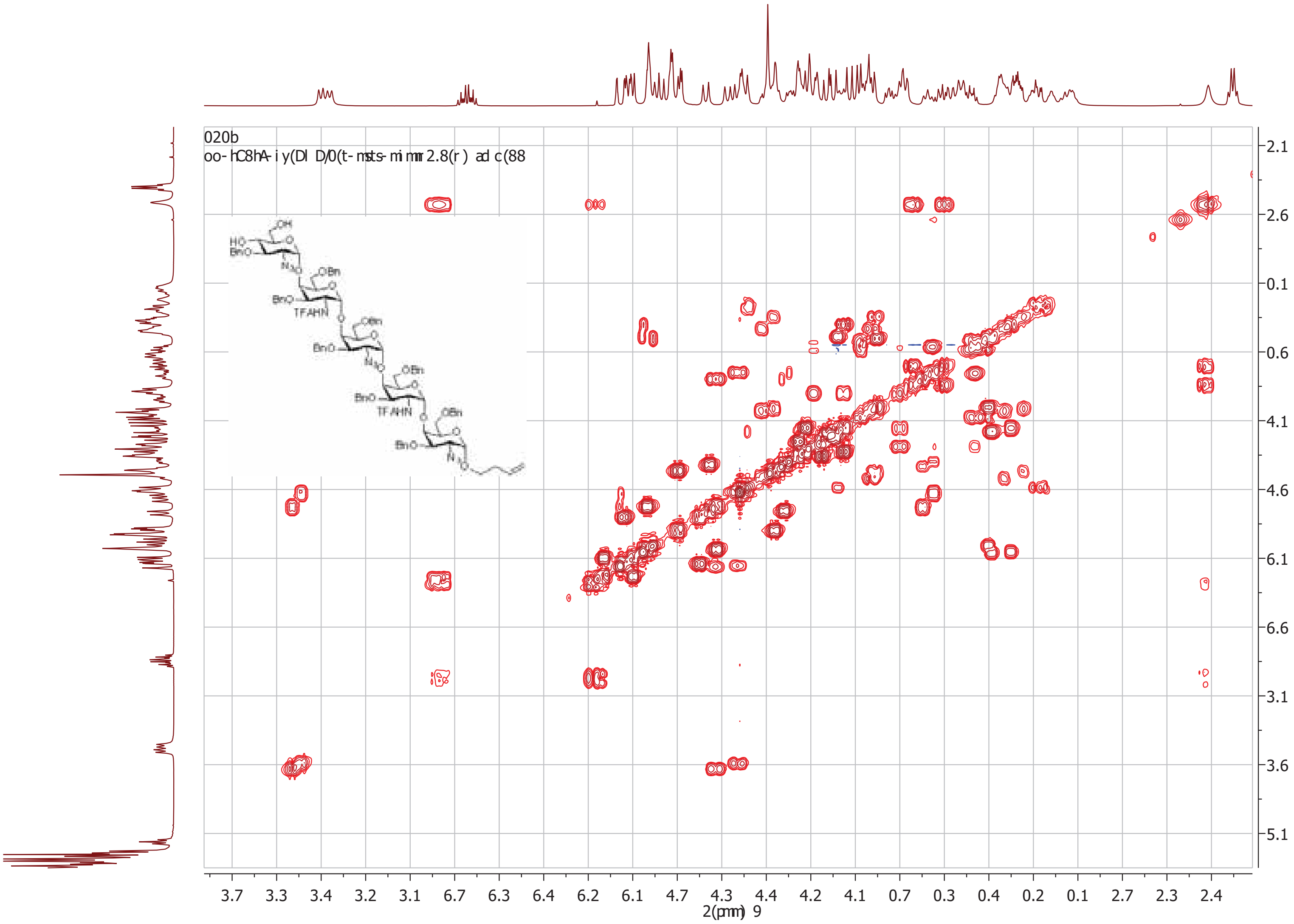
020b
oo-hC8hA-i y(DI D/0(t-msts-mi mr 2.8(r) ad c(88
OH
HO
BnO
OBn
TFAHN
2(pm) 9
8(pm) 9

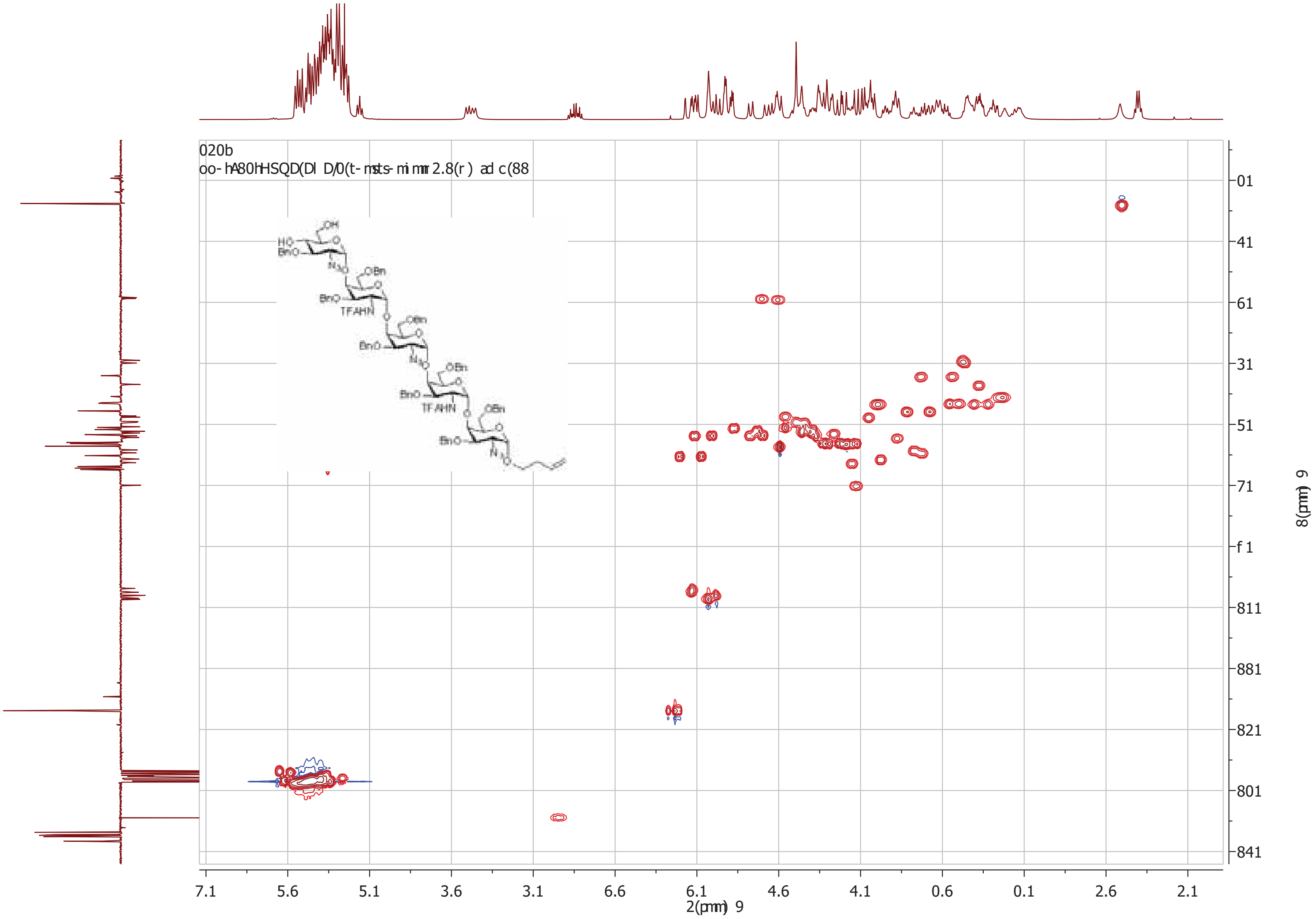
020b
oo- hA80hHSQD(DI D/0(t- mstxs- mi mr 2.8(r) ad c(88
HO
BnO
OH
OBn
TFAHN
2(pmm) 9
8(pmm) 9

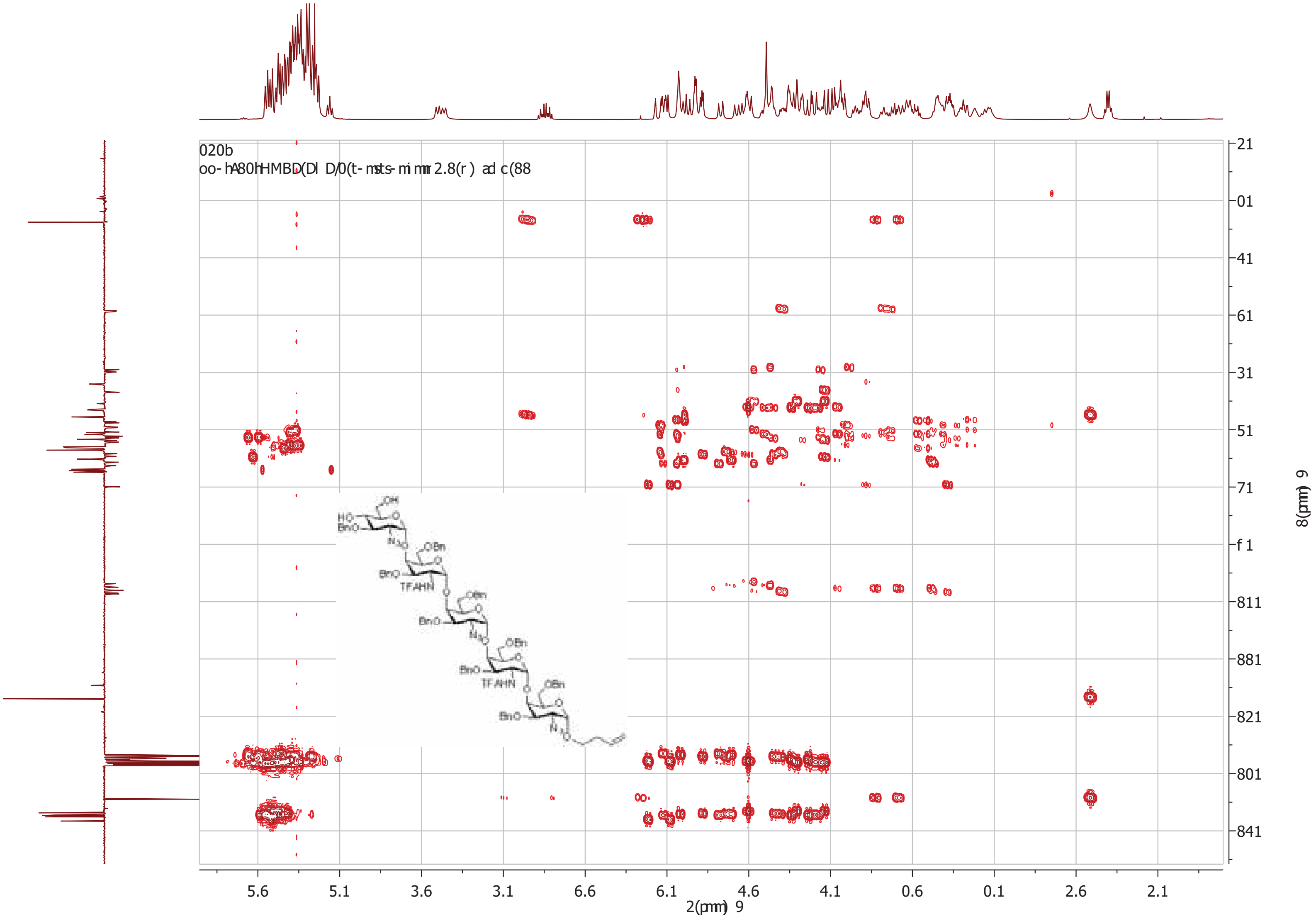
020b
oo- hA80hHMBD(DI D/0(t- msts- mi mr 2.8(r) ad c(88
2(pmm) 9
8(pmm) 9

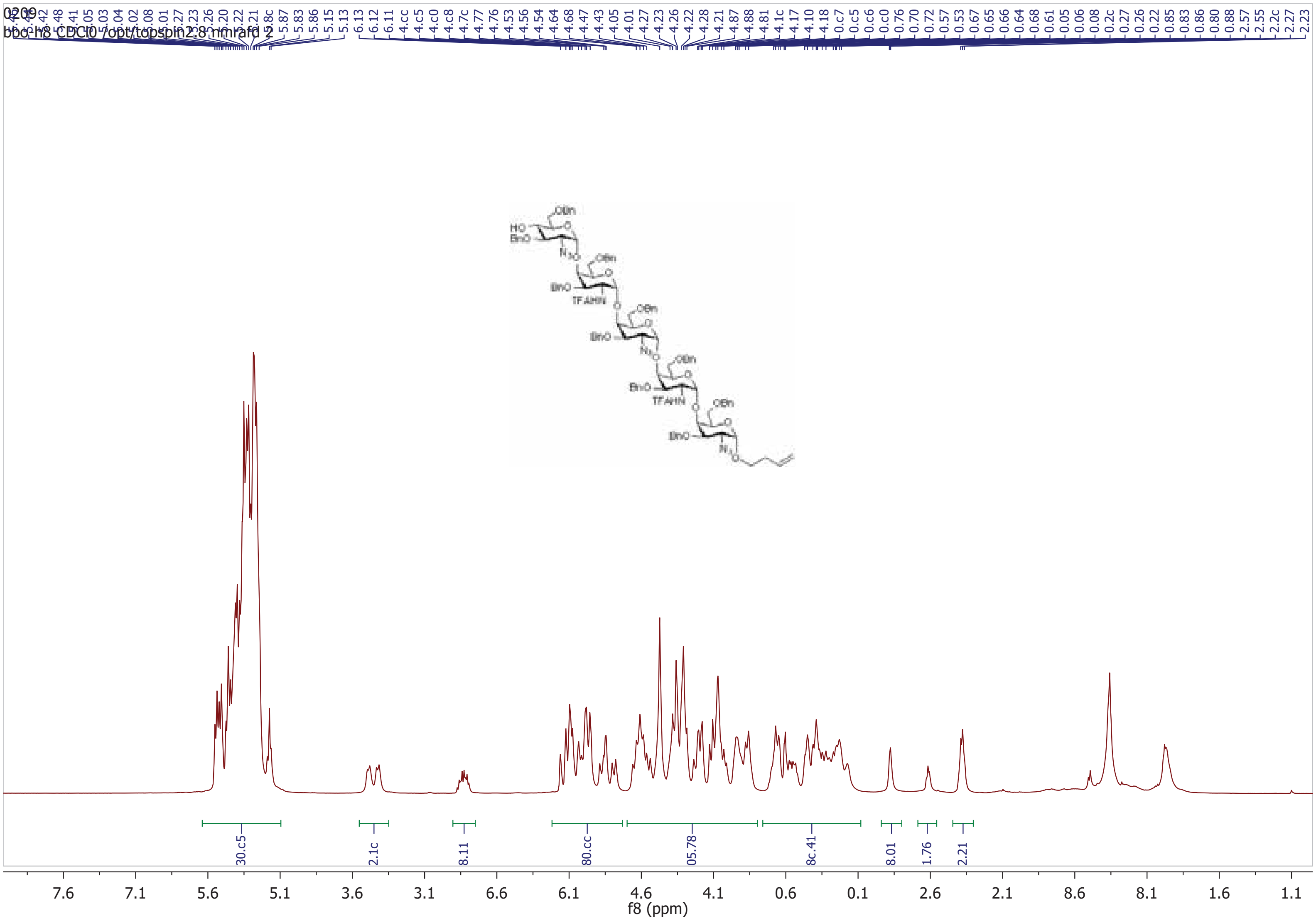

30.c5
2.1c
8.11
80.cc
05.78
8c.41
8.01
1.76
2.21
7.6
7.1
5.6
5.1
3.6
3.1
6.6
6.1
4.6
4.1
0.6
0.1
2.6
2.1
8.6
8.1
1.6
1.1
f8 (ppm)

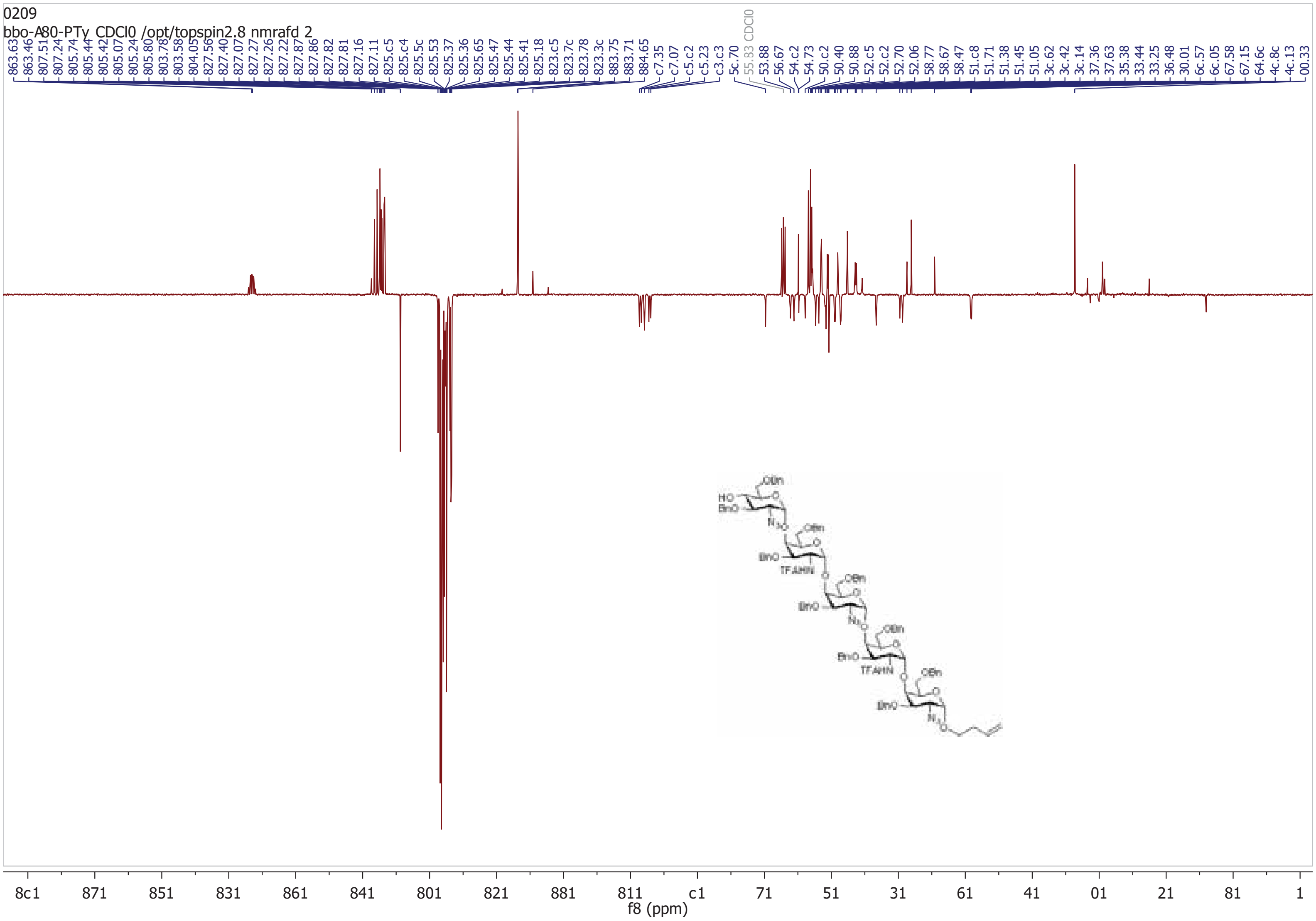
0209
bbo-A80-PTy CDCl0 /opt/topspin2.8 nmrafd 2
8c1
871
851
831
861
841
801
821
881
811
c1
71
51
31
61
41
01
21
81
1
f8 (ppm)
HO
BnO
OBn
TFAHN
N3

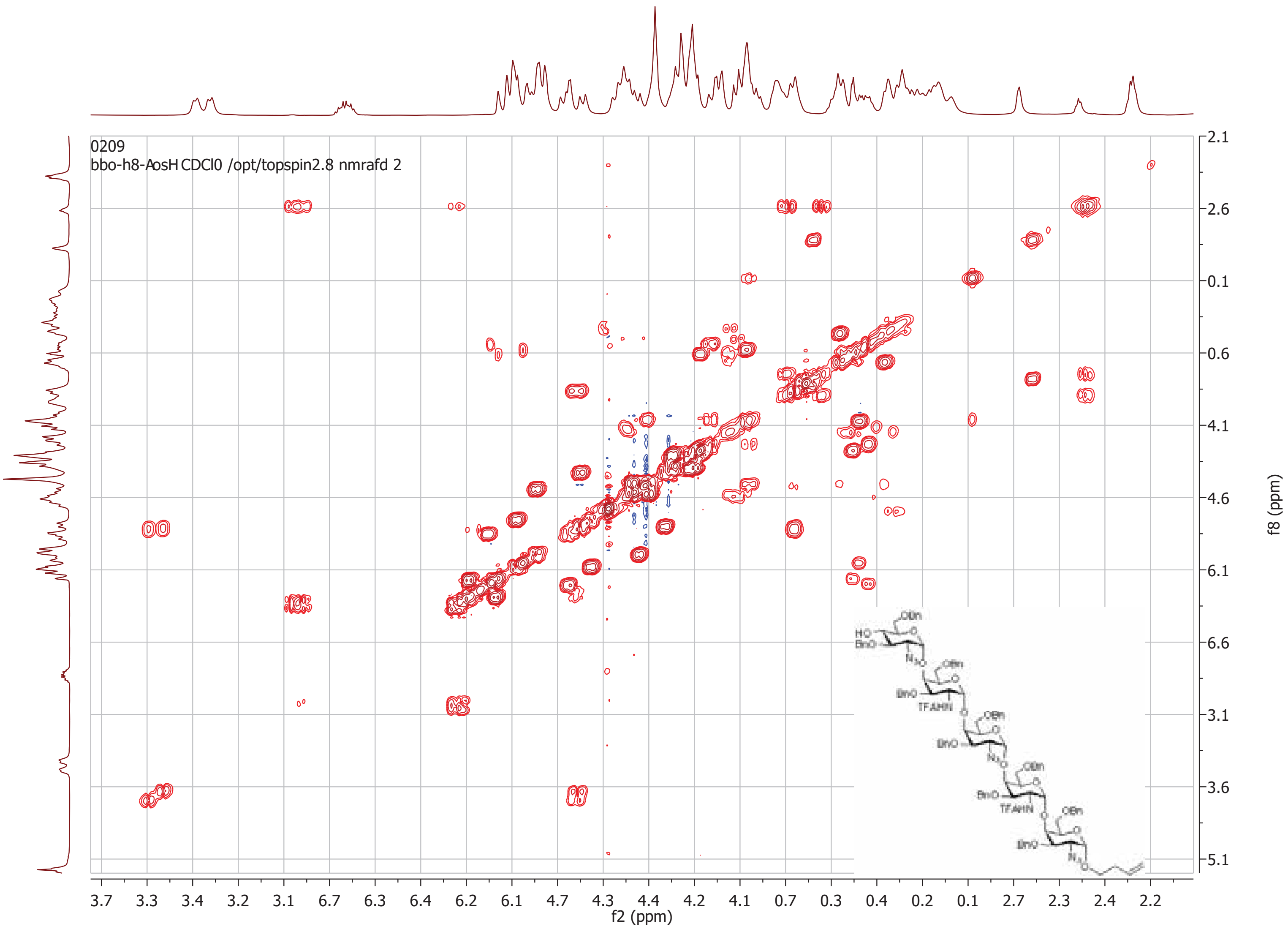
0209
bbo-h8-AosH CDCl0 /opt/topspin2.8 nmrafd 2
3.7 3.3 3.4 3.2 3.1 6.7 6.3 6.4 6.2 6.1 4.7 4.3 4.4 4.2 4.1 0.7 0.3 0.4 0.2 0.1 2.7 2.3 2.4 2.2
f2 (ppm)
2.1 2.6 0.1 0.6 4.1 4.6 6.1 6.6 3.1 3.6 5.1
f8 (ppm)
OBn
HO
BnO
N3
TFAHN

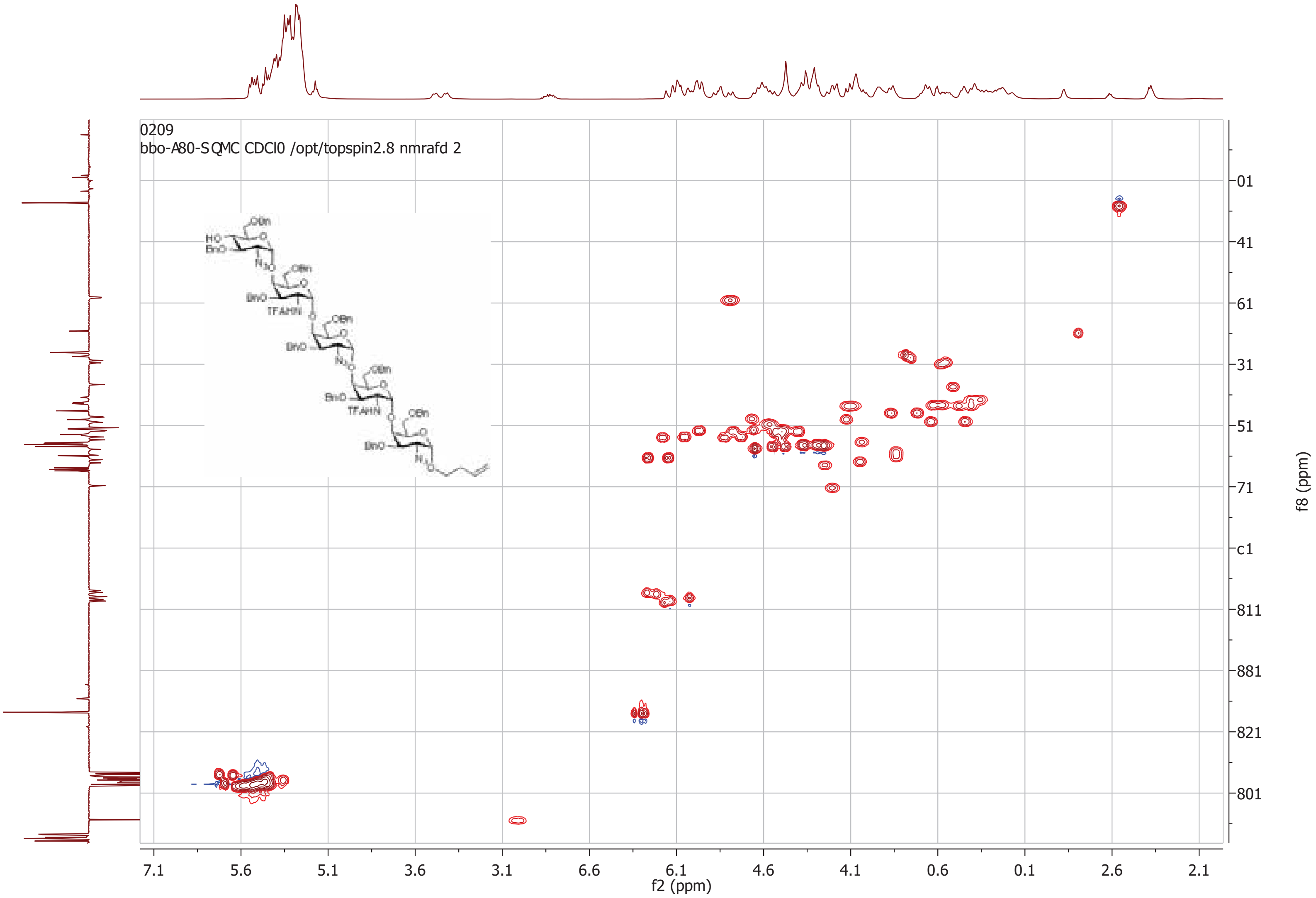

0209
bbo-A80-SQMC CDCl0 /opt/topspin2.8 nmrafd 2
f2 (ppm)
f8 (ppm)
7.1
5.6
5.1
3.6
3.1
6.6
6.1
4.6
4.1
0.6
0.1
2.6
2.1
01
41
61
31
51
71
c1
811
881
821
801

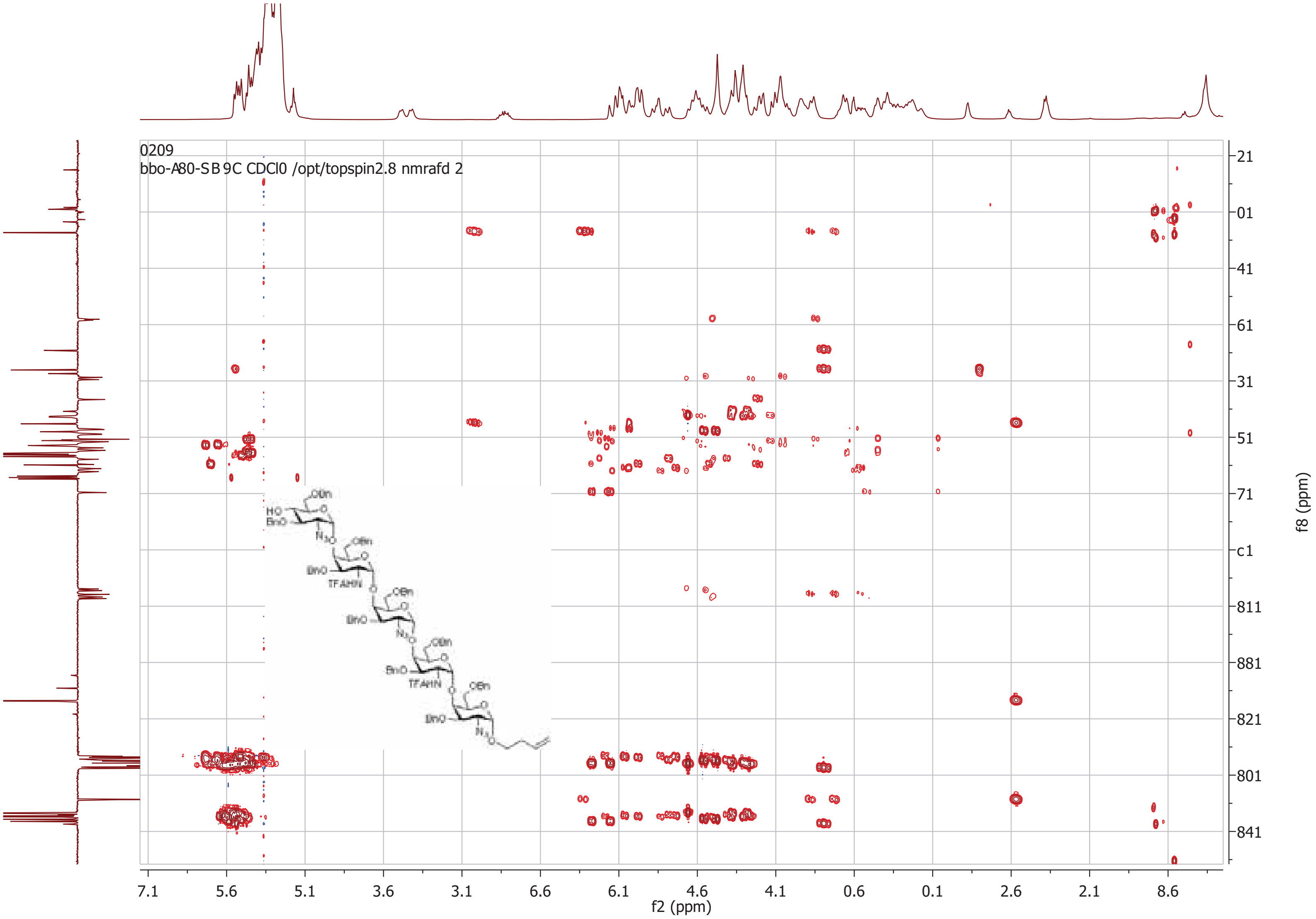
0209
bbo-A80-SB 9C CDCl0 /opt/topspin2.8 nmrafd 2
f2 (ppm)
f8 (ppm)
HO
BnO
OBn
N3
TFAHN

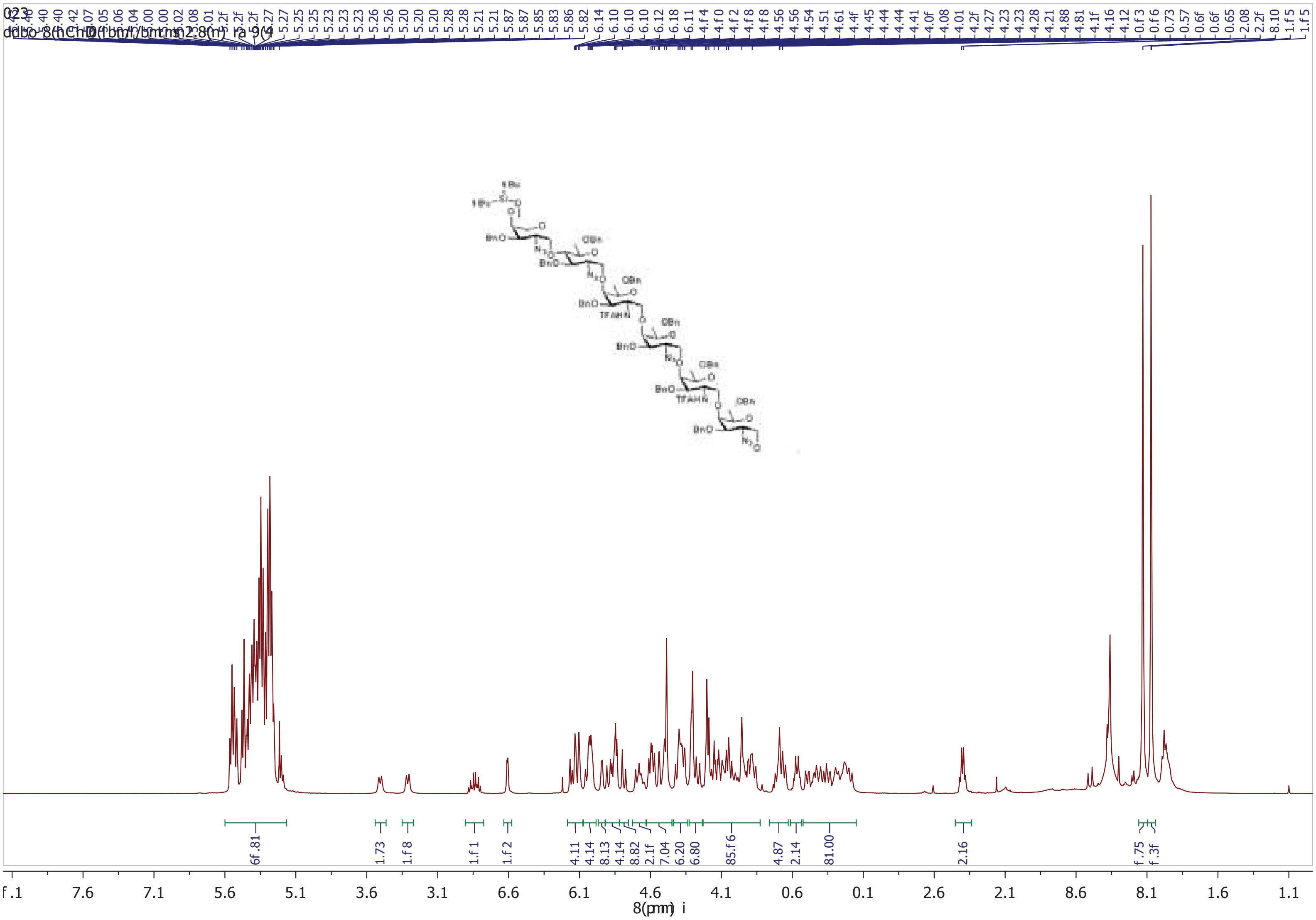

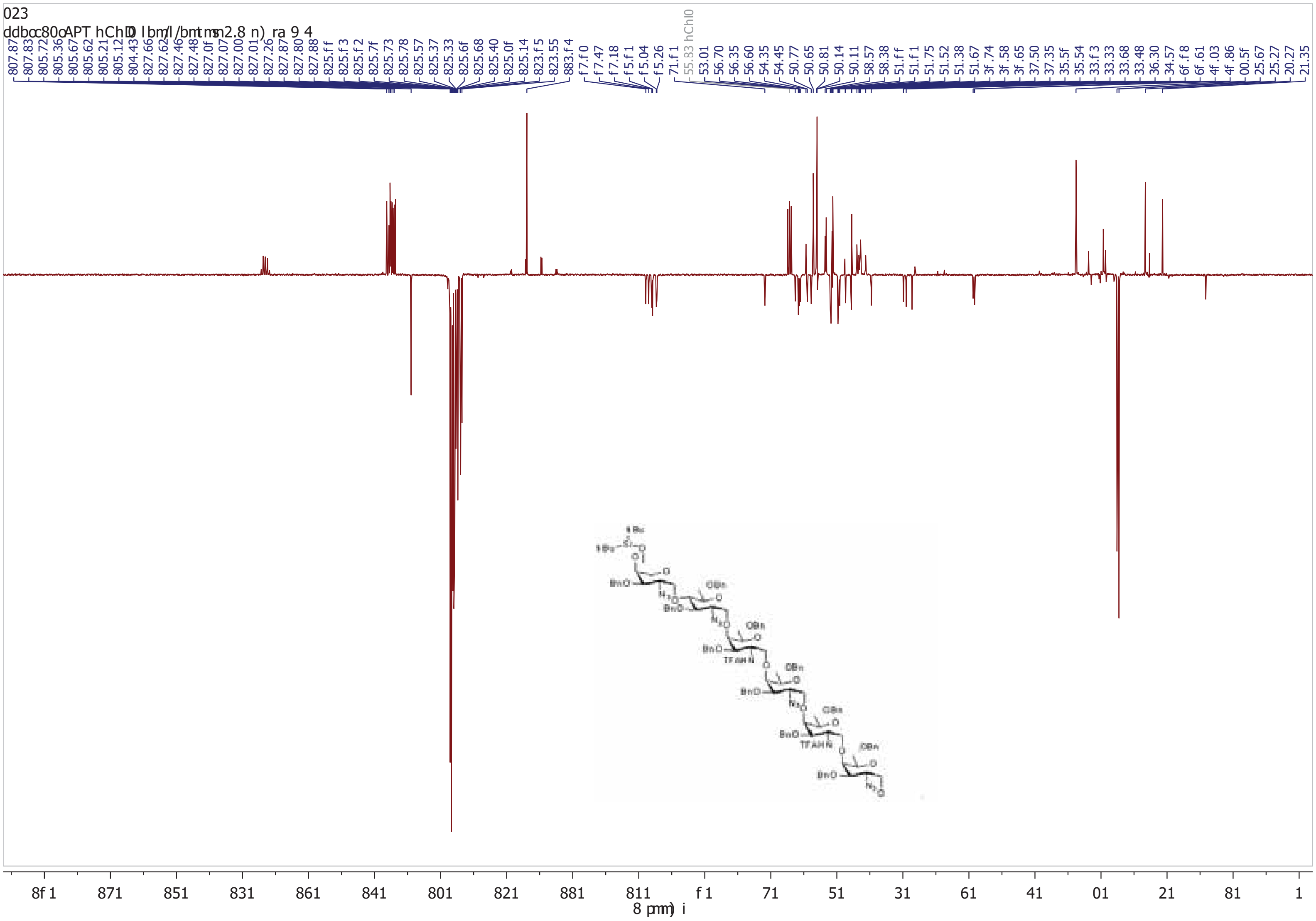
023
ddbcc80oAPT hChl0 lbm/l/bmt ms2.8 n) ra 9 4
807.87
807.83
805.72
805.36
805.67
805.62
805.21
805.12
804.43
827.66
827.62
827.46
827.48
827.0f
827.07
827.00
827.01
827.26
827.87
827.80
827.88
825.f f
825.f 3
825.f 2
825.7f
825.73
825.78
825.57
825.37
825.33
825.6f
825.68
825.40
825.0f
825.14
823.f 5
823.55
883.f 4
f 7.f 0
f 7.47
f 7.18
f 5.f 1
f 5.04
f 5.26
71.f 1
55.83 hChl0
53.01
56.70
56.35
56.60
54.35
54.45
50.77
50.65
50.81
50.14
50.11
58.57
58.38
51.f f
51.f 1
51.75
51.52
51.38
51.67
3f.74
3f.58
3f.65
37.50
37.35
35.5f
35.54
33.f 3
33.33
33.68
33.48
36.30
34.57
6f.f 8
6f.61
4f.03
4f.86
00.5f
25.67
25.27
20.27
21.35
8f 1
871
851
831
861
841
801
821
881
811
f 1
71
51
31
61
41
01
21
81
1
8 pmm) i

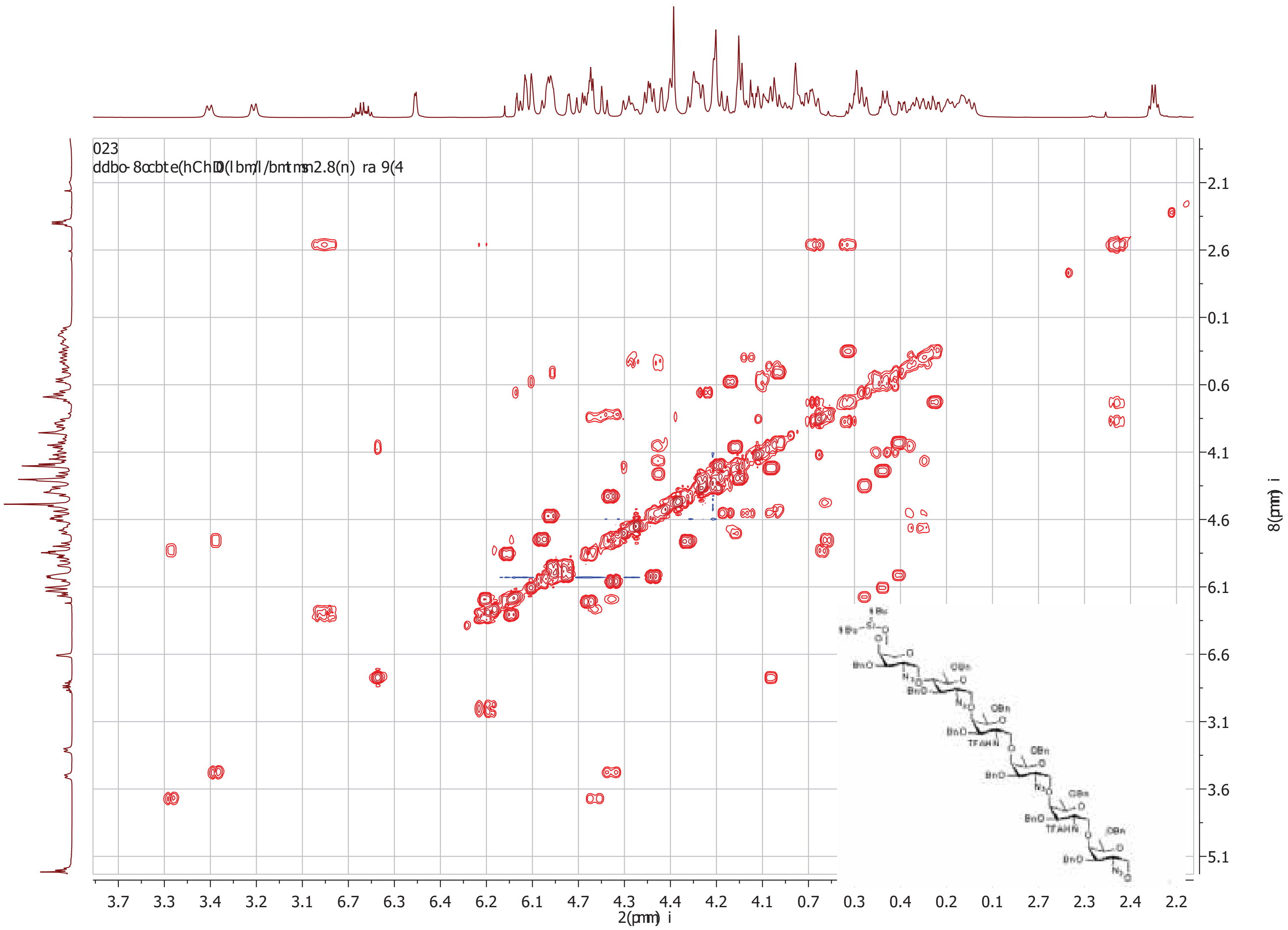
023
BnO
OBn
TFAHN

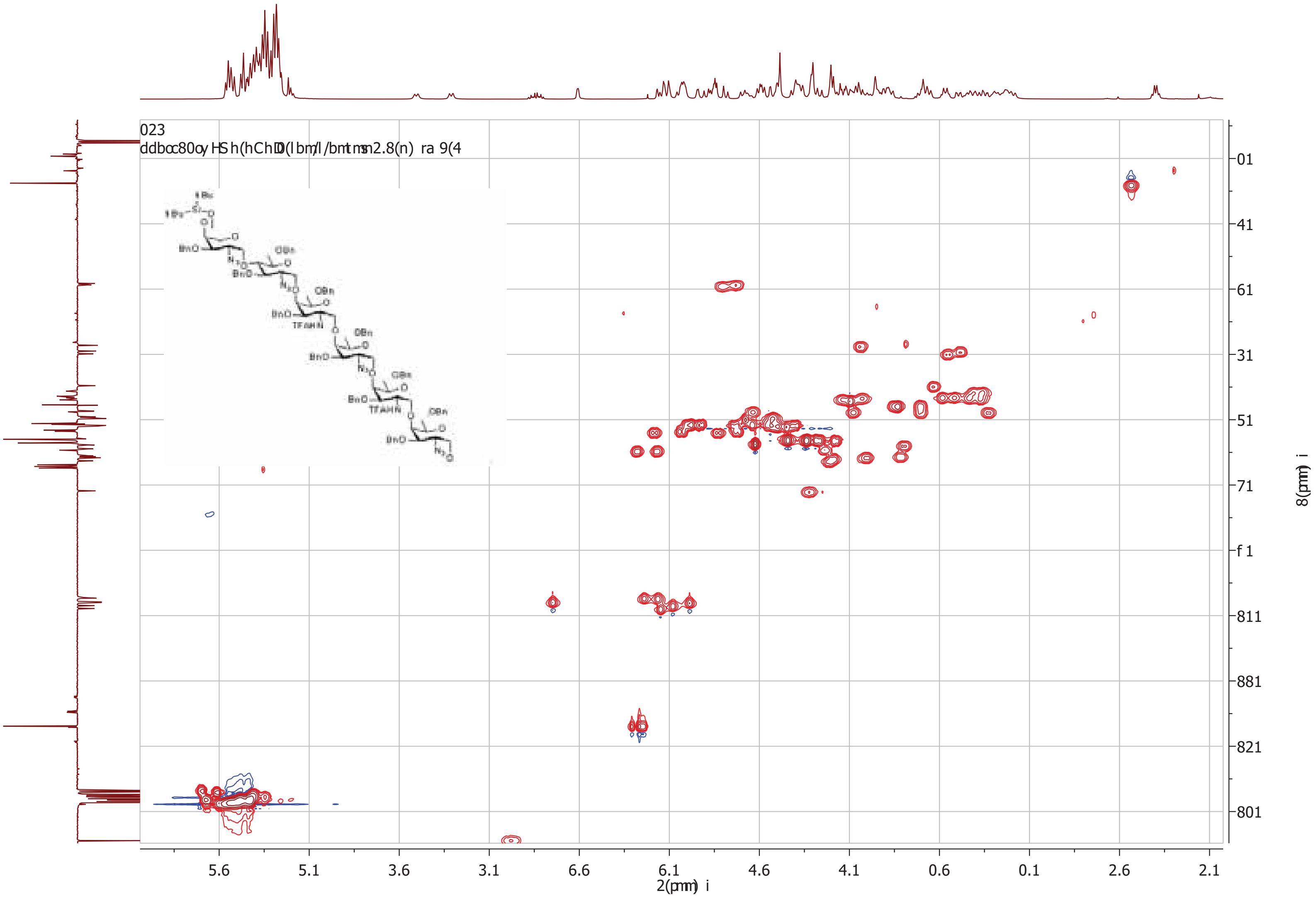
023
ddbcc80oy HS h(hChD0(lbm/l/bmtms2.8(n) ra 9(4
5.6
5.1
3.6
3.1
6.6
6.1
4.6
4.1
0.6
0.1
2.6
2.1
2(pmm) i
01
41
61
31
51
71
f1
811
881
821
801
8(pmm) i

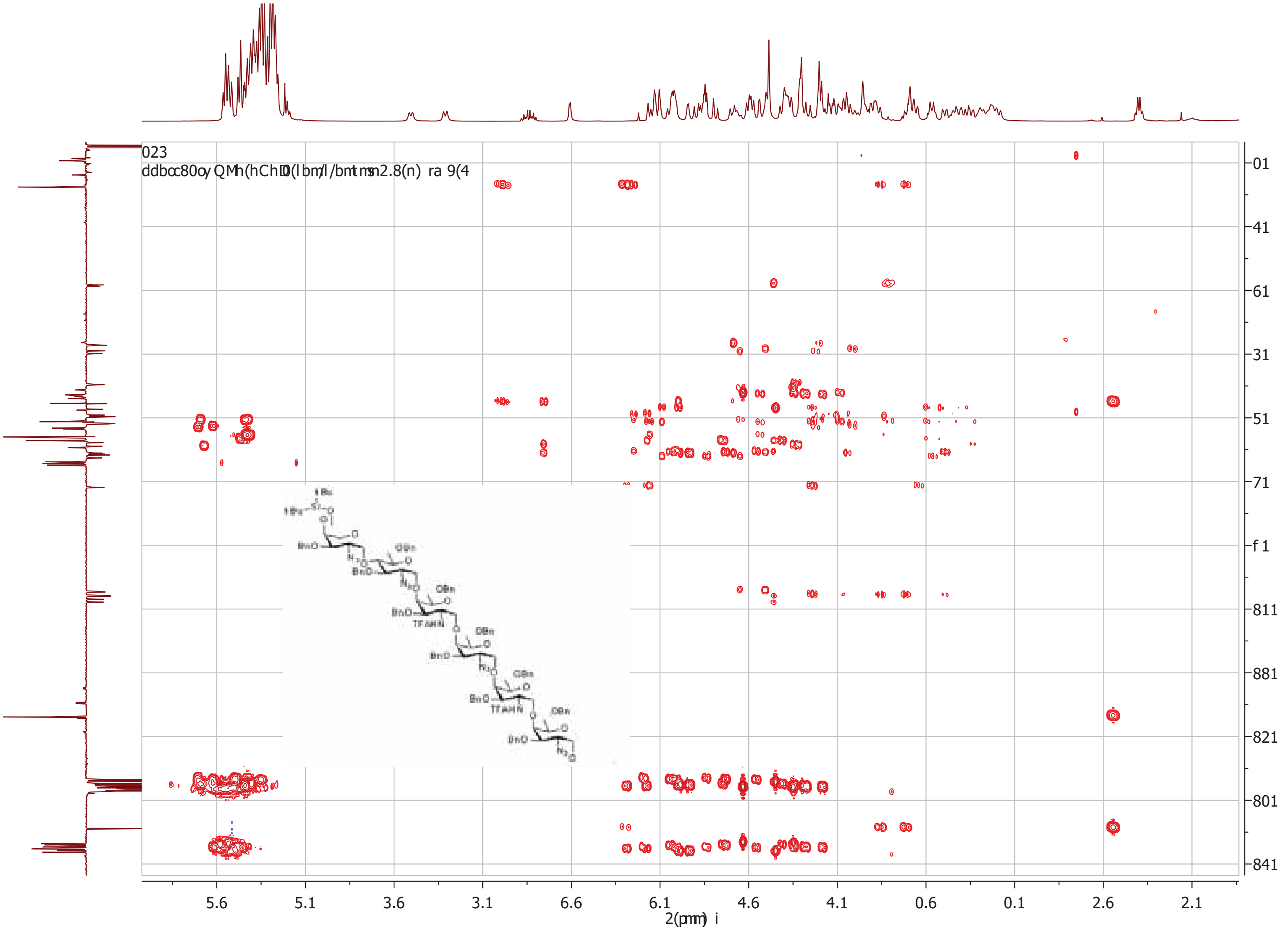
023
ddbcc80oy QMh(hChD(lbm/l/bmtms2.8(n) ra 9(4
2(pmm) i
8(pmm) i
tBu
BnO
OBn
TFAHN
N3

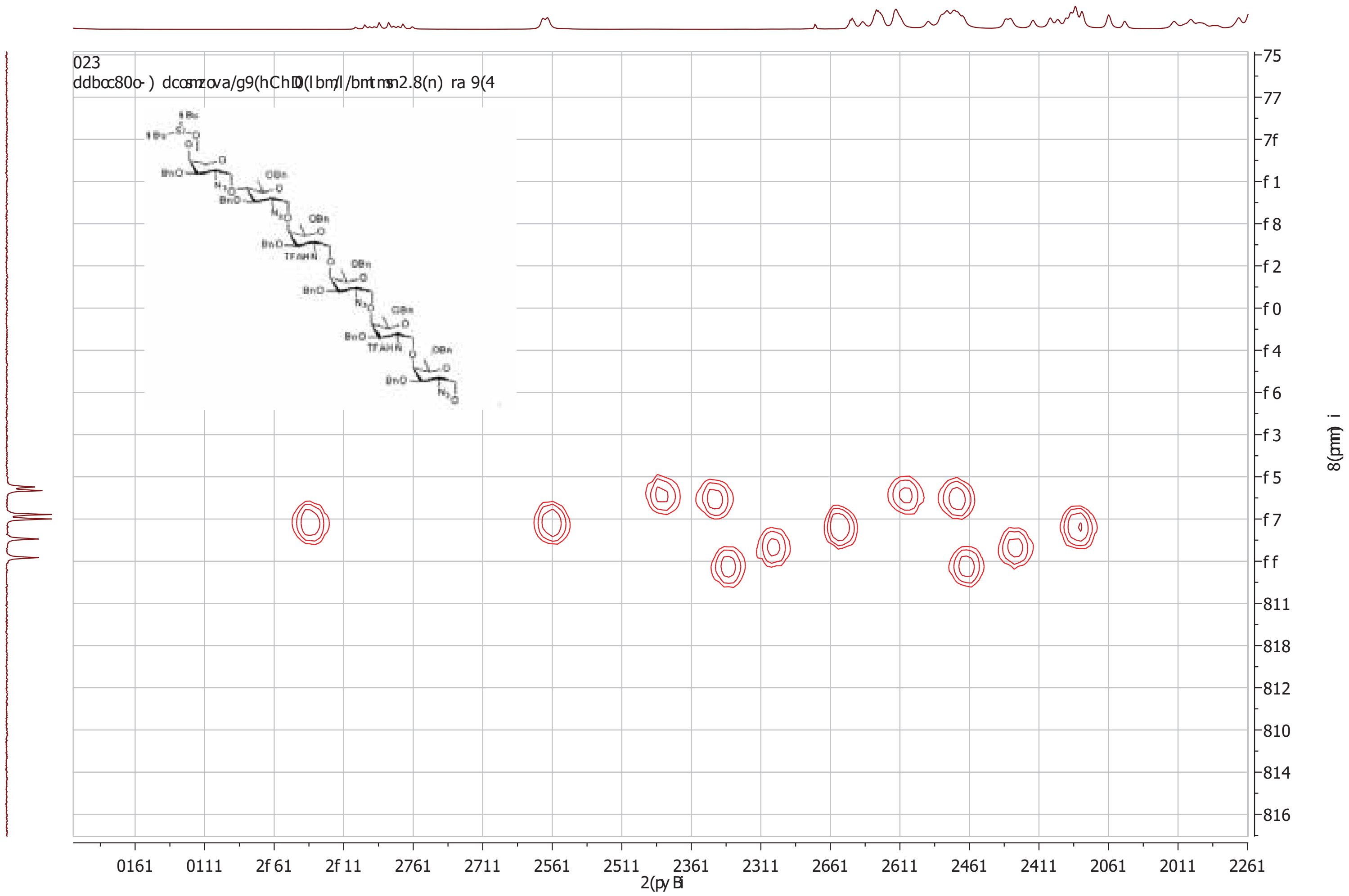
023
ddbcc80o-) dcosmzova/g9(hChD0(lbm/l/bmtms2.8(n) ra 9(4

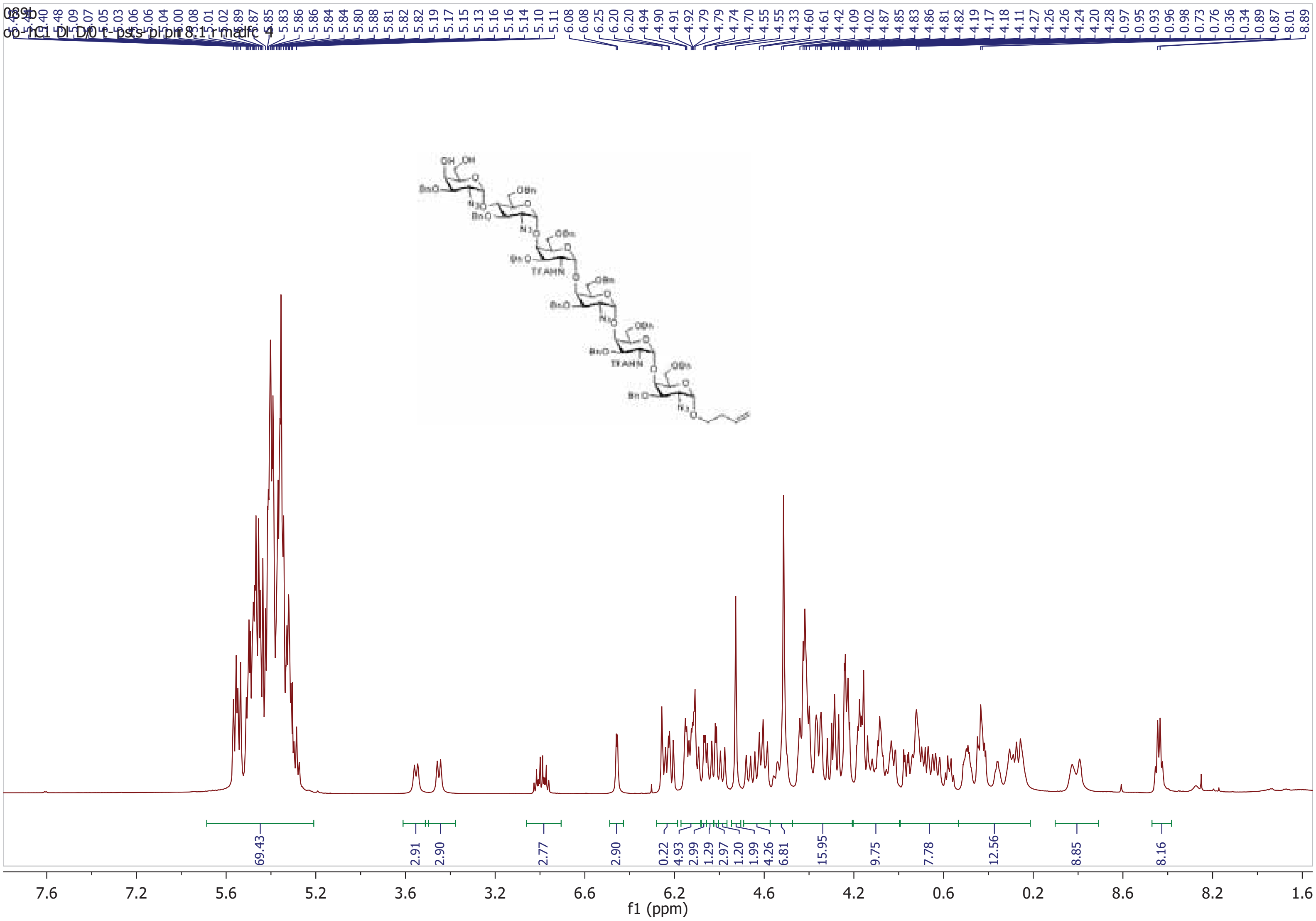

5.48 5.09 5.07 5.05 5.03 5.06 5.04 5.00 5.08 5.01 5.02 5.89 5.87 5.85 5.83 5.86 5.86 5.84 5.80 5.88 5.81 5.82 5.82 5.19 5.17 5.15 5.13 5.16 5.16 5.14 5.10 5.11 6.08 6.08 6.25 6.20 6.20 4.94 4.90 4.91 4.92 4.79 4.79 4.74 4.70 4.55 4.55 4.33 4.60 4.61 4.42 4.09 4.02 4.87 4.85 4.83 4.86 4.81 4.82 4.19 4.17 4.18 4.11 4.27 4.26 4.26 4.24 4.20 4.28 0.97 0.95 0.93 0.96 0.98 0.73 0.76 0.36 0.34 0.89 0.87 8.01 8.89
OH OH
BnO
N3
OBn
BnO
TFAHN
N3
OBn
BnO
TFAHN
N3
69.43
2.91
2.90
2.77
2.90
0.22
4.93
2.99
1.29
2.97
1.20
1.99
4.26
6.81
15.95
9.75
7.78
12.56
8.85
8.16
7.6 7.2 5.6 5.2 3.6 3.2 6.6 6.2 4.6 4.2 0.6 0.2 8.6 8.2 1.6
f1 (ppm)

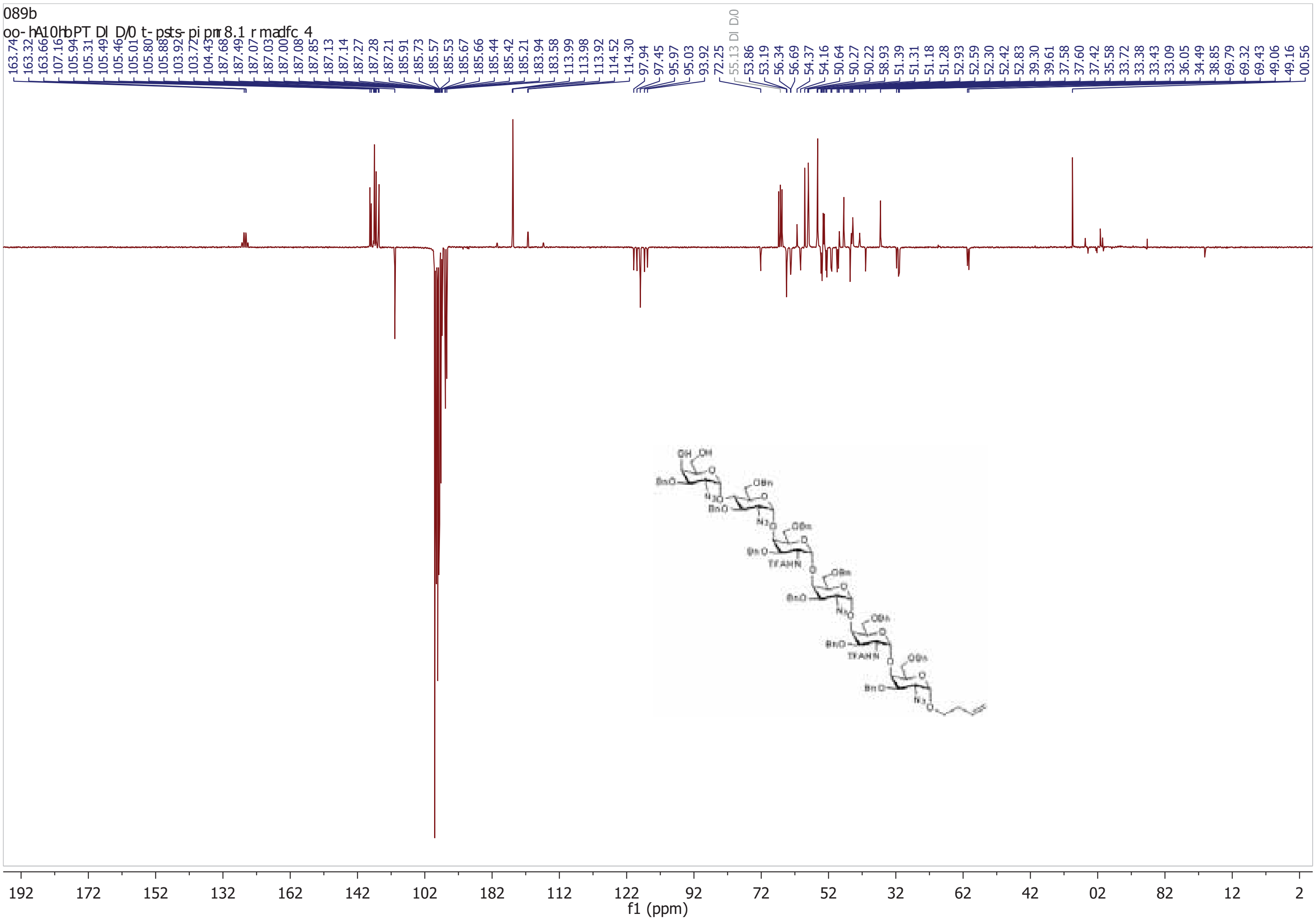
089b
oo-hA10hbPT Dl D/0 t-psts-pi pm 8.1 r madfc 4
f1 (ppm)

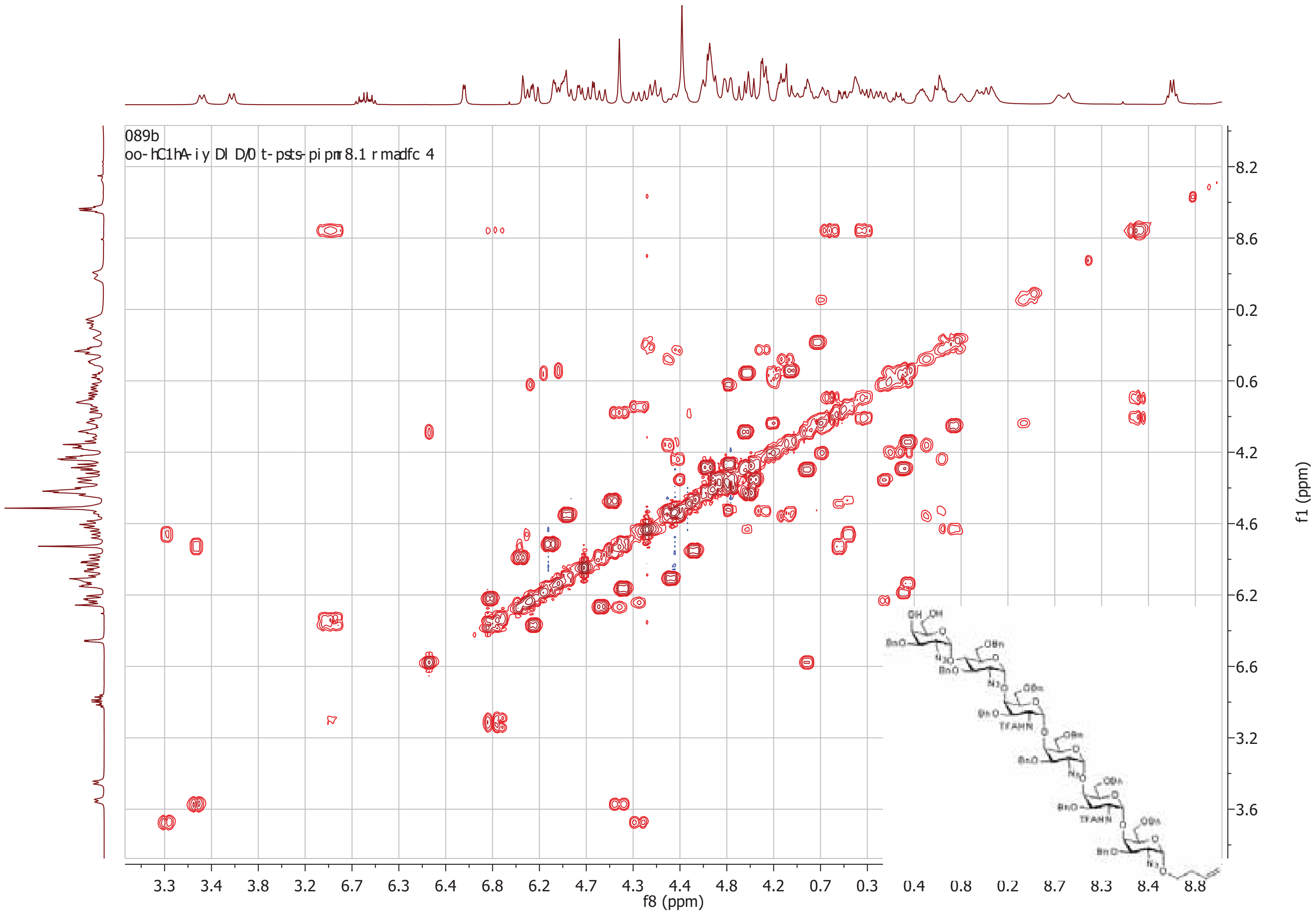
089b
oo-hC1hA-i y Dl D/0 t-psts-pi pmr8.1 r madfc 4
f8 (ppm)
f1 (ppm)

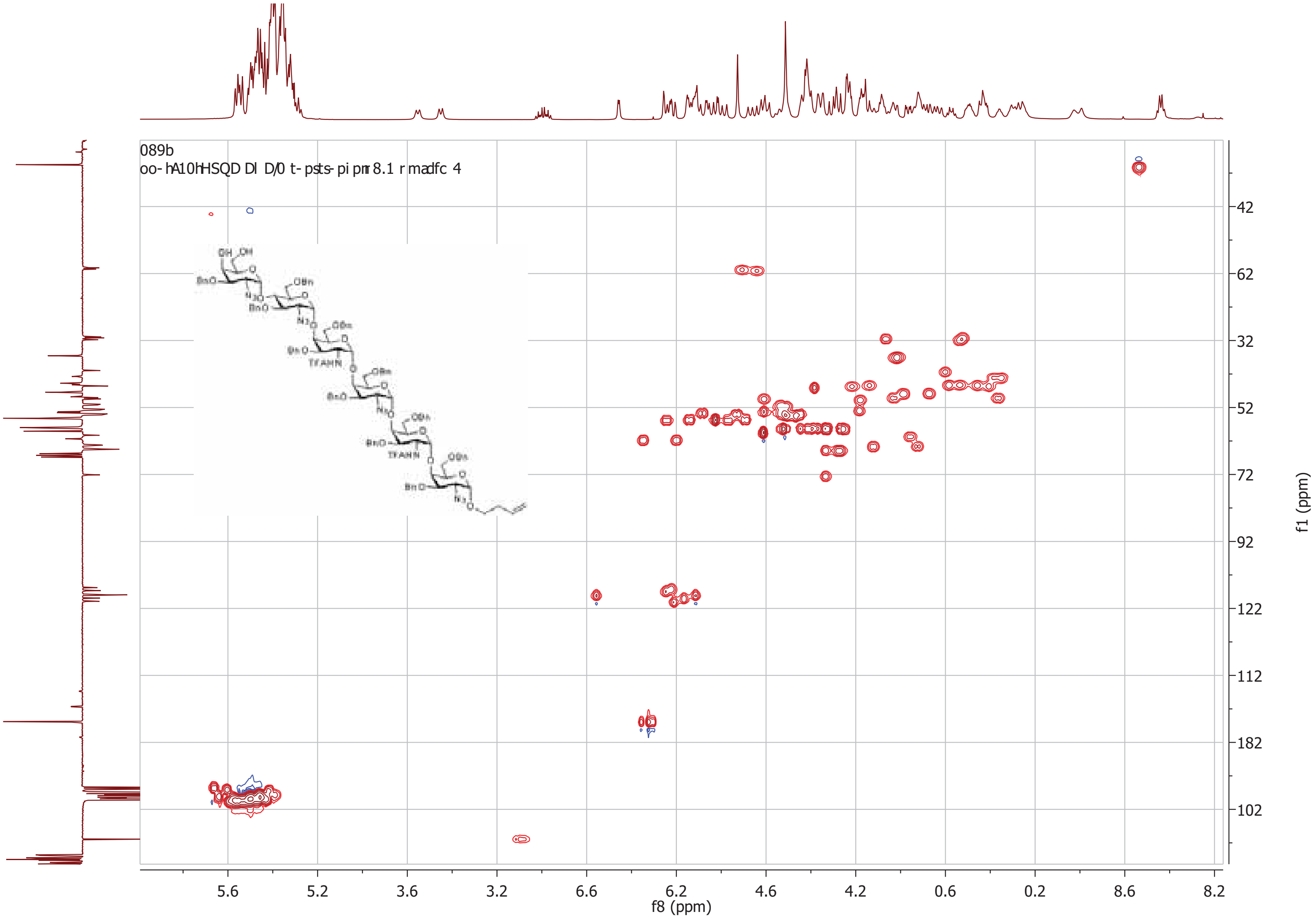
089b
oo- hA10hHSQD Dl D/0 t- psts- pi pmr 8.1 r madfc 4
f8 (ppm)
f1 (ppm)
5.6
5.2
3.6
3.2
6.6
6.2
4.6
4.2
0.6
0.2
8.6
8.2
42
62
32
52
72
92
122
112
182
102
BnO
N3
TFAHN
OBn
OH

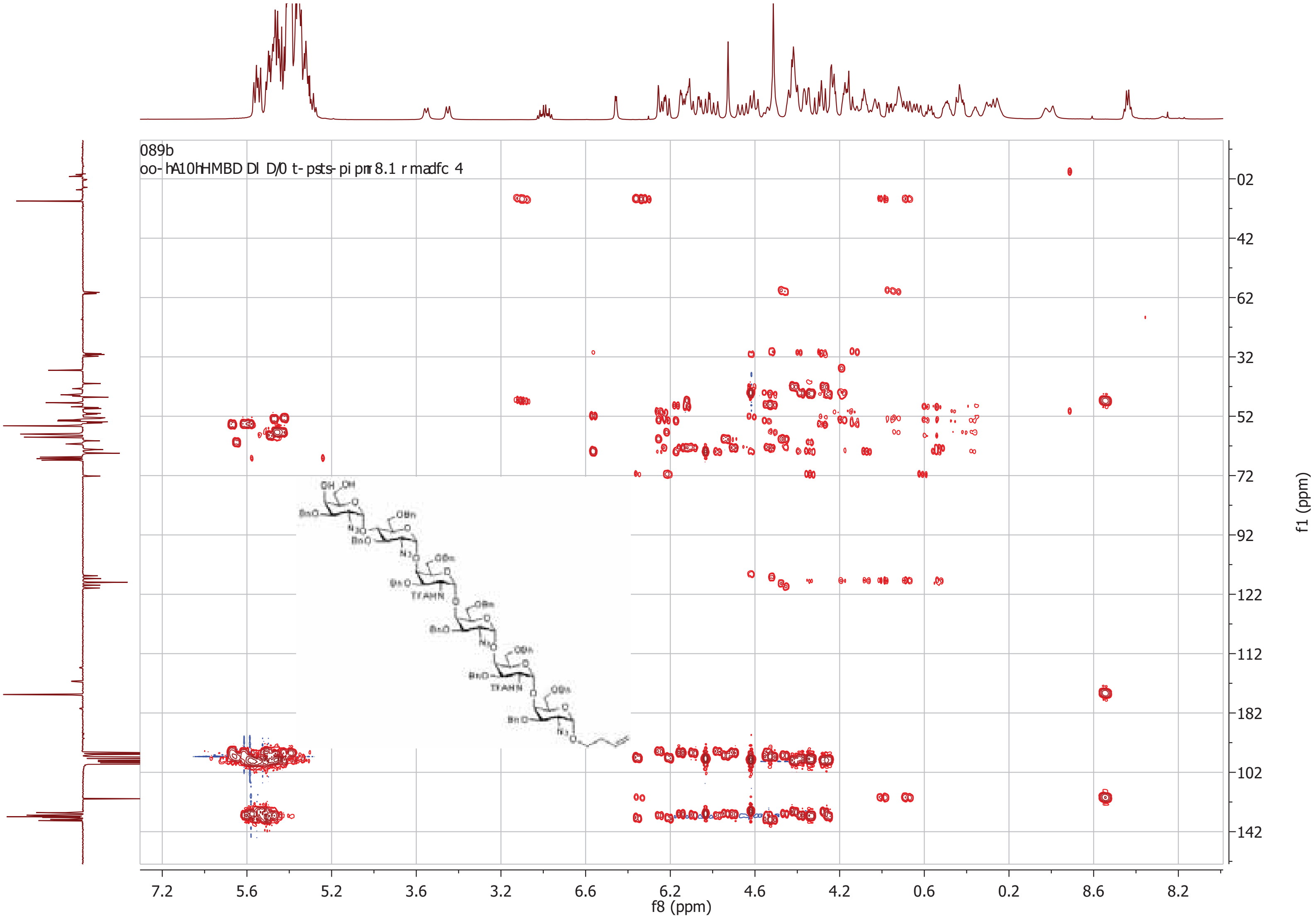
089b
oo-hA10hHMBD DI D/0 t-psts-pi pmr8.1 rmadfc 4
f8 (ppm)
f1 (ppm)

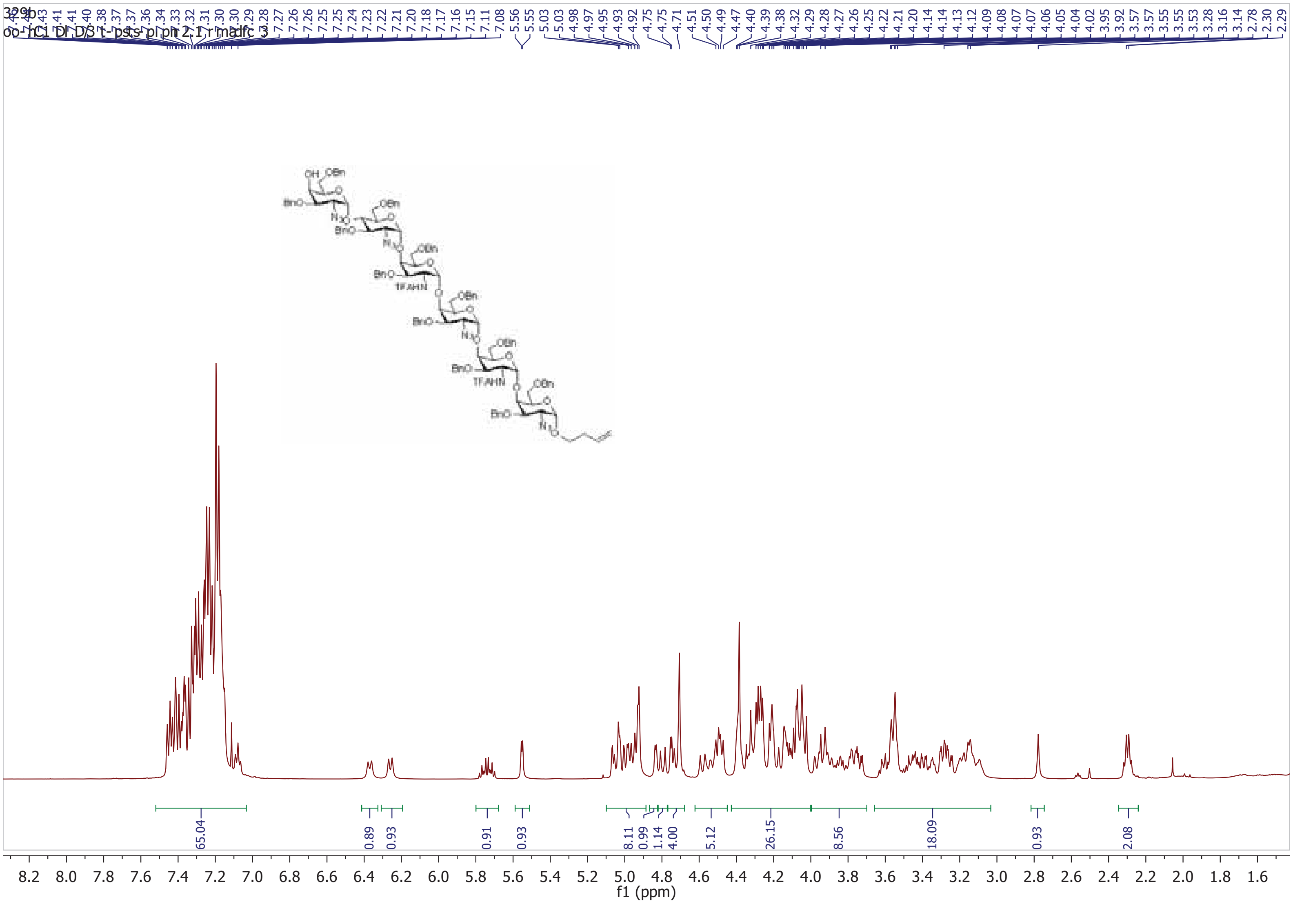
329b
OH
OBn
BnO
TFAHN
65.04
0.89
0.93
0.91
0.93
8.11
0.99
1.14
4.00
5.12
26.15
8.56
18.09
0.93
2.08
8.2 8.0 7.8 7.6 7.4 7.2 7.0 6.8 6.6 6.4 6.2 6.0 5.8 5.6 5.4 5.2 5.0 4.8 4.6 4.4 4.2 4.0 3.8 3.6 3.4 3.2 3.0 2.8 2.6 2.4 2.2 2.0 1.8 1.6
f1 (ppm)

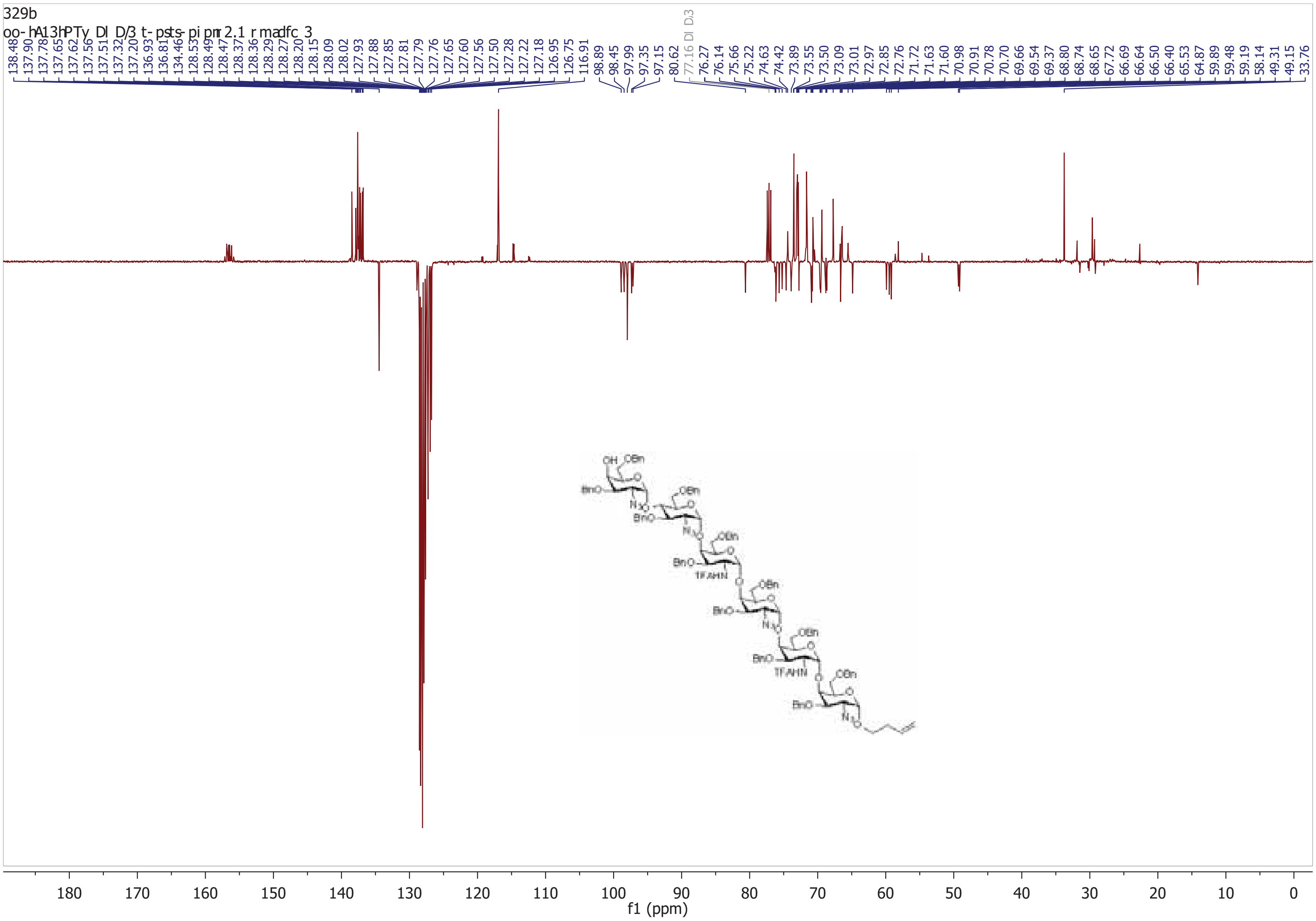
329b
138.48
137.90
137.78
137.65
137.62
137.56
137.51
137.32
137.20
136.93
136.81
134.46
128.53
128.49
128.47
128.37
128.36
128.29
128.27
128.20
128.15
128.09
128.02
127.93
127.88
127.85
127.81
127.79
127.76
127.65
127.60
127.56
127.50
127.28
127.22
127.18
126.95
126.75
116.91
98.89
98.45
97.99
97.35
97.15
80.62
77.16
76.27
76.14
75.66
75.22
74.63
74.42
73.89
73.55
73.50
73.09
73.01
72.97
72.85
72.76
71.72
71.63
71.60
70.98
70.91
70.78
70.70
69.66
69.54
69.37
68.80
68.74
68.65
67.72
66.69
66.64
66.50
66.40
65.53
64.87
59.89
59.48
59.19
58.14
49.31
49.15
33.76
180
170
160
150
140
130
120
110
100
90
80
70
60
50
40
30
20
10
0
f1 (ppm)
OH
OBn
BnO
TFAHN

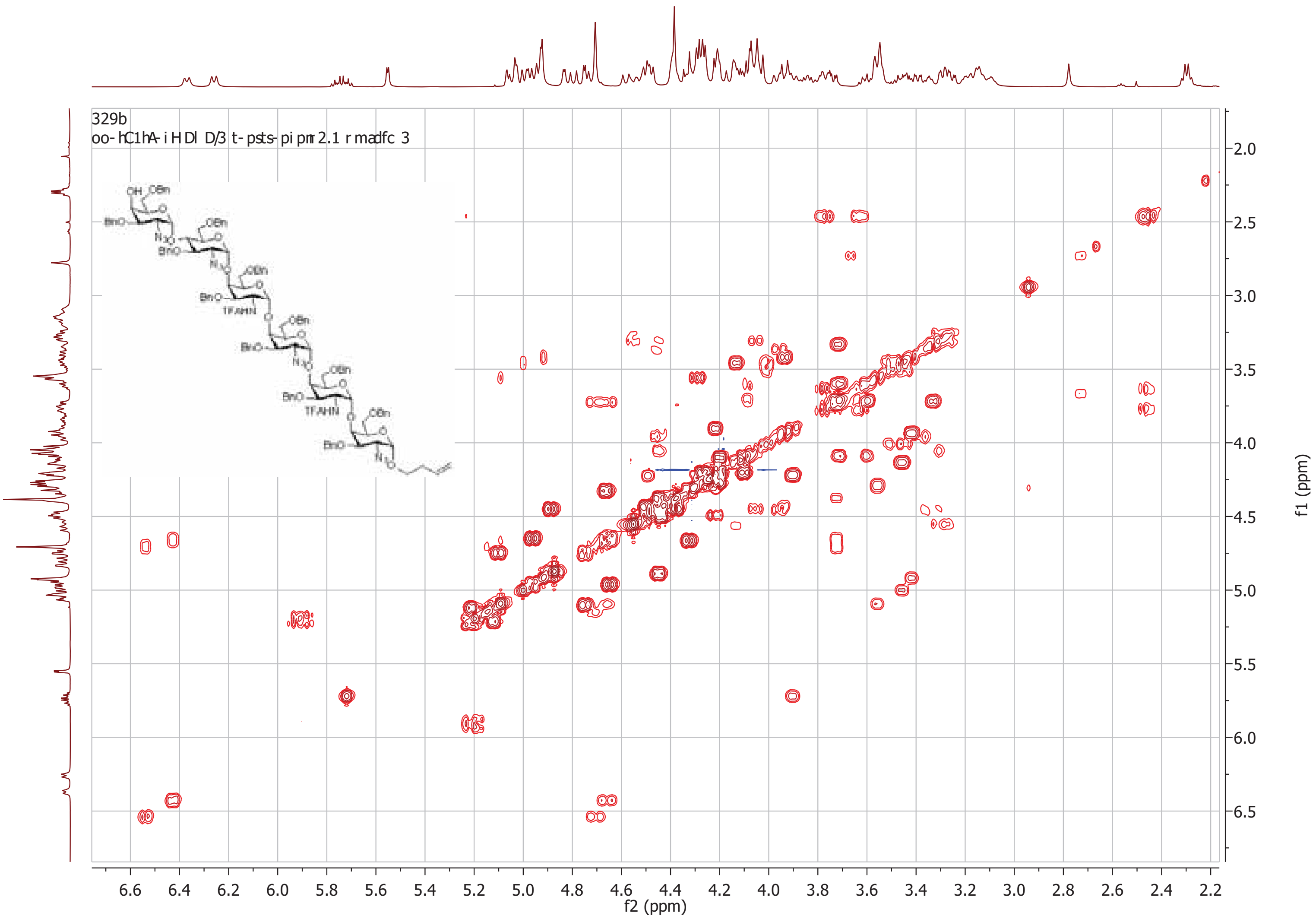
329b
f2 (ppm)
f1 (ppm)

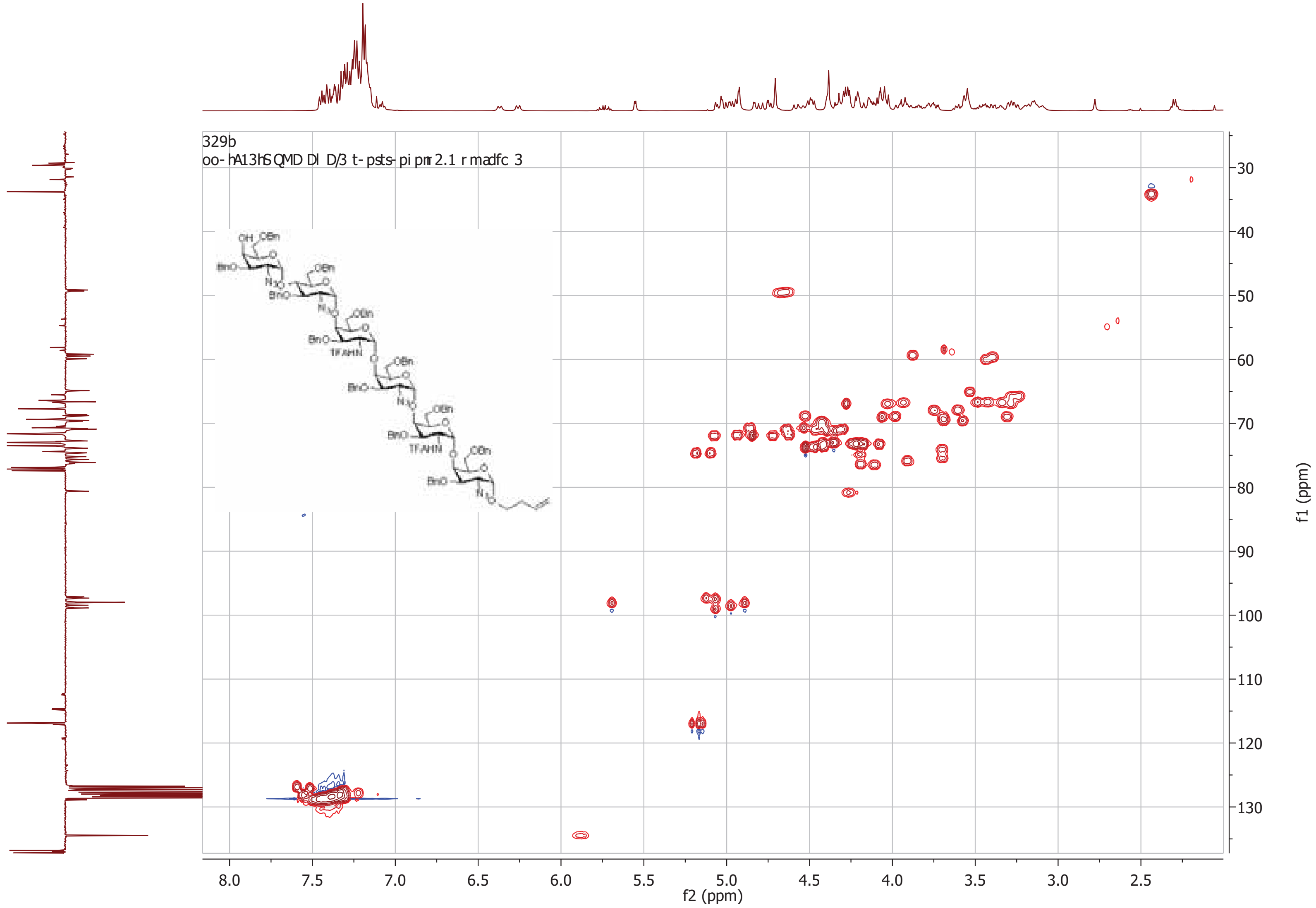
329b
oo- hA13hSQMD DI D/3 t- psts- pi pmr 2.1 r madfc 3
f2 (ppm)
f1 (ppm)

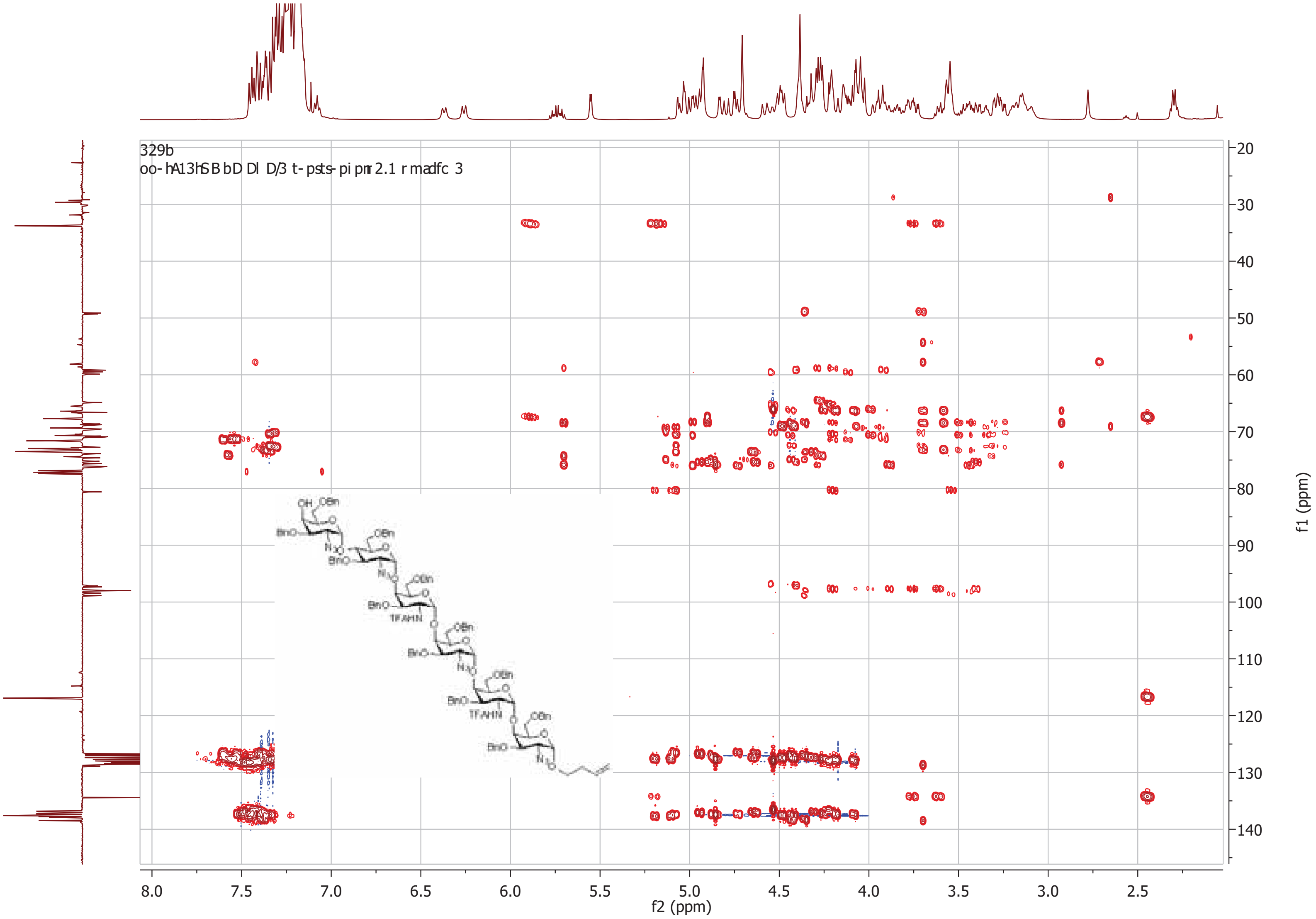

329b
oo- hA13hSB bD DI D/3 t- psts- pi pmr 2.1 r madfc 3
f2 (ppm)
f1 (ppm)
OH
OBn
BnO
N3
TFAHN

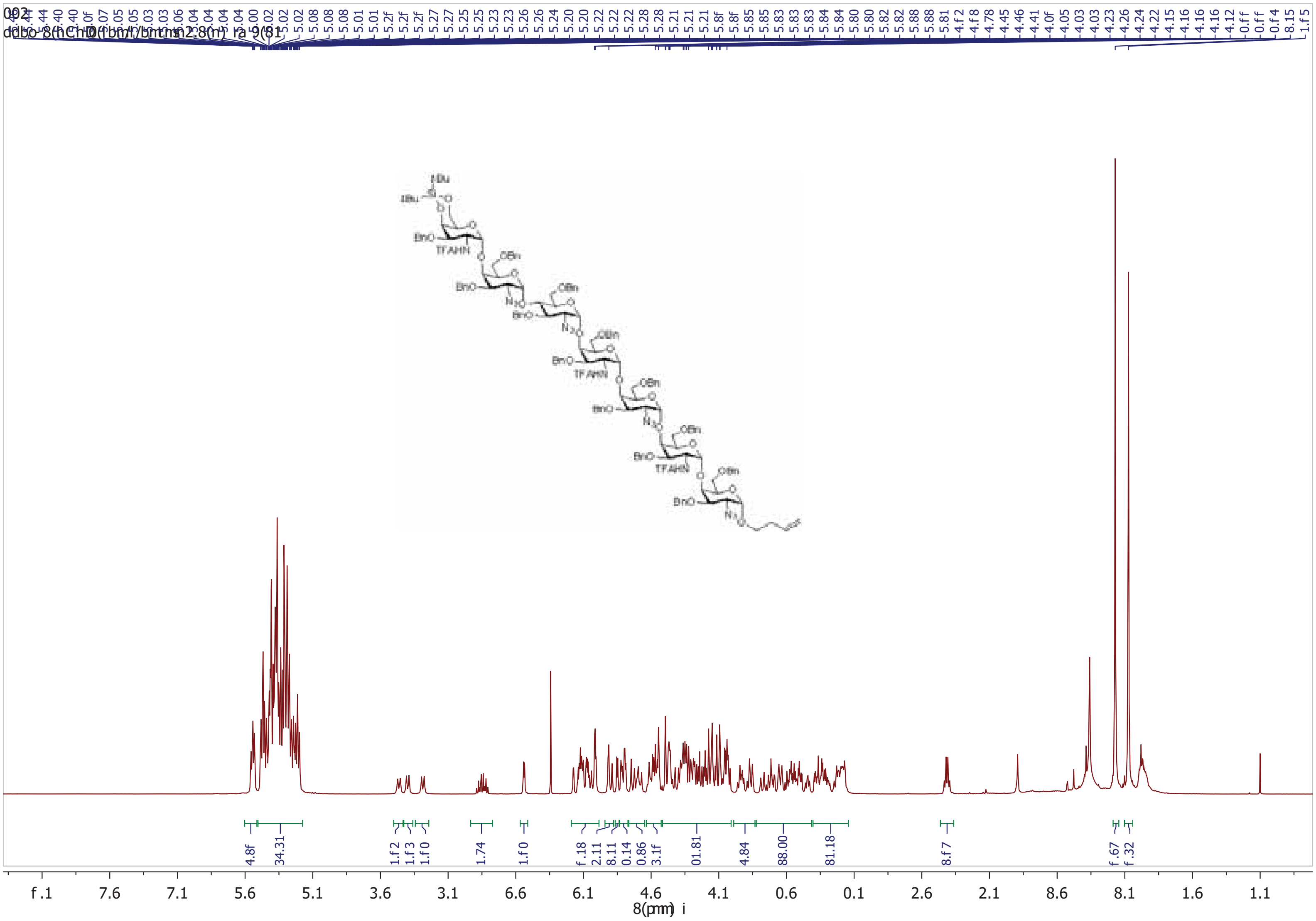

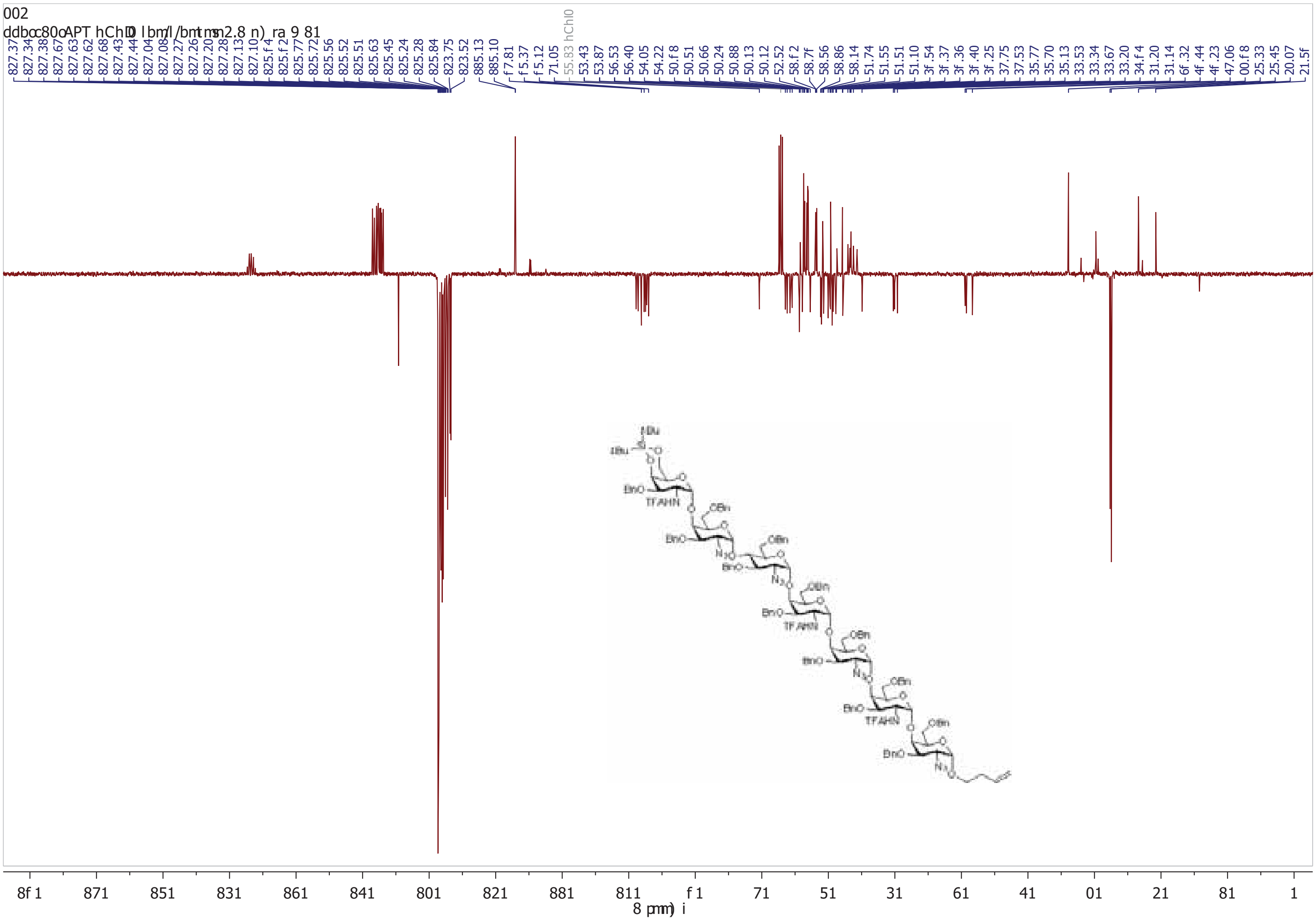
002
ddbc c80c APT hChl0 lbm/l/bmtms 2.8 n) ra 9 81
827.37 827.34 827.38 827.67 827.63 827.62 827.68 827.43 827.44 827.04 827.08 827.27 827.26 827.20 827.28 827.13 827.10 825.f4 825.f2 825.77 825.72 825.56 825.52 825.51 825.63 825.45 825.24 825.28 825.84 823.75 823.52 885.13 885.10 f7.81 f5.37 f5.12 71.05 55.83 hChl0 53.43 53.87 56.53 56.40 54.05 54.22 50.f8 50.51 50.66 50.24 50.88 50.13 50.12 52.52 58.f2 58.7f 58.56 58.86 58.14 51.74 51.55 51.51 51.10 3f.54 3f.37 3f.36 3f.40 3f.25 37.75 37.53 35.77 35.70 35.13 33.53 33.34 33.67 33.20 34.f4 31.20 31.14 6f.32 4f.44 4f.23 47.06 00.f8 25.33 25.45 20.07 21.5f
8f1 871 851 831 861 841 801 821 881 811 f1 71 51 31 61 41 01 21 81 1
8 pmm) i
tBu
tBu
BnO
TFAHN
OBn
BnO
N3
OBn
BnO
N3
OBn
BnO
TFAHN
OBn
BnO
N3
OBn
BnO
TFAHN
OBn
BnO
N3

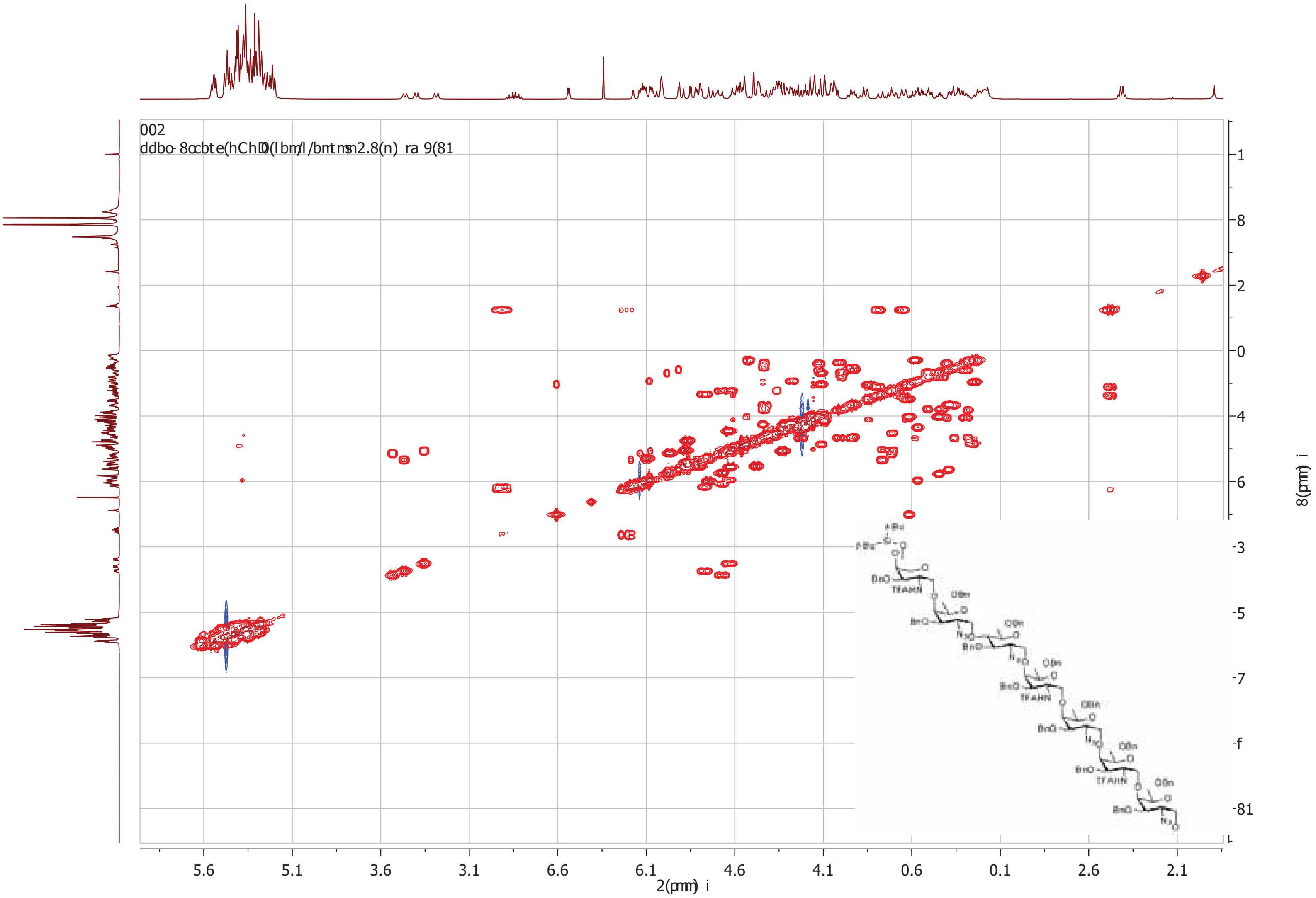
002
ddbo- 8ccbte(hChD (l bm/l /bmt ms n2.8(n) ra 9(81
2(pm) i
8(pm) i

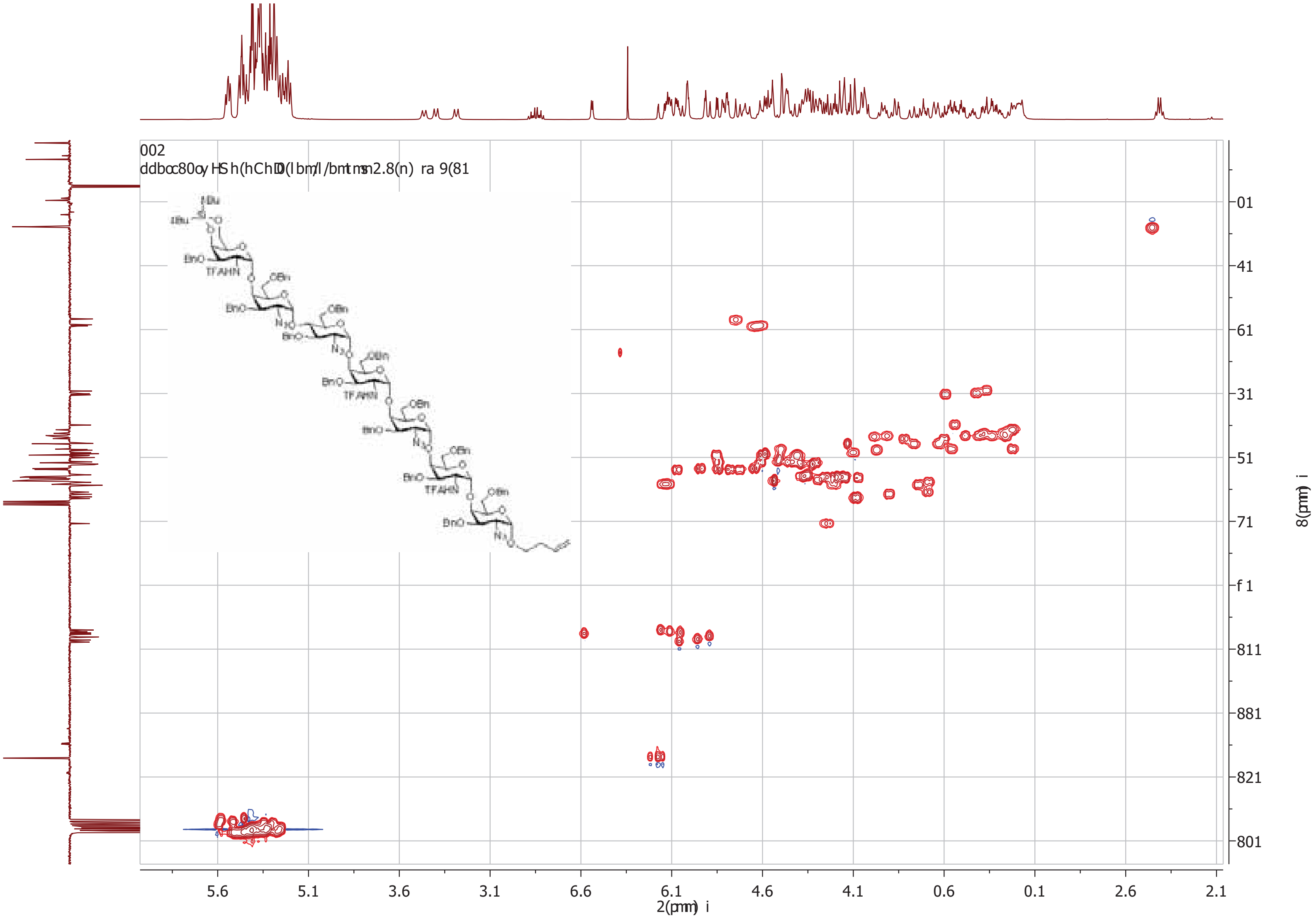

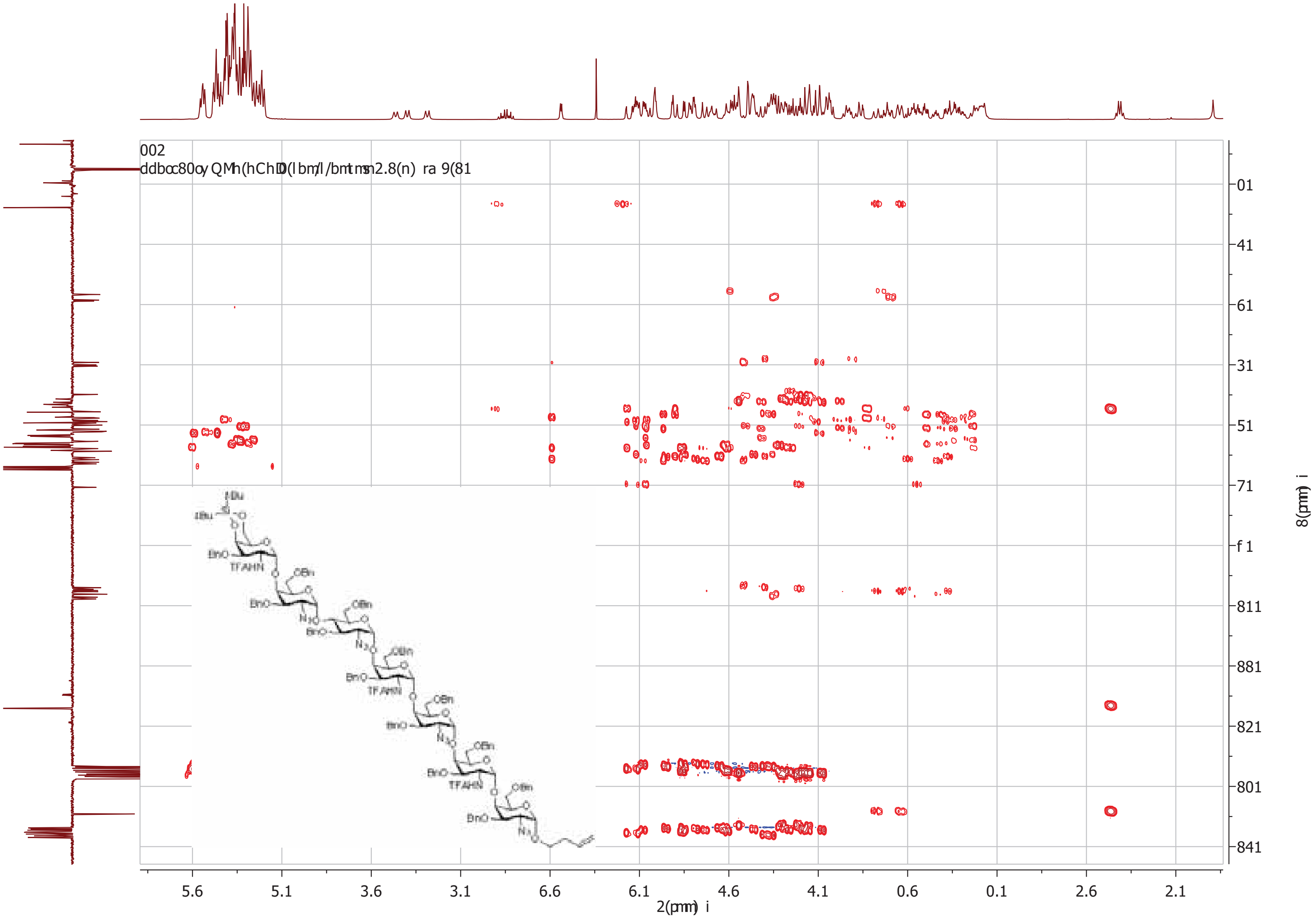

tBu
tBu
BnO
TFAHN
OBn
N3

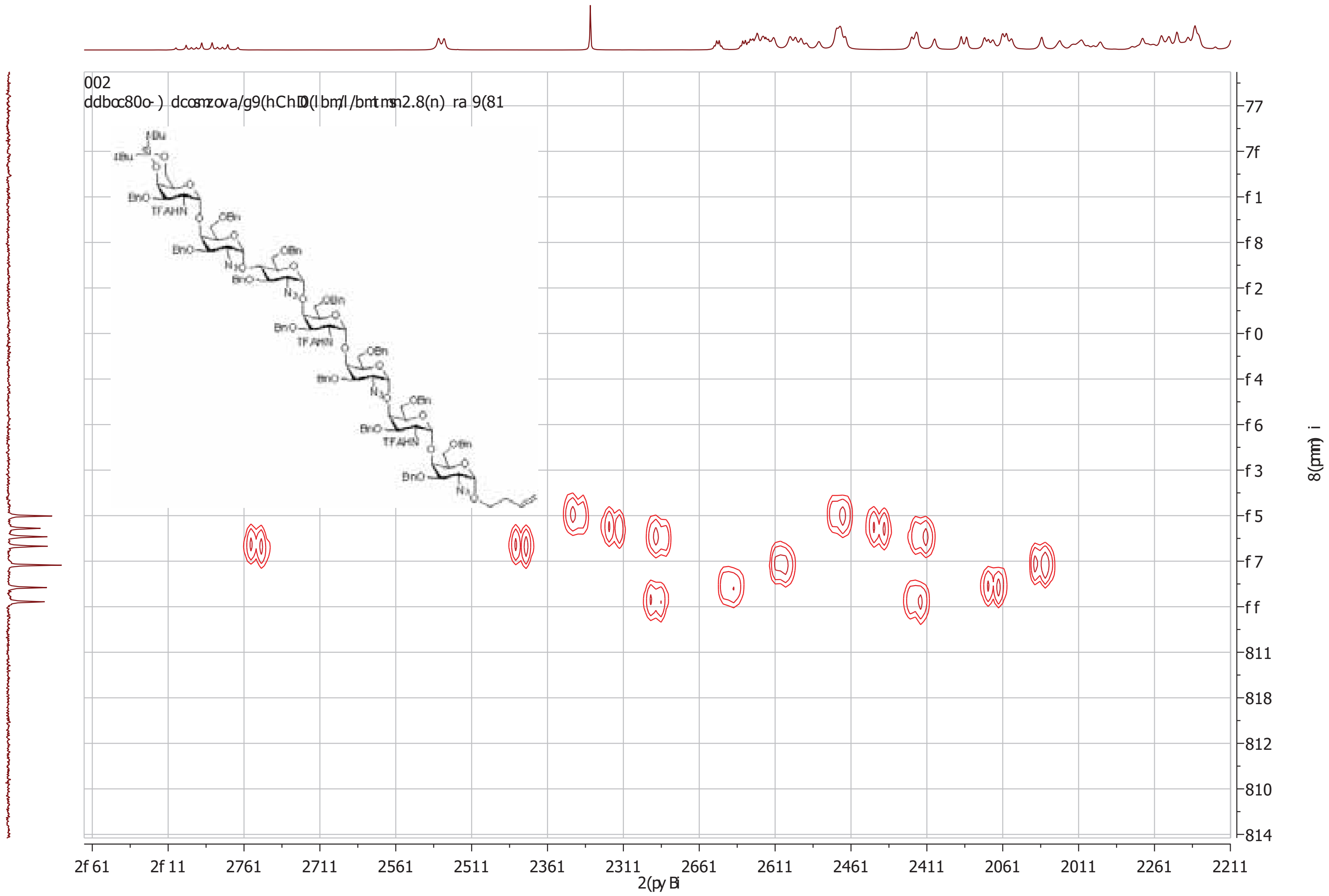
tBu
BnO
TFAHN
OBn
N3

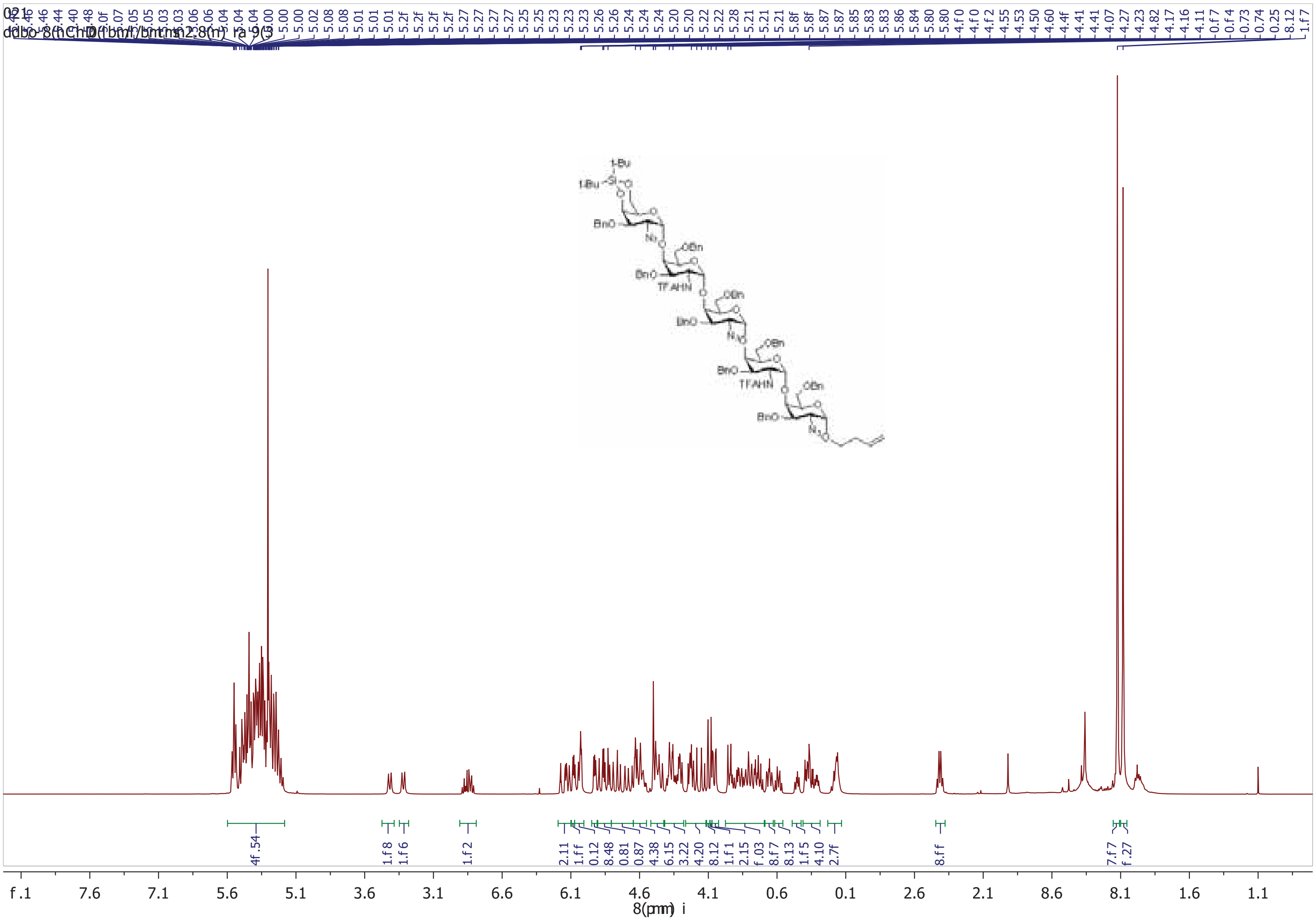

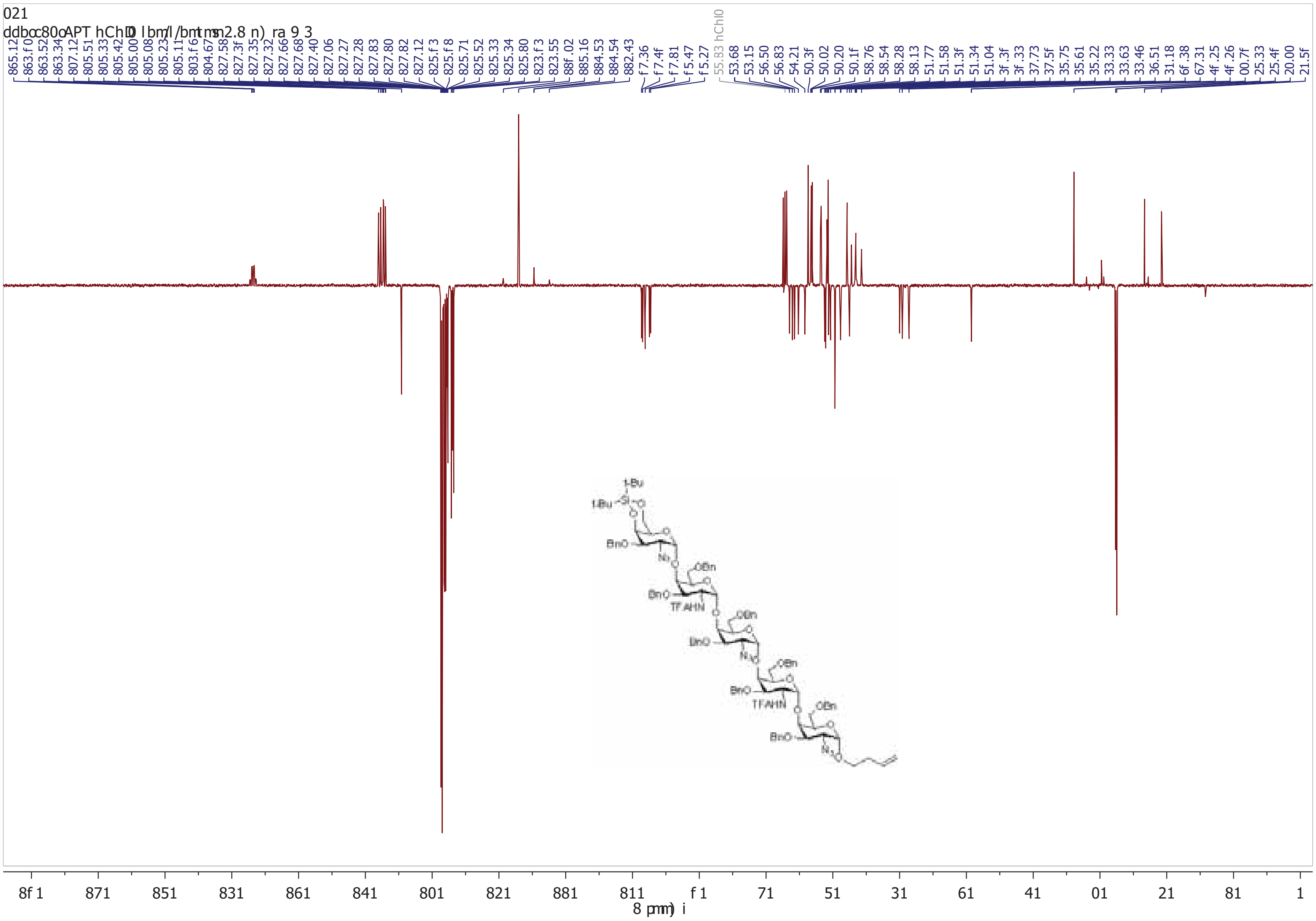
021
ddbc c80c APT hChlD lbm/l/bmtms2.8 n) ra 9 3
t-Bu
t-Bu
Si
BnO
N3
OBn
BnO
TFAHN
OBn
BnO
N3
OBn
BnO
TFAHN
OBn
BnO
N3
8 pmm) i

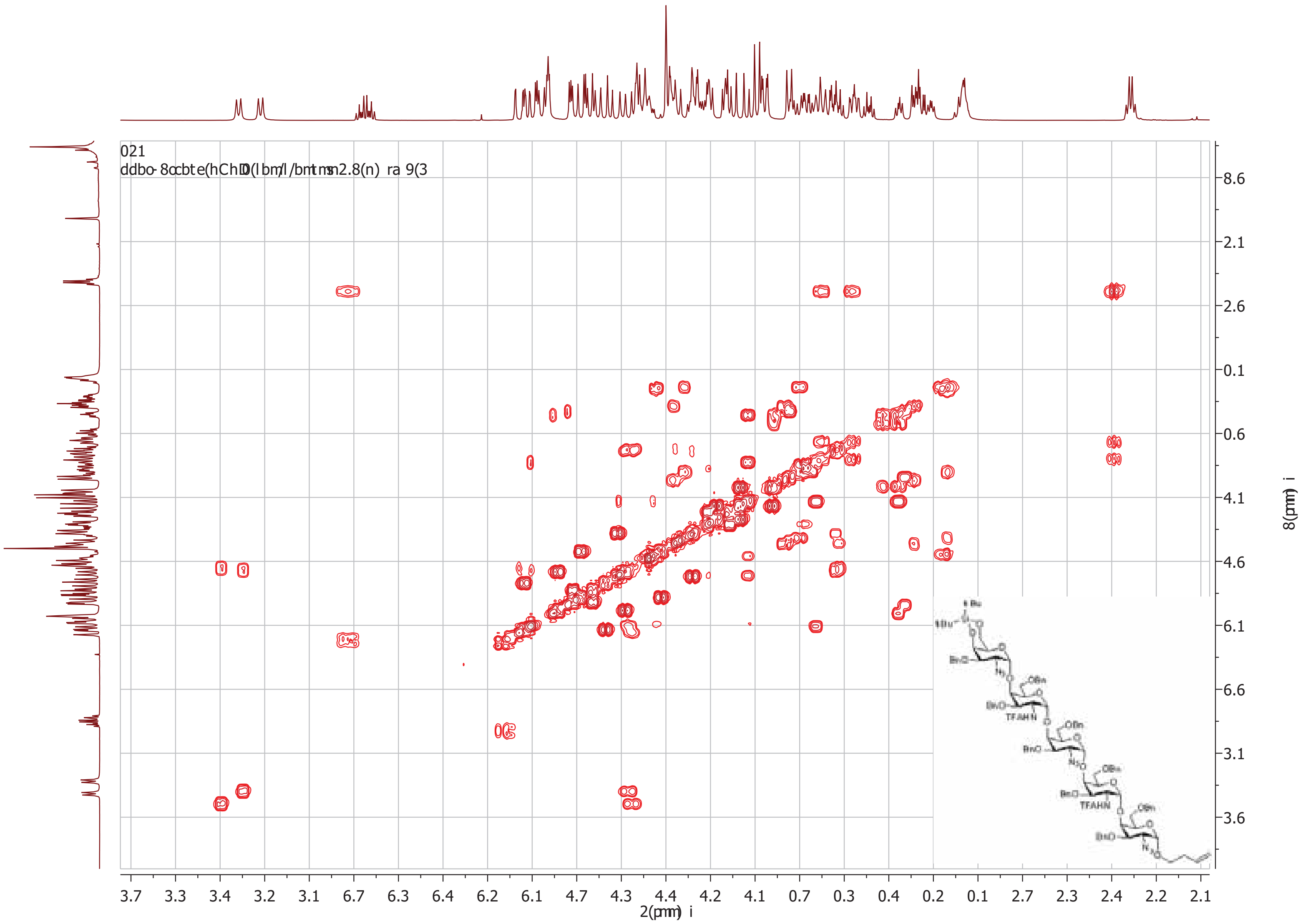

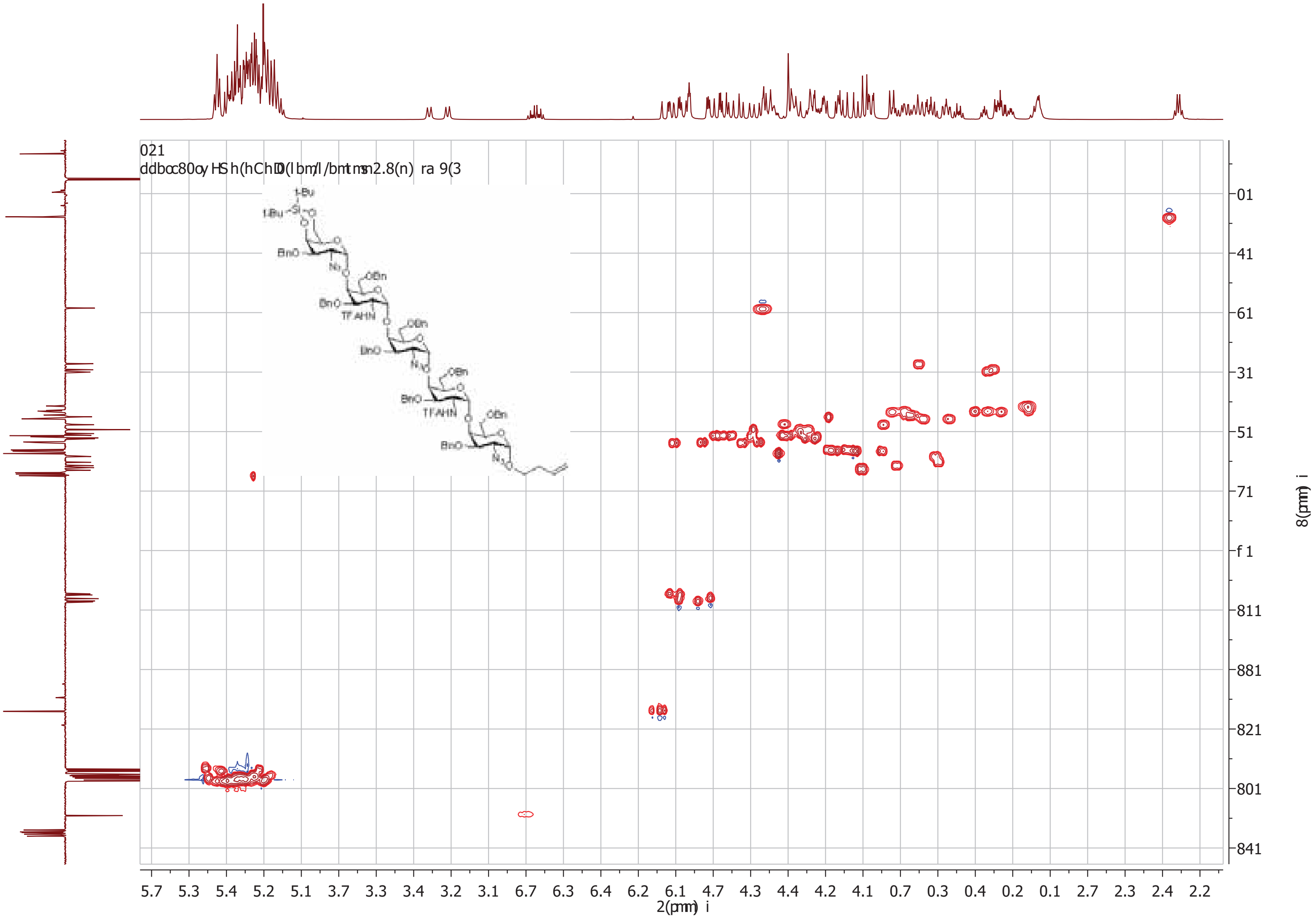

021
ddbcc80oy HS h(hChD(lbm/l/bmtms2.8(n) ra 9(3
t-Bu
t-Bu
Si
BnO
N3
OBn
TFAHN
2(pmn) i
8(pmn) i

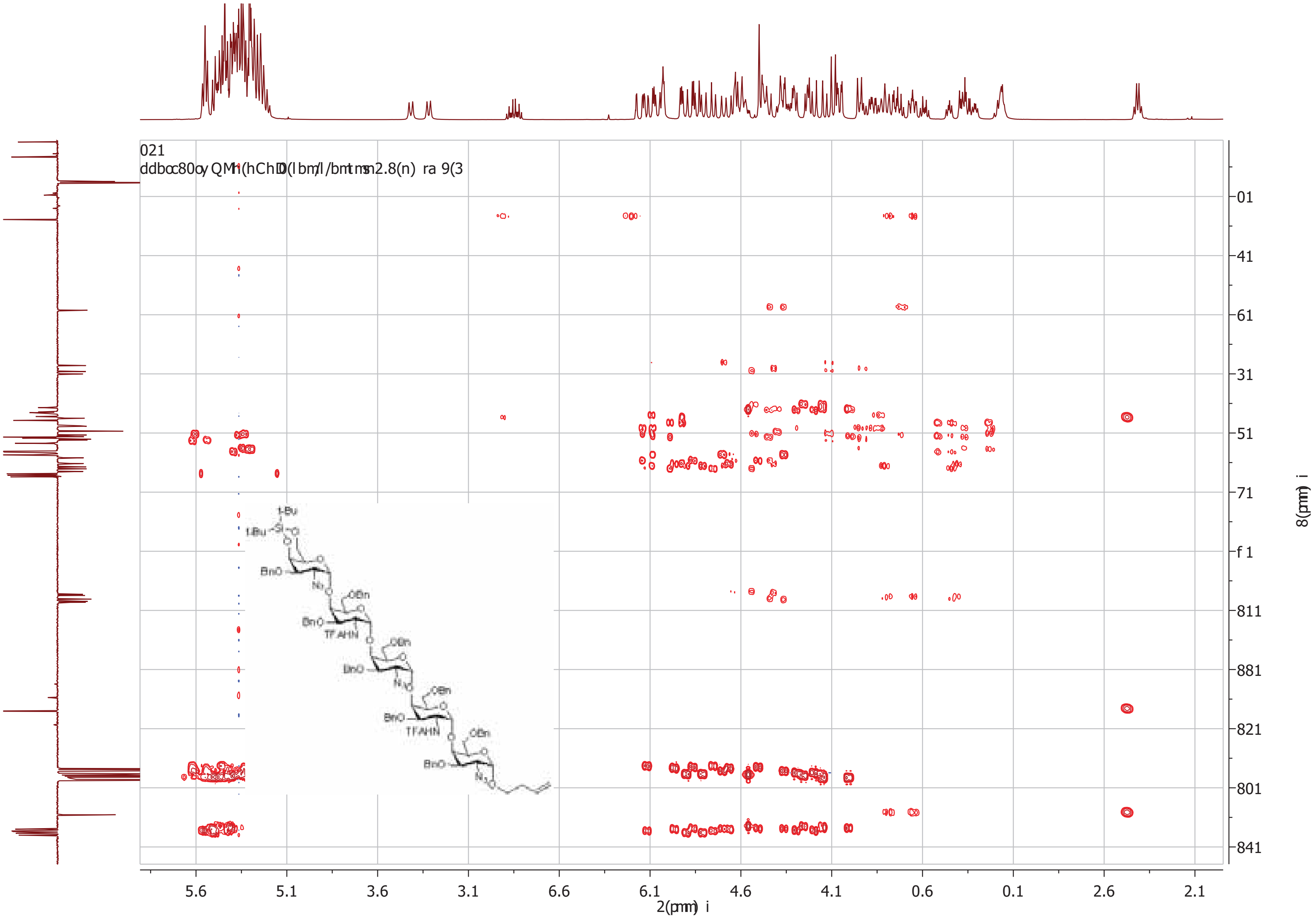
t-Bu
t-Bu
Si
BnO
N3
OBn
TFAHN

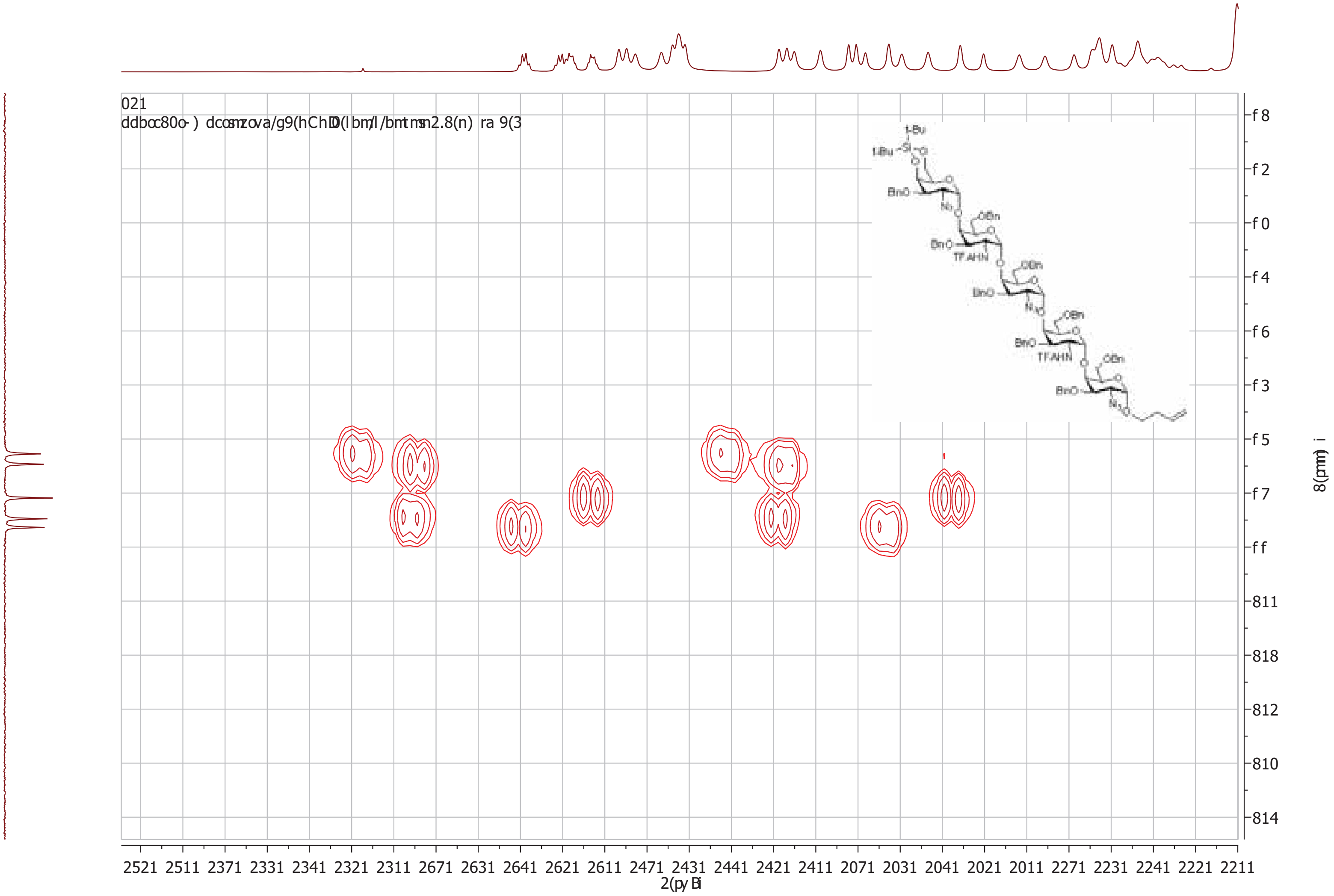

021
ddbcc80o-) dcosmzova/g9(hChD0(lbm/l/bmtms n2.8(n) ra 9(3
t-Bu
t-Bu
Si
BnO
N3
OBn
BnO
TFAHN
OBn
BnO
N3
OBn
BnO
TFAHN
OBn
BnO
N3
8(pm) i
2(py B

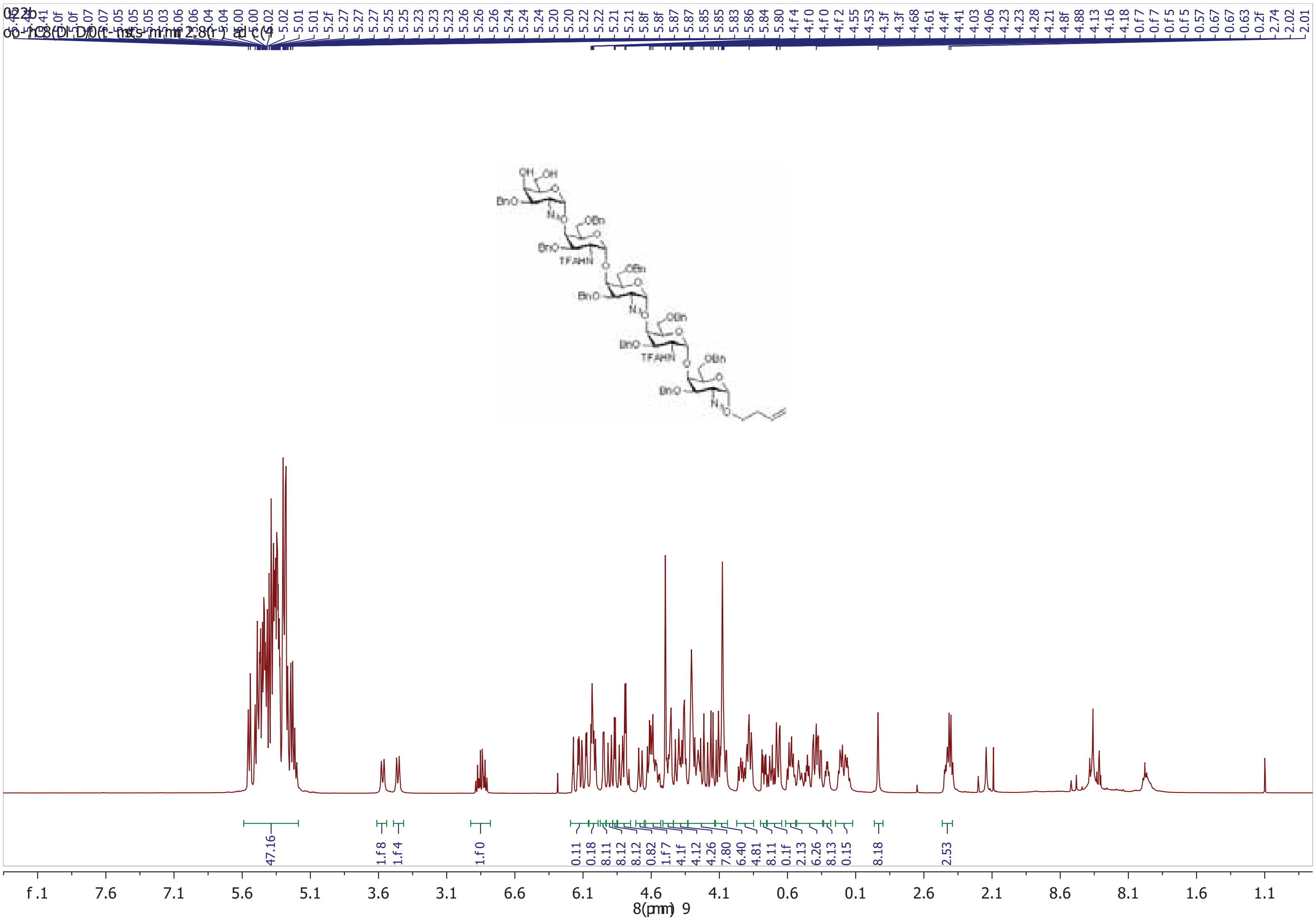

BnO
TFAHN
OBn
47.16
8.18
2.53

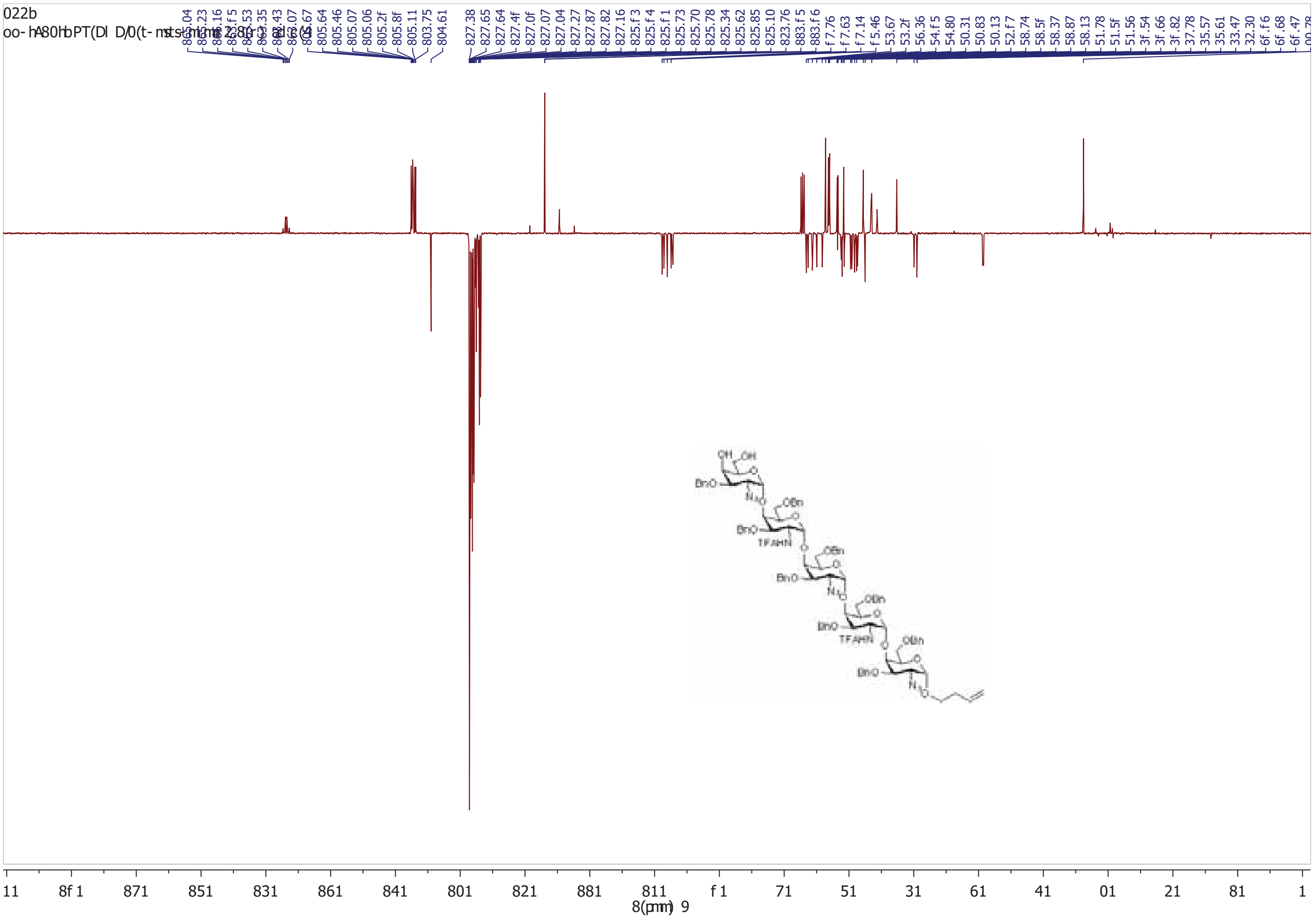
022b
OH
OBn
BnO
TFAHN

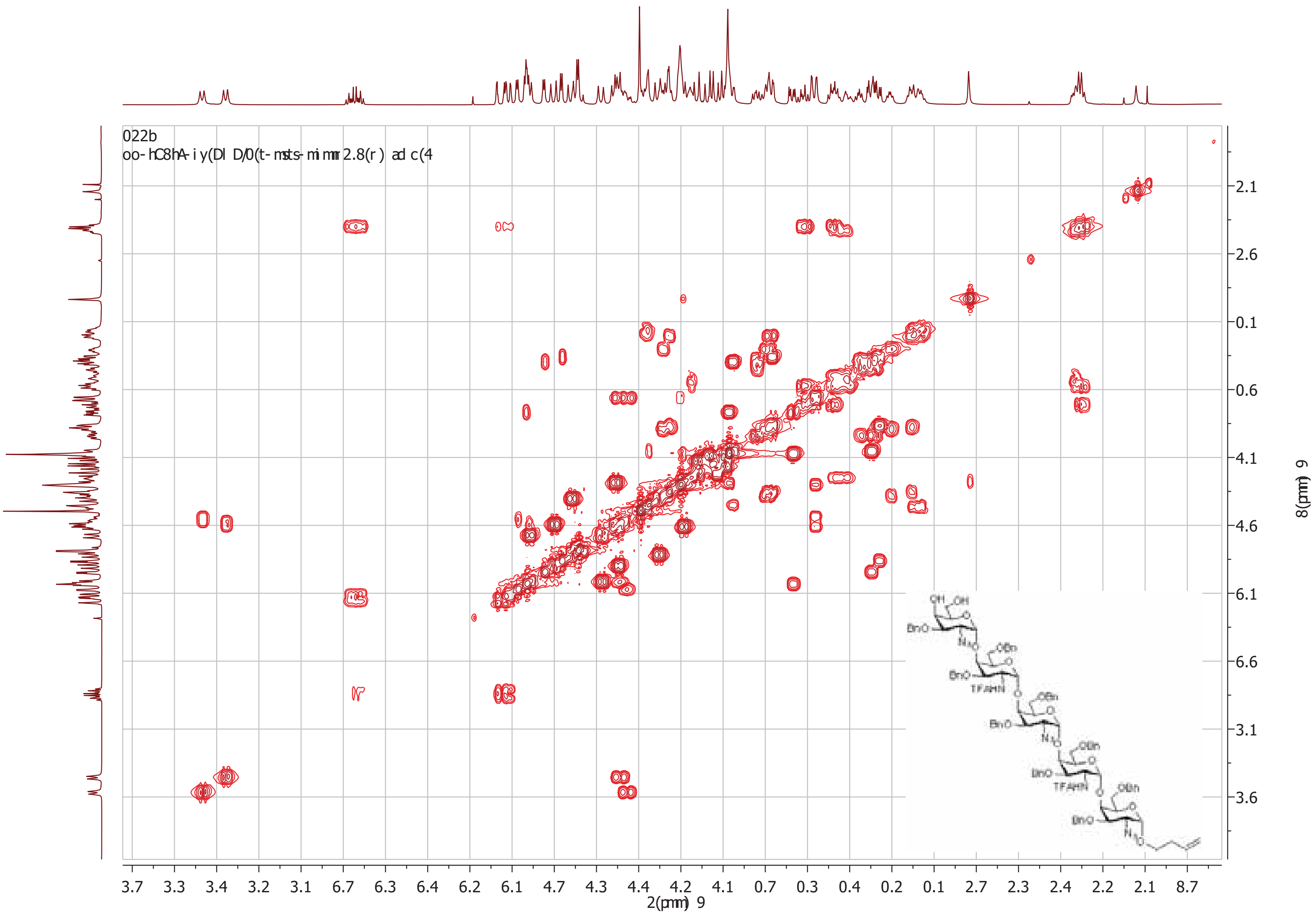
022b
BnO
TFAHN
OBn

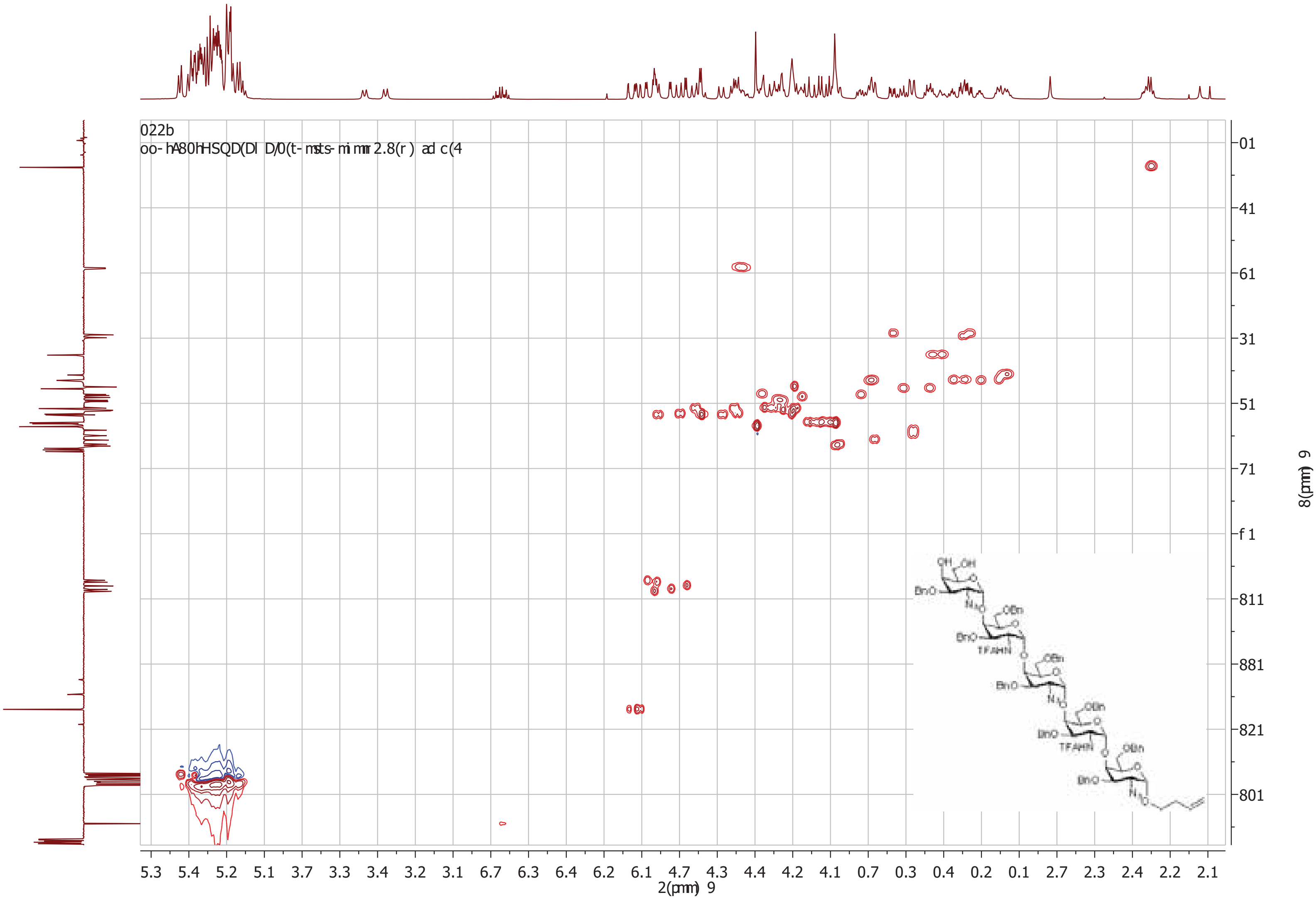
022b
2(pm) 9
8(pm) 9
OH
BnO
OBn
TFAHN

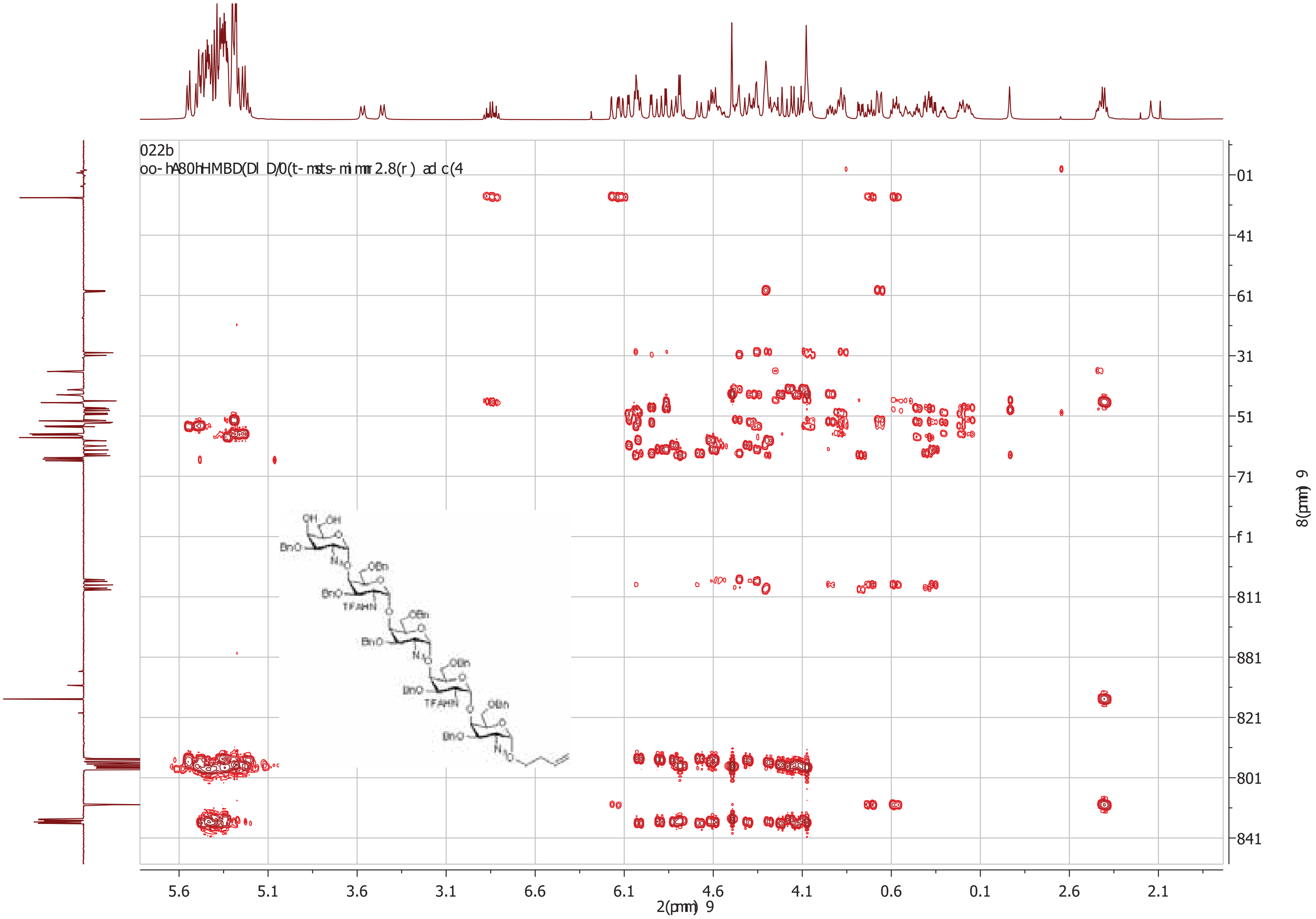
022b
oo- hA80hHMBD(DI D/0(t- msts- mi mr 2.8(r) ad c(4
2(pm) 9
8(pm) 9
OH
OBn
BnO
TFAHN

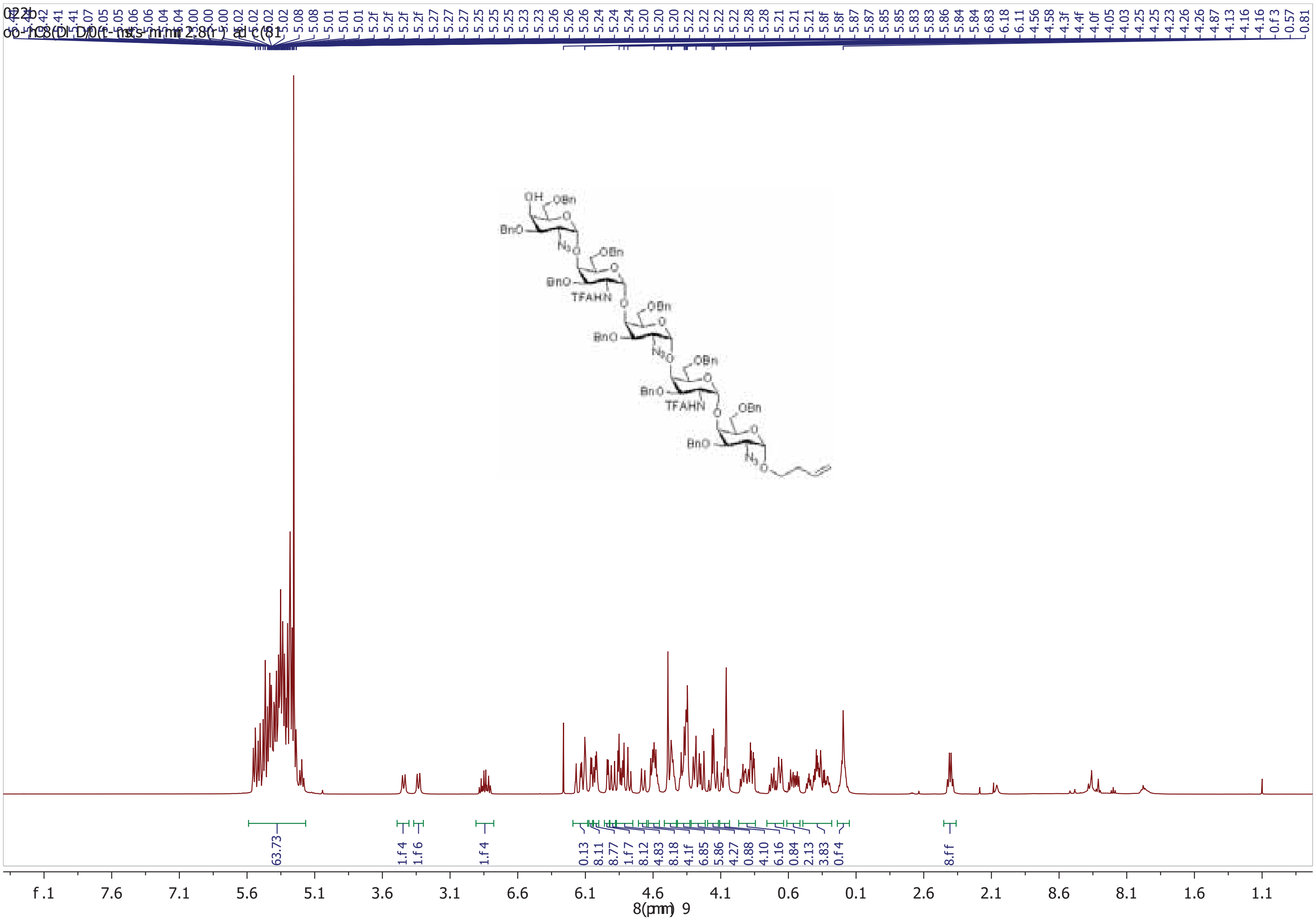
OH
OBn
BnO
N3
TFAHN
63.73

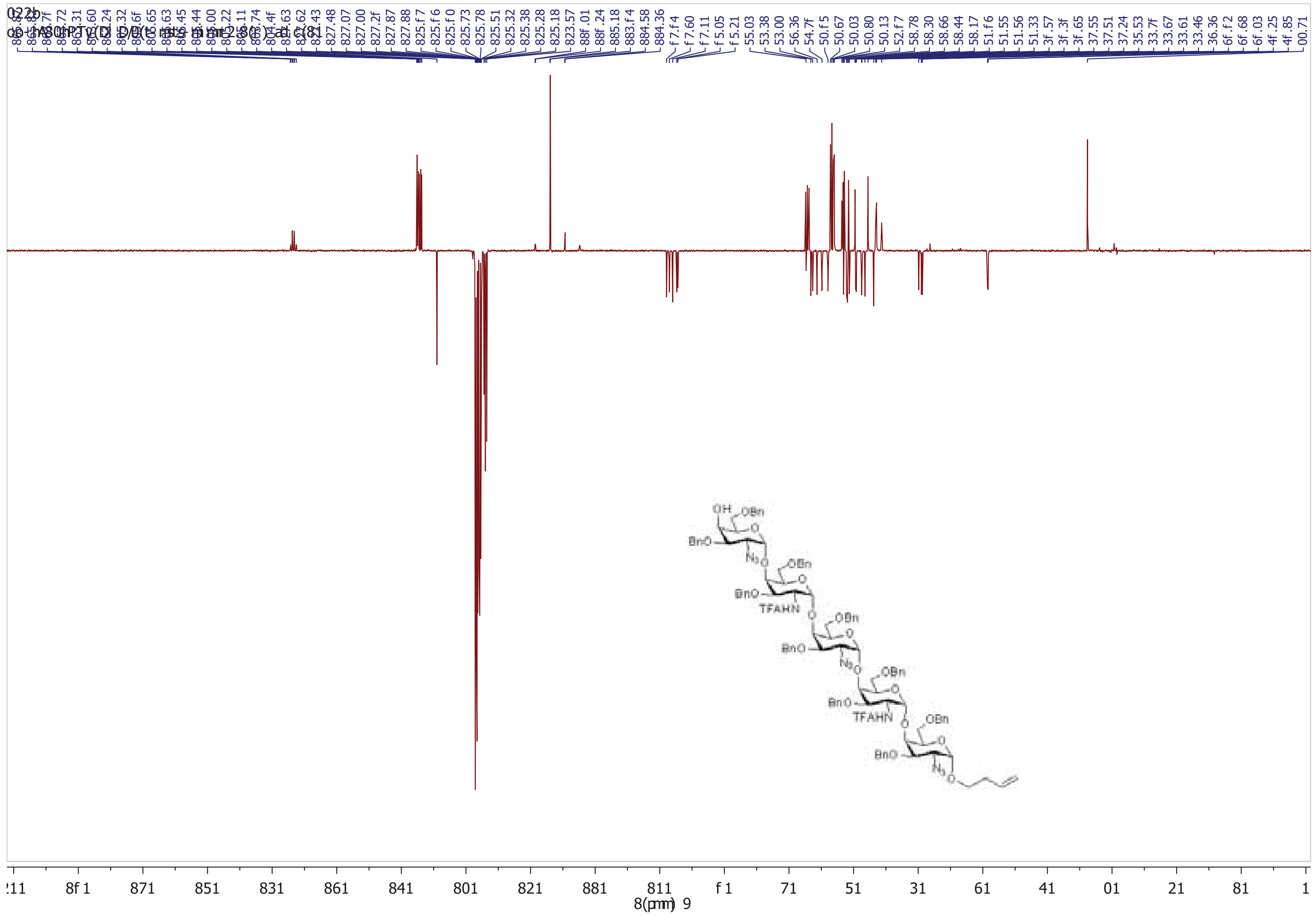
OH
OBn
BnO
N3
TFAHN

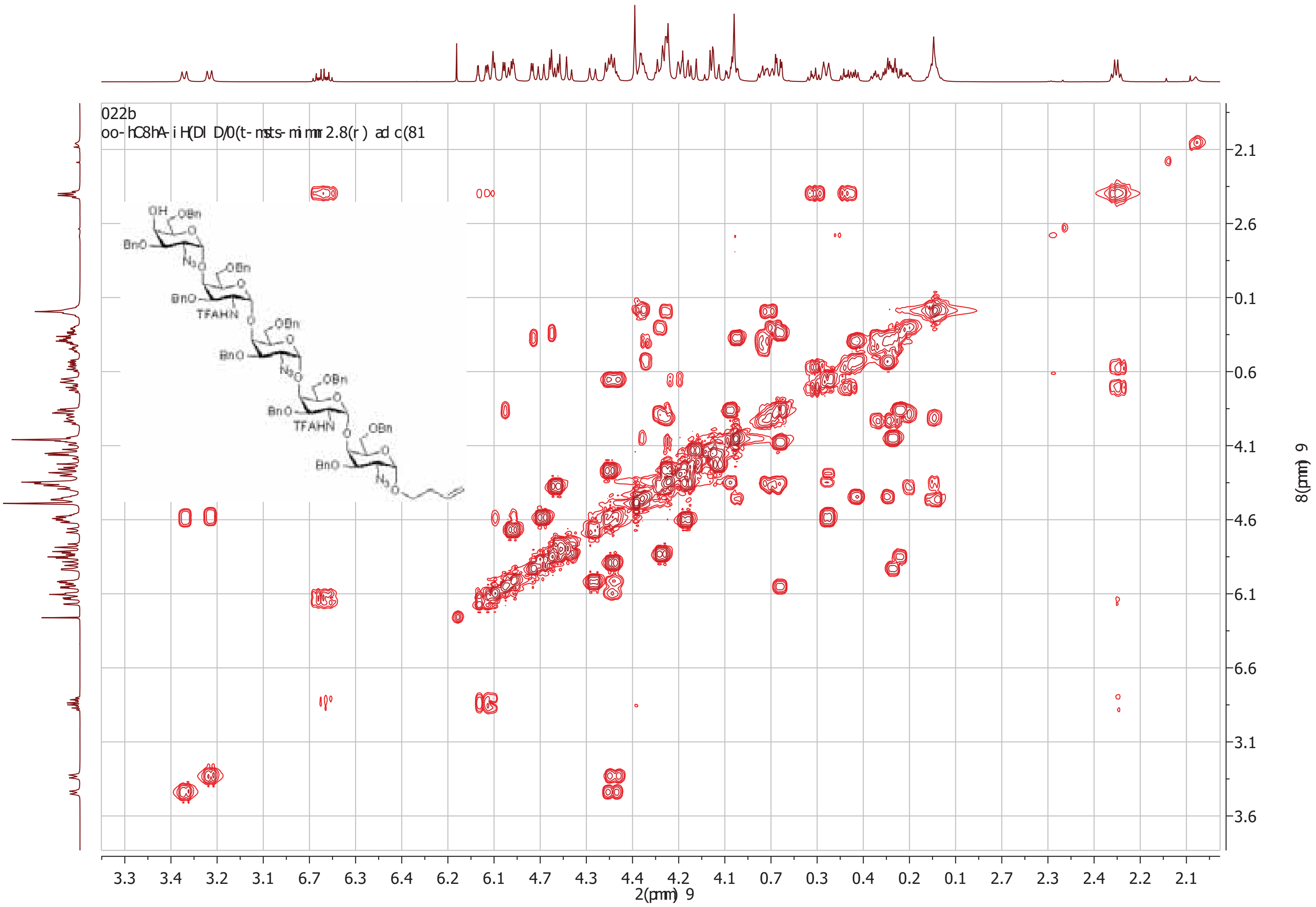
022b
OH
OBn
BnO
N3
TFAHN

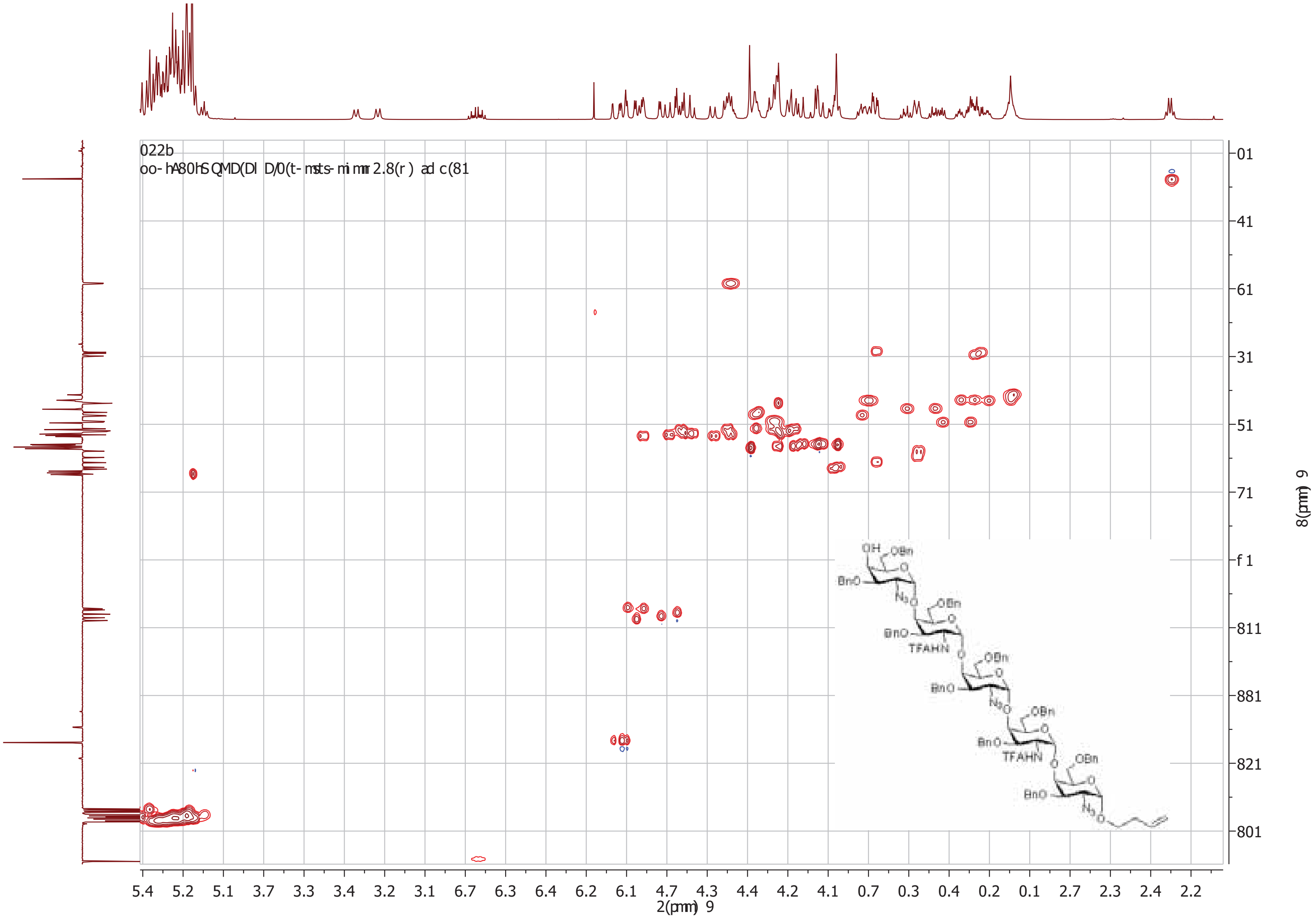
022b
oo- hA80hSQMD(DI D/0(t- msts- mi mr 2.8(r) ad c(81
2(pm) 9
8(pm) 9
OH
OBn
BnO
N3
TFAHN
O

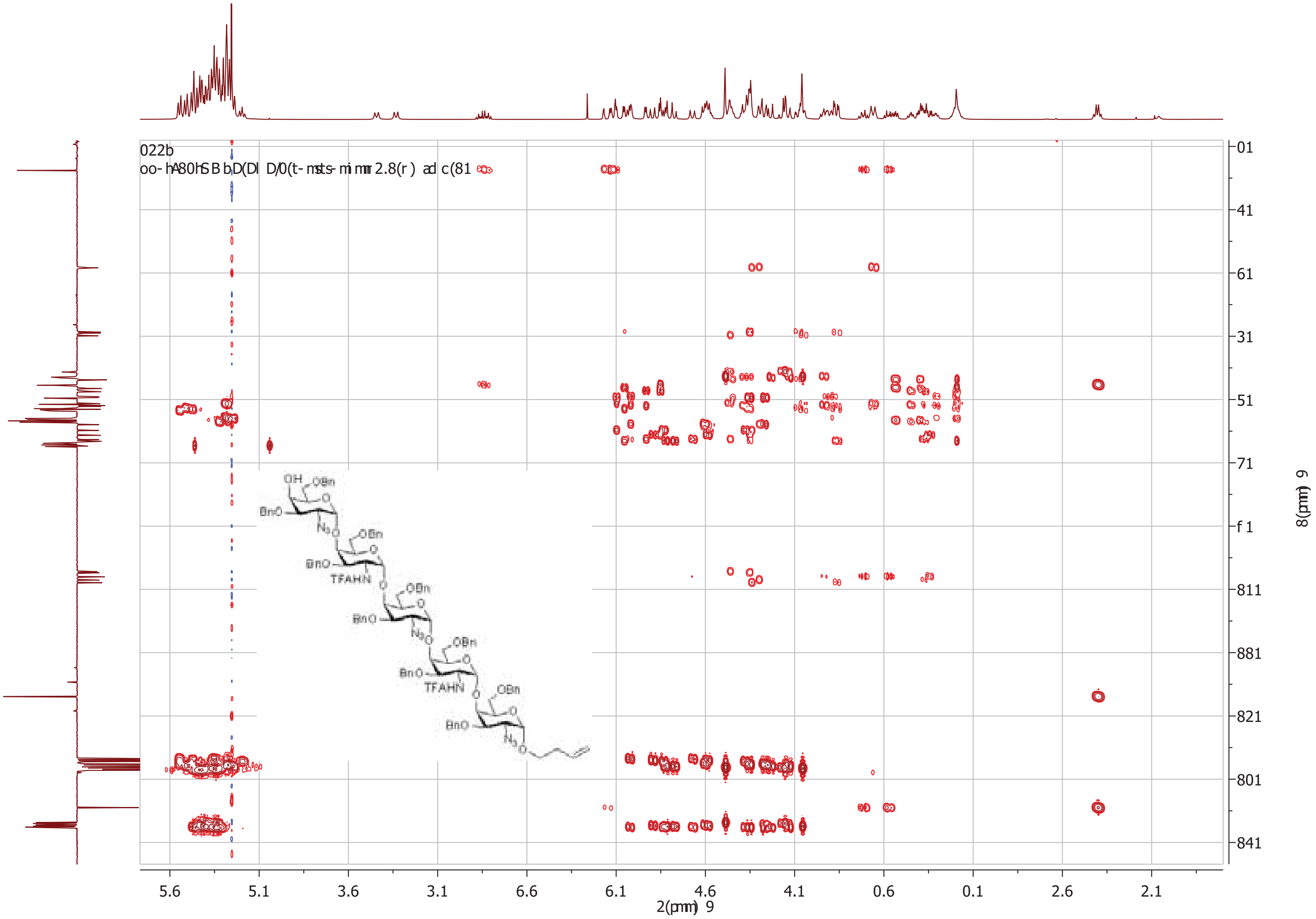

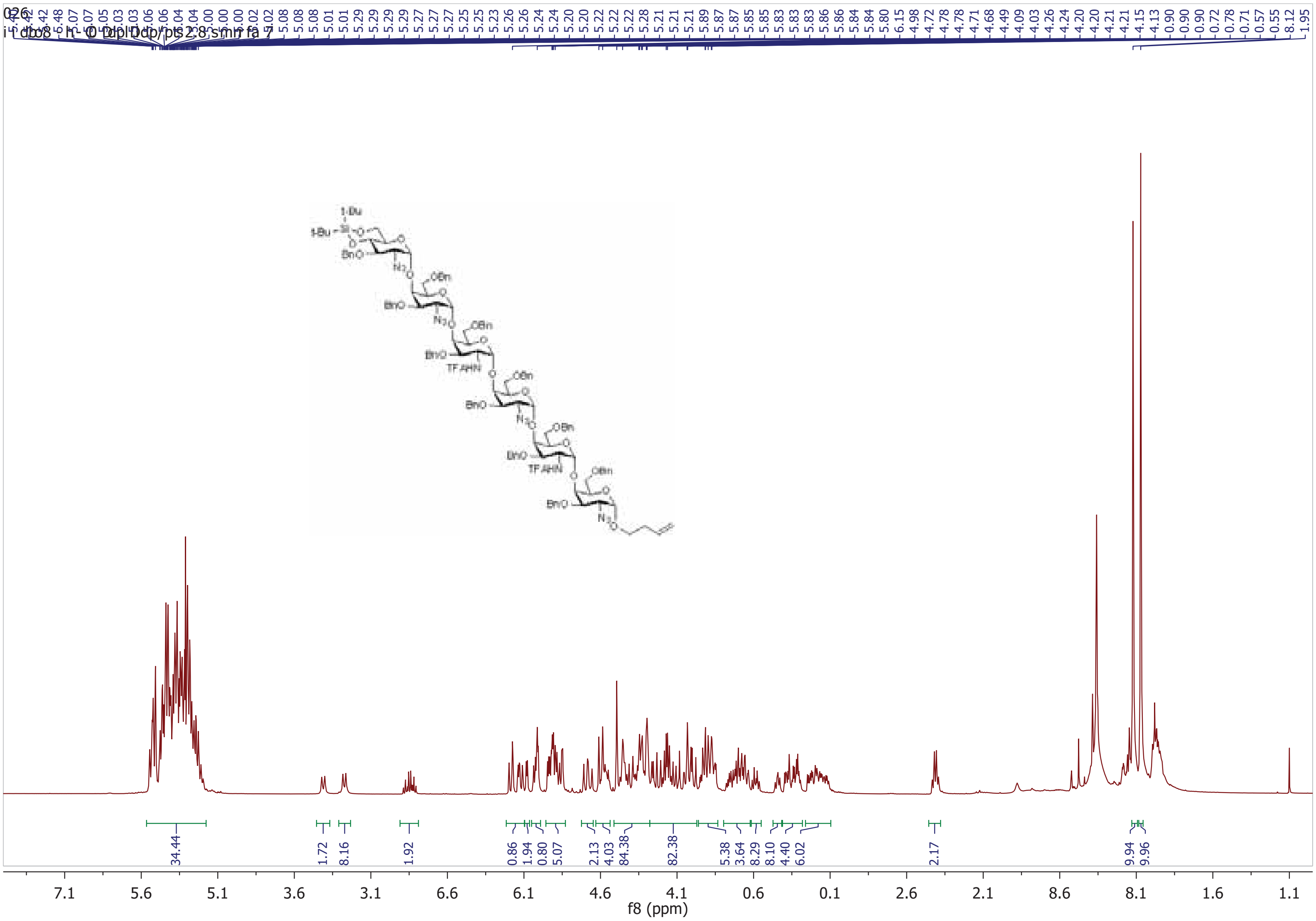

34.44
1.72
8.16
1.92
0.86
1.94
0.80
5.07
2.13
4.03
84.38
82.38
5.38
3.64
8.29
8.10
4.40
6.02
2.17
9.94
9.96
7.1 5.6 5.1 3.6 3.1 6.6 6.1 4.6 4.1 0.6 0.1 2.6 2.1 8.6 8.1 1.6 1.1
f8 (ppm)

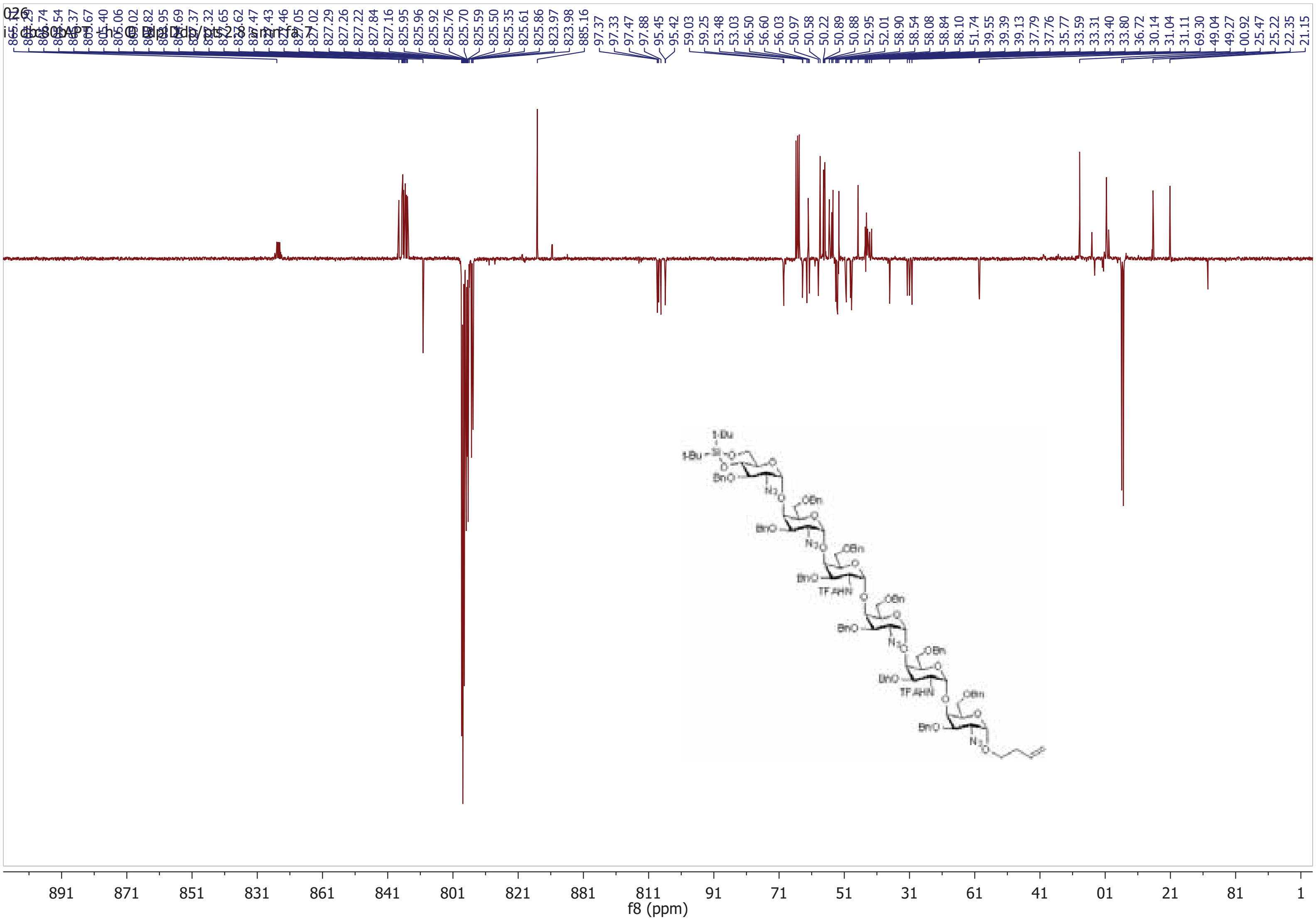
f8 (ppm)

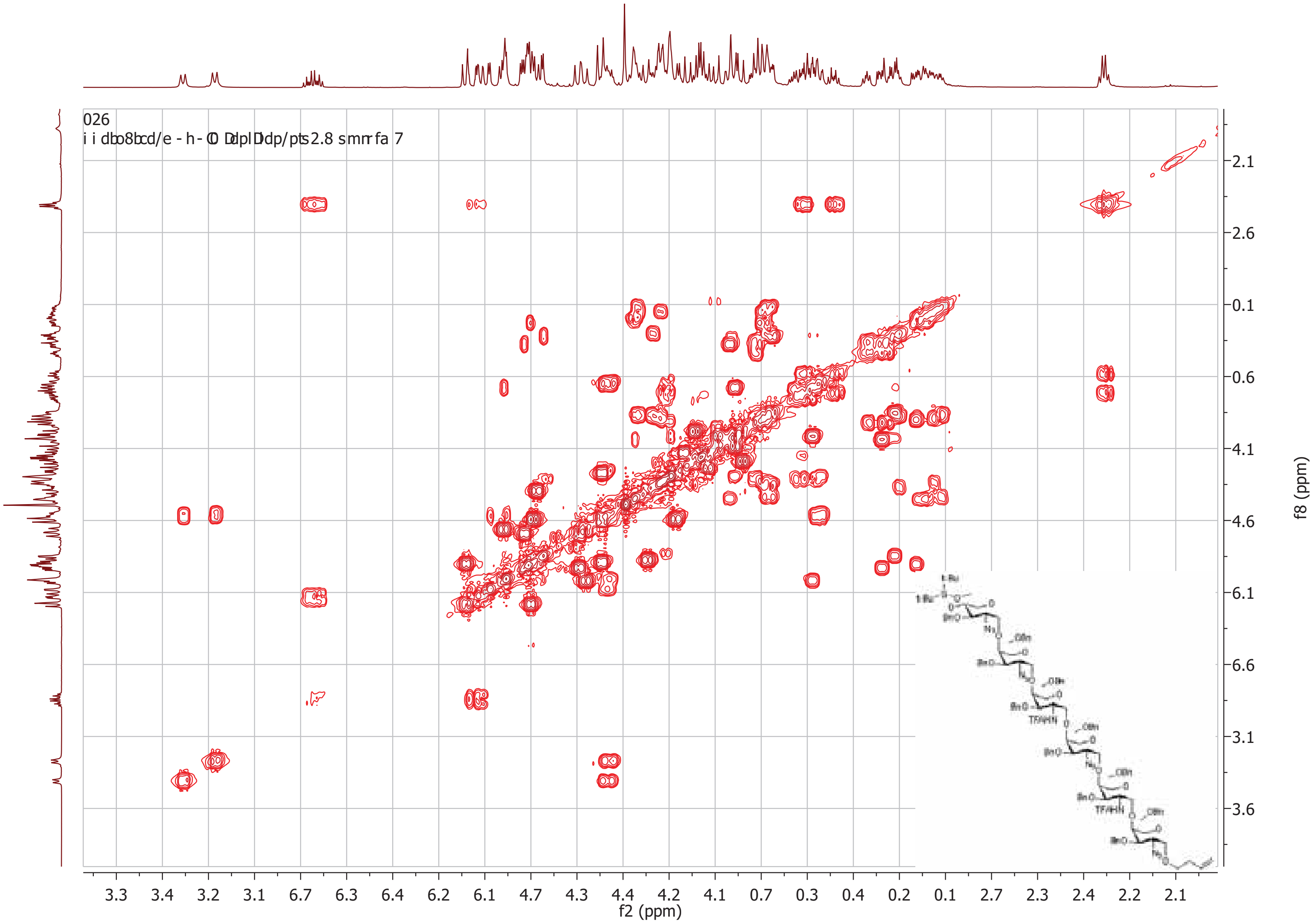
026
f2 (ppm)
f8 (ppm)

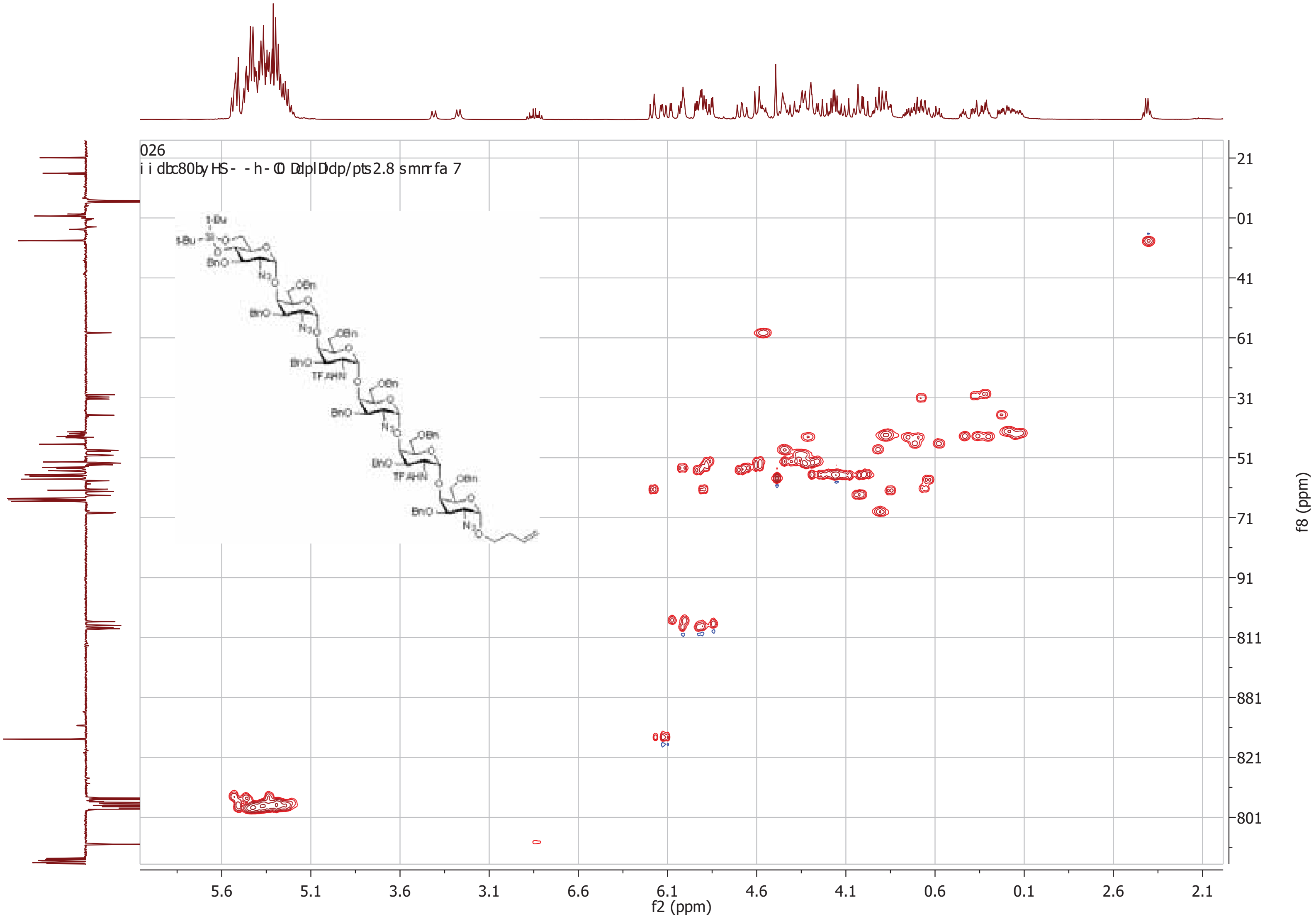

026
f2 (ppm)
f8 (ppm)
t-Bu
t-Bu
Si
BnO
N3
OBn
TFAHN

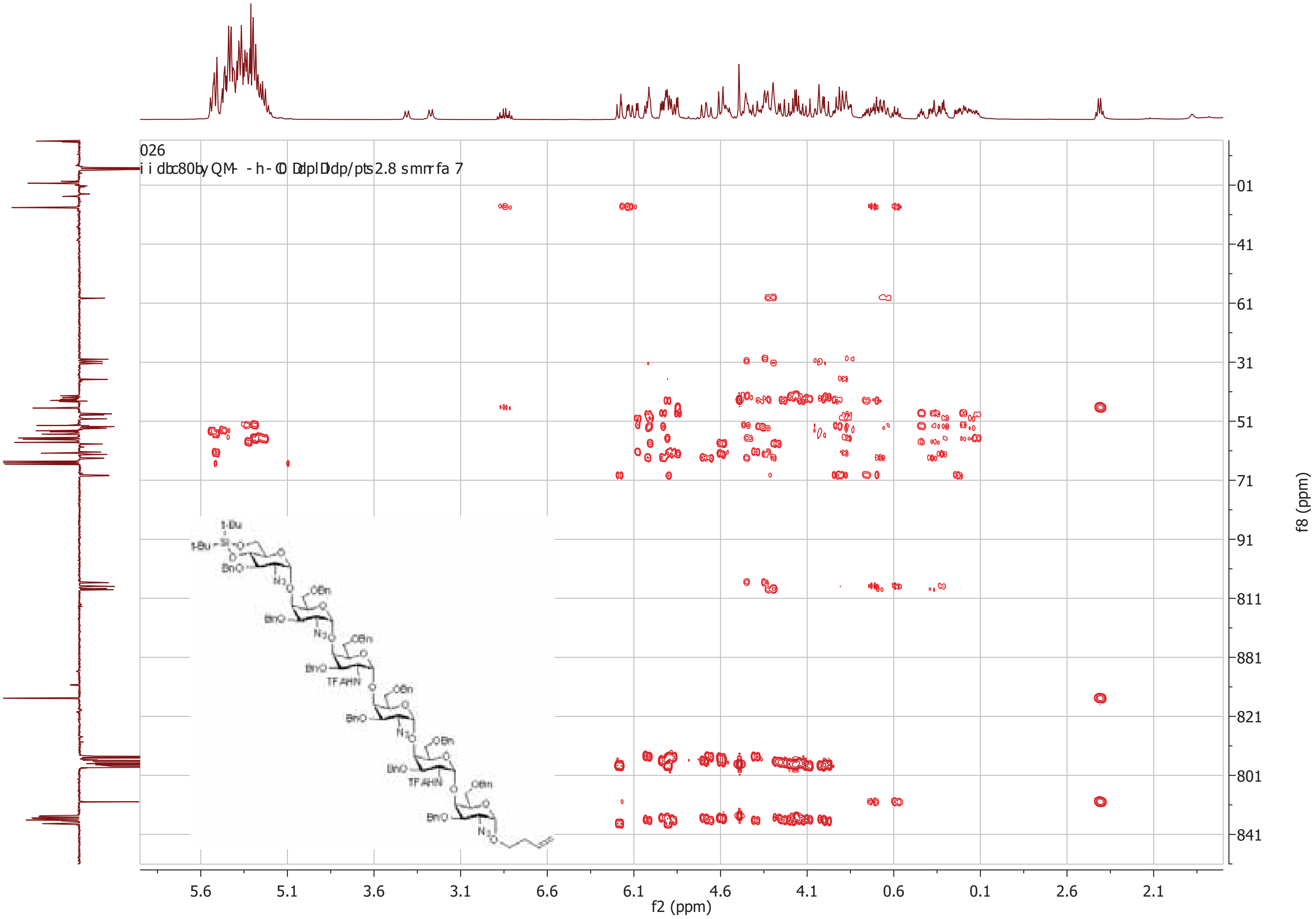

f2 (ppm)
f8 (ppm)
t-Bu
BnO
OBn
TFAHN

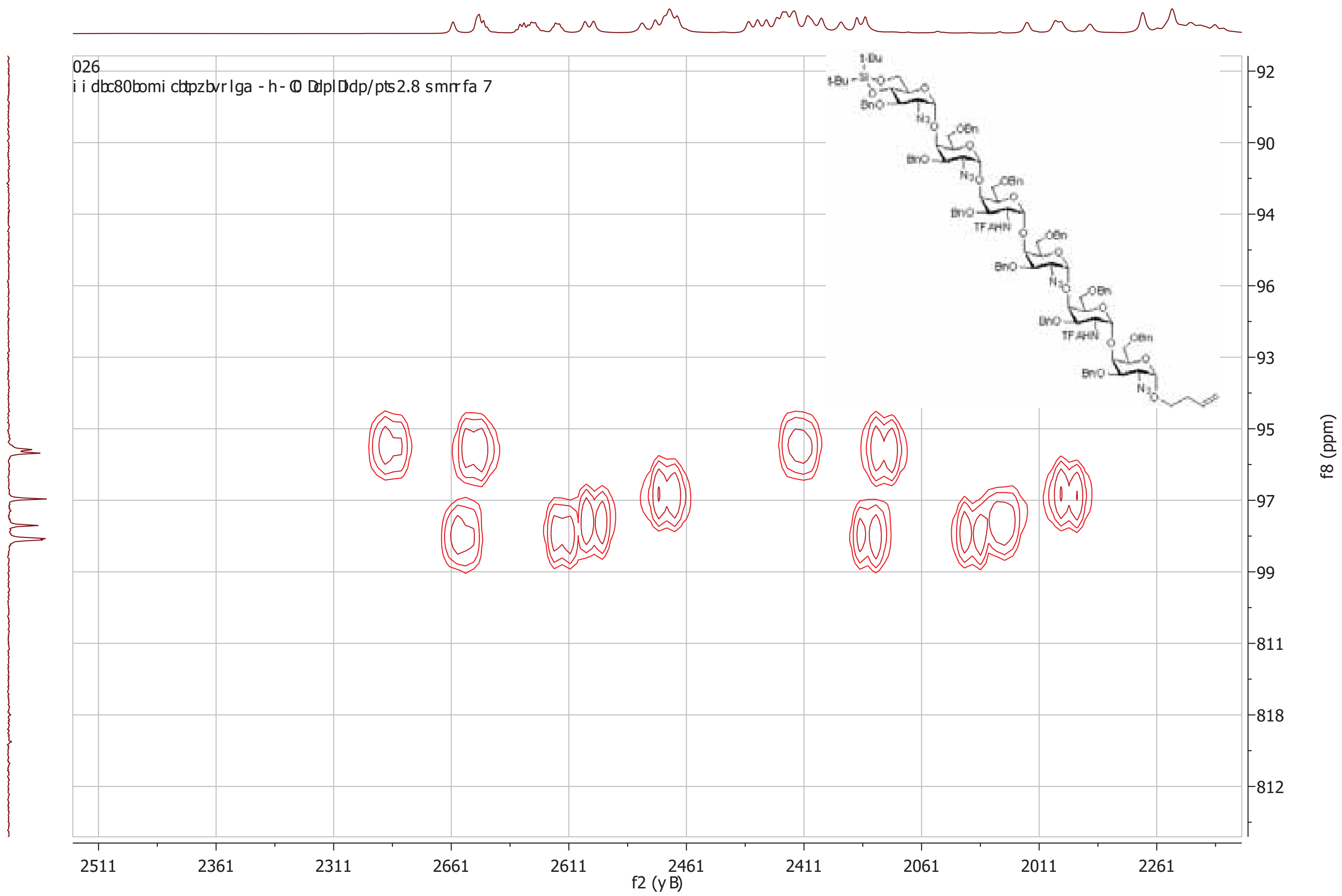

026
i i dbc80bomi cbtpzbvrlga - h- ℂ Ddpl Ddp/pts2.8 smrrfa 7
t-Bu
t-Bu
Si
BnO
N3
OBn
TFAHN
f2 (y B)
f8 (ppm)

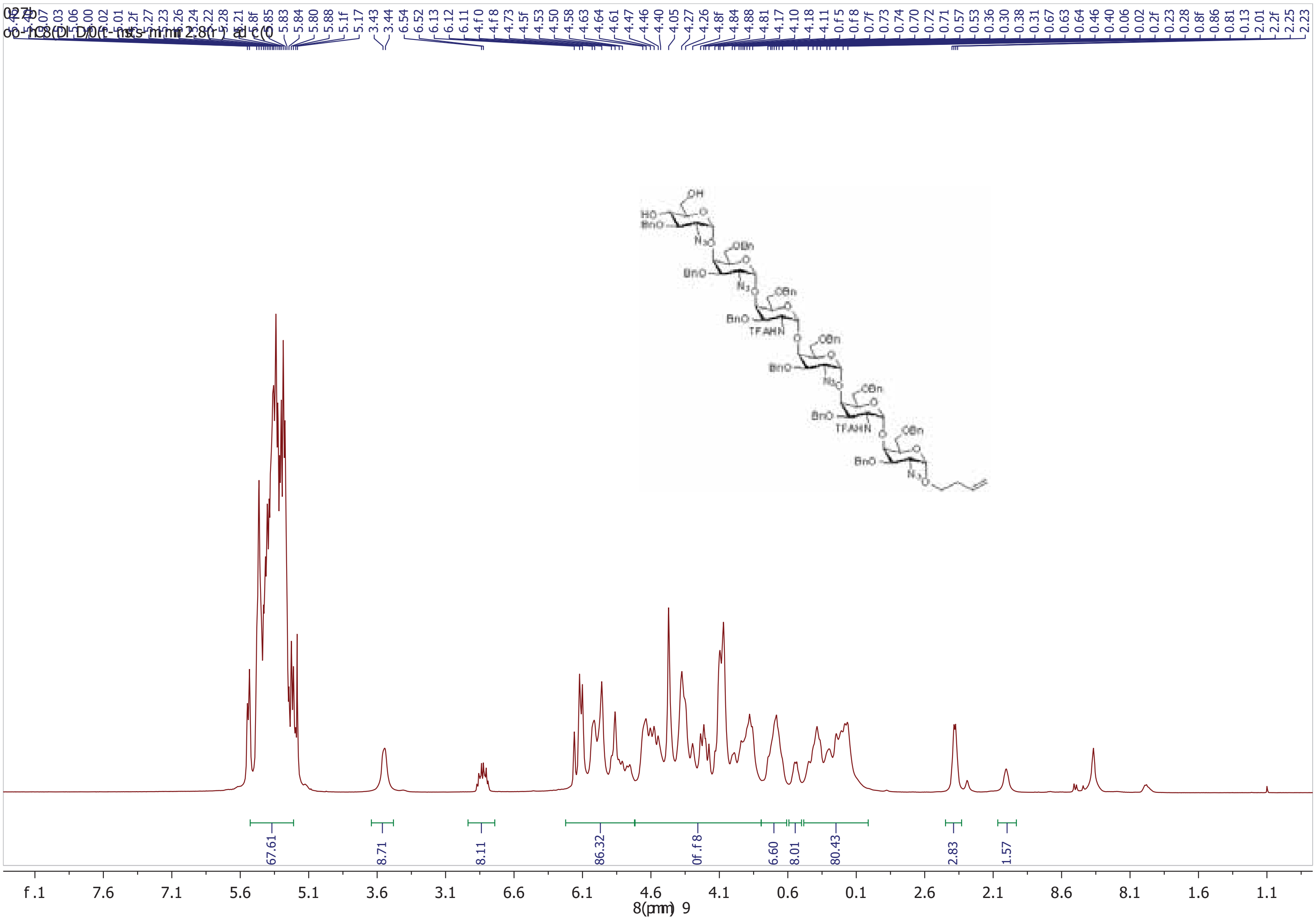

OH
HO
BnO
N3
OBn
TFAHN
67.61
8.71
8.11
86.32
6.60
8.01
80.43
2.83
1.57

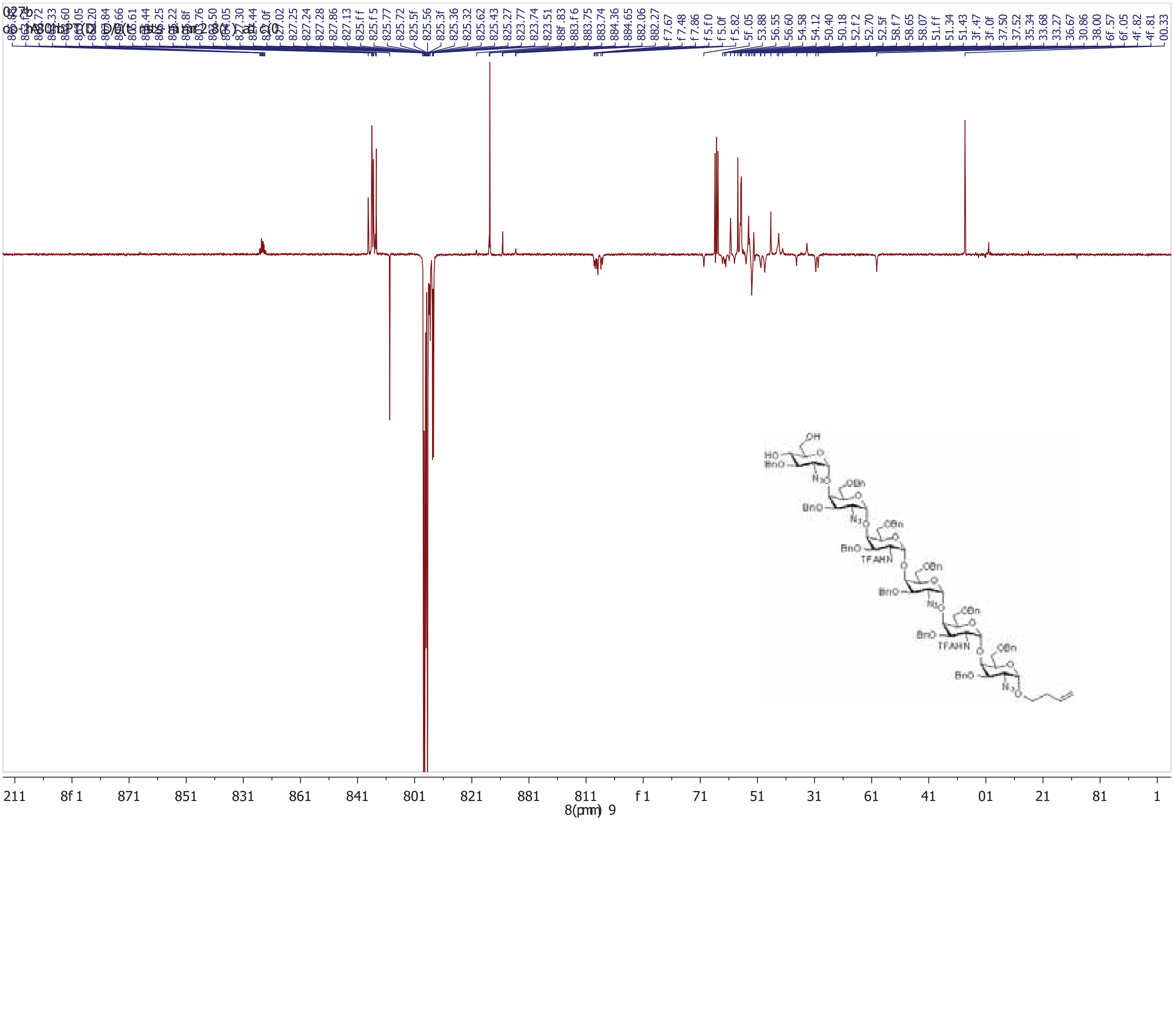
OH
HO
BnO
N3
OBn
BnO
N3
OBn
BnO
TFAHN
OBn
BnO
N3
OBn
BnO
TFAHN
OBn
BnO
N3

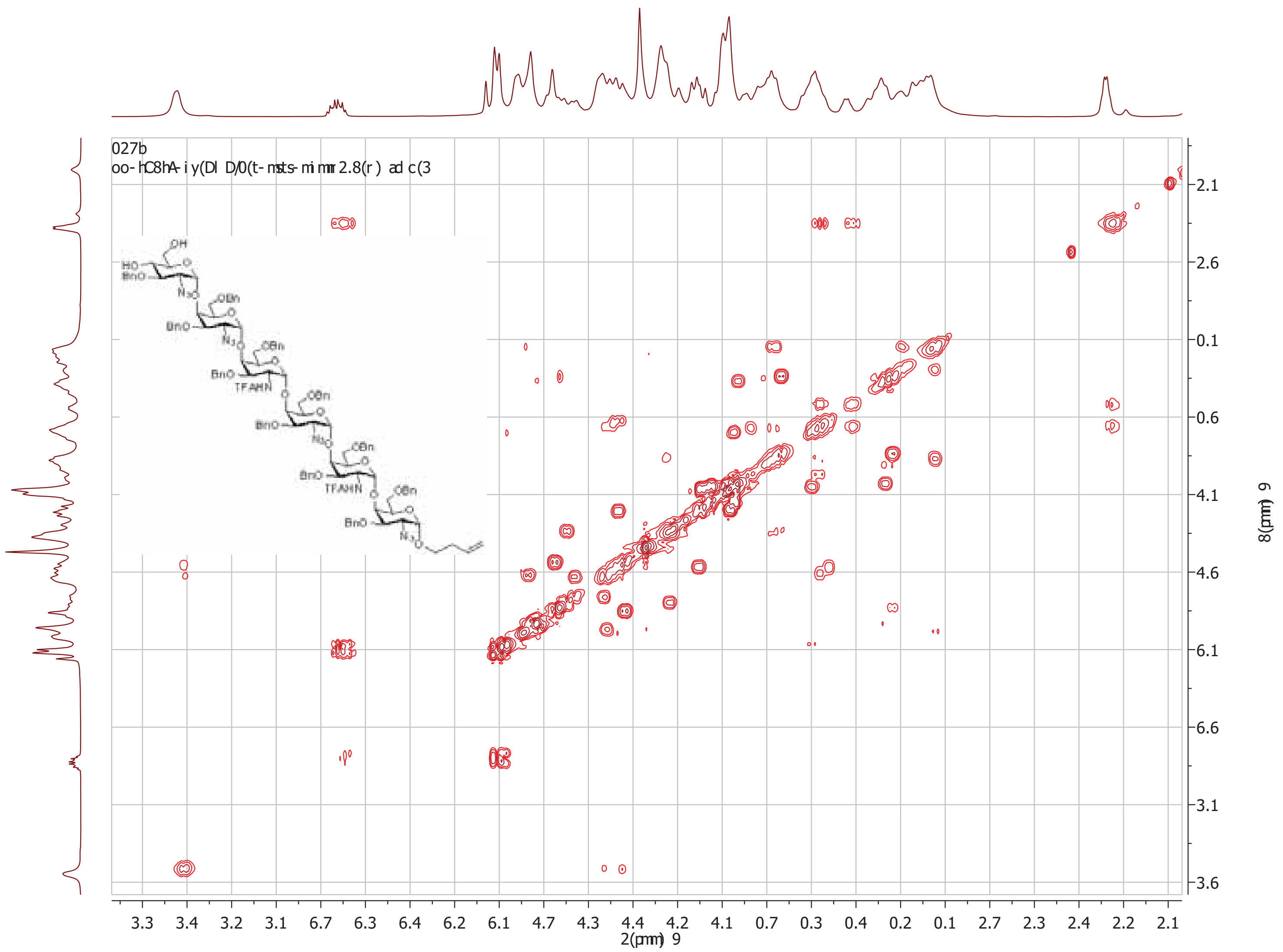
027b
OH
HO
BnO
N3
OBn
TFAHN

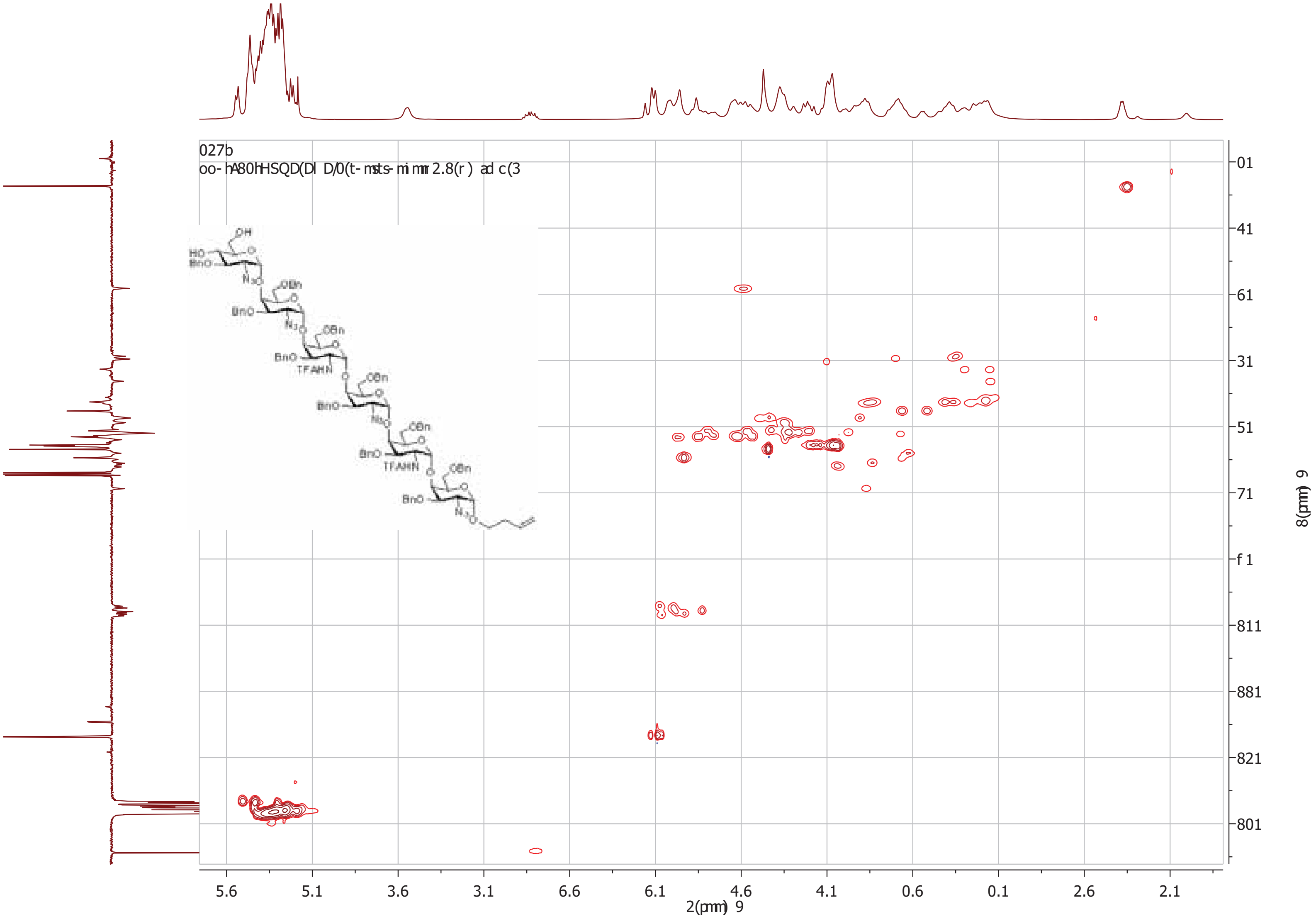
027b
oo- hA80hHSQD(DI D/0(t- msts- mi mr 2.8(r) ad c(3
OH
HO
BnO
N3
OBn
BnO
N3
OBn
BnO
TFAHN
OBn
BnO
N3
OBn
BnO
TFAHN
OBn
BnO
N3
2(pm) 9
8(pm) 9

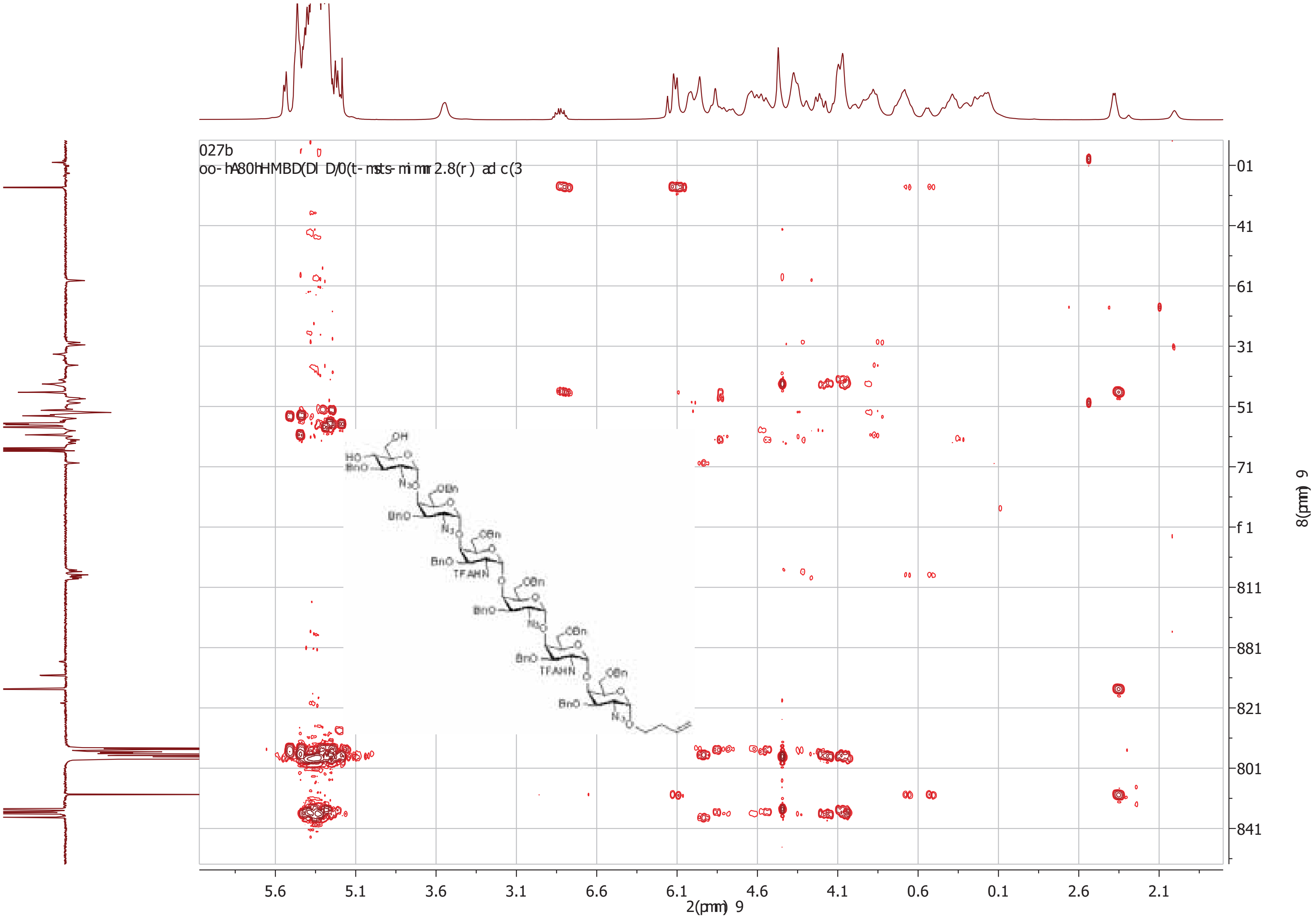
027b
oo- hA80hHMBD(Dl D/0(t- mstsmimr2.8(r) ad c(3
2(pm) 9
8(pm) 9
OH
HO
BnO
N_3
OBn
TFAHN

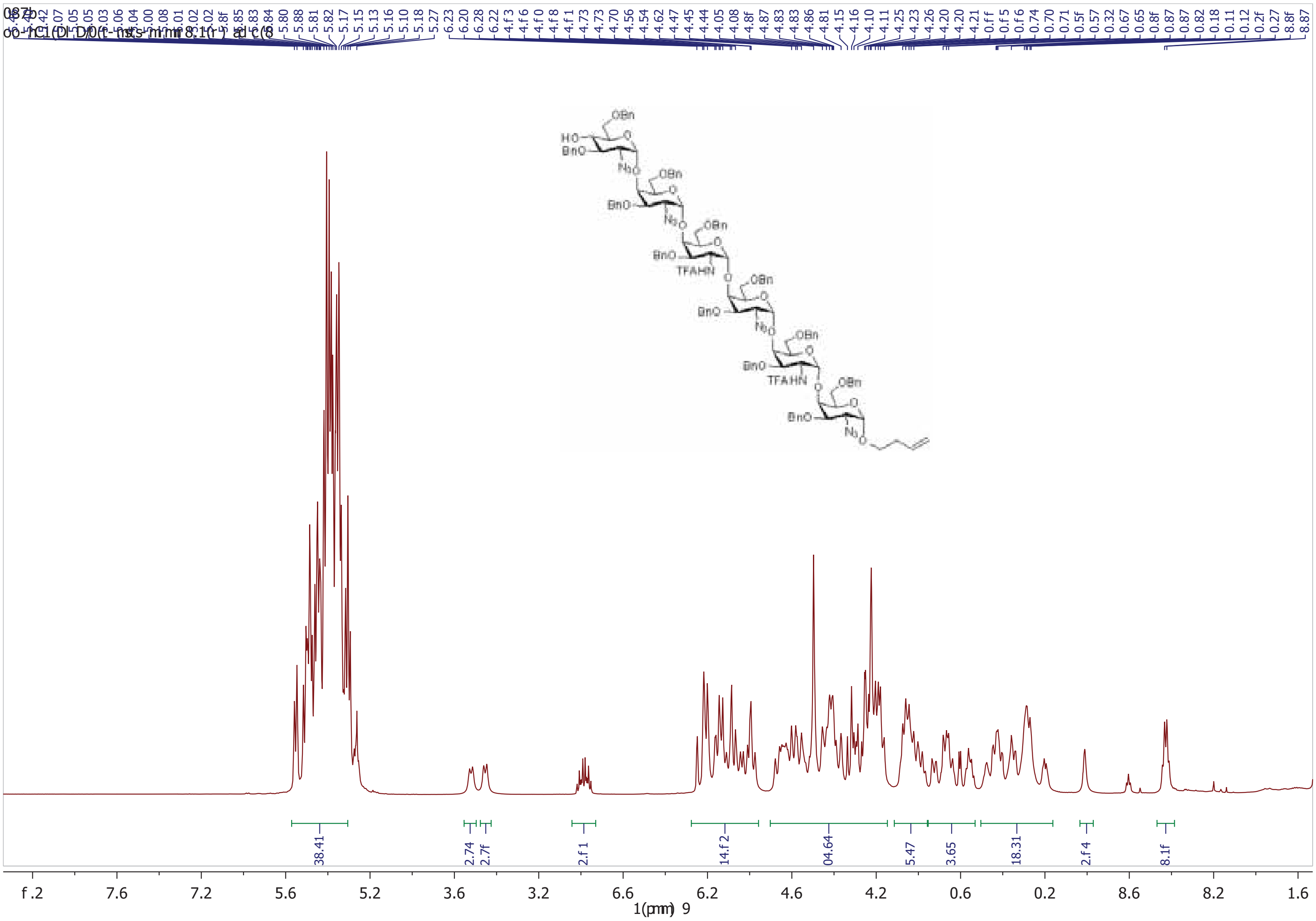
OBn
HO
BnO
N3
TFAHN
1(pmm) 9

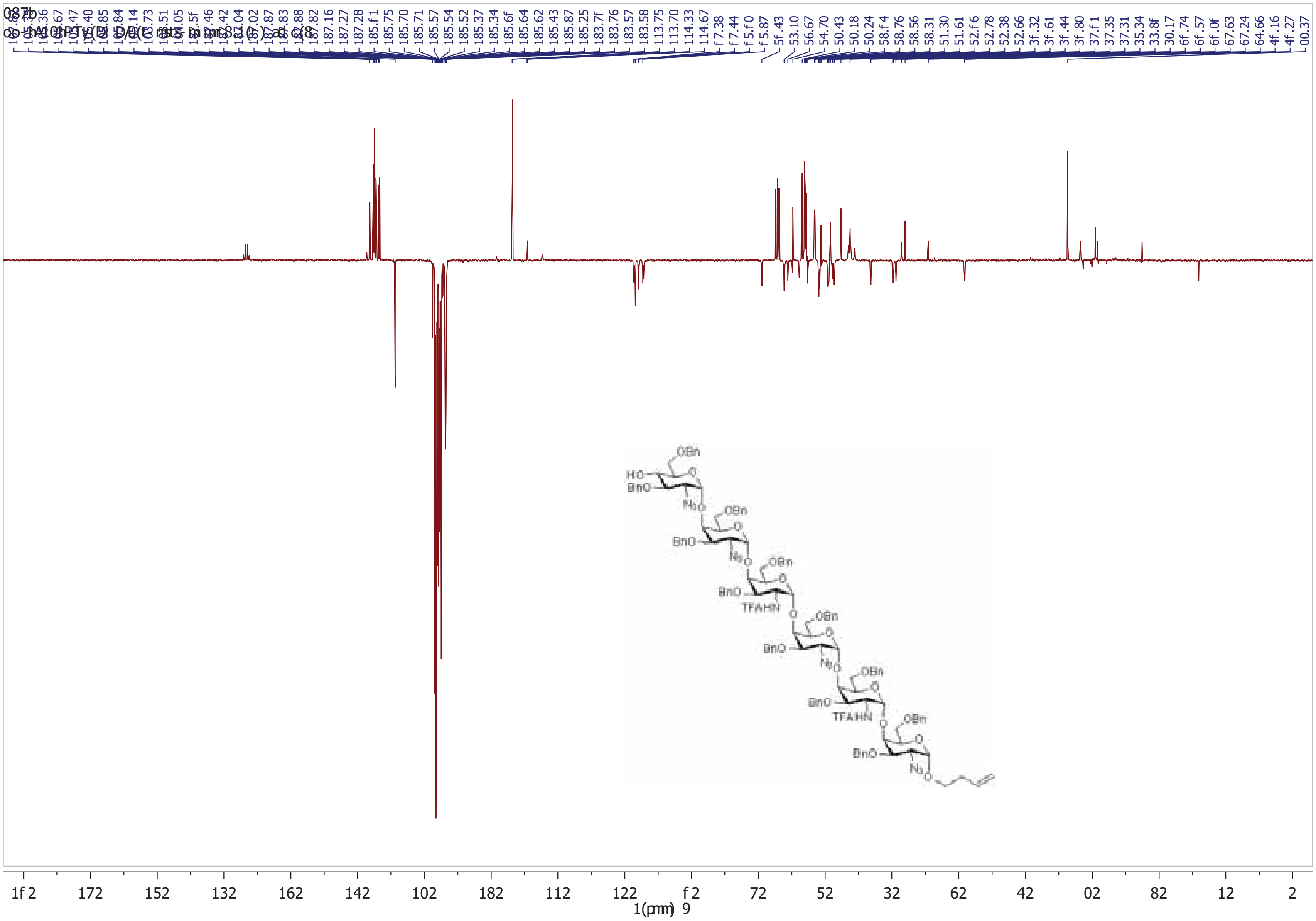
087b
OBn
HO
BnO
N3
TFAHN

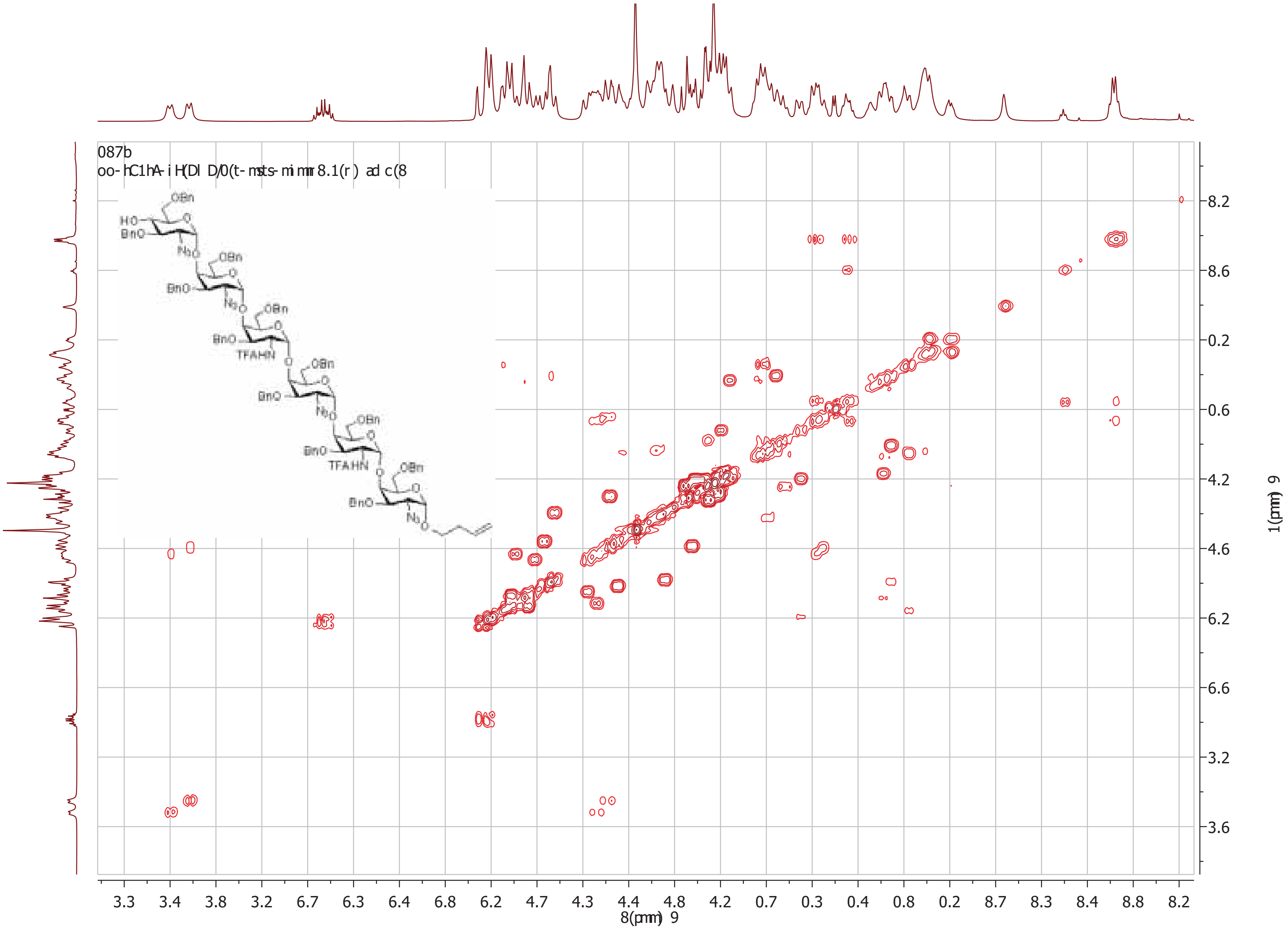
087b
oo-hC1hA-i H(DI D/0(t-msts-mimr 8.1(r) ad c(8
OBn
HO
BnO
N3
TFAHN

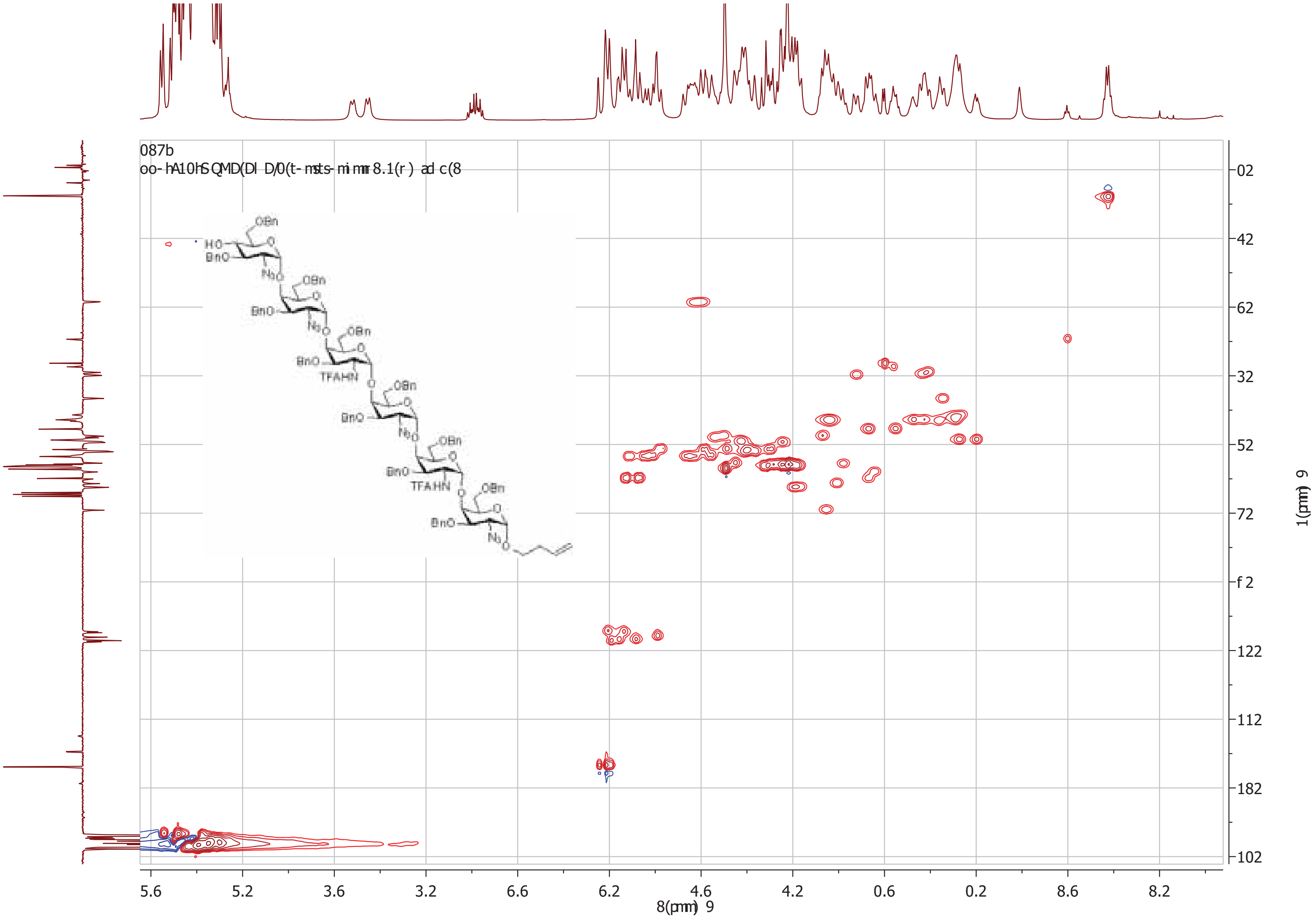
087b
OBn
HO
BnO
N3
TFAHN

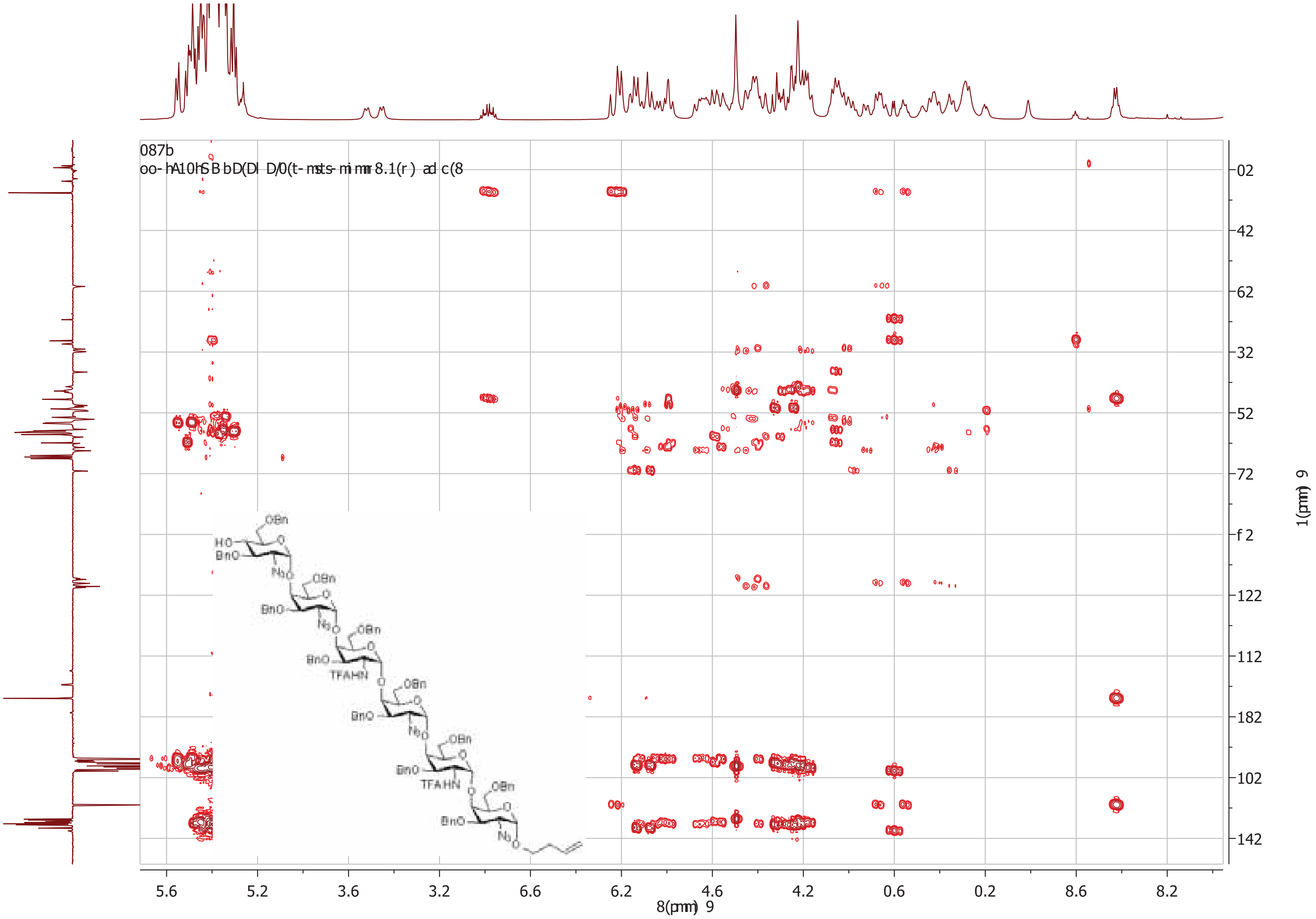
087b
HO
OBn
BnO
N3
TFAHN

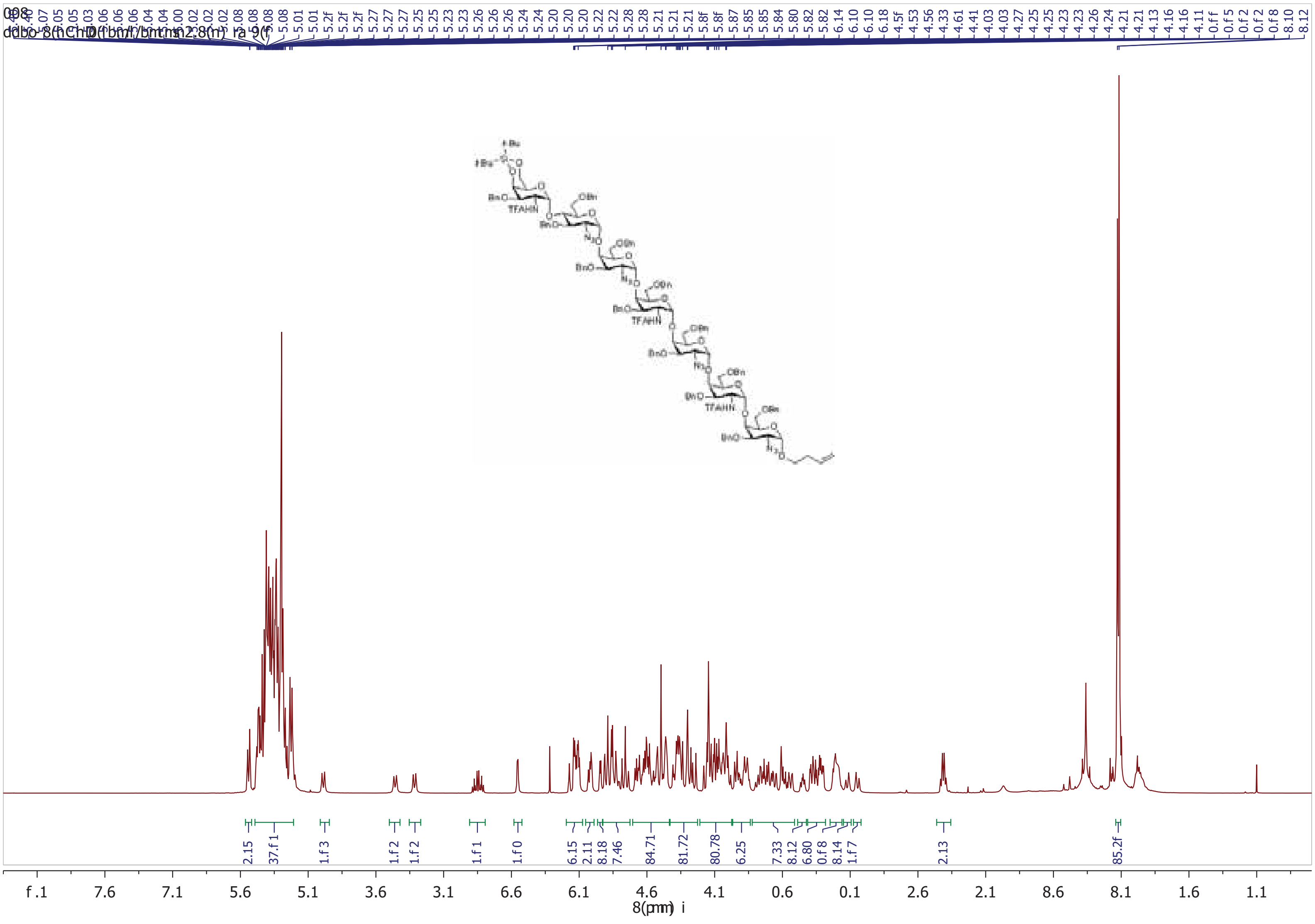
2.15
37.f1
1.f3
1.f2
1.f2
1.f1
1.f0
6.15
2.11
8.18
7.46
84.71
81.72
80.78
6.25
7.33
8.12
6.80
0.f8
8.14
1.f7
2.13
85.2f
f .1 7.6 7.1 5.6 5.1 3.6 3.1 6.6 6.1 4.6 4.1 0.6 0.1 2.6 2.1 8.6 8.1 1.6 1.1
8(pm) i

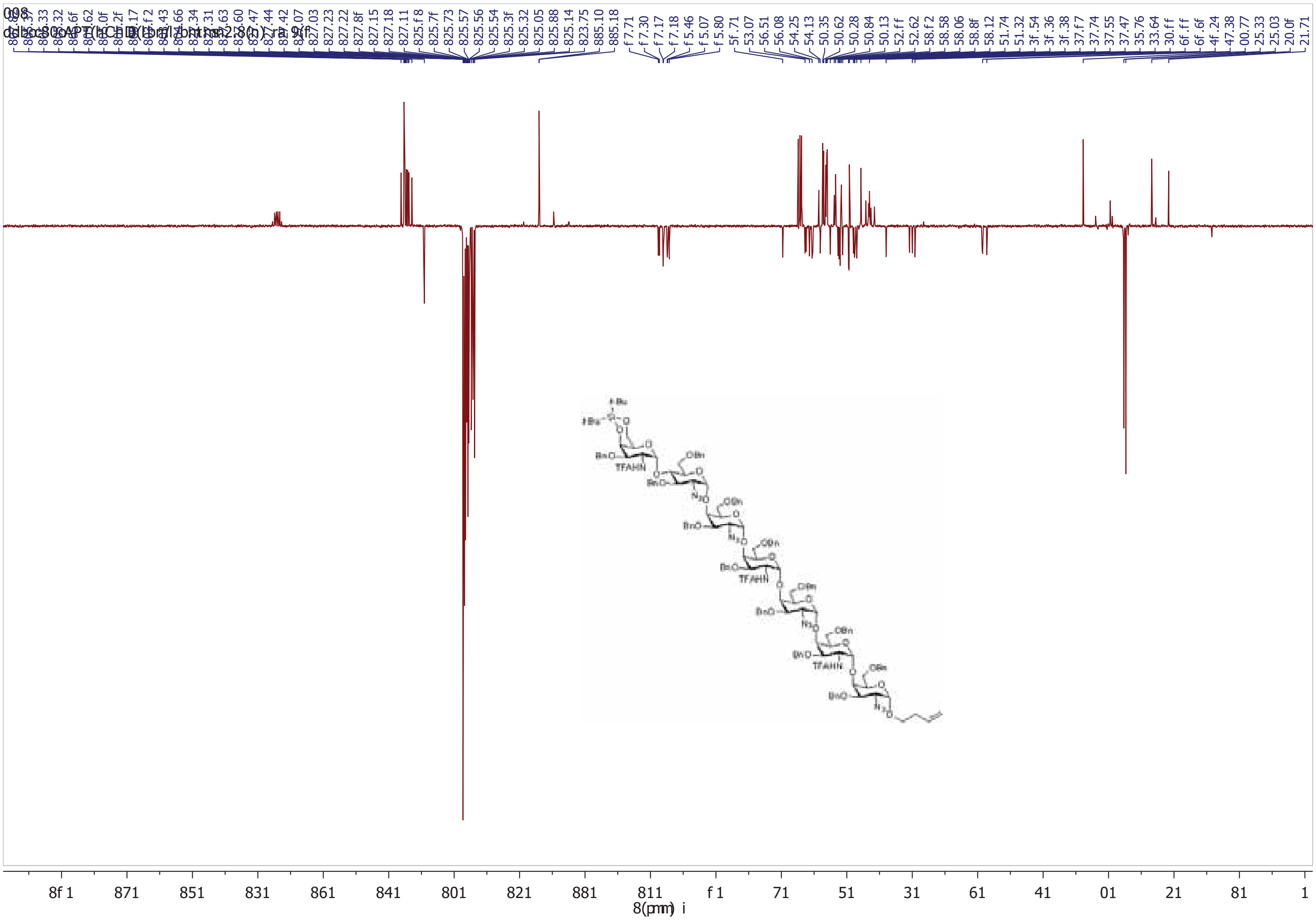

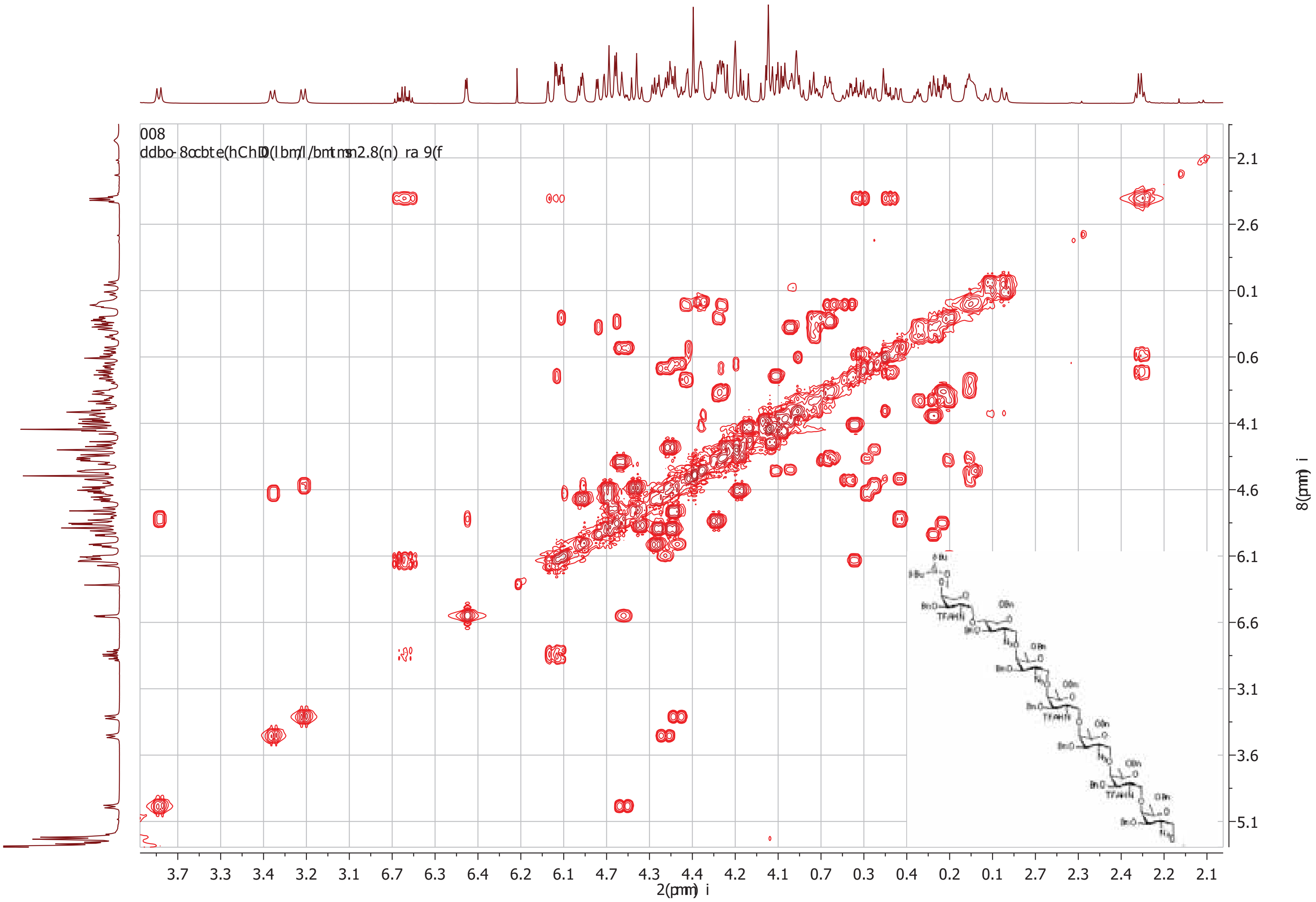

008
ddbo- 8ccbte(hChD0(lbm/l/bmtms n2.8(n) ra 9(f
2(pmm) i
8(pmm) i

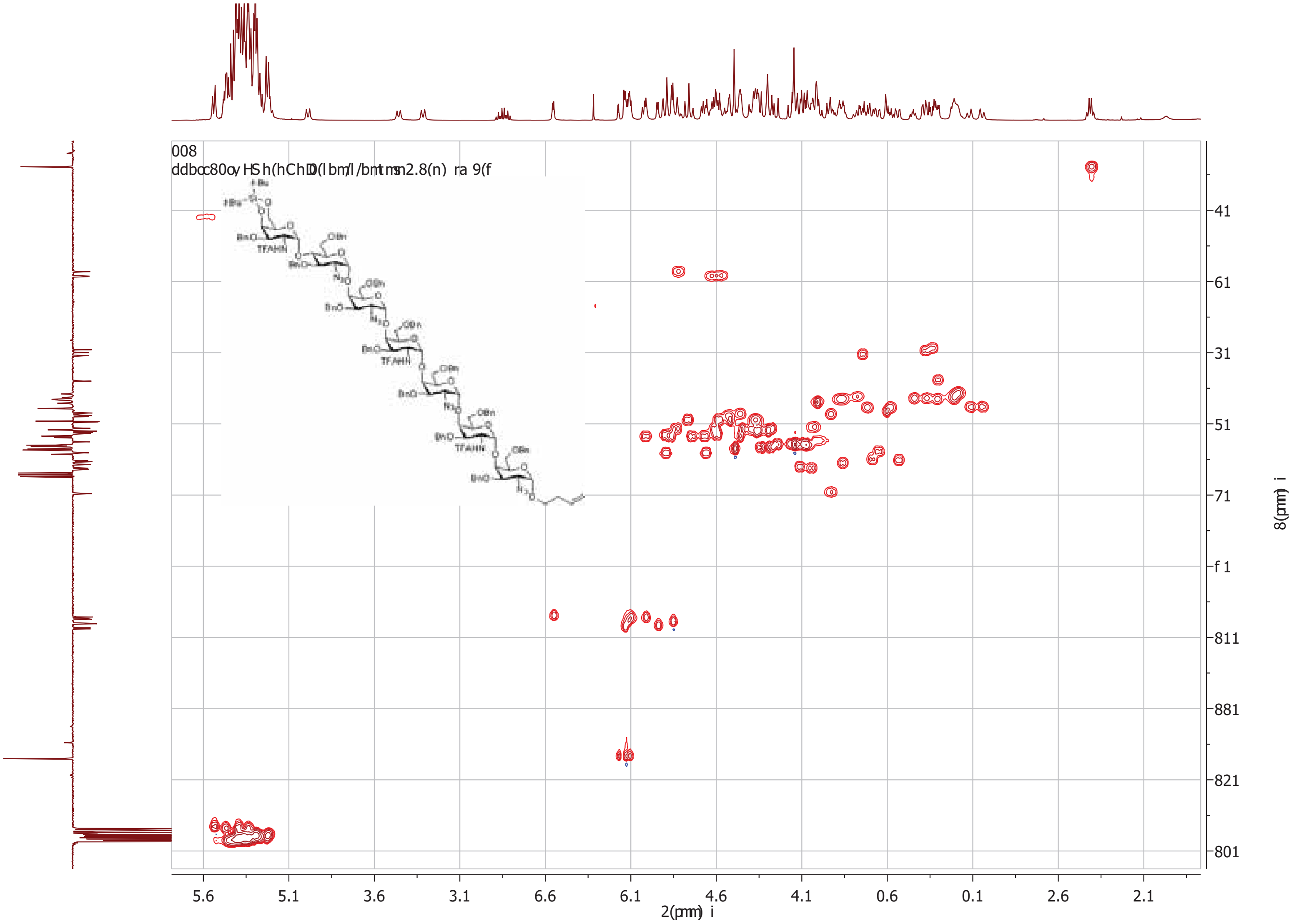
008
ddbc c80o, HS h(hChD(l bm/l /bmt ms2.8(n) ra 9(f
t-Bu
t-Bu
BnO
TFAHN
OBn
N3
5.6
5.1
3.6
3.1
6.6
6.1
4.6
4.1
0.6
0.1
2.6
2.1
2(pm) i
41
61
31
51
71
f 1
811
881
821
801
8(pm) i

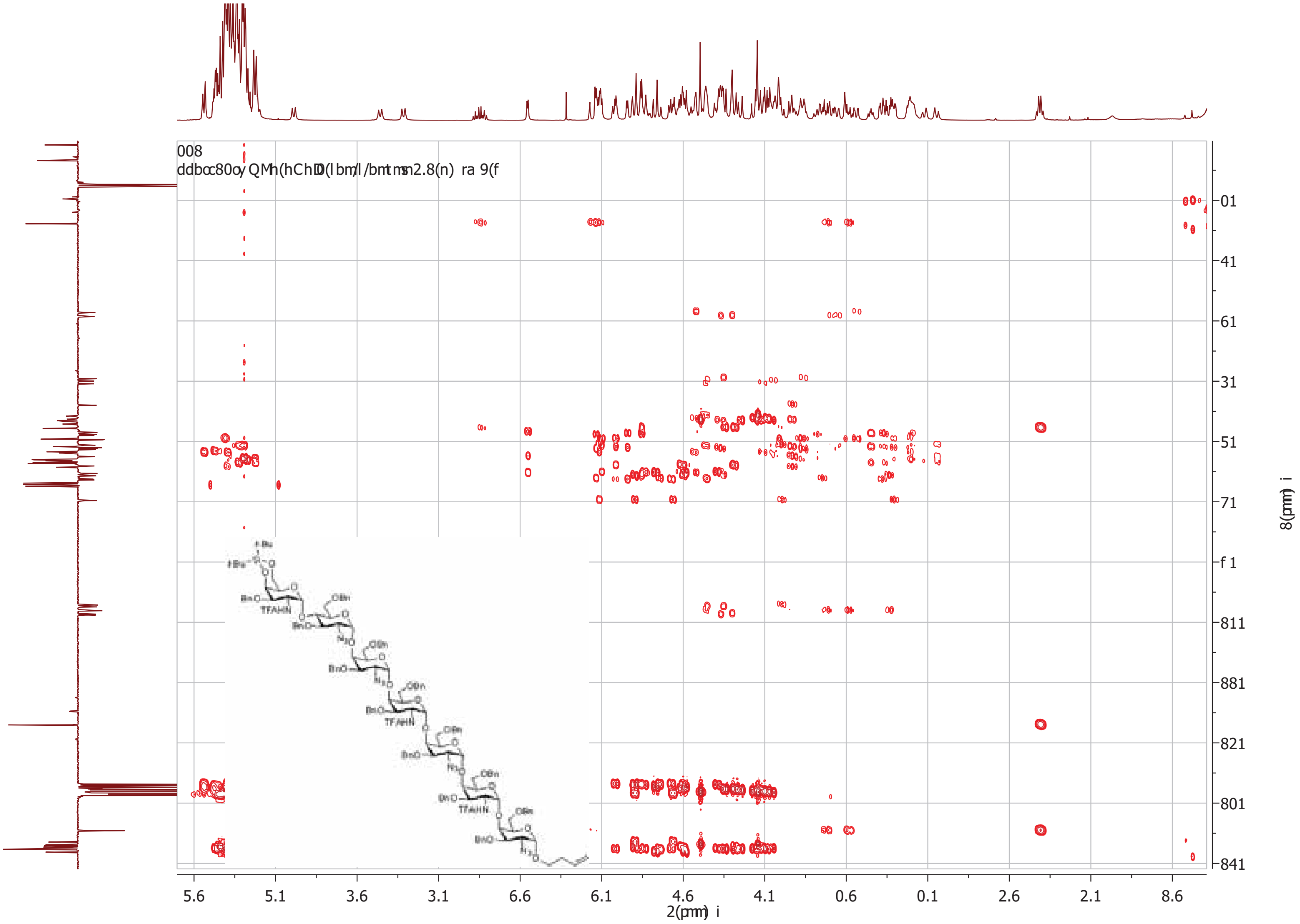
008
ddboc80oy QMh(hChD0(l bm/l /bmt ms2.8(n) ra 9(f
t-Bu
BnO
TFAHN
OBn
N3
2(pmm) i
8(pmm) i

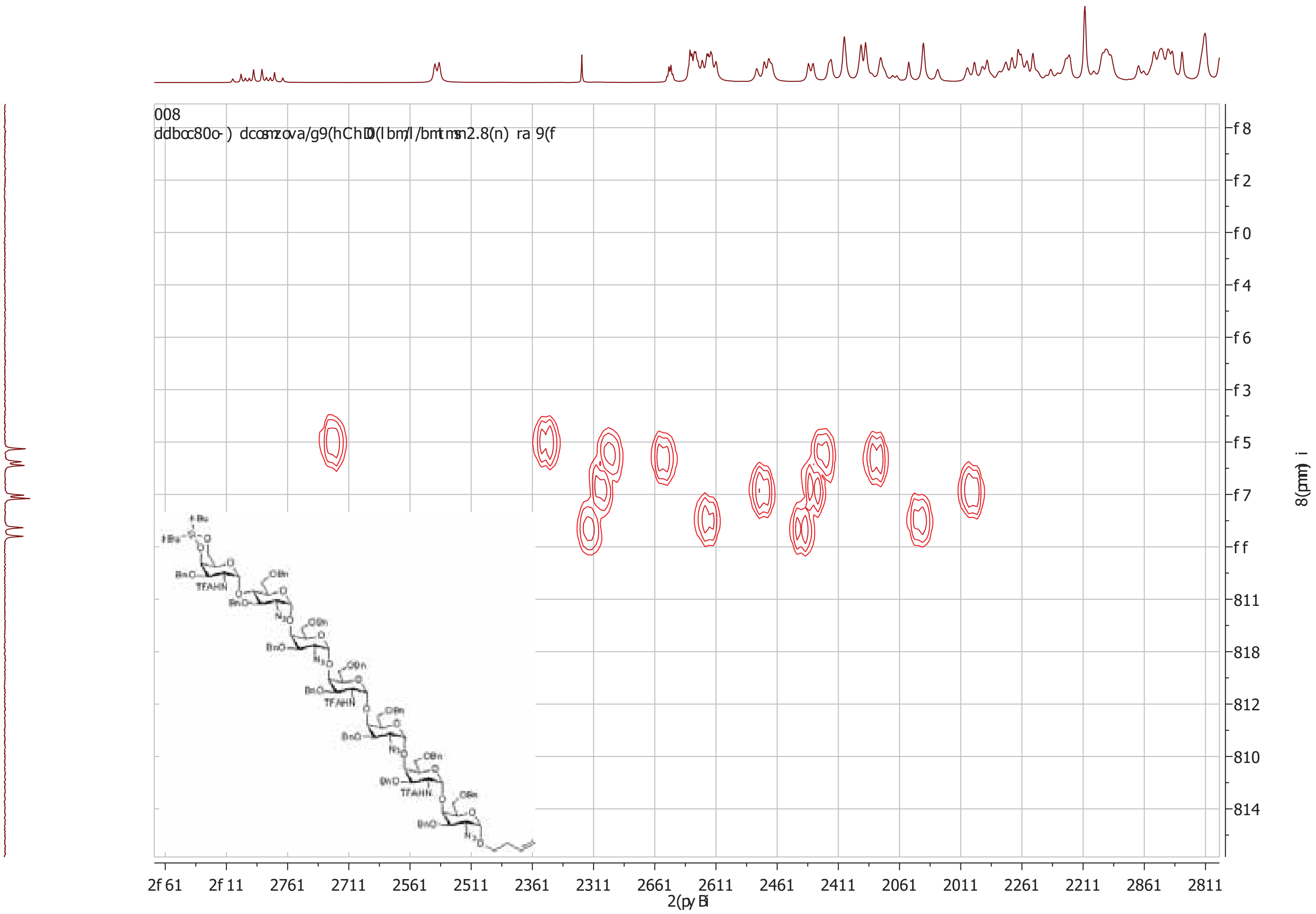

008
t-Bu
BnO
TFAHN
OBn
N3

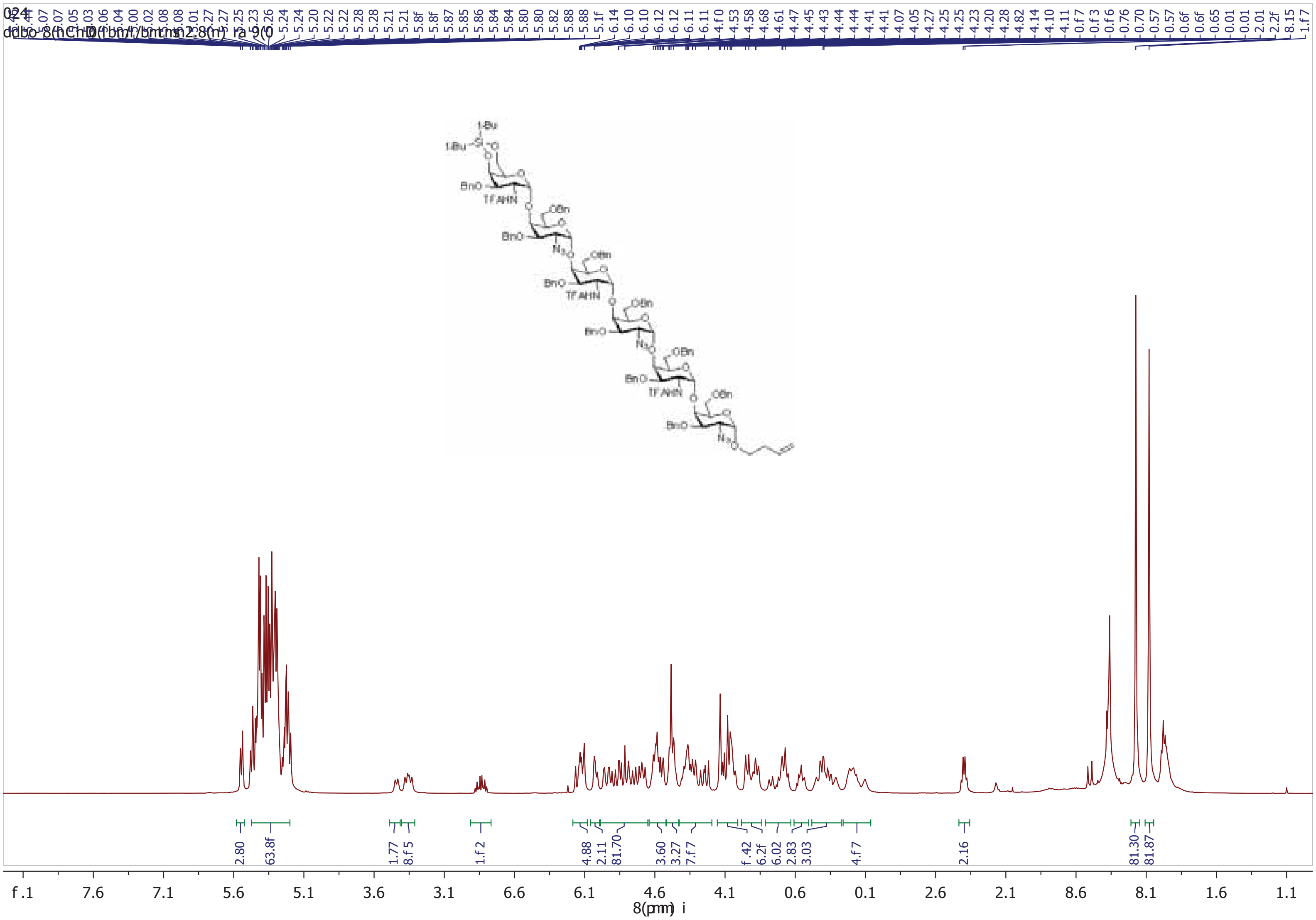

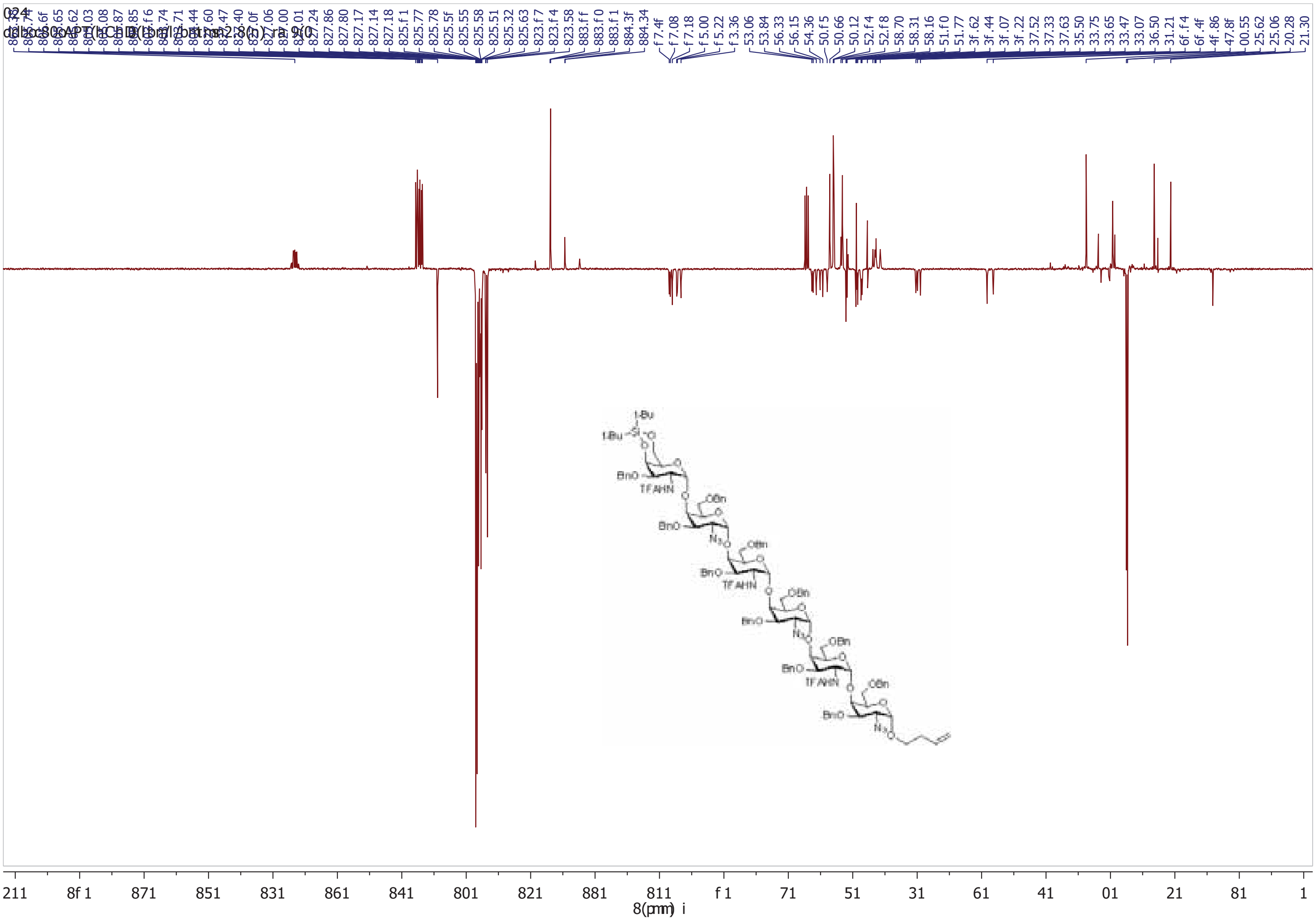

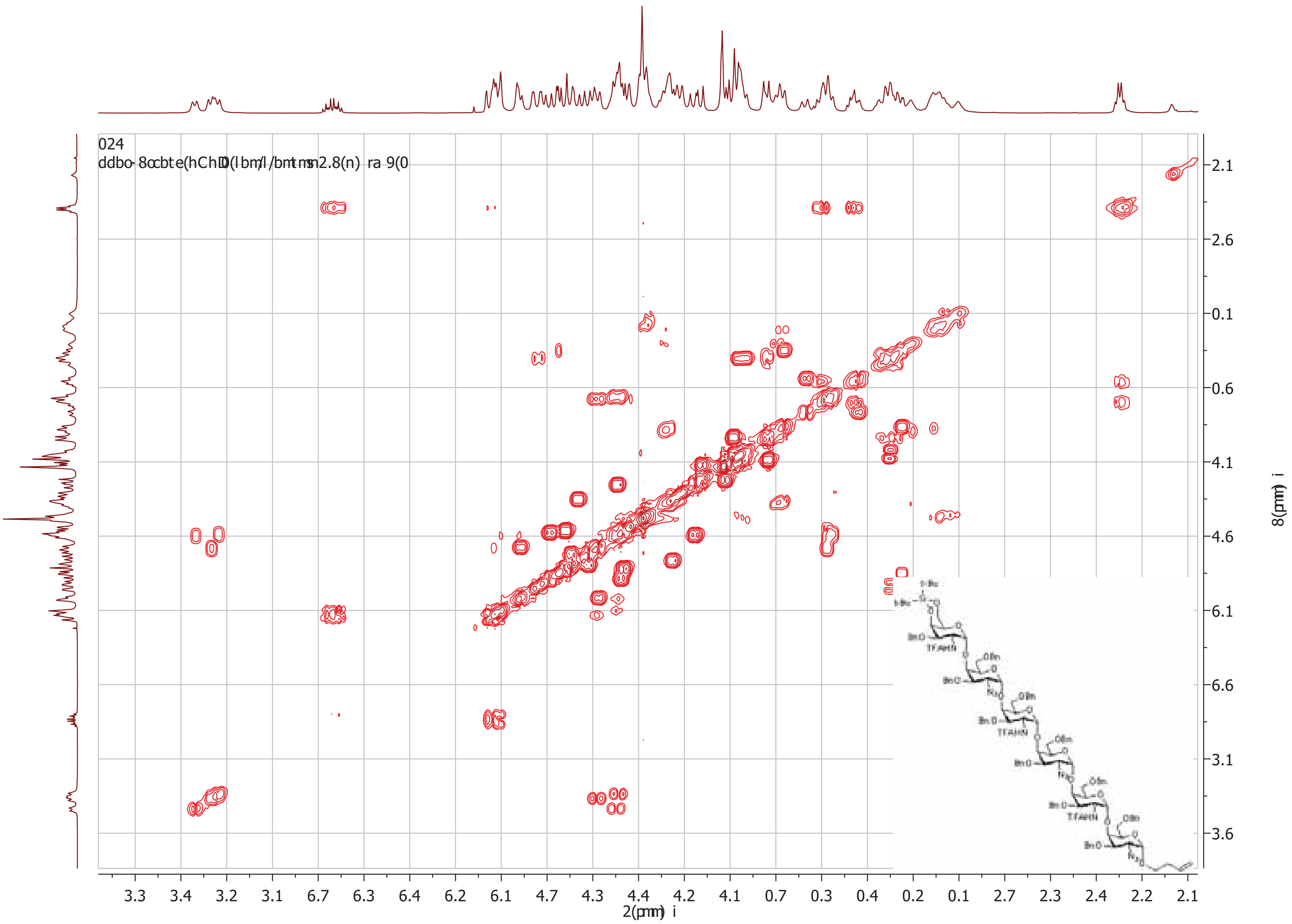

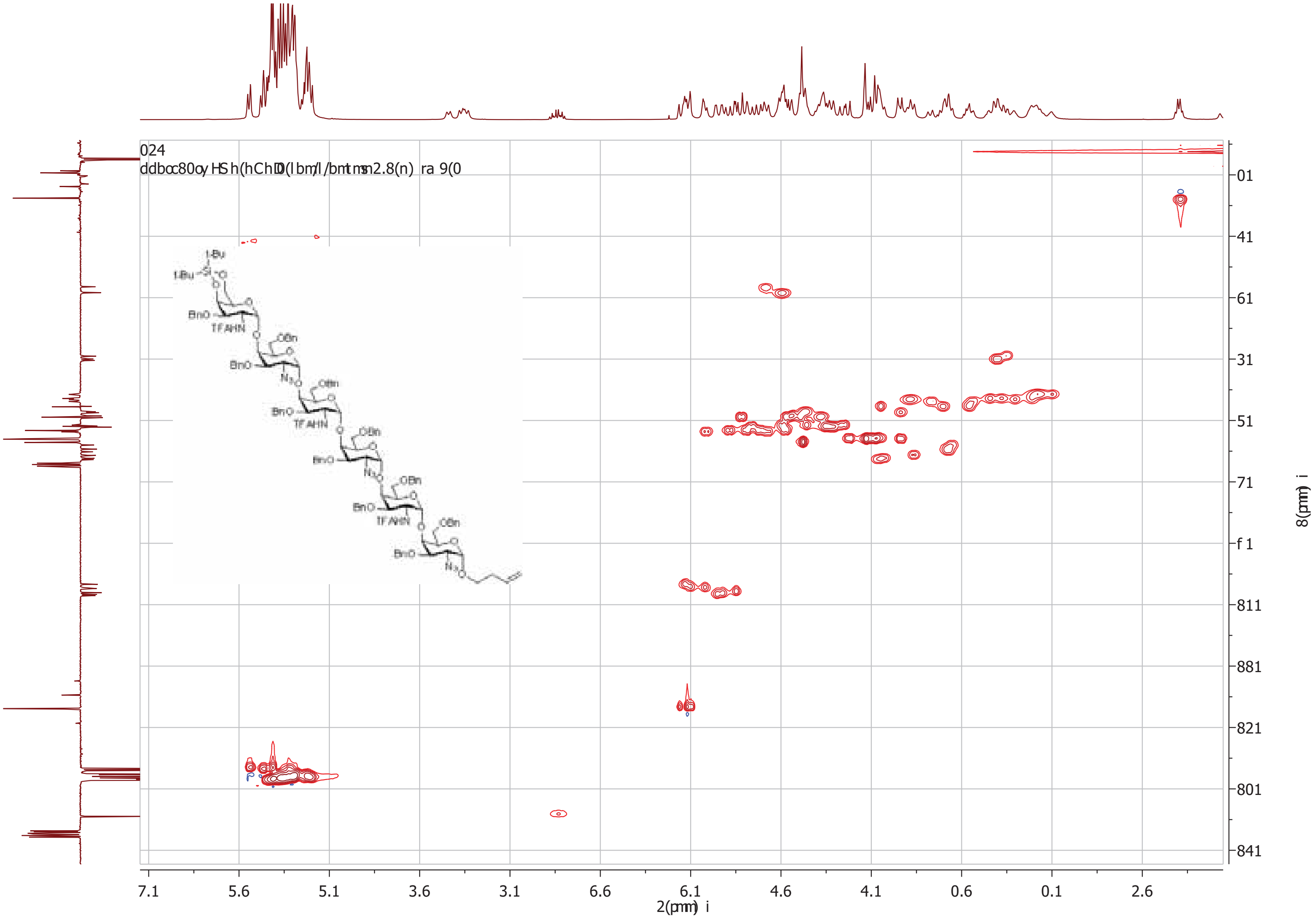

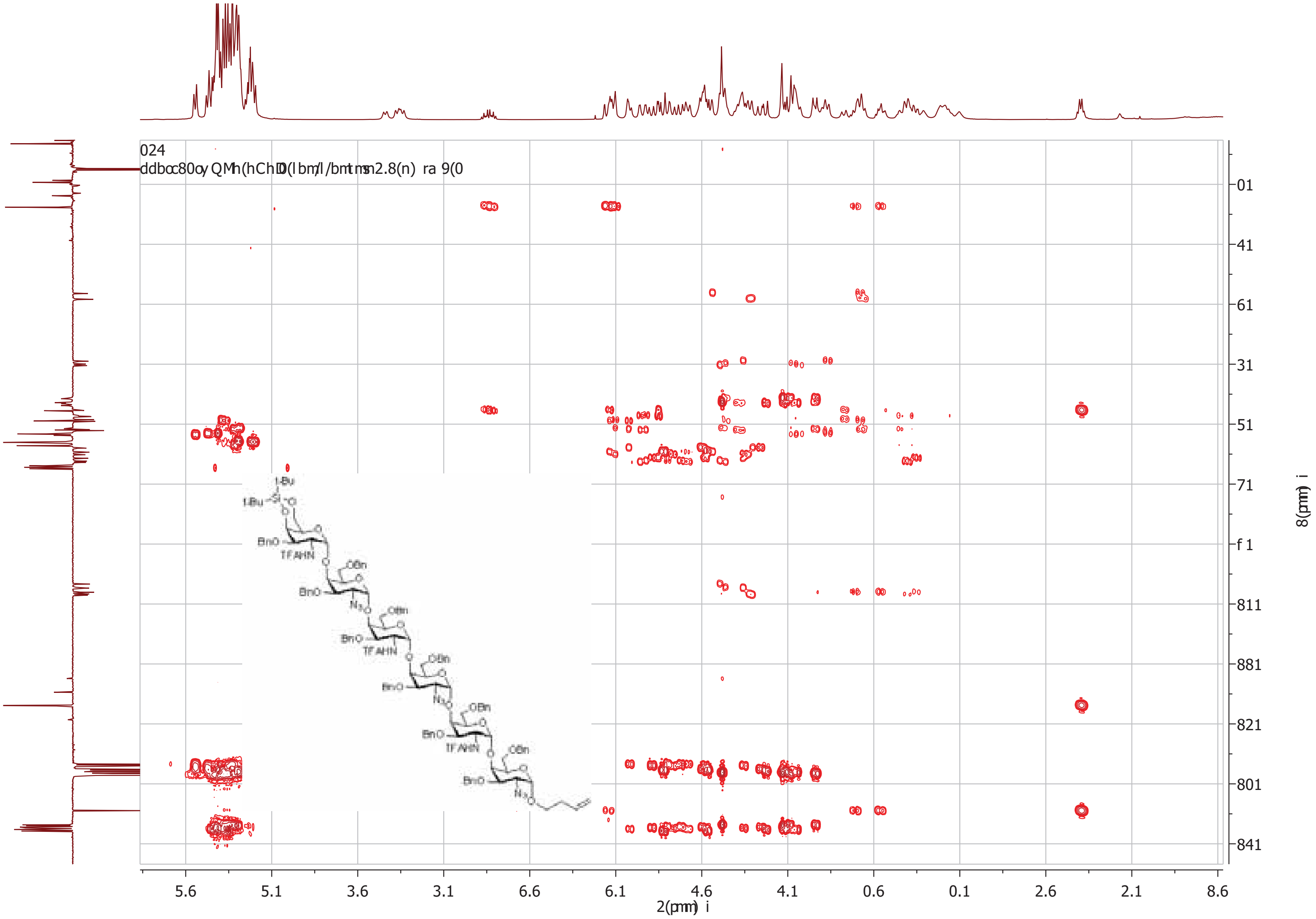
t-Bu
t-Bu
Si
BnO
TFAHN
OBn
BnO
N3
OBn
BnO
TFAHN
OBn
BnO
N3
OBn
BnO
TFAHN
OBn
BnO
N3

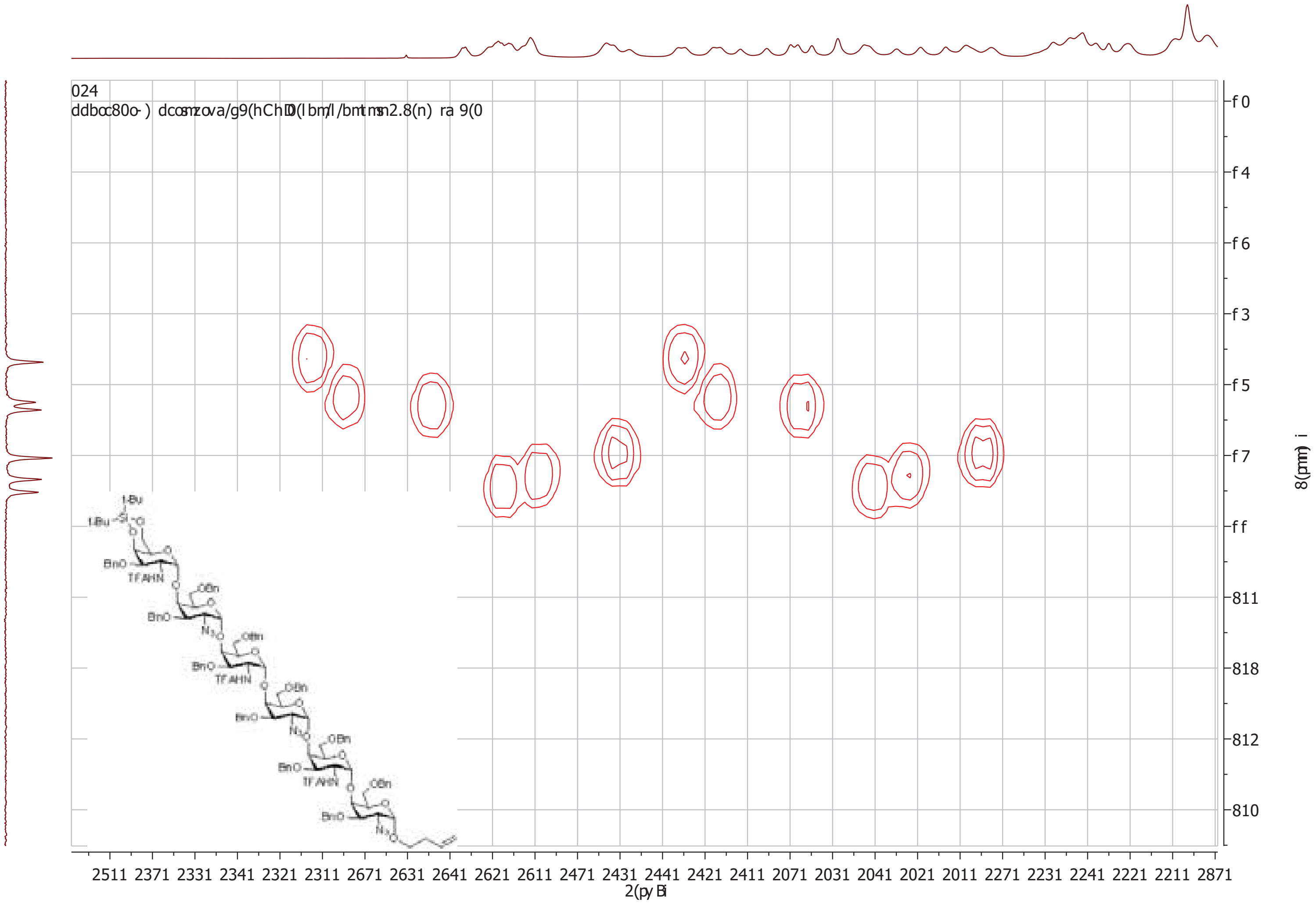
t-Bu
t-Bu
Si
BnO
TFAHN
OBn
N3
OBn
BnO
TFAHN
OBn
BnO
N3
OBn
BnO
TFAHN
OBn
BnO
N3

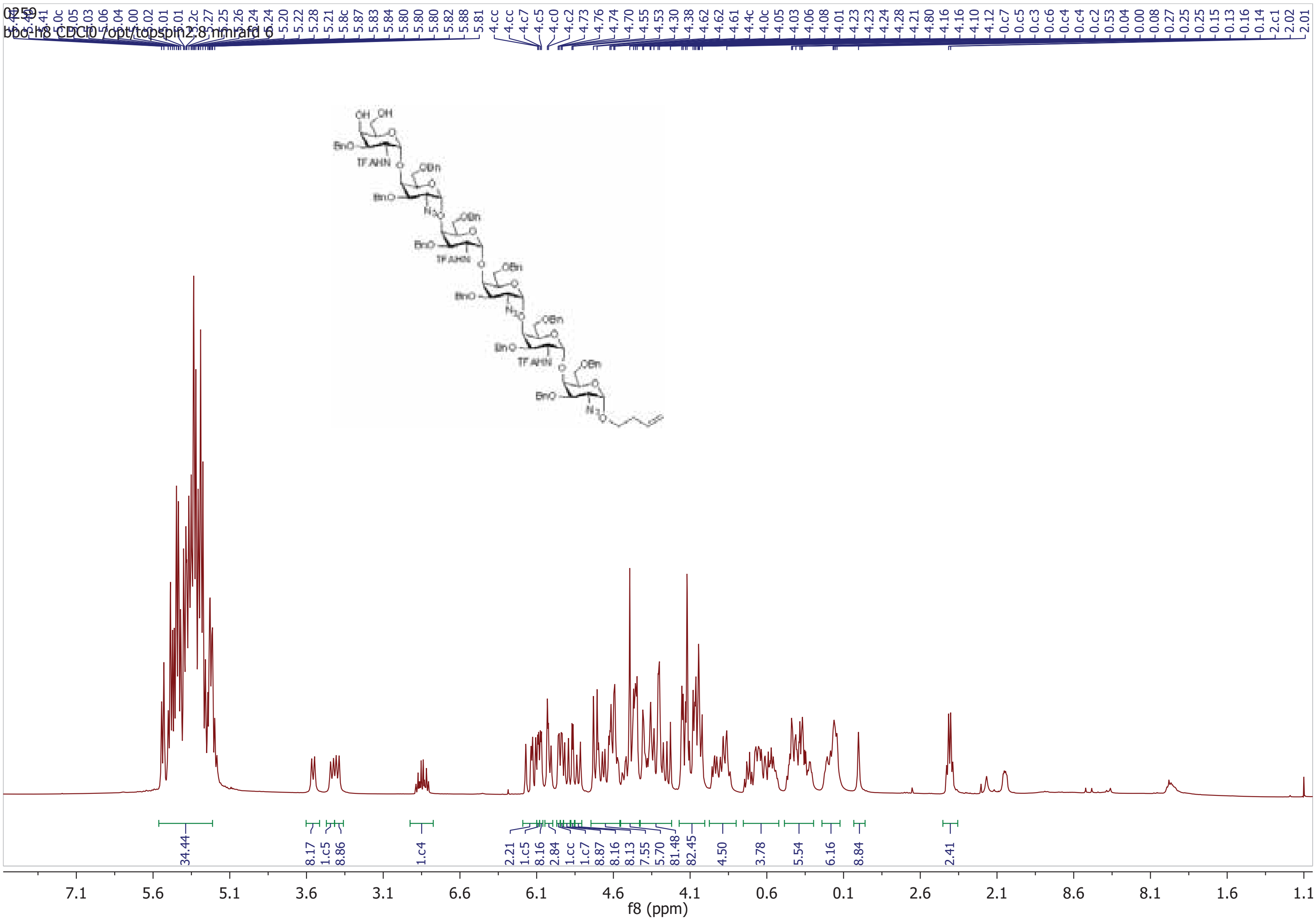
0259
bbo-h8 CDCl0 /opt/topspin2.8 nmrafd 6
7.1 5.6 5.1 3.6 3.1 6.6 6.1 4.6 4.1 0.6 0.1 2.6 2.1 8.6 8.1 1.6 1.1
f8 (ppm)

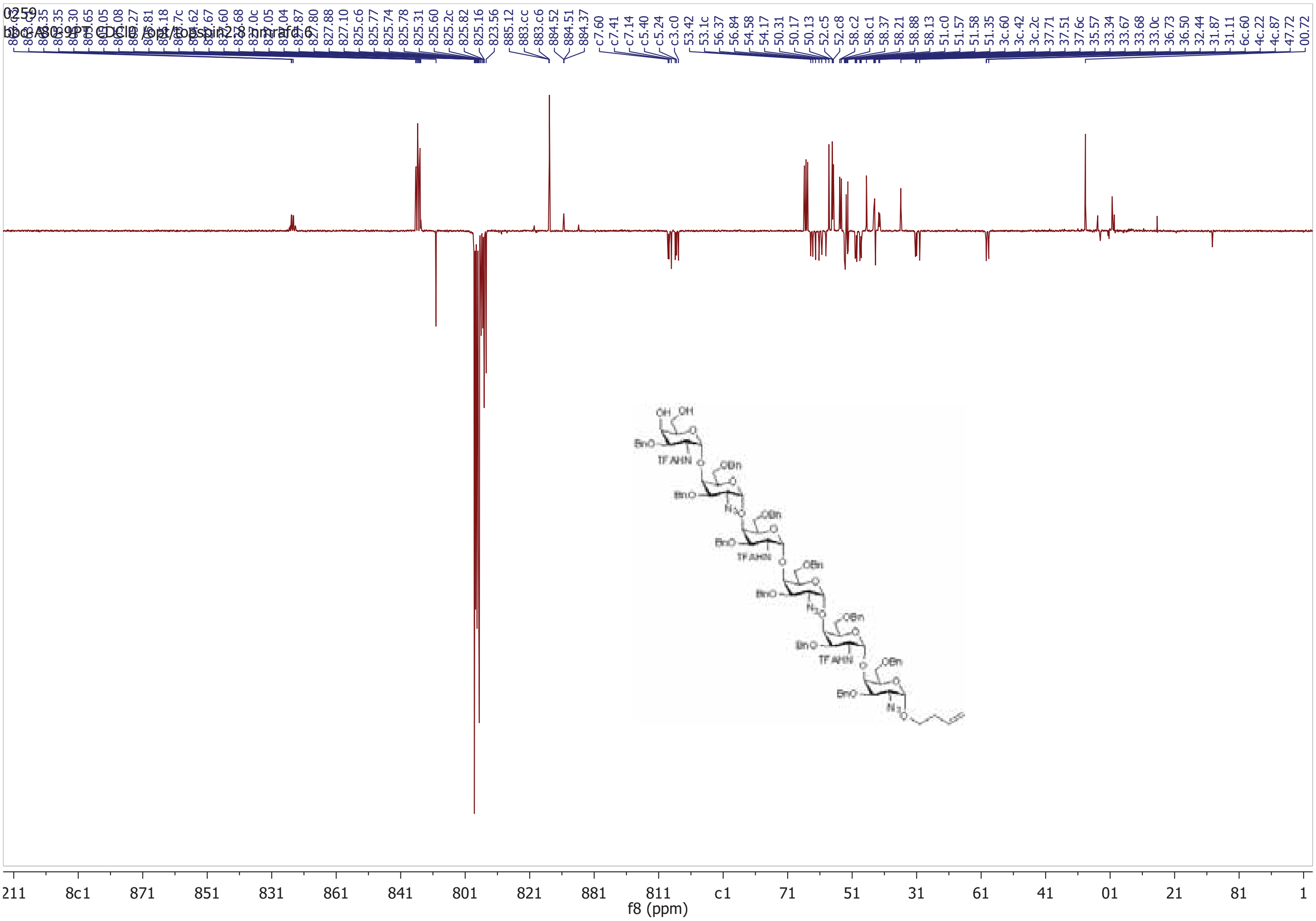
OH OH
BnO
TFAHN
OBn
N3
f8 (ppm)

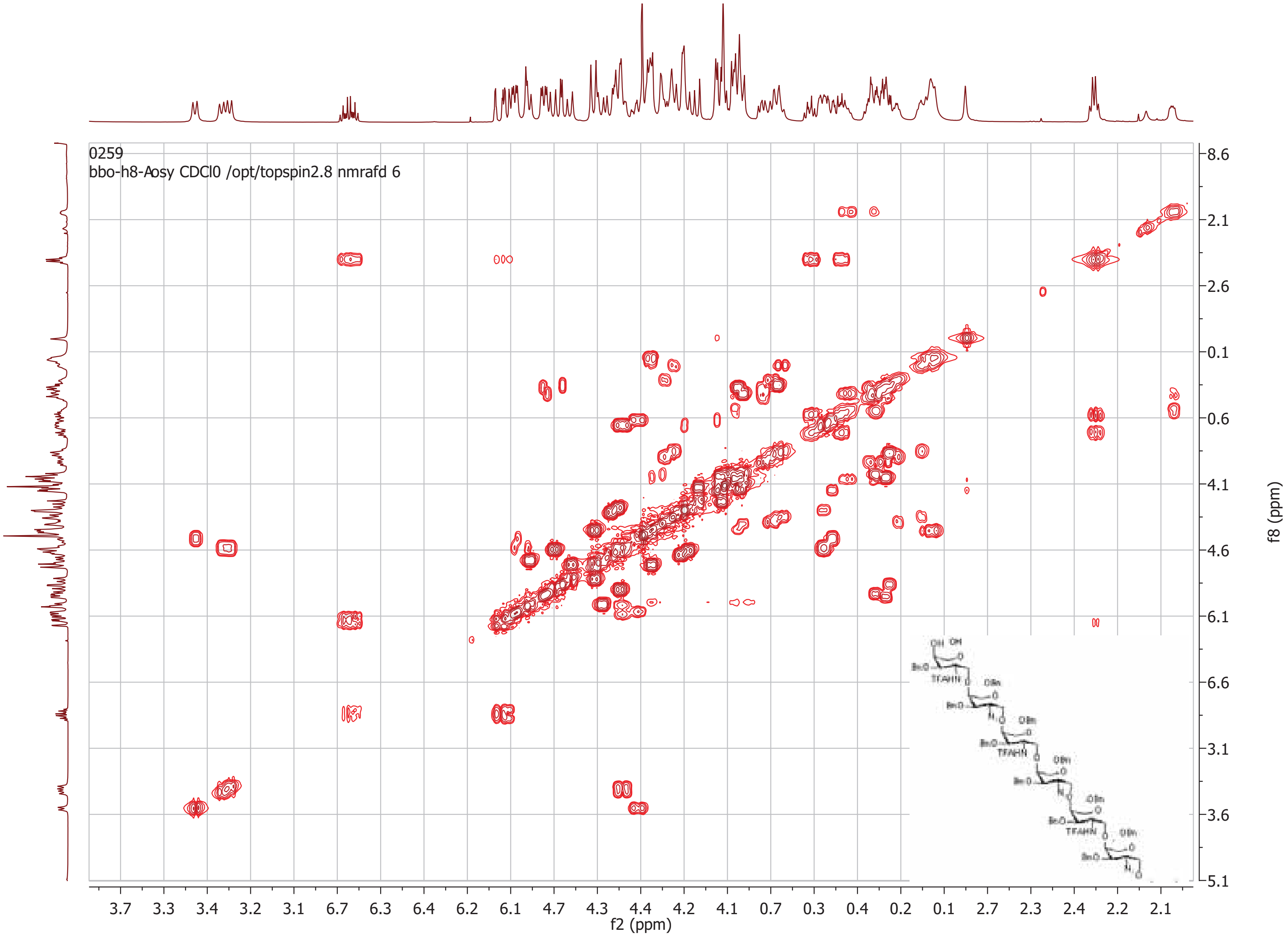
0259
bbo-h8-Aosy CDCl0 /opt/topspin2.8 nmrafd 6
f2 (ppm)
f8 (ppm)

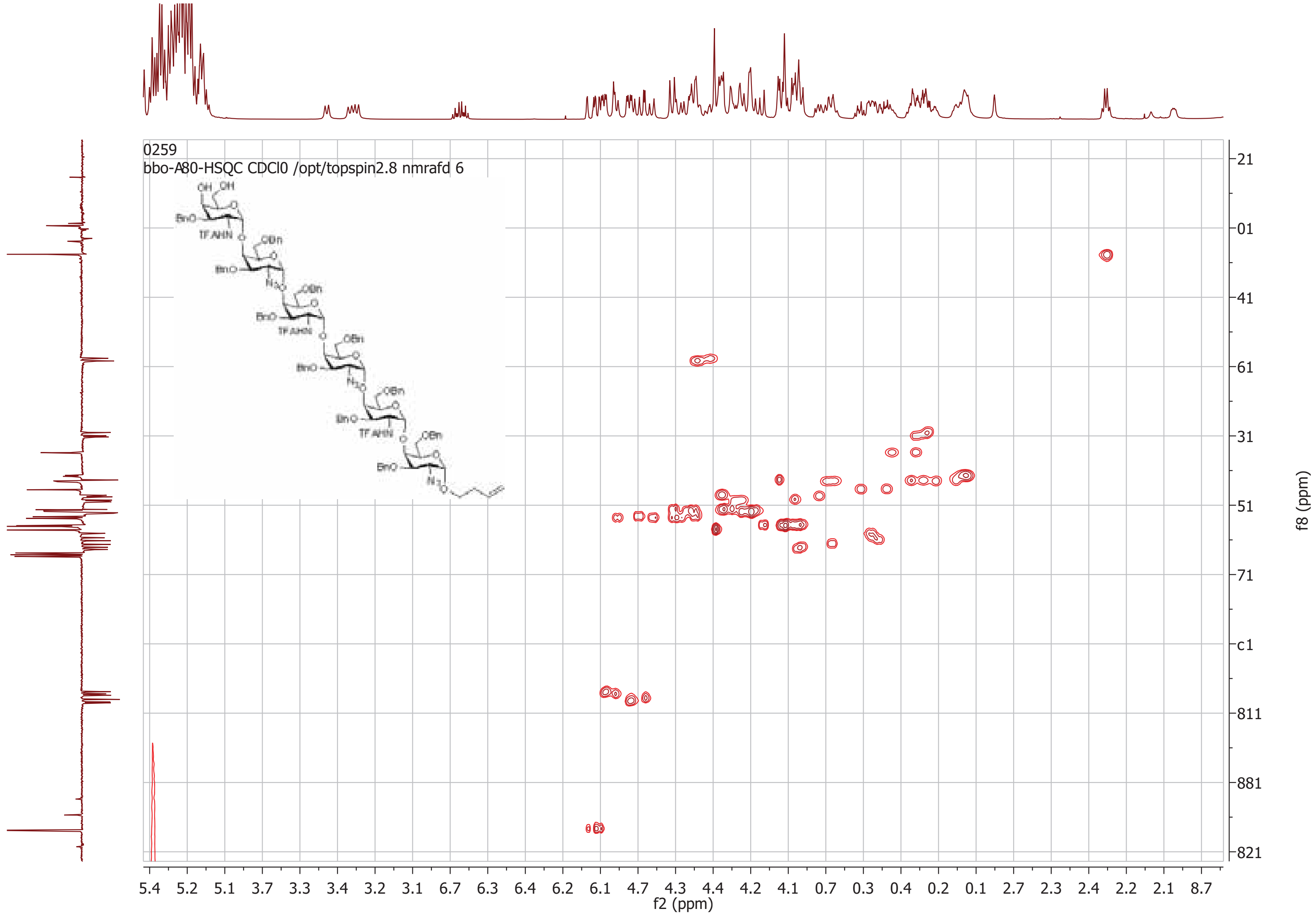
0259
bbo-A80-HSQC CDCl0 /opt/topspin2.8 nmrafd 6
f2 (ppm)
f8 (ppm)

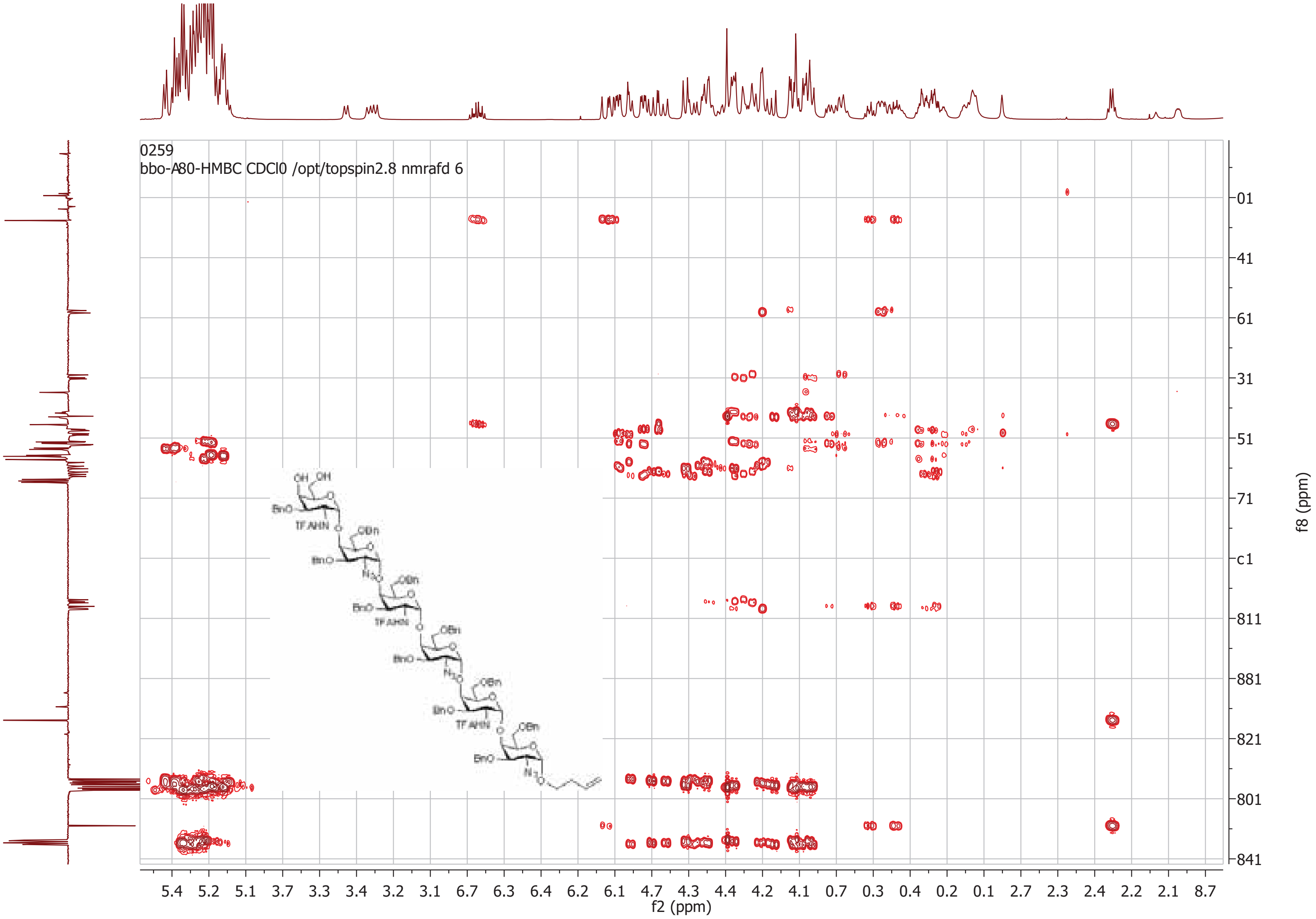
0259
bbo-A80-HMBC CDCl0 /opt/topspin2.8 nmrafd 6
f2 (ppm)
f8 (ppm)
OH OH
BnO
TFAHN
OBn
BnO
OBn
BnO
TFAHN
OBn
BnO
OBn
BnO
TFAHN
OBn
BnO

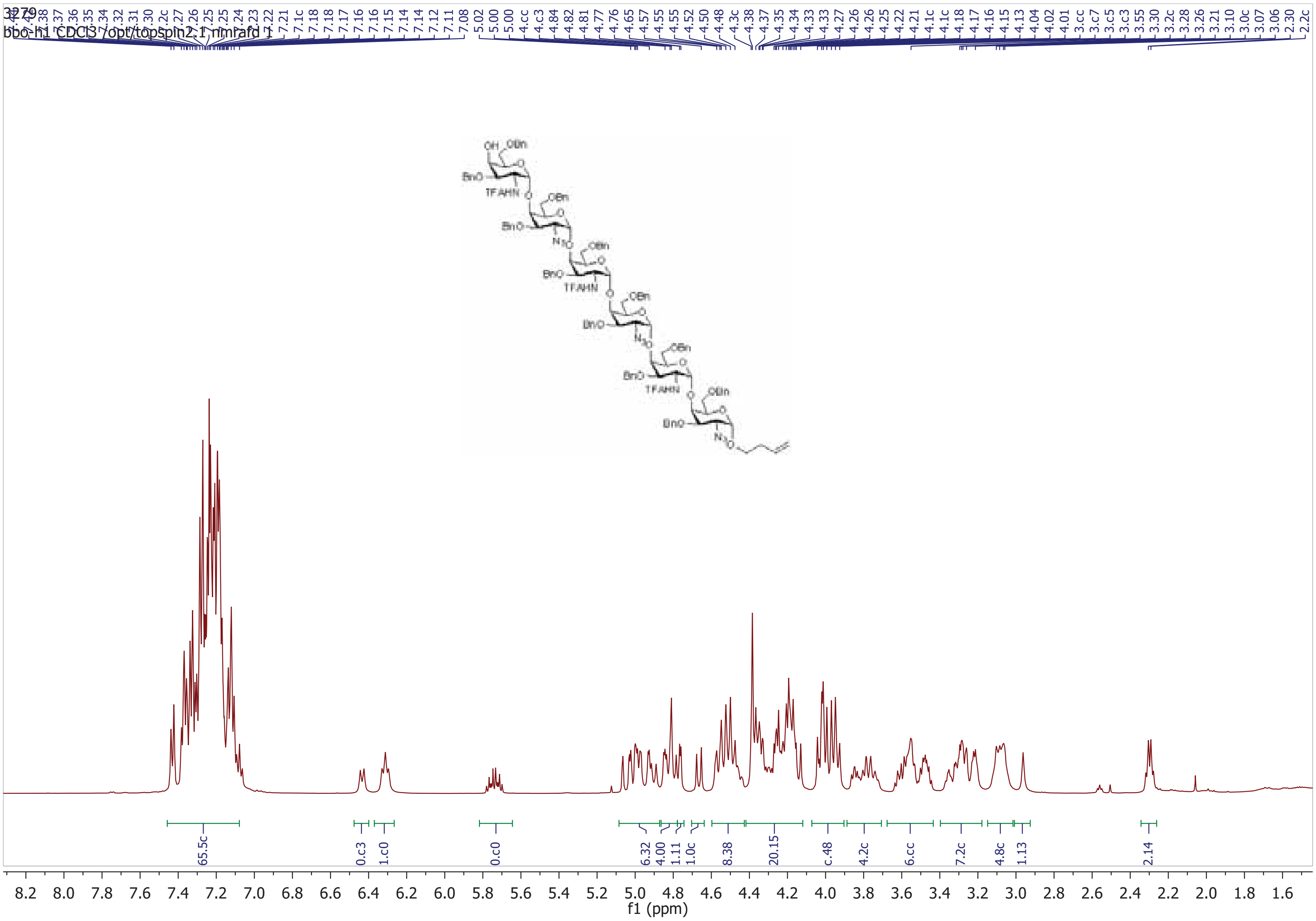

3279
bbo-h1 CDCl3 /opt/topspin2.1 nmrafd 1
OH
OBn
BnO
TFAHN
N3
f1 (ppm)

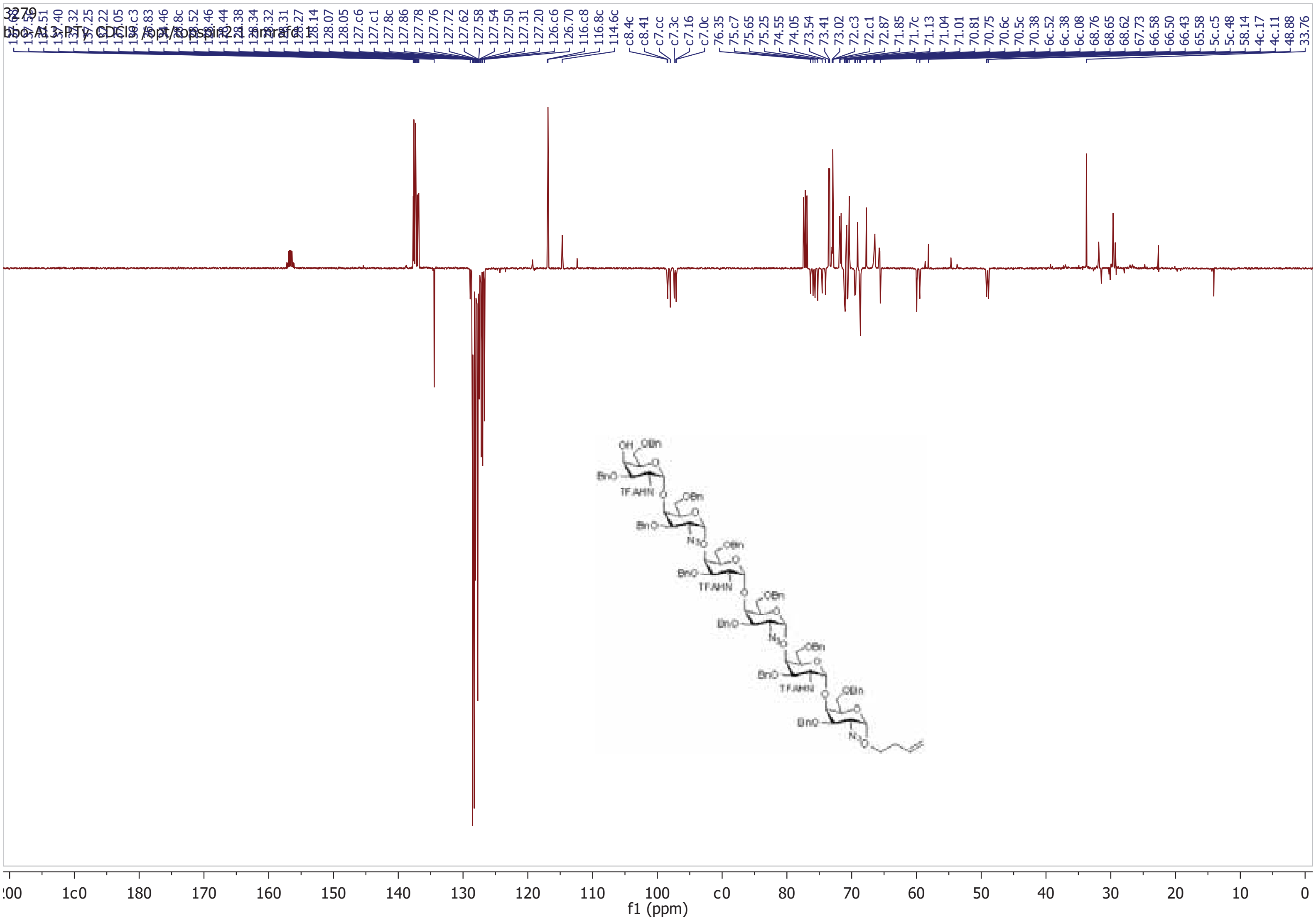

3279
f1 (ppm)
200 1c0 180 170 160 150 140 130 120 110 100 c0 80 70 60 50 40 30 20 10 0
OH
OBn
BnO
TFAHN
N3

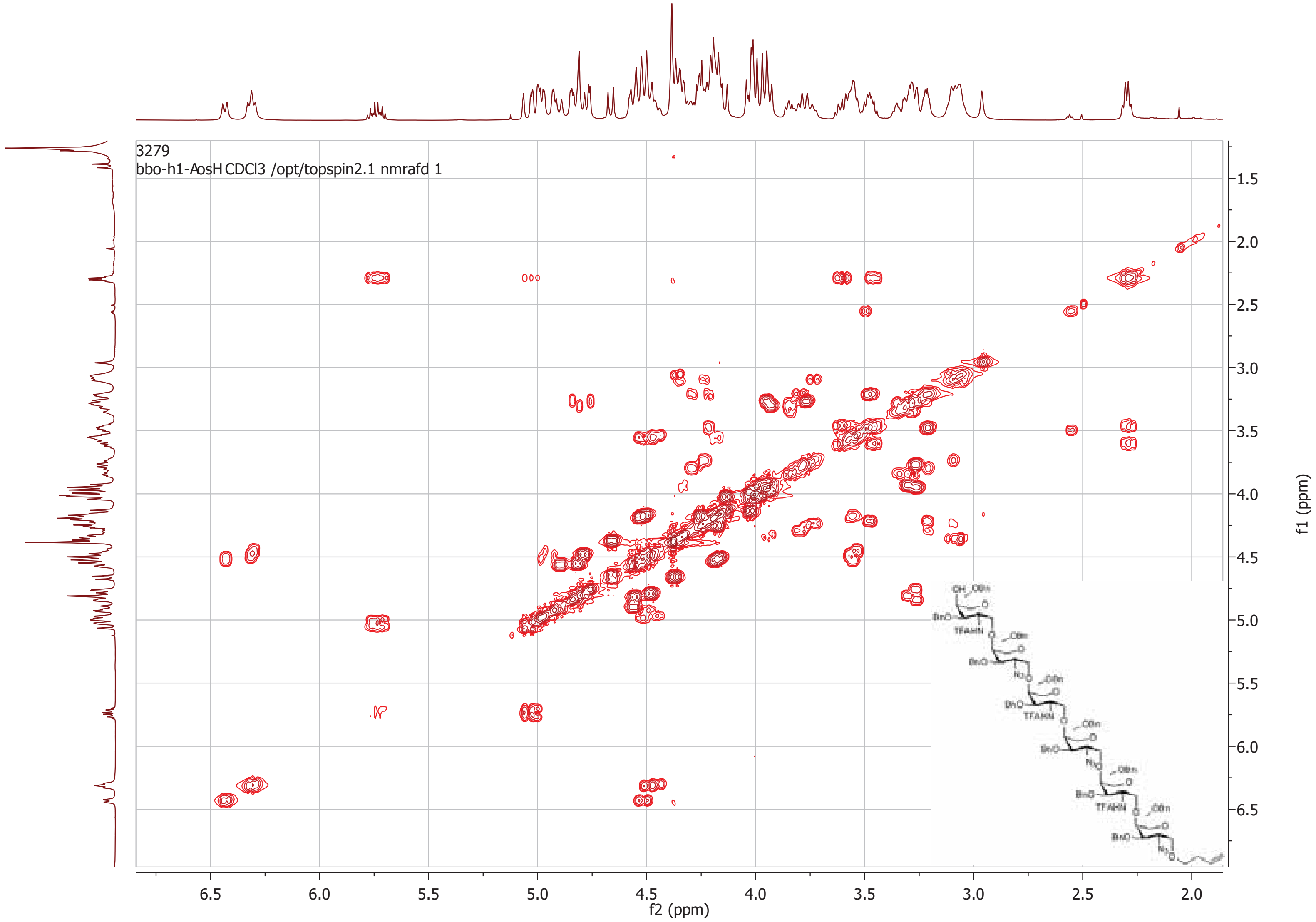
3279
bbo-h1-AosH CDCl3 /opt/topspin2.1 nmrafd 1
f2 (ppm)
f1 (ppm)
6.5
6.0
5.5
5.0
4.5
4.0
3.5
3.0
2.5
2.0
1.5
OH
OBn
BnO
TFAHN
N3
O

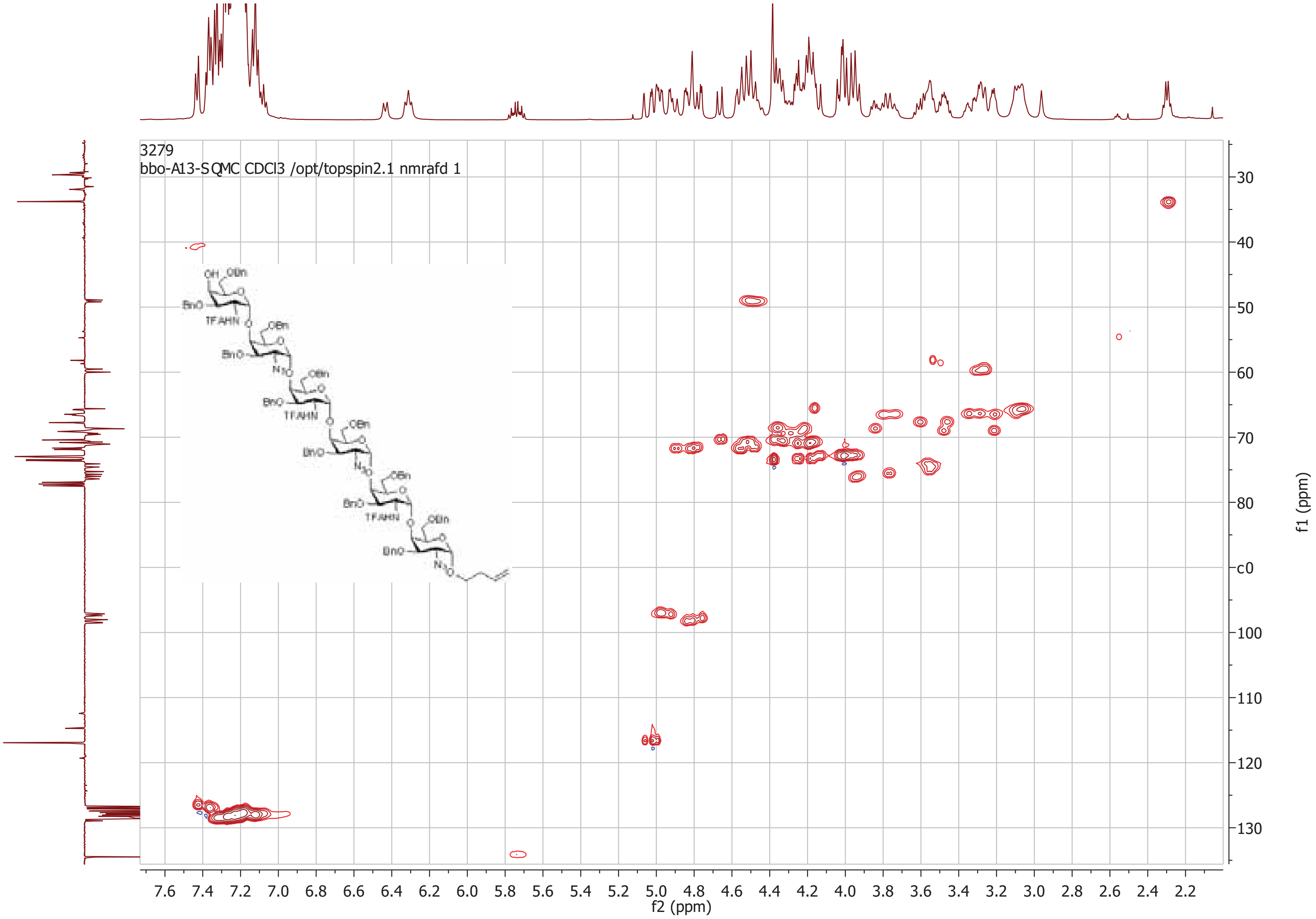
3279
bbo-A13-SQMC CDCl3 /opt/topspin2.1 nmrafd 1
f2 (ppm)
f1 (ppm)
OH
OBn
BnO
TFAHN
N3

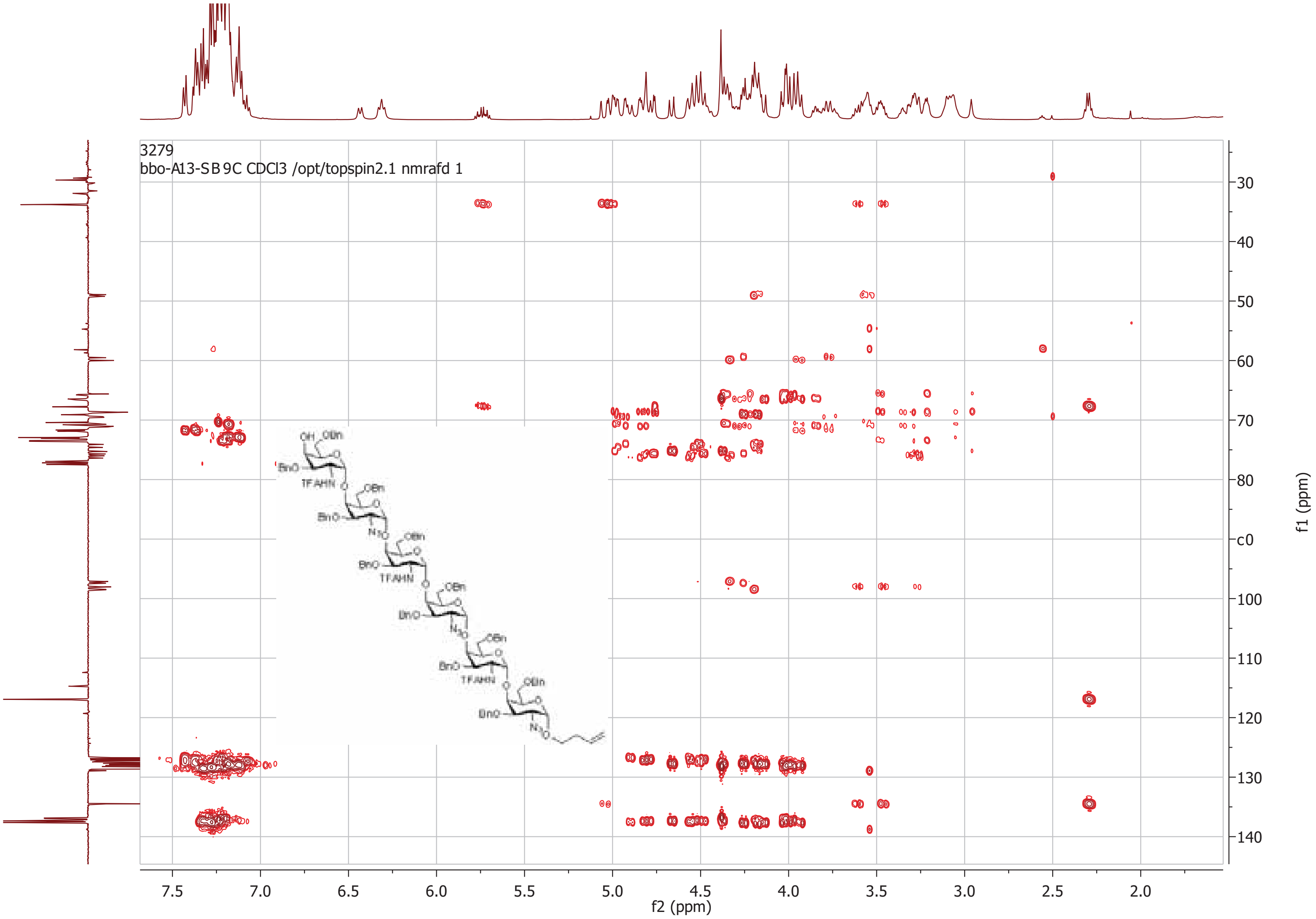
3279
bbo-A13-SB 9C CDCl3 /opt/topspin2.1 nmrafd 1
f2 (ppm)
f1 (ppm)
OH
OBn
BnO
TFAHN
N3

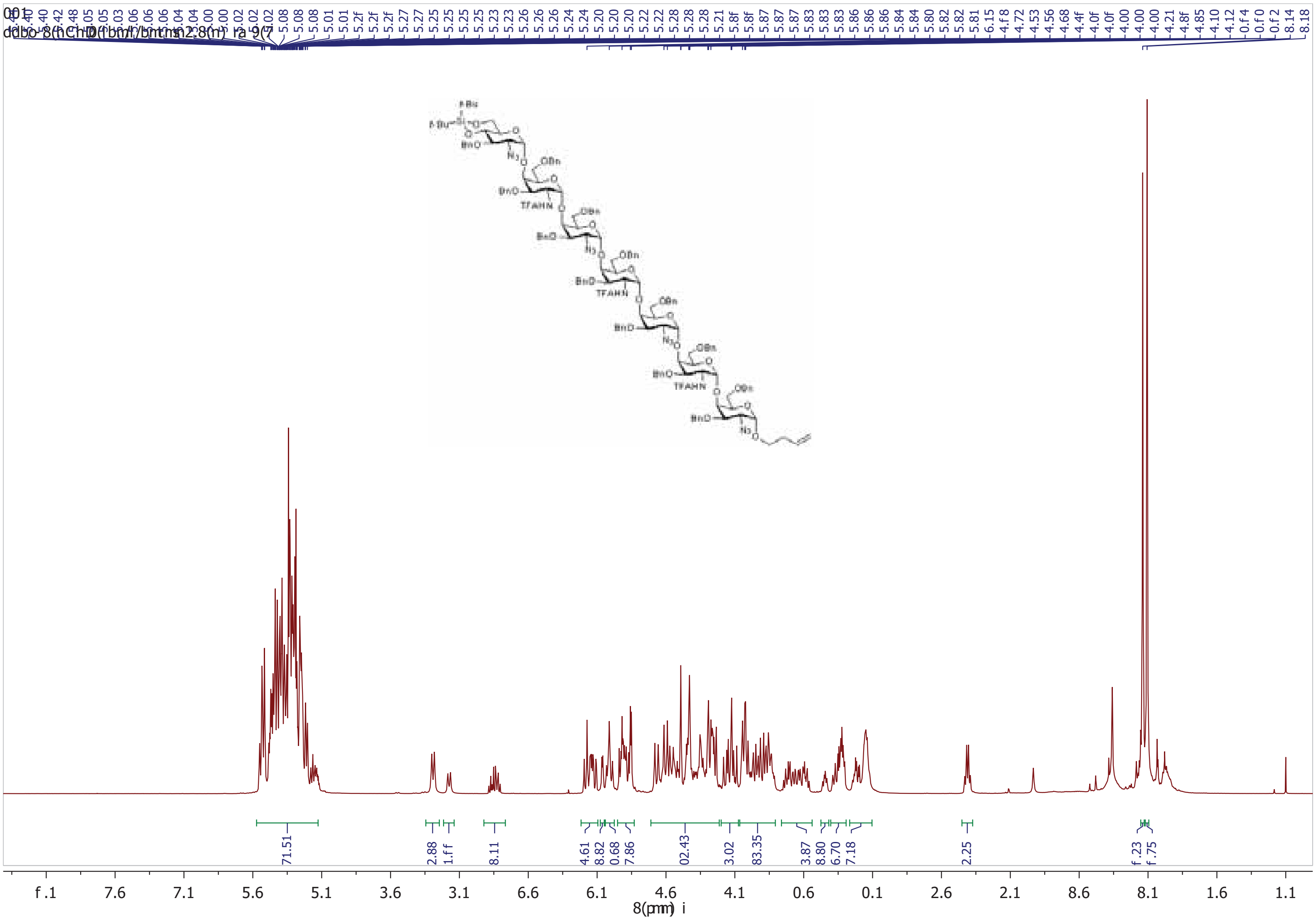

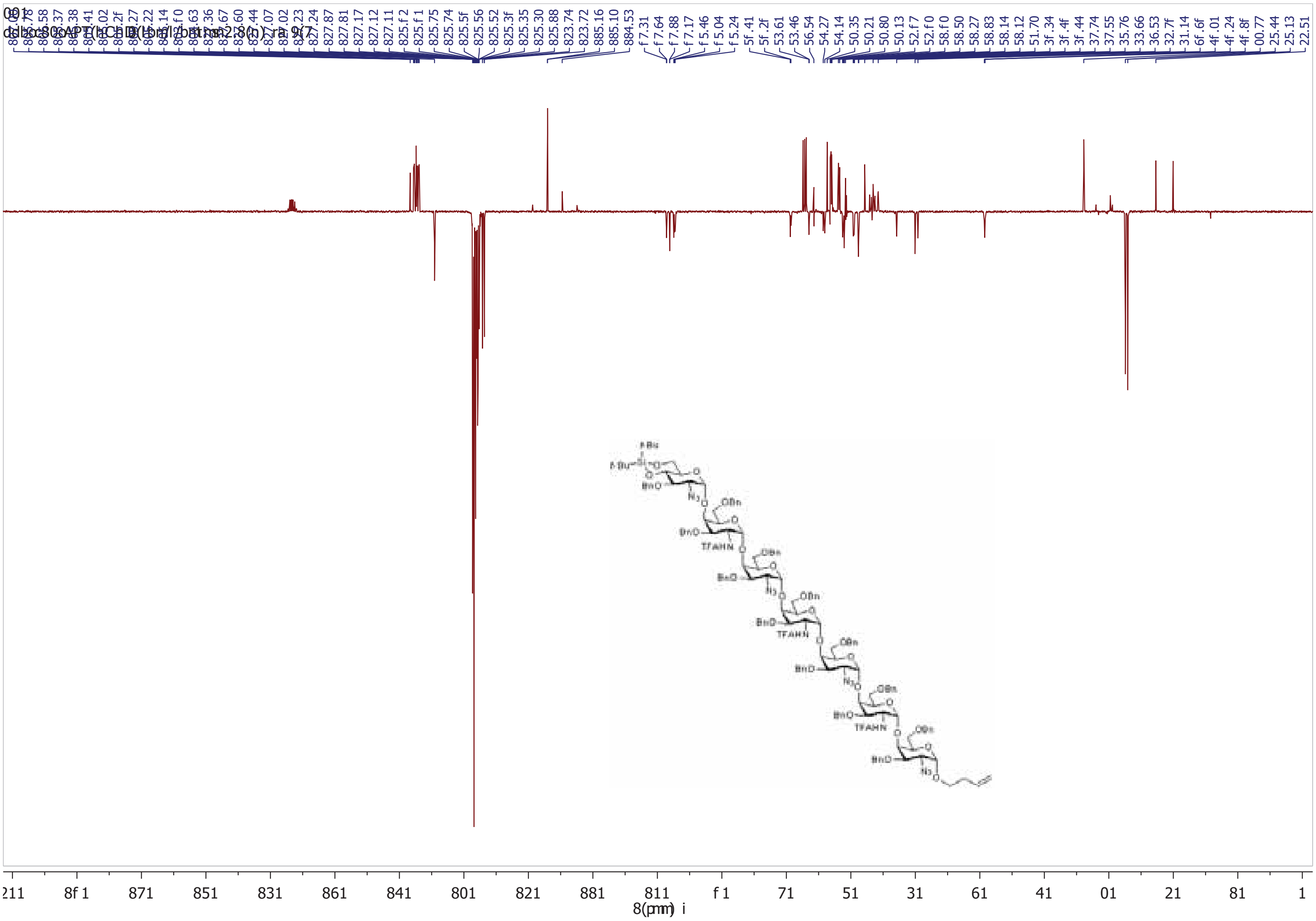

OBn
BnO
TFAHN
N3

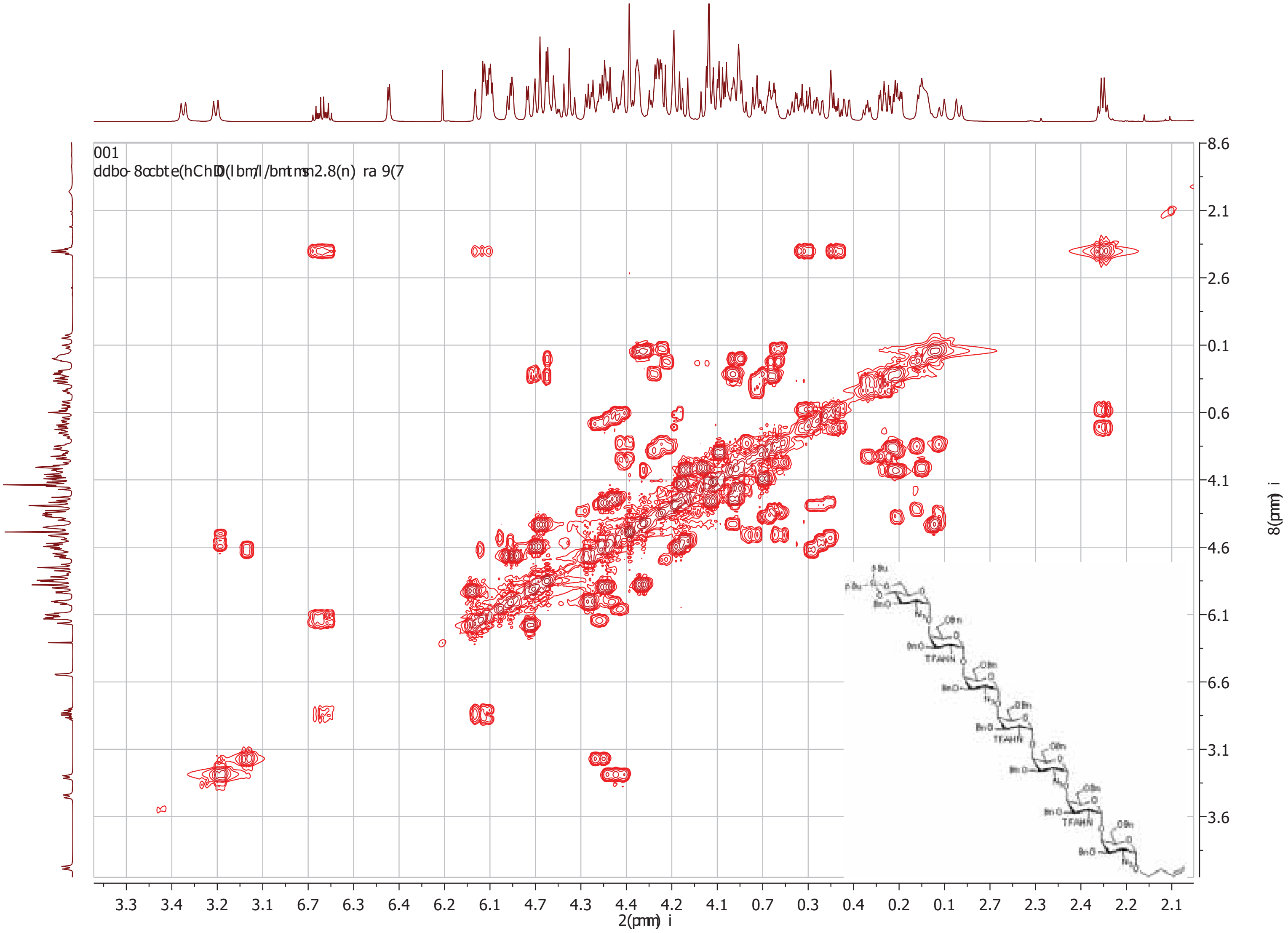

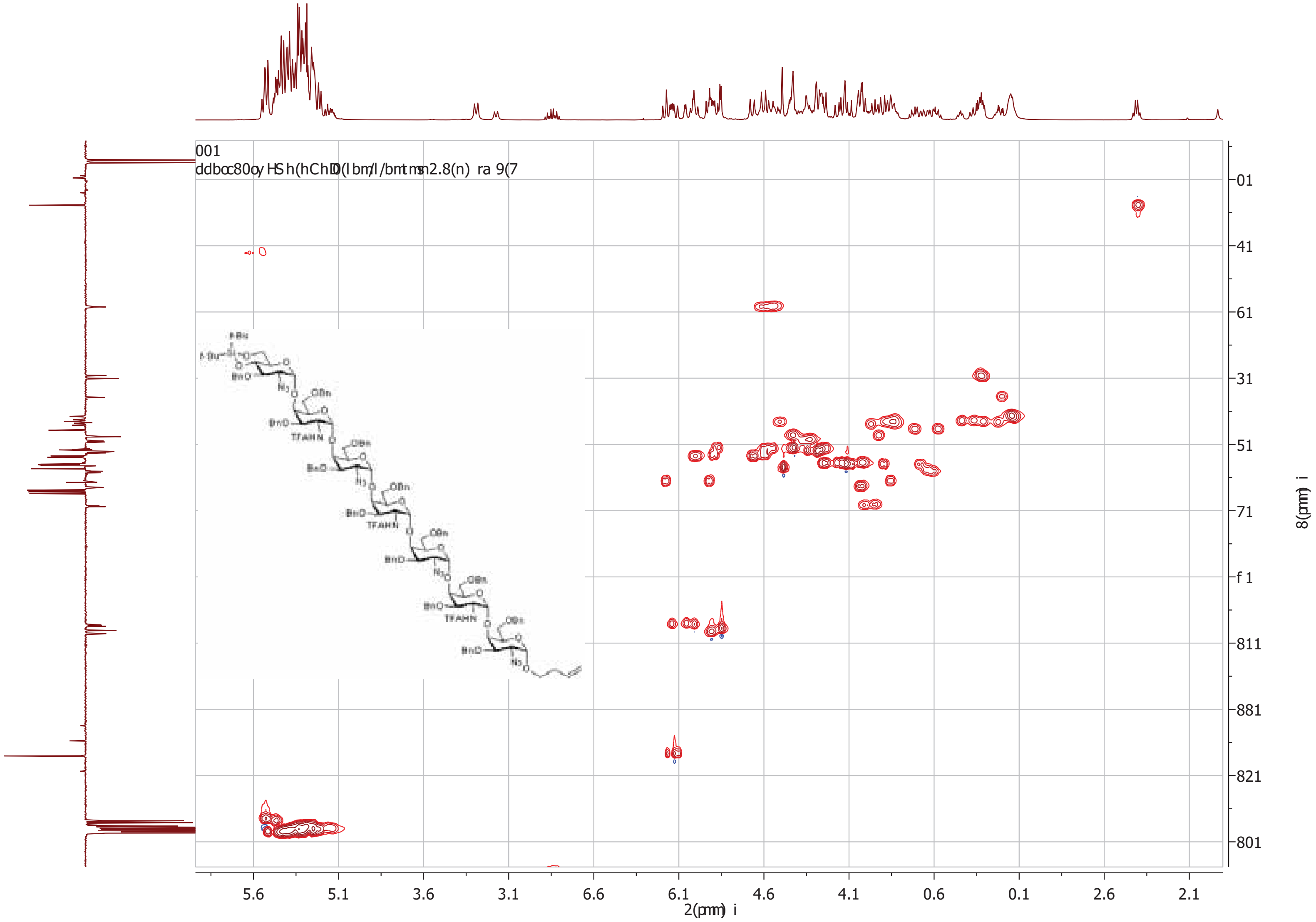

001
ddbcc80oy HS h(hChD(l bm/l /bmt ms2.8(n) ra 9(7
t-Bu
BnO
N3
OBn
TFAHN
2(pmm) i
8(pmm) i

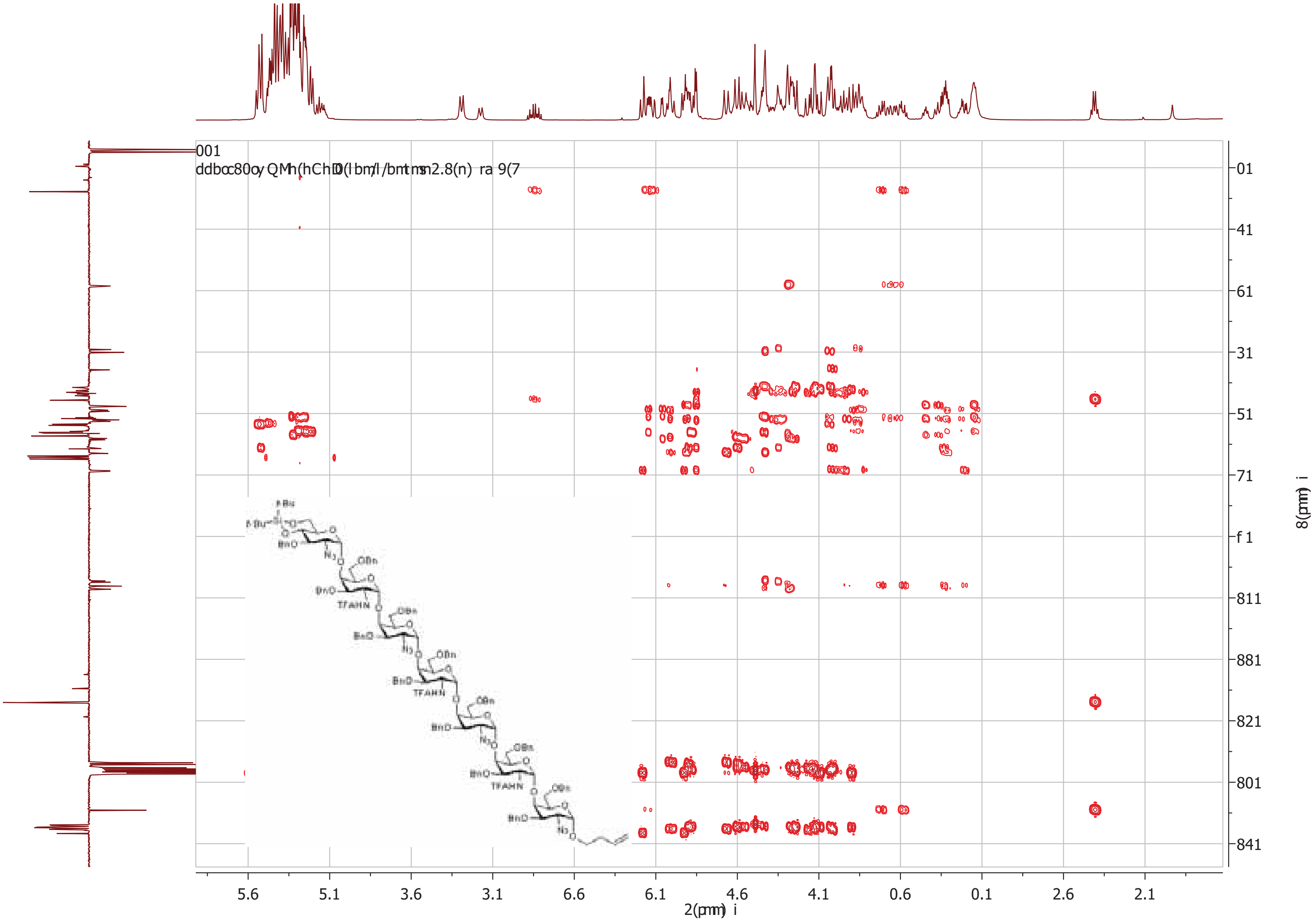
t-Bu
t-Bu
Si
BnO
N3
OBn
TFAHN

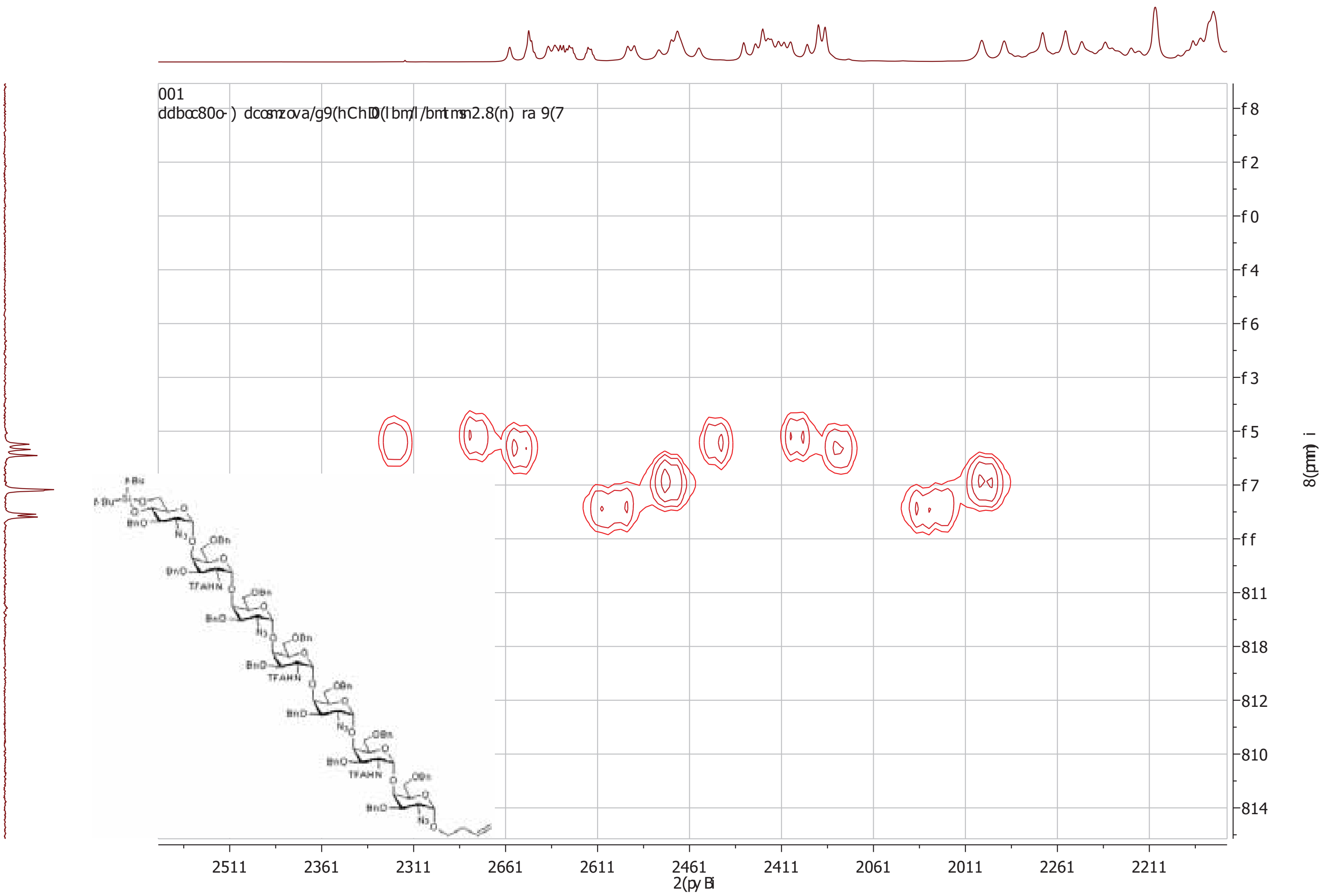

001
2511
2361
2311
2661
2611
2461
2411
2061
2011
2261
2211
811
818
812
810
814
OBn
BnO
TFAHN

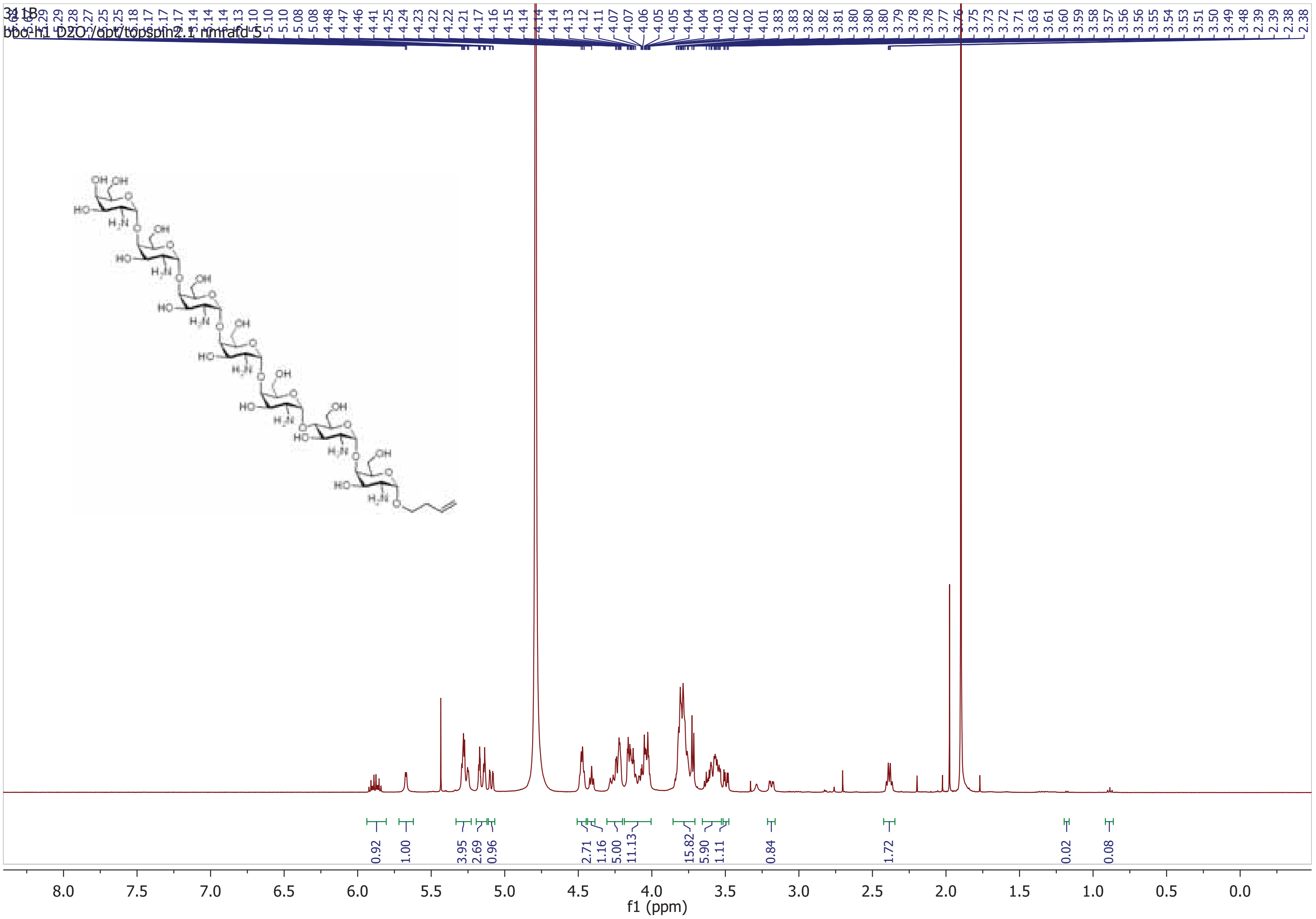
311B
bbo h1 D2O /opt/topspin2.1 nmrafd 5
5.29 5.28 5.27 5.25 5.25 5.18 5.17 5.17 5.17 5.14 5.14 5.14 5.13 5.10 5.10 5.08 5.08 4.48 4.47 4.46 4.41 4.25 4.24 4.23 4.22 4.22 4.21 4.17 4.16 4.15 4.14 4.14 4.14 4.13 4.12 4.11 4.07 4.07 4.06 4.05 4.05 4.04 4.04 4.03 4.02 4.02 4.01 3.83 3.83 3.82 3.82 3.81 3.80 3.80 3.80 3.79 3.78 3.78 3.77 3.76 3.75 3.73 3.72 3.71 3.63 3.61 3.60 3.59 3.58 3.57 3.56 3.55 3.54 3.53 3.51 3.50 3.49 3.48 2.39 2.39 2.38 2.38
0.92 1.00 3.95 2.69 0.96 2.71 1.16 5.00 11.13 15.82 5.90 1.11 0.84 1.72 0.02 0.08
8.0 7.5 7.0 6.5 6.0 5.5 5.0 4.5 4.0 3.5 3.0 2.5 2.0 1.5 1.0 0.5 0.0
f1 (ppm)
HO
OH
H2N
O

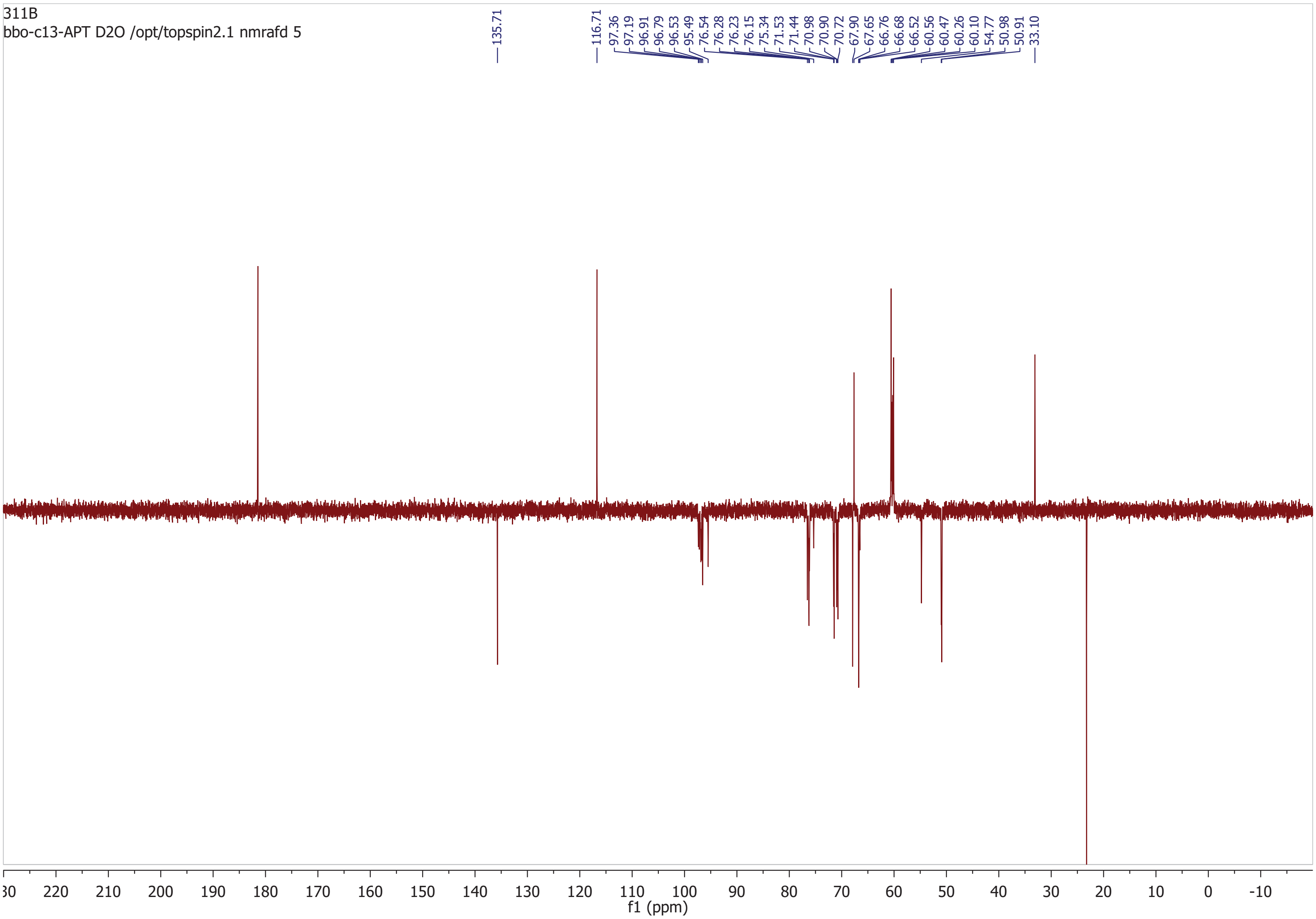
311B
bbo-c13-APT D2O /opt/topspin2.1 nmrafd 5
135.71
116.71
97.36
97.19
96.91
96.79
96.53
95.49
76.54
76.28
76.23
76.15
75.34
71.53
71.44
70.98
70.90
70.72
67.90
67.65
66.76
66.68
66.52
60.56
60.47
60.26
60.10
54.77
50.98
50.91
33.10
f1 (ppm)

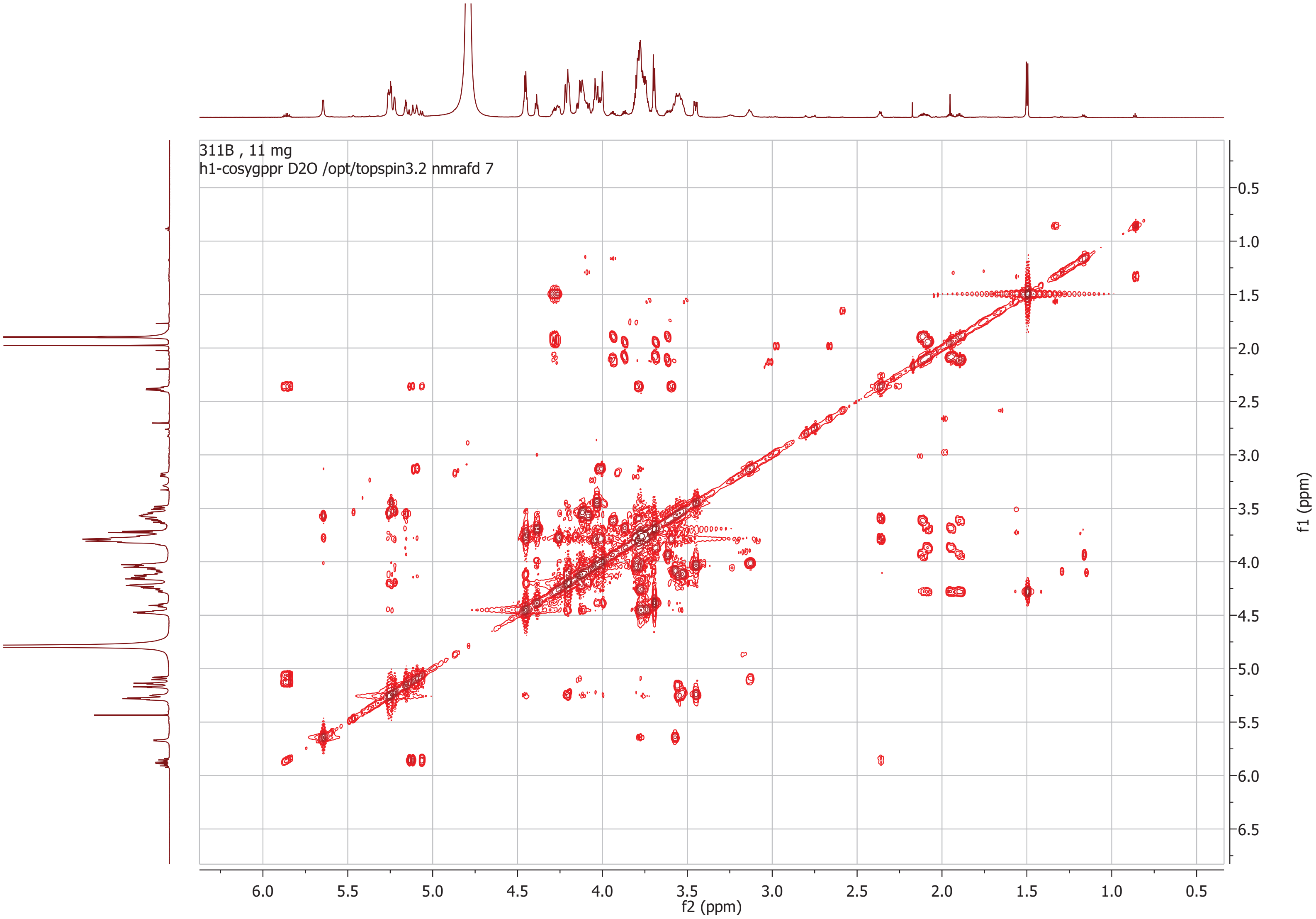

311B , 11 mg
h1-cosygppr D2O /opt/topspin3.2 nmrafd 7
f2 (ppm)
f1 (ppm)
6.0
5.5
5.0
4.5
4.0
3.5
3.0
2.5
2.0
1.5
1.0
0.5
6.5

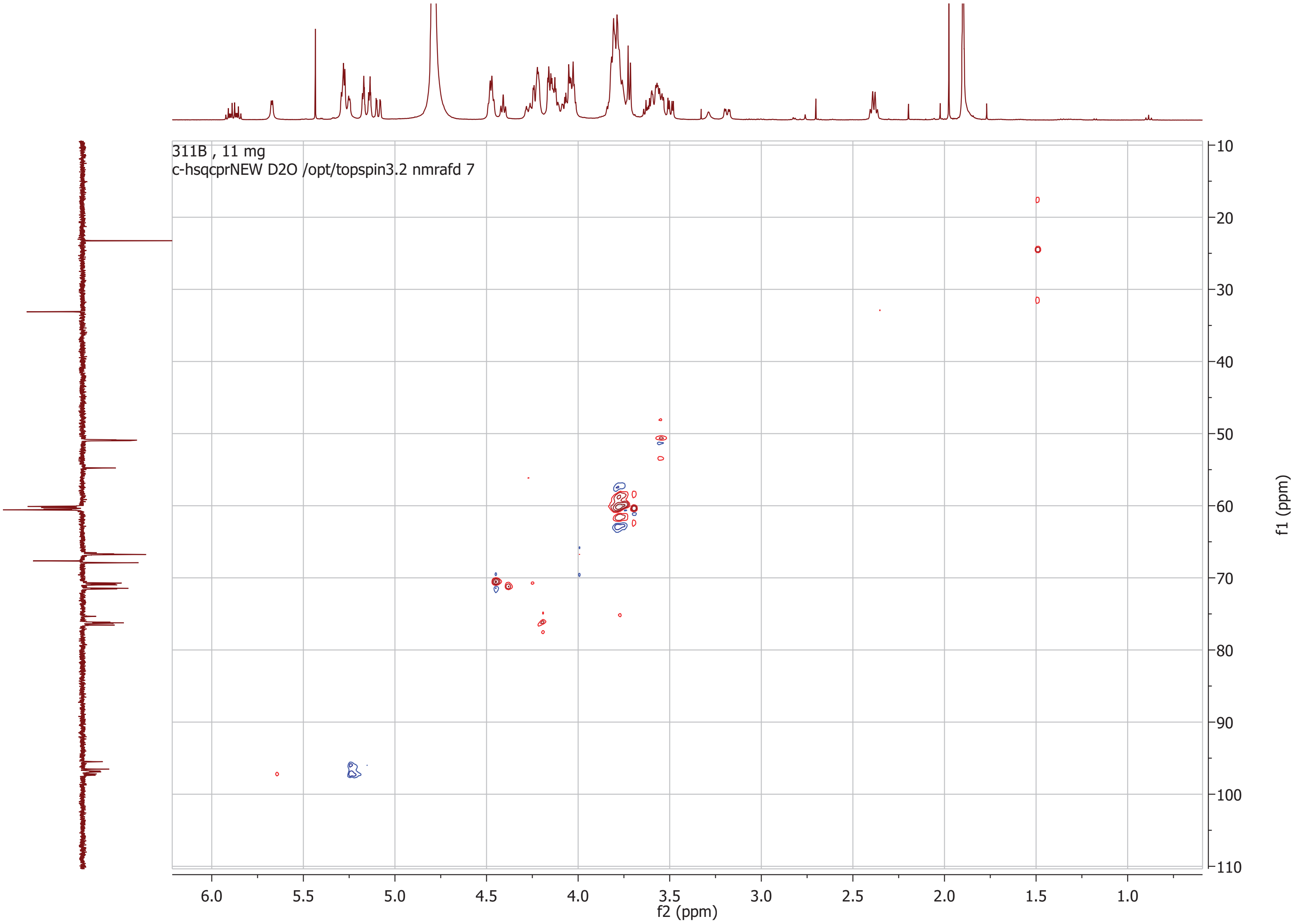

311B , 11 mg
c-hsqcprNEW D2O /opt/topspin3.2 nmrafd 7
f2 (ppm)
f1 (ppm)
6.0
5.5
5.0
4.5
4.0
3.5
3.0
2.5
2.0
1.5
1.0
10
20
30
40
50
60
70
80
90
100
110

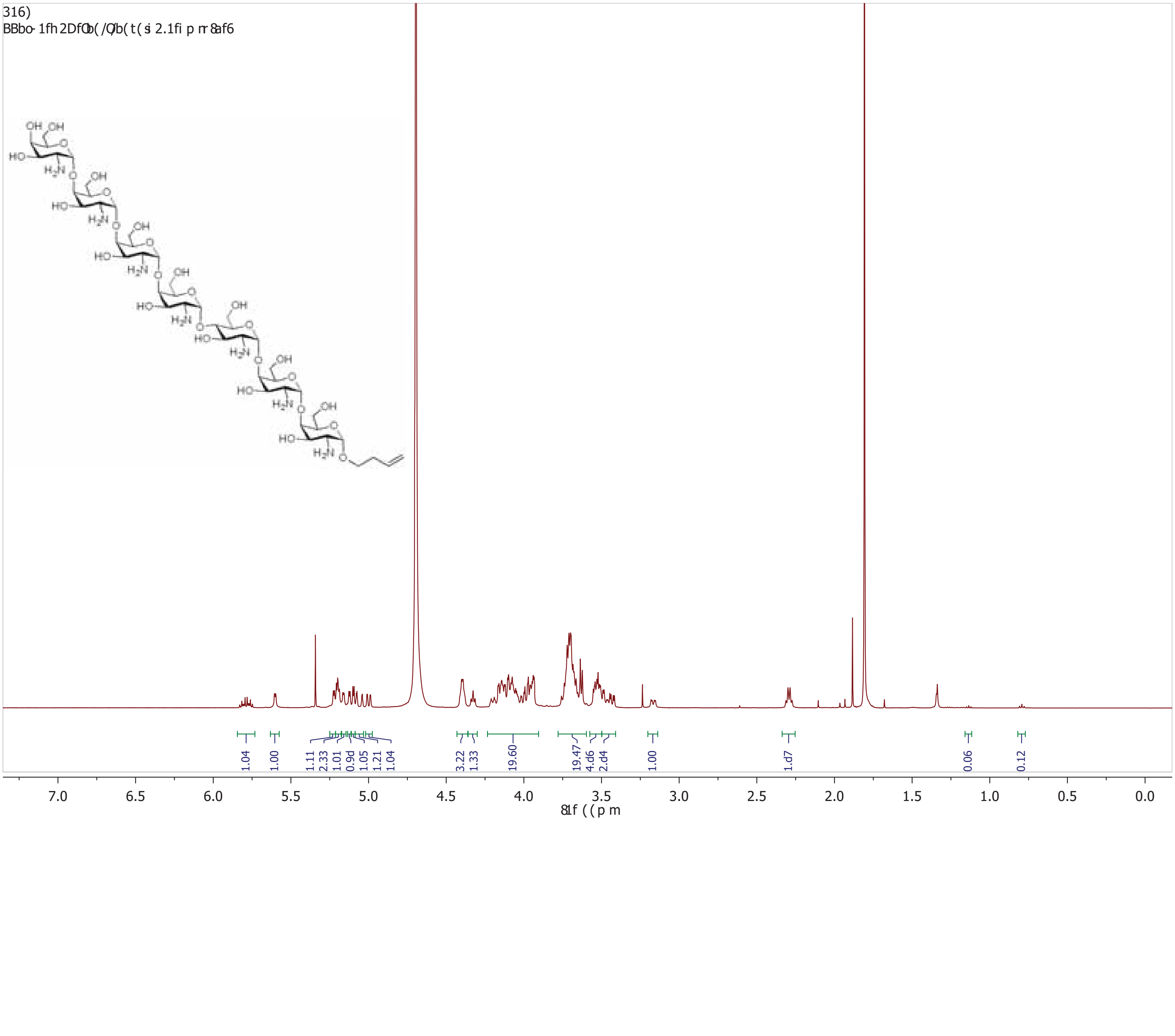
316)
1.04
1.00
1.11
2.33
1.01
0.9d
1.05
1.21
1.04
3.22
1.33
19.60
19.47
4.d6
2.d4
1.00
1.d7
0.06
0.12
7.0 6.5 6.0 5.5 5.0 4.5 4.0 3.5 3.0 2.5 2.0 1.5 1.0 0.5 0.0
OH
HO
H2N
O

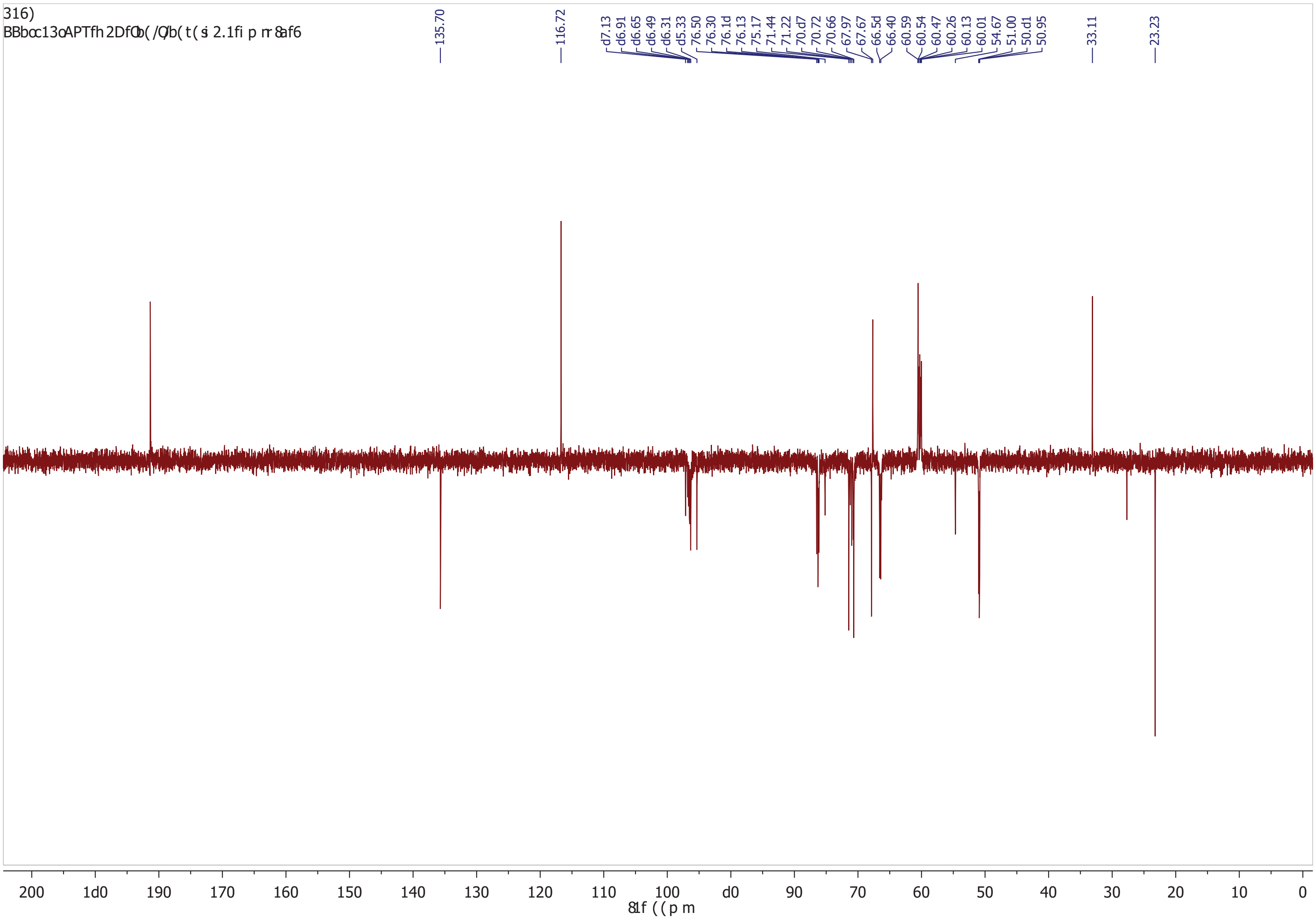
316)
BBbcc13oAPTfh 2DfOb(/Q/b(t(si 2.1fi p rr 8af6
135.70
116.72
d7.13
d6.91
d6.65
d6.49
d6.31
d5.33
76.50
76.30
76.1d
76.13
75.17
71.44
71.22
70.d7
70.72
70.66
67.97
67.67
66.5d
66.40
60.59
60.54
60.47
60.26
60.13
60.01
54.67
51.00
50.d1
50.95
33.11
23.23
200
1d0
190
170
160
150
140
130
120
110
100
d0
90
70
60
50
40
30
20
10
0
8f1 ((p m

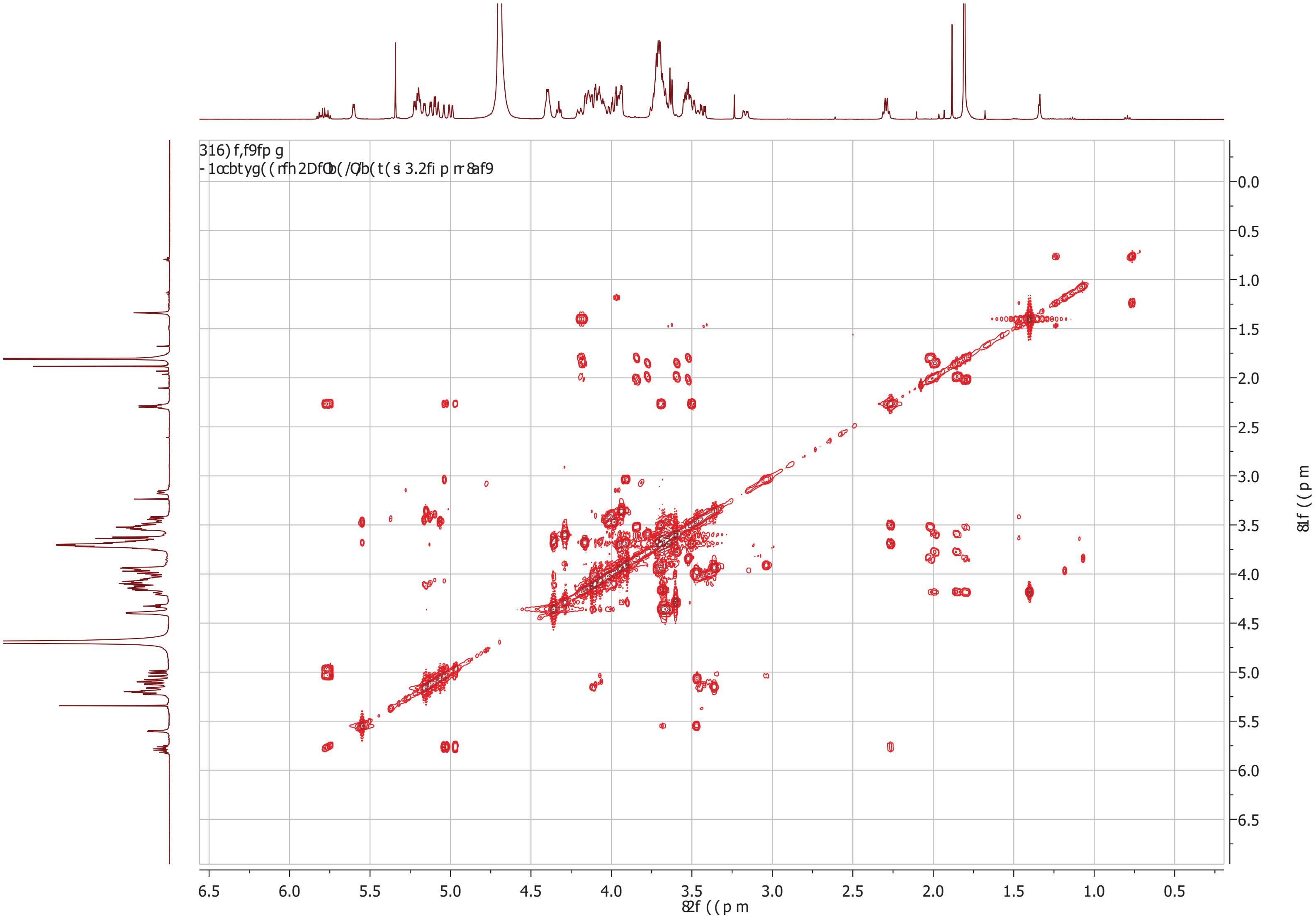
316) f,f9fp g
- 1ccbtyg((rrfh 2DfO b(/Q/b(t(si 3.2fi p rrr 8af9
6.5
6.0
5.5
5.0
4.5
4.0
3.5
3.0
2.5
2.0
1.5
1.0
0.5
82f ((p m
0.0
0.5
1.0
1.5
2.0
2.5
3.0
3.5
4.0
4.5
5.0
5.5
6.0
6.5
81f ((p m

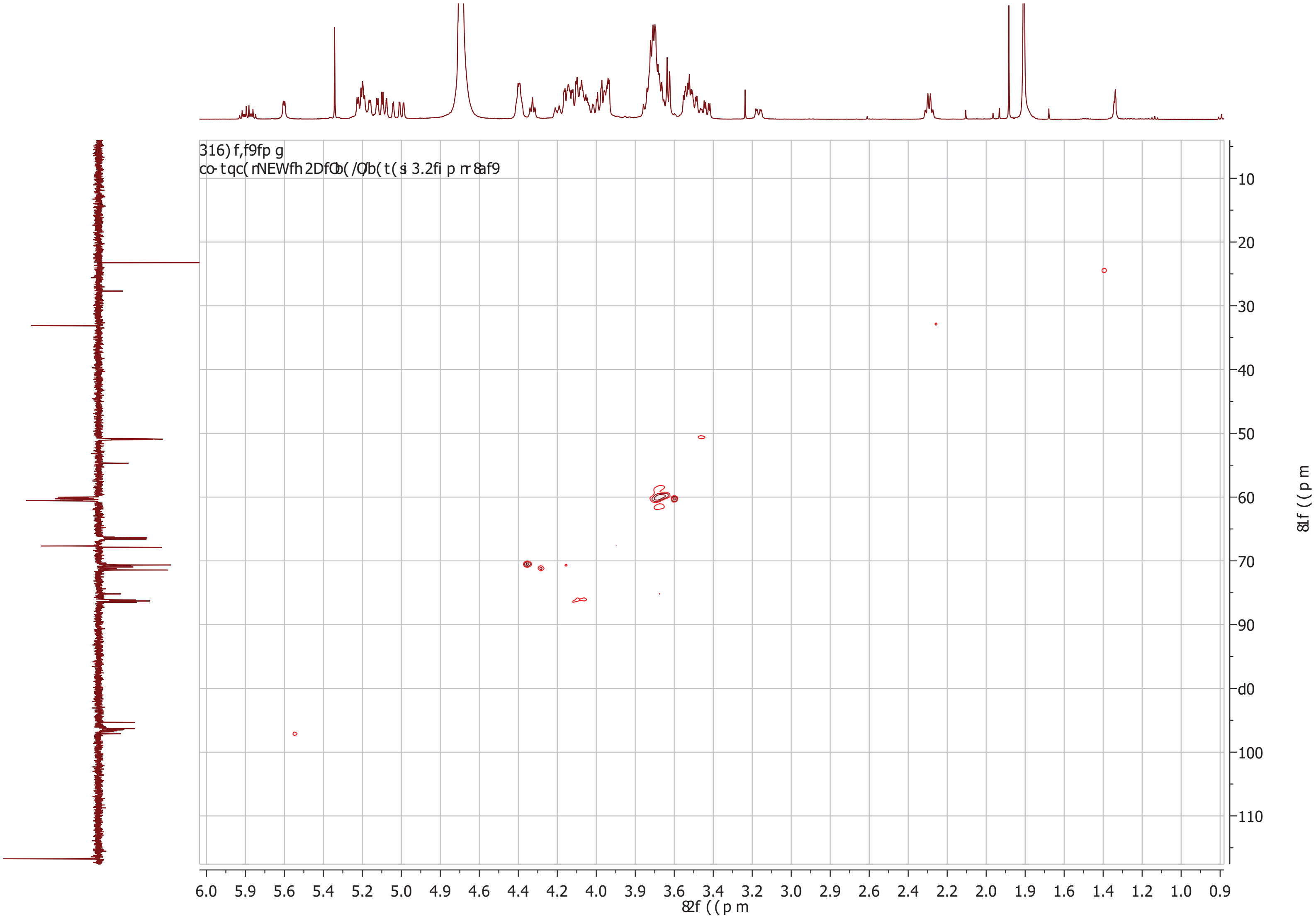
6.0 5.9 5.6 5.4 5.2 5.0 4.9 4.6 4.4 4.2 4.0 3.9 3.6 3.4 3.2 3.0 2.9 2.6 2.4 2.2 2.0 1.9 1.6 1.4 1.2 1.0 0.9
10 20 30 40 50 60 70 90 100 110

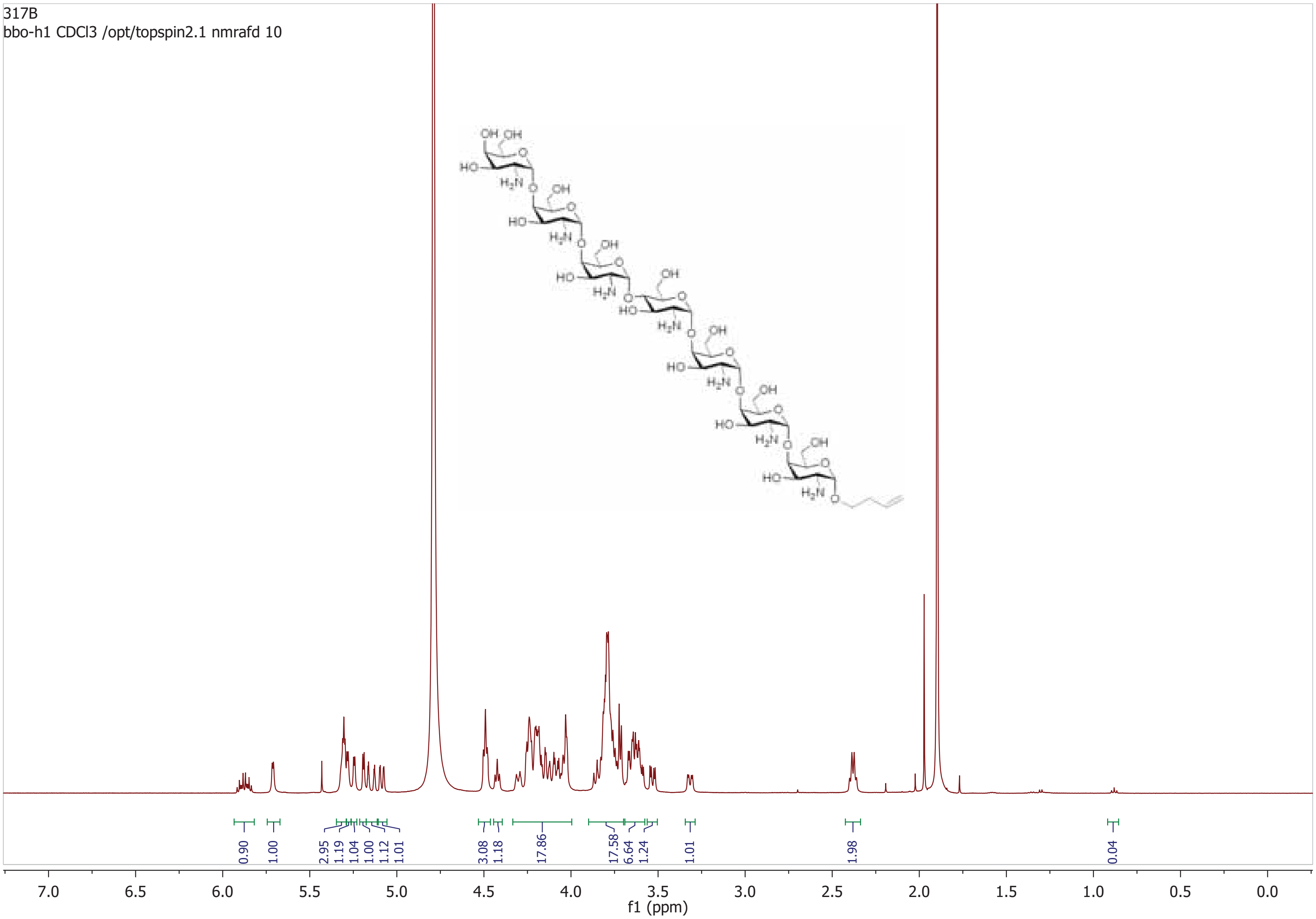
317B
bbo-h1 CDCl3 /opt/topspin2.1 nmrafd 10
0.90
1.00
2.95
1.19
1.04
1.00
1.12
1.01
3.08
1.18
17.86
17.58
6.64
1.24
1.01
1.98
0.04
7.0 6.5 6.0 5.5 5.0 4.5 4.0 3.5 3.0 2.5 2.0 1.5 1.0 0.5 0.0
f1 (ppm)

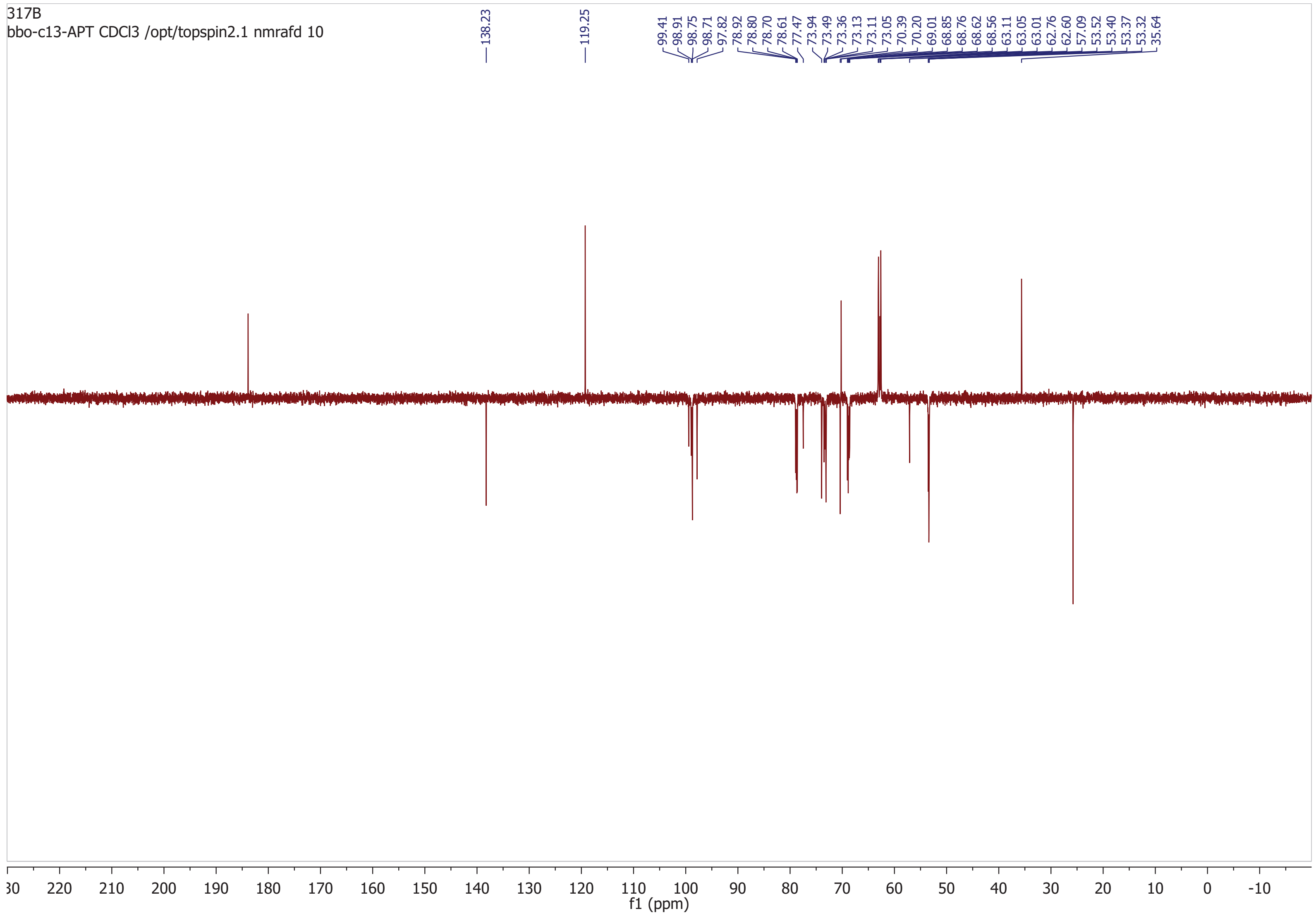
317B
bbo-c13-APT CDCl3 /opt/topspin2.1 nmrafd 10
138.23
119.25
99.41
98.91
98.75
98.71
97.82
78.92
78.80
78.70
78.61
77.47
73.94
73.49
73.36
73.13
73.11
73.05
70.39
70.20
69.01
68.85
68.76
68.62
68.56
63.11
63.05
63.01
62.76
62.60
57.09
53.52
53.40
53.37
53.32
35.64
f1 (ppm)

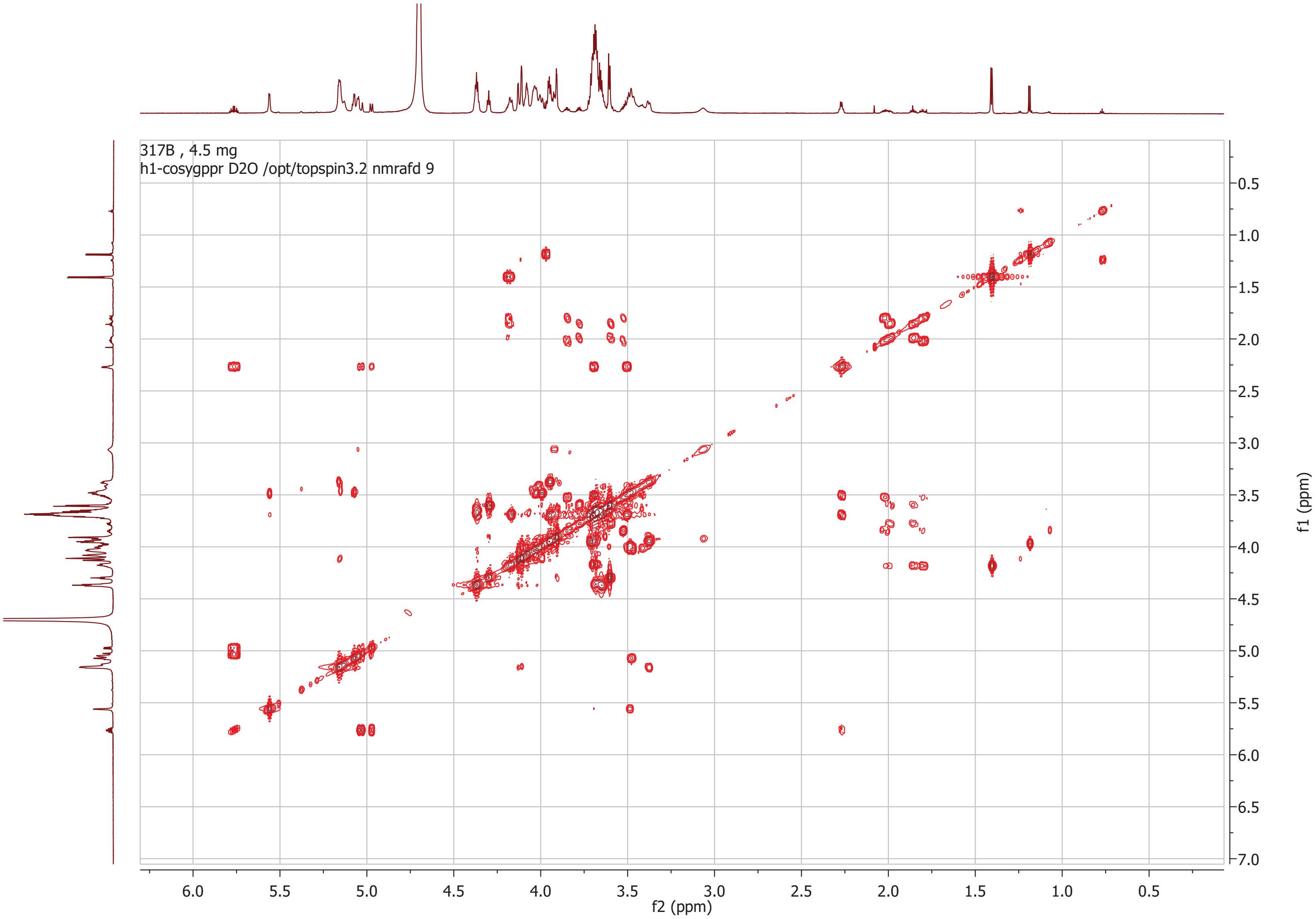
317B , 4.5 mg
h1-cosygppr D2O /opt/topspin3.2 nmrafd 9
f2 (ppm)
f1 (ppm)

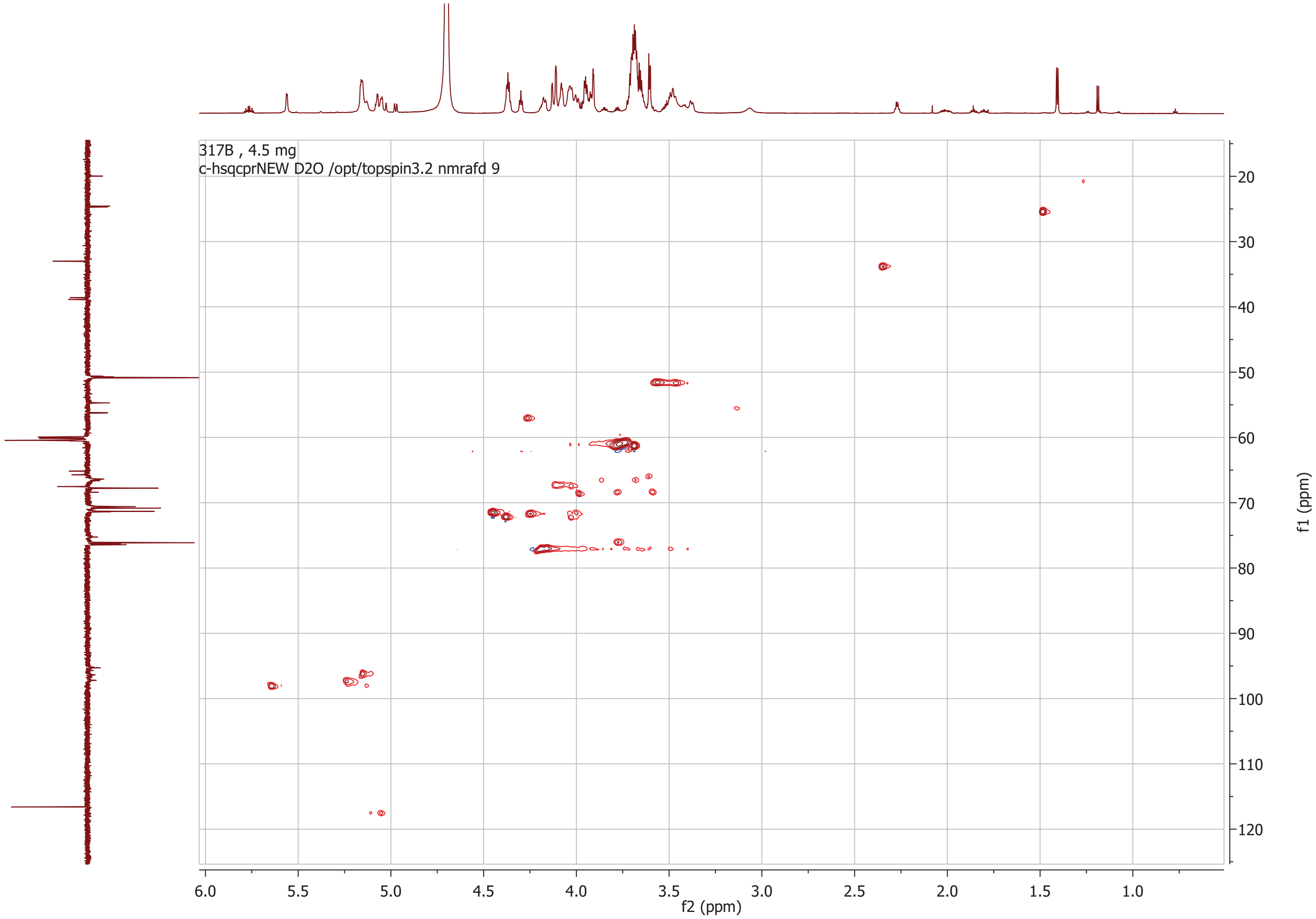

317B , 4.5 mg
c-hsqcprNEW D2O /opt/topspin3.2 nmrafd 9
20
30
40
50
60
70
80
90
100
110
120
f1 (ppm)
6.0
5.5
5.0
4.5
4.0
3.5
3.0
2.5
2.0
1.5
1.0
f2 (ppm)

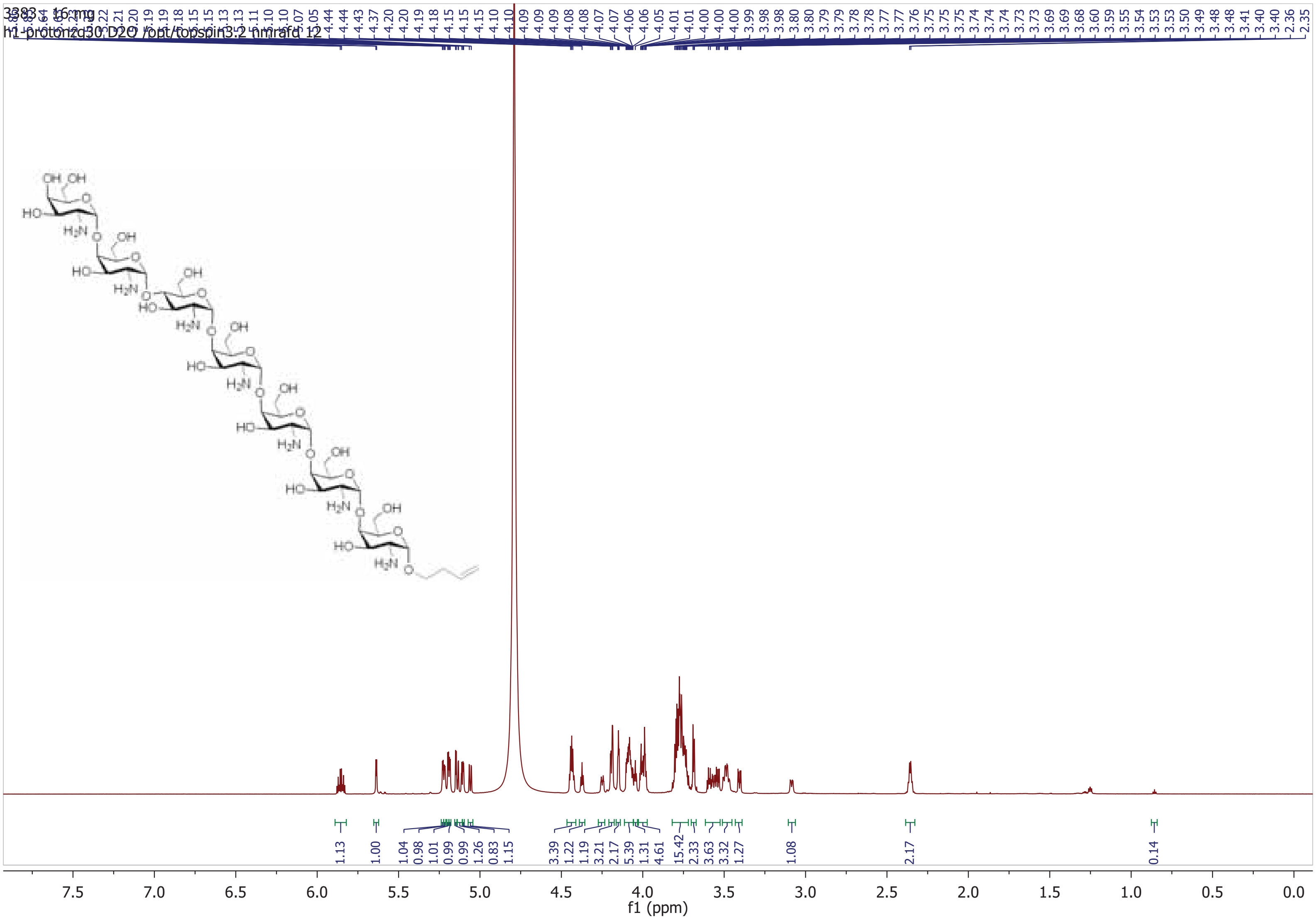
3383 - 16 mg
h1-protonzg30.D2O /opt/topspin3.2 nmrafd 12
f1 (ppm)

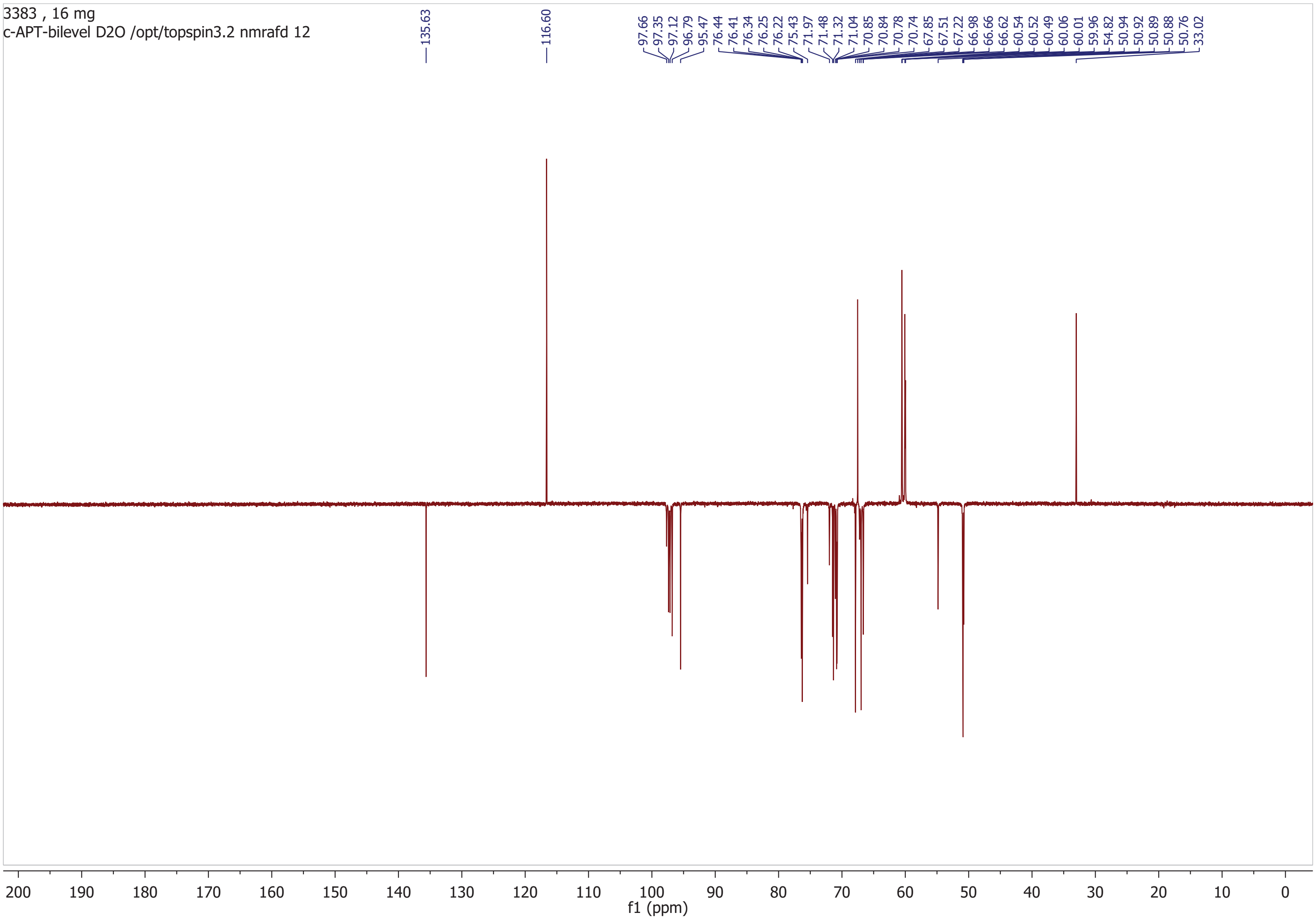

3383 , 16 mg
c-APT-bilevel D2O /opt/topspin3.2 nmrafd 12
135.63
116.60
97.66
97.35
97.12
96.79
95.47
76.44
76.41
76.34
76.25
76.22
75.43
71.97
71.48
71.32
71.04
70.85
70.84
70.78
70.74
67.85
67.51
67.22
66.98
66.66
66.62
60.54
60.52
60.49
60.06
60.01
59.96
54.82
50.94
50.92
50.89
50.88
50.76
33.02
200 190 180 170 160 150 140 130 120 110 100 90 80 70 60 50 40 30 20 10 0
f1 (ppm)

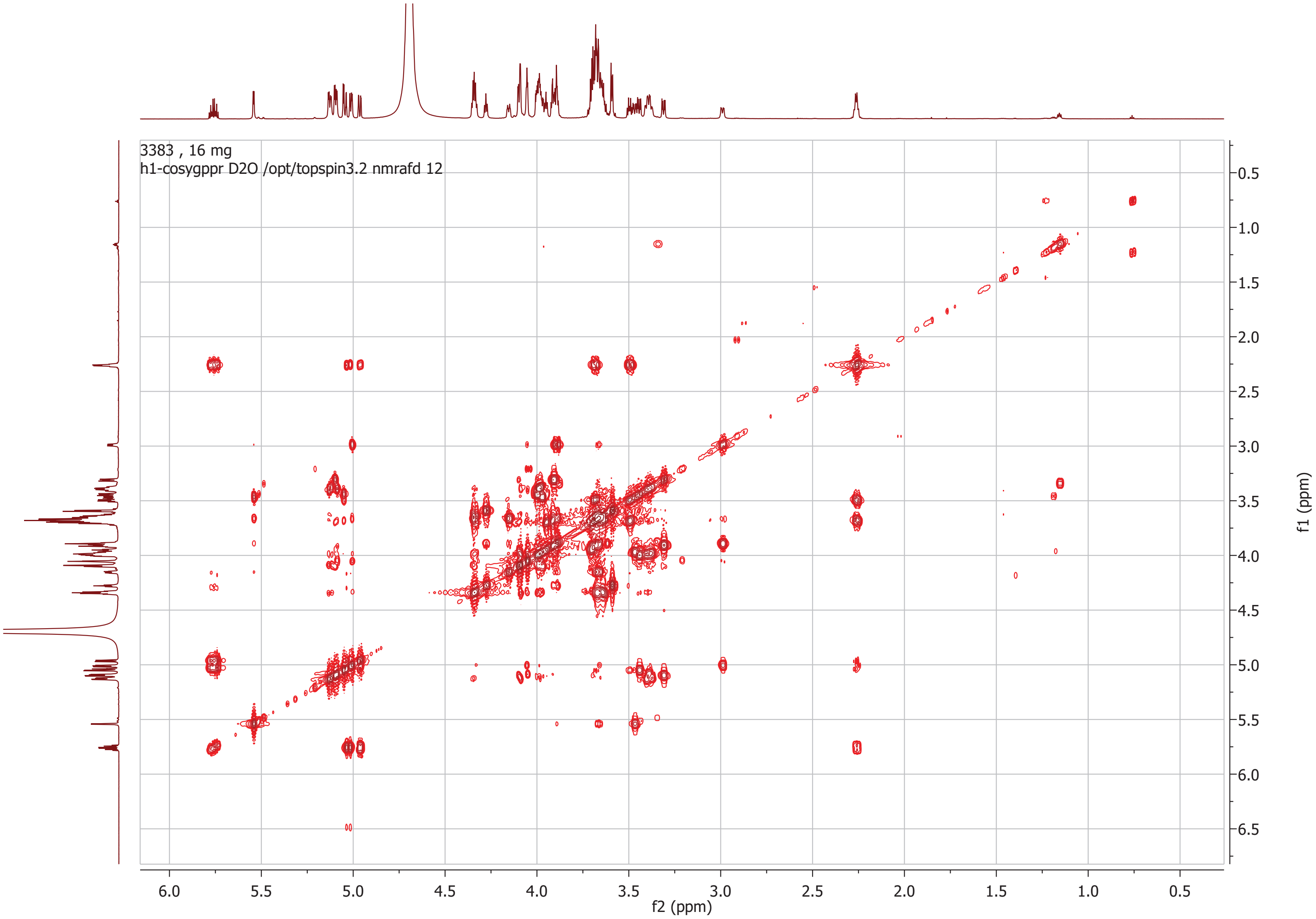

3383 , 16 mg
h1-cosygppr D2O /opt/topspin3.2 nmrafd 12
0.5
1.0
1.5
2.0
2.5
3.0
3.5
4.0
4.5
5.0
5.5
6.0
6.5
f1 (ppm)
6.0
5.5
5.0
4.5
4.0
3.5
3.0
2.5
2.0
1.5
1.0
0.5
f2 (ppm)

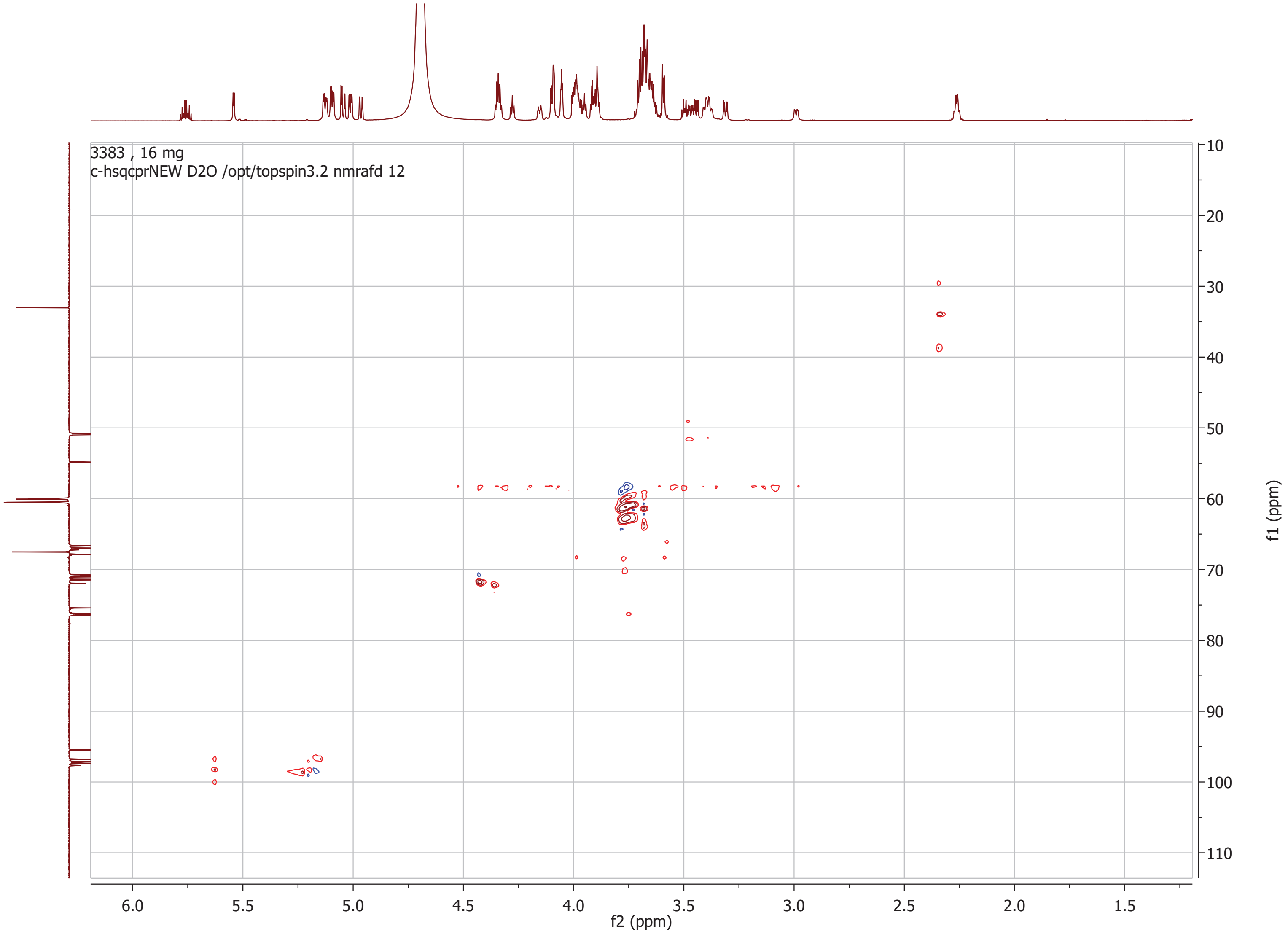

3383 , 16 mg
c-hsqcprNEW D2O /opt/topspin3.2 nmrafd 12
f2 (ppm)
f1 (ppm)

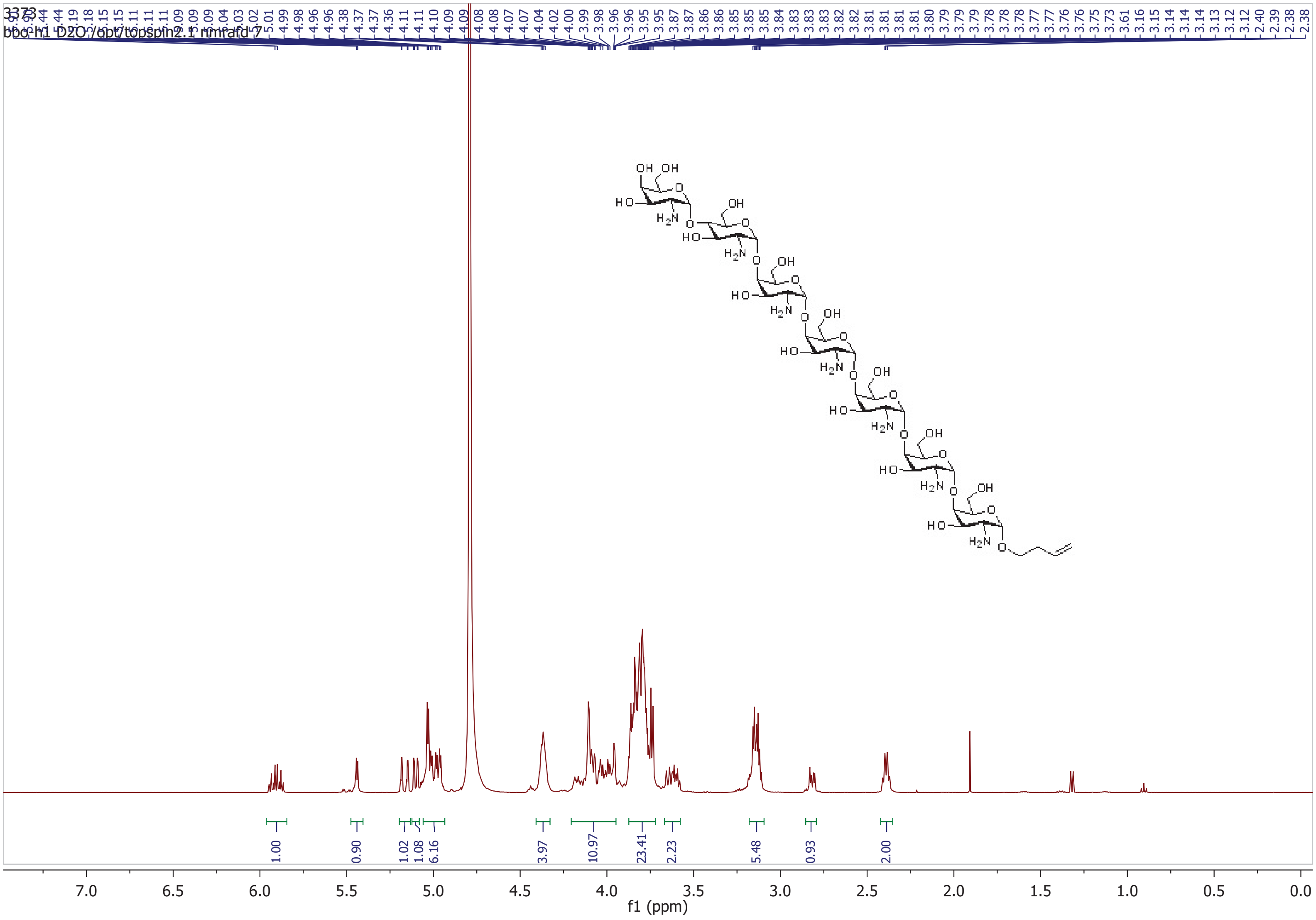

3373
bbo h1 D2O /opt/topspin2.1 nmrafd 7
5.44 5.19 5.18 5.15 5.15 5.11 5.11 5.11 5.09 5.09 5.09 5.04 5.03 5.02 5.01 4.99 4.98 4.96 4.96 4.38 4.37 4.37 4.36 4.11 4.11 4.10 4.09 4.09 4.08 4.08 4.07 4.07 4.04 4.02 4.00 3.99 3.98 3.96 3.96 3.95 3.95 3.87 3.86 3.86 3.85 3.85 3.85 3.84 3.83 3.83 3.83 3.82 3.82 3.81 3.81 3.81 3.81 3.80 3.79 3.79 3.79 3.78 3.78 3.77 3.77 3.76 3.76 3.75 3.73 3.61 3.16 3.15 3.15 3.14 3.14 3.13 3.12 3.12 2.40 2.39 2.38 2.38
OH OH
HO
O
H2N
OH
1.00
0.90
1.02
1.08
6.16
3.97
10.97
23.41
2.23
5.48
0.93
2.00
7.0 6.5 6.0 5.5 5.0 4.5 4.0 3.5 3.0 2.5 2.0 1.5 1.0 0.5 0.0
f1 (ppm)

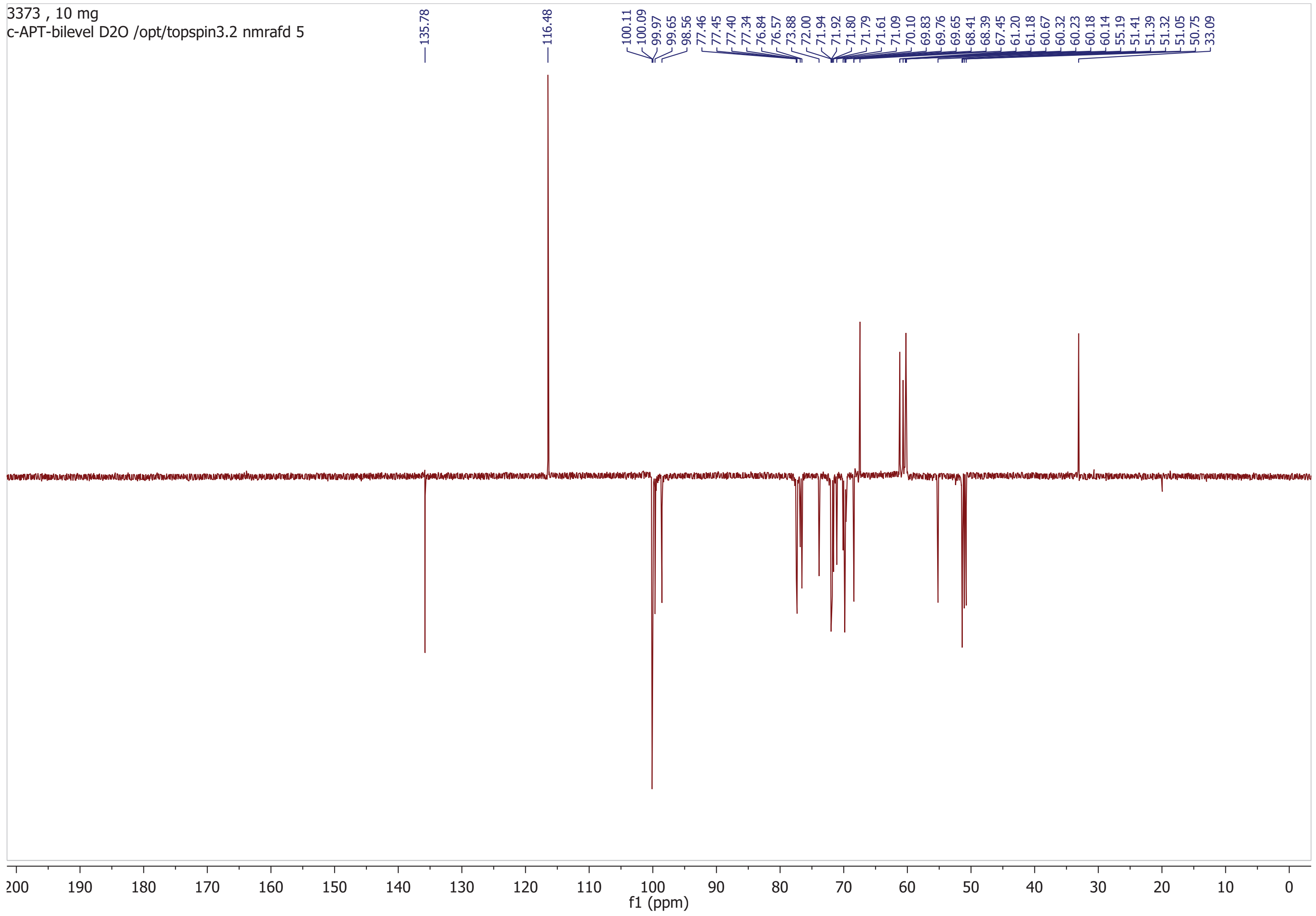
3373 , 10 mg
c-APT-bilevel D2O /opt/topspin3.2 nmrafd 5
135.78
116.48
100.11
100.09
99.97
99.65
98.56
77.46
77.45
77.40
77.34
76.84
76.57
73.88
72.00
71.94
71.92
71.80
71.79
71.61
71.09
70.10
69.83
69.76
69.65
68.41
68.39
67.45
61.20
61.18
60.67
60.32
60.23
60.18
60.14
55.19
51.41
51.39
51.32
51.05
50.75
33.09
f1 (ppm)

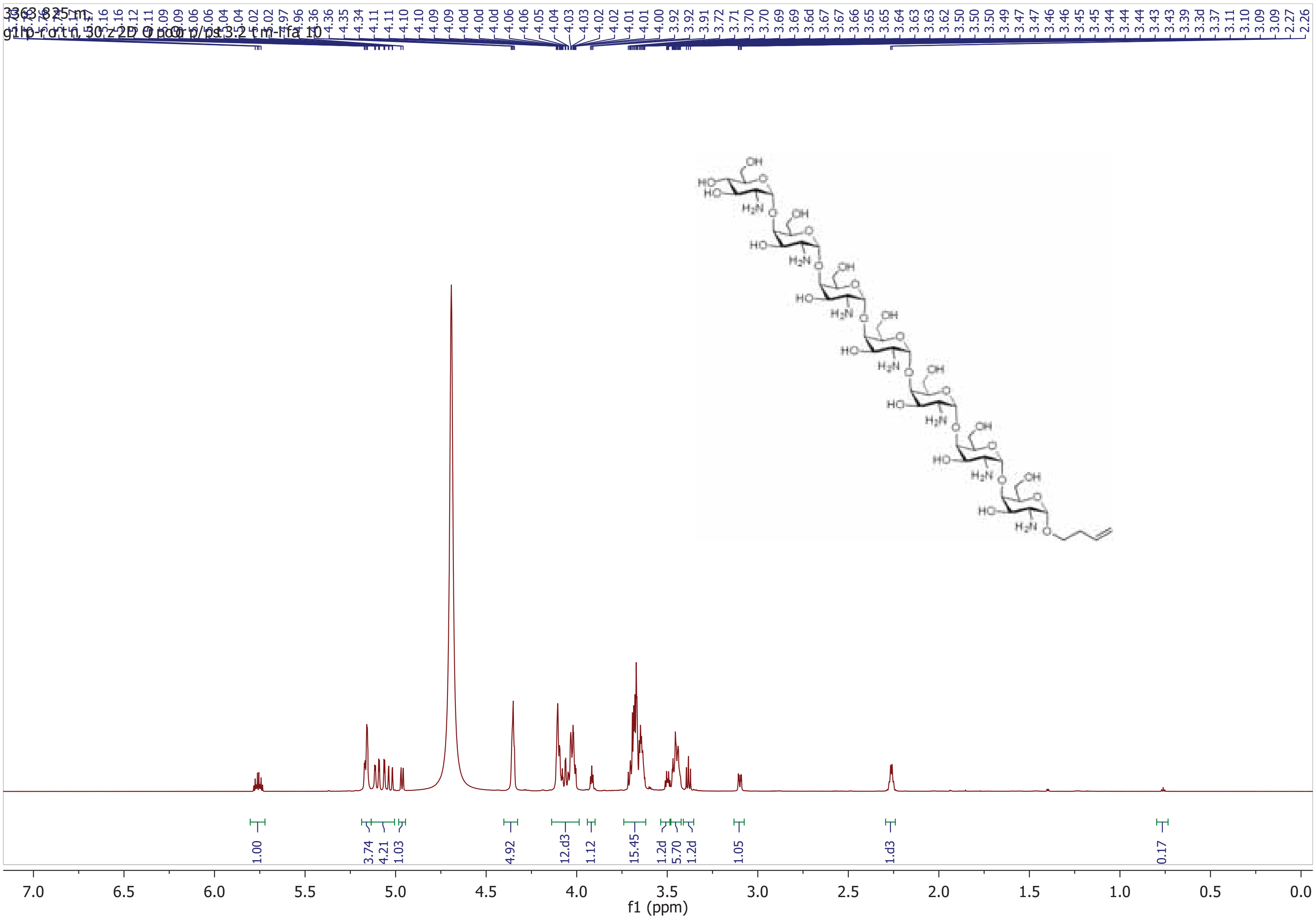

4.36 4.35 4.34 4.11 4.11 4.10 4.10 4.09 4.09 4.06 4.06 4.05 4.04 4.03 4.03 4.02 4.02 4.01 4.01 4.00 3.92 3.92 3.91 3.72 3.71 3.70 3.70 3.69 3.69 3.67 3.66 3.65 3.65 3.64 3.63 3.63 3.62 3.50 3.50 3.49 3.47 3.46 3.46 3.45 3.45 3.44 3.44 3.44 3.43 3.43 3.39 3.37 3.11 3.10 3.09 3.09 2.27 2.26
1.00
3.74
4.21
1.03
4.92
1.12
15.45
5.70
1.05
0.17
7.0 6.5 6.0 5.5 5.0 4.5 4.0 3.5 3.0 2.5 2.0 1.5 1.0 0.5 0.0
f1 (ppm)
HO
OH
H2N
O

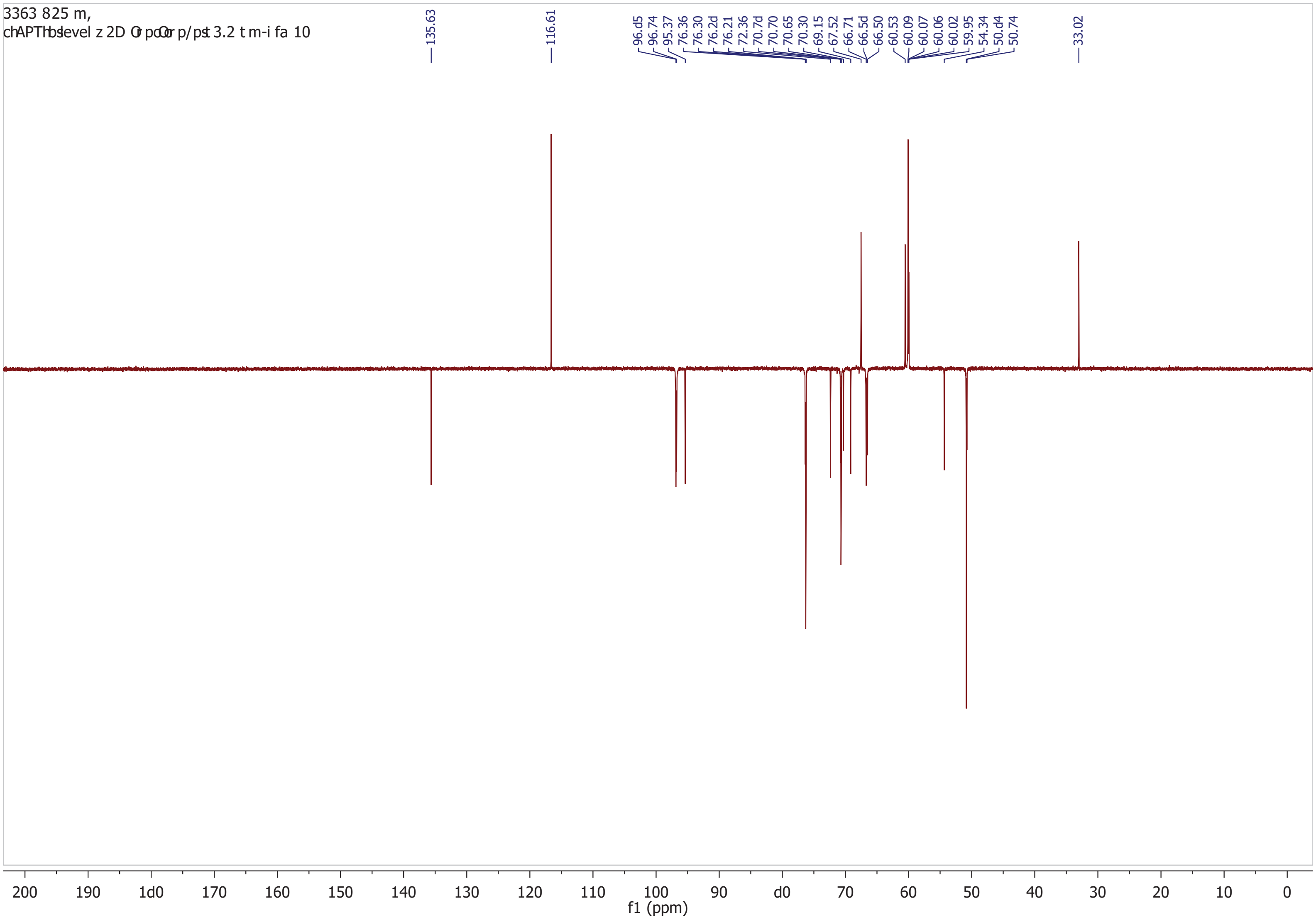
3363 825 m,
chAPThbslevel z 2D Orpo Or p/pst 3.2 t m-i fa 10
135.63
116.61
96.d5
96.74
95.37
76.36
76.30
76.2d
76.21
72.36
70.7d
70.70
70.65
70.30
69.15
67.52
66.71
66.5d
66.50
60.53
60.09
60.07
60.06
60.02
59.95
54.34
50.d4
50.74
33.02
200
190
1d0
170
160
150
140
130
120
110
100
90
d0
70
60
50
40
30
20
10
0
f1 (ppm)

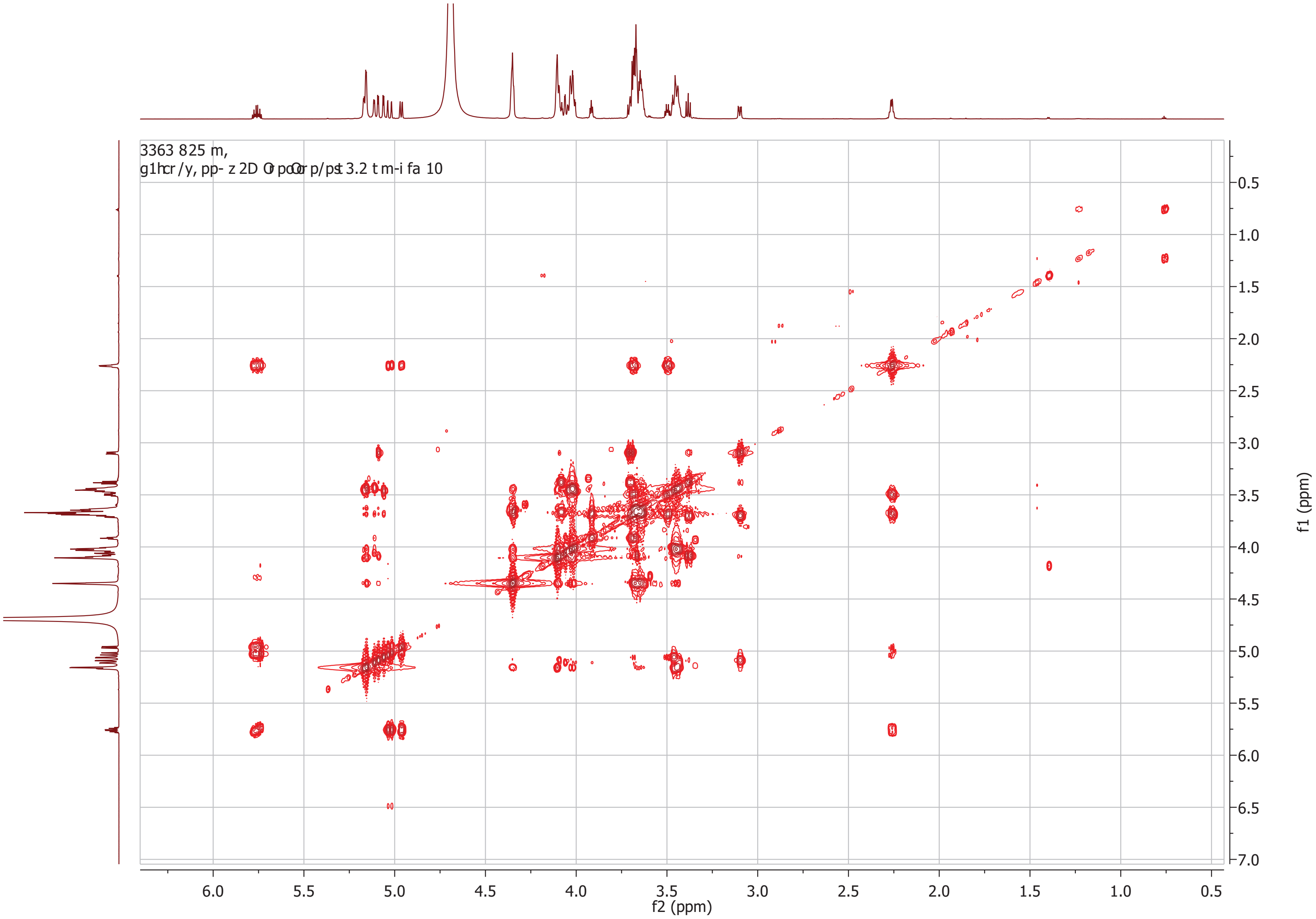
3363 825 m,
g1hcr /y, pp- z 2D Or poOr p/pst 3.2 t m-i fa 10
f2 (ppm)
f1 (ppm)

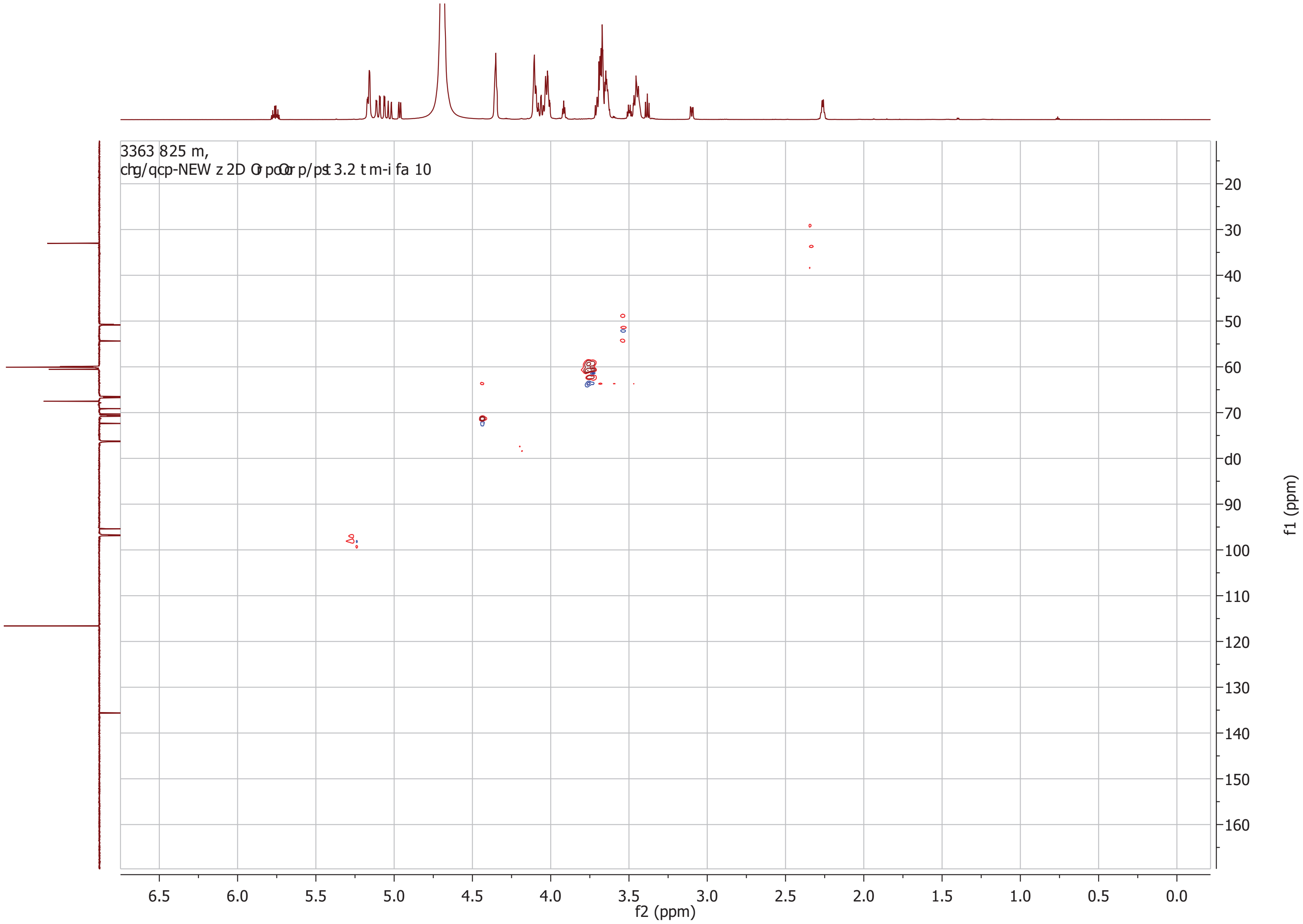

3363 825 m,
chg/qcp-NEW z 2D Or poOr p/pst 3.2 t m-i fa 10
6.5
6.0
5.5
5.0
4.5
4.0
3.5
3.0
2.5
2.0
1.5
1.0
0.5
0.0
f2 (ppm)
20
30
40
50
60
70
d0
90
100
110
120
130
140
150
160
f1 (ppm)

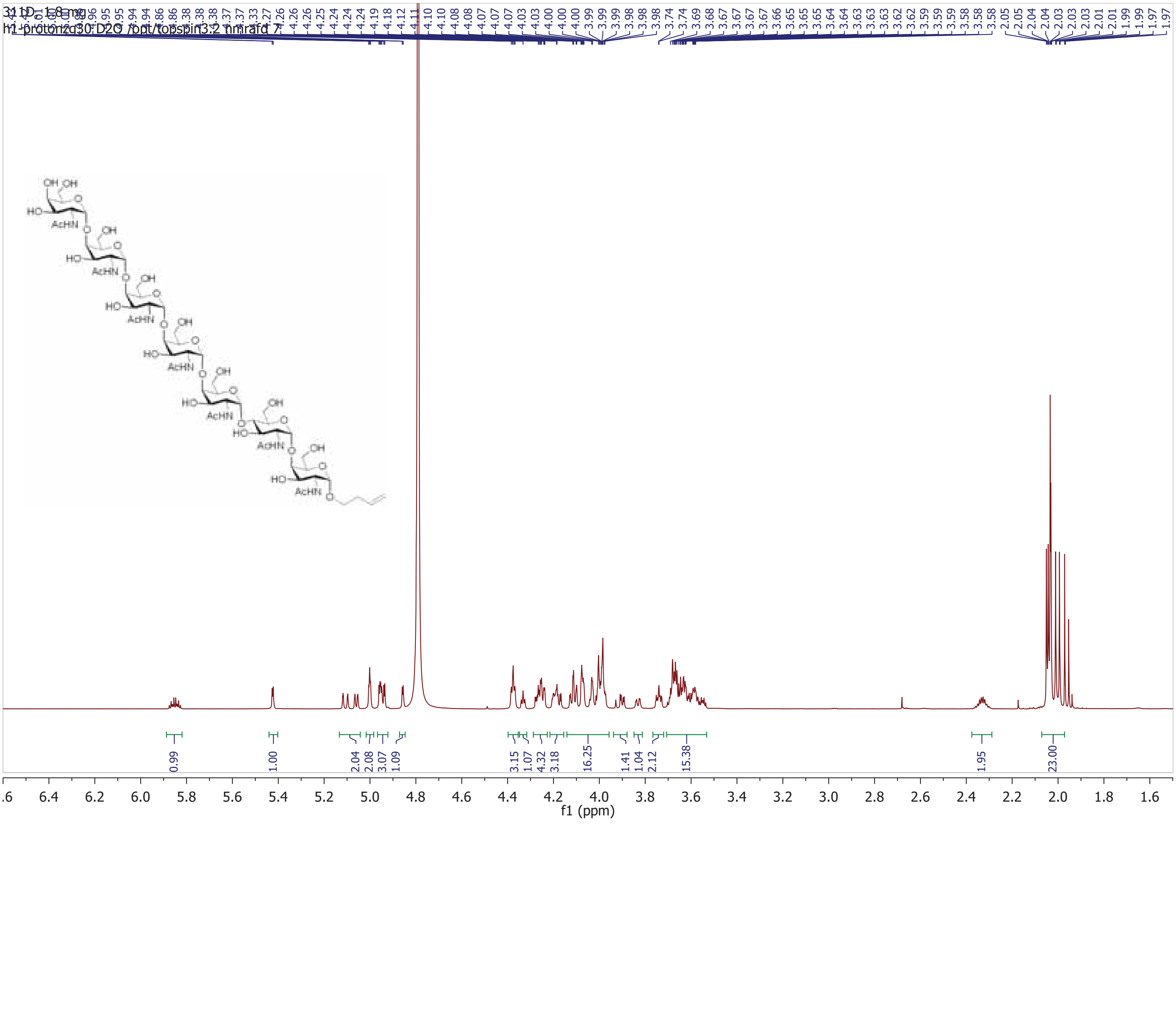

3.96
3.95
3.95
3.94
3.94
3.86
4.38
4.38
4.38
4.37
4.37
4.33
4.27
4.26
4.26
4.25
4.24
4.24
4.24
4.19
4.18
4.12
4.11
4.10
4.10
4.08
4.08
4.07
4.07
4.07
4.03
4.03
4.00
4.00
3.99
3.99
3.99
3.98
3.98
3.74
3.74
3.69
3.68
3.67
3.67
3.67
3.66
3.65
3.65
3.64
3.64
3.63
3.63
3.63
3.62
3.59
3.59
3.59
3.58
3.58
3.58
2.05
2.04
2.04
2.03
2.03
2.01
2.01
1.99
1.99
1.97
1.97
OH
OH
HO
AcHN
0.99
1.00
2.04
2.08
3.07
1.09
3.15
1.07
4.32
3.18
16.25
1.41
1.04
2.12
15.38
1.95
23.00
6.4
6.2
6.0
5.8
5.6
5.4
5.2
5.0
4.8
4.6
4.4
4.2
4.0
3.8
3.6
3.4
3.2
3.0
2.8
2.6
2.4
2.2
2.0
1.8
1.6
f1 (ppm)

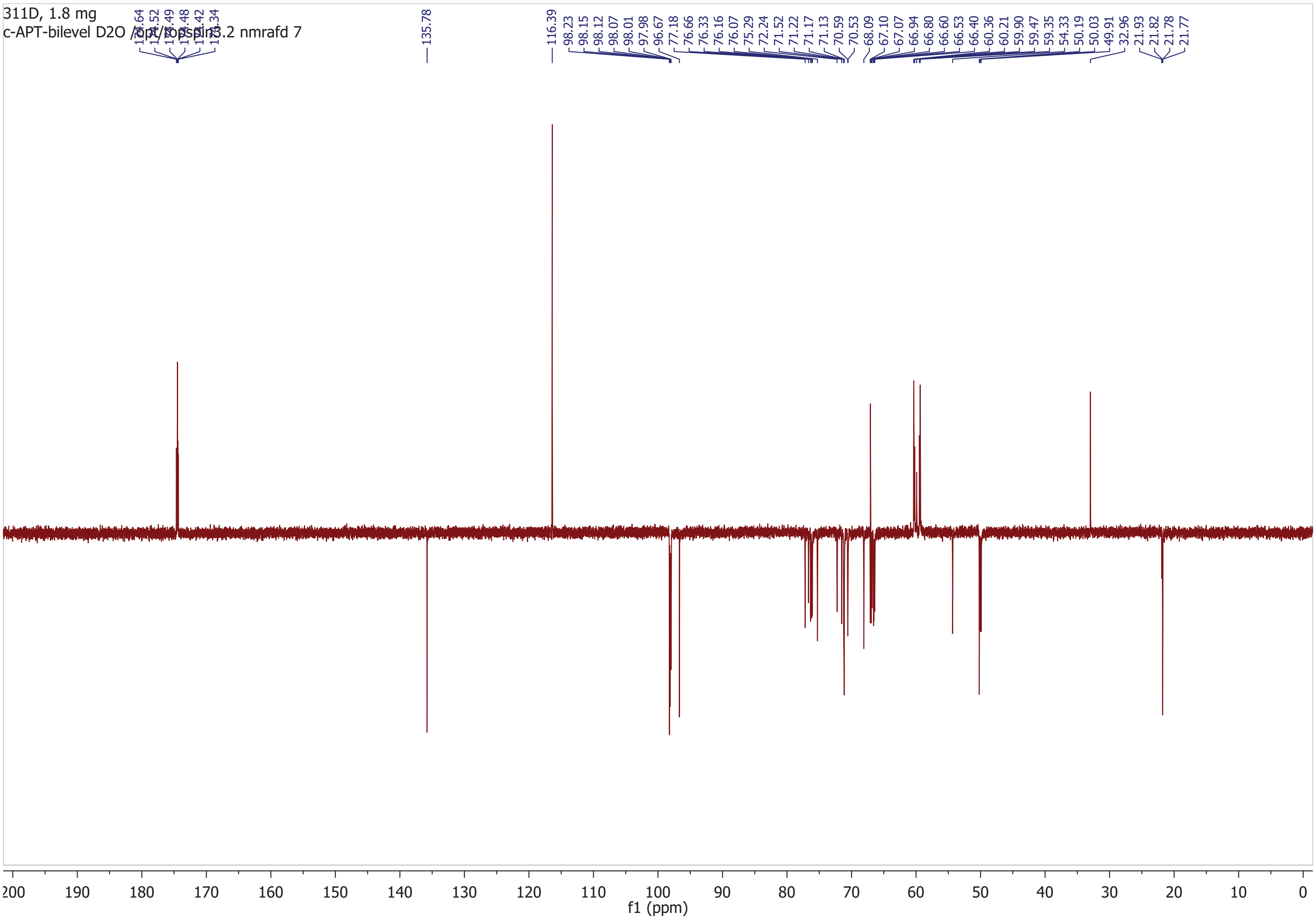

311D, 1.8 mg
c-APT-bilevel D2O /opt/topspin3.2 nmrafd 7
174.64
174.52
174.49
174.48
174.42
174.34
135.78
116.39
98.23
98.15
98.12
98.07
98.01
97.98
96.67
77.18
76.66
76.33
76.16
76.07
75.29
72.24
71.52
71.22
71.17
71.13
70.59
70.53
68.09
67.10
67.07
66.94
66.80
66.60
66.53
66.40
60.36
60.21
59.90
59.47
59.35
54.33
50.19
50.03
49.91
32.96
21.93
21.82
21.78
21.77
200
190
180
170
160
150
140
130
120
110
100
90
80
70
60
50
40
30
20
10
0
f1 (ppm)

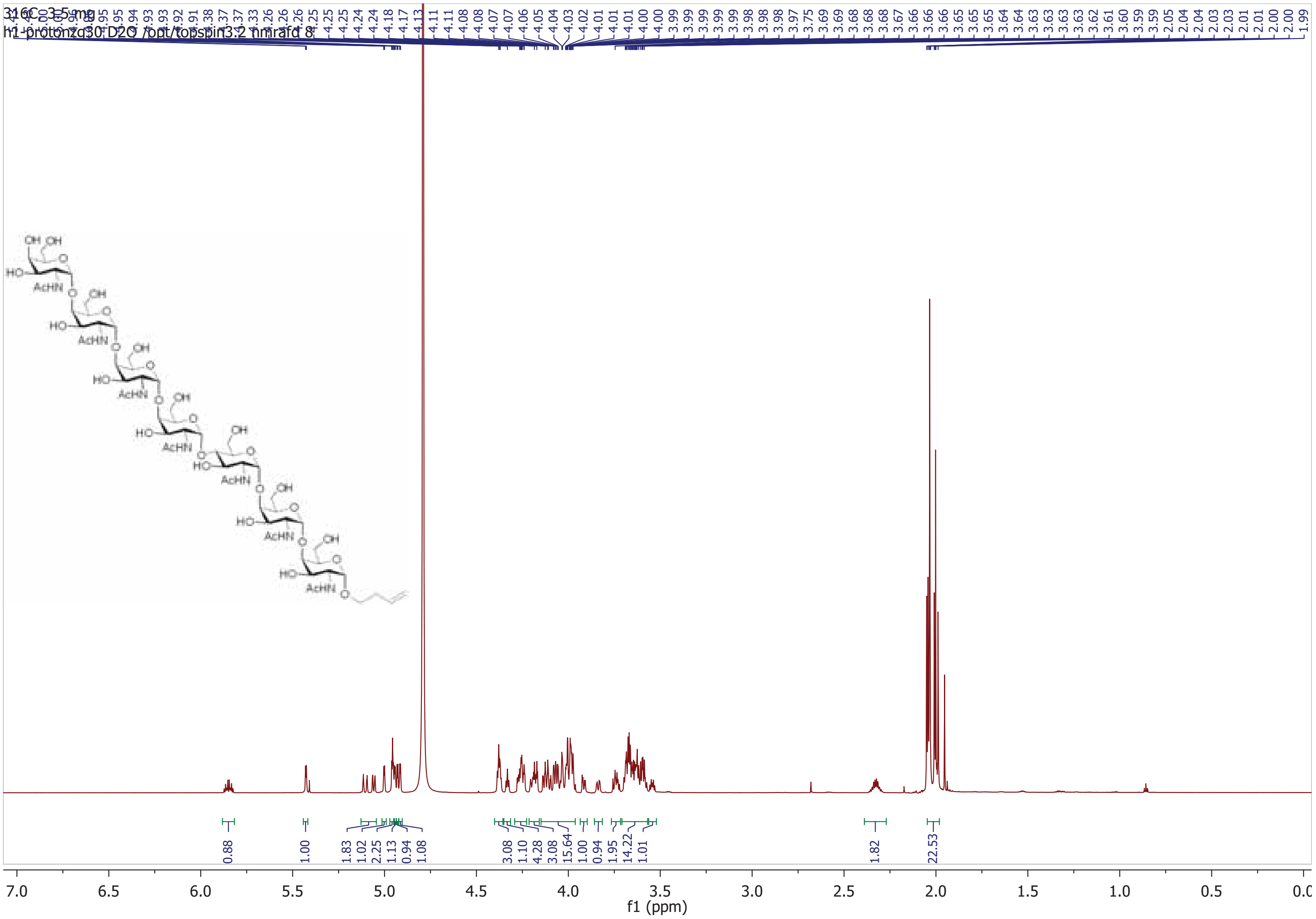

316C 3.5 mg
h1-protonzg30 D2O /opt/topspin3.2 nmrafd 8
4.38 4.37 4.37 4.33 4.26 4.26 4.26 4.25 4.25 4.25 4.24 4.24 4.18 4.17 4.13 4.11 4.11 4.08 4.08 4.07 4.07 4.06 4.05 4.04 4.03 4.02 4.01 4.01 4.01 4.00 4.00 3.99 3.99 3.99 3.99 3.99 3.98 3.98 3.98 3.97 3.75 3.69 3.69 3.68 3.68 3.68 3.67 3.66 3.66 3.66 3.65 3.65 3.64 3.63 3.63 3.63 3.63 3.62 3.61 3.60 3.59 3.59 2.05 2.04 2.04 2.03 2.03 2.01 2.01 2.00 2.00 1.99
OH OH
HO
AcHN
O
OH
AcHN
0.88
1.00
1.83
1.02
2.25
1.13
0.94
1.08
3.08
1.10
4.28
3.08
15.64
1.00
0.94
1.95
14.22
1.01
1.82
22.53
7.0 6.5 6.0 5.5 5.0 4.5 4.0 3.5 3.0 2.5 2.0 1.5 1.0 0.5
f1 (ppm)

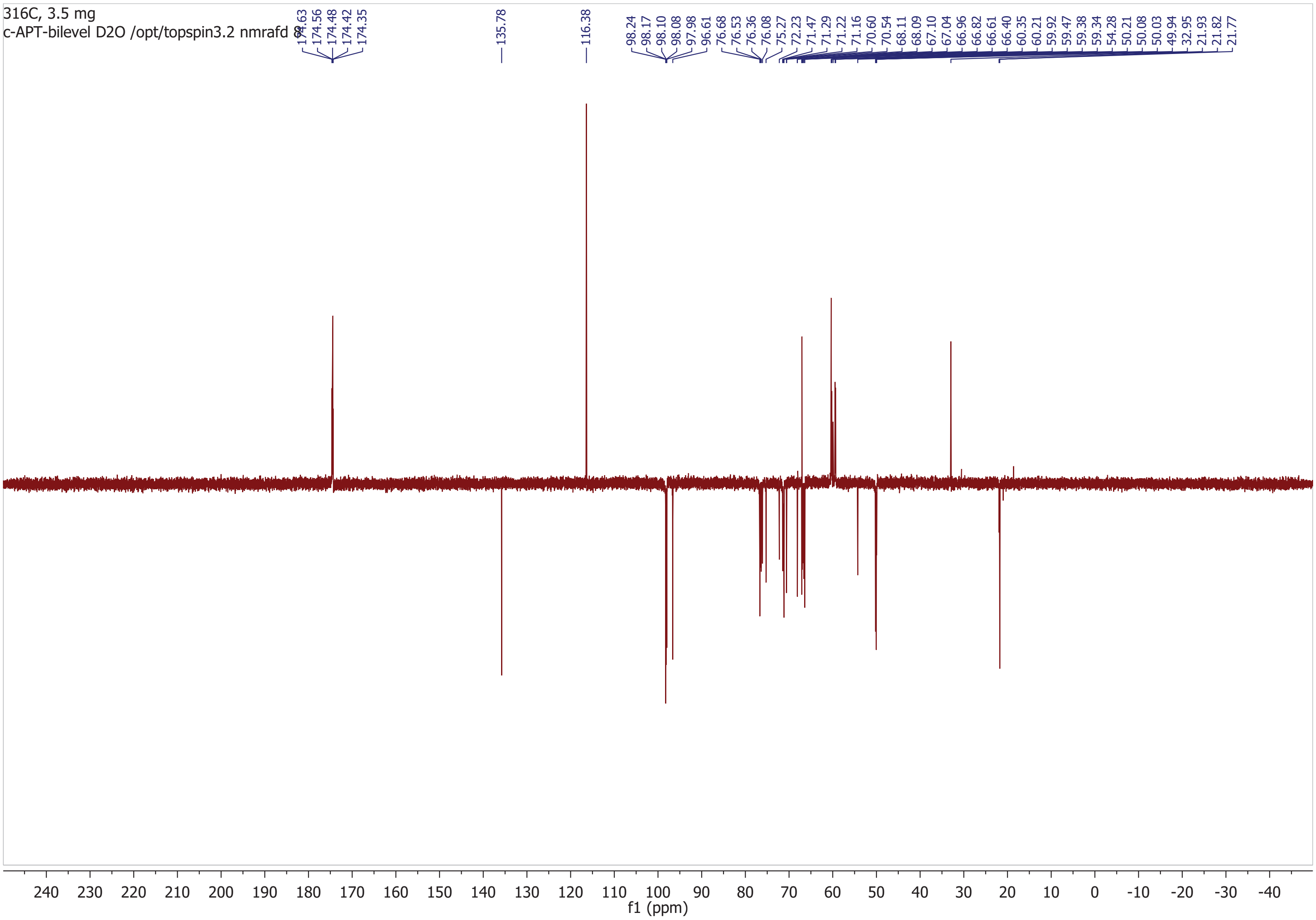
316C, 3.5 mg
c-APT-bilevel D2O /opt/topspin3.2 nmrafd 8
174.63
174.56
174.48
174.42
174.35
135.78
116.38
98.24
98.17
98.10
98.08
97.98
96.61
76.68
76.53
76.36
76.08
75.27
72.23
71.47
71.29
71.22
71.16
70.60
70.54
68.11
68.09
67.10
67.04
66.96
66.82
66.61
66.40
60.35
60.21
59.92
59.47
59.38
59.34
54.28
50.21
50.08
50.03
49.94
32.95
21.93
21.82
21.77
240 230 220 210 200 190 180 170 160 150 140 130 120 110 100 90 80 70 60 50 40 30 20 10 0 -10 -20 -30 -40
f1 (ppm)

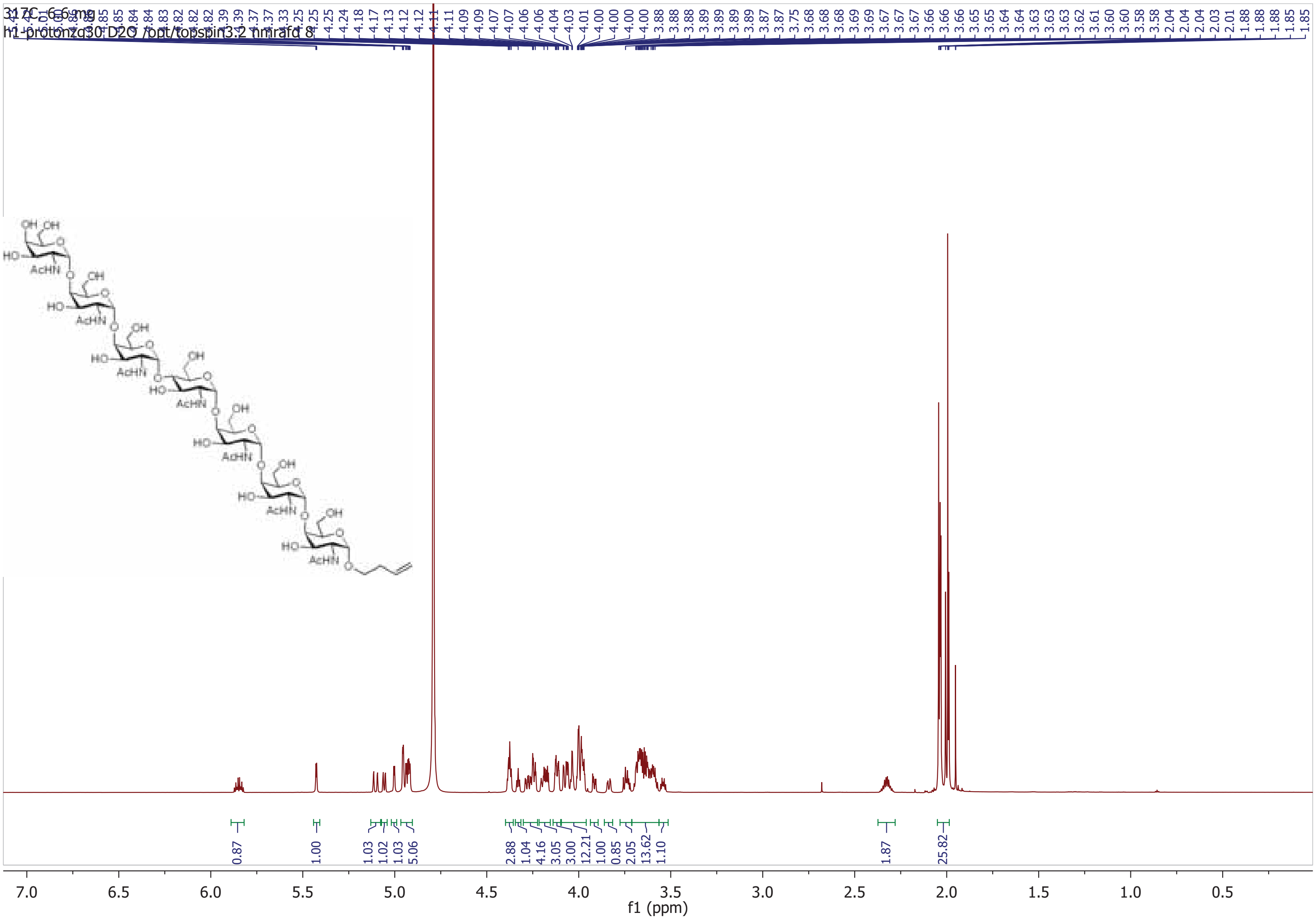

317C_6.6mg
h1-protonzg30.D2O /opt/topspin3.2 nmrafd 8
3.85 3.85 3.84 3.83 3.82 3.82 3.82 4.39 4.39 4.37 4.37 4.33 4.25 4.25 4.24 4.18 4.17 4.13 4.12 4.12 4.11 4.11 4.09 4.09 4.07 4.06 4.06 4.04 4.03 4.01 4.00 4.00 4.00 4.00 3.88 3.88 3.89 3.89 3.89 3.89 3.87 3.87 3.75 3.68 3.68 3.69 3.69 3.67 3.67 3.67 3.66 3.66 3.66 3.65 3.65 3.64 3.63 3.63 3.63 3.62 3.61 3.60 3.60 3.58 3.58 2.04 2.04 2.03 2.01 1.88 1.88 1.88 1.85 1.85
OH OH
HO
AcHN
O
OH
AcHN
0.87
1.00
1.03
1.02
1.03
5.06
2.88
1.04
4.16
3.05
3.00
12.21
1.00
0.85
2.05
13.62
1.10
1.87
25.82
7.0 6.5 6.0 5.5 5.0 4.5 4.0 3.5 3.0 2.5 2.0 1.5 1.0 0.5
f1 (ppm)

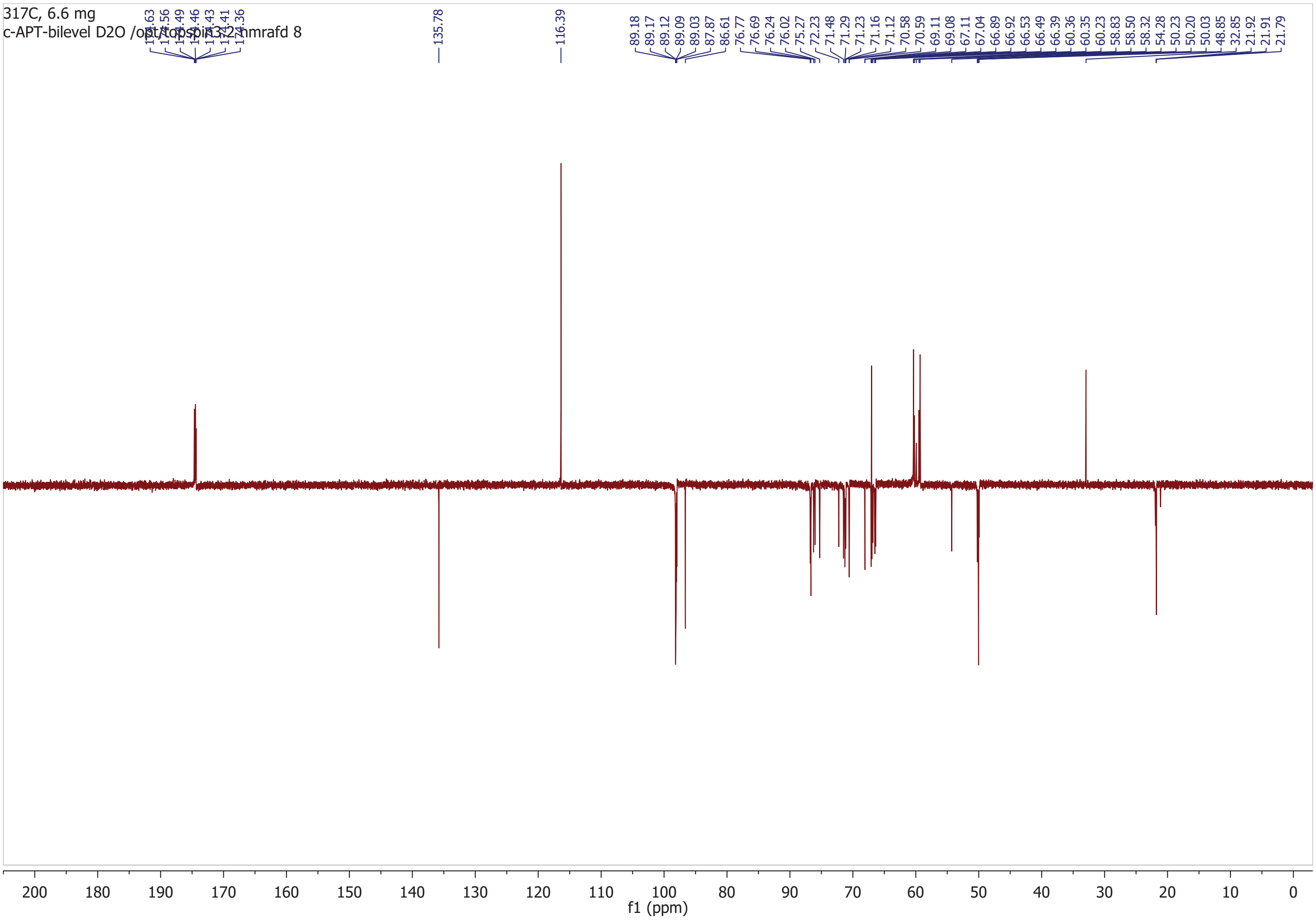
317C, 6.6 mg
c-APT-bilevel D2O /opt/topspin3.2 nmrafd 8
174.56
174.49
174.46
174.43
174.41
174.36
135.78
116.39
89.18
89.17
89.12
89.09
89.03
87.87
86.61
76.77
76.69
76.24
76.02
75.27
72.23
71.48
71.29
71.23
71.16
71.12
70.58
70.59
69.11
69.08
67.11
67.04
66.89
66.92
66.53
66.49
66.39
60.36
60.35
60.23
58.83
58.50
58.32
54.28
50.23
50.20
50.03
48.85
32.85
21.92
21.91
21.79
f1 (ppm)

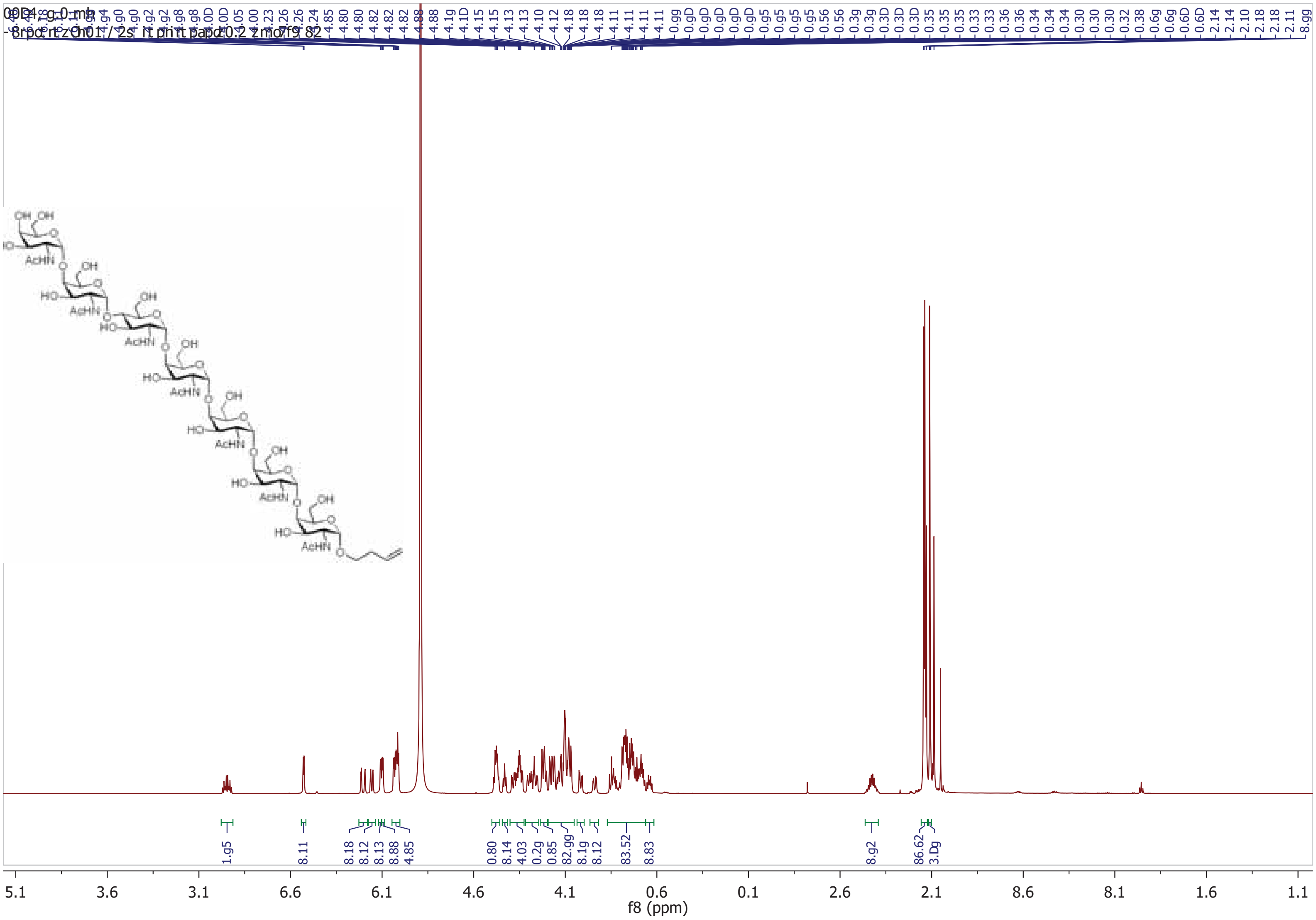
f8 (ppm)
AcHN
HO
OH

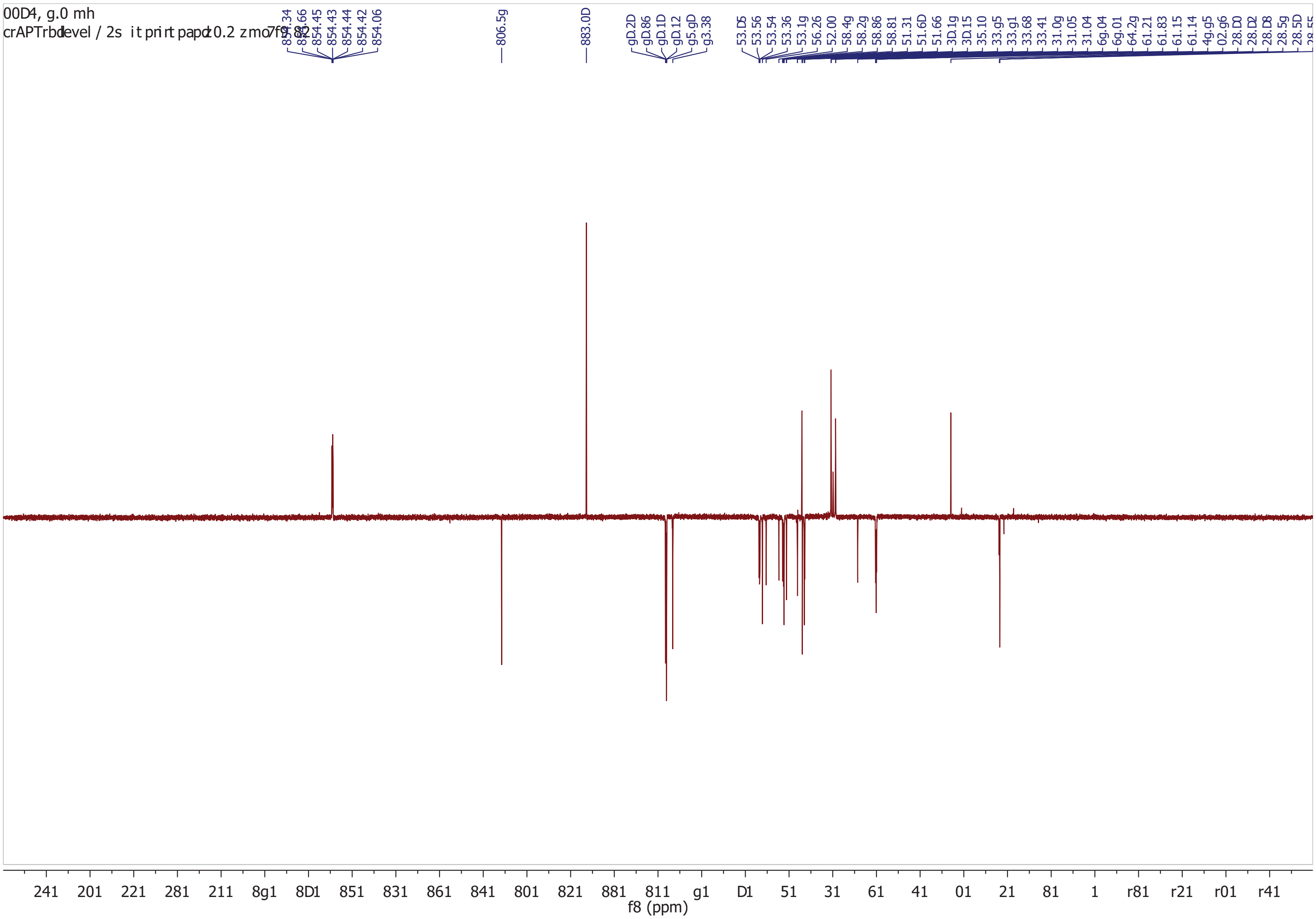

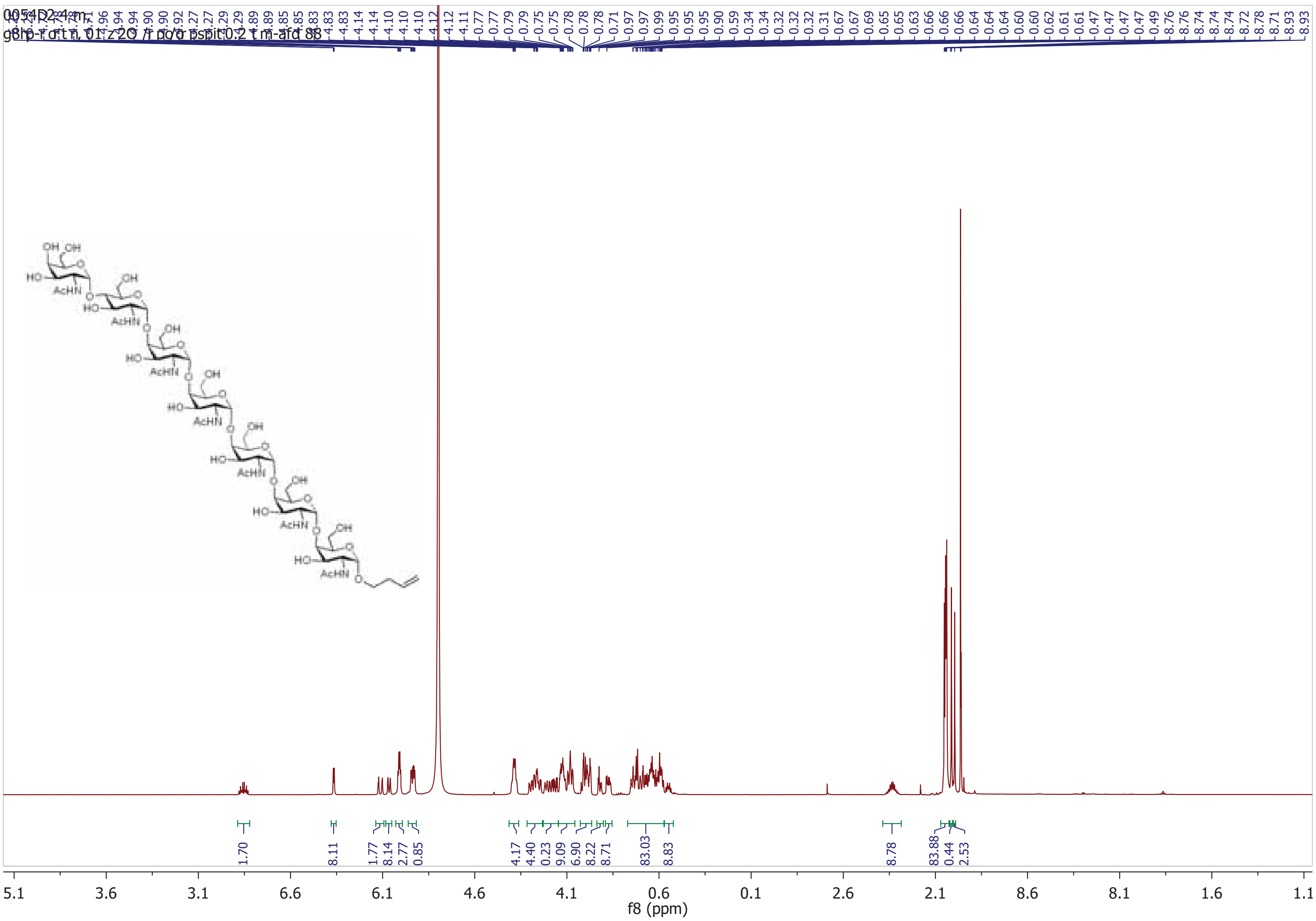

1.70
8.11
1.77
8.14
2.77
0.85
4.17
4.40
0.23
9.09
6.90
8.22
8.71
83.03
8.83
8.78
83.88
0.44
2.53
5.1
3.6
3.1
6.6
6.1
4.6
4.1
0.6
0.1
2.6
2.1
8.6
8.1
1.6
1.1
f8 (ppm)

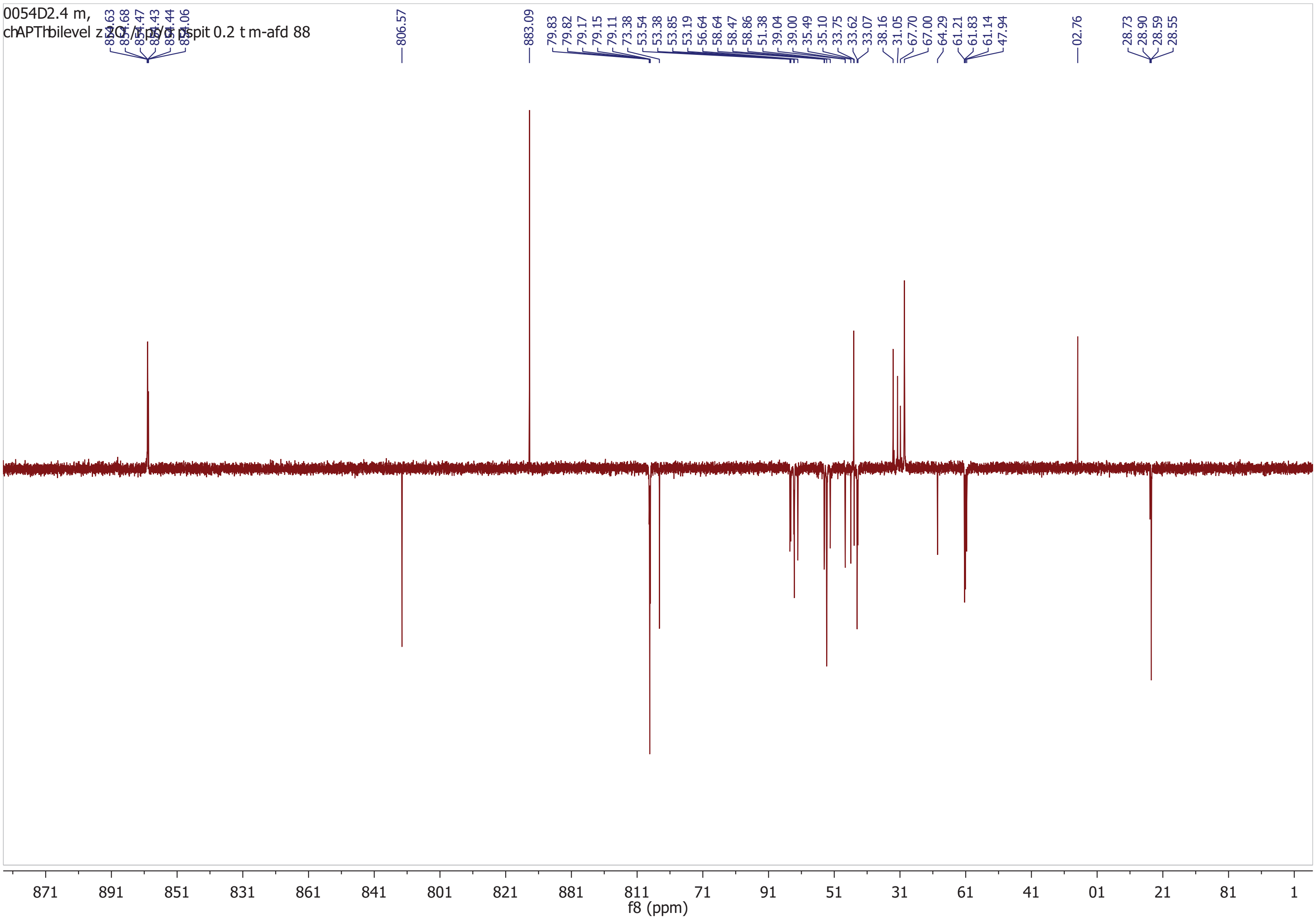
0054D2.4 m,
chAPTHbilevel z 2O /r pp/d pspit 0.2 t m-afd 88
854.63
854.68
854.47
854.43
854.44
854.06
806.57
883.09
79.83
79.82
79.17
79.15
79.11
73.38
53.54
53.38
53.85
53.19
56.64
58.64
58.47
58.86
51.38
39.04
39.00
35.49
35.10
33.75
33.62
33.07
38.16
31.05
67.70
67.00
64.29
61.21
61.83
61.14
47.94
02.76
28.73
28.90
28.59
28.55
871
891
851
831
861
841
801
821
881
811
71
91
51
31
61
41
01
21
81
1
f8 (ppm)

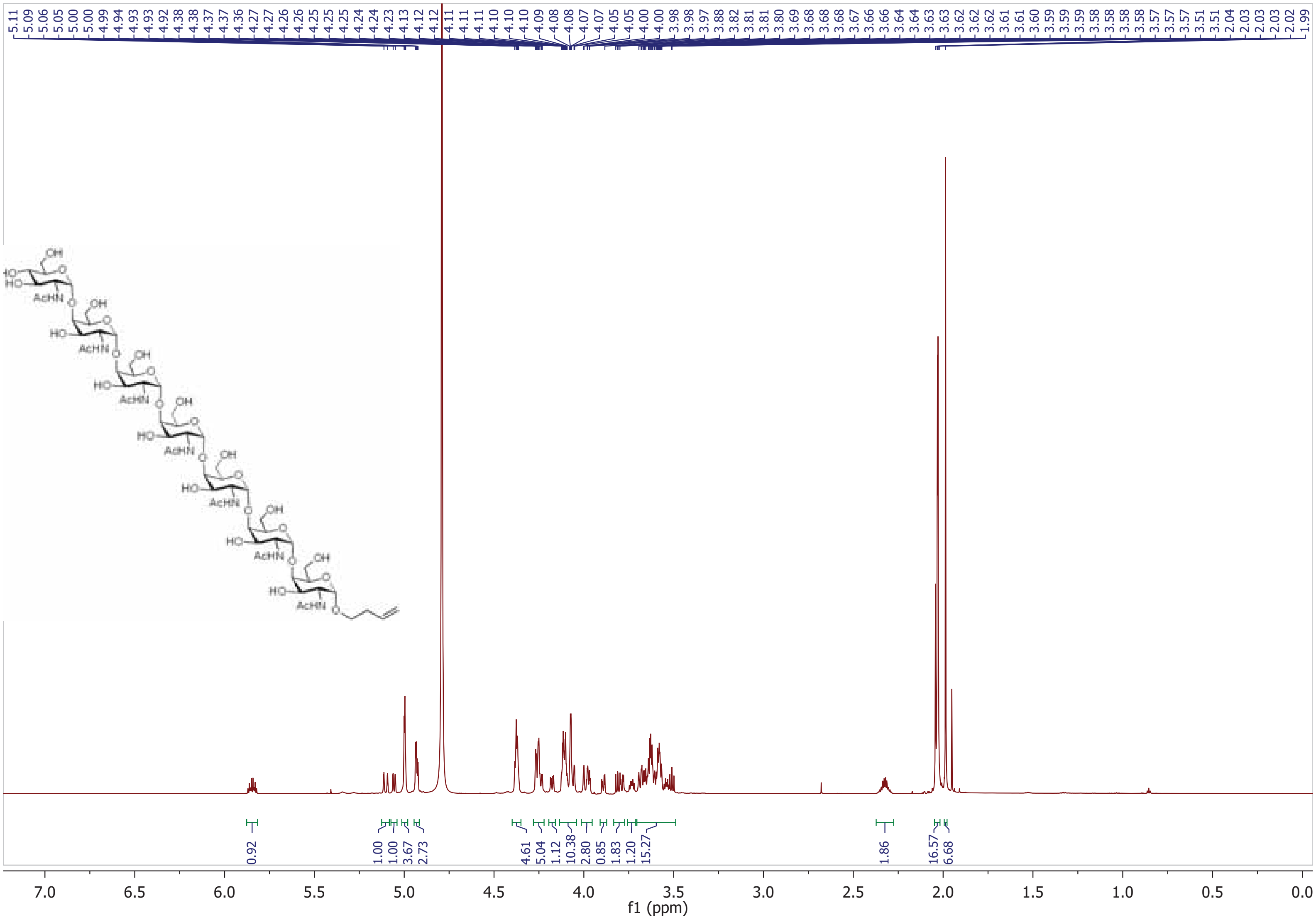
5.11 5.09 5.06 5.05 5.00 5.00 4.99 4.94 4.93 4.93 4.92 4.38 4.38 4.37 4.36 4.27 4.27 4.26 4.25 4.25 4.25 4.24 4.23 4.13 4.12 4.12 4.11 4.11 4.11 4.10 4.10 4.09 4.08 4.08 4.07 4.05 4.05 4.00 4.00 3.98 3.97 3.88 3.82 3.81 3.81 3.80 3.69 3.68 3.68 3.67 3.66 3.66 3.64 3.64 3.63 3.63 3.62 3.62 3.61 3.61 3.60 3.59 3.59 3.58 3.58 3.58 3.58 3.57 3.57 3.51 3.51 2.04 2.03 2.03 2.02 1.99
0.92
1.00
1.00
3.67
2.73
4.61
5.04
1.12
10.38
2.80
0.85
1.83
1.20
15.27
1.86
16.57
6.68
7.0 6.5 6.0 5.5 5.0 4.5 4.0 3.5 3.0 2.5 2.0 1.5 1.0 0.5 0.0
f1 (ppm)
HO
OH
AcHN

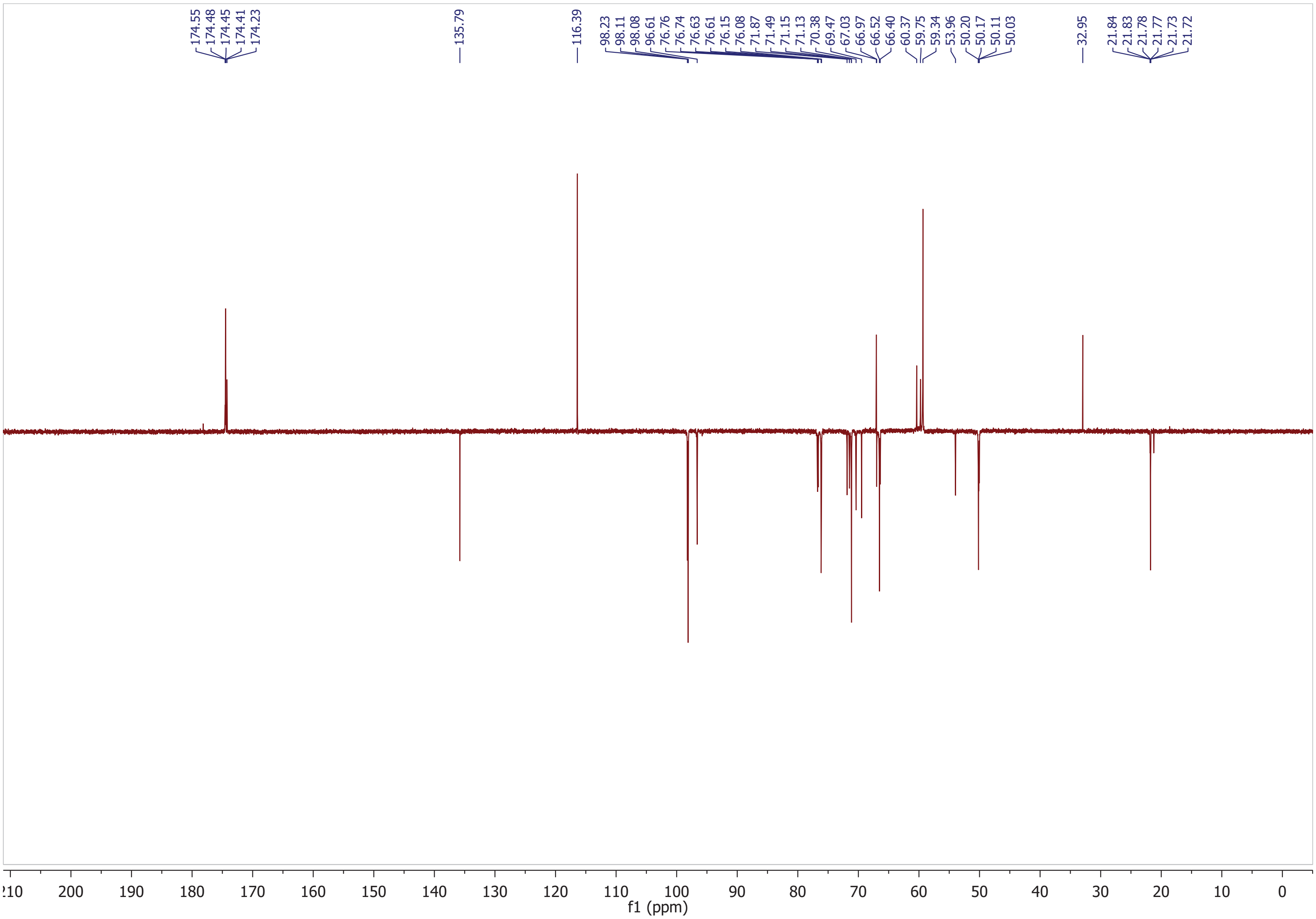

174.55
174.48
174.45
174.41
174.23
135.79
116.39
98.23
98.11
98.08
96.61
76.76
76.74
76.63
76.61
76.15
76.08
71.87
71.49
71.15
71.13
70.38
69.47
67.03
66.97
66.52
66.40
60.37
59.75
59.34
53.96
50.20
50.17
50.11
50.03
32.95
21.84
21.83
21.78
21.77
21.73
21.72
210 200 190 180 170 160 150 140 130 120 110 100 90 80 70 60 50 40 30 20 10 0
f1 (ppm)

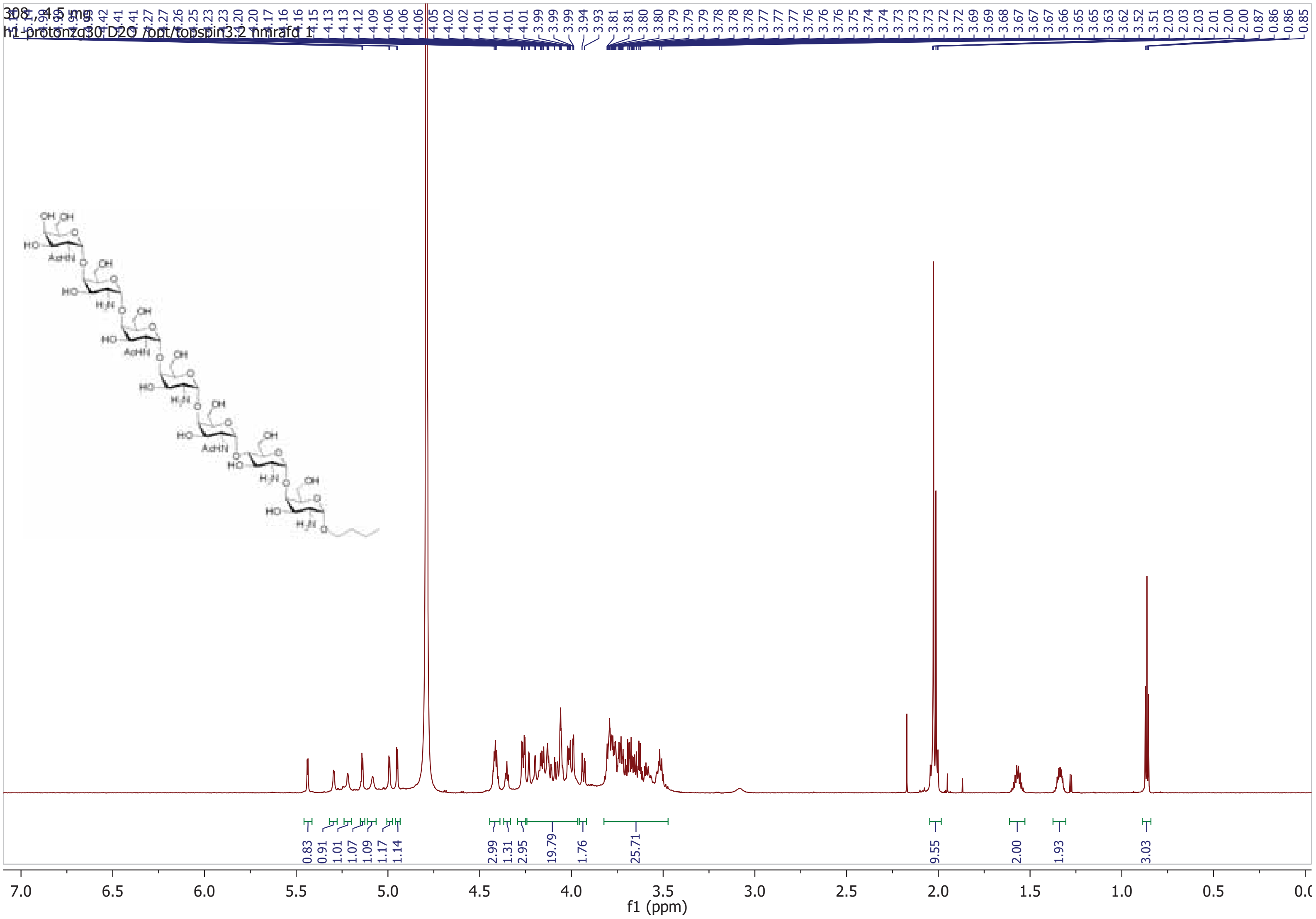

308, 4.5 mg
h1-protonzg30 D2O /opt/topspin3.2 nmrafd 1
0.83 0.91 1.01 1.07 1.09 1.17 1.14
2.99 1.31 2.95 19.79 1.76 25.71
9.55 2.00 1.93 3.03
7.0 6.5 6.0 5.5 5.0 4.5 4.0 3.5 3.0 2.5 2.0 1.5 1.0 0.5
f1 (ppm)

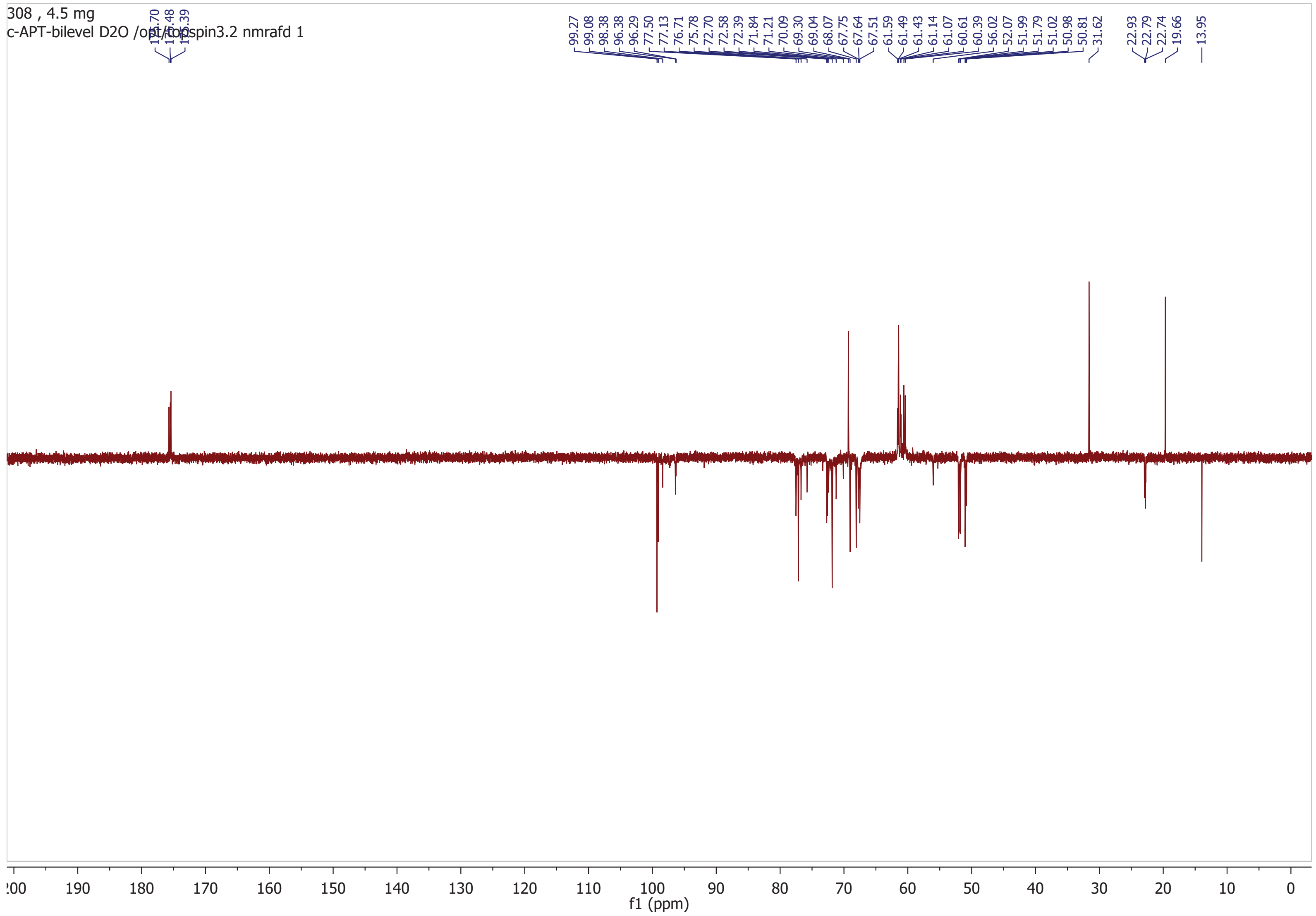
308 , 4.5 mg
c-APT-bilevel D2O /opt/topspin3.2 nmrafd 1
175.70
175.48
175.39
99.27
99.08
98.38
96.38
96.29
77.50
77.13
76.71
75.78
72.70
72.58
72.39
71.84
71.21
70.09
69.30
69.04
68.07
67.75
67.64
67.51
61.59
61.49
61.43
61.14
61.07
60.61
60.39
56.02
52.07
51.99
51.79
51.02
50.98
50.81
31.62
22.93
22.79
22.74
19.66
13.95
f1 (ppm)

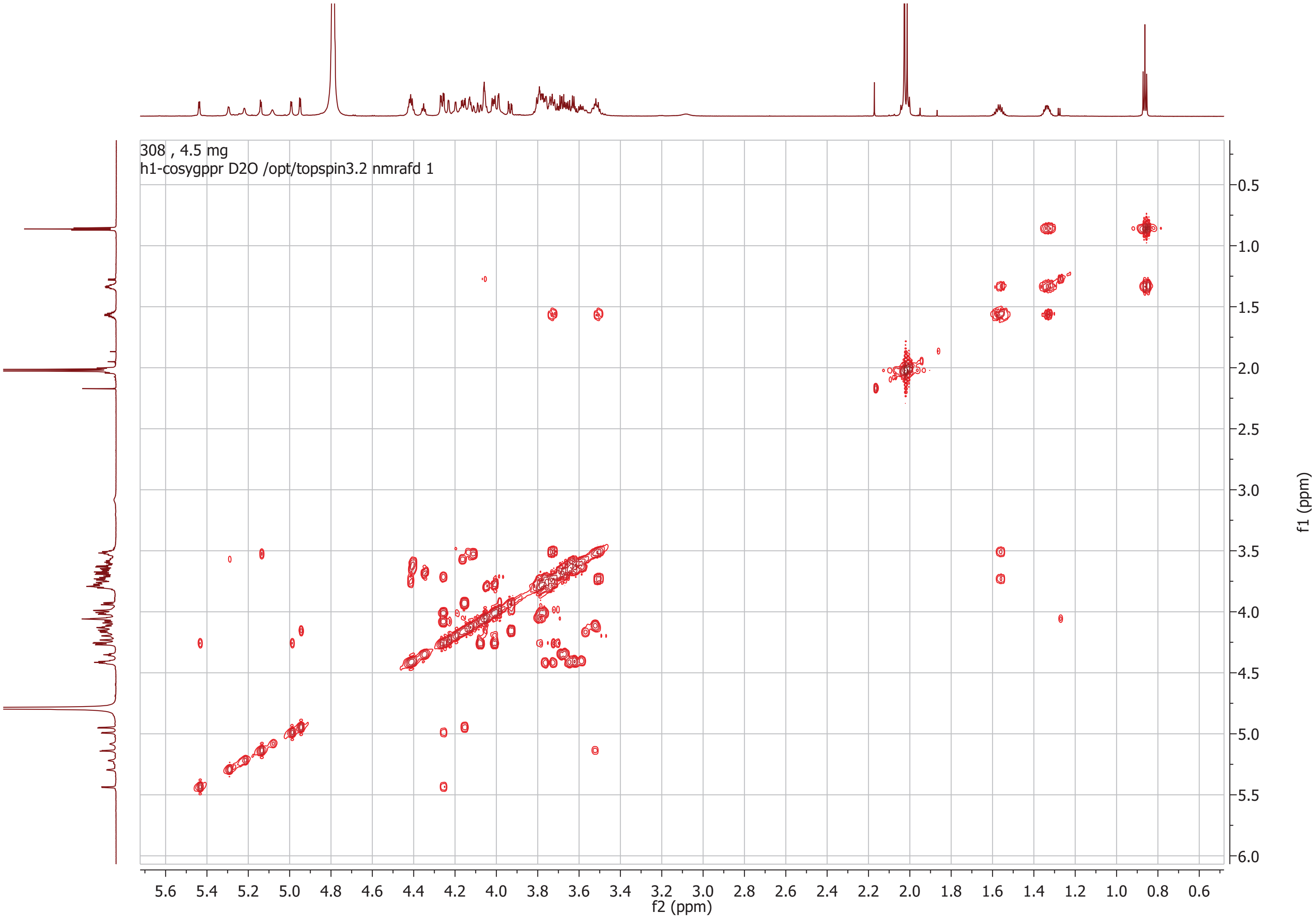

308 , 4.5 mg
h1-cosygppr D2O /opt/topspin3.2 nmrafd 1
f2 (ppm)
f1 (ppm)

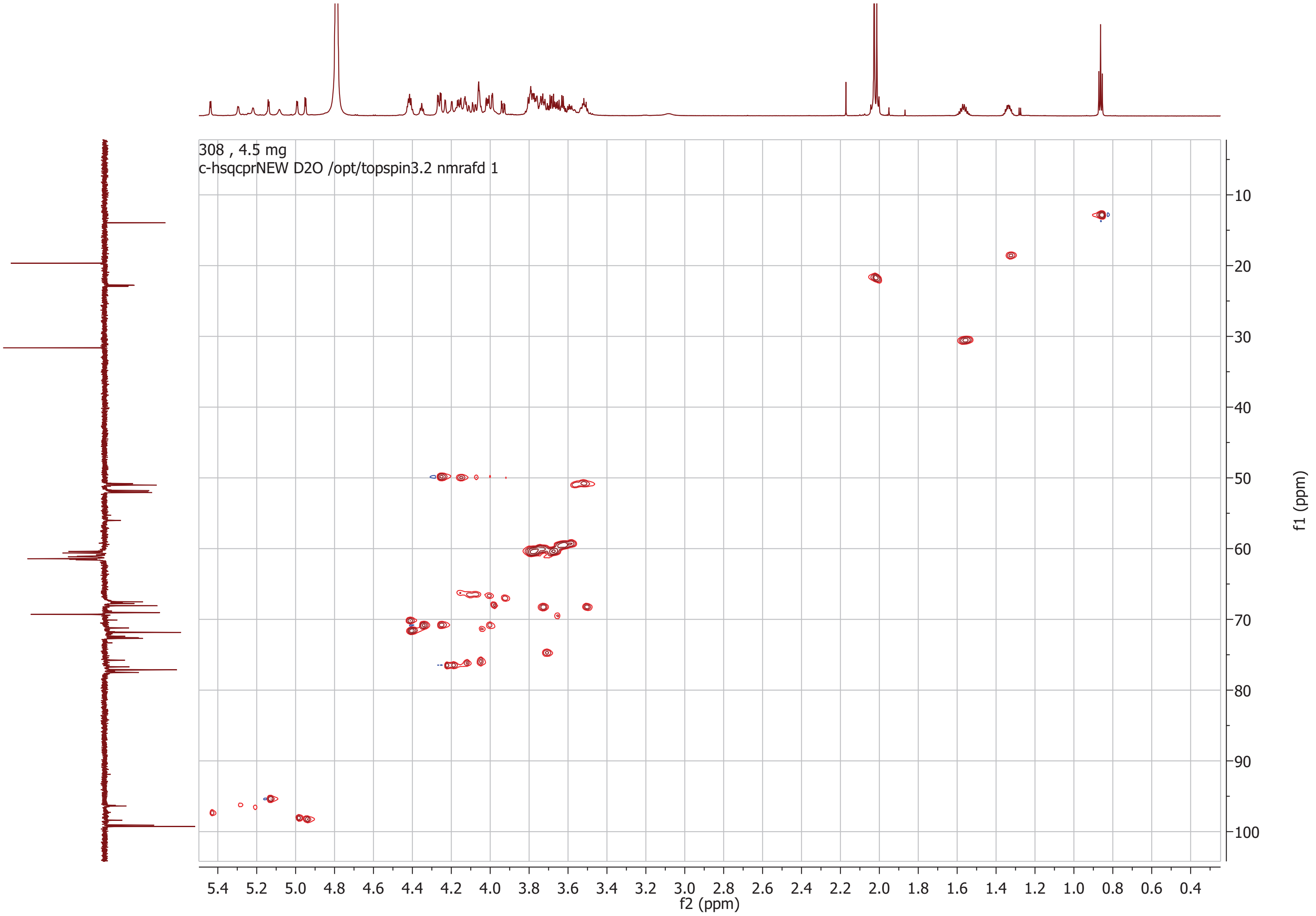

308 , 4.5 mg
c-hsqcprNEW D2O /opt/topspin3.2 nmrafd 1
f1 (ppm)
f2 (ppm)

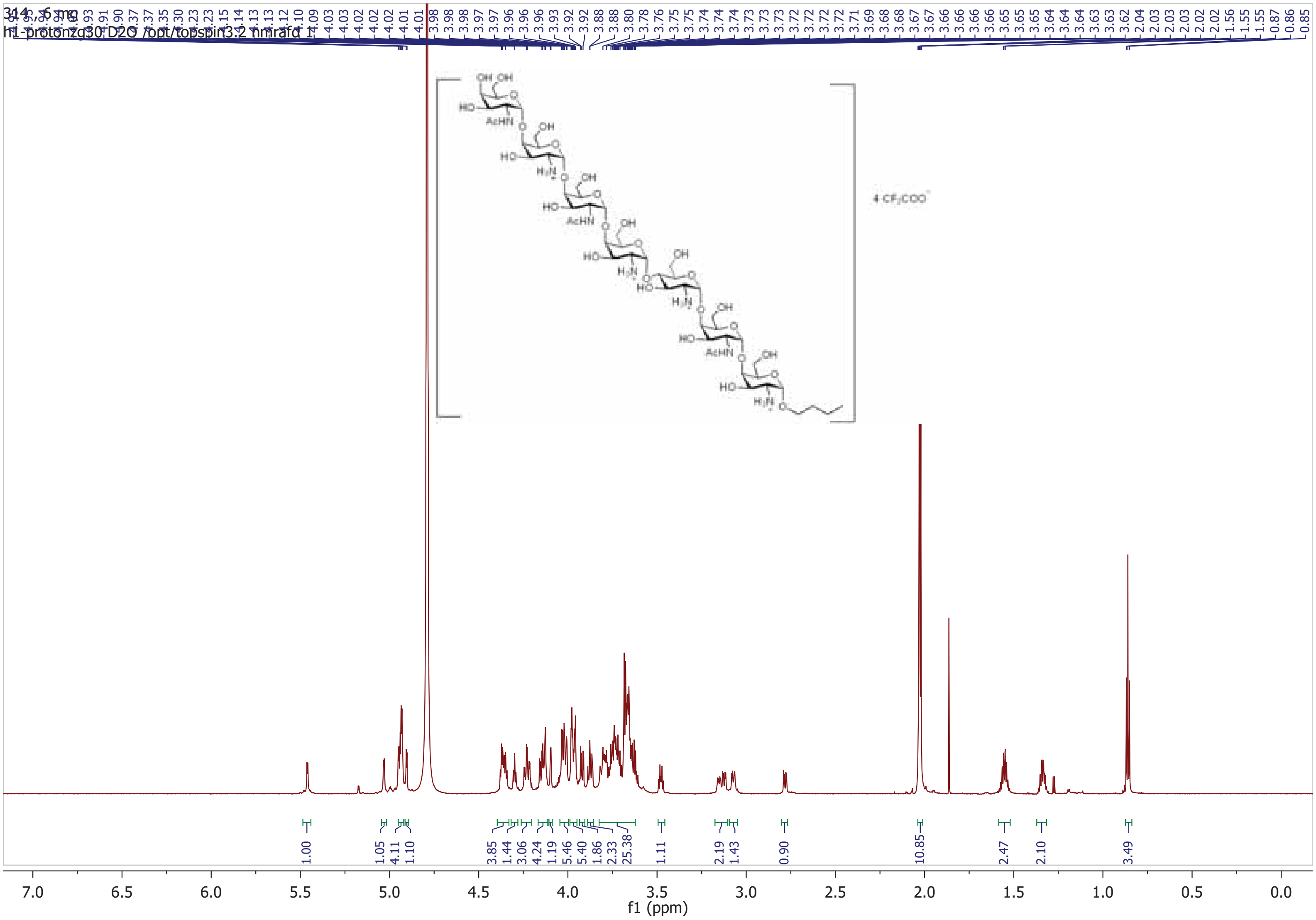

344 6 mg
h1-protonzg30 D2O /opt/topspin3.2 nmrafd 1
4.01 3.98 3.98 3.98 3.97 3.97 3.96 3.96 3.96 3.93 3.92 3.92 3.88 3.88 3.80 3.78 3.76 3.75 3.74 3.74 3.74 3.73 3.73 3.73 3.72 3.72 3.72 3.72 3.71 3.69 3.68 3.68 3.67 3.67 3.66 3.66 3.66 3.66 3.65 3.65 3.65 3.64 3.64 3.63 3.63 3.62 2.04 2.03 2.03 2.03 2.02 2.02 1.56 1.55 1.55 0.87 0.86 0.85
4 CF3COO-
1.00 1.05 4.11 1.10 3.85 1.44 3.06 4.24 1.19 5.46 5.40 1.86 2.33 25.38 1.11 2.19 1.43 0.90 10.85 2.47 2.10 3.49
7.0 6.5 6.0 5.5 5.0 4.5 4.0 3.5 3.0 2.5 2.0 1.5 1.0 0.5 0.0
f1 (ppm)

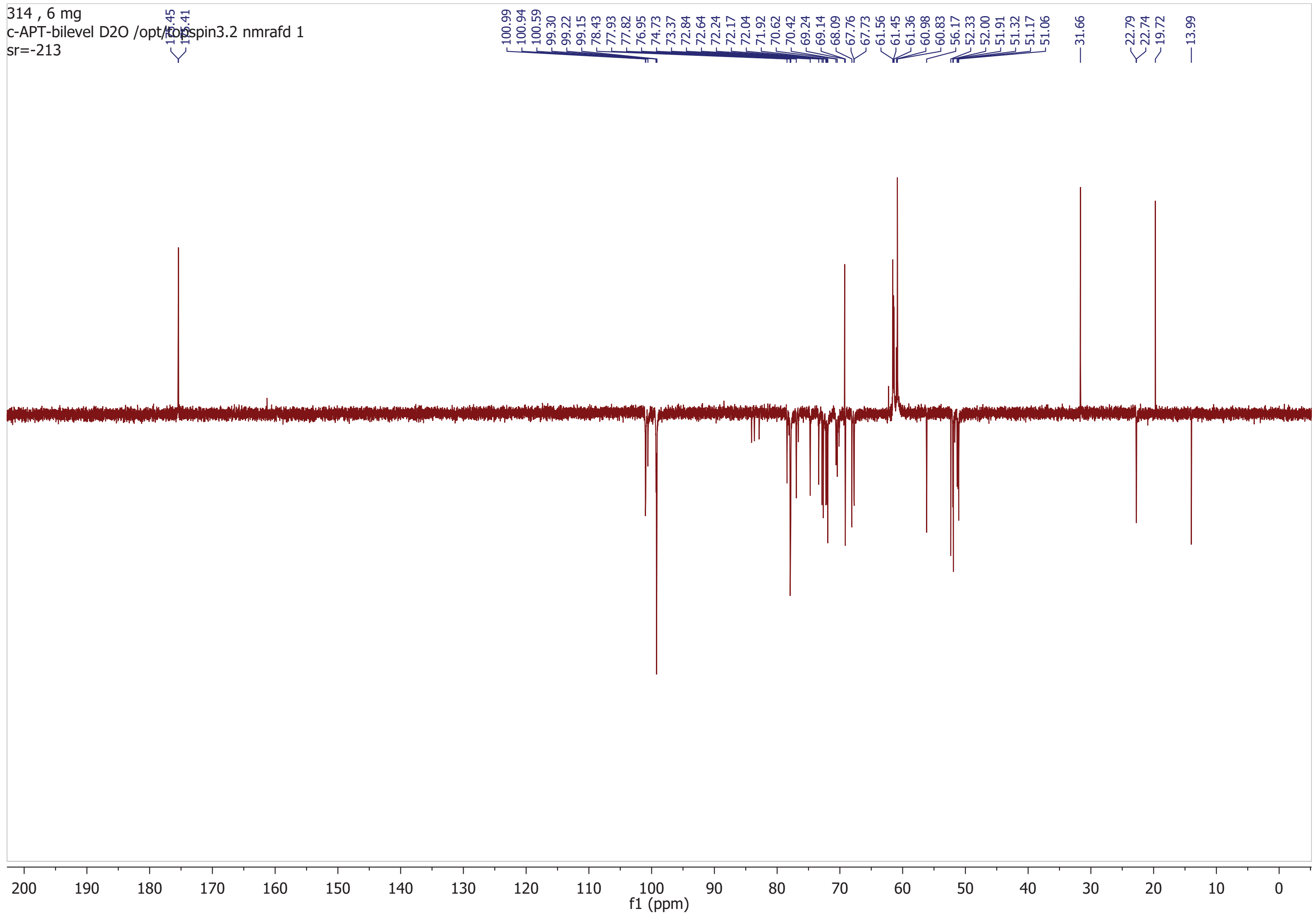
314 , 6 mg
c-APT-bilevel D2O /opt/topspin3.2 nmrafd 1
sr=-213
175.45
175.41
100.99
100.94
100.59
99.30
99.22
99.15
78.43
77.93
77.82
76.95
74.73
73.37
72.84
72.64
72.24
72.17
72.04
71.92
70.62
70.42
69.24
69.14
68.09
67.76
67.73
61.56
61.45
61.36
60.98
60.83
56.17
52.33
52.00
51.91
51.32
51.17
51.06
31.66
22.79
22.74
19.72
13.99
200 190 180 170 160 150 140 130 120 110 100 90 80 70 60 50 40 30 20 10 0
f1 (ppm)

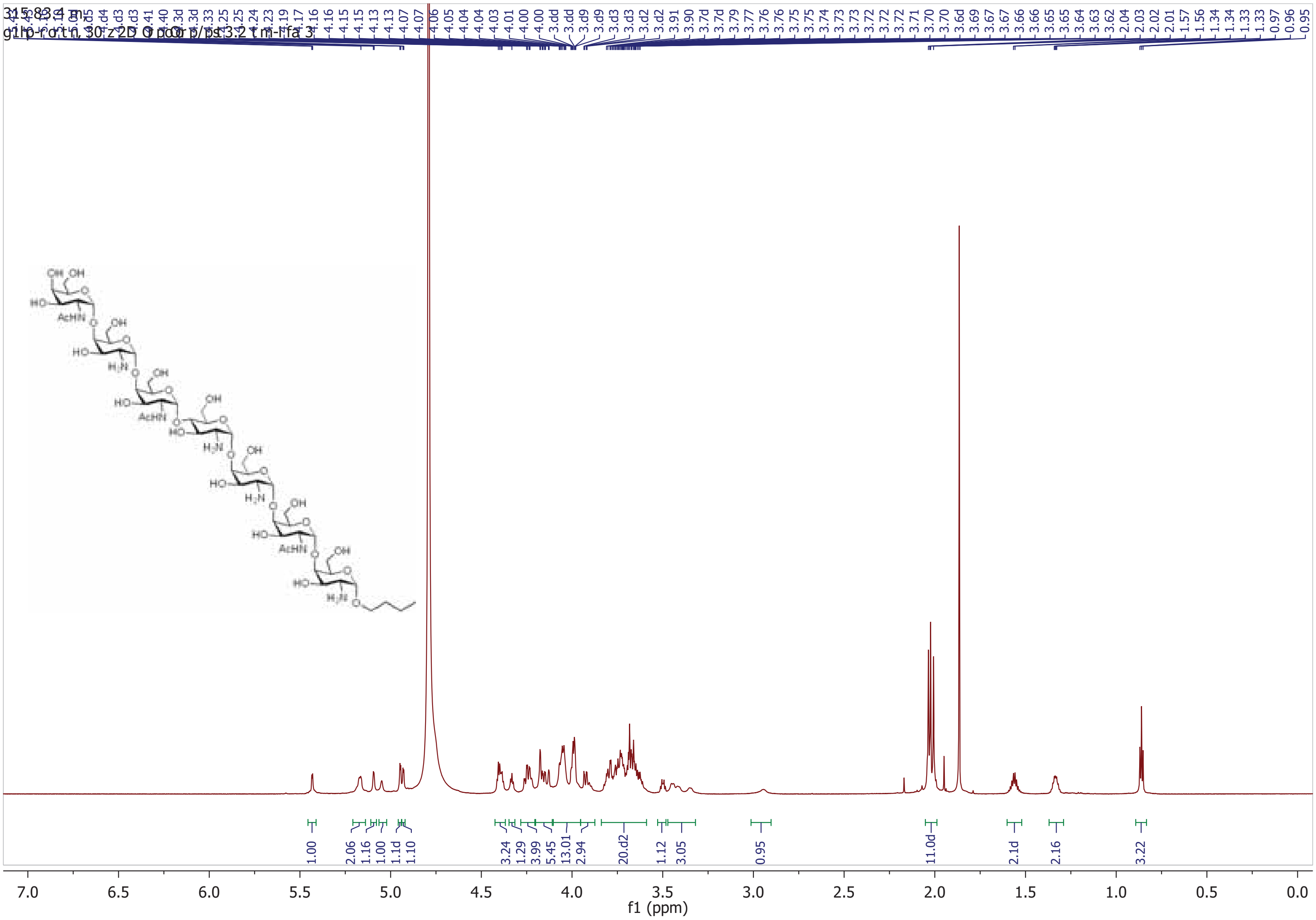

1.00
2.06
1.16
1.00
1.10
3.24
1.29
3.99
5.45
13.01
2.94
1.12
3.05
0.95
2.16
3.22
7.0
6.5
6.0
5.5
5.0
4.5
4.0
3.5
3.0
2.5
2.0
1.5
1.0
0.5
0.0
f1 (ppm)

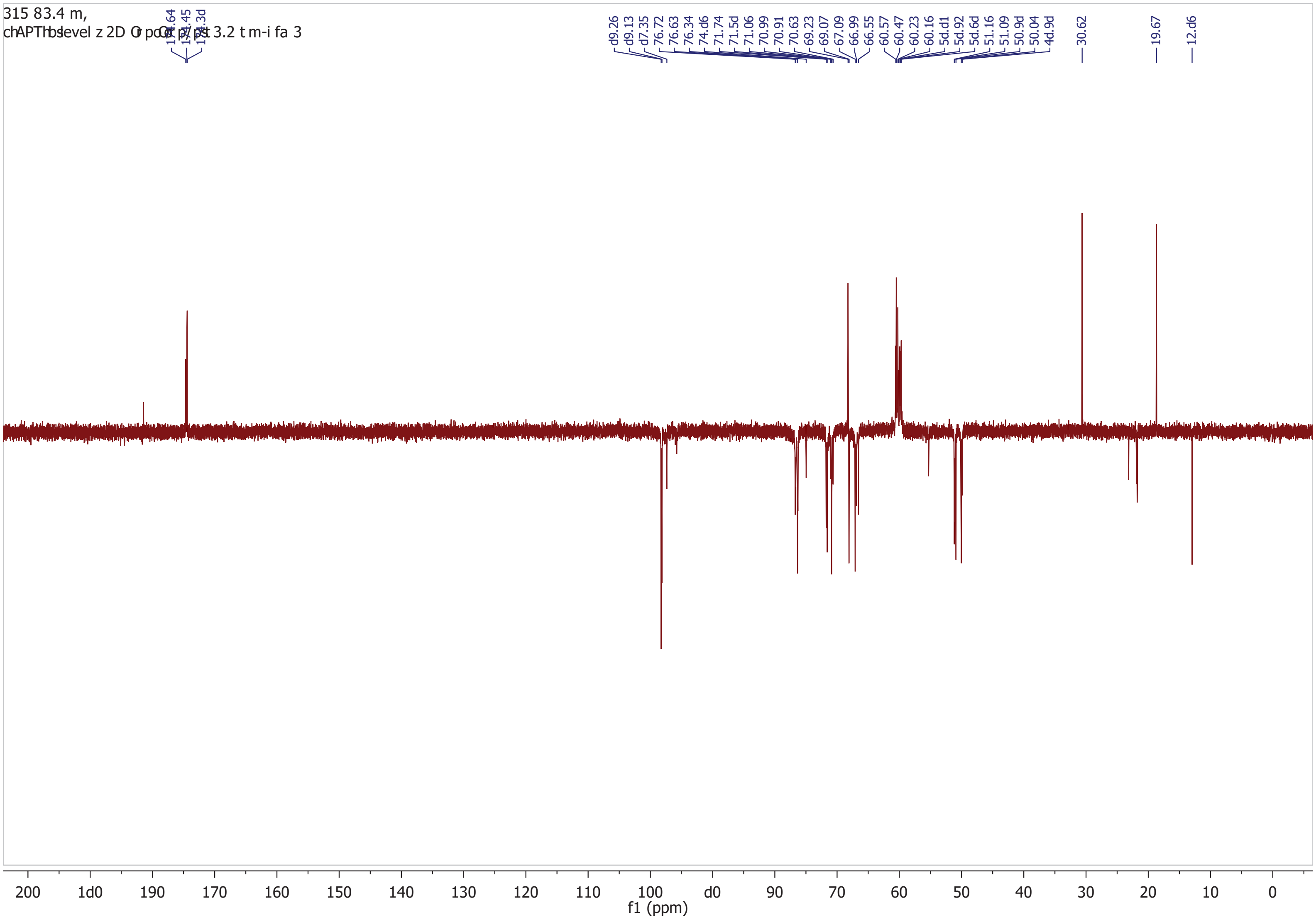
30.62
19.67
f1 (ppm)

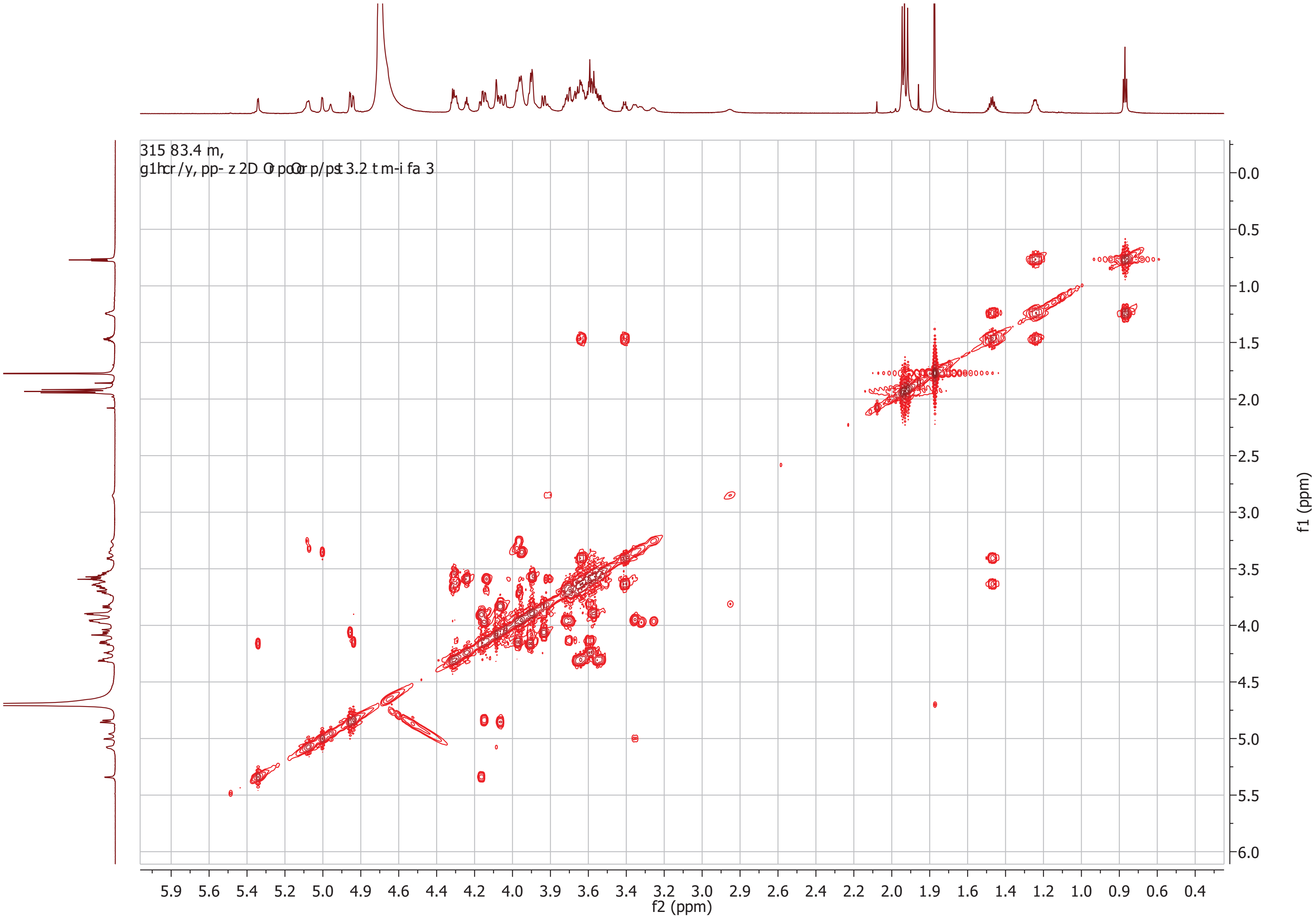
315 83.4 m,
g1hcr /y, pp- z 2D Or poOr p/pst 3.2 t m-i fa 3
f1 (ppm)
f2 (ppm)

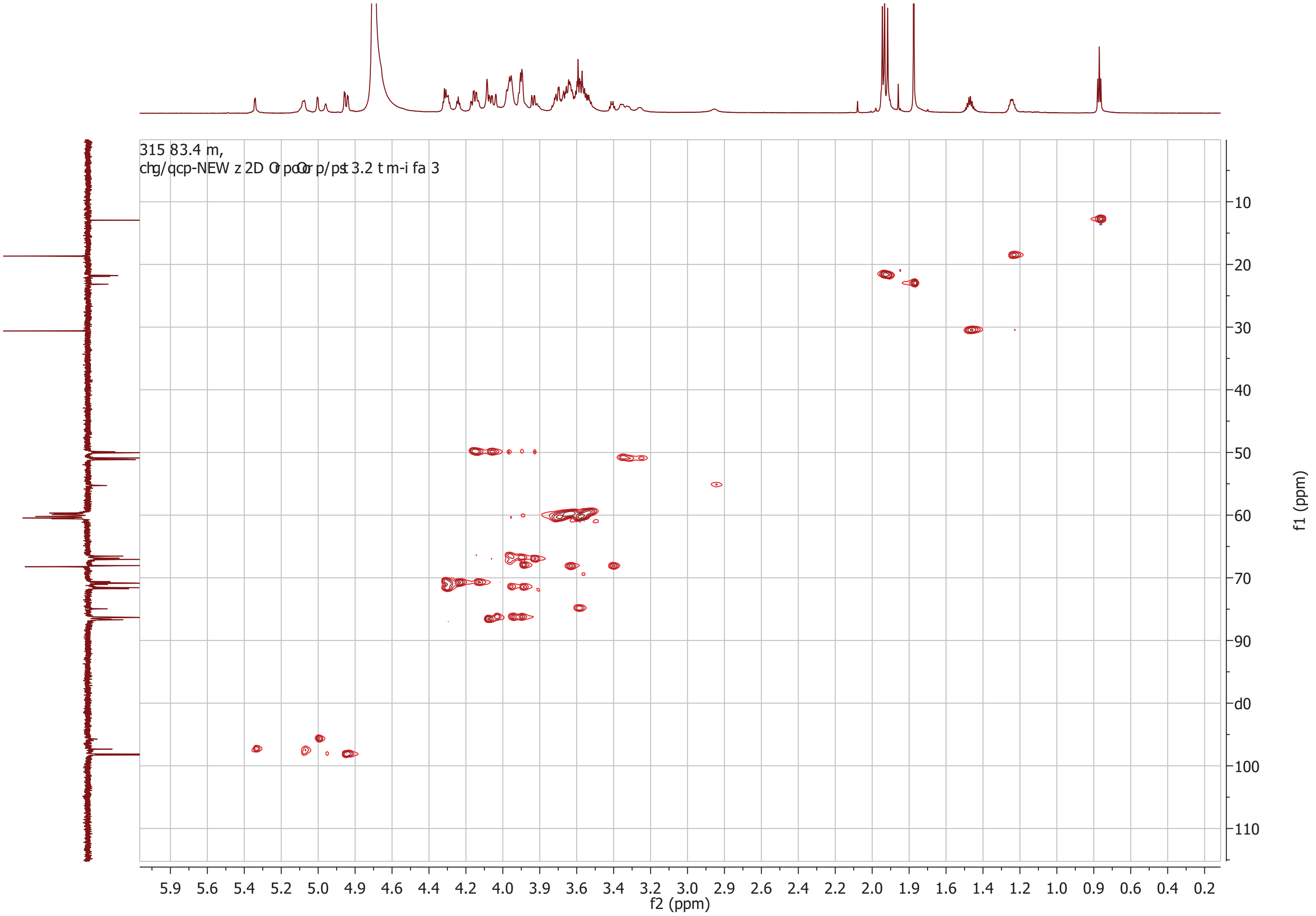

315 83.4 m,
chg/qcp-NEW z 2D Or poOr p/pst 3.2 t m-i fa 3
f2 (ppm)
f1 (ppm)

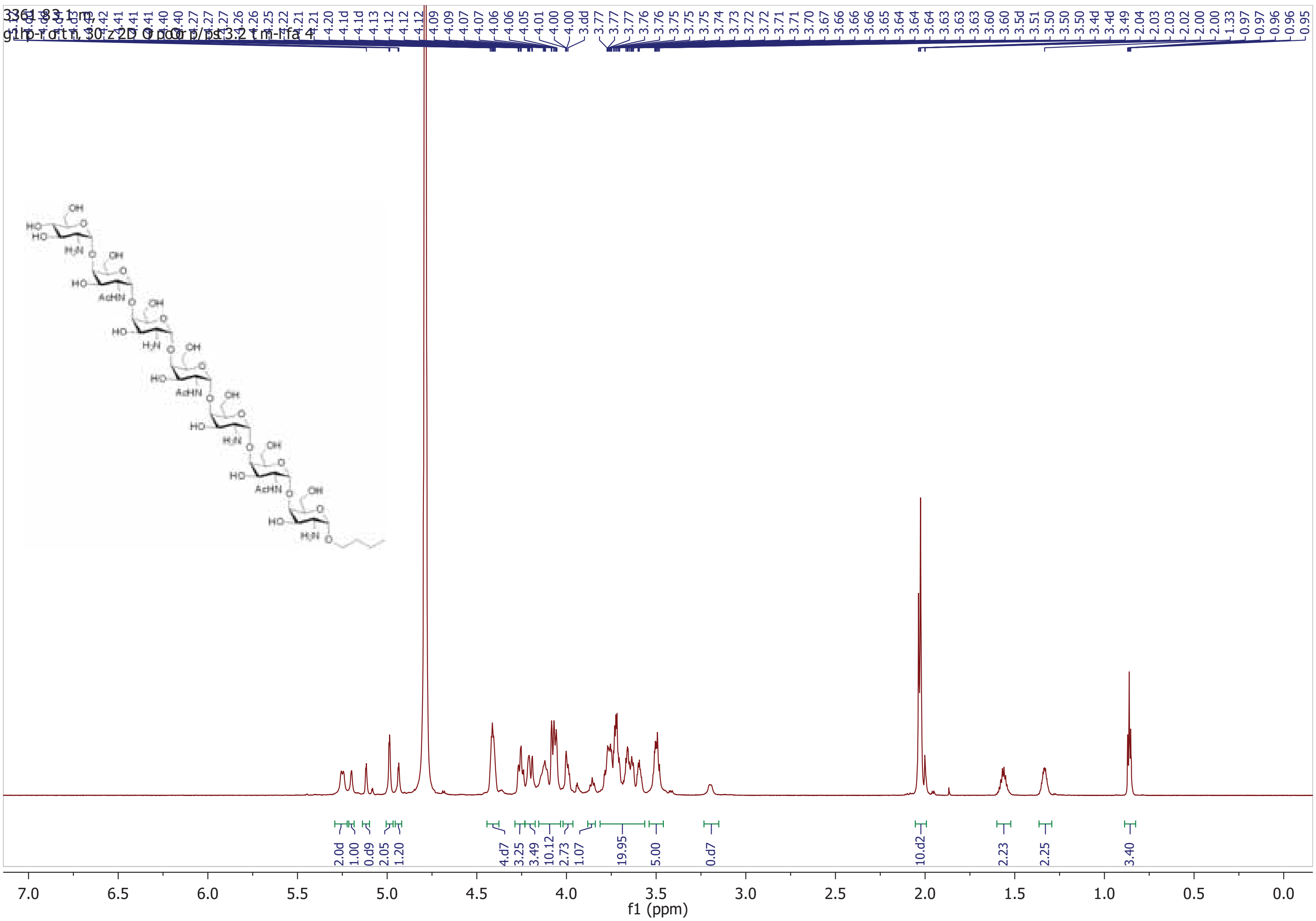
f1 (ppm)
7.0 6.5 6.0 5.5 5.0 4.5 4.0 3.5 3.0 2.5 2.0 1.5 1.0 0.5 0.0

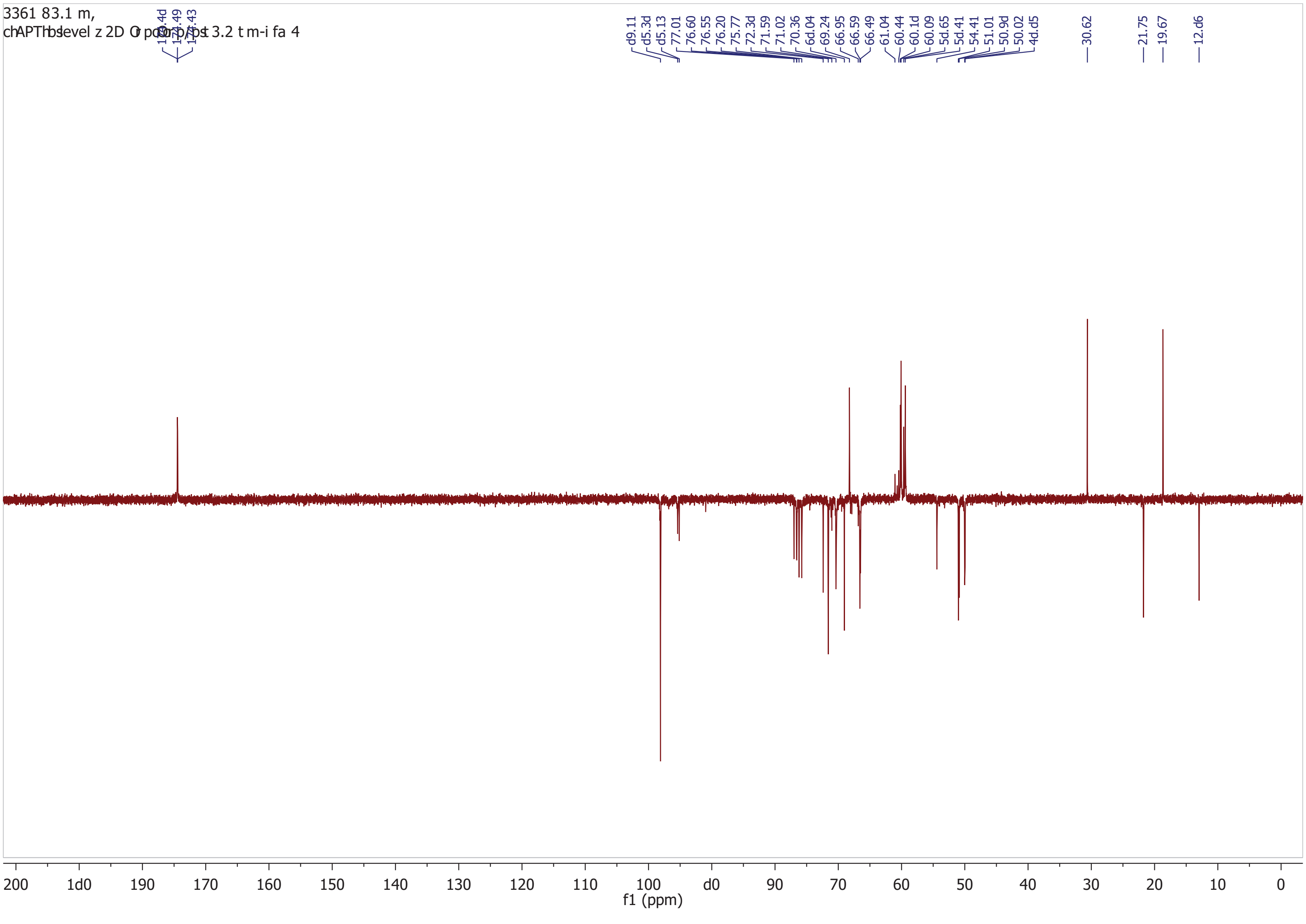
3361 83.1 m,
chAPThbslevel z 2D Or pocor p/ps t 3.2 t m-i fa 4
174.49
174.43
d9.11
d5.3d
d5.13
77.01
76.60
76.55
76.20
75.77
72.3d
71.59
71.02
70.36
6d.04
69.24
66.95
66.59
66.49
61.04
60.44
60.1d
60.09
5d.65
5d.41
54.41
51.01
50.9d
50.02
4d.d5
30.62
21.75
19.67
12.d6
200 1d0 190 170 160 150 140 130 120 110 100 d0 90 70 60 50 40 30 20 10 0
f1 (ppm)

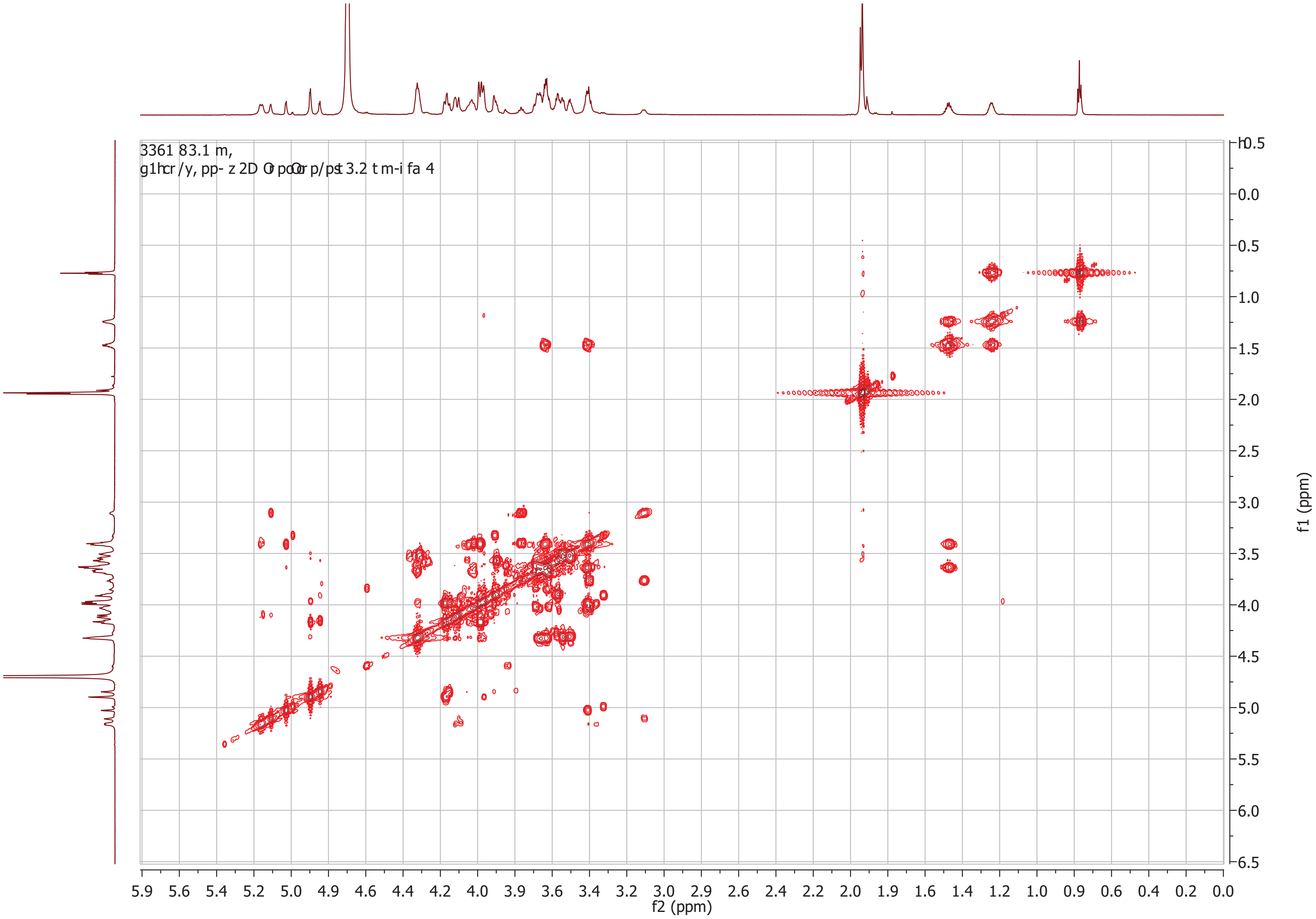
3361 83.1 m,
f2 (ppm)
f1 (ppm)

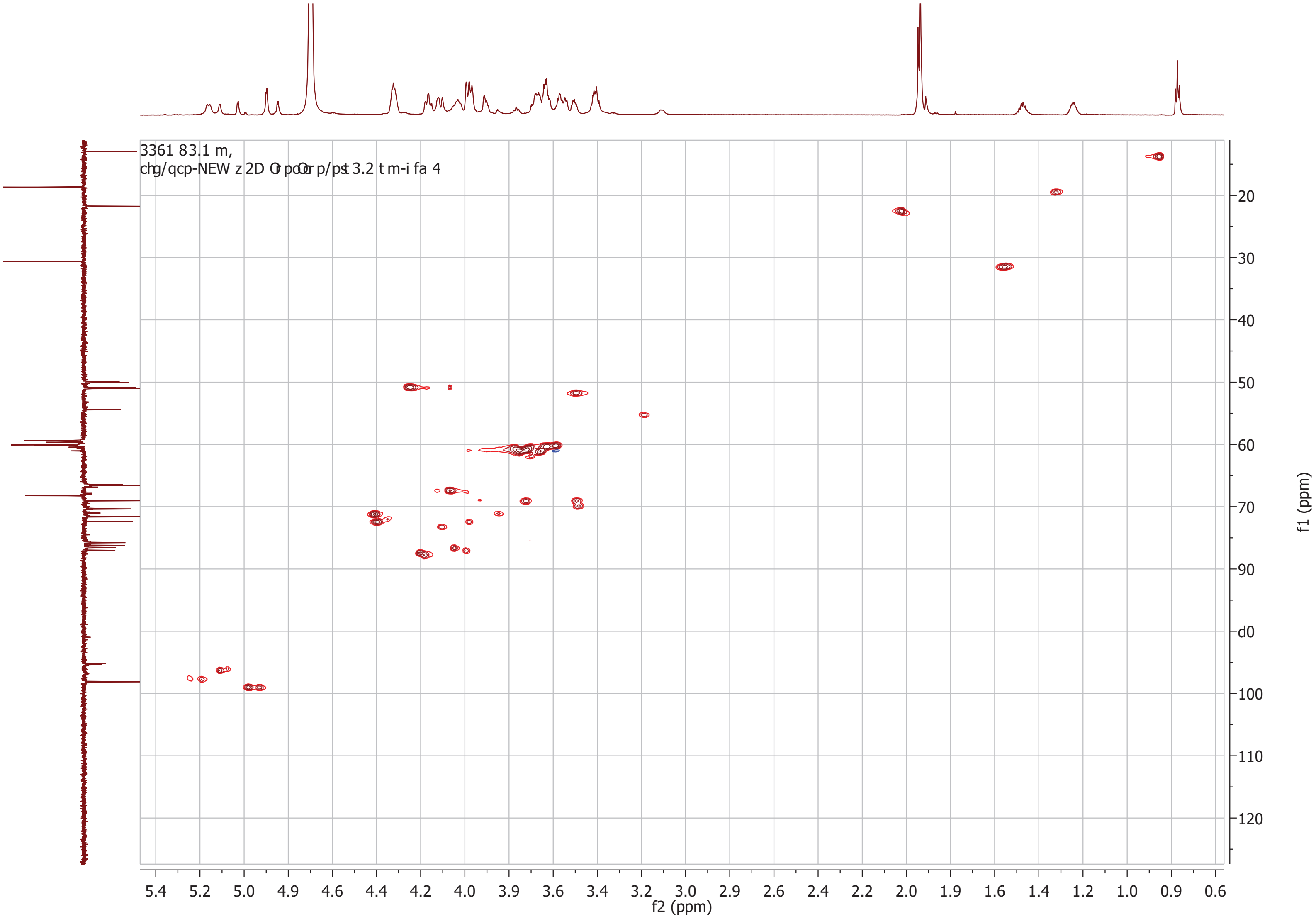
3361 83.1 m,
chg/qcp-NEW z 2D Or poOr p/pst 3.2 t m-i fa 4
5.4 5.2 5.0 4.9 4.6 4.4 4.2 4.0 3.9 3.6 3.4 3.2 3.0 2.9 2.6 2.4 2.2 2.0 1.9 1.6 1.4 1.2 1.0 0.9 0.6
f2 (ppm)
20 30 40 50 60 70 90 d0 100 110 120
f1 (ppm)

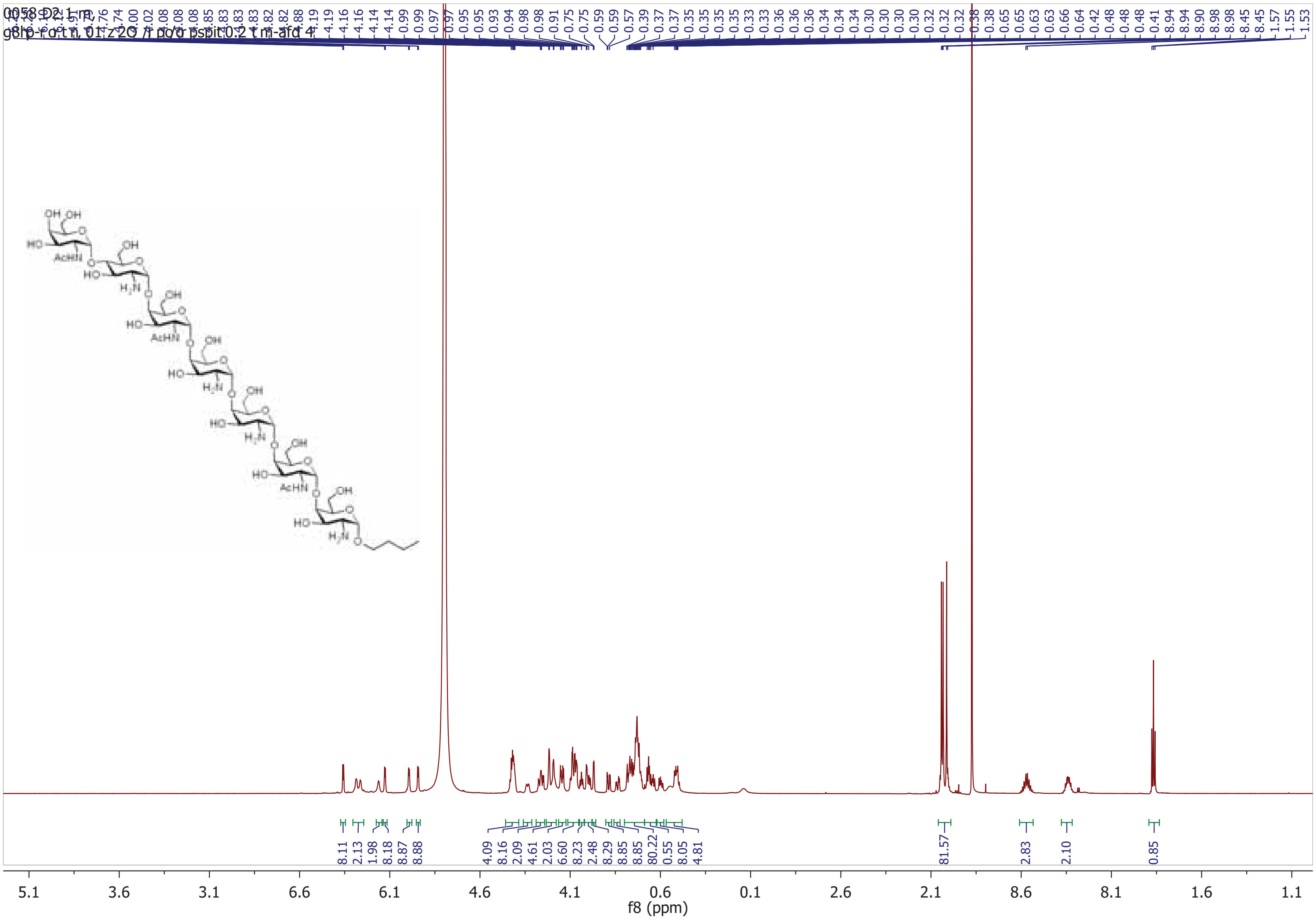

8.11
2.13
1.98
8.18
8.87
8.88
4.09
8.16
2.09
4.61
2.03
6.60
8.23
2.48
8.29
8.85
8.85
80.22
0.55
8.05
4.81
81.57
2.83
2.10
0.85
5.1
3.6
3.1
6.6
6.1
4.6
4.1
0.6
0.1
2.6
2.1
8.6
8.1
1.6
1.1
f8 (ppm)

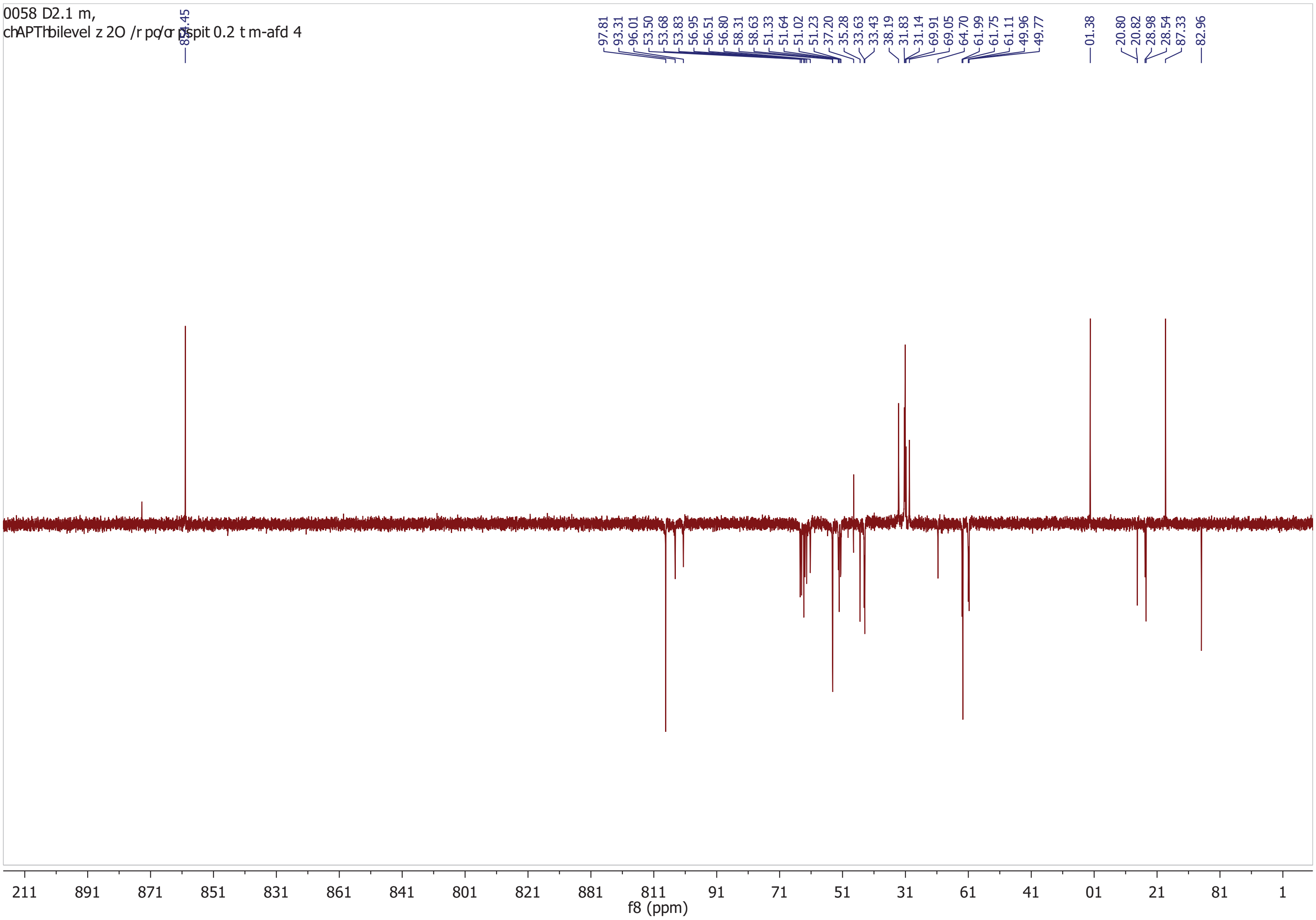

0058 D2.1 m,
chAPTHbilevel z 2O /r pq/σ pspit 0.2 t m-afd 4
83.45
97.81
93.31
96.01
53.50
53.68
53.83
56.95
56.51
56.80
58.31
58.63
51.33
51.64
51.02
51.23
37.20
35.28
33.63
33.43
38.19
31.83
31.14
69.91
69.05
64.70
61.99
61.75
61.11
49.96
49.77
01.38
20.80
20.82
28.98
28.54
87.33
82.96
211 891 871 851 831 861 841 801 821 881 811 91 71 51 31 61 41 01 21 81 1
f8 (ppm)

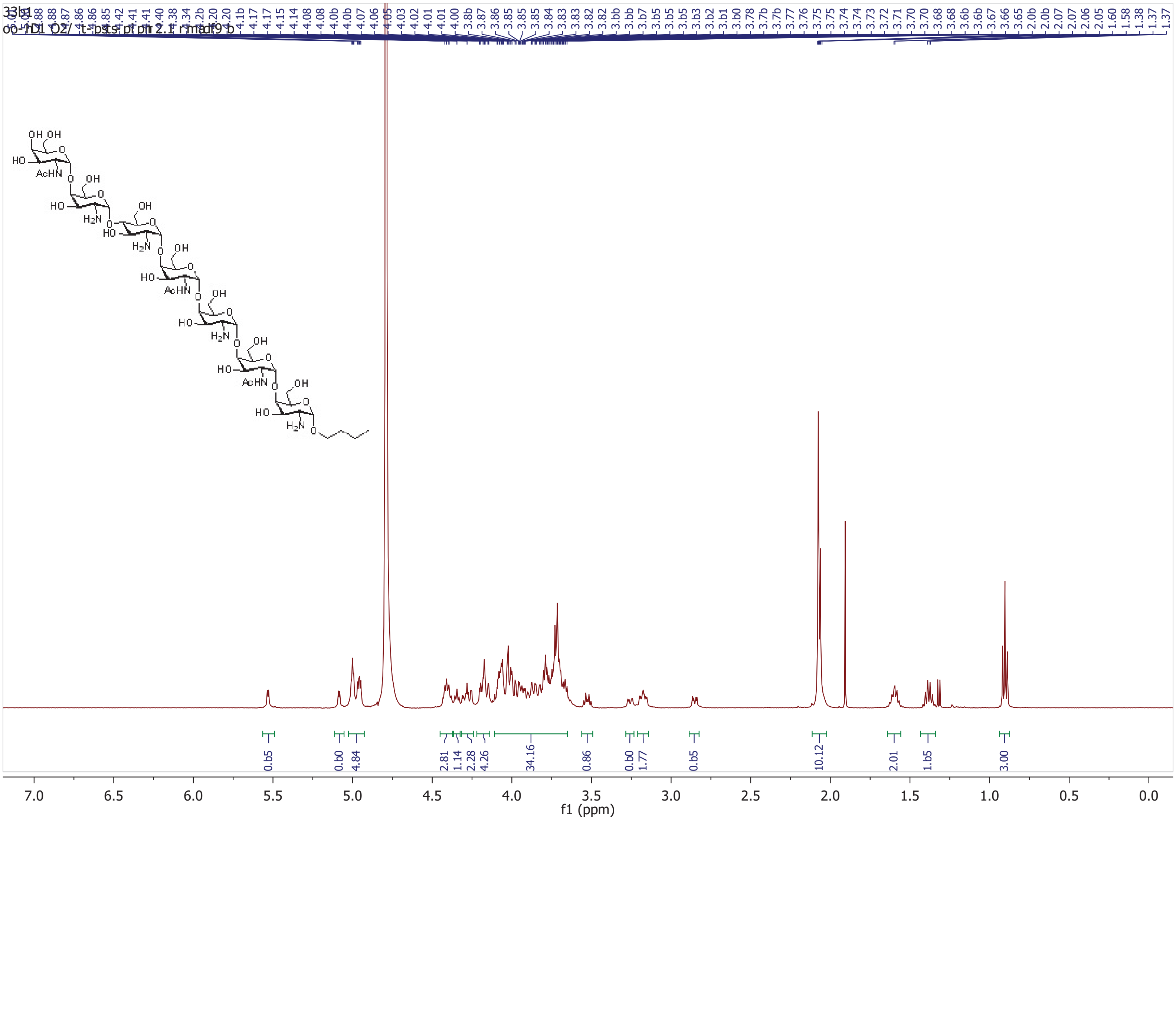
7.0 6.5 6.0 5.5 5.0 4.5 4.0 3.5 3.0 2.5 2.0 1.5 1.0 0.5 0.0
f1 (ppm)

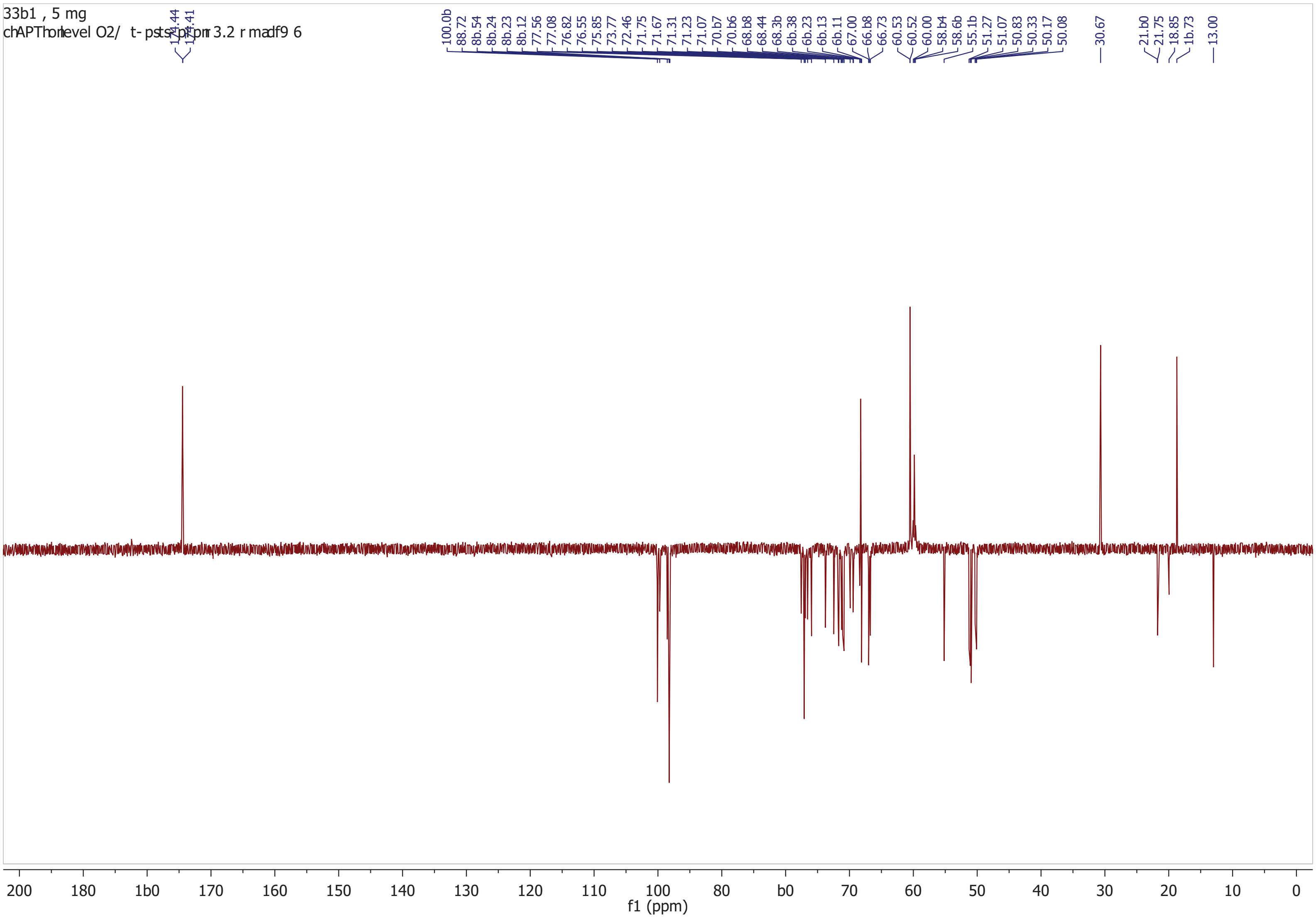
33b1 , 5 mg
chAPThonlevel O2/ t- psts-prpm 3.2 r madf9 6
174.44
174.41
100.0b
88.72
8b.54
8b.24
8b.23
8b.12
77.56
77.08
76.82
76.55
75.85
73.77
72.46
71.75
71.67
71.31
71.23
71.07
70.b7
70.b6
68.b8
68.44
68.3b
6b.38
6b.23
6b.13
6b.11
67.00
66.b8
66.73
60.53
60.52
60.00
58.b4
58.6b
55.1b
51.27
51.07
50.83
50.33
50.17
50.08
30.67
21.b0
21.75
18.85
1b.73
13.00
200
180
1b0
170
160
150
140
130
120
110
100
90
80
b0
70
60
50
40
30
20
10
0
f1 (ppm)
